# State-level needs for social distancing and contact tracing to contain COVID-19 in the United States

**DOI:** 10.21203/rs.3.rs-40364/v2

**Published:** 2020-09-16

**Authors:** Weihsueh A. Chiu, Rebecca Fischer, Martial L. Ndeffo-Mbah

**Affiliations:** 1 Department of Veterinary Integrative Biosciences, College of Veterinary Medicine and Biomedical Sciences, Texas A&M University, College Station, TX 77845; 2 Department of Epidemiology and Biostatistics, School of Public Health, Texas A&M University, College Station, TX 77845

**Keywords:** COVID-19, social distancing, testing, contact tracing, mathematical modeling, Bayesian analysis

## Abstract

Starting in mid-May 2020, many US states began relaxing social distancing measures that were put in place to mitigate the spread of COVID-19. To evaluate the impact of relaxation of restrictions on COVID-19 dynamics and control, we developed a transmission dynamic model and calibrated it to US state-level COVID-19 cases and deaths. We used this model to evaluate the impact of social distancing, testing and contact tracing on the COVID-19 epidemic in each state. As of July 22, 2020, we found only three states were on track to curtail their epidemic curve. Thirty-nine states and the District of Columbia may have to double their testing and/or tracing rates and/or rolling back reopening by 25%, while eight states require an even greater measure of combined testing, tracing, and distancing. Increased testing and contact tracing capacity is paramount for mitigating the recent large-scale increases in U.S. cases and deaths.

The novel coronavirus pandemic (COVID-19) emerged in Wuhan, China in December 2019 and has now reached pandemic status, with spread to more than 210 countries and territories, including the United States (US) ^1^. The US reported its first imported case of COVID-19 on January 20, 2020, arriving via an international flight from China ^2^. Since then, the disease has spread rapidly within the US, with every state reporting confirmed cases within three weeks of the first reported community transmission. As of August 1^st^, the US has exceeded 4.5 million cases and 150,000 deaths, heterogeneously distributed across all states ^1^. So far, states such as New York, New Jersey, and California have borne the highest burden with more than 420,000, 183,000, and 510,000 cases and 32,000, 15,000, and 9,000 deaths, respectively, while Alaska and Hawaii have each reported less than 4000 cases and 25 deaths each ^1^.

COVID-19 is caused by a newly described and highly transmissible SARS-like coronavirus (SARS-CoV-2). Severe clinical outcomes have been observed in approximately 20% of symptomatic cases ^3,4^. There is no vaccine and no cure or approved pharmaceutical intervention for this disease, making the fight against the pandemic reliant on non-pharmaceutical interventions (NPIs). These NPIs include: case-driven measures, such as testing, contact tracing, and isolation ^5^; personal preventive measures such as hand hygiene, cough etiquette, face mask use, eye protection, physical distancing, and surface cleaning, which aim to reduce the risk of transmission during contact with potentially-infectious individuals ^6^; and social distancing measures to reduce interpersonal contact in the population. In the US, social distancing measures have included policies and guidelines to close schools and workplaces, cancel and restrict mass gatherings and group events, restrict travel, maintain physical separation from others (e.g. keeping six feet distance), and stay-at-home orders ^7^.

NPIs and other responses to COVID-19, especially stay-at-home orders, have varied widely across states, leading to spatial and temporal variation in the timing and implementation of mitigation strategies. This variation in policies and response efforts may have contributed to the observed heterogeneity in COVID-19 morbidity and mortality across states ^8^. Recent studies suggest that statewide social distancing measures have likely contributed to reducing the spread COVID-19 epidemic in the US ^9,10^. Understanding the extent to which NPIs, such as social distance, testing, contact tracing, and self-quarantine, influence COVID-19 transmission in a local context is pivotal for predicting and better managing the future course of the epidemic on a state-by-state basis. This in turn will inform how these NPIs should be optimized to mitigate the spread and burden of COVID-19 while awaiting development of pharmaceutical interventions (e.g. therapeutics and vaccines).

After several weeks of statewide stay-at-home orders, most US states began to ease their social distancing requirements in May-June, 2020 ^11^, while attempting to increase their testing and contact tracing capacities ^12^. Mathematical modeling is a unique tool to help answer these important and timely questions. Models can contribute valuable insight for public health decision-makers by providing an evaluation of the effectiveness of ongoing control strategies along with predictions of the potential impact of alternative policy scenarios ^13^.

To address these needs, we developed and validated a data-driven transmission dynamic model to evaluate the impact of social distancing, state-reopening, testing, and contact tracing on the state-level dynamics of COVID-19 infections and mortality in the US, shown schematically in Figure 1. Like many other COVID-19 transmission models ^14–17^, we used an extended SEIR (susceptible, exposed, infectious, removed) compartmental model. The model divides the population into several disease compartments and tracts movements of individuals between the compartments through different transition rates. The main model compartments include: *S*, susceptible, *E*, exposed, *A*, infectious and asymptomatic, *I*, infectious and symptomatic, *R*, recovered, and *F*, dead. In addition to disease progression stages, our model incorporates social distancing informed by several public sources of mobility data, case identification via testing, isolation of detected cases, and contact tracing. This is a mean-field epidemiological modeling approach that captures the average disease dynamics behavior within a population ^18,19^. We used Bayesian inference methods to calibrate and validate our model prediction to state-level daily reported COVID-19 cases and fatality data. Model parameters, prior distributions, and their sources are shown in Table 1. We used the calibrated model to evaluate the transmissibility of COVID-19 in each state from March, 2020 to late July, 2020, to estimate the state-level impact of shelter-in-place and reopening on COVID-19 transmission. Finally, we evaluated the degree to which increasing testing efforts (rate of identification of infected cases) and/or contact tracing could curtail the spread of the diseases and enable greater relaxation of social distancing restrictions while preventing a resurgence of infections and deaths. A detailed description of the model considerations, parameterization, and analysis is provided in Methods.

## Results

### Model performance and validation

**Figure 1.**
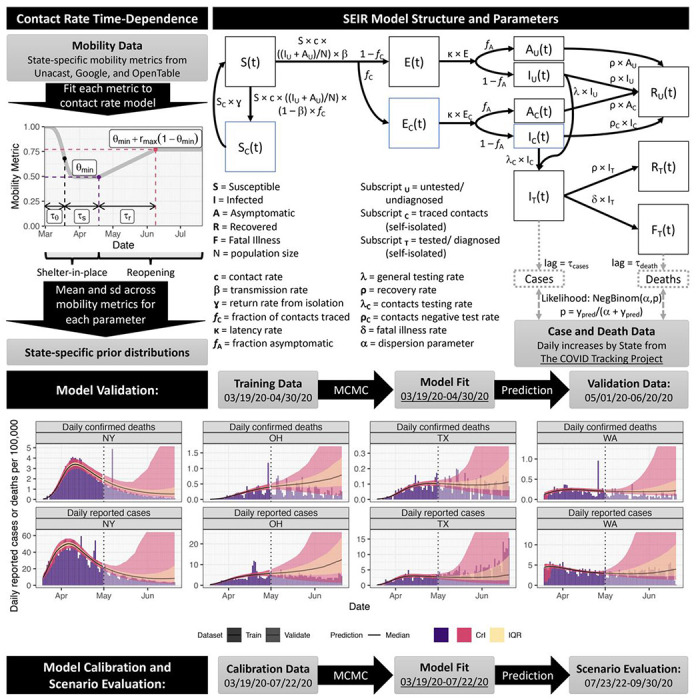
SEIR model structure, parameter, data sources, and fitting/validation methods. We used mobility data to constrain the time-dependence of the contact rate. We fitted the model to daily reported cases and confirmed deaths from March 19^th^ to April 30^th^ and validated its projections against data from May 1^st^ to June 20^th^. On the model projections, the black solid line is the median, the pink band is the 95% credible interval (CrI) and the orange is the interquartile range (IQR). We show model fitting and validation for four states: New York (NY), Ohio (OH), Texas (TX), and Washington (WA).

We used state-level mobility data from Unacast, Google, and OpenTable to calibrate a parametric model of shelter-in-place and reopening (Supplementary Figure 1), and used the results to inform prior distributions for the transmission model (Figure 1). We fit our model to state-level daily cases and deaths data using a Bayesian inference approach (see Methods). Model performance assessment for several representative states is shown in Figure 1, with full results in Supplementary Figures 2 and 3. With respect to validation, the posterior 95% credible interval of our model projections, estimated using data through April 30^th^, 2020, covered 84% of the data points from May 1^st^ through June 20^th^, 2020. For seven states, validation had low coverage (<50%) because of insufficient training data through April 30 to adequately inform sheltering and reopening in those states. Specifically, for Alaska, Montana, and South Dakota, a greater surge of cases due to a greater reopening occurred than predicted, and in Iowa, Illinois, Michigan, and Minnesota, shelter-in-place continued longer than predicted. This inaccuracy was not unexpected because the length of sheltering and the degree of reopening could not have been known on April 30th, and thus our model predictions were based on generic prior distributions. However, during model calibration to data through July 22nd, these parameters were informed by updated state-specific mobility data. Model performance for fitting all data through July 22^nd^ is shown in Supplementary Figures 4-6, with posterior parameter distributions shown in Supplementary Figure 7. Good fits with high coverage were obtained for all states.

### Estimations of effective reproduction number

**Figure 2.**
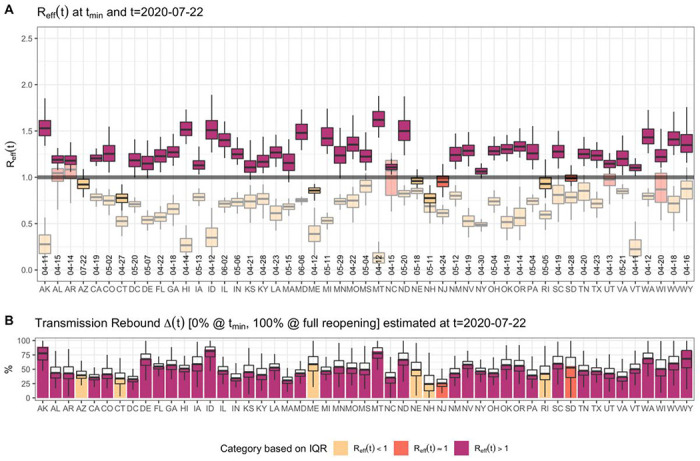
Estimated effective reproduction number *R_eff_* and the level of reopening/rebound in transmission as of July 22^nd^, 2020 for all states. (A) shows estimated *R_eff_* (median, IQR, and 95% CrI) across States. The figure shows the value of *R_eff_* on July 22^nd^, 2020, as well as the “minimum” value of *R_eff_* between March 19^th^, 2020 and July 22^nd^, 2020, in lighter shades of each color. It also includes the date of the minimum *R_eff_*. (B) shows the level of reopening/rebound in disease transmission in each state relative to its minimum value during state shelter-in-place (median, IQR, and 95% CrI).

The effective reproduction number, *R_eff_* the average number of secondary infection cases generated by a single infectious individual during her infectious period ^18^. When *R_eff_* the epidemic curve is increasing, and when *R_eff_*, the epidemic curve is decreasing ^18^. Using the posterior distribution of our model parameters we estimated the effective reproduction number *R_eff_* from March 19^th^ to late July, 2020 and identified the minimum level of transmission achieved in each state (Figure 2A). We found that for all except five states (Alabama, Arkansas, North Carolina, Wisconsin, and Utah), the inter-quartile range for the minimum *R_eff_* value was less than 1 and these values were mainly achieved during the state shelter-in-place (Figure 2A). Following states’ relaxations of social distancing measures, disease transmission started to re-increase. By July 22^nd^, 2020, 42 states and the District of Columbia had at least a 75% probability that *R_eff_*>1. Thus, the model predicts that as states are reopening, a majority of states are at risk of continued increases in the scale of the outbreak and require additional mitigation to contain the spread of the disease.

We conducted an analysis of variance to evaluate the contribution of each parameter to the variation in *R_eff_* value (Supplementary Table 1). Across states, we found that the largest drivers of variation in *R_eff_* are the power parameter for relating social distancing to hygiene-associated reduction in transmission, *η*, degree of mitigation during shelter-in-place, *θ_min_*, the maximum relative increase in contact after shelter-in-place orders, *r_max_*, and the fraction of contact traced, *f_c_*, which together contribute over 50% of variance (Extended Data Figure 1). This observation is consistent with mobility data alone being insufficient to account for the combined effect of multiple control measures, and suggest that the degree of adoption of non-mobility-related measures, such as enhanced hygiene practices and contact tracing, play a large role in the extent to which a state may reduce disease transmission.

For each state, we defined *Δ* as the level of reopening/rebound (*Δ* = 0% at minimum, 100% at full reopening) in disease transmission relative to its lowest transmission rate observed during shelter-in-place, and estimated the current level of reopening/rebound (Figure 2B). We found that 24 states had a 50% or more rebound in COVID-19 transmission by July 22^nd^, 2020, while no states had a 25% or less rebound in transmission (Figure 2B).

### Impact of testing and contact tracing on easing of social distancing

Bringing and keeping the effective reproduction number, *R_eff_*, below 1 is necessary to curtail the spread of an outbreak. We evaluated the probability of keeping *R_eff_*<1 for different levels of testing and contact tracing under the July 22^nd^, 2020 level of state reopening. We found that for most states bringing and keeping *R_eff_*<1 may not be possible without increased contact tracing efforts, as increasing testing and isolation alone would be sufficient or require extremely high coverage to curtail the epidemic curve with a 0.975 probability (Extended Data Figures 2 & 3, and Supplementary Table 2). The challenges are even greater to ensure continued control of the epidemic with full reopening, which require much larger increases in tracing and testing (Extended Data Figure 4, Supplementary Table 3).

**Figure 3.**
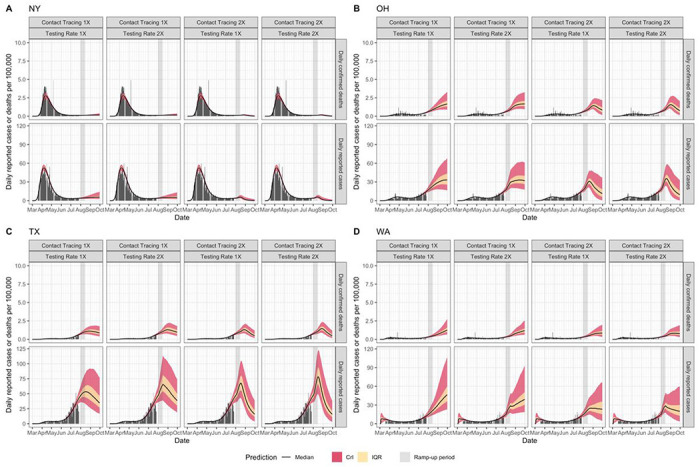
Predicted time-course (median, IQR, and 95% CrI) of daily reported cases and deaths under different testing and contact tracing rates (1X and 2X) in New York (A), Ohio (B), Texas (C), and Washington State (D).

To evaluate the impact of scaling up testing and contact tracing on the epidemic dynamics in each state, we assumed a linear “ramp-up” of either testing and/or contact tracing from August 1^st^ – 14^th^, 2020, after which both parameters remain constant. We then predicted the daily number of reported cases and deaths (Figure 3 and Supplementary Figure 8). We found that under current levels of reopening and control, almost all states would see a continued increase in reported cases and deaths (Supplementary Figure 8). Even with increased testing and contact tracing, some of these states will still experience a short-term increase in reported cases and deaths (Figure 3 and Supplementary Figure 8). For example, Ohio, Texas, and Washington may experience a substantial short-term increase of cases and deaths even if their current testing and contact tracing rate were doubled within the next two weeks (Figure 3B–D). Moreover, reported cases increase during the “ramp-up” period (Figure 3). We also found that in most states additional relaxation of restrictions without simultaneously increasing contact tracing may exacerbate disease dynamics and result in large-scale outbreaks (Supplementary Figure 10).

**Figure 4.**
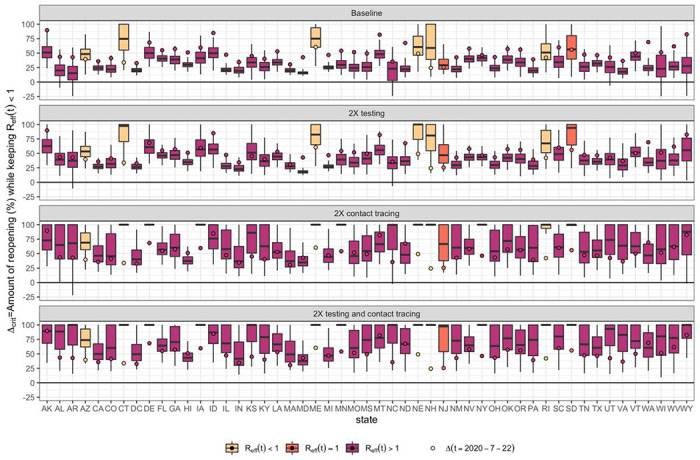
Reopening/rebound in transmission *Δ_crit_* permitted (0% = minimum shelter-in-place value, 100% = return to no restrictions) to keep *R_eff_* < 1. (A) If testing and contact rates are unchanged, (B) testing rate is doubled, (C) contact tracing is doubled, or (D) both testing and contact tracing are doubled. *Δ* (*t*) the level of reopening/rebound in transmission on July 22^nd^, 2020 is shown by the circle. All boxplots show median, IQR, and 95% CrI.

We next evaluated the maximal degree of rebound in transmission (i.e., level of reopening) permitted while keeping *R_eff_*<1 under different testing and contact tracing scenarios (Figure 4). We found that under the current level of testing and contact tracing rate, 27 states cannot keep their *R_eff_*<1 (at 75% confidence) even with only 25% reopening/rebound in transmission (Figure 4A). By doubling the current testing rate, eight states could keep their *R_eff_*<1 even with a 50% level of reopening (Figure 4B). By doubling contact tracing, nine states could remove all mobility restrictions while keeping *R_eff_*<1 (Figure 4C). By doubling both testing rate and contact tracing, ten states could remove all mobility restrictions while keeping *R_eff_*<1 (Figure 4D).

**Figure 5.**
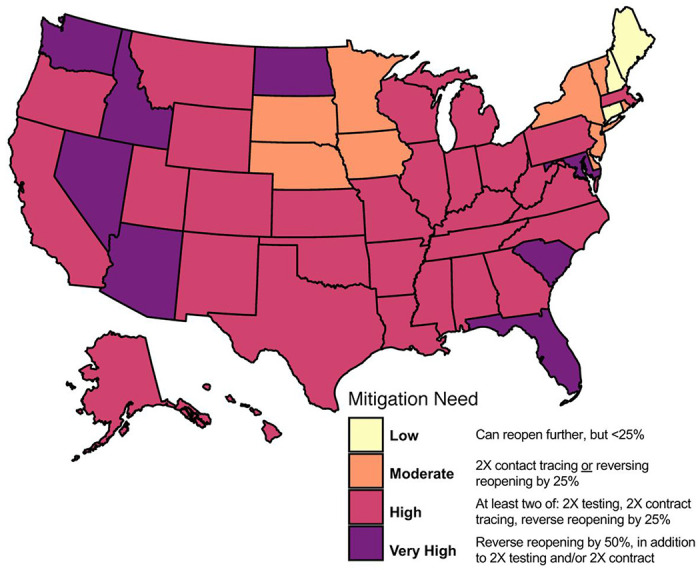
State-specific level of mitigation needed as a July 22^nd^, 2020 to curtail the spread of COVID-19 (keeping *R* < 1 with at least 75% confidence, equivalent to the upper bound of the Interquartile range (IQR)). Categories based on evaluating scenarios with different combinations of baseline/doubling testing, baseline/doubling contact tracing, and baseline/+25%/−25% in the reopening parameter *Δ*. Categories are defined as follows: *Very Low (no states)*: Can reopen further by >25% while keeping *R*(t)<1; *Low (3 states)*: Can reopen further by < 25% with up to 2X increase in testing while keeping *R*(t)<1; *Moderate (9 states)*: Requires 2X contact tracing or reversal of reopening by 25% to keep *R*(t)<1; *High (30 states and DC)*: Requires multiple interventions (2X testing, 2X contract tracing, reversal of reopening by 25%) to keep *R*(t)<1; *Very High (8 states)*: Reverse of reopening by 50%, combined with 2X testing and/or 2X contact tracing with to keep *R*(t)<1.

We categorized states by the additional amount of mitigation efforts needed to keep *R*(*t*) < 1 with at least 75% confidence (Figure 5 and Supplementary Figure 8). We found that under current control efforts, no states could reduce and keep *R*(*t*) < 1 if their current level of reopening was relaxed by an additional 25% (“Very Low” category), and three states (Connecticut, Maine, New Hampshire) could reduce and keep *R*(*t*) < 1 without additional reopening (“Low” category). Eight states could reduce and keep *R*(*t*) < 1 by doubling their contact tracing rate or implementing additional social distancing restrictions, a 25% reversal of current level of reopening (“Moderate” category), while 30 states and the District of Columbia need a combined intervention of doubling both testing and contact tracing and/or 25% reversal of current reopening to reduce and keep *R*(*t*) < 1(“High” category). For the remaining eight states (Arizona, Florida, Idaho, Maryland, North Dakota, Nevada, South Carolina, and Washington) a 50% reversal of current reopening in addition to increased testing and/or contact tracing are needed in order to to reduce and keep *R*(*t*) < 1 (“Very High” Category).

## Discussion

There is a delicate and continuous balance to strike between the use of social distancing measures to mitigate the spread of an emerging and deadly disease such as COVID-19 and the need for re/opening various sectors of activities for the social, economic, mental, and physical well-being of a community. To address this issue, it is imperative to design measurable, data-driven, and flexible milestones for identifying when to make specific transitions with regard to easing or retightening specific social distancing measures. We developed a data-driven SARS-CoV-2 transmission dynamic model not only to make short-term predictions on COVID-19 incidence and mortality in the US, but more importantly to evaluate the impact that relaxing social distancing measures and increasing testing and contact tracing would have on the epidemic in each state.

We showed that in most states, control strategies implemented during their “shelter-in-place” period were sufficient to contain the outbreak, defined as reducing and ultimately maintaining the effective reproduction number below 1 (*R_eff_*<1). However, for the majority of states, our modelling suggests that “reopening” has proceeded too rapidly and/or without adequate testing and contact tracing to prevent a resurgence of the epidemic. Our model suggests for some states, a substantial fraction of the population may have already been infected such that even without additional intervention, *R_eff_*(*t*) is declining towards (or below) 1 even as *R*(*t*)>1. The most extreme example is Arizona, where *R_eff_*(*t*) is estimated to have declined below the previous minimum *R_eff_* value achieved during shelter-in-place. However, accurate estimation of the susceptible fraction of the population is difficult due to uncertain degree of undercounting in the reported case data. Thus, we used *R*(*t*) to categorize the mitigation needs in each state and evaluate the level of control effort needed to curtail the spread of the epidemic in each state.

Moreover, even in states with currently decreasing incidence and mortality, such as Maine and New Jersey, additional relaxation of restrictions is likely to “bend the epidemic curve upwards” in the absence of increased testing or tracing. However, our model predicts that a combination of increased testing, increased contact tracing, and/or scaling back reopening will be sufficient for curtailing the spread of COVID-19 in most states. Specifically, doubling of current testing and contact tracing rates would enable the majority of states to either maintain or increase the easing of social distancing restrictions in a “safe” manner in the short term. Scaling back the current level of reopening by 25% in combination with doubling of testing and tracing will be sufficient to control the epidemic in the long term in all but eight “Very High” risk states. The impact of these interventions on the epidemic curve was evaluated by computing their probability of reducing and keeping the reproduction number below one. However, in states with high overdispersion in disease transmission, epidemic with high superpreadability characteristics, the reproduction number may be subject to large fluctuation as the number of infection cases decreases. This may more likely be the case for states with lower dispersion parameters posterior values such as Arkansas, Connecticut, Idaho, Kansas, Kentucky, Louisiana, Mississippi, New Hampshire, South Carolina, and Wyoming (see Supplementary Figure 7).

Increasing testing and contact tracing rates entails both increasing the number of tests performed per day as well as requiring early identification and effective isolation of COVID-19 infected individuals. This can be accomplished through active case detection via efficient contact tracing strategies. However, it should also be noted that increased testing and contact tracing will lead to a short-term increase in reported cases because a larger fraction of the infected population is being observed, and that several weeks may pass before these rates begin to show a decline. Therefore, it is imperative that policymakers and the public recognize that such a surge is actually a sign that testing and tracing efforts are succeeding, and exercise the patience to wait several weeks before these successes are reflected as declining rates of reported cases.

Other modeling studies have used SEIR-type compartmental models to assess the impact of social distancing, testing and contact tracing to curb the epidemic curve in Italy and the United Kingdom ^14–17^. Consistent with our results, these studies have shown that rapid reopening of the economy without adequate testing and contact tracing could lead to a resurgence of the epidemic ^14–17^. Specifically, they show that high testing and contact tracing rates may enable to maintain/increase the easing of social distancing restrictions without an increased rate of COVID-19 transmission ^14^.

Our study has several limitations due to modelling assumptions and the quality of available data. Like most COVID-19 transmission models ^14–17^, we used a compartmental SEIR-type model to model the spread of SARS-CoV-2 because of its simplicity and ability to capture population average dynamics. This modeling approach does not account for heterogeneity in individual-level behavior, overdispersion due to “super-spreaders,” social contact networks, and inherent stochasticity which may play an important role in SARS-CoV-2 transmission dynamics. These factors can be modeled through the use of individual-based models ^20–22^. However, individual-based modeling is a more complex modeling framework and may require a substantial amount of individual-level data for model parameterization, calibration, and validation.

To characterize the limitations of using cell phone-based mobility data to infer (prior distributions for) contact rates, we examined the state-to-state variation in mobility data to the corresponding posterior distributions for each mobility-related parameter (see Supplementary Figure 9). Three parameters of particular interest are the minimum relative contact rate *θ_min_*, the duration of the shelter-in-place phase *τ_S_*, and the maximum amount of reopening *r_max_*. For *θ_min_*, none of the r^2^ values were consistently less than 0.2, although the slope and intercept of the regression line for the Unacast Visitation metric were within 15% of 1 and 0, respectively. Similarly, for *τ_s_*, the highest r^2^ value was 0.37, for OpenTable Bookings data, which also had a relatively accurate regression line (again within 15%). For *r_max_*, the highest r^2^ values were for Google retail and recreation (0.49), and Unacast Visitation (0.52) metrics, but the Google data were much more accurate, with a slope close to 1 and intercept close to 0. Overall, these results suggest that cell-phone based mobility data vary substantially in their accuracy (slope and intercept near 1 and 0, respectively) and overall have low precision (no r^2^ more than about 0.5), and supports our use of the range across multiple sources in developing prior distributions, rather than using such data directly for modeling contact rates.

The initiation of social distancing measures, such as stay-at-home orders in the US, for mitigating the spread of COVID-19 has occurred concurrently with increased promotion and application of other NPIs, such as hygiene practices (e.g. hand hygiene, surface cleaning, cough etiquette, and wearing of face mask). These hygiene practices coupled with the avoidance of physical contact whenever possible (keeping six feet apart) could impact the spread of COVID-19 by reducing both the risk of exposure and the risk of transmission of SARS-CoV-2 from infected patients ^23,24^. Though our model explicitly accounts for the differential contribution of social distancing (mobility reduction) versus hygiene practices and physical distancing to reducing COVID-19 transmission, we assume that the impact of hygiene practices and physical distancing was a function of social distancing (mobility reduction). While cell phone mobility data may continue to be informative as to contact rates, at least in aggregate, the impact of enhanced hygiene practices is more difficult to measure independently. As several states have eased their social distancing requirements, especially their stay-at-home orders, compliance with hygiene practices would become even more important for reducing individuals’ risk of getting or transmitting the pathogen. However, keeping a high population-level adherence to these measures is required to mitigate the spread of the COVID-19 epidemic ^25^. As states are reopening various aspects of their economy, data on compliance with enhanced hygiene practices and physical distancing are needed to improve the estimation of these measures’ population-level impact on reducing disease transmission.

Additionally, consistent with previous COVID-19 modeling studies ^26–28^, our model uses a simple functional form to model increases in testing rate from early March to June, 2020. This testing rate was estimated through model fitting to daily reported case and mortality data. Particularly in states that have seen a substantial increase in testing capability and efforts during the month of May, our simple time varying assumption may underestimate the current level of testing and contact tracing. However, it should be noted that increased testing capacity does not necessarily lead to increased rate of testing if individuals are unaware, unwilling, or unable to be tested ^29^. Having contact tracing and date of symptoms onset data would enable us to compute a better estimate of the current testing and contact tracing rate in each state. Our model also assumes that all individuals who test positive to COVID-19 are effectively isolated for the rest of their infectious period and no longer contribute to disease transmission. Though voluntary compliance to COVID-19 self-quarantine recommendations may be high across the US, it is likely not 100%. Therefore, the assumption of effective isolation of all identified cases may cause our model to slightly overestimate the impact of increased testing rate on disease dynamics. However, we anticipate that this assumption would only have a marginal impact on the qualitative nature of our results.

Finally, our model does not explicitly account for age-stratified risk of disease transmission and mortality. This age-stratification is important for designing and evaluating social distancing and testing strategies that are targeted towards the elderly population, which is at higher risk of COVID-19-induced hospitalization and death ^30^. As reopening the economy becomes an imperative for states across the US, age- or risk-targeted interventions may be a valuable tool to mitigate the burden of the pandemic. Future modeling studies could investigate the effectiveness of age- or risk-targeted non-pharmaceutical and potential pharmaceutical (vaccine or therapeutic) interventions for controlling the spread and burden of COVID-19.

In sum, we use a data-driven mathematical modeling approach to study the impacts of social distancing, testing, and contact tracing on the transmission dynamics of SARS-CoV-2. Our findings emphasize the importance for public health authorities not only to monitor the case and mortality dynamics of SARS-CoV-2 in their state, but also to understand the impact of their existing social distancing measures on SARS-CoV-2 transmission and evaluate the effectiveness of their testing and contact tracing programs for promptly identifying and isolating new cases of COVID-19. As reported case rates are increasing widely across US states because social distancing restrictions have been eased to allow more economic activity to resume, we find that most states need to either significantly scale back reopening or enhance their capacity and scale of testing, case isolation, and contact tracing programs in order to mitigate large-scale increases in COVID-19 cases and deaths.

## Methods

Our overall approach is as follows: 1) develop a mathematical model (an SEIR-type compartmental model)^18,19^ that incorporates social distancing data, case identification via testing, isolation of detected cases, and contact tracing; 2) assess the model’s predictive performance by training (calibrating) it to reported cases and mortality data from March 19^th^ to April 30^th^, 2020 and validating its predictions against data from May 1^st^ to June 20^th^, 2020; and 3) use the model, trained on data through July 22^nd^, 2020, to predict future incidence and mortality. The final stage of our approach predicts future events under a set of scenarios that include increased case detection through expanded testing rate, contact tracing, and relaxation or increase of measures to promote social distancing. All model fitting is performed in a Bayesian framework in order to incorporate available prior information and address multivariate uncertainty in model parameters.

### Model formulation

We modified the standard SEIR model to address testing and contact tracing, as well as asymptomatic individuals. A fraction *f_A_* of those exposed (*E*) to enter the asymptomatic *A* class (divided into *A_U_* for untested, and *A_C_* for contact traced) instead of the infected *I* class, which in our model formulation also includes infectious pre-symptomatic individuals. With respect to testing, separate compartments were added for untested, “freely roaming” infected individuals (*I_U_*), tested/isolated cases *I_T_*, fatalities *F_T_*. Upon recovery, untested infected individuals *I_U_*) and all asymptomatic individuals move to the untested recovered compartment *I_U_*, and tested infected individuals move to the tested recovered compartment *I_T_*. In balancing considerations of model fidelity and parameter identifiability, we made the reasonably conservative assumptions that all tested cases are effectively isolated (through self-quarantine or hospitalization) and thus unavailable for transmission, and that all COVID-related deaths are identified/tested.

With respect to contact tracing, the additional compartment *S_C_* represents unexposed contacts, who undergo a period of isolation during which they are not susceptible before returning to *S*; while *E_C_, A_C_*, and *I_C_* represent contacts who were exposed. Again, the reasonably conservative assumption was made that all exposed contacts undergo testing, with an accelerated testing rate compared to the general population. We assume a closed population of constant size *N* for each state.

The ordinary differential equations governing our model are as follows:

$$\begin{array}{c} \frac{dS}{dt}=-S\cdot c\cdot \left[\beta +(1-\beta )\cdot f_{C}\right]\cdot \left(I_{U}+A_{U}\right)/N+S_{C}\cdot \gamma \\ \frac{dS_{C}}{dt}=-S_{C}\cdot \gamma +S\cdot c\cdot (1-\beta )\cdot f_{C}\cdot \left(I_{U}+A_{U}\right)/N\\ \frac{dE}{dt}=-E\cdot \kappa +S\cdot c\cdot \beta \cdot \left(1-f_{C}\right)\cdot \left(I_{U}+A_{U}\right)/N\\ \frac{dE_{C}}{dt}=-E_{C}\cdot \kappa +S\cdot c\cdot \beta \cdot f_{C}\cdot \left(I_{U}+A_{U}\right)/N\\ \frac{dI_{U}}{dt}=-I_{U}\cdot (\lambda +\rho )+E\cdot \kappa \cdot \left(1-f_{A}\right)\\ \frac{dA_{U}}{dt}=-A_{U}\cdot \rho +E\cdot \kappa \cdot f_{A}\\ \frac{dI_{C}}{dt}=-I_{C}\cdot \left(\lambda_{C}+\rho_{C}\right)+E_{C}\cdot \kappa \cdot \left(1-f_{A}\right)\\ \frac{dA_{C}}{dt}=-A_{C}\cdot \rho_{C}+E_{C}\cdot \kappa \cdot f_{A}\\ \frac{dR_{U}}{dt}=\left(I_{U}+A_{U}+A_{C}\right)\cdot \rho +I_{C}\cdot \rho_{C}\\ \frac{dI_{T}}{dt}=-I_{T}\cdot (\rho +\delta )+I_{U}\cdot \lambda +I_{C}\cdot \lambda_{C}\\ \frac{dR_{T}}{dt}=I_{T}\cdot \rho \\ \frac{dF_{T}}{dt}=I_{T}\cdot \delta \end{array}$$

*c* is the contact rate between individuals, *β* is the transmission probability per infected contact, *f_C_* is the fraction of contacts identified through contact tracing, 1/*γ* is the duration of self-isolation after contact tracing, 1/*κ* is the latent period, *f_A_* is the fraction of exposed who are asymptomatic, *λ* is the testing rate, *δ* is the fatality rate, *ρ* is the recovery rate, *λ_C_* and *ρ_C_* is the testing rate and recovery rate of contact traced individuals, respectively. The testing rates *λ* and *λ_C_*, the fatality rate *δ*, and the recovery rate of traced contacts *ρ_C_* are each composites of several underlying parameters. The testing rate defined as

$$\lambda (t)=F_{test,0}\cdot \left[1-\frac{1}{1+e^{\left(t-T50_{T}\right)/\tau_{T}}}\right]\cdot {Sens}_{test}\cdot k_{test},$$

where *F*_*test*,0_ is the current testing coverage (fraction of infected individuals tested), *Sens_test_* is the test sensitivity (true positive rate), and *k_test_* is rate of testing for those tested, with a typical time-to-test equal to 1/*k_test_*. The time-dependence term models the “ramp-up” of testing using a logistic function with a growth rate of 1/*τ_T_ days*^−1^, where *T*50_*T*_ is the time where 50% of the current testing rate is achieved. Similarly, for testing of traced contacts, the same definition is used with the assumption that all identified contacts are tested, *F*_*test*,0_ = 1 and at a faster assumed testing rate *k_C,test_*:

$$\lambda_{C}(t)=\left[1-\frac{1}{1+e^{\left(t-T50_{T}\right)/\tau_{T}}}\right]\cdot {Sens}_{test}\cdot k_{C,test},$$

Because all contacts are assumed to be tested, the rate *ρ_C_* at which they enter the “recovered” compartment *R_U_* is simply the rate of false negative test results:

$$\rho_{C}(t)=\left[1-\frac{1}{1+e^{\left(t-T50_{T}\right)/\tau_{T}}}\right]\cdot \left(1-{Sens}_{test}\right)\cdot k_{test}$$

The fatality rate is adjusted to maintain consistency with the assumption that all COVID-19 deaths are identified, assuming a constant infected fatality rate (*IFR*). Specifically, we first calculated the fraction of infected that are tested and positive

$$f_{pos}(t)=f_{C}\frac{\lambda_{C}(t)}{\lambda_{C}(t)+\rho_{C}(t)}+\left(1-f_{C}\right)\frac{\lambda (t)}{\lambda (t)+\rho }.$$

Then the case fatality rate *CFR*(*t*) = *IFR/f_pos_*(*t*). Because the *CFR* = *δ*/(*δ* + *ρ*), this implies

$$\delta (t)=\rho \frac{CFR(t)}{1-CFR(t)}=\rho \frac{IFR}{f_{pos}(t)-IFR}.$$

The model is “seeded” *N_initial_* cases on February 29^th^, 2020. Because in the early stages of the outbreak, there may be multiple “imported” cases, we only fit to data from March 19^th^, 2020 onwards, one week after the U.S. travel ban was put in place ^31^.

Our model is fit to daily case *y_c_* and death *y_d_* data (cumulative data are not used for fitting because of autocorrelation). To adequately fit the case and mortality data, we accounted for two lag times. First, a lag is assumed between leaving the *I_U_* compartment and public reporting of a positive test result, accounting for the time it takes to seek a test, obtaining testing, and have the result reported. No lag is assumed for tests from contact tracing. Second, a lag time is assumed between entering the fatally ill compartment *F_T_* and publically reported deaths. Additionally, we use a negative binomial likelihood in order to account for the substantial day-to-day overdispersion in reporting results. The corresponding equations are as follows:

$$\begin{array}{c} y_{obs,[c,d]}(t)∼NegBin\left[\alpha_{\left[c,d\right]},p_{\left[c,d\right]}(t)\right]\\ p_{\left[c,d\right]}(t)=\frac{y_{pred,[c,d]}(t)}{\alpha_{\left[c,d\right]}+y_{pred,[c,d]}(t)}\\ y_{pred,c}(t)=I_{U}\left(t-\tau_{case}\right)\cdot \lambda (t)+I_{C}(t)\cdot \lambda_{C}(t)\\ y_{pred,d}(t)=I_{T}\left(t-\tau_{death}\right)\cdot \delta (t) \end{array}$$

In this parameterization, as the dispersion parameter *α* → ∞, the likelihood becomes a Poisson distribution with expected value *y*_*pred*,[*c,d*]_, whereas for small values of *α* there is substantial interindividual variability. Case and death data were sourced from The COVID Tracking Project ^32^.

Finally, we derived the time-dependent reproduction number, *R*(*t*) and the effective reproduction number,*R_eff_*(*t*) of this model, given by

$$R(t)=c\cdot \beta \cdot \left(1-f_{C}\right)\left(\frac{1-f_{A}}{\lambda +\rho }+\frac{f_{A}}{\rho }\right)$$

and

$$R_{eff}(t)=R(t)\cdot \frac{S(t)}{N}.$$

Using posterior samples for all 50 states and the District of Columbia, we conducted an analysis of variance using a linear model to characterize the contributions to the combined inter- and intra-state variation in *R_eff_*. Specifically, we used a linear model for *R_eff_* with the model parameters R_0_, |, ⎝_min_, r_max_, *f_C_*, *f_A_*, ⌊, and 〉 as predictors, and evaluated the percentage of variance in *R_eff_* contributed by each parameter.

### Incorporating social distancing, enhanced hygiene practices, and reopening

The impact of social distancing, hygiene practices, and reopening were modeled through a time-dependence in the contact rate *c* and the transmission probability per infected contact *β*:

$$\begin{array}{c} c(t)=c_{0}\cdot \left[\theta (t)+\left(1-\theta_{min}\right)\cdot r(t)\right]\\ \beta (t)=\beta_{0}\cdot \theta {\left(t\right)}^{\eta } \end{array}$$

The *θ*(*t*) function parameterized social distancing during the progression to shelter-in-place, and is modeled as a Weibull function

$$\theta (t)=\theta_{min}+\left(1-\theta_{min}\right)e^{-{\left(t/\tau_{\theta }\right)}^{n}\theta },$$

which starts a unity and decreases to *θ_min_*, with *τ_θ_* being Weibull scale parameter and *n_θ_* the Weibull shape parameter (Figure 1).

The *r*(*t*) function parameterized relative increase in contacts due to reopening after shelter-in-place, with *r* = 1 corresponding to a return to baseline *c* = *c*_0_.


$$\begin{array}{c} r(t)=r_{max}\frac{t-\tau_{\theta }-\tau_{s}}{\tau_{r}}\left[u\left(t-t_{r}\right)-u\left(t-t_{rmax}\right)\right]+u\left(t-t_{rmax}\right)\\ u(t)=Heaviside(t)\approx 1-\frac{1}{1+e^{4t}}\\ t_{r}=\tau_{\theta }+\tau_{s}\\ t_{rmax}=\tau_{\theta }+\tau_{s}+\tau_{r} \end{array}$$

The term *r*(*t*) is 0 before *t_r_*, linear between *t_r_* and *t_rmax_*, and constant at a value of *r_max_* after that, and made continuous by approximating the Heaviside function by a logistic function. The reopening time is defined as *τ_S_* days after *τ_θ_*, and the maximum relative increase in contacts *r_max_* happens *τ_r_* days after that.

We selected the functional form above for *c*(*t*) because it was found to be able to represent a wide variety of social distancing data, including cell phone mobility data from Unacast ^33^ and Google ^34^, as well as restaurant booking data from OpenTable ^35^. We used these different mobility sources to derive state-specific prior distributions because different social distancing datasets had different values for *θ_min_, τ_θ_, n_θ_*, *τ_S_, r_max_*, and *τ_R_* (Figure S1).

With respect to the reduction in transmission probability *β*, we assumed that during the “shelter-in-place” phase, hygiene-based mitigation paralleled this decline with an effectiveness power *η*, and that this mitigation continued through re-opening.

Finally, we define an overall “reopening” parameter *Δ* that measures the “rebound” in disease transmission *c* ⋅ *β* relative to its minimum, defined to be 0 during shelter-in-place (i.e., *R*(*t*) is at a minimum), and 1 when all restrictions are removed (when *R*(*t*) = *R*_0_), which can be derived as:

$$\Delta (t)=\frac{c\cdot \beta /\left(c_{0}\cdot \beta_{0}\right)-\theta_{min}^{1+\eta }}{1-\theta_{min}^{1+\eta }}.$$

Our model is illustrated in Figure 1, with parameters and prior distributions listed in Table 1.

### Scenario evaluation

We used the model to make several inferences about the current and future course of the pandemic in each state. First, we consider the effective reproduction number. Two time points of particular interest are the time of minimum *R_eff_*, reflecting the degree to which shelter-in-place and other interventions were effective in reducing transmission, and the final time of the simulation, July 22^nd^, 2020, reflecting the extent to which reopening has increased *R_eff_*. Additional parameters of interest are the current levels of reopening *Δ*(*t*), testing *λ*, and contact tracing *f_C_*.

We then conducted scenario-based prospective predictions using our model’s parameters as estimated through July 22^nd^, 2020. We asked the following questions:

- Assuming current levels of reopening, what increases in general testing *λ* and/or contact tracing *f_C_* would be necessary to bring *R_eff_* < 1?
- What amount reopening *Δ* can maintain *R_eff_* < 1 under four different scenarios: current values of testing and contact tracing, doubling testing, double tracing, and doubling both testing and tracing?
- What will the rates of new cases and deaths be under different scenarios? Specifically, we evaluate the impact of increases in testing and contact tracing under current levels of reopening, as well as increases or decreases of 25% or 50%.

For (a), we evaluated the posterior probability that *R_eff_* < 1 under scaling transformations *λ* → *λ* · *μ_λ_* and *f_C_* → *f_C_* ⋅ *μ_C_* with scaling factors *μ_λ_* and *μ_C_*:

$$R_{eff}(t)=S(t)\cdot c\cdot \beta \cdot \left(1-\mu_{C}\cdot f_{C}\right)\left(\frac{1-f_{A}}{\mu_{\lambda }\cdot \lambda +\rho }+\frac{f_{A}}{\rho }\right)$$

We additionally derived “critical” values of *μ_C_* and *μ_λ_* where *R_eff_* (*t*) < 1 under the conditions of increased testing only (*μ_C_* = 1), increased contact tracing only (*μ_λ_* = 1), and equal increases in testing and tracing (*μ_C_* = *μ_λ_*). We also performed the same analysis under a full re-opening scenario (i.e., setting *S*(*t*) = 1, *c* = *c*_0_, and *β* = *β*_0_).

For (b), we re-arranged the equation for *R_eff_* in terms of the reopening parameter *Δ*

$$R_{eff}(t)=S(t)\cdot c_{0}\cdot \beta_{0}\cdot \left(1-\mu_{C}\cdot f_{C}\right)\left(\frac{1-f_{A}}{\mu_{\lambda }\cdot \lambda +\rho }+\frac{f_{A}}{\rho }\right)\left[\Delta \cdot \left(1-\theta_{min}^{1+\eta }\right)+\theta_{min}^{1+\eta }\right]$$

We then fixed the scaling factors at 1 or 2, and solved the above equation for *Δ_crit_* such that *R_eff_* < 1. Values of *Δ_crit_* ≥ *Δ*(*t*) indicate the additional degree of reopening possible while maintaining *R_eff_* < 1, while values of *Δ_crit_* < *Δ*(*t*) indicate a reduction of reopening is needed. To convert back to testing and contact tracing rates, we multiplied the scaling factors *μ_C_* and *μ_λ_* by the original values of *f_C_* and *λ*, respectively.

Finally, for (c), we additionally evaluated changes in reopening *Δ* → *Δ* + *Δ_Δ_* for *Δ_Δ_* values of +25% (+50%) or −25% (−50%), for a total of 20 scenarios (4 different levels of testing and tracing, and 5 different levels of reopening). We then ran the SEIR model forward in time until September 30th, 2020. For all three intervention parameters *μ_C_, μ_λ_*, and *Δ_Δ_*, we assumed a “ramp-up” period of 2 weeks from August 1^st^-14^th^, 2020.

To summarize the relative need for mitigation in each state, we categorized states based on which scenarios resulted in the IQR of *R*(*t*) being < 1 on August 15^th^, 2020. The categories were defined as follows:

- **Very Low:** Can reopen further by >25% while keeping *R*(*t*) < 1;
- **Low:** Can reopen further by < 25% with up to 2X increase in testing while keeping *R*(*t*) < 1;
- **Moderate:** Requires 2X contact tracing or reversal of reopening by 25% to bring and keep *R*(*t*) < 1;
- **High:** Requires multiple interventions (2X testing, 2X contract tracing, reversal of reopening by 25%) to bring and keep *R*(*t*) < 1;
- **Very High:** Combining 2X testing, 2X contact tracing, and reversal of reopening by 50% is needed to bring and keep *R*(*t*) < 1.

We use *R*(*t*) instead of *R_eff_*(*t*) minimize the impact of heterogeneity and uncertainty in the value of *S*(*t*)/*N* on our results. Thus, requiring *R*(*t*) < 1 provides greater assurance of state-wide control of the epidemic.

## Figures and Tables

**Table 1.** Model inputs, parameters and prior distributions for Bayesian analysis.

Symbol	Definition (units)	Calibrated parameter(s)	Prior [Truncation]	Notes
*N*	Population size	Input (not calibrated)	Constant	^40^
*N_init_*	Initial I_U_ on 2020-02-29	*N_init_*	LogN(1000, 10) [1, 10000]	¶
1/ɣ	Self-isolation time after contact tracing	T_isolation_ = 1/ɣ	LogN(14, 2) [7, 21]	ϯ
1/κ	Latent period (d)	T_latent_ = 1/κ	N(4,1) [2,7]	^41,42^
c_0_	Baseline contact rate (contacts d^−1^)	c_0_	N(13, 5) [7, 20]	^43^
ρ	Recovery rate (d^−1^)	T_recover_ = 1/ρ	LogN(10, 1.5) [5, 30]	^42,44^
β_0_	Transmission probability per contact (unitless)	R_0_ = c_0_β_0_/ρ	N(2.9, 0.78) [1.46, 4.5]	^45–47^
f_C_	Fraction of contacts traced (unitless)	f_C_	LogN(0.25, 2) [0.05, 1]	^48^
f_A_	Fraction of infected asymptomatic (unitless)	f_A_	N(0.295,0.275) [0.02, 0.57]	^49^
T50_T_	Date of 50% of final testing rate (d)	T50_T_	U(60, 106) (Mar 1 – Apr 15)	¶
λ	General positive diagnosis rate (d^−1^)	λ = F_test_ Sens_test_ k_test_	Derived	^45,50,51^
F_test_	General test coverage (unitless)	F_test_	Beta(2,2)	^45,50,51^
Sens_test_	Test sensitivity (unitless)	Sens_test_	N(0.7, 0.1) [0.6, 0.95]	^52^
k_test_	General testing rate (d^−1^)	τ_test_ = 1/k_test_	N(7, 3) [2, 12]	^53,54^
λ_C_	Contacts positive diagnosis rate (d^−1^)	λ_C_ = Sens_test_ k_test,C_	Derived	
k_C,test_	Contacts testing rate (d^−1^)	τ_C,test_ = 1/k_C,test_	N(2, 1) [1, 3]	¶
ρ_C_	Rate of infected contacts testing negative (d^−1^)	ρ_C_ = (1 − Sens_test_) k_test,C_	Derived	
δ	Fatal illness rate (d^−1^)	IFR (infected fatality rate)*	LogN(0.01, 2) [0.001, 0.1]	^44,55^
θ_min_	Minimum of θ(t)	θ_min_	Validation: Beta(2,2) Calibration: State-specific	¶ Ƣ
τ_θ_	Weibull scale parameter	τ_θ_	Validation: N(21, 7) [7, 35] Calibration: State-specific	¶ Ƣ
n_θ_	Weibull shape parameter	n_θ_	Validation: LogN(6, 2) [1,11] Calibration: State-specific	¶ Ƣ
η	Hygiene effectiveness relative to social distancing (unitless)	η	Beta(2,4)	¶
τ_s_	Duration of shelter in place (d)	τ_s_	Validation: N(45, 30) [21, 90] Calibration: State-specific	^56^
τ_r_	Duration of linear increase after shelter-in-place (d)	τ_r_	Validation: N(45, 30) [14, 105] Calibration: State-specific	¶ Ƣ
r_max_	Maximum relative increase in contacts from shelter-in-place (unitless)	r_max_	Validation: N(1, 1) [0, 2] Calibration: State-specific	¶ Ƣ
τ_case_	Lag time for observing confirmed case	τ_case_	LogN(7, 2) [1, 14]	¶
τ_death_	Lag time for observing confirmed death	τ_death_	LogN(7, 2) [1, 14]	¶
α_pos_	Negative Binomial shape parameter for cases likelihood function	α_pos_	LogU(0.1, 40)	¶
α_death_	Negative Binomial shape parameter for deaths likelihood function	α_death_	LogU(0.1, 40)	¶

LogN(GM, GSD) = lognormal distribution with geometric mean GM and geometric standard deviation GSD

N(M,SD) = normal distribution with mean M and standard deviation SD

U(MIN,MAX) = uniform distribution with minimum MIN and maximum MAX

LogU(MIN, MAX) = log-uniform distribution with minimum MIN and maximum MAX

Beta(a,b) = beta distribution with shape parameters a and b

Time (t) is measured from t=1 corresponds to 2020-01-01.

¶ Assumed, non-informative prior wide enough to have adequate validation coverage.

ϯ Standard contact tracing guidance is to self-isolate for 2 weeks.

Ƣ For calibration to 6/20/20, state-specific priors were derived by fitting to different social distancing data sets, with each parameter’s mean, standard deviation, and range used to define a normal distribution prior.

* See Methods for relationship between IFR and δ.

## Data Availability

The following publicly available datasets are used:
Mobility data from Unacast were sourced from https://covid19-scoreboard-api.unacastapis.com/api/search/covidstateaggregates_v3. Mobility data from Google were sourced from https://www.gstatic.com/covid19/mobility/Global_Mobility_Report.csv. Restaurant booking data were sourced from OpenTable https://www.opentable.com/state-of-industry. Case and death data were sourced from The COVID Tracking Project (https://covidtracking.com/). Mobility data are shown in Supplemental Figure 1. Case and death data are shown in Figures 1 and 3, and Supplemental Figures 3-6, 8. All data used are also available in the software and code repository.

## References

[1] Johns Hopkins Coronavirus Resource Center. Available at: https://coronavirus.jhu.edu/. (Accessed: 18th May 2020)

[2] Holshue M. L. First Case of 2019 Novel Coronavirus in the United States. N. Engl. J. Med. 382, 929–936 (2020). 32004427 10.1056/NEJMoa2001191 PMC7092802

[3] Weiss P. & Murdoch D. R. Clinical course and mortality risk of severe COVID-19. The Lancet 395, 1014–1015 (2020). 10.1016/S0140-6736(20)30633-4 PMC7138151 32197108

[4] Wu Z. & McGoogan J. M. Characteristics of and Important Lessons from the Coronavirus Disease 2019 (COVID-19) Outbreak in China: Summary of a Report of 72314 Cases from the Chinese Center for Disease Control and Prevention. JAMA - Journal of the American Medical Association 323, 1239–1242 (2020). 32091533 10.1001/jama.2020.2648

[5] Kucharski A. J. Effectiveness of isolation, testing, contact tracing and physical distancing on reducing transmission of SARS-CoV-2 in different settings. medRxiv 2020.04.23.20077024 (2020). doi:10.1101/2020.04.23.20077024 PMC7511527 32559451

[6] Chu D. K. Physical distancing, face masks, and eye protection to prevent person-to-person transmission of SARS-CoV-2 and COVID-19: a systematic review and meta-analysis. Lancet 0, (2020). 10.1016/S0140-6736(20)31142-9 PMC7263814 32497510

[7] What is social distancing and how can it slow the spread of COVID-19? | Hub. Available at: https://hub.jhu.edu/2020/03/13/what-is-social-distancing/. (Accessed: 18th May 2020)

[8] Bialek S. Geographic Differences in COVID-19 Cases, Deaths, and Incidence — United States, February 12—April 7, 2020. MMWR. Morb. Mortal. Wkly. Rep. 69, 465–471 (2020). 32298250 10.15585/mmwr.mm6915e4 PMC7755058

[9] Siedner M. J. Social distancing to slow the U.S. COVID-19 epidemic: an interrupted time-series analysis. medRxiv 2020.04.03.20052373 (2020). doi:10.1101/2020.04.03.20052373

[10] Wagner A. B. Social Distancing Has Merely Stabilized COVID-19 in the US. medRxiv 2020.04.27.20081836 (2020). doi:10.1101/2020.04.27.20081836

[11] Lifting Social Distancing Measures in America: State Actions & Metrics | The Henry J. Kaiser Family Foundation. Available at: https://www.kff.org/coronavirus-policy-watch/lifting-social-distancing-measures-in-america-state-actions-metrics/. (Accessed: 18th May 2020)

[12] Testing in the U.S. | CDC. Available at: https://www.cdc.gov/coronavirus/2019-ncov/cases-updates/testing-in-us.html. (Accessed: 18th May 2020)

[13] Garnett G. P., Cousens S., Hallett T. B., Steketee R. & Walker N. Mathematical models in the evaluation of health programmes. The Lancet 378, 515–525 (2011). 10.1016/S0140-6736(10)61505-X 21481448

[14] Giordano G. Modelling the COVID-19 epidemic and implementation of population-wide interventions in Italy. Nat. Med. 26, 855–860 (2020). 32322102 10.1038/s41591-020-0883-7 PMC7175834

[15] Russo L. Tracing DAY-ZERO and Forecasting the Fade out of the COVID-19 Outbreak in Lombardy, Italy: A Compartmental Modelling and Numerical Optimization Approach. medRxiv (Cold Spring Harbor Laboratory Press, 2020). doi:10.1101/2020.03.17.20037689 PMC7598513 33125393

[16] Gevertz J., Greene J., Tapia C. H. S. & Sontag E. D. A novel COVID-19 epidemiological model with explicit susceptible and asymptomatic isolation compartments reveals unexpected consequences of timing social distancing. medRxiv 2020.05.11.20098335-2020.05.11.20098335 (2020). doi:10.1101/2020.05.11.20098335 PMC7840295 33242489

[17] Colbourn T. Modelling the Health and Economic Impacts of Population-Wide Testing, Contact Tracing and Isolation (PTTI) Strategies for COVID-19 in the UK. SSRN Electron. J. (2020). doi:10.2139/ssrn.3627273

[18] Anderson R. M., May R. M. & Anderson B. Infectious Diseases of Humans: Dynamics and Control (Oxford Science Publications). (Oxford University Press, USA, 1992).

[19] Murray J. D. Mathematical Biology I. An introduction. Interdisciplinary Applied Mathematics 17, (2002).

[20] Moghadas S. M. The implications of silent transmission for the control of COVID-19 outbreaks. Proc. Natl. Acad. Sci. 202008373 (2020). doi:10.1073/pnas.2008373117 PMC7395516 32632012

[21] Chinazzi M. The effect of travel restrictions on the spread of the 2019 novel coronavirus (COVID-19) outbreak. Science (80-. ). 368, 395–400 (2020). 10.1126/science.aba9757 PMC7164386 32144116

[22] Kerr C. C. Covasim: an agent-based model of COVID-19 dynamics and interventions. medRxiv 2020.05.10.20097469 (2020). doi:10.1101/2020.05.10.20097469 PMC8341708 34310589

[23] Leung N. H. L. Respiratory virus shedding in exhaled breath and efficacy of face masks. Nat. Med. 26, 676–680 (2020). 32371934 10.1038/s41591-020-0843-2 PMC8238571

[24] Greenhalgh T., Schmid M. B., Czypionka T., Bassler D. & Gruer L. Face masks for the public during the covid-19 crisis. BMJ 369, (2020). 10.1136/bmj.m1435 32273267

[25] Eikenberry S. E. To mask or not to mask: Modeling the potential for face mask use by the general public to curtail the COVID-19 pandemic. Infect. Dis. Model. 5, 293–308 (2020). 32355904 10.1016/j.idm.2020.04.001 PMC7186508

[26] Pitzer V. E. The impact of changes in diagnostic testing practices on estimates of COVID-19 transmission in the United States. medRxiv 2020.04.20.20073338 (2020). doi:10.1101/2020.04.20.20073338 PMC8083380 33831148

[27] Yamana T., Pei S., Kandula S. & Shaman J. Projection of COVID-19 Cases and Deaths in the US as Individual States Re-open May 4, 2020. medRxiv 2020.05.04.20090670 (2020). doi:10.1101/2020.05.04.20090670

[28] Peirlinck M., Linka K., Sahli Costabal F. & Kuhl E. Outbreak dynamics of COVID-19 in China and the United States. Biomech. Model. Mechanobiol. 1–15 (2020). doi:10.1007/s10237-020-01332-5 PMC7185268 32342242

[29] U.S. states see COVID-19 testing supply improvements, but challenges abound - The Washington Post. Available at: https://www.washingtonpost.com/health/as-coronavirus-testing-expands-a-new-problem-arises-not-enough-people-to-test/2020/05/17/3f3297de-8bcd-11ea-8ac1-bfb250876b7a_story.html. (Accessed: 18th May 2020)

[30] Keeling M. J. Predictions of COVID-19 dynamics in the UK: short-term forecasting and analysis of potential exit strategies. doi:10.1101/2020.05.10.20083683 PMC7857604 33481773

[31] Presidential Proclamation — Travel From Europe. Available at: https://travel.state.gov/content/travel/en/traveladvisories/presidential-proclamation-travel-from-europe.html. (Accessed: 18th May 2020)

[32] The COVID Tracking Project | The COVID Tracking Project. Available at: https://covidtracking.com/. (Accessed: 18th May 2020)

[33] Data for Good. Available at: https://www.unacast.com/data-for-good. (Accessed: 18th May 2020)

[34] COVID-19 Community Mobility Reports. Available at: https://www.google.com/covid19/mobility/. (Accessed: 15th June 2020)

[35] State of the Industry | OpenTable. Available at: https://www.opentable.com/state-of-industry. (Accessed: 18th May 2020)

[36] Bois F. Y. GNU MCSim: Bayesian statistical inference for SBML-coded systems biology models. Bioinformatics 25, 1453–1454 (2009). 19304877 10.1093/bioinformatics/btp162

[37] Gelman A. & Rubin D. B. Inference from Iterative Simulation Using Multiple Sequences. Statistical Science 7, 457–472

[38] RStudio | Open source & professional software for data science teams - RStudio. Available at: https://rstudio.com/. (Accessed: 18th May 2020)

[39] R: The R Project for Statistical Computing. Available at: https://www.r-project.org/. (Accessed: 18th May 2020)

[40] programs-surveys/popest/datasets/2010-2019/state/detail. Available at: https://www2.census.gov/programs-surveys/popest/datasets/2010-2019/state/detail/. (Accessed: 19th May 2020)

[41] Lauer S. A. The Incubation Period of Coronavirus Disease 2019 (COVID-19) From Publicly Reported Confirmed Cases: Estimation and Application. Ann. Intern. Med. (2020). doi:10.7326/M20-0504 PMC7081172 32150748

[42] He X. Temporal dynamics in viral shedding and transmissibility of COVID-19. Nat. Med. 26, 672–675 (2020). 32296168 10.1038/s41591-020-0869-5

[43] Mossong J. Social contacts and mixing patterns relevant to the spread of infectious diseases. PLoS Med. 5, e74 (2008). 18366252 10.1371/journal.pmed.0050074 PMC2270306

[44] Verity R. Estimates of the severity of coronavirus disease 2019: a model-based analysis. Lancet. Infect. Dis. 0, (2020). 10.1016/S1473-3099(20)30243-7 PMC7158570 32240634

[45] Li Q. Early Transmission Dynamics in Wuhan, China, of Novel Coronavirus–Infected Pneumonia. N. Engl. J. Med. 382, 1199–1207 (2020). 31995857 10.1056/NEJMoa2001316 PMC7121484

[46] Riou J. & Althaus C. L. Pattern of early human-to-human transmission of Wuhan 2019 novel coronavirus (2019-nCoV), December 2019 to January 2020. Eurosurveillance 25, 2000058 (2020). 32019669 10.2807/1560-7917.ES.2020.25.4.2000058 PMC7001239

[47] Park S. W. Reconciling early-outbreak estimates of the basic reproductive number and its uncertainty: a new framework and applications to the novel coronavirus (2019-nCoV) outbreak. medRxiv 2020.01.30.20019877 (2020). doi:10.1101/2020.01.30.20019877 PMC7423425 32693748

[48] Covid Act Now. Available at: https://covidactnow.org/?s=49762. (Accessed: 15th June 2020)

[49] Gao Z. A systematic review of asymptomatic infections with COVID-19. Journal of Microbiology, Immunology and Infection (2020). doi:10.1016/j.jmii.2020.05.001 PMC7227597 32425996

[50] Arons M. M. Presymptomatic SARS-CoV-2 Infections and Transmission in a Skilled Nursing Facility. N. Engl. J. Med. (2020). doi:10.1056/nejmoa2008457 PMC7200056 32329971

[51] Nishiura H. Estimation of the asymptomatic ratio of novel coronavirus infections (COVID-19). (2020). doi:10.1016/j.ijid.2020.03.020 PMC7270890 32179137

[52] Watson J., Whiting P. F. & Brush J. E. Interpreting a covid-19 test result. BMJ 369, ml808 (2020). 10.1136/bmj.m1808 32398230

[53] Cummings M. J. Epidemiology, clinical course, and outcomes of critically ill adults with COVID-19 in New York City: a prospective cohort study. Lancet 395, 1763–1770 (2020). 32442528 10.1016/S0140-6736(20)31189-2 PMC7237188

[54] Sun K., Chen J. & Viboud C. Early epidemiological analysis of the coronavirus disease 2019 outbreak based on crowdsourced data: a population-level observational study. Lancet Digit. Heal. 2, e201–e208 (2020). 10.1016/S2589-7500(20)30026-1 PMC7158945 32309796

[55] Onder G., Rezza G. & Brusaferro S. Case-Fatality Rate and Characteristics of Patients Dying in Relation to COVID-19 in Italy. JAMA - Journal of the American Medical Association 323, 1775–1776 (2020). 32203977 10.1001/jama.2020.4683

[56] Map: How states are reopening after coronavirus shutdown - Washington Post. Available at: https://www.washingtonpost.com/graphics/2020/national/states-reopening-coronavirus-map/. (Accessed: 15th June 2020)

