# Supplementary material for: State-level impact of social distancing and testing on COVID-19 in the United States
Source: Res Sq. 2020 Jul 7:rs.3.rs-40364. Preprint. [Version 1] doi: 10.21203/rs.3.rs-40364/v1 (PMC7362894; doi:10.21203/rs.3.rs-40364/v1)
Supplement: Supplementary file 1 [file SupplementalMaterials.docx]

**Supplemental Materials: State-level impact of social distancing and testing on COVID-19 in the United States**

Weihsueh A. Chiu, Ph.D^1^, Rebecca Fischer, PhD, MPH^2^, Martial L. Ndeffo-Mbah, Ph.D^1,2^

^1^Department of Veterinary Integrative Biosciences, College of Veterinary Medicine and Biomedical Sciences, Texas A&M University, College Station, TX 77845

^2^Department of Epidemiology and Biostatistics, School of Public Health, Texas A&M University, College Station, TX 77845

Figure S1. Schematic of model for fitting mobility data.  The same model formulation is used for contact rate.  Using March 1, 2020 as t=1, the initial decline is modeled as a Weibull function, with scale parameter τ_θ_ shape parameter n_θ_, and minimum value θ_min_.  Then after a shelter-in-place period τ_s_, a linear increase begins that lasts for a duration τ_r_ and plateaus at a value θ_min_ + r_max _(1 - θ_min_), were r_max_ is the maximum percentage increase relative to the minimum.

Figure S2. Model fitting of mobility data each state. We fitted the model to daily reported cases and confirmed deaths until April 30th and validated its projections against data from May 1st to June 20th. On the model projections, the black solid line is the median, the pink band is the 95% credible interval (CrI) and the orange is the inter-quartile range (IQR). [**Provided as separate file**]

*Figure S3. Validation runs: Comparison of data and predictions overall. Each panel shows data versus predictions for daily deaths (left column) or cases (right column), with calibration results shown in the top panels (March 19 through April 30) and validation results in the bottom panels (May 1 through June 20). Each point includes the 95% Credible Interval (CrI) for the prediction. The band centered on the y=x line shows the additional 95% CrI for the negative binomial likelihood, which is used to account for day-to-day variation in reporting.*

Figure S4. Model fitting and validation for each state. We fitted the model to daily reported cases and confirmed deaths until April 30th and validated its projections against data from May 1st to June 20th. On the model projections, the black solid line is the median, the pink band is the 95% credible interval (CrI) and the orange is the inter-quartile range (IQR). [**Provided as separate file**]

Figure S5. Model calibration through June 20, 2020: time-courses for selected States.

Figure S6. Model calibration through June 20, 2020: comparisons of data and predictions for all States.

Figure S7. Comparison of prior and posterior parameter distributions for each state for model calibration through June 20, 2020. **[Provided as separate file]**

Figure S8. Correlations across states between R_eff_(t) and (A) θ_min_, (B) η, (C) Δ, and (D) f_C_.  For each state, 500 posterior samples are shown.  Substantial state-to-state heterogeneity is evident in all parameters, with η, r_max_, and  f_C_ contribute over 60% of the variance in R_eff_(t) under a linear model (estimated from ANOVA table [Table S1] using the sum-of-squares relative to the total sum-of-squares).  For θ_min_, non-linearity is evident, as a positive correlation is apparent for states with R_eff_(t)<1 and a negative one for states with R_eff_(t)>1.  The other parameters correlate as expected: higher R_eff_(t) is correlated with lower contribution from hygiene practices (smaller η), more reopening (larger Δ), and lower rates of contact tracing (smaller f_C_).

Table S1. ANOVA table for R_eff_(t) with respect to key parameters

|  | Df | Sum Sq | PctSumSq | Mean Sq | F value |  | Pr(>F) |
| --- | --- | --- | --- | --- | --- | --- | --- |
| R_0_ | 1 | 200 | 7.7% | 200 | 13766.9 | < | 2.20E-16 |
| η | 1 | 627.48 | 24.1% | 627.48 | 43192.9 | < | 2.20E-16 |
| θ_min_ | 1 | 64.52 | 2.5% | 64.52 | 4441.4 | < | 2.20E-16 |
| Δ | 1 | 580.43 | 22.3% | 580.43 | 39954.1 | < | 2.20E-16 |
| *f*_C_ | 1 | 537.84 | 20.6% | 537.84 | 37022.7 | < | 2.20E-16 |
| λ | 1 | 156.66 | 6.0% | 156.66 | 10783.6 | < | 2.20E-16 |
| ρ | 1 | 70.21 | 2.7% | 70.21 | 4833 | < | 2.20E-16 |
| Residuals | 25492 | 370.33 | 14.2% | 0.01 |  |  |  |
| TOTAL |  | 2607.47 |  |  |  |  |  |

Figure S9. Contour maps for each state of the probability that R_eff_(t) at different levels contact tracing f_C_ and testing λ. Contours are labeled as by median and 95% Credible interval, and current median estimates of f_C_ and λ are shown by the circle.

Figure S10. Predicted time-courses across states of daily reported cases (A, C) and deaths (B, D) under different testing rate (1X and 2X) and contact tracing (1X and 2X) scenarios, given current value of restriction relaxation (current Δ). [**Provided as separate file**]
