# Supplementary material for: State-level impact of social distancing and testing on COVID-19 in the United States
Source: Res Sq. 2020 Jul 7:rs.3.rs-40364. Preprint. [Version 1] doi: 10.21203/rs.3.rs-40364/v1 (PMC7362894; doi:10.21203/rs.3.rs-40364/v1)
Supplement: Supplementary file 1 [file FigS10ScenariosIncNegReopeningResultsFigures202006201.pdf]

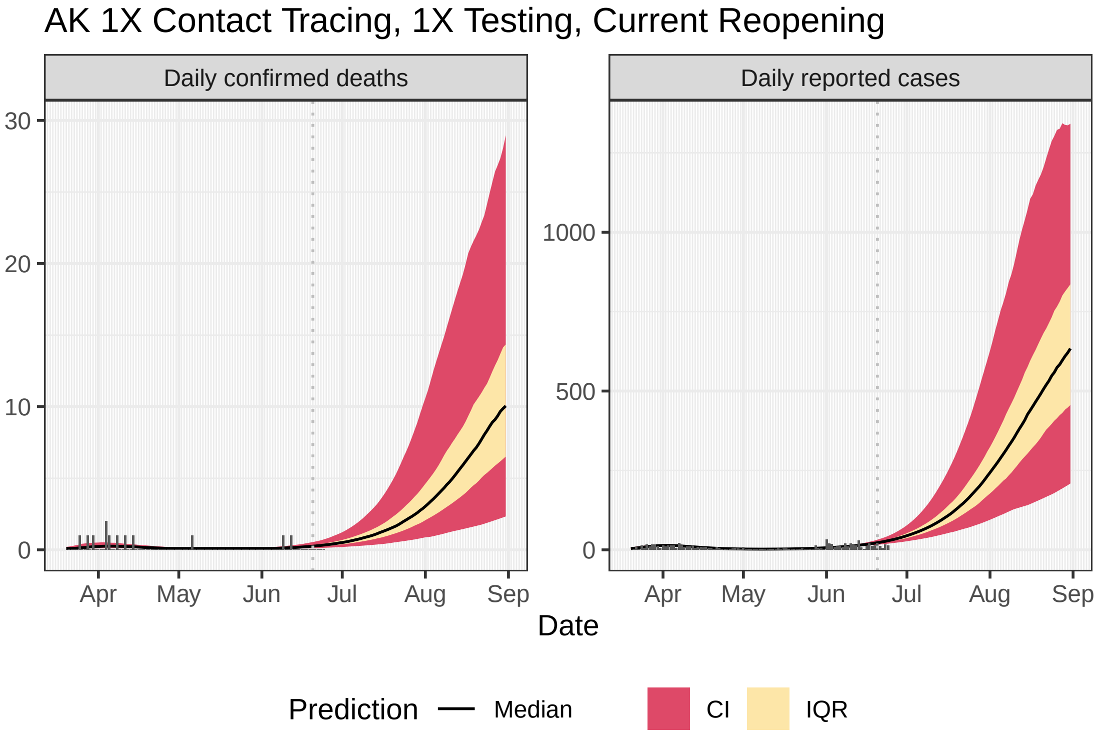

AK 1X Contact Tracing, 1X Testing, Current Reopening
Daily confirmed deaths
Daily reported cases
30
20
10
0
1000
500
0
Apr
May
Jun
Jul
Aug
Sep
Date
Prediction
Median
CI
IQR

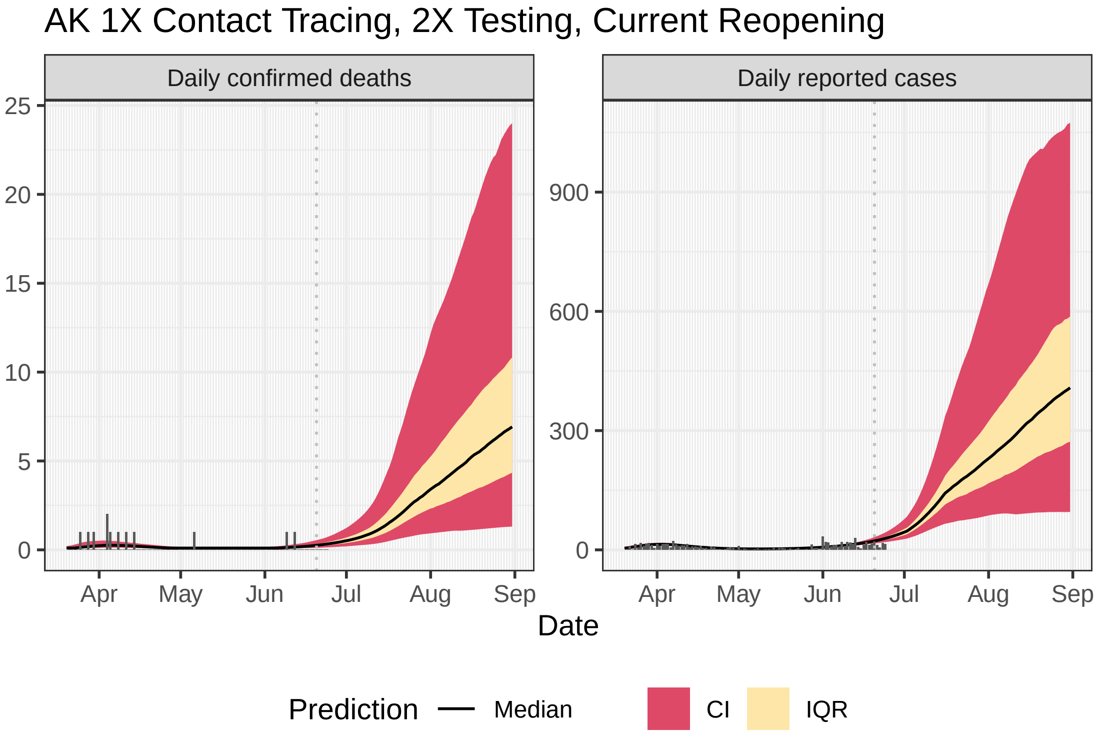
AK 1X Contact Tracing, 2X Testing, Current Reopening
Daily confirmed deaths
Daily reported cases
25
20
15
10
5
0
900
600
300
0
Apr
May
Jun
Jul
Aug
Sep
Date
Prediction
Median
CI
IQR

AK 2X Contact Tracing, 1X Testing, Current Reopening

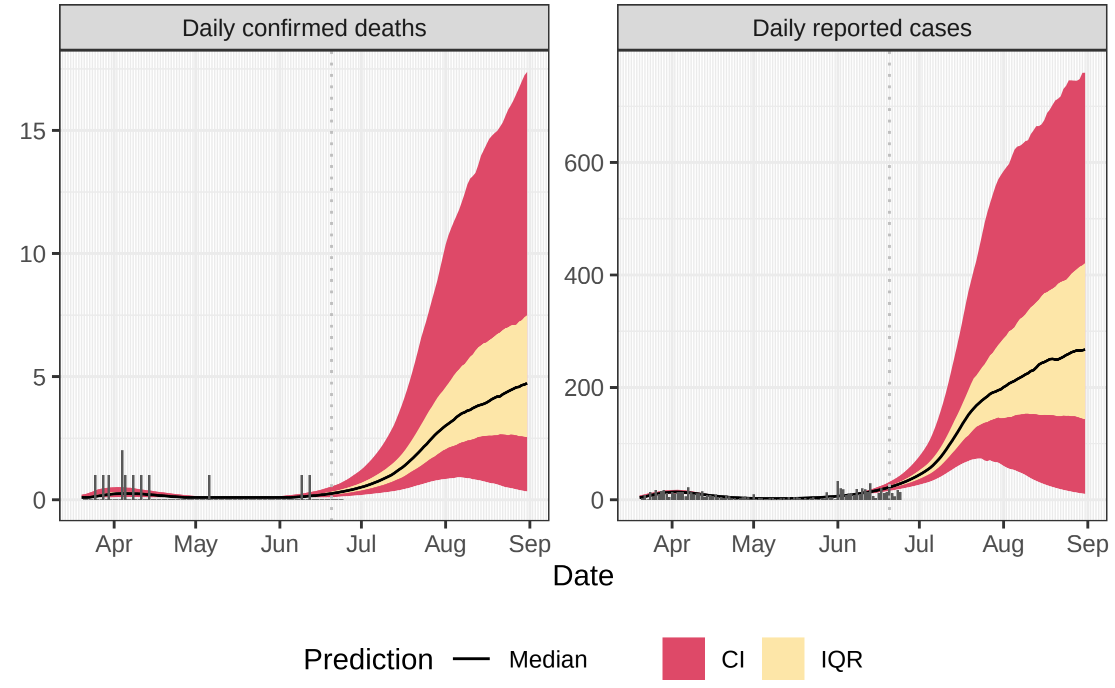

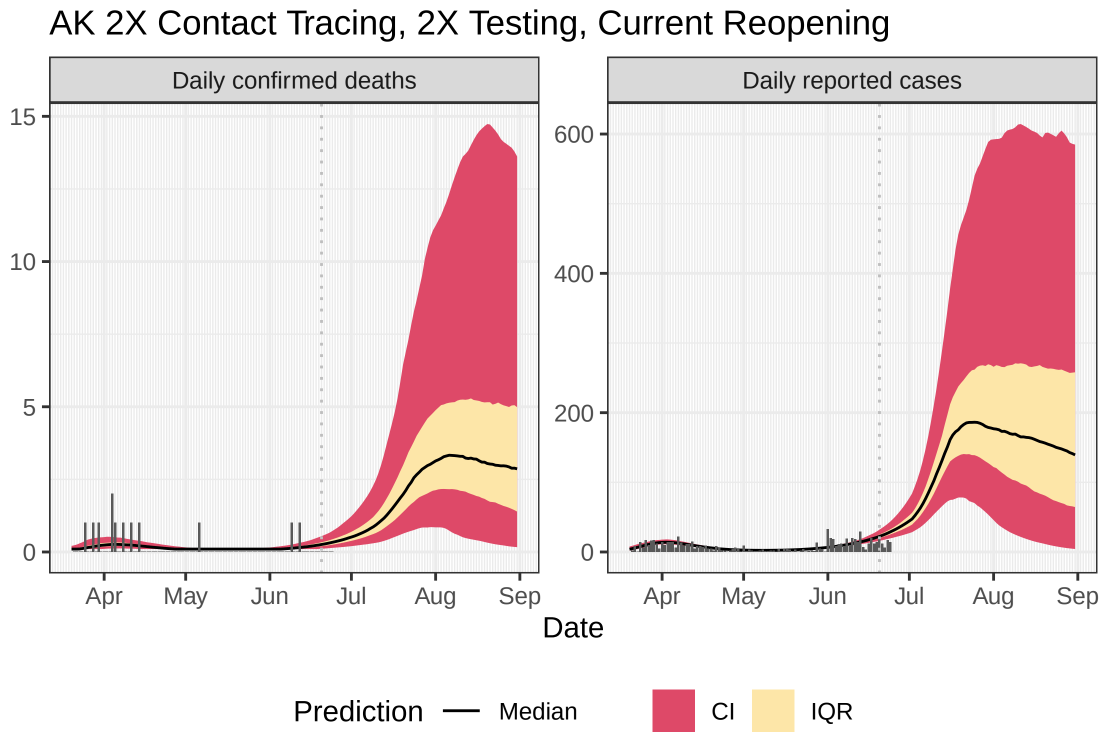
AK 2X Contact Tracing, 2X Testing, Current Reopening
Daily confirmed deaths
Daily reported cases
15
10
5
0
600
400
200
0
Apr
May
Jun
Jul
Aug
Sep
Date
Prediction
Median
CI
IQR

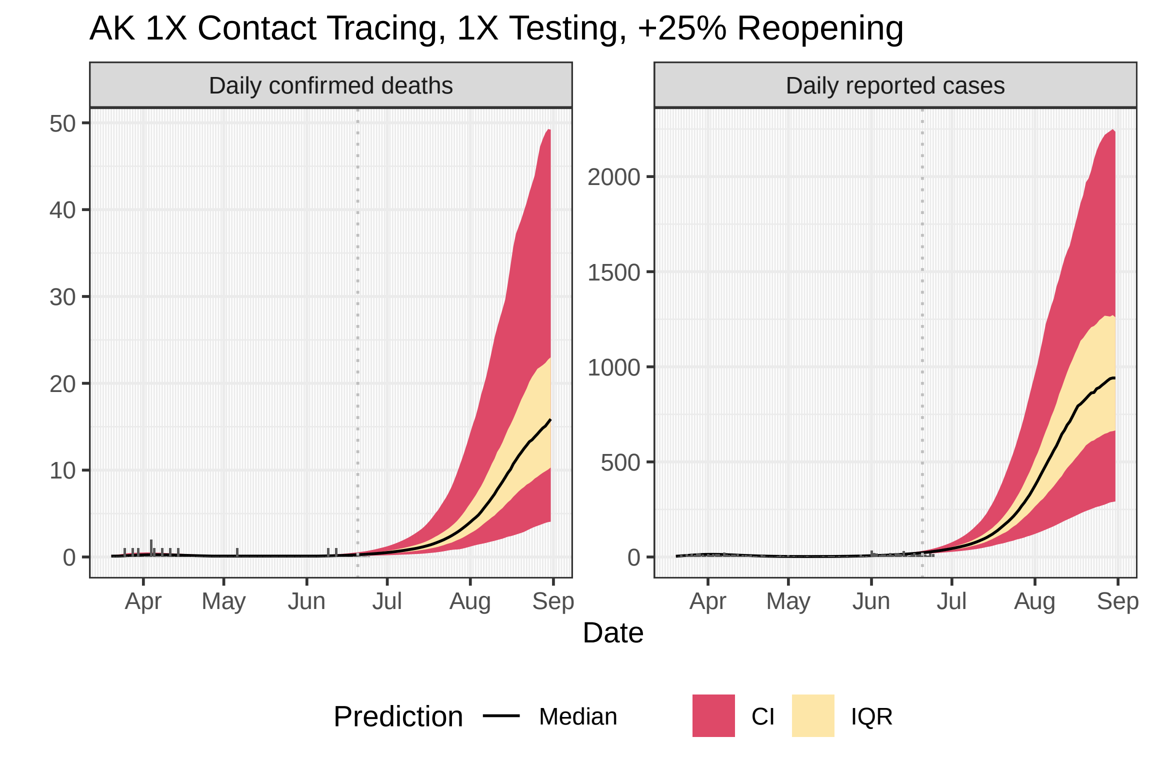

AK 1X Contact Tracing, 1X Testing, +25% Reopening
Daily confirmed deaths
Daily reported cases
50
40
30
20
10
0
2000
1500
1000
500
0
Apr
May
Jun
Jul
Aug
Sep
Date
Prediction
Median
CI
IQR

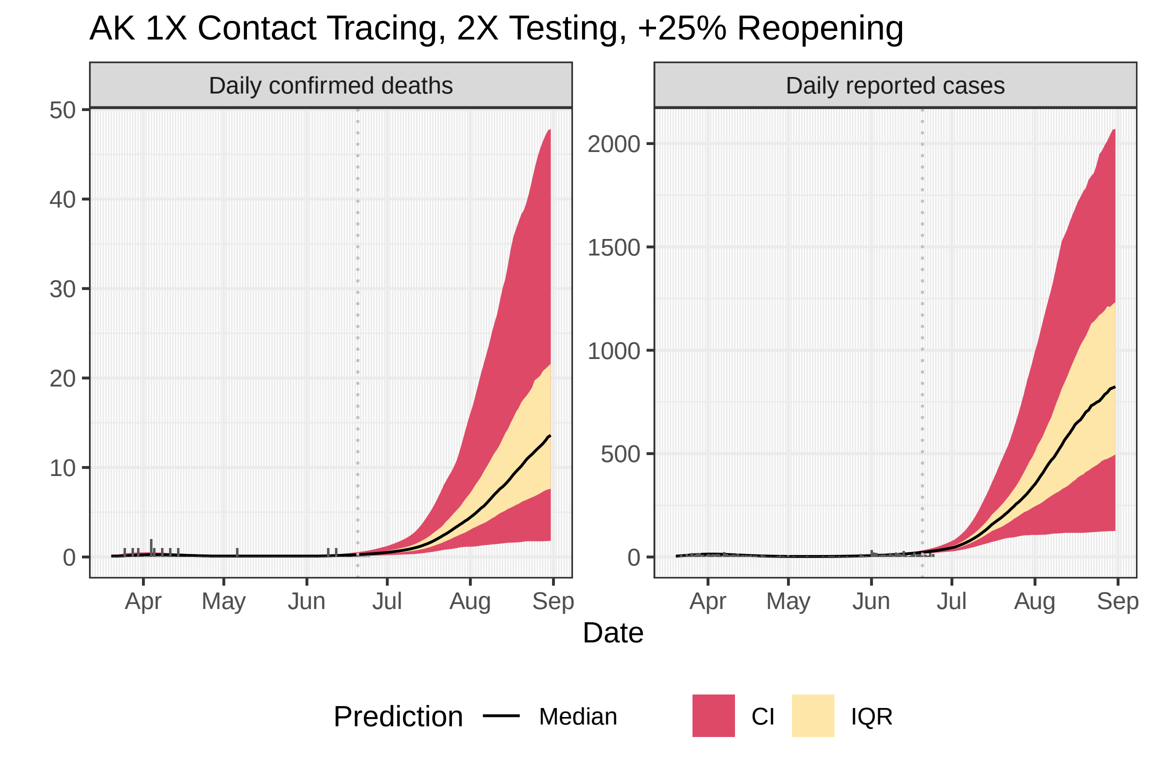

AK 1X Contact Tracing, 2X Testing, +25% Reopening
Daily confirmed deaths
Daily reported cases
50
40
30
20
10
0
2000
1500
1000
500
0
Apr
May
Jun
Jul
Aug
Sep
Date
Prediction
Median
CI
IQR

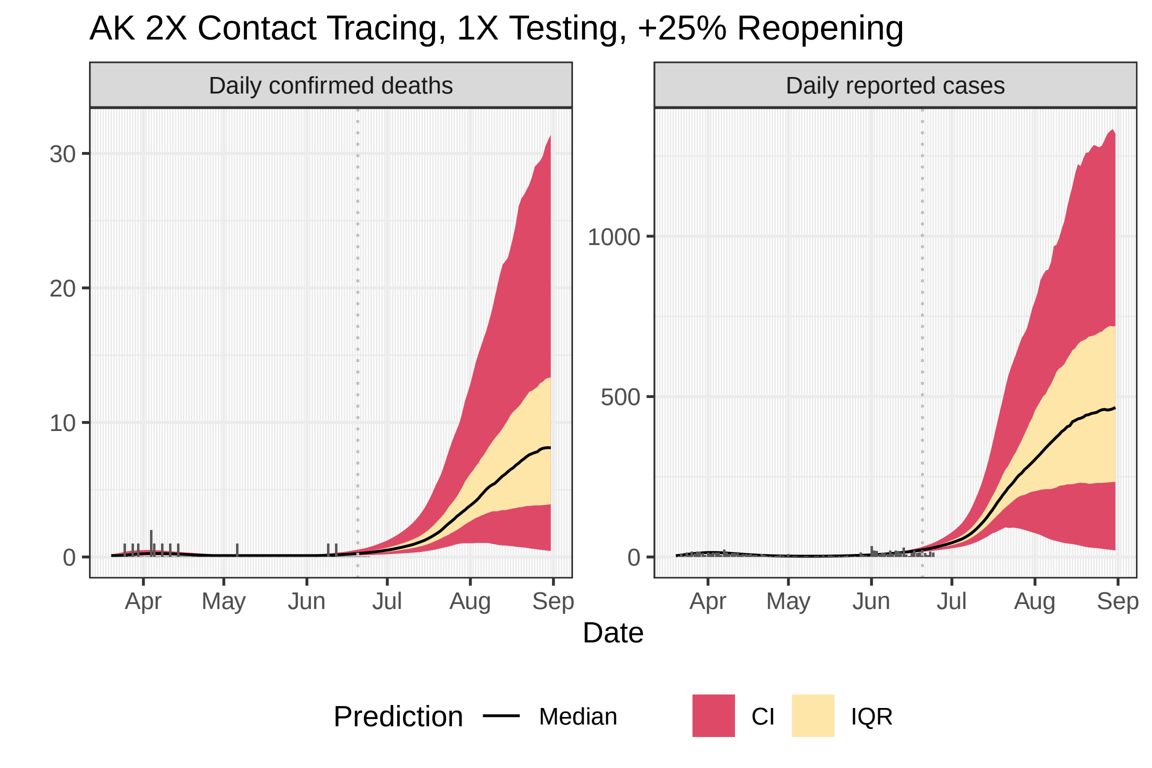
AK 2X Contact Tracing, 1X Testing, +25% Reopening
Daily confirmed deaths
Daily reported cases
30
20
10
0
1000
500
0
Apr
May
Jun
Jul
Aug
Sep
Date
Prediction
Median
CI
IQR

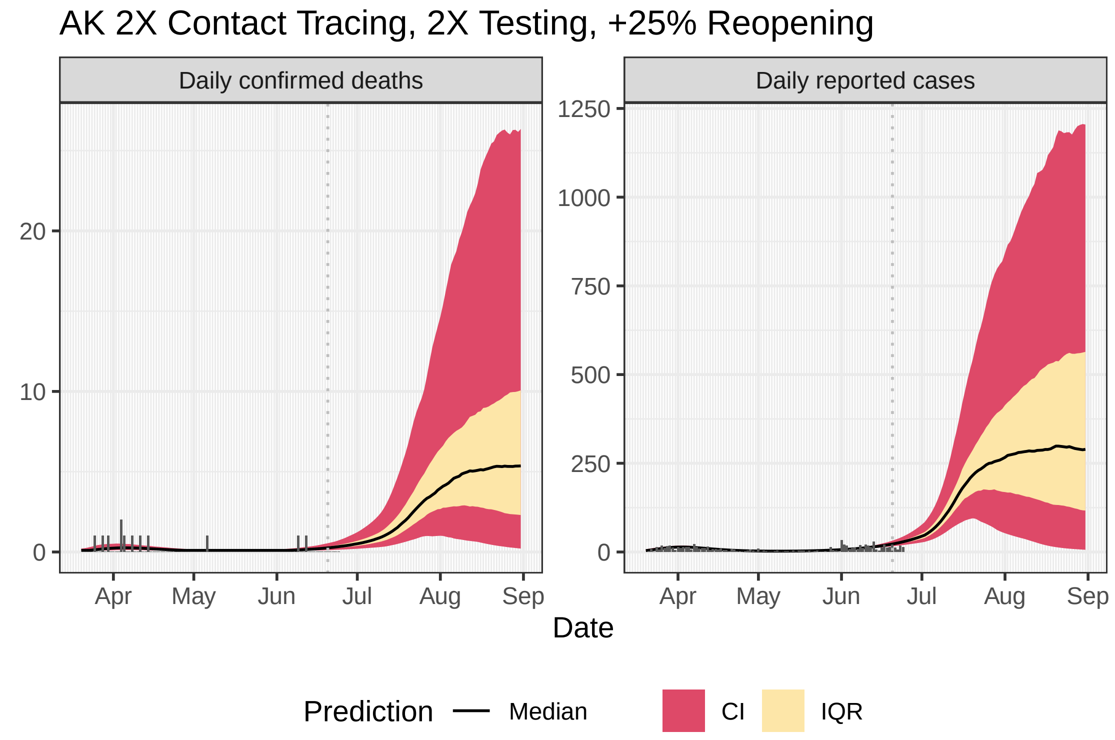

AK 2X Contact Tracing, 2X Testing, +25% Reopening
Daily confirmed deaths
Daily reported cases
0
10
20
0
250
500
750
1000
1250
Apr
May
Jun
Jul
Aug
Sep
Date
Prediction
Median
CI
IQR

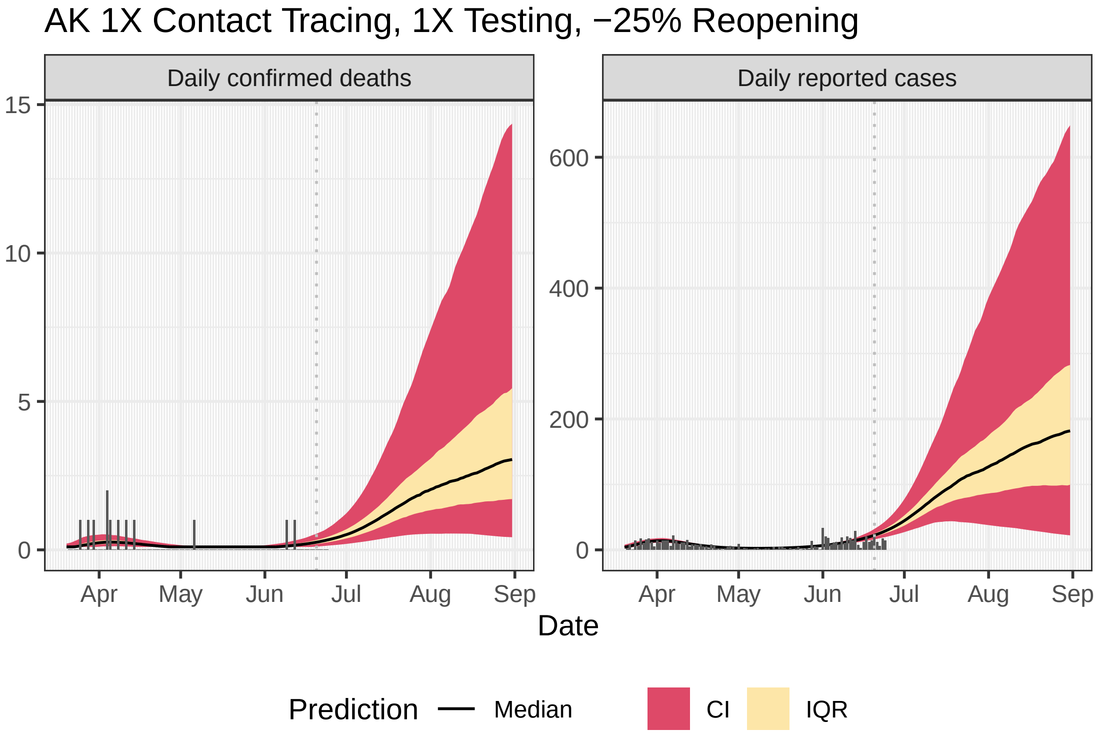
AK 1X Contact Tracing, 1X Testing, −25% Reopening
Daily confirmed deaths
Daily reported cases
0
5
10
15
0
200
400
600
Apr
May
Jun
Jul
Aug
Sep
Date
Prediction
Median
CI
IQR

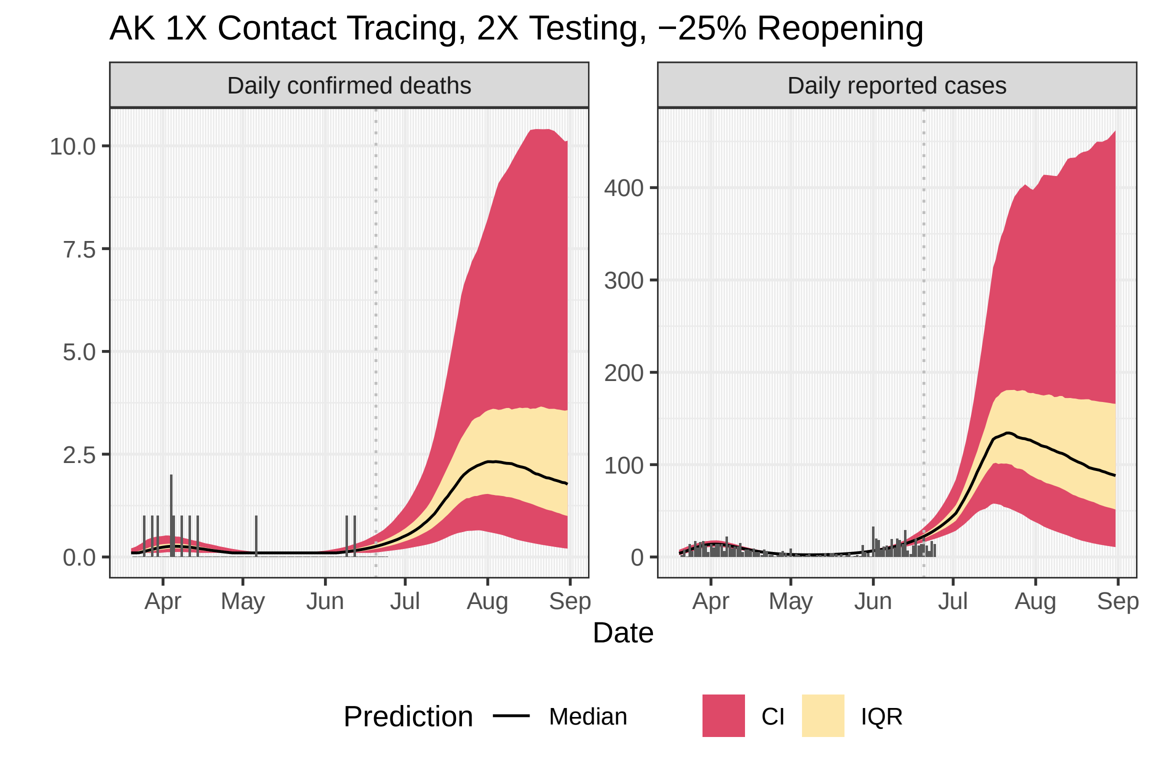

AK 1X Contact Tracing, 2X Testing, −25% Reopening
Daily confirmed deaths
Daily reported cases
10.0
7.5
5.0
2.5
0.0
400
300
200
100
0
Apr
May
Jun
Jul
Aug
Sep
Date
Prediction
Median
CI
IQR

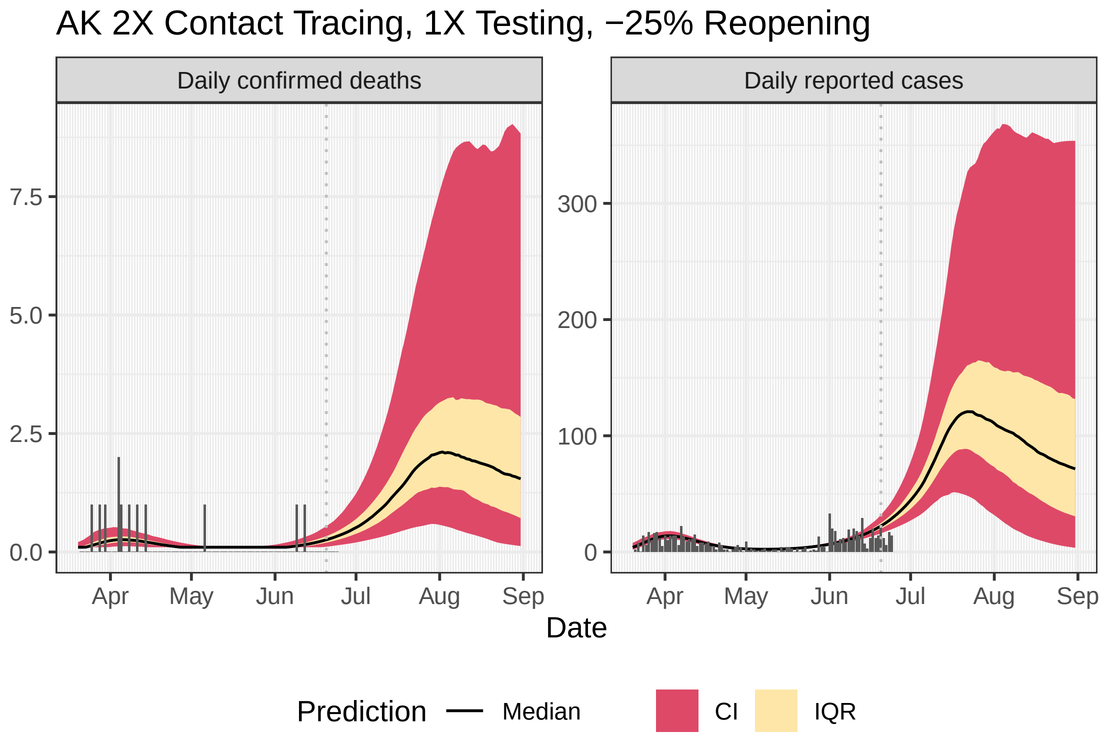

AK 2X Contact Tracing, 1X Testing, −25% Reopening
Daily confirmed deaths
Daily reported cases
7.5
5.0
2.5
0.0
300
200
100
0
Apr
May
Jun
Jul
Aug
Sep
Date
Prediction
Median
CI
IQR

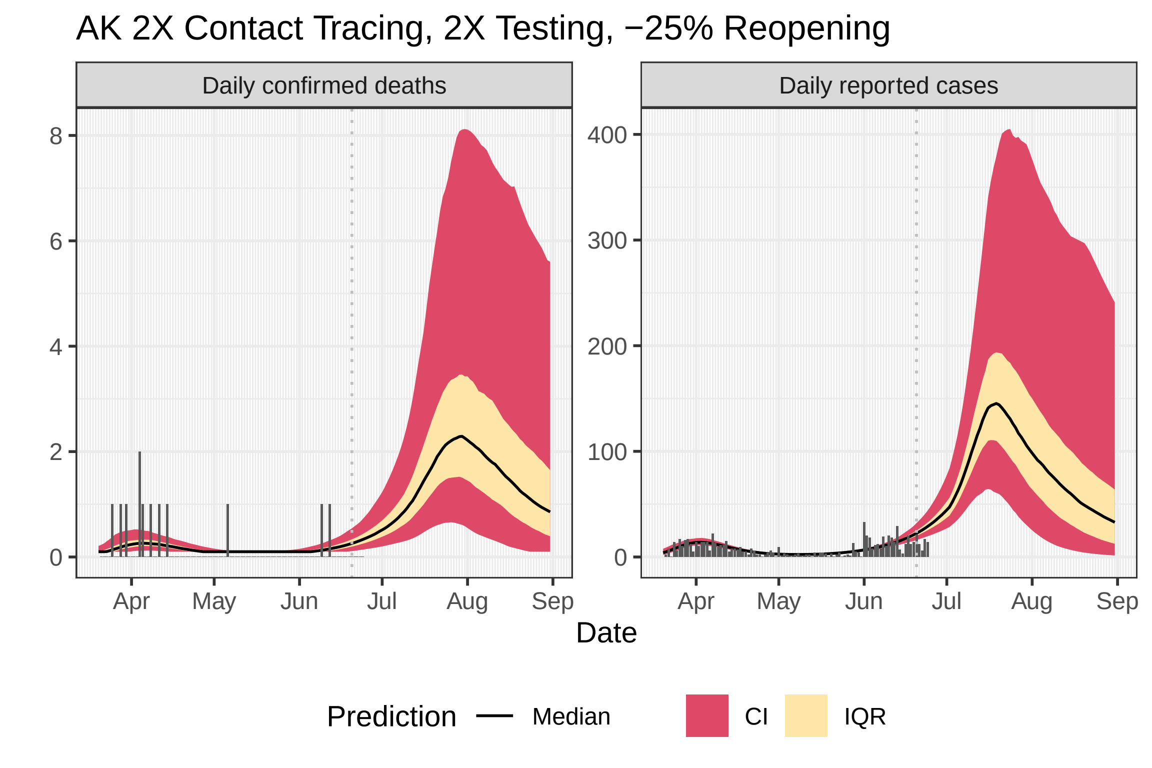

AK 2X Contact Tracing, 2X Testing, −25% Reopening
Daily confirmed deaths
Daily reported cases
0
2
4
6
8
0
100
200
300
400
Apr
May
Jun
Jul
Aug
Sep
Date
Prediction
Median
CI
IQR

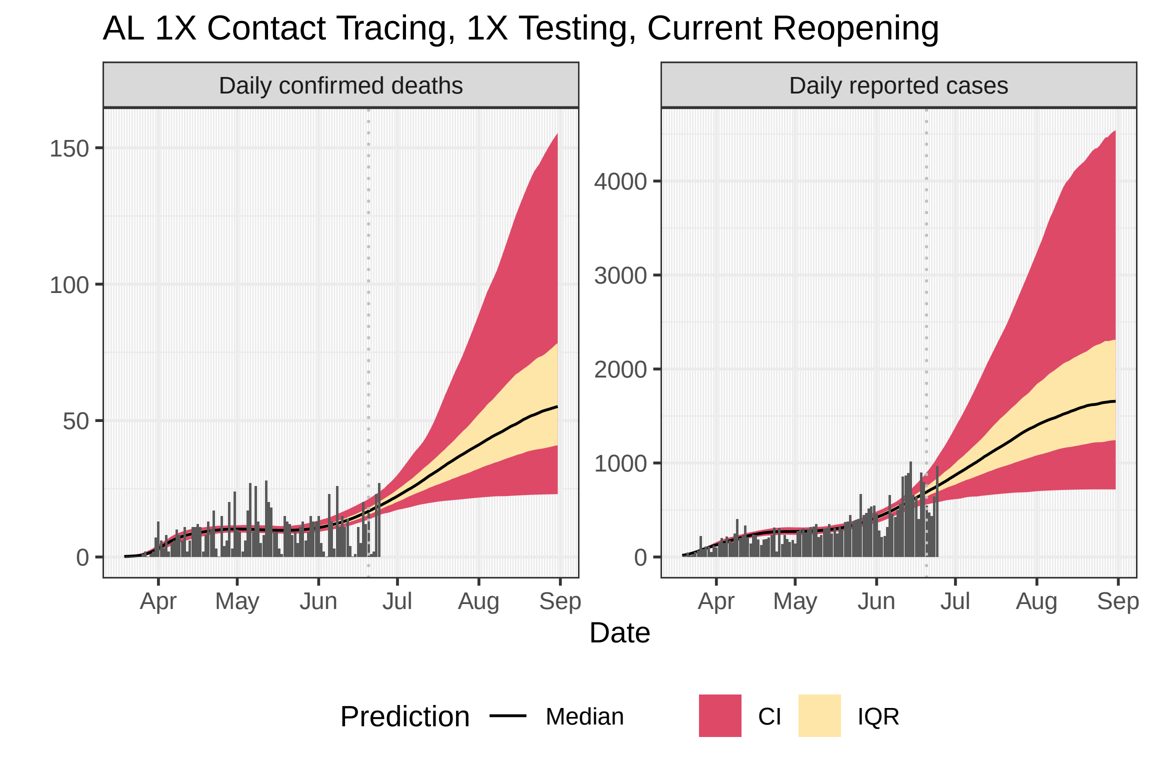
AL 1X Contact Tracing, 1X Testing, Current Reopening
Daily confirmed deaths
Daily reported cases
150
100
50
0
4000
3000
2000
1000
0
Apr
May
Jun
Jul
Aug
Sep
Date
Prediction
Median
CI
IQR

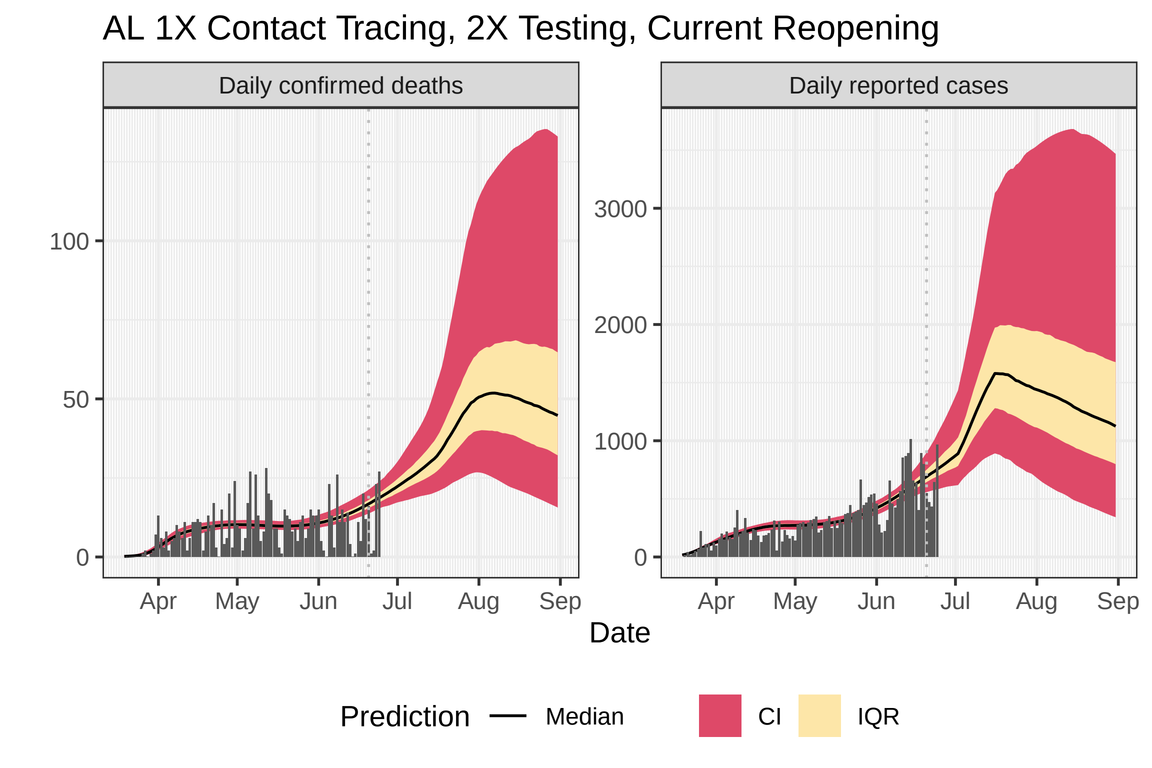

AL 1X Contact Tracing, 2X Testing, Current Reopening
Daily confirmed deaths
Daily reported cases
100
50
0
3000
2000
1000
0
Apr
May
Jun
Jul
Aug
Sep
Date
Prediction
Median
CI
IQR

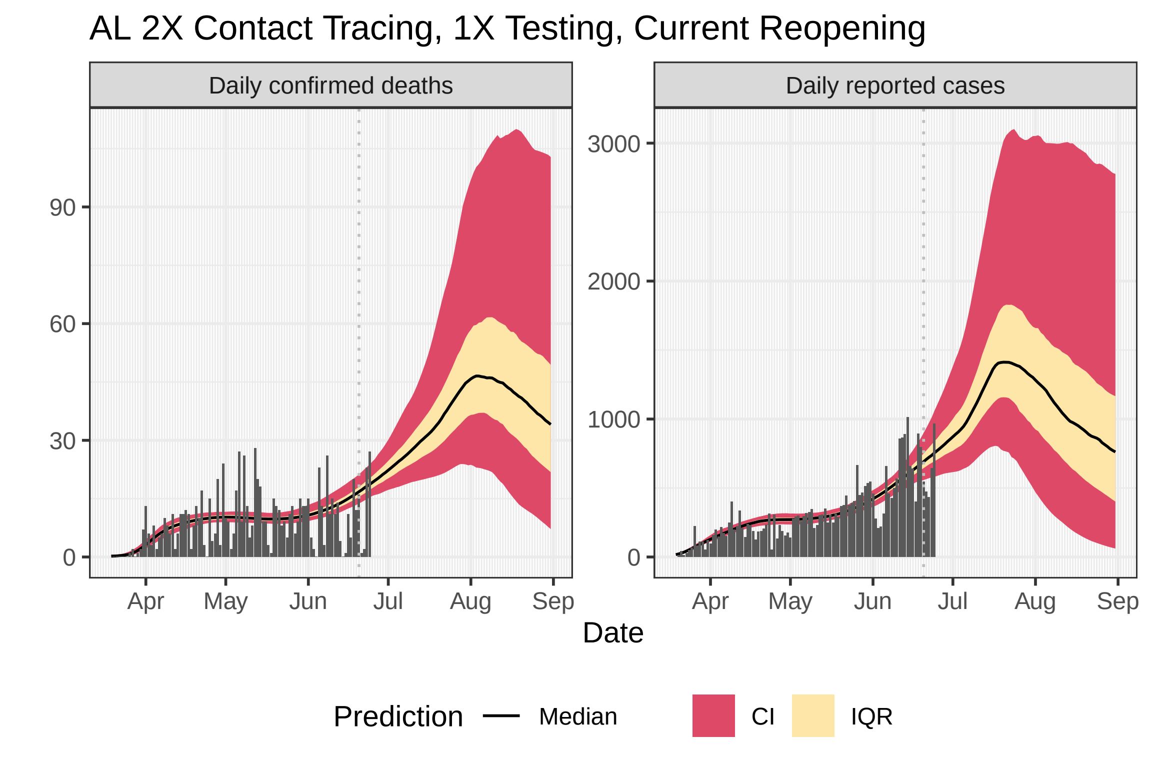

AL 2X Contact Tracing, 1X Testing, Current Reopening
Daily confirmed deaths
Daily reported cases
90
60
30
0
3000
2000
1000
0
Apr
May
Jun
Jul
Aug
Sep
Date
Prediction
Median
CI
IQR

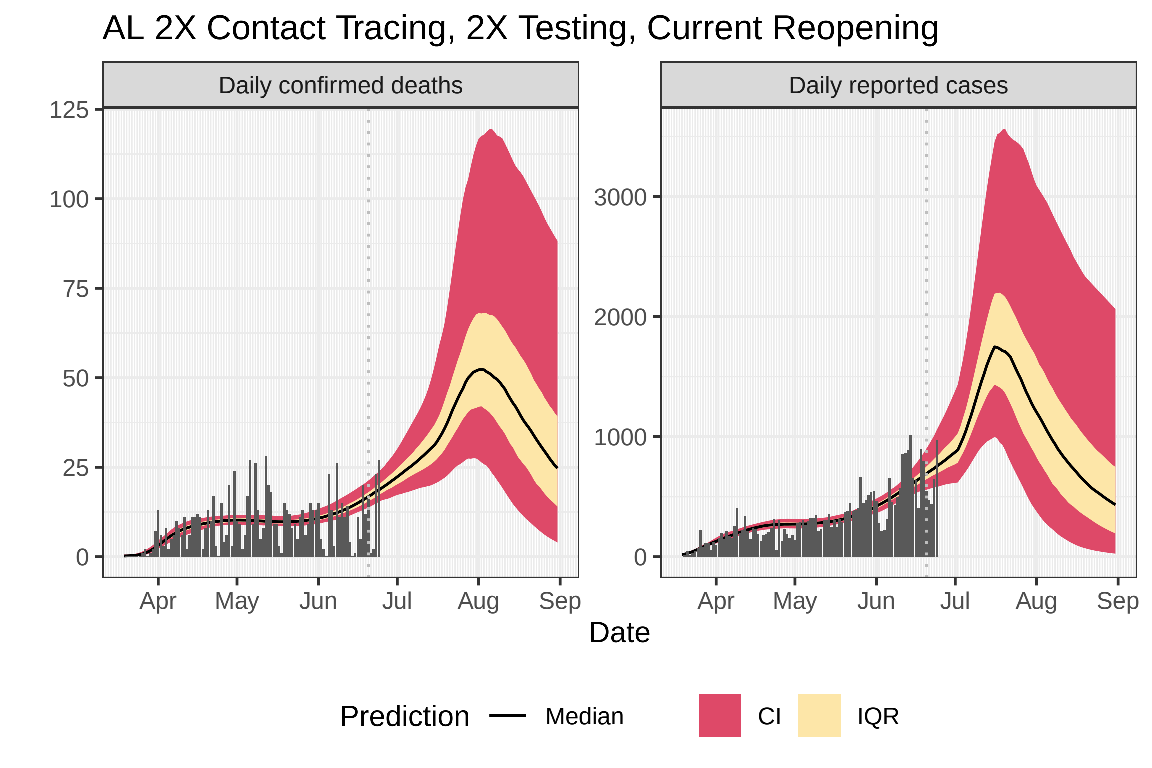

AL 2X Contact Tracing, 2X Testing, Current Reopening
Daily confirmed deaths
Daily reported cases
125
100
75
50
25
0
3000
2000
1000
0
Apr
May
Jun
Jul
Aug
Sep
Date
Prediction
Median
CI
IQR

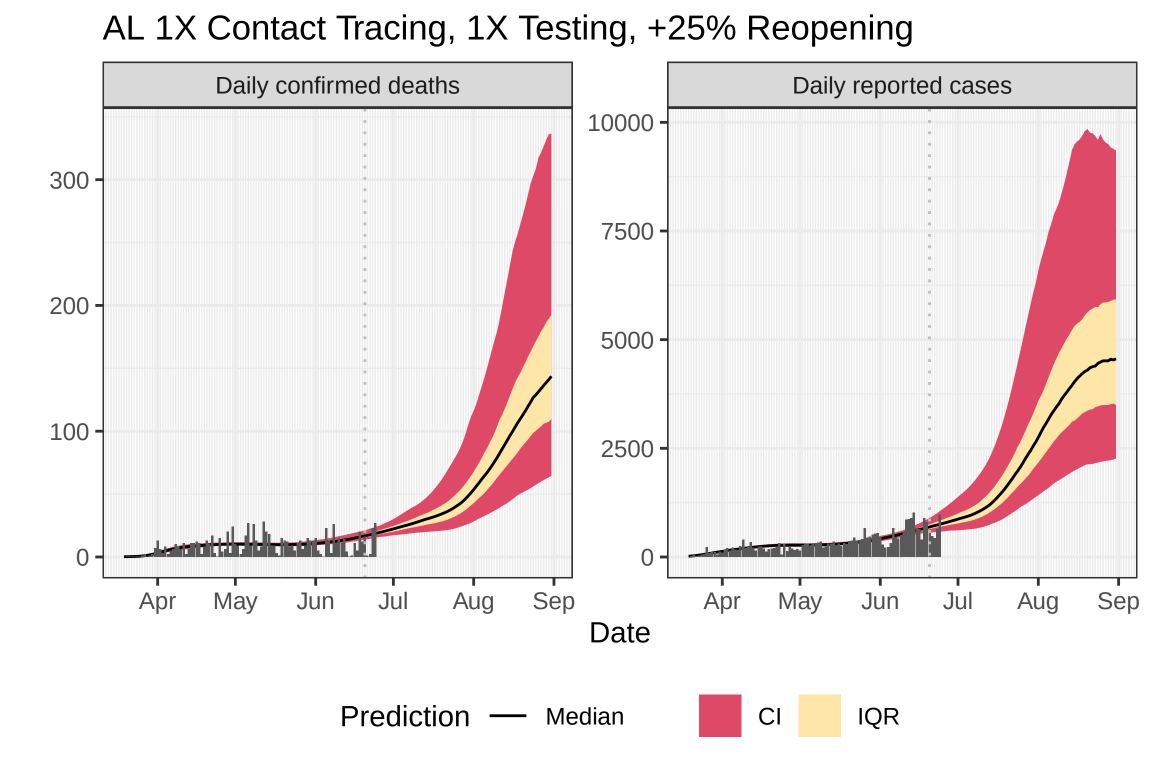
AL 1X Contact Tracing, 1X Testing, +25% Reopening
Daily confirmed deaths
Daily reported cases
300
200
100
0
10000
7500
5000
2500
0
Apr
May
Jun
Jul
Aug
Sep
Date
Prediction
Median
CI
IQR

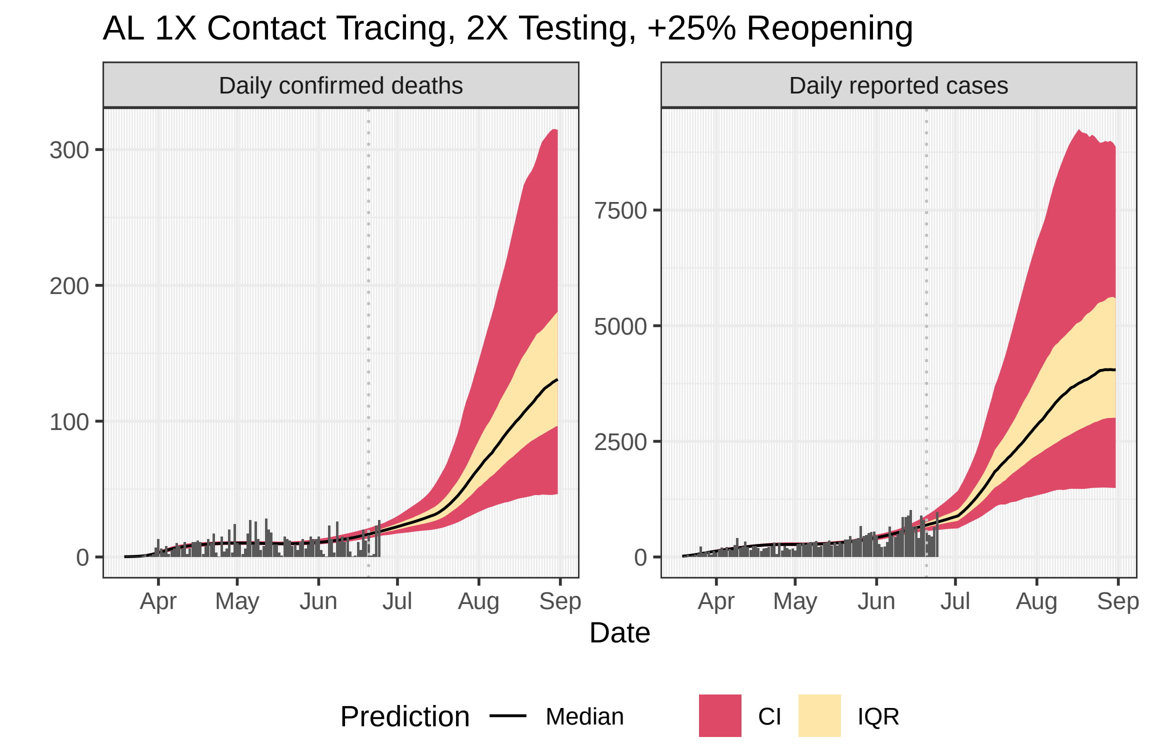
AL 1X Contact Tracing, 2X Testing, +25% Reopening
Daily confirmed deaths
Daily reported cases
300
200
100
0
7500
5000
2500
0
Apr
May
Jun
Jul
Aug
Sep
Date
Prediction
Median
CI
IQR

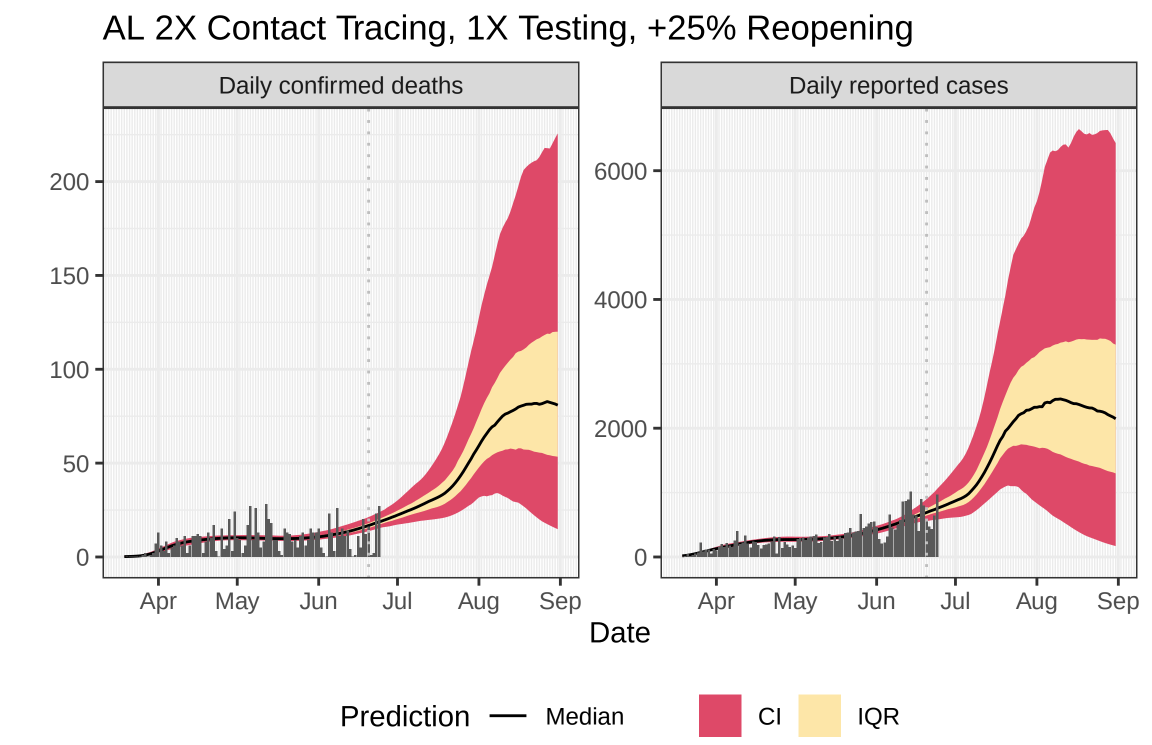

AL 2X Contact Tracing, 1X Testing, +25% Reopening
Daily confirmed deaths
Daily reported cases
0
50
100
150
200
0
2000
4000
6000
Apr
May
Jun
Jul
Aug
Sep
Date
Prediction
Median
CI
IQR

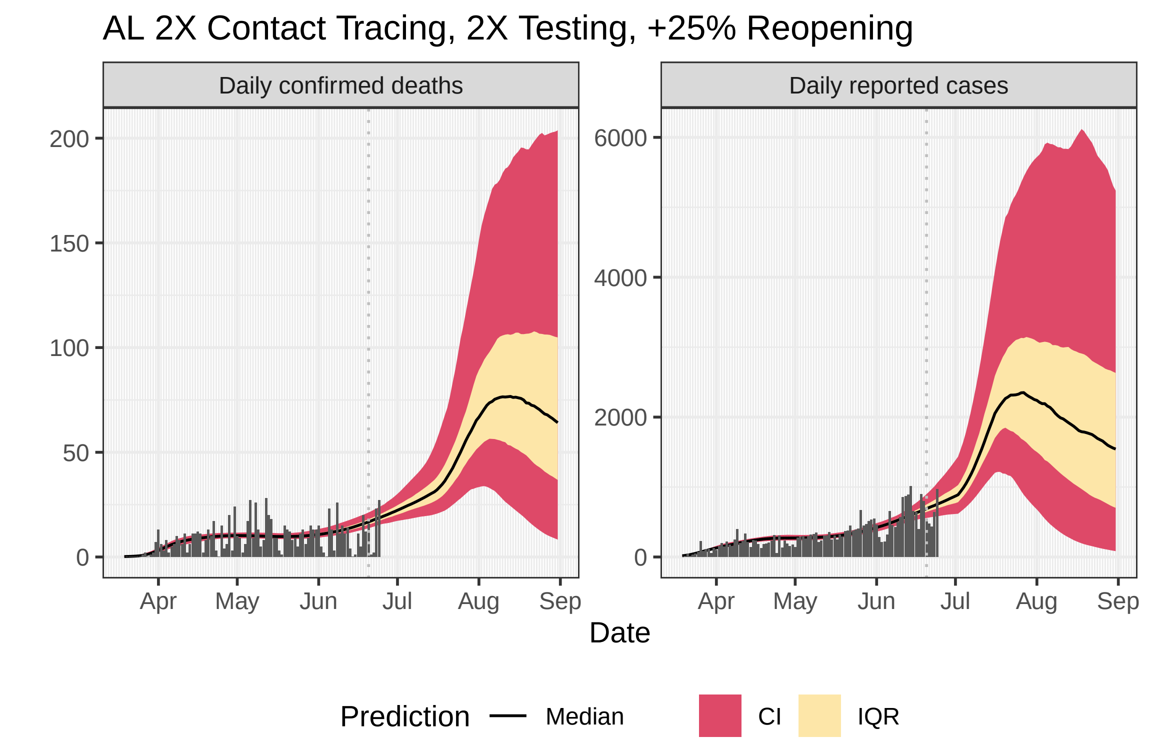
AL 2X Contact Tracing, 2X Testing, +25% Reopening
Daily confirmed deaths
Daily reported cases
200
150
100
50
0
6000
4000
2000
0
Apr
May
Jun
Jul
Aug
Sep
Date
Prediction
Median
CI
IQR

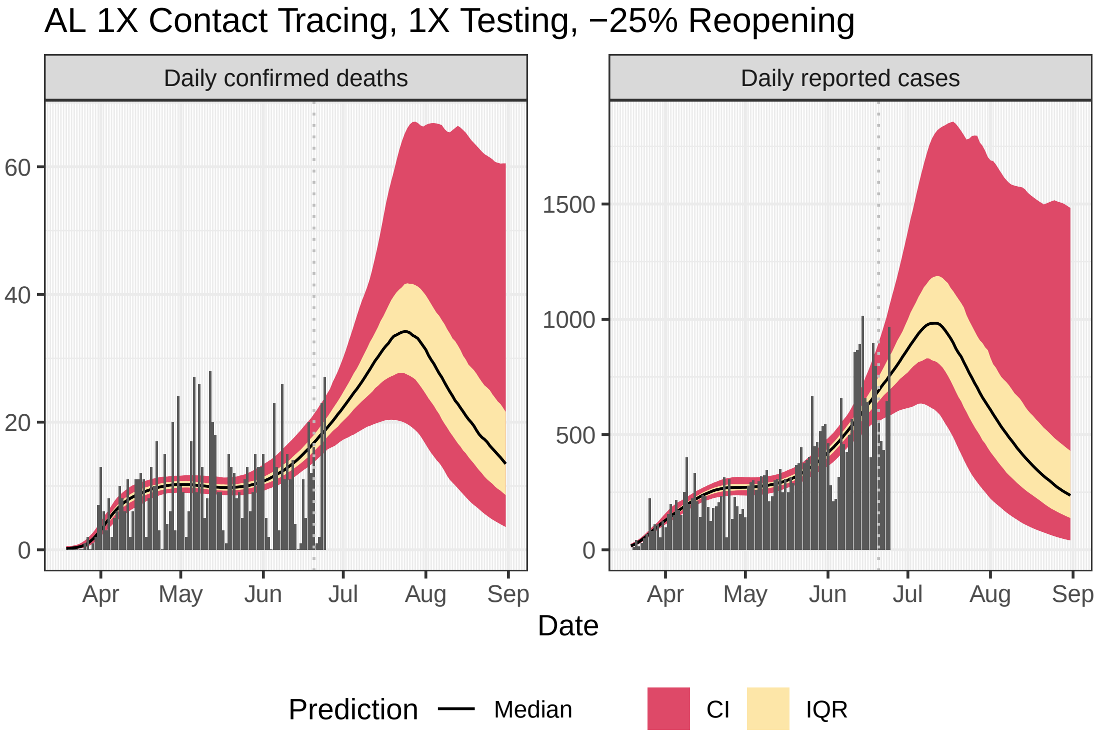
AL 1X Contact Tracing, 1X Testing, −25% Reopening
Daily confirmed deaths
Daily reported cases
60
40
20
0
1500
1000
500
0
Apr
May
Jun
Jul
Aug
Sep
Date
Prediction
Median
CI
IQR

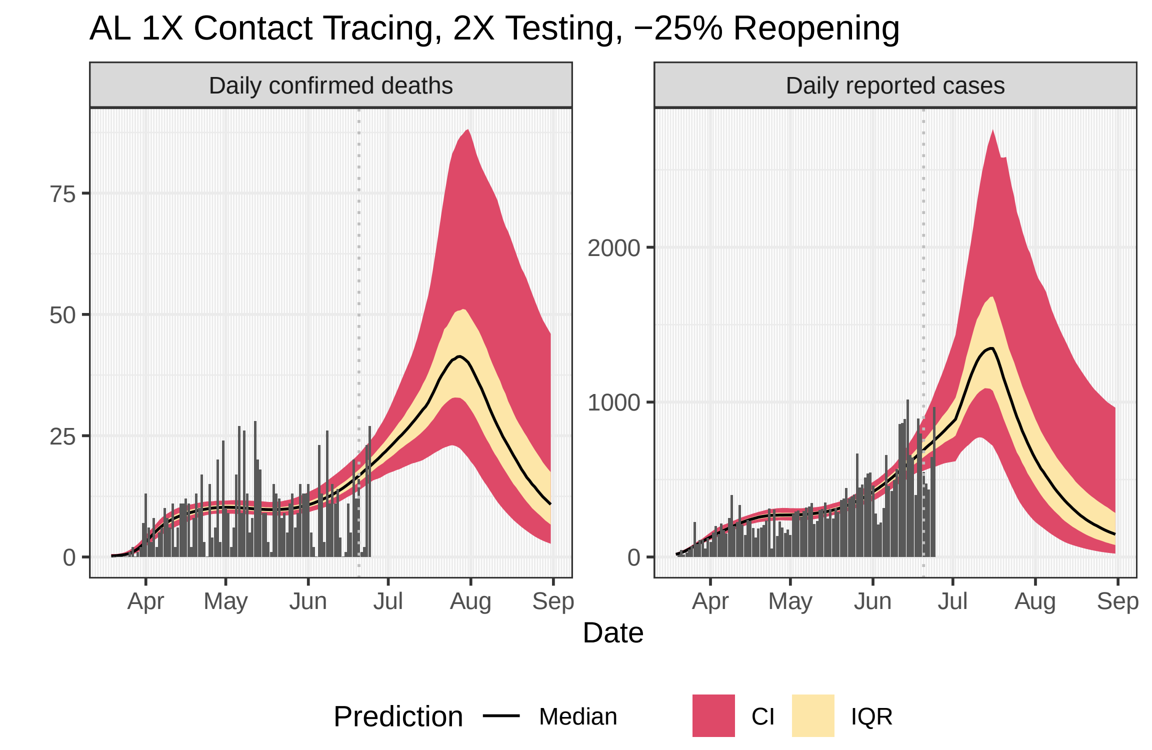

AL 1X Contact Tracing, 2X Testing, −25% Reopening
Daily confirmed deaths
Daily reported cases
0
25
50
75
0
1000
2000
Apr
May
Jun
Jul
Aug
Sep
Date
Prediction
Median
CI
IQR

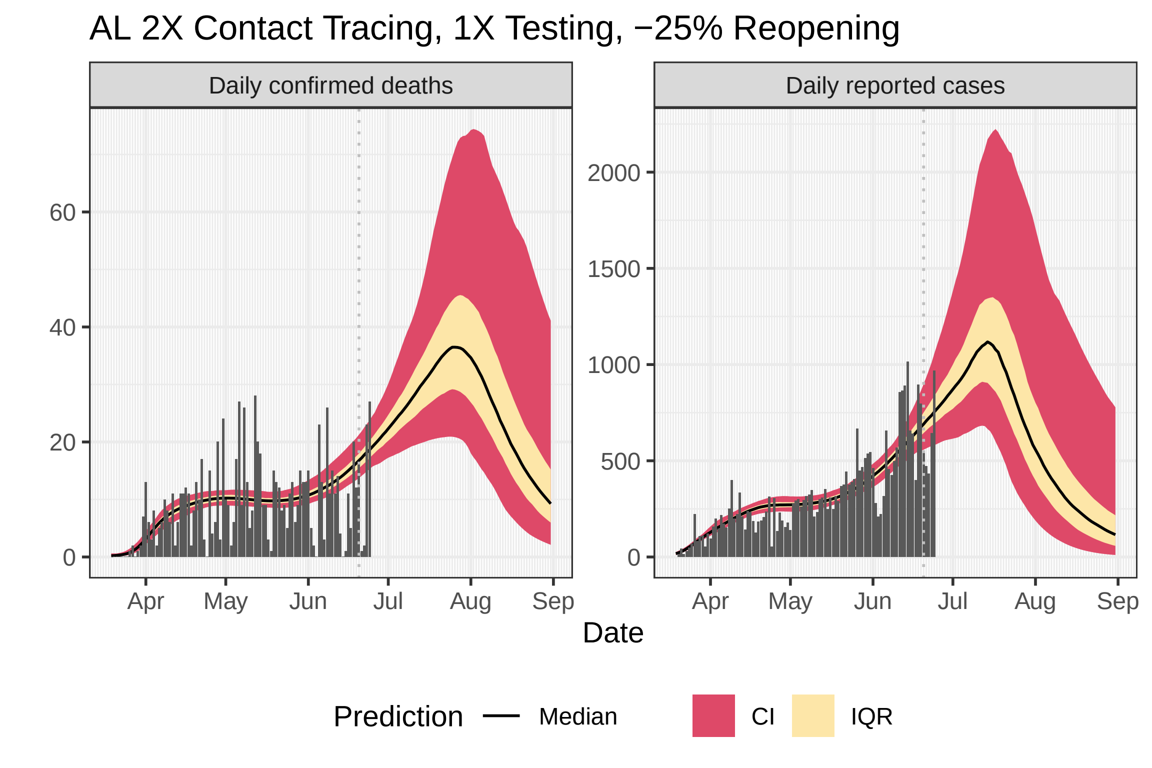

AL 2X Contact Tracing, 1X Testing, −25% Reopening
Daily confirmed deaths
Daily reported cases
0
20
40
60
0
500
1000
1500
2000
Apr
May
Jun
Jul
Aug
Sep
Date
Prediction
Median
CI
IQR

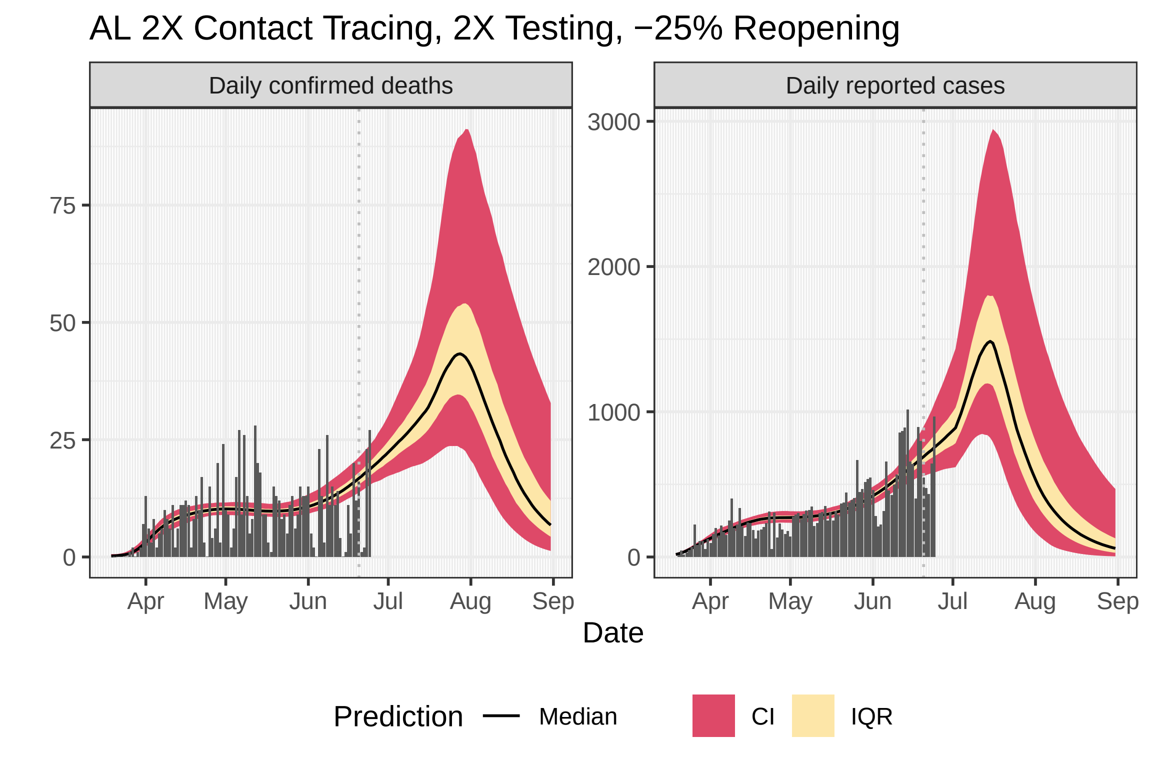
AL 2X Contact Tracing, 2X Testing, −25% Reopening
Daily confirmed deaths
Daily reported cases
0
25
50
75
0
1000
2000
3000
Apr
May
Jun
Jul
Aug
Sep
Date
Prediction
Median
CI
IQR

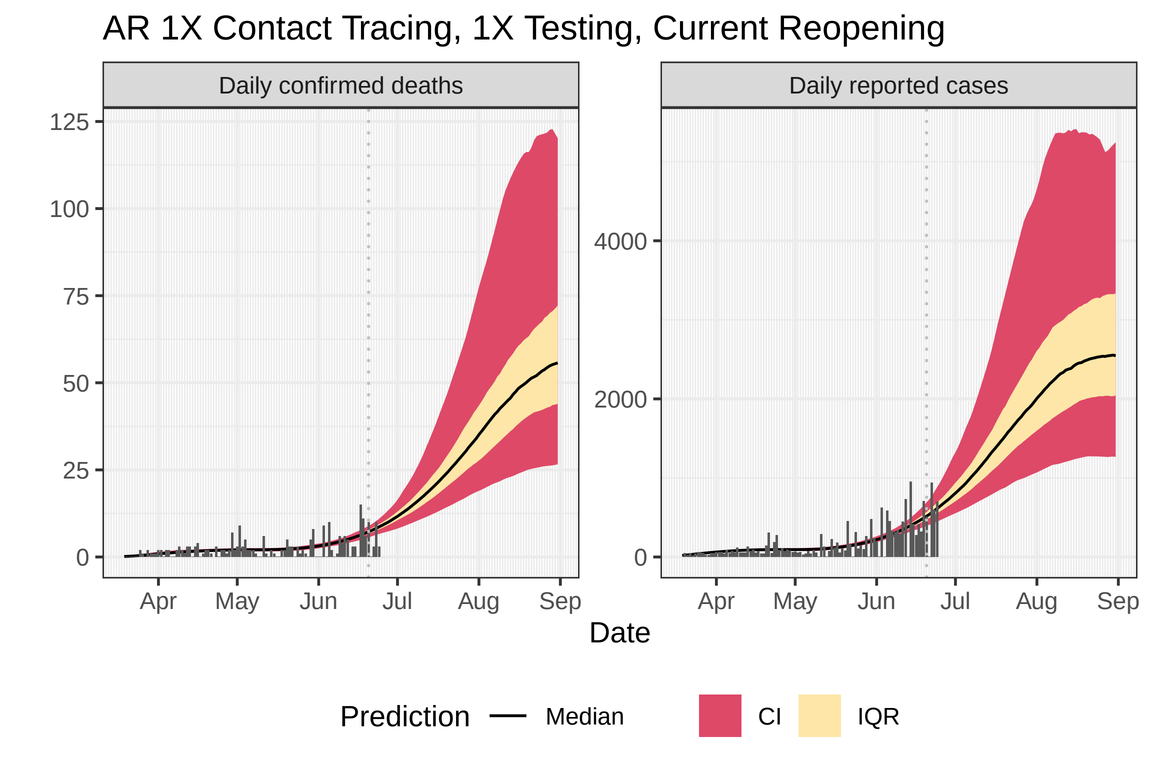
AR 1X Contact Tracing, 1X Testing, Current Reopening
Daily confirmed deaths
Daily reported cases
125
100
75
50
25
0
4000
2000
0
Apr
May
Jun
Jul
Aug
Sep
Date
Prediction
Median
CI
IQR

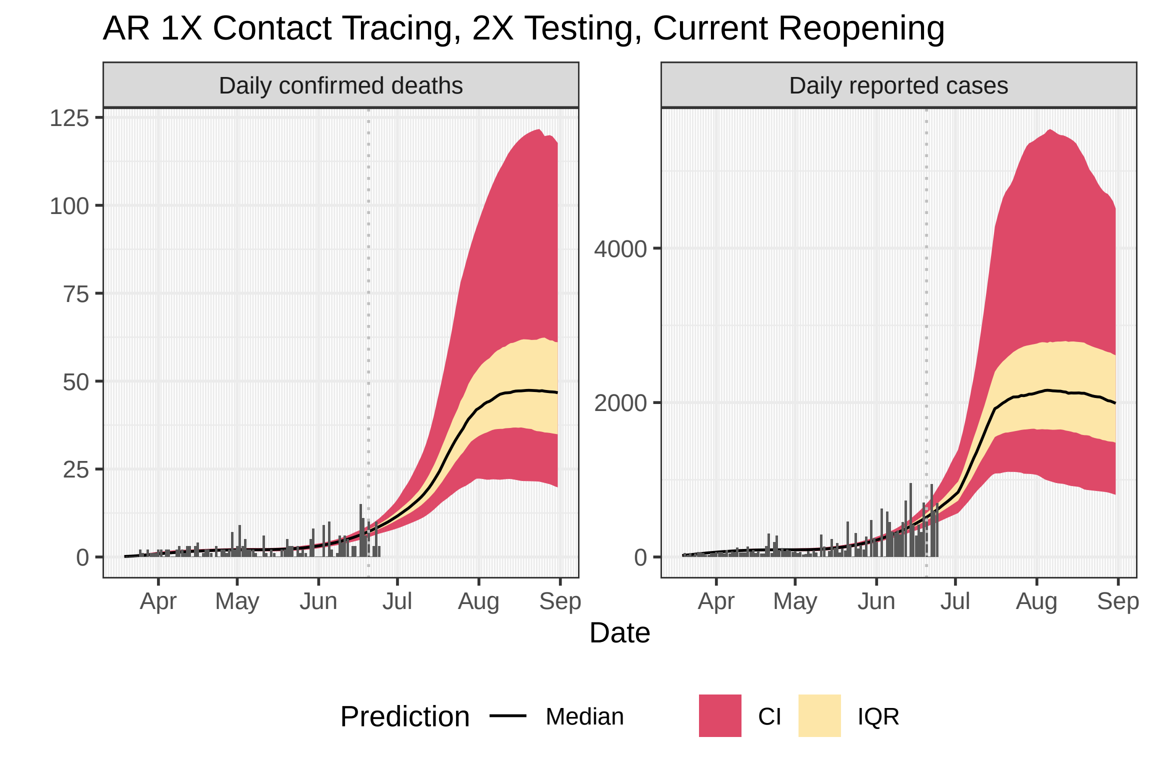

AR 1X Contact Tracing, 2X Testing, Current Reopening
Daily confirmed deaths
Daily reported cases
125
100
75
50
25
0
4000
2000
0
Apr
May
Jun
Jul
Aug
Sep
Date
Prediction
Median
CI
IQR

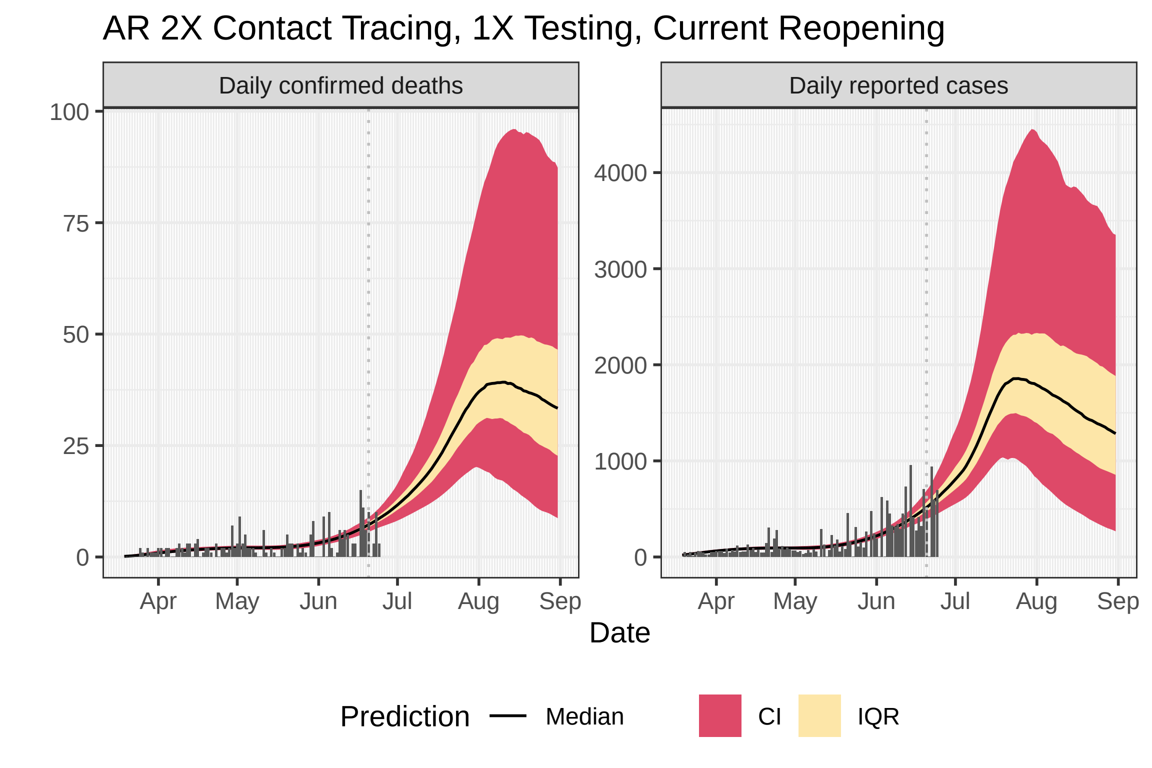

AR 2X Contact Tracing, 1X Testing, Current Reopening
Daily confirmed deaths
Daily reported cases
100
75
50
25
0
4000
3000
2000
1000
0
Apr
May
Jun
Jul
Aug
Sep
Date
Prediction
Median
CI
IQR

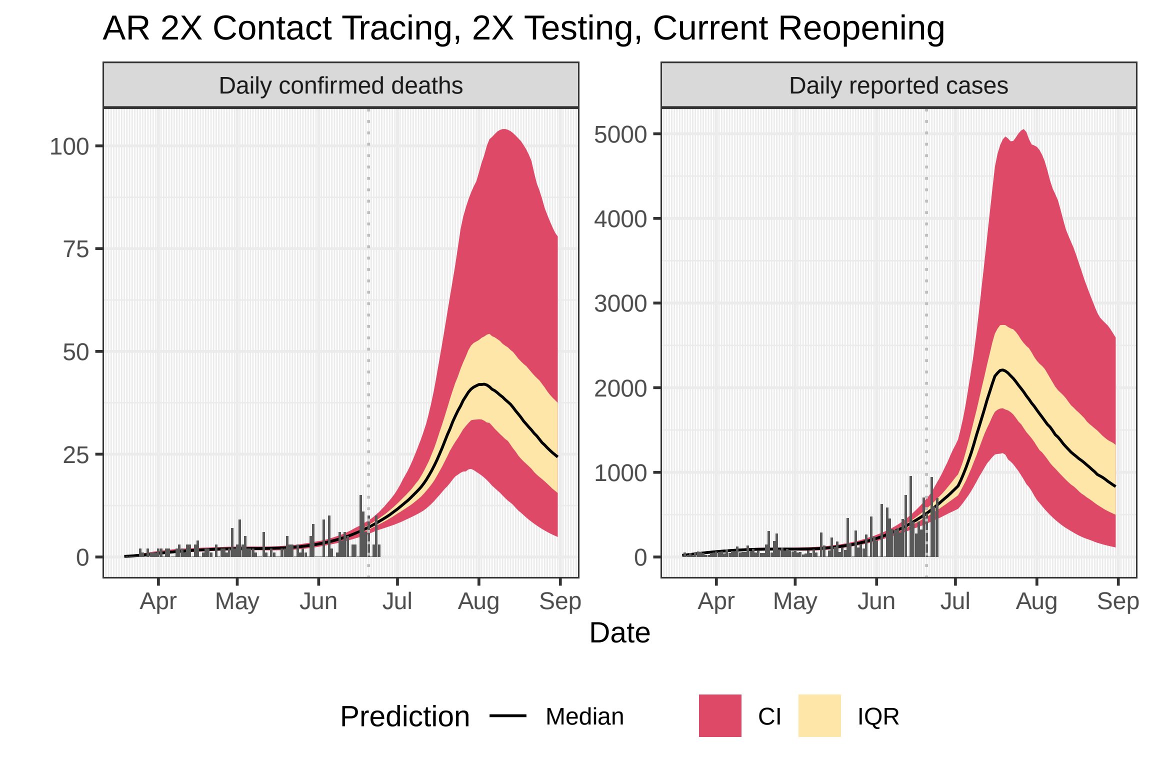

AR 2X Contact Tracing, 2X Testing, Current Reopening
Daily confirmed deaths
Daily reported cases
100
75
50
25
0
5000
4000
3000
2000
1000
0
Apr
May
Jun
Jul
Aug
Sep
Date
Prediction
Median
CI
IQR

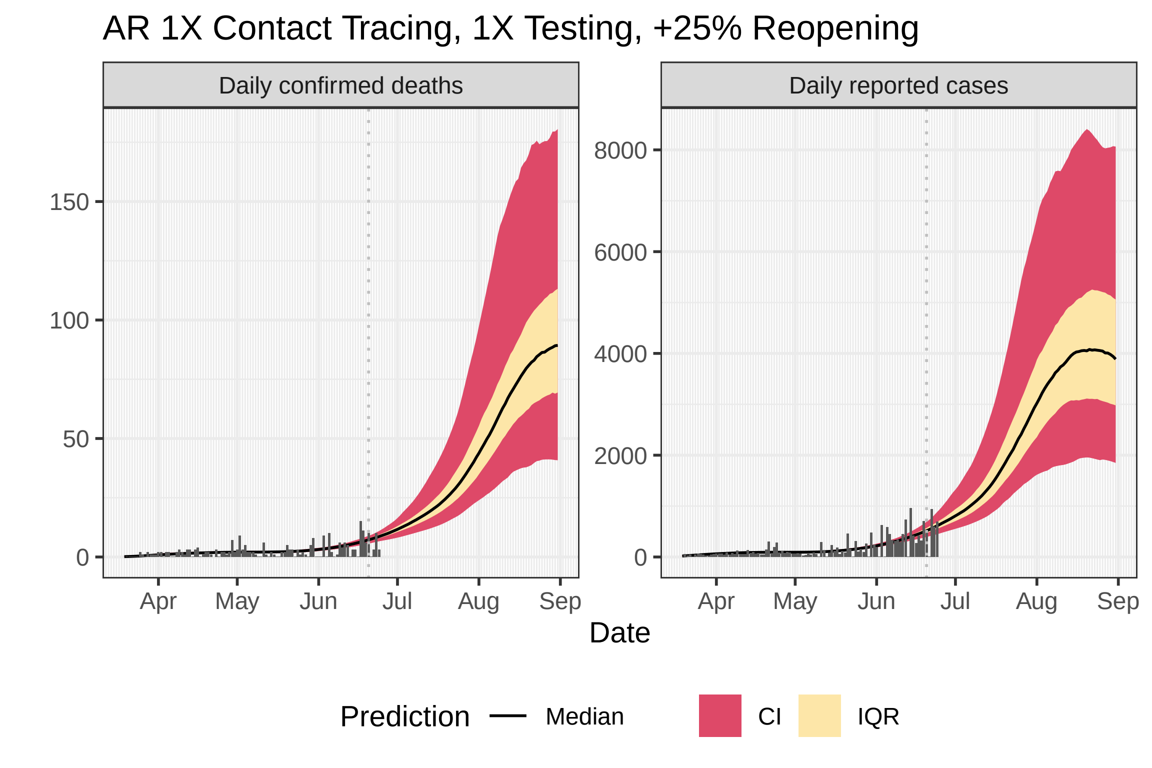
AR 1X Contact Tracing, 1X Testing, +25% Reopening
Daily confirmed deaths
Daily reported cases
0
50
100
150
0
2000
4000
6000
8000
Apr
May
Jun
Jul
Aug
Sep
Date
Prediction
Median
CI
IQR

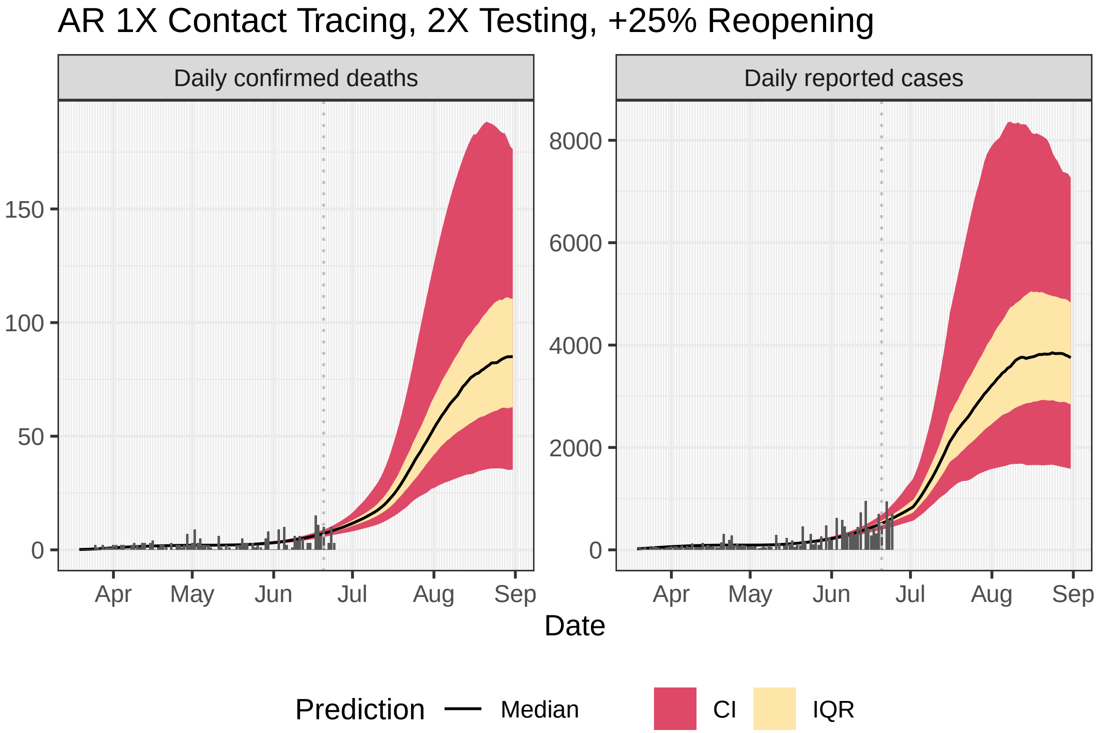
AR 1X Contact Tracing, 2X Testing, +25% Reopening
Daily confirmed deaths
Daily reported cases
0
50
100
150
0
2000
4000
6000
8000
Apr
May
Jun
Jul
Aug
Sep
Date
Prediction
Median
CI
IQR

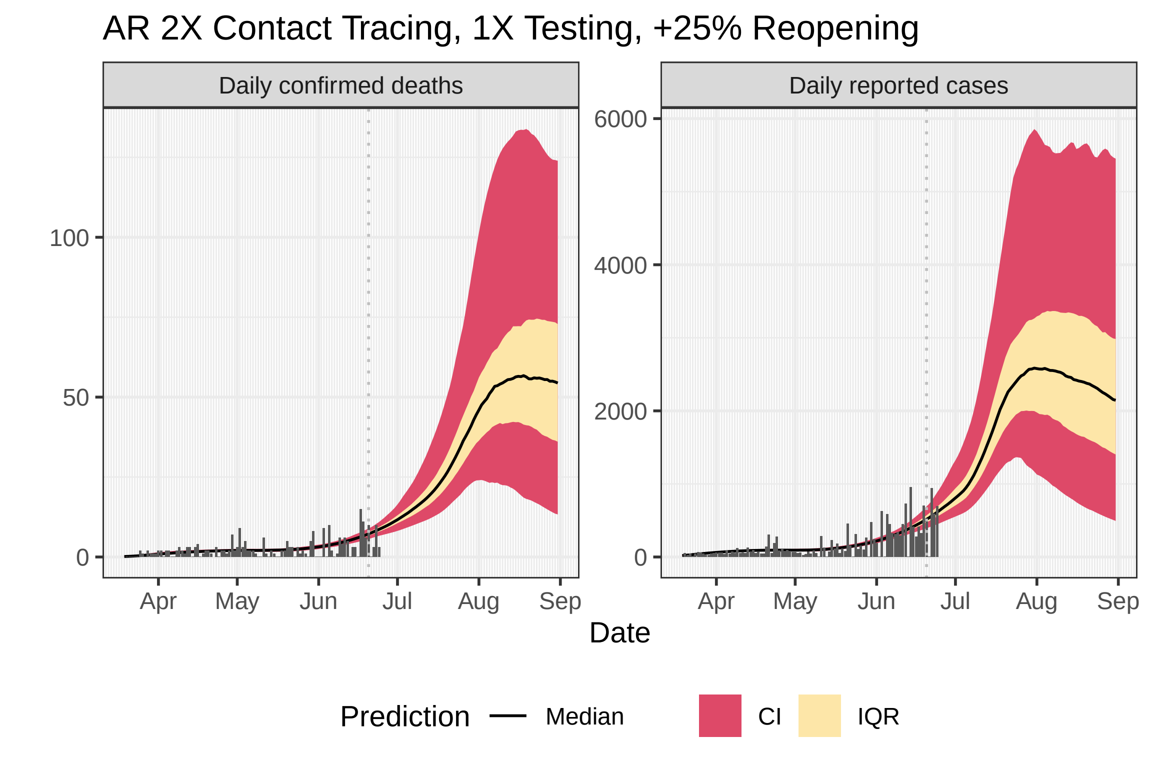

AR 2X Contact Tracing, 1X Testing, +25% Reopening
Daily confirmed deaths
Daily reported cases
100
50
0
6000
4000
2000
0
Apr
May
Jun
Jul
Aug
Sep
Date
Prediction
Median
CI
IQR

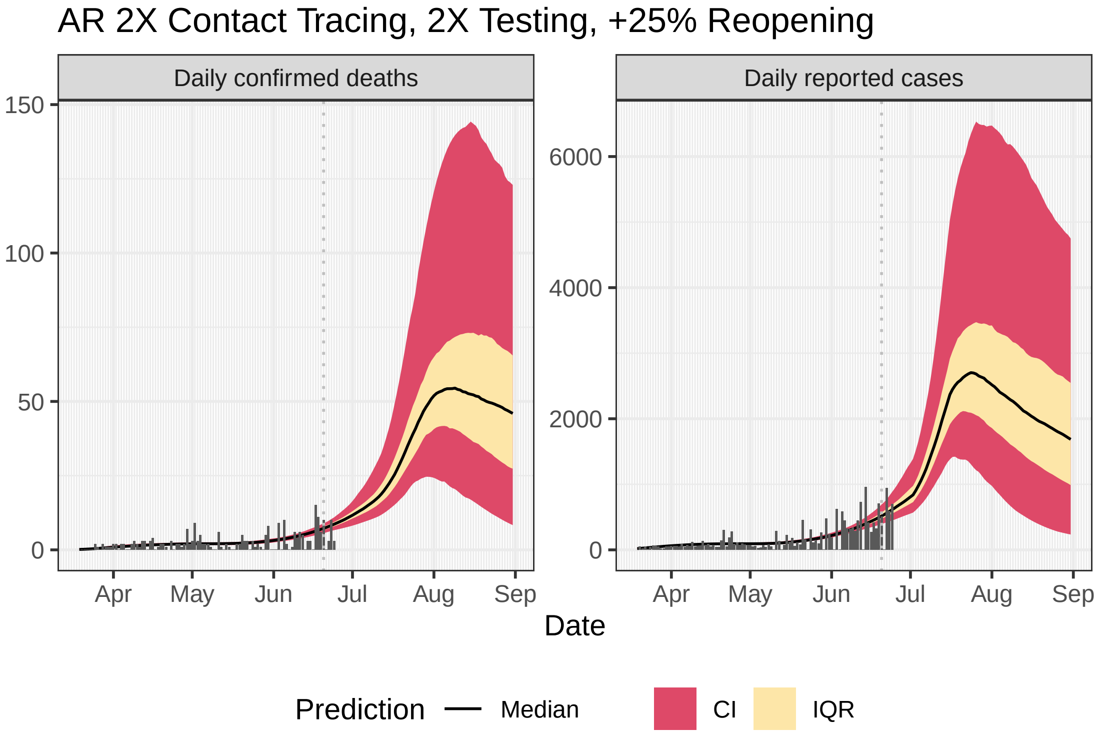
AR 2X Contact Tracing, 2X Testing, +25% Reopening
Daily confirmed deaths
Daily reported cases
150
100
50
0
6000
4000
2000
0
Apr
May
Jun
Jul
Aug
Sep
Date
Prediction
Median
CI
IQR

AR 1X Contact Tracing, 1X Testing, −25% Reopening

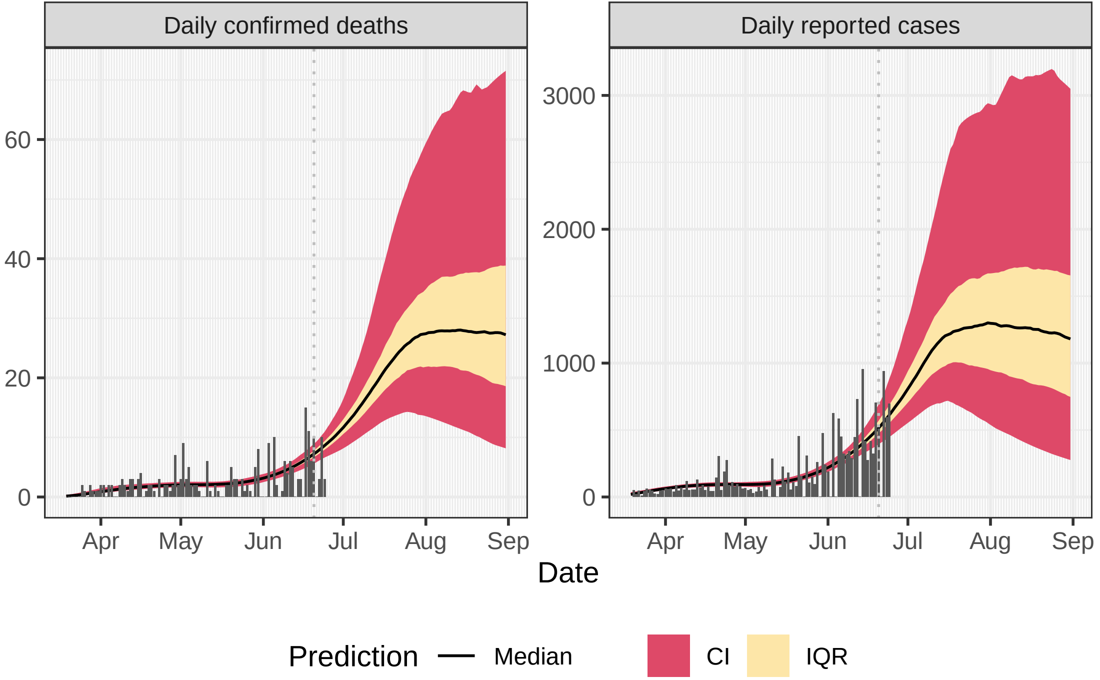

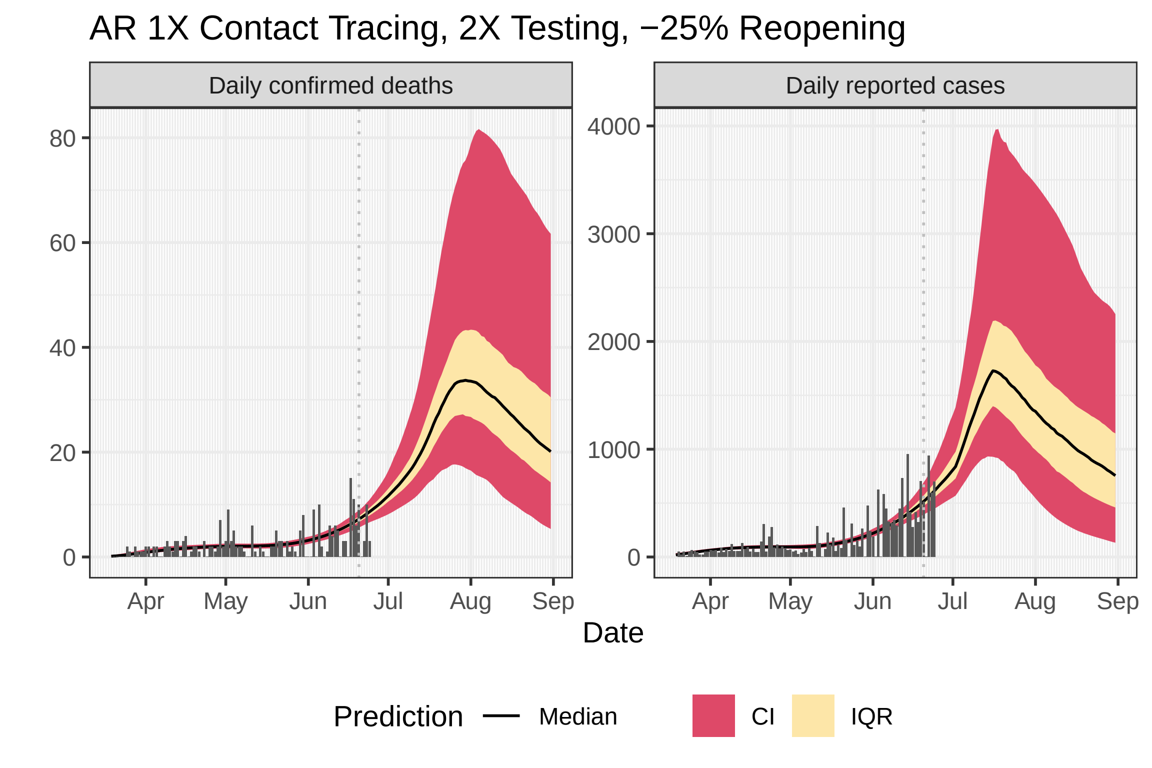

AR 1X Contact Tracing, 2X Testing, −25% Reopening
Daily confirmed deaths
Daily reported cases
80
60
40
20
0
4000
3000
2000
1000
0
Apr
May
Jun
Jul
Aug
Sep
Date
Prediction
Median
CI
IQR

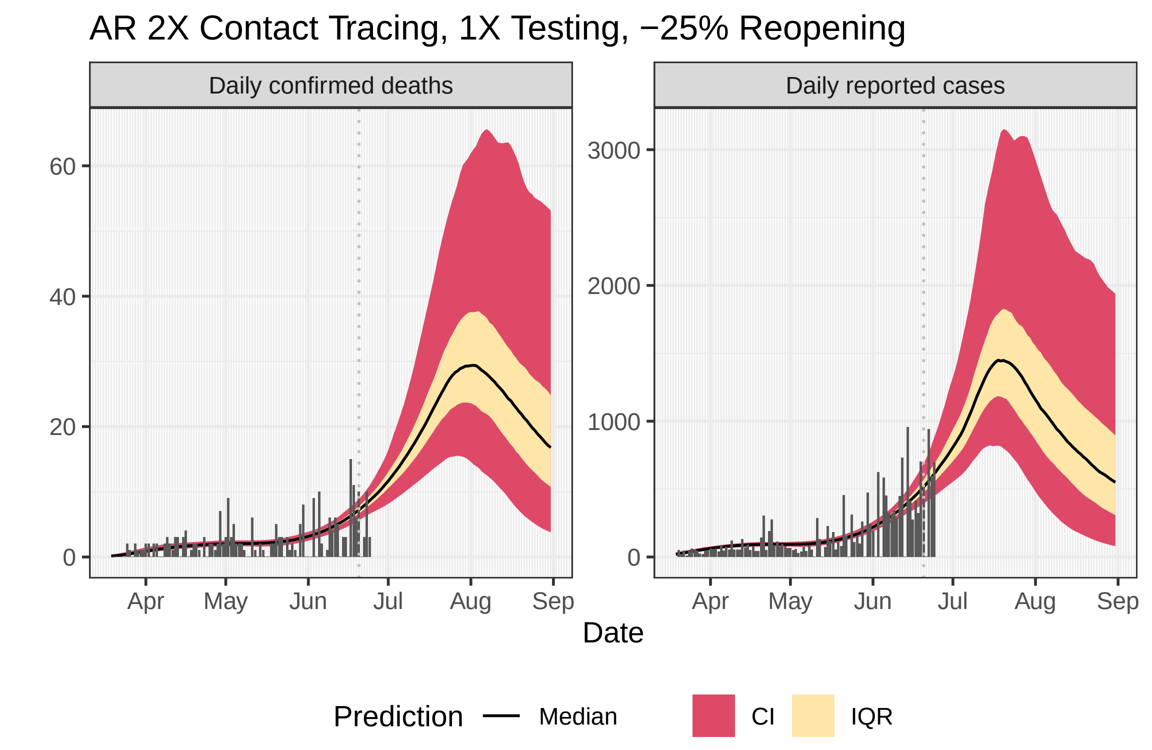
AR 2X Contact Tracing, 1X Testing, −25% Reopening
Daily confirmed deaths
Daily reported cases
0
20
40
60
0
1000
2000
3000
Apr
May
Jun
Jul
Aug
Sep
Date
Prediction
Median
CI
IQR

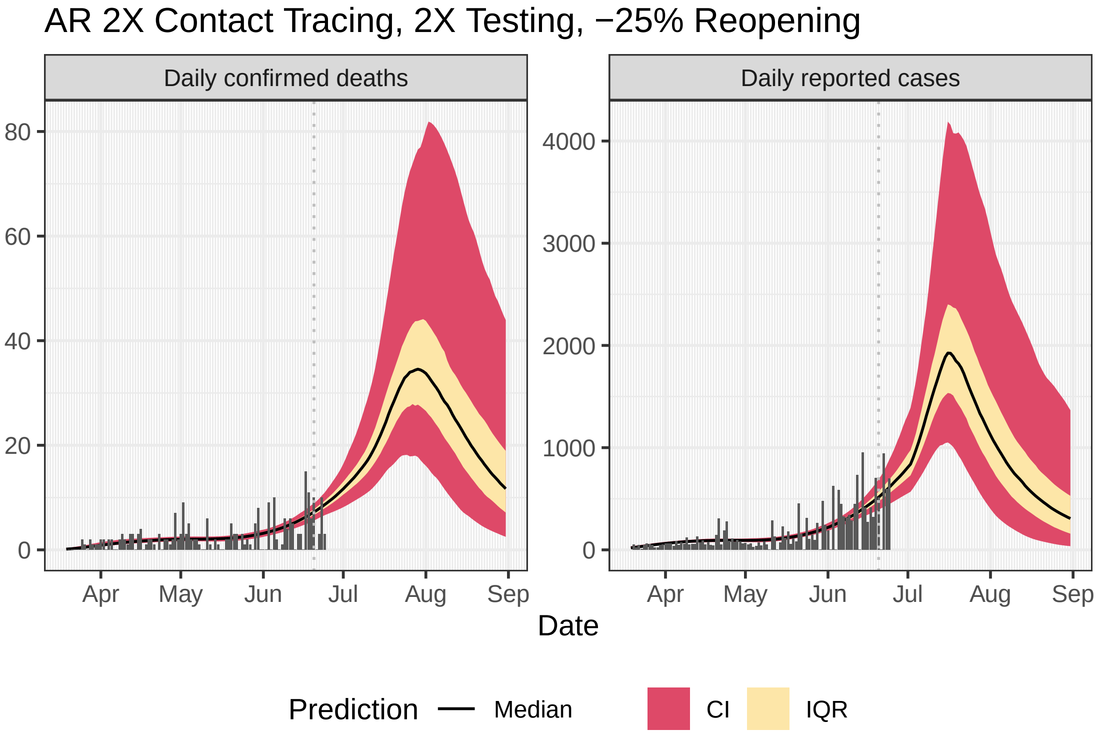

AR 2X Contact Tracing, 2X Testing, −25% Reopening
Daily confirmed deaths
Daily reported cases
80
60
40
20
0
4000
3000
2000
1000
0
Apr
May
Jun
Jul
Aug
Sep
Date
Prediction
Median
CI
IQR

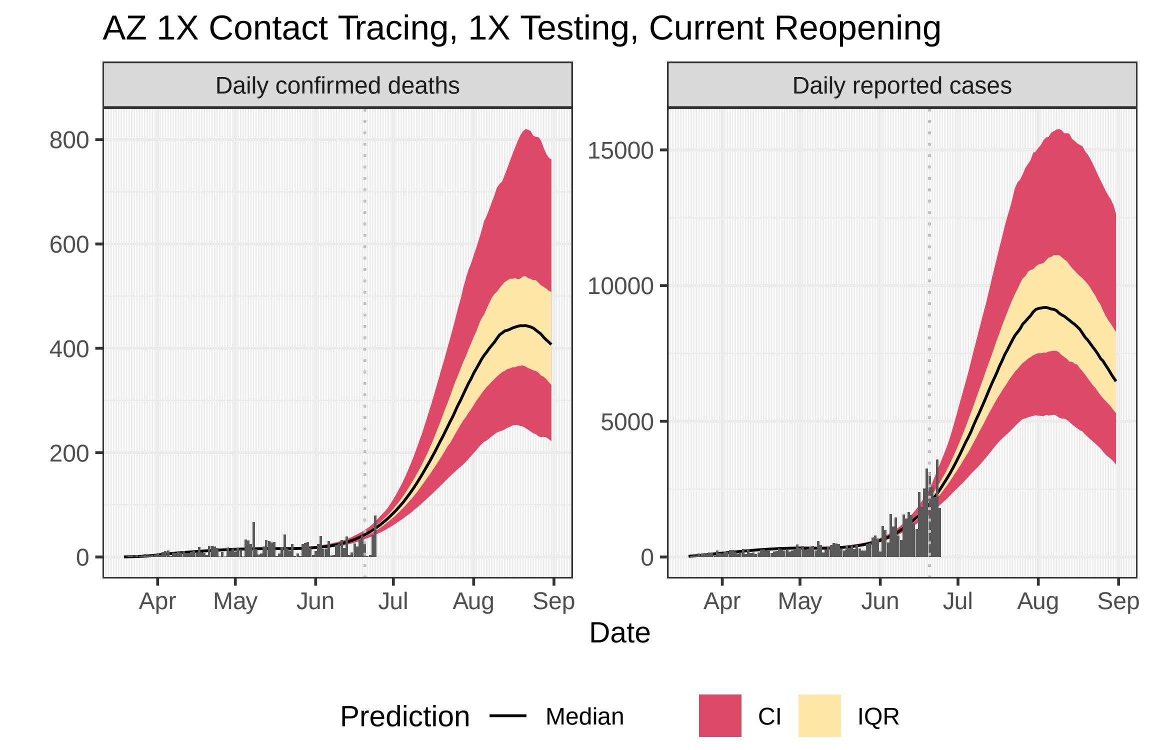

AZ 1X Contact Tracing, 1X Testing, Current Reopening
Daily confirmed deaths
Daily reported cases
800
600
400
200
0
15000
10000
5000
0
Apr
May
Jun
Jul
Aug
Sep
Date
Prediction
Median
CI
IQR

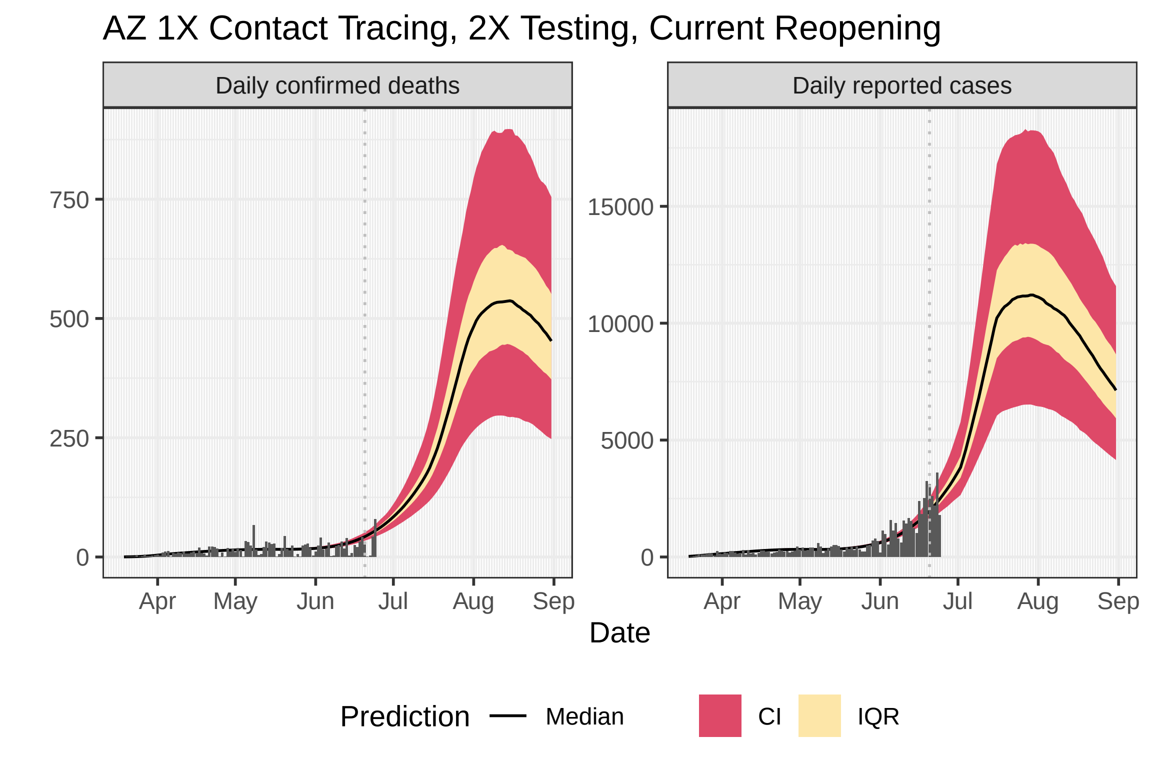

AZ 1X Contact Tracing, 2X Testing, Current Reopening
Daily confirmed deaths
Daily reported cases
750
500
250
0
15000
10000
5000
0
Apr
May
Jun
Jul
Aug
Sep
Date
Prediction
Median
CI
IQR

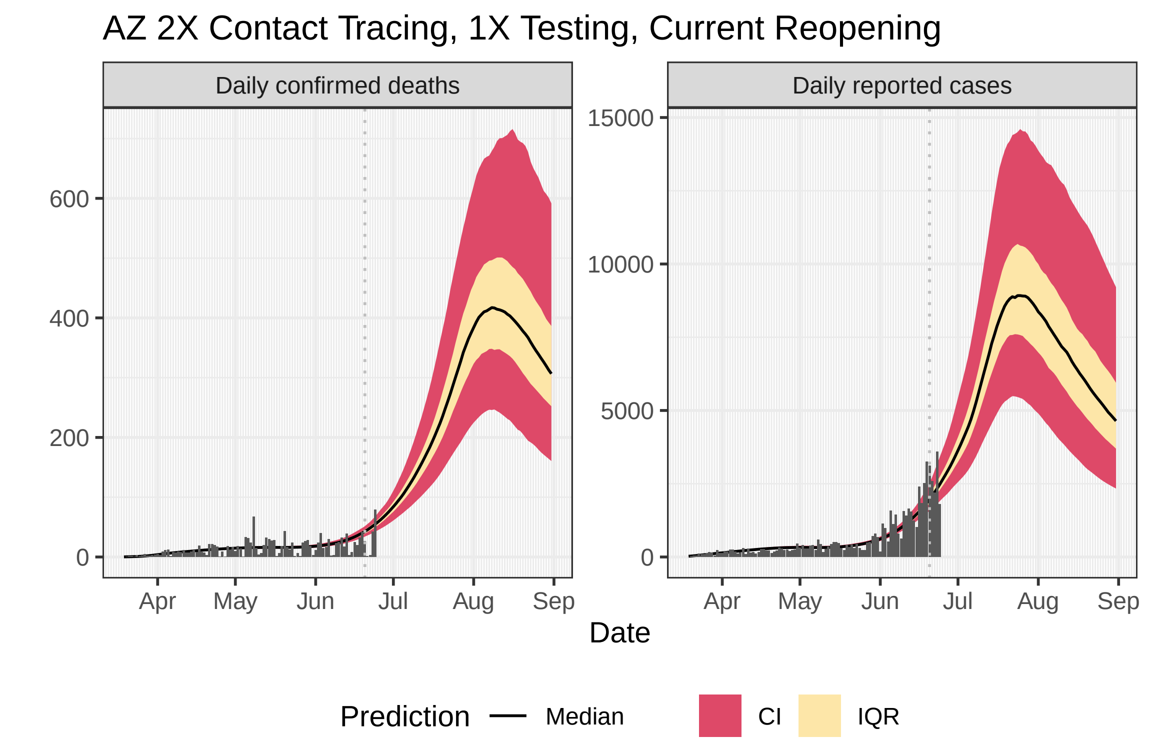

AZ 2X Contact Tracing, 1X Testing, Current Reopening
Daily confirmed deaths
Daily reported cases
0
200
400
600
0
5000
10000
15000
Apr
May
Jun
Jul
Aug
Sep
Date
Prediction
Median
CI
IQR

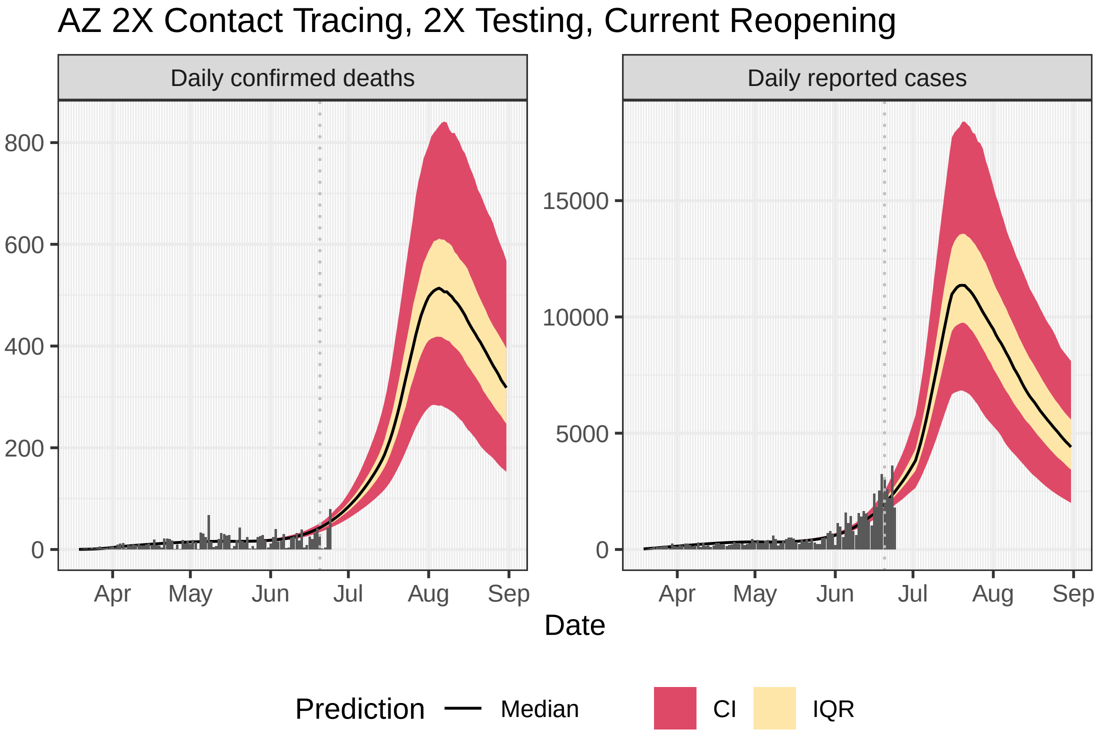
AZ 2X Contact Tracing, 2X Testing, Current Reopening
Daily confirmed deaths
Daily reported cases
0
200
400
600
800
0
5000
10000
15000
Apr
May
Jun
Jul
Aug
Sep
Date
Prediction
Median
CI
IQR

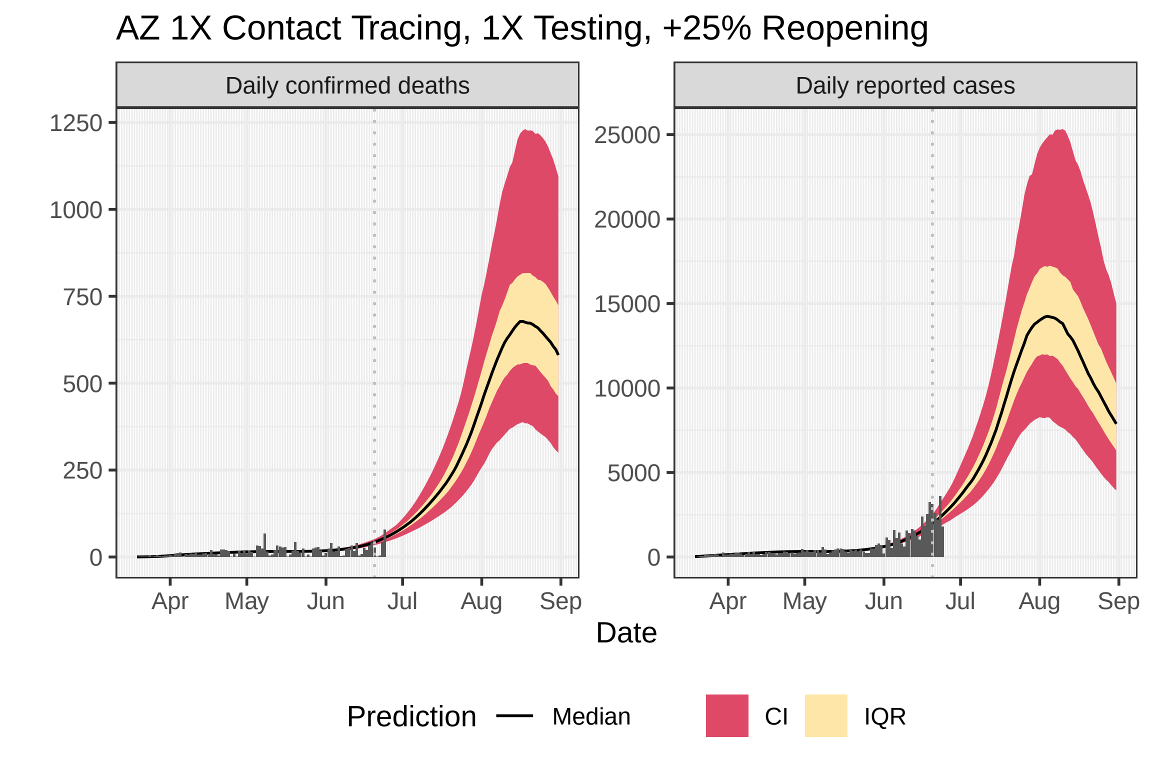

AZ 1X Contact Tracing, 1X Testing, +25% Reopening
Daily confirmed deaths
Daily reported cases
1250
1000
750
500
250
0
25000
20000
15000
10000
5000
0
Apr
May
Jun
Jul
Aug
Sep
Date
Prediction
Median
CI
IQR

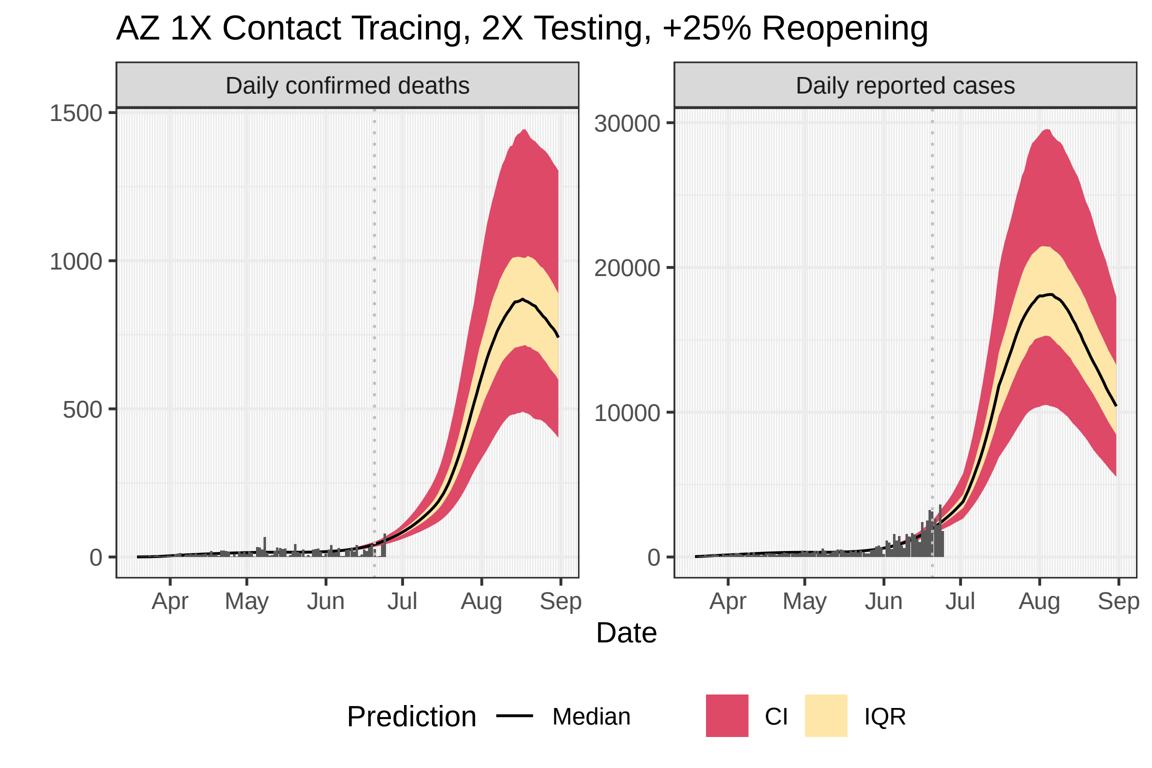
AZ 1X Contact Tracing, 2X Testing, +25% Reopening
Daily confirmed deaths
Daily reported cases
1500
1000
500
0
30000
20000
10000
0
Apr
May
Jun
Jul
Aug
Sep
Date
Prediction
Median
CI
IQR

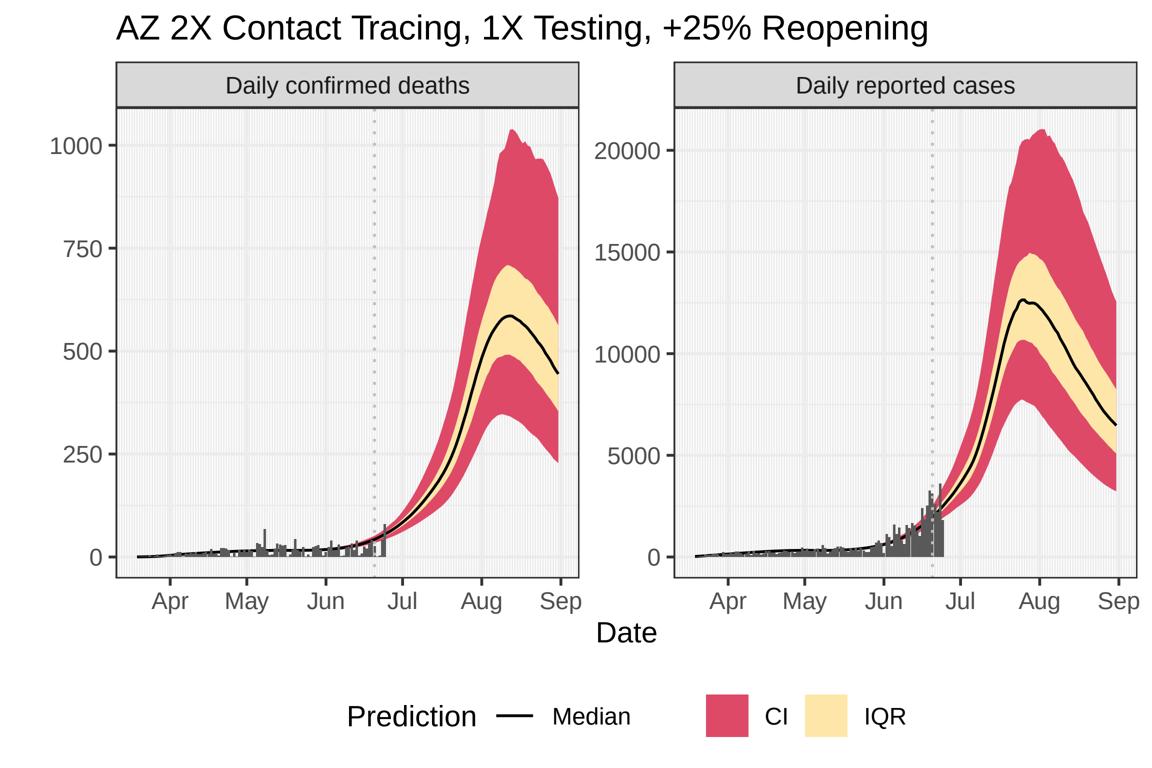

AZ 2X Contact Tracing, 1X Testing, +25% Reopening
Daily confirmed deaths
Daily reported cases
1000
750
500
250
0
20000
15000
10000
5000
0
Apr
May
Jun
Jul
Aug
Sep
Date
Prediction
Median
CI
IQR

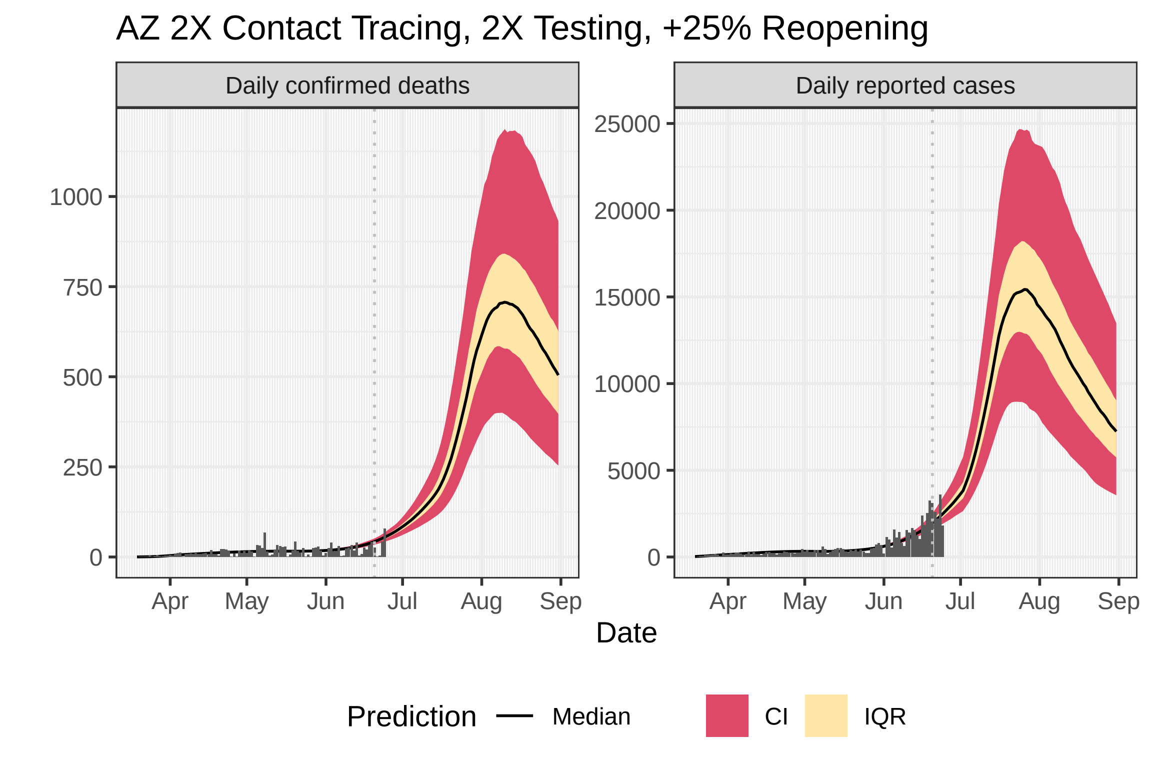

AZ 2X Contact Tracing, 2X Testing, +25% Reopening
Daily confirmed deaths
Daily reported cases
1000
750
500
250
0
25000
20000
15000
10000
5000
0
Apr
May
Jun
Jul
Aug
Sep
Date
Prediction
Median
CI
IQR

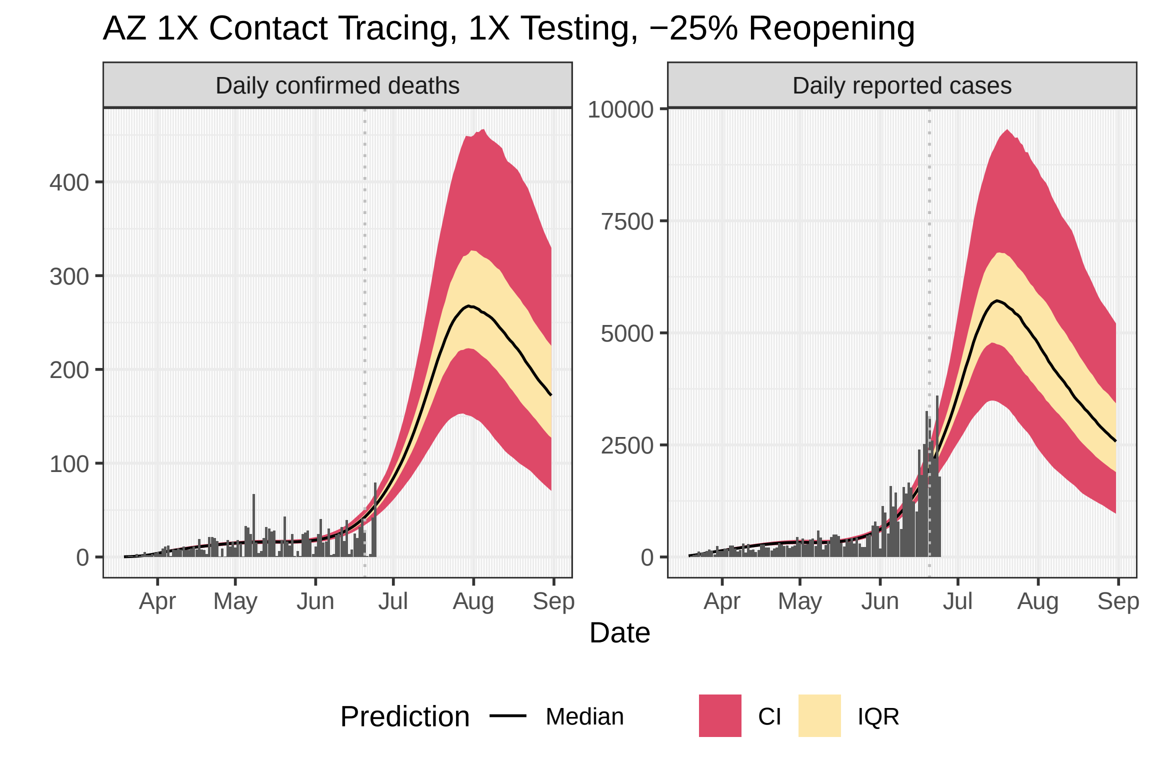
AZ 1X Contact Tracing, 1X Testing, −25% Reopening
Daily confirmed deaths
Daily reported cases
400
300
200
100
0
10000
7500
5000
2500
0
Apr
May
Jun
Jul
Aug
Sep
Date
Prediction
Median
CI
IQR

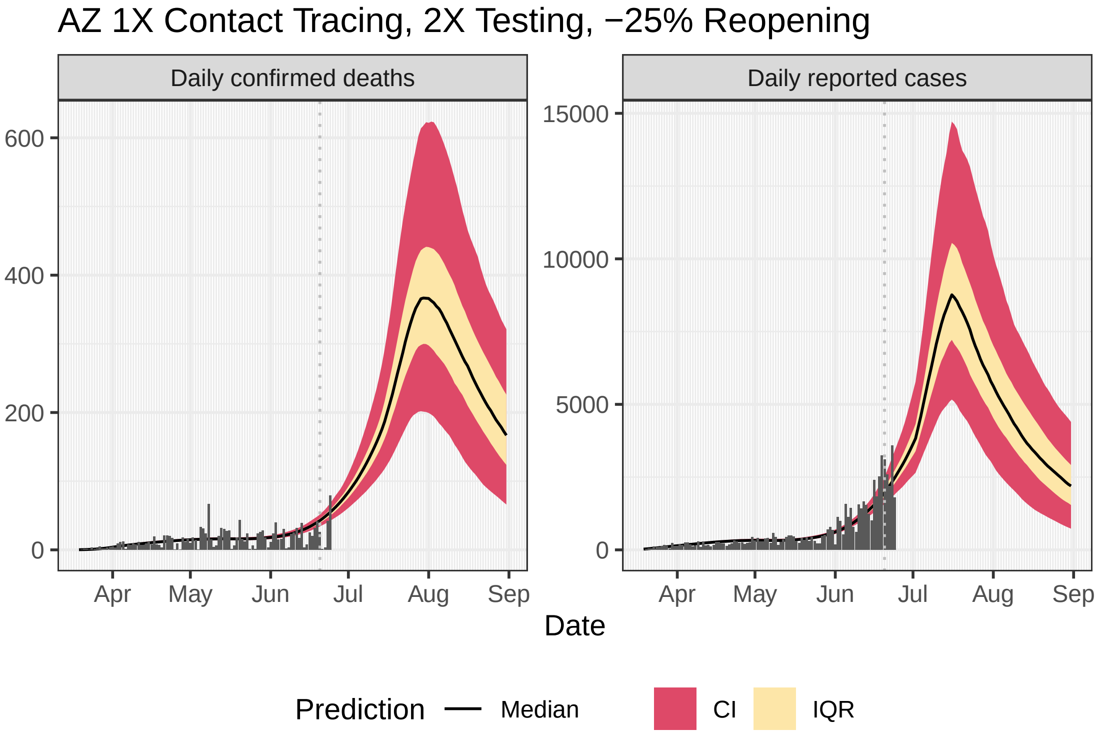
AZ 1X Contact Tracing, 2X Testing, −25% Reopening
Daily confirmed deaths
Daily reported cases
600
400
200
0
15000
10000
5000
0
Apr
May
Jun
Jul
Aug
Sep
Date
Prediction
Median
CI
IQR

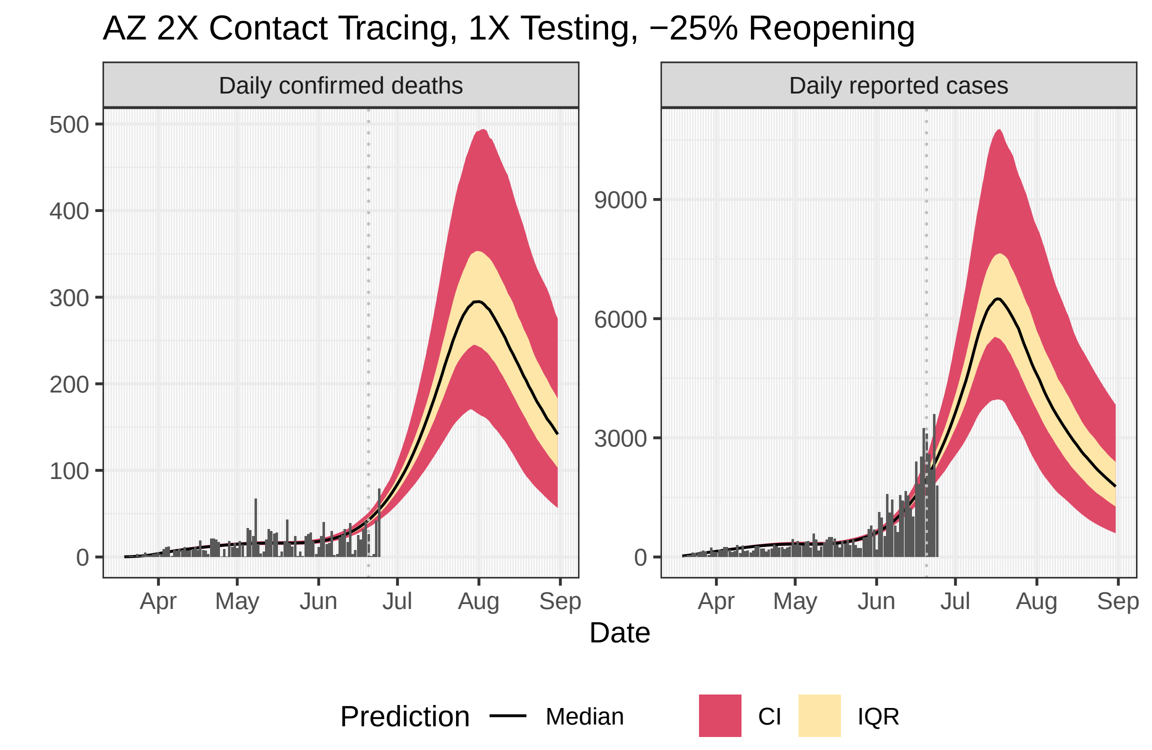

AZ 2X Contact Tracing, 1X Testing, −25% Reopening
Daily confirmed deaths
Daily reported cases
500
400
300
200
100
0
9000
6000
3000
0
Apr
May
Jun
Jul
Aug
Sep
Date
Prediction
Median
CI
IQR

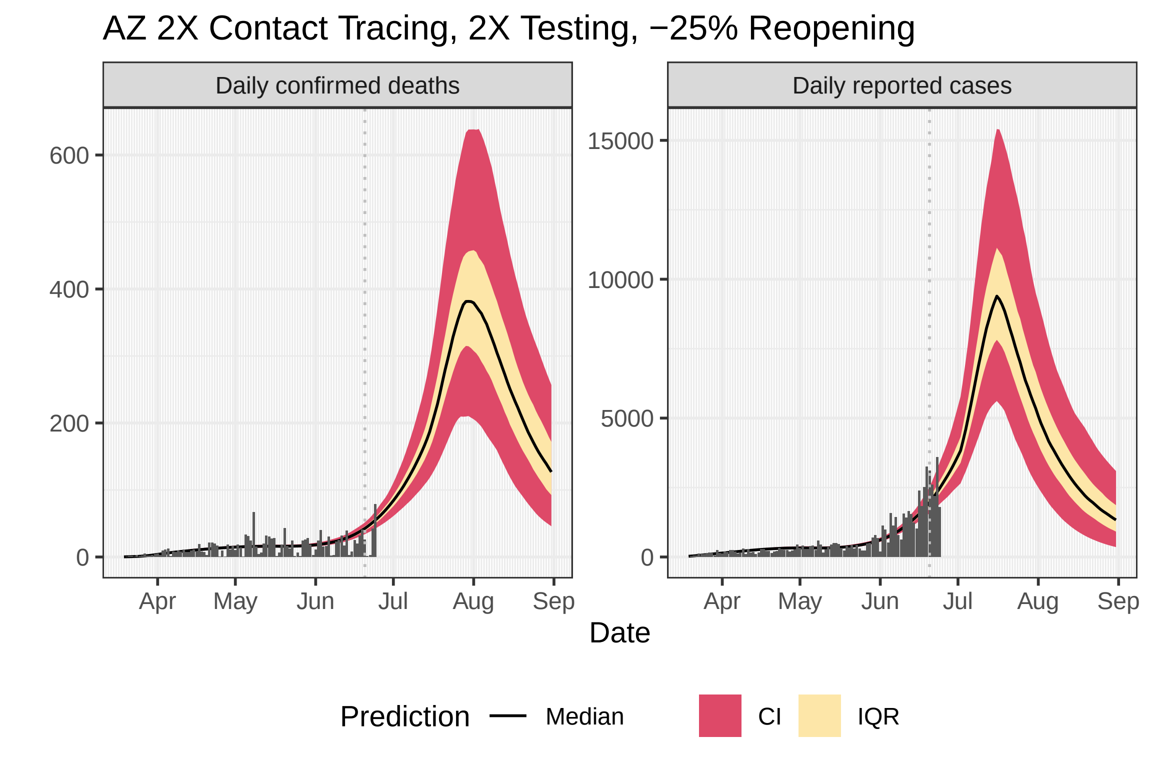
AZ 2X Contact Tracing, 2X Testing, −25% Reopening
Daily confirmed deaths
Daily reported cases
600
400
200
0
15000
10000
5000
0
Apr
May
Jun
Jul
Aug
Sep
Date
Prediction
Median
CI
IQR

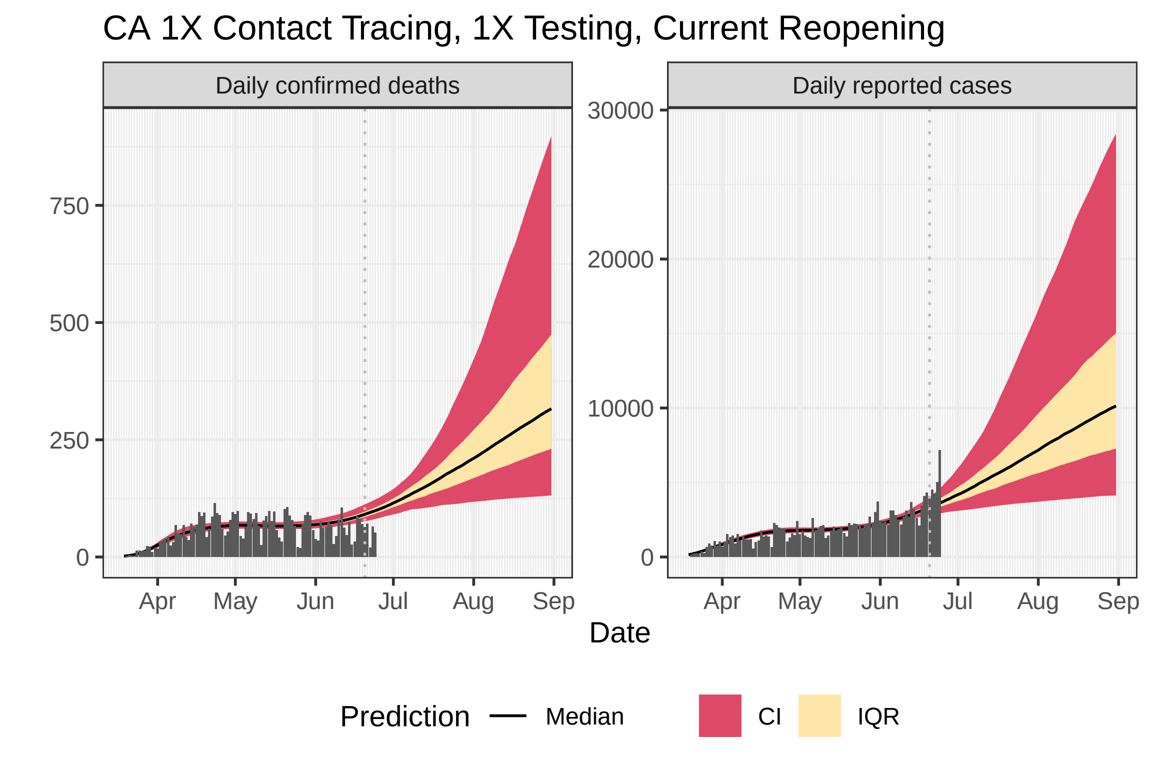

CA 1X Contact Tracing, 1X Testing, Current Reopening
Daily confirmed deaths
Daily reported cases
750
500
250
0
30000
20000
10000
0
Apr
May
Jun
Jul
Aug
Sep
Date
Prediction
Median
CI
IQR

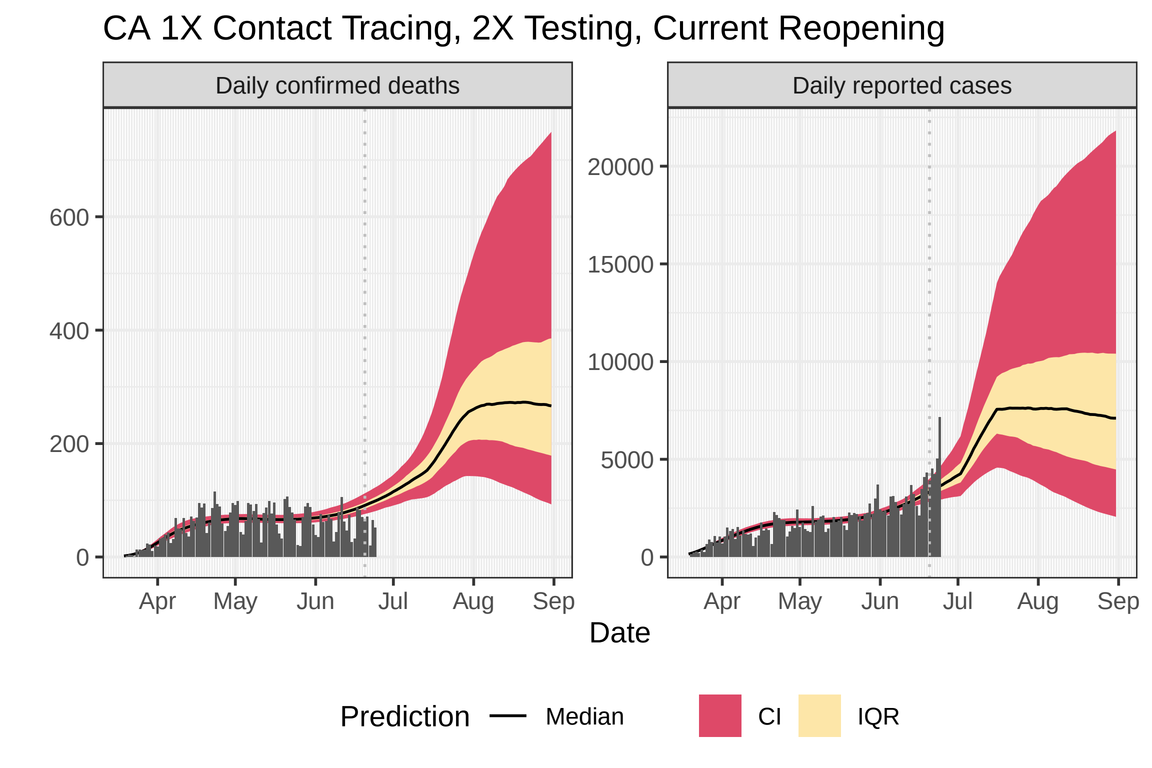
CA 1X Contact Tracing, 2X Testing, Current Reopening
Daily confirmed deaths
Daily reported cases
0
200
400
600
0
5000
10000
15000
20000
Apr
May
Jun
Jul
Aug
Sep
Date
Prediction
Median
CI
IQR

CA 2X Contact Tracing, 1X Testing, Current Reopening

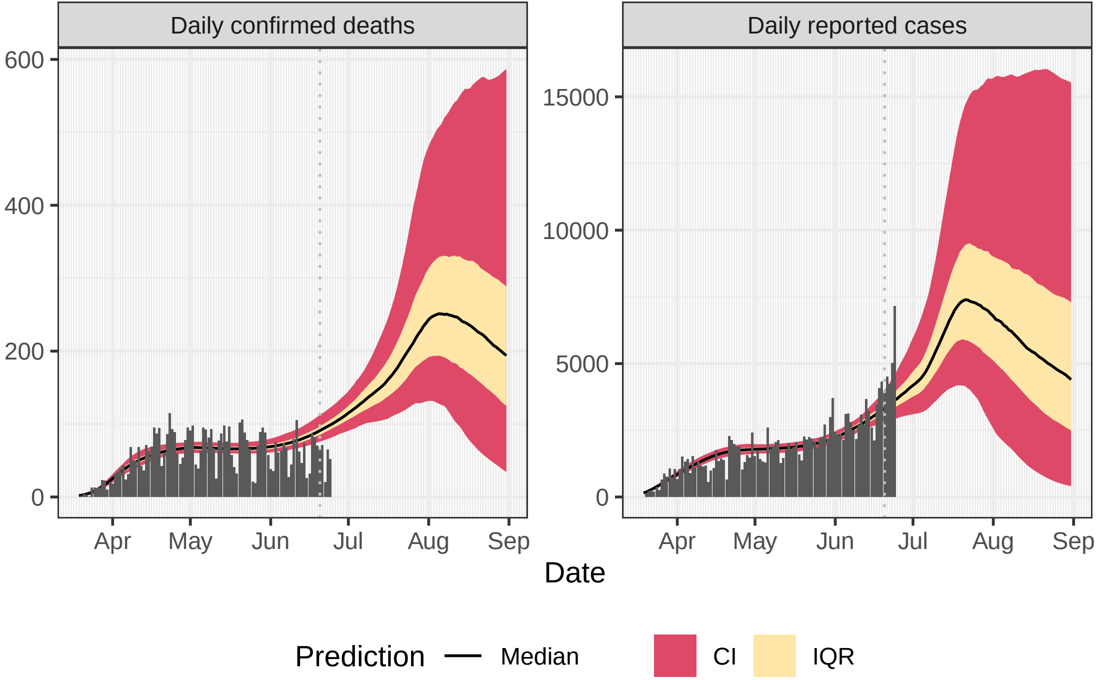

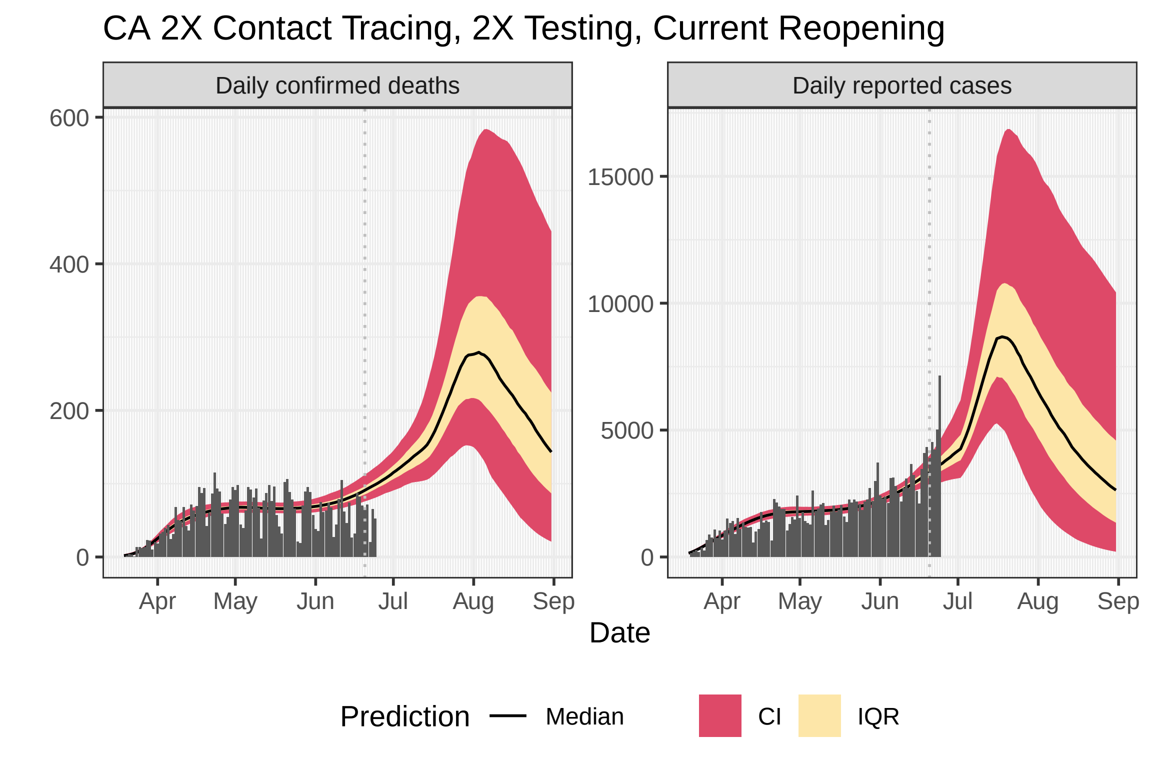
CA 2X Contact Tracing, 2X Testing, Current Reopening
Daily confirmed deaths
Daily reported cases
600
400
200
0
15000
10000
5000
0
Apr
May
Jun
Jul
Aug
Sep
Date
Prediction
Median
CI
IQR

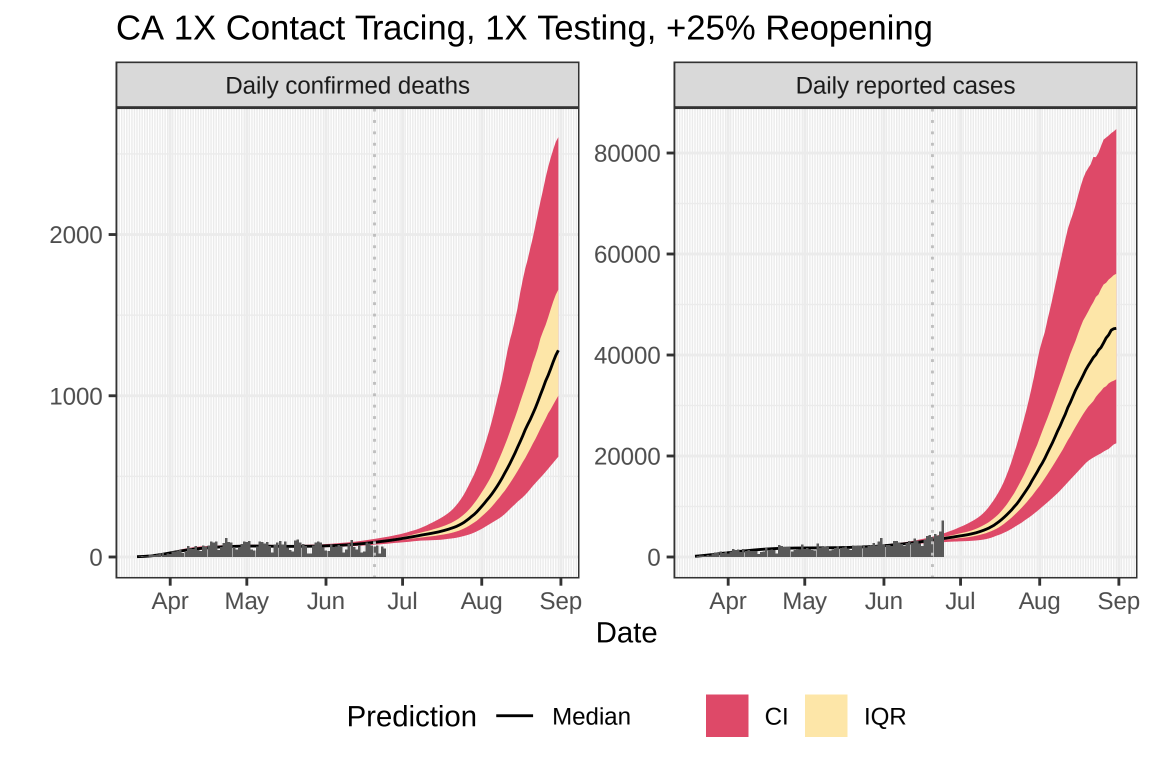

CA 1X Contact Tracing, 1X Testing, +25% Reopening
Daily confirmed deaths
Daily reported cases
0
1000
2000
0
20000
40000
60000
80000
Apr
May
Jun
Jul
Aug
Sep
Date
Prediction
Median
CI
IQR

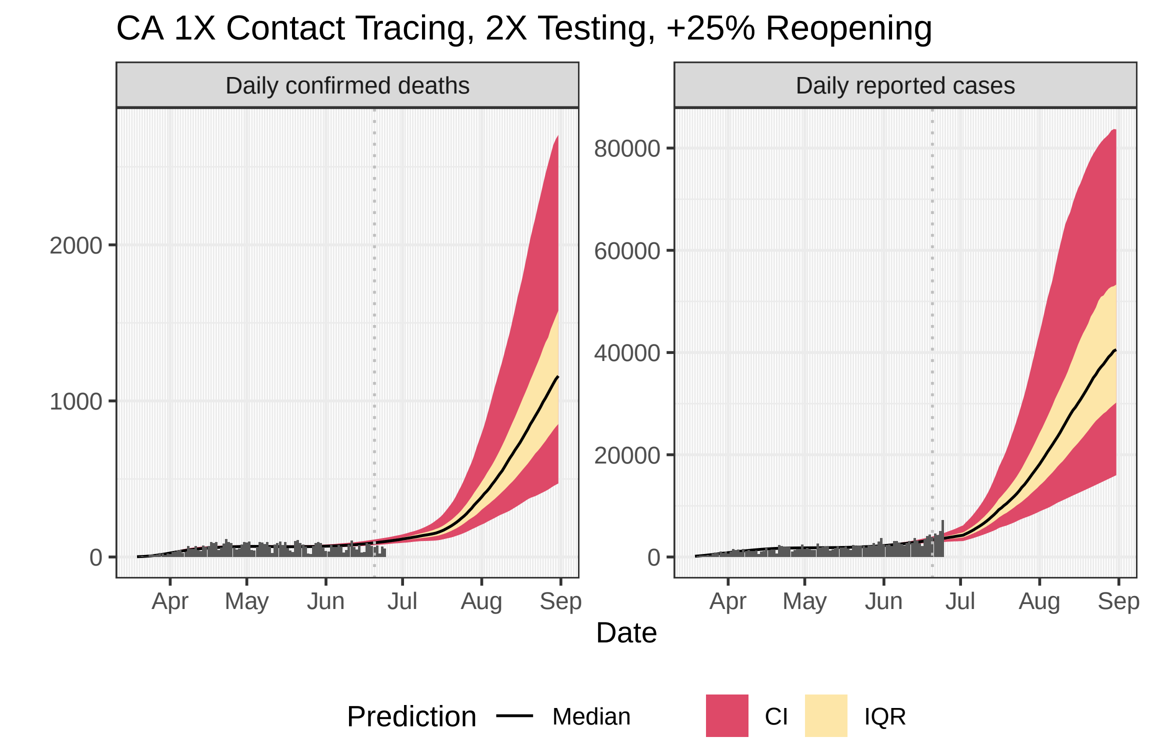

CA 1X Contact Tracing, 2X Testing, +25% Reopening
Daily confirmed deaths
Daily reported cases
0
1000
2000
0
20000
40000
60000
80000
Apr
May
Jun
Jul
Aug
Sep
Date
Prediction
Median
CI
IQR

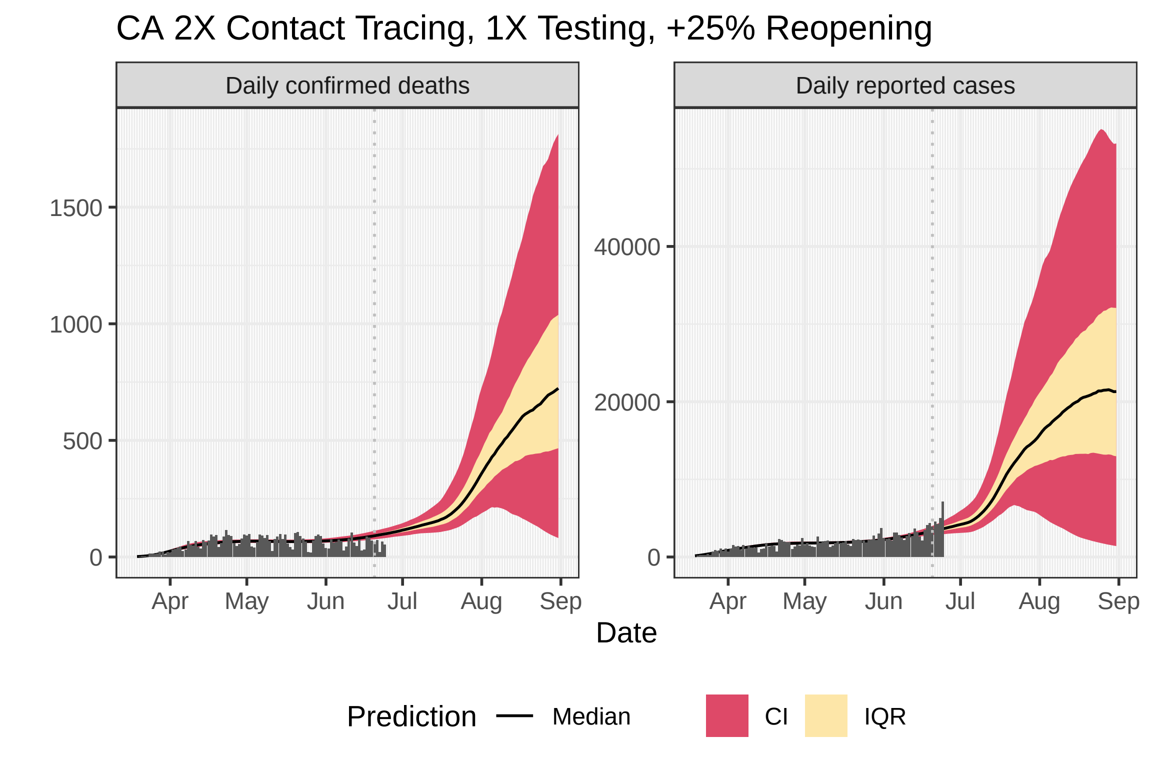

CA 2X Contact Tracing, 1X Testing, +25% Reopening
Daily confirmed deaths
Daily reported cases
1500
1000
500
0
40000
20000
0
Apr
May
Jun
Jul
Aug
Sep
Date
Prediction
Median
CI
IQR

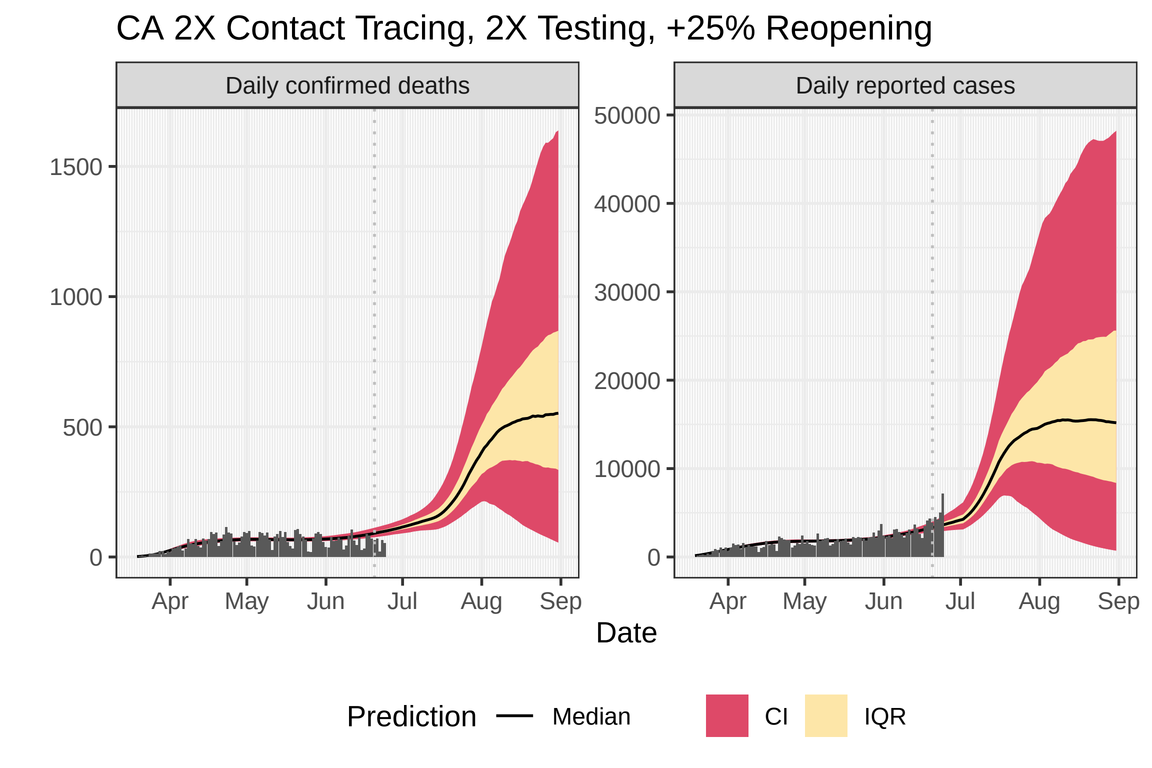
CA 2X Contact Tracing, 2X Testing, +25% Reopening
Daily confirmed deaths
Daily reported cases
1500
1000
500
0
50000
40000
30000
20000
10000
0
Apr
May
Jun
Jul
Aug
Sep
Date
Prediction
Median
CI
IQR

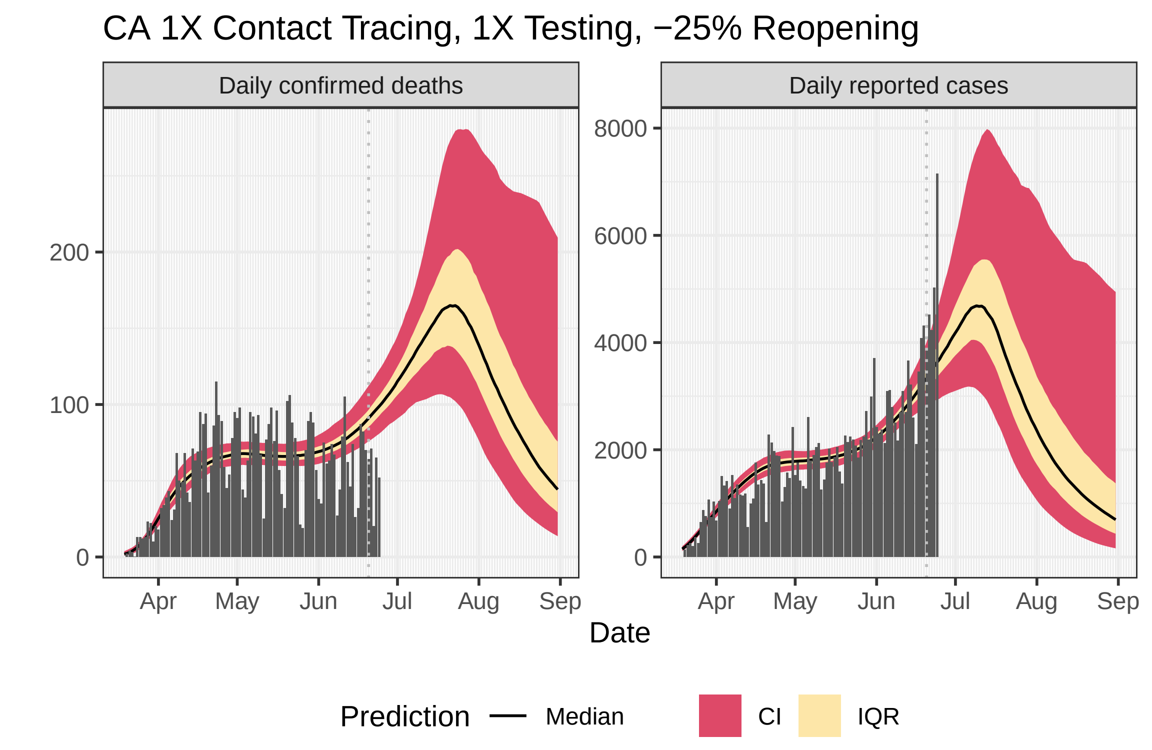
CA 1X Contact Tracing, 1X Testing, −25% Reopening
Daily confirmed deaths
Daily reported cases
0
100
200
0
2000
4000
6000
8000
Apr
May
Jun
Jul
Aug
Sep
Date
Prediction
Median
CI
IQR

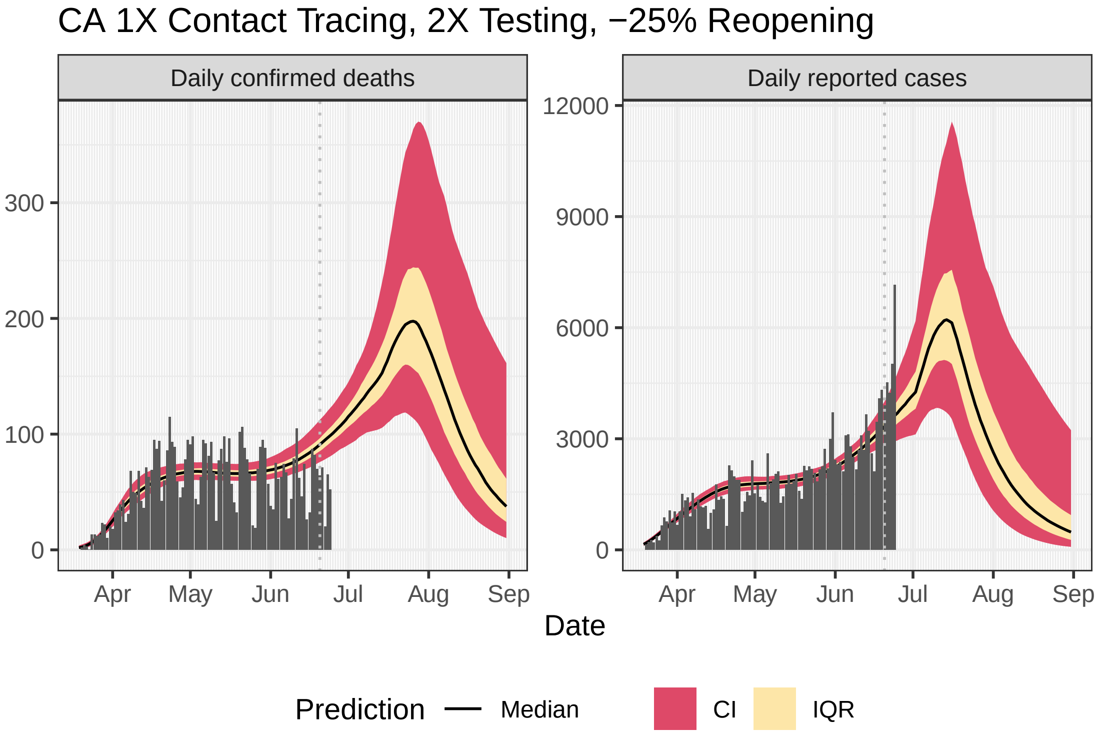

CA 1X Contact Tracing, 2X Testing, −25% Reopening
Daily confirmed deaths
Daily reported cases
300
200
100
0
12000
9000
6000
3000
0
Apr
May
Jun
Jul
Aug
Sep
Date
Prediction
Median
CI
IQR

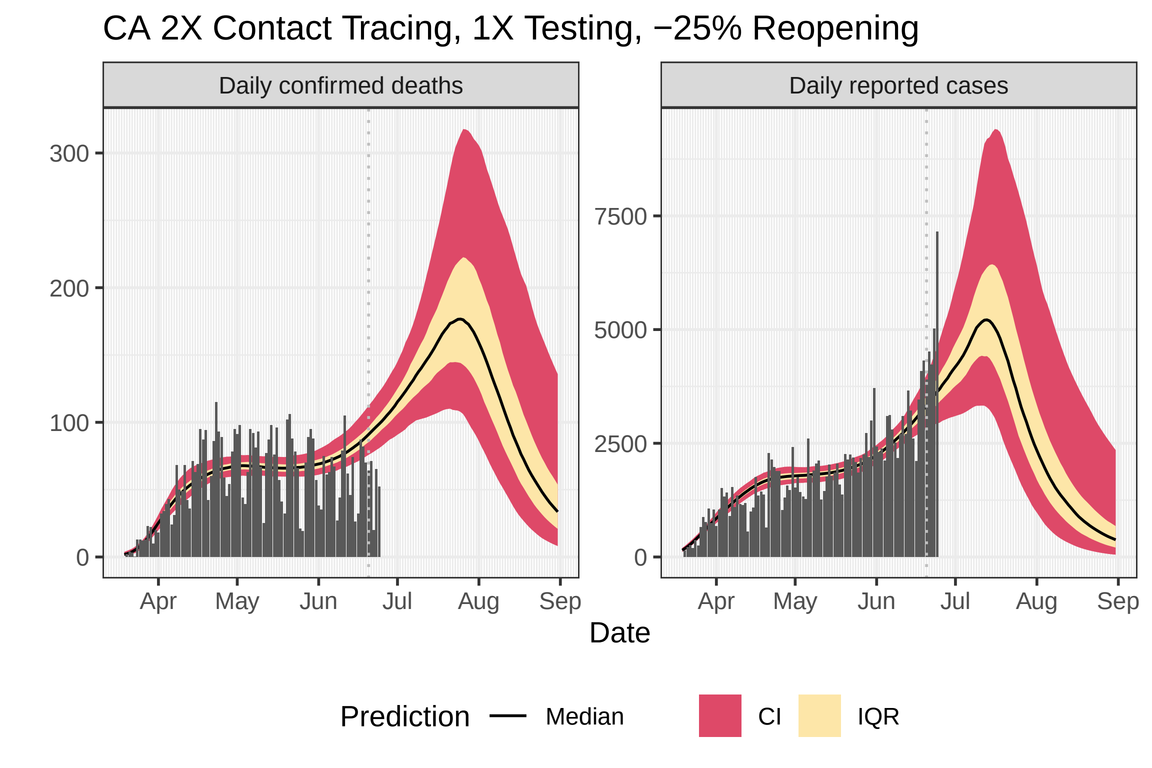
CA 2X Contact Tracing, 1X Testing, −25% Reopening
Daily confirmed deaths
Daily reported cases
300
200
100
0
7500
5000
2500
0
Apr
May
Jun
Jul
Aug
Sep
Date
Prediction
Median
CI
IQR

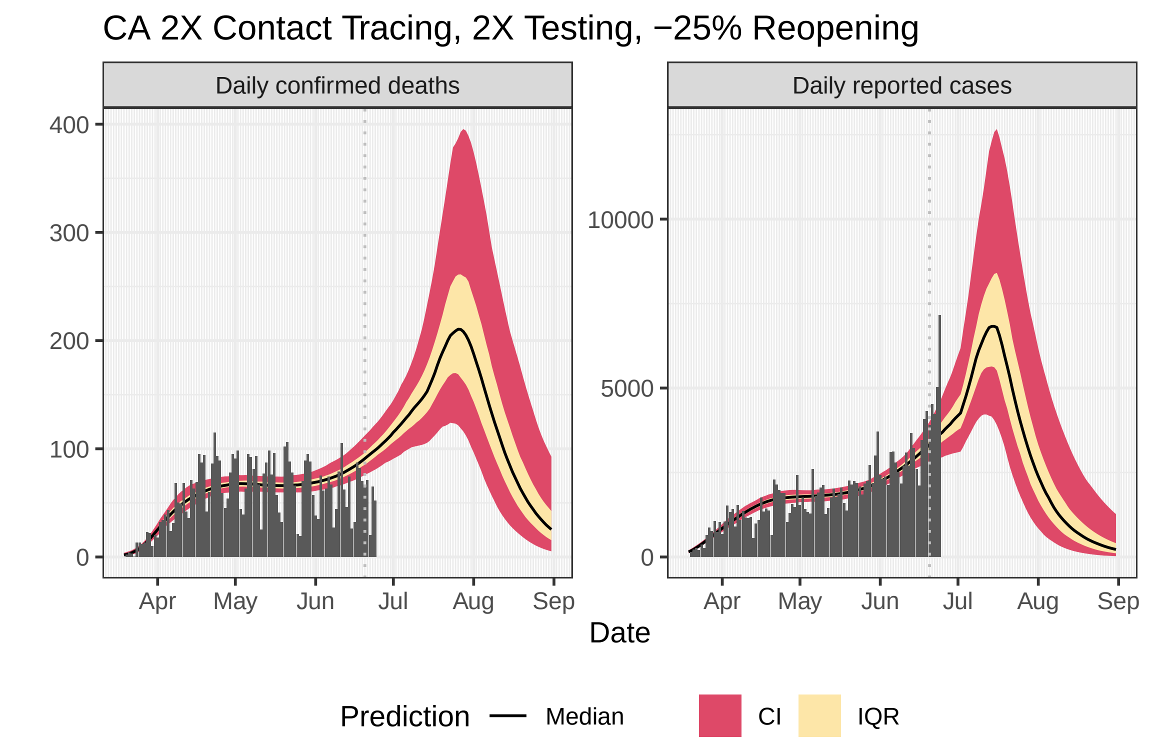

CA 2X Contact Tracing, 2X Testing, −25% Reopening
Daily confirmed deaths
Daily reported cases
400
300
200
100
0
10000
5000
0
Apr
May
Jun
Jul
Aug
Sep
Date
Prediction
Median
CI
IQR

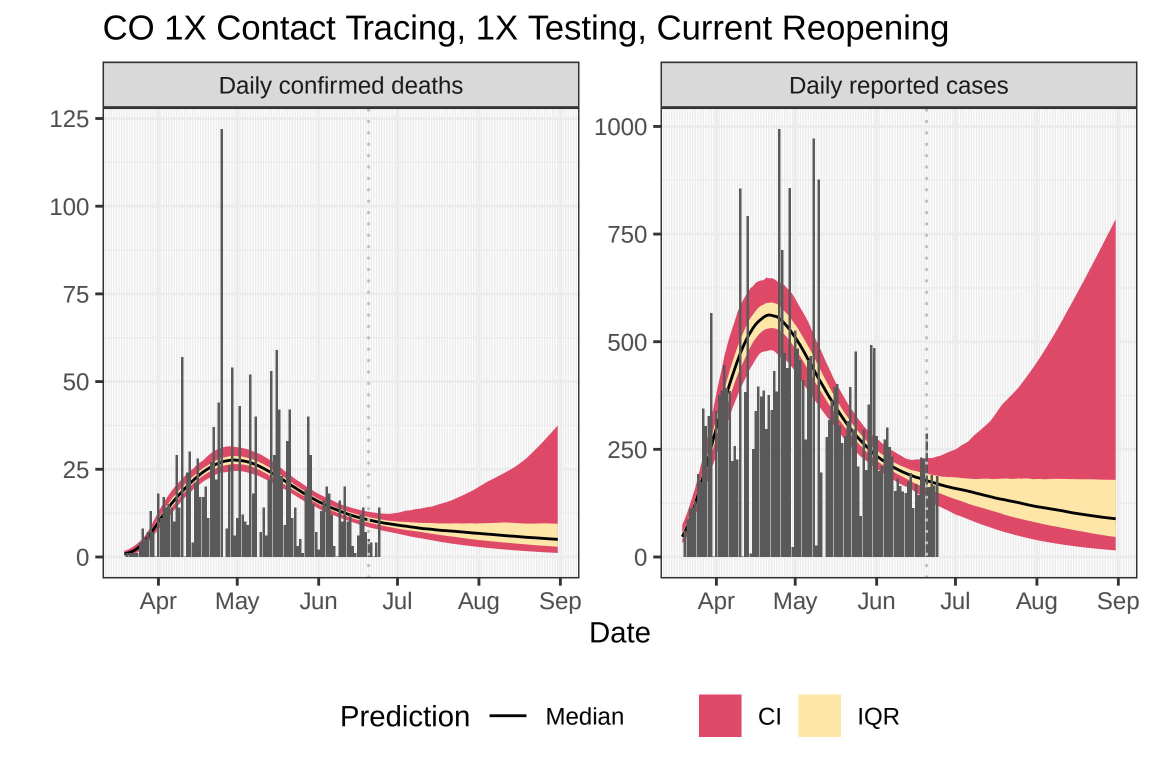

CO 1X Contact Tracing, 1X Testing, Current Reopening
Daily confirmed deaths
Daily reported cases
125
100
75
50
25
0
1000
750
500
250
0
Apr
May
Jun
Jul
Aug
Sep
Date
Prediction
Median
CI
IQR

CO 1X Contact Tracing, 2X Testing, Current Reopening

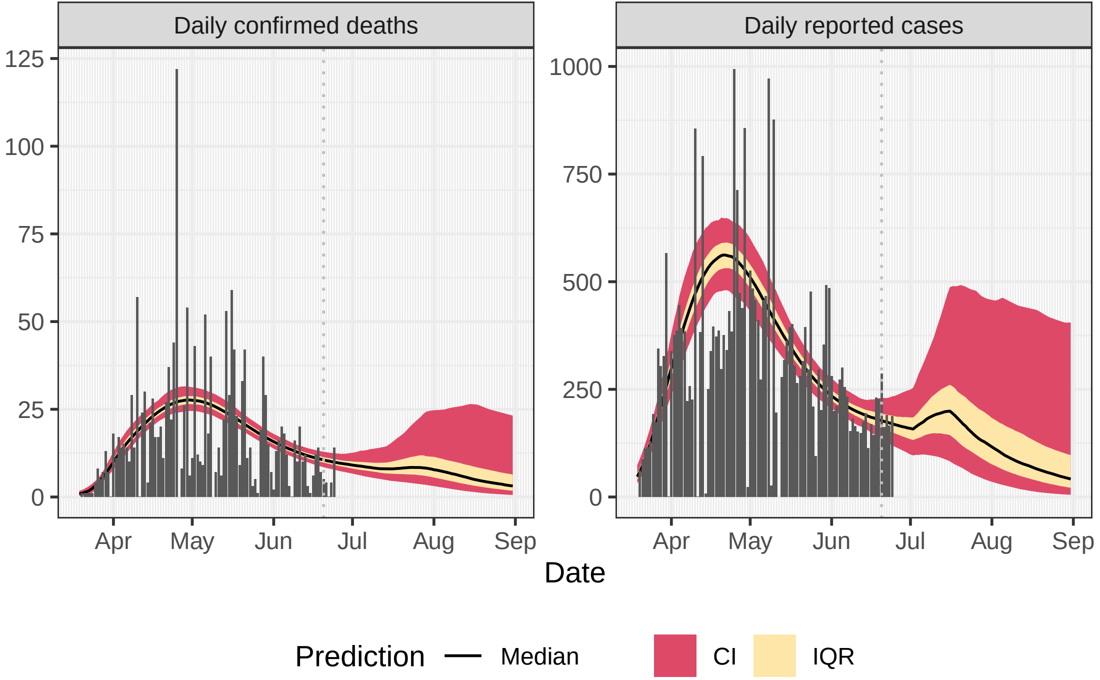

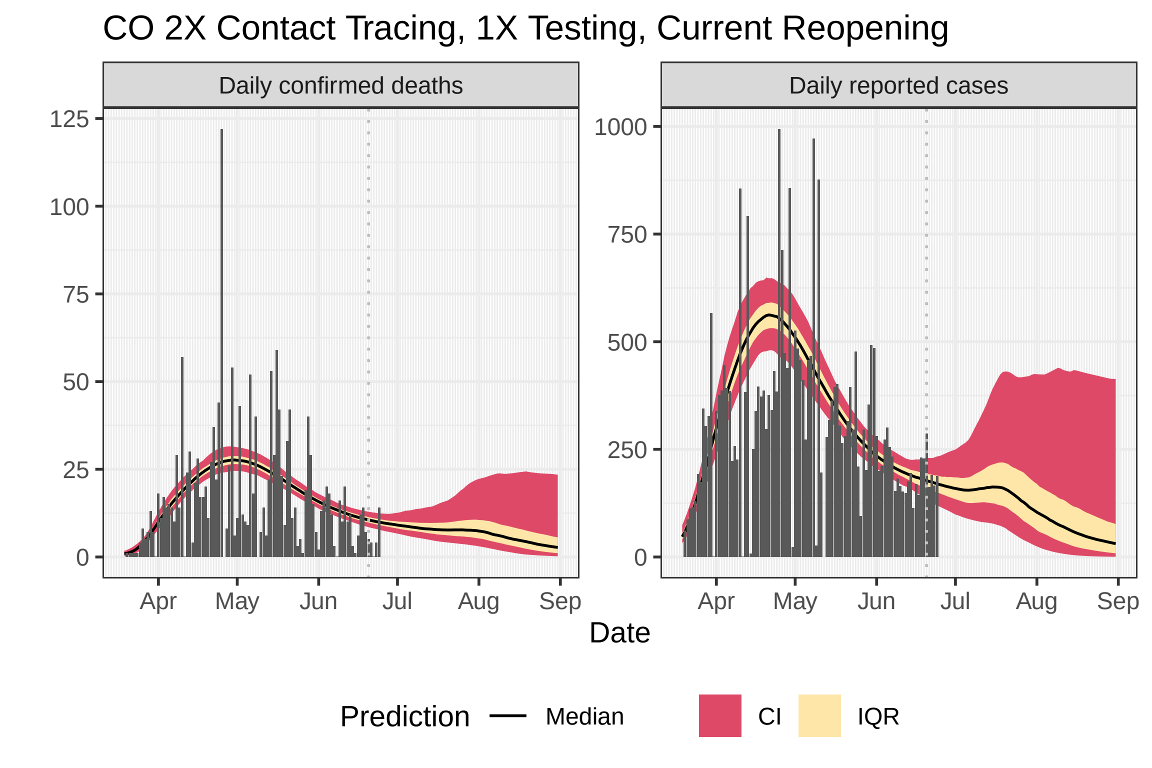

CO 2X Contact Tracing, 1X Testing, Current Reopening
Daily confirmed deaths
Daily reported cases
125
100
75
50
25
0
1000
750
500
250
0
Apr
May
Jun
Jul
Aug
Sep
Date
Prediction
Median
CI
IQR

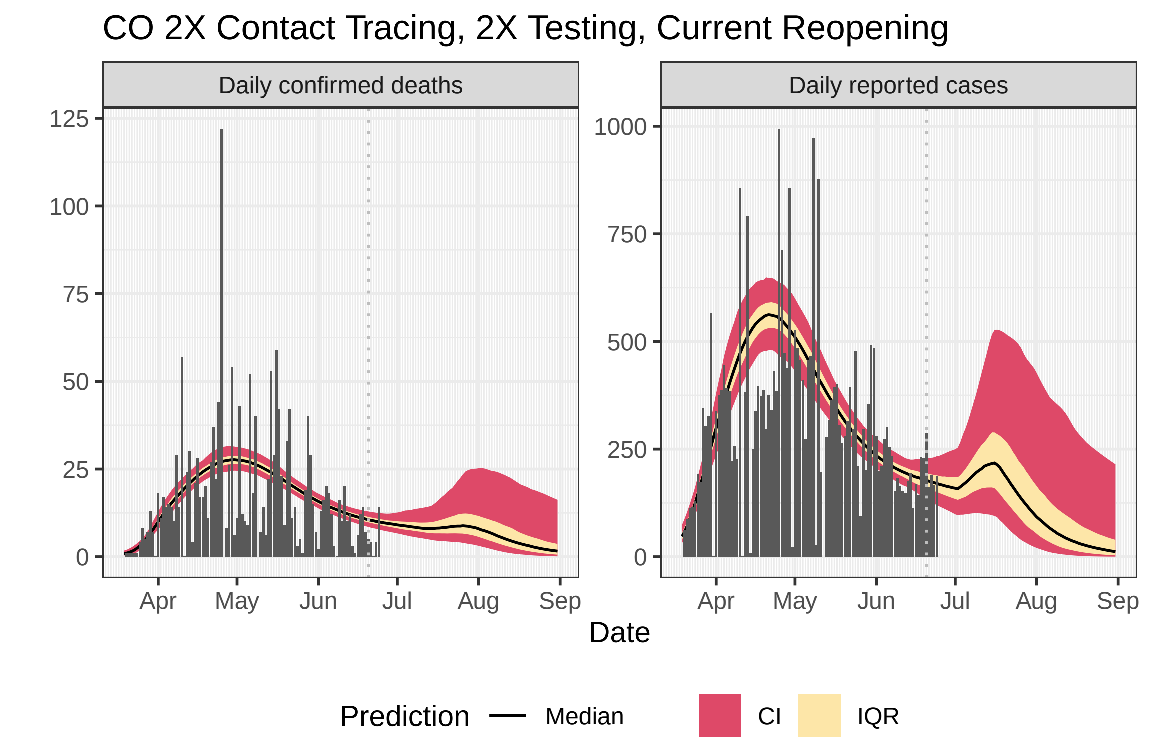

CO 2X Contact Tracing, 2X Testing, Current Reopening
Daily confirmed deaths
Daily reported cases
125
100
75
50
25
0
1000
750
500
250
0
Apr
May
Jun
Jul
Aug
Sep
Date
Prediction
Median
CI
IQR

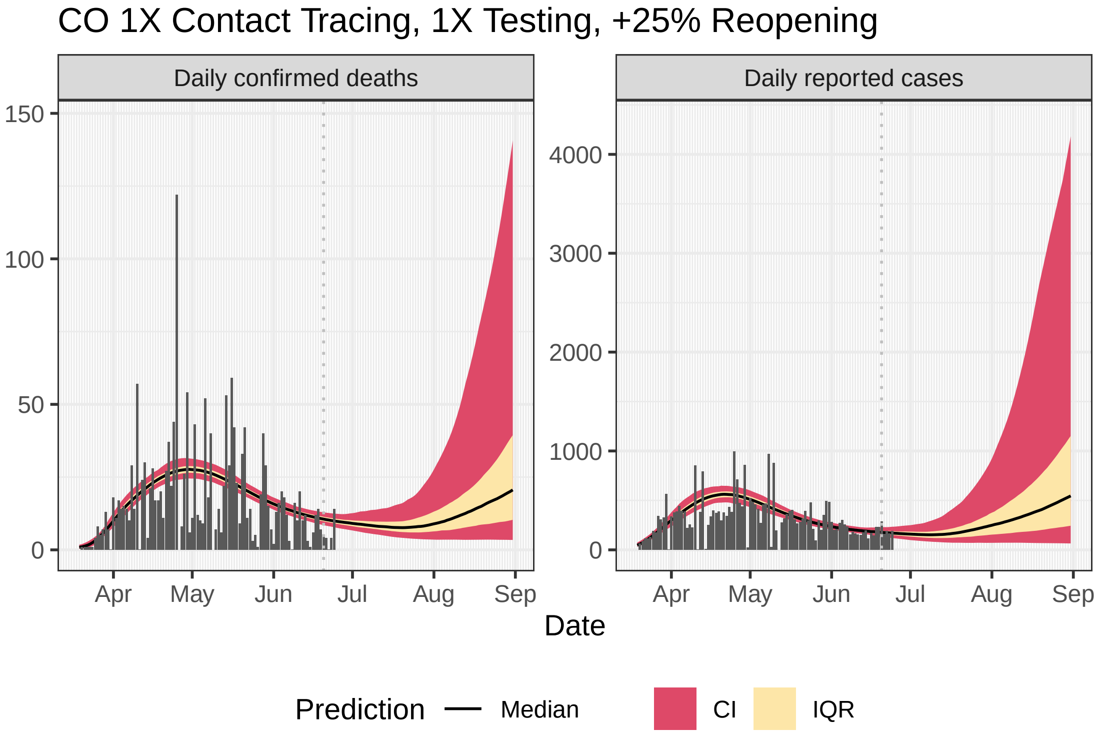

CO 1X Contact Tracing, 1X Testing, +25% Reopening
Daily confirmed deaths
Daily reported cases
150
100
50
0
4000
3000
2000
1000
0
Apr
May
Jun
Jul
Aug
Sep
Date
Prediction
Median
CI
IQR

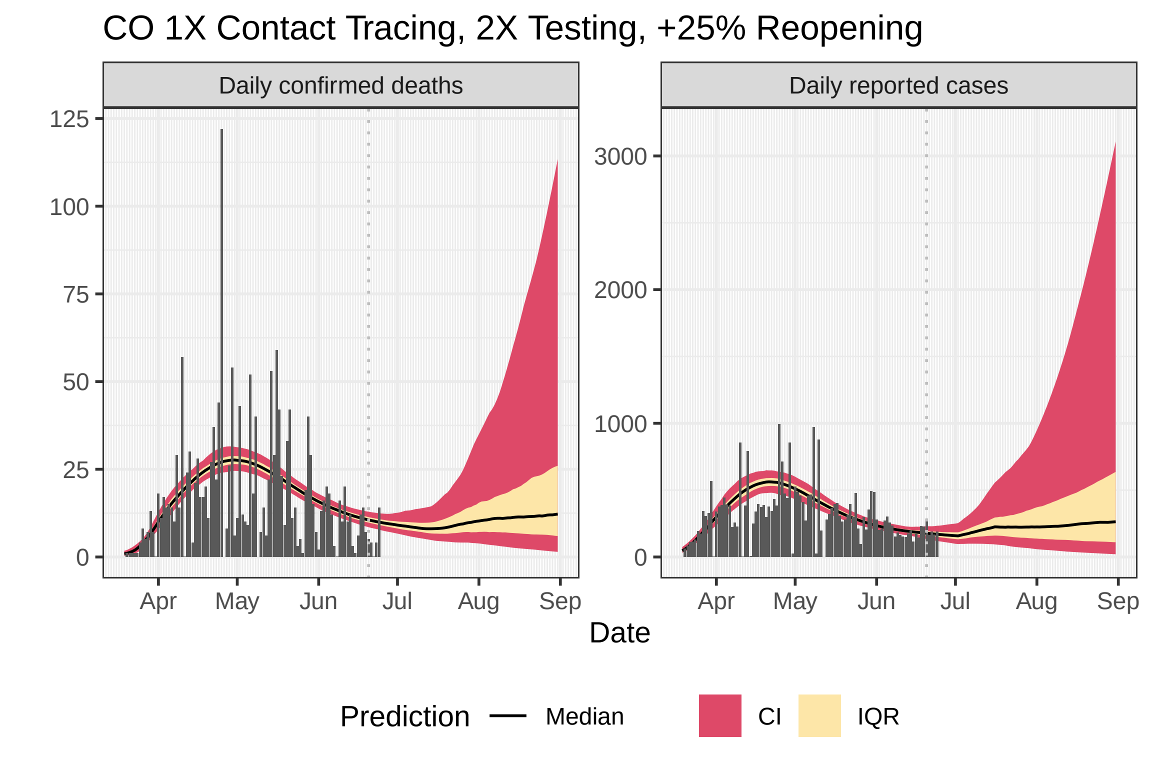
CO 1X Contact Tracing, 2X Testing, +25% Reopening
Daily confirmed deaths
Daily reported cases
125
100
75
50
25
0
3000
2000
1000
0
Apr
May
Jun
Jul
Aug
Sep
Date
Prediction
Median
CI
IQR

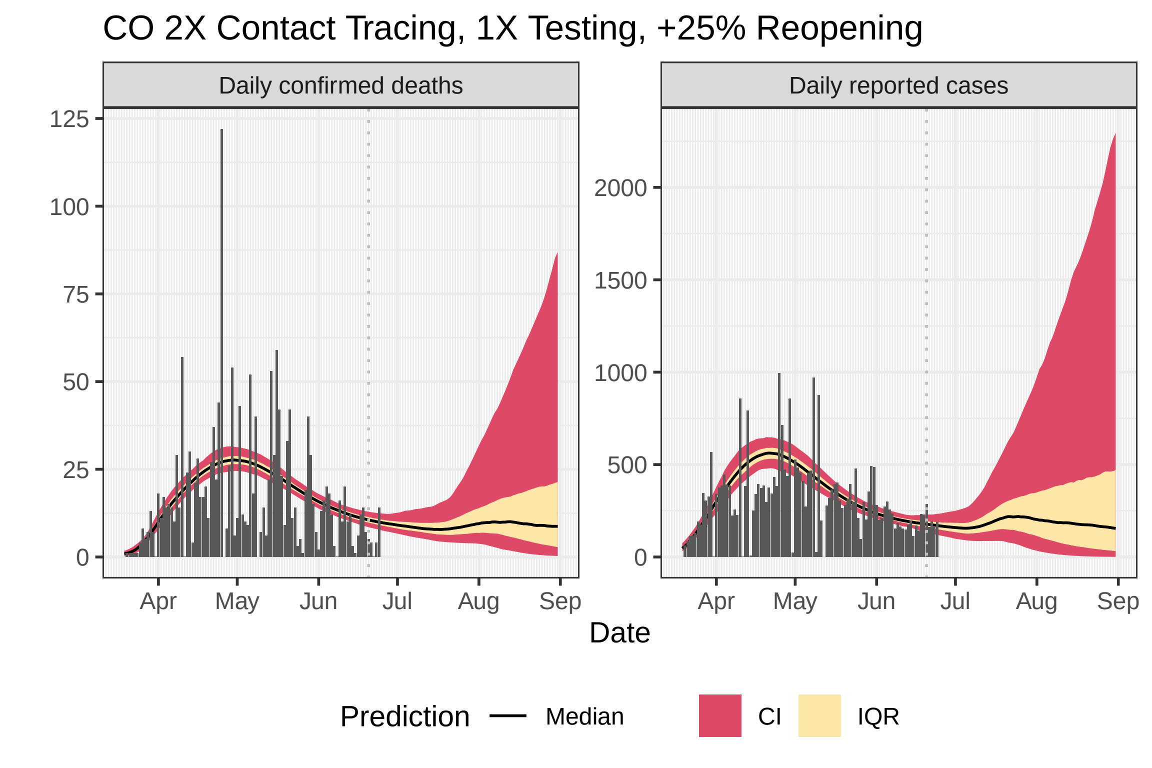

CO 2X Contact Tracing, 1X Testing, +25% Reopening
Daily confirmed deaths
Daily reported cases
0
25
50
75
100
125
0
500
1000
1500
2000
Apr
May
Jun
Jul
Aug
Sep
Date
Prediction
Median
CI
IQR

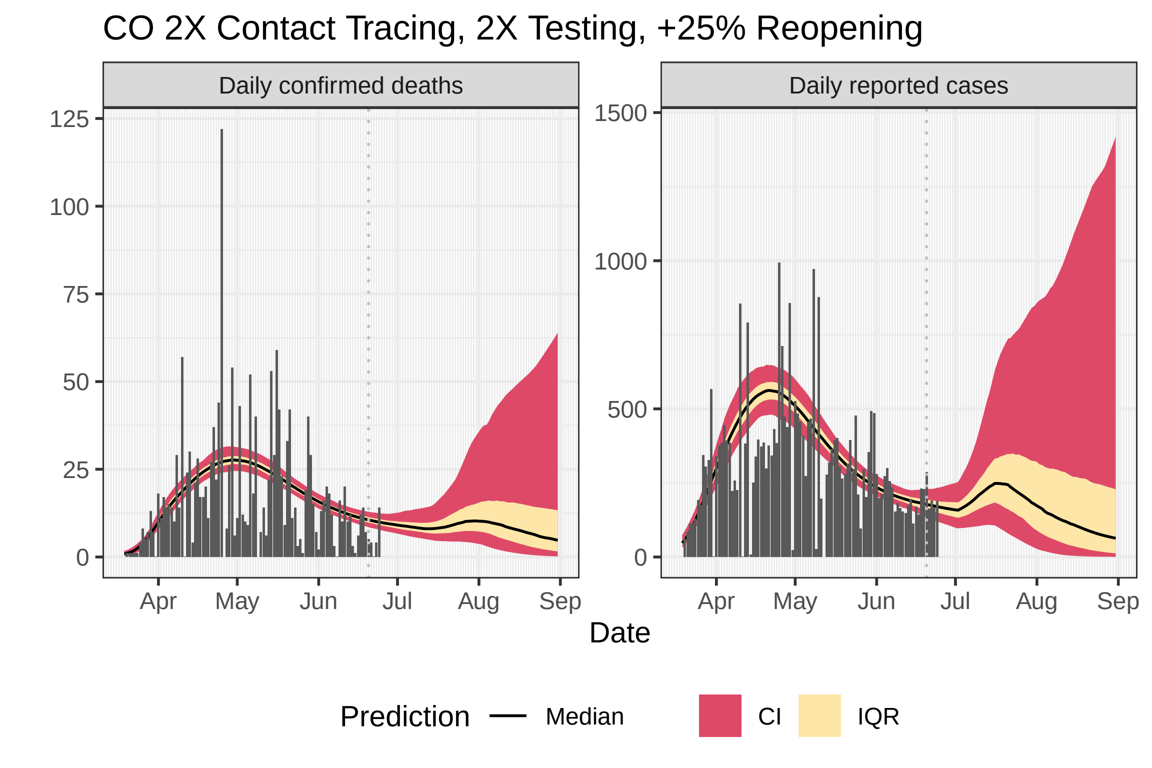

CO 2X Contact Tracing, 2X Testing, +25% Reopening
Daily confirmed deaths
Daily reported cases
125
100
75
50
25
0
1500
1000
500
0
Apr
May
Jun
Jul
Aug
Sep
Date
Prediction
Median
CI
IQR

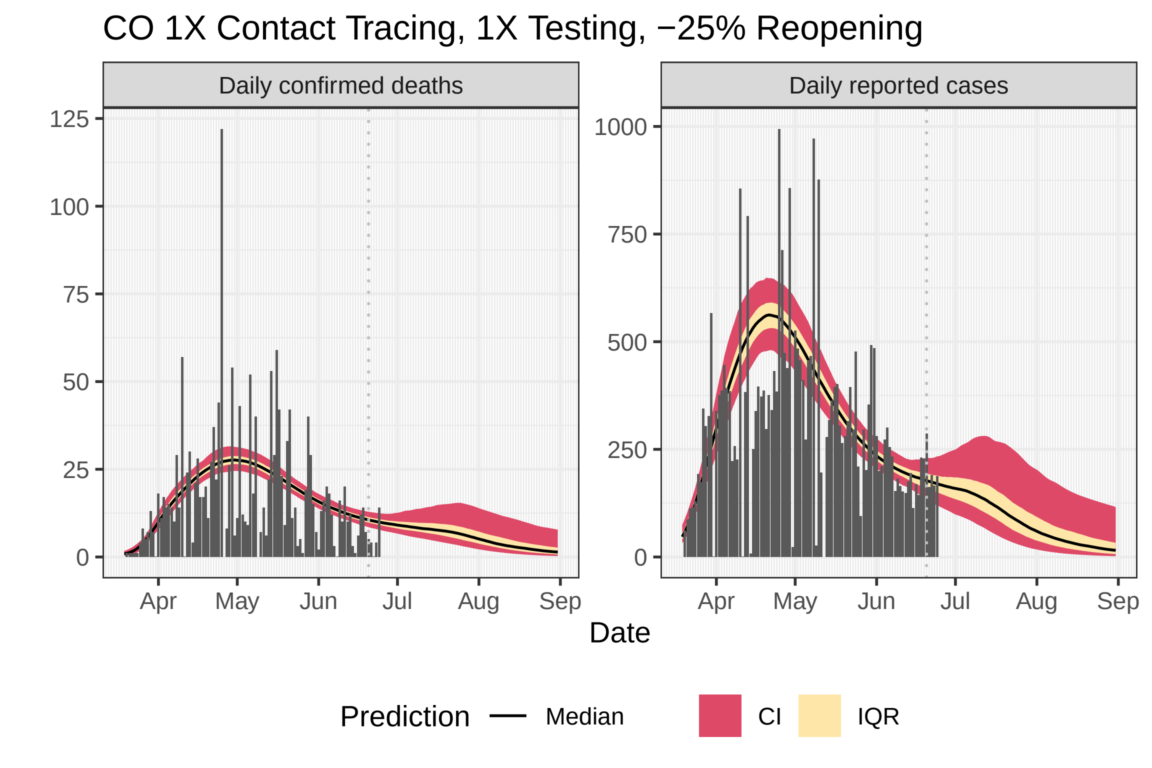

CO 1X Contact Tracing, 1X Testing, −25% Reopening
Daily confirmed deaths
Daily reported cases
125
100
75
50
25
0
1000
750
500
250
0
Apr
May
Jun
Jul
Aug
Sep
Date
Prediction
Median
CI
IQR

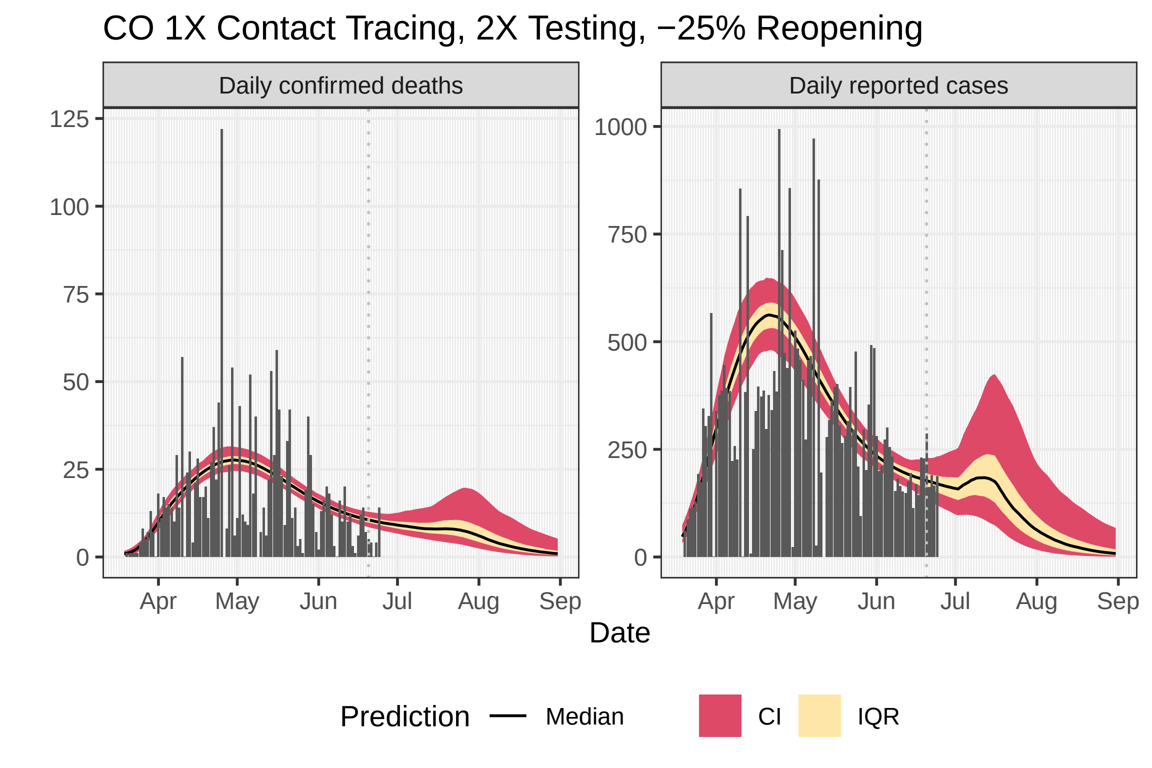
CO 1X Contact Tracing, 2X Testing, −25% Reopening
Daily confirmed deaths
Daily reported cases
125
100
75
50
25
0
1000
750
500
250
0
Apr
May
Jun
Jul
Aug
Sep
Date
Prediction
Median
CI
IQR

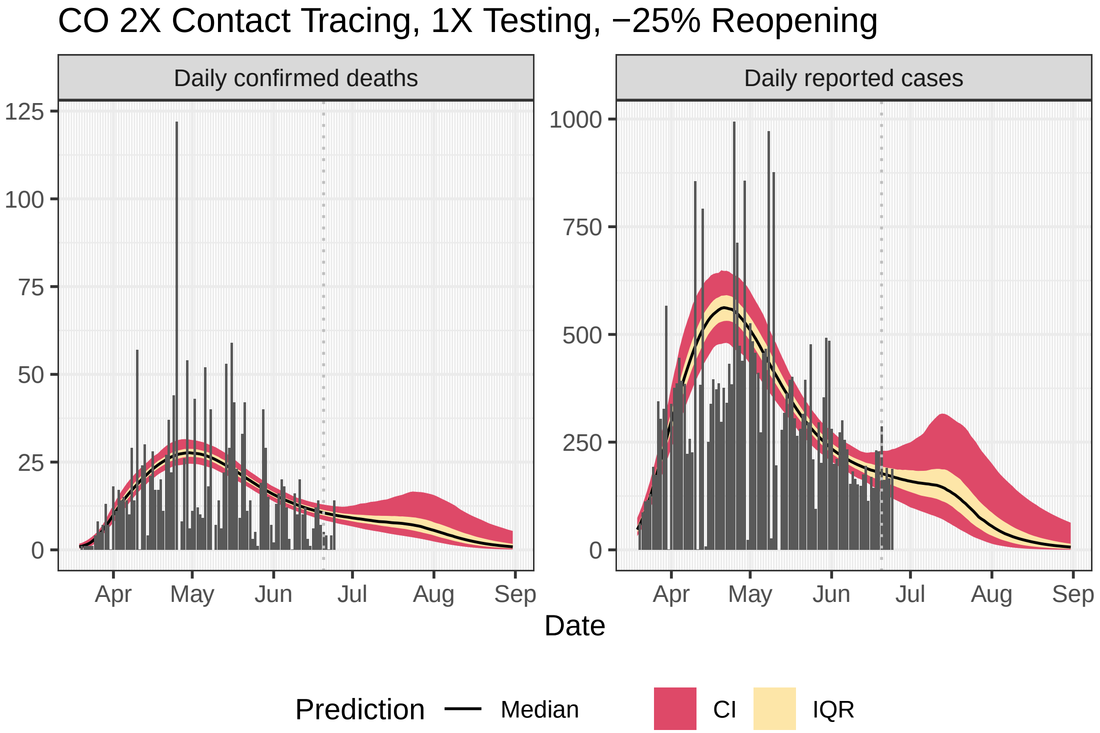

CO 2X Contact Tracing, 1X Testing, −25% Reopening
Daily confirmed deaths
Daily reported cases
125
100
75
50
25
0
1000
750
500
250
0
Apr
May
Jun
Jul
Aug
Sep
Date
Prediction
Median
CI
IQR

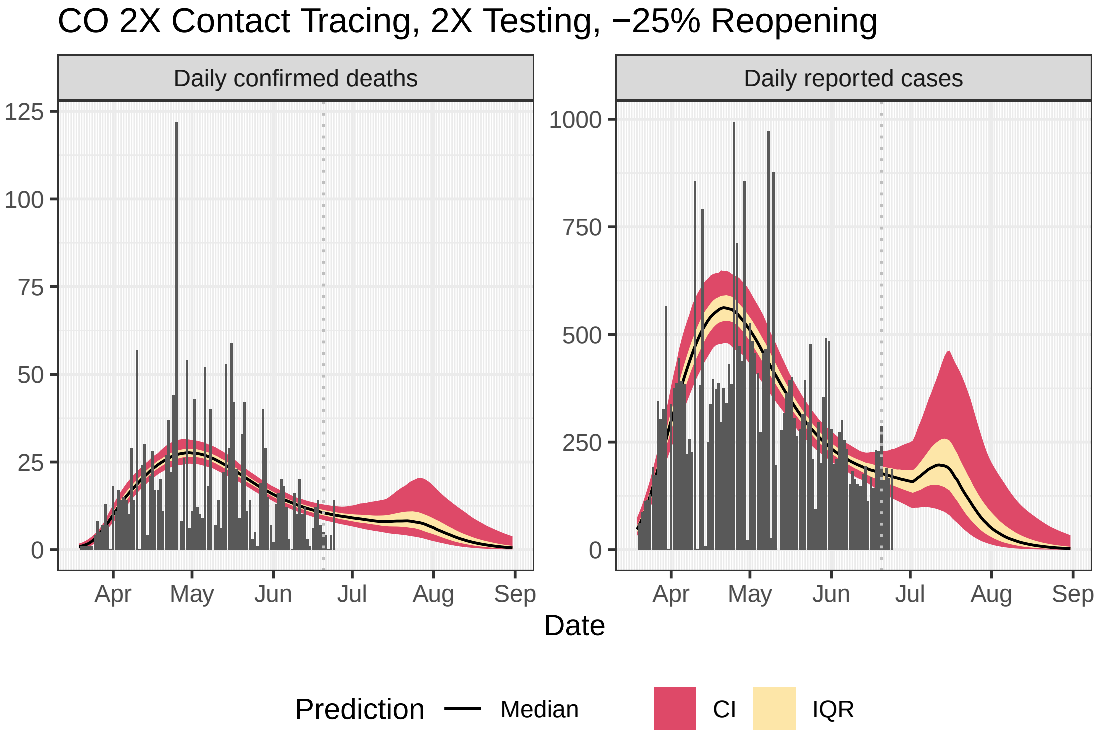

CO 2X Contact Tracing, 2X Testing, −25% Reopening
Daily confirmed deaths
Daily reported cases
125
100
75
50
25
0
1000
750
500
250
0
Apr
May
Jun
Jul
Aug
Sep
Date
Prediction
Median
CI
IQR

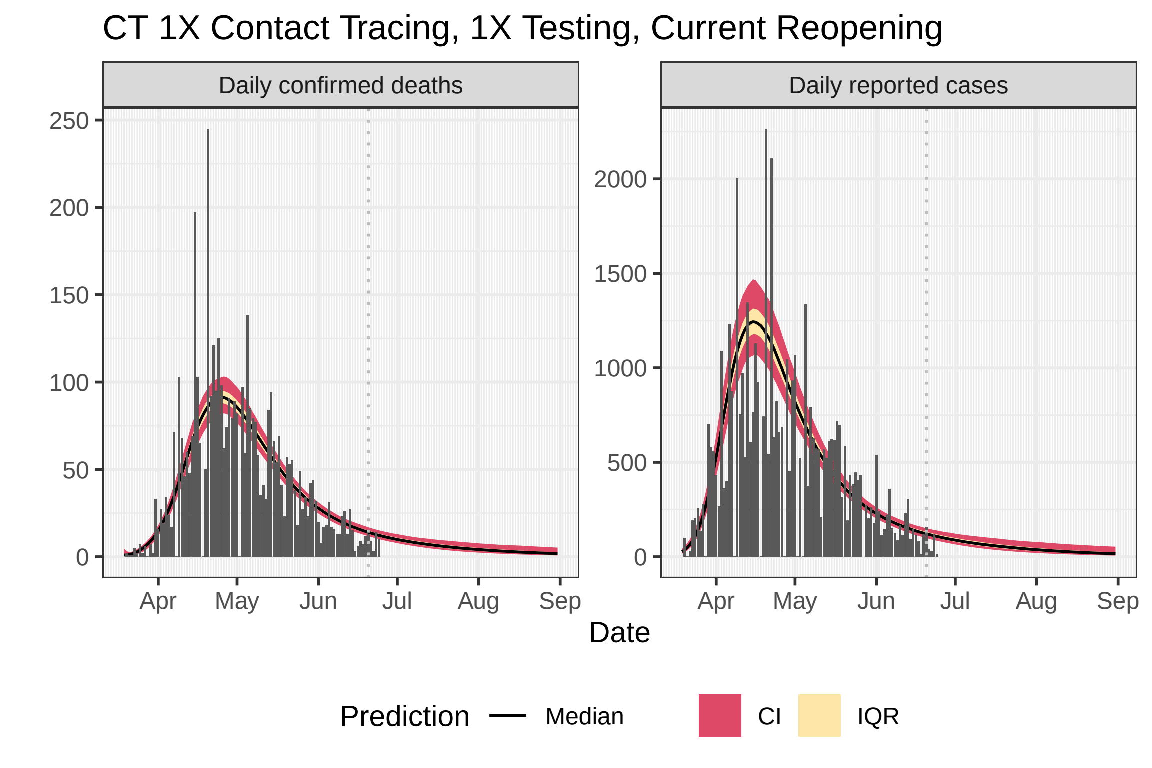

CT 1X Contact Tracing, 1X Testing, Current Reopening
Daily confirmed deaths
Daily reported cases
250
200
150
100
50
0
2000
1500
1000
500
0
Apr
May
Jun
Jul
Aug
Sep
Date
Prediction
Median
CI
IQR

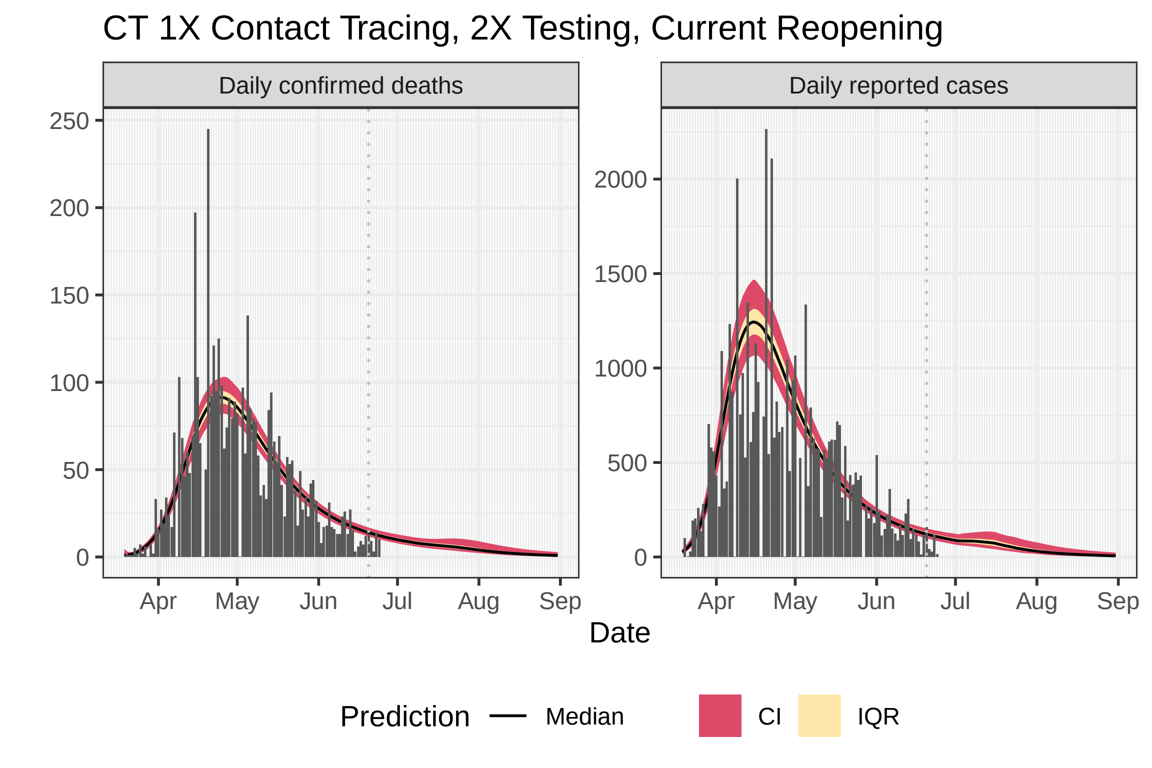

CT 1X Contact Tracing, 2X Testing, Current Reopening
Daily confirmed deaths
Daily reported cases
250
200
150
100
50
0
2000
1500
1000
500
0
Apr
May
Jun
Jul
Aug
Sep
Date
Prediction
Median
CI
IQR

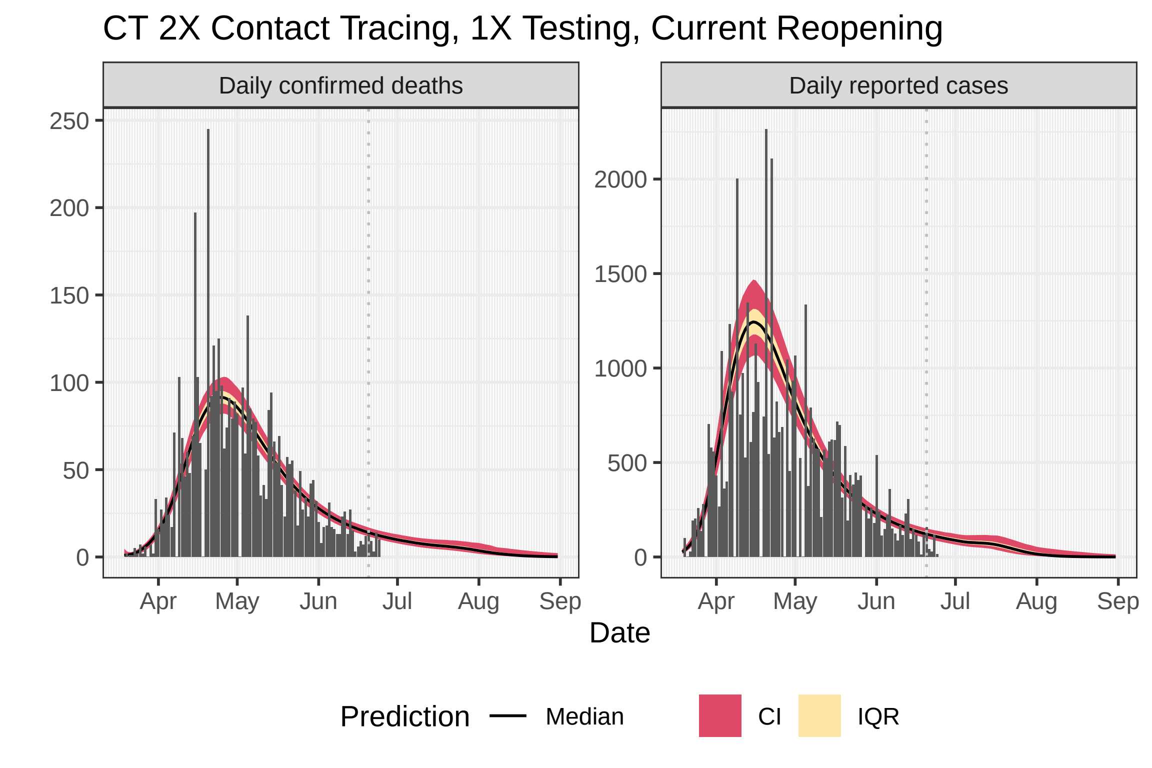
CT 2X Contact Tracing, 1X Testing, Current Reopening
Daily confirmed deaths
Daily reported cases
250
200
150
100
50
0
2000
1500
1000
500
0
Apr
May
Jun
Jul
Aug
Sep
Date
Prediction
Median
CI
IQR

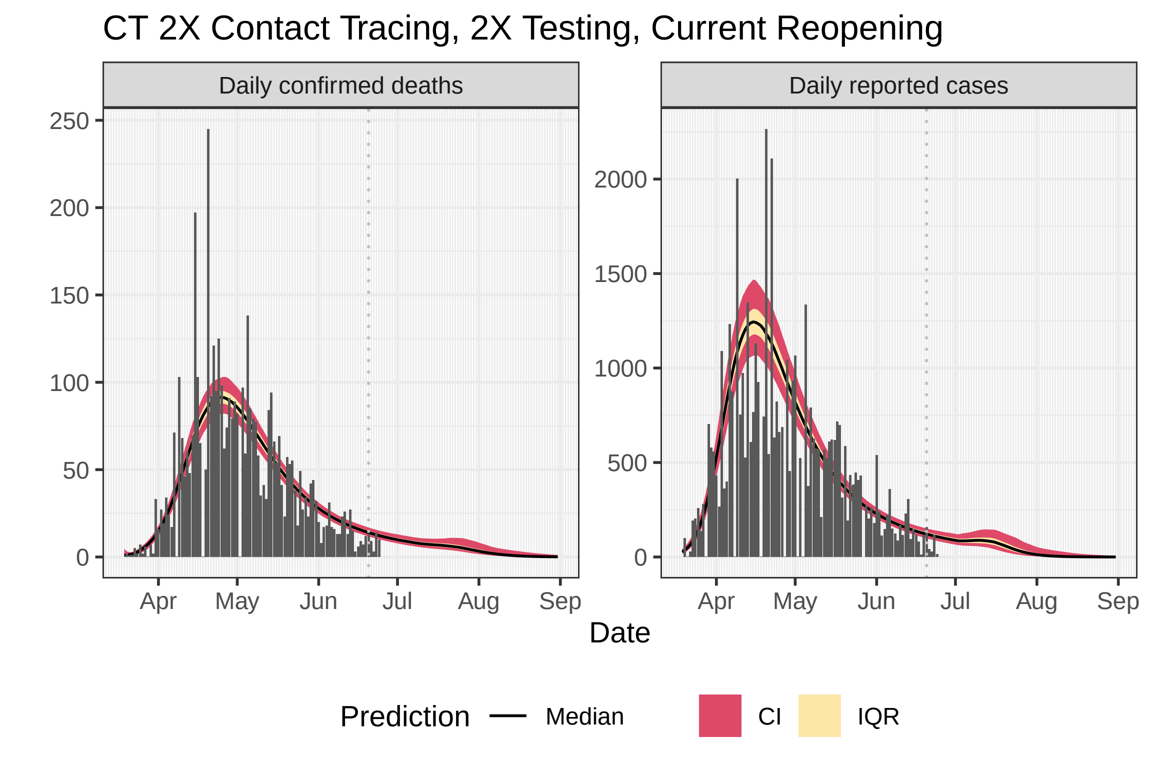
CT 2X Contact Tracing, 2X Testing, Current Reopening
Daily confirmed deaths
Daily reported cases
250
200
150
100
50
0
2000
1500
1000
500
0
Apr
May
Jun
Jul
Aug
Sep
Date
Prediction
Median
CI
IQR

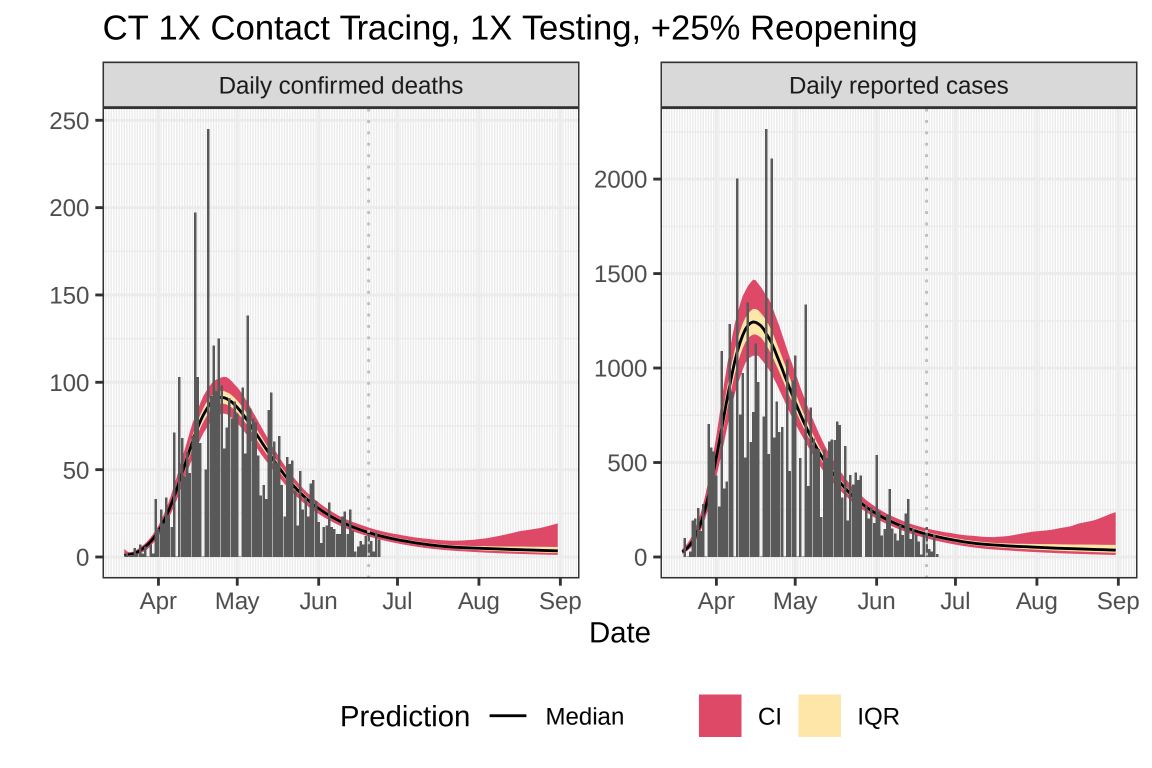

CT 1X Contact Tracing, 1X Testing, +25% Reopening
Daily confirmed deaths
Daily reported cases
250
200
150
100
50
0
2000
1500
1000
500
0
Apr
May
Jun
Jul
Aug
Sep
Date
Prediction
Median
CI
IQR

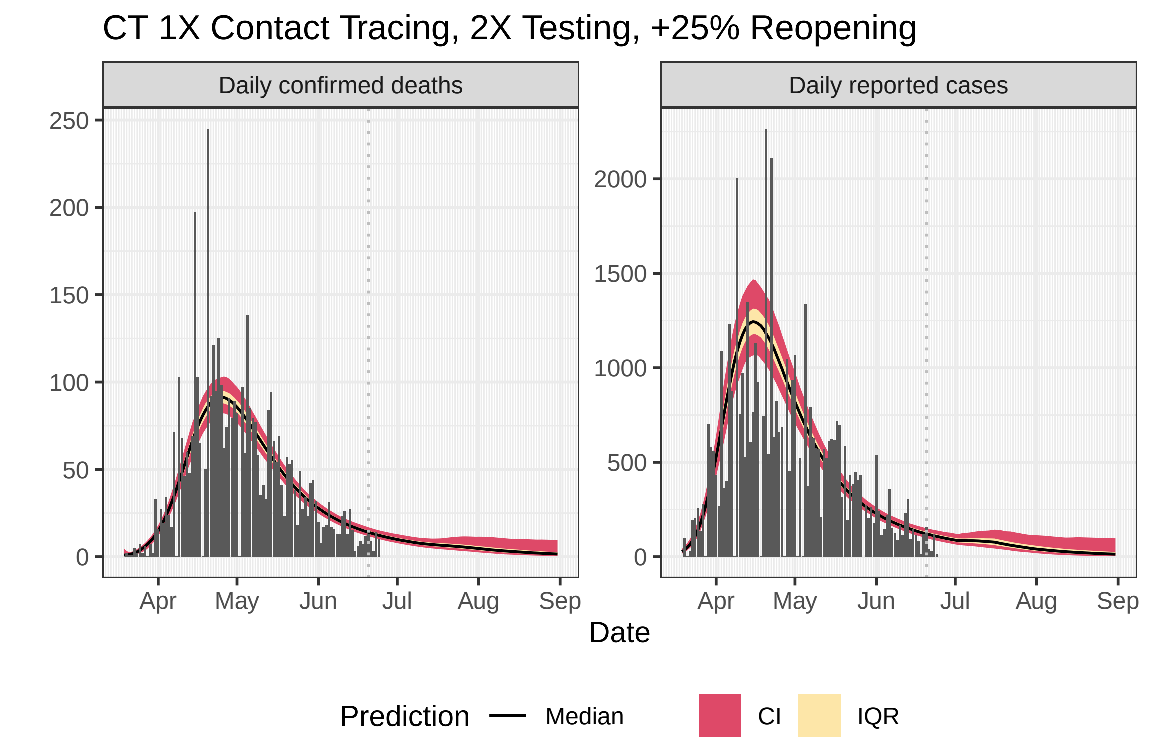

CT 1X Contact Tracing, 2X Testing, +25% Reopening
Daily confirmed deaths
Daily reported cases
250
200
150
100
50
0
2000
1500
1000
500
0
Apr
May
Jun
Jul
Aug
Sep
Date
Prediction
Median
CI
IQR

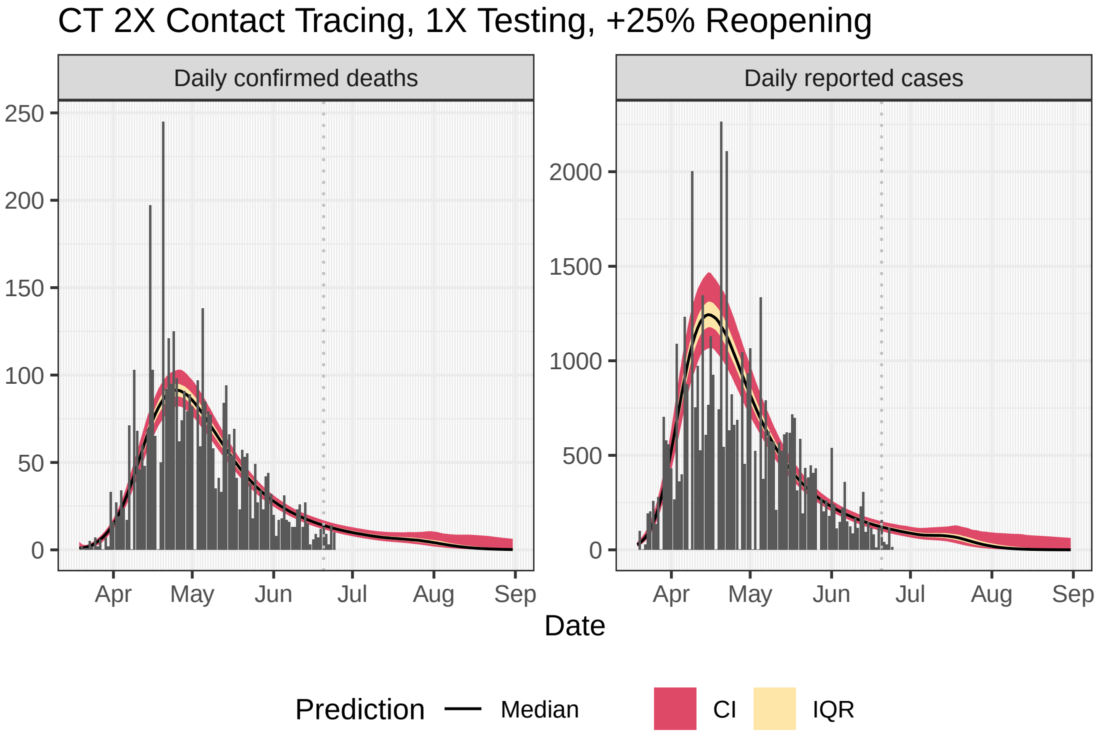
CT 2X Contact Tracing, 1X Testing, +25% Reopening
Daily confirmed deaths
Daily reported cases
250
200
150
100
50
0
2000
1500
1000
500
0
Apr
May
Jun
Jul
Aug
Sep
Date
Prediction
Median
CI
IQR

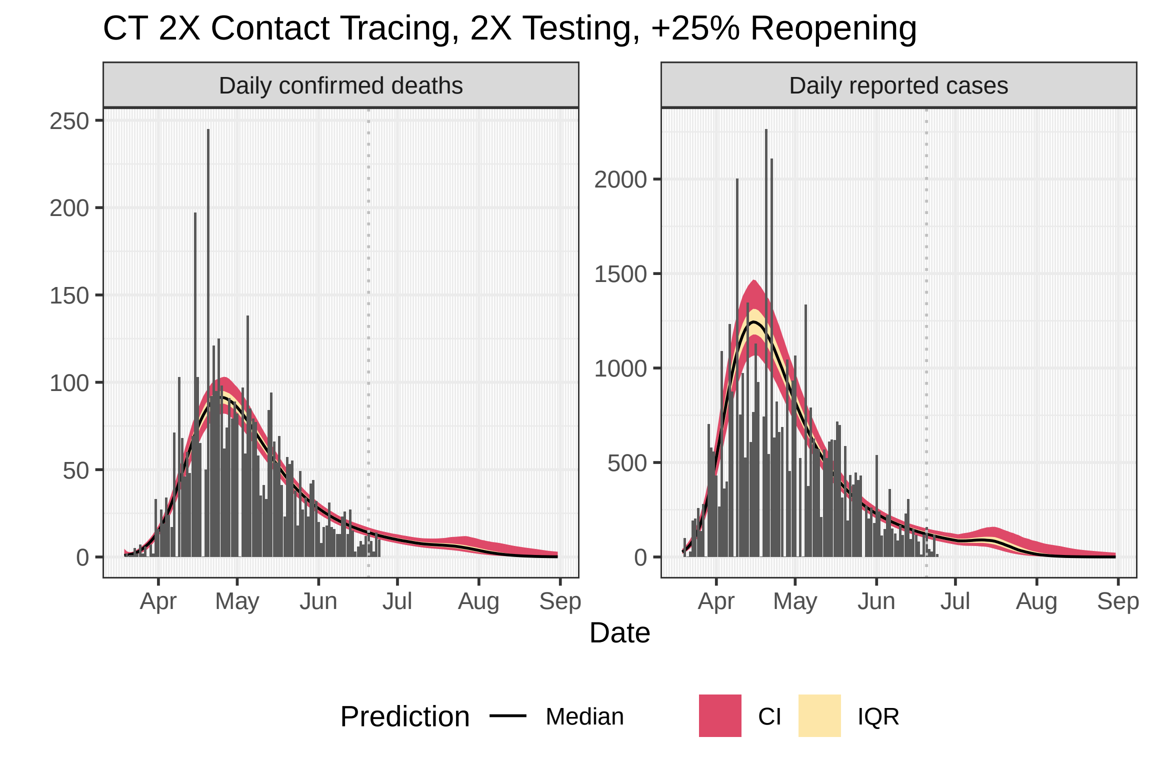
CT 2X Contact Tracing, 2X Testing, +25% Reopening
Daily confirmed deaths
Daily reported cases
250
200
150
100
50
0
2000
1500
1000
500
0
Apr
May
Jun
Jul
Aug
Sep
Date
Prediction
Median
CI
IQR

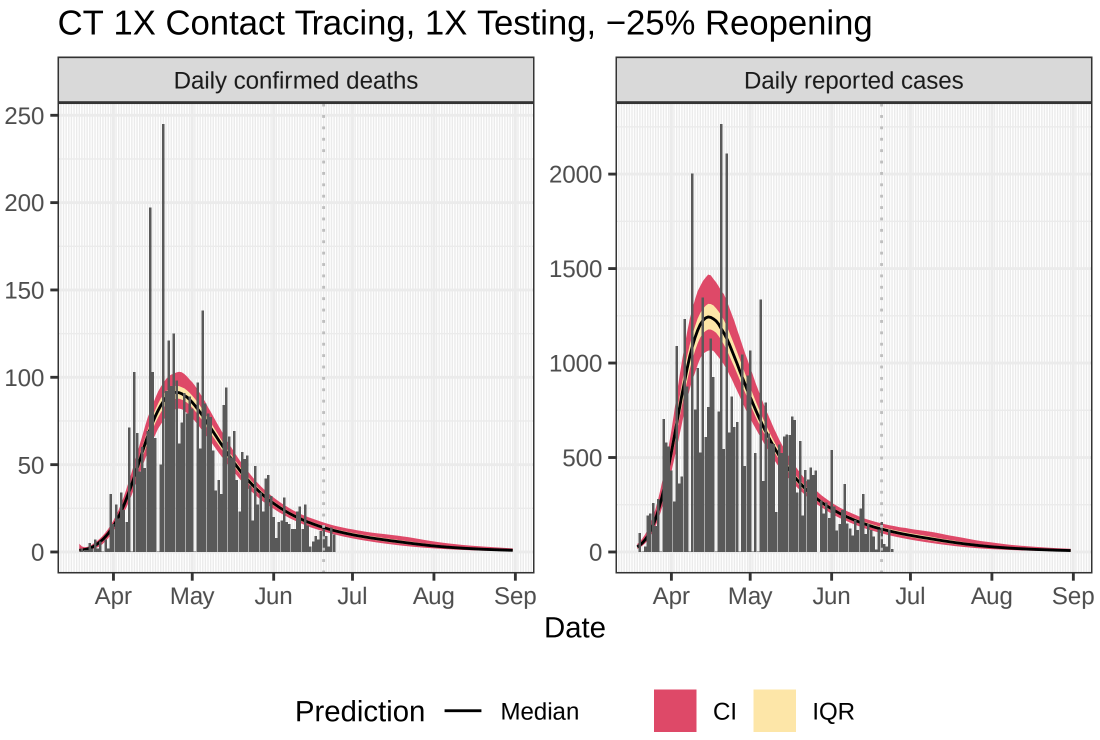

CT 1X Contact Tracing, 1X Testing, −25% Reopening
Daily confirmed deaths
Daily reported cases
250
200
150
100
50
0
2000
1500
1000
500
0
Apr
May
Jun
Jul
Aug
Sep
Date
Prediction
Median
CI
IQR

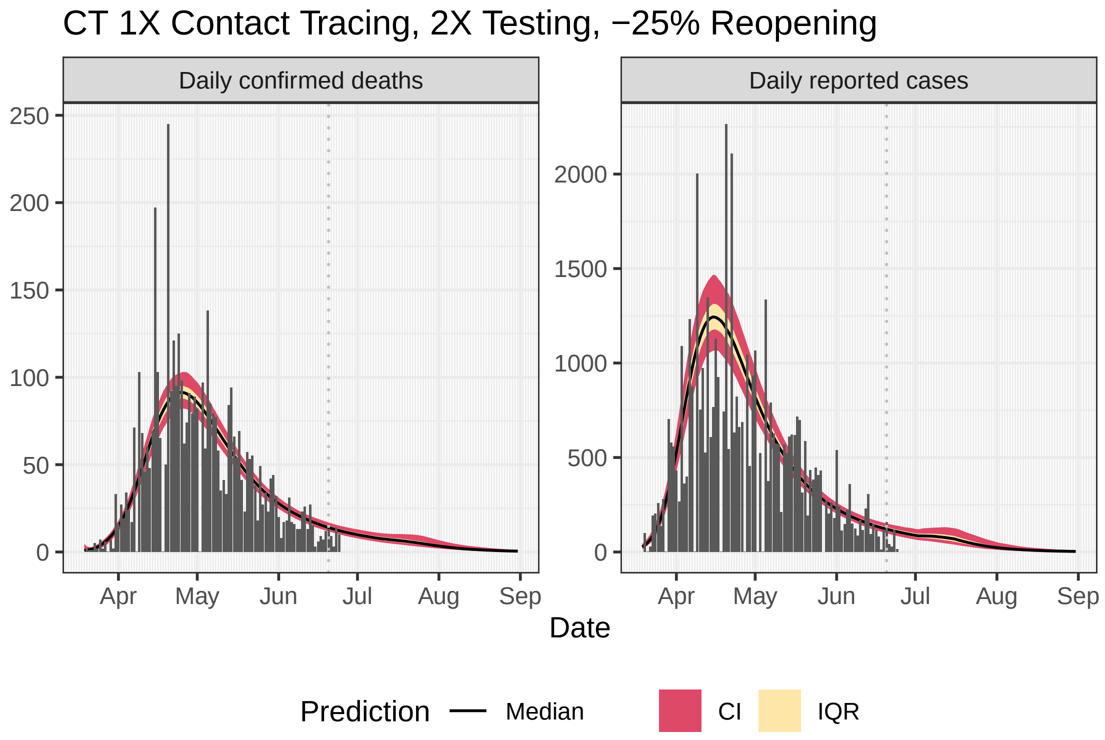

CT 1X Contact Tracing, 2X Testing, −25% Reopening
Daily confirmed deaths
Daily reported cases
250
200
150
100
50
0
2000
1500
1000
500
0
Apr
May
Jun
Jul
Aug
Sep
Date
Prediction
Median
CI
IQR

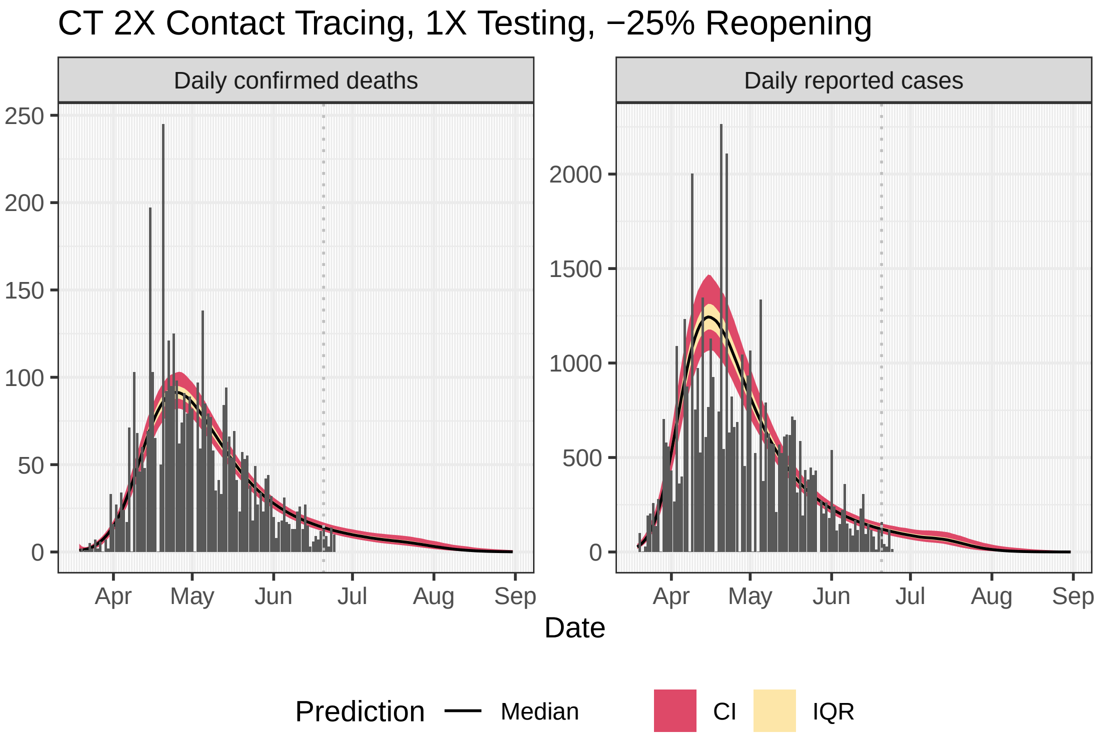

CT 2X Contact Tracing, 1X Testing, −25% Reopening
Daily confirmed deaths
Daily reported cases
250
200
150
100
50
0
2000
1500
1000
500
0
Apr
May
Jun
Jul
Aug
Sep
Date
Prediction
Median
CI
IQR

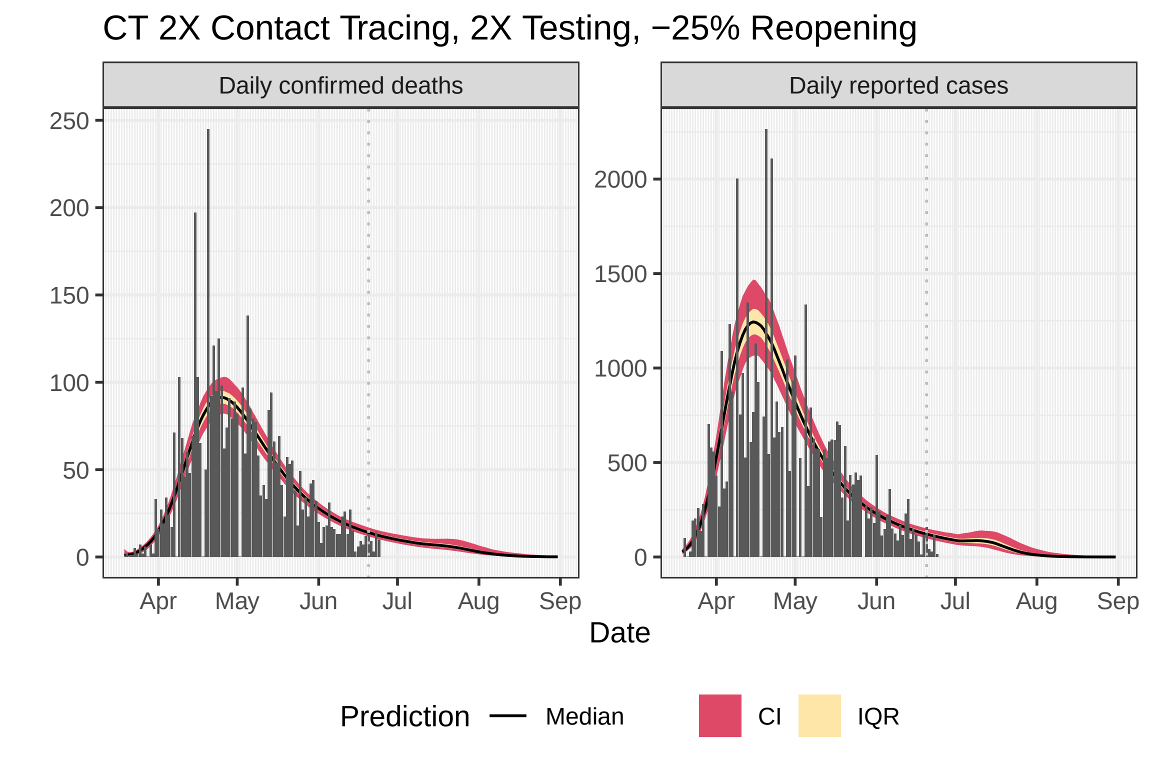

CT 2X Contact Tracing, 2X Testing, −25% Reopening
Daily confirmed deaths
Daily reported cases
250
200
150
100
50
0
2000
1500
1000
500
0
Apr
May
Jun
Jul
Aug
Sep
Date
Prediction
Median
CI
IQR

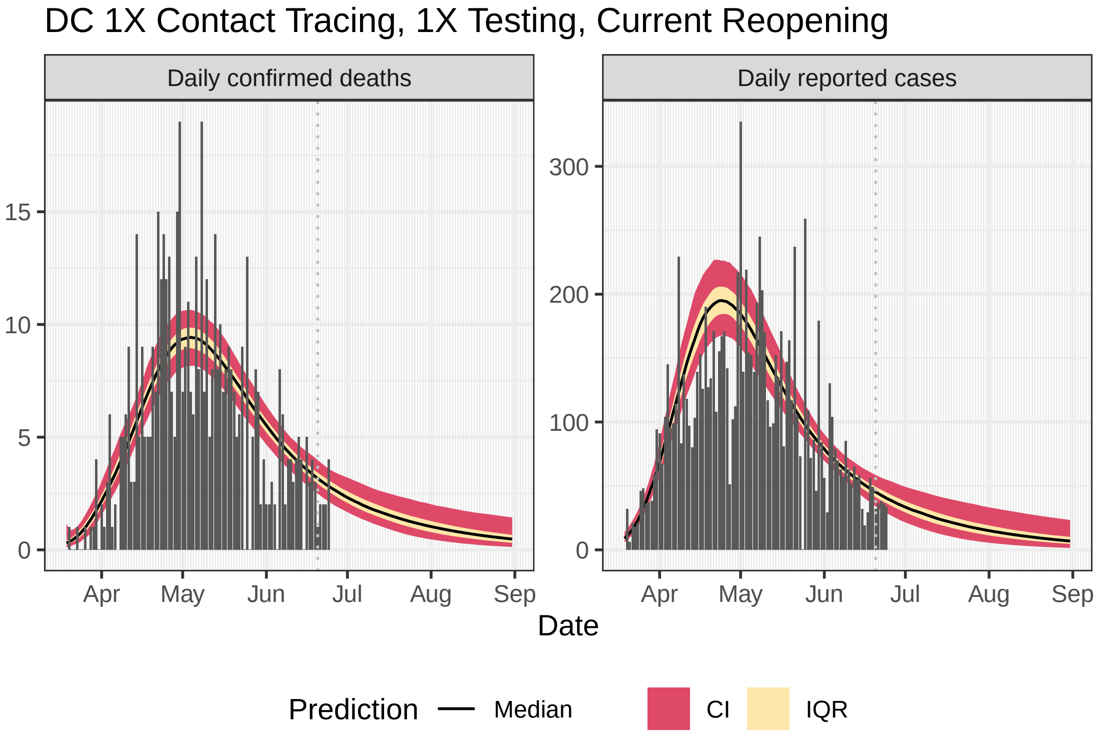
DC 1X Contact Tracing, 1X Testing, Current Reopening
Daily confirmed deaths
Daily reported cases
0
5
10
15
0
100
200
300
Apr
May
Jun
Jul
Aug
Sep
Date
Prediction
Median
CI
IQR

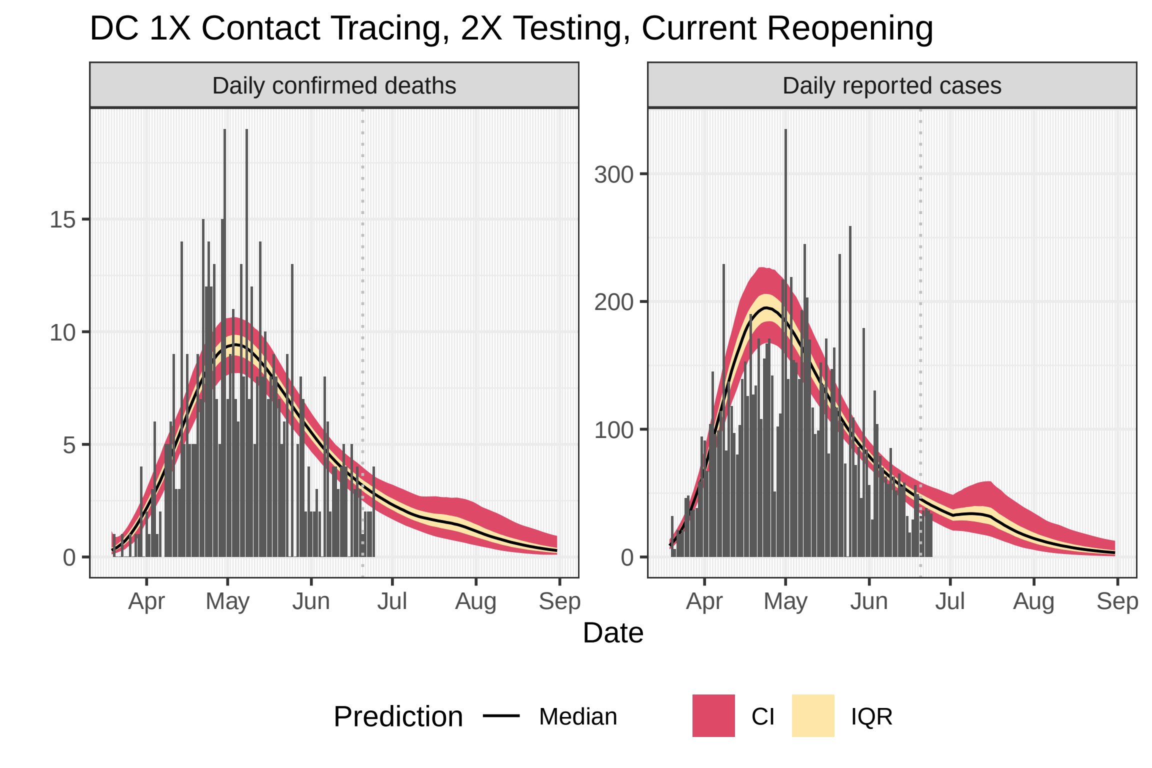

DC 1X Contact Tracing, 2X Testing, Current Reopening
Daily confirmed deaths
Daily reported cases
0
5
10
15
0
100
200
300
Apr
May
Jun
Jul
Aug
Sep
Date
Prediction
Median
CI
IQR

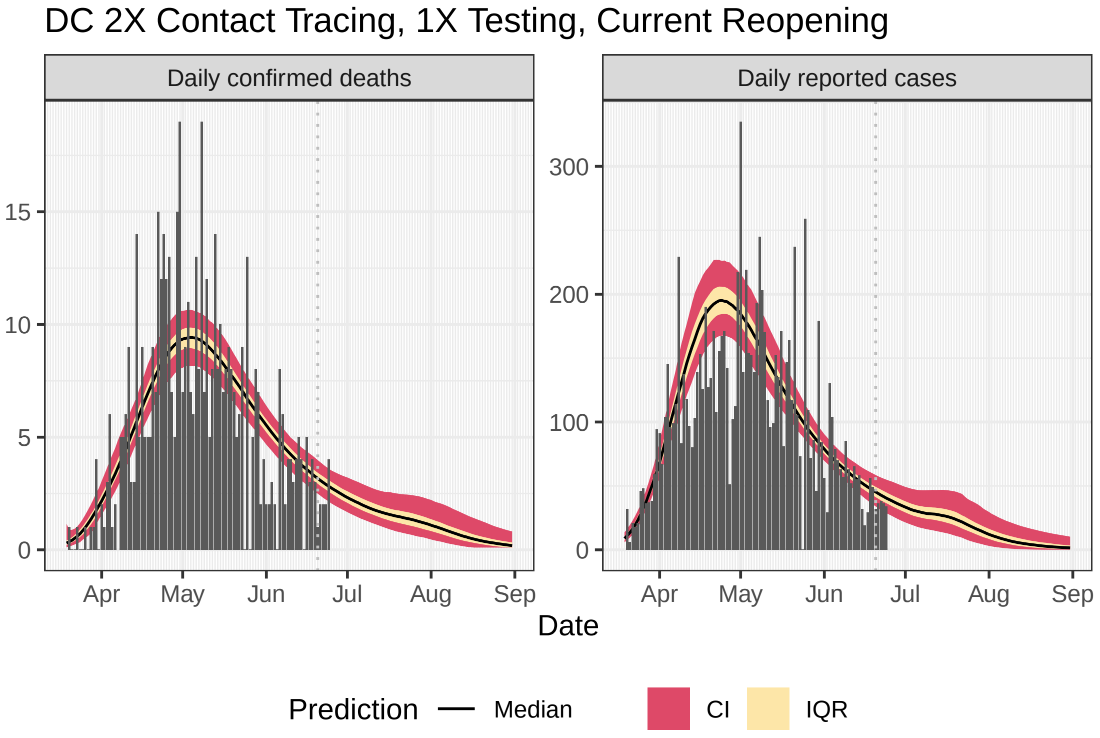

DC 2X Contact Tracing, 1X Testing, Current Reopening
Daily confirmed deaths
Daily reported cases
0
5
10
15
0
100
200
300
Apr
May
Jun
Jul
Aug
Sep
Date
Prediction
Median
CI
IQR

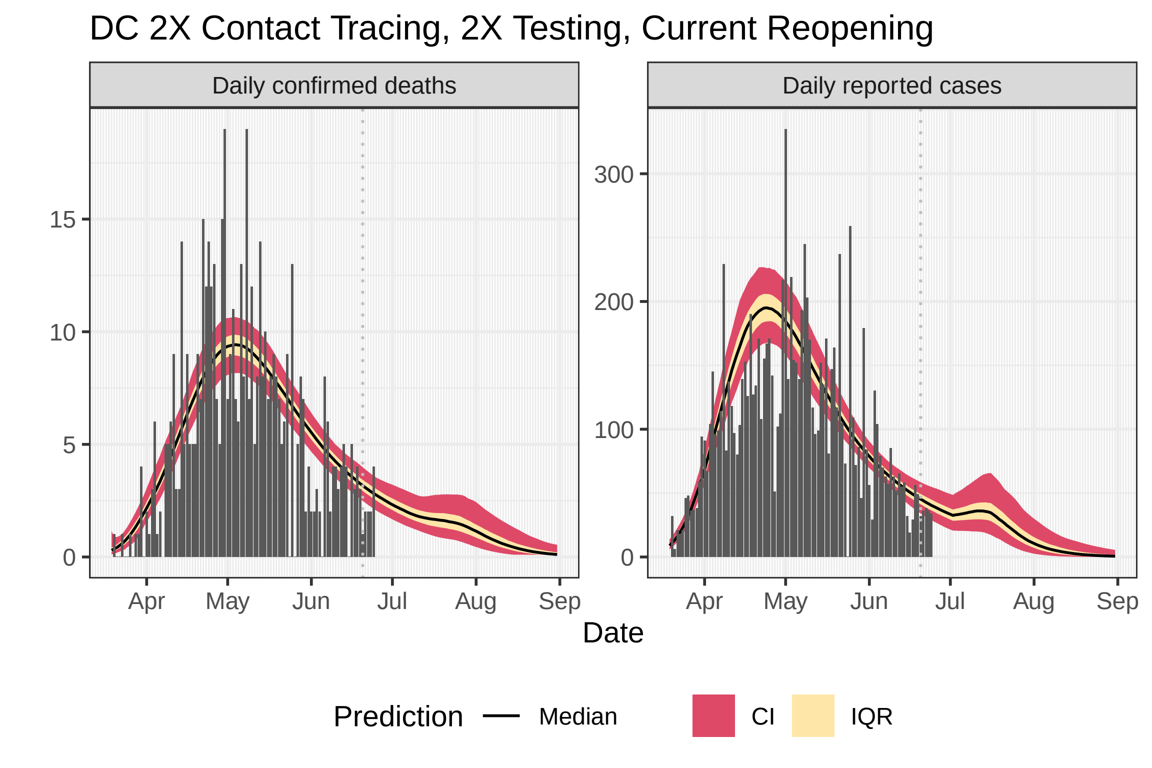

DC 2X Contact Tracing, 2X Testing, Current Reopening
Daily confirmed deaths
Daily reported cases
0
5
10
15
100
200
300
Apr
May
Jun
Jul
Aug
Sep
Date
Prediction
Median
CI
IQR

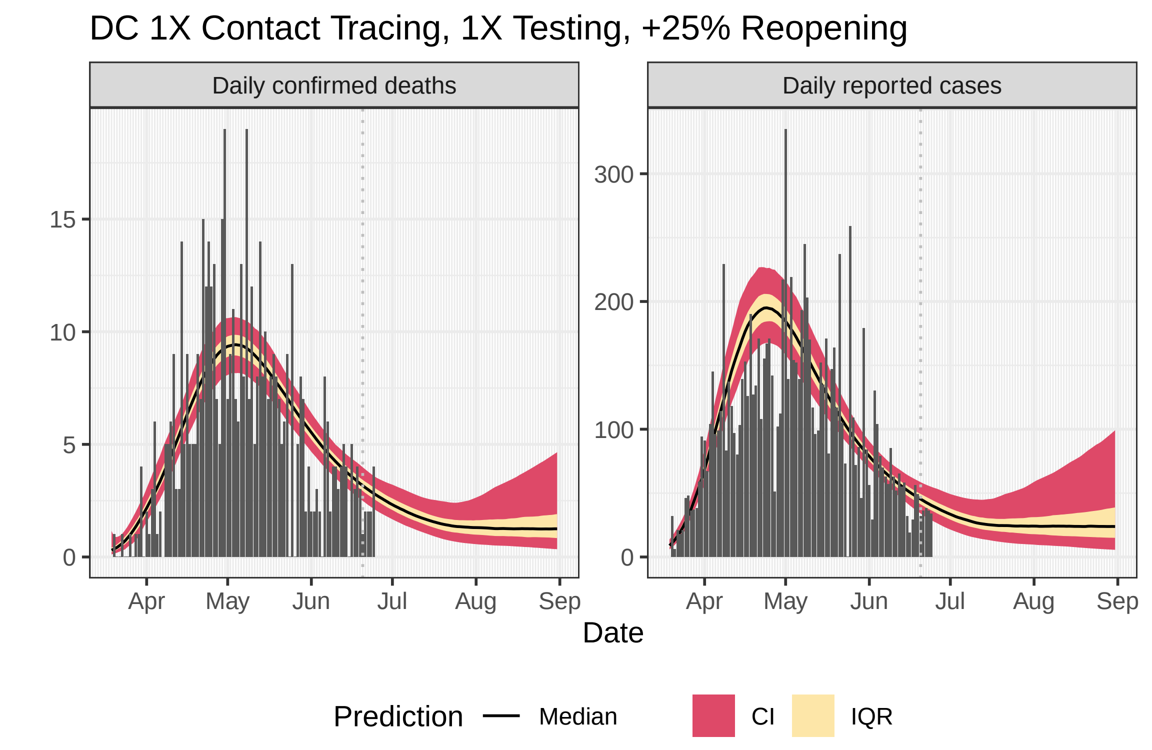
DC 1X Contact Tracing, 1X Testing, +25% Reopening
Daily confirmed deaths
Daily reported cases
0
5
10
15
0
100
200
300
Apr
May
Jun
Jul
Aug
Sep
Date
Prediction
Median
CI
IQR

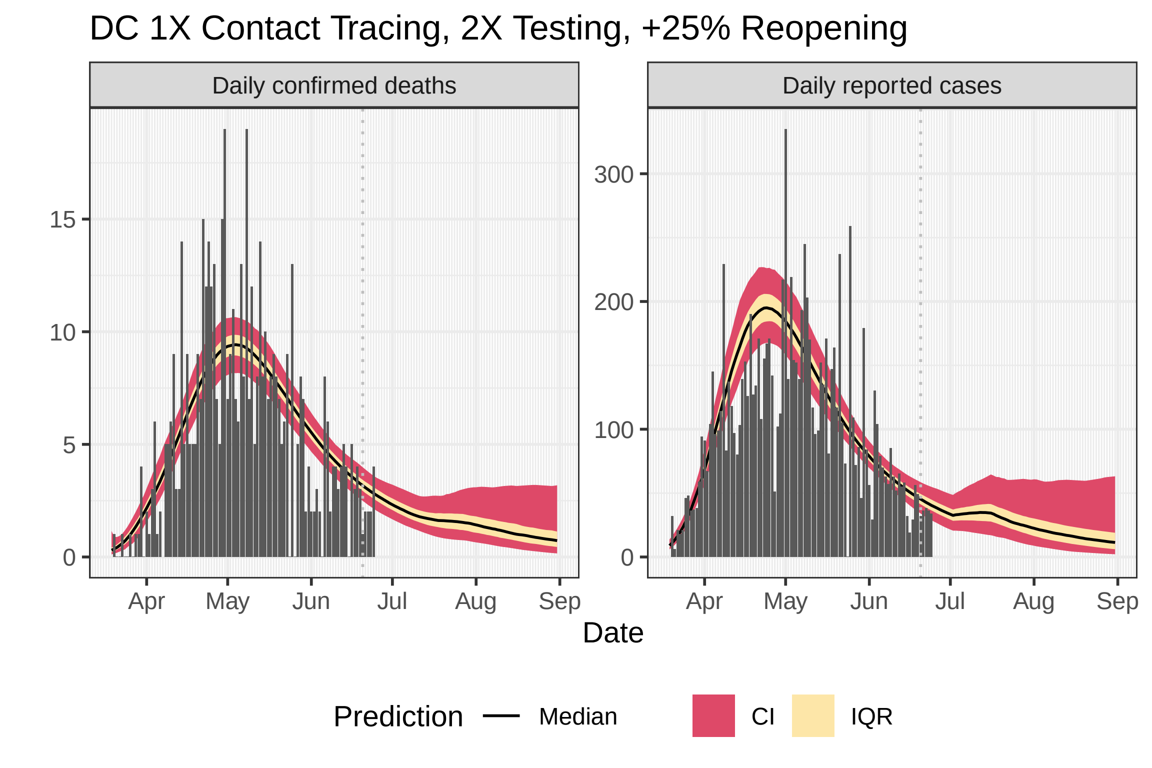
DC 1X Contact Tracing, 2X Testing, +25% Reopening
Daily confirmed deaths
Daily reported cases
0
5
10
15
0
100
200
300
Apr
May
Jun
Jul
Aug
Sep
Date
Prediction
Median
CI
IQR

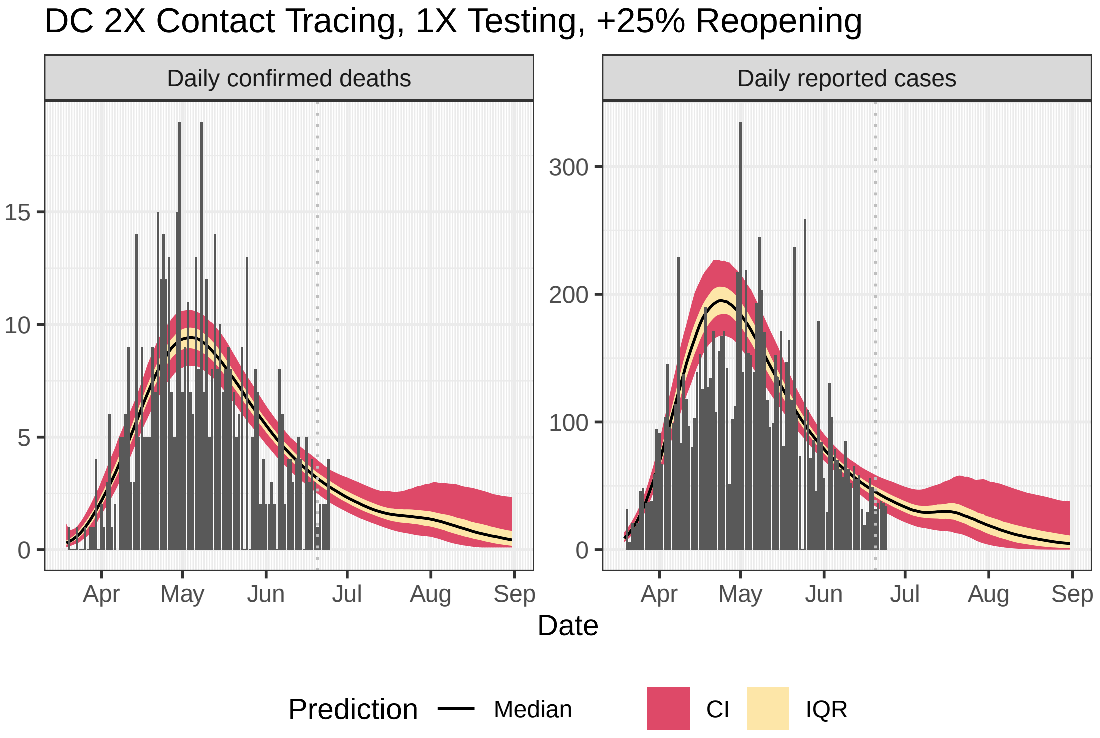
DC 2X Contact Tracing, 1X Testing, +25% Reopening
Daily confirmed deaths
Daily reported cases
0
5
10
15
0
100
200
300
Apr
May
Jun
Jul
Aug
Sep
Date
Prediction
Median
CI
IQR

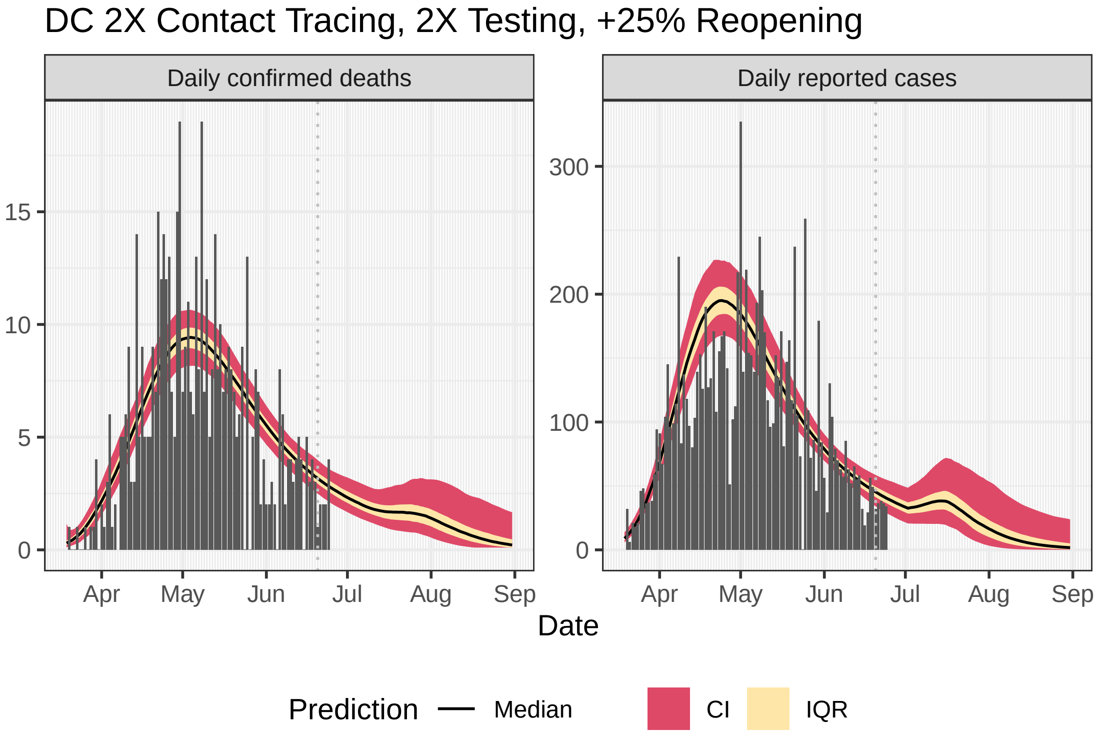
DC 2X Contact Tracing, 2X Testing, +25% Reopening
Daily confirmed deaths
Daily reported cases
15
10
5
0
300
200
100
0
Apr
May
Jun
Jul
Aug
Sep
Date
Prediction
Median
CI
IQR

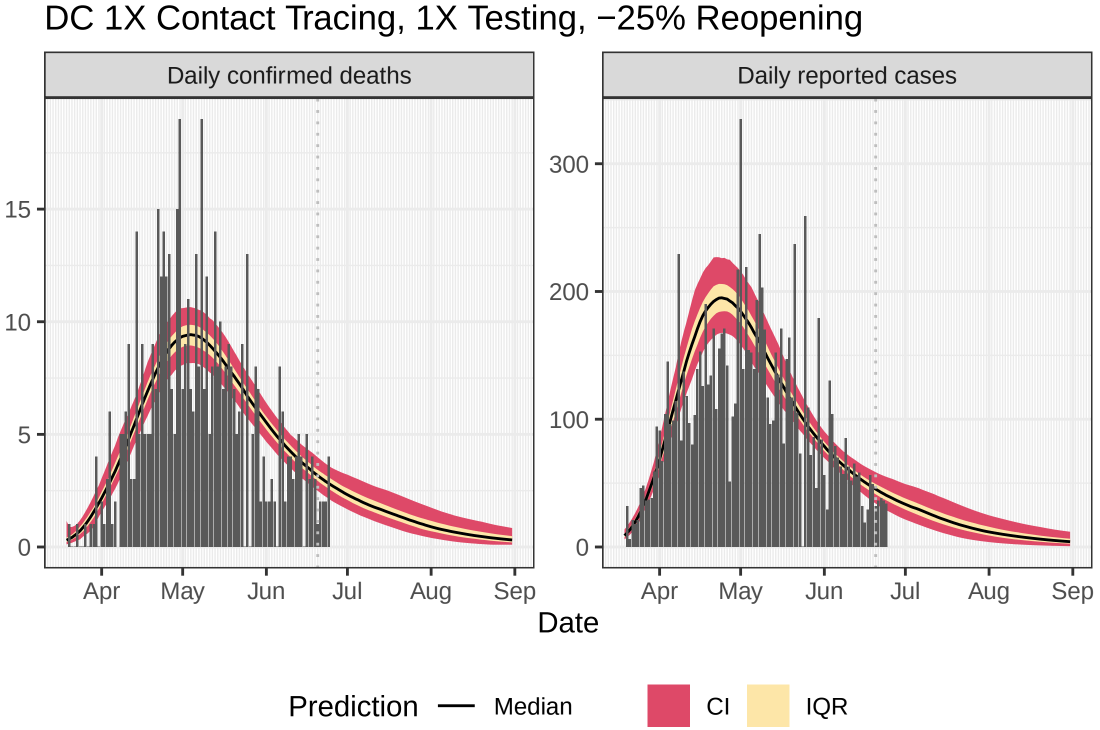
DC 1X Contact Tracing, 1X Testing, −25% Reopening
Daily confirmed deaths
Daily reported cases
15
10
5
0
300
200
100
0
Apr
May
Jun
Jul
Aug
Sep
Date
Prediction
Median
CI
IQR

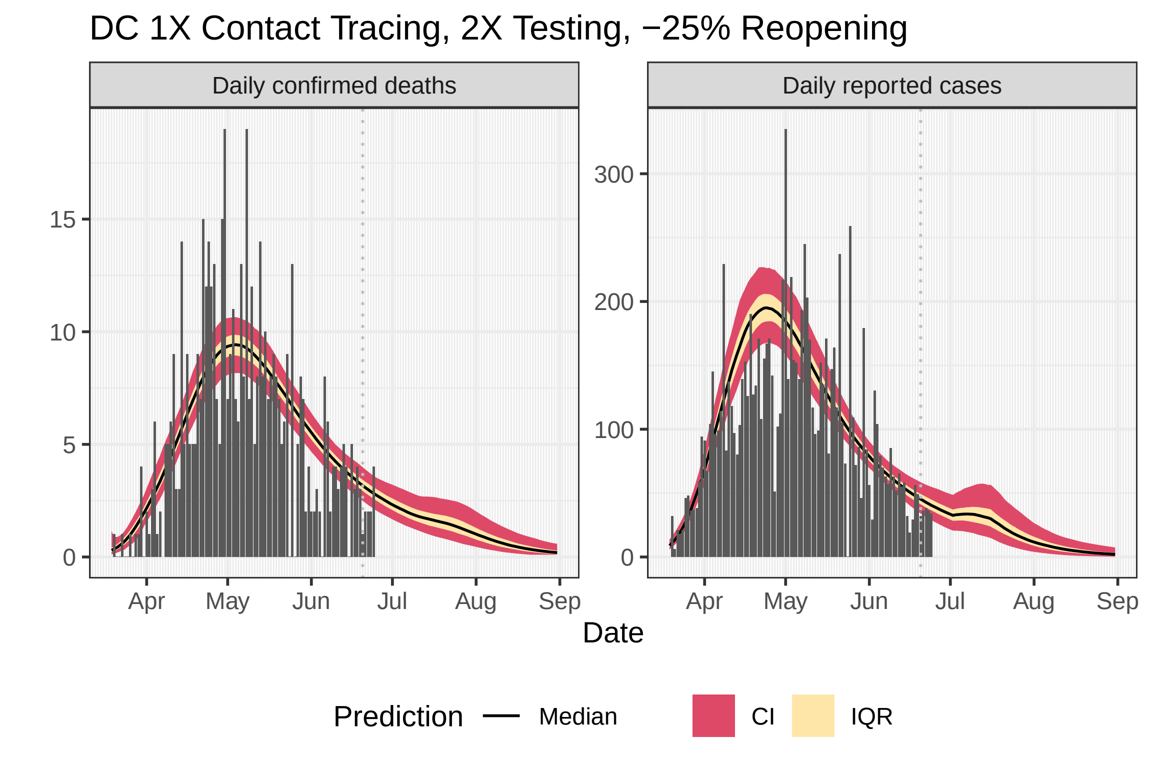

DC 1X Contact Tracing, 2X Testing, −25% Reopening
Daily confirmed deaths
Daily reported cases
0
5
10
15
0
100
200
300
Apr
May
Jun
Jul
Aug
Sep
Date
Prediction
Median
CI
IQR

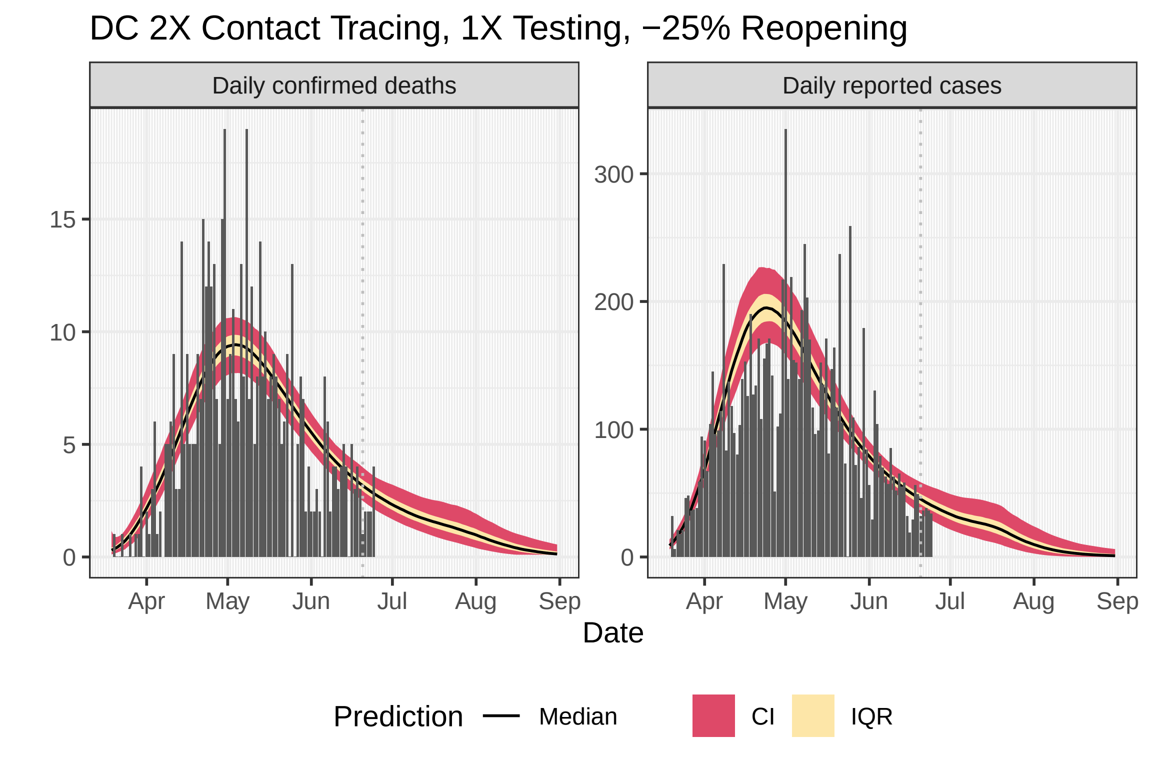

DC 2X Contact Tracing, 1X Testing, −25% Reopening
Daily confirmed deaths
Daily reported cases
0
5
10
15
100
200
300
Apr
May
Jun
Jul
Aug
Sep
Date
Prediction
Median
CI
IQR

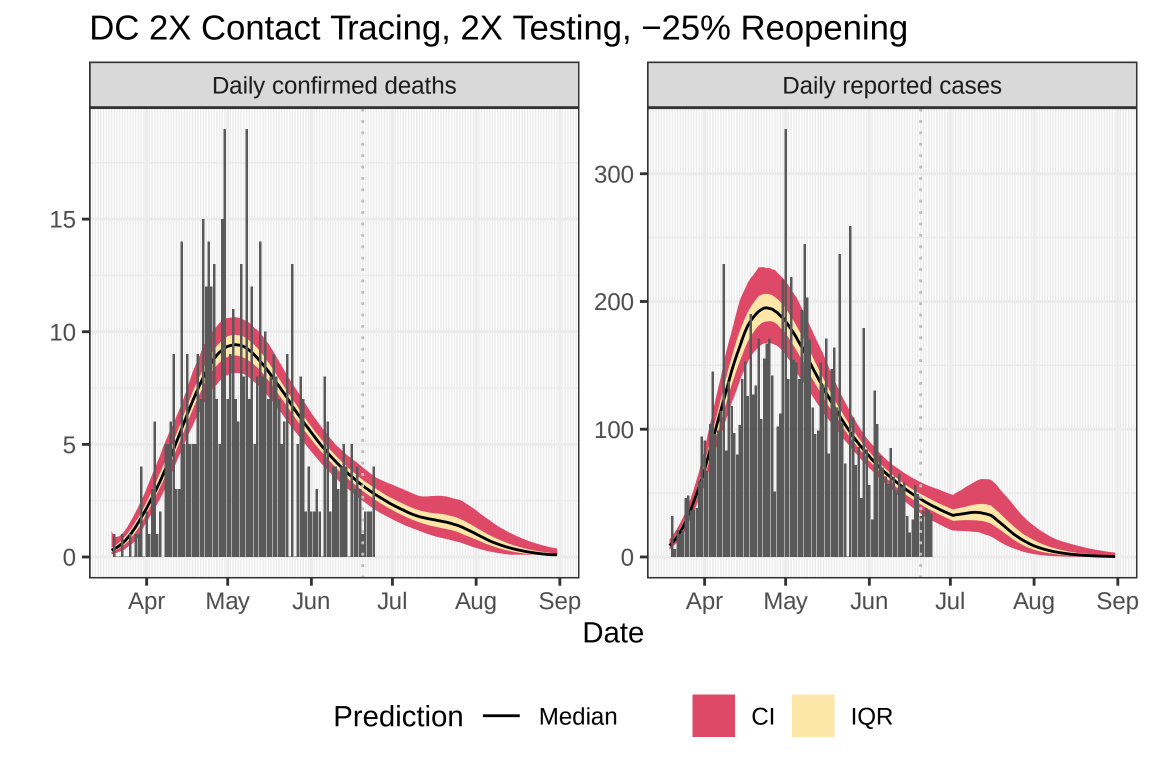

DC 2X Contact Tracing, 2X Testing, −25% Reopening
Daily confirmed deaths
Daily reported cases
15
10
5
0
300
200
100
0
Apr
May
Jun
Jul
Aug
Sep
Date
Prediction
Median
CI
IQR

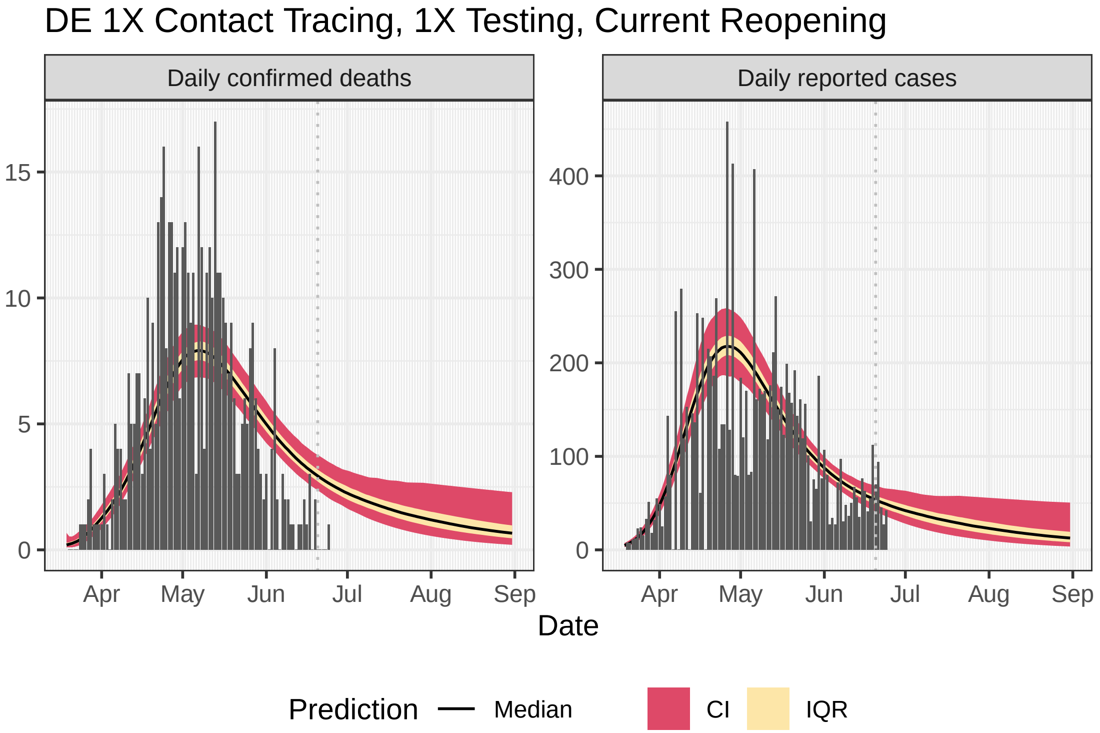
DE 1X Contact Tracing, 1X Testing, Current Reopening
Daily confirmed deaths
Daily reported cases
0
5
10
15
0
100
200
300
400
Apr
May
Jun
Jul
Aug
Sep
Date
Prediction
Median
CI
IQR

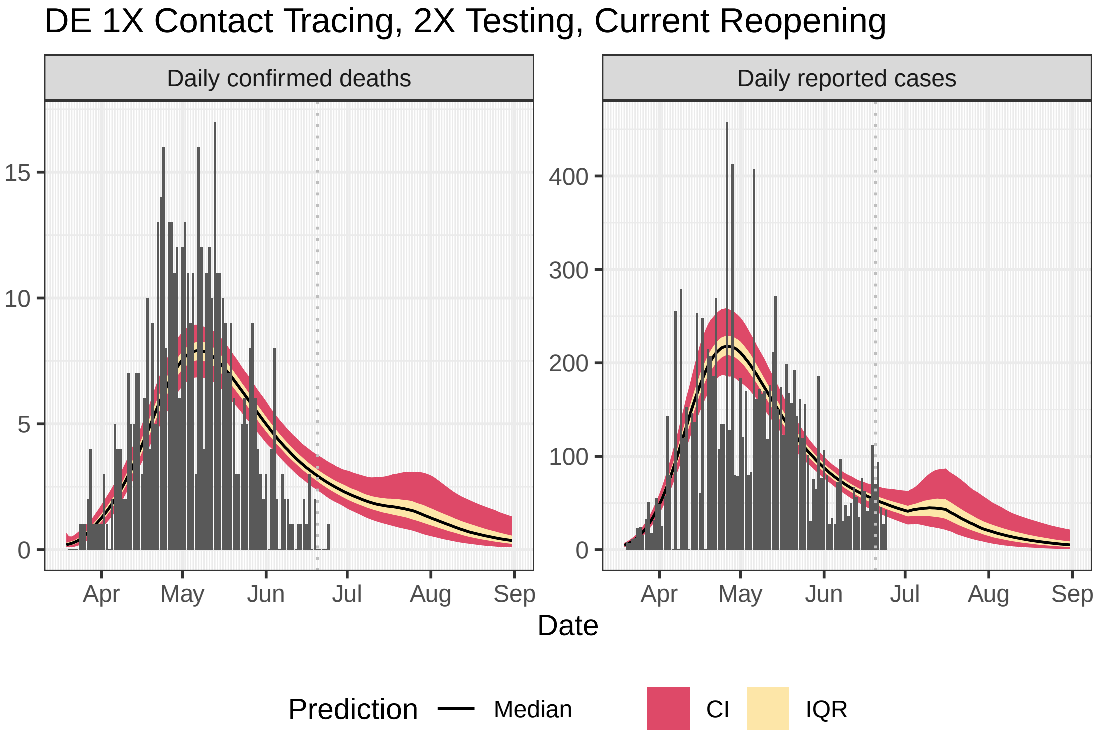
DE 1X Contact Tracing, 2X Testing, Current Reopening
Daily confirmed deaths
Daily reported cases
0
5
10
15
0
100
200
300
400
Apr
May
Jun
Jul
Aug
Sep
Date
Prediction
Median
CI
IQR

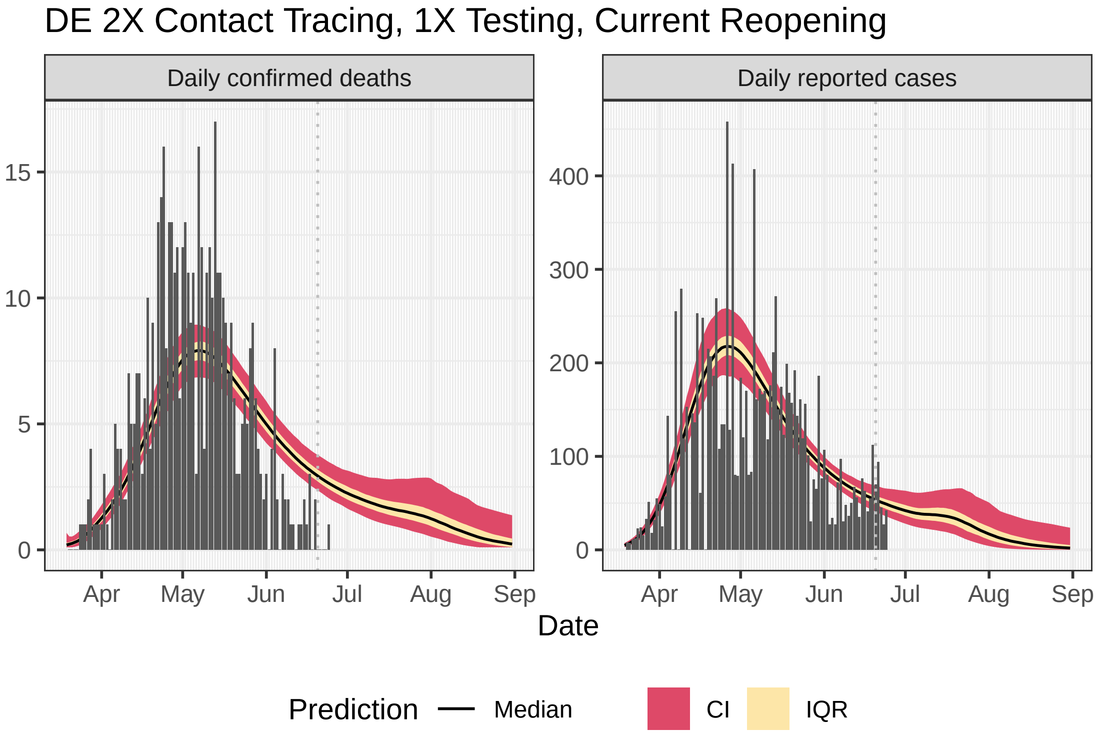
DE 2X Contact Tracing, 1X Testing, Current Reopening
Daily confirmed deaths
Daily reported cases
15
10
5
0
400
300
200
100
0
Apr
May
Jun
Jul
Aug
Sep
Date
Prediction
Median
CI
IQR

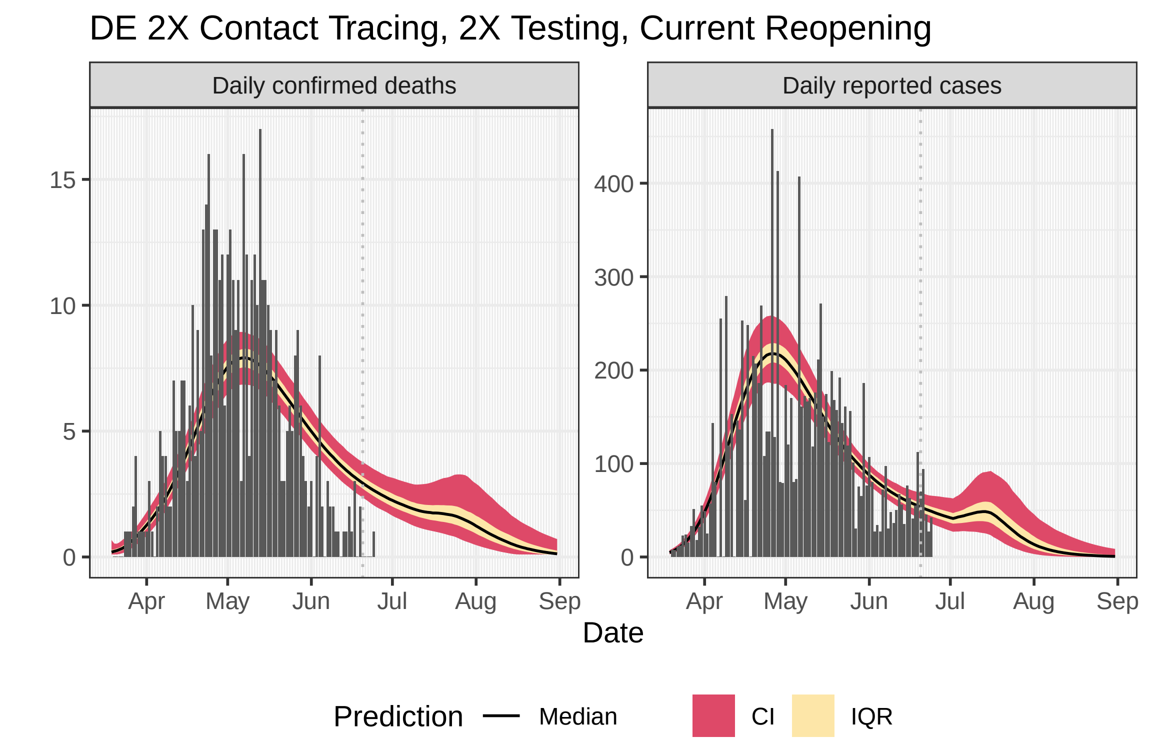

DE 2X Contact Tracing, 2X Testing, Current Reopening
Daily confirmed deaths
Daily reported cases
15
10
5
0
400
300
200
100
0
Apr
May
Jun
Jul
Aug
Sep
Date
Prediction
Median
CI
IQR

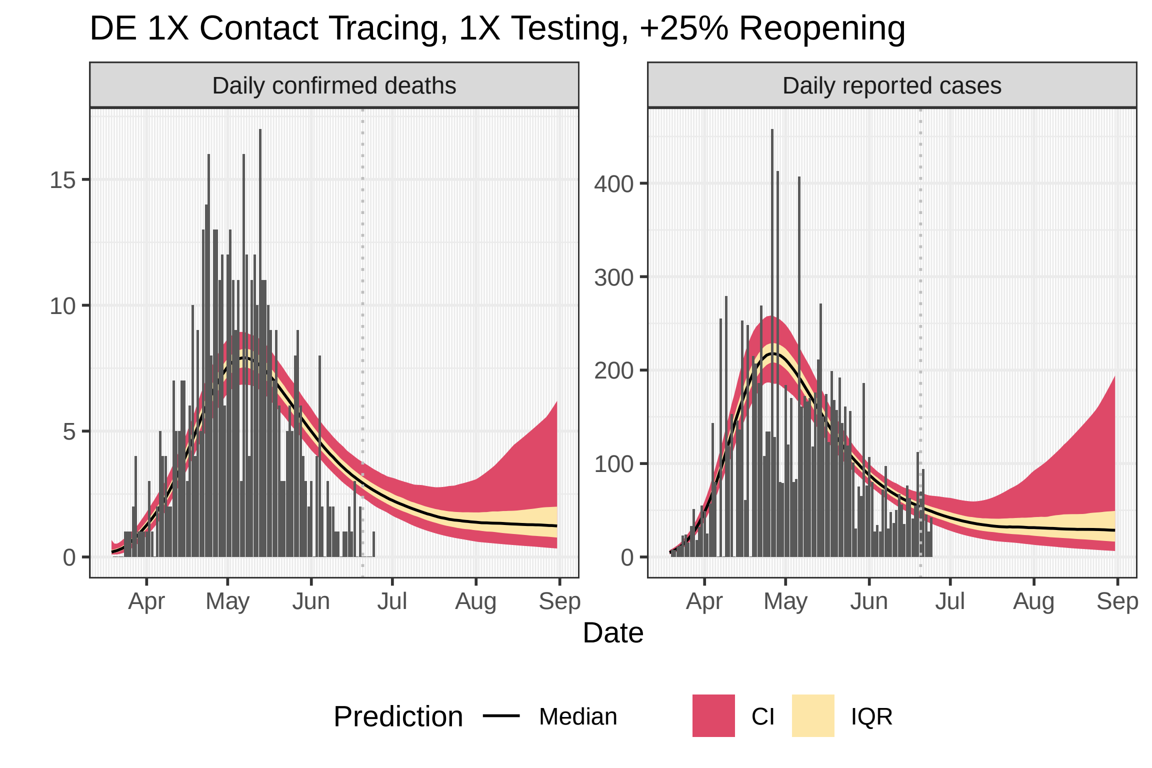
DE 1X Contact Tracing, 1X Testing, +25% Reopening
Daily confirmed deaths
Daily reported cases
15
10
5
0
400
300
200
100
0
Apr
May
Jun
Jul
Aug
Sep
Date
Prediction
Median
CI
IQR

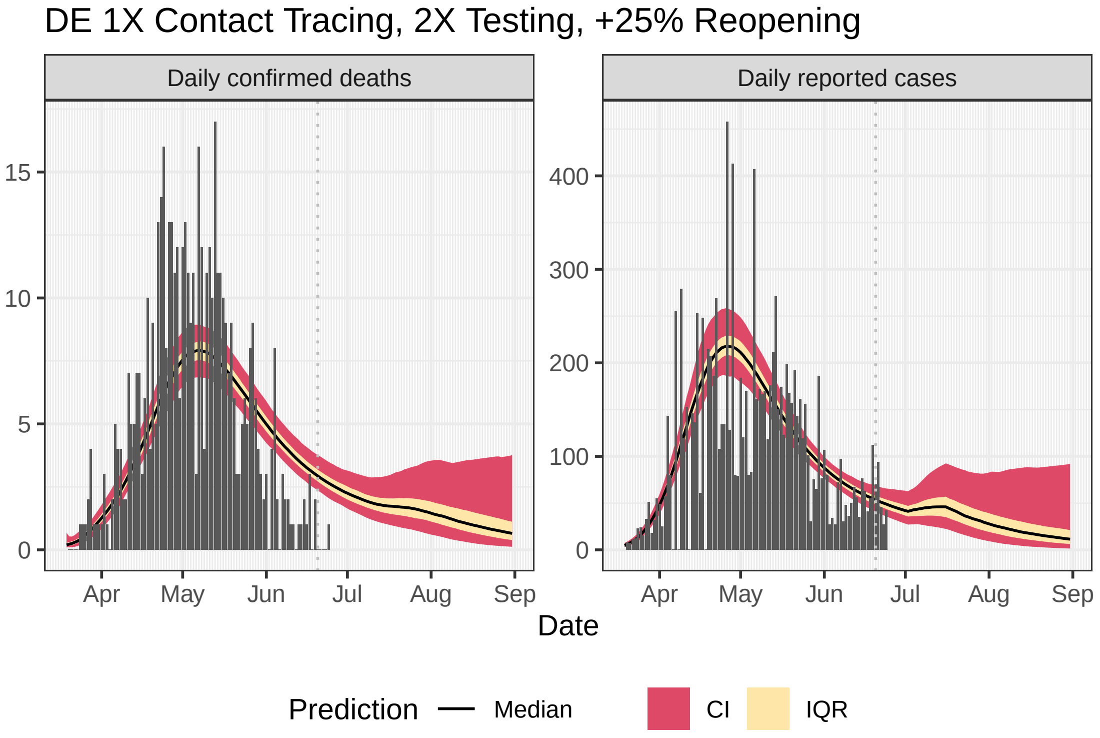

DE 1X Contact Tracing, 2X Testing, +25% Reopening
Daily confirmed deaths
Daily reported cases
0
5
10
15
0
100
200
300
400
Apr
May
Jun
Jul
Aug
Sep
Date
Prediction
Median
CI
IQR

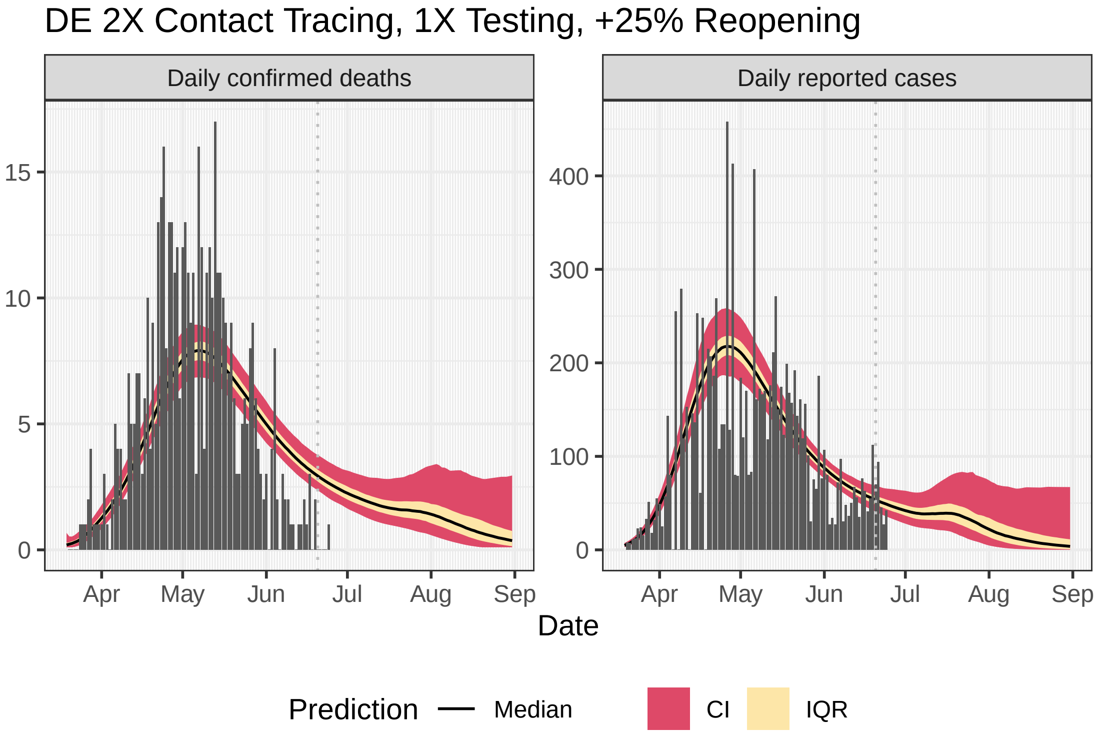
DE 2X Contact Tracing, 1X Testing, +25% Reopening
Daily confirmed deaths
Daily reported cases
15
10
5
0
400
300
200
100
0
Apr
May
Jun
Jul
Aug
Sep
Date
Prediction
Median
CI
IQR

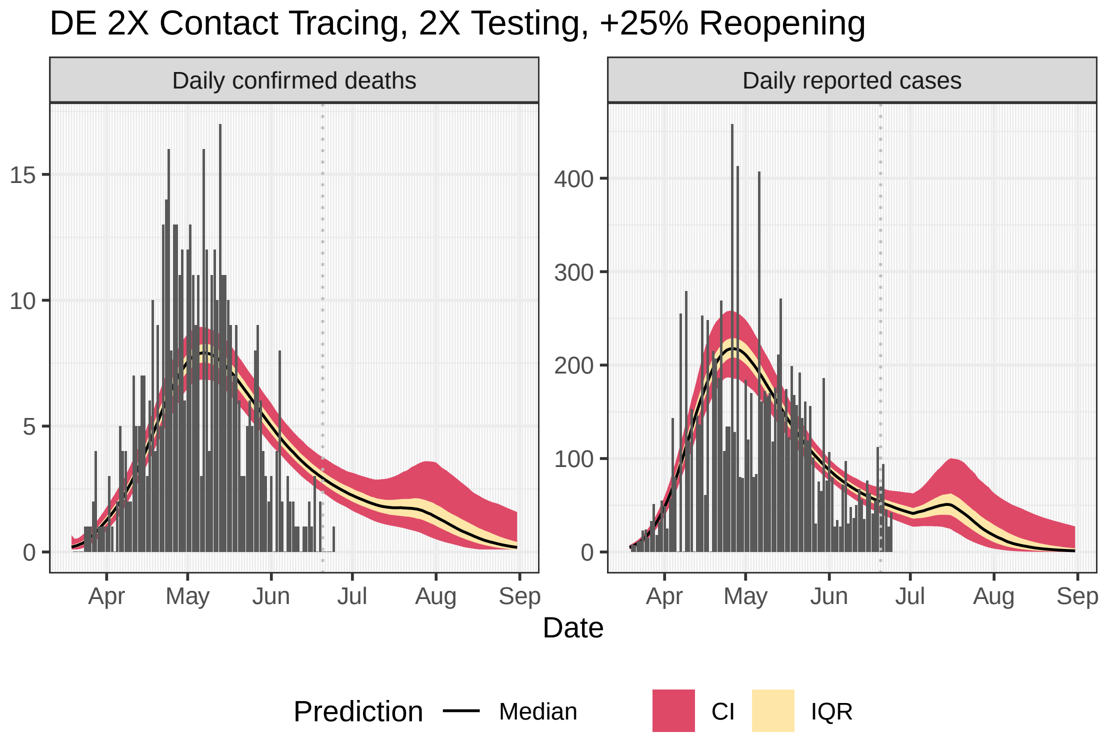

DE 2X Contact Tracing, 2X Testing, +25% Reopening
Daily confirmed deaths
Daily reported cases
15
10
5
0
400
300
200
100
0
Apr
May
Jun
Jul
Aug
Sep
Date
Prediction
Median
CI
IQR

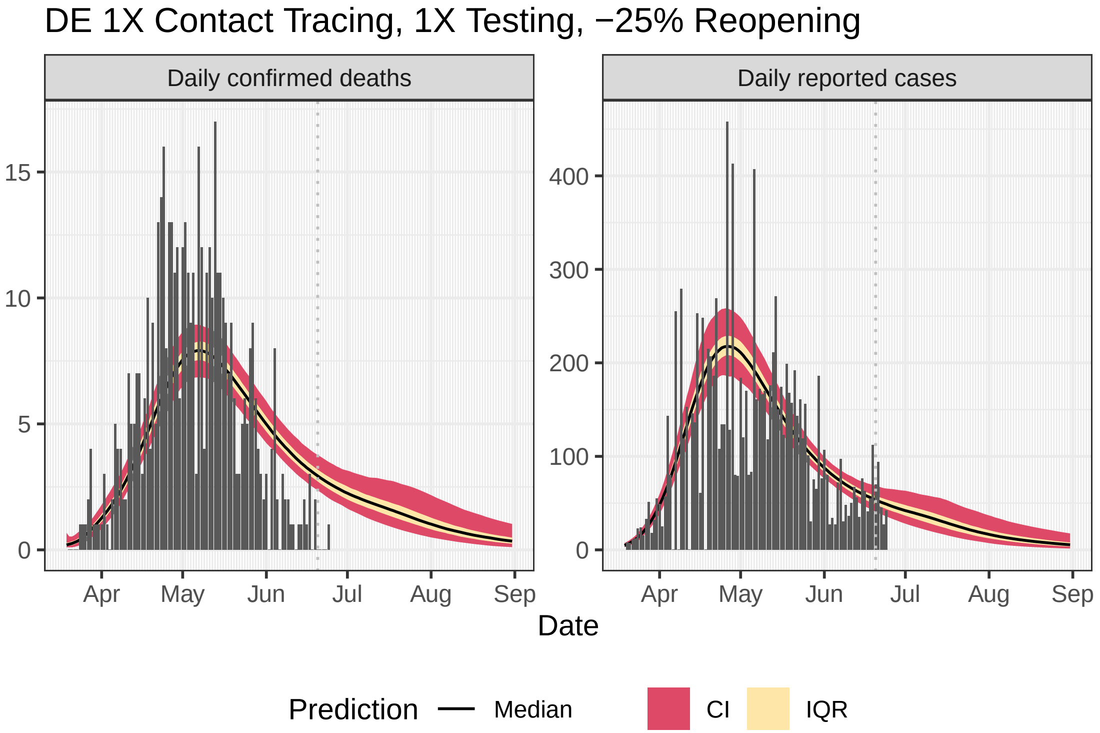

DE 1X Contact Tracing, 1X Testing, −25% Reopening
Daily confirmed deaths
Daily reported cases
0
5
10
15
0
100
200
300
400
Apr
May
Jun
Jul
Aug
Sep
Date
Prediction
Median
CI
IQR

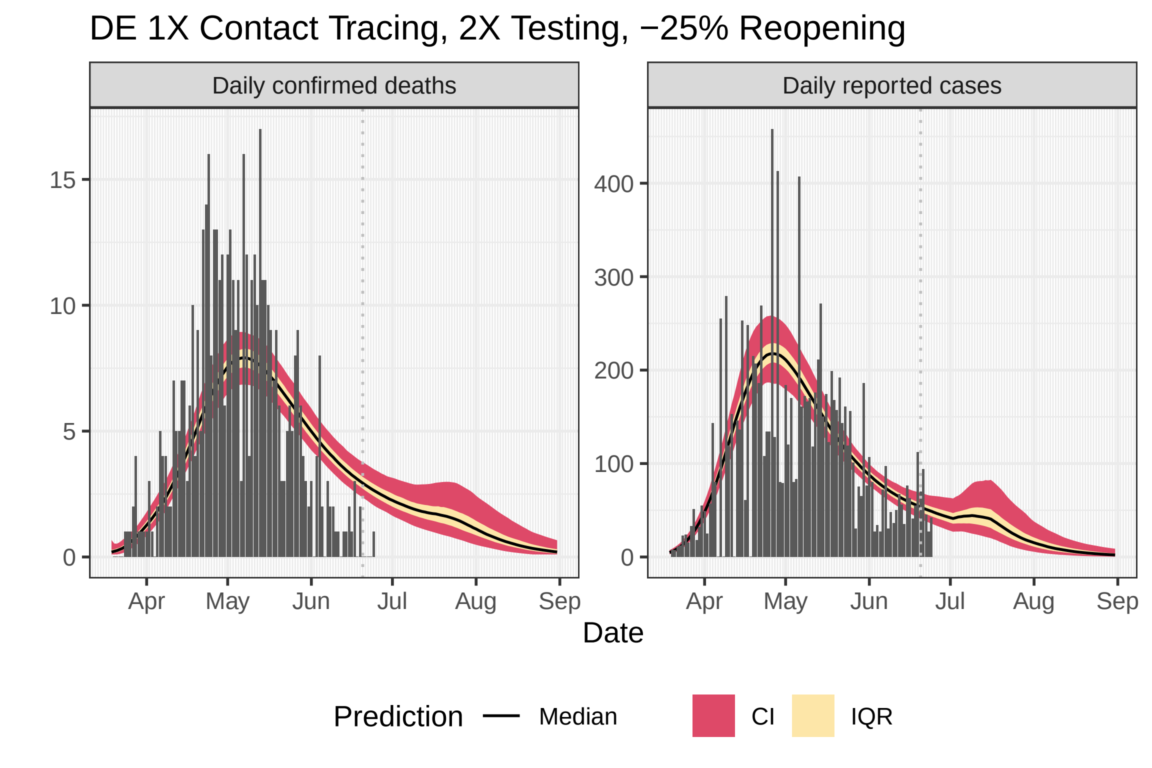
DE 1X Contact Tracing, 2X Testing, −25% Reopening
Daily confirmed deaths
Daily reported cases
15
10
5
0
400
300
200
100
0
Apr
May
Jun
Jul
Aug
Sep
Date
Prediction
Median
CI
IQR

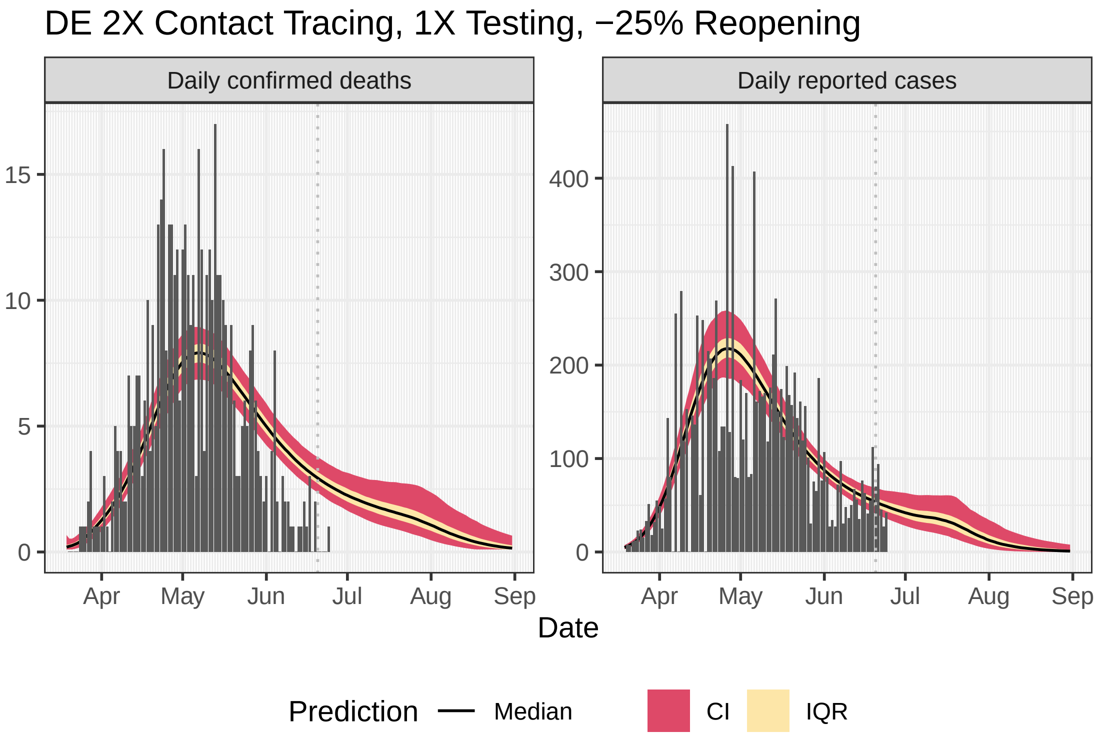
DE 2X Contact Tracing, 1X Testing, −25% Reopening
Daily confirmed deaths
Daily reported cases
15
10
5
0
400
300
200
100
0
Apr
May
Jun
Jul
Aug
Sep
Date
Prediction
Median
CI
IQR

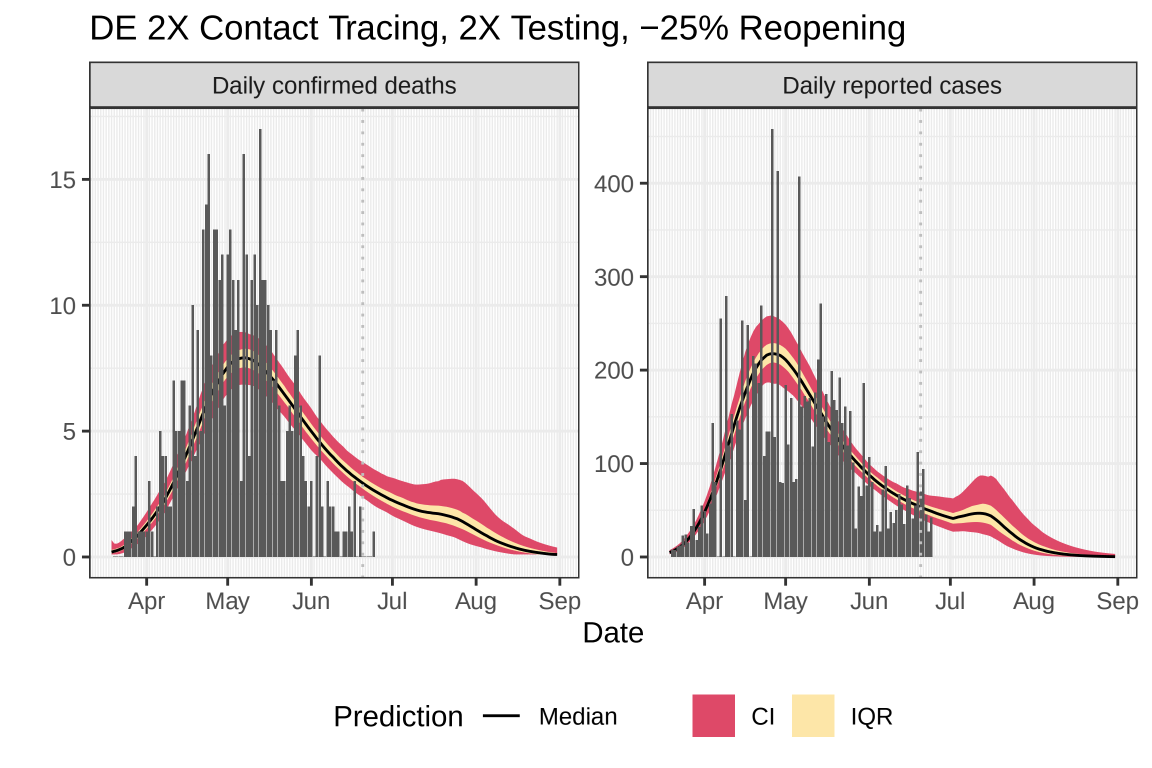

DE 2X Contact Tracing, 2X Testing, −25% Reopening
Daily confirmed deaths
Daily reported cases
15
10
5
0
400
300
200
100
0
Apr
May
Jun
Jul
Aug
Sep
Date
Prediction
Median
CI
IQR

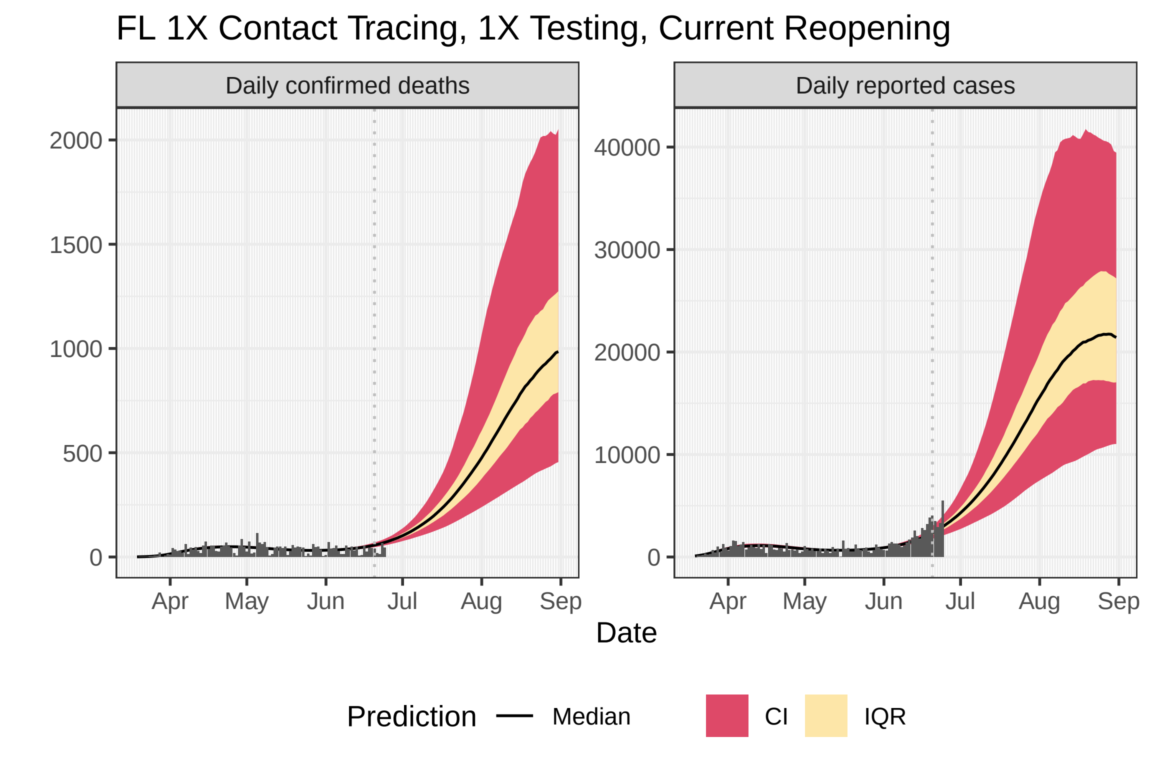
FL 1X Contact Tracing, 1X Testing, Current Reopening
Daily confirmed deaths
Daily reported cases
2000
1500
1000
500
0
40000
30000
20000
10000
0
Apr
May
Jun
Jul
Aug
Sep
Date
Prediction
Median
CI
IQR

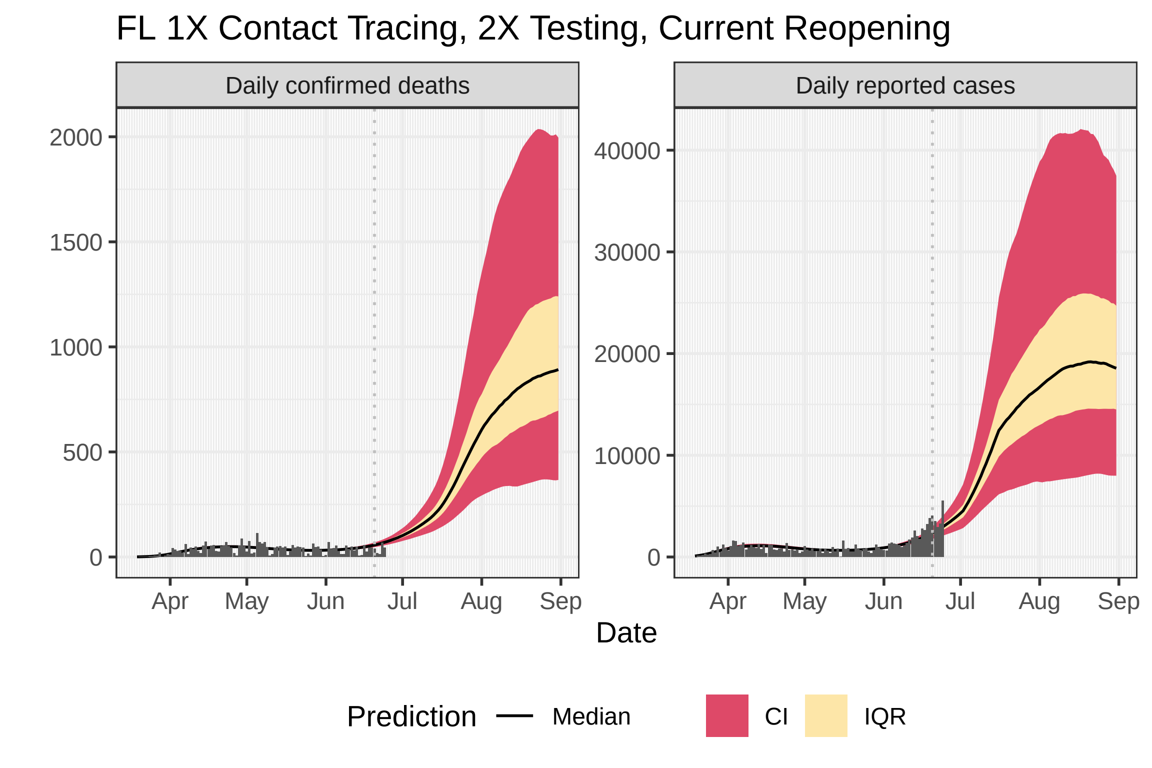

FL 1X Contact Tracing, 2X Testing, Current Reopening
Daily confirmed deaths
Daily reported cases
2000
1500
1000
500
0
40000
30000
20000
10000
0
Apr
May
Jun
Jul
Aug
Sep
Date
Prediction
Median
CI
IQR

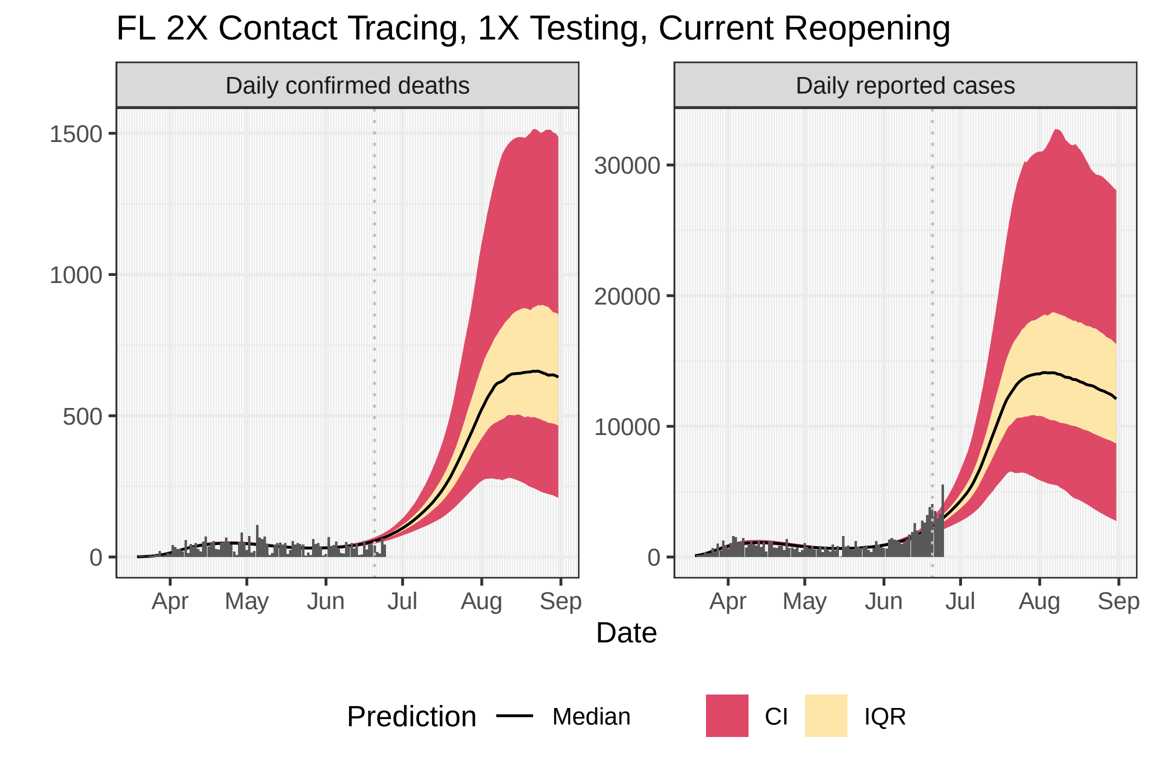
FL 2X Contact Tracing, 1X Testing, Current Reopening
Daily confirmed deaths
Daily reported cases
1500
1000
500
0
30000
20000
10000
0
Apr
May
Jun
Jul
Aug
Sep
Date
Prediction
Median
CI
IQR

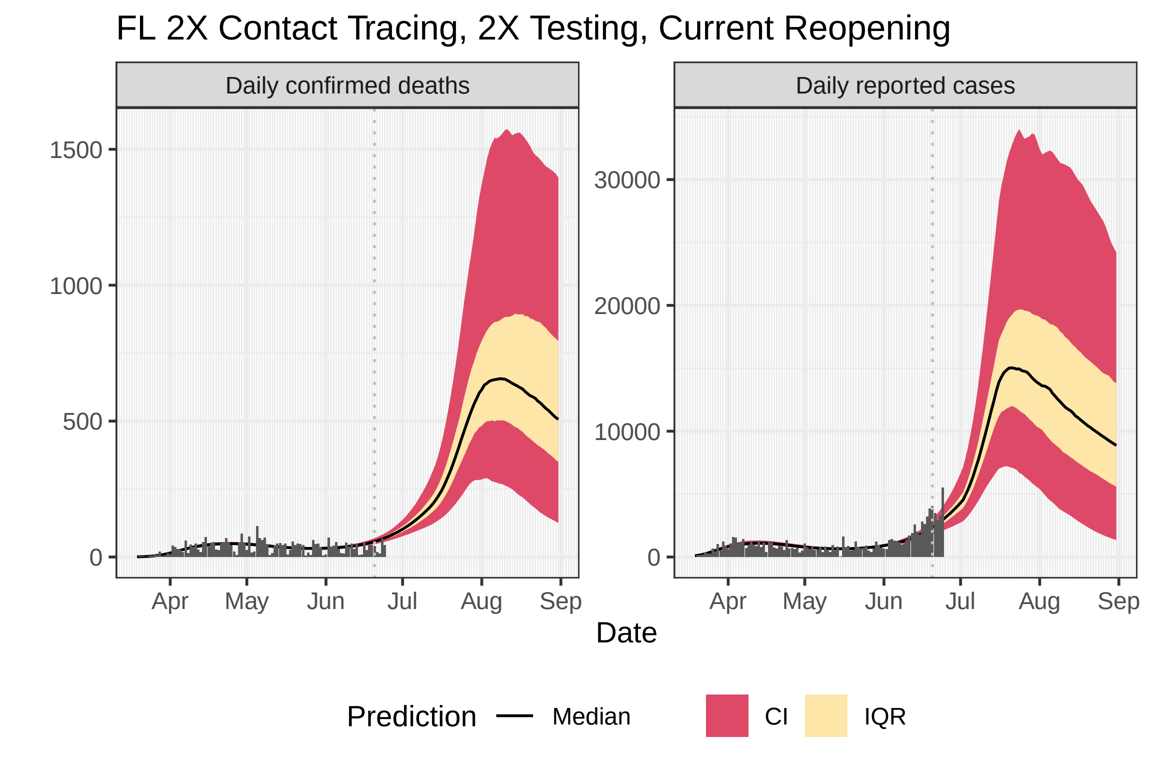
FL 2X Contact Tracing, 2X Testing, Current Reopening
Daily confirmed deaths
Daily reported cases
1500
1000
500
0
30000
20000
10000
0
Apr
May
Jun
Jul
Aug
Sep
Date
Prediction
Median
CI
IQR

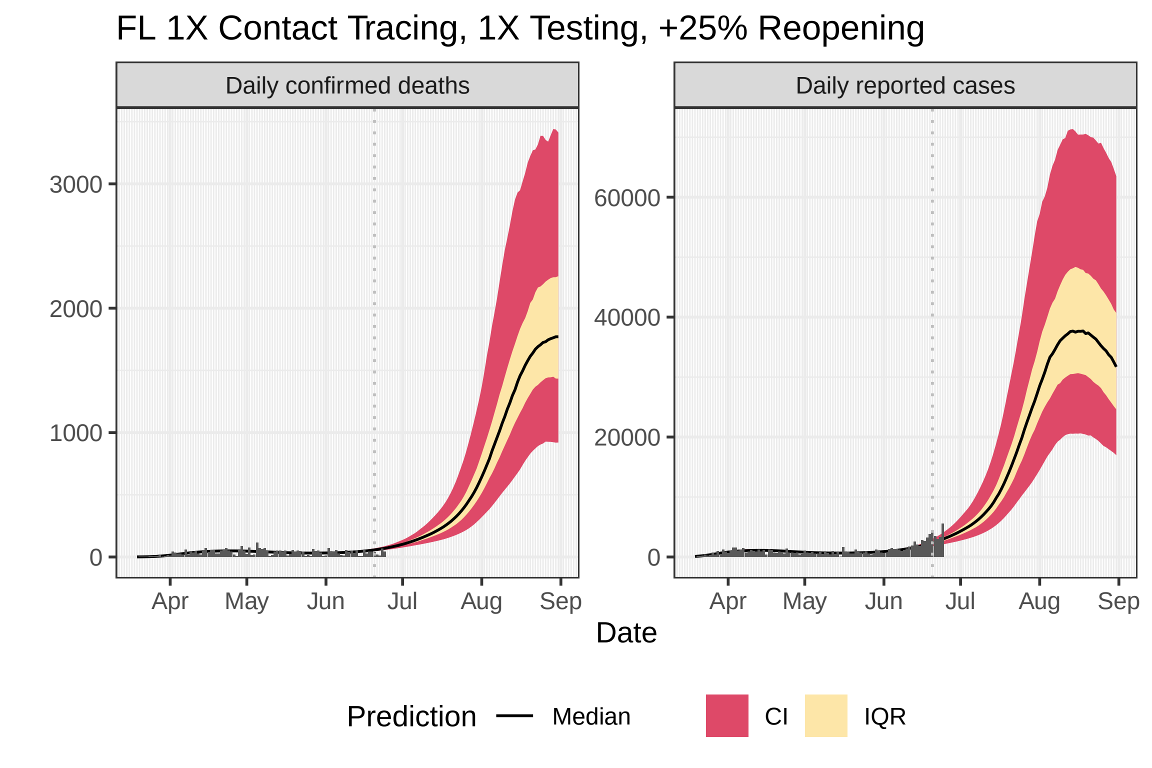
FL 1X Contact Tracing, 1X Testing, +25% Reopening
Daily confirmed deaths
Daily reported cases
3000
2000
1000
0
60000
40000
20000
0
Apr
May
Jun
Jul
Aug
Sep
Date
Prediction
Median
CI
IQR

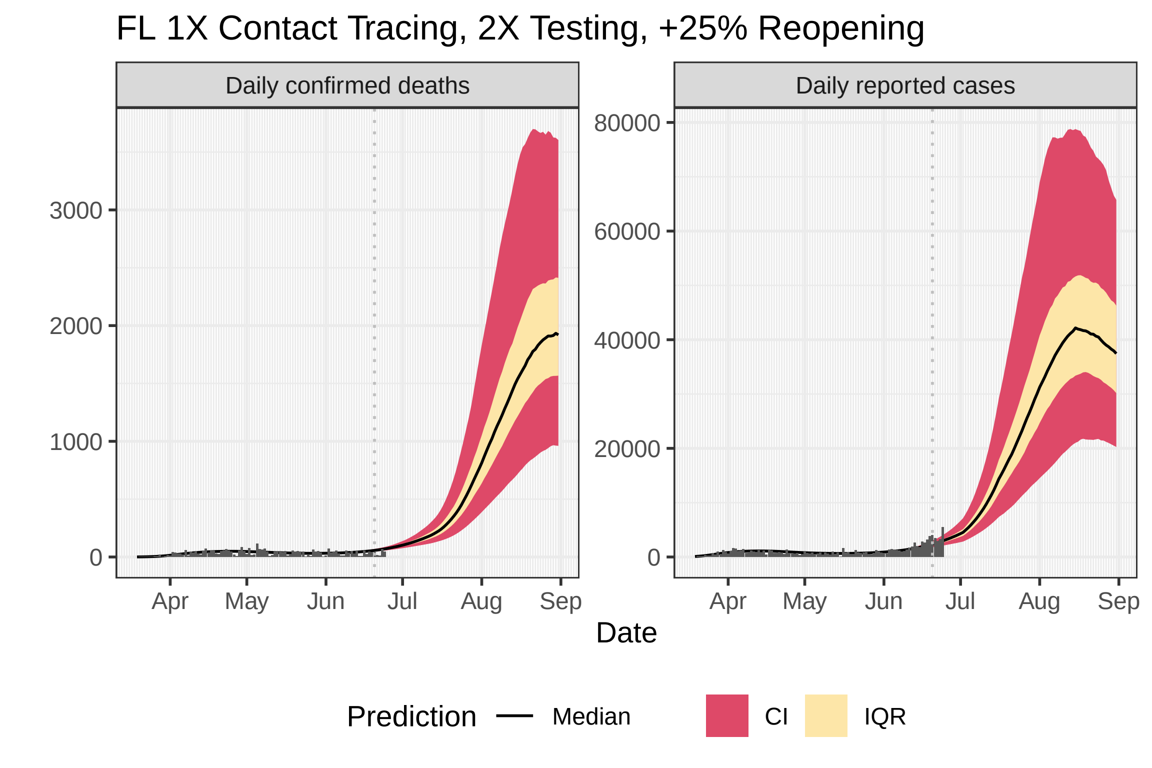
FL 1X Contact Tracing, 2X Testing, +25% Reopening
Daily confirmed deaths
Daily reported cases
3000
2000
1000
0
80000
60000
40000
20000
0
Apr
May
Jun
Jul
Aug
Sep
Date
Prediction
Median
CI
IQR

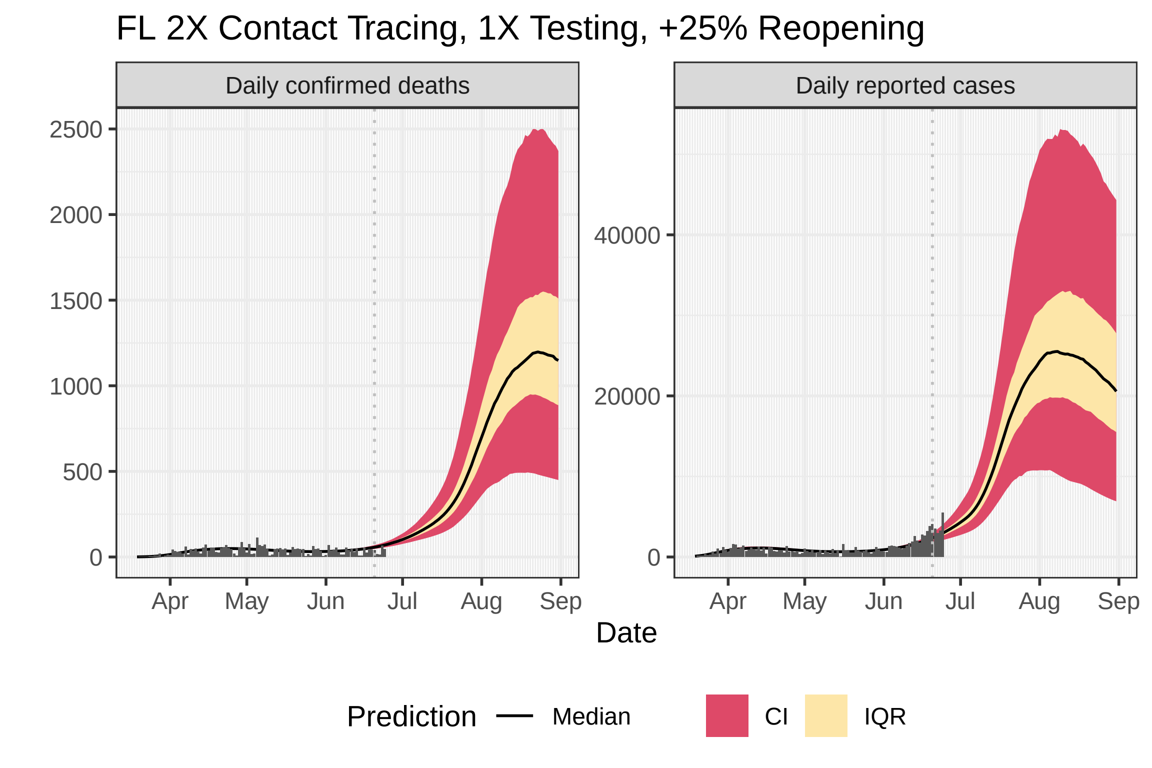

FL 2X Contact Tracing, 1X Testing, +25% Reopening
Daily confirmed deaths
Daily reported cases
2500
2000
1500
1000
500
0
40000
20000
0
Apr
May
Jun
Jul
Aug
Sep
Date
Prediction
Median
CI
IQR

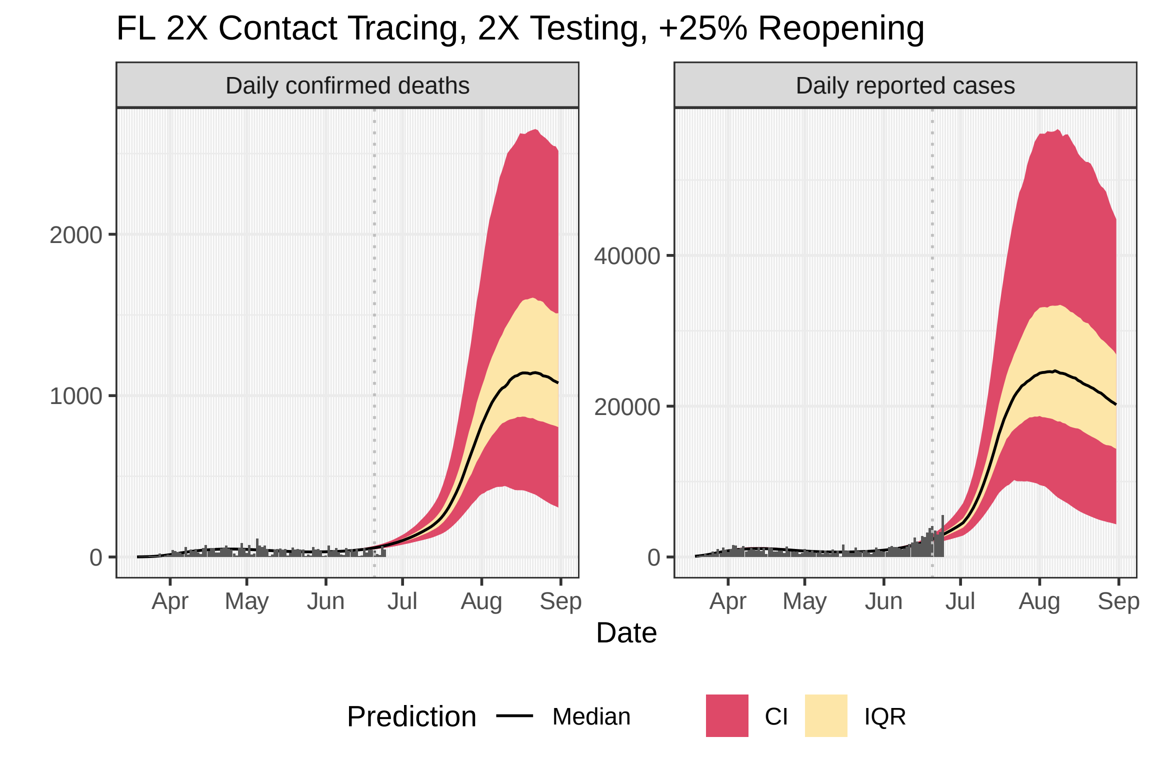
FL 2X Contact Tracing, 2X Testing, +25% Reopening
Daily confirmed deaths
Daily reported cases
2000
1000
0
40000
20000
0
Apr
May
Jun
Jul
Aug
Sep
Date
Prediction
Median
CI
IQR

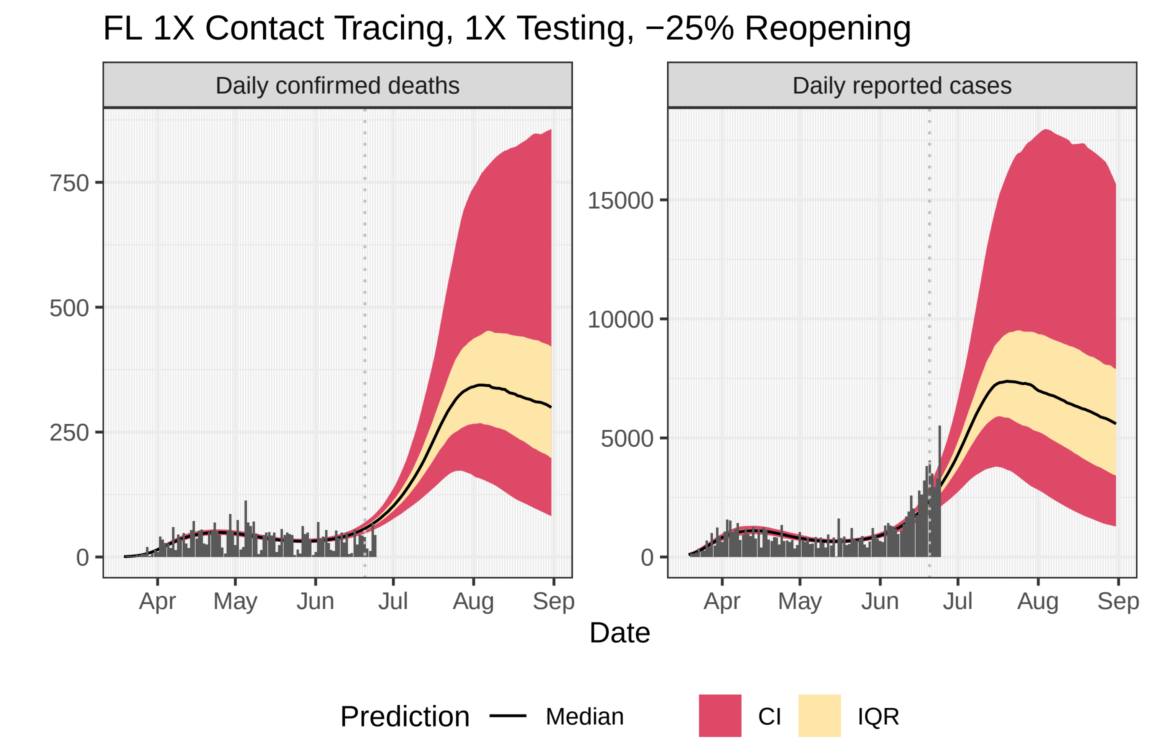
FL 1X Contact Tracing, 1X Testing, −25% Reopening
Daily confirmed deaths
Daily reported cases
750
500
250
0
15000
10000
5000
0
Apr
May
Jun
Jul
Aug
Sep
Date
Prediction
Median
CI
IQR

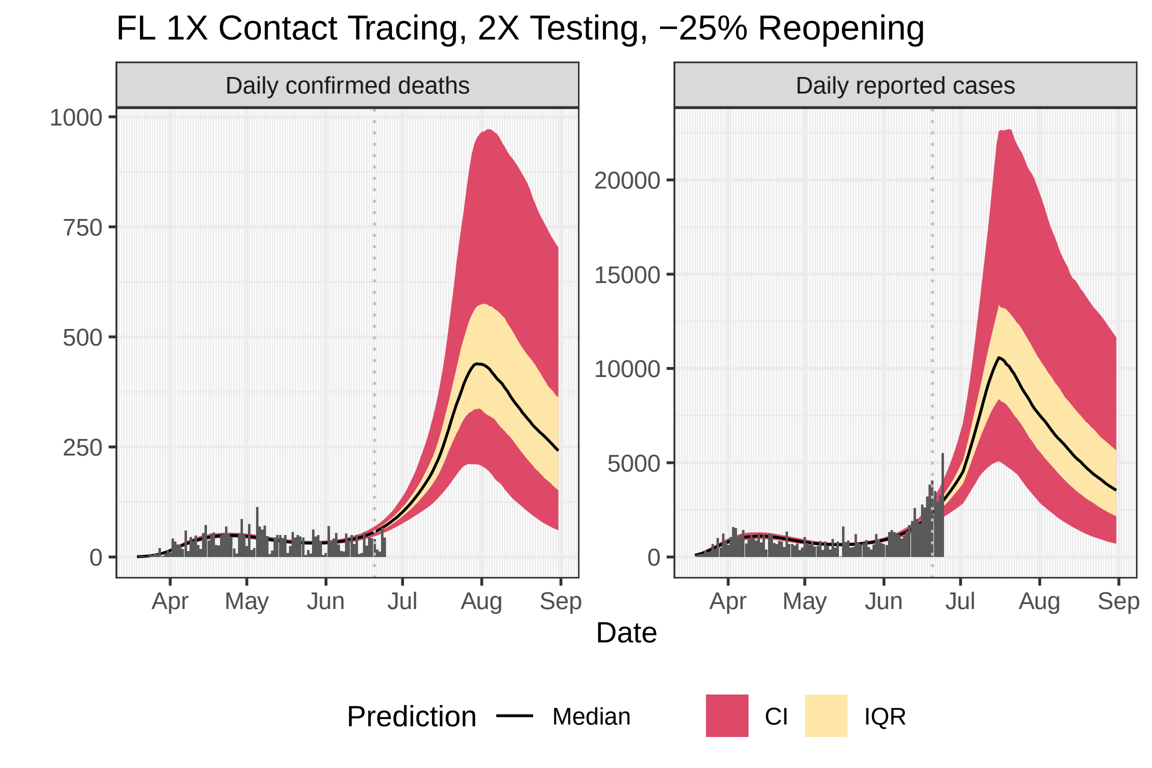

FL 1X Contact Tracing, 2X Testing, −25% Reopening
Daily confirmed deaths
Daily reported cases
1000
750
500
250
0
20000
15000
10000
5000
0
Apr
May
Jun
Jul
Aug
Sep
Date
Prediction
Median
CI
IQR

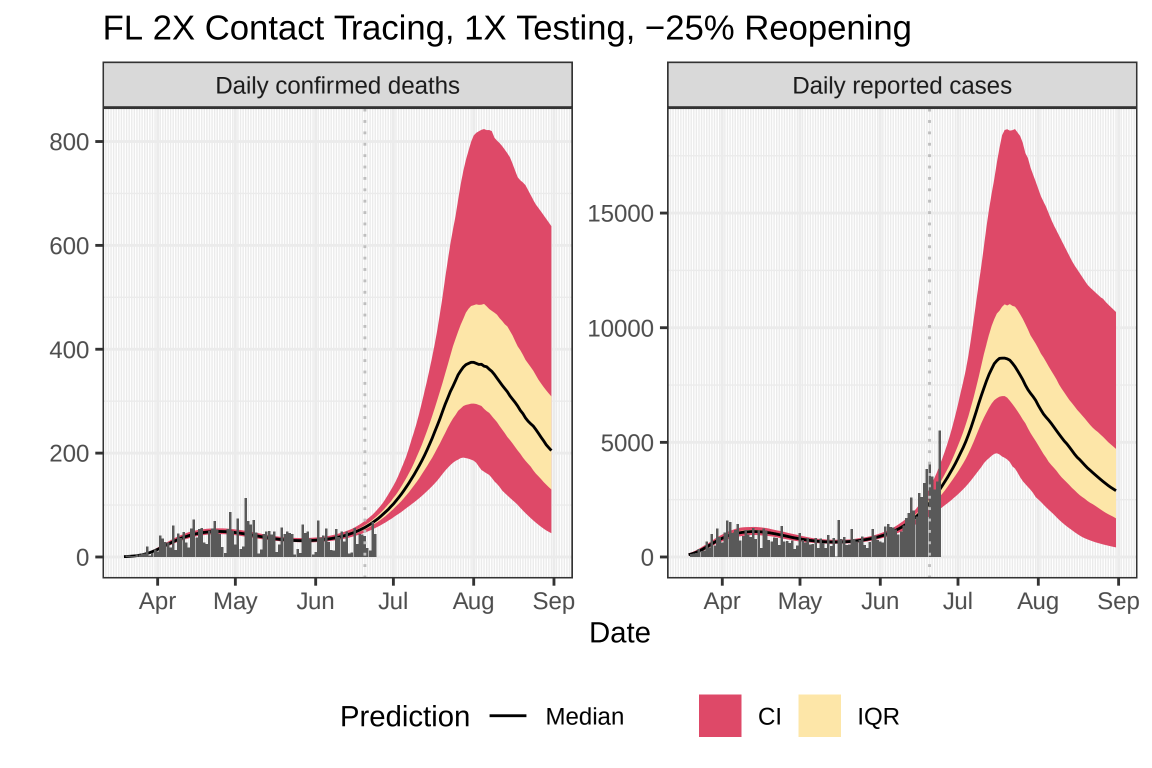

FL 2X Contact Tracing, 1X Testing, −25% Reopening
Daily confirmed deaths
Daily reported cases
800
600
400
200
0
15000
10000
5000
0
Apr
May
Jun
Jul
Aug
Sep
Date
Prediction
Median
CI
IQR

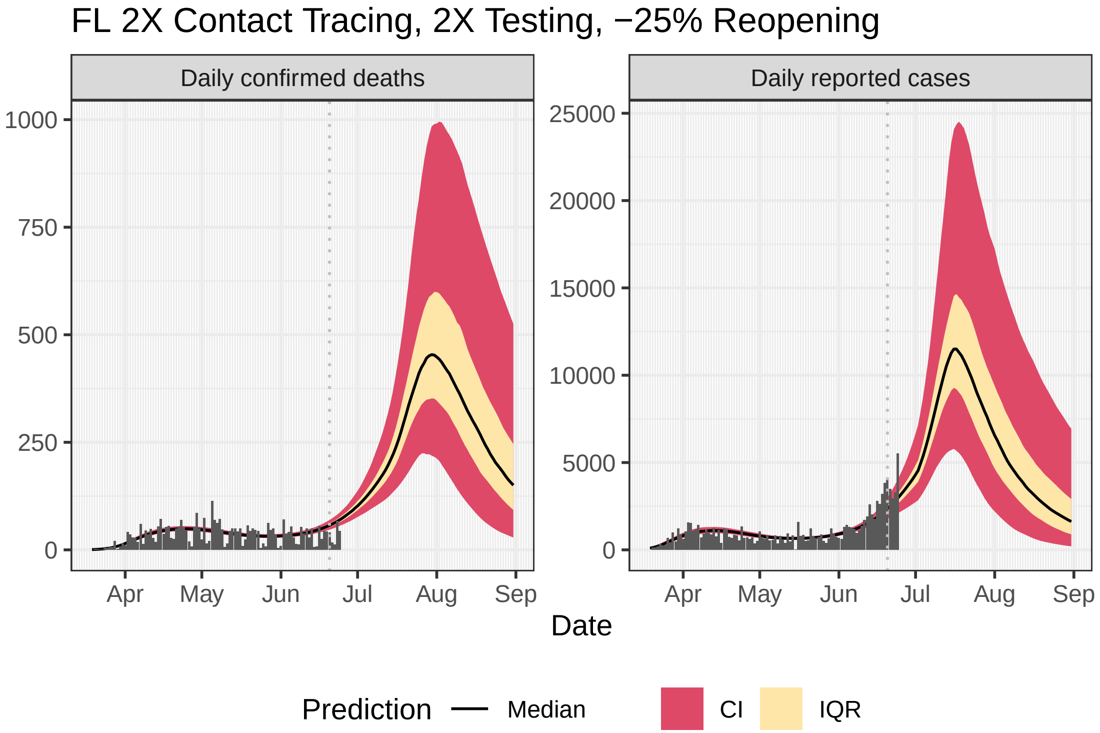
FL 2X Contact Tracing, 2X Testing, −25% Reopening
Daily confirmed deaths
Daily reported cases
1000
750
500
250
0
25000
20000
15000
10000
5000
0
Apr
May
Jun
Jul
Aug
Sep
Date
Prediction
Median
CI
IQR

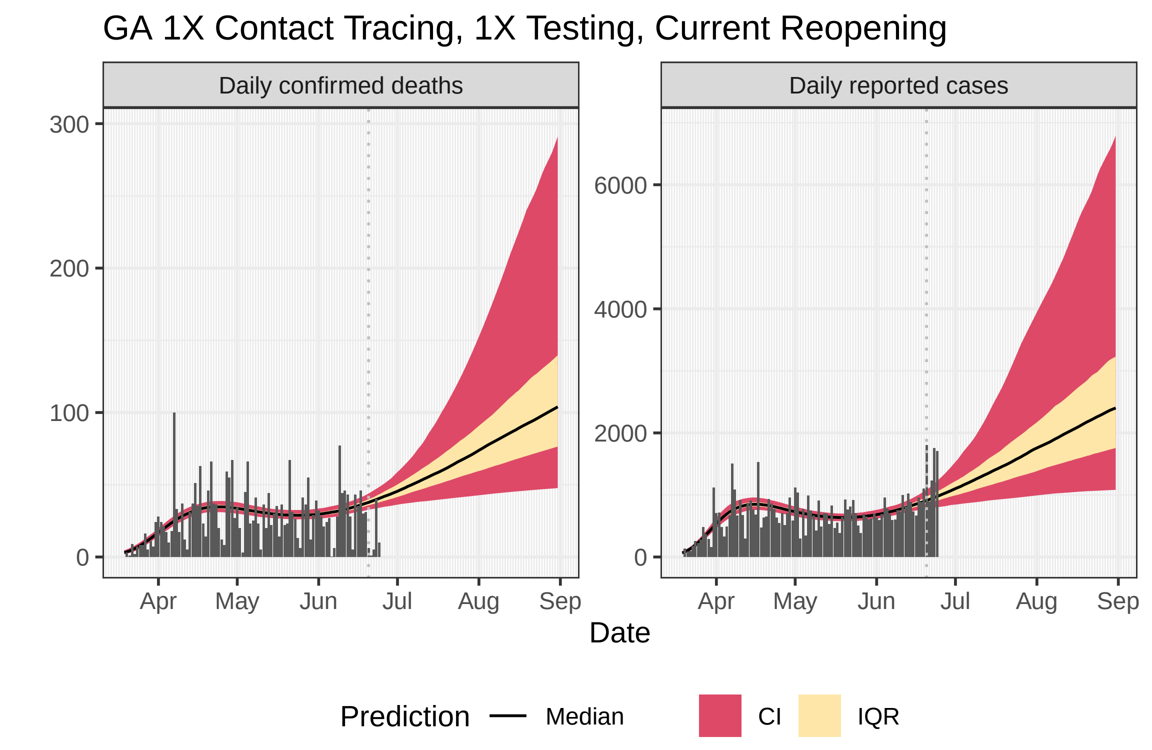
GA 1X Contact Tracing, 1X Testing, Current Reopening
Daily confirmed deaths
Daily reported cases
300
200
100
0
6000
4000
2000
0
Apr
May
Jun
Jul
Aug
Sep
Date
Prediction
Median
CI
IQR

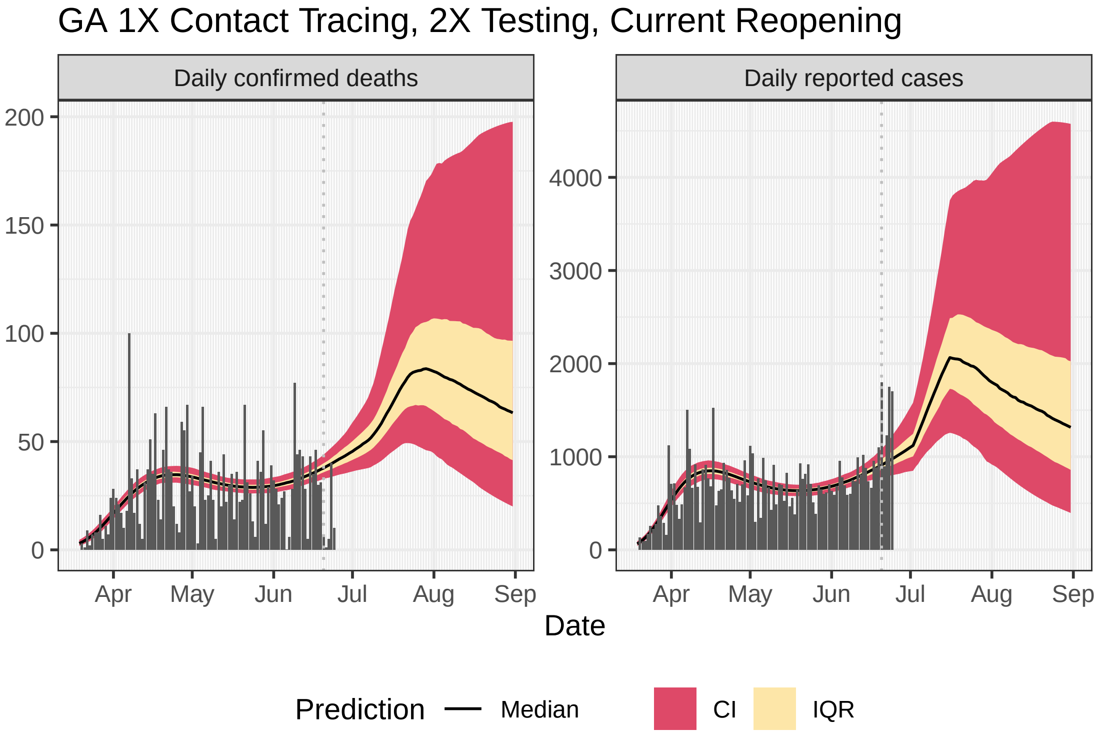
GA 1X Contact Tracing, 2X Testing, Current Reopening
Daily confirmed deaths
Daily reported cases
200
150
100
50
0
4000
3000
2000
1000
0
Apr
May
Jun
Jul
Aug
Sep
Date
Prediction
Median
CI
IQR

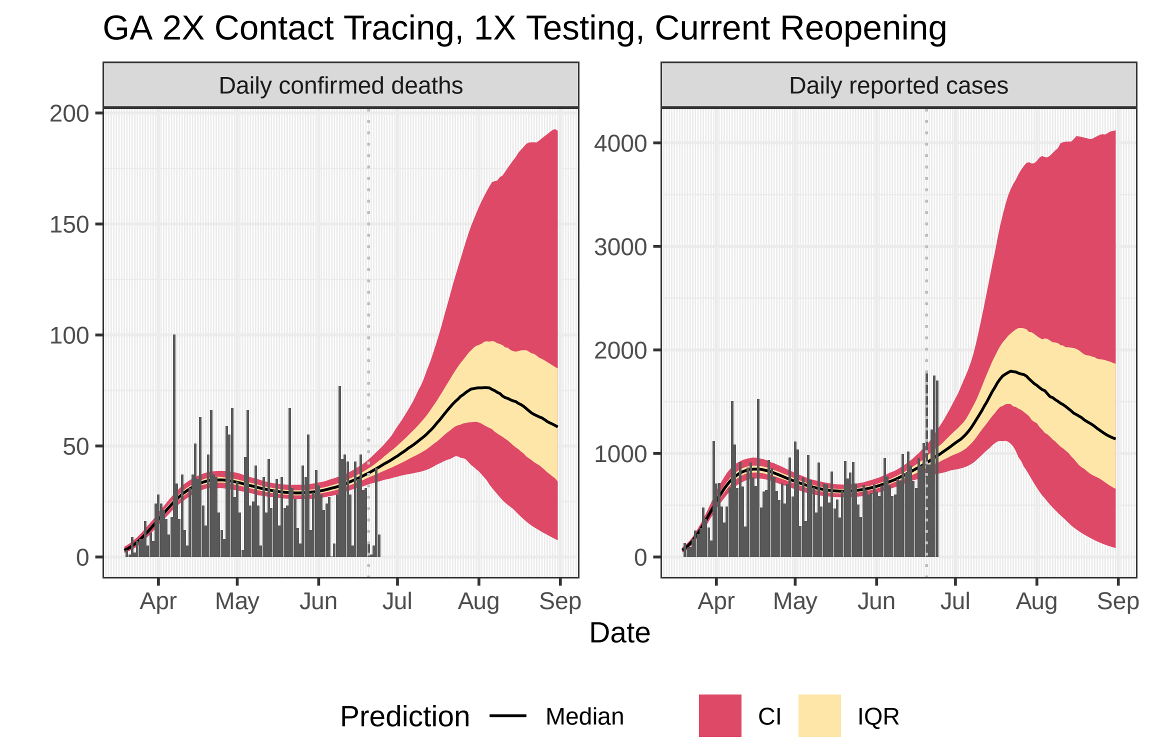

GA 2X Contact Tracing, 1X Testing, Current Reopening
Daily confirmed deaths
Daily reported cases
200
150
100
50
0
4000
3000
2000
1000
0
Apr
May
Jun
Jul
Aug
Sep
Date
Prediction
Median
CI
IQR

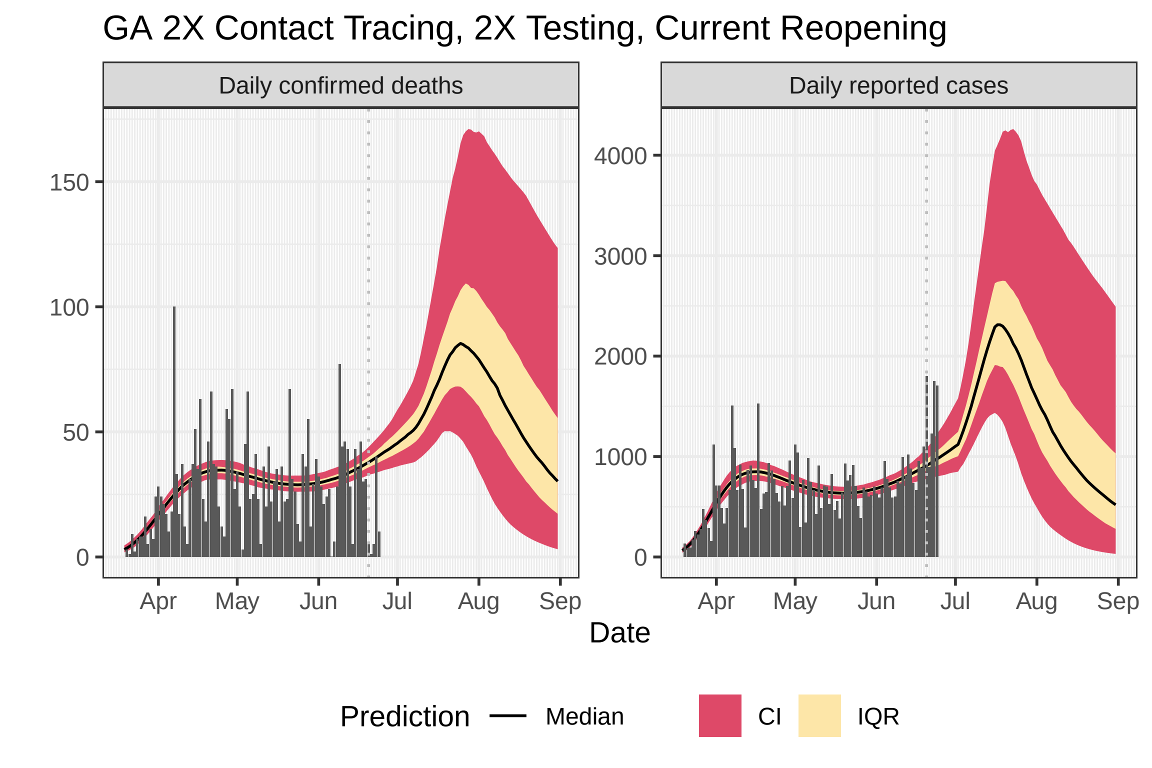
GA 2X Contact Tracing, 2X Testing, Current Reopening
Daily confirmed deaths
Daily reported cases
150
100
50
0
4000
3000
2000
1000
0
Apr
May
Jun
Jul
Aug
Sep
Date
Prediction
Median
CI
IQR

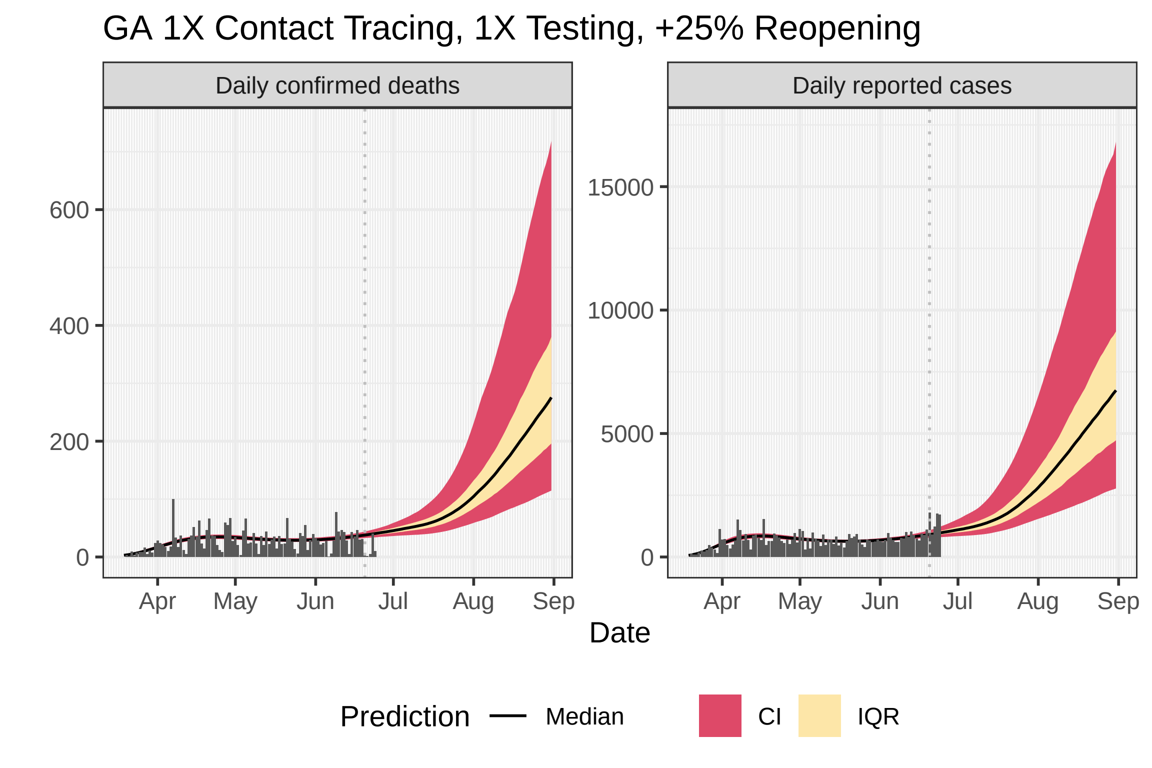
GA 1X Contact Tracing, 1X Testing, +25% Reopening
Daily confirmed deaths
Daily reported cases
600
400
200
0
15000
10000
5000
0
Apr
May
Jun
Jul
Aug
Sep
Date
Prediction
Median
CI
IQR

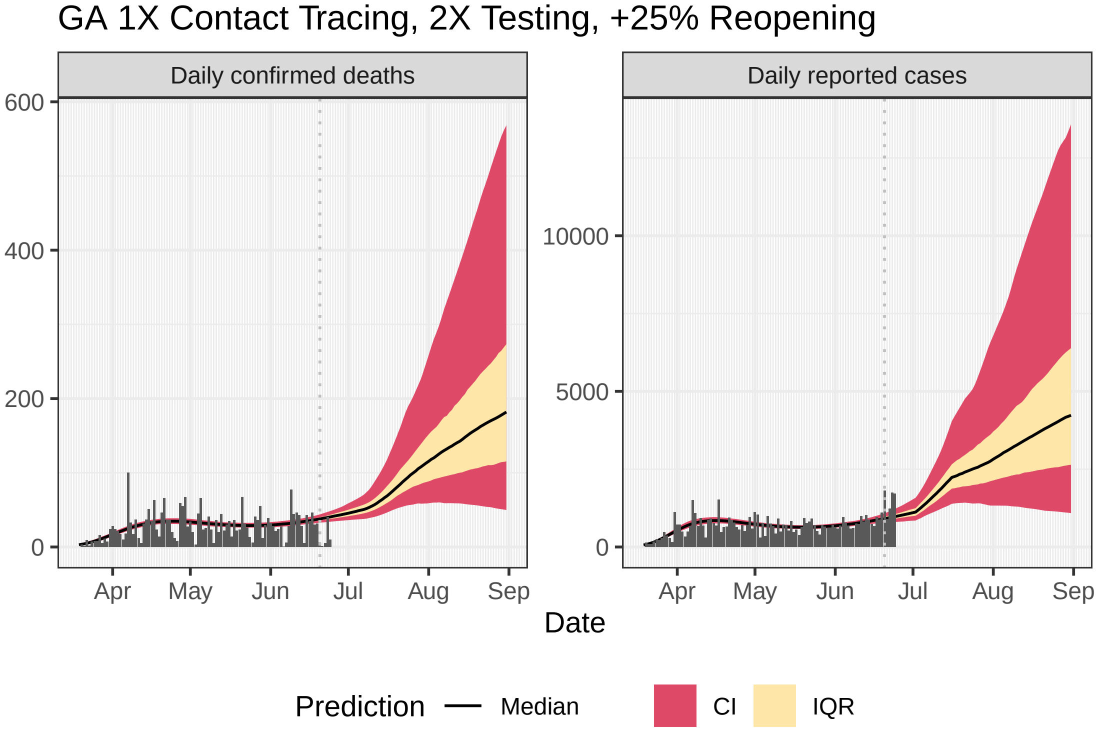

GA 1X Contact Tracing, 2X Testing, +25% Reopening
Daily confirmed deaths
Daily reported cases
600
400
200
0
10000
5000
0
Apr
May
Jun
Jul
Aug
Sep
Date
Prediction
Median
CI
IQR

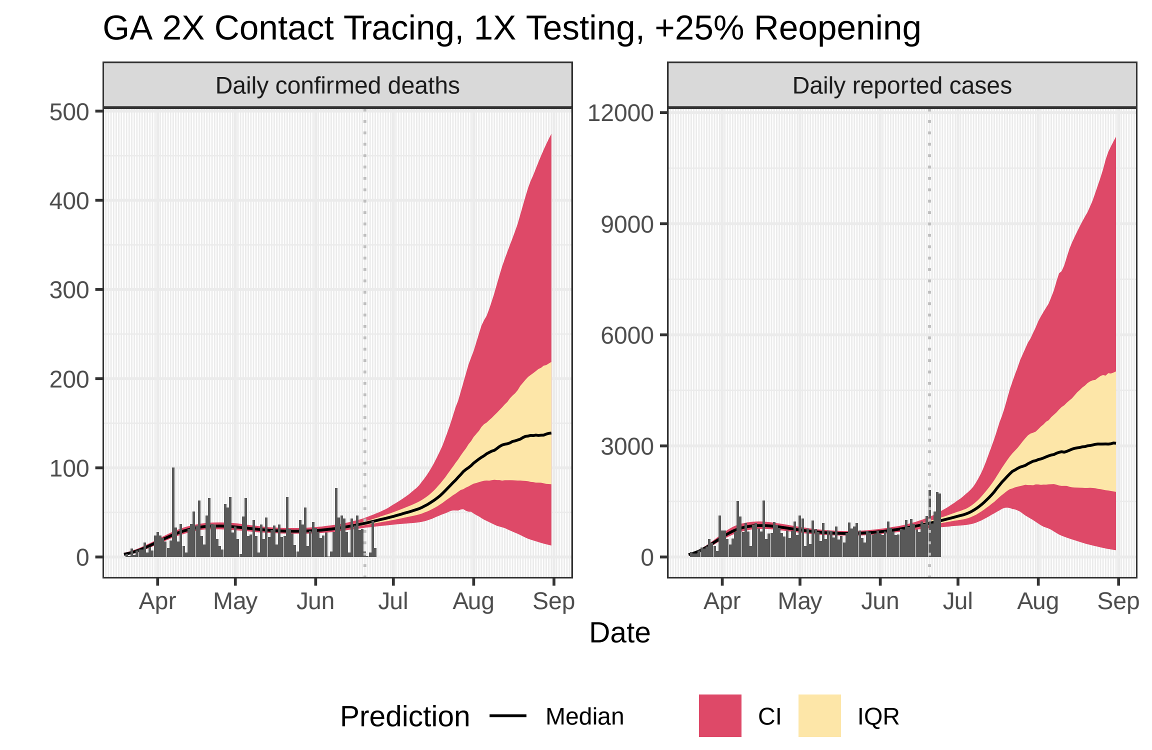
GA 2X Contact Tracing, 1X Testing, +25% Reopening
Daily confirmed deaths
Daily reported cases
500
400
300
200
100
0
12000
9000
6000
3000
0
Apr
May
Jun
Jul
Aug
Sep
Date
Prediction
Median
CI
IQR

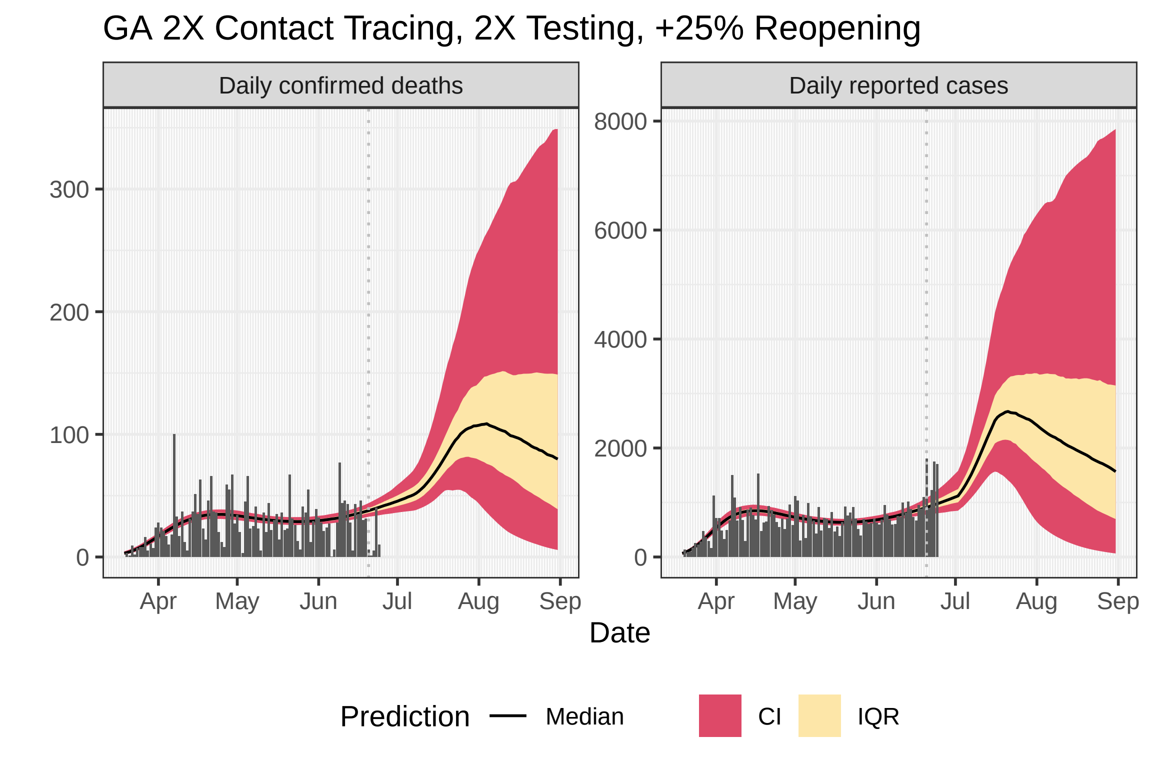

GA 2X Contact Tracing, 2X Testing, +25% Reopening
Daily confirmed deaths
Daily reported cases
0
100
200
300
0
2000
4000
6000
8000
Apr
May
Jun
Jul
Aug
Sep
Date
Prediction
Median
CI
IQR

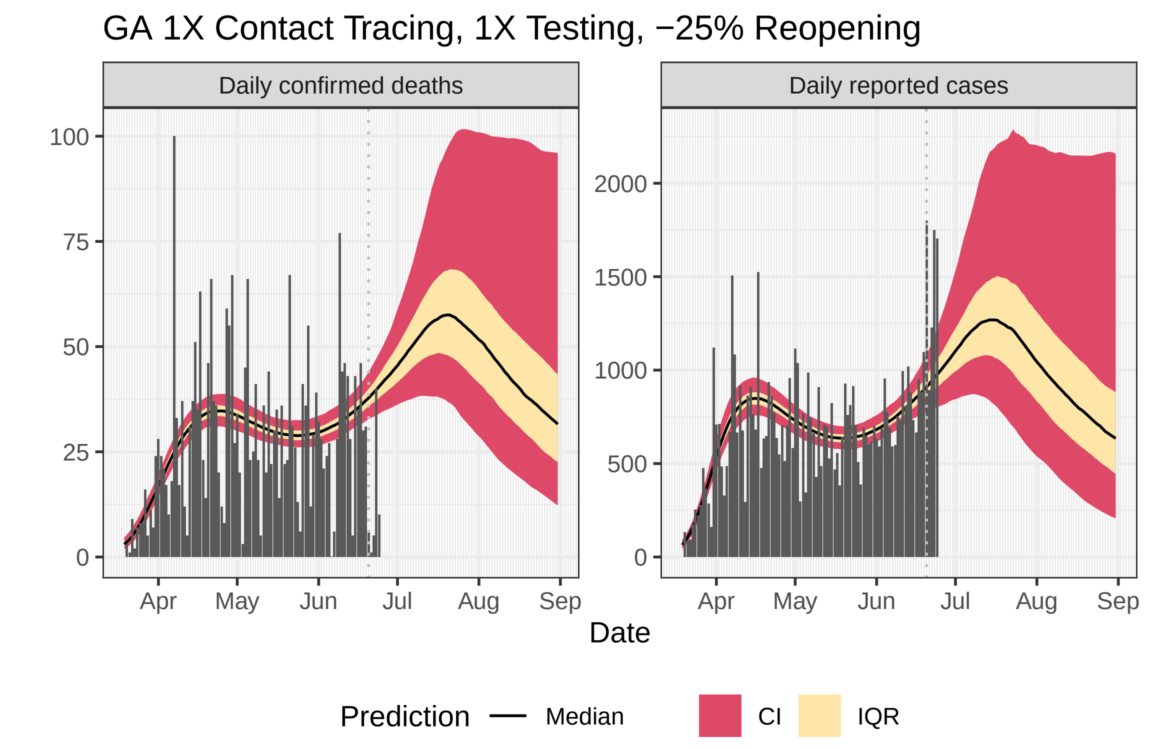
GA 1X Contact Tracing, 1X Testing, −25% Reopening
Daily confirmed deaths
Daily reported cases
100
75
50
25
0
2000
1500
1000
500
0
Apr
May
Jun
Jul
Aug
Sep
Date
Prediction
Median
CI
IQR

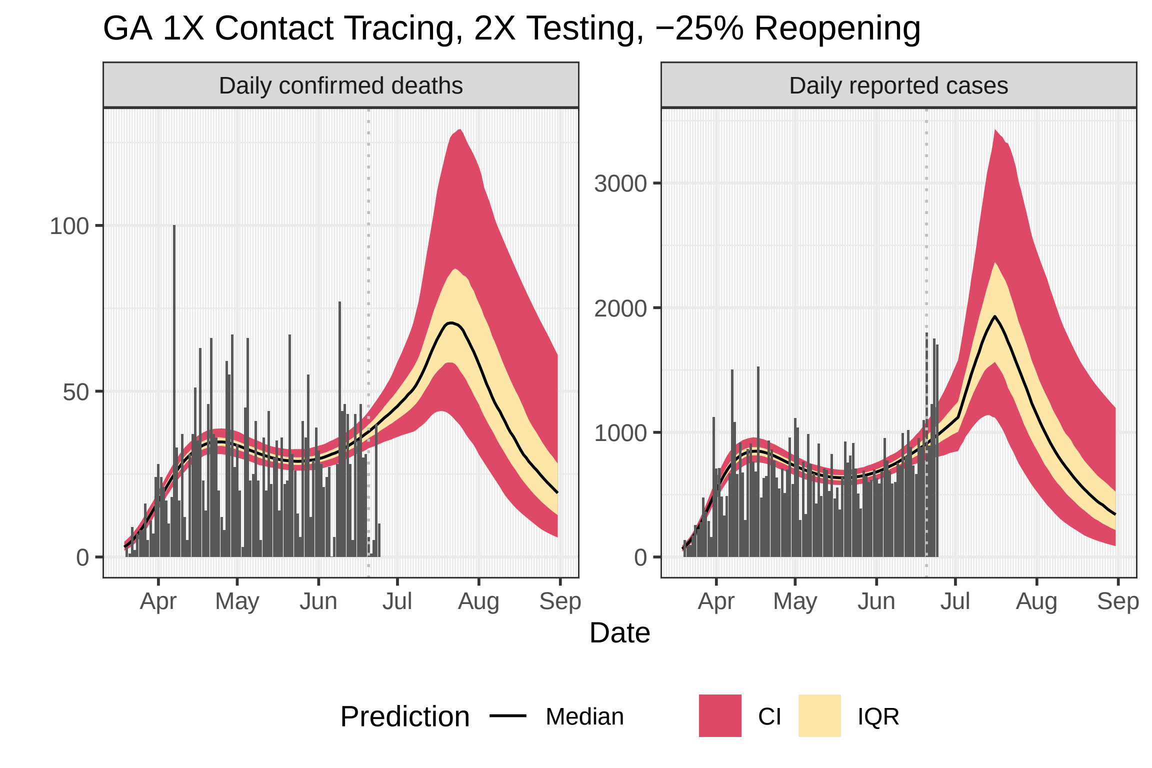

GA 1X Contact Tracing, 2X Testing, −25% Reopening
Daily confirmed deaths
Daily reported cases
100
50
0
3000
2000
1000
0
Apr
May
Jun
Jul
Aug
Sep
Date
Prediction
Median
CI
IQR

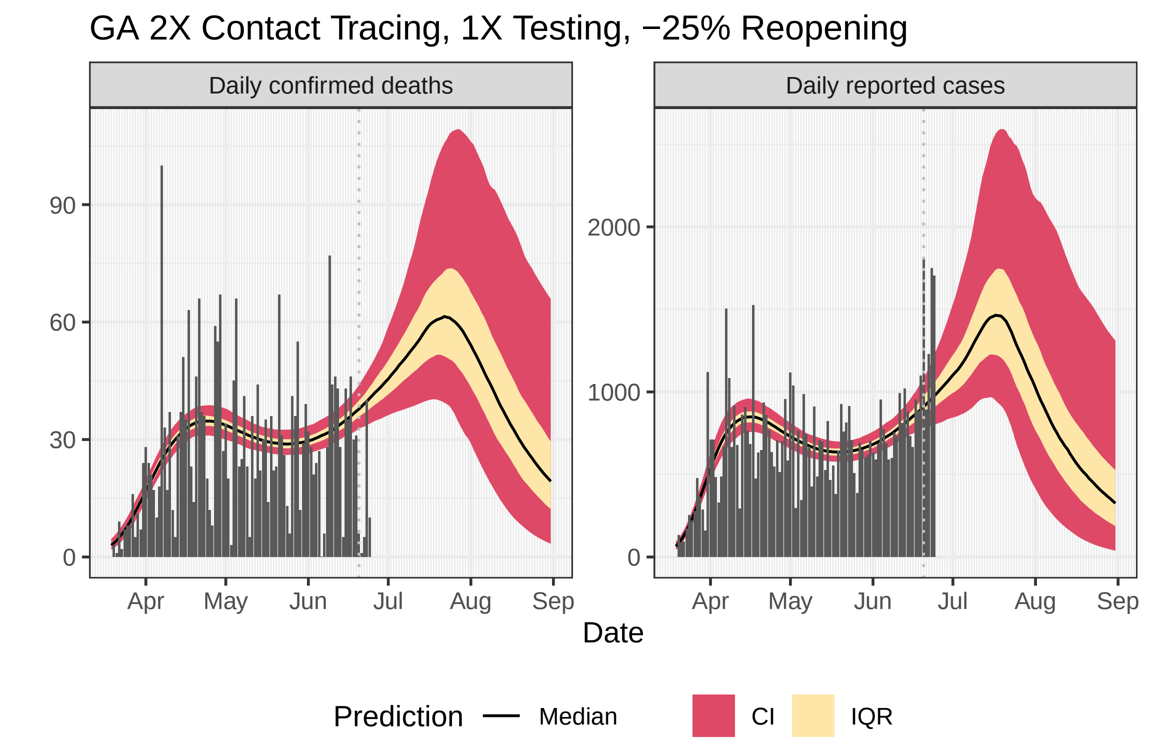
GA 2X Contact Tracing, 1X Testing, −25% Reopening
Daily confirmed deaths
Daily reported cases
90
60
30
0
2000
1000
0
Apr
May
Jun
Jul
Aug
Sep
Date
Prediction
Median
CI
IQR

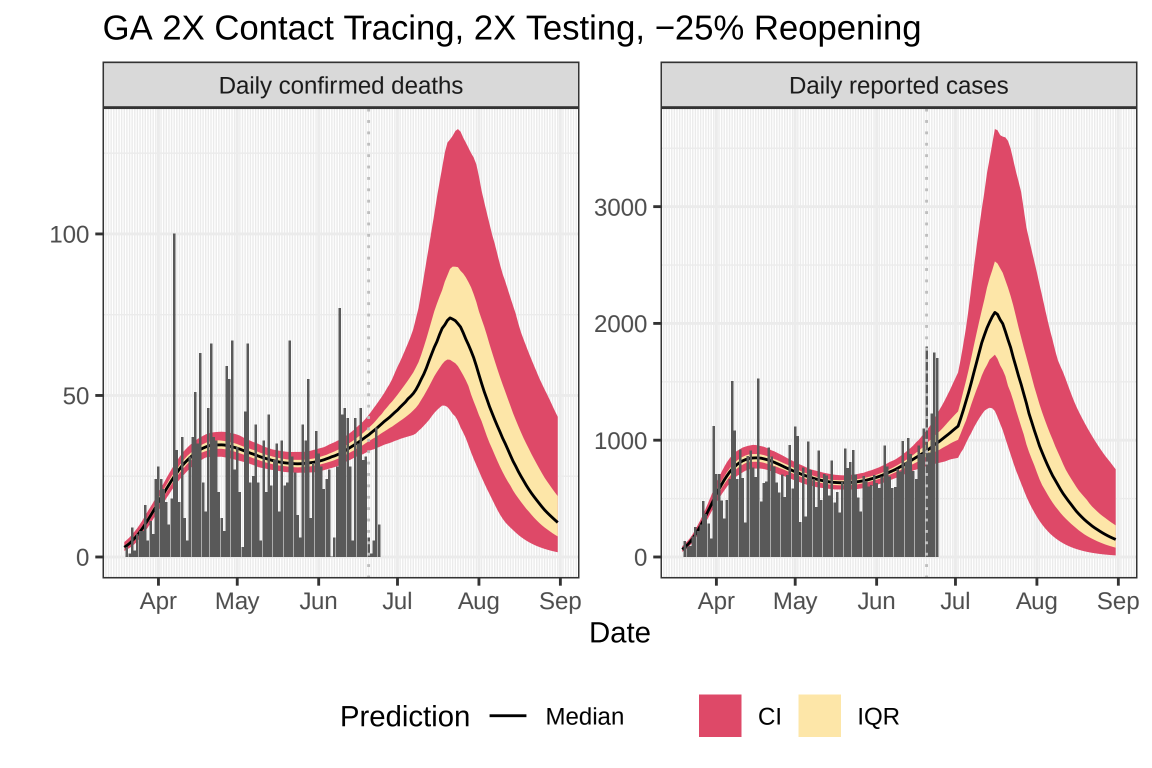
GA 2X Contact Tracing, 2X Testing, −25% Reopening
Daily confirmed deaths
Daily reported cases
100
50
0
3000
2000
1000
0
Apr
May
Jun
Jul
Aug
Sep
Date
Prediction
Median
CI
IQR

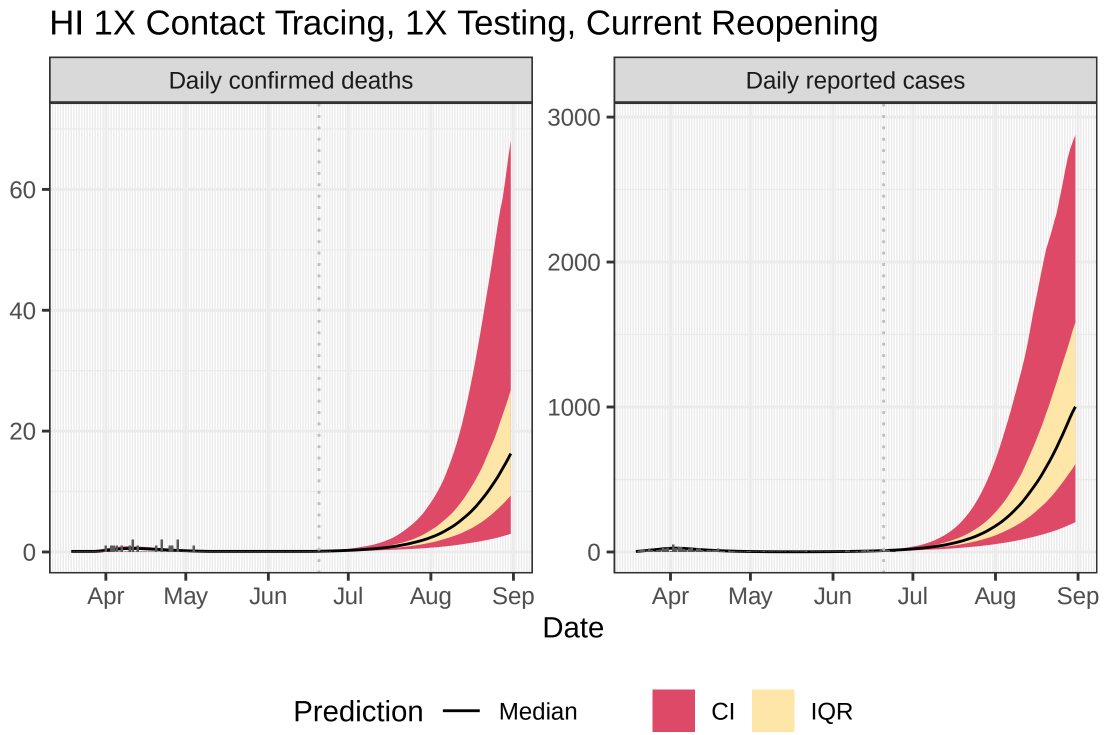
HI 1X Contact Tracing, 1X Testing, Current Reopening
Daily confirmed deaths
Daily reported cases
60
40
20
0
3000
2000
1000
0
Apr
May
Jun
Jul
Aug
Sep
Date
Prediction
Median
CI
IQR

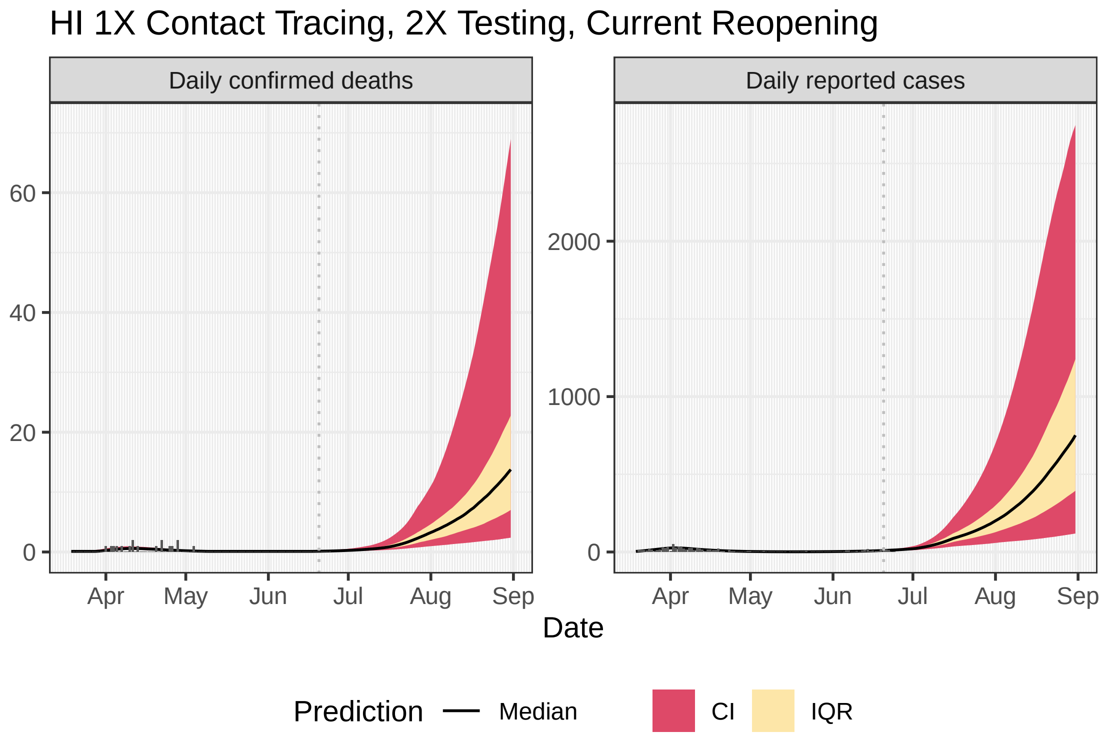
HI 1X Contact Tracing, 2X Testing, Current Reopening
Daily confirmed deaths
Daily reported cases
60
40
20
0
2000
1000
0
Apr
May
Jun
Jul
Aug
Sep
Date
Prediction
Median
CI
IQR

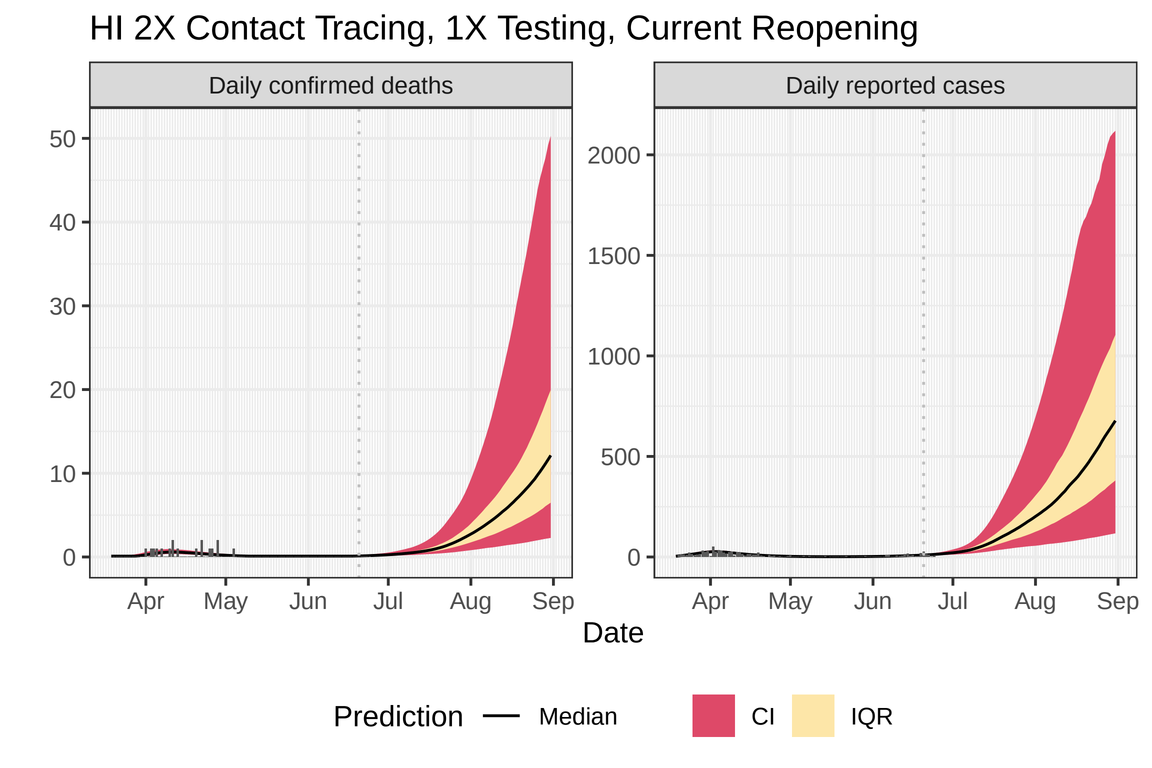

HI 2X Contact Tracing, 1X Testing, Current Reopening
Daily confirmed deaths
Daily reported cases
50
40
30
20
10
0
2000
1500
1000
500
0
Apr
May
Jun
Jul
Aug
Sep
Date
Prediction
Median
CI
IQR

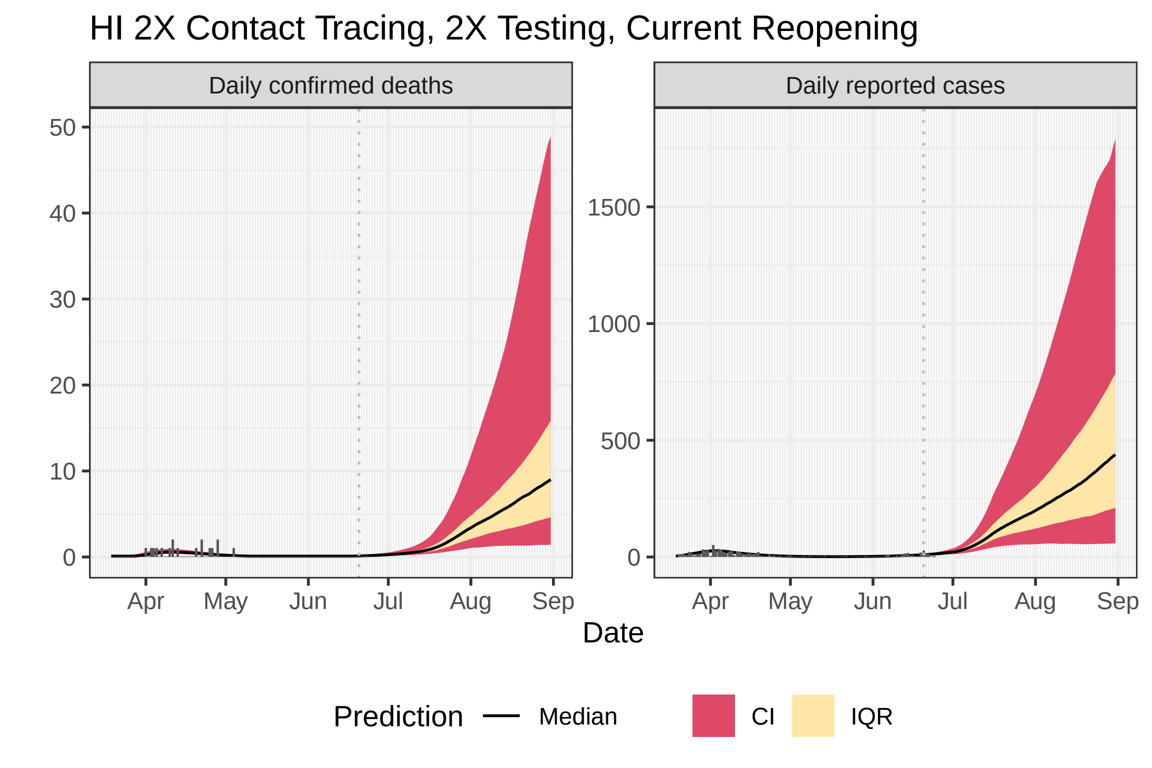
HI 2X Contact Tracing, 2X Testing, Current Reopening
Daily confirmed deaths
Daily reported cases
50
40
30
20
10
0
1500
1000
500
0
Apr
May
Jun
Jul
Aug
Sep
Date
Prediction
Median
CI
IQR

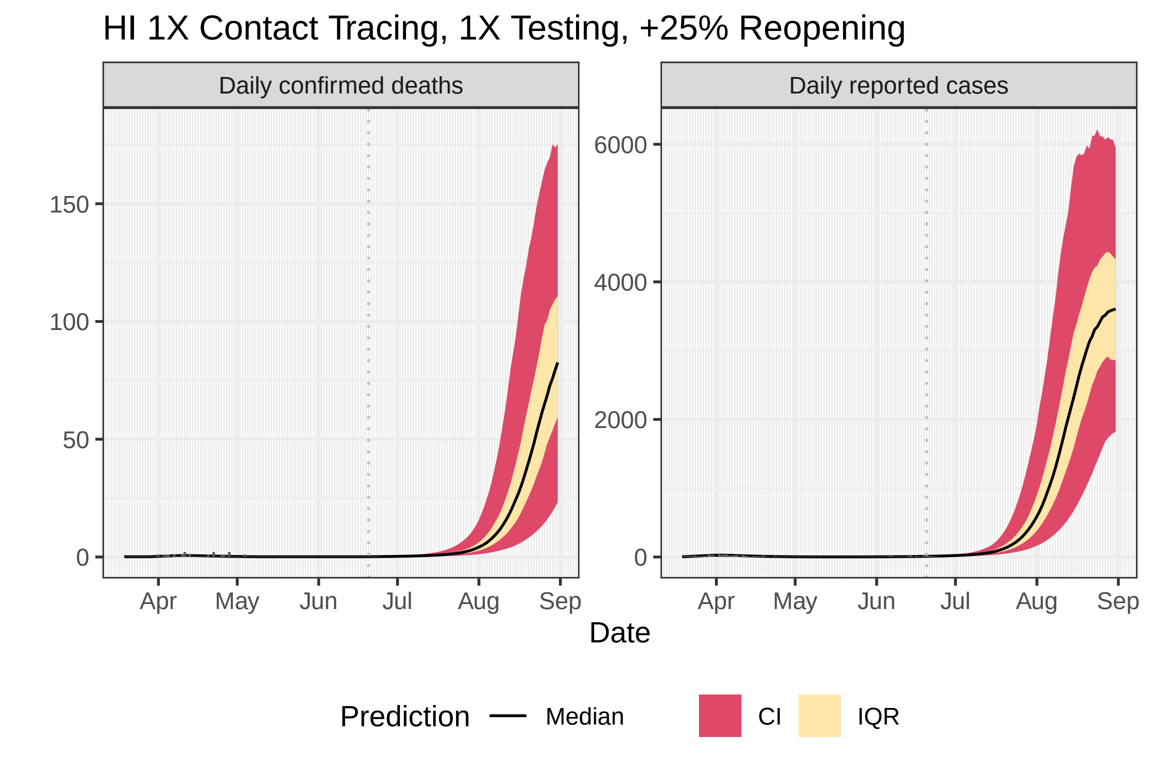

HI 1X Contact Tracing, 1X Testing, +25% Reopening
Daily confirmed deaths
Daily reported cases
0
50
100
150
0
2000
4000
6000
Apr
May
Jun
Jul
Aug
Sep
Date
Prediction
Median
CI
IQR

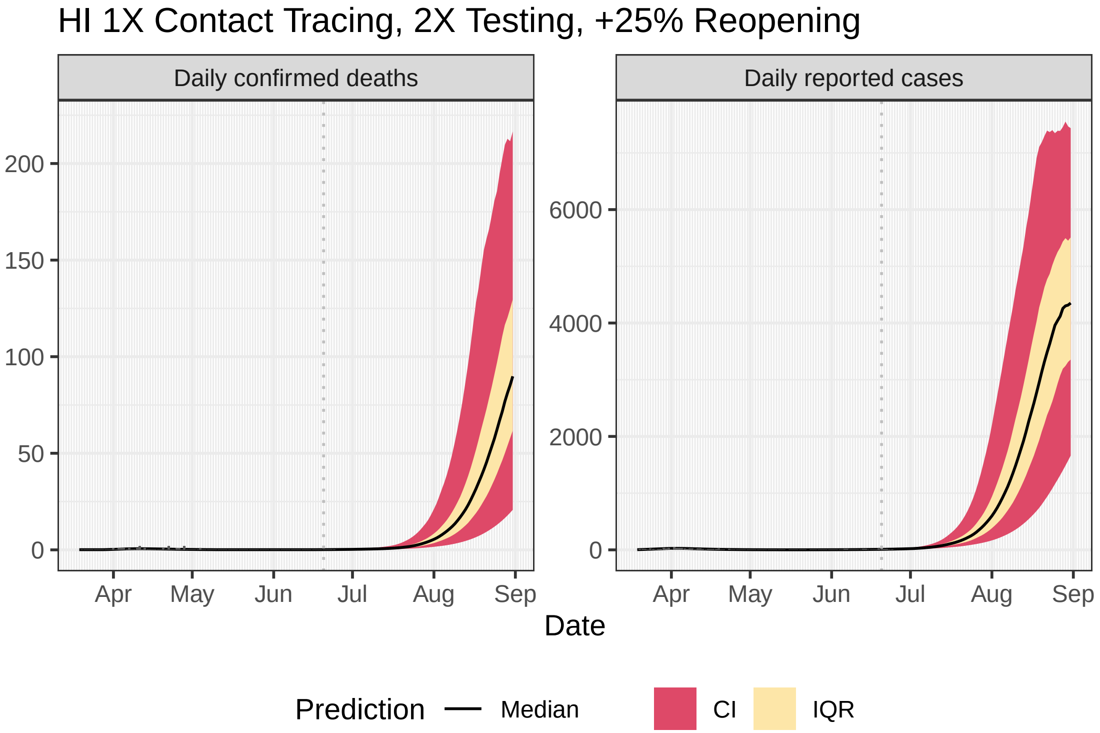

HI 1X Contact Tracing, 2X Testing, +25% Reopening
Daily confirmed deaths
Daily reported cases
200
150
100
50
0
6000
4000
2000
0
Apr
May
Jun
Jul
Aug
Sep
Date
Prediction
Median
CI
IQR

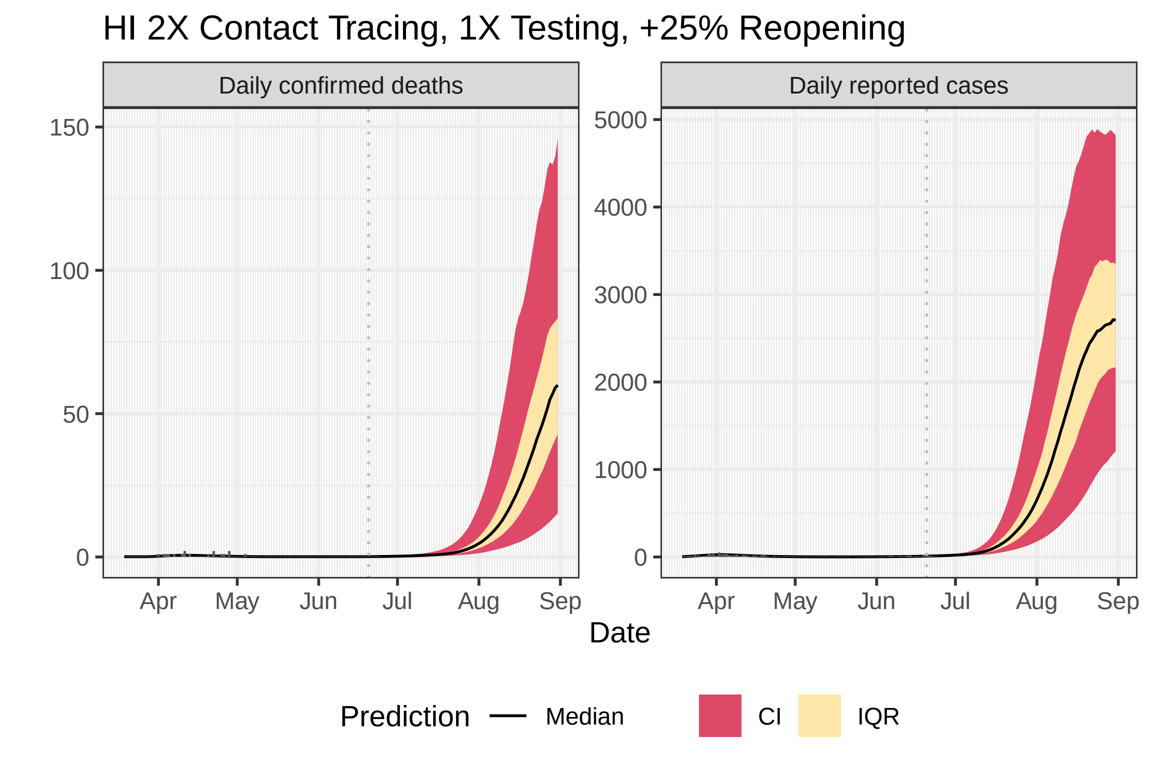

HI 2X Contact Tracing, 1X Testing, +25% Reopening
Daily confirmed deaths
Daily reported cases
150
100
50
0
5000
4000
3000
2000
1000
0
Apr
May
Jun
Jul
Aug
Sep
Date
Prediction
Median
CI
IQR

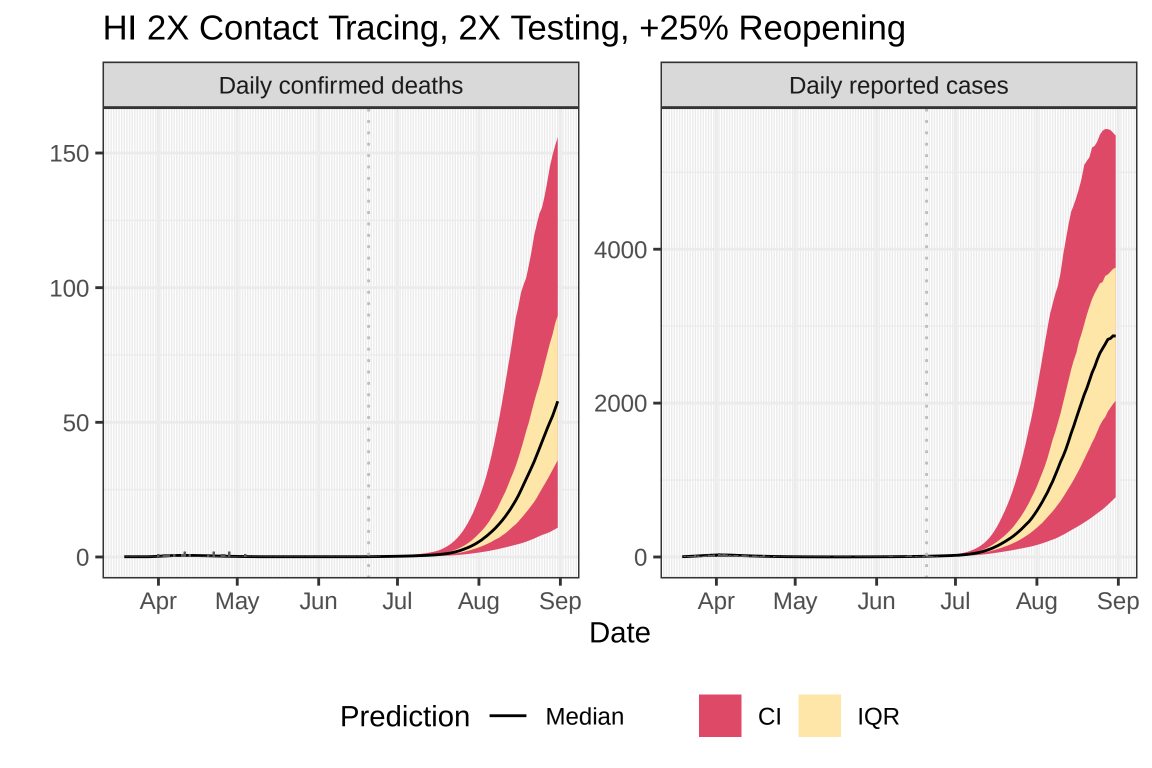

HI 2X Contact Tracing, 2X Testing, +25% Reopening
Daily confirmed deaths
Daily reported cases
150
100
50
0
4000
2000
0
Apr
May
Jun
Jul
Aug
Sep
Date
Prediction
Median
CI
IQR

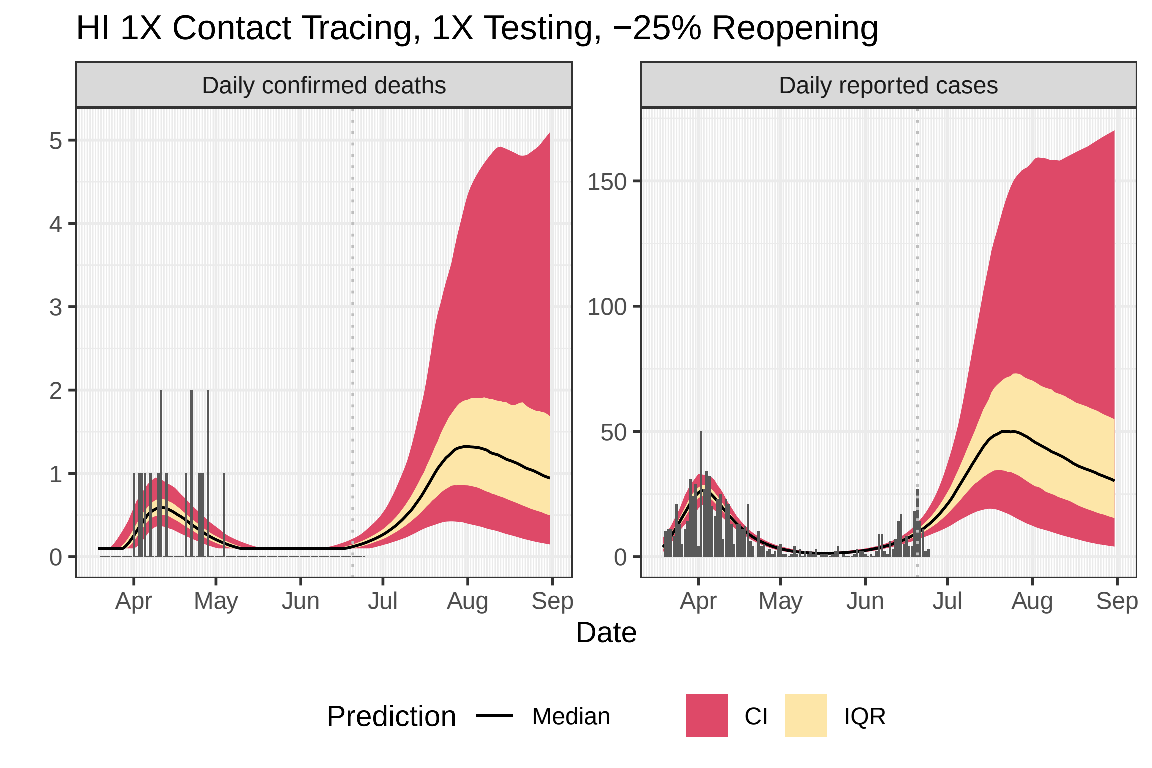
HI 1X Contact Tracing, 1X Testing, −25% Reopening
Daily confirmed deaths
Daily reported cases
0
1
2
3
4
5
0
50
100
150
Apr
May
Jun
Jul
Aug
Sep
Date
Prediction
Median
CI
IQR

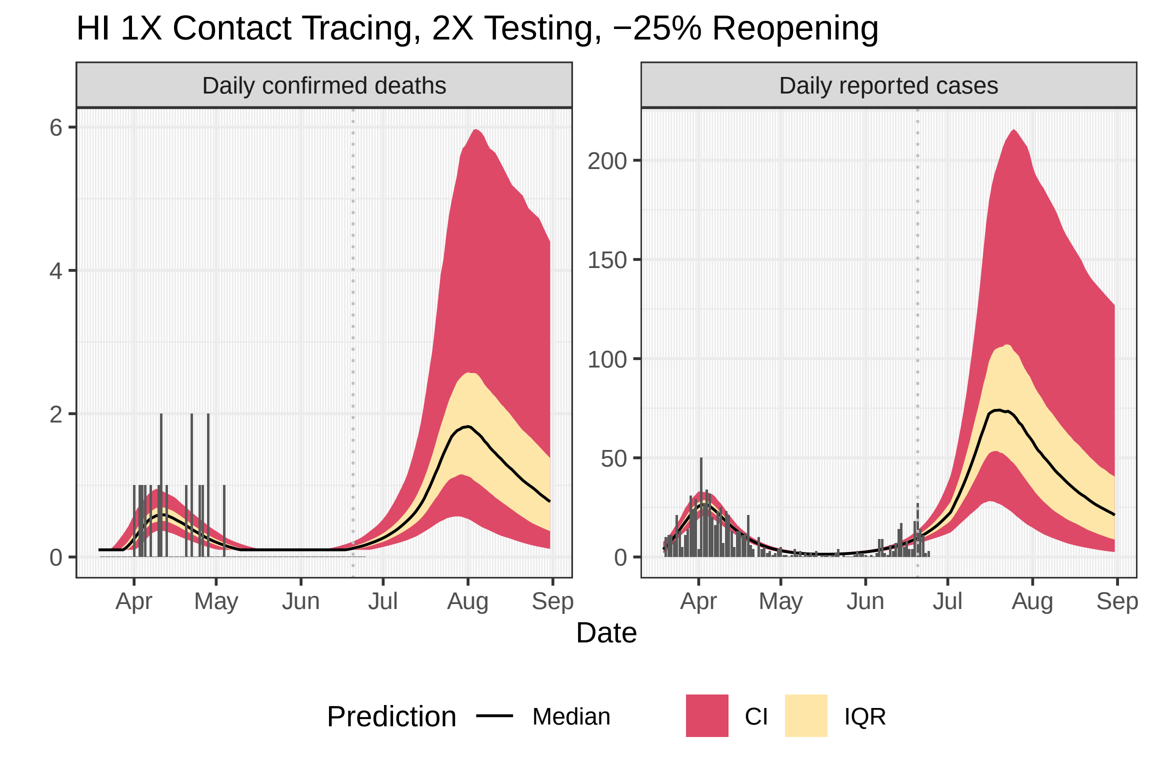

HI 1X Contact Tracing, 2X Testing, −25% Reopening
Daily confirmed deaths
Daily reported cases
0
2
4
6
0
50
100
150
200
Apr
May
Jun
Jul
Aug
Sep
Date
Prediction
Median
CI
IQR

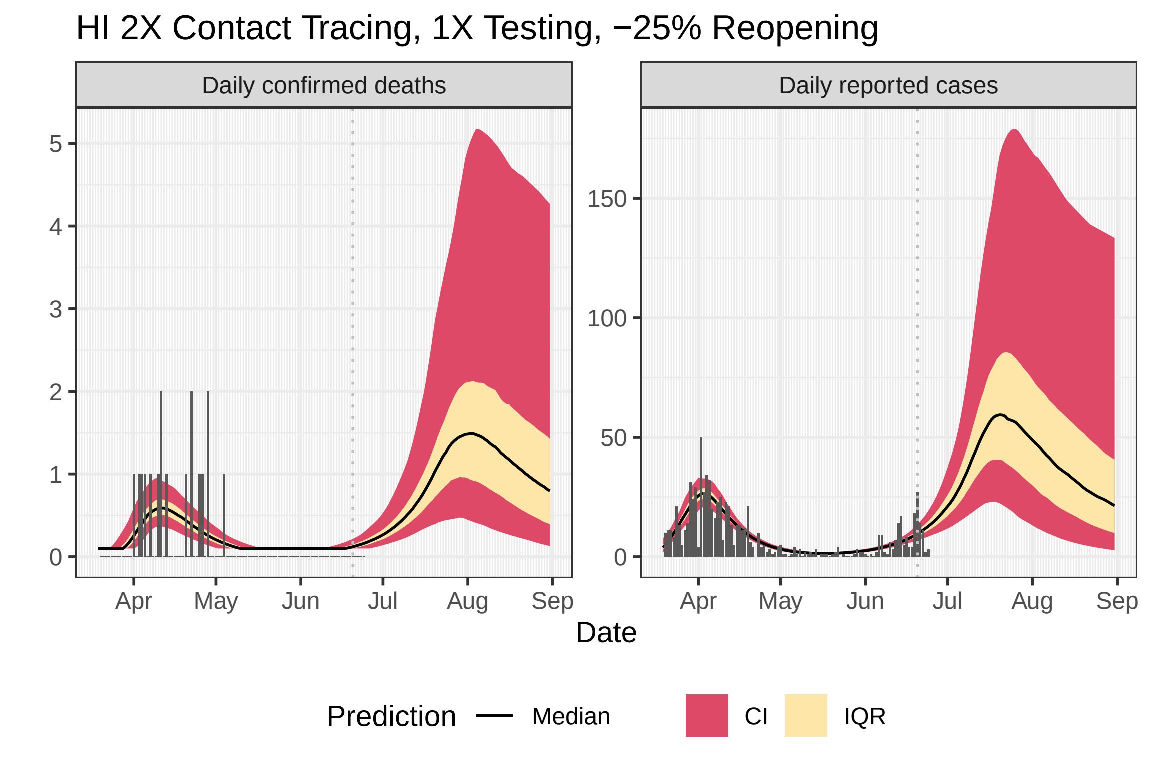

HI 2X Contact Tracing, 1X Testing, −25% Reopening
Daily confirmed deaths
Daily reported cases
0
1
2
3
4
5
0
50
100
150
Apr
May
Jun
Jul
Aug
Sep
Date
Prediction
Median
CI
IQR

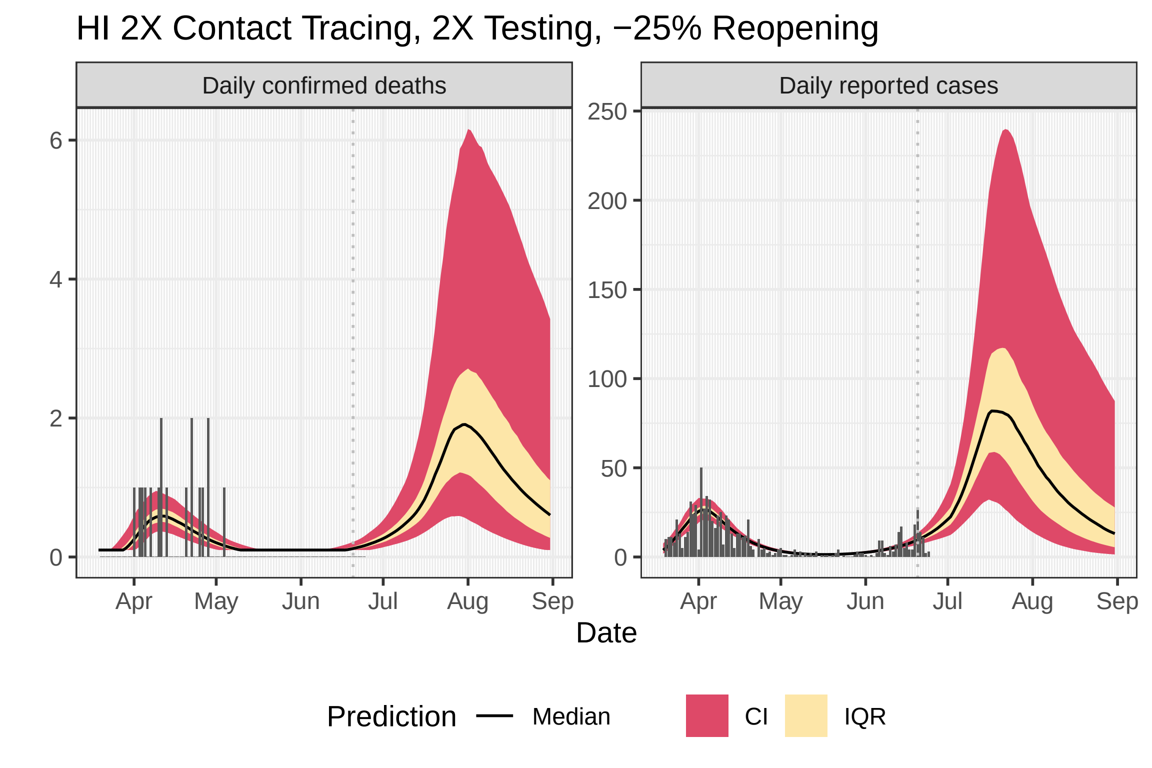

HI 2X Contact Tracing, 2X Testing, −25% Reopening
Daily confirmed deaths
Daily reported cases
6
4
2
0
250
200
150
100
50
0
Apr
May
Jun
Jul
Aug
Sep
Date
Prediction
Median
CI
IQR

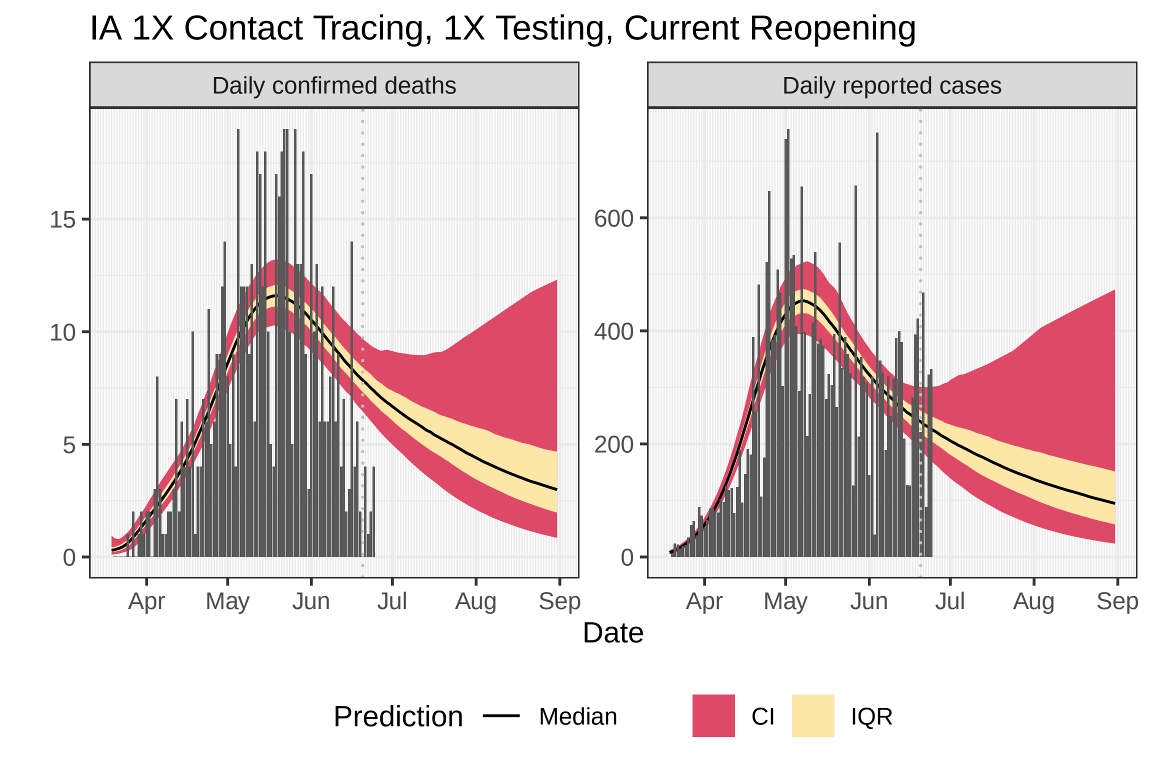
IA 1X Contact Tracing, 1X Testing, Current Reopening
Daily confirmed deaths
Daily reported cases
0
5
10
15
0
200
400
600
Apr
May
Jun
Jul
Aug
Sep
Date
Prediction
Median
CI
IQR

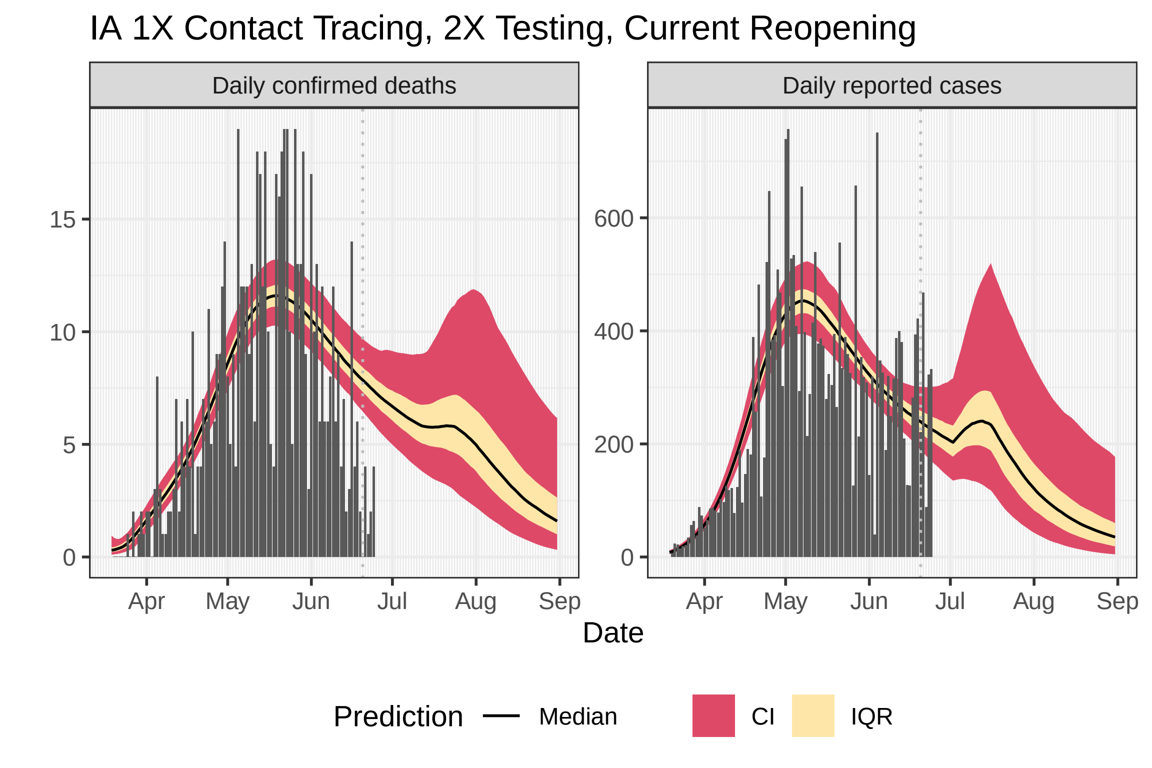

IA 1X Contact Tracing, 2X Testing, Current Reopening
Daily confirmed deaths
Daily reported cases
15
10
5
0
600
400
200
0
Apr
May
Jun
Jul
Aug
Sep
Date
Prediction
Median
CI
IQR

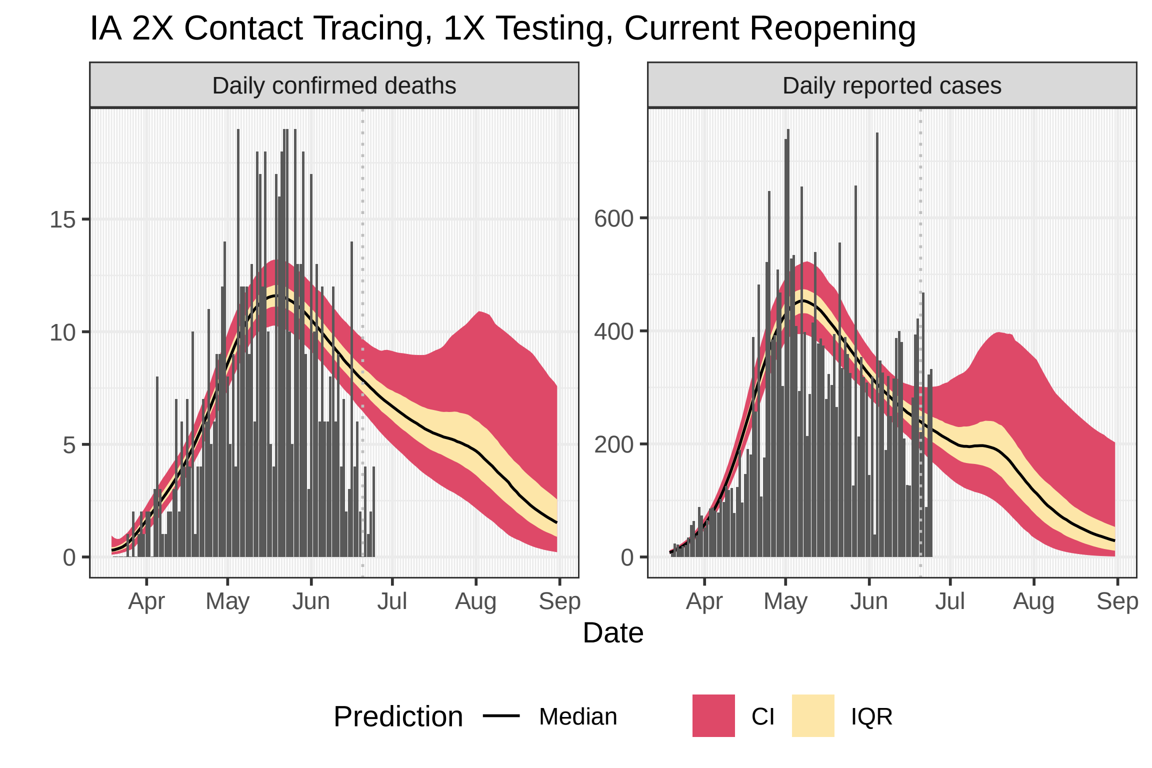
IA 2X Contact Tracing, 1X Testing, Current Reopening
Daily confirmed deaths
Daily reported cases
15
10
5
0
600
400
200
0
Apr
May
Jun
Jul
Aug
Sep
Date
Prediction
Median
CI
IQR

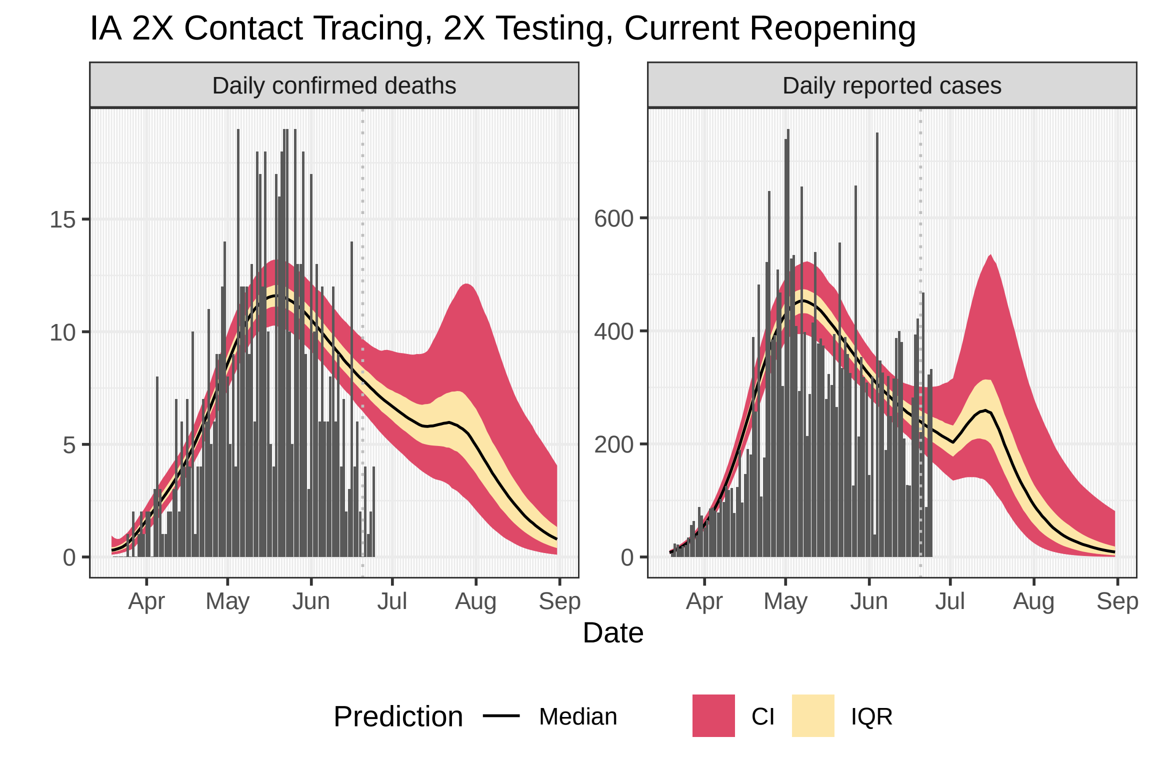
IA 2X Contact Tracing, 2X Testing, Current Reopening
Daily confirmed deaths
Daily reported cases
15
10
5
0
600
400
200
0
Apr
May
Jun
Jul
Aug
Sep
Date
Prediction
Median
CI
IQR

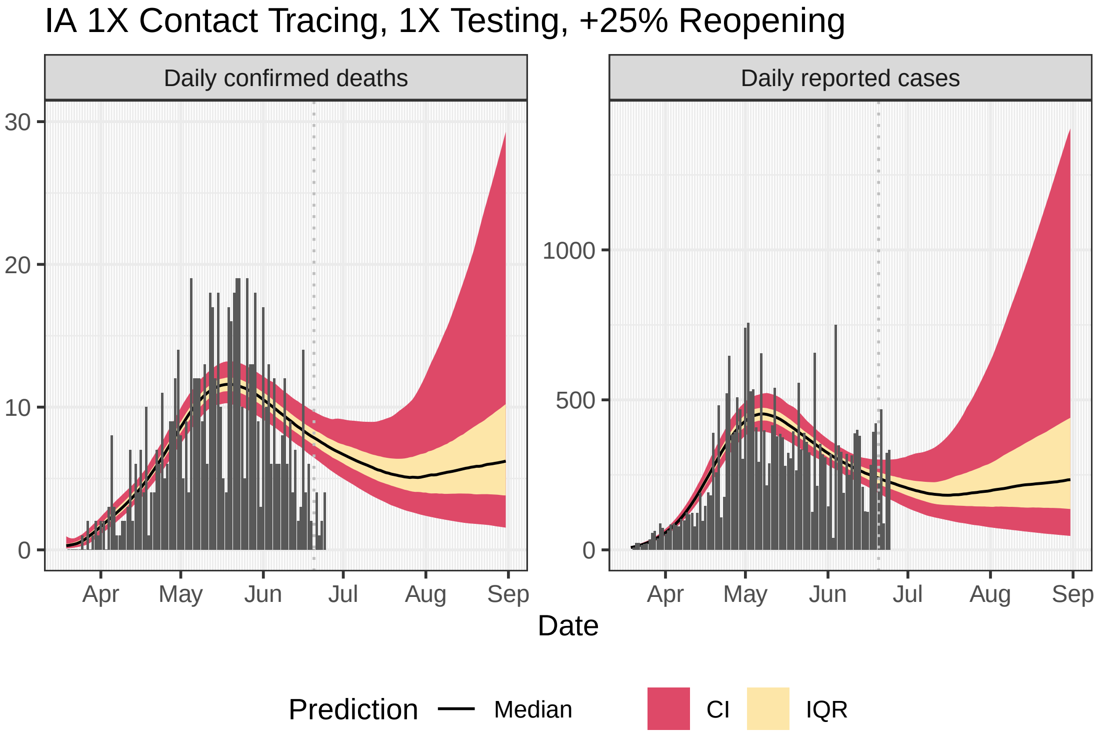
IA 1X Contact Tracing, 1X Testing, +25% Reopening
Daily confirmed deaths
Daily reported cases
30
20
10
0
1000
500
0
Apr
May
Jun
Jul
Aug
Sep
Date
Prediction
Median
CI
IQR

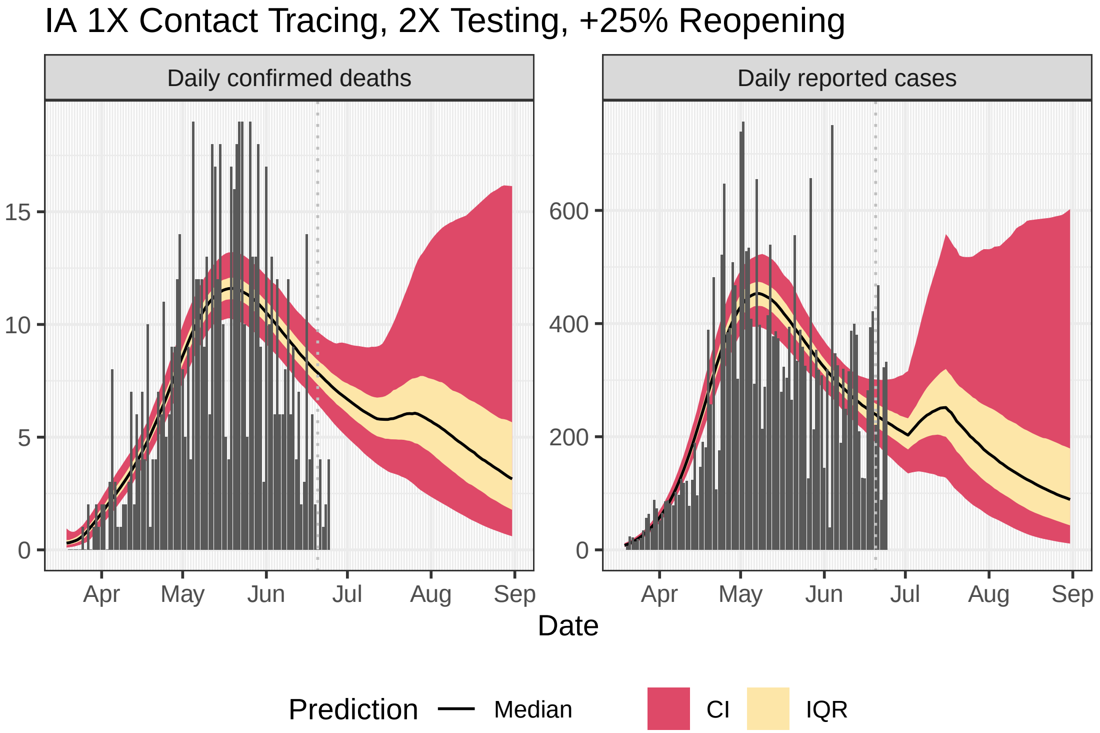

IA 1X Contact Tracing, 2X Testing, +25% Reopening
Daily confirmed deaths
Daily reported cases
0
5
10
15
0
200
400
600
Apr
May
Jun
Jul
Aug
Sep
Date
Prediction
Median
CI
IQR

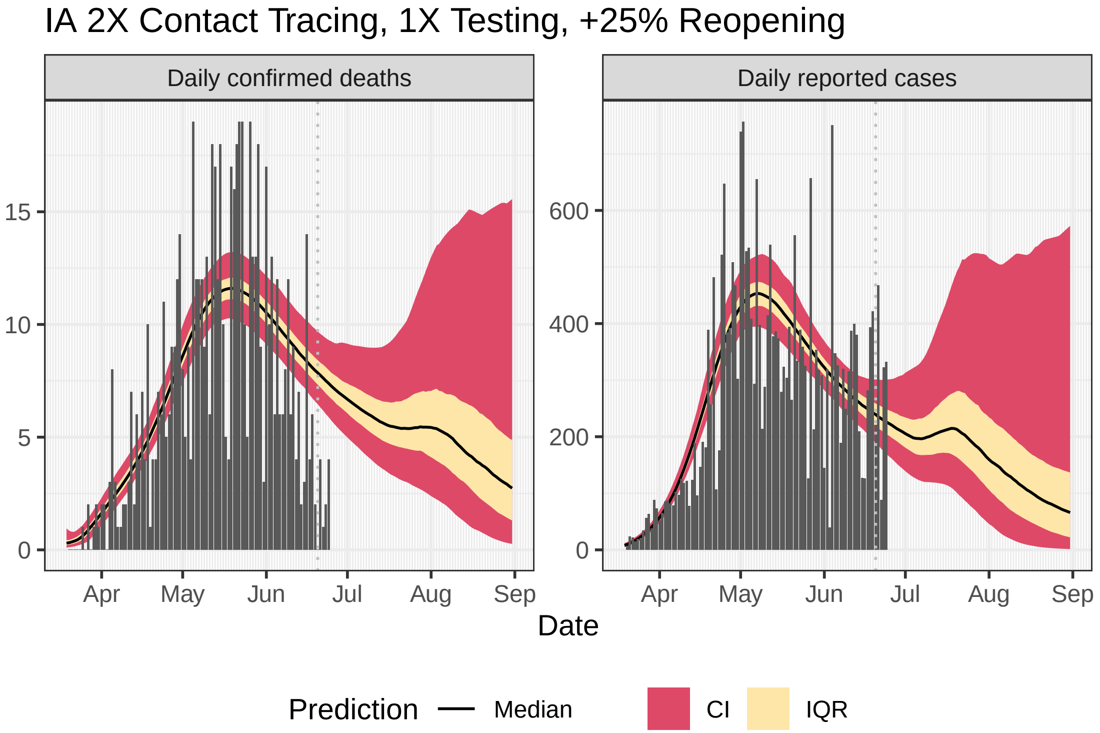

IA 2X Contact Tracing, 1X Testing, +25% Reopening
Daily confirmed deaths
Daily reported cases
15
10
5
0
600
400
200
0
Apr
May
Jun
Jul
Aug
Sep
Date
Prediction
Median
CI
IQR

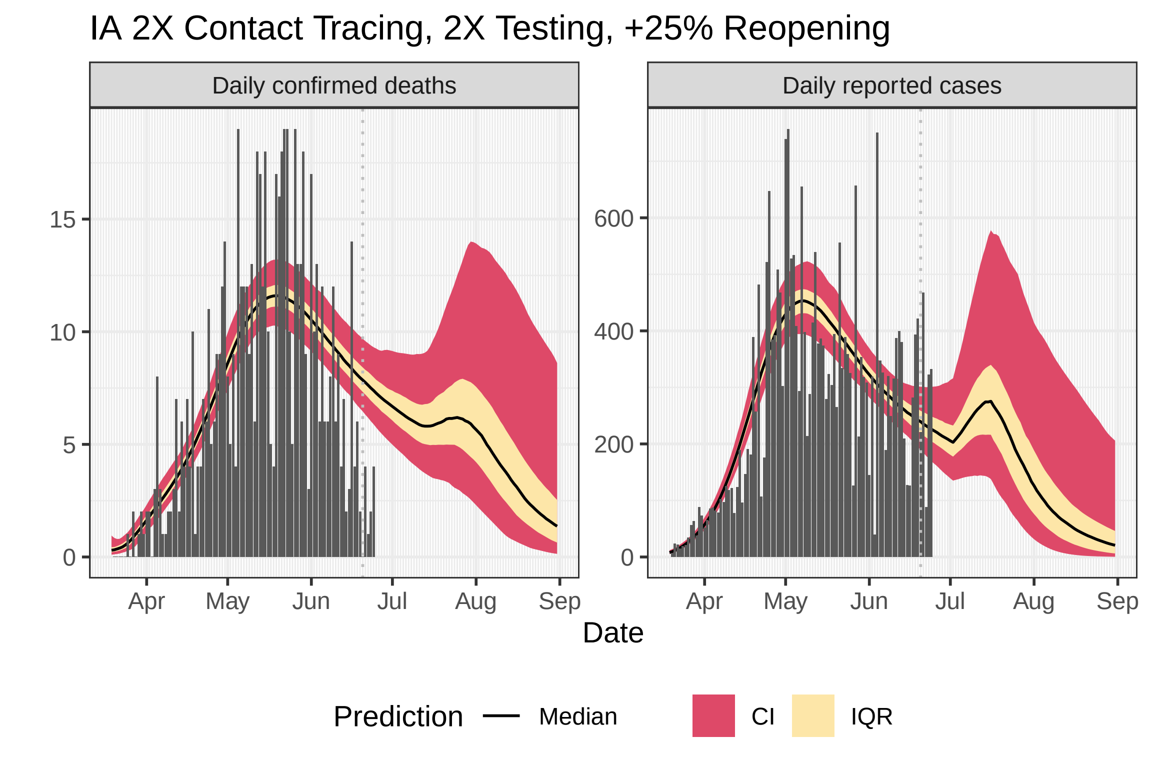
IA 2X Contact Tracing, 2X Testing, +25% Reopening
Daily confirmed deaths
Daily reported cases
15
10
5
0
600
400
200
0
Apr
May
Jun
Jul
Aug
Sep
Date
Prediction
Median
CI
IQR

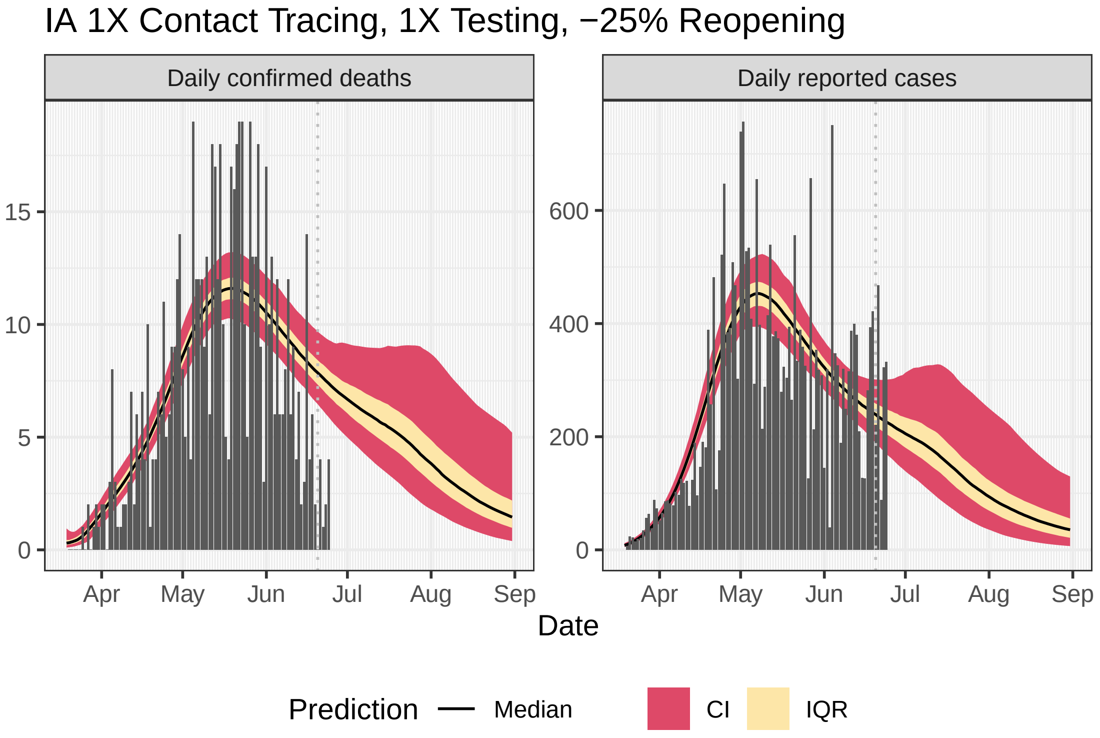
IA 1X Contact Tracing, 1X Testing, −25% Reopening
Daily confirmed deaths
Daily reported cases
0
5
10
15
0
200
400
600
Apr
May
Jun
Jul
Aug
Sep
Date
Prediction
Median
CI
IQR

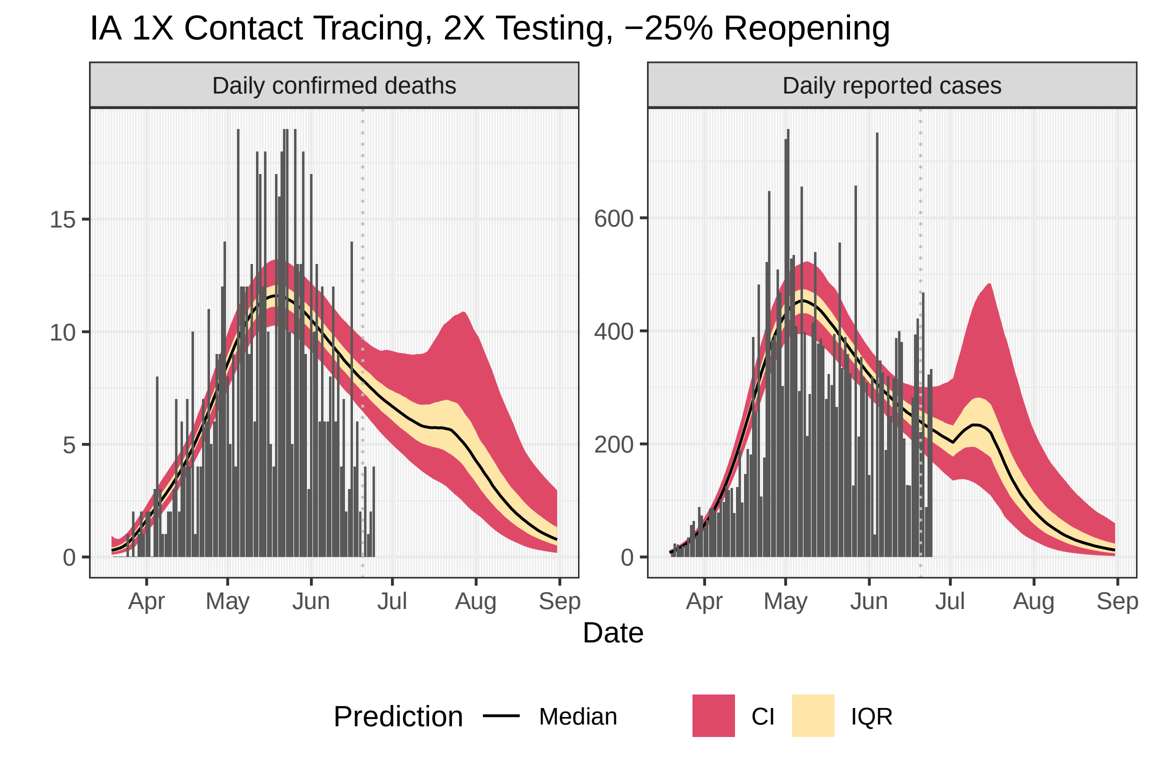
IA 1X Contact Tracing, 2X Testing, −25% Reopening
Daily confirmed deaths
Daily reported cases
15
10
5
0
600
400
200
0
Apr
May
Jun
Jul
Aug
Sep
Date
Prediction
Median
CI
IQR

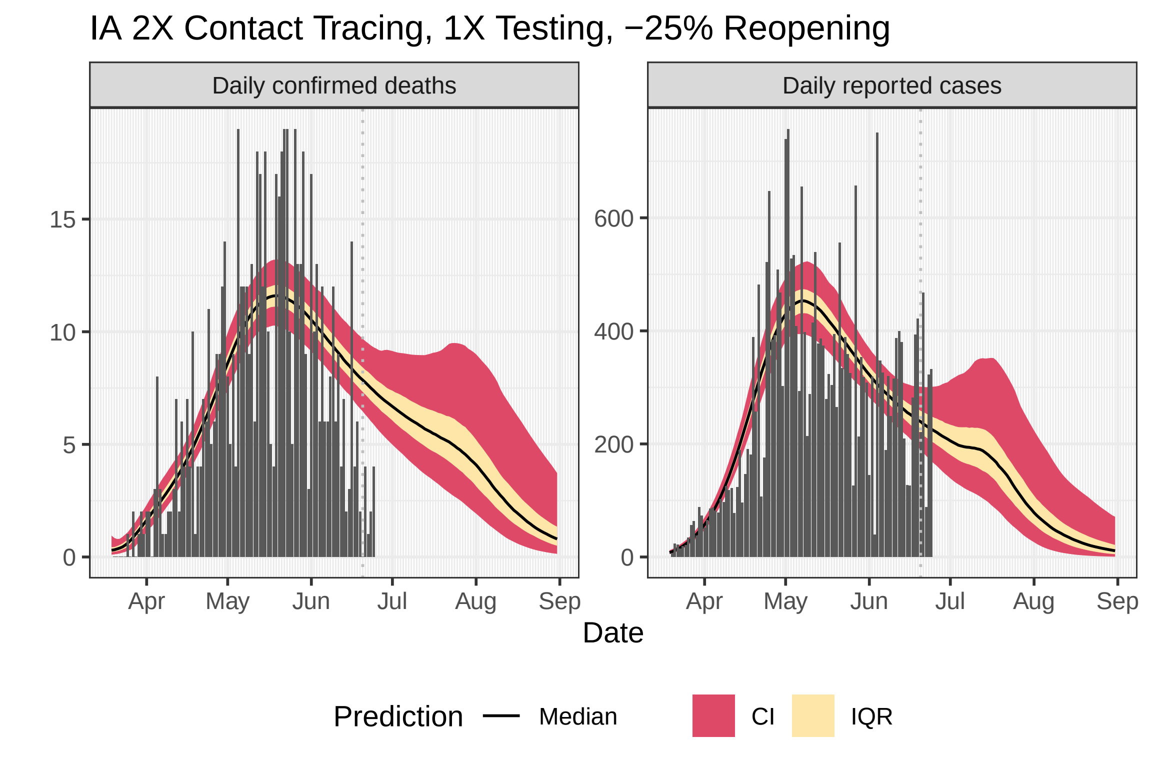
IA 2X Contact Tracing, 1X Testing, −25% Reopening
Daily confirmed deaths
Daily reported cases
0
5
10
15
0
200
400
600
Apr
May
Jun
Jul
Aug
Sep
Date
Prediction
Median
CI
IQR

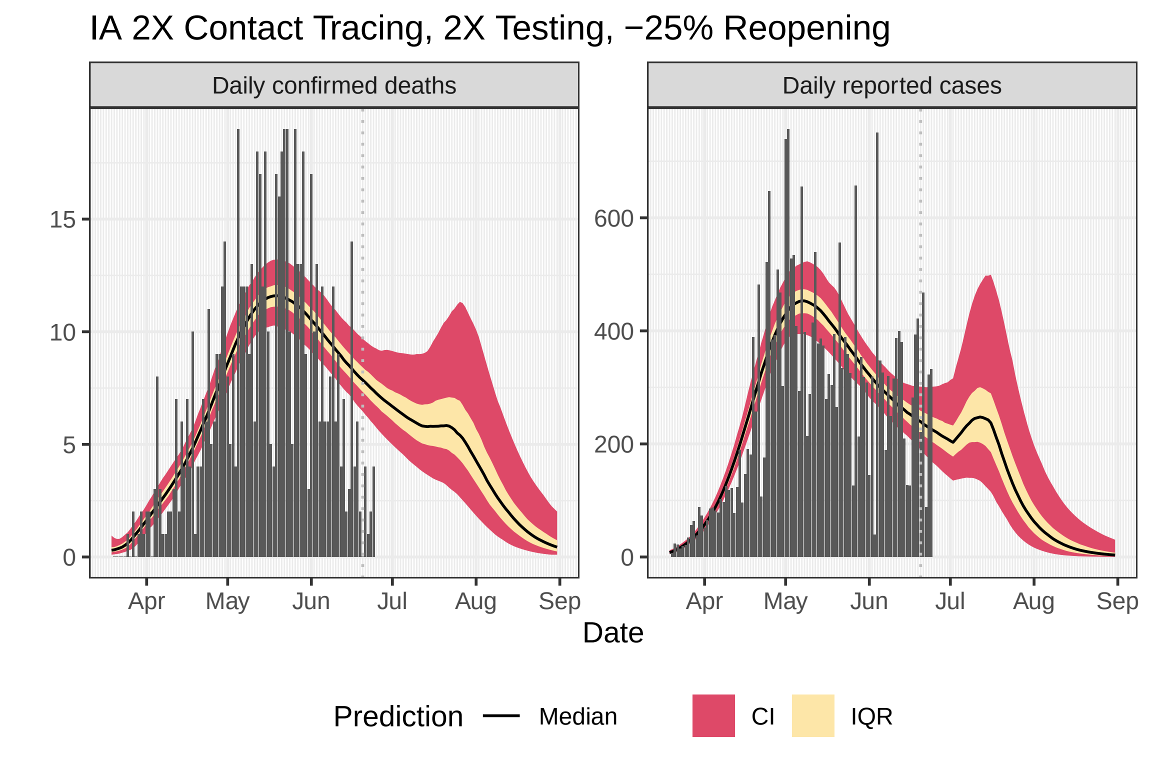

IA 2X Contact Tracing, 2X Testing, −25% Reopening
Daily confirmed deaths
Daily reported cases
15
10
5
0
600
400
200
0
Apr
May
Jun
Jul
Aug
Sep
Date
Prediction
Median
CI
IQR

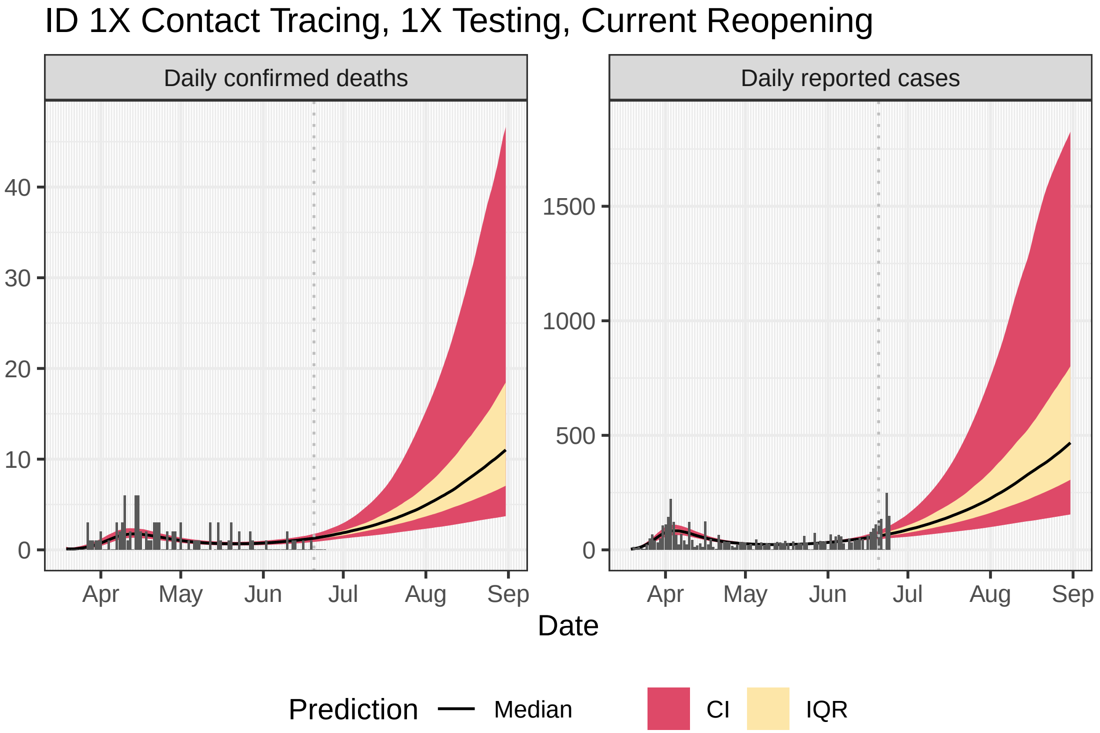

ID 1X Contact Tracing, 1X Testing, Current Reopening
Daily confirmed deaths
Daily reported cases
40
30
20
10
0
1500
1000
500
0
Apr
May
Jun
Jul
Aug
Sep
Date
Prediction
Median
CI
IQR

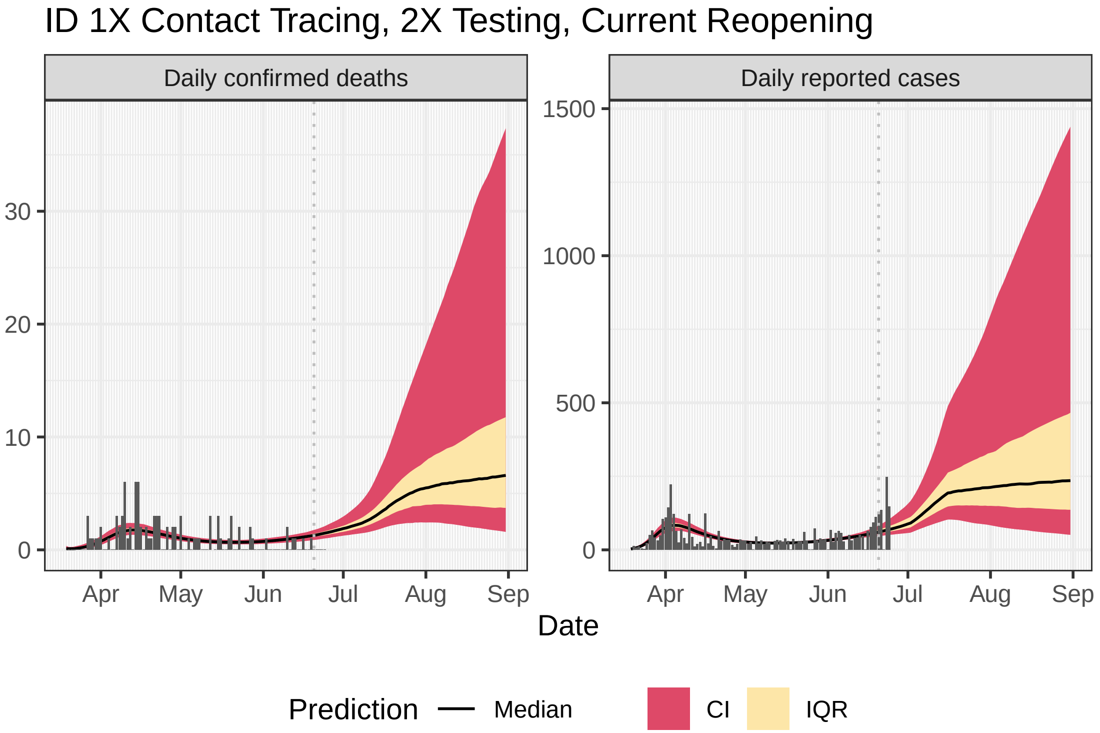

ID 1X Contact Tracing, 2X Testing, Current Reopening
Daily confirmed deaths
Daily reported cases
30
20
10
0
1500
1000
500
0
Apr
May
Jun
Jul
Aug
Sep
Date
Prediction
Median
CI
IQR

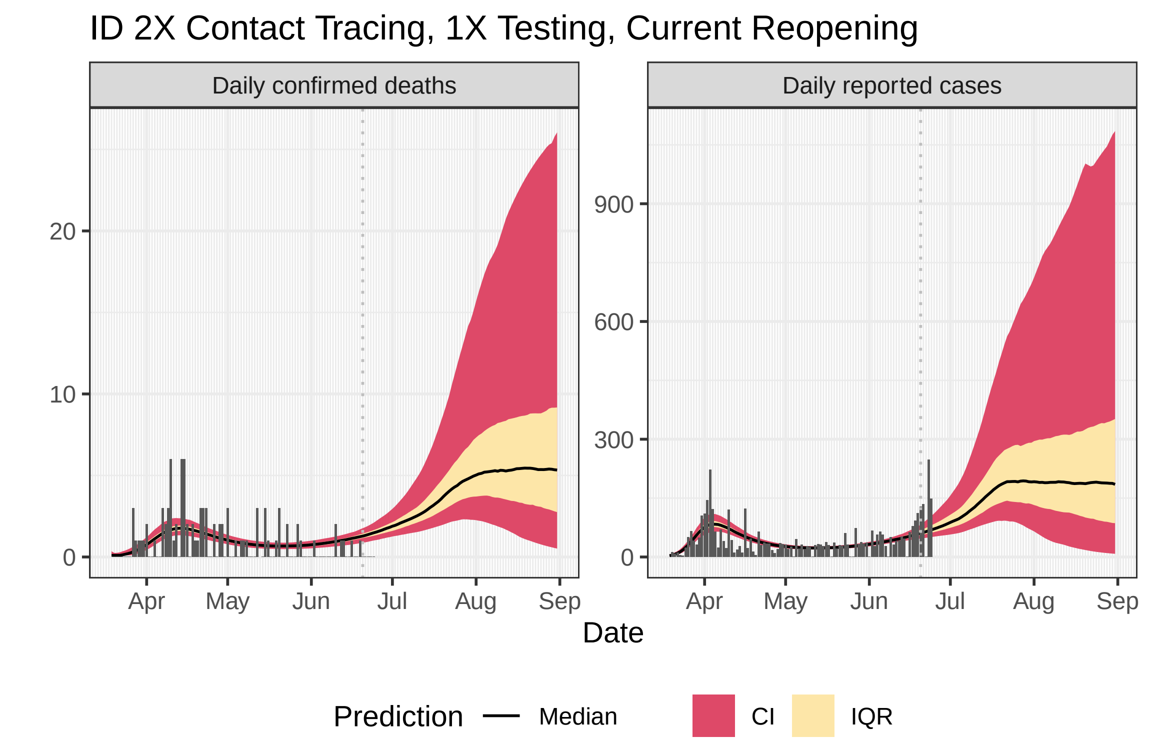
ID 2X Contact Tracing, 1X Testing, Current Reopening
Daily confirmed deaths
Daily reported cases
0
10
20
0
300
600
900
Apr
May
Jun
Jul
Aug
Sep
Date
Prediction
Median
CI
IQR

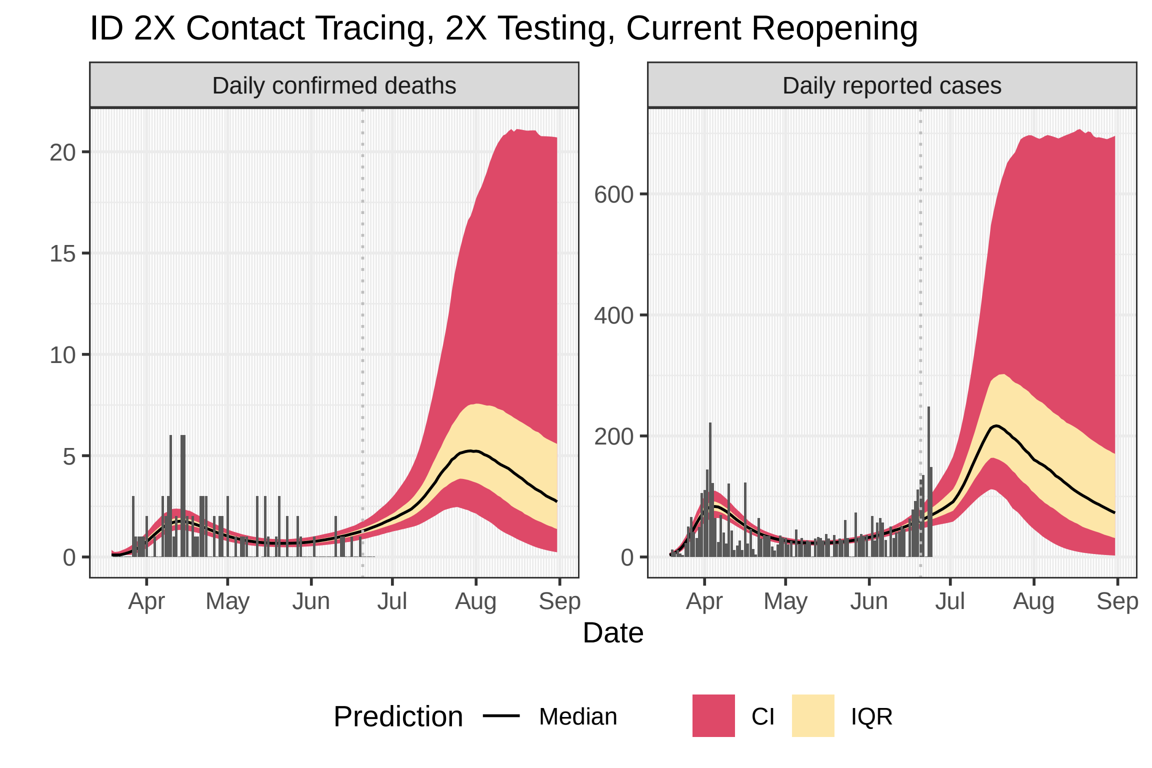
ID 2X Contact Tracing, 2X Testing, Current Reopening
Daily confirmed deaths
Daily reported cases
0
5
10
15
20
0
200
400
600
Apr
May
Jun
Jul
Aug
Sep
Date
Prediction
Median
CI
IQR

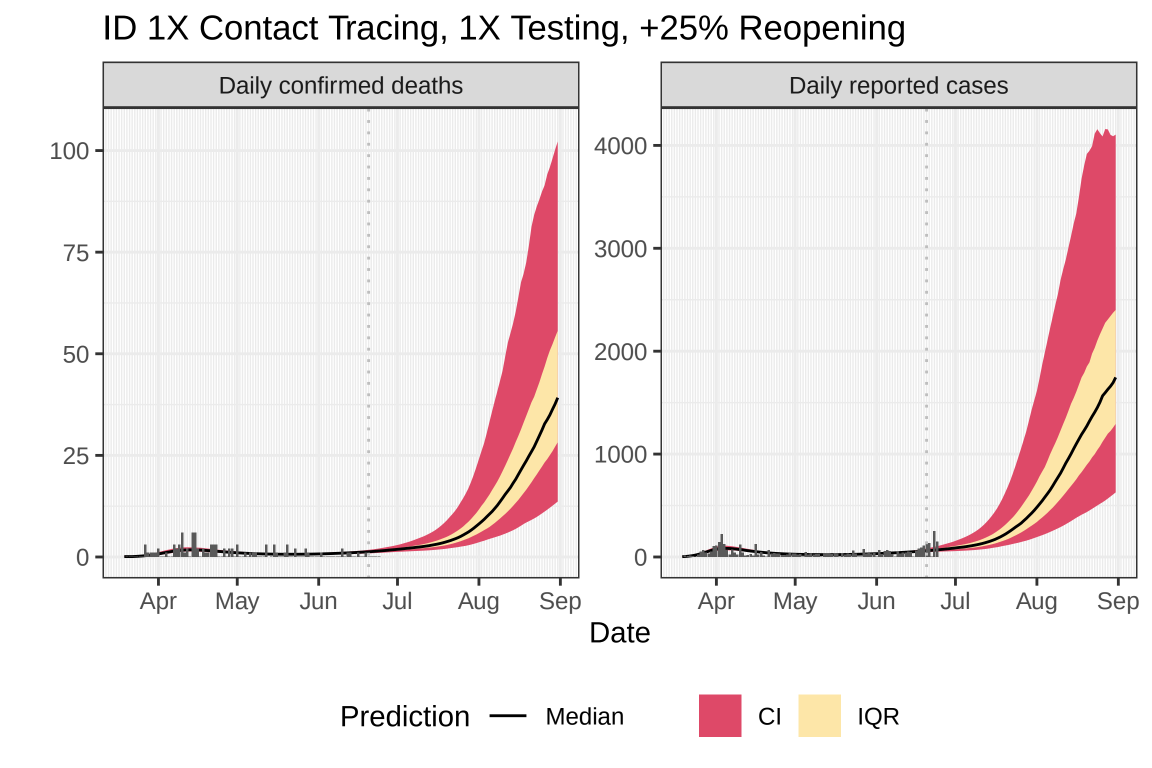
ID 1X Contact Tracing, 1X Testing, +25% Reopening
Daily confirmed deaths
Daily reported cases
100
75
50
25
0
4000
3000
2000
1000
0
Apr
May
Jun
Jul
Aug
Sep
Date
Prediction
Median
CI
IQR

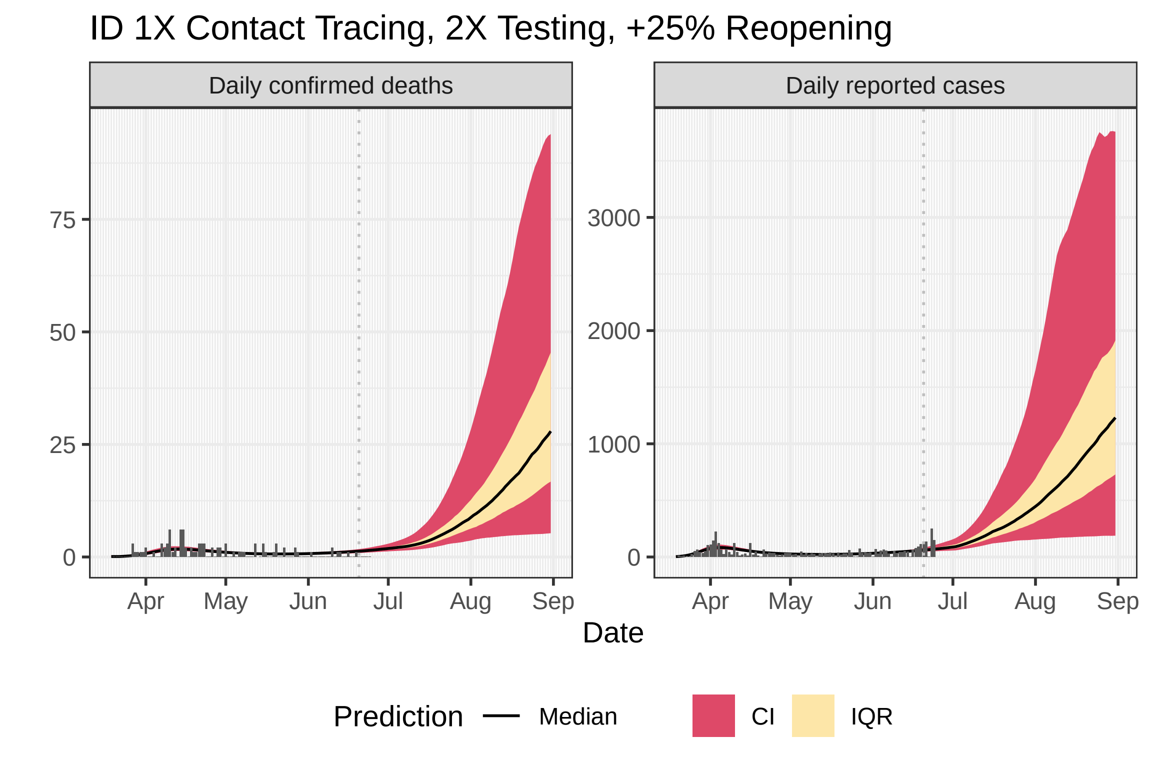

ID 1X Contact Tracing, 2X Testing, +25% Reopening
Daily confirmed deaths
Daily reported cases
75
50
25
0
3000
2000
1000
0
Apr
May
Jun
Jul
Aug
Sep
Date
Prediction
Median
CI
IQR

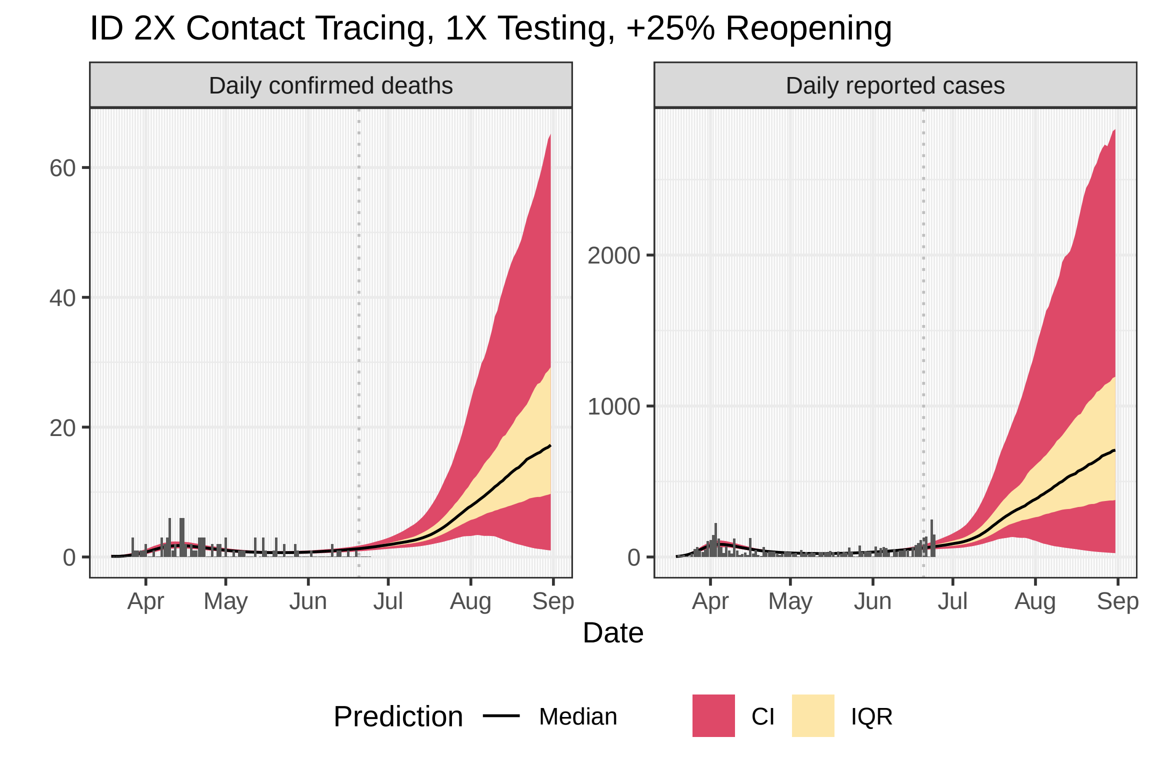

ID 2X Contact Tracing, 1X Testing, +25% Reopening
Daily confirmed deaths
Daily reported cases
60
40
20
0
2000
1000
0
Apr
May
Jun
Jul
Aug
Sep
Date
Prediction
Median
CI
IQR

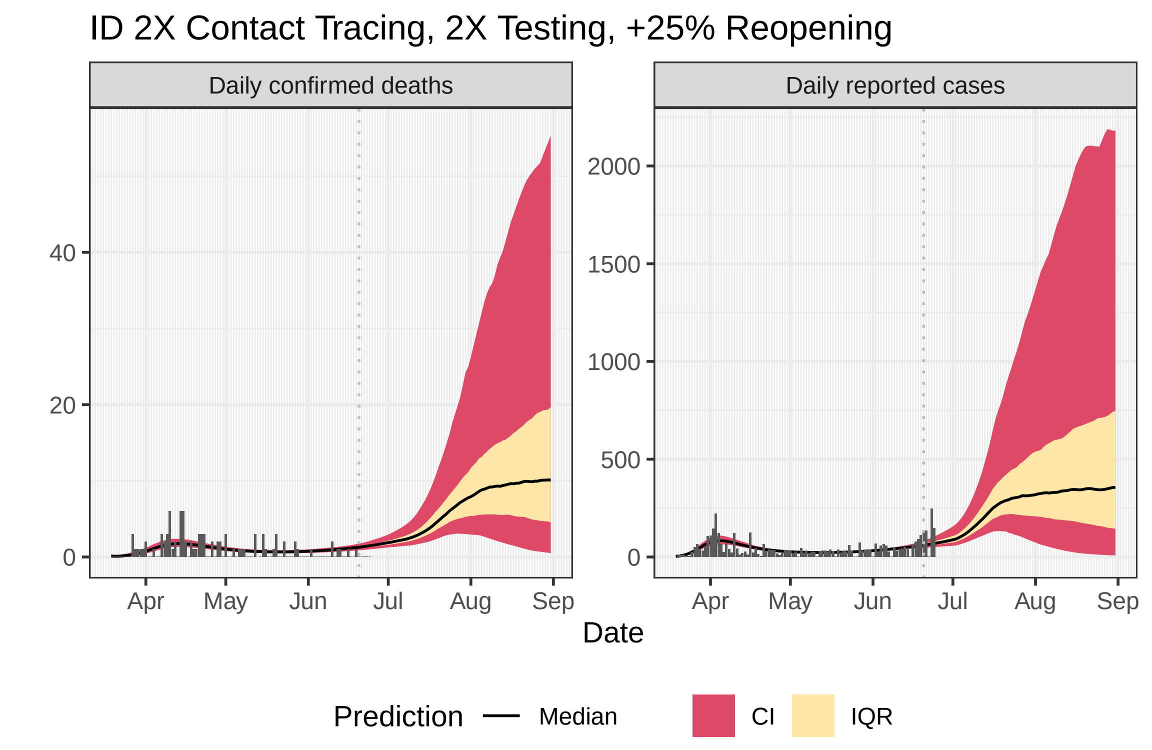
ID 2X Contact Tracing, 2X Testing, +25% Reopening
Daily confirmed deaths
Daily reported cases
0
20
40
0
500
1000
1500
2000
Apr
May
Jun
Jul
Aug
Sep
Date
Prediction
Median
CI
IQR

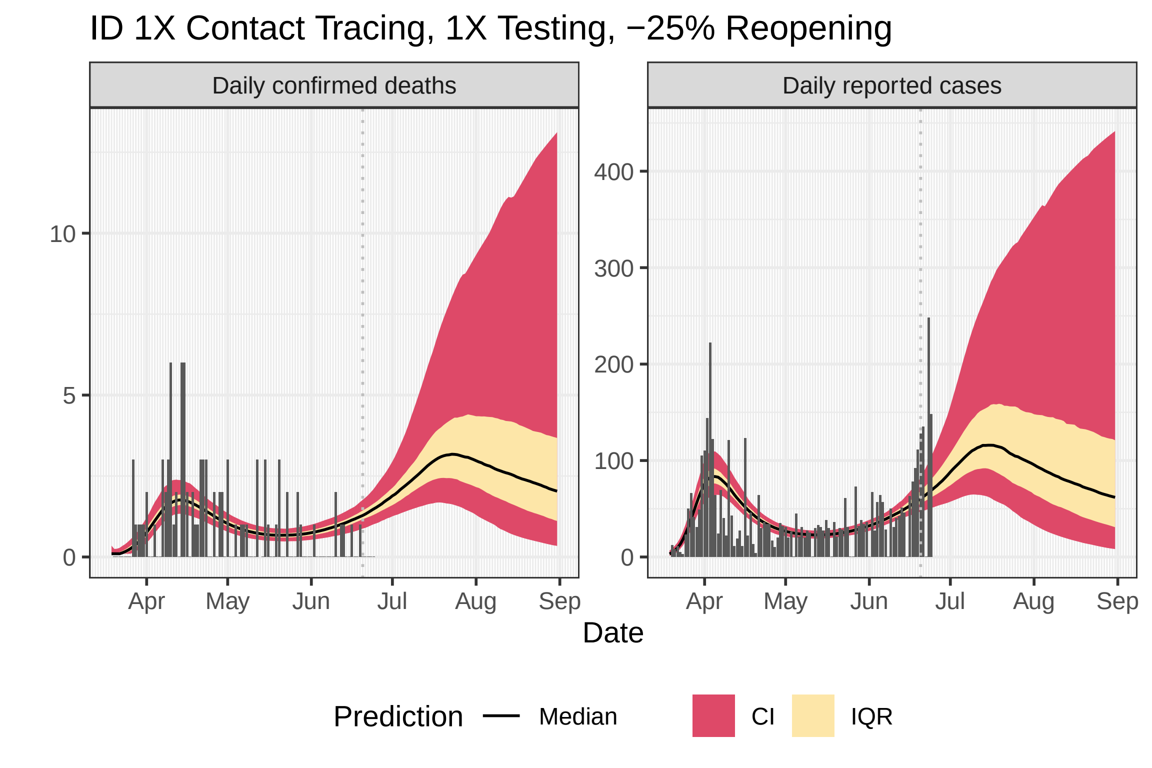

ID 1X Contact Tracing, 1X Testing, −25% Reopening
Daily confirmed deaths
Daily reported cases
0
5
10
100
200
300
400
Apr
May
Jun
Jul
Aug
Sep
Date
Prediction
Median
CI
IQR

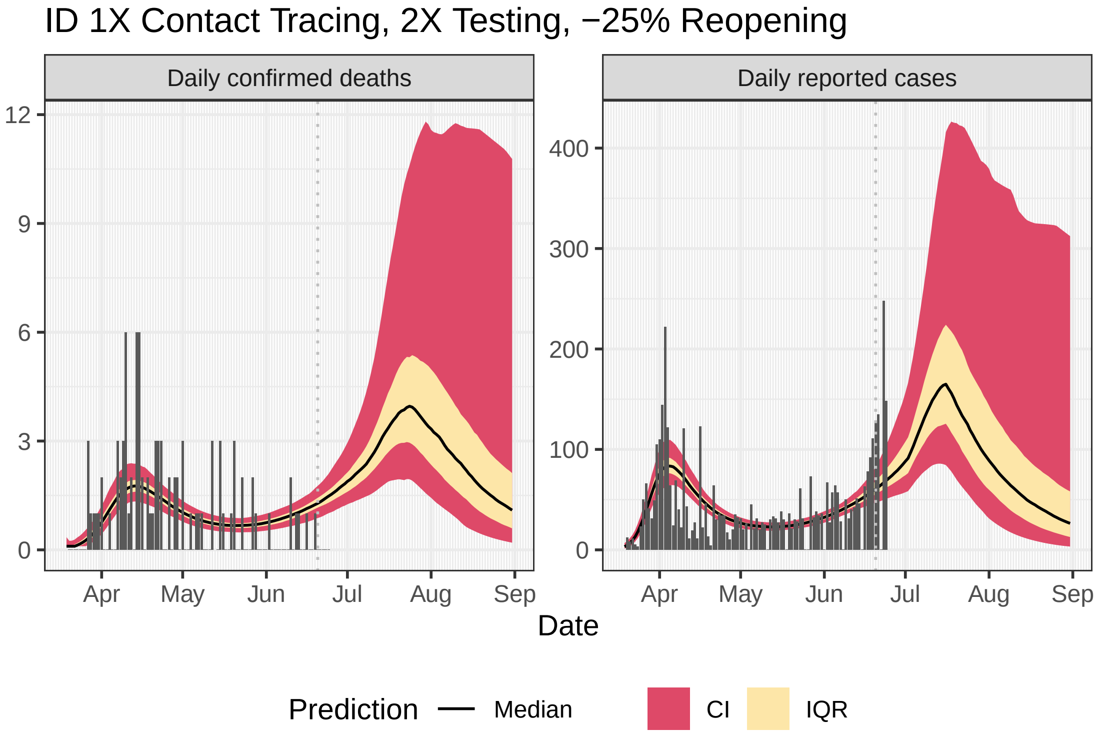
ID 1X Contact Tracing, 2X Testing, −25% Reopening
Daily confirmed deaths
Daily reported cases
12
9
6
3
0
400
300
200
100
0
Apr
May
Jun
Jul
Aug
Sep
Date
Prediction
Median
CI
IQR

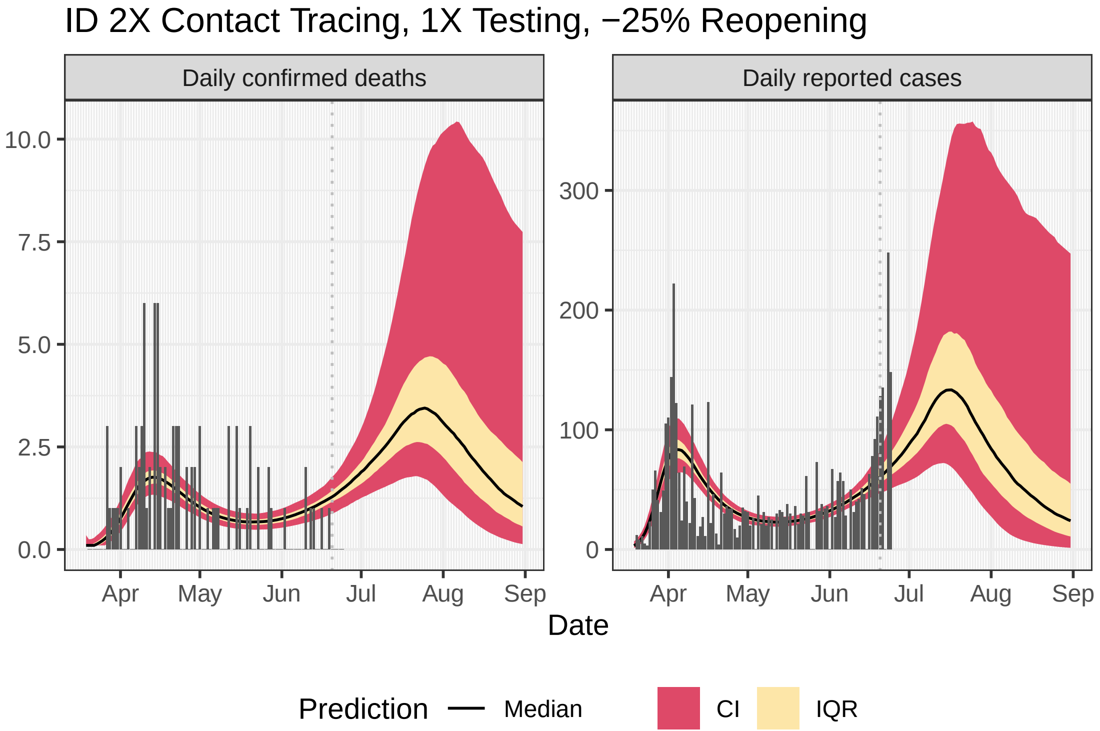

ID 2X Contact Tracing, 1X Testing, −25% Reopening
Daily confirmed deaths
Daily reported cases
10.0
7.5
5.0
2.5
0.0
300
200
100
0
Apr
May
Jun
Jul
Aug
Sep
Date
Prediction
Median
CI
IQR

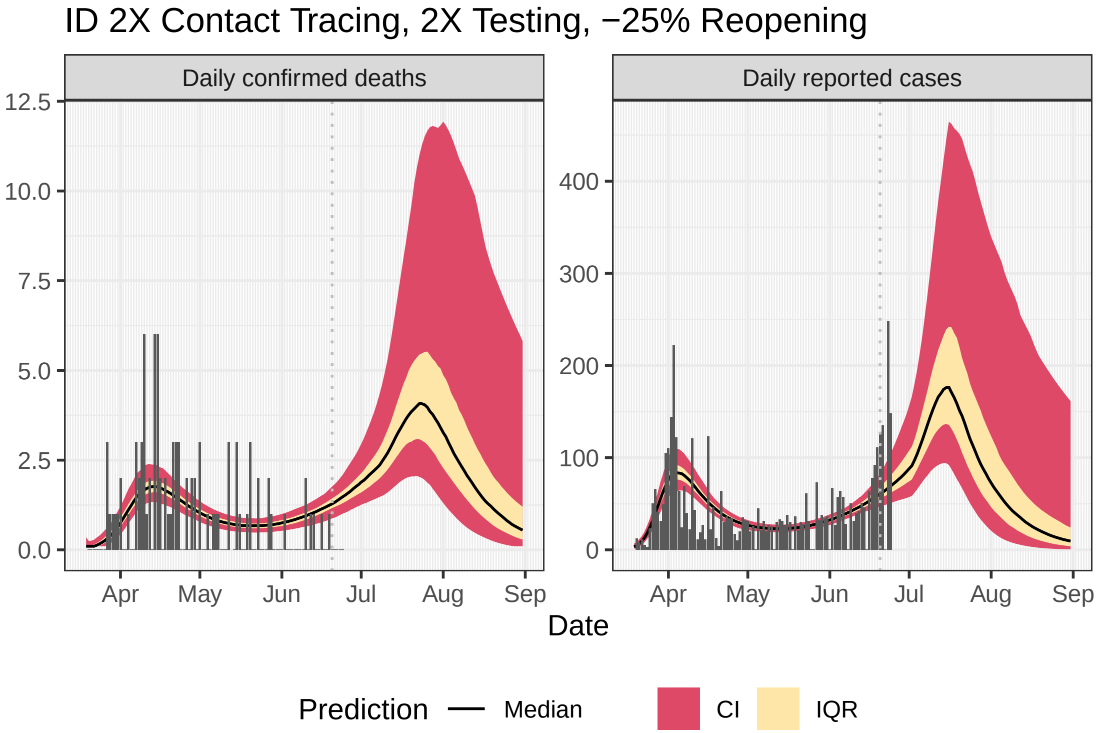

ID 2X Contact Tracing, 2X Testing, −25% Reopening
Daily confirmed deaths
Daily reported cases
12.5
10.0
7.5
5.0
2.5
0.0
400
300
200
100
0
Apr
May
Jun
Jul
Aug
Sep
Date
Prediction
Median
CI
IQR

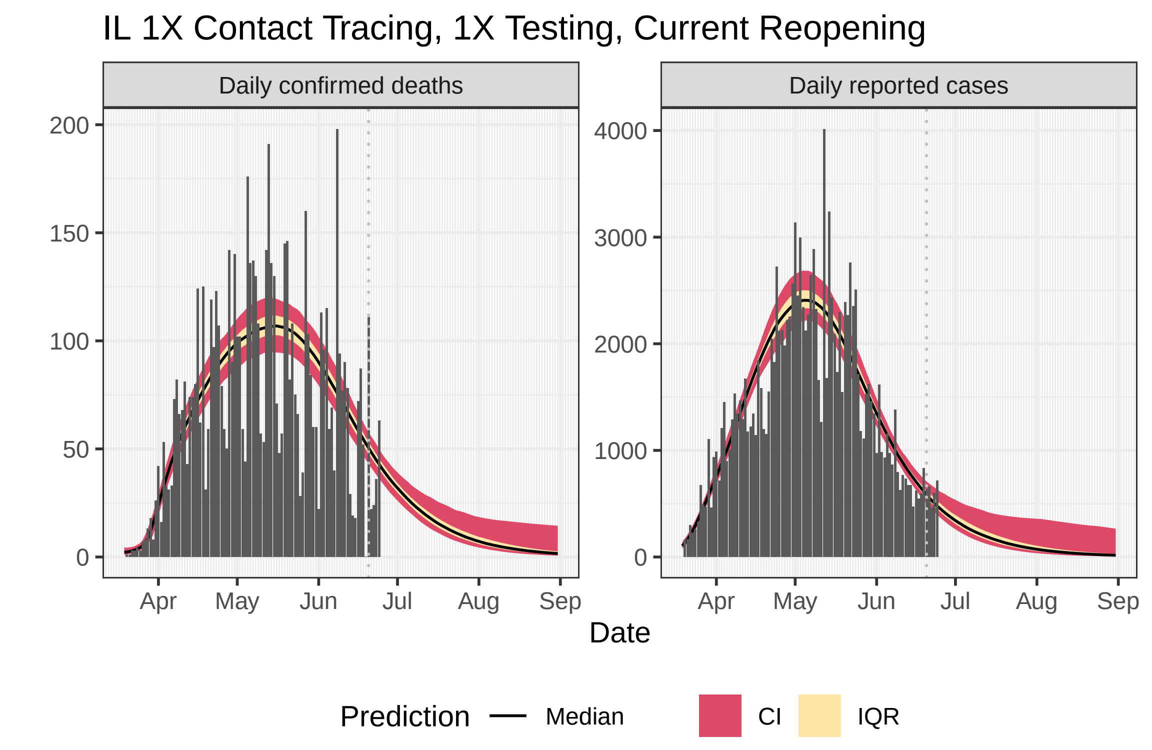
IL 1X Contact Tracing, 1X Testing, Current Reopening
Daily confirmed deaths
Daily reported cases
200
150
100
50
0
4000
3000
2000
1000
0
Apr
May
Jun
Jul
Aug
Sep
Date
Prediction
Median
CI
IQR

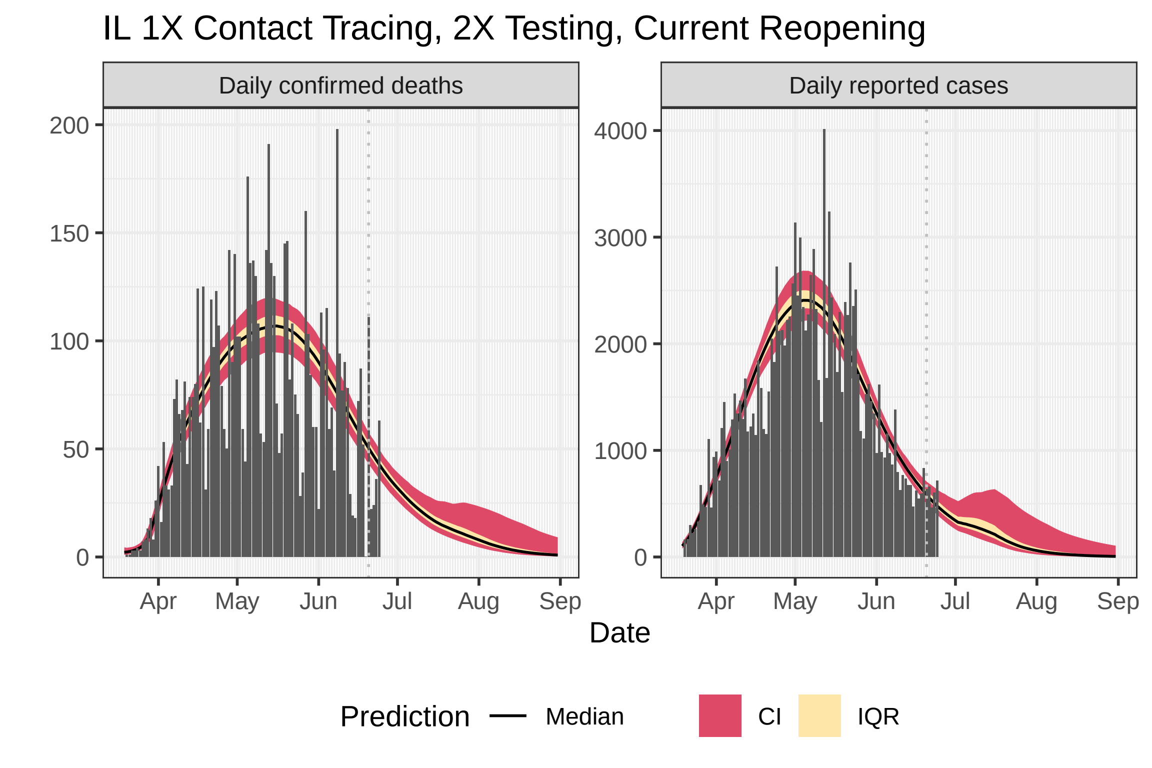
IL 1X Contact Tracing, 2X Testing, Current Reopening
Daily confirmed deaths
Daily reported cases
200
150
100
50
0
4000
3000
2000
1000
0
Apr
May
Jun
Jul
Aug
Sep
Date
Prediction
Median
CI
IQR

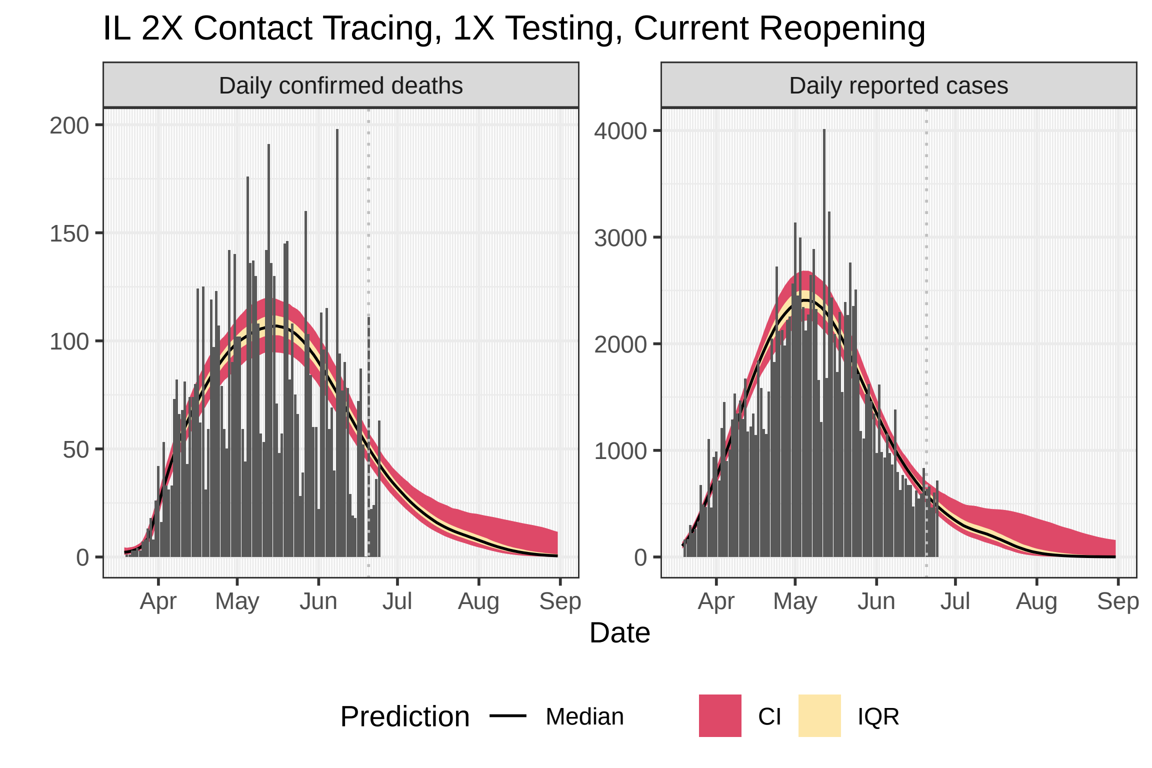
IL 2X Contact Tracing, 1X Testing, Current Reopening
Daily confirmed deaths
Daily reported cases
200
150
100
50
0
4000
3000
2000
1000
0
Apr
May
Jun
Jul
Aug
Sep
Date
Prediction
Median
CI
IQR

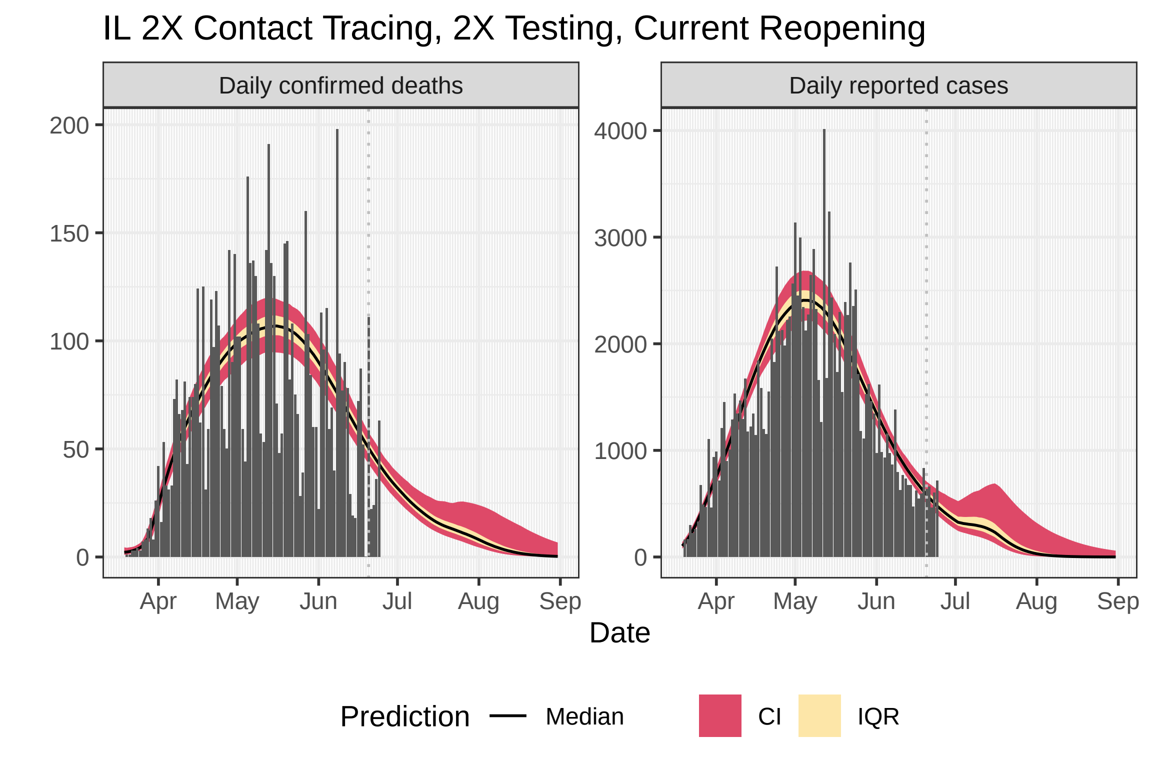
IL 2X Contact Tracing, 2X Testing, Current Reopening
Daily confirmed deaths
Daily reported cases
200
150
100
50
0
4000
3000
2000
1000
0
Apr
May
Jun
Jul
Aug
Sep
Date
Prediction
Median
CI
IQR

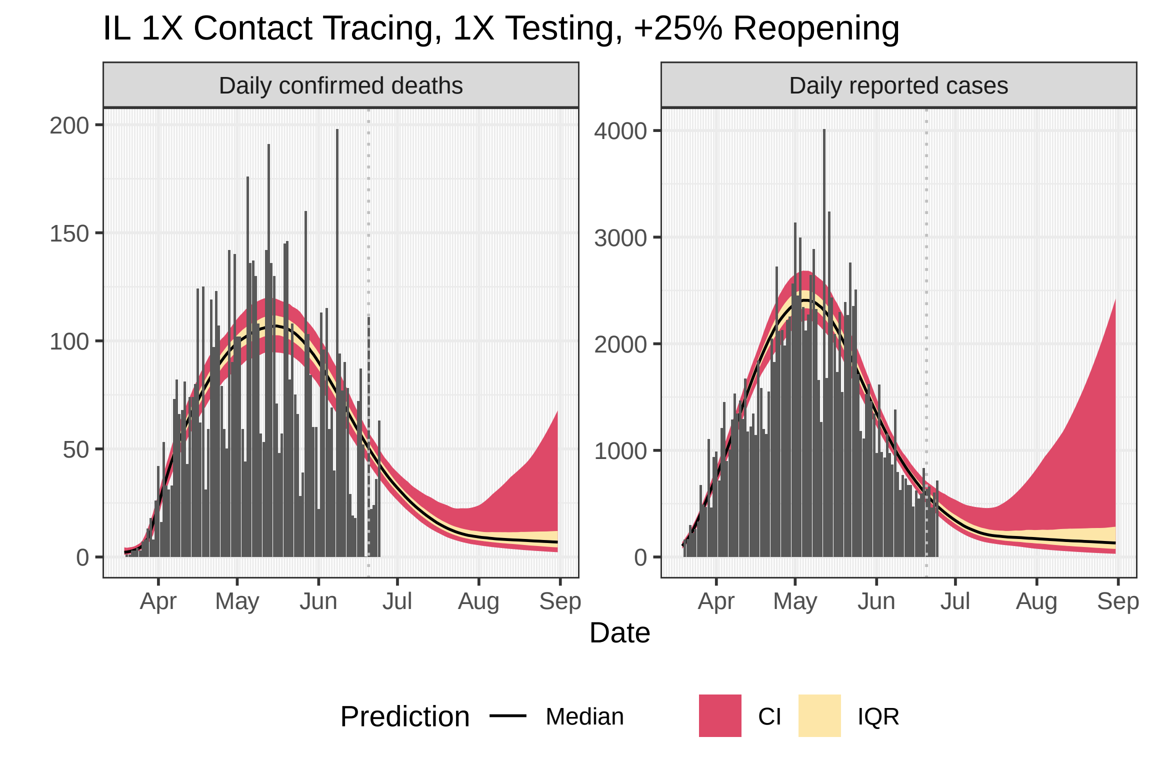

IL 1X Contact Tracing, 1X Testing, +25% Reopening
Daily confirmed deaths
Daily reported cases
200
150
100
50
0
4000
3000
2000
1000
0
Apr
May
Jun
Jul
Aug
Sep
Date
Prediction
Median
CI
IQR

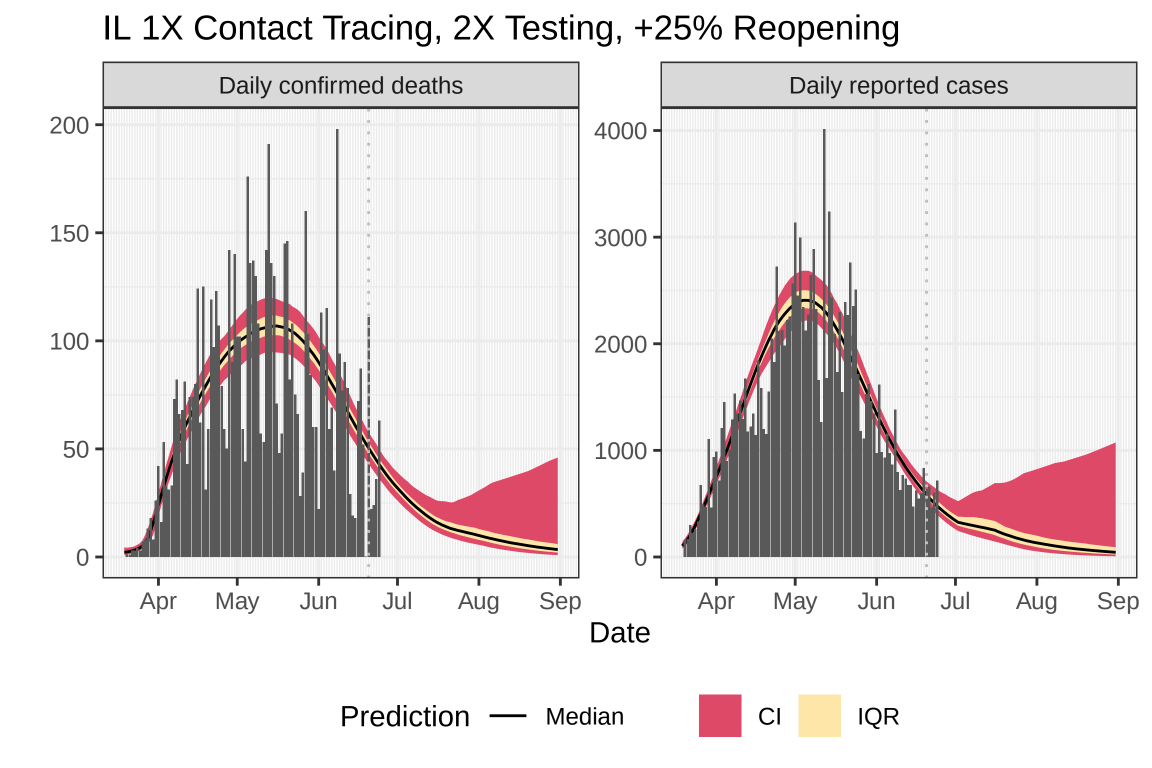

IL 1X Contact Tracing, 2X Testing, +25% Reopening
Daily confirmed deaths
Daily reported cases
200
150
100
50
0
4000
3000
2000
1000
0
Apr
May
Jun
Jul
Aug
Sep
Date
Prediction
Median
CI
IQR

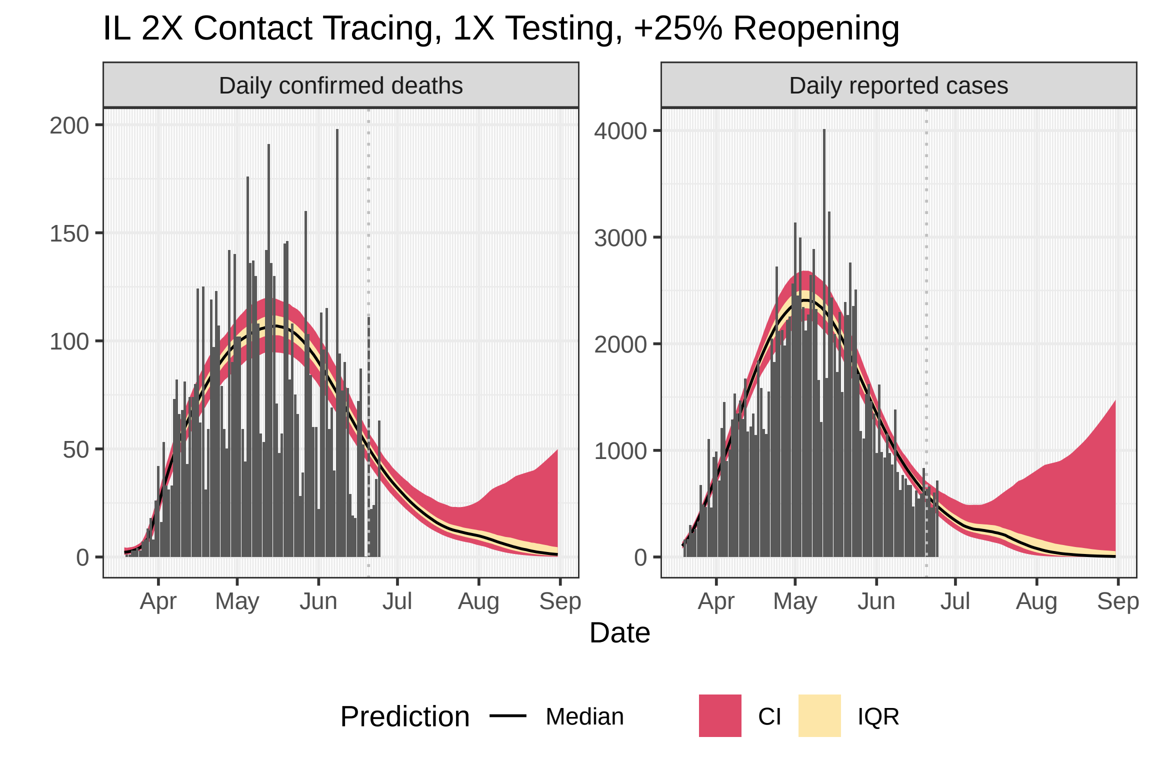
IL 2X Contact Tracing, 1X Testing, +25% Reopening
Daily confirmed deaths
Daily reported cases
200
150
100
50
0
4000
3000
2000
1000
0
Apr
May
Jun
Jul
Aug
Sep
Date
Prediction
Median
CI
IQR

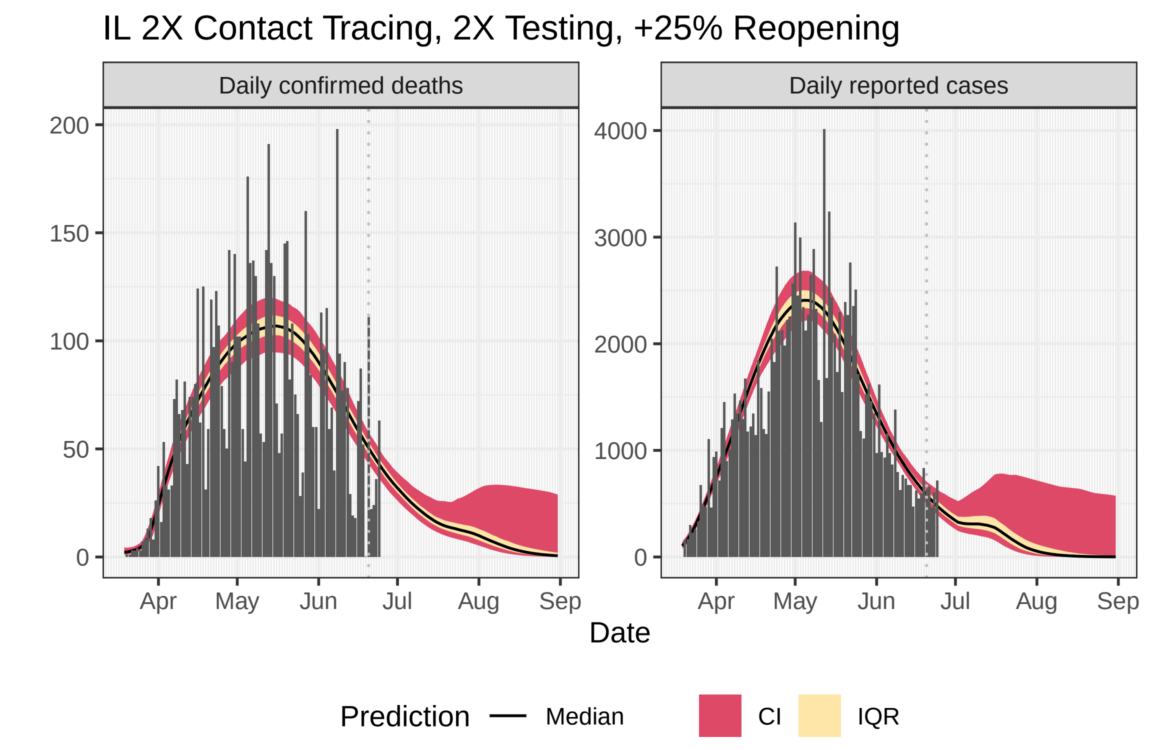

IL 2X Contact Tracing, 2X Testing, +25% Reopening
Daily confirmed deaths
Daily reported cases
200
150
100
50
0
4000
3000
2000
1000
0
Apr
May
Jun
Jul
Aug
Sep
Date
Prediction
Median
CI
IQR

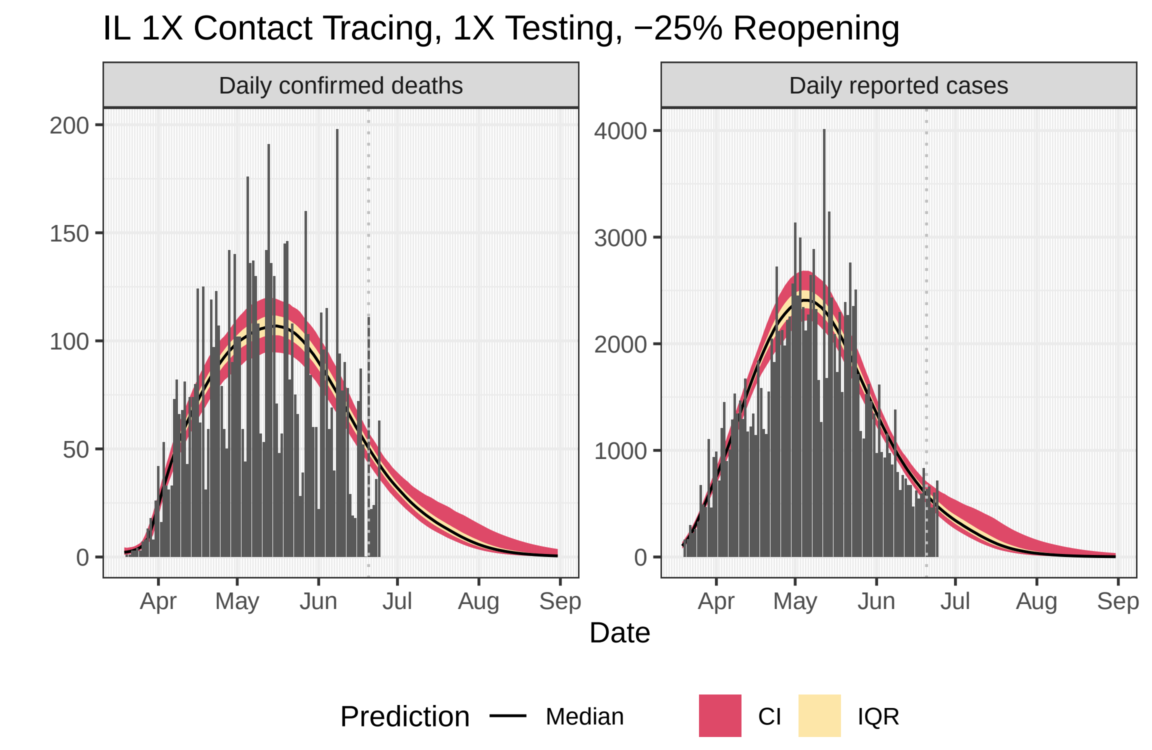
IL 1X Contact Tracing, 1X Testing, −25% Reopening
Daily confirmed deaths
Daily reported cases
200
150
100
50
0
4000
3000
2000
1000
0
Apr
May
Jun
Jul
Aug
Sep
Date
Prediction
Median
CI
IQR

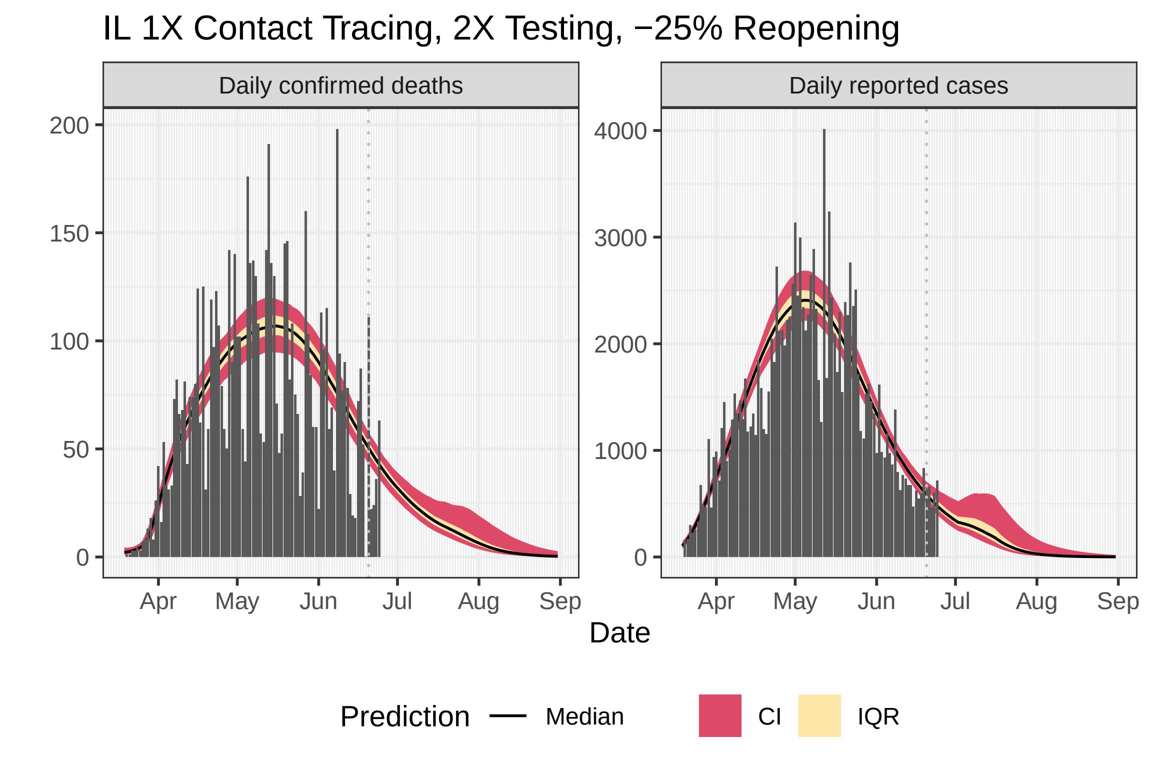

IL 1X Contact Tracing, 2X Testing, −25% Reopening
Daily confirmed deaths
Daily reported cases
200
150
100
50
0
4000
3000
2000
1000
0
Apr
May
Jun
Jul
Aug
Sep
Date
Prediction
Median
CI
IQR

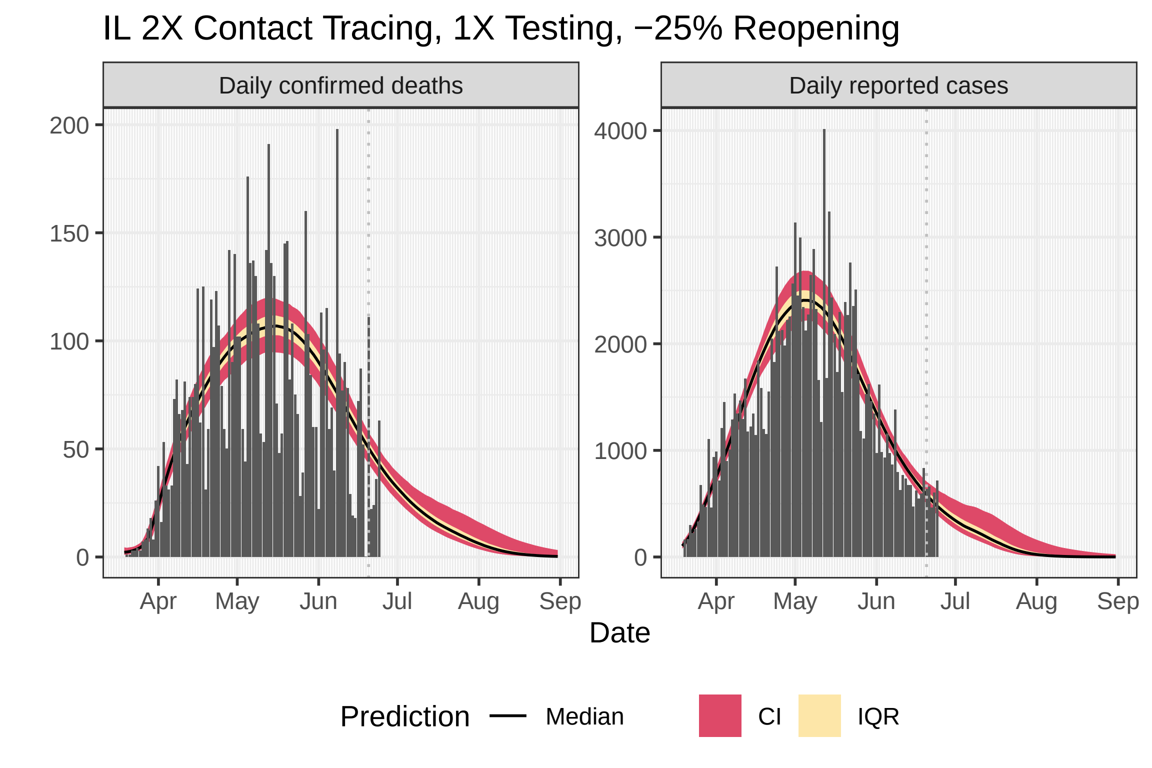

IL 2X Contact Tracing, 1X Testing, −25% Reopening
Daily confirmed deaths
Daily reported cases
200
150
100
50
0
4000
3000
2000
1000
0
Apr
May
Jun
Jul
Aug
Sep
Date
Prediction
Median
CI
IQR

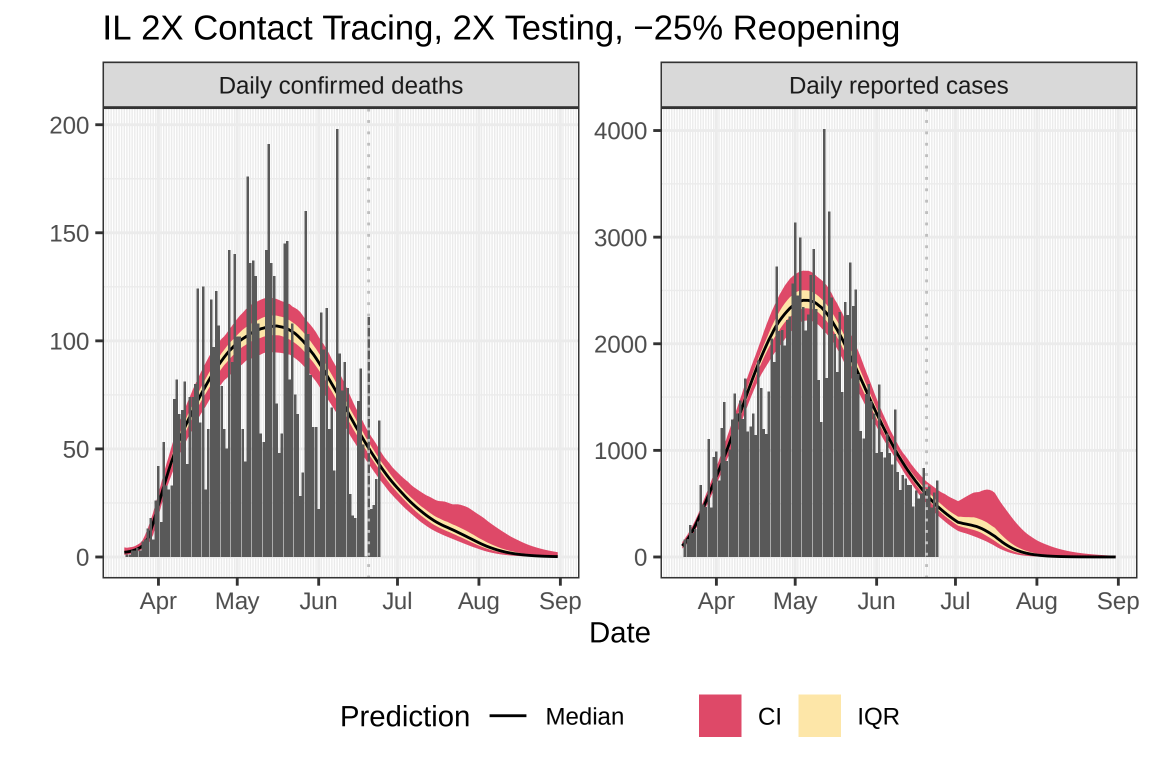
IL 2X Contact Tracing, 2X Testing, −25% Reopening
Daily confirmed deaths
Daily reported cases
200
150
100
50
0
4000
3000
2000
1000
0
Apr
May
Jun
Jul
Aug
Sep
Date
Prediction
Median
CI
IQR

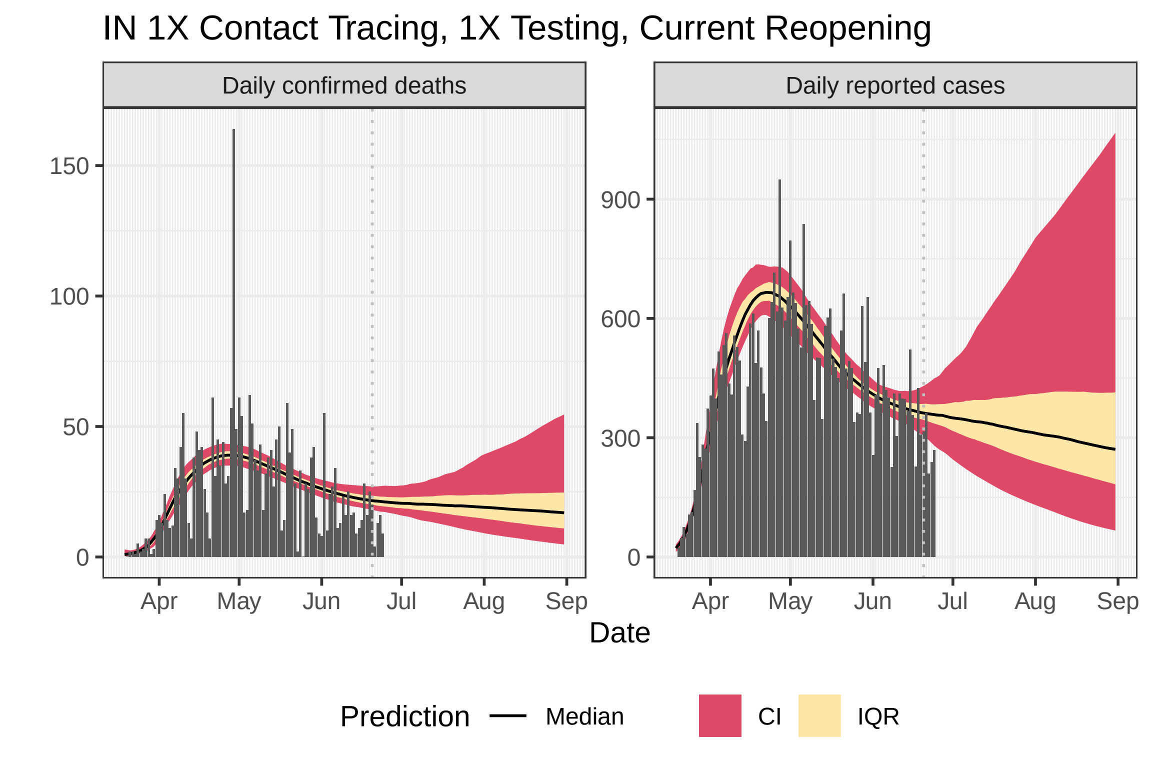

IN 1X Contact Tracing, 1X Testing, Current Reopening
Daily confirmed deaths
Daily reported cases
150
100
50
0
900
600
300
0
Apr
May
Jun
Jul
Aug
Sep
Date
Prediction
Median
CI
IQR

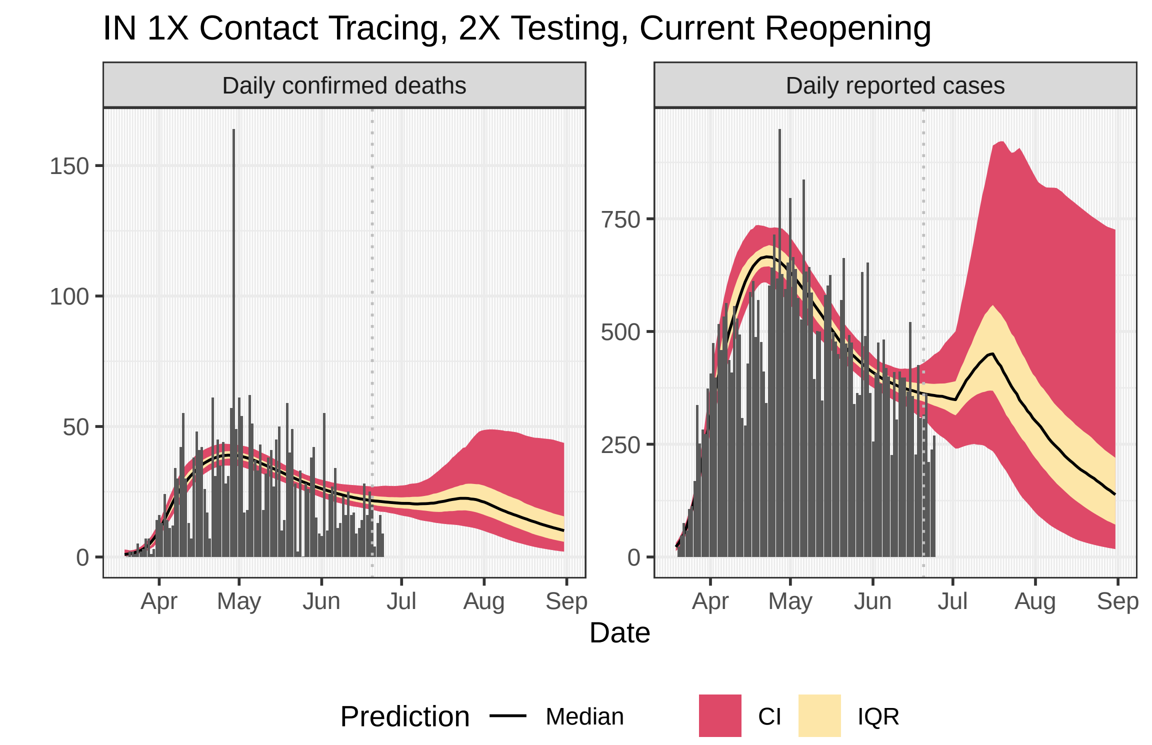

IN 1X Contact Tracing, 2X Testing, Current Reopening
Daily confirmed deaths
Daily reported cases
150
100
50
0
750
500
250
0
Apr
May
Jun
Jul
Aug
Sep
Date
Prediction
Median
CI
IQR

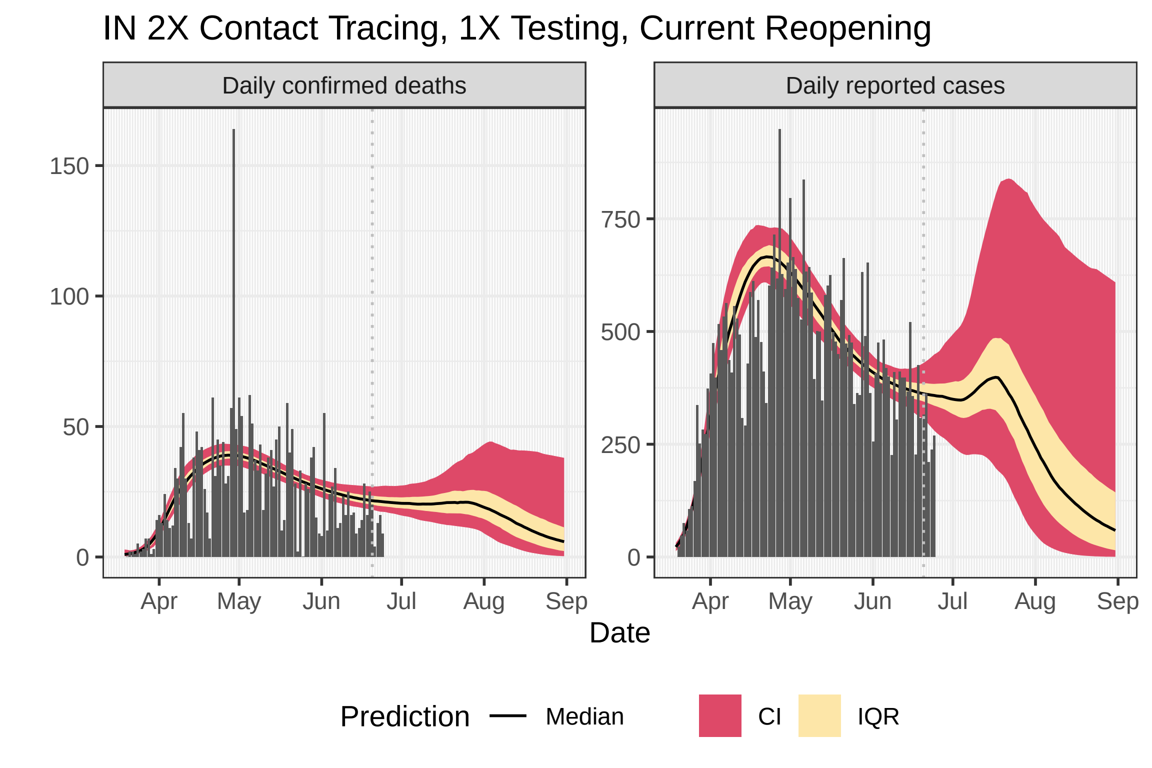

IN 2X Contact Tracing, 1X Testing, Current Reopening
Daily confirmed deaths
Daily reported cases
0
50
100
150
0
250
500
750
Apr
May
Jun
Jul
Aug
Sep
Date
Prediction
Median
CI
IQR

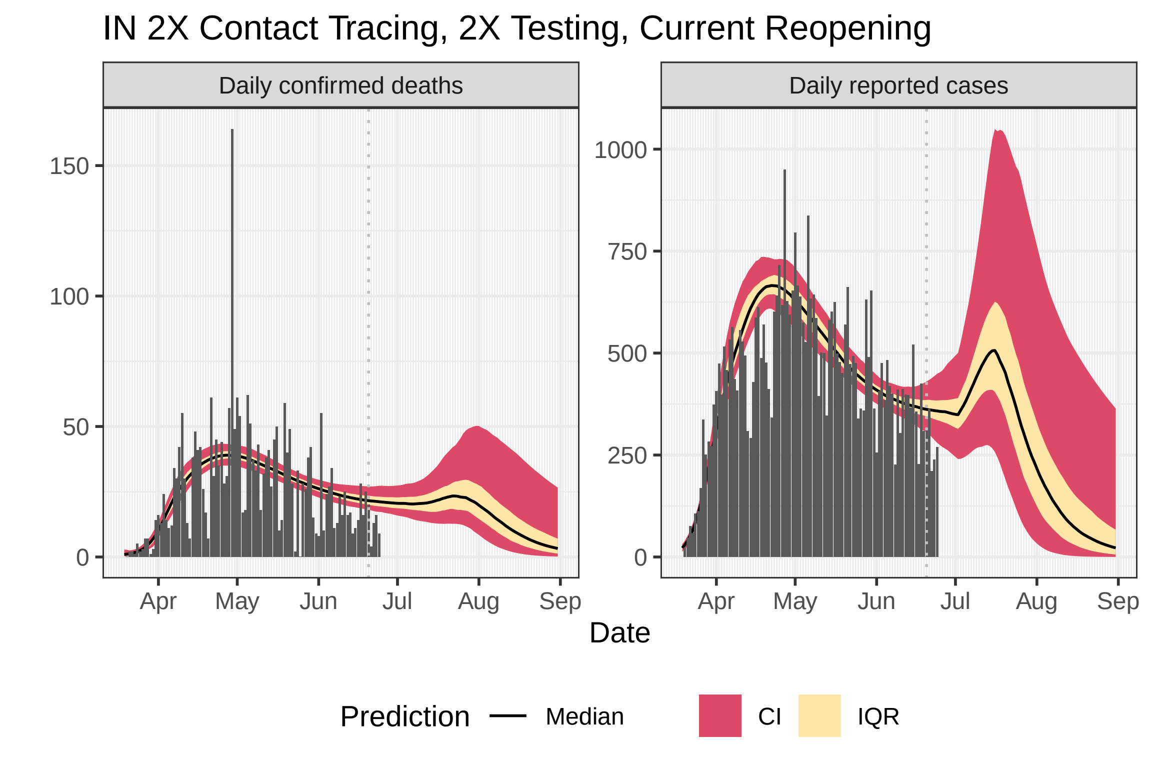

IN 2X Contact Tracing, 2X Testing, Current Reopening
Daily confirmed deaths
Daily reported cases
150
100
50
0
1000
750
500
250
0
Apr
May
Jun
Jul
Aug
Sep
Date
Prediction
Median
CI
IQR

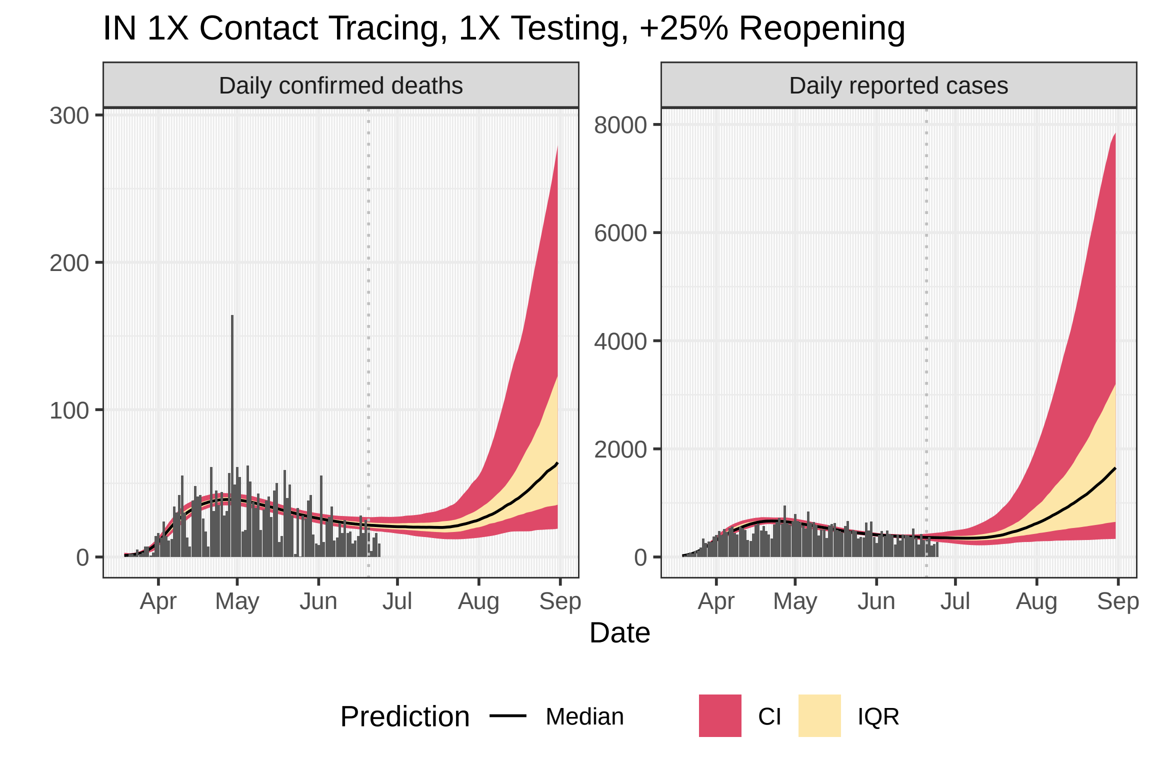

IN 1X Contact Tracing, 1X Testing, +25% Reopening
Daily confirmed deaths
Daily reported cases
300
200
100
0
8000
6000
4000
2000
0
Apr
May
Jun
Jul
Aug
Sep
Date
Prediction
Median
CI
IQR

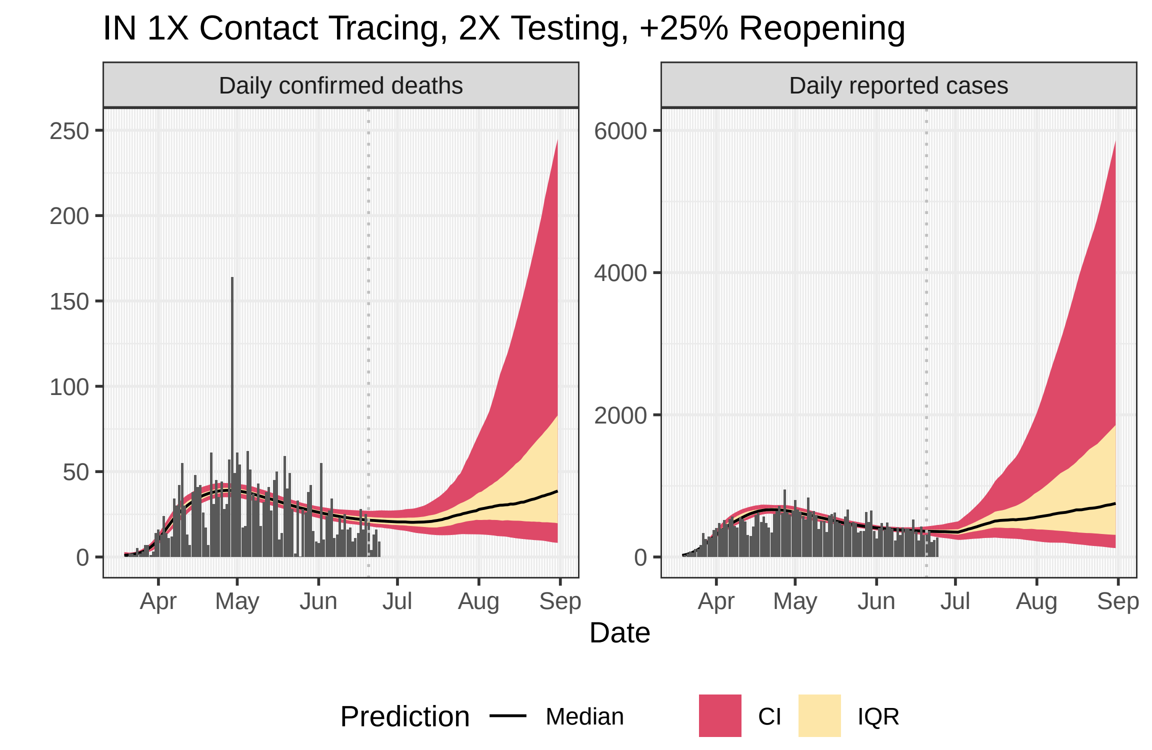
IN 1X Contact Tracing, 2X Testing, +25% Reopening
Daily confirmed deaths
Daily reported cases
0
50
100
150
200
250
0
2000
4000
6000
Apr
May
Jun
Jul
Aug
Sep
Date
Prediction
Median
CI
IQR

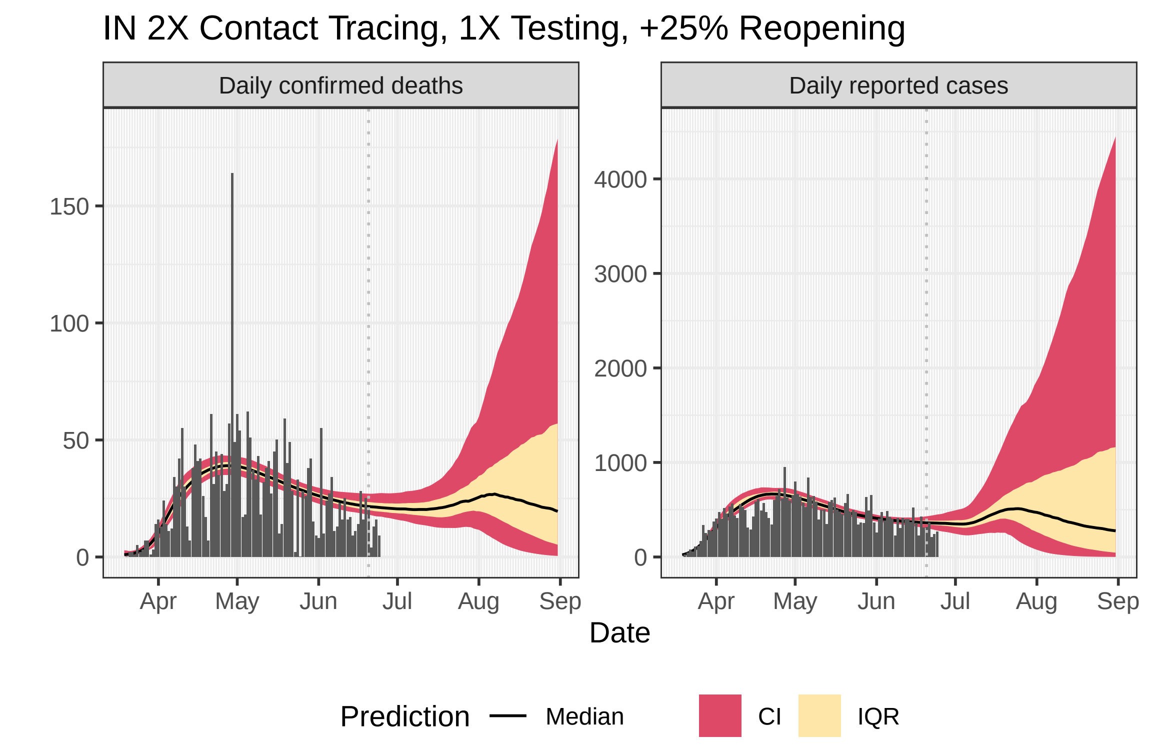

IN 2X Contact Tracing, 1X Testing, +25% Reopening
Daily confirmed deaths
Daily reported cases
0
50
100
150
0
1000
2000
3000
4000
Apr
May
Jun
Jul
Aug
Sep
Date
Prediction
Median
CI
IQR

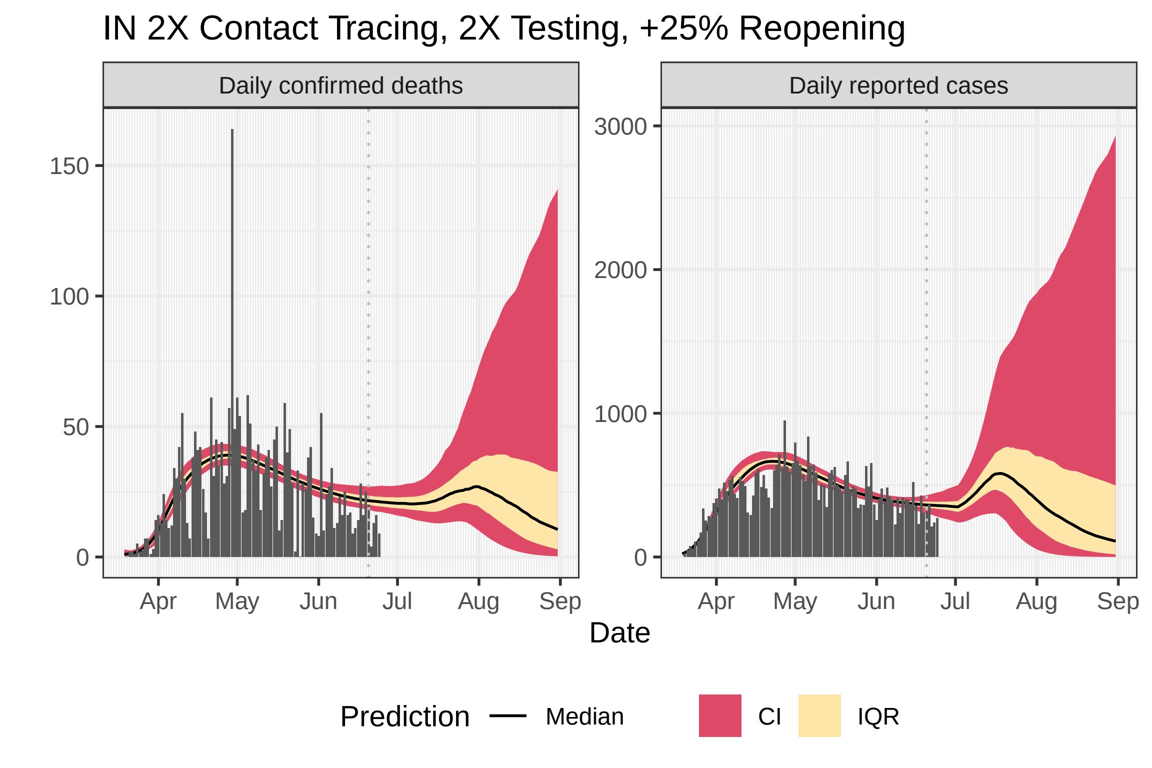
IN 2X Contact Tracing, 2X Testing, +25% Reopening
Daily confirmed deaths
Daily reported cases
150
100
50
0
3000
2000
1000
0
Apr
May
Jun
Jul
Aug
Sep
Date
Prediction
Median
CI
IQR

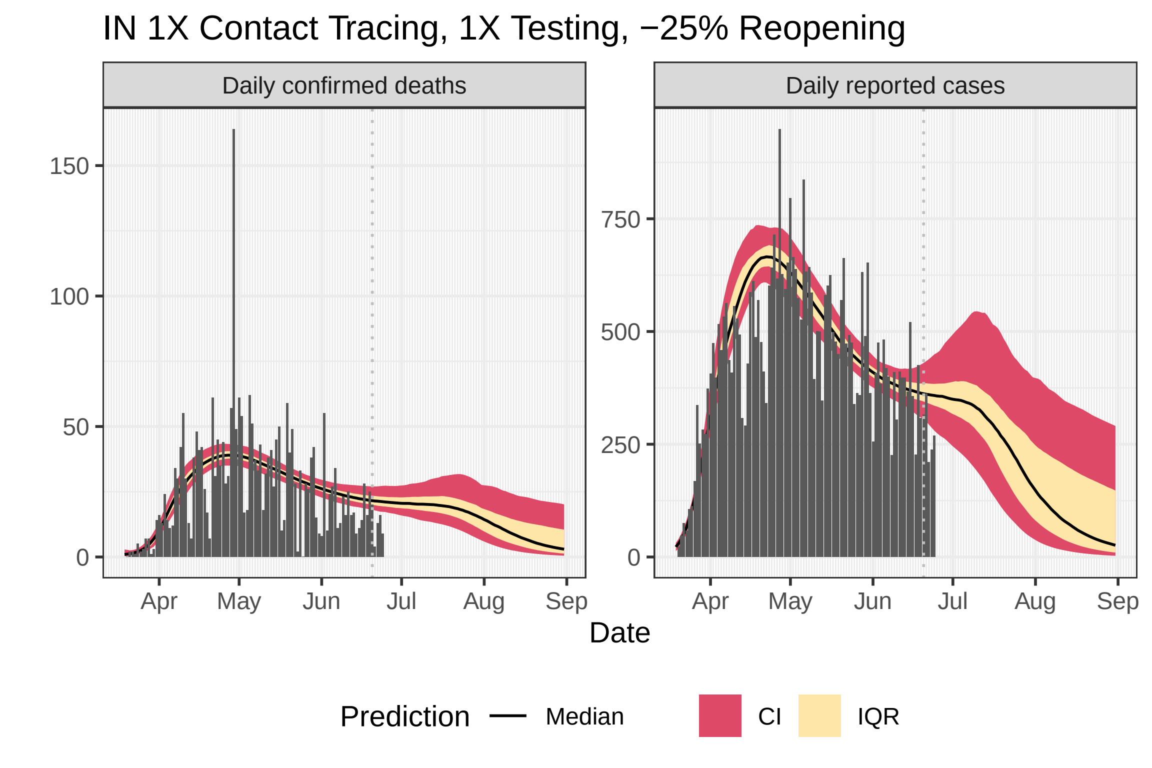
IN 1X Contact Tracing, 1X Testing, −25% Reopening
Daily confirmed deaths
Daily reported cases
150
100
50
0
750
500
250
0
Apr
May
Jun
Jul
Aug
Sep
Date
Prediction
Median
CI
IQR

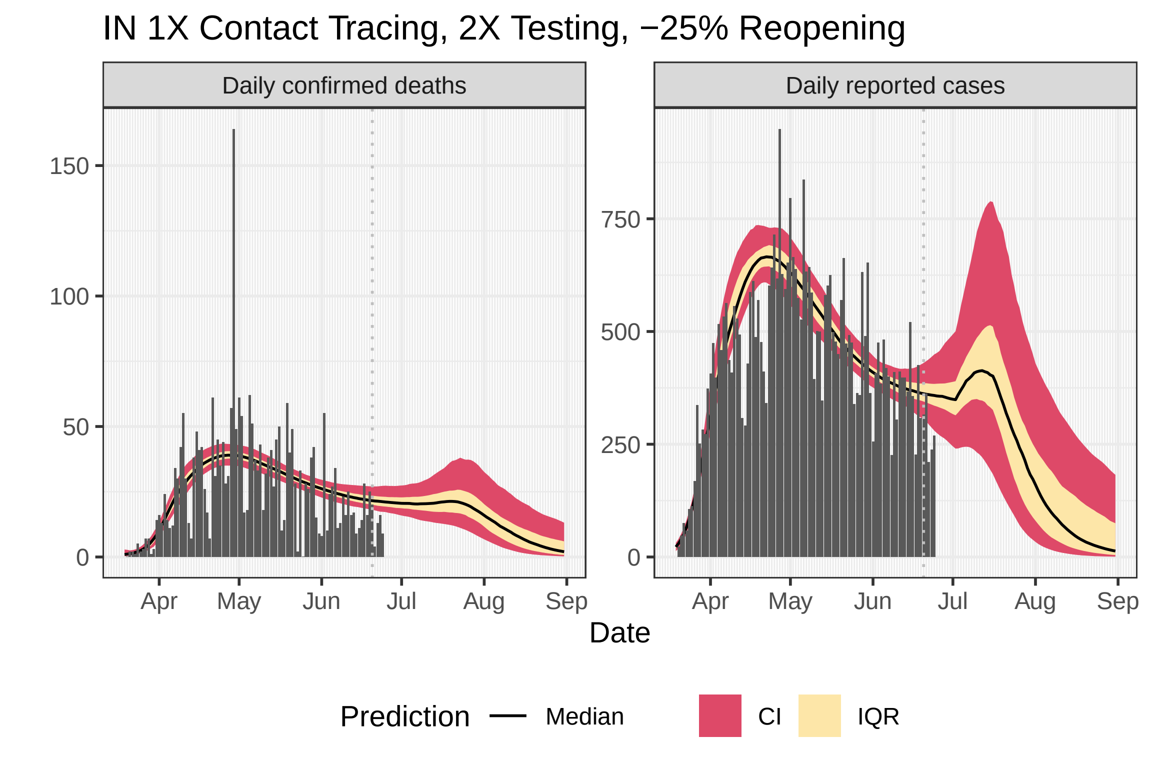
IN 1X Contact Tracing, 2X Testing, −25% Reopening
Daily confirmed deaths
Daily reported cases
150
100
50
0
750
500
250
0
Apr
May
Jun
Jul
Aug
Sep
Date
Prediction
Median
CI
IQR

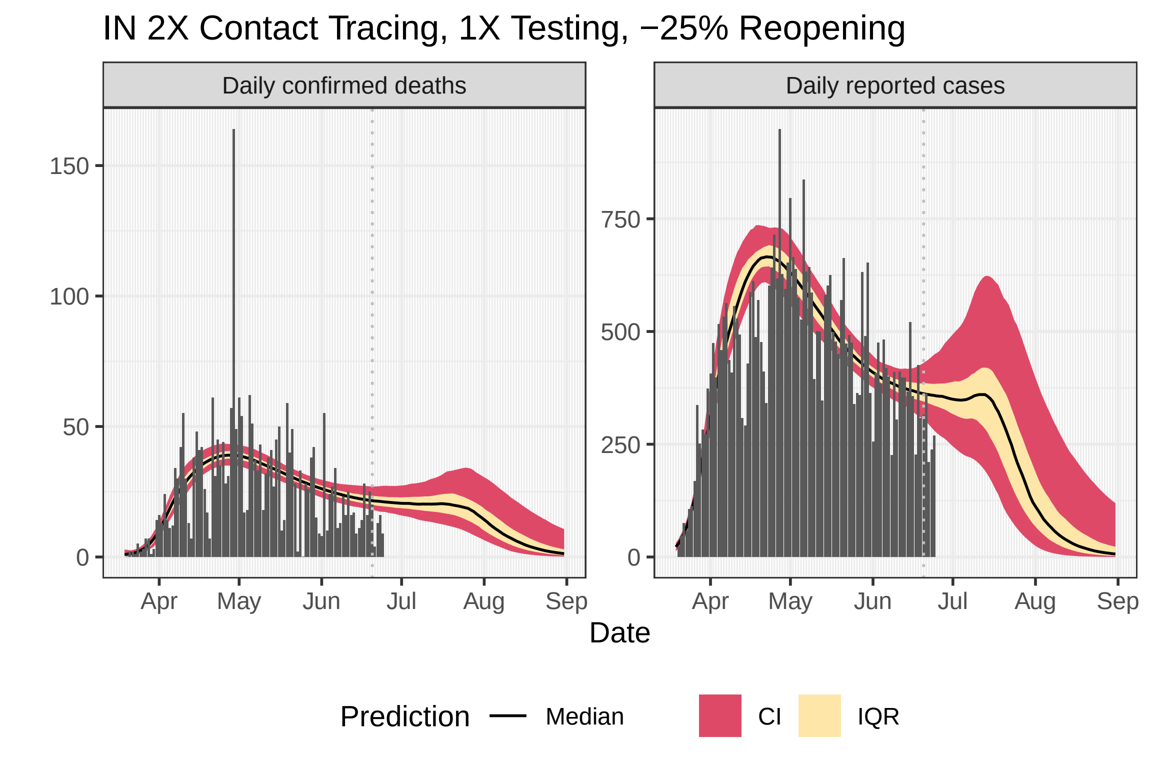
IN 2X Contact Tracing, 1X Testing, −25% Reopening
Daily confirmed deaths
Daily reported cases
150
100
50
0
750
500
250
0
Apr
May
Jun
Jul
Aug
Sep
Date
Prediction
Median
CI
IQR

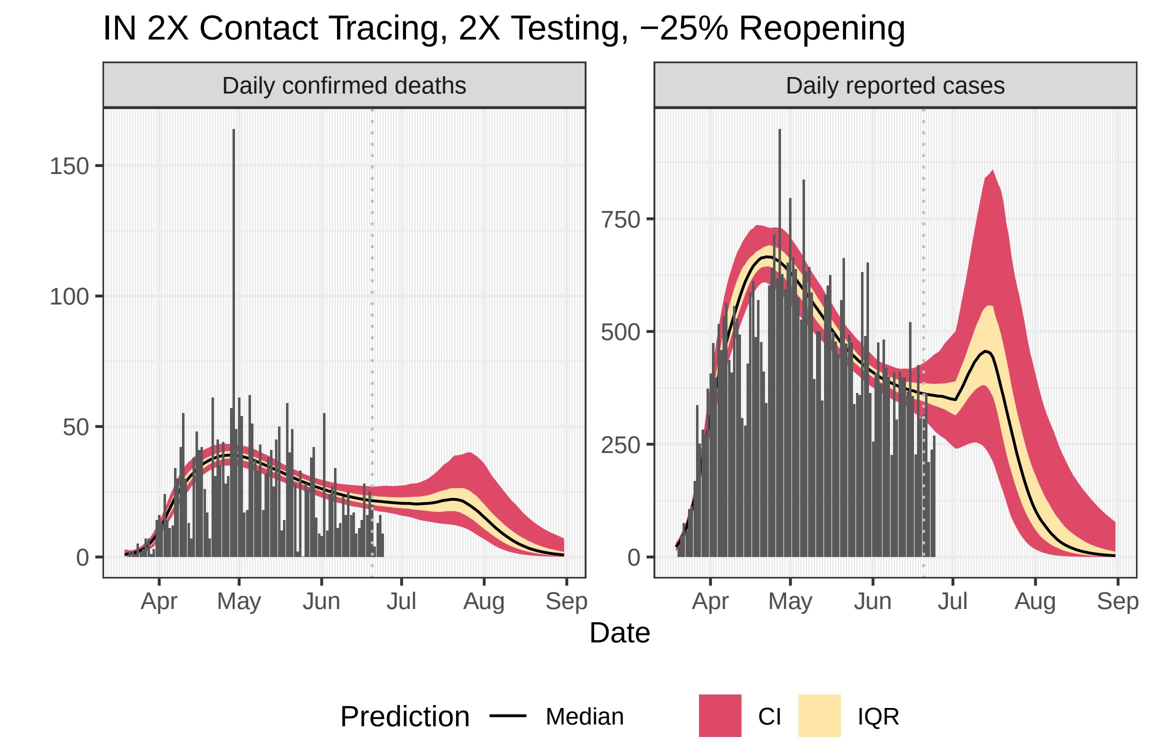
IN 2X Contact Tracing, 2X Testing, −25% Reopening
Daily confirmed deaths
Daily reported cases
150
100
50
0
750
500
250
0
Apr
May
Jun
Jul
Aug
Sep
Date
Prediction
Median
CI
IQR

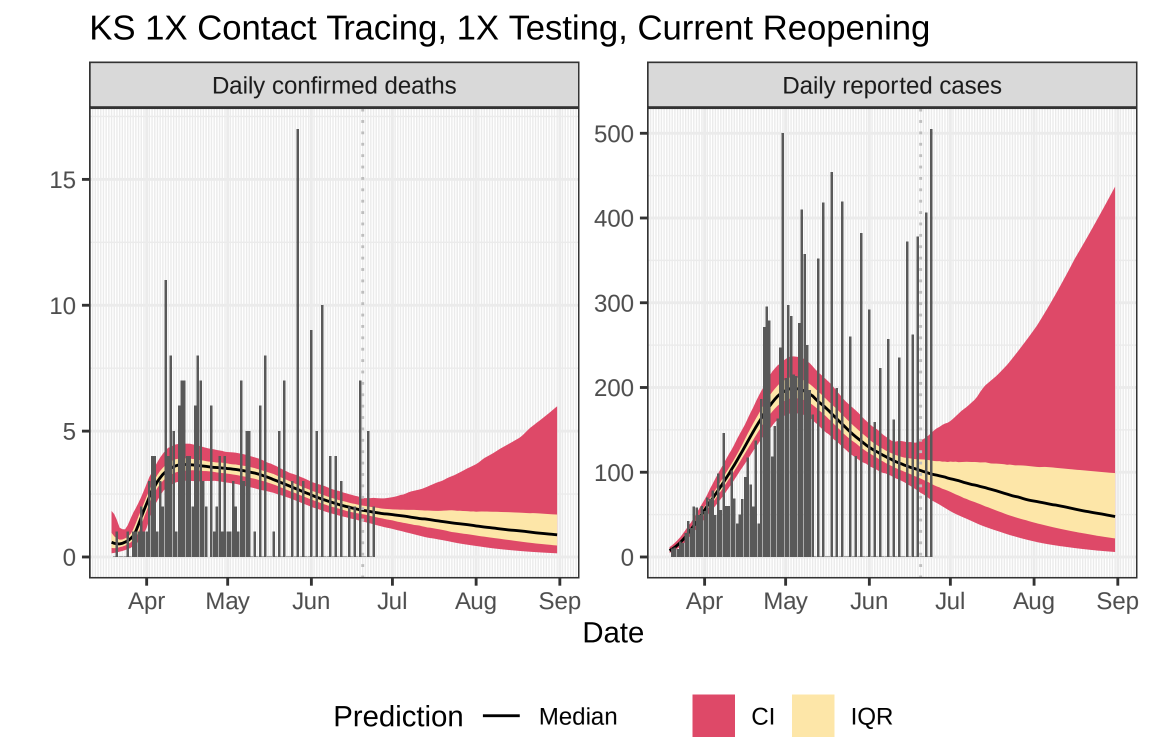

KS 1X Contact Tracing, 1X Testing, Current Reopening
Daily confirmed deaths
Daily reported cases
0
5
10
15
0
100
200
300
400
500
Apr
May
Jun
Jul
Aug
Sep
Date
Prediction
Median
CI
IQR

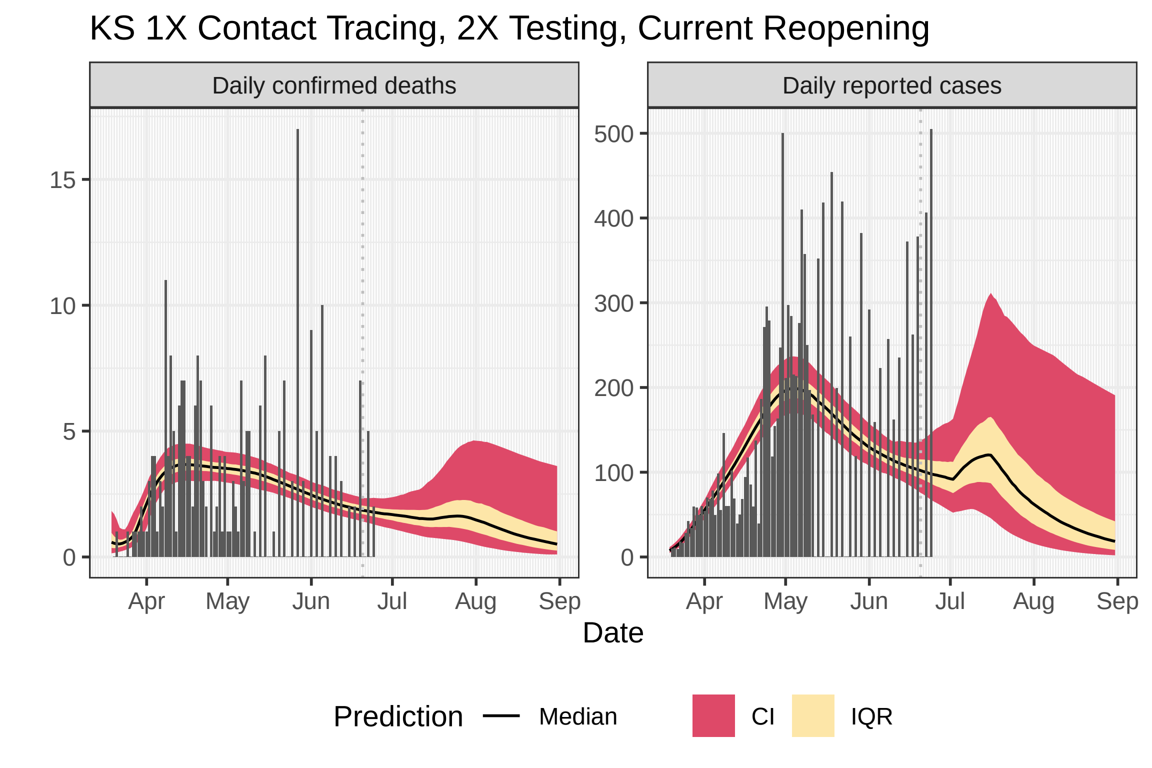

KS 1X Contact Tracing, 2X Testing, Current Reopening
Daily confirmed deaths
Daily reported cases
0
5
10
15
0
100
200
300
400
500
Apr
May
Jun
Jul
Aug
Sep
Date
Prediction
Median
CI
IQR

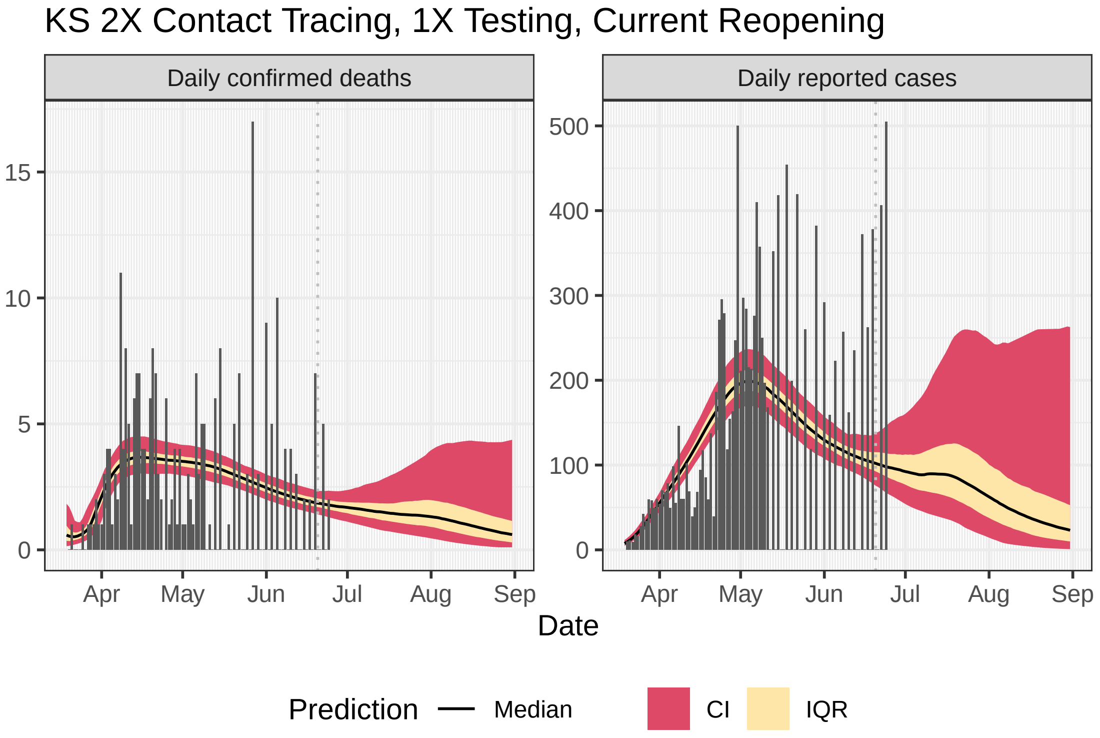

KS 2X Contact Tracing, 1X Testing, Current Reopening
Daily confirmed deaths
Daily reported cases
0
5
10
15
100
200
300
400
500
Apr
May
Jun
Jul
Aug
Sep
Date
Prediction
Median
CI
IQR

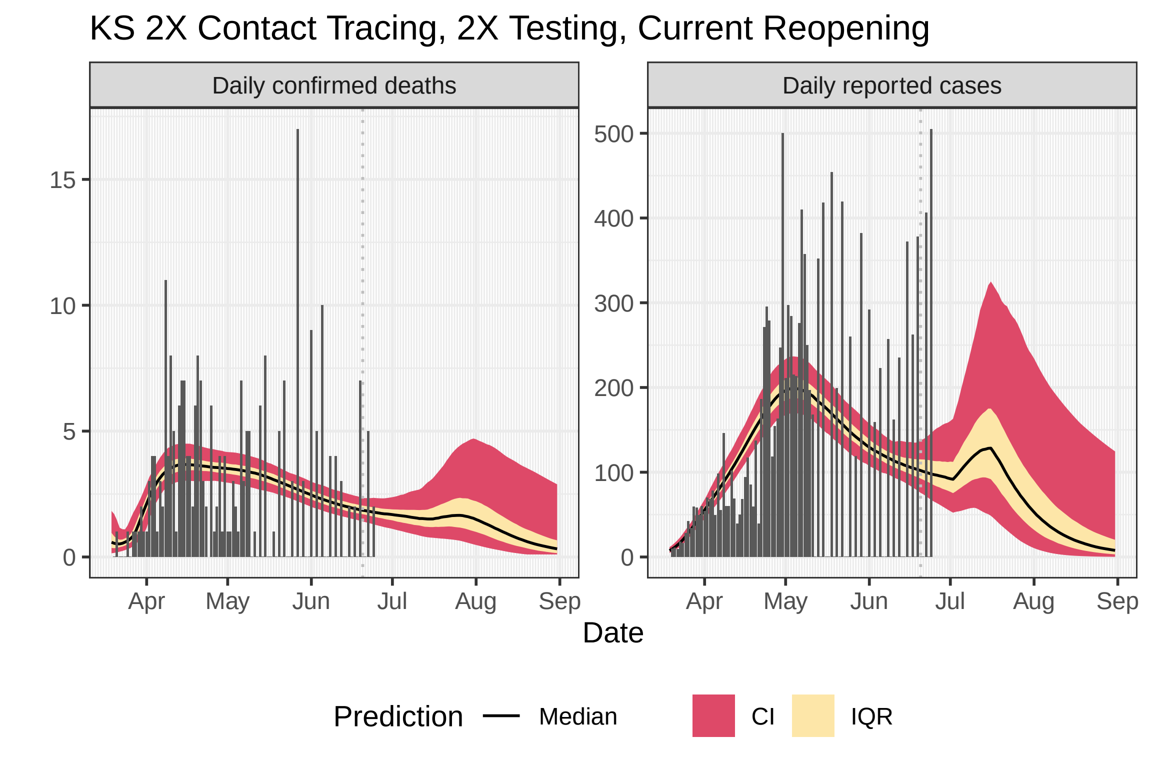

KS 2X Contact Tracing, 2X Testing, Current Reopening
Daily confirmed deaths
Daily reported cases
0
5
10
15
0
100
200
300
400
500
Apr
May
Jun
Jul
Aug
Sep
Date
Prediction
Median
CI
IQR

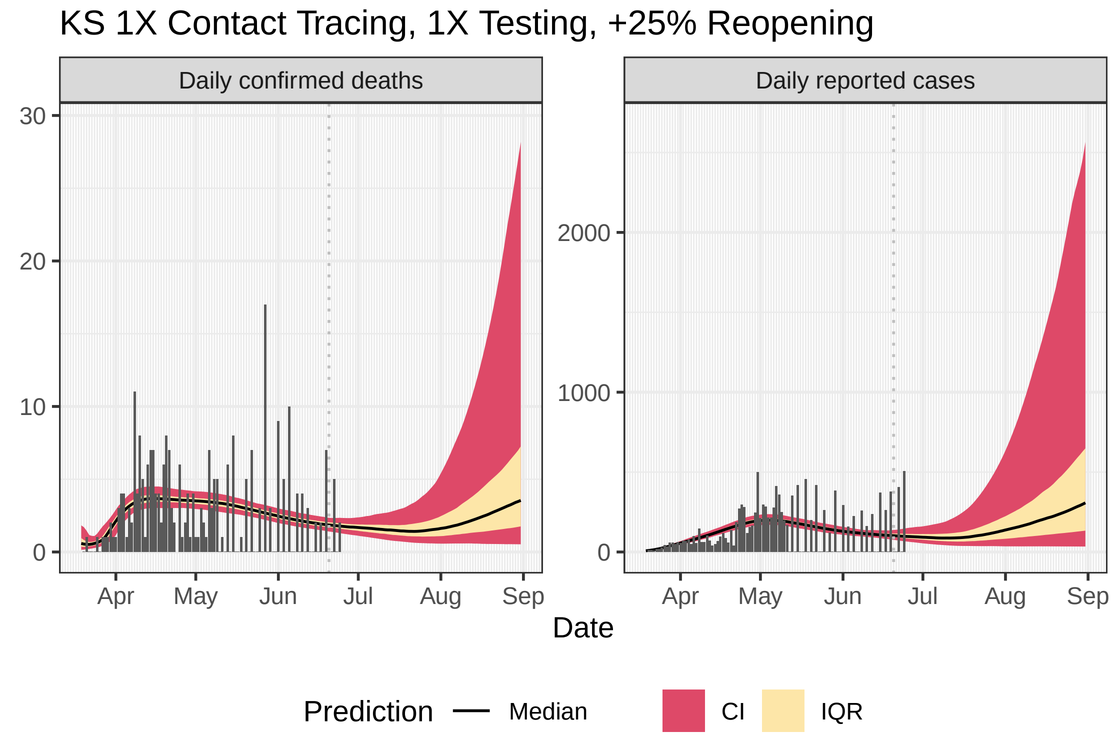

KS 1X Contact Tracing, 1X Testing, +25% Reopening
Daily confirmed deaths
Daily reported cases
30
20
10
0
2000
1000
0
Apr
May
Jun
Jul
Aug
Sep
Date
Prediction
Median
CI
IQR

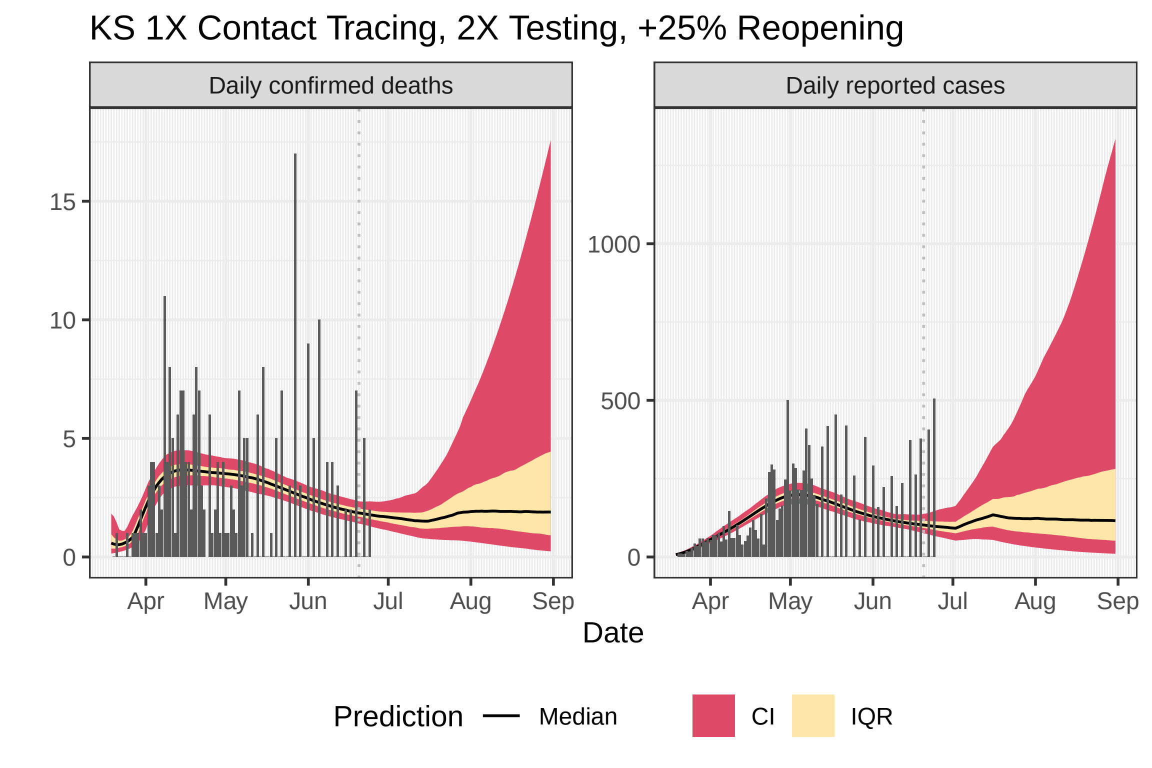

KS 1X Contact Tracing, 2X Testing, +25% Reopening
Daily confirmed deaths
Daily reported cases
0
5
10
15
0
500
1000
Apr
May
Jun
Jul
Aug
Sep
Date
Prediction
Median
CI
IQR

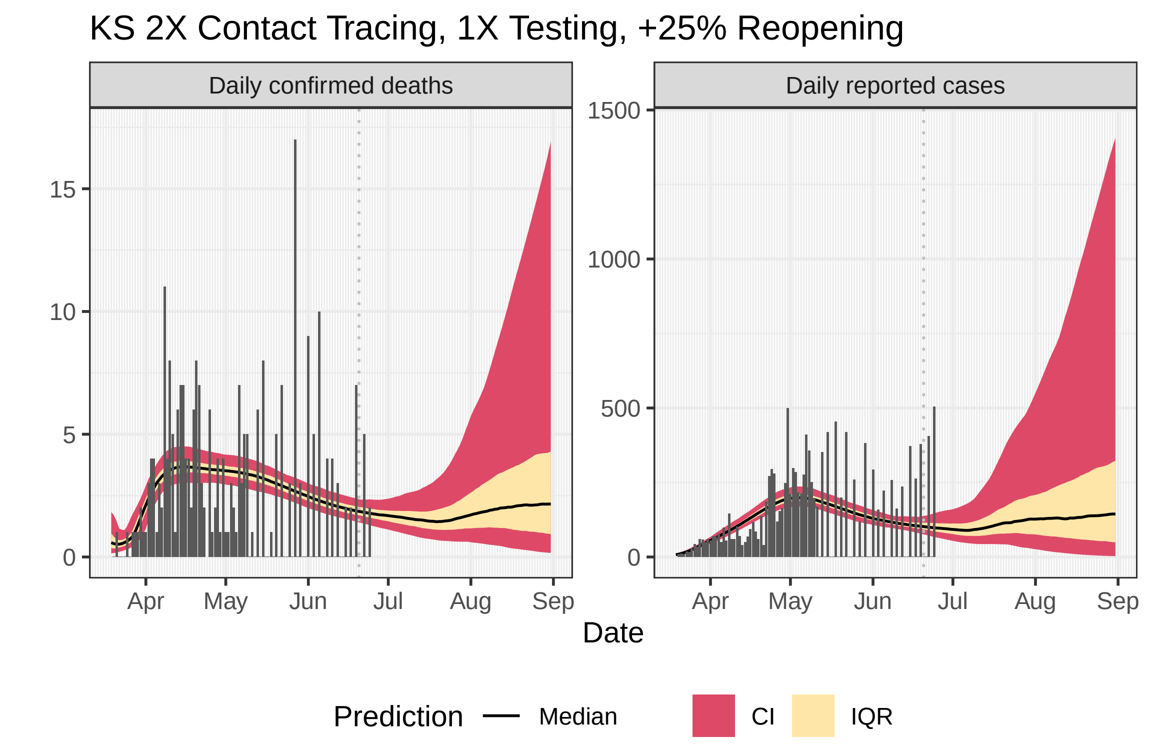

KS 2X Contact Tracing, 1X Testing, +25% Reopening
Daily confirmed deaths
Daily reported cases
0
5
10
15
0
500
1000
1500
Apr
May
Jun
Jul
Aug
Sep
Date
Prediction
Median
CI
IQR

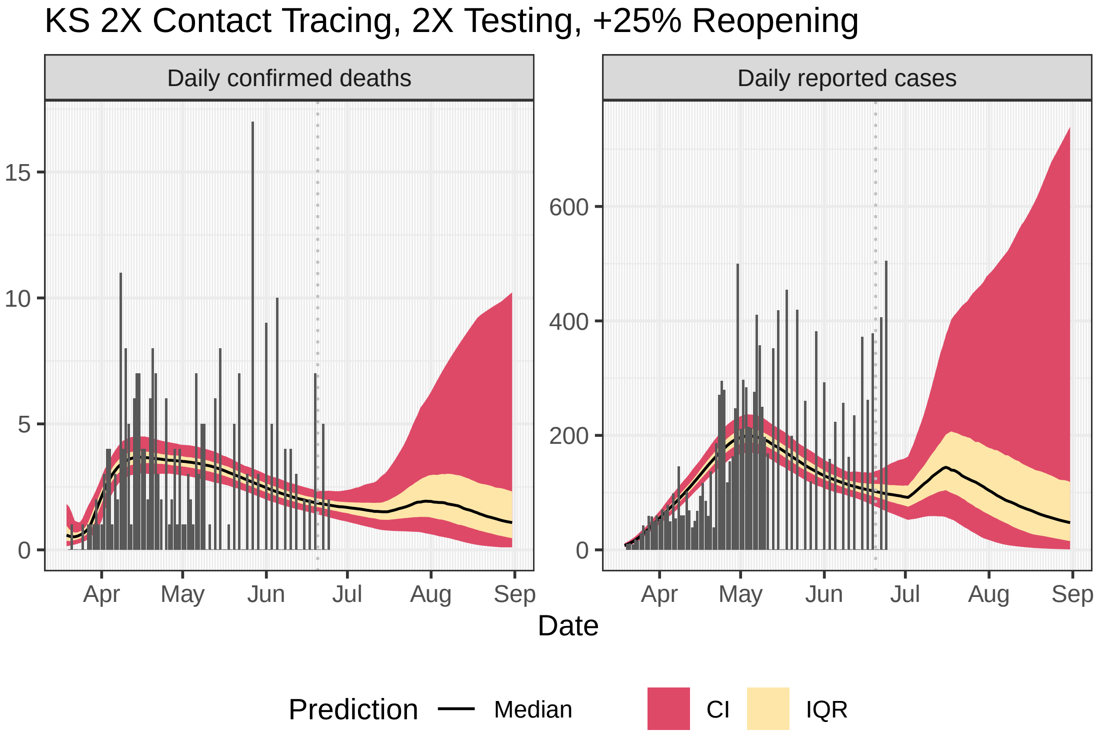

KS 2X Contact Tracing, 2X Testing, +25% Reopening
Daily confirmed deaths
Daily reported cases
15
10
5
0
600
400
200
0
Apr
May
Jun
Jul
Aug
Sep
Date
Prediction
Median
CI
IQR

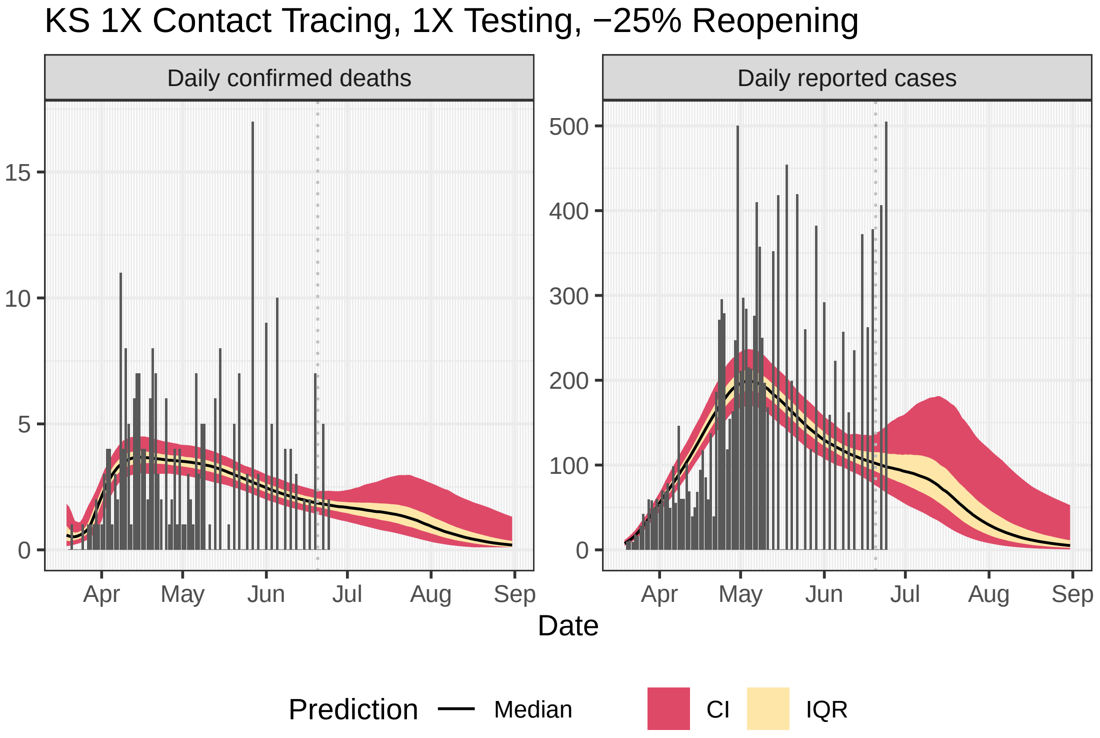

KS 1X Contact Tracing, 1X Testing, −25% Reopening
Daily confirmed deaths
Daily reported cases
0
5
10
15
0
100
200
300
400
500
Apr
May
Jun
Jul
Aug
Sep
Date
Prediction
Median
CI
IQR

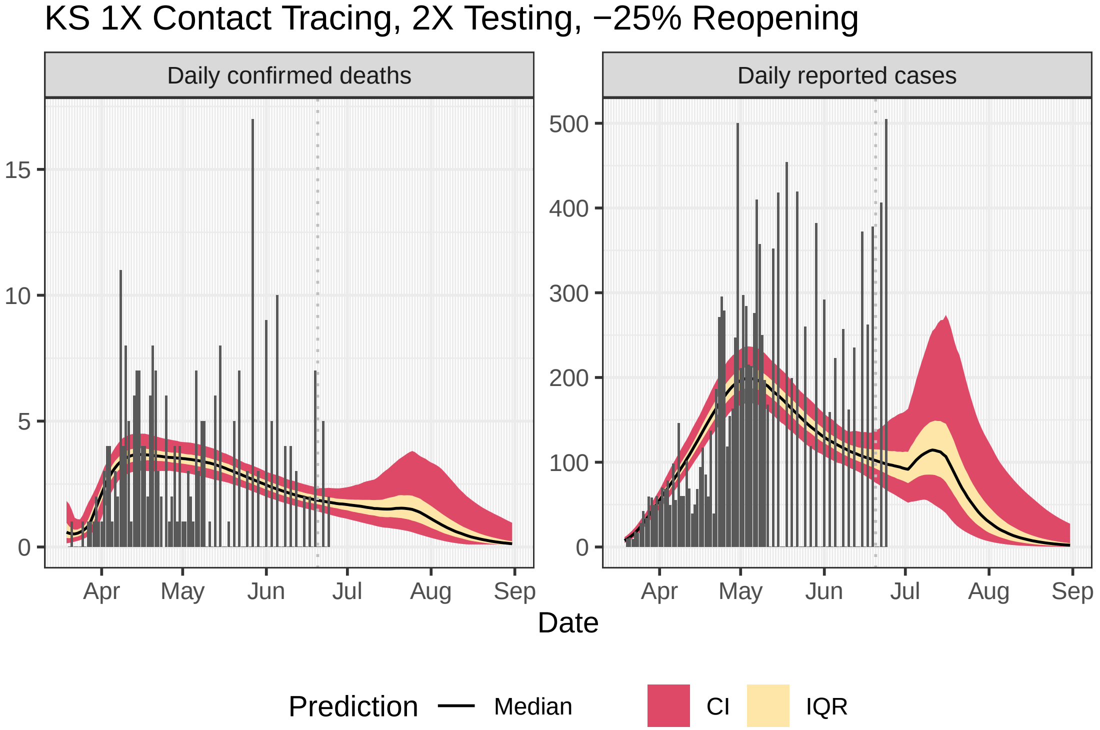
KS 1X Contact Tracing, 2X Testing, −25% Reopening
Daily confirmed deaths
Daily reported cases
0
5
10
15
0
100
200
300
400
500
Apr
May
Jun
Jul
Aug
Sep
Date
Prediction
Median
CI
IQR

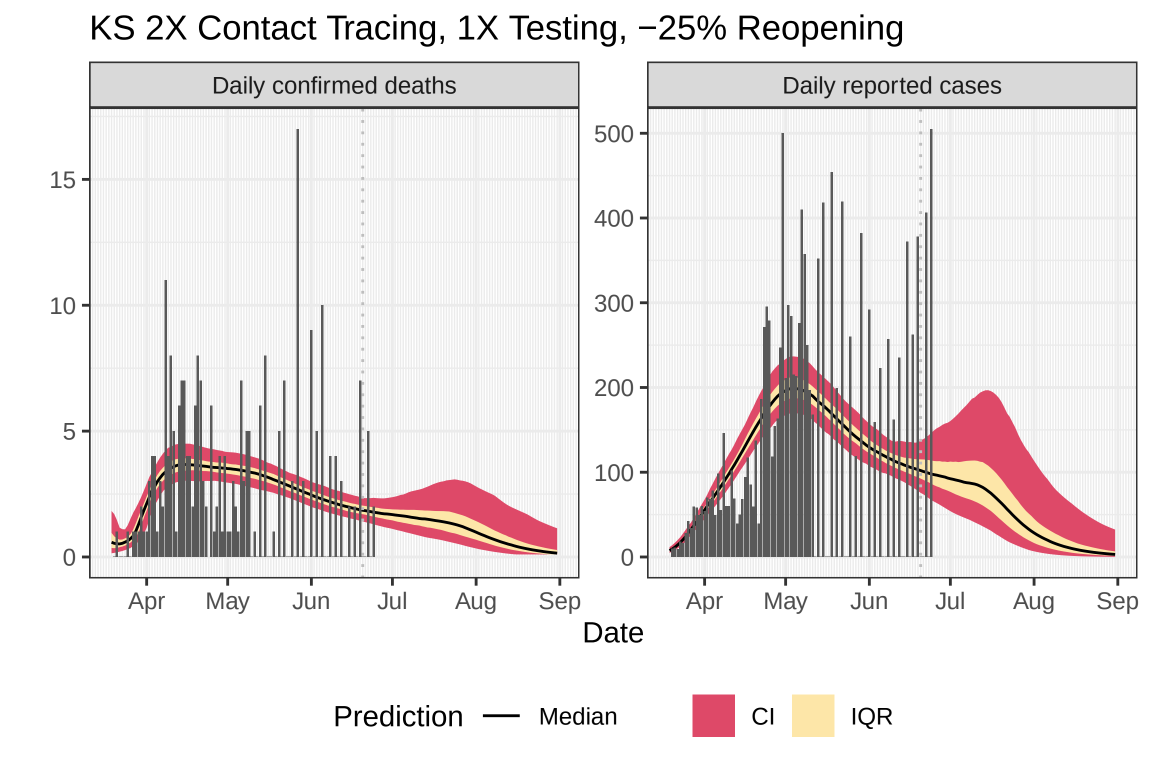
KS 2X Contact Tracing, 1X Testing, −25% Reopening
Daily confirmed deaths
Daily reported cases
15
10
5
0
500
400
300
200
100
0
Apr
May
Jun
Jul
Aug
Sep
Date
Prediction
Median
CI
IQR

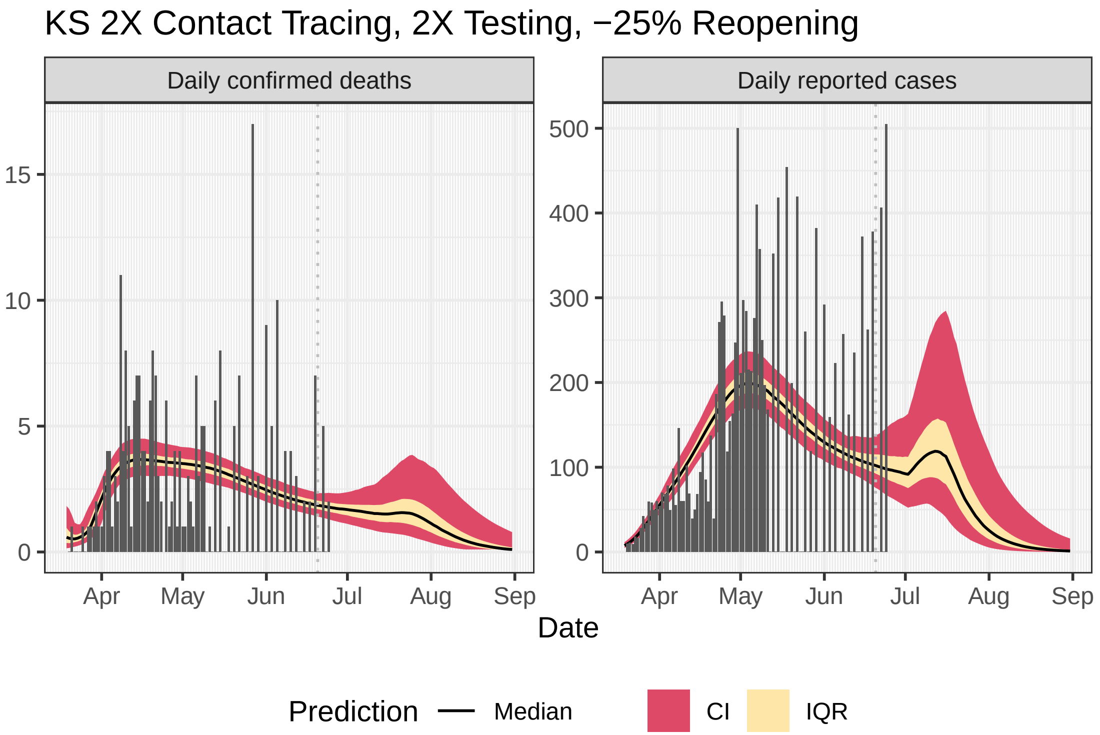
KS 2X Contact Tracing, 2X Testing, −25% Reopening
Daily confirmed deaths
Daily reported cases
15
10
5
0
500
400
300
200
100
0
Apr
May
Jun
Jul
Aug
Sep
Date
Prediction
Median
CI
IQR

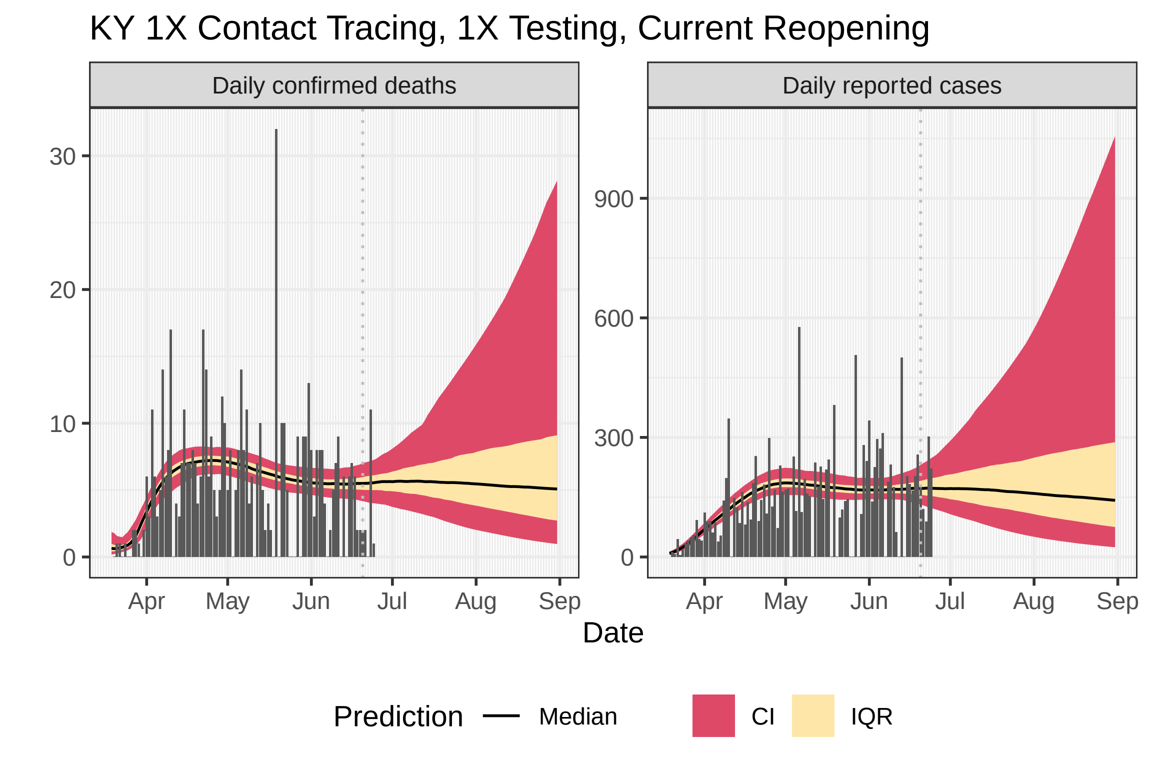
KY 1X Contact Tracing, 1X Testing, Current Reopening
Daily confirmed deaths
Daily reported cases
30
20
10
0
900
600
300
0
Apr
May
Jun
Jul
Aug
Sep
Date
Prediction
Median
CI
IQR

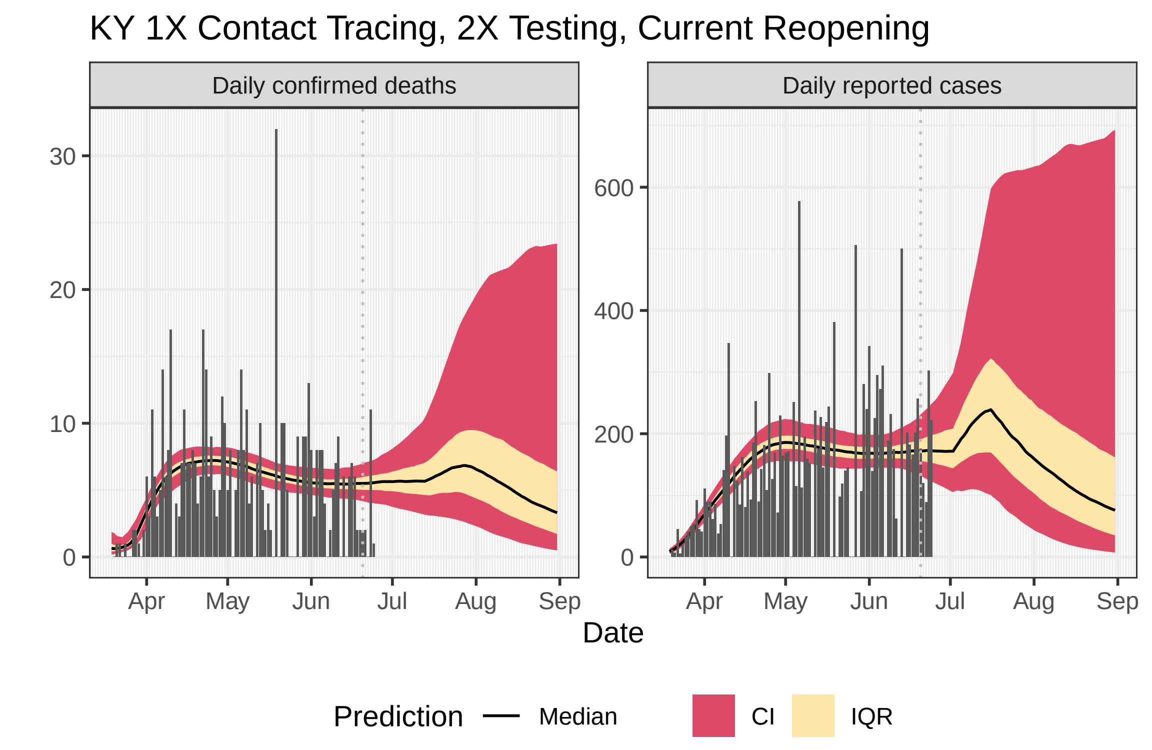

KY 1X Contact Tracing, 2X Testing, Current Reopening
Daily confirmed deaths
Daily reported cases
30
20
10
0
600
400
200
0
Apr
May
Jun
Jul
Aug
Sep
Date
Prediction
Median
CI
IQR

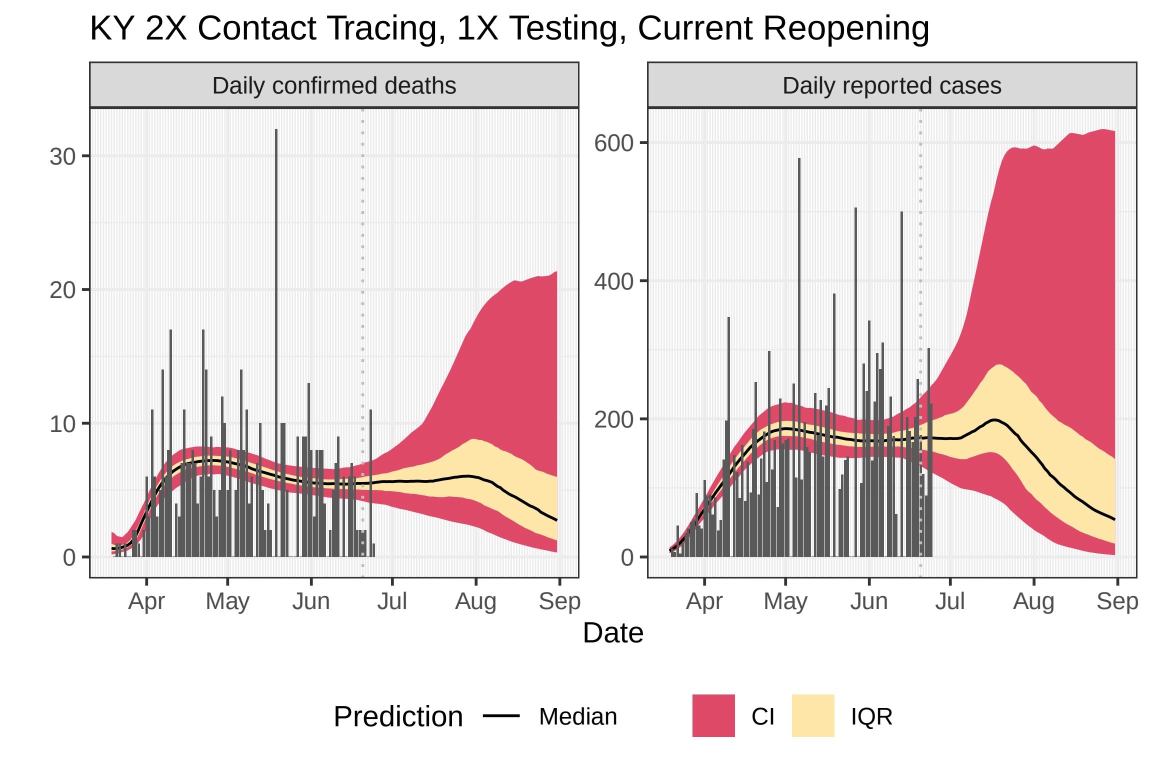
KY 2X Contact Tracing, 1X Testing, Current Reopening
Daily confirmed deaths
Daily reported cases
30
20
10
0
600
400
200
0
Apr
May
Jun
Jul
Aug
Sep
Date
Prediction
Median
CI
IQR

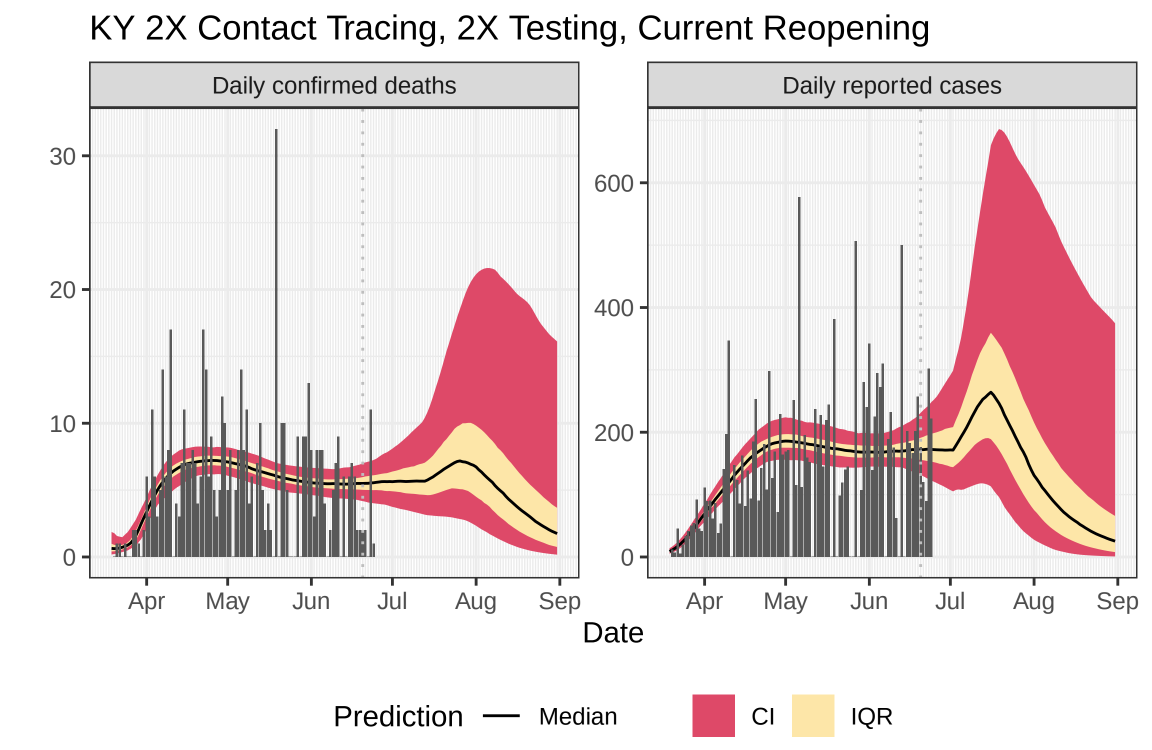

KY 2X Contact Tracing, 2X Testing, Current Reopening
Daily confirmed deaths
Daily reported cases
30
20
10
0
600
400
200
0
Apr
May
Jun
Jul
Aug
Sep
Date
Prediction
Median
CI
IQR

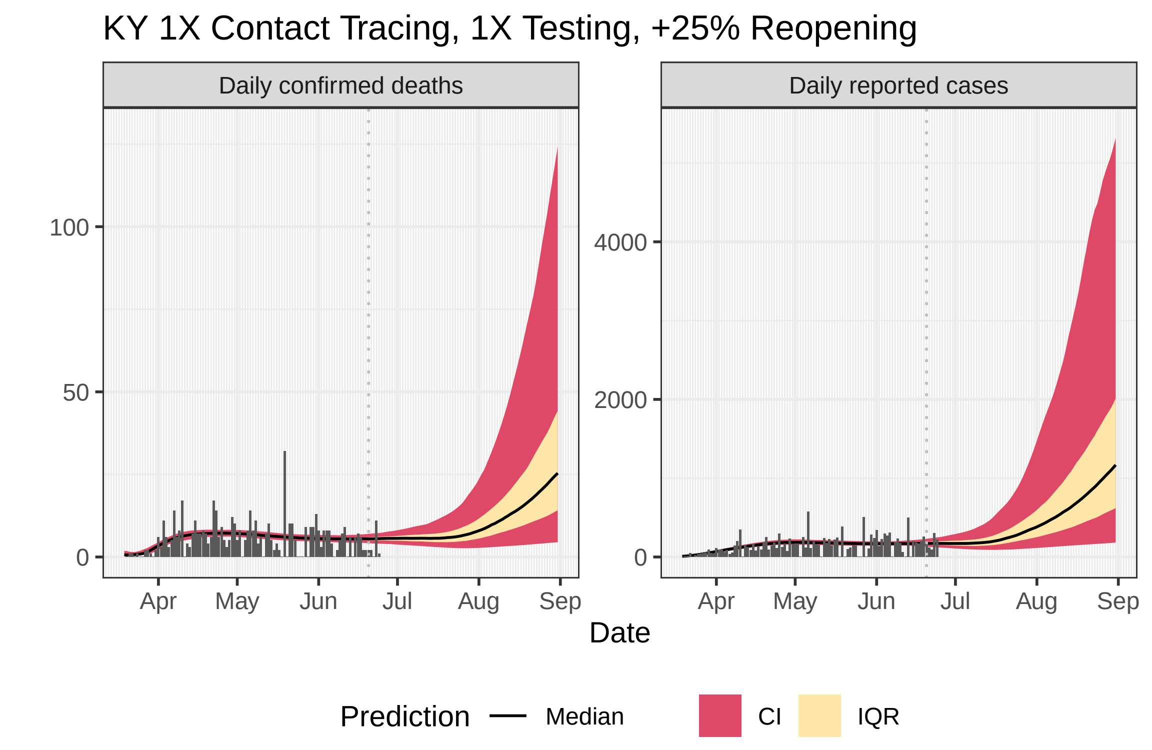

KY 1X Contact Tracing, 1X Testing, +25% Reopening
Daily confirmed deaths
Daily reported cases
100
50
0
4000
2000
0
Apr
May
Jun
Jul
Aug
Sep
Date
Prediction
Median
CI
IQR

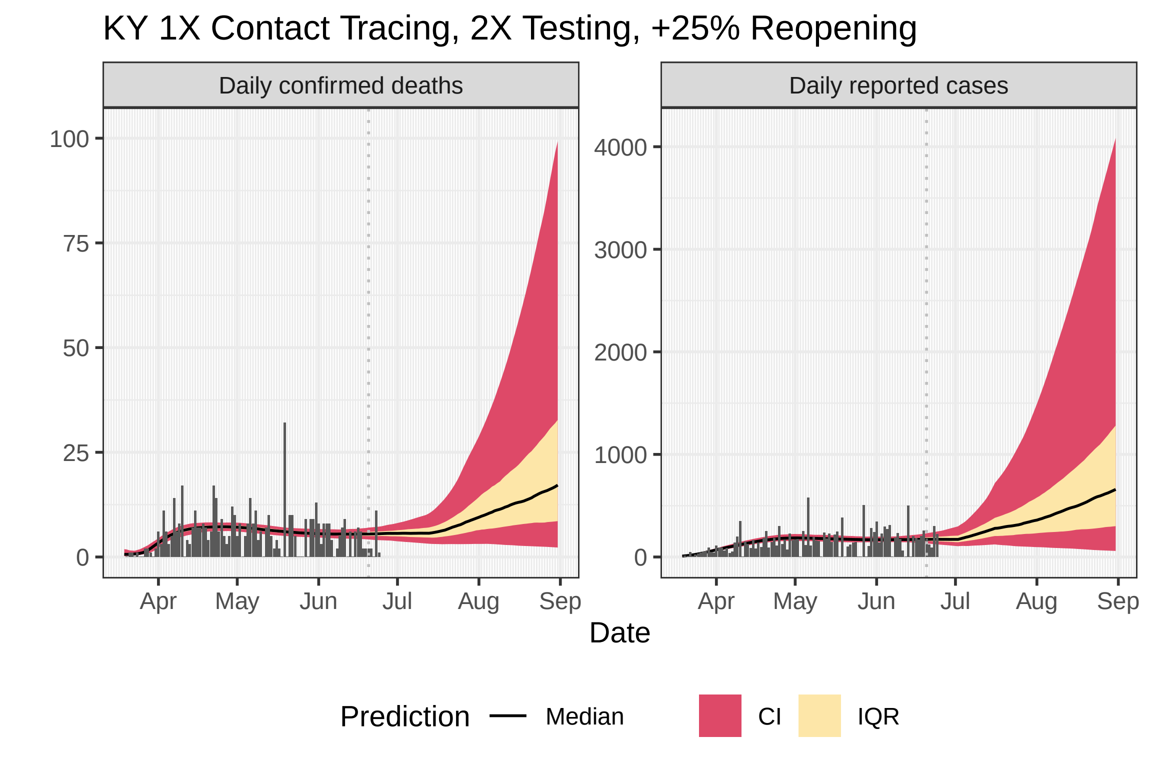
KY 1X Contact Tracing, 2X Testing, +25% Reopening
Daily confirmed deaths
Daily reported cases
100
75
50
25
0
4000
3000
2000
1000
0
Apr
May
Jun
Jul
Aug
Sep
Date
Prediction
Median
CI
IQR

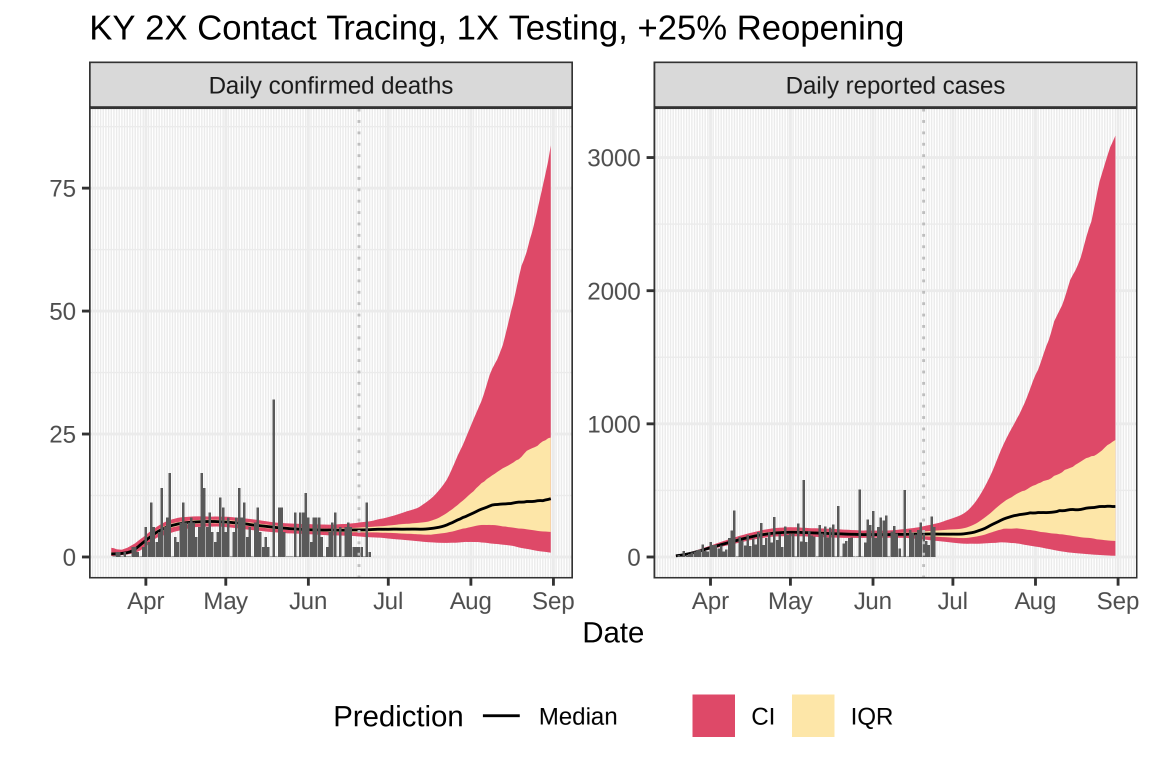

KY 2X Contact Tracing, 1X Testing, +25% Reopening
Daily confirmed deaths
Daily reported cases
0
25
50
75
0
1000
2000
3000
Apr
May
Jun
Jul
Aug
Sep
Date
Prediction
Median
CI
IQR

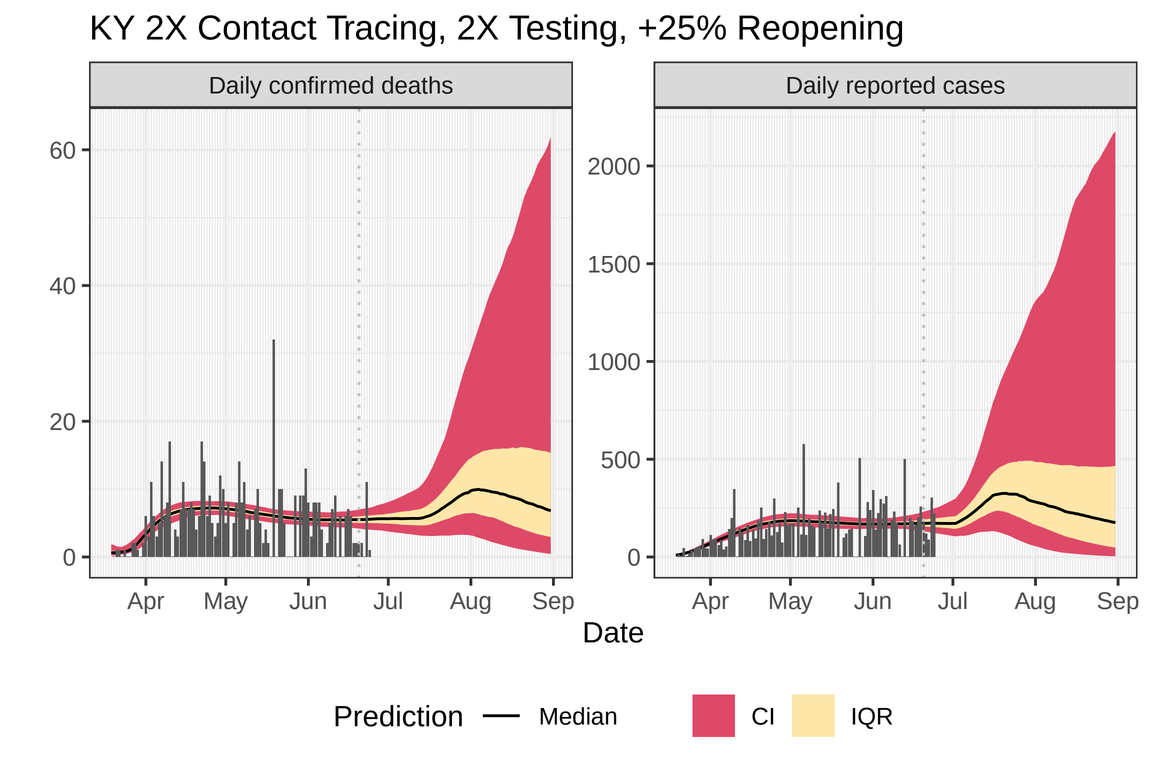
KY 2X Contact Tracing, 2X Testing, +25% Reopening
Daily confirmed deaths
Daily reported cases
0
20
40
60
0
500
1000
1500
2000
Apr
May
Jun
Jul
Aug
Sep
Date
Prediction
Median
CI
IQR

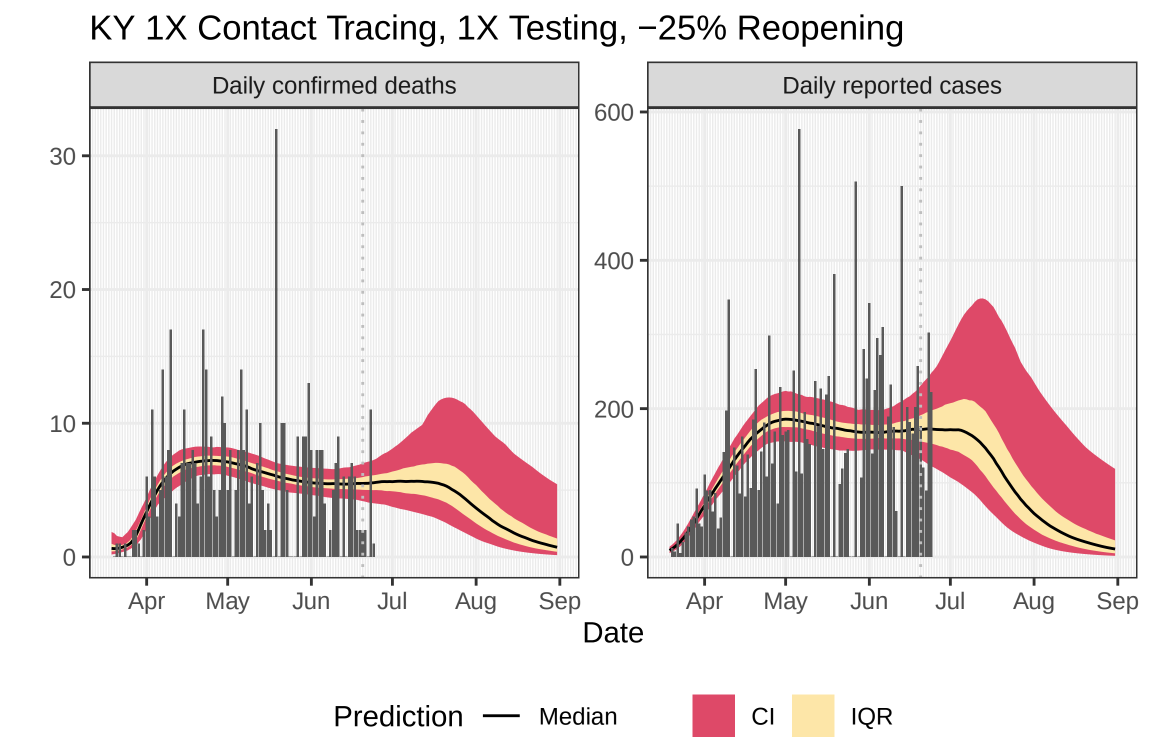

KY 1X Contact Tracing, 1X Testing, −25% Reopening
Daily confirmed deaths
Daily reported cases
30
20
10
0
600
400
200
0
Apr
May
Jun
Jul
Aug
Sep
Date
Prediction
Median
CI
IQR

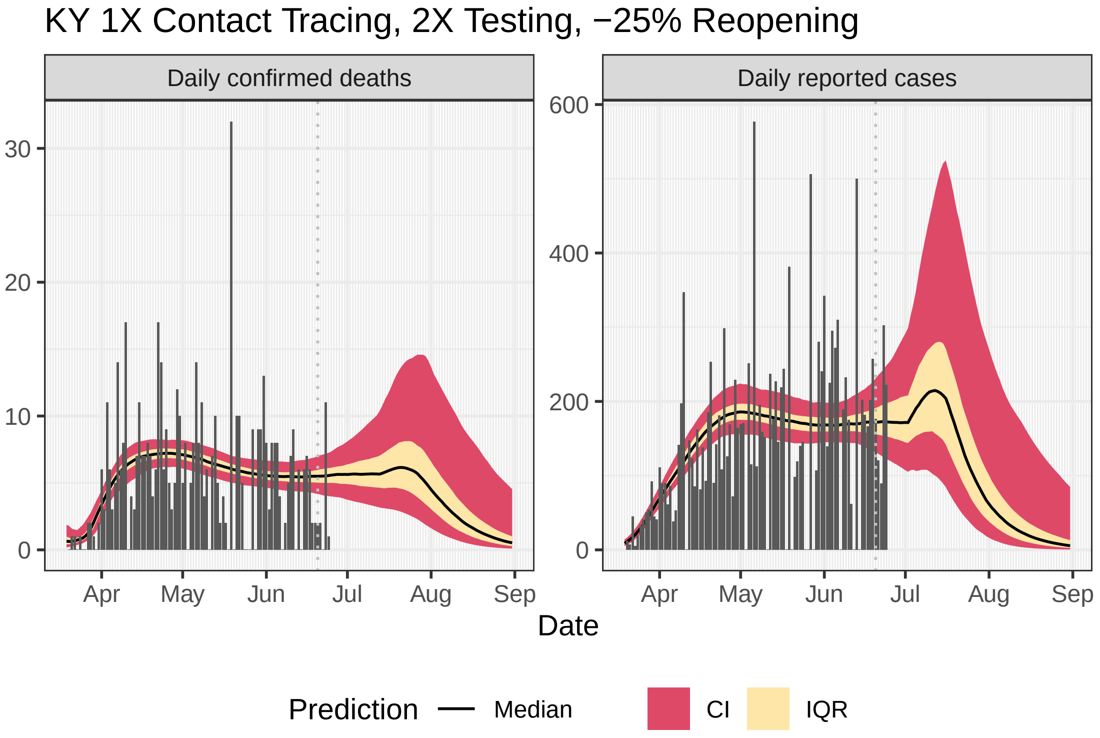

KY 1X Contact Tracing, 2X Testing, −25% Reopening
Daily confirmed deaths
Daily reported cases
30
20
10
0
600
400
200
0
Apr
May
Jun
Jul
Aug
Sep
Date
Prediction
Median
CI
IQR

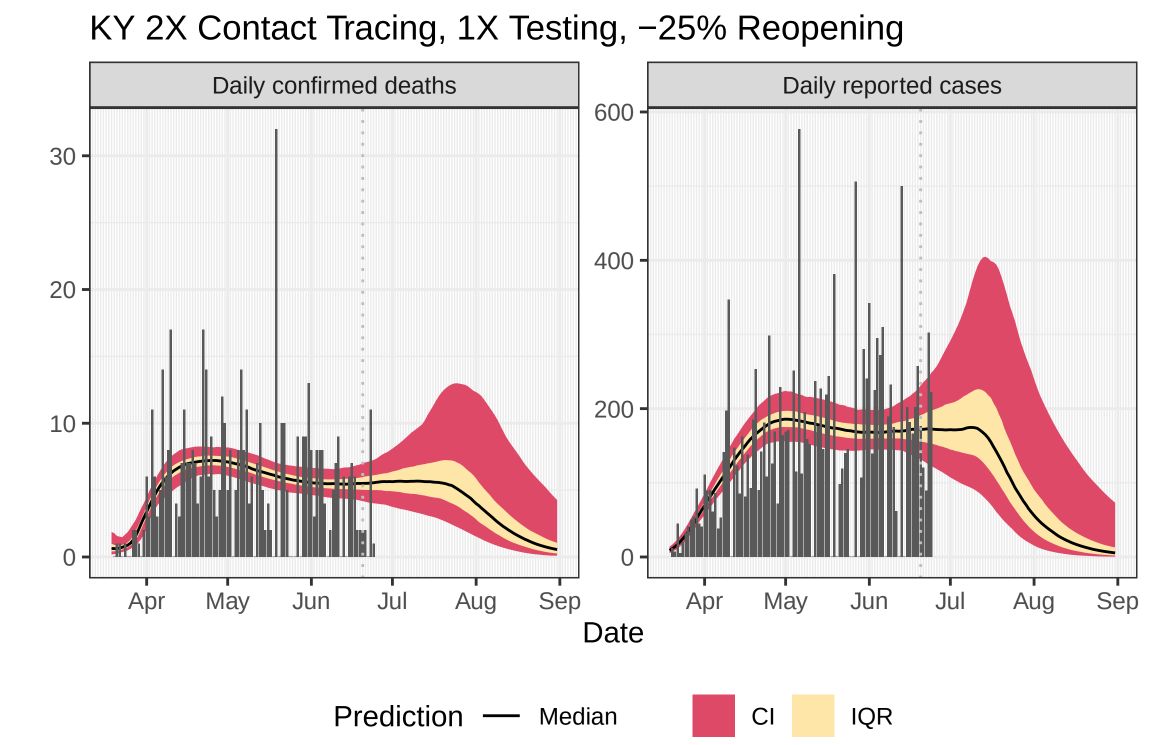
KY 2X Contact Tracing, 1X Testing, −25% Reopening
Daily confirmed deaths
Daily reported cases
30
20
10
0
600
400
200
0
Apr
May
Jun
Jul
Aug
Sep
Date
Prediction
Median
CI
IQR

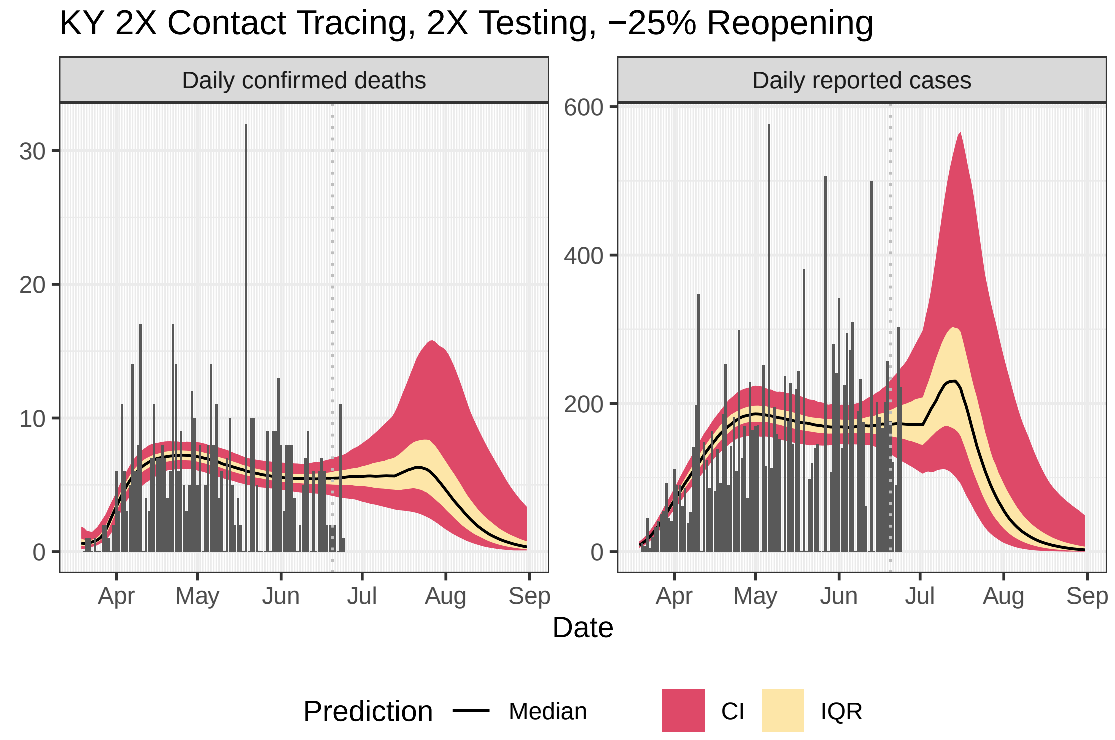

KY 2X Contact Tracing, 2X Testing, −25% Reopening
Daily confirmed deaths
Daily reported cases
0
10
20
30
0
200
400
600
Apr
May
Jun
Jul
Aug
Sep
Date
Prediction
Median
CI
IQR

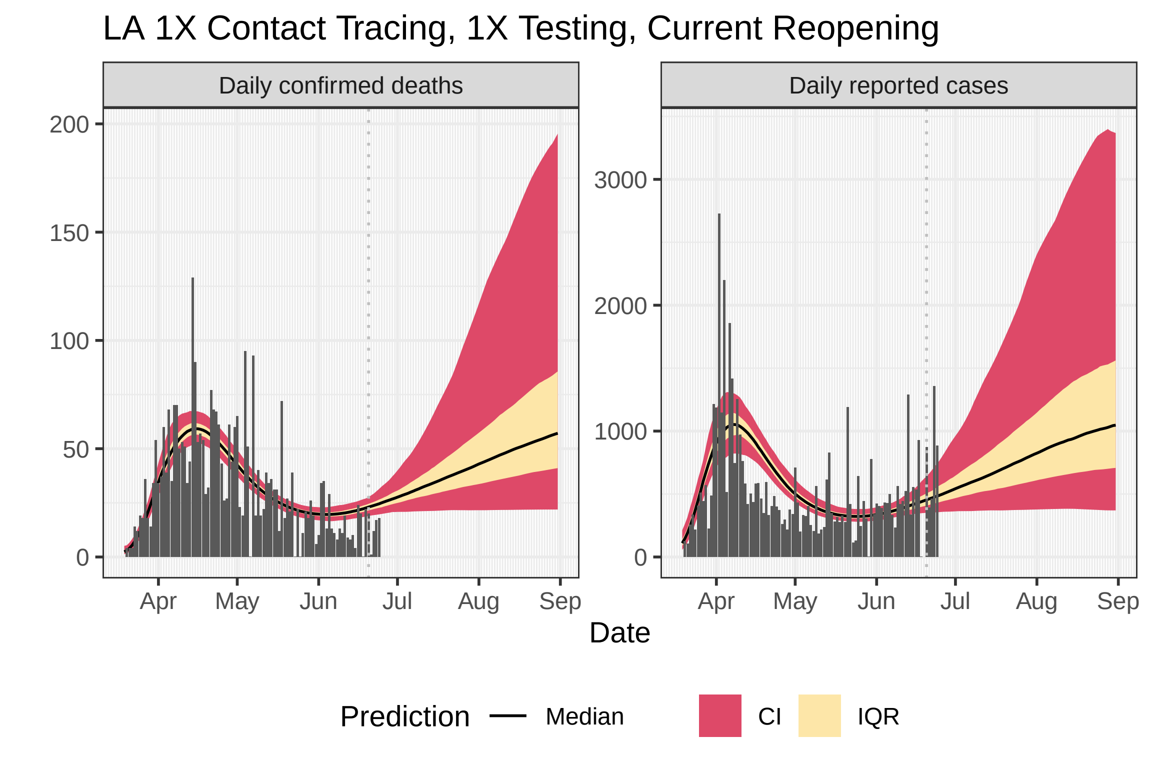
LA 1X Contact Tracing, 1X Testing, Current Reopening
Daily confirmed deaths
Daily reported cases
200
150
100
50
0
3000
2000
1000
0
Apr
May
Jun
Jul
Aug
Sep
Date
Prediction
Median
CI
IQR

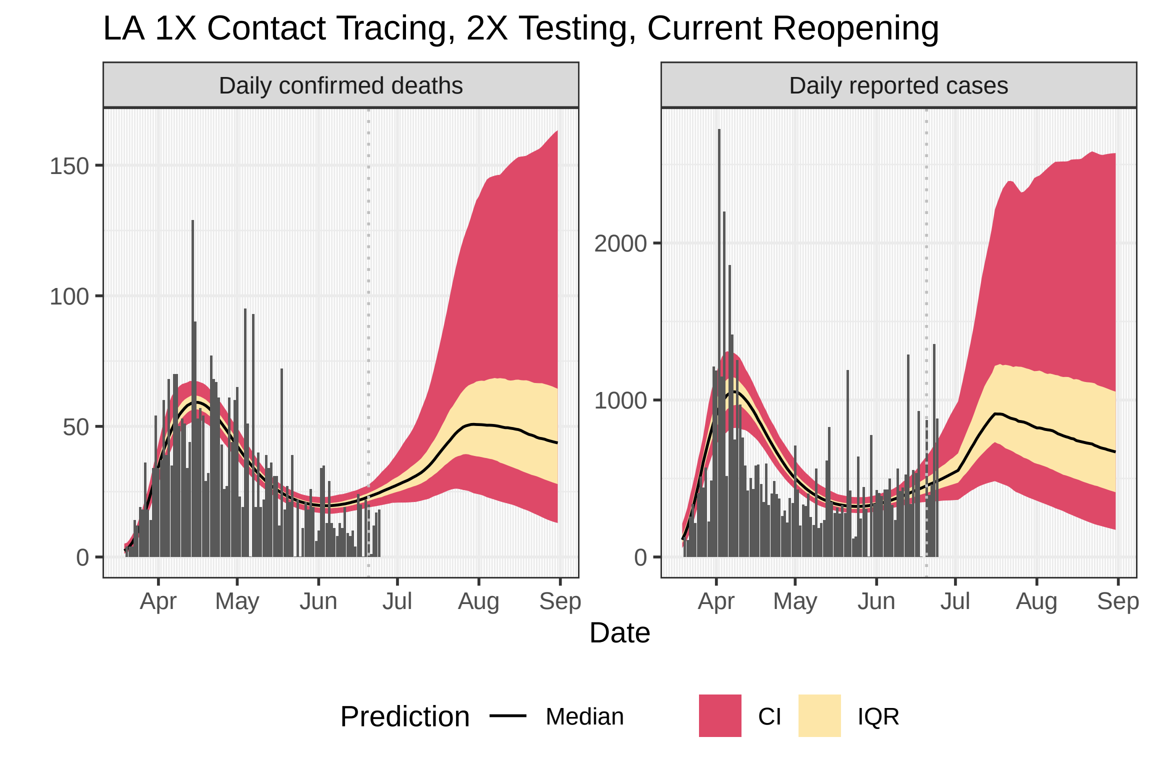
LA 1X Contact Tracing, 2X Testing, Current Reopening
Daily confirmed deaths
Daily reported cases
150
100
50
0
2000
1000
0
Apr
May
Jun
Jul
Aug
Sep
Date
Prediction
Median
CI
IQR

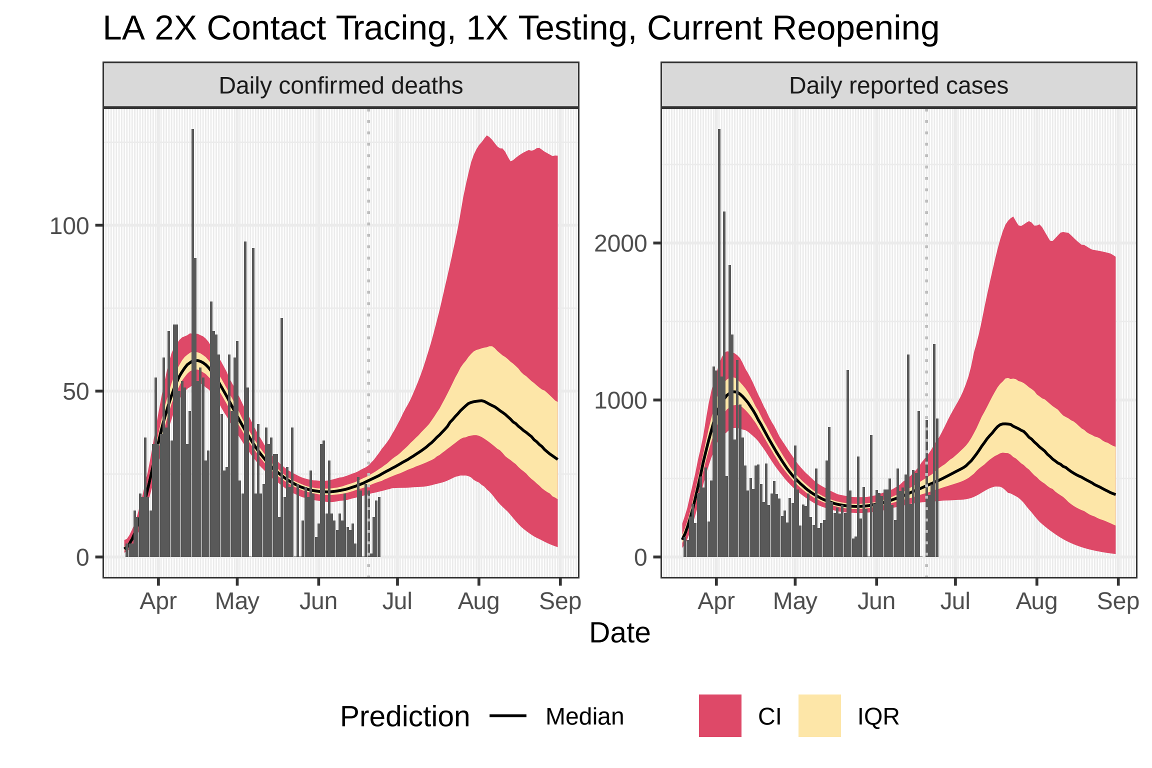

LA 2X Contact Tracing, 1X Testing, Current Reopening
Daily confirmed deaths
Daily reported cases
0
50
100
0
1000
2000
Apr
May
Jun
Jul
Aug
Sep
Date
Prediction
Median
CI
IQR

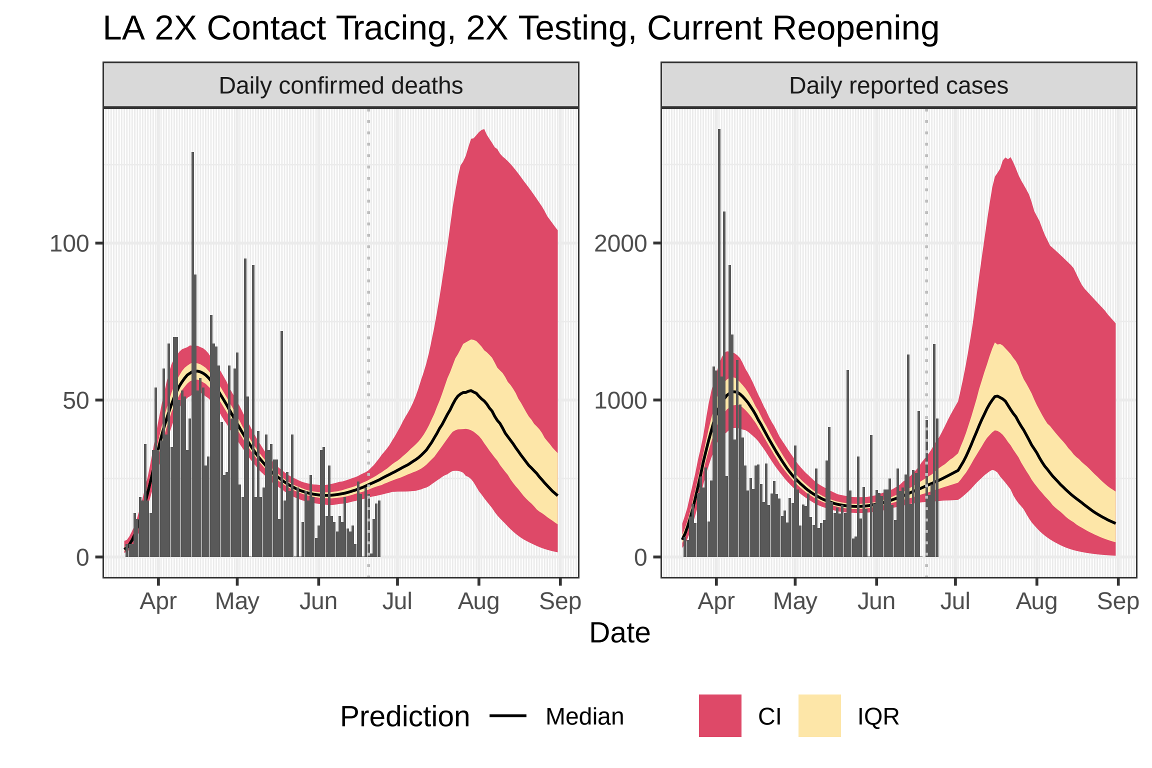

LA 2X Contact Tracing, 2X Testing, Current Reopening
Daily confirmed deaths
Daily reported cases
0
50
100
0
1000
2000
Apr
May
Jun
Jul
Aug
Sep
Date
Prediction
Median
CI
IQR

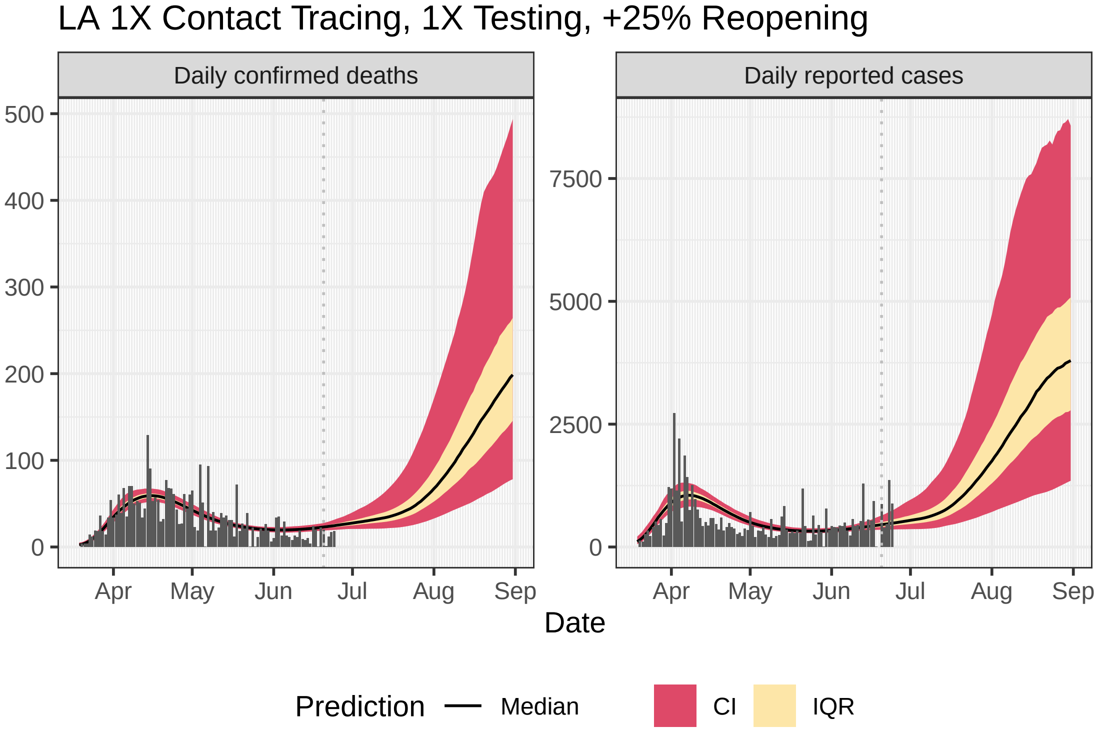

LA 1X Contact Tracing, 1X Testing, +25% Reopening
Daily confirmed deaths
Daily reported cases
500
400
300
200
100
0
7500
5000
2500
0
Apr
May
Jun
Jul
Aug
Sep
Date
Prediction
Median
CI
IQR

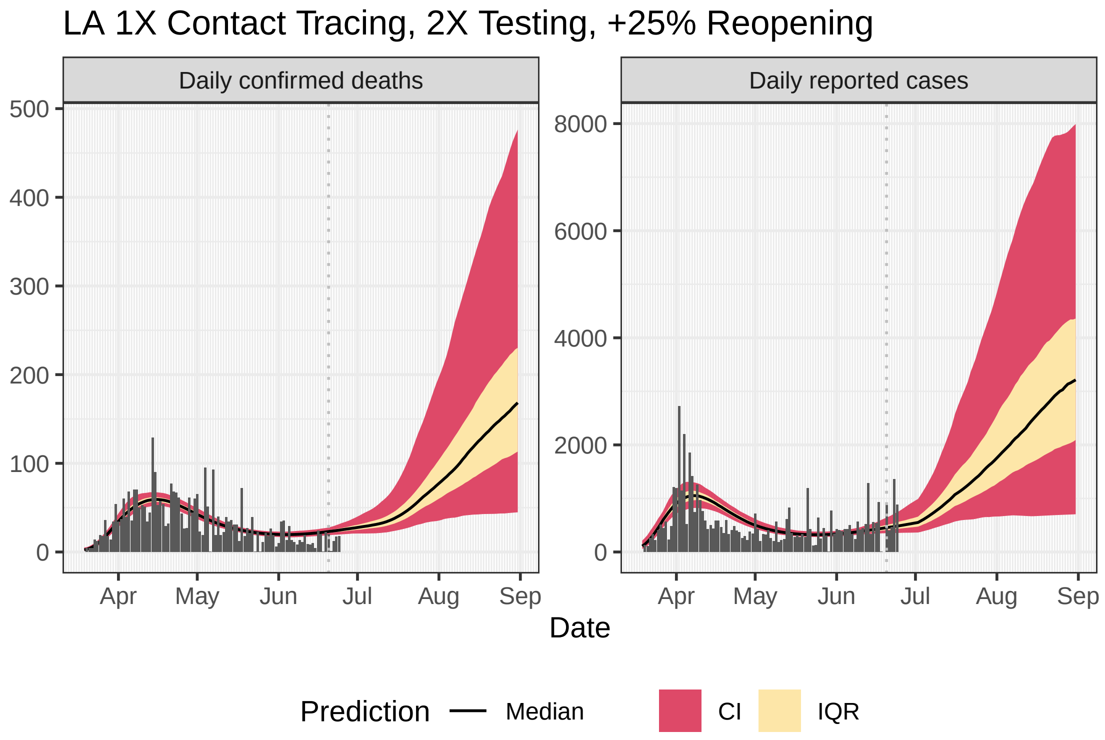

LA 1X Contact Tracing, 2X Testing, +25% Reopening
Daily confirmed deaths
Daily reported cases
500
400
300
200
100
0
8000
6000
4000
2000
0
Apr
May
Jun
Jul
Aug
Sep
Date
Prediction
Median
CI
IQR

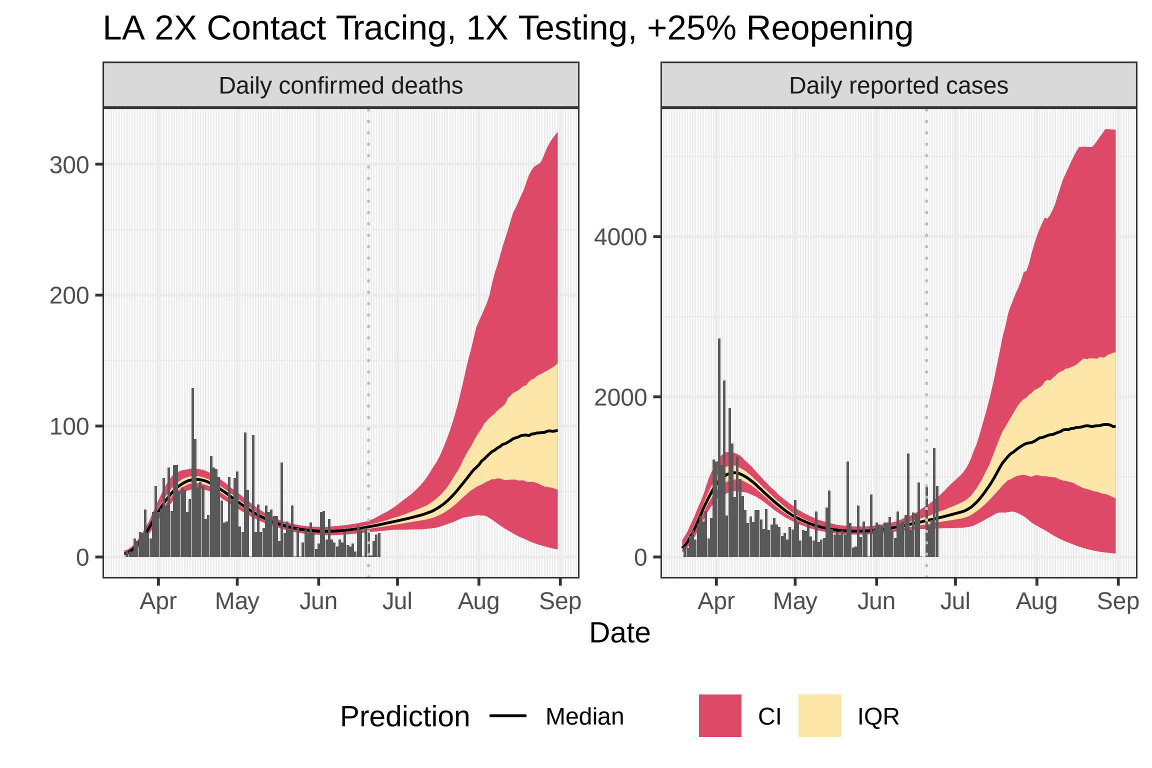

LA 2X Contact Tracing, 1X Testing, +25% Reopening
Daily confirmed deaths
Daily reported cases
300
200
100
0
4000
2000
0
Apr
May
Jun
Jul
Aug
Sep
Date
Prediction
Median
CI
IQR

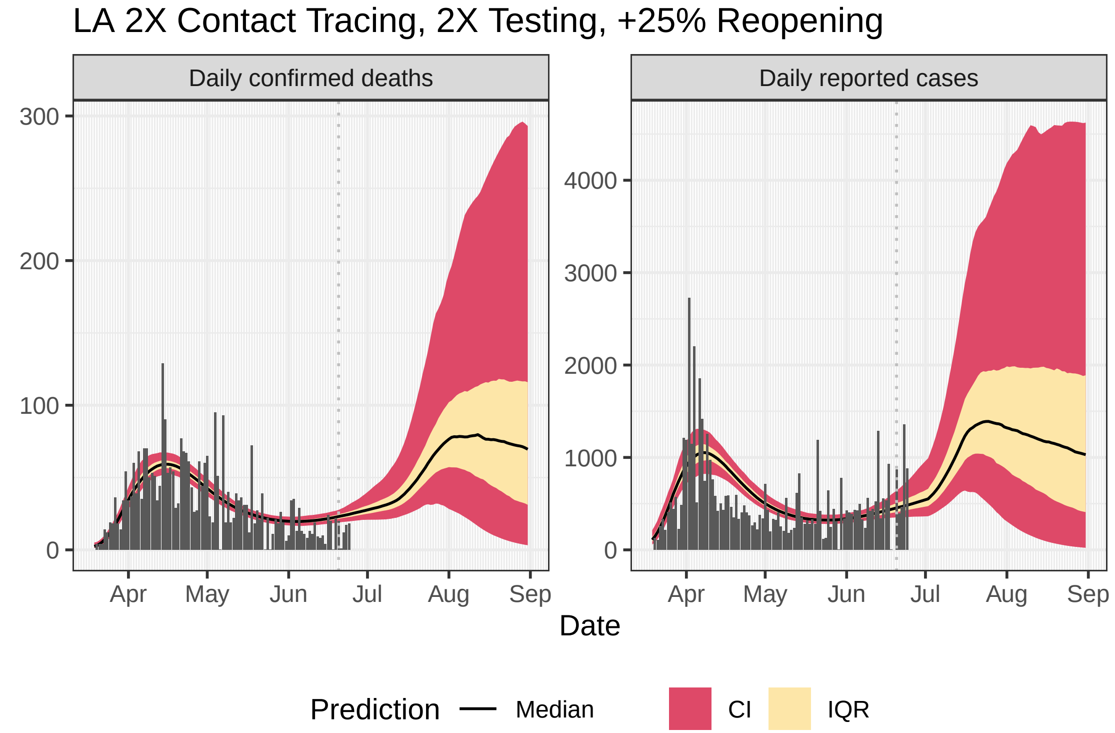
LA 2X Contact Tracing, 2X Testing, +25% Reopening
Daily confirmed deaths
Daily reported cases
300
200
100
0
4000
3000
2000
1000
0
Apr
May
Jun
Jul
Aug
Sep
Date
Prediction
Median
CI
IQR

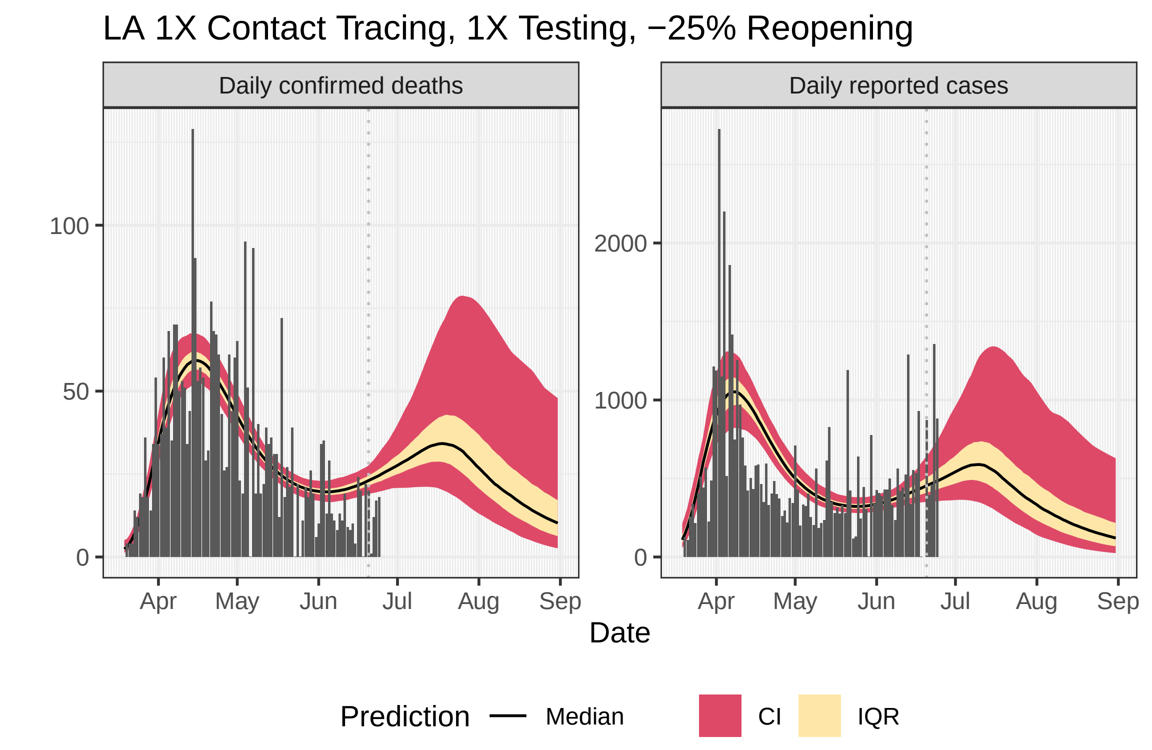

LA 1X Contact Tracing, 1X Testing, −25% Reopening
Daily confirmed deaths
Daily reported cases
100
50
0
2000
1000
0
Apr
May
Jun
Jul
Aug
Sep
Date
Prediction
Median
CI
IQR

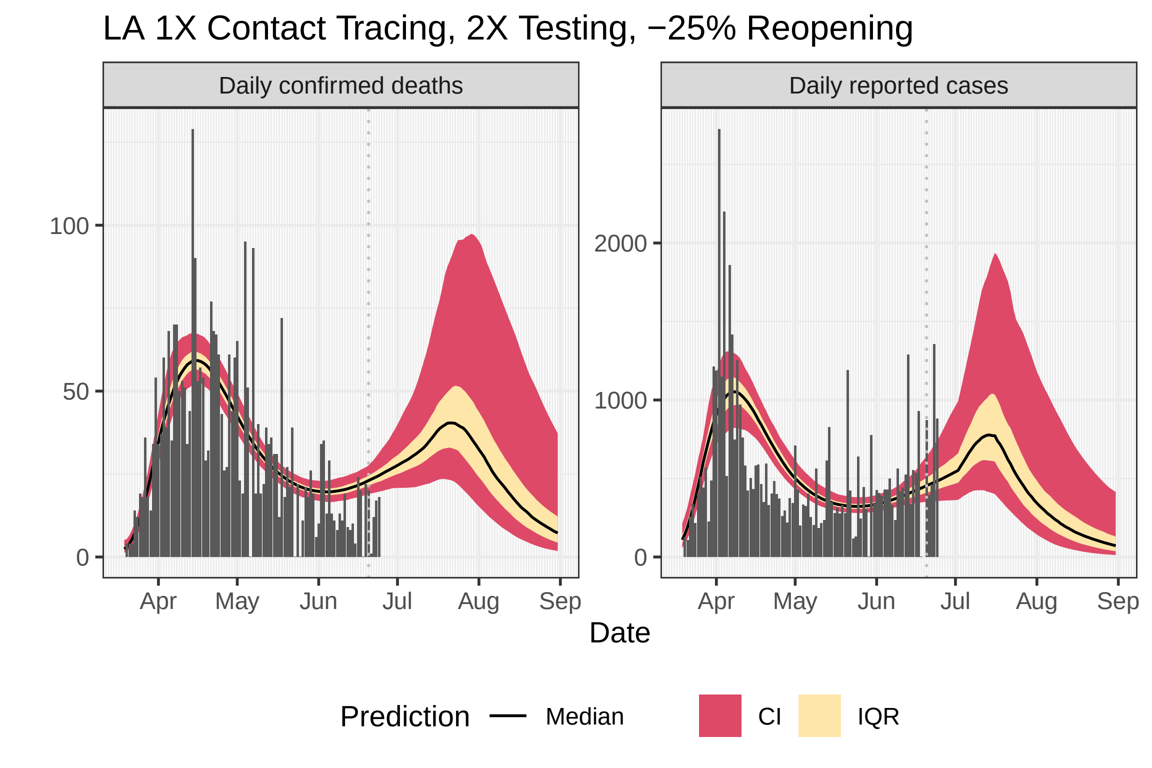

LA 1X Contact Tracing, 2X Testing, −25% Reopening
Daily confirmed deaths
Daily reported cases
100
50
0
2000
1000
0
Apr
May
Jun
Jul
Aug
Sep
Date
Prediction
Median
CI
IQR

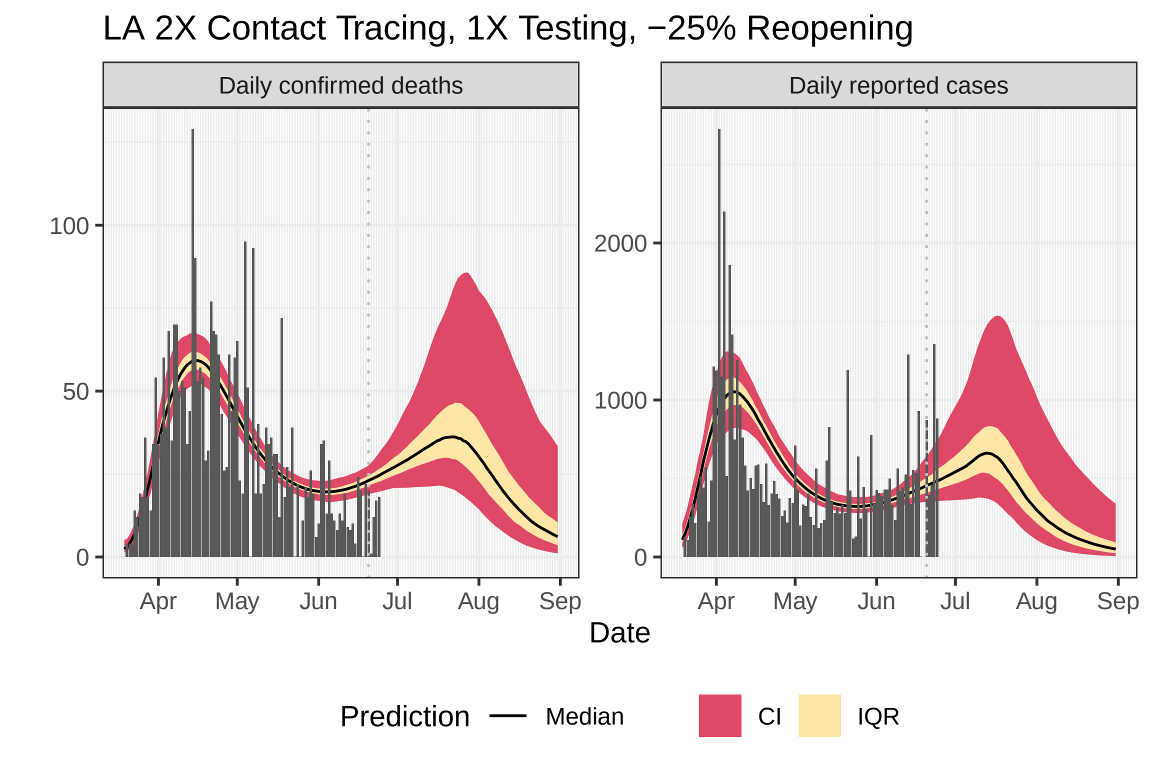

LA 2X Contact Tracing, 1X Testing, −25% Reopening
Daily confirmed deaths
Daily reported cases
100
50
0
2000
1000
0
Apr
May
Jun
Jul
Aug
Sep
Date
Prediction
Median
CI
IQR

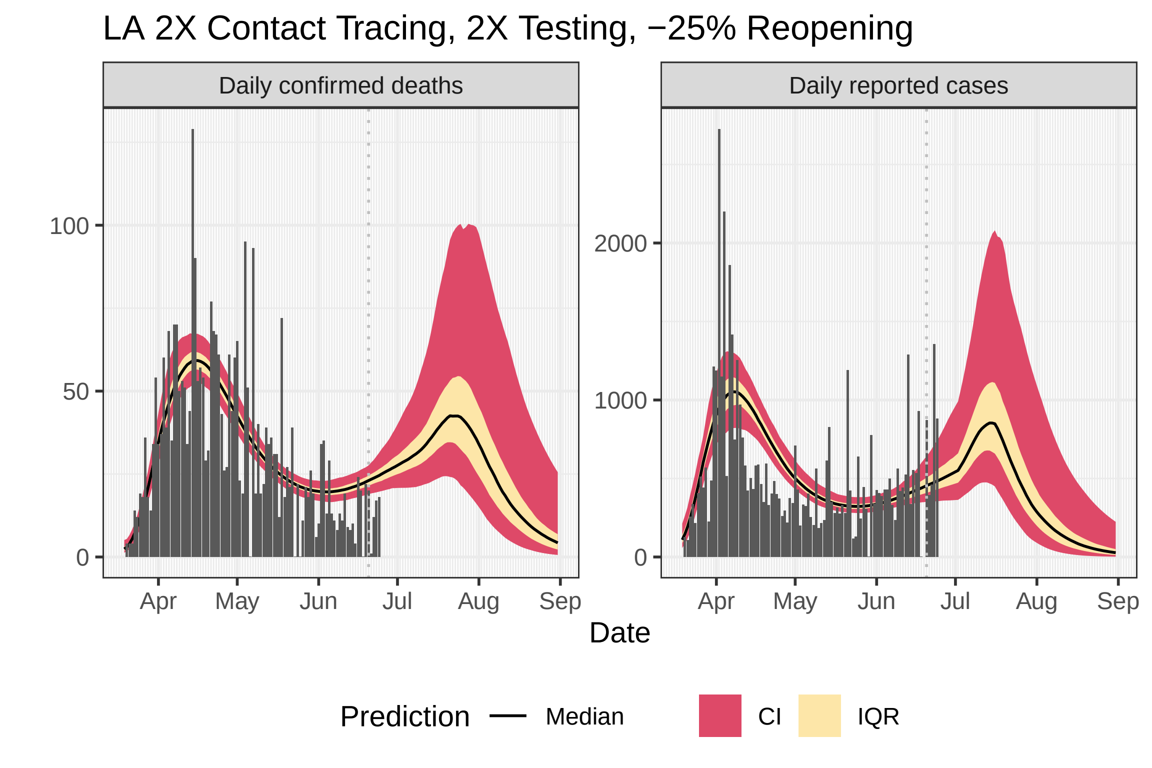

LA 2X Contact Tracing, 2X Testing, −25% Reopening
Daily confirmed deaths
Daily reported cases
0
50
100
1000
2000
Apr
May
Jun
Jul
Aug
Sep
Date
Prediction
Median
CI
IQR

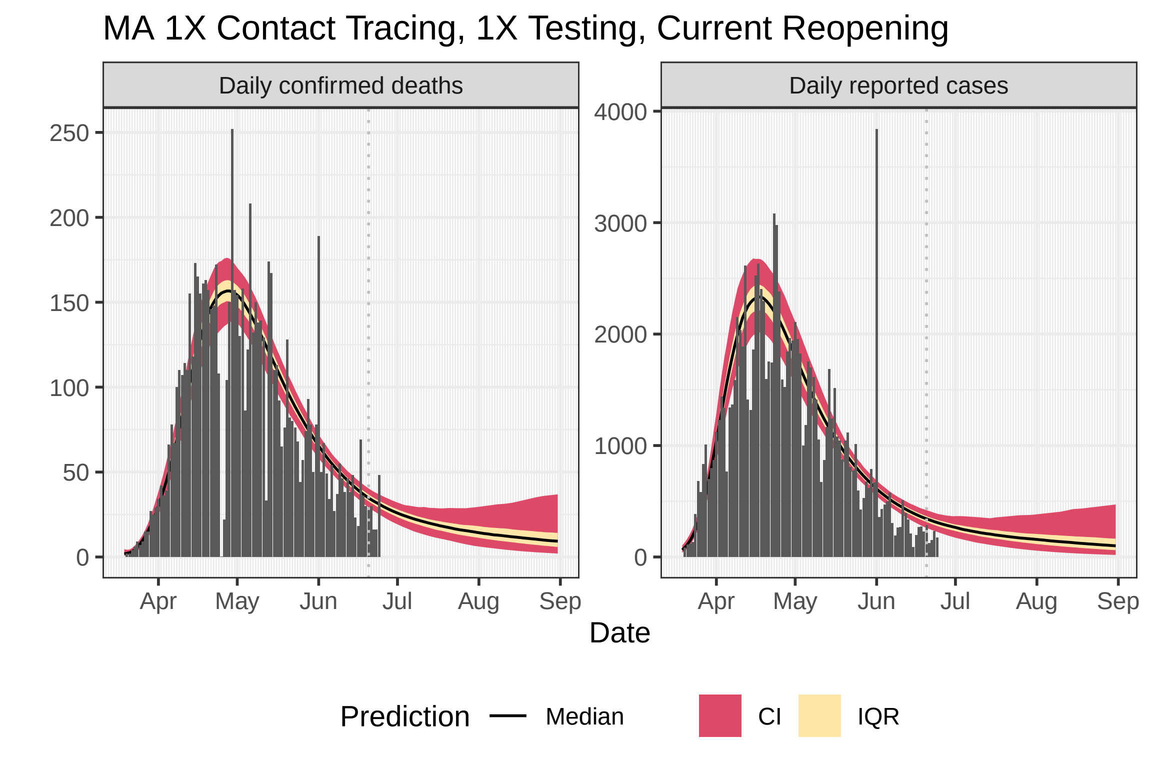

MA 1X Contact Tracing, 1X Testing, Current Reopening
Daily confirmed deaths
Daily reported cases
250
200
150
100
50
0
4000
3000
2000
1000
0
Apr
May
Jun
Jul
Aug
Sep
Date
Prediction
Median
CI
IQR

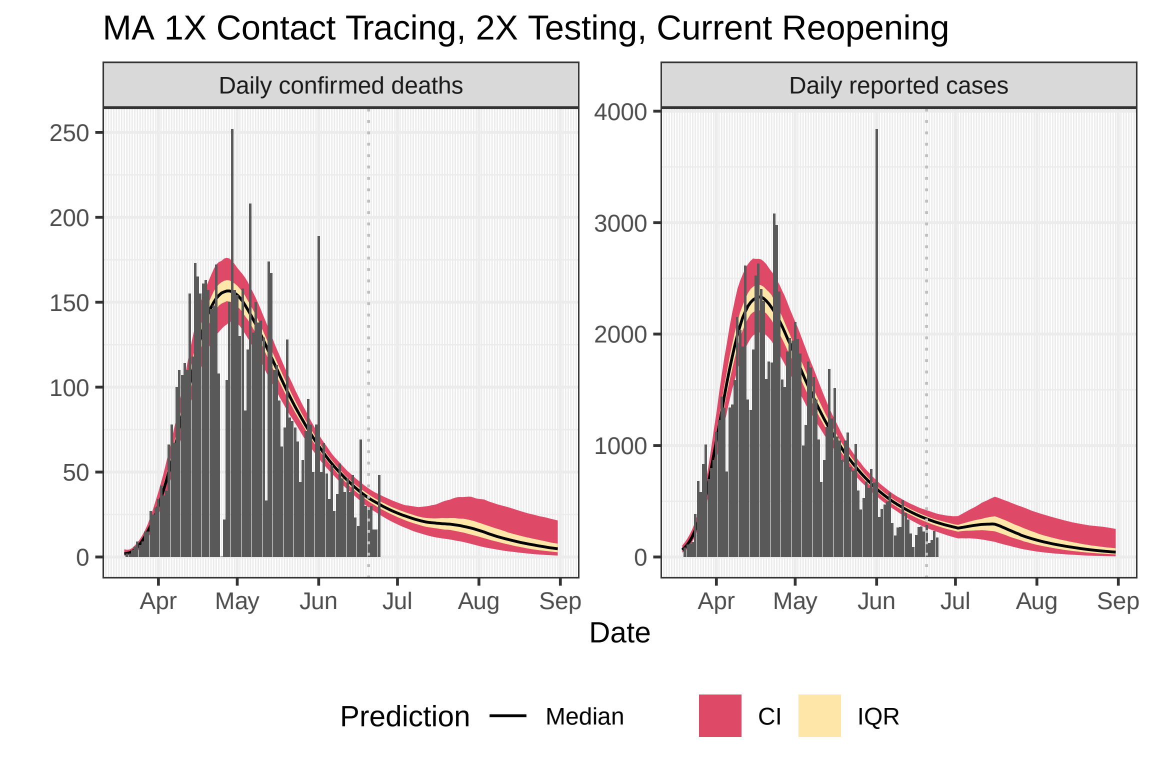
MA 1X Contact Tracing, 2X Testing, Current Reopening
Daily confirmed deaths
Daily reported cases
250
200
150
100
50
0
4000
3000
2000
1000
0
Apr
May
Jun
Jul
Aug
Sep
Date
Prediction
Median
CI
IQR

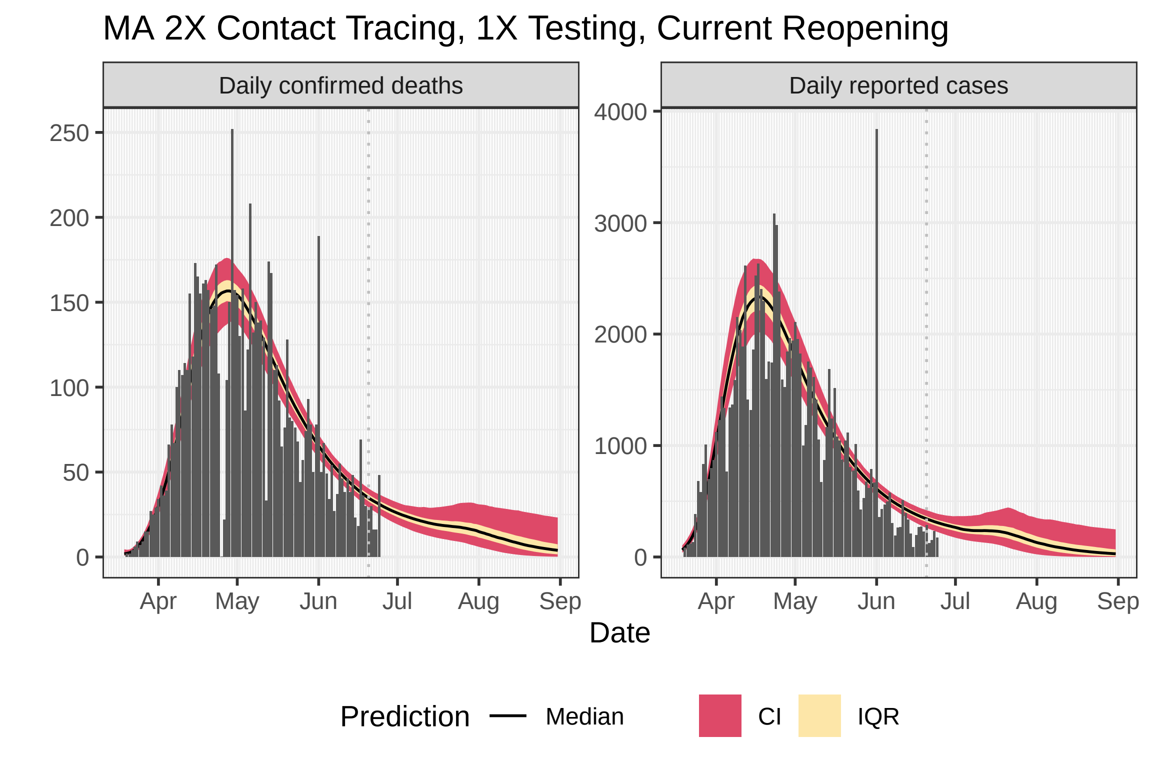

MA 2X Contact Tracing, 1X Testing, Current Reopening
Daily confirmed deaths
Daily reported cases
250
200
150
100
50
0
4000
3000
2000
1000
0
Apr
May
Jun
Jul
Aug
Sep
Date
Prediction
Median
CI
IQR

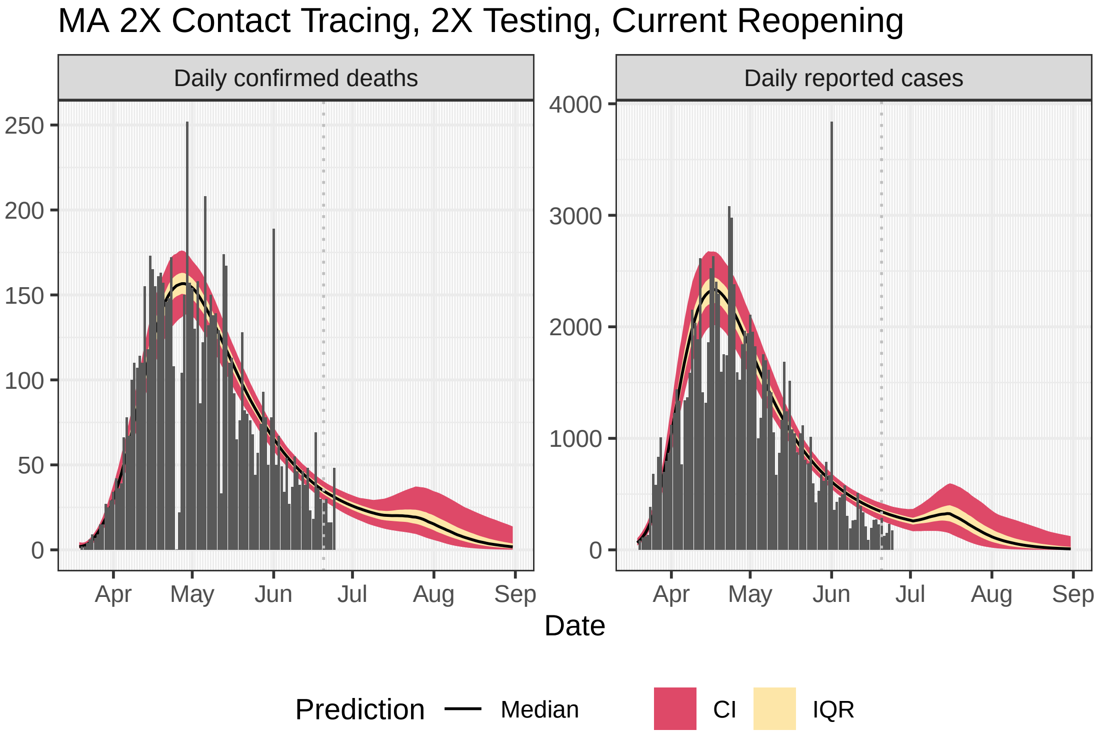

MA 2X Contact Tracing, 2X Testing, Current Reopening
Daily confirmed deaths
Daily reported cases
250
200
150
100
50
0
4000
3000
2000
1000
0
Apr
May
Jun
Jul
Aug
Sep
Date
Prediction
Median
CI
IQR

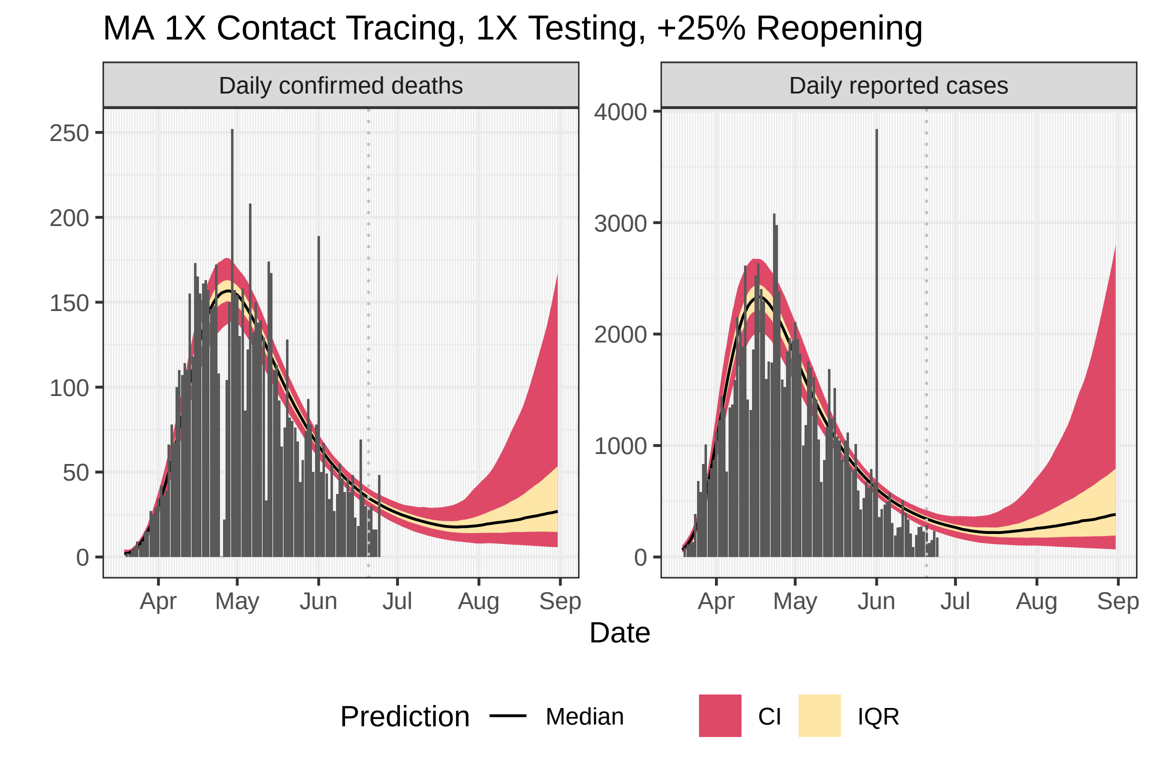
MA 1X Contact Tracing, 1X Testing, +25% Reopening
Daily confirmed deaths
Daily reported cases
250
200
150
100
50
0
4000
3000
2000
1000
0
Apr
May
Jun
Jul
Aug
Sep
Date
Prediction
Median
CI
IQR

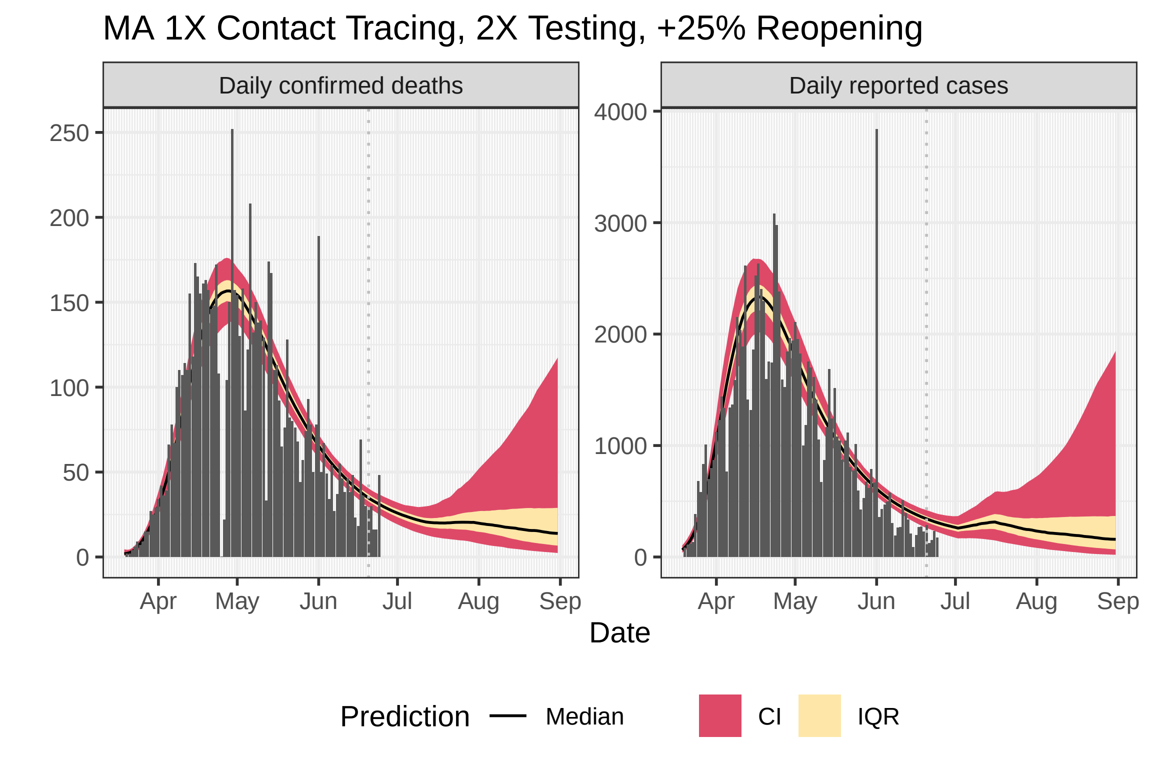

MA 1X Contact Tracing, 2X Testing, +25% Reopening
Daily confirmed deaths
Daily reported cases
0
50
100
150
200
250
0
1000
2000
3000
4000
Apr
May
Jun
Jul
Aug
Sep
Date
Prediction
Median
CI
IQR

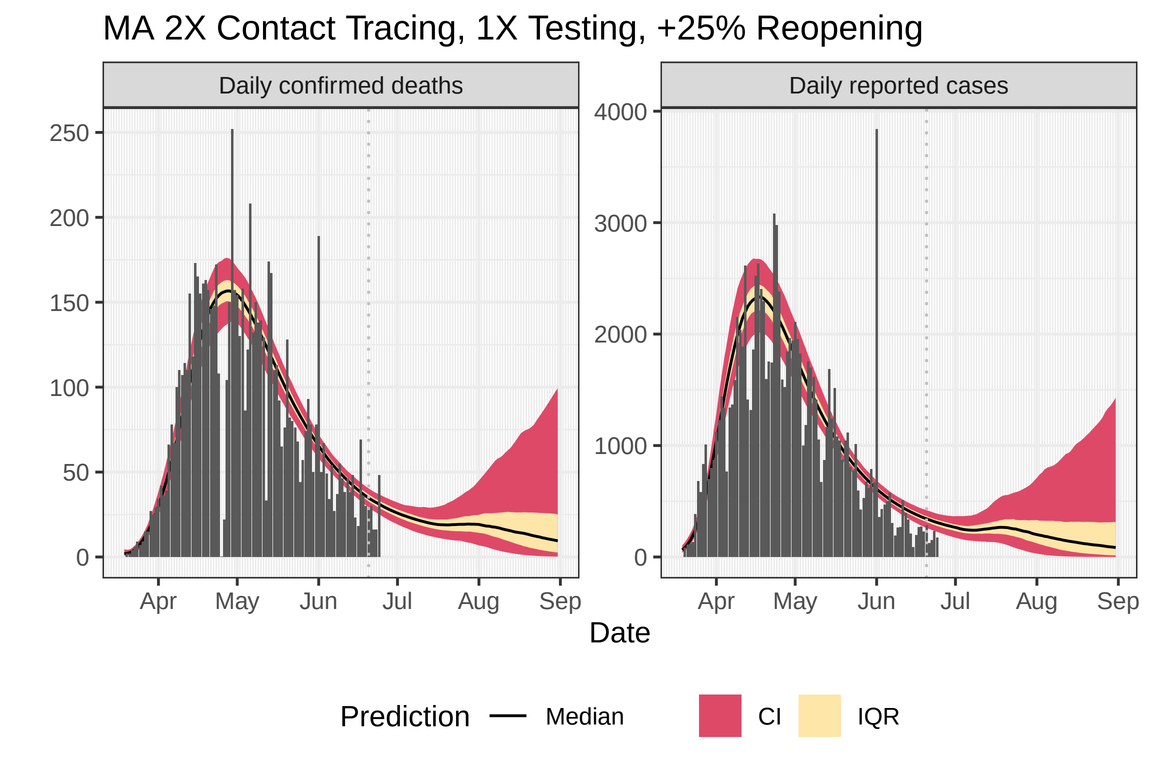

MA 2X Contact Tracing, 1X Testing, +25% Reopening
Daily confirmed deaths
Daily reported cases
250
200
150
100
50
0
4000
3000
2000
1000
0
Apr
May
Jun
Jul
Aug
Sep
Date
Prediction
Median
CI
IQR

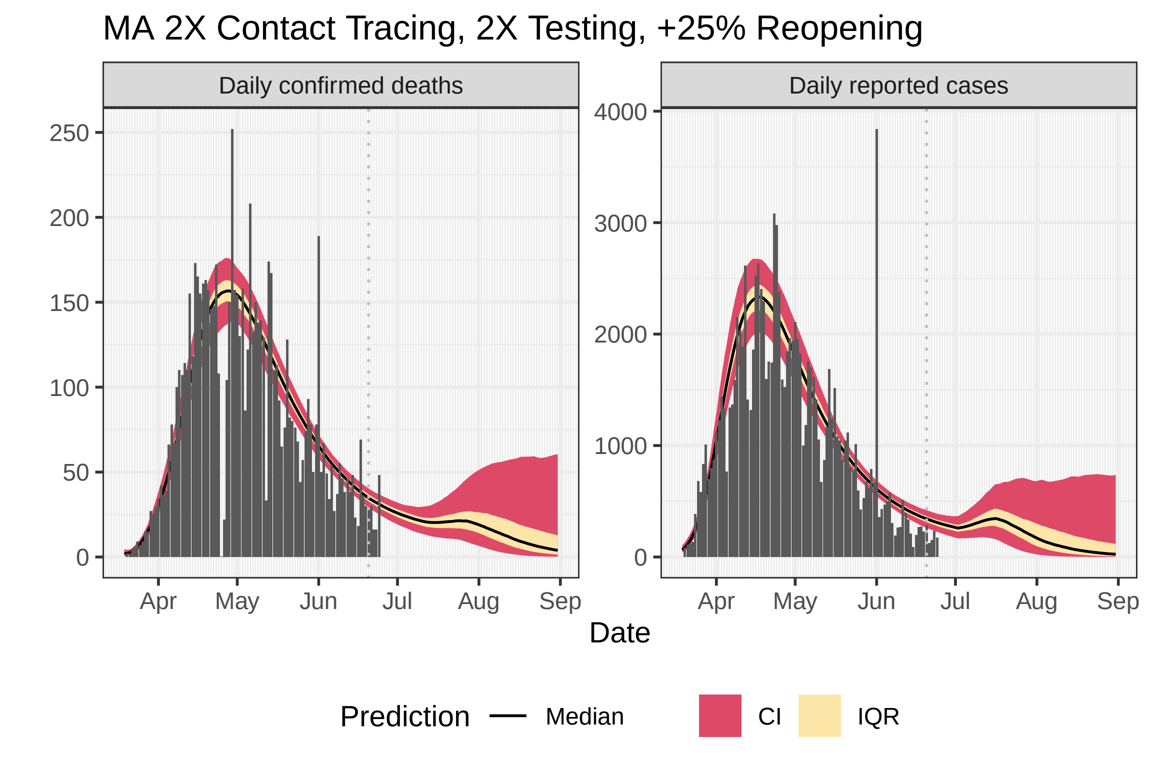

MA 2X Contact Tracing, 2X Testing, +25% Reopening
Daily confirmed deaths
Daily reported cases
250
200
150
100
50
0
4000
3000
2000
1000
0
Apr
May
Jun
Jul
Aug
Sep
Date
Prediction
Median
CI
IQR

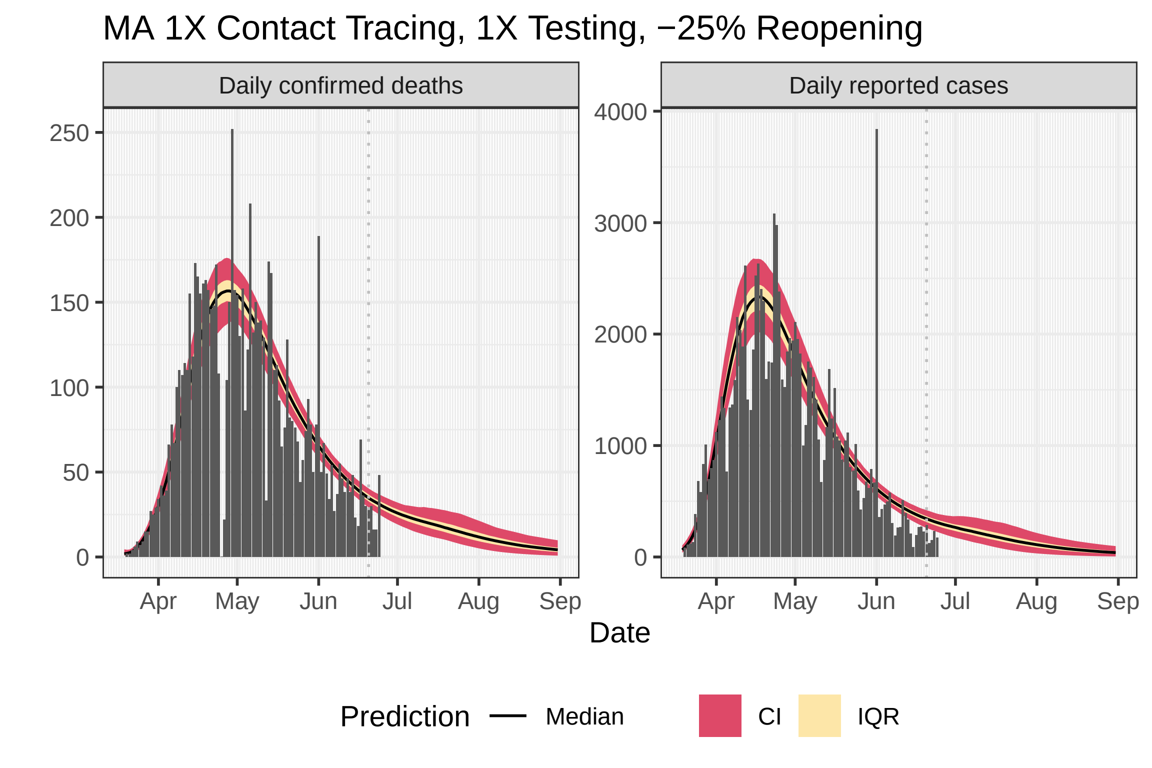

MA 1X Contact Tracing, 1X Testing, −25% Reopening
Daily confirmed deaths
Daily reported cases
250
200
150
100
50
0
4000
3000
2000
1000
0
Apr
May
Jun
Jul
Aug
Sep
Date
Prediction
Median
CI
IQR

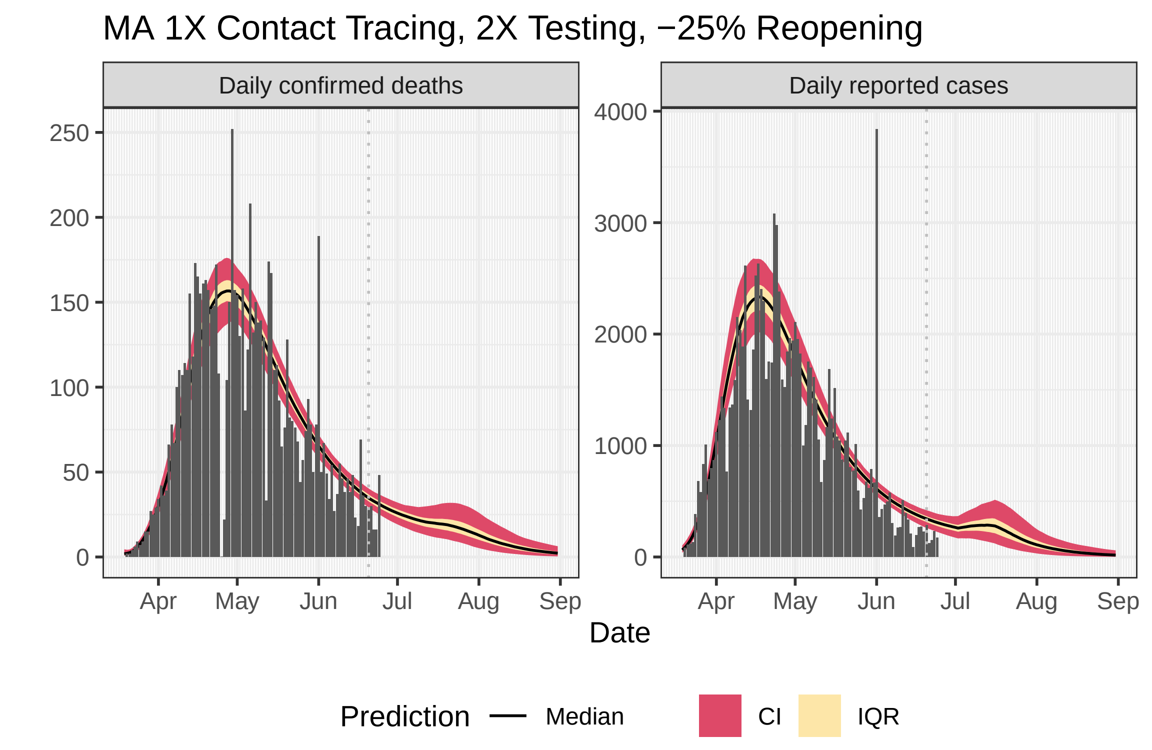
MA 1X Contact Tracing, 2X Testing, −25% Reopening
Daily confirmed deaths
Daily reported cases
250
200
150
100
50
0
4000
3000
2000
1000
0
Apr
May
Jun
Jul
Aug
Sep
Date
Prediction
Median
CI
IQR

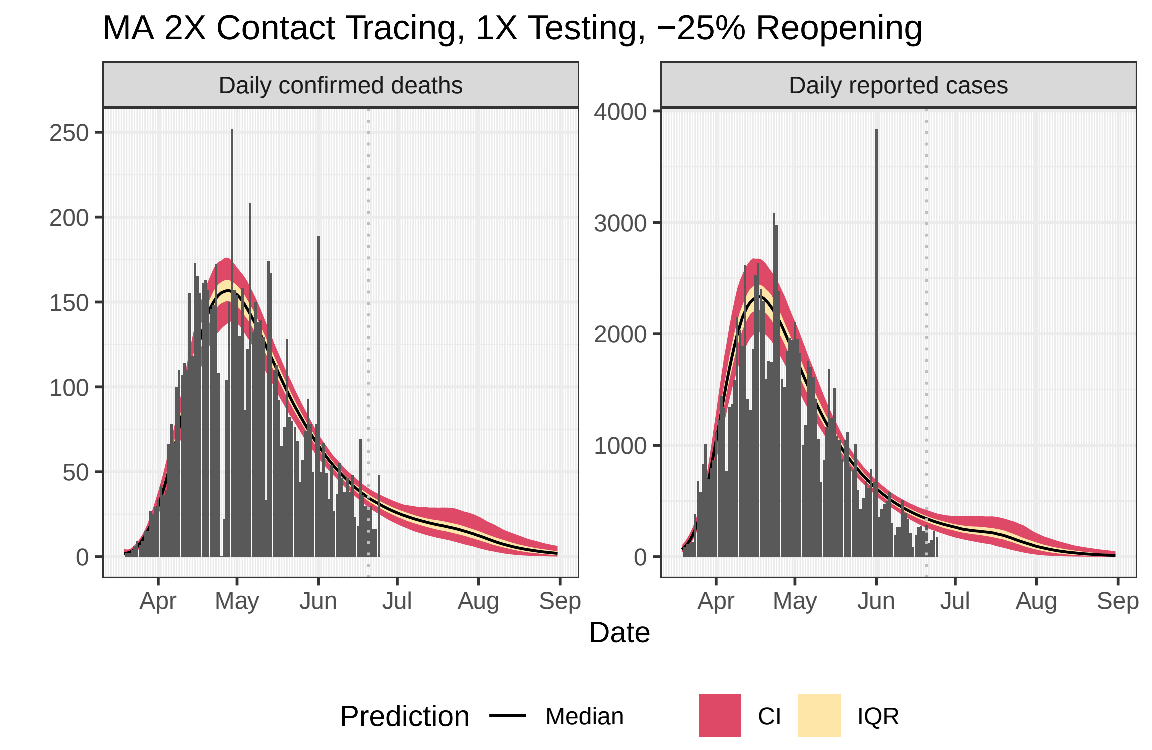

MA 2X Contact Tracing, 1X Testing, −25% Reopening
Daily confirmed deaths
Daily reported cases
250
200
150
100
50
0
4000
3000
2000
1000
0
Apr
May
Jun
Jul
Aug
Sep
Date
Prediction
Median
CI
IQR

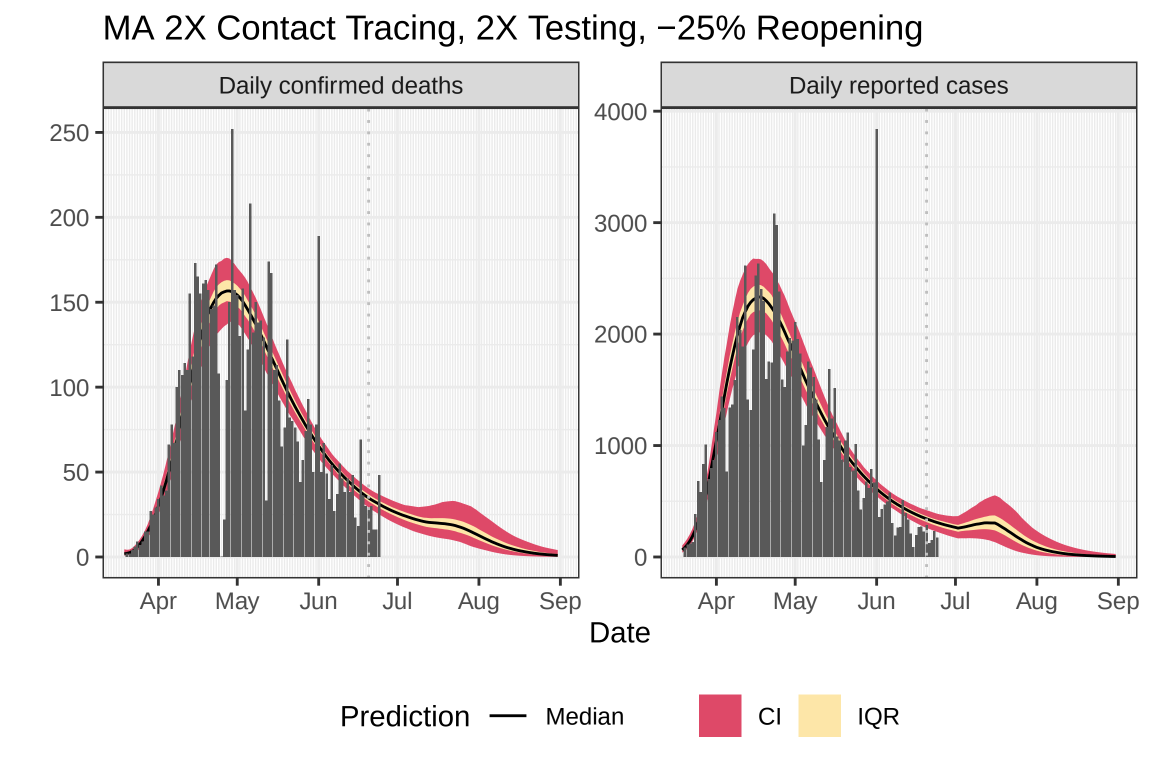
MA 2X Contact Tracing, 2X Testing, −25% Reopening
Daily confirmed deaths
Daily reported cases
250
200
150
100
50
0
4000
3000
2000
1000
0
Apr
May
Jun
Jul
Aug
Sep
Date
Prediction
Median
CI
IQR

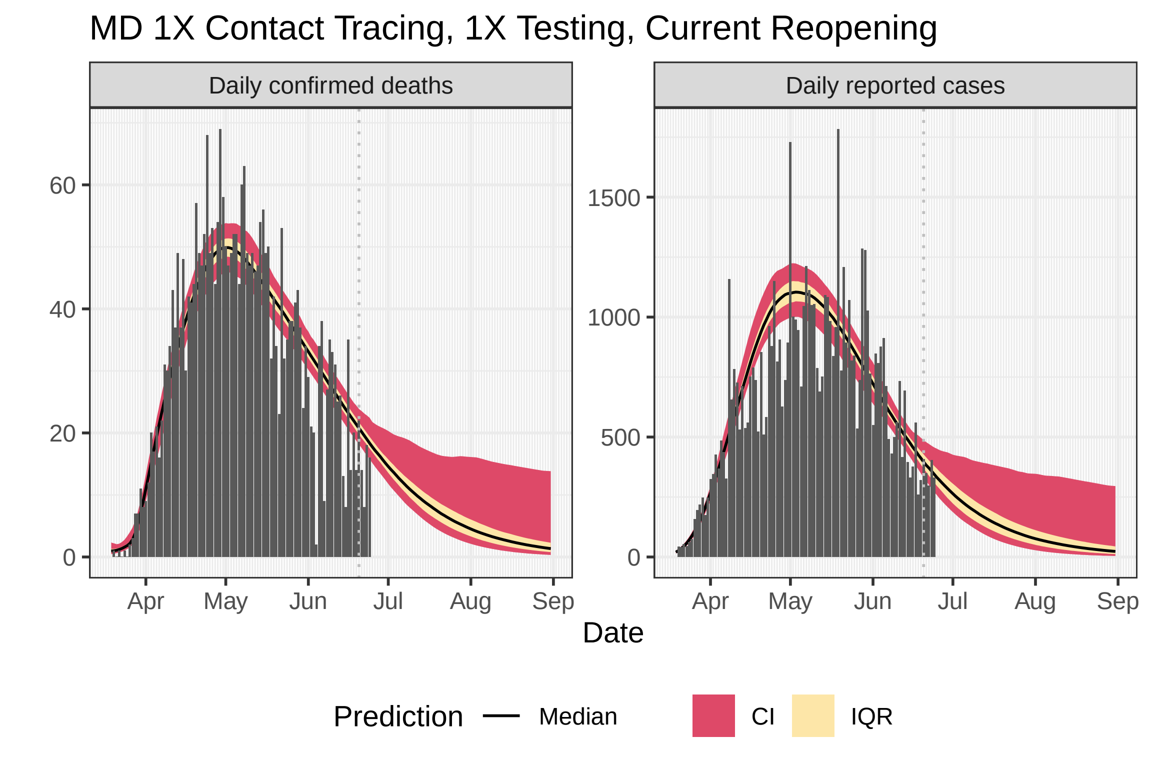

MD 1X Contact Tracing, 1X Testing, Current Reopening
Daily confirmed deaths
Daily reported cases
0
20
40
60
0
500
1000
1500
Apr
May
Jun
Jul
Aug
Sep
Date
Prediction
Median
CI
IQR

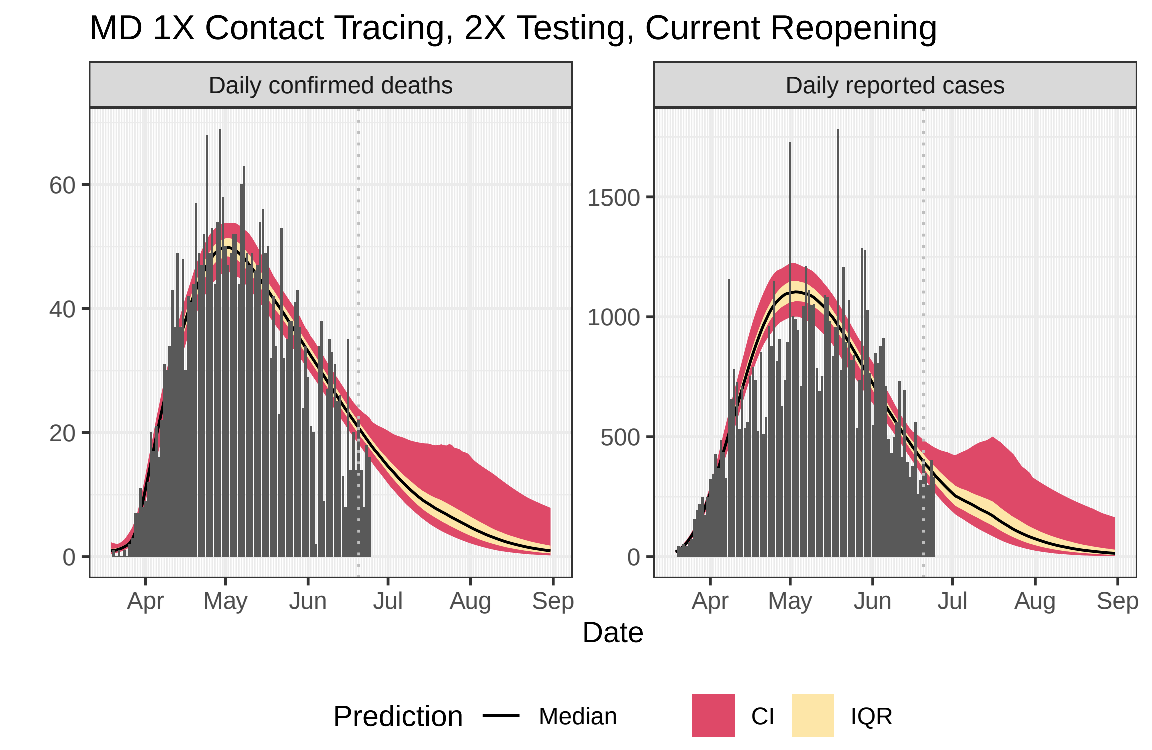

MD 1X Contact Tracing, 2X Testing, Current Reopening
Daily confirmed deaths
Daily reported cases
0
20
40
60
0
500
1000
1500
Apr
May
Jun
Jul
Aug
Sep
Date
Prediction
Median
CI
IQR

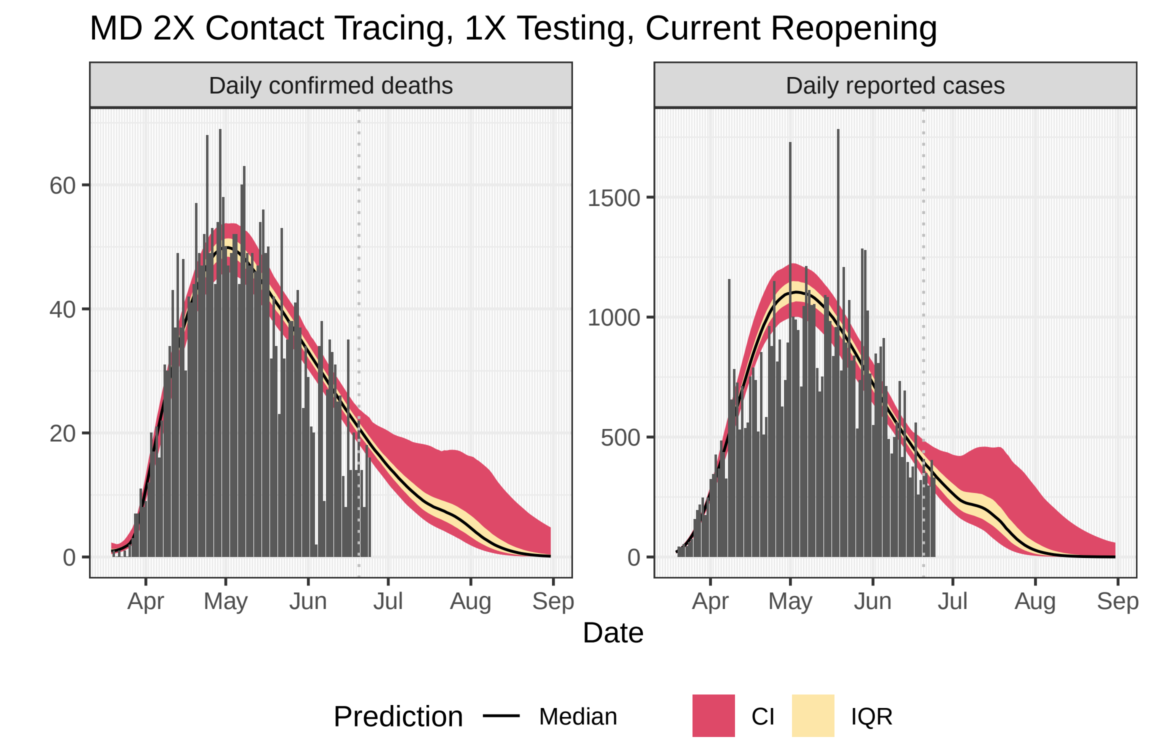
MD 2X Contact Tracing, 1X Testing, Current Reopening
Daily confirmed deaths
Daily reported cases
0
20
40
60
0
500
1000
1500
Apr
May
Jun
Jul
Aug
Sep
Date
Prediction
Median
CI
IQR

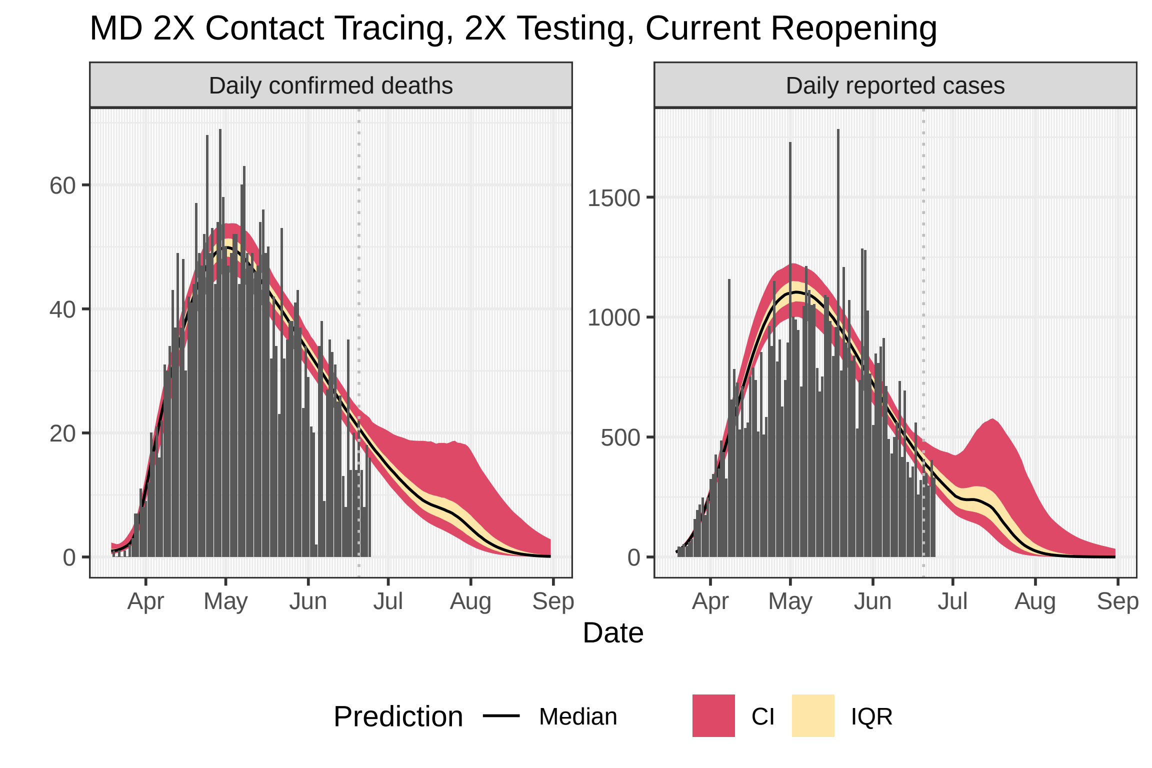
MD 2X Contact Tracing, 2X Testing, Current Reopening
Daily confirmed deaths
Daily reported cases
0
20
40
60
0
500
1000
1500
Apr
May
Jun
Jul
Aug
Sep
Date
Prediction
Median
CI
IQR

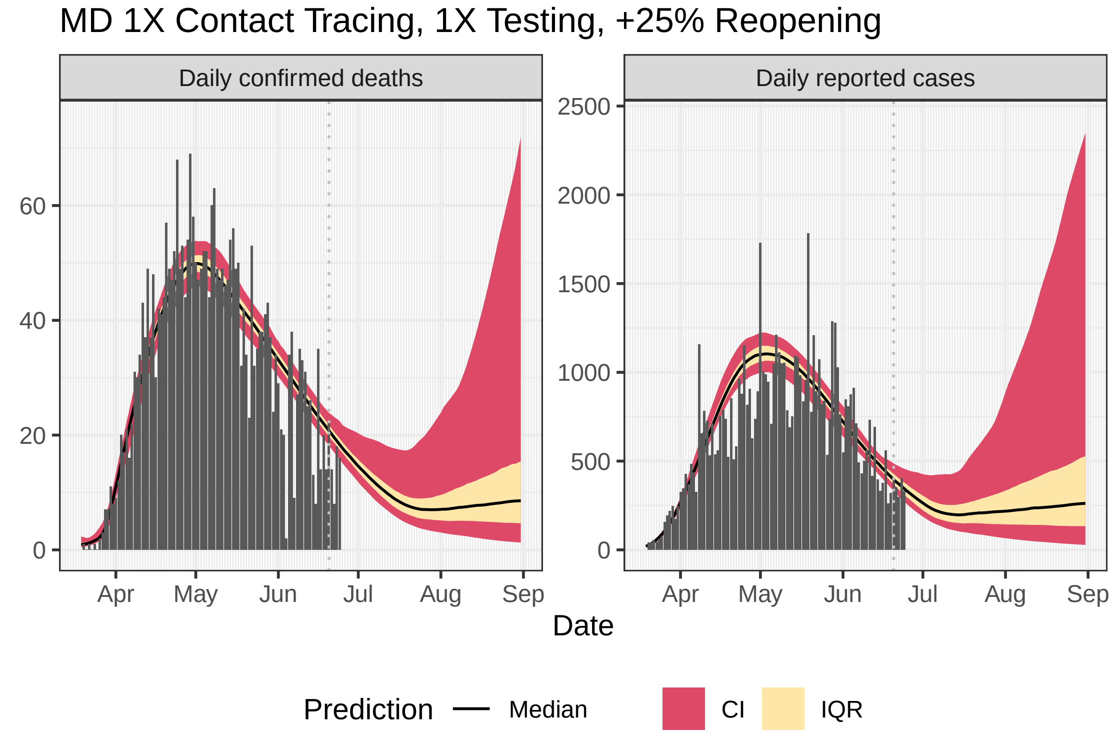

MD 1X Contact Tracing, 1X Testing, +25% Reopening
Daily confirmed deaths
Daily reported cases
0
20
40
60
0
500
1000
1500
2000
2500
Apr
May
Jun
Jul
Aug
Sep
Date
Prediction
Median
CI
IQR

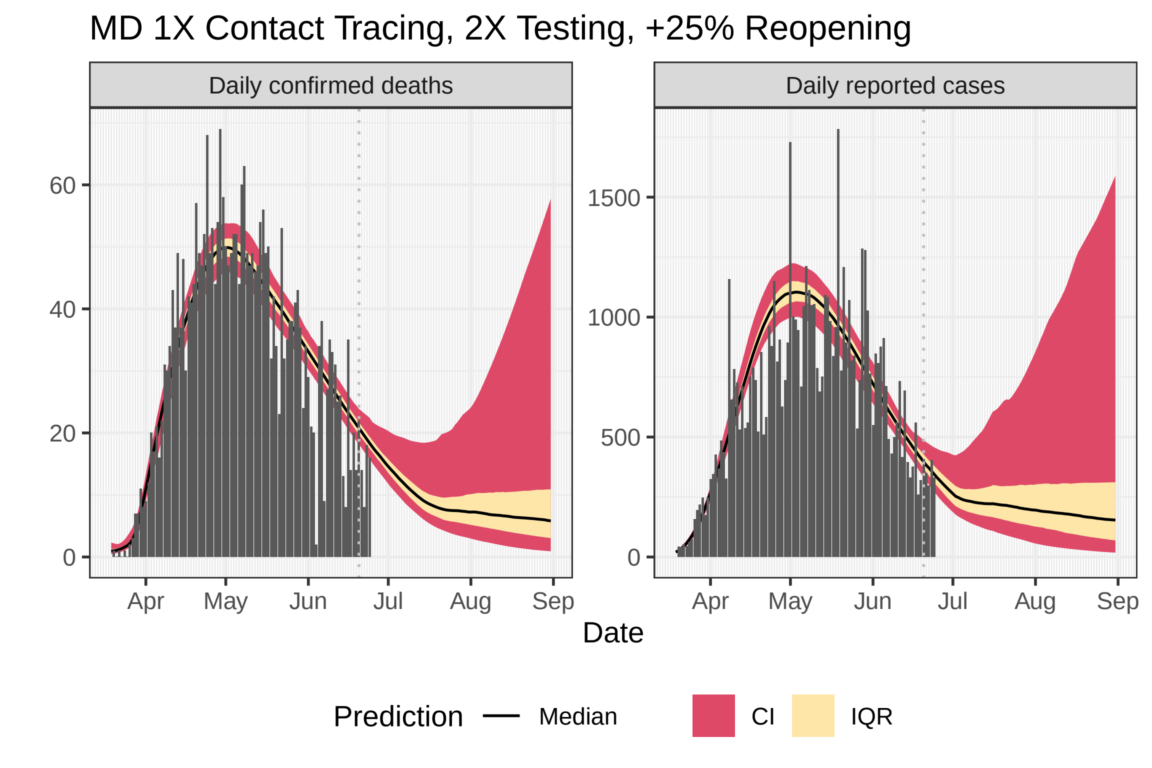

MD 1X Contact Tracing, 2X Testing, +25% Reopening
Daily confirmed deaths
Daily reported cases
0
20
40
60
0
500
1000
1500
Apr
May
Jun
Jul
Aug
Sep
Date
Prediction
Median
CI
IQR

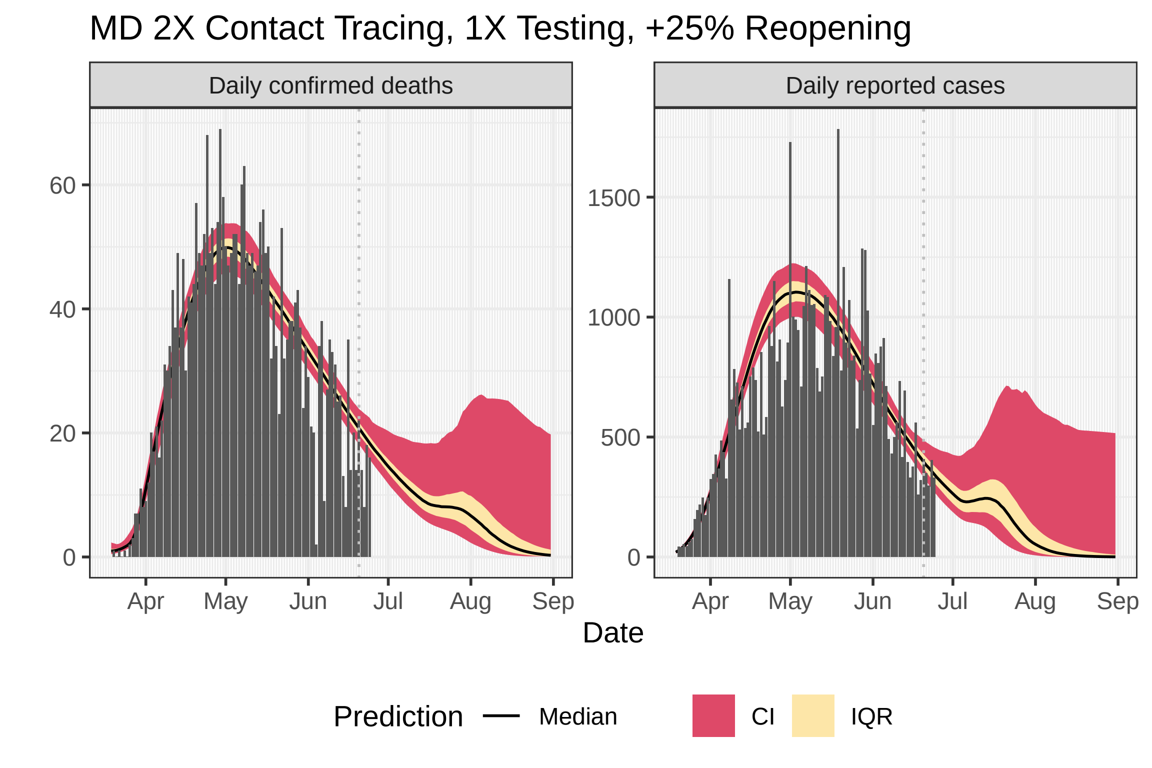

MD 2X Contact Tracing, 1X Testing, +25% Reopening
Daily confirmed deaths
Daily reported cases
60
40
20
0
1500
1000
500
0
Apr
May
Jun
Jul
Aug
Sep
Date
Prediction
Median
CI
IQR

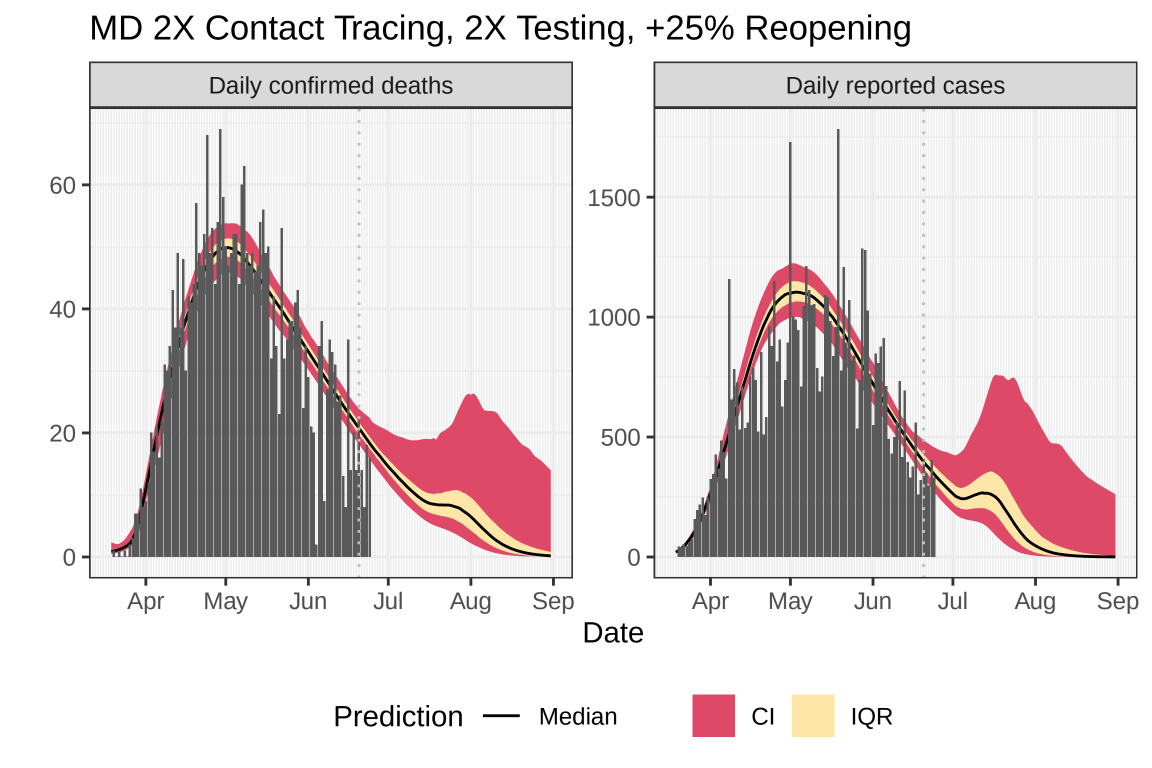

MD 2X Contact Tracing, 2X Testing, +25% Reopening
Daily confirmed deaths
Daily reported cases
60
40
20
0
1500
1000
500
0
Apr
May
Jun
Jul
Aug
Sep
Date
Prediction
Median
CI
IQR

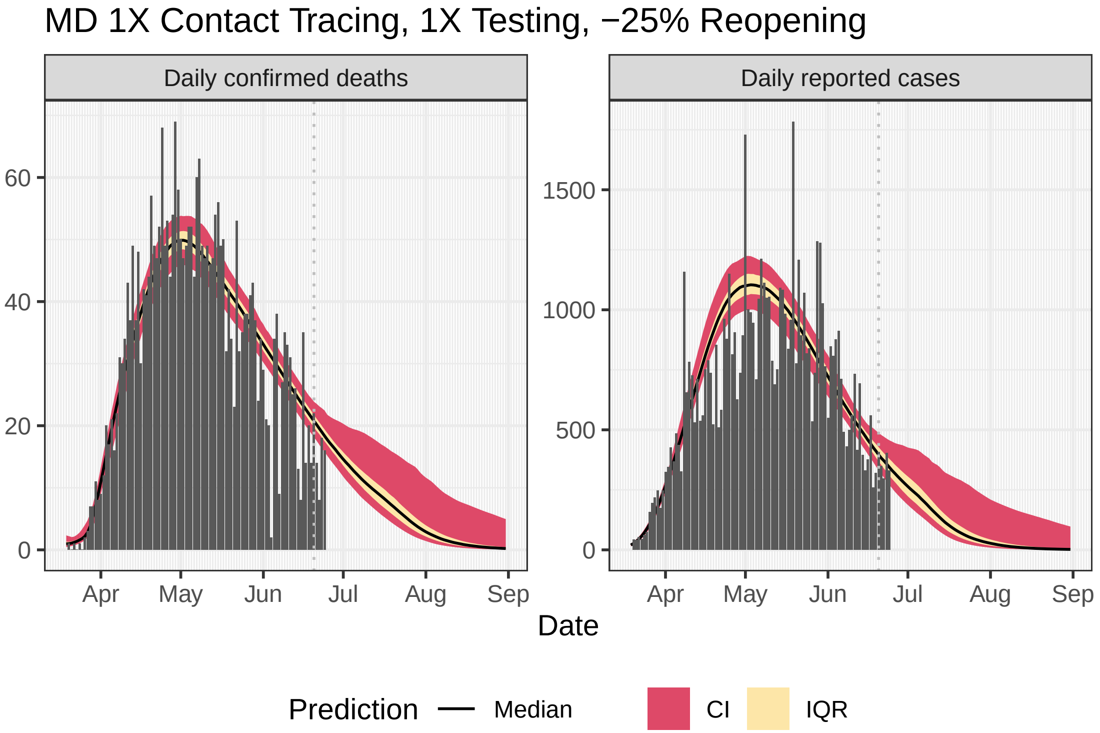

MD 1X Contact Tracing, 1X Testing, −25% Reopening
Daily confirmed deaths
Daily reported cases
60
40
20
0
1500
1000
500
0
Apr
May
Jun
Jul
Aug
Sep
Date
Prediction
Median
CI
IQR

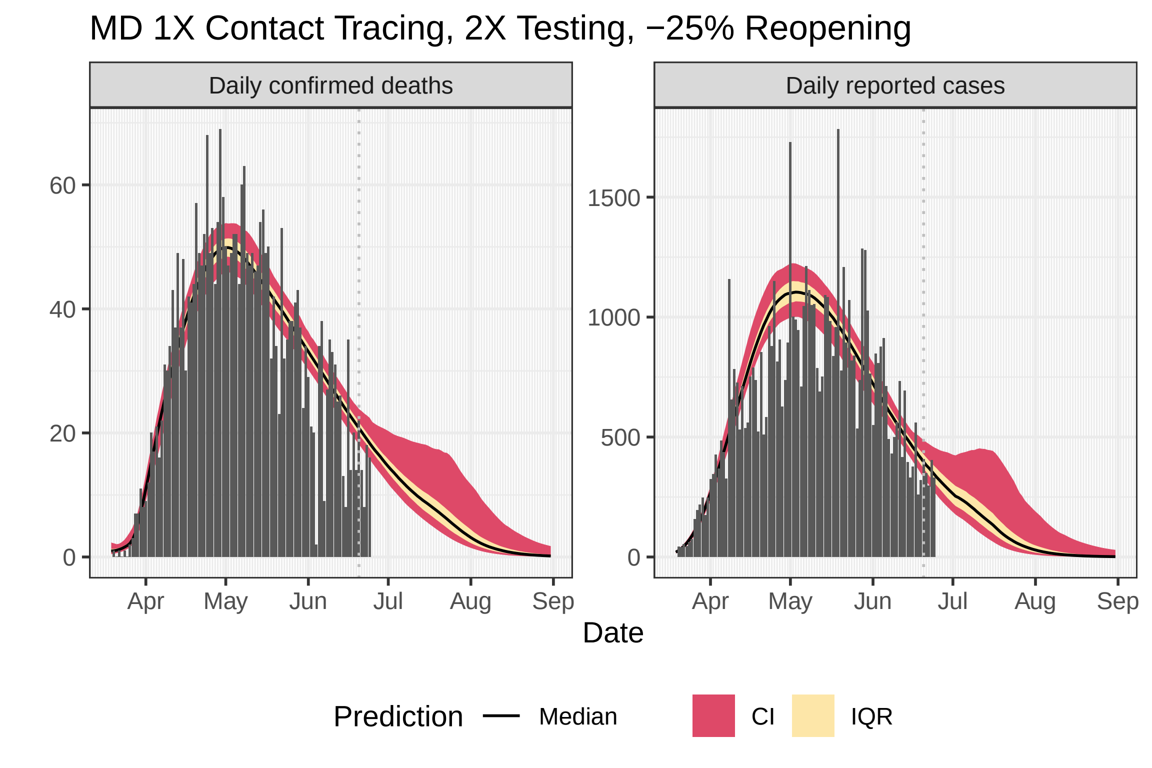

MD 1X Contact Tracing, 2X Testing, −25% Reopening
Daily confirmed deaths
Daily reported cases
60
40
20
0
1500
1000
500
0
Apr
May
Jun
Jul
Aug
Sep
Date
Prediction
Median
CI
IQR

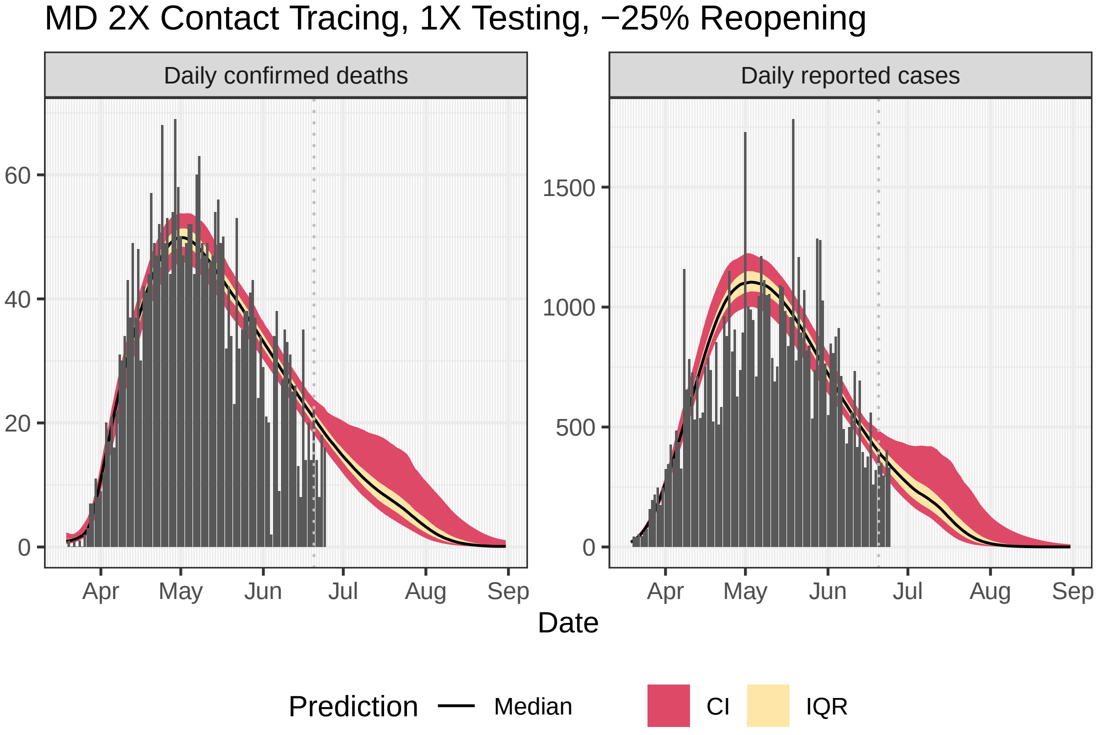

MD 2X Contact Tracing, 1X Testing, −25% Reopening
Daily confirmed deaths
Daily reported cases
0
20
40
60
0
500
1000
1500
Apr
May
Jun
Jul
Aug
Sep
Date
Prediction
Median
CI
IQR

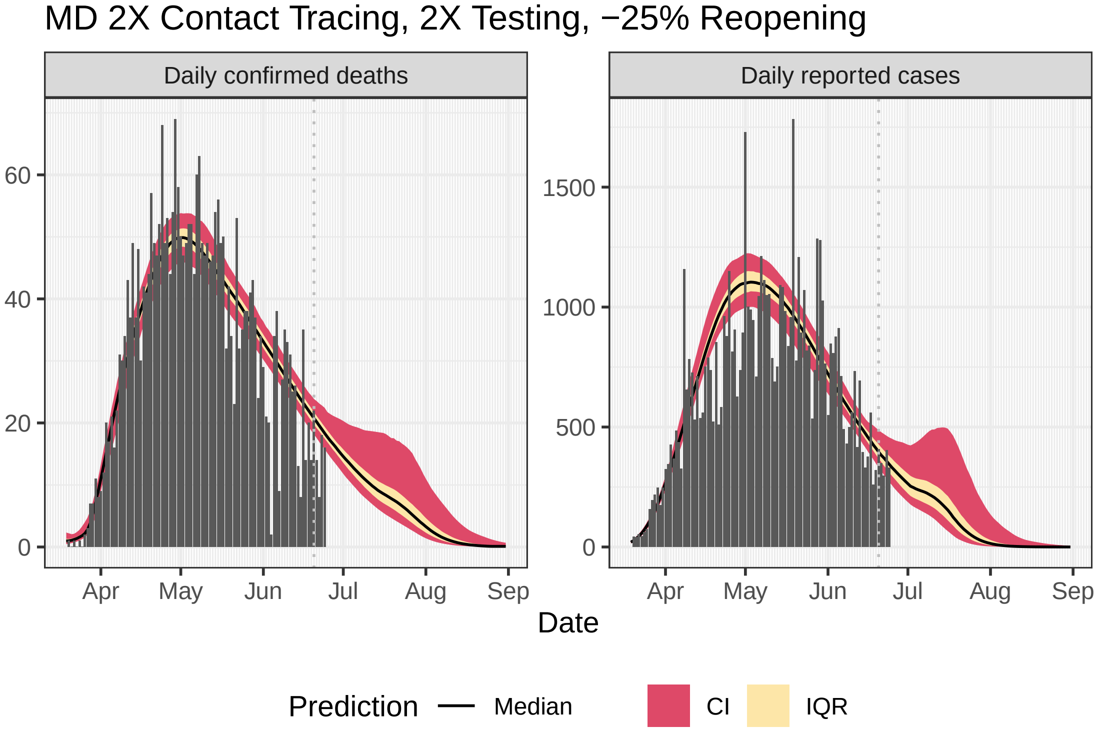

MD 2X Contact Tracing, 2X Testing, −25% Reopening
Daily confirmed deaths
Daily reported cases
0
20
40
60
0
500
1000
1500
Apr
May
Jun
Jul
Aug
Sep
Date
Prediction
Median
CI
IQR

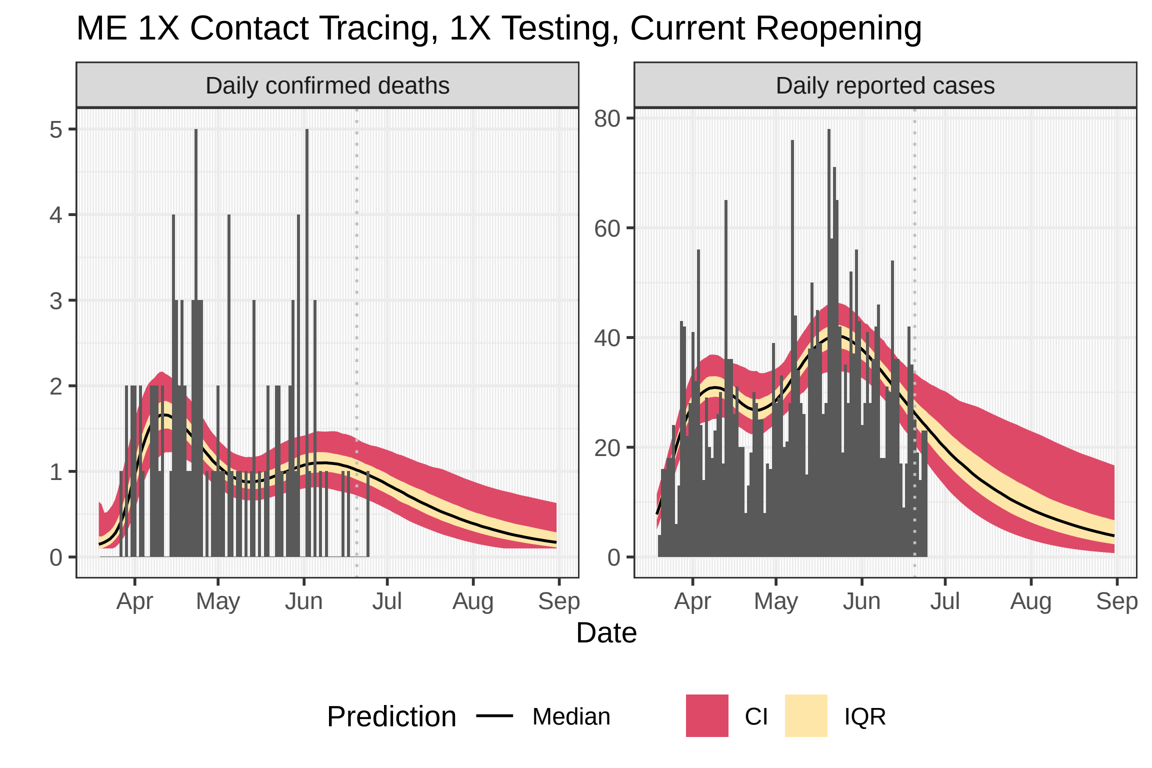
ME 1X Contact Tracing, 1X Testing, Current Reopening
Daily confirmed deaths
Daily reported cases
0
1
2
3
4
5
0
20
40
60
80
Apr
May
Jun
Jul
Aug
Sep
Date
Prediction
Median
CI
IQR

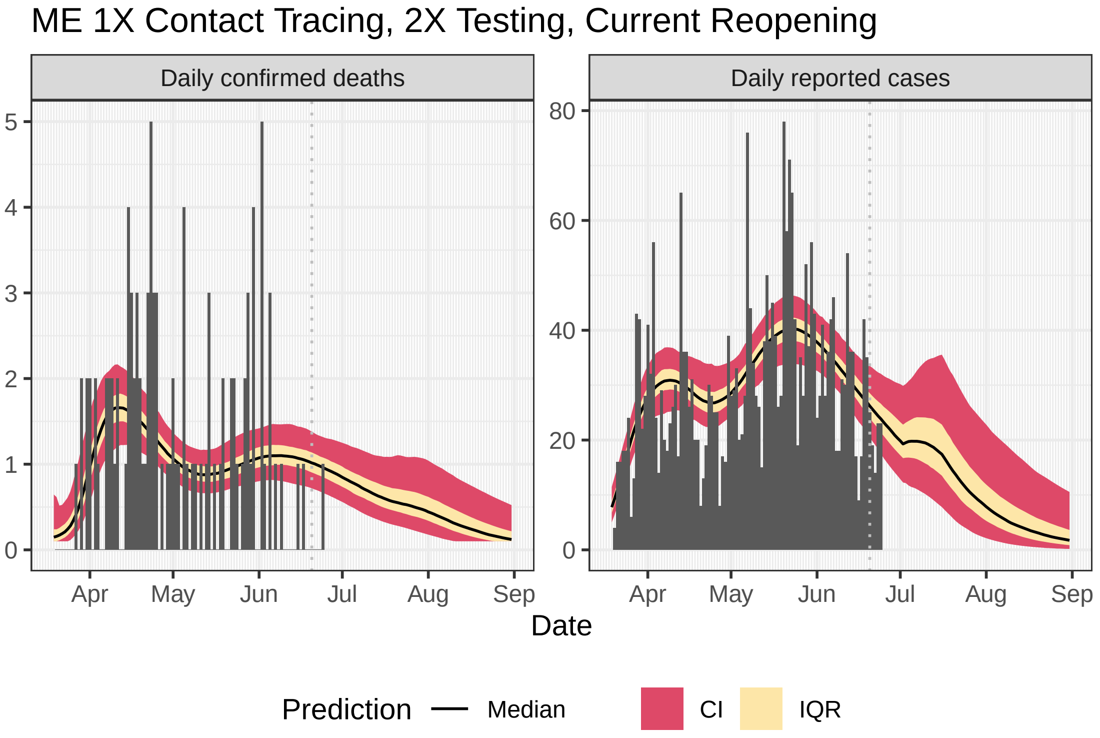

ME 1X Contact Tracing, 2X Testing, Current Reopening
Daily confirmed deaths
Daily reported cases
5
4
3
2
1
0
80
60
40
20
0
Apr
May
Jun
Jul
Aug
Sep
Date
Prediction
Median
CI
IQR

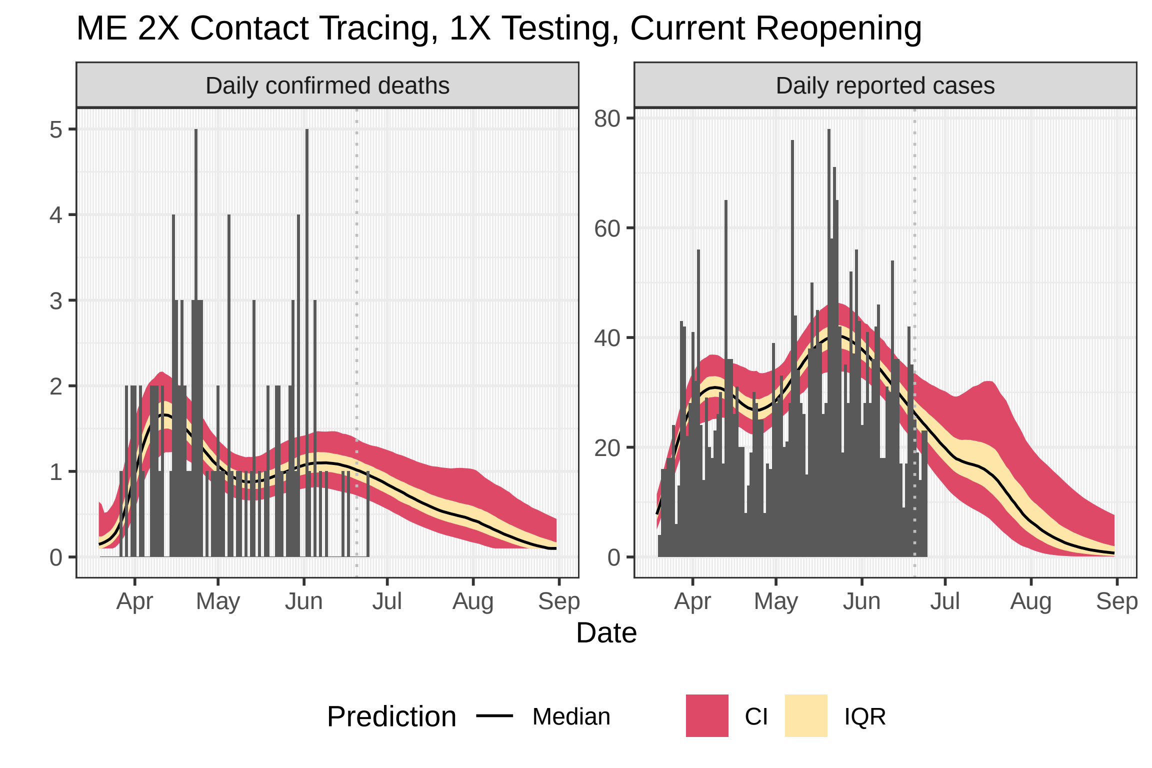

ME 2X Contact Tracing, 1X Testing, Current Reopening
Daily confirmed deaths
Daily reported cases
0
1
2
3
4
5
0
20
40
60
80
Apr
May
Jun
Jul
Aug
Sep
Date
Prediction
Median
CI
IQR

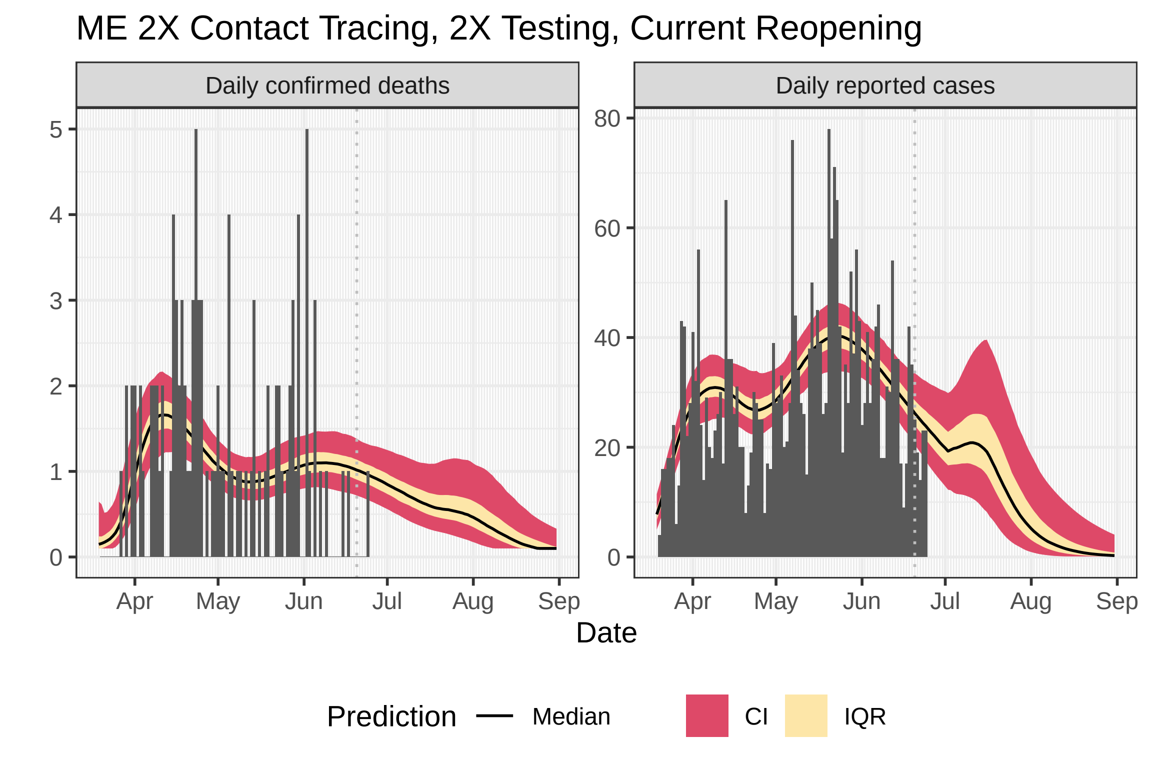

ME 2X Contact Tracing, 2X Testing, Current Reopening
Daily confirmed deaths
Daily reported cases
0
1
2
3
4
5
0
20
40
60
80
Apr
May
Jun
Jul
Aug
Sep
Date
Prediction
Median
CI
IQR

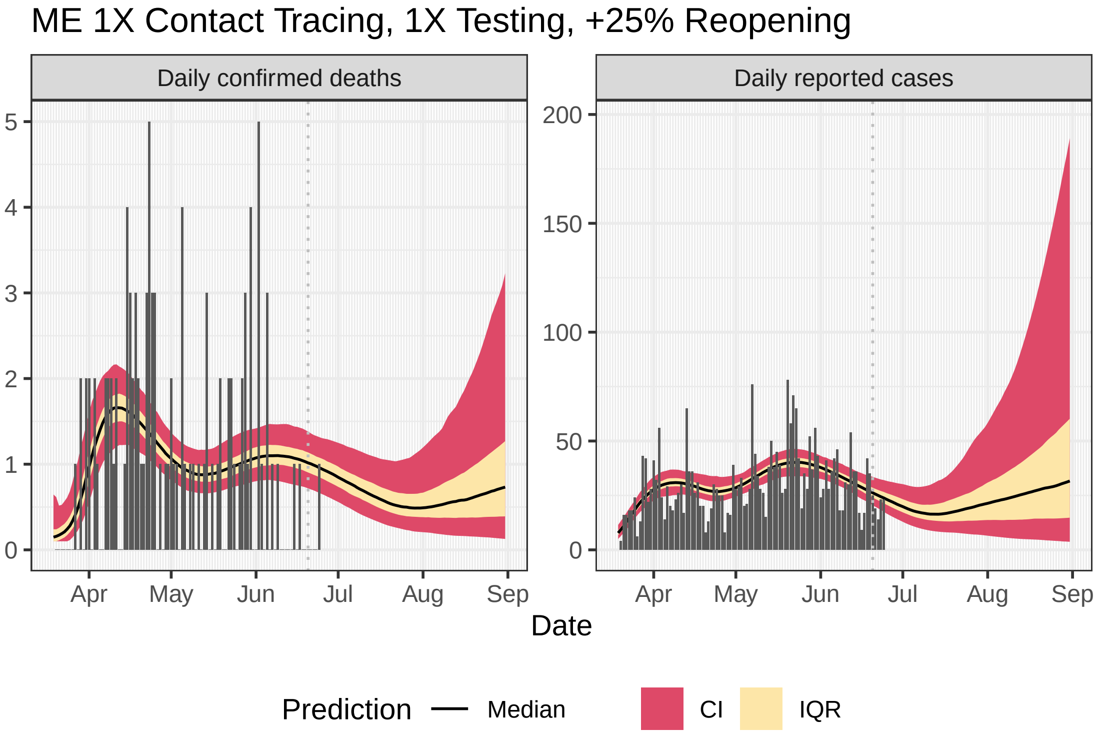
ME 1X Contact Tracing, 1X Testing, +25% Reopening
Daily confirmed deaths
Daily reported cases
0
1
2
3
4
5
0
50
100
150
200
Apr
May
Jun
Jul
Aug
Sep
Date
Prediction
Median
CI
IQR

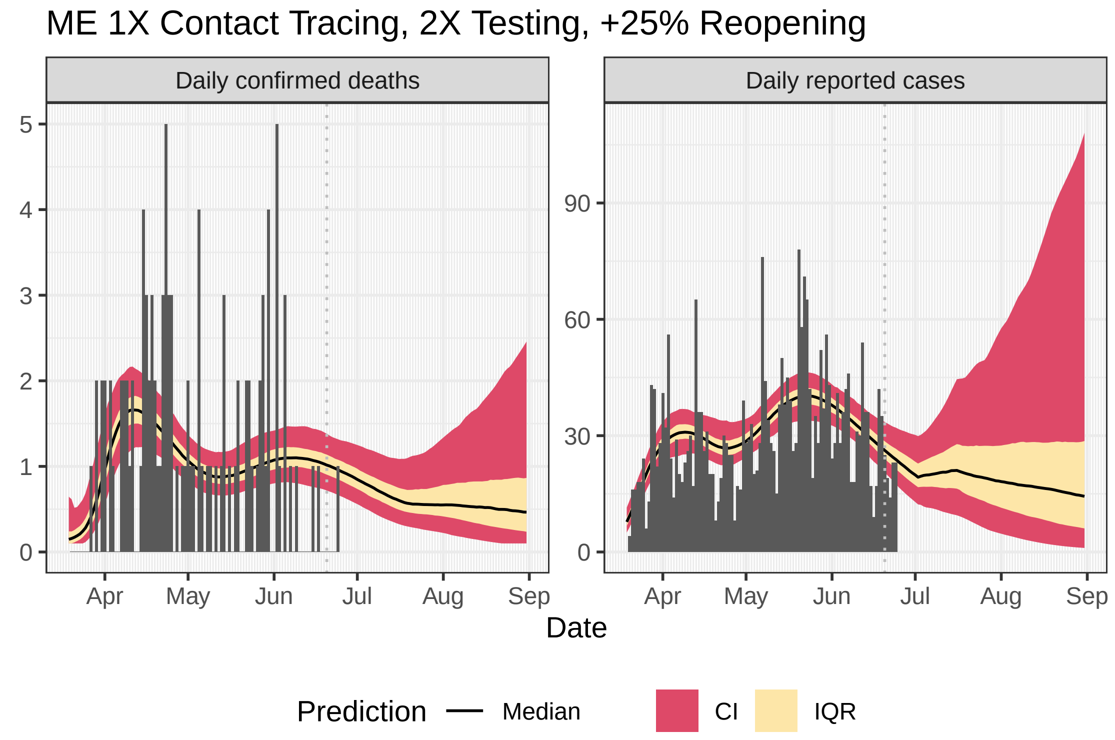

ME 1X Contact Tracing, 2X Testing, +25% Reopening
Daily confirmed deaths
Daily reported cases
5
4
3
2
1
0
90
60
30
0
Apr
May
Jun
Jul
Aug
Sep
Date
Prediction
Median
CI
IQR

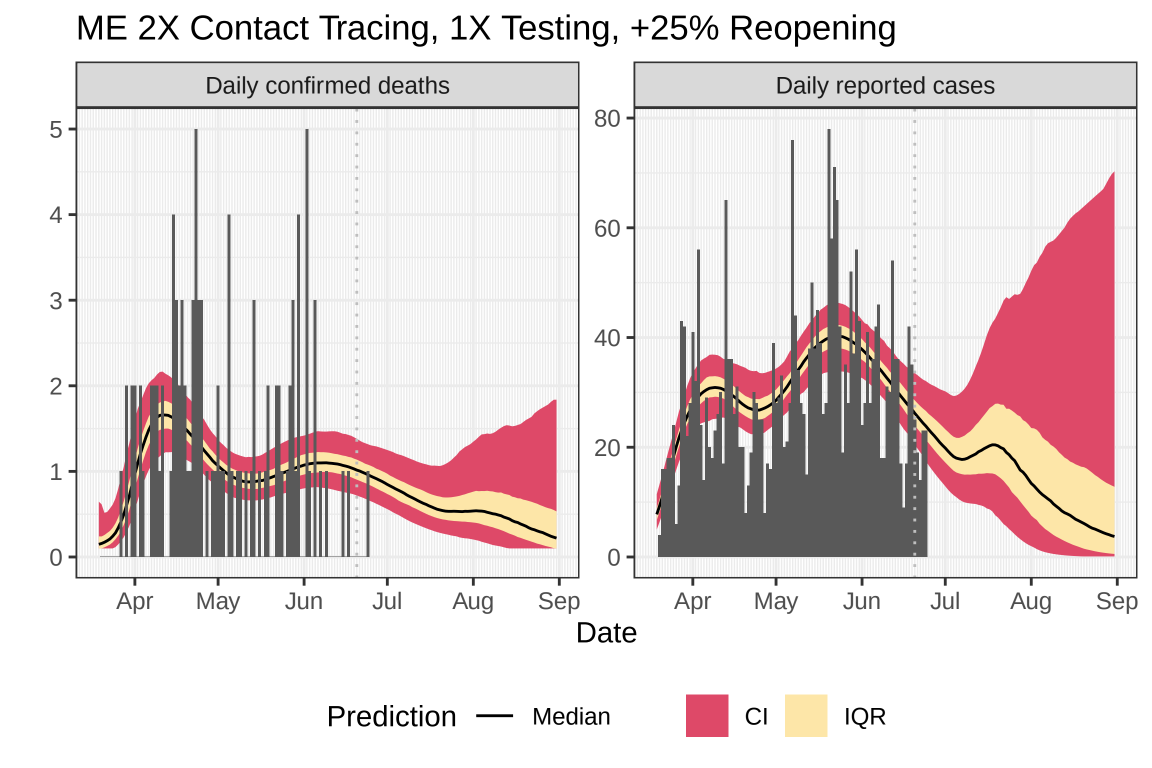
ME 2X Contact Tracing, 1X Testing, +25% Reopening
Daily confirmed deaths
Daily reported cases
0
1
2
3
4
5
0
20
40
60
80
Apr
May
Jun
Jul
Aug
Sep
Date
Prediction
Median
CI
IQR

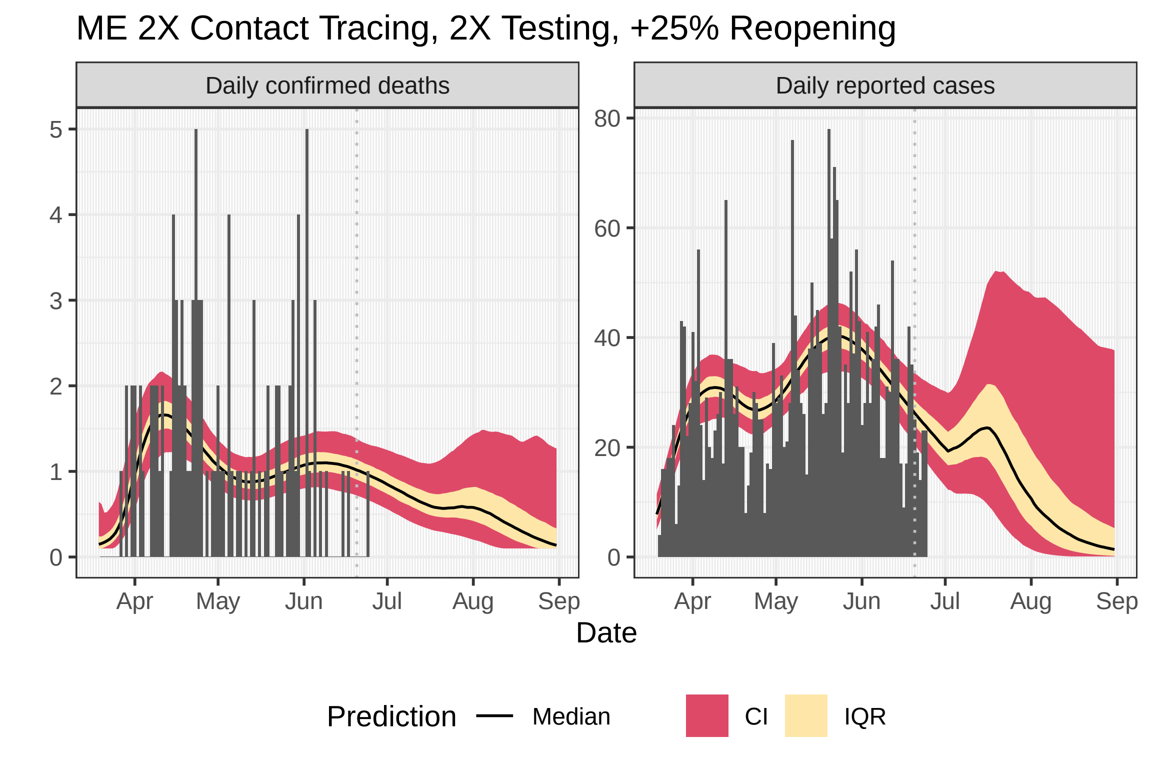

ME 2X Contact Tracing, 2X Testing, +25% Reopening
Daily confirmed deaths
Daily reported cases
0
1
2
3
4
5
0
20
40
60
80
Apr
May
Jun
Jul
Aug
Sep
Date
Prediction
Median
CI
IQR

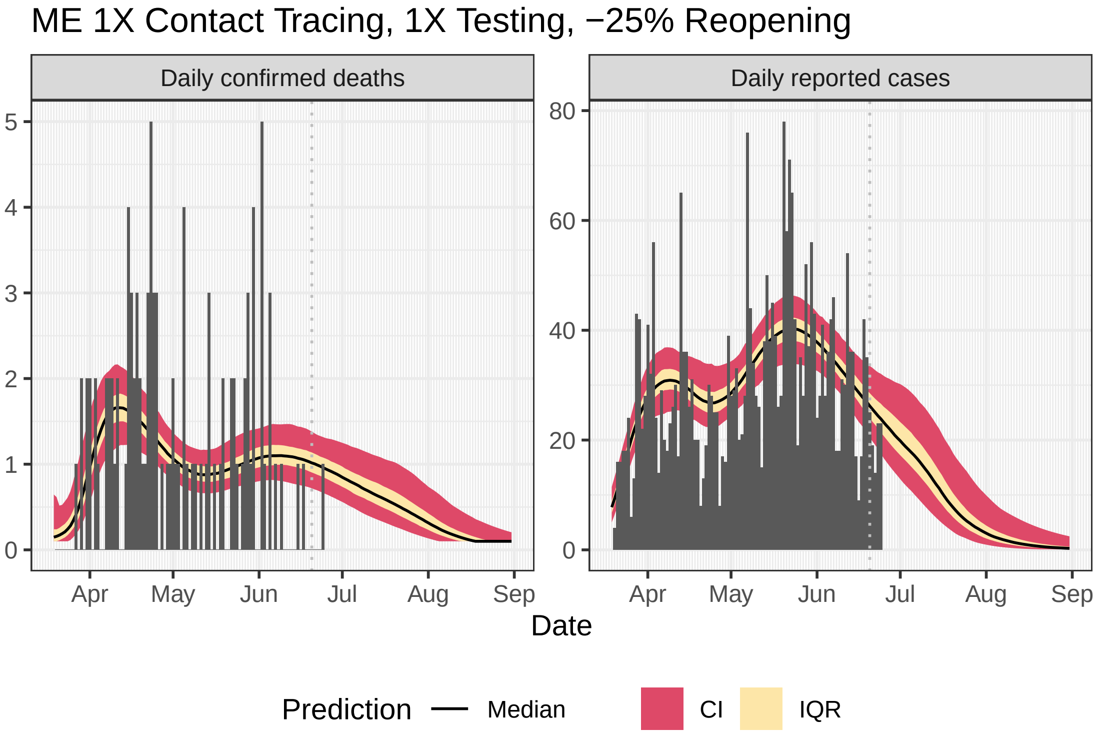
ME 1X Contact Tracing, 1X Testing, −25% Reopening
Daily confirmed deaths
Daily reported cases
0
1
2
3
4
5
0
20
40
60
80
Apr
May
Jun
Jul
Aug
Sep
Date
Prediction
Median
CI
IQR

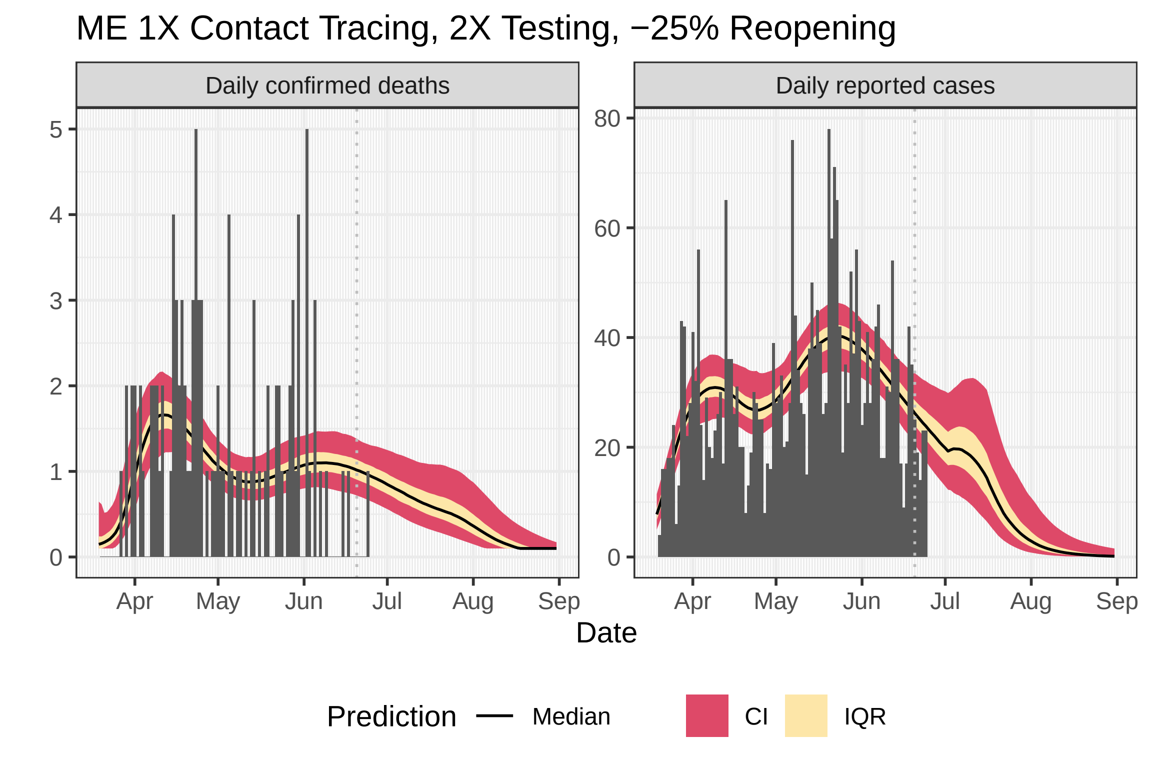

ME 1X Contact Tracing, 2X Testing, −25% Reopening
Daily confirmed deaths
Daily reported cases
0
1
2
3
4
5
0
20
40
60
80
Apr
May
Jun
Jul
Aug
Sep
Date
Prediction
Median
CI
IQR

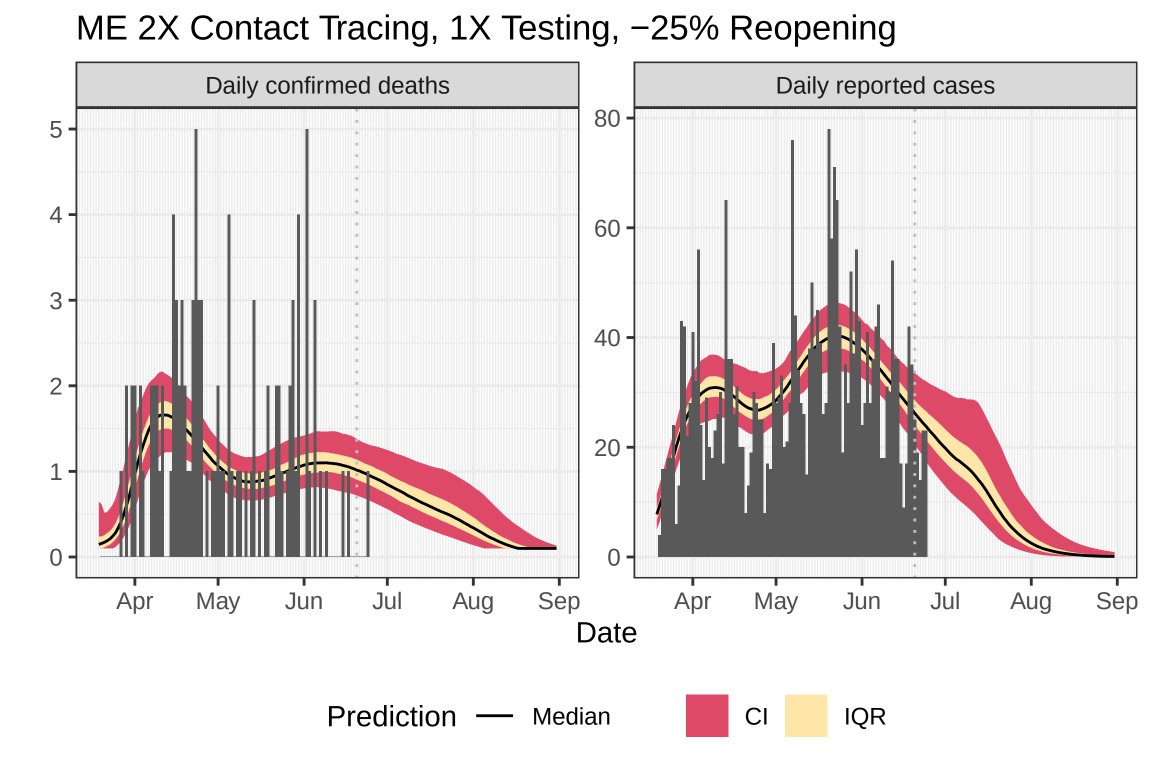

ME 2X Contact Tracing, 1X Testing, −25% Reopening
Daily confirmed deaths
Daily reported cases
5
4
3
2
1
0
80
60
40
20
0
Apr
May
Jun
Jul
Aug
Sep
Date
Prediction
Median
CI
IQR

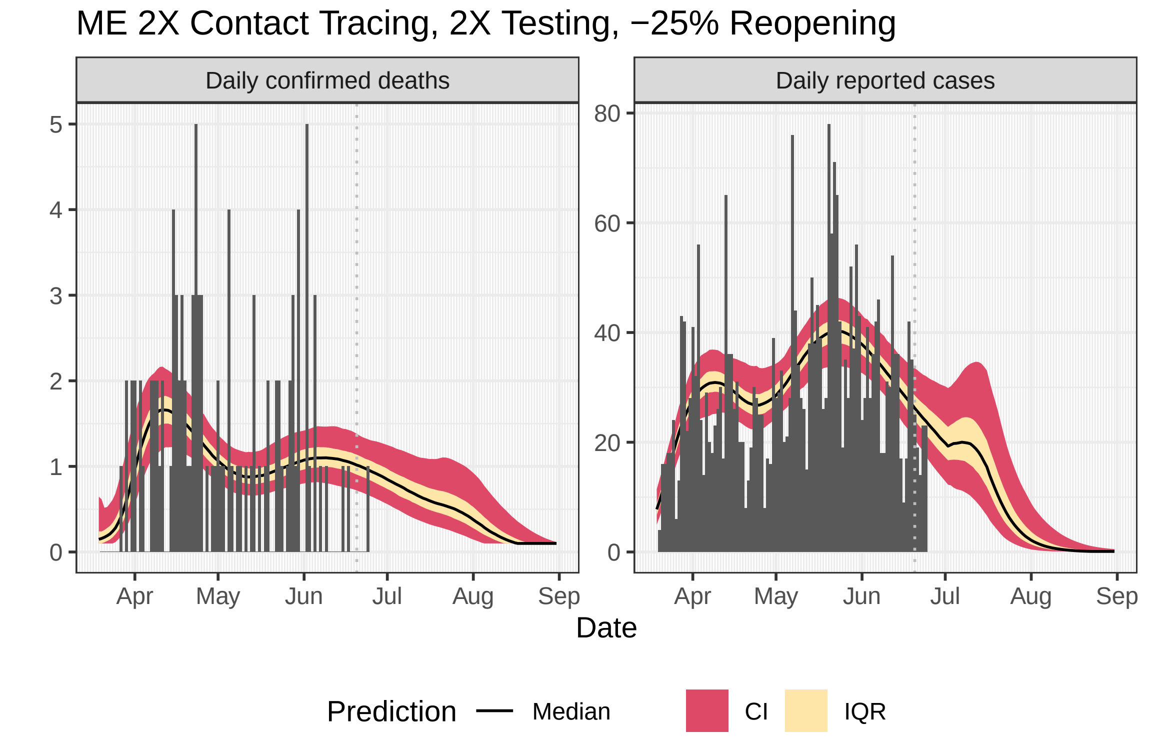
ME 2X Contact Tracing, 2X Testing, −25% Reopening
Daily confirmed deaths
Daily reported cases
0
1
2
3
4
5
0
20
40
60
80
Apr
May
Jun
Jul
Aug
Sep
Date
Prediction
Median
CI
IQR

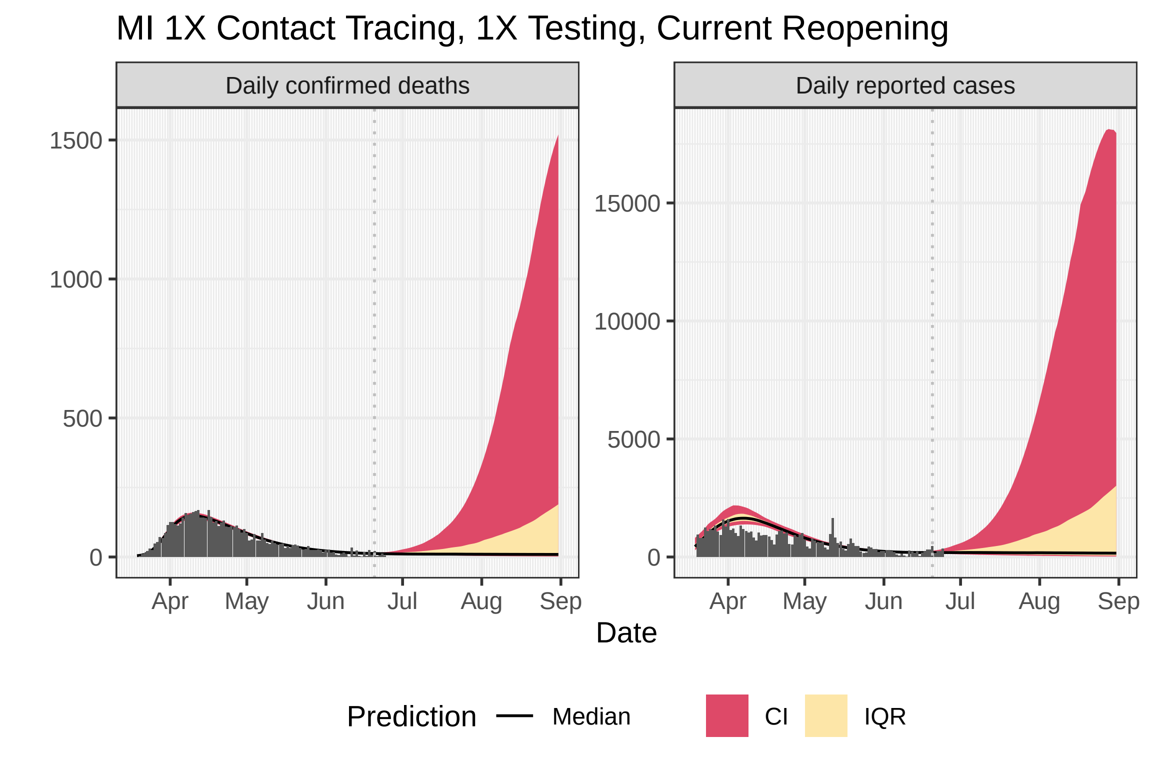

MI 1X Contact Tracing, 1X Testing, Current Reopening
Daily confirmed deaths
Daily reported cases
1500
1000
500
0
15000
10000
5000
0
Apr
May
Jun
Jul
Aug
Sep
Date
Prediction
Median
CI
IQR

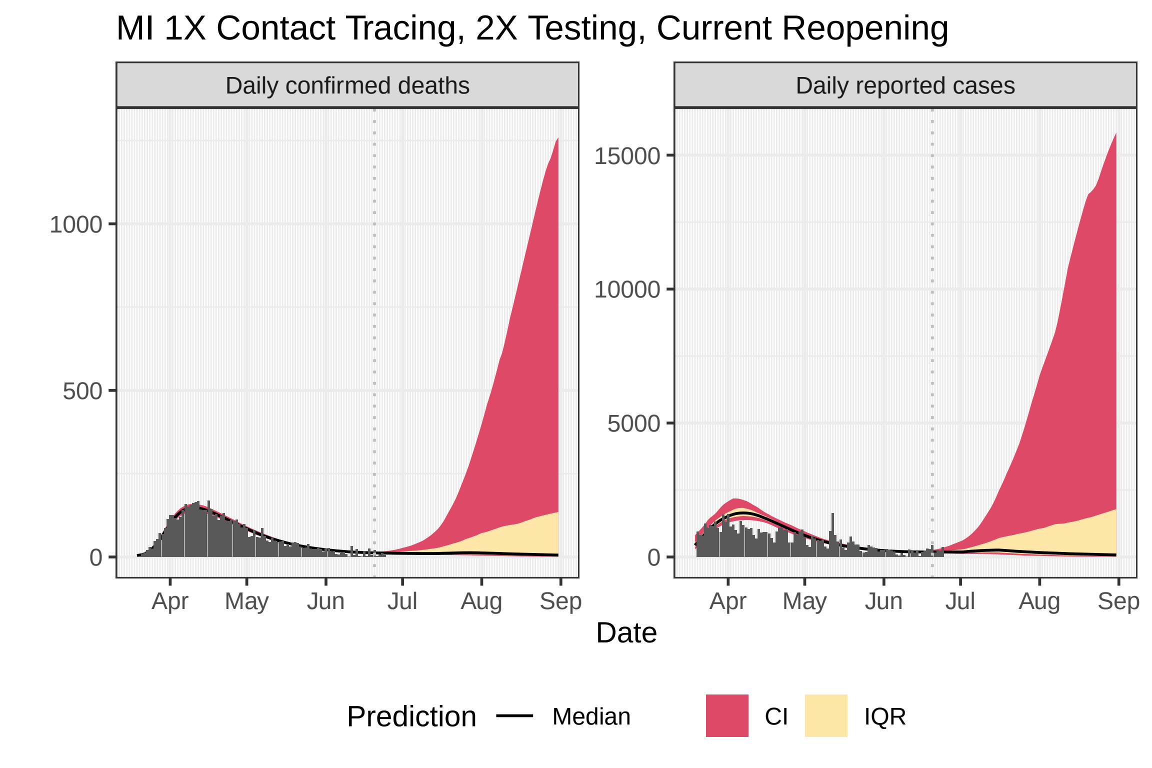
MI 1X Contact Tracing, 2X Testing, Current Reopening
Daily confirmed deaths
Daily reported cases
1000
500
0
15000
10000
5000
0
Apr
May
Jun
Jul
Aug
Sep
Date
Prediction
Median
CI
IQR

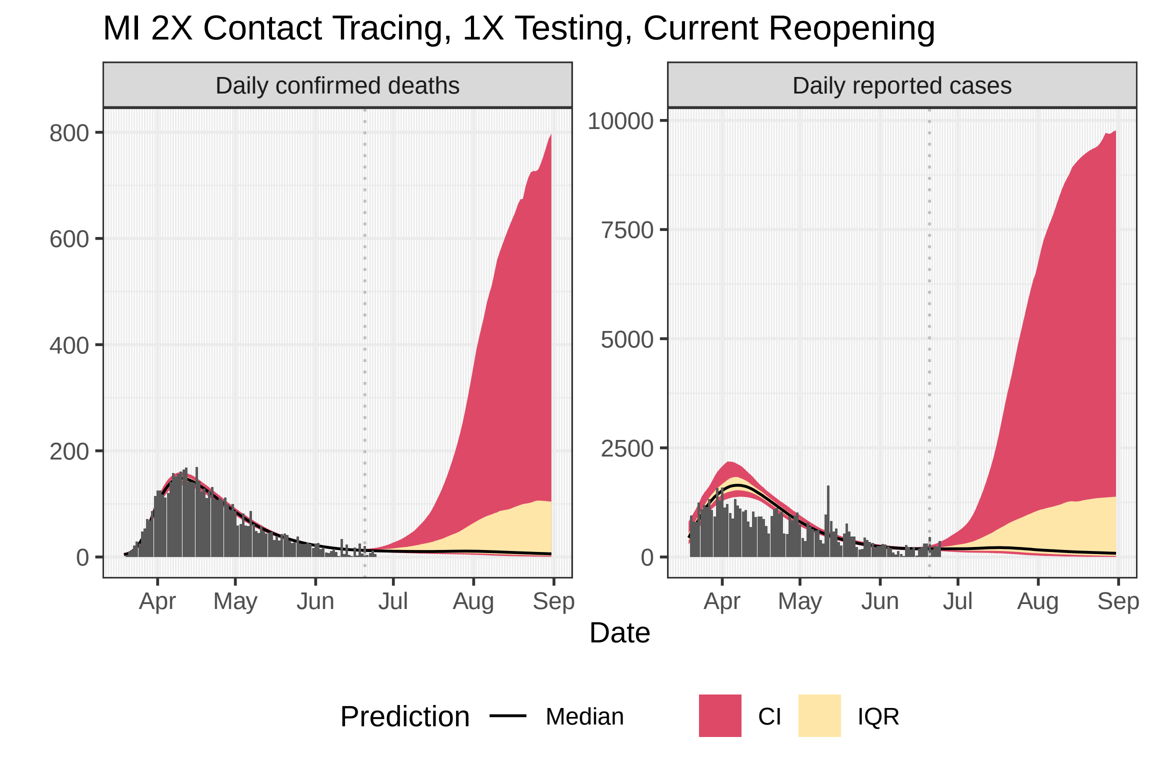

MI 2X Contact Tracing, 1X Testing, Current Reopening
Daily confirmed deaths
Daily reported cases
800
600
400
200
0
10000
7500
5000
2500
0
Apr
May
Jun
Jul
Aug
Sep
Date
Prediction
Median
CI
IQR

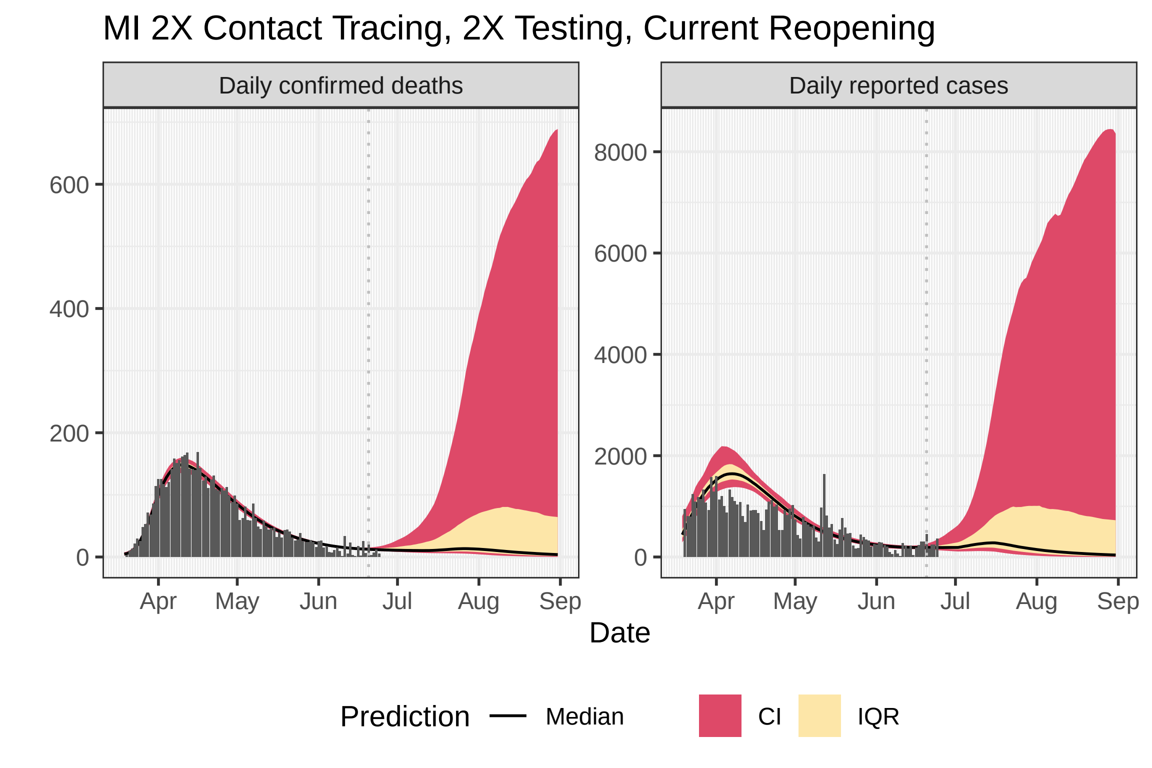

MI 2X Contact Tracing, 2X Testing, Current Reopening
Daily confirmed deaths
Daily reported cases
600
400
200
0
8000
6000
4000
2000
0
Apr
May
Jun
Jul
Aug
Sep
Date
Prediction
Median
CI
IQR

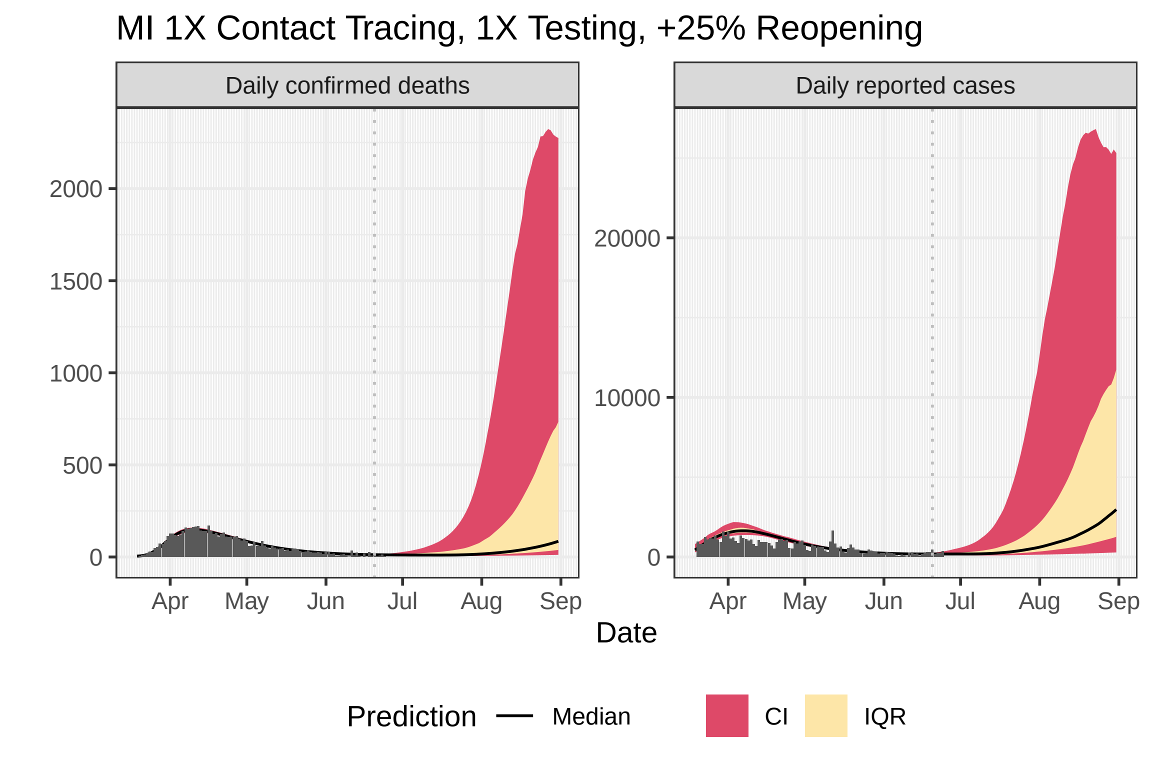
MI 1X Contact Tracing, 1X Testing, +25% Reopening
Daily confirmed deaths
Daily reported cases
2000
1500
1000
500
0
20000
10000
0
Apr
May
Jun
Jul
Aug
Sep
Date
Prediction
Median
CI
IQR

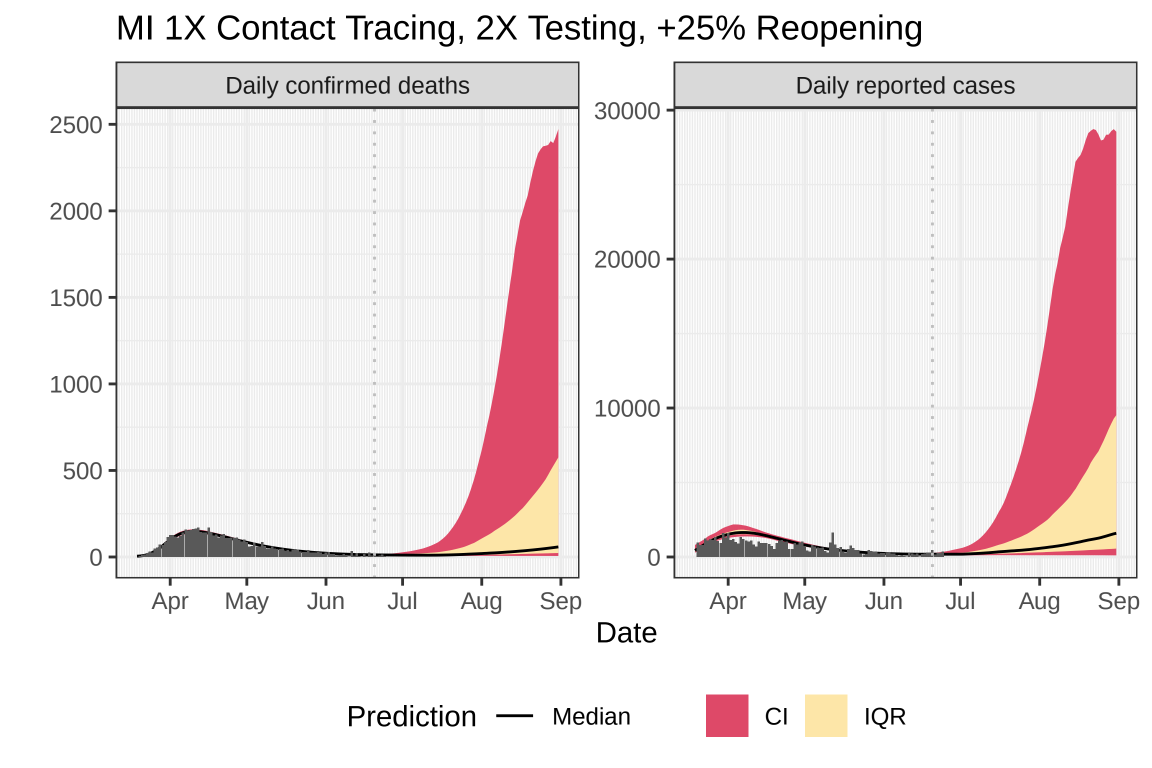

MI 1X Contact Tracing, 2X Testing, +25% Reopening
Daily confirmed deaths
Daily reported cases
2500
2000
1500
1000
500
0
30000
20000
10000
0
Apr
May
Jun
Jul
Aug
Sep
Date
Prediction
Median
CI
IQR

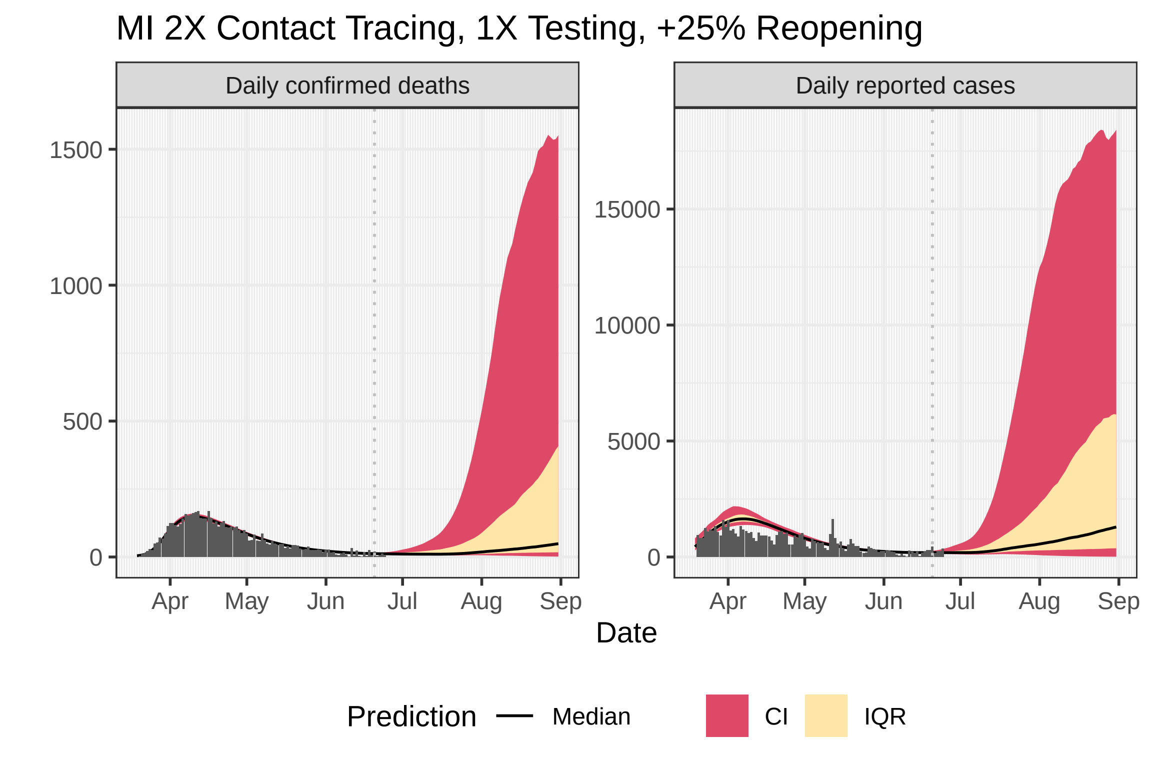
MI 2X Contact Tracing, 1X Testing, +25% Reopening
Daily confirmed deaths
Daily reported cases
1500
1000
500
0
15000
10000
5000
0
Apr
May
Jun
Jul
Aug
Sep
Date
Prediction
Median
CI
IQR

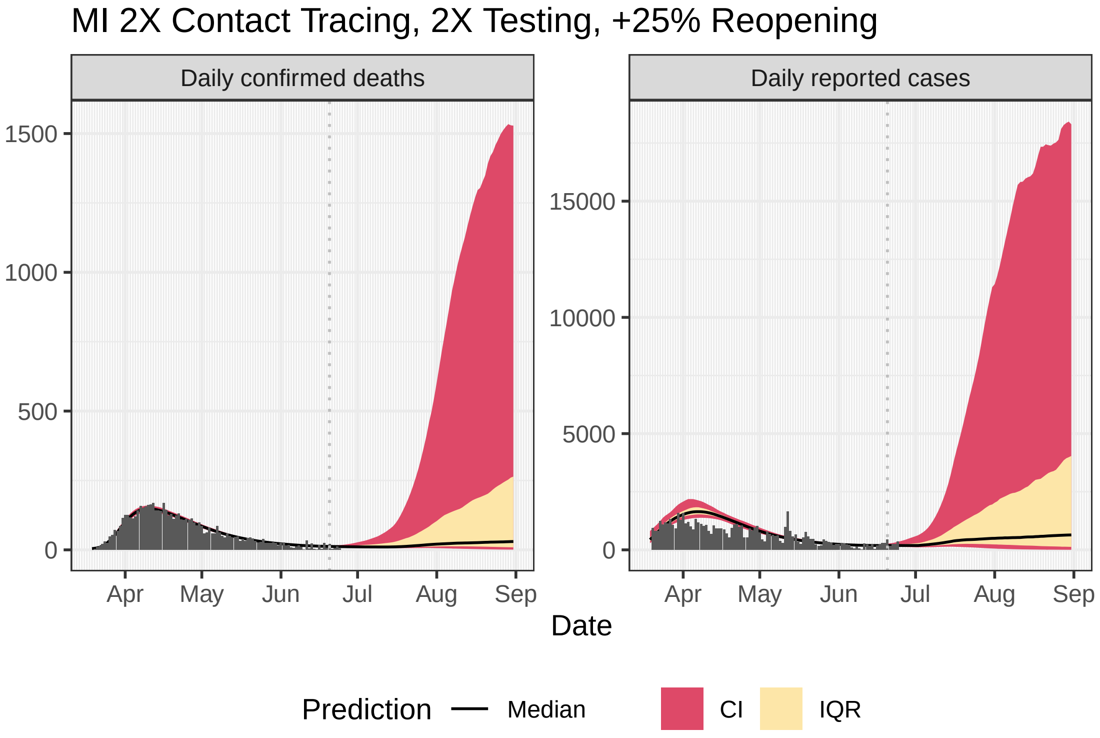
MI 2X Contact Tracing, 2X Testing, +25% Reopening
Daily confirmed deaths
Daily reported cases
1500
1000
500
0
15000
10000
5000
0
Apr
May
Jun
Jul
Aug
Sep
Date
Prediction
Median
CI
IQR

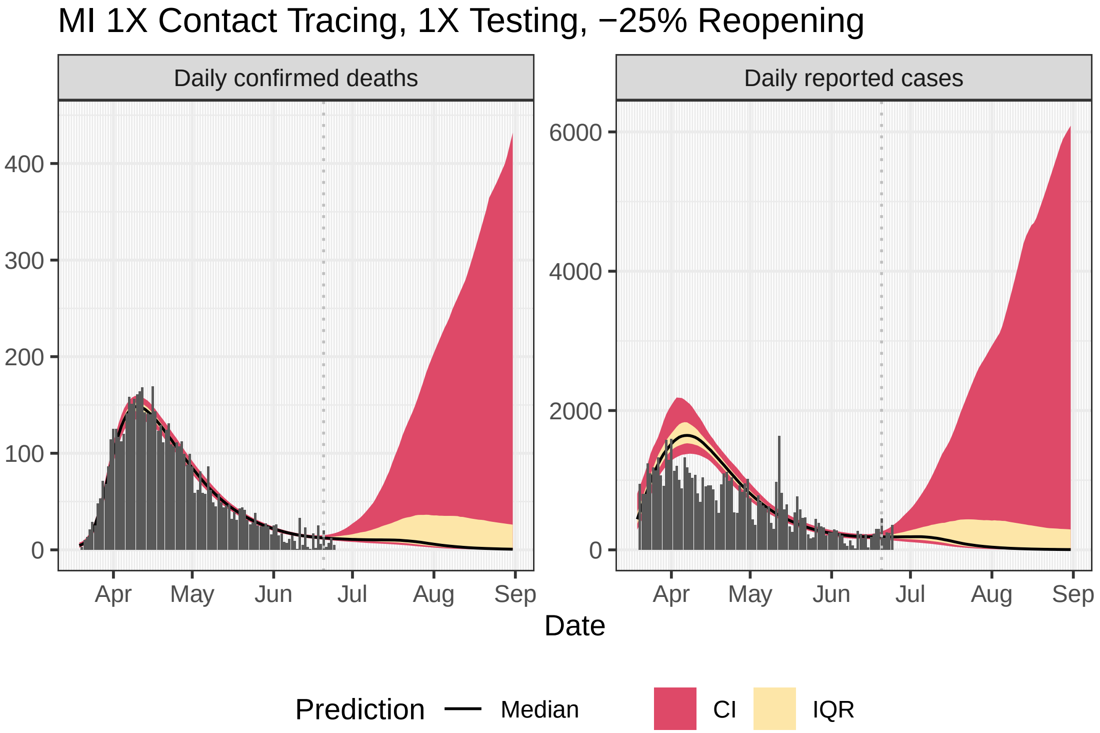

MI 1X Contact Tracing, 1X Testing, −25% Reopening
Daily confirmed deaths
Daily reported cases
400
300
200
100
0
6000
4000
2000
0
Apr
May
Jun
Jul
Aug
Sep
Date
Prediction
Median
CI
IQR

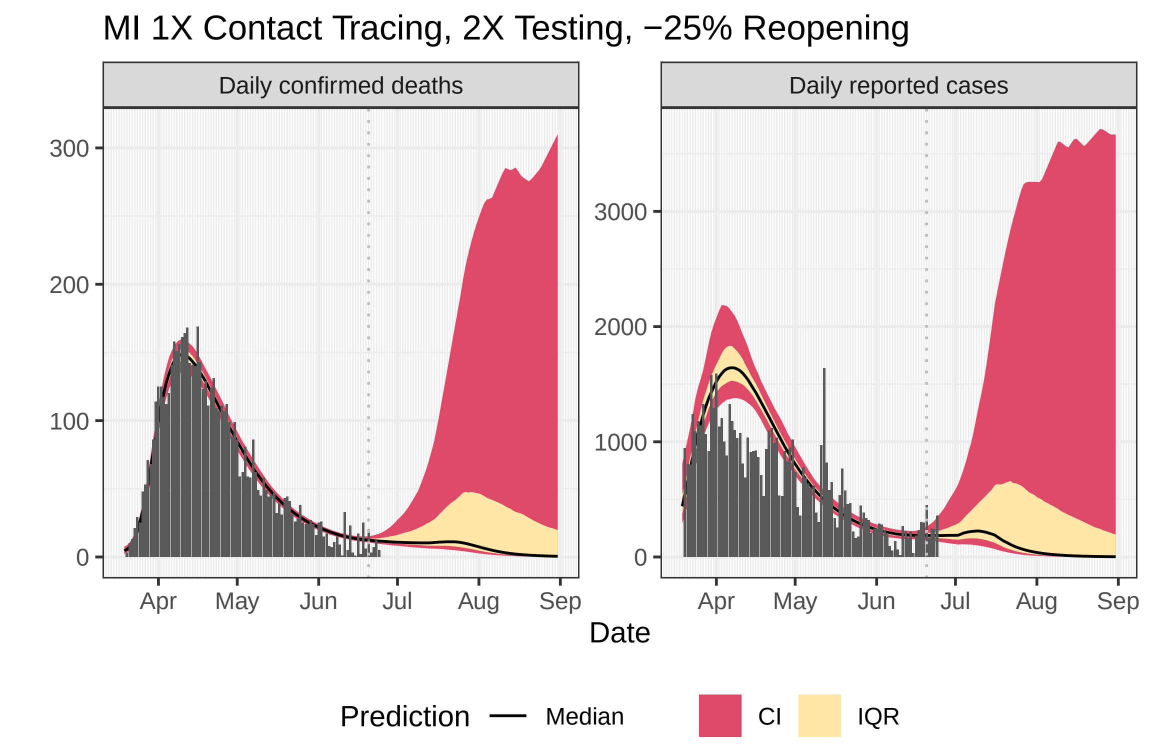

MI 1X Contact Tracing, 2X Testing, −25% Reopening
Daily confirmed deaths
Daily reported cases
300
200
100
0
3000
2000
1000
0
Apr
May
Jun
Jul
Aug
Sep
Date
Prediction
Median
CI
IQR

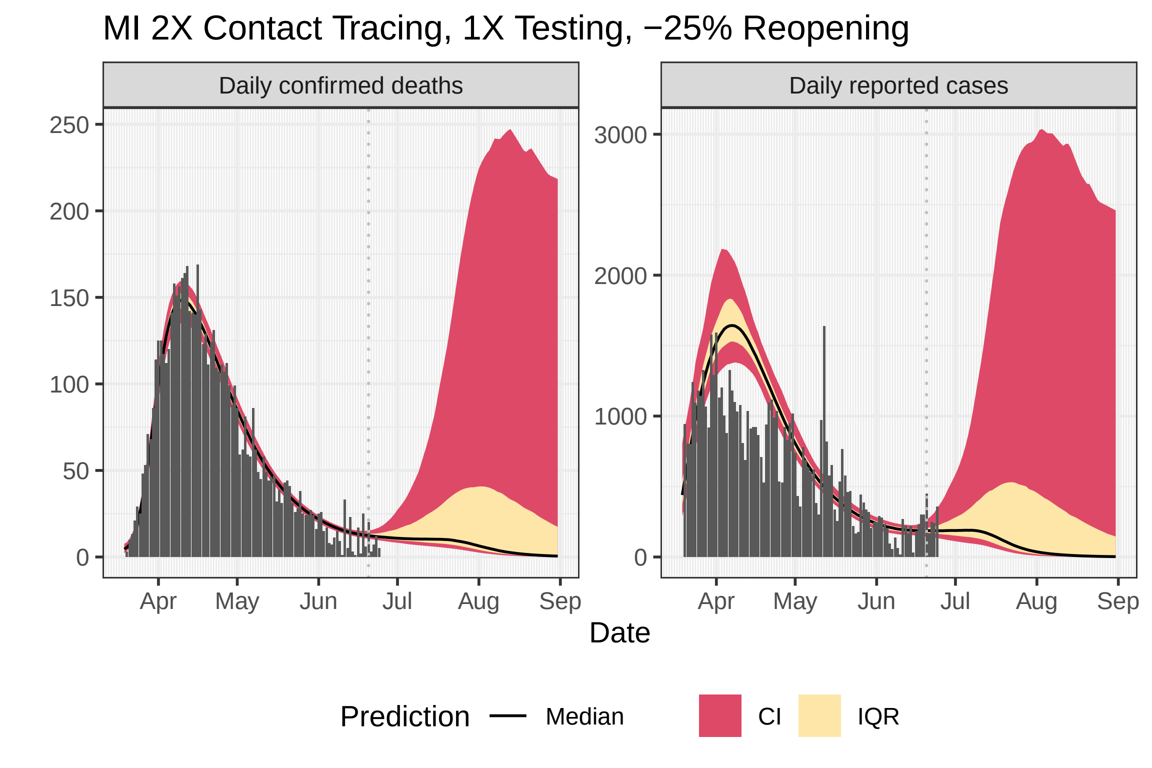
MI 2X Contact Tracing, 1X Testing, −25% Reopening
Daily confirmed deaths
Daily reported cases
250
200
150
100
50
0
3000
2000
1000
0
Apr
May
Jun
Jul
Aug
Sep
Date
Prediction
Median
CI
IQR

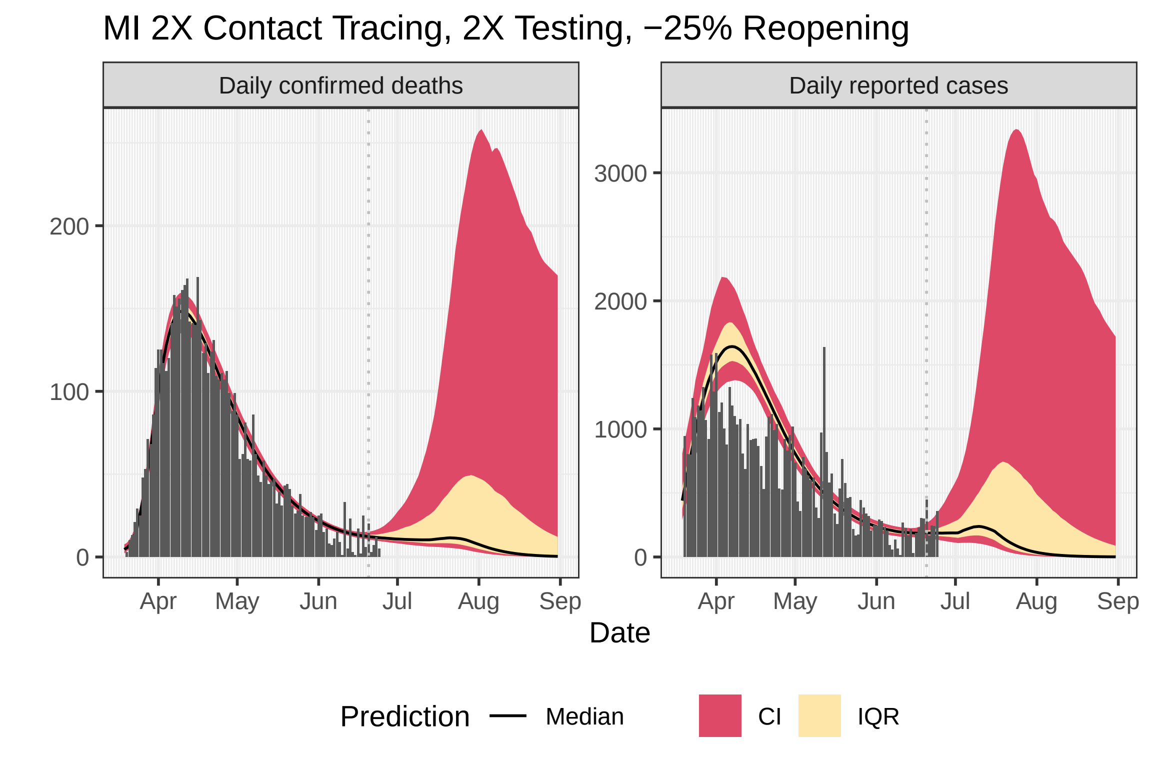

MI 2X Contact Tracing, 2X Testing, −25% Reopening
Daily confirmed deaths
Daily reported cases
200
100
0
3000
2000
1000
0
Apr
May
Jun
Jul
Aug
Sep
Date
Prediction
Median
CI
IQR

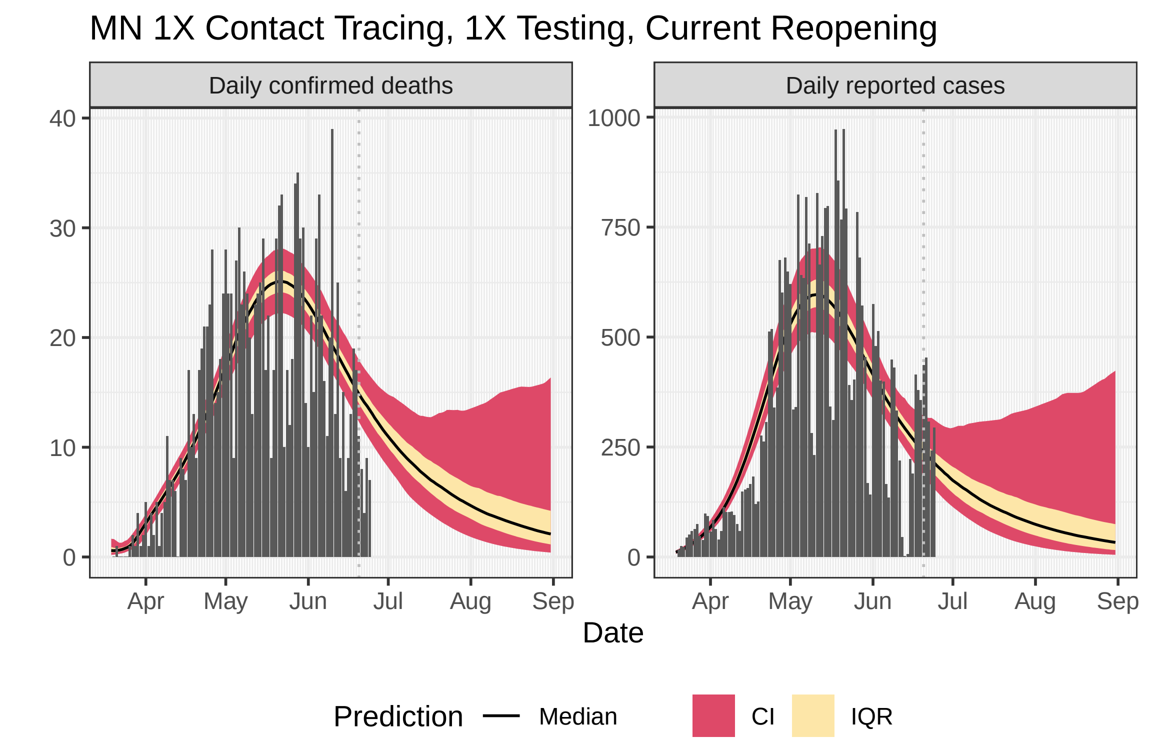

MN 1X Contact Tracing, 1X Testing, Current Reopening
Daily confirmed deaths
Daily reported cases
40
30
20
10
0
1000
750
500
250
0
Apr
May
Jun
Jul
Aug
Sep
Date
Prediction
Median
CI
IQR

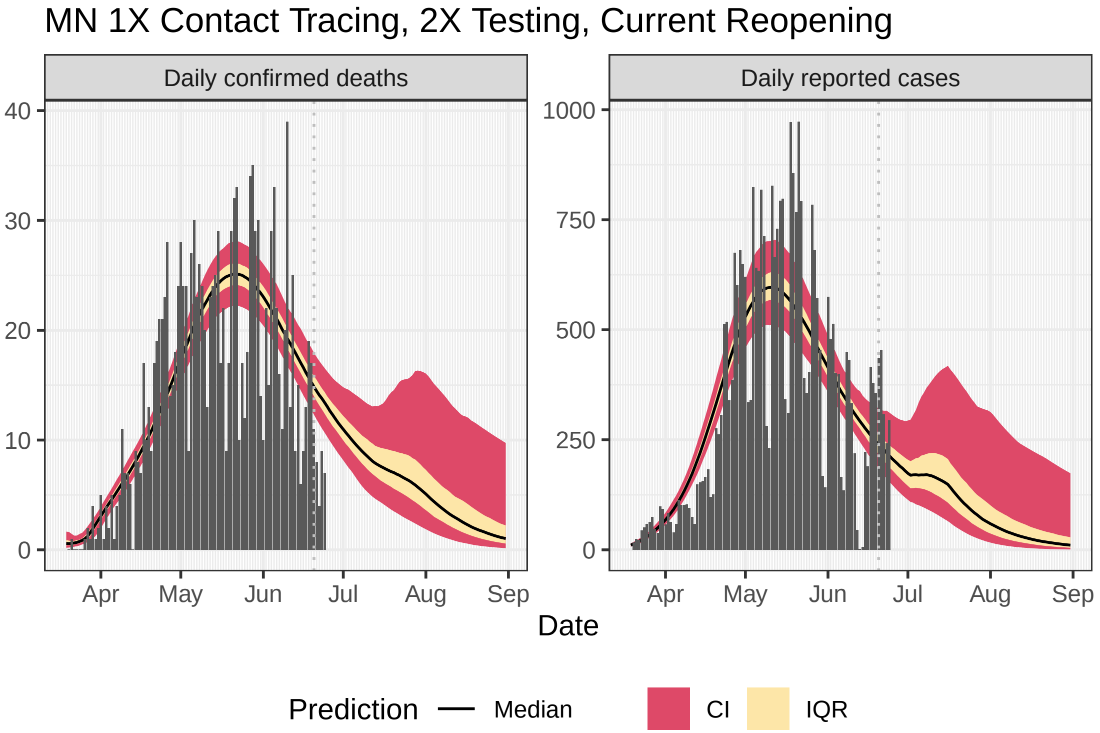

MN 1X Contact Tracing, 2X Testing, Current Reopening
Daily confirmed deaths
Daily reported cases
40
30
20
10
0
1000
750
500
250
0
Apr
May
Jun
Jul
Aug
Sep
Date
Prediction
Median
CI
IQR

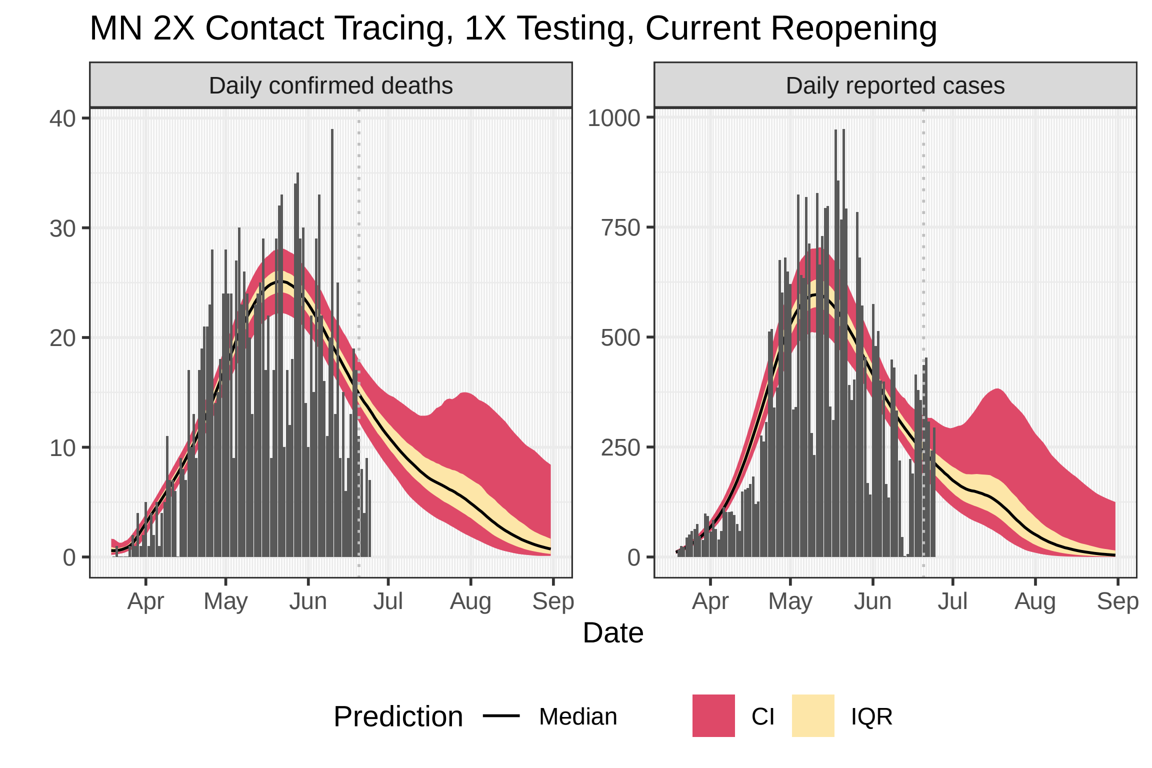
MN 2X Contact Tracing, 1X Testing, Current Reopening
Daily confirmed deaths
Daily reported cases
40
30
20
10
0
1000
750
500
250
0
Apr
May
Jun
Jul
Aug
Sep
Date
Prediction
Median
CI
IQR

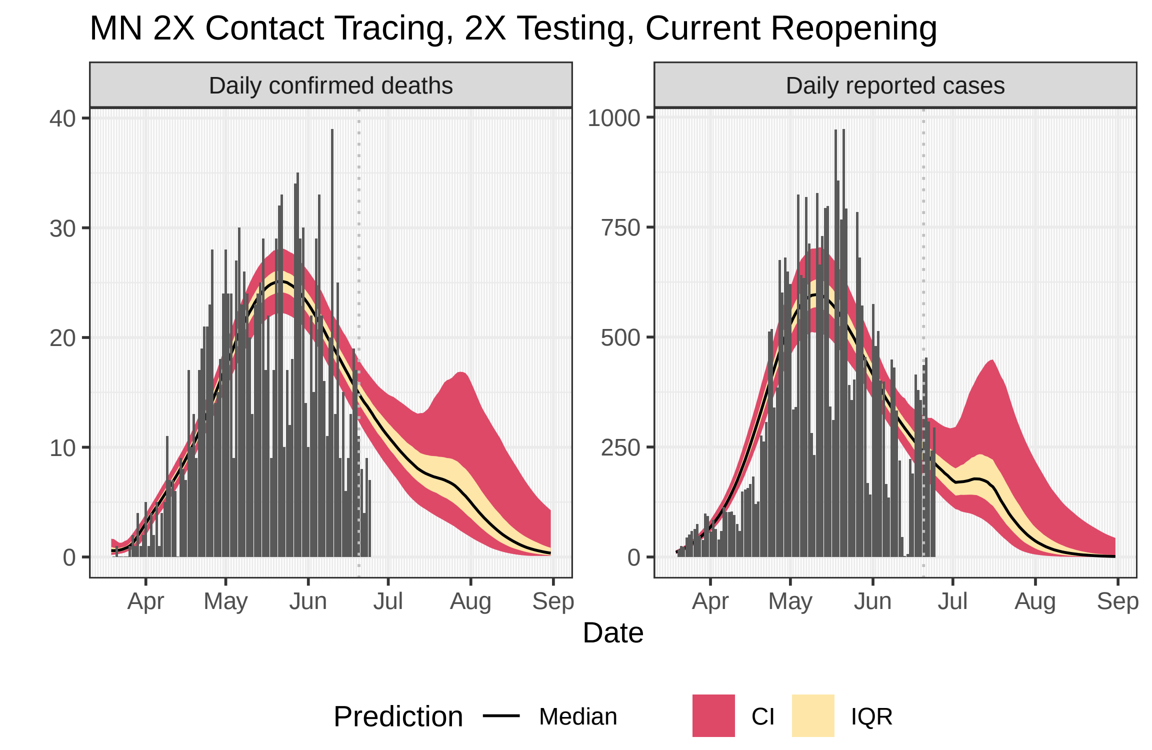
MN 2X Contact Tracing, 2X Testing, Current Reopening
Daily confirmed deaths
Daily reported cases
40
30
20
10
0
1000
750
500
250
0
Apr
May
Jun
Jul
Aug
Sep
Date
Prediction
Median
CI
IQR

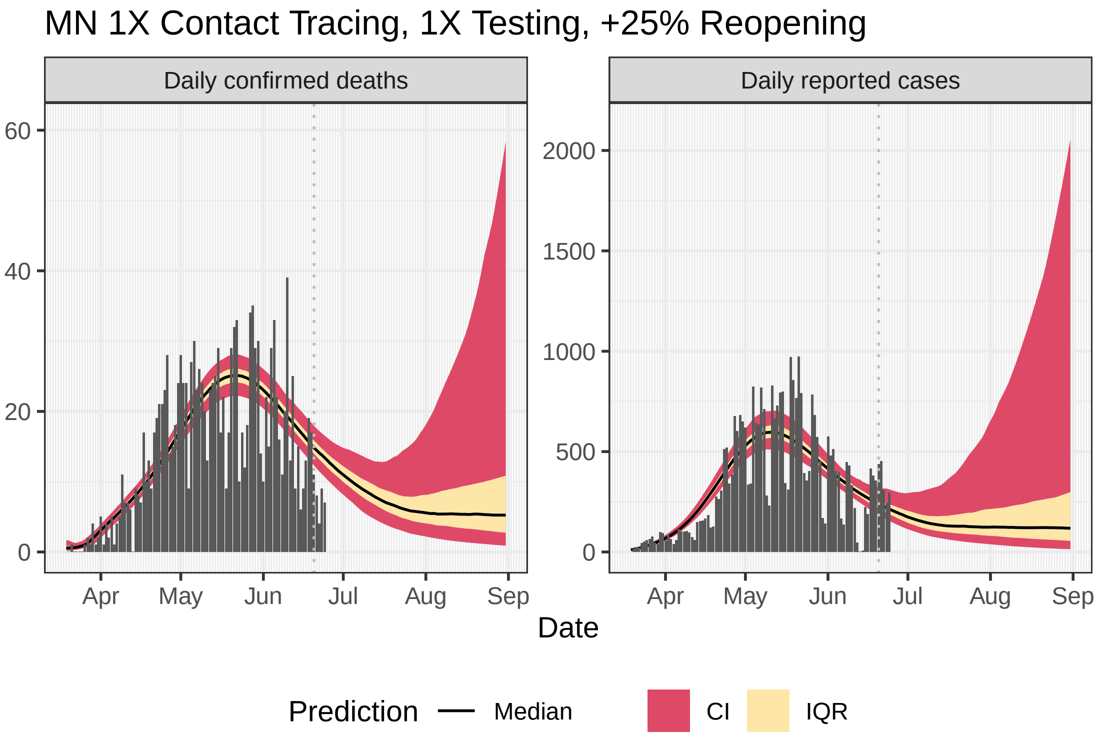

MN 1X Contact Tracing, 1X Testing, +25% Reopening
Daily confirmed deaths
Daily reported cases
60
40
20
0
2000
1500
1000
500
0
Apr
May
Jun
Jul
Aug
Sep
Date
Prediction
Median
CI
IQR

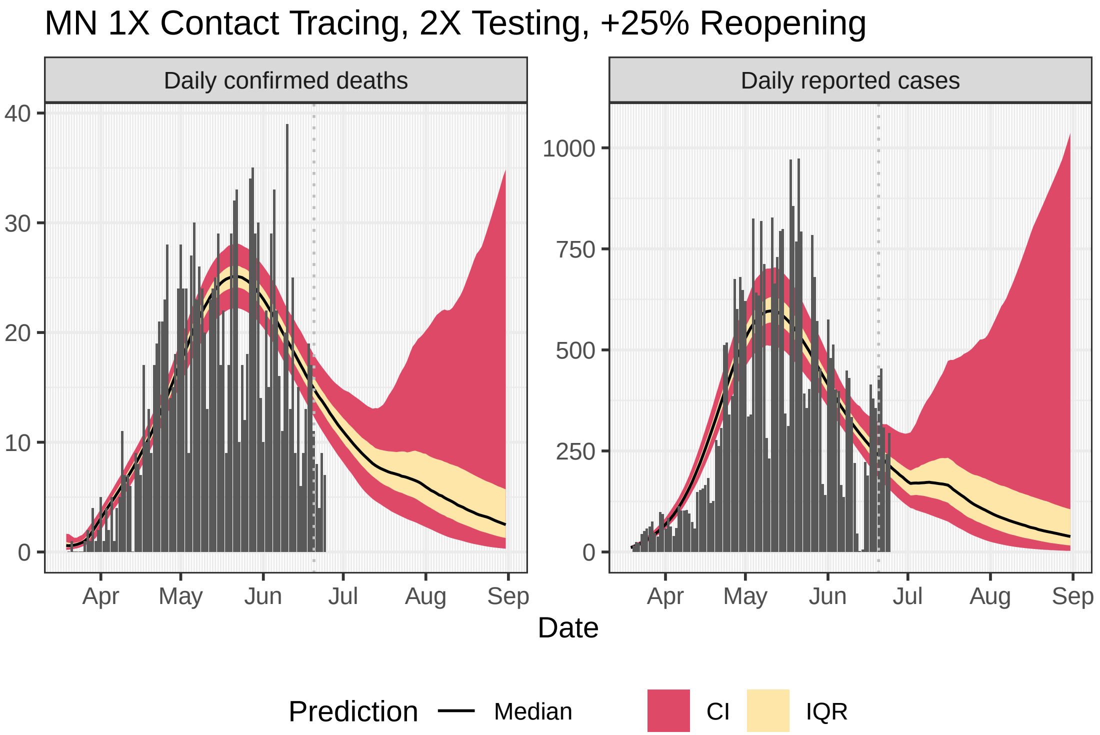
MN 1X Contact Tracing, 2X Testing, +25% Reopening
Daily confirmed deaths
Daily reported cases
0
10
20
30
40
0
250
500
750
1000
Apr
May
Jun
Jul
Aug
Sep
Date
Prediction
Median
CI
IQR

MN 2X Contact Tracing, 1X Testing, +25% Reopening

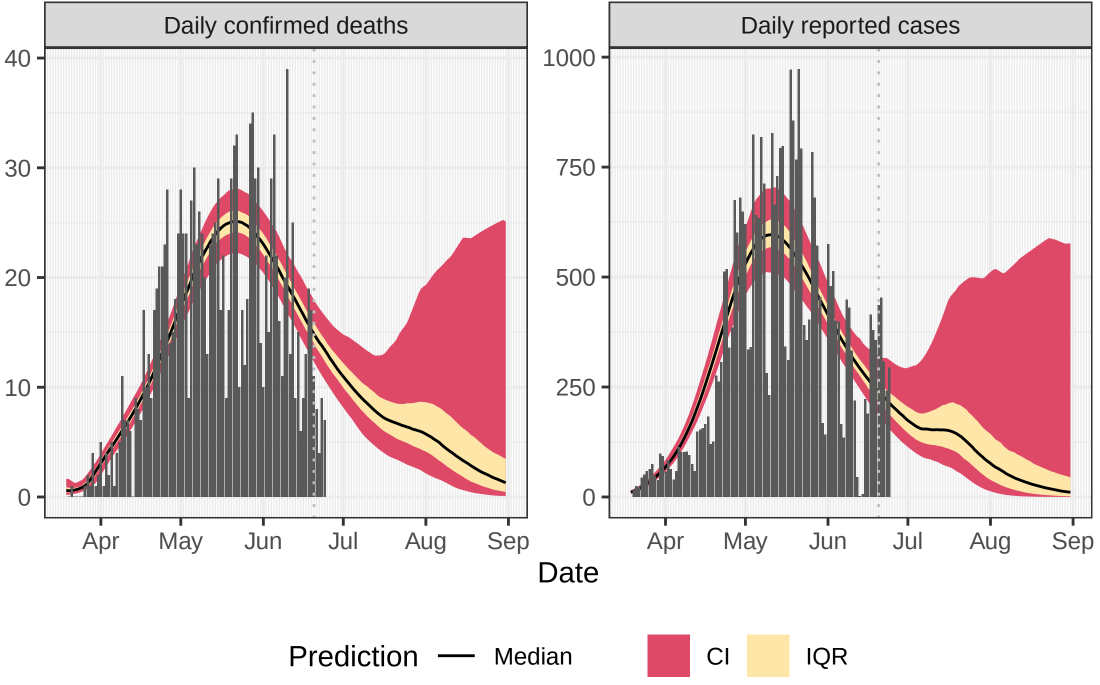

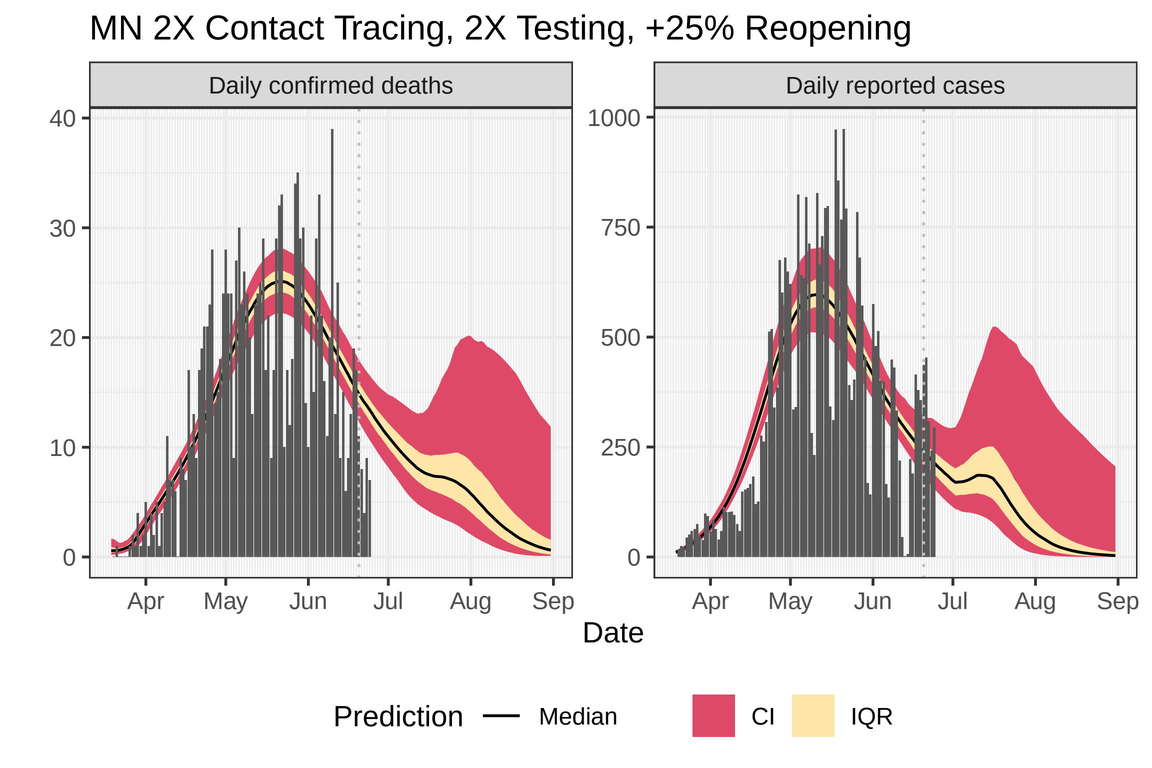
MN 2X Contact Tracing, 2X Testing, +25% Reopening
Daily confirmed deaths
Daily reported cases
40
30
20
10
0
1000
750
500
250
0
Apr
May
Jun
Jul
Aug
Sep
Date
Prediction
Median
CI
IQR

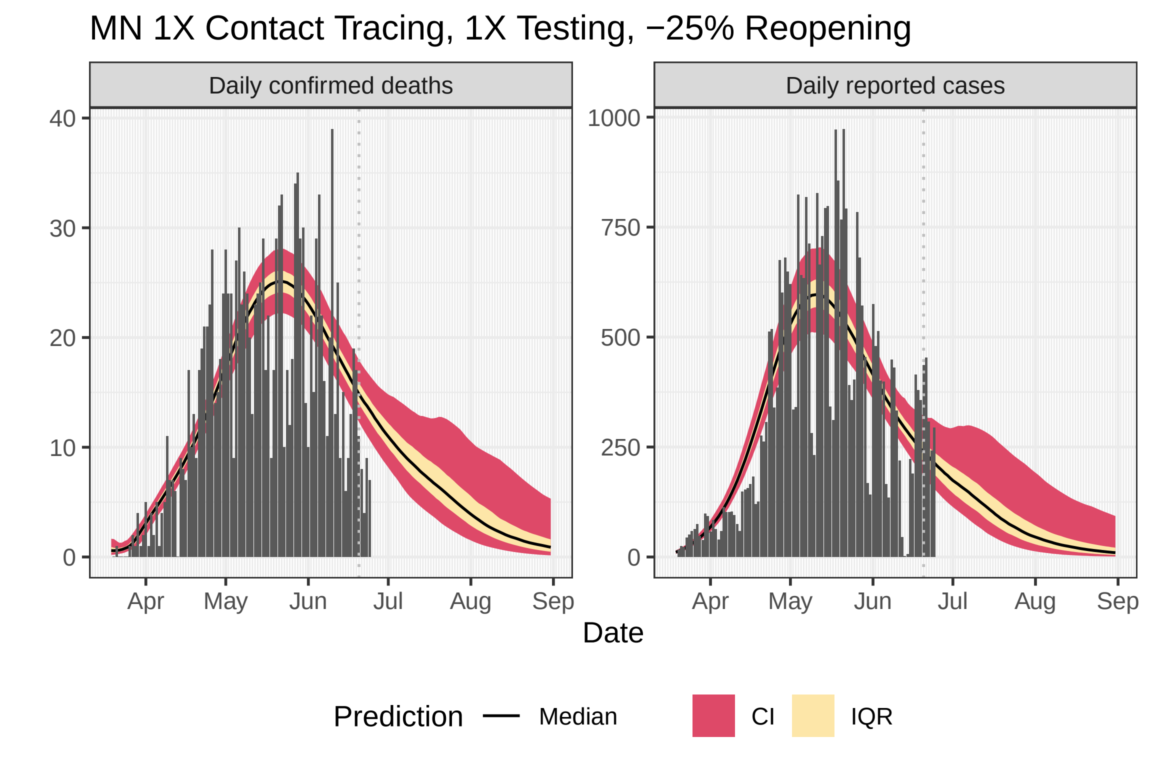
MN 1X Contact Tracing, 1X Testing, −25% Reopening
Daily confirmed deaths
Daily reported cases
40
30
20
10
0
1000
750
500
250
0
Apr
May
Jun
Jul
Aug
Sep
Date
Prediction
Median
CI
IQR

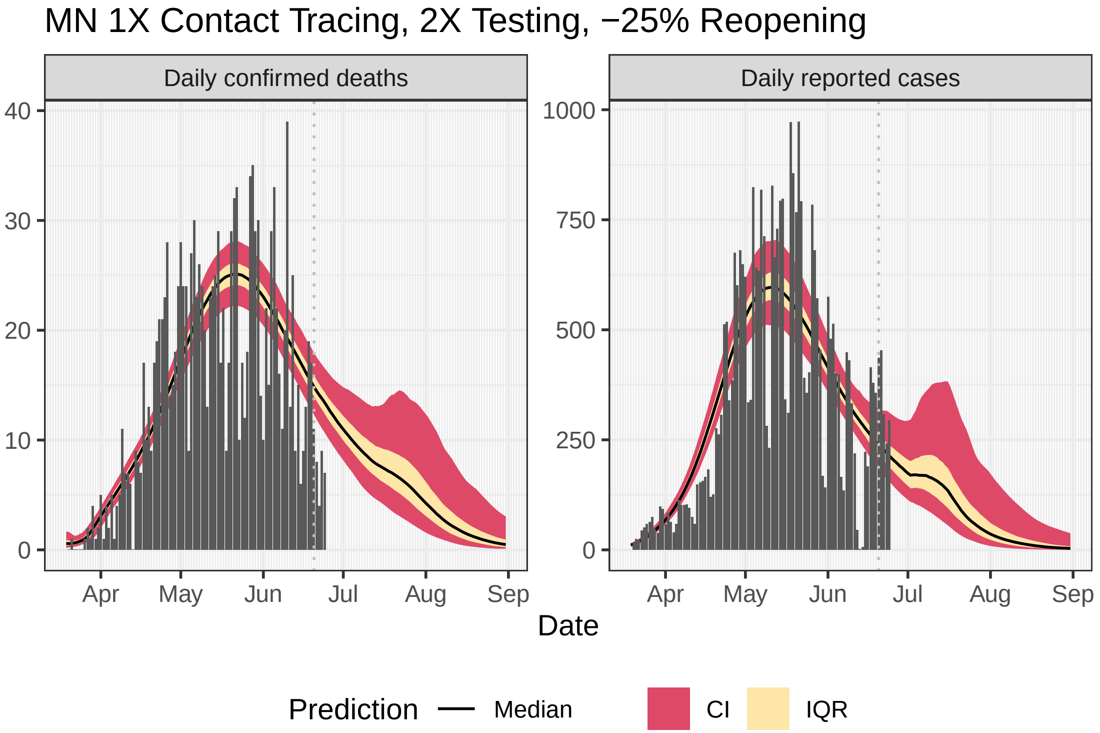
MN 1X Contact Tracing, 2X Testing, −25% Reopening
Daily confirmed deaths
Daily reported cases
0
10
20
30
40
0
250
500
750
1000
Apr
May
Jun
Jul
Aug
Sep
Date
Prediction
Median
CI
IQR

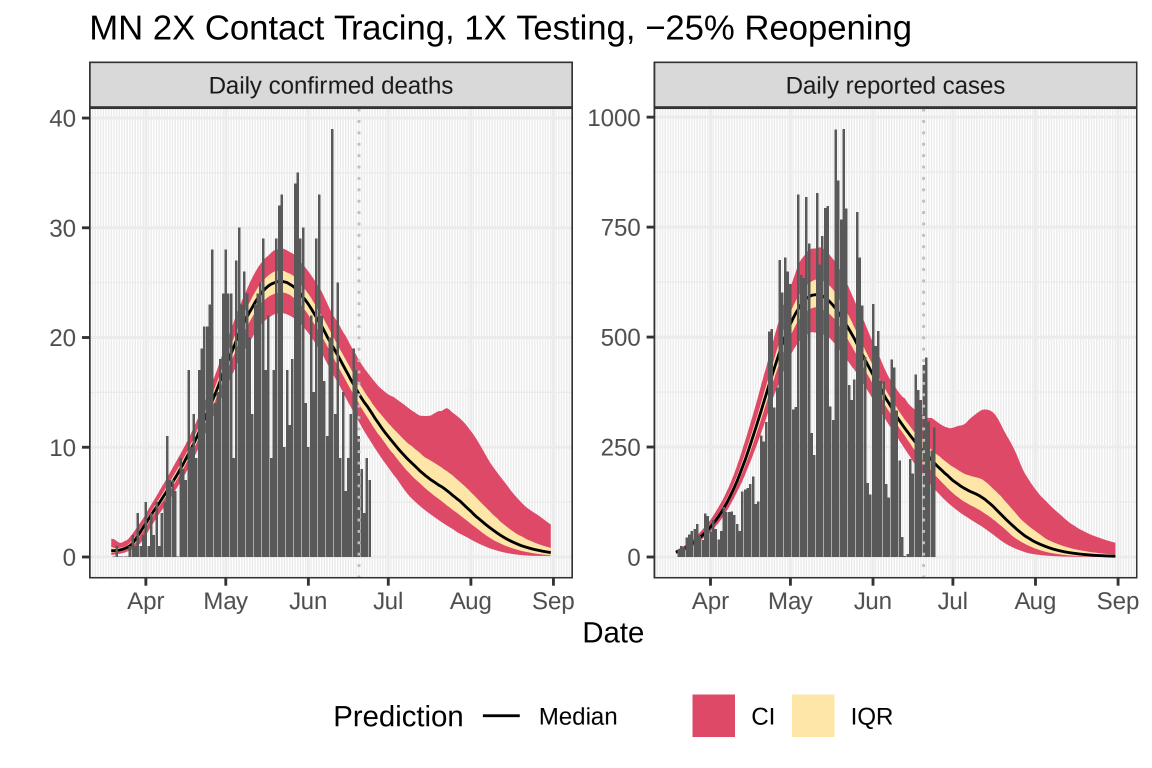

MN 2X Contact Tracing, 1X Testing, −25% Reopening
Daily confirmed deaths
Daily reported cases
40
30
20
10
0
1000
750
500
250
0
Apr
May
Jun
Jul
Aug
Sep
Date
Prediction
Median
CI
IQR

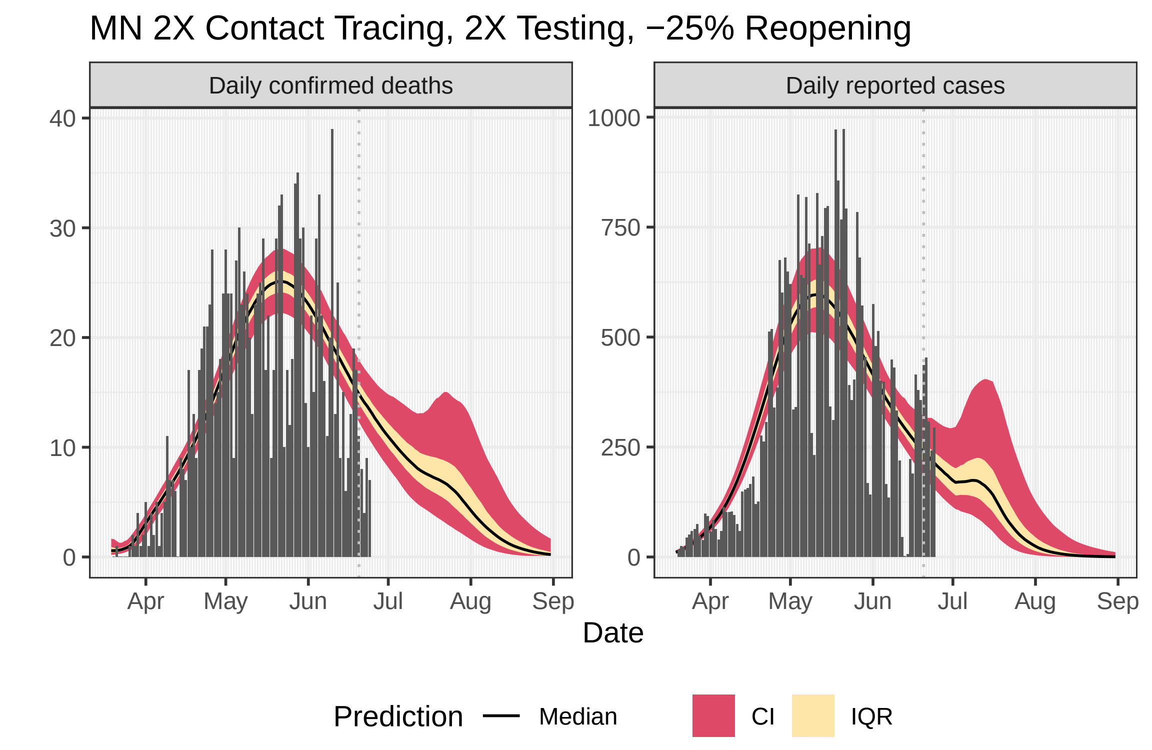

MN 2X Contact Tracing, 2X Testing, −25% Reopening
Daily confirmed deaths
Daily reported cases
40
30
20
10
0
1000
750
500
250
0
Apr
May
Jun
Jul
Aug
Sep
Date
Prediction
Median
CI
IQR

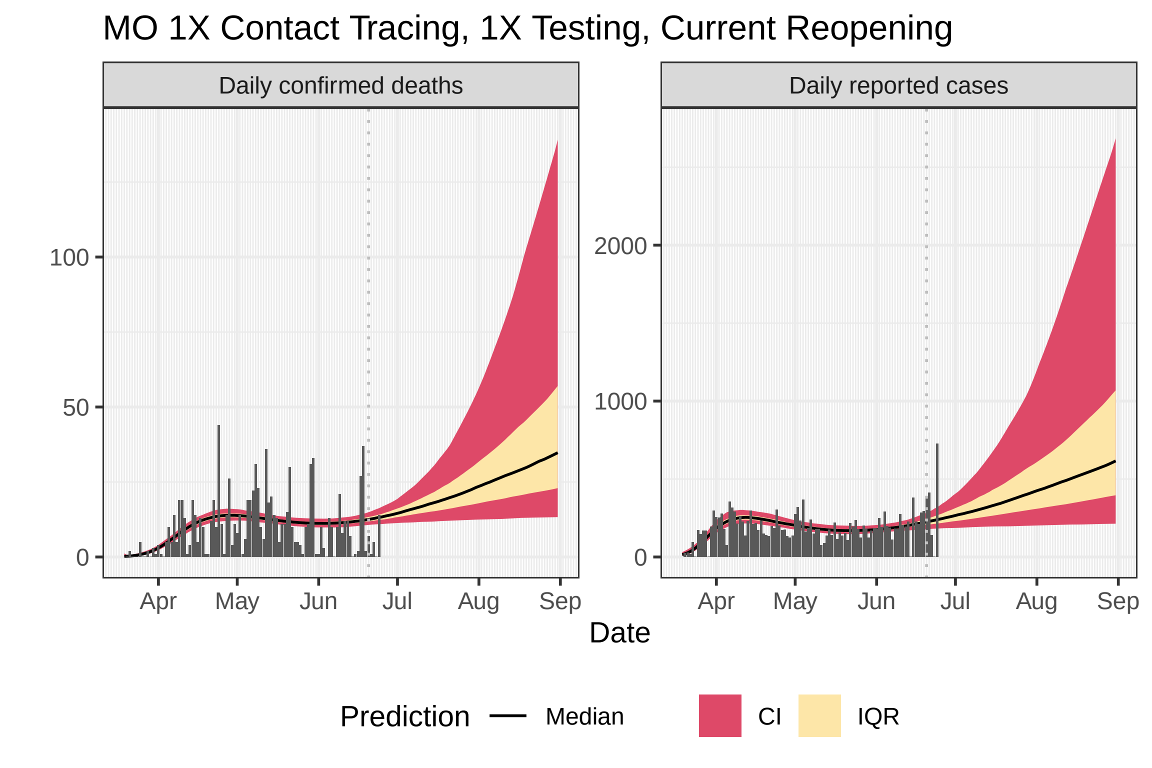

MO 1X Contact Tracing, 1X Testing, Current Reopening
Daily confirmed deaths
Daily reported cases
0
50
100
0
1000
2000
Apr
May
Jun
Jul
Aug
Sep
Date
Prediction
Median
CI
IQR

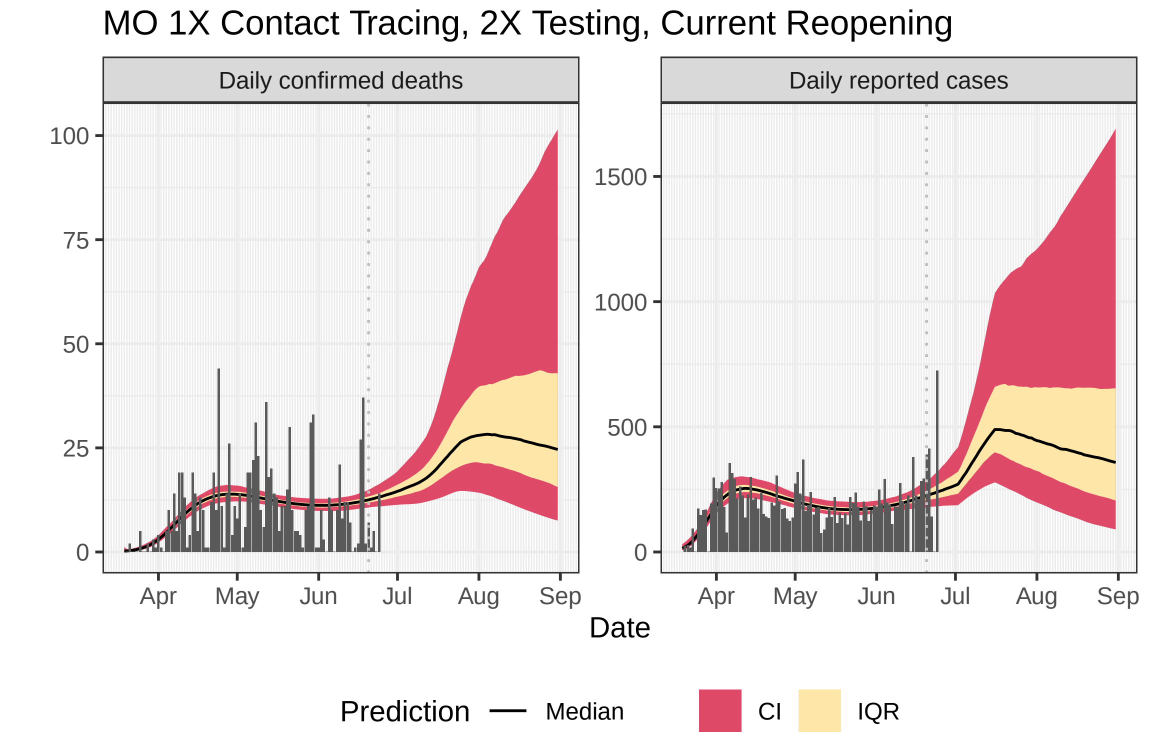
MO 1X Contact Tracing, 2X Testing, Current Reopening
Daily confirmed deaths
Daily reported cases
100
75
50
25
0
1500
1000
500
0
Apr
May
Jun
Jul
Aug
Sep
Date
Prediction
Median
CI
IQR

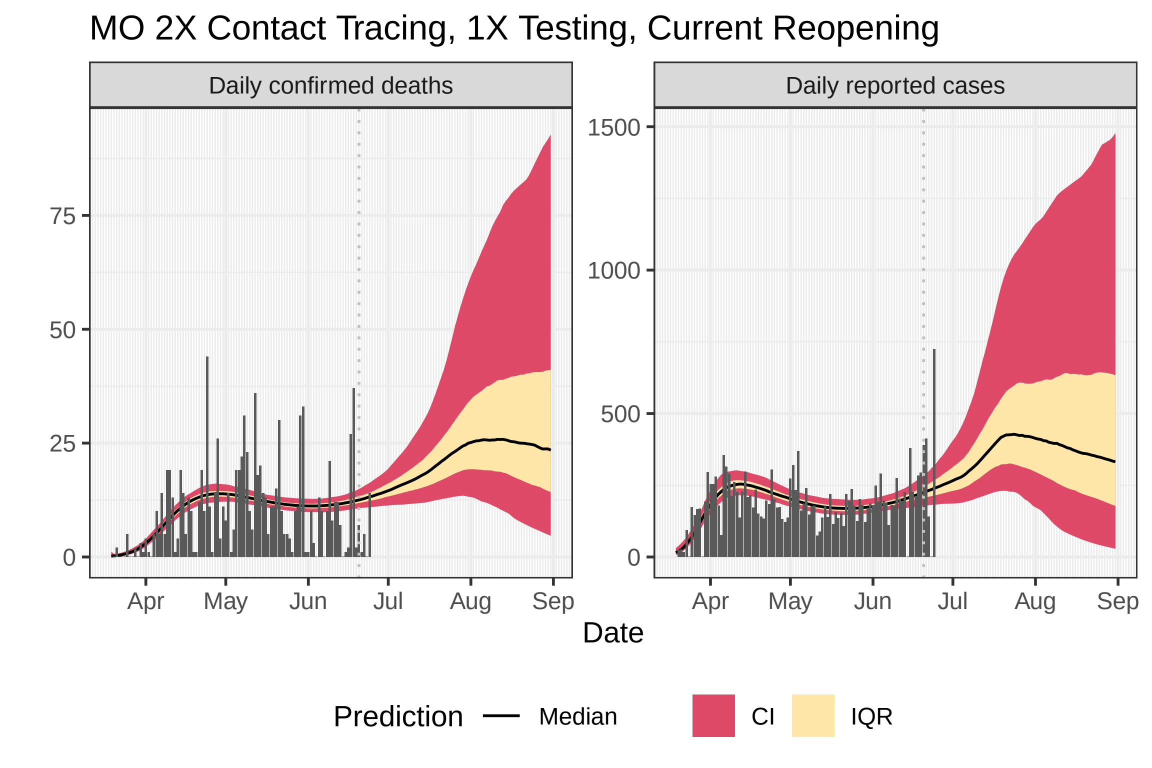

MO 2X Contact Tracing, 1X Testing, Current Reopening
Daily confirmed deaths
Daily reported cases
0
25
50
75
0
500
1000
1500
Apr
May
Jun
Jul
Aug
Sep
Date
Prediction
Median
CI
IQR

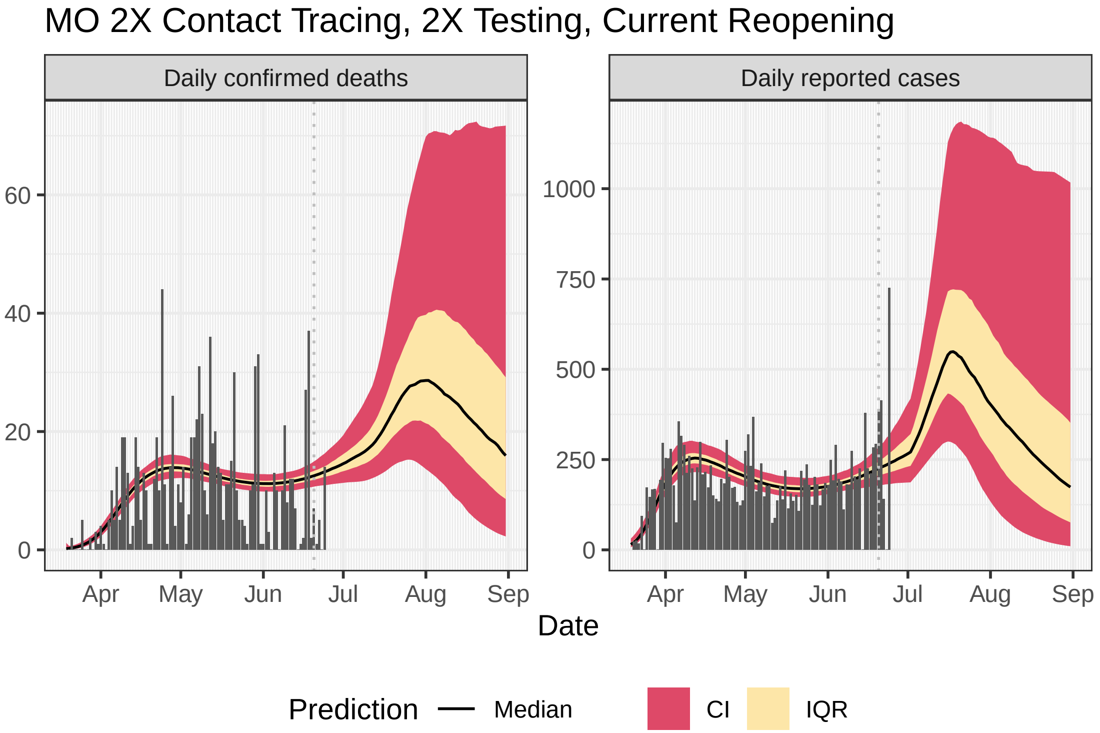
MO 2X Contact Tracing, 2X Testing, Current Reopening
Daily confirmed deaths
Daily reported cases
0
20
40
60
0
250
500
750
1000
Apr
May
Jun
Jul
Aug
Sep
Date
Prediction
Median
CI
IQR

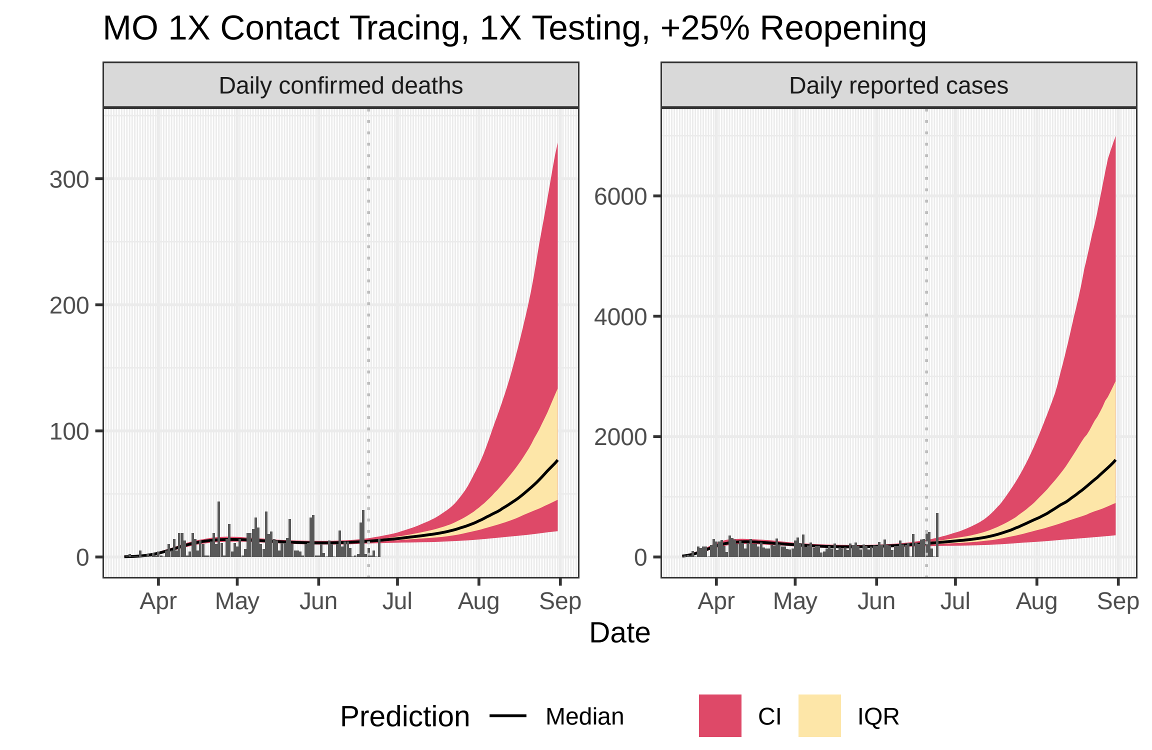
MO 1X Contact Tracing, 1X Testing, +25% Reopening
Daily confirmed deaths
Daily reported cases
0
100
200
300
0
2000
4000
6000
Apr
May
Jun
Jul
Aug
Sep
Date
Prediction
Median
CI
IQR

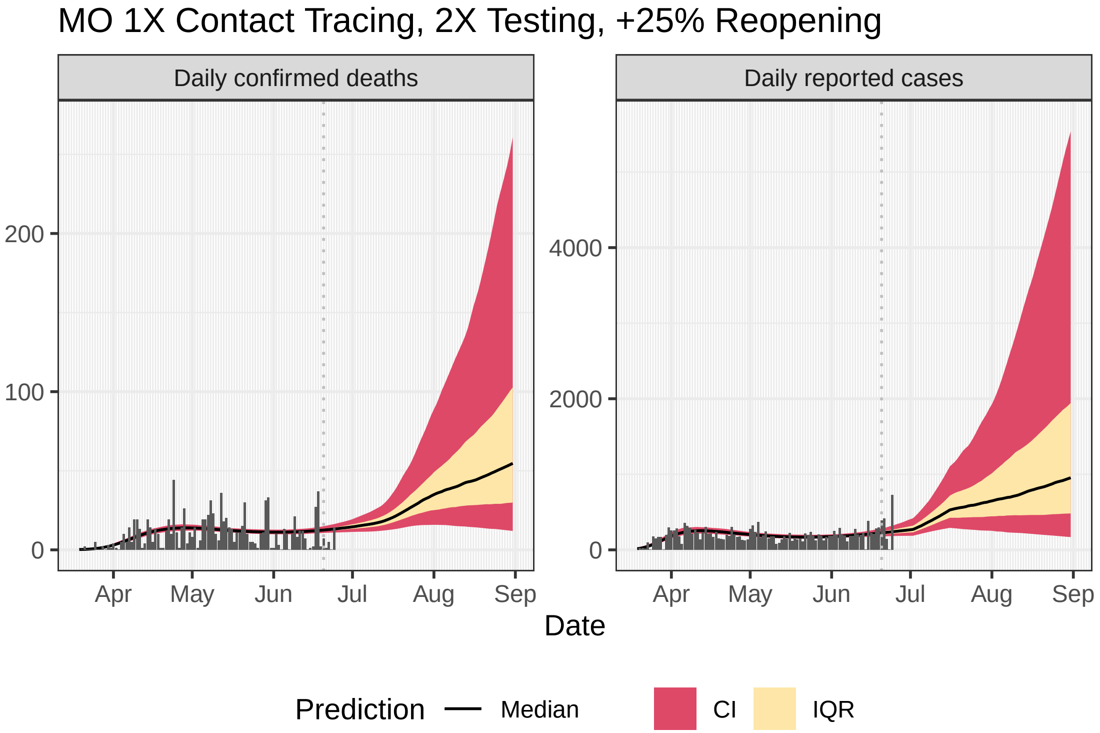
MO 1X Contact Tracing, 2X Testing, +25% Reopening
Daily confirmed deaths
Daily reported cases
200
100
0
4000
2000
0
Apr
May
Jun
Jul
Aug
Sep
Date
Prediction
Median
CI
IQR

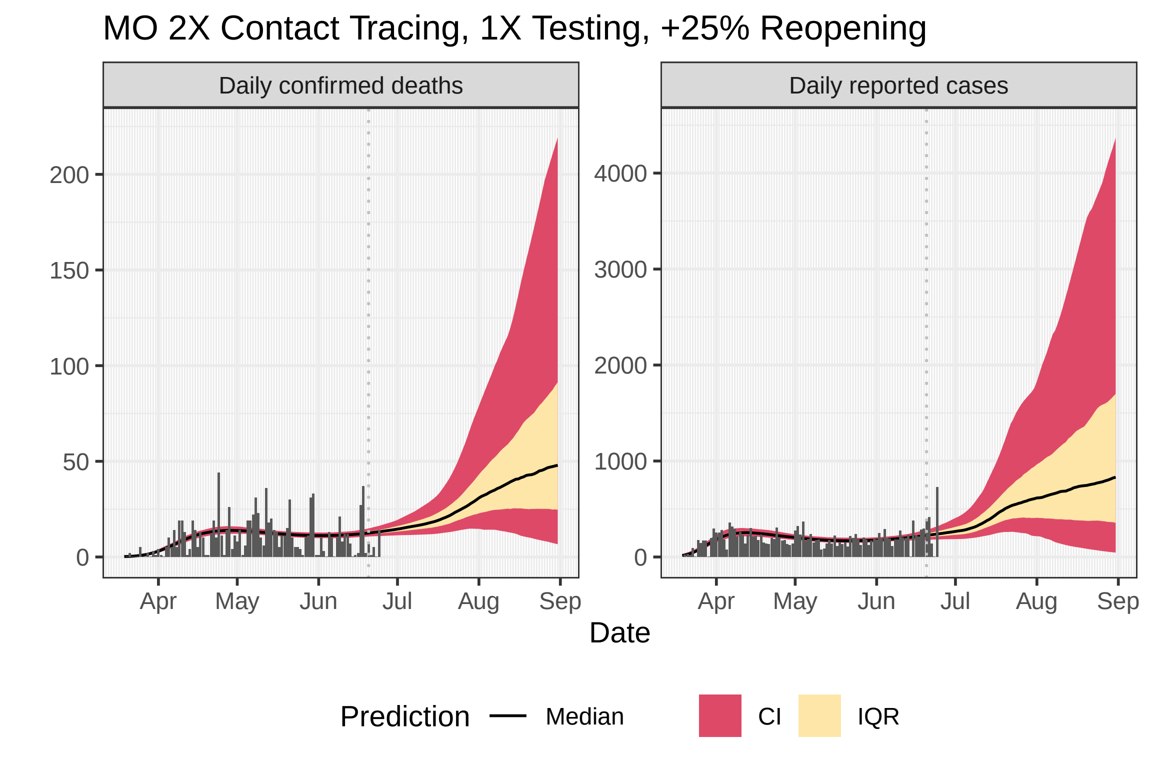
MO 2X Contact Tracing, 1X Testing, +25% Reopening
Daily confirmed deaths
Daily reported cases
0
50
100
150
200
0
1000
2000
3000
4000
Apr
May
Jun
Jul
Aug
Sep
Date
Prediction
Median
CI
IQR

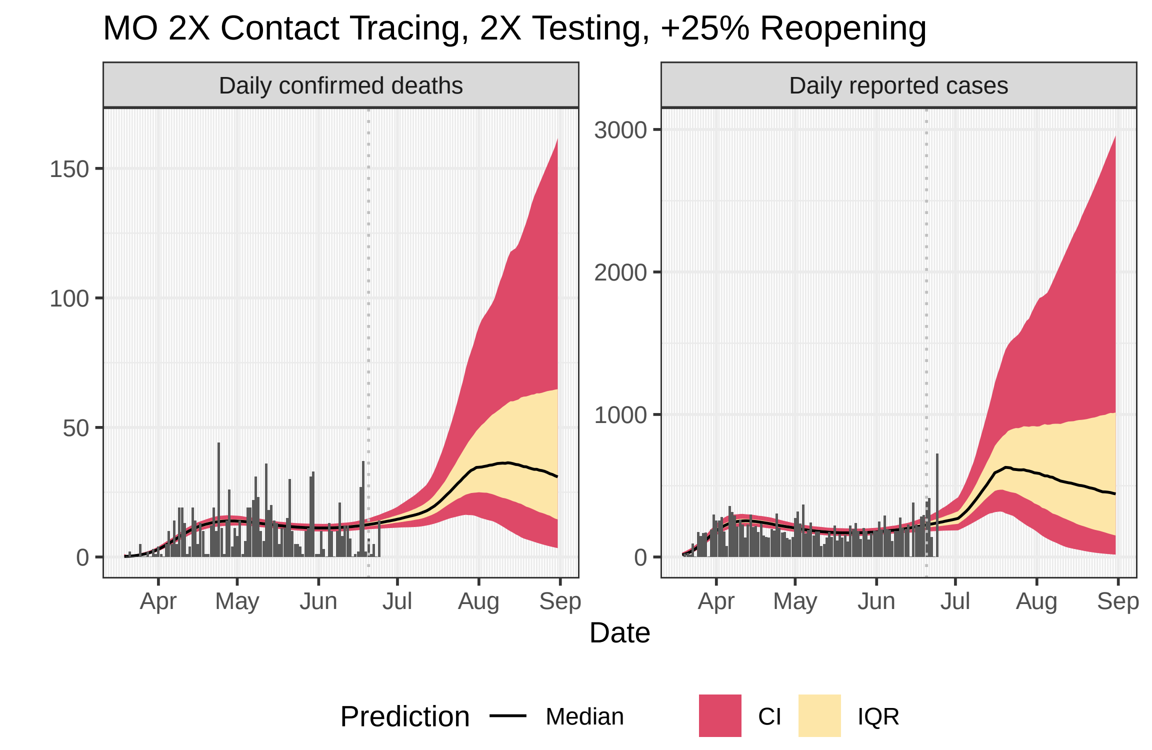
MO 2X Contact Tracing, 2X Testing, +25% Reopening
Daily confirmed deaths
Daily reported cases
150
100
50
0
3000
2000
1000
0
Apr
May
Jun
Jul
Aug
Sep
Date
Prediction
Median
CI
IQR

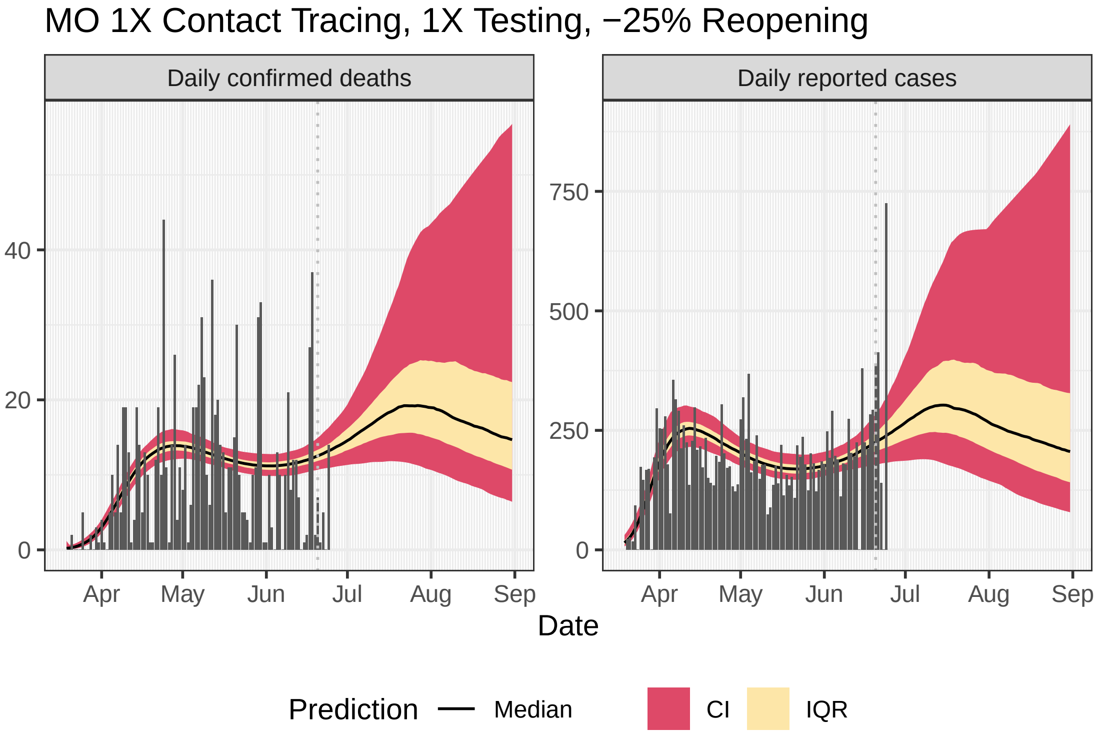

MO 1X Contact Tracing, 1X Testing, −25% Reopening
Daily confirmed deaths
Daily reported cases
0
20
40
0
250
500
750
Apr
May
Jun
Jul
Aug
Sep
Date
Prediction
Median
CI
IQR

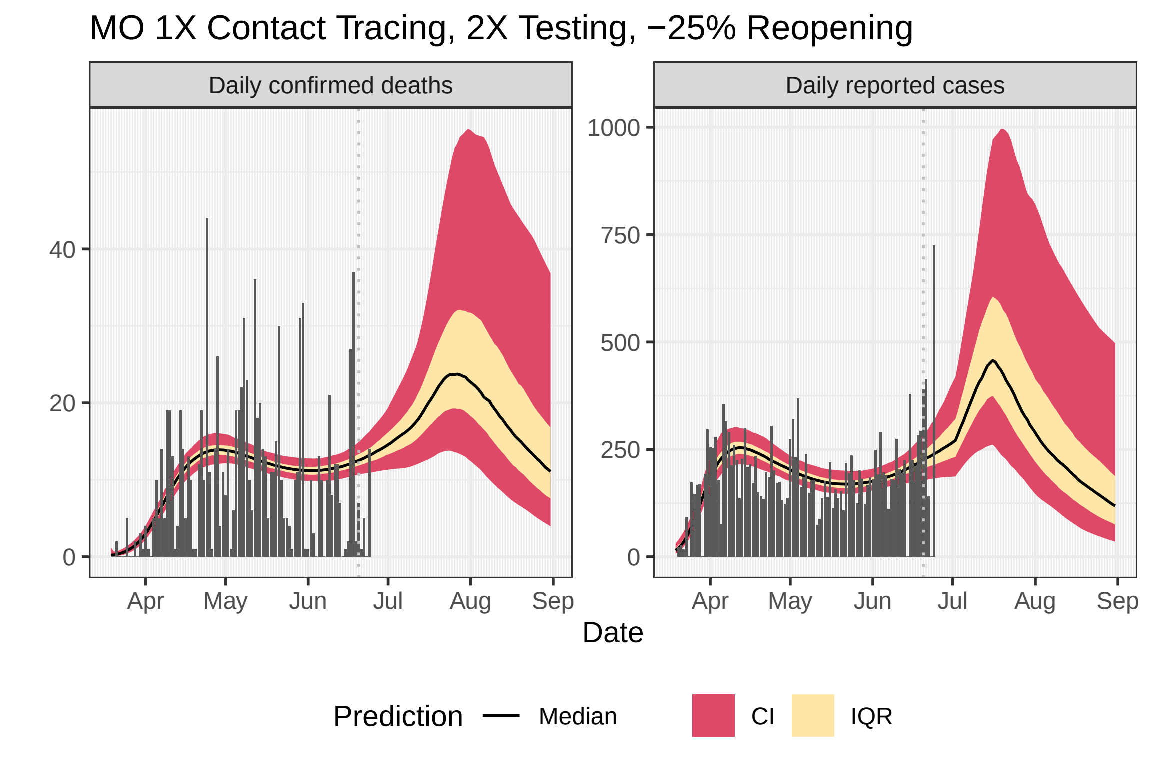

MO 1X Contact Tracing, 2X Testing, −25% Reopening
Daily confirmed deaths
Daily reported cases
0
20
40
0
250
500
750
1000
Apr
May
Jun
Jul
Aug
Sep
Date
Prediction
Median
CI
IQR

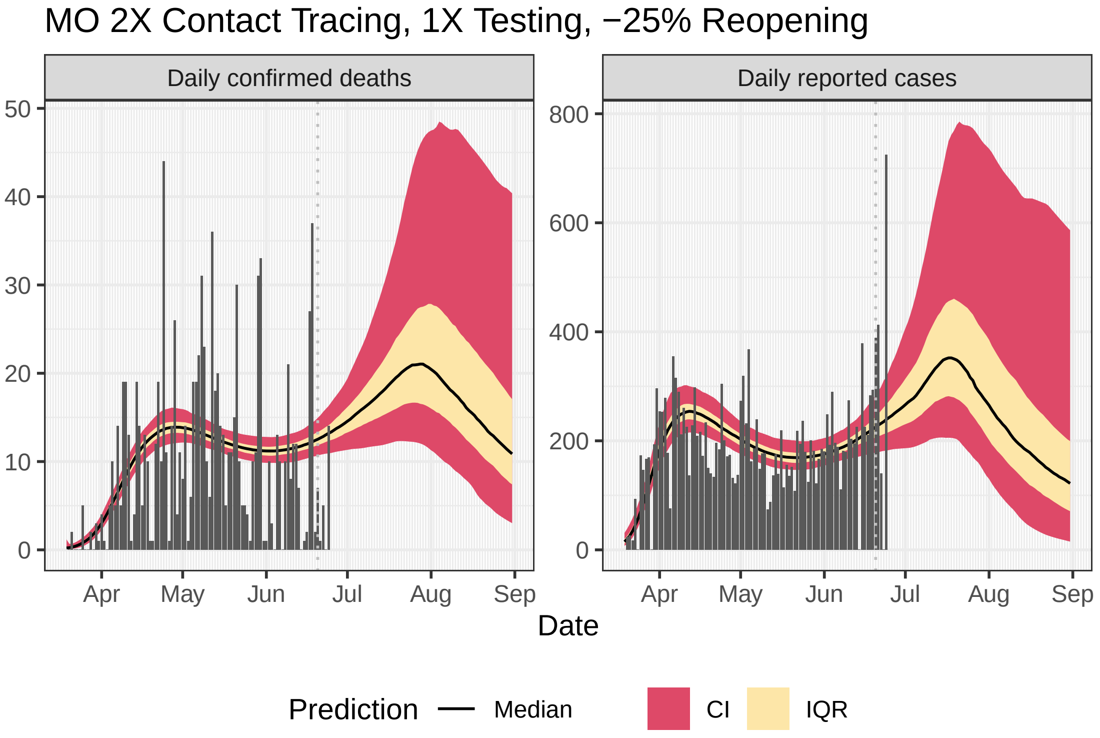
MO 2X Contact Tracing, 1X Testing, −25% Reopening
Daily confirmed deaths
Daily reported cases
50
40
30
20
10
0
800
600
400
200
0
Apr
May
Jun
Jul
Aug
Sep
Date
Prediction
Median
CI
IQR

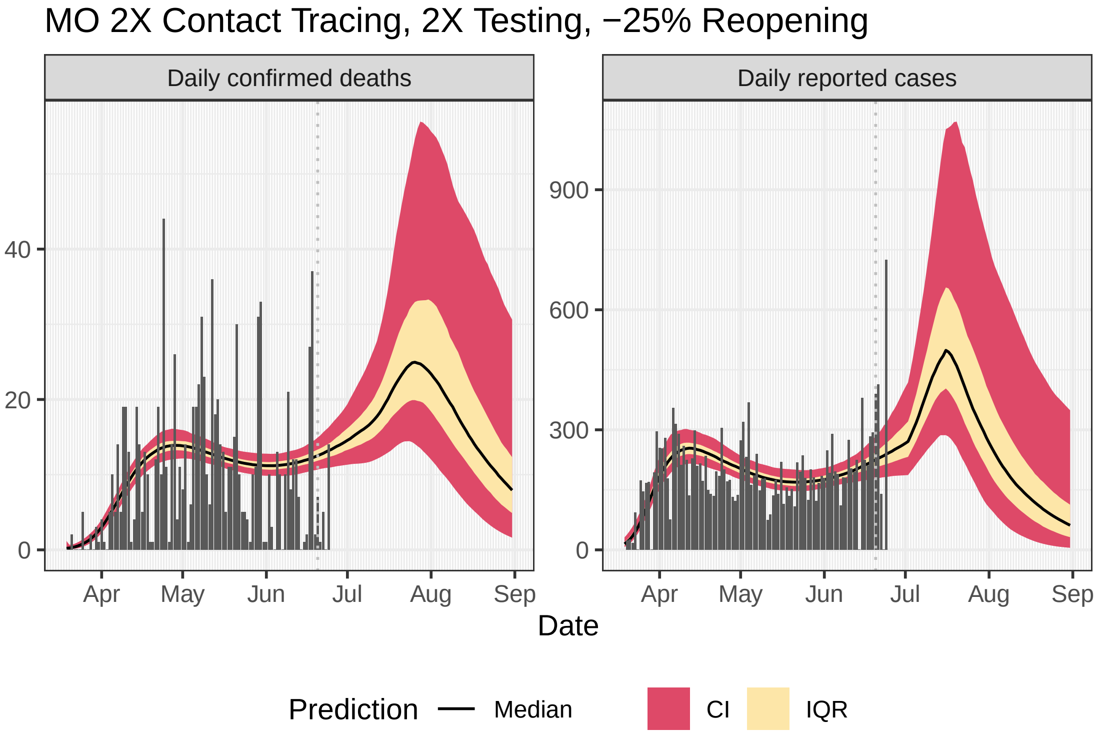

MO 2X Contact Tracing, 2X Testing, −25% Reopening
Daily confirmed deaths
Daily reported cases
0
20
40
0
300
600
900
Apr
May
Jun
Jul
Aug
Sep
Date
Prediction
Median
CI
IQR

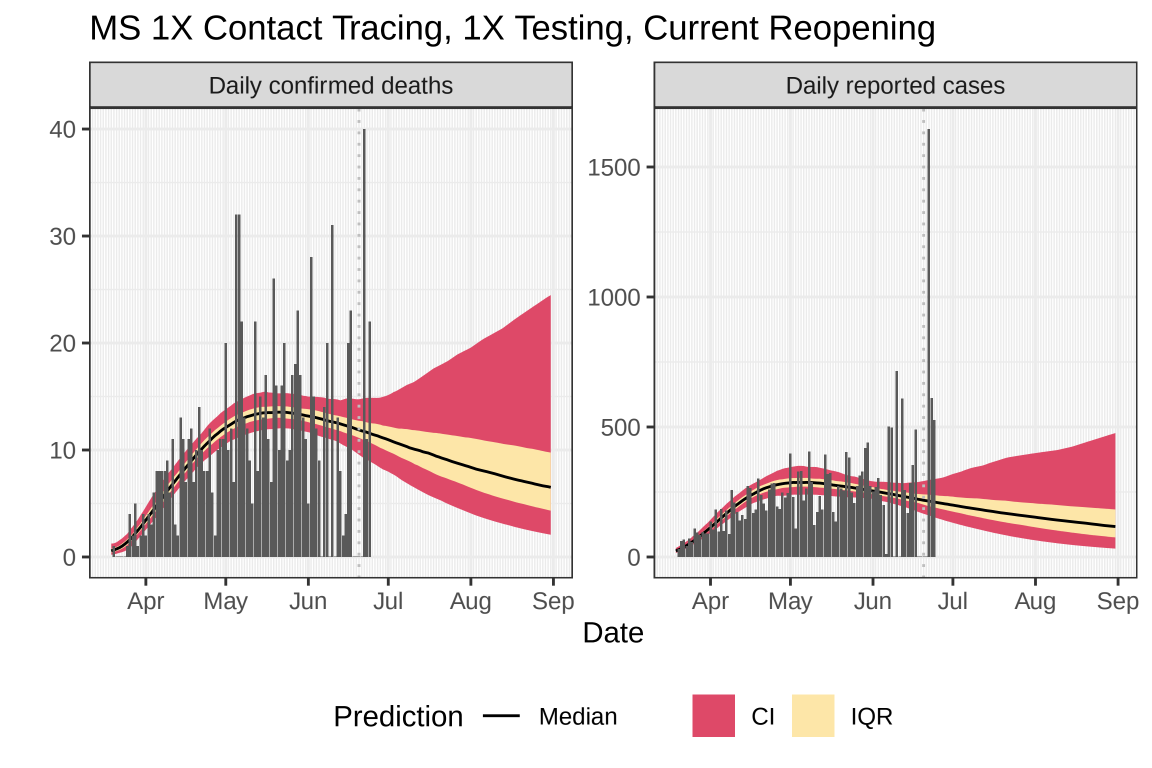
MS 1X Contact Tracing, 1X Testing, Current Reopening
Daily confirmed deaths
Daily reported cases
40
30
20
10
0
1500
1000
500
0
Apr
May
Jun
Jul
Aug
Sep
Date
Prediction
Median
CI
IQR

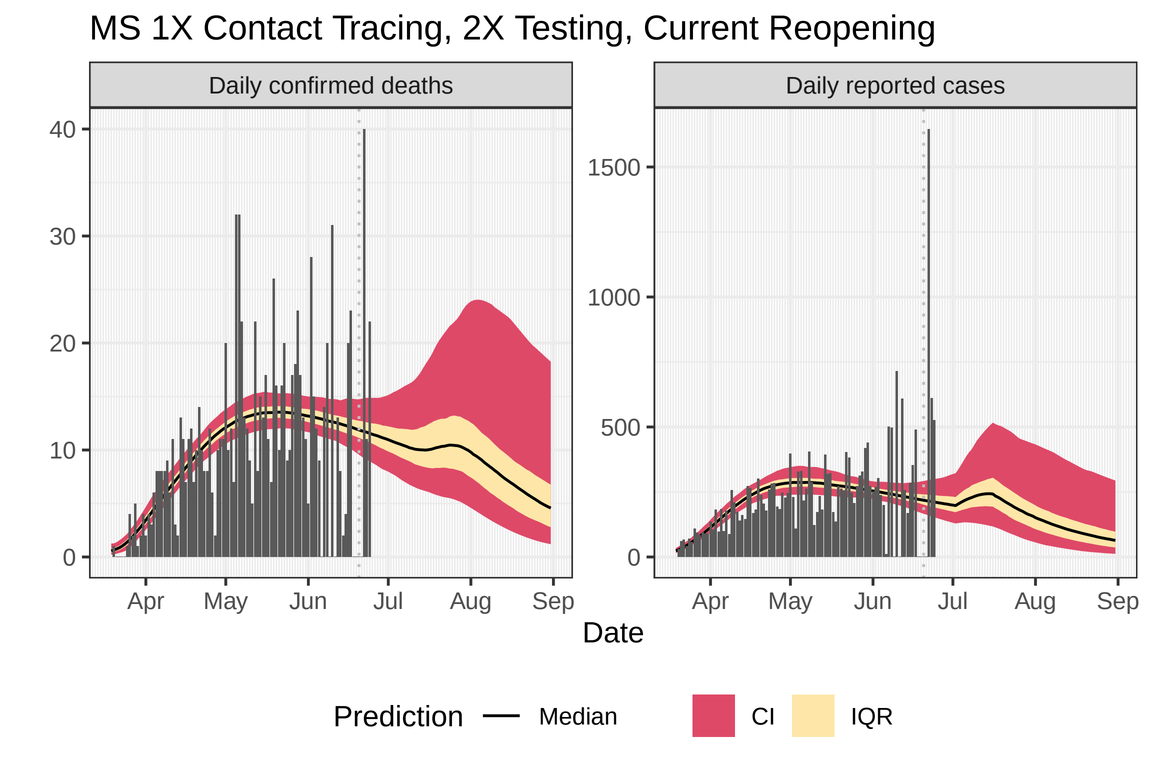
MS 1X Contact Tracing, 2X Testing, Current Reopening
Daily confirmed deaths
Daily reported cases
0
10
20
30
40
0
500
1000
1500
Apr
May
Jun
Jul
Aug
Sep
Date
Prediction
Median
CI
IQR

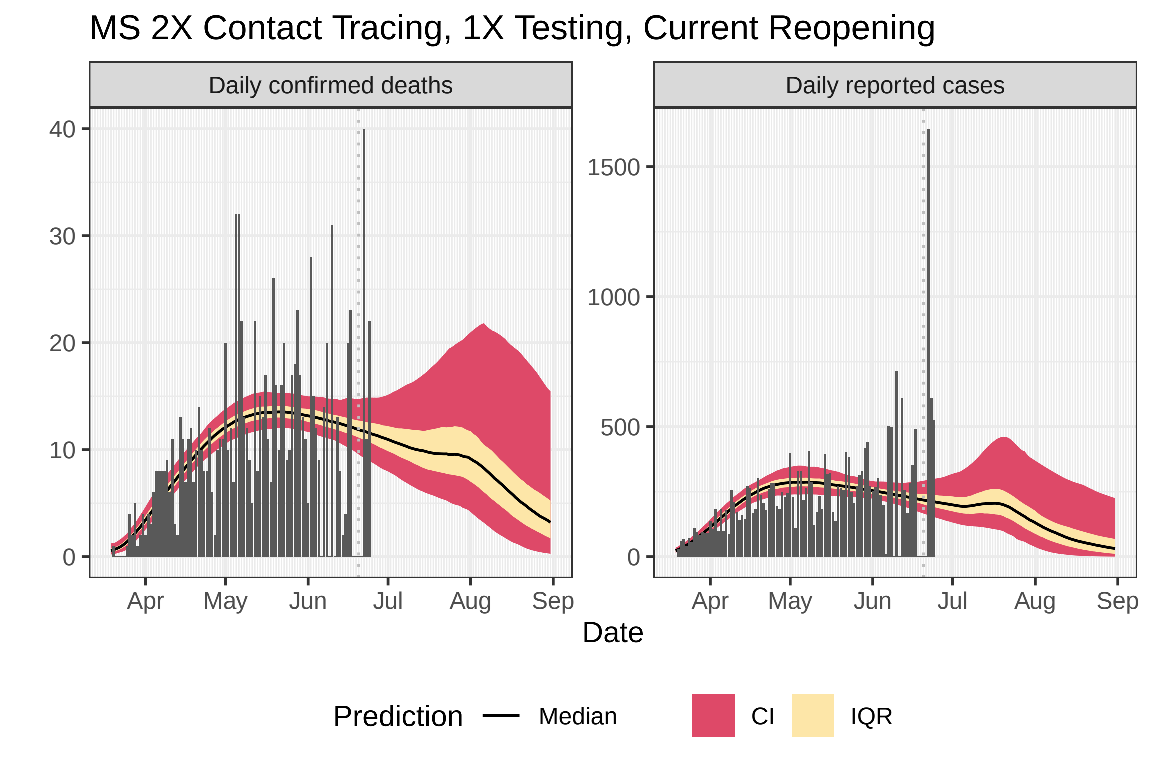

MS 2X Contact Tracing, 1X Testing, Current Reopening
Daily confirmed deaths
Daily reported cases
40
30
20
10
0
1500
1000
500
0
Apr
May
Jun
Jul
Aug
Sep
Date
Prediction
Median
CI
IQR

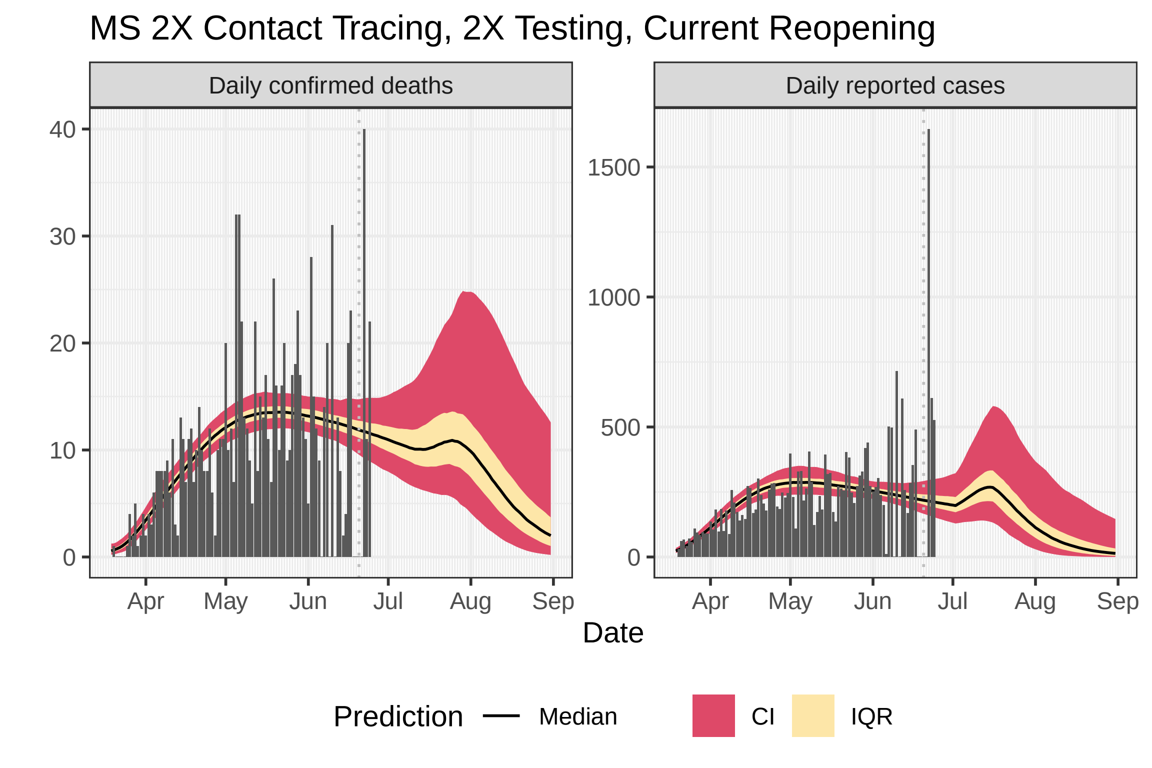

MS 2X Contact Tracing, 2X Testing, Current Reopening
Daily confirmed deaths
Daily reported cases
40
30
20
10
0
1500
1000
500
0
Apr
May
Jun
Jul
Aug
Sep
Date
Prediction
Median
CI
IQR

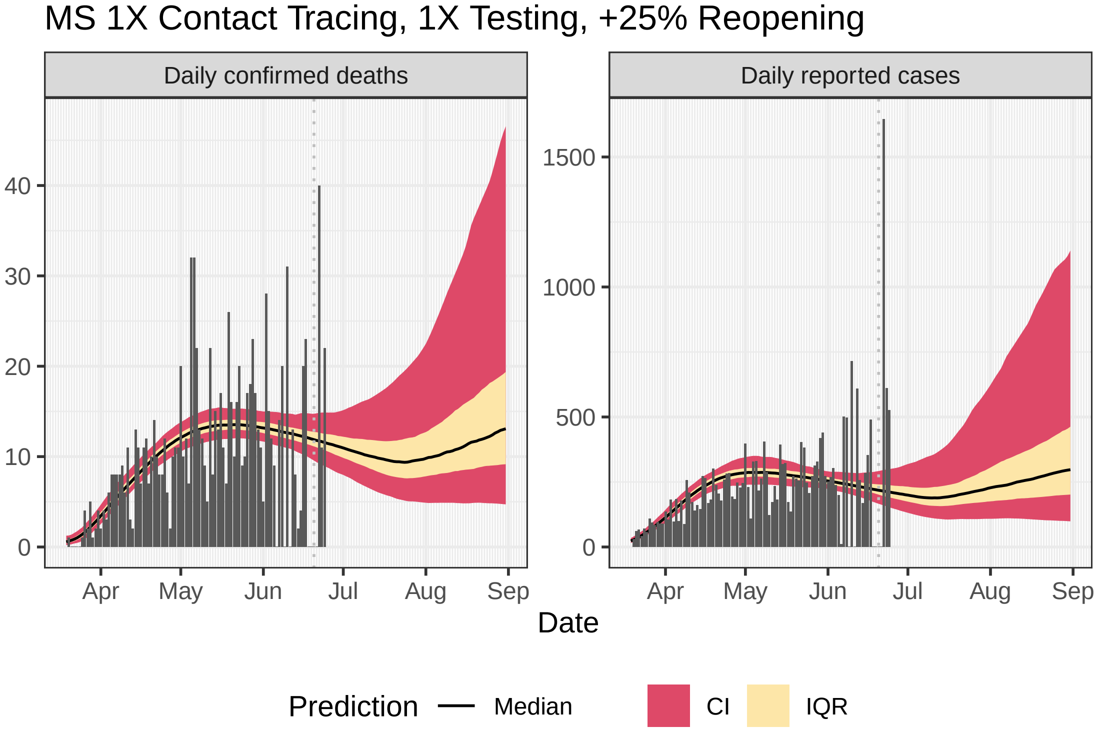

MS 1X Contact Tracing, 1X Testing, +25% Reopening
Daily confirmed deaths
Daily reported cases
0
10
20
30
40
500
1000
1500
Apr
May
Jun
Jul
Aug
Sep
Date
Prediction
Median
CI
IQR

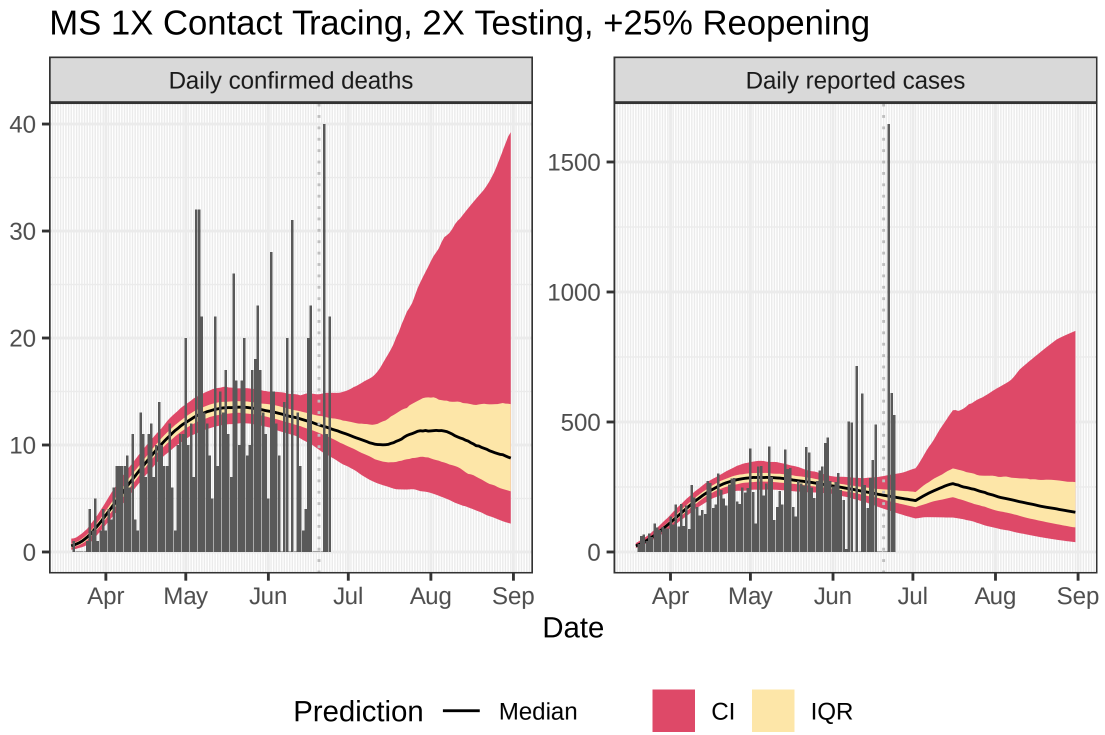

MS 1X Contact Tracing, 2X Testing, +25% Reopening
Daily confirmed deaths
Daily reported cases
40
30
20
10
0
1500
1000
500
0
Apr
May
Jun
Jul
Aug
Sep
Date
Prediction
Median
CI
IQR

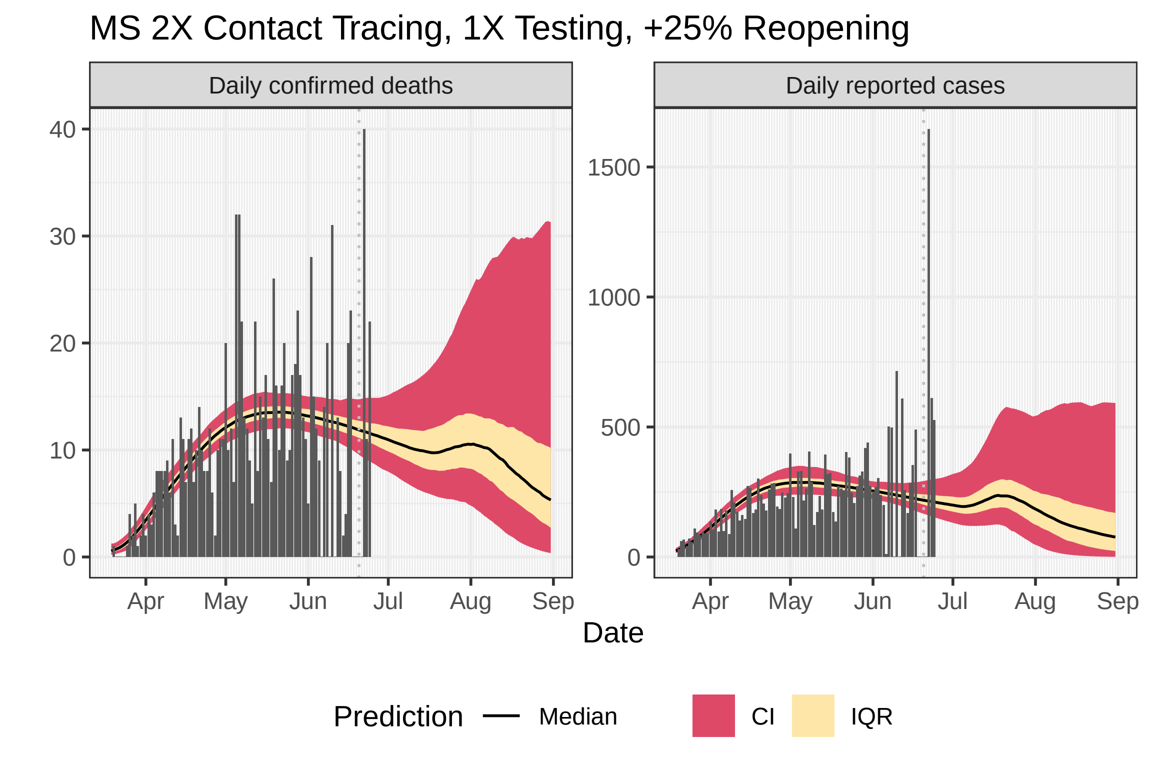
MS 2X Contact Tracing, 1X Testing, +25% Reopening
Daily confirmed deaths
Daily reported cases
40
30
20
10
0
1500
1000
500
0
Apr
May
Jun
Jul
Aug
Sep
Date
Prediction
Median
CI
IQR

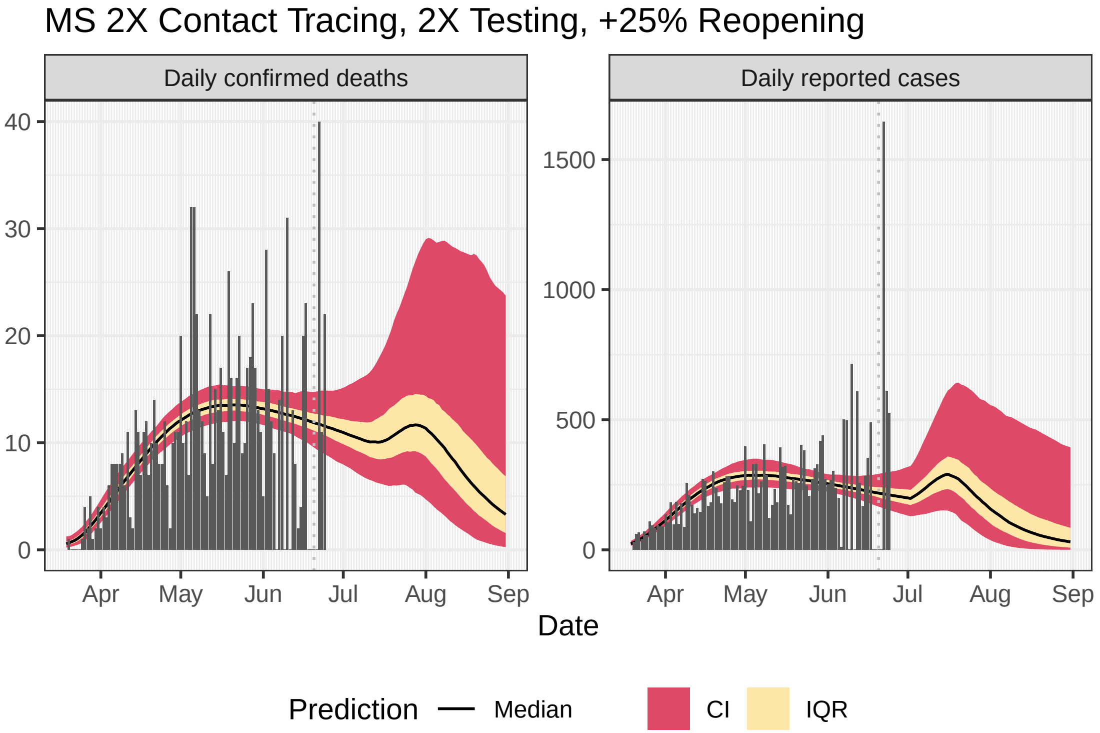
MS 2X Contact Tracing, 2X Testing, +25% Reopening
Daily confirmed deaths
Daily reported cases
40
30
20
10
0
1500
1000
500
0
Apr
May
Jun
Jul
Aug
Sep
Date
Prediction
Median
CI
IQR

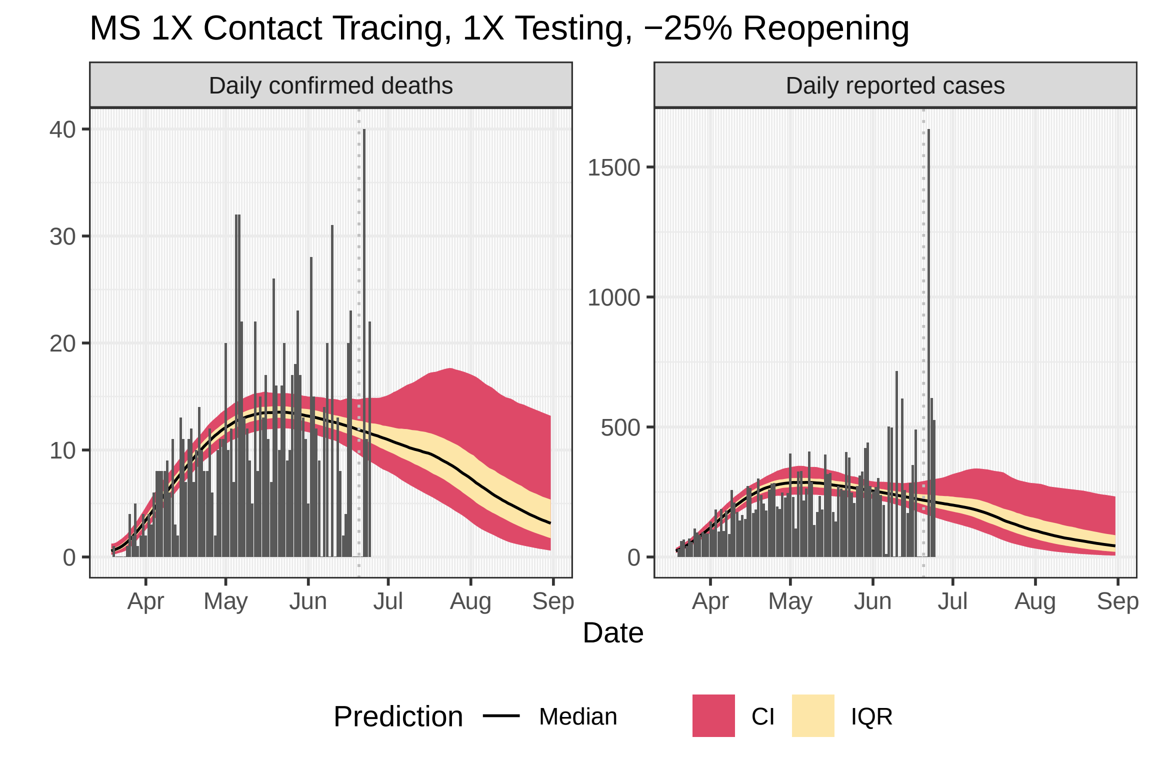

MS 1X Contact Tracing, 1X Testing, −25% Reopening
Daily confirmed deaths
Daily reported cases
0
10
20
30
40
0
500
1000
1500
Apr
May
Jun
Jul
Aug
Sep
Date
Prediction
Median
CI
IQR

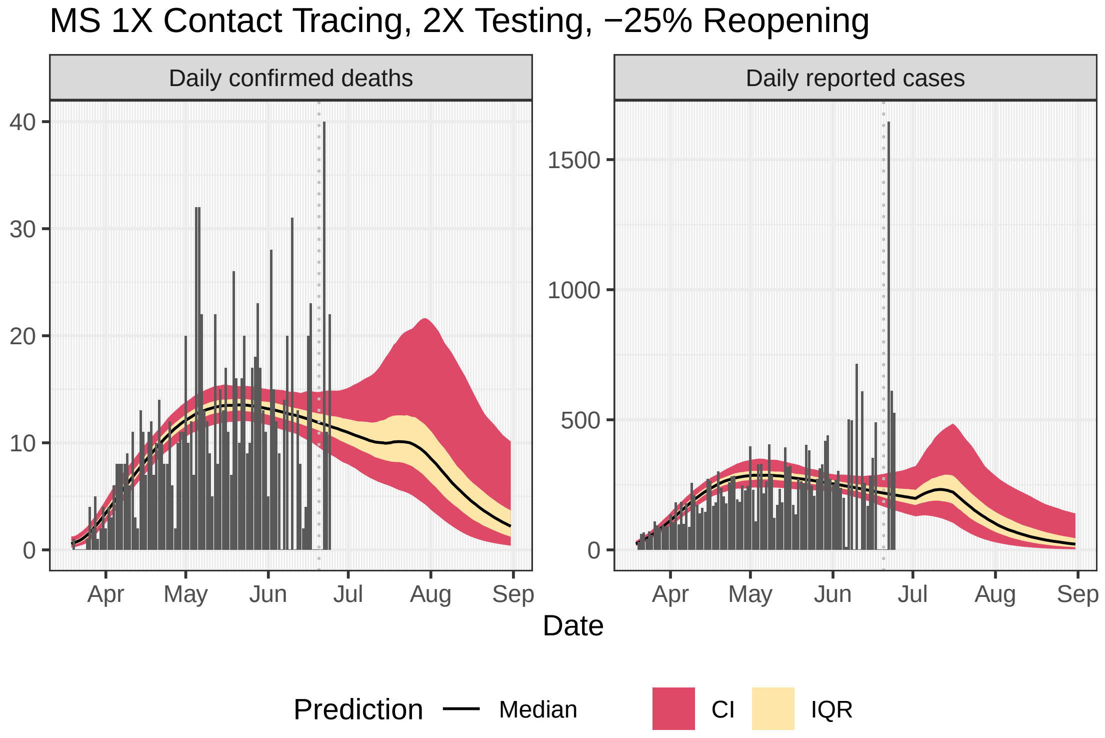

MS 1X Contact Tracing, 2X Testing, −25% Reopening
Daily confirmed deaths
Daily reported cases
0
10
20
30
40
0
500
1000
1500
Apr
May
Jun
Jul
Aug
Sep
Date
Prediction
Median
CI
IQR

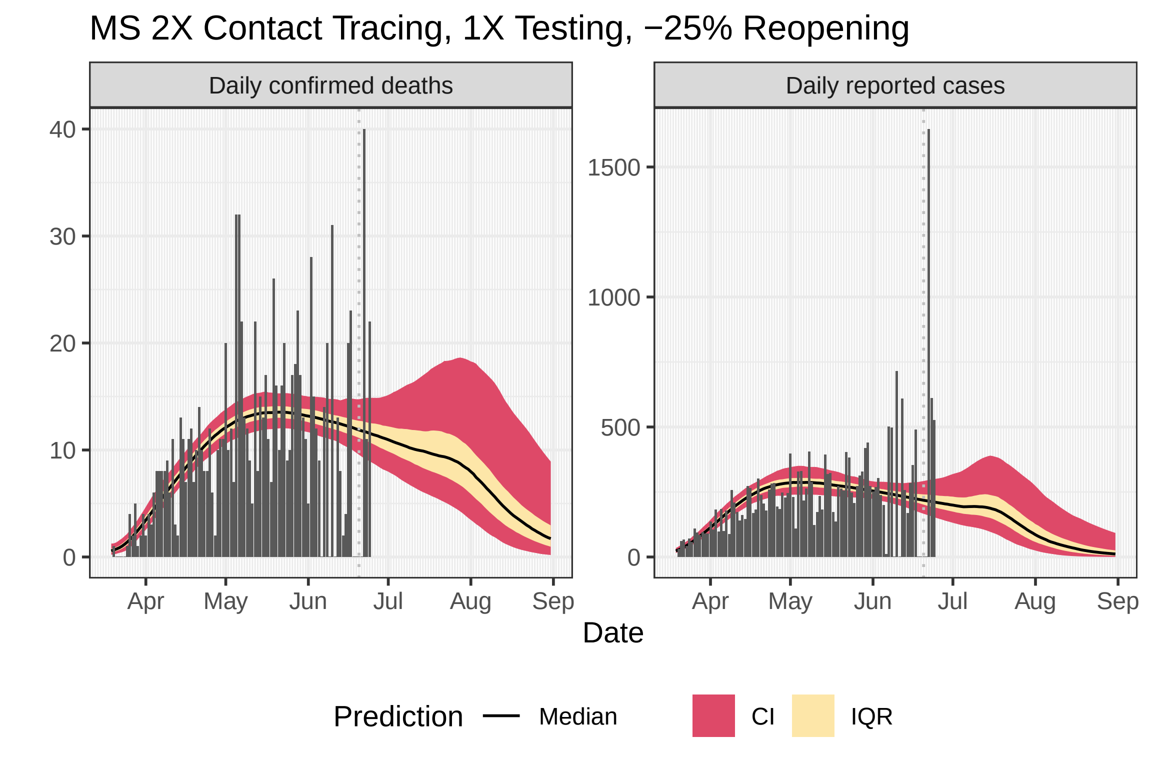
MS 2X Contact Tracing, 1X Testing, −25% Reopening
Daily confirmed deaths
Daily reported cases
40
30
20
10
0
1500
1000
500
0
Apr
May
Jun
Jul
Aug
Sep
Date
Prediction
Median
CI
IQR

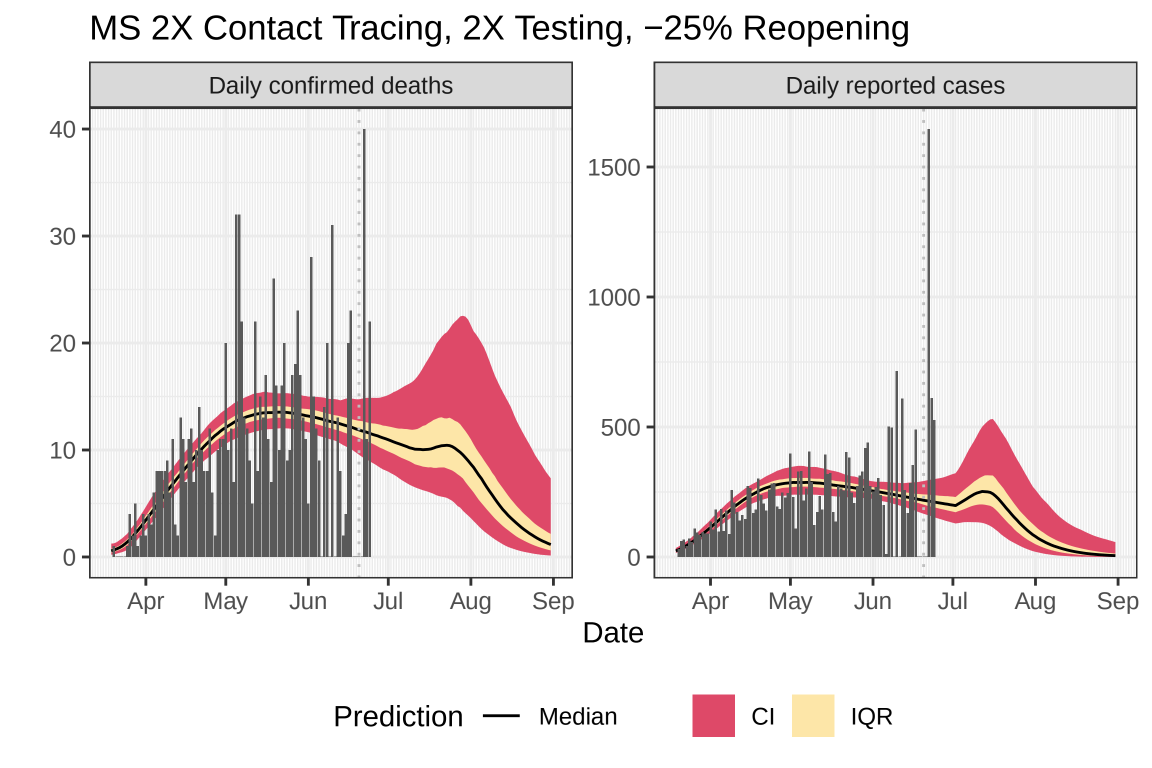
MS 2X Contact Tracing, 2X Testing, −25% Reopening
Daily confirmed deaths
Daily reported cases
40
30
20
10
0
1500
1000
500
0
Apr
May
Jun
Jul
Aug
Sep
Date
Prediction
Median
CI
IQR

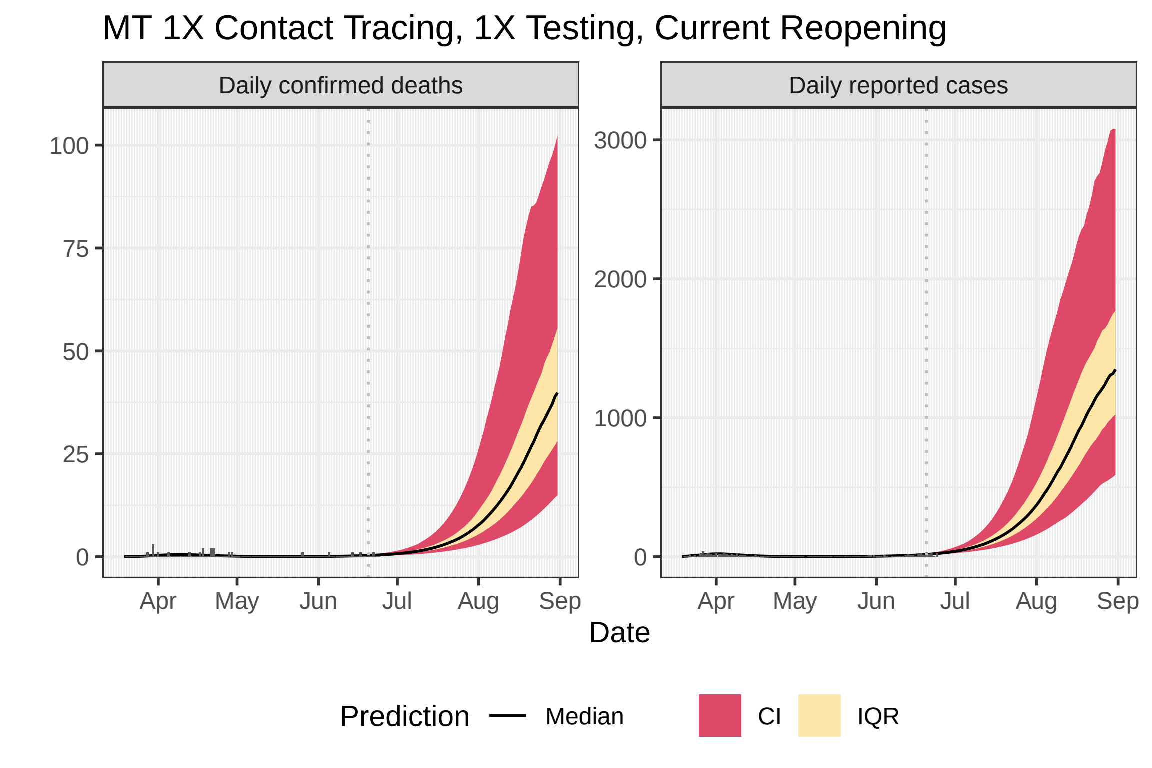

MT 1X Contact Tracing, 1X Testing, Current Reopening
Daily confirmed deaths
Daily reported cases
100
75
50
25
0
3000
2000
1000
0
Apr
May
Jun
Jul
Aug
Sep
Date
Prediction
Median
CI
IQR

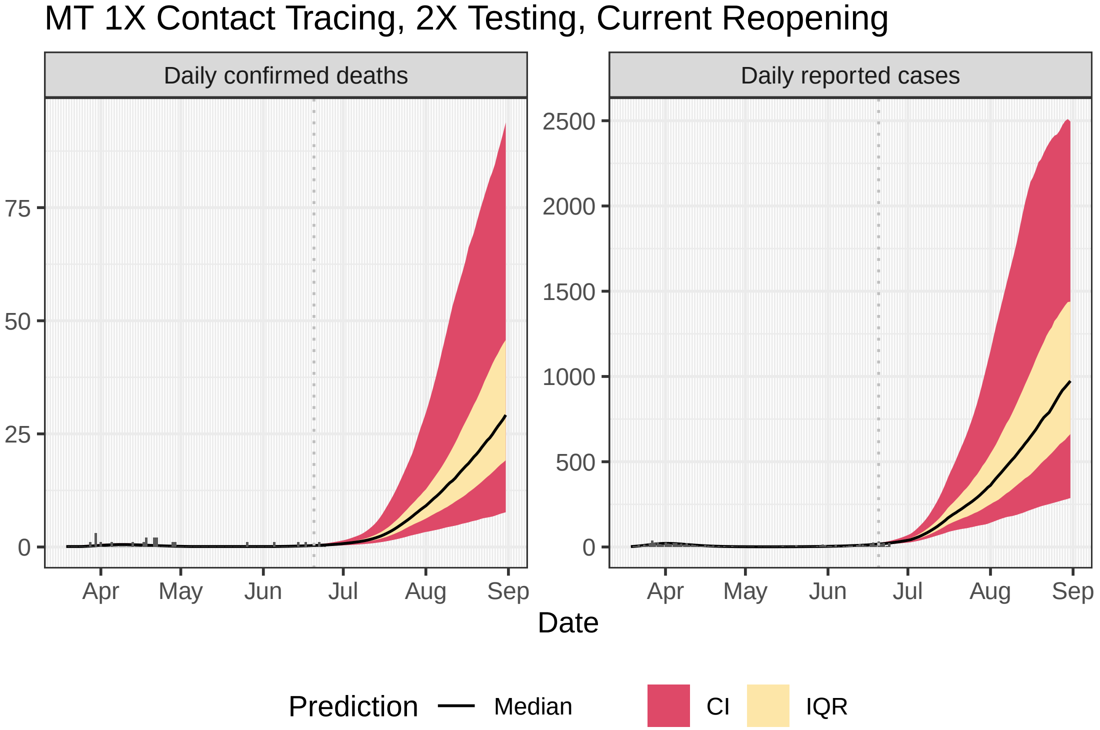

MT 1X Contact Tracing, 2X Testing, Current Reopening
Daily confirmed deaths
Daily reported cases
0
25
50
75
0
500
1000
1500
2000
2500
Apr
May
Jun
Jul
Aug
Sep
Date
Prediction
Median
CI
IQR

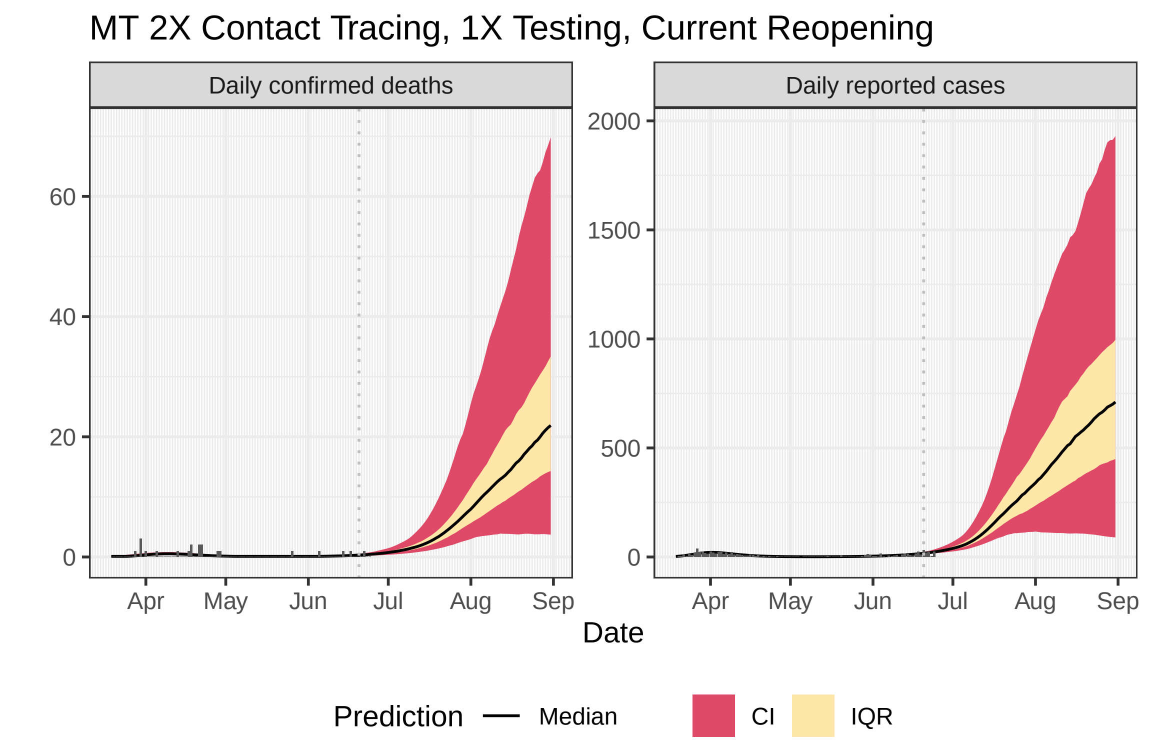
MT 2X Contact Tracing, 1X Testing, Current Reopening
Daily confirmed deaths
Daily reported cases
60
40
20
0
2000
1500
1000
500
0
Apr
May
Jun
Jul
Aug
Sep
Date
Prediction
Median
CI
IQR

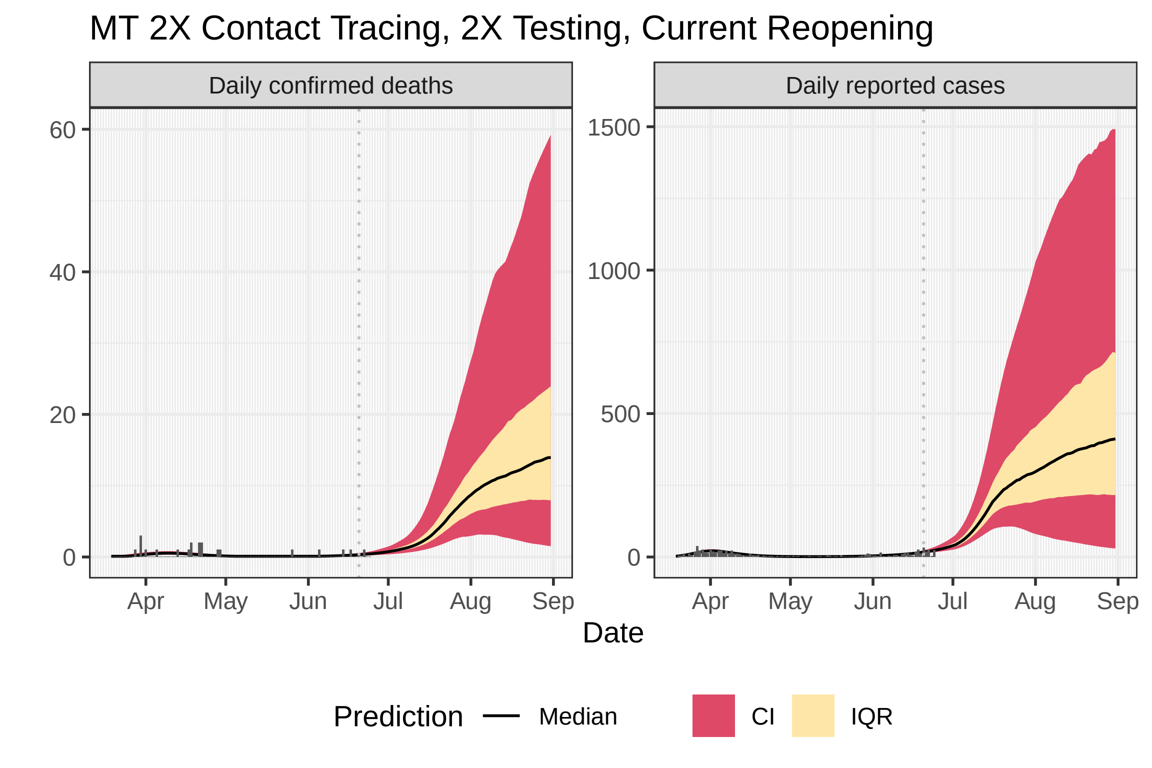
MT 2X Contact Tracing, 2X Testing, Current Reopening
Daily confirmed deaths
Daily reported cases
60
40
20
0
1500
1000
500
0
Apr
May
Jun
Jul
Aug
Sep
Date
Prediction
Median
CI
IQR

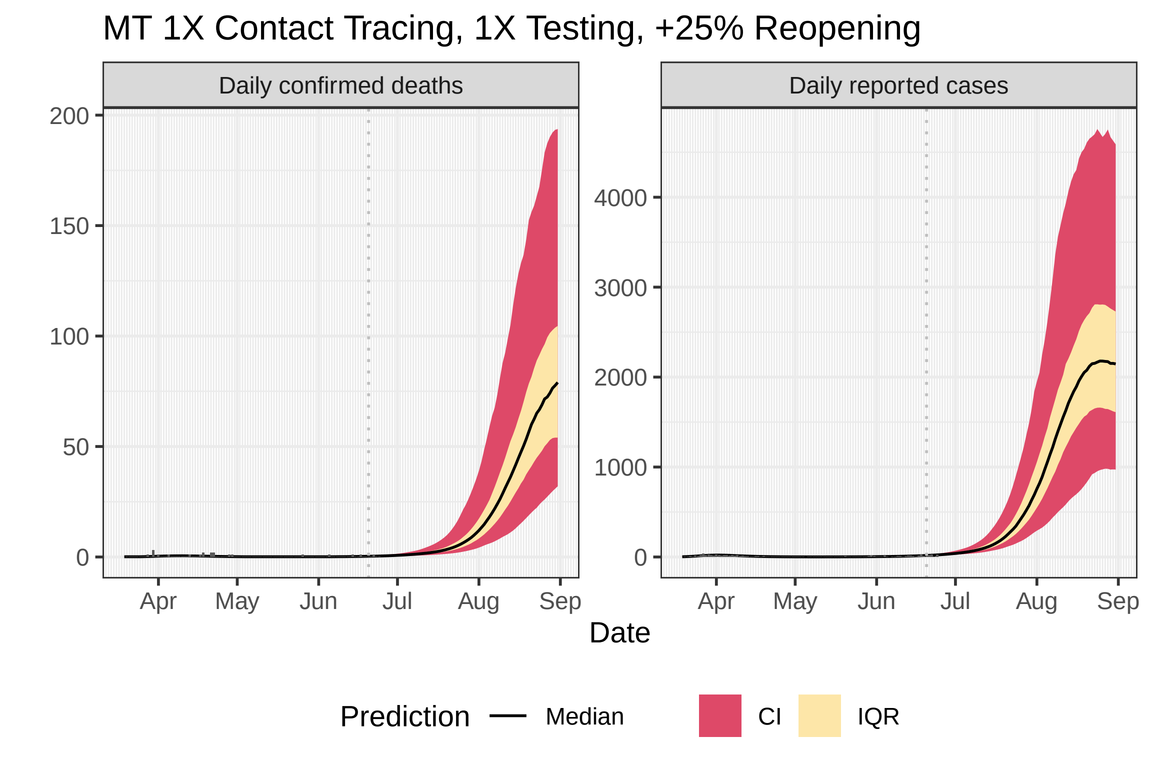
MT 1X Contact Tracing, 1X Testing, +25% Reopening
Daily confirmed deaths
Daily reported cases
200
150
100
50
0
4000
3000
2000
1000
0
Apr
May
Jun
Jul
Aug
Sep
Date
Prediction
Median
CI
IQR

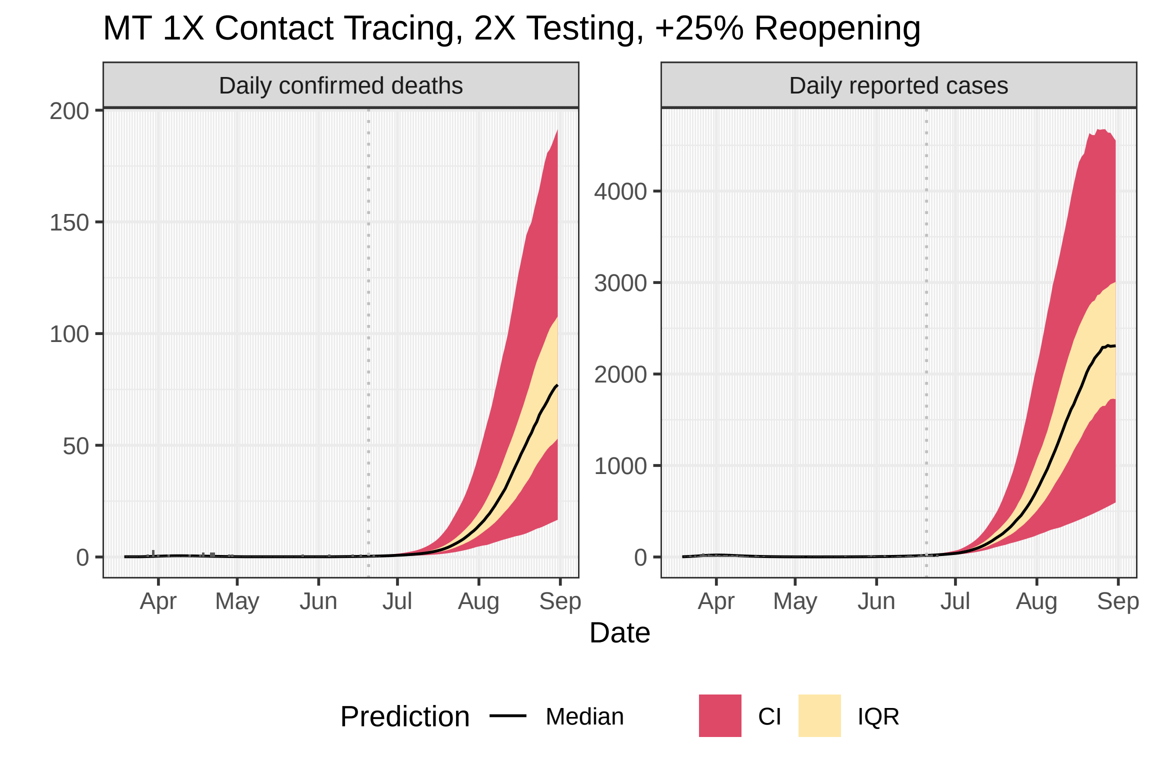

MT 1X Contact Tracing, 2X Testing, +25% Reopening
Daily confirmed deaths
Daily reported cases
200
150
100
50
0
4000
3000
2000
1000
0
Apr
May
Jun
Jul
Aug
Sep
Date
Prediction
Median
CI
IQR

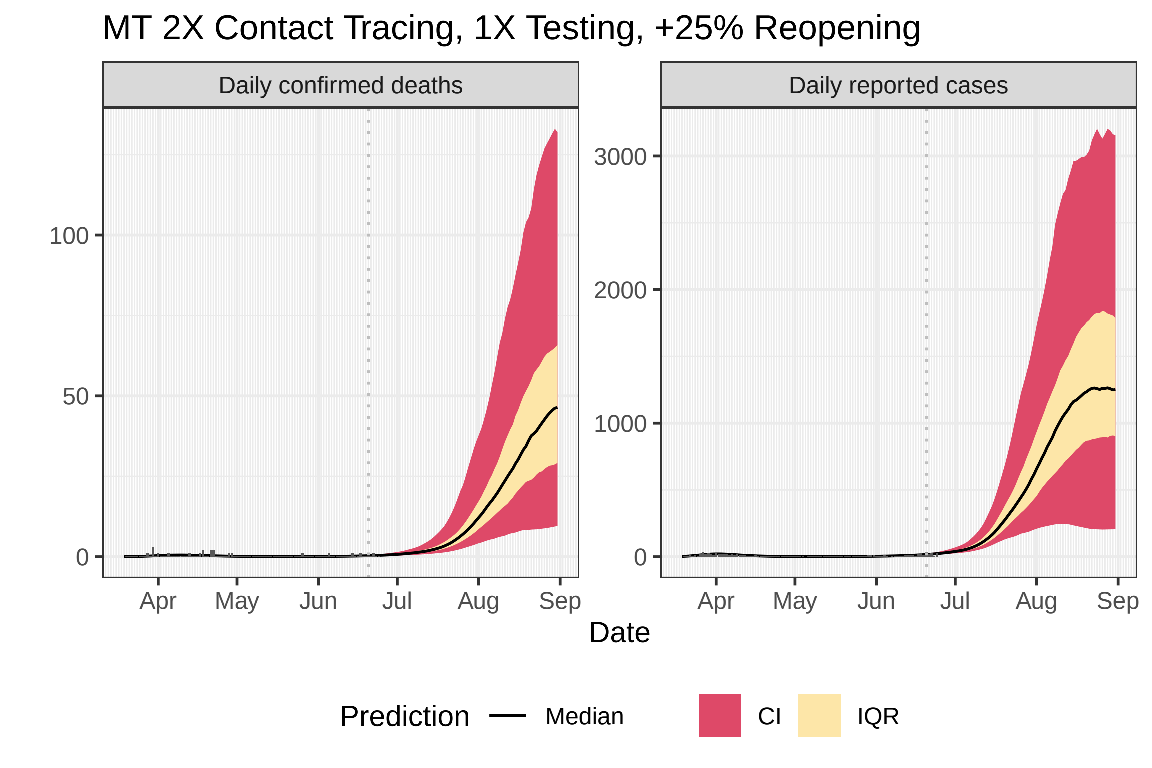

MT 2X Contact Tracing, 1X Testing, +25% Reopening
Daily confirmed deaths
Daily reported cases
100
50
0
3000
2000
1000
0
Apr
May
Jun
Jul
Aug
Sep
Date
Prediction
Median
CI
IQR

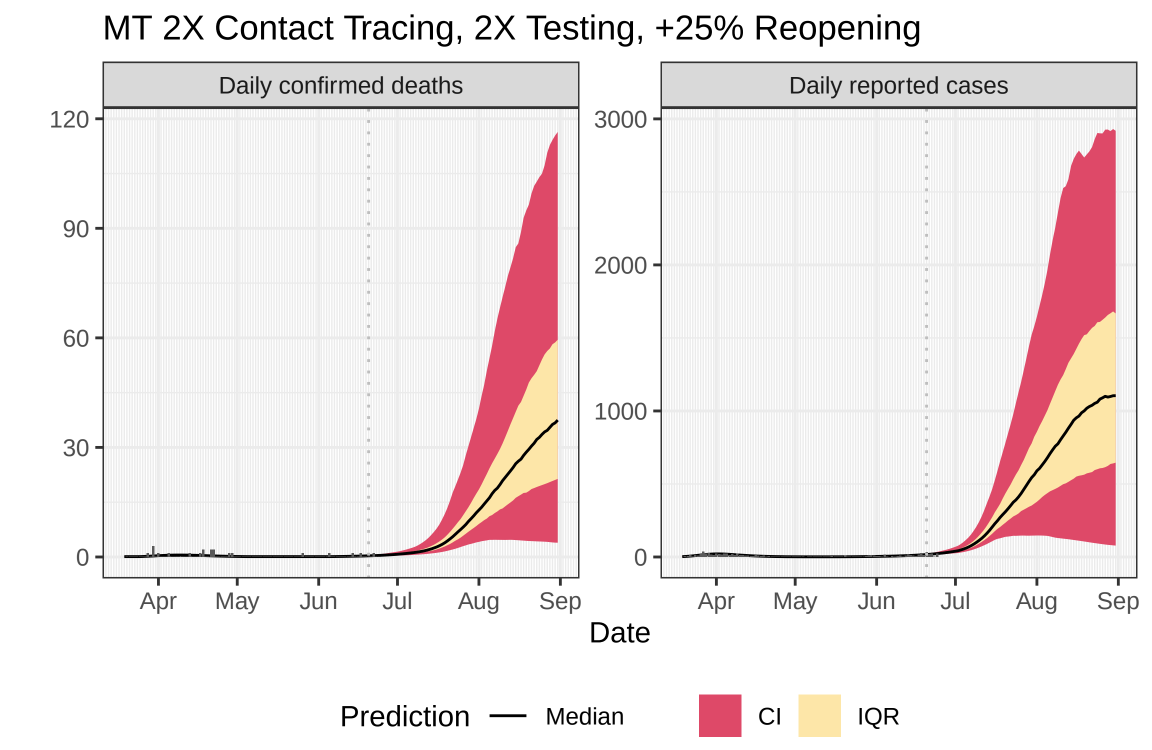

MT 2X Contact Tracing, 2X Testing, +25% Reopening
Daily confirmed deaths
Daily reported cases
120
90
60
30
0
3000
2000
1000
0
Apr
May
Jun
Jul
Aug
Sep
Date
Prediction
Median
CI
IQR

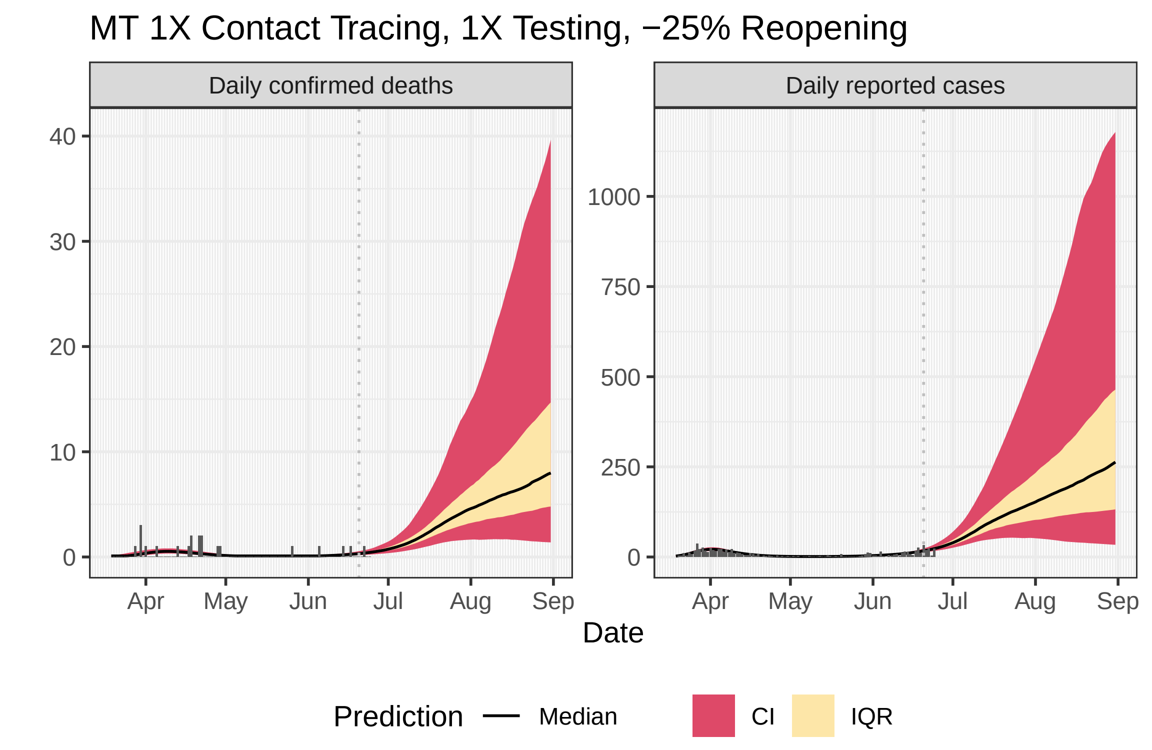

MT 1X Contact Tracing, 1X Testing, −25% Reopening
Daily confirmed deaths
Daily reported cases
40
30
20
10
0
1000
750
500
250
0
Apr
May
Jun
Jul
Aug
Sep
Date
Prediction
Median
CI
IQR

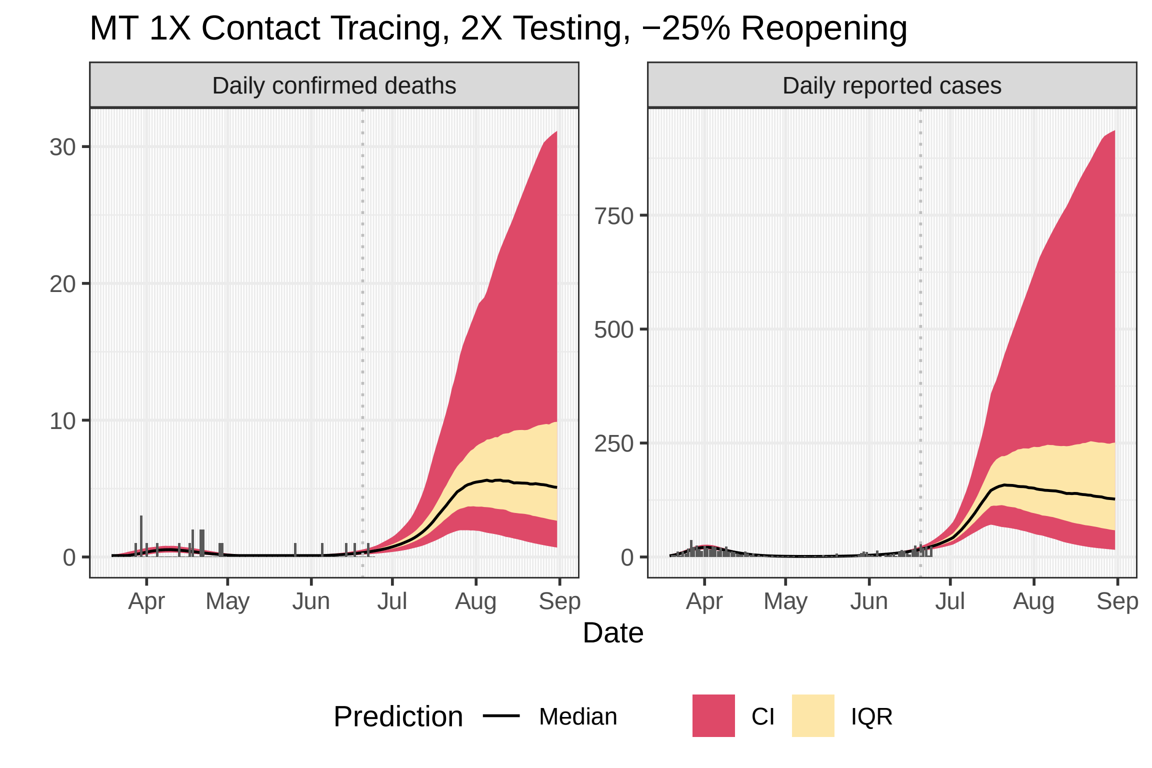

MT 1X Contact Tracing, 2X Testing, −25% Reopening
Daily confirmed deaths
Daily reported cases
30
20
10
0
750
500
250
0
Apr
May
Jun
Jul
Aug
Sep
Date
Prediction
Median
CI
IQR

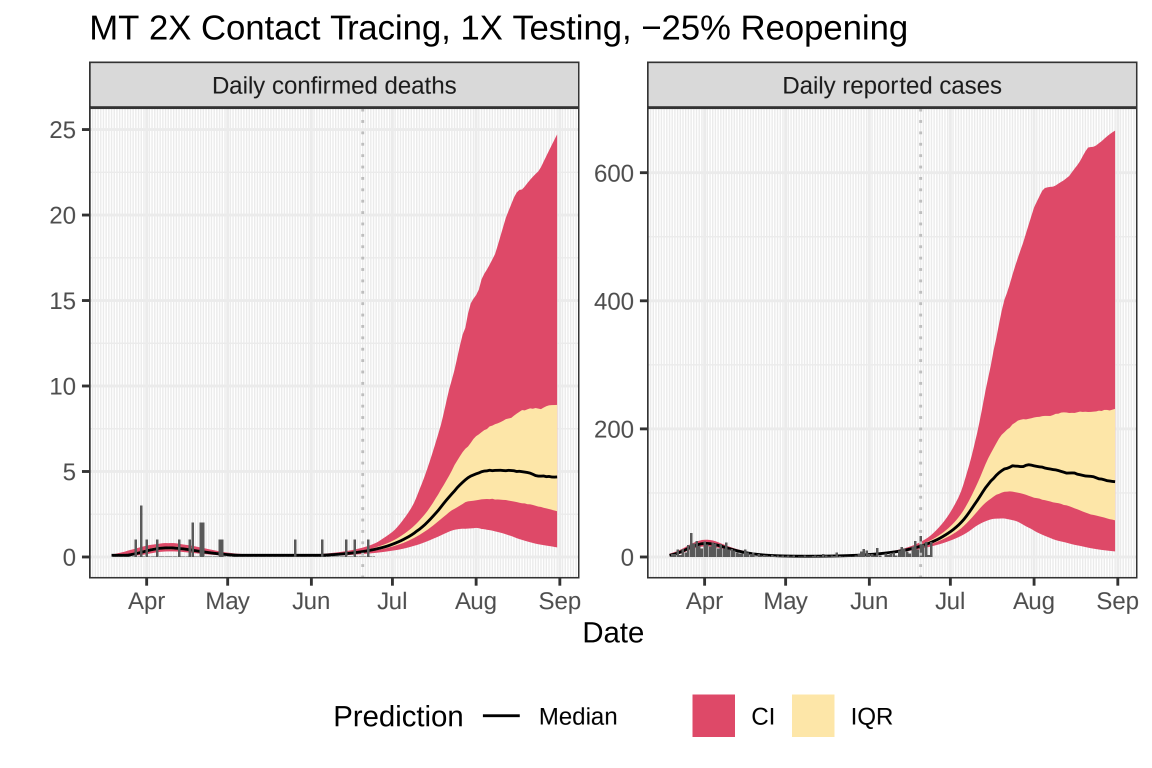
MT 2X Contact Tracing, 1X Testing, −25% Reopening
Daily confirmed deaths
Daily reported cases
25
20
15
10
5
0
600
400
200
0
Apr
May
Jun
Jul
Aug
Sep
Date
Prediction
Median
CI
IQR

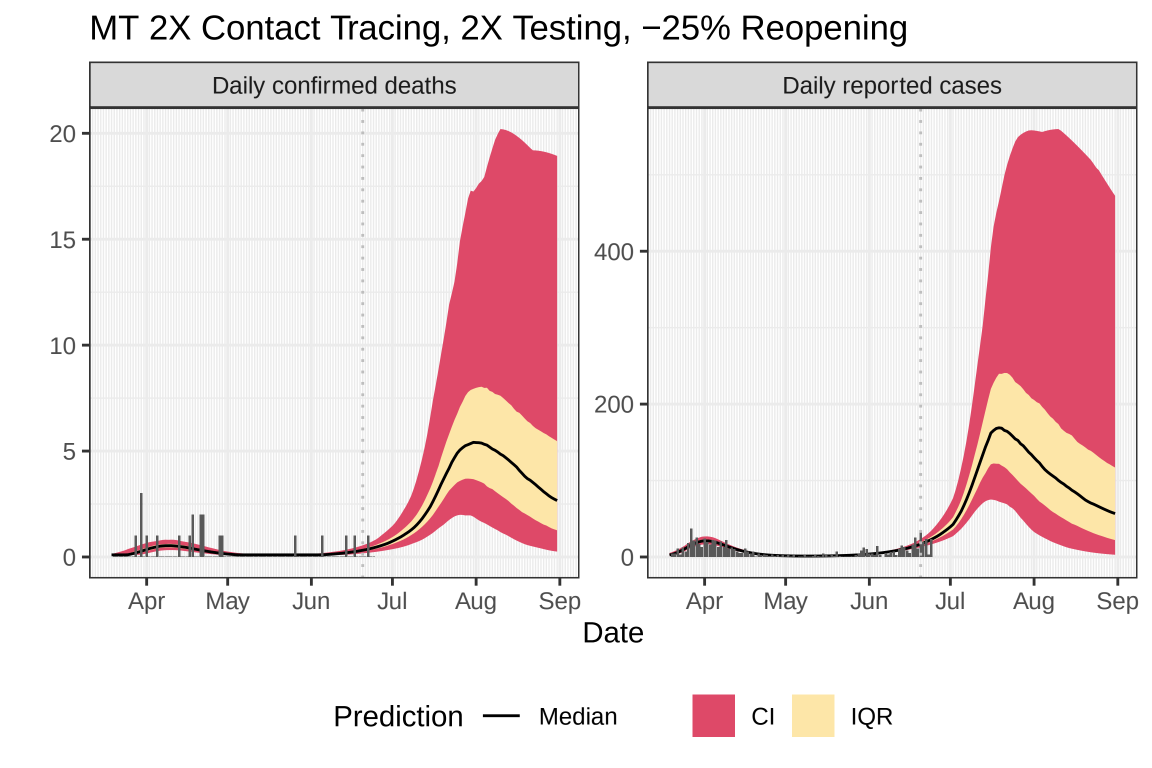

MT 2X Contact Tracing, 2X Testing, −25% Reopening
Daily confirmed deaths
Daily reported cases
20
15
10
5
0
400
200
0
Apr
May
Jun
Jul
Aug
Sep
Date
Prediction
Median
CI
IQR

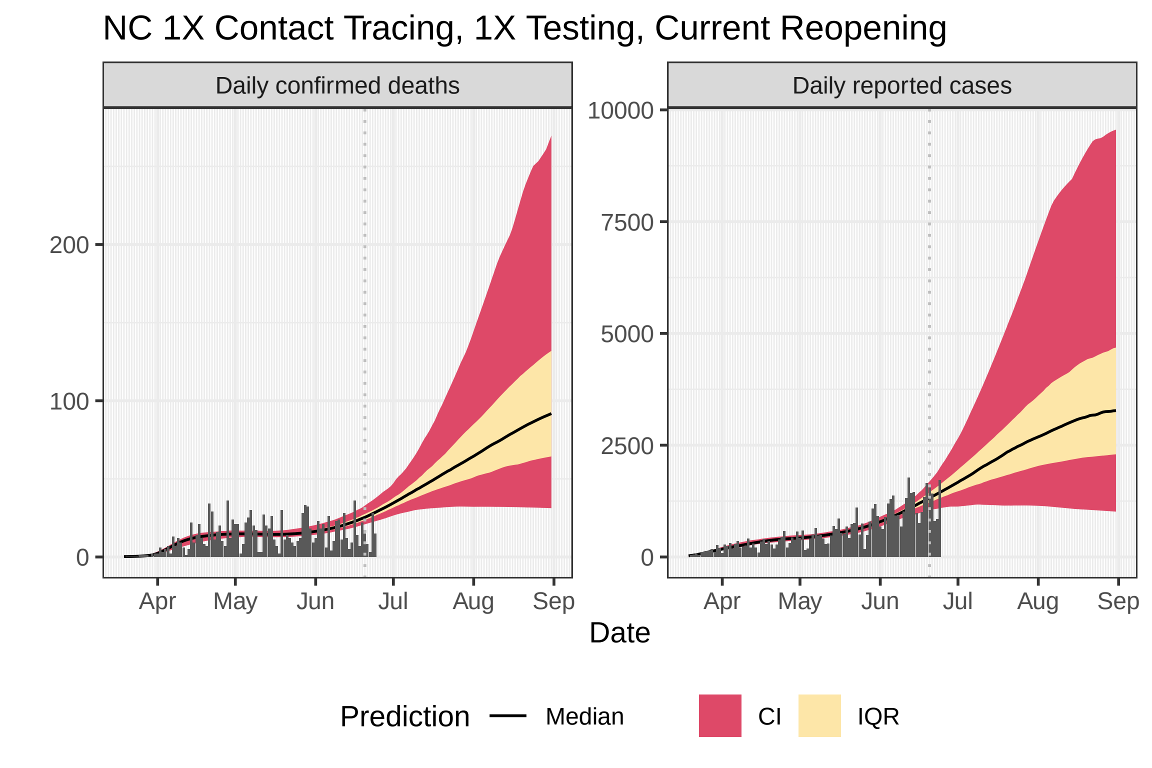

NC 1X Contact Tracing, 1X Testing, Current Reopening
Daily confirmed deaths
Daily reported cases
200
100
0
10000
7500
5000
2500
0
Apr
May
Jun
Jul
Aug
Sep
Date
Prediction
Median
CI
IQR

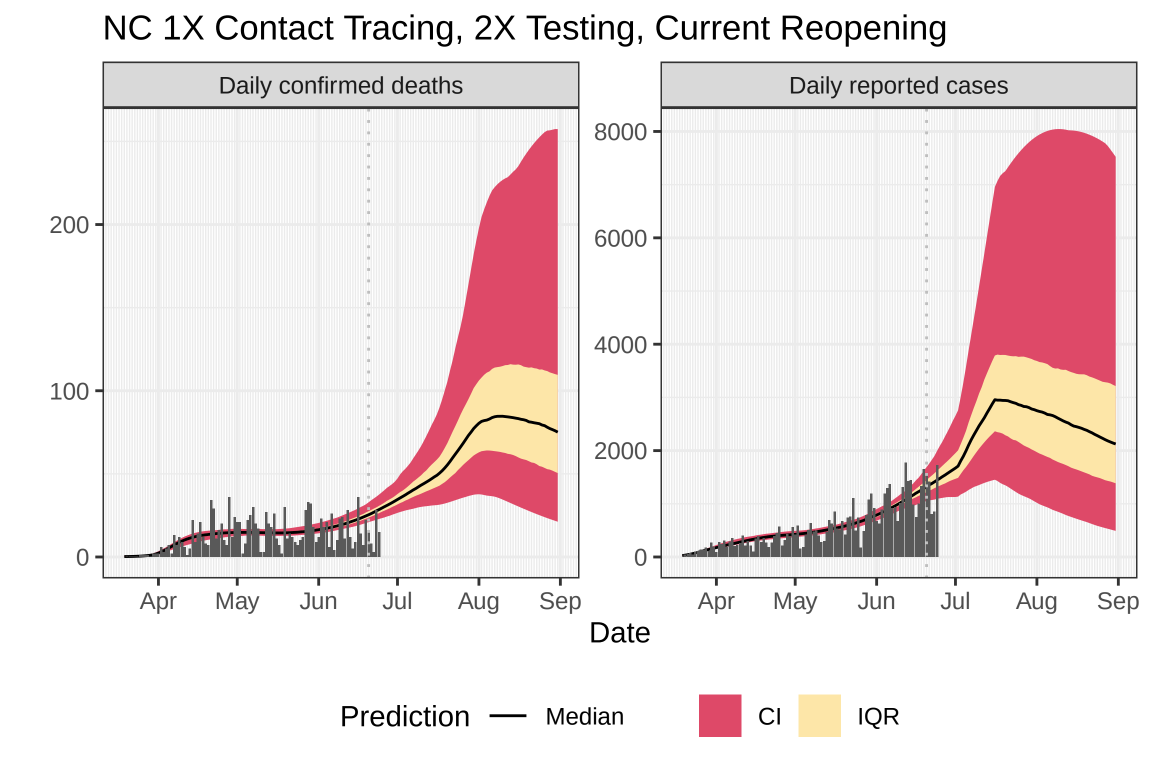
NC 1X Contact Tracing, 2X Testing, Current Reopening
Daily confirmed deaths
Daily reported cases
0
100
200
0
2000
4000
6000
8000
Apr
May
Jun
Jul
Aug
Sep
Date
Prediction
Median
CI
IQR

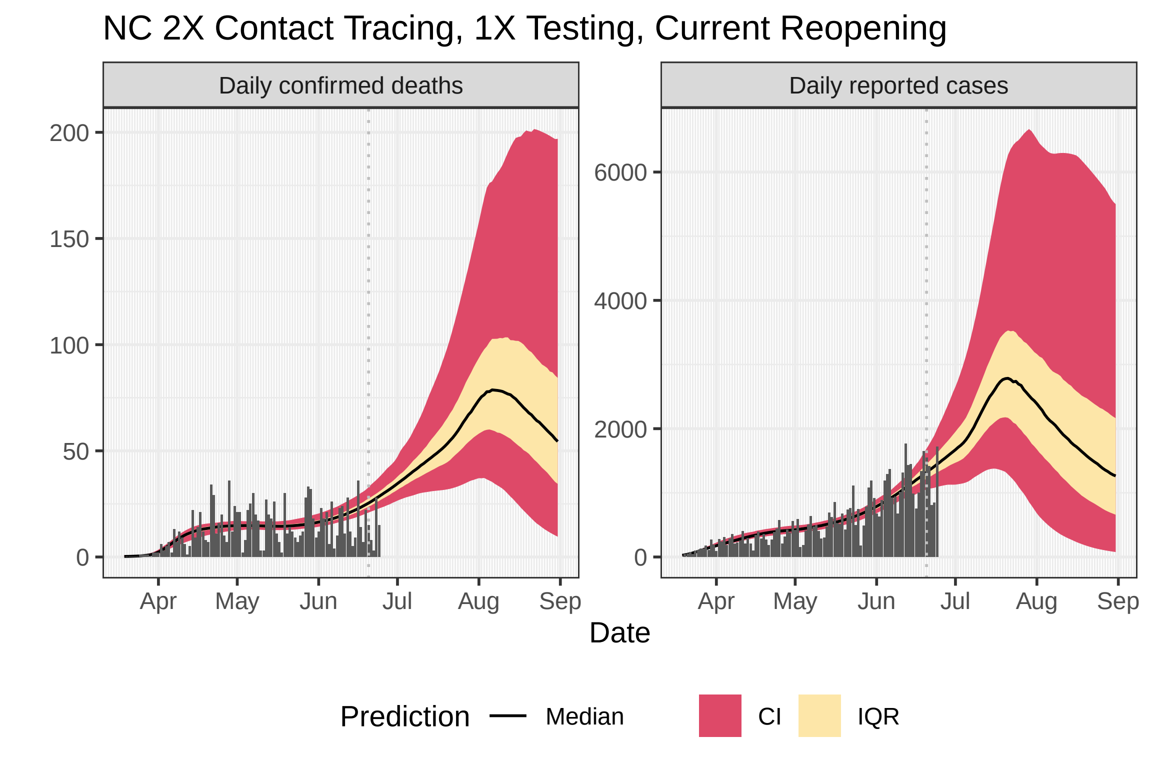
NC 2X Contact Tracing, 1X Testing, Current Reopening
Daily confirmed deaths
Daily reported cases
200
150
100
50
0
6000
4000
2000
0
Apr
May
Jun
Jul
Aug
Sep
Date
Prediction
Median
CI
IQR

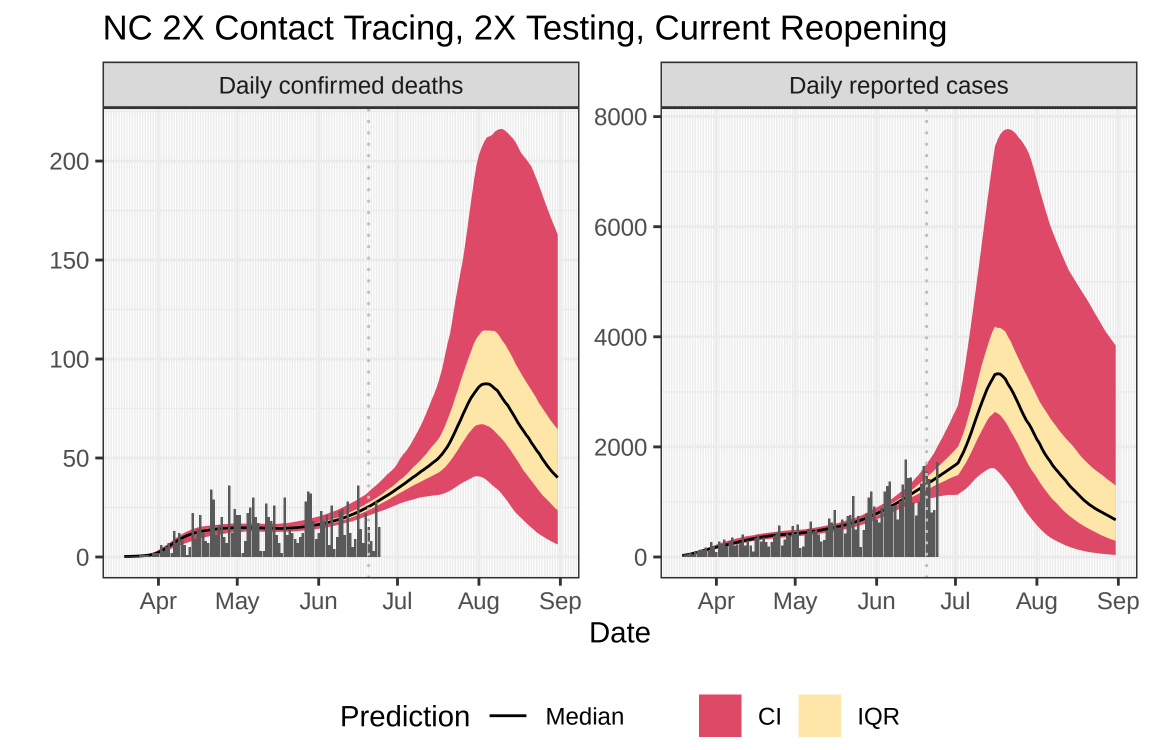

NC 2X Contact Tracing, 2X Testing, Current Reopening
Daily confirmed deaths
Daily reported cases
200
150
100
50
0
8000
6000
4000
2000
0
Apr
May
Jun
Jul
Aug
Sep
Date
Prediction
Median
CI
IQR

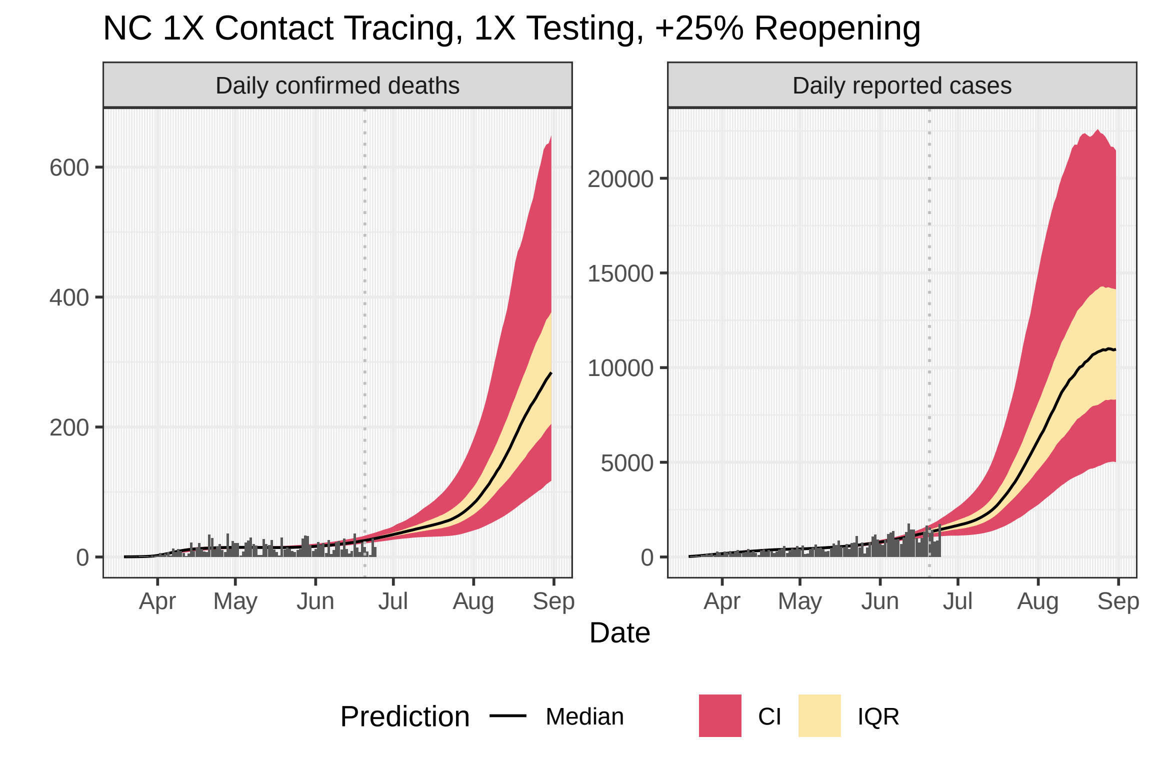

NC 1X Contact Tracing, 1X Testing, +25% Reopening
Daily confirmed deaths
Daily reported cases
600
400
200
0
20000
15000
10000
5000
0
Apr
May
Jun
Jul
Aug
Sep
Date
Prediction
Median
CI
IQR

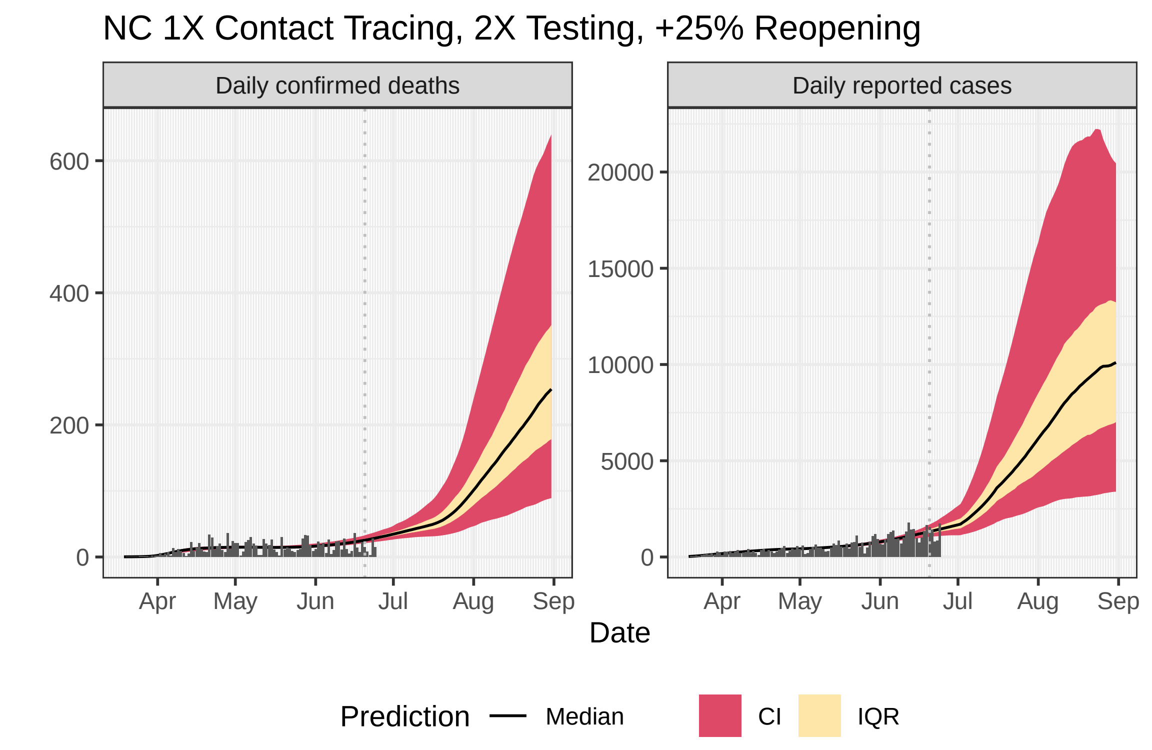
NC 1X Contact Tracing, 2X Testing, +25% Reopening
Daily confirmed deaths
Daily reported cases
600
400
200
0
20000
15000
10000
5000
0
Apr
May
Jun
Jul
Aug
Sep
Date
Prediction
Median
CI
IQR

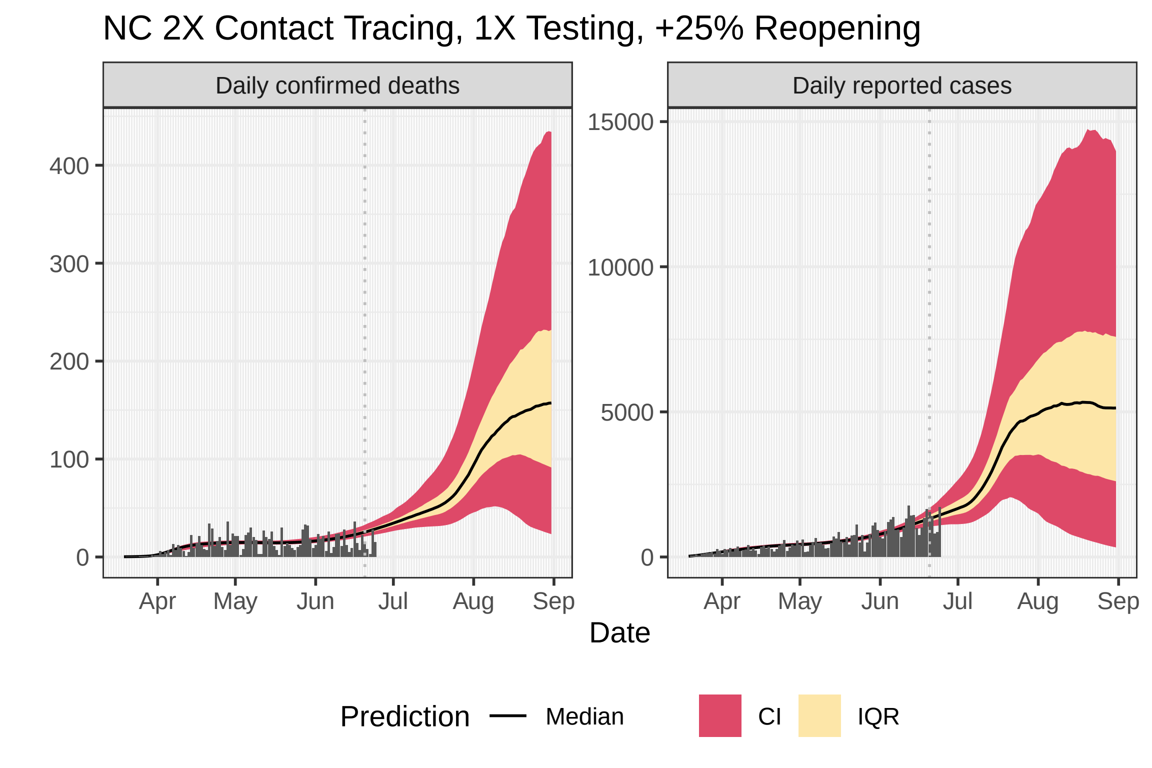
NC 2X Contact Tracing, 1X Testing, +25% Reopening
Daily confirmed deaths
Daily reported cases
400
300
200
100
0
15000
10000
5000
0
Apr
May
Jun
Jul
Aug
Sep
Date
Prediction
Median
CI
IQR

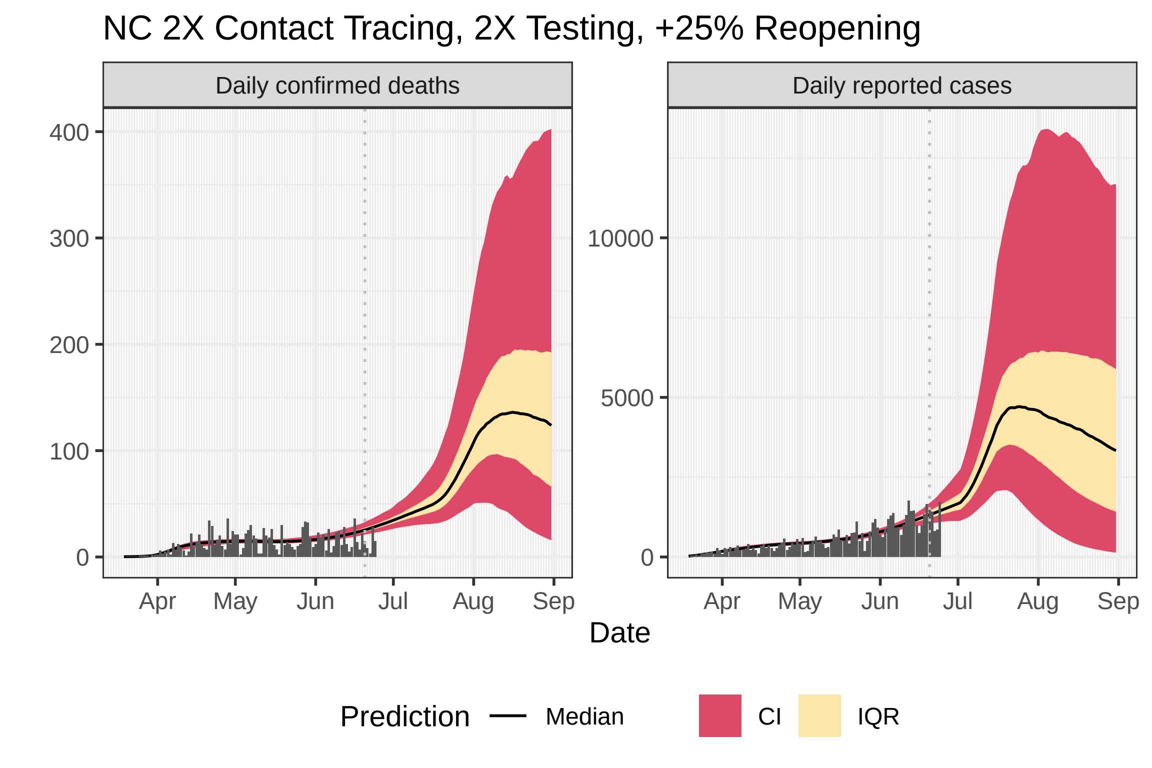
NC 2X Contact Tracing, 2X Testing, +25% Reopening
Daily confirmed deaths
Daily reported cases
400
300
200
100
0
10000
5000
0
Apr
May
Jun
Jul
Aug
Sep
Date
Prediction
Median
CI
IQR

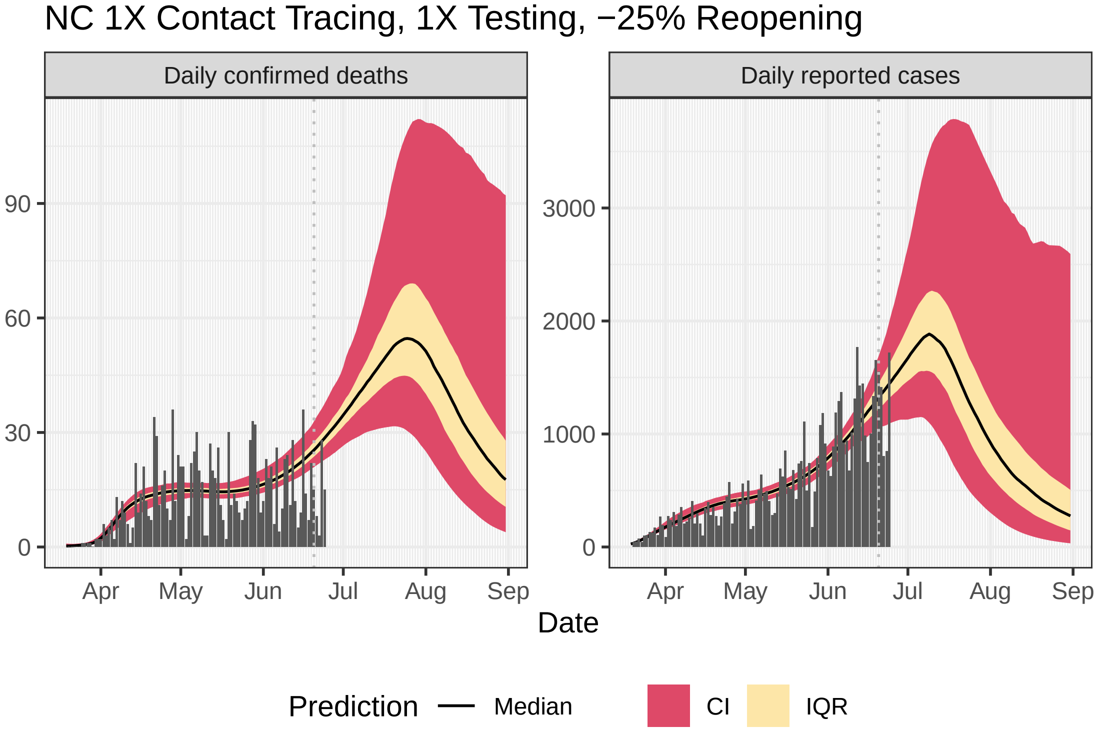
NC 1X Contact Tracing, 1X Testing, −25% Reopening
Daily confirmed deaths
Daily reported cases
0
30
60
90
0
1000
2000
3000
Apr
May
Jun
Jul
Aug
Sep
Date
Prediction
Median
CI
IQR

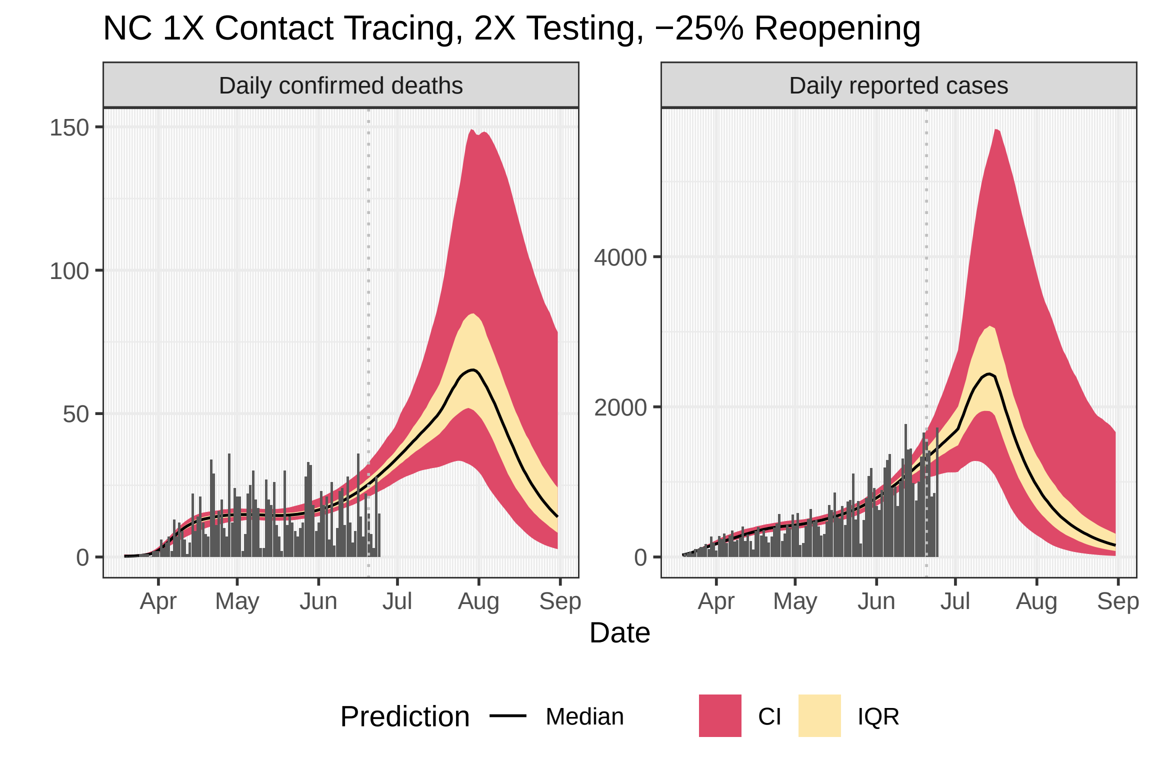
NC 1X Contact Tracing, 2X Testing, −25% Reopening
Daily confirmed deaths
Daily reported cases
150
100
50
0
4000
2000
0
Apr
May
Jun
Jul
Aug
Sep
Date
Prediction
Median
CI
IQR

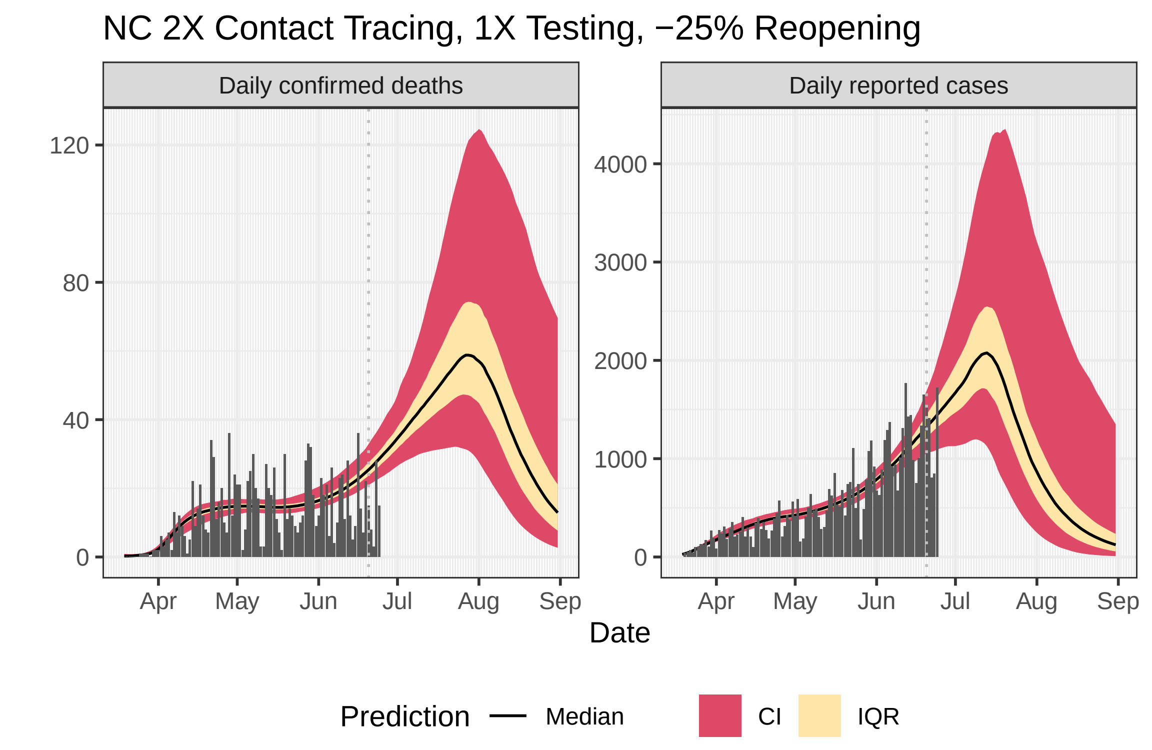

NC 2X Contact Tracing, 1X Testing, −25% Reopening
Daily confirmed deaths
Daily reported cases
120
80
40
0
4000
3000
2000
1000
0
Apr
May
Jun
Jul
Aug
Sep
Date
Prediction
Median
CI
IQR

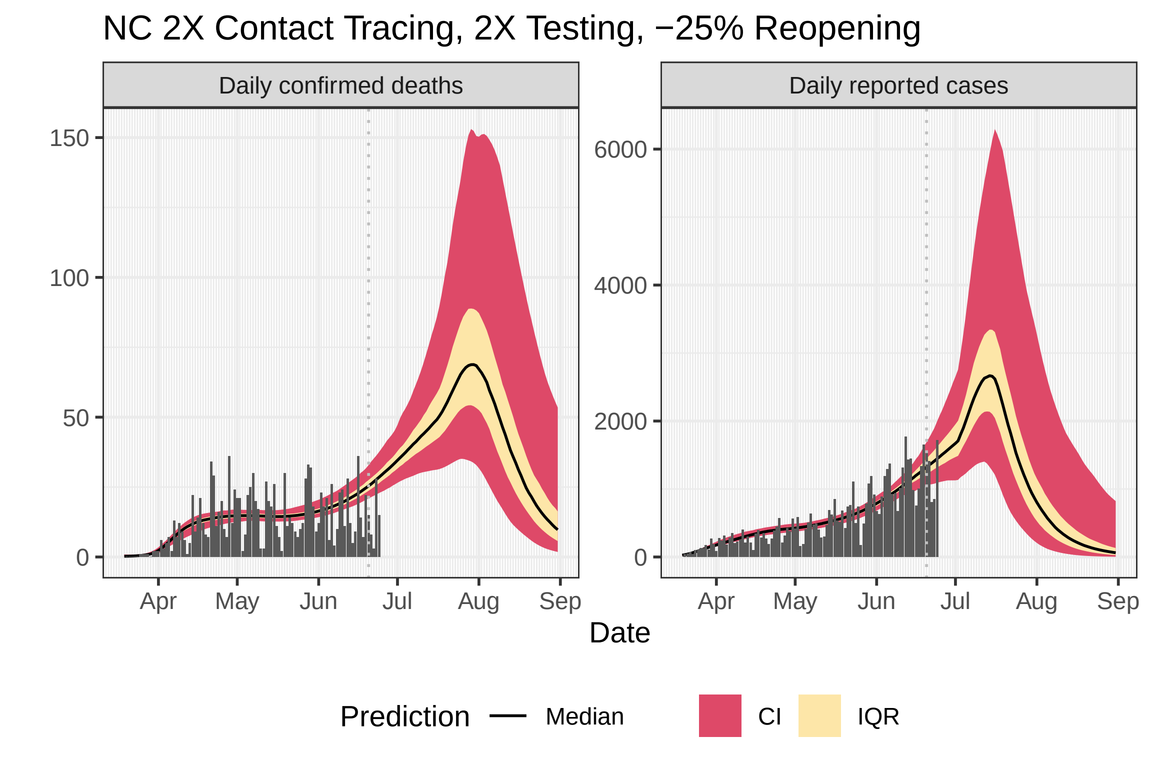
NC 2X Contact Tracing, 2X Testing, −25% Reopening
Daily confirmed deaths
Daily reported cases
150
100
50
0
6000
4000
2000
0
Apr
May
Jun
Jul
Aug
Sep
Date
Prediction
Median
CI
IQR

ND 1X Contact Tracing, 1X Testing, Current Reopening

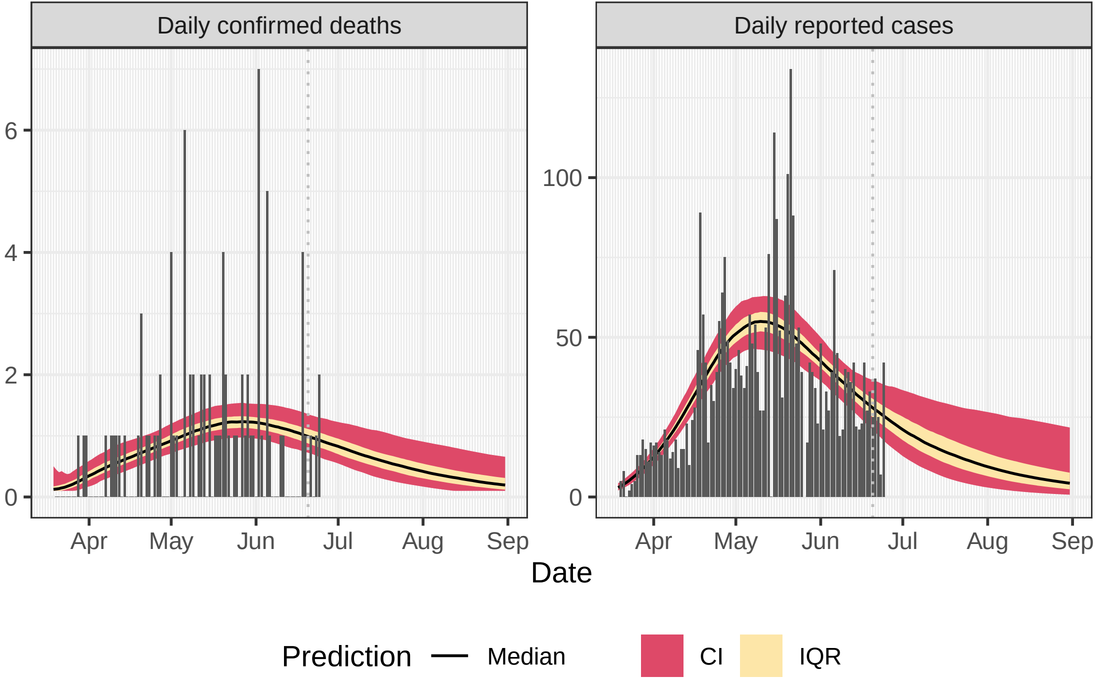

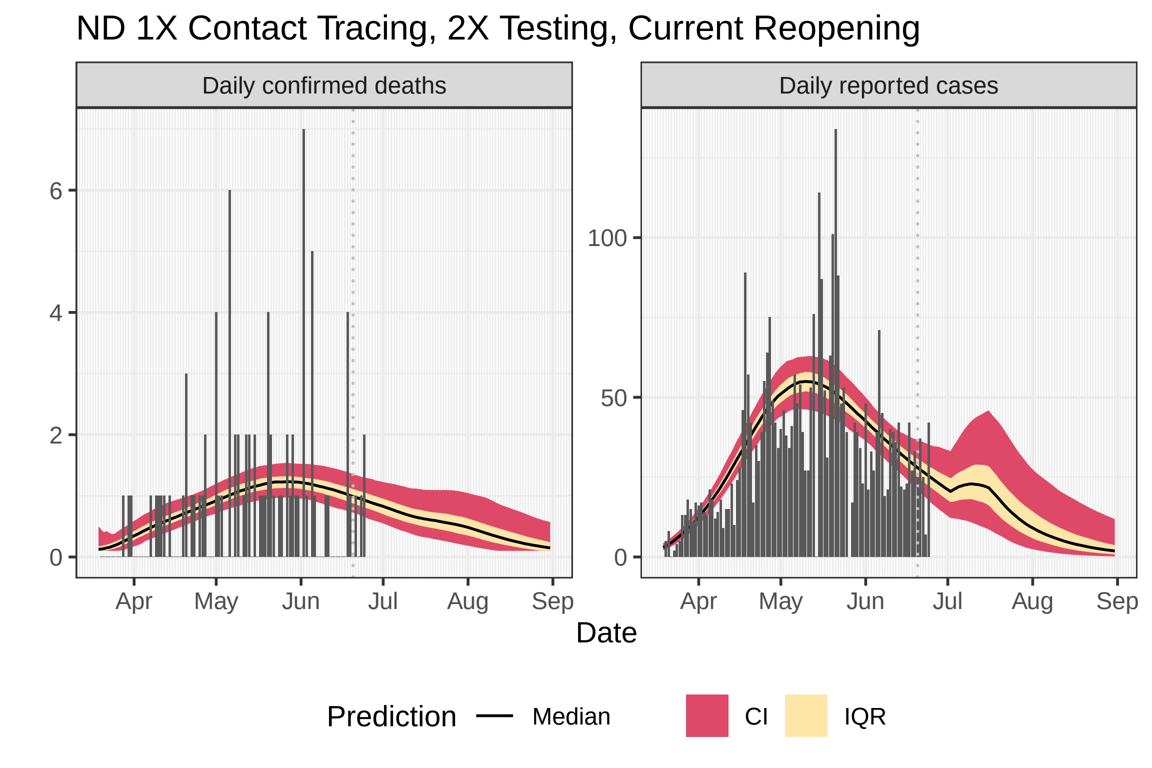
ND 1X Contact Tracing, 2X Testing, Current Reopening
Daily confirmed deaths
Daily reported cases
0
2
4
6
0
50
100
Apr
May
Jun
Jul
Aug
Sep
Date
Prediction
Median
CI
IQR

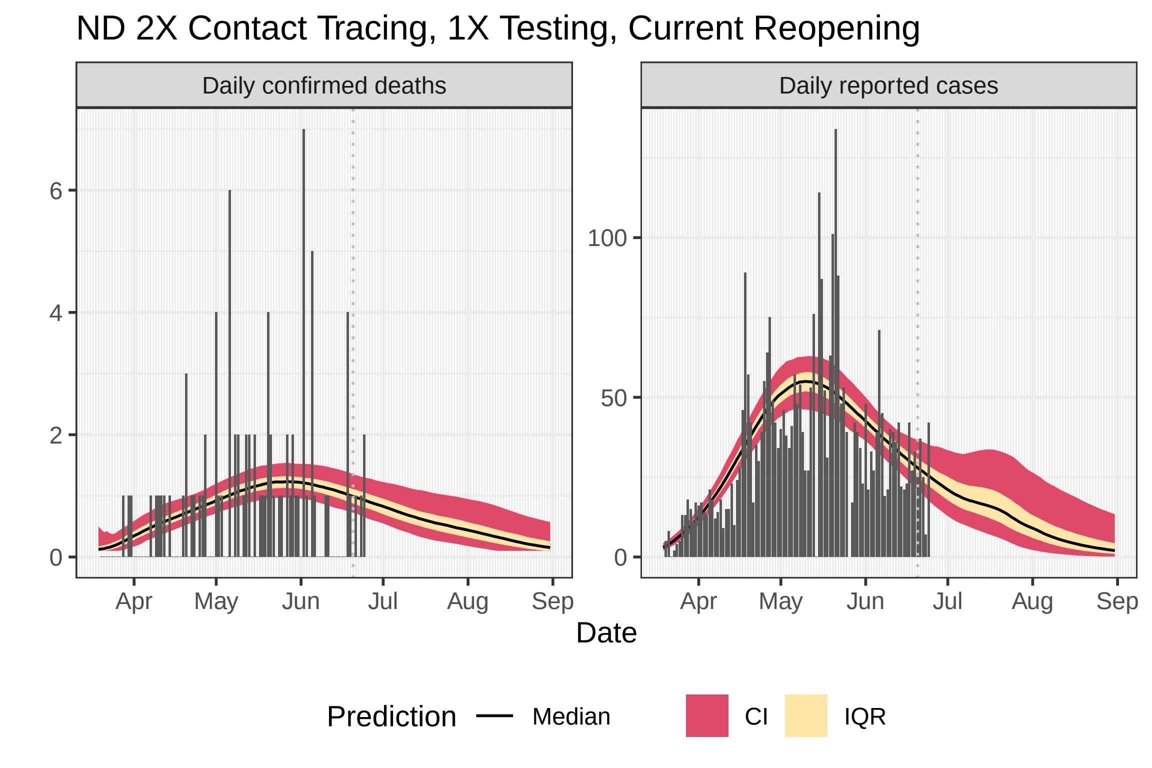
ND 2X Contact Tracing, 1X Testing, Current Reopening
Daily confirmed deaths
Daily reported cases
0
2
4
6
0
50
100
Apr
May
Jun
Jul
Aug
Sep
Apr
May
Jun
Jul
Aug
Sep
Date
Prediction
Median
CI
IQR

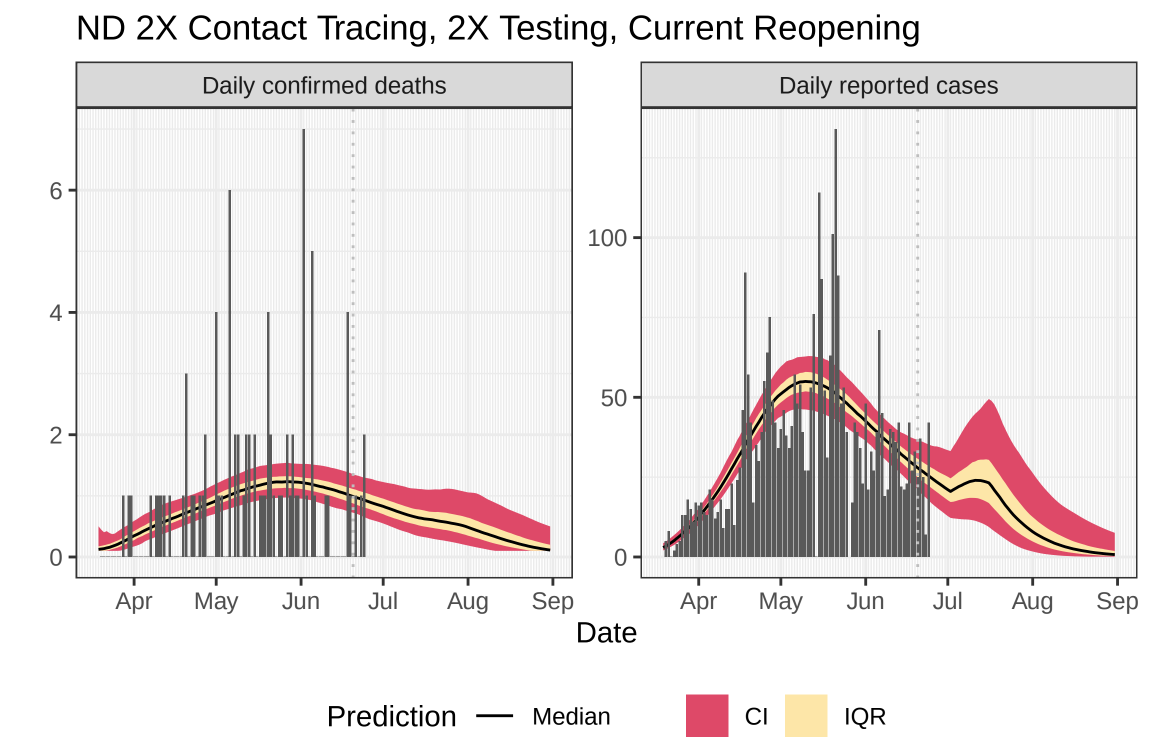

ND 2X Contact Tracing, 2X Testing, Current Reopening
Daily confirmed deaths
Daily reported cases
0
2
4
6
0
50
100
Apr
May
Jun
Jul
Aug
Sep
Date
Prediction
Median
CI
IQR

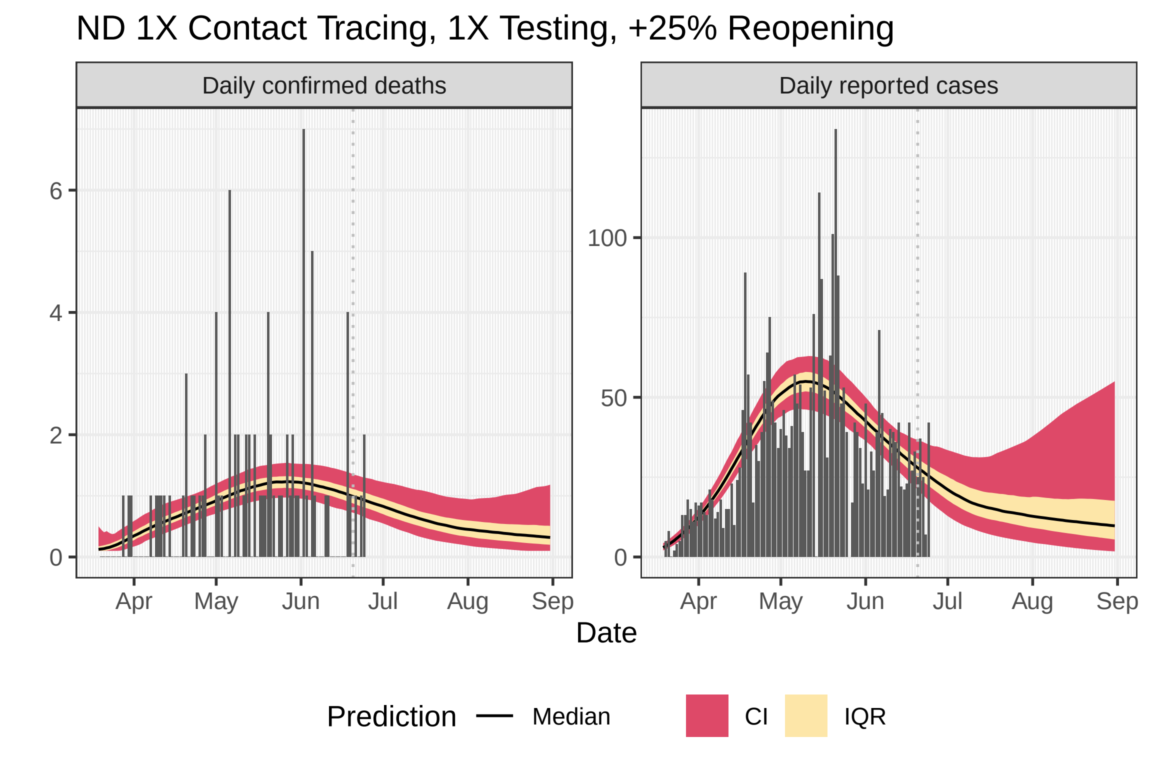
ND 1X Contact Tracing, 1X Testing, +25% Reopening
Daily confirmed deaths
Daily reported cases
0
2
4
6
0
50
100
Apr
May
Jun
Jul
Aug
Sep
Date
Prediction
Median
CI
IQR

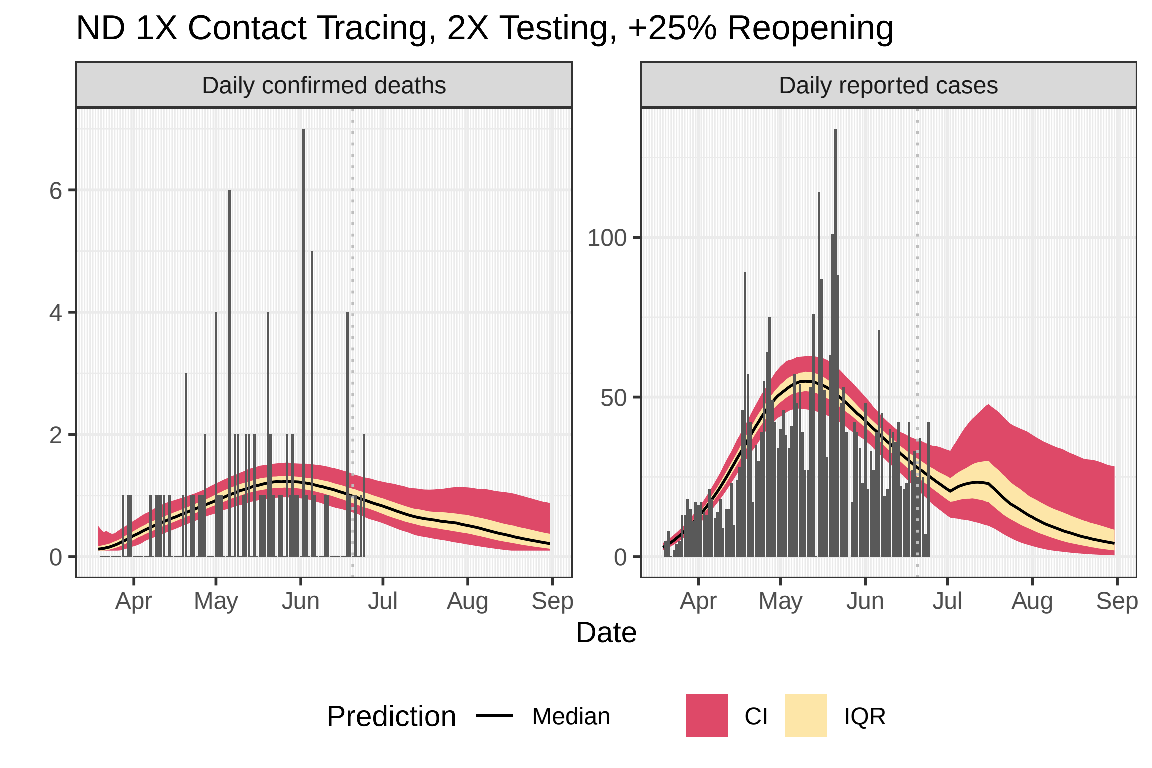
ND 1X Contact Tracing, 2X Testing, +25% Reopening
Daily confirmed deaths
Daily reported cases
0
2
4
6
0
50
100
Apr
May
Jun
Jul
Aug
Sep
Date
Prediction
Median
CI
IQR

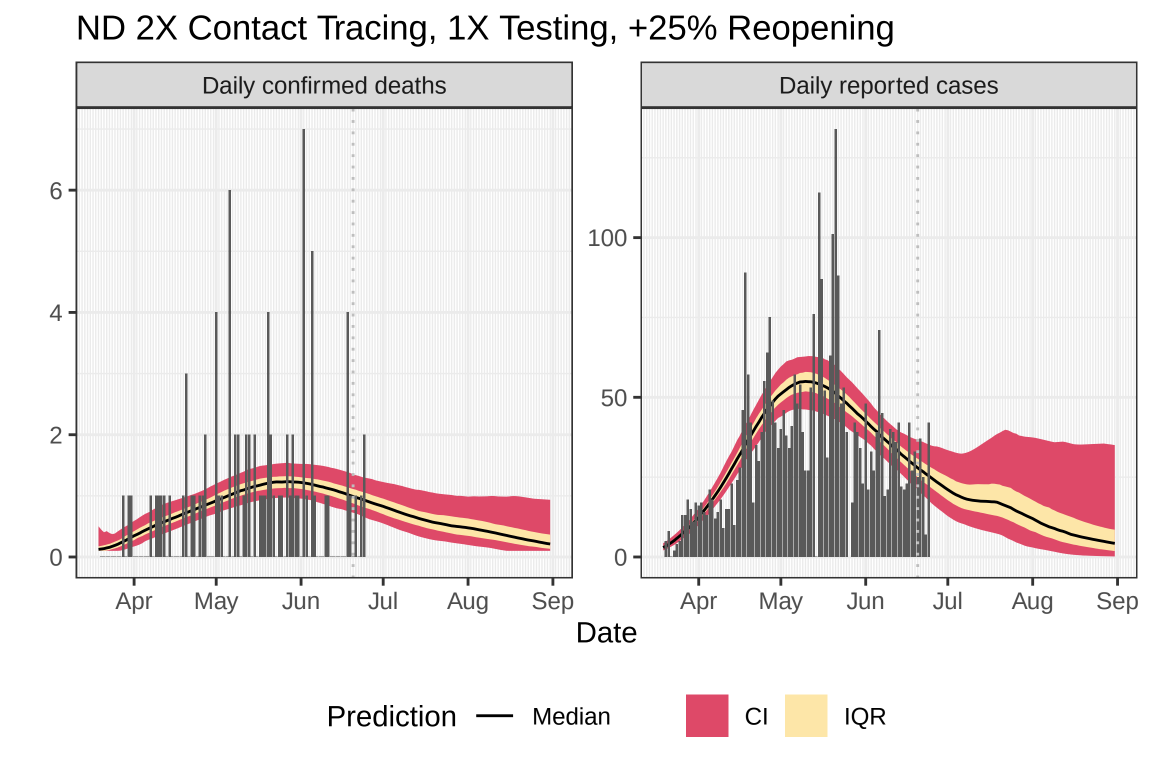
ND 2X Contact Tracing, 1X Testing, +25% Reopening
Daily confirmed deaths
Daily reported cases
0
2
4
6
0
50
100
Apr
May
Jun
Jul
Aug
Sep
Date
Prediction
Median
CI
IQR

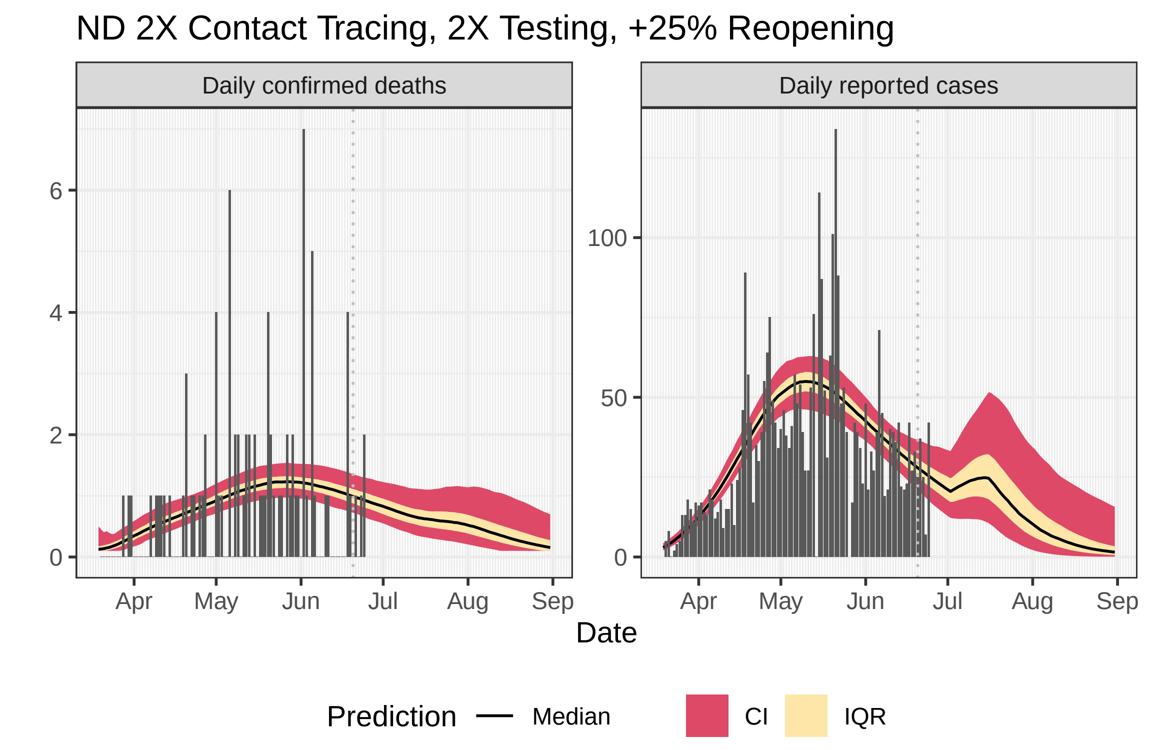
ND 2X Contact Tracing, 2X Testing, +25% Reopening
Daily confirmed deaths
Daily reported cases
0
2
4
6
0
50
100
Apr
May
Jun
Jul
Aug
Sep
Date
Prediction
Median
CI
IQR

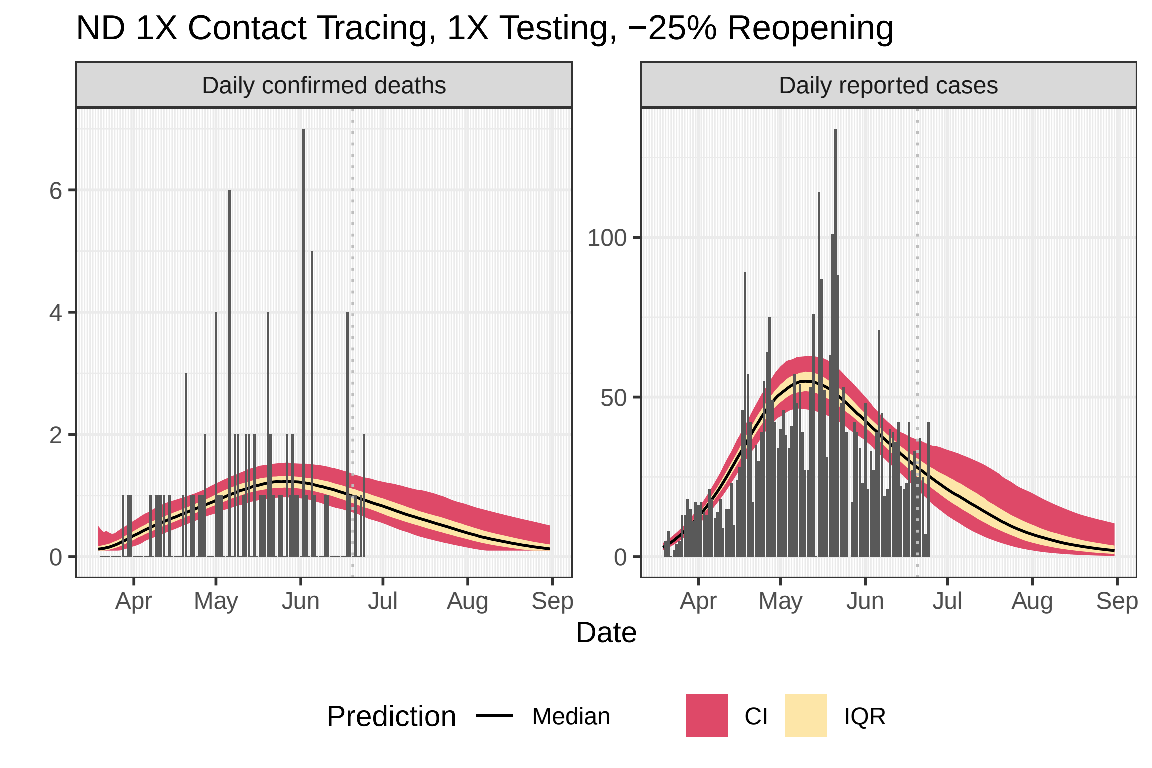
ND 1X Contact Tracing, 1X Testing, −25% Reopening
Daily confirmed deaths
Daily reported cases
0
2
4
6
50
100
Apr
May
Jun
Jul
Aug
Sep
Date
Prediction
Median
CI
IQR

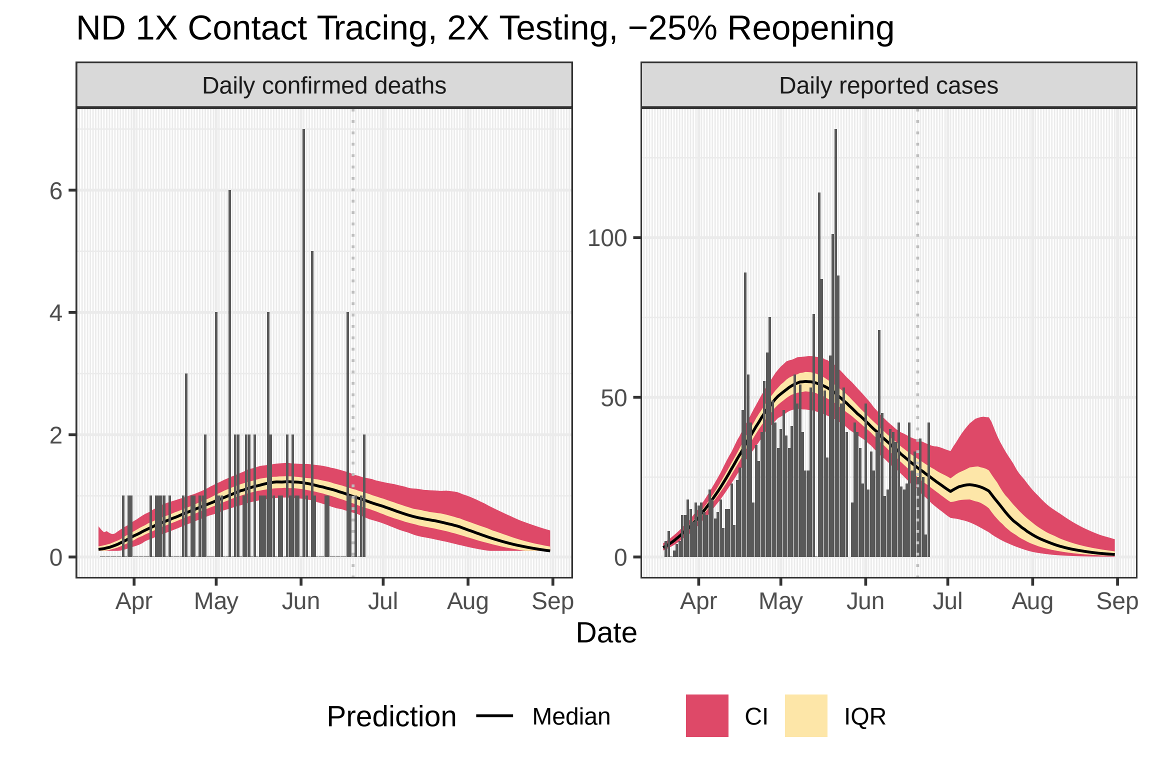

ND 1X Contact Tracing, 2X Testing, −25% Reopening
Daily confirmed deaths
Daily reported cases
0
2
4
6
0
50
100
Apr
May
Jun
Jul
Aug
Sep
Date
Prediction
Median
CI
IQR

ND 2X Contact Tracing, 1X Testing, −25% Reopening

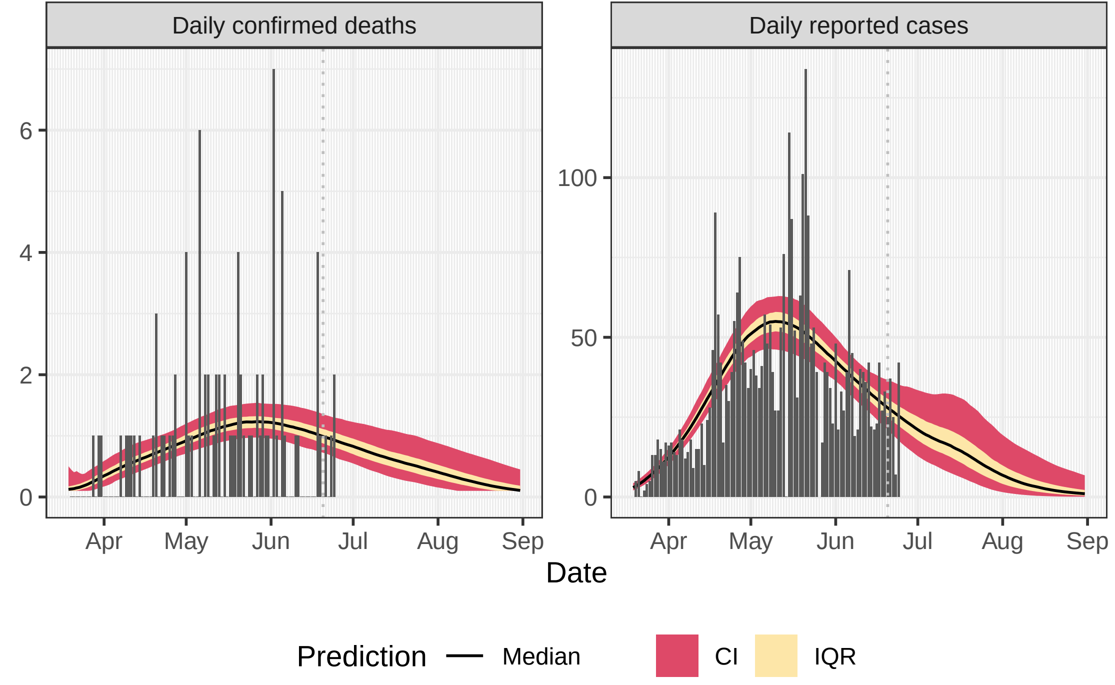

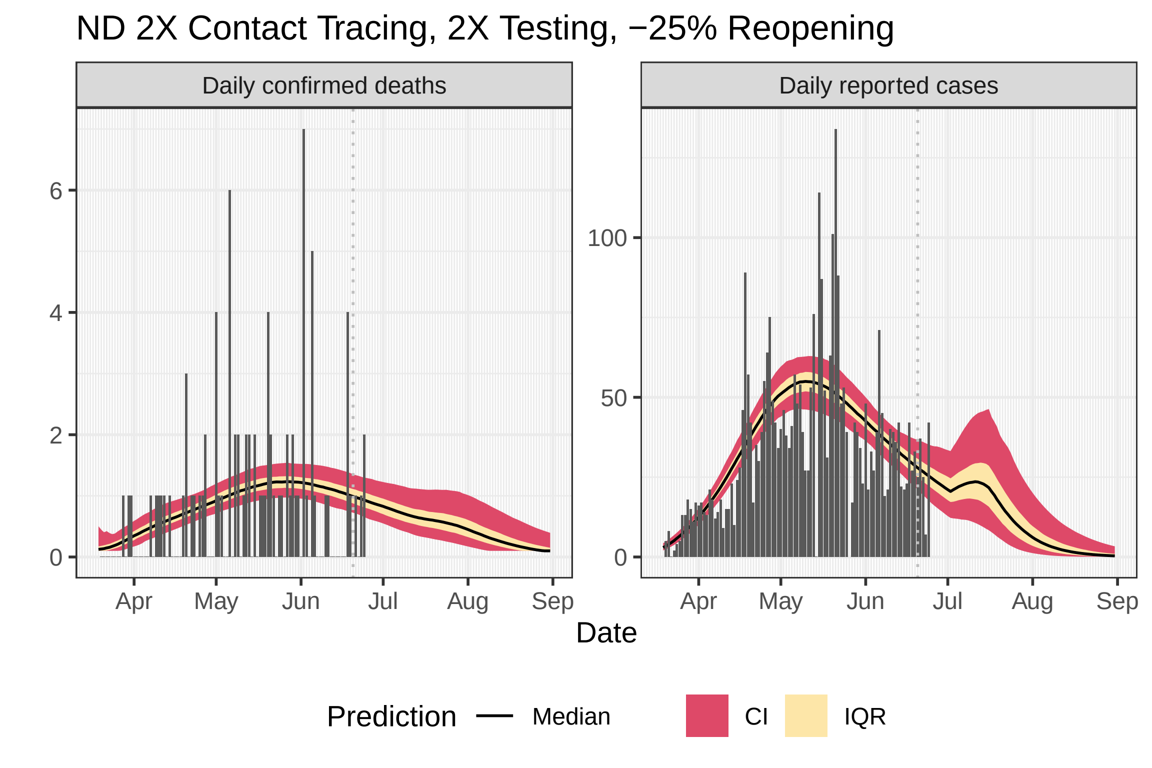
ND 2X Contact Tracing, 2X Testing, −25% Reopening
Daily confirmed deaths
Daily reported cases
0
2
4
6
0
50
100
Apr
May
Jun
Jul
Aug
Sep
Date
Prediction
Median
CI
IQR

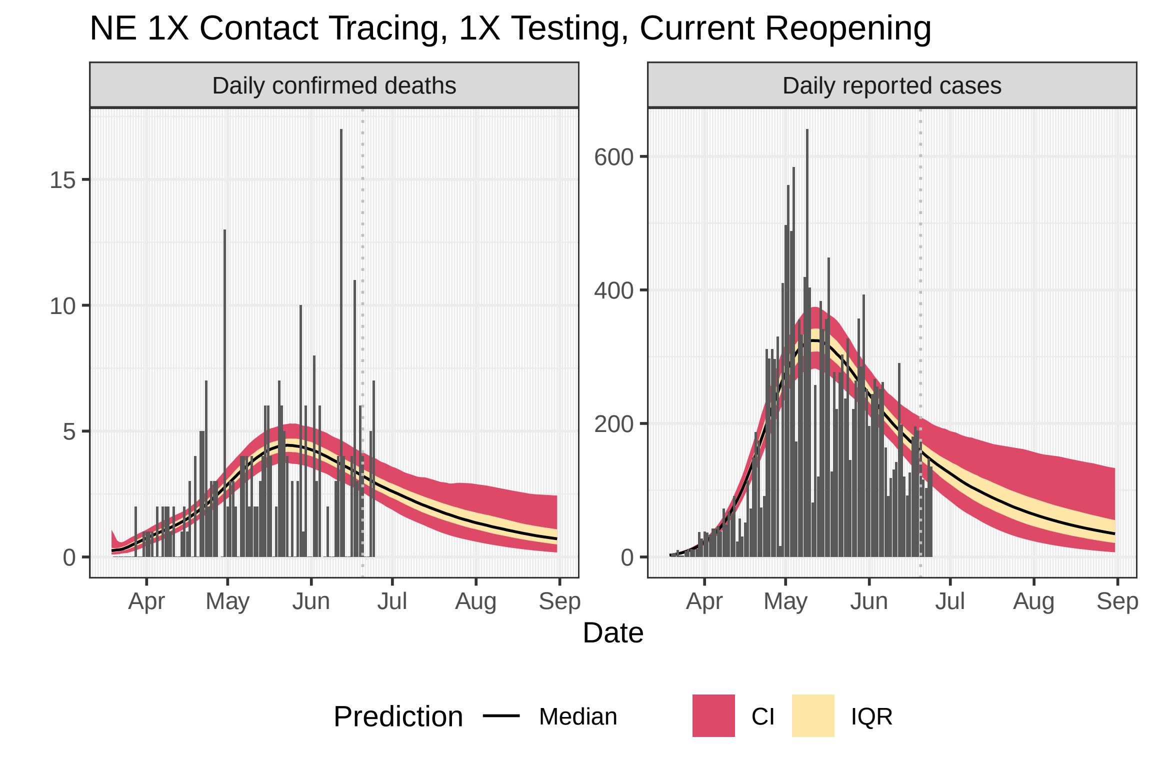

NE 1X Contact Tracing, 1X Testing, Current Reopening
Daily confirmed deaths
Daily reported cases
0
5
10
15
0
200
400
600
Apr
May
Jun
Jul
Aug
Sep
Date
Prediction
Median
CI
IQR

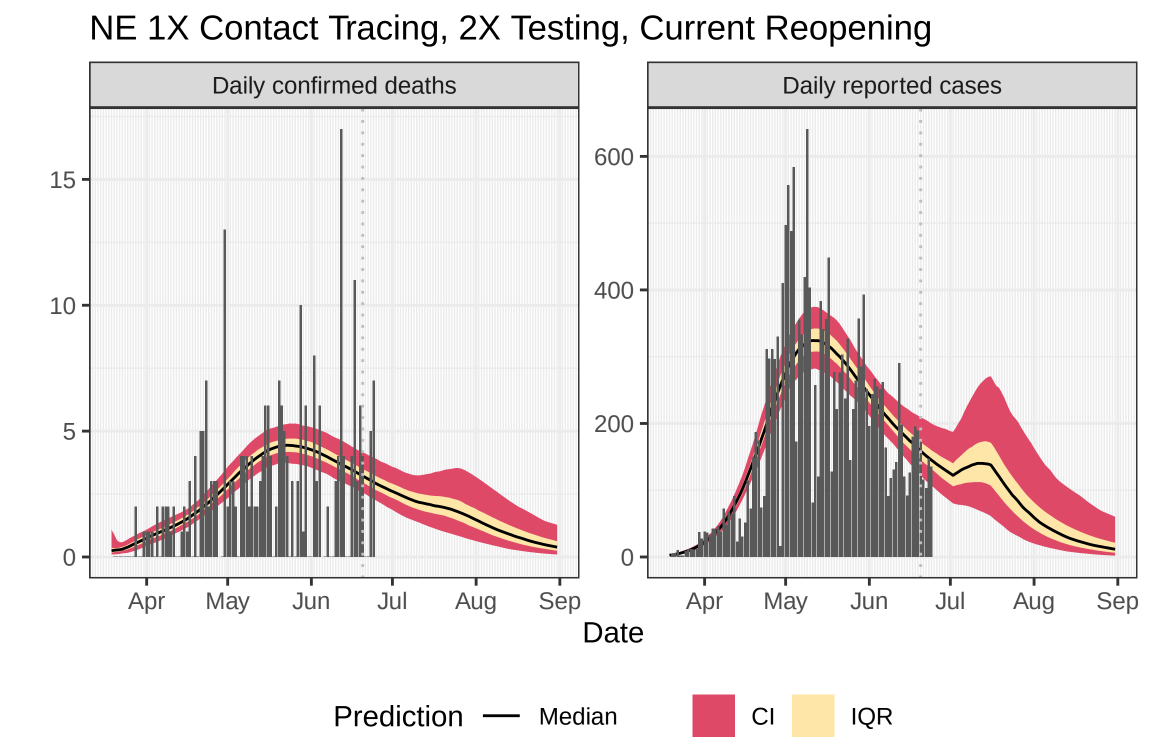

NE 1X Contact Tracing, 2X Testing, Current Reopening
Daily confirmed deaths
Daily reported cases
0
5
10
15
0
200
400
600
Apr
May
Jun
Jul
Aug
Sep
Date
Prediction
Median
CI
IQR

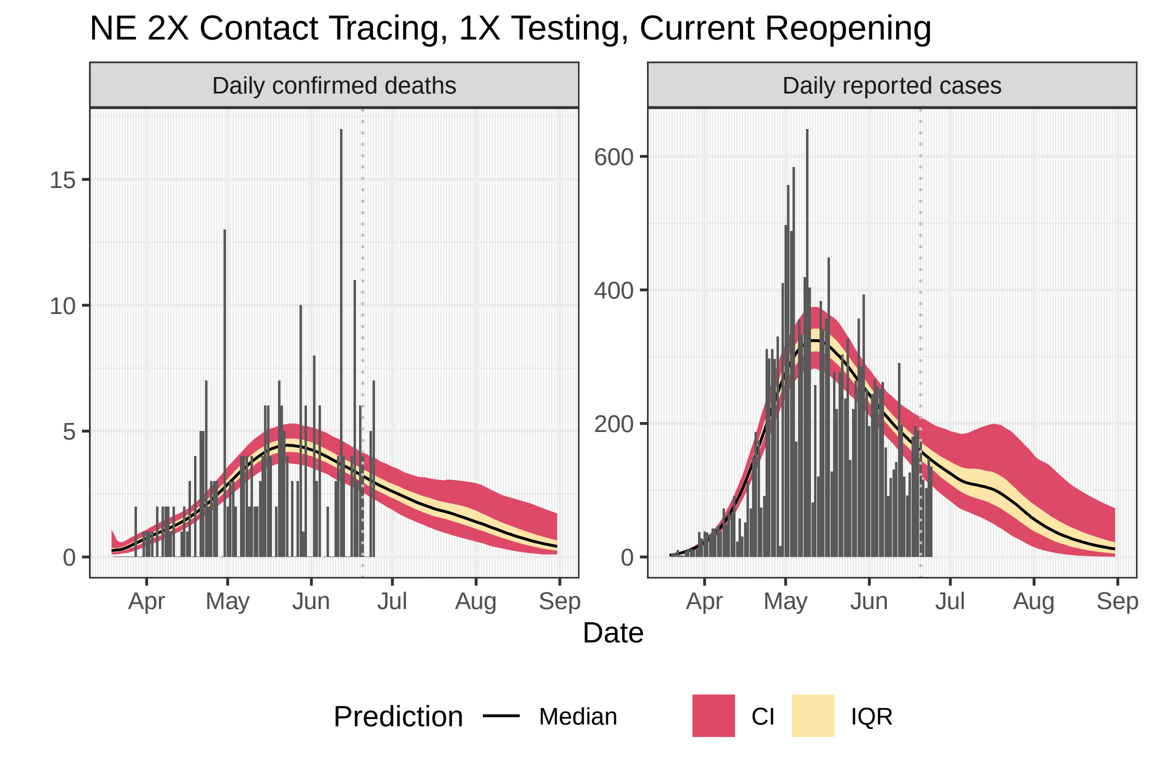
NE 2X Contact Tracing, 1X Testing, Current Reopening
Daily confirmed deaths
Daily reported cases
0
5
10
15
0
200
400
600
Apr
May
Jun
Jul
Aug
Sep
Date
Prediction
Median
CI
IQR

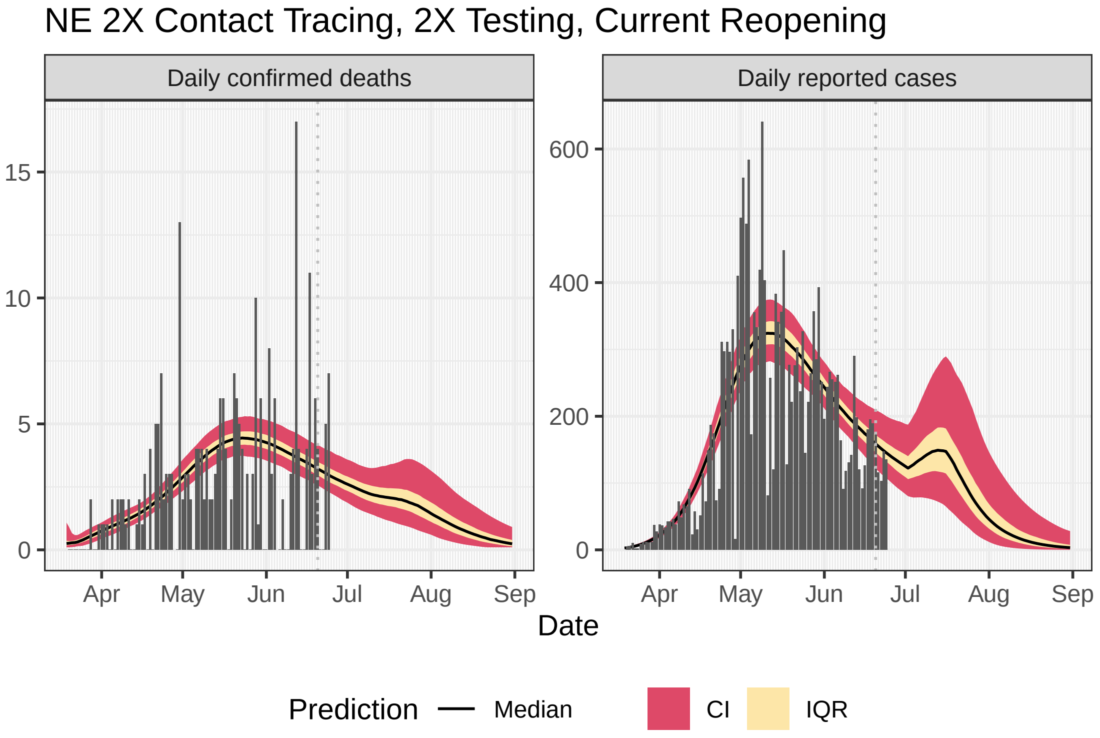
NE 2X Contact Tracing, 2X Testing, Current Reopening
Daily confirmed deaths
Daily reported cases
0
5
10
15
0
200
400
600
Apr
May
Jun
Jul
Aug
Sep
Date
Prediction
Median
CI
IQR

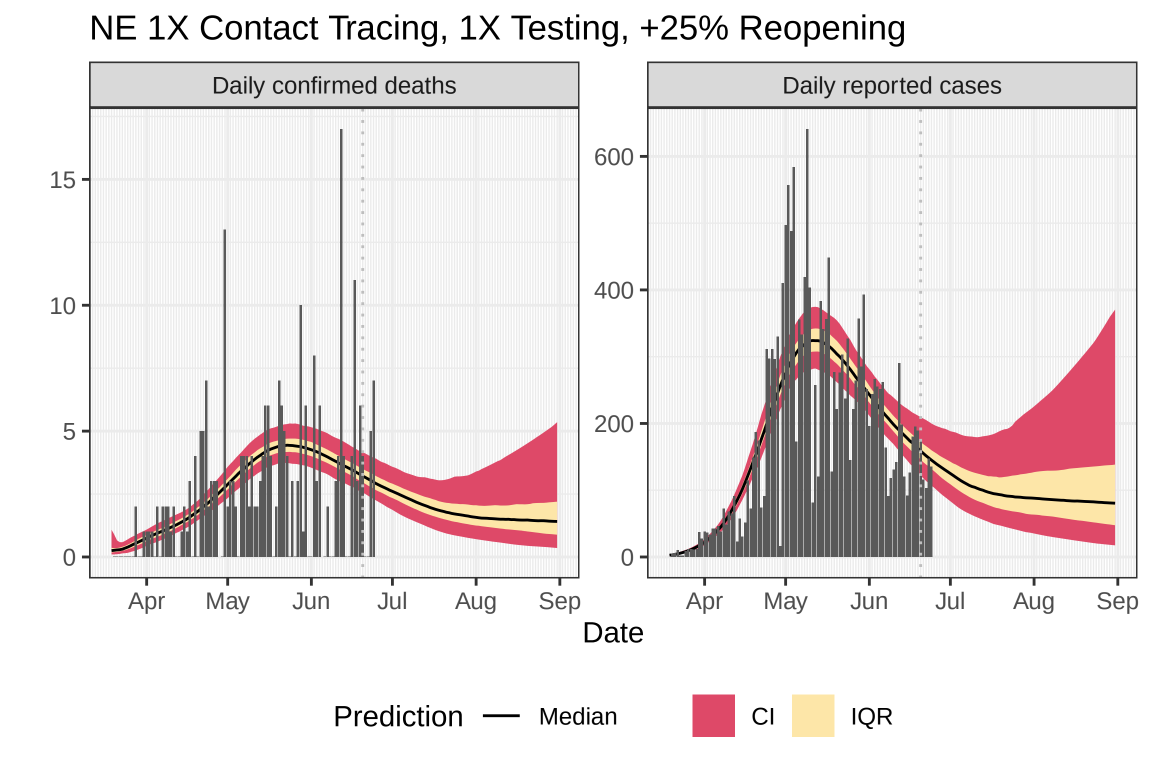

NE 1X Contact Tracing, 1X Testing, +25% Reopening
Daily confirmed deaths
Daily reported cases
0
5
10
15
0
200
400
600
Apr
May
Jun
Jul
Aug
Sep
Date
Prediction
Median
CI
IQR

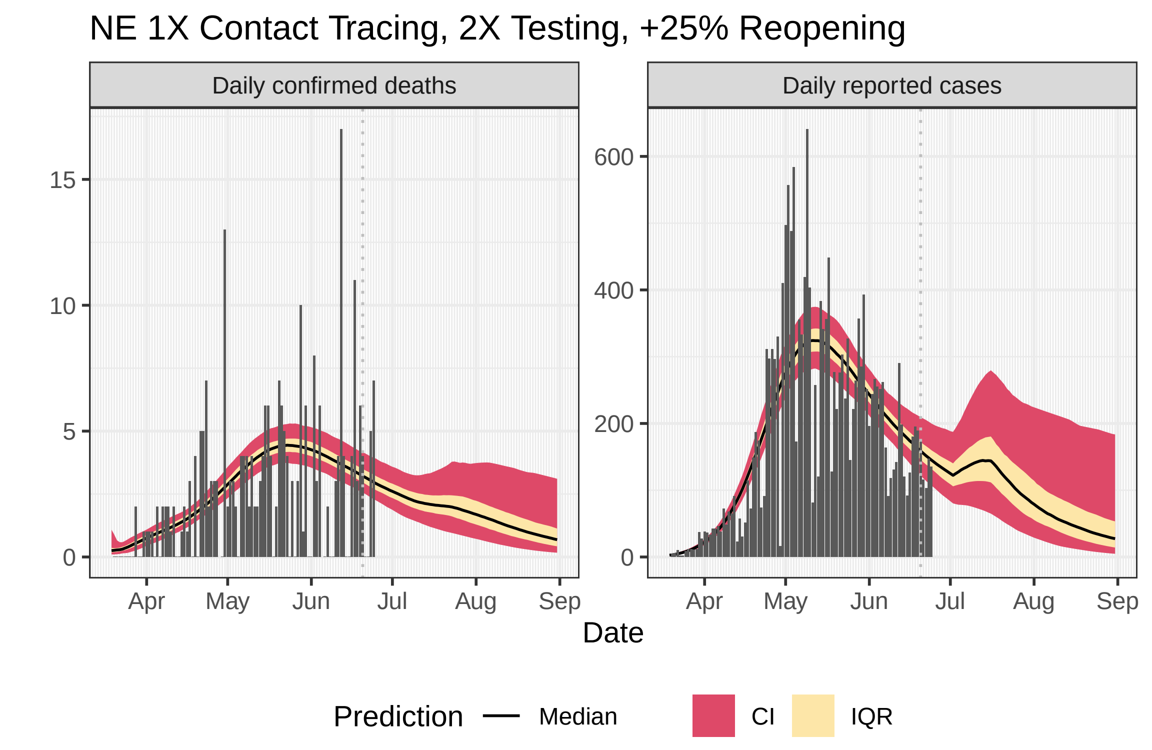
NE 1X Contact Tracing, 2X Testing, +25% Reopening
Daily confirmed deaths
Daily reported cases
0
5
10
15
0
200
400
600
Apr
May
Jun
Jul
Aug
Sep
Date
Prediction
Median
CI
IQR

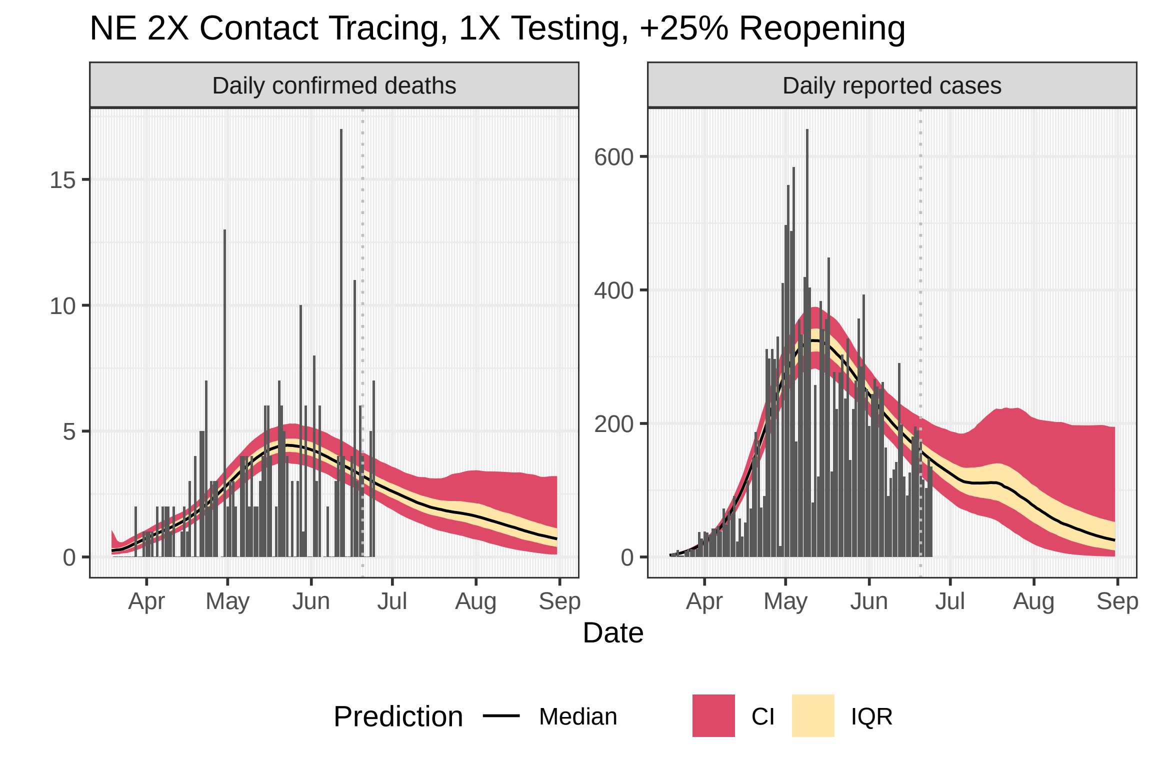

NE 2X Contact Tracing, 1X Testing, +25% Reopening
Daily confirmed deaths
Daily reported cases
0
5
10
15
0
200
400
600
Apr
May
Jun
Jul
Aug
Sep
Date
Prediction
Median
CI
IQR

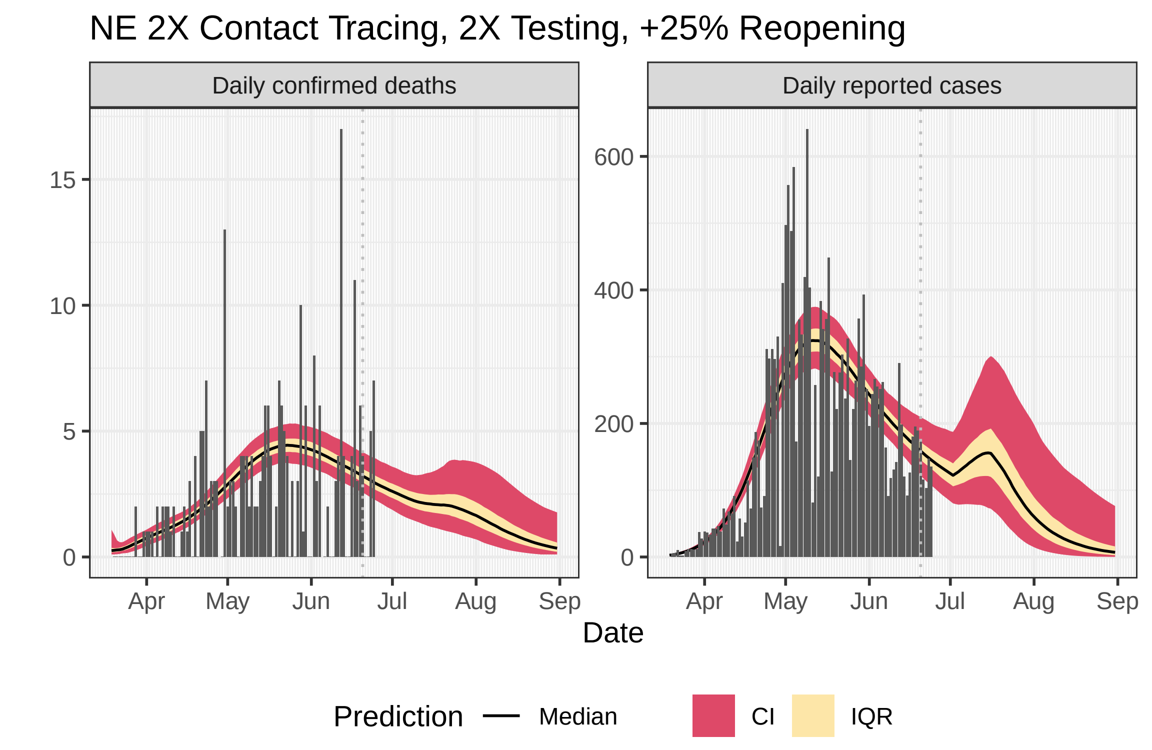

NE 2X Contact Tracing, 2X Testing, +25% Reopening
Daily confirmed deaths
Daily reported cases
0
5
10
15
0
200
400
600
Apr
May
Jun
Jul
Aug
Sep
Date
Prediction
Median
CI
IQR

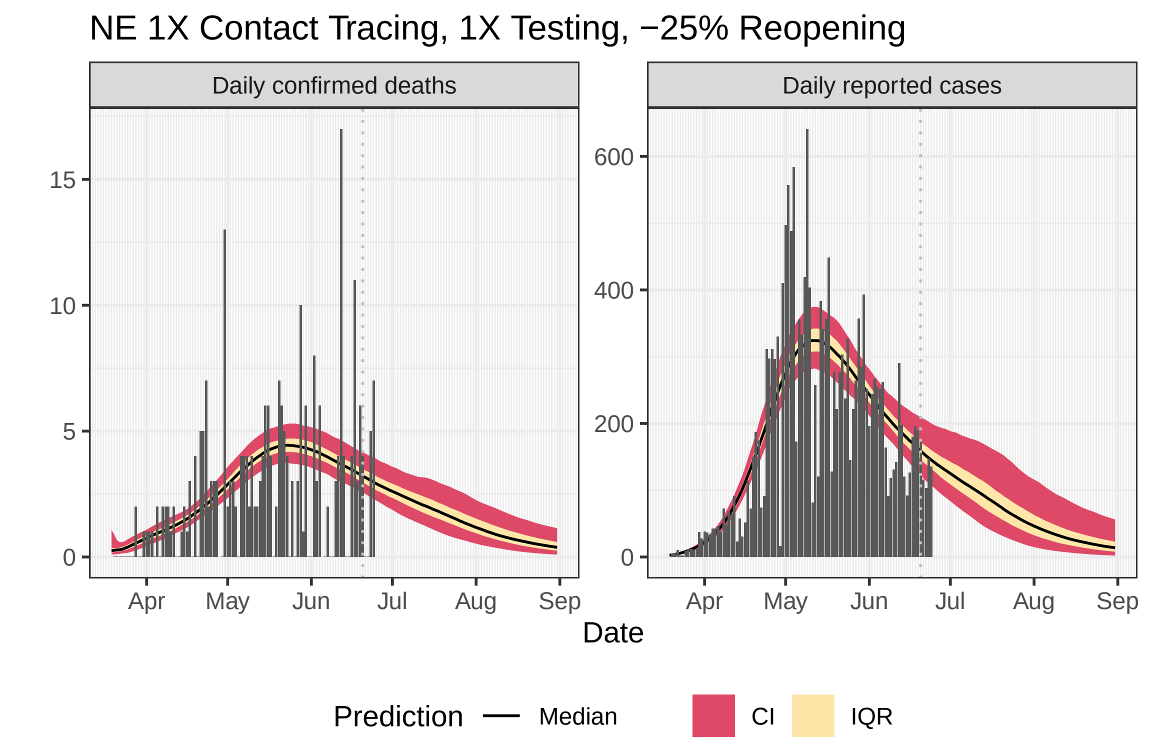
NE 1X Contact Tracing, 1X Testing, −25% Reopening
Daily confirmed deaths
Daily reported cases
0
5
10
15
0
200
400
600
Apr
May
Jun
Jul
Aug
Sep
Date
Prediction
Median
CI
IQR

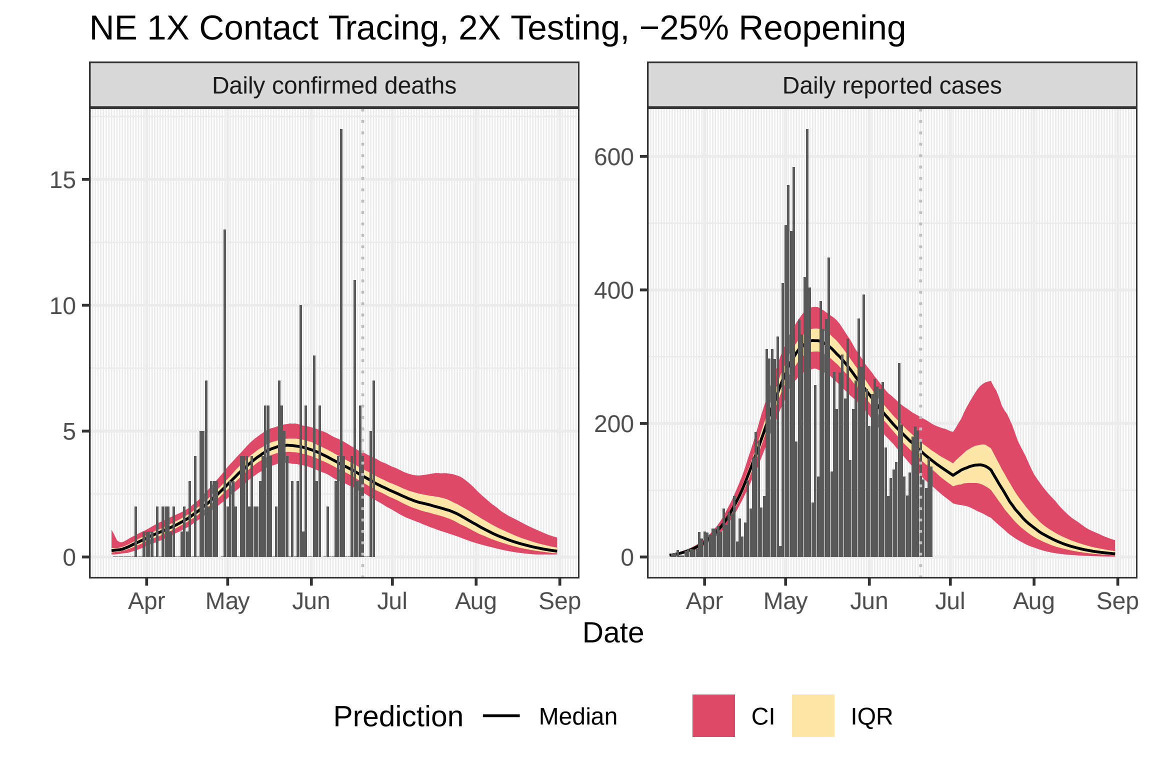

NE 1X Contact Tracing, 2X Testing, −25% Reopening
Daily confirmed deaths
Daily reported cases
0
5
10
15
0
200
400
600
Apr
May
Jun
Jul
Aug
Sep
Date
Prediction
Median
CI
IQR

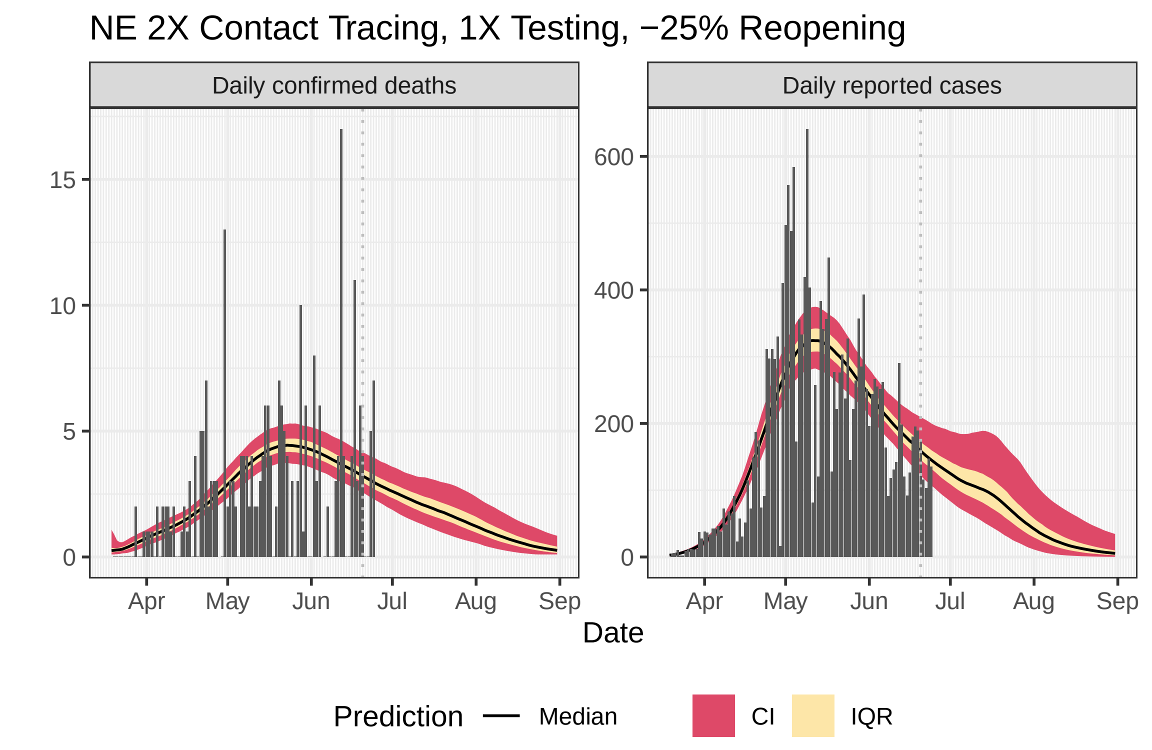

NE 2X Contact Tracing, 1X Testing, −25% Reopening
Daily confirmed deaths
Daily reported cases
15
10
5
0
600
400
200
0
Apr
May
Jun
Jul
Aug
Sep
Date
Prediction
Median
CI
IQR

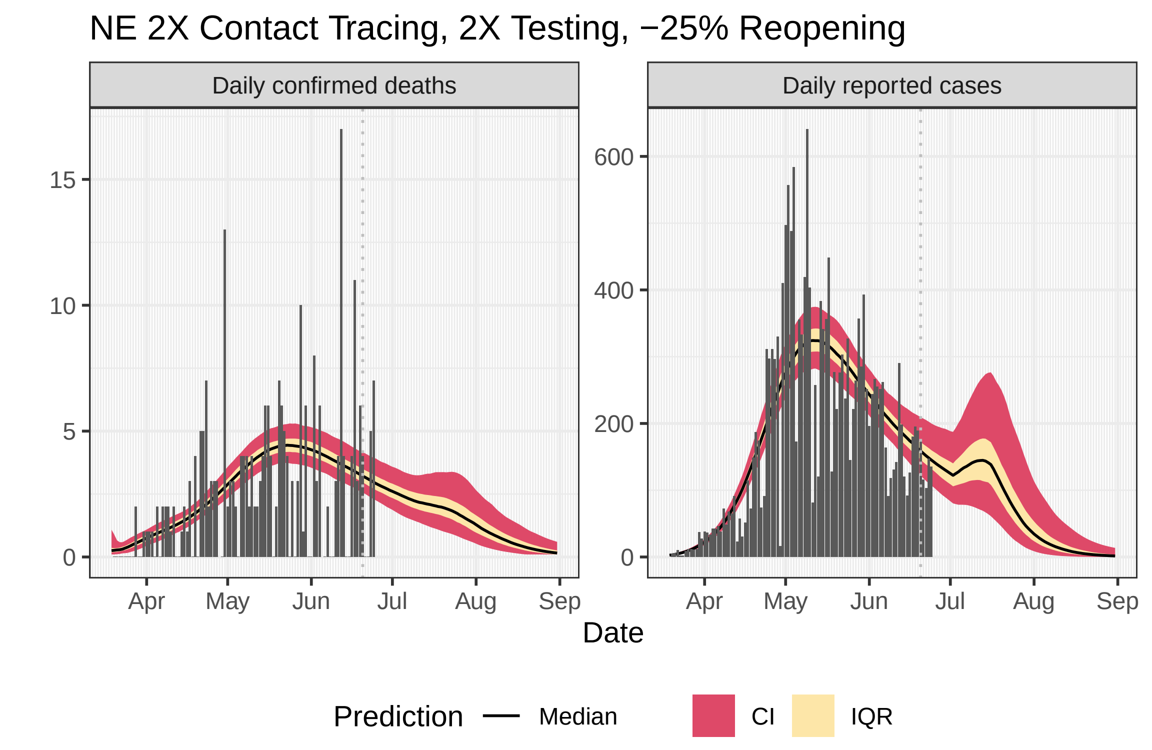
NE 2X Contact Tracing, 2X Testing, −25% Reopening
Daily confirmed deaths
Daily reported cases
0
5
10
15
0
200
400
600
Apr
May
Jun
Jul
Aug
Sep
Date
Prediction
Median
CI
IQR

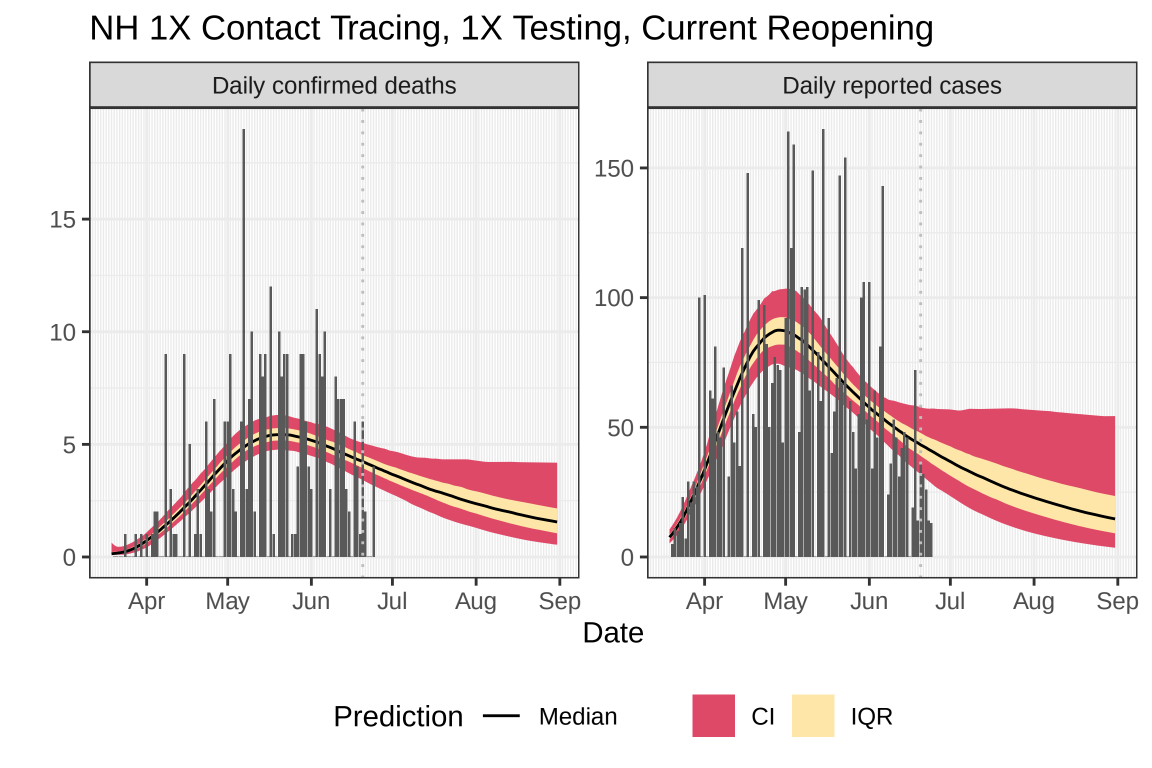
NH 1X Contact Tracing, 1X Testing, Current Reopening
Daily confirmed deaths
Daily reported cases
0
5
10
15
0
50
100
150
Apr
May
Jun
Jul
Aug
Sep
Date
Prediction
Median
CI
IQR

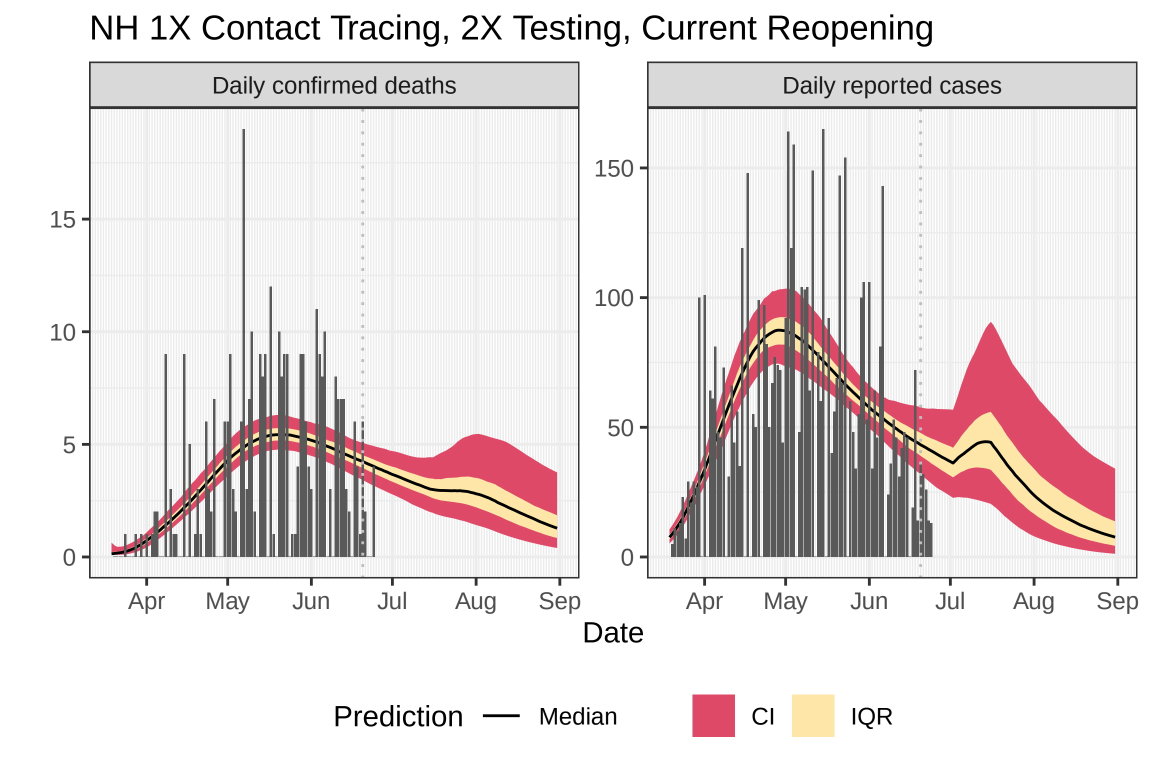
NH 1X Contact Tracing, 2X Testing, Current Reopening
Daily confirmed deaths
Daily reported cases
0
5
10
15
0
50
100
150
Apr
May
Jun
Jul
Aug
Sep
Date
Prediction
Median
CI
IQR

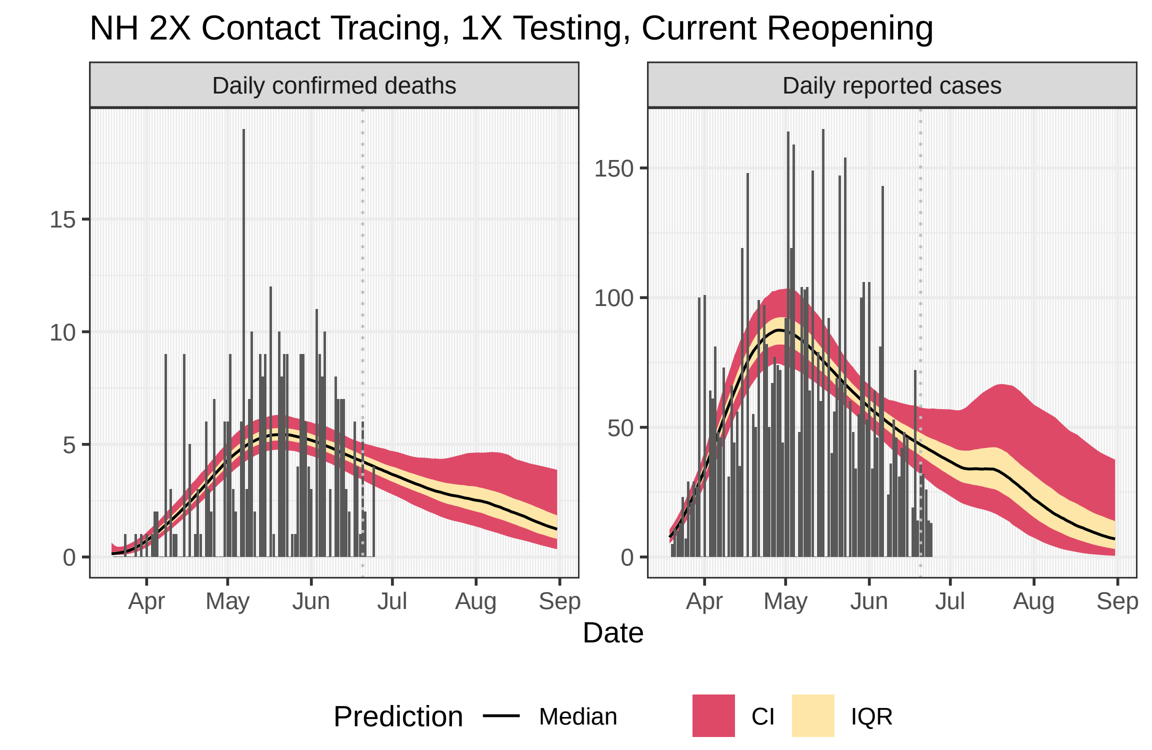
NH 2X Contact Tracing, 1X Testing, Current Reopening
Daily confirmed deaths
Daily reported cases
0
5
10
15
0
50
100
150
Apr
May
Jun
Jul
Aug
Sep
Date
Prediction
Median
CI
IQR

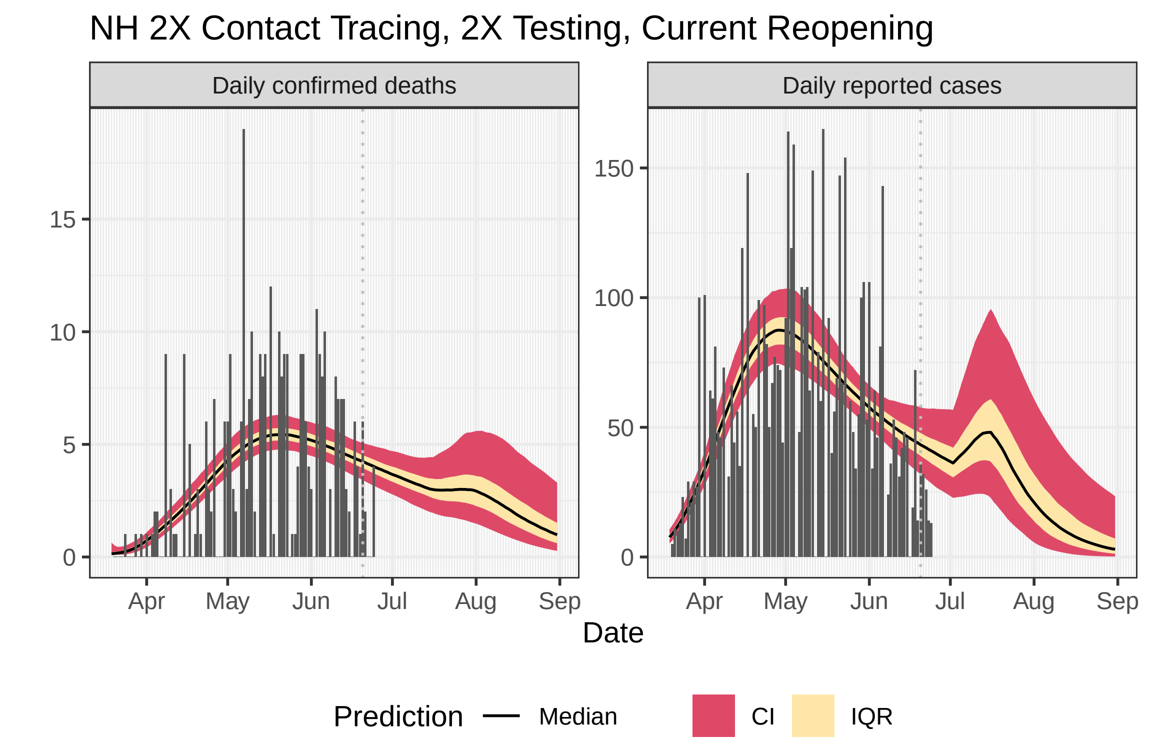

NH 2X Contact Tracing, 2X Testing, Current Reopening
Daily confirmed deaths
Daily reported cases
0
5
10
15
50
100
150
Apr
May
Jun
Jul
Aug
Sep
Date
Prediction
Median
CI
IQR

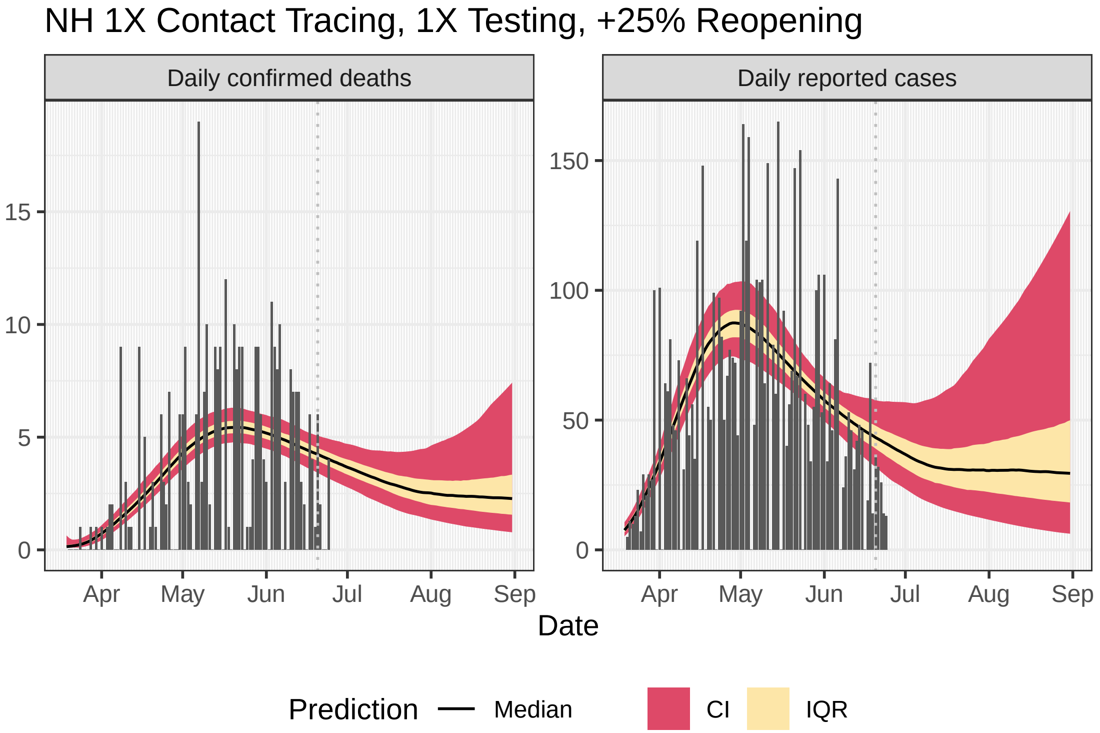
NH 1X Contact Tracing, 1X Testing, +25% Reopening
Daily confirmed deaths
Daily reported cases
0
5
10
15
0
50
100
150
Apr
May
Jun
Jul
Aug
Sep
Date
Prediction
Median
CI
IQR

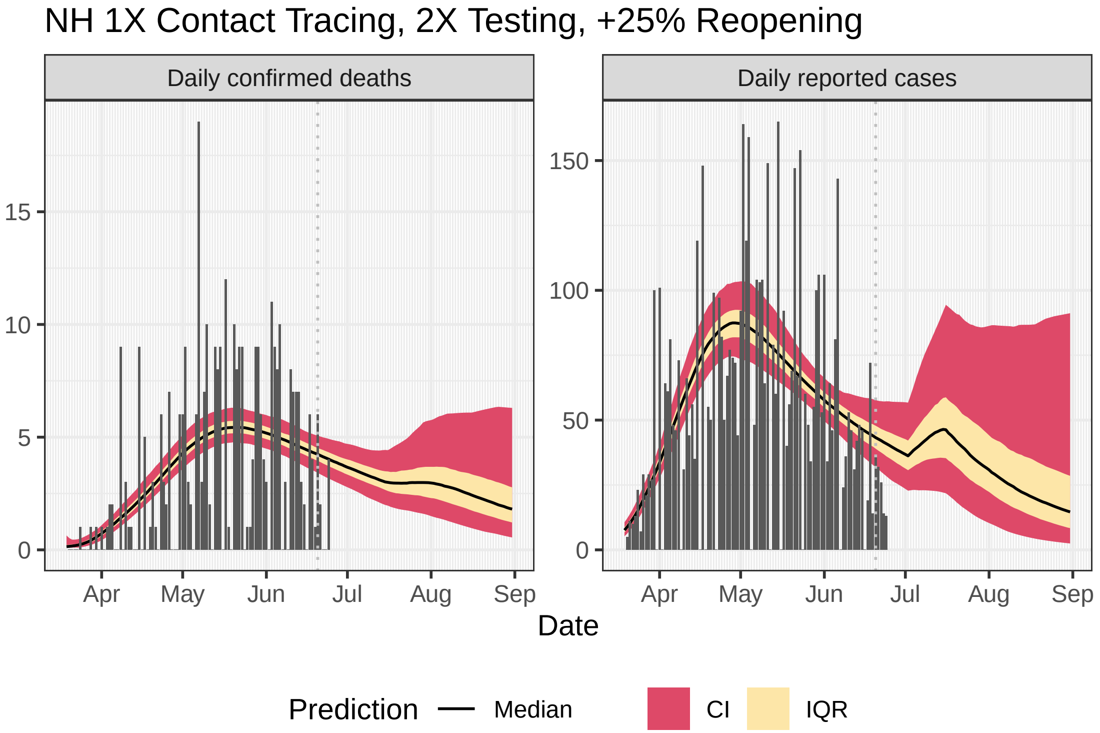

NH 1X Contact Tracing, 2X Testing, +25% Reopening
Daily confirmed deaths
Daily reported cases
0
5
10
15
0
50
100
150
Apr
May
Jun
Jul
Aug
Sep
Date
Prediction
Median
CI
IQR

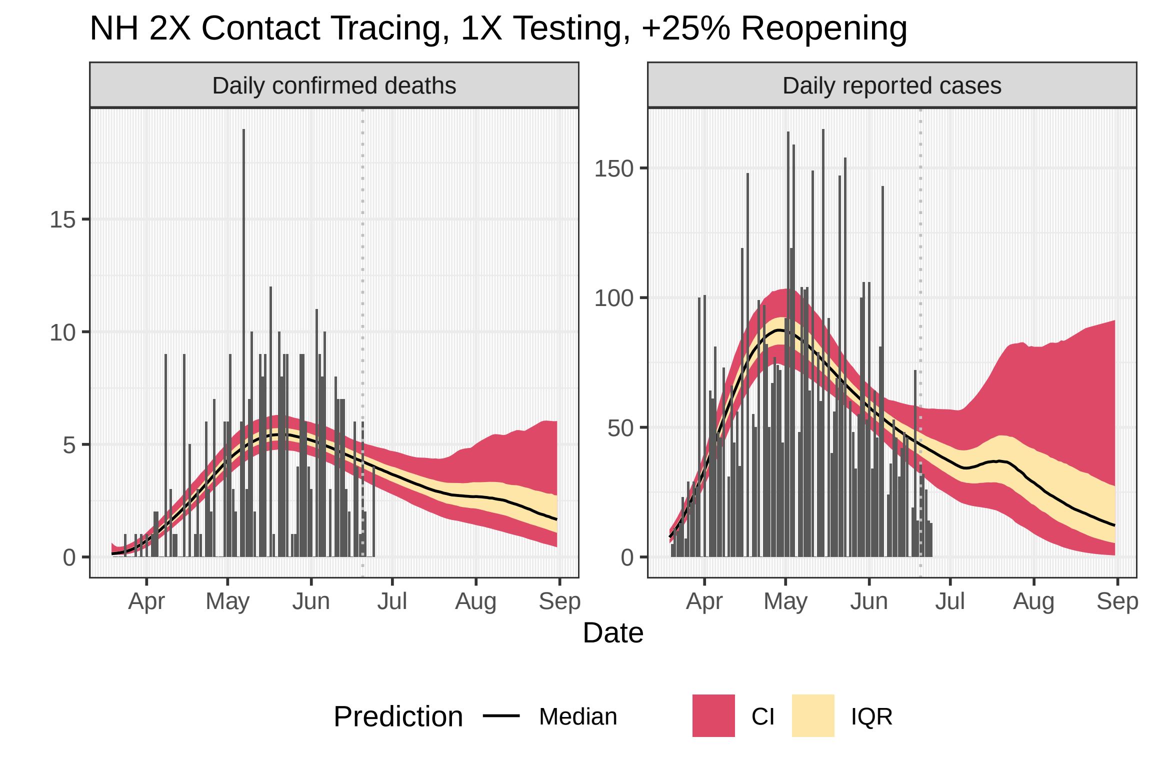
NH 2X Contact Tracing, 1X Testing, +25% Reopening
Daily confirmed deaths
Daily reported cases
0
5
10
15
0
50
100
150
Apr
May
Jun
Jul
Aug
Sep
Date
Prediction
Median
CI
IQR

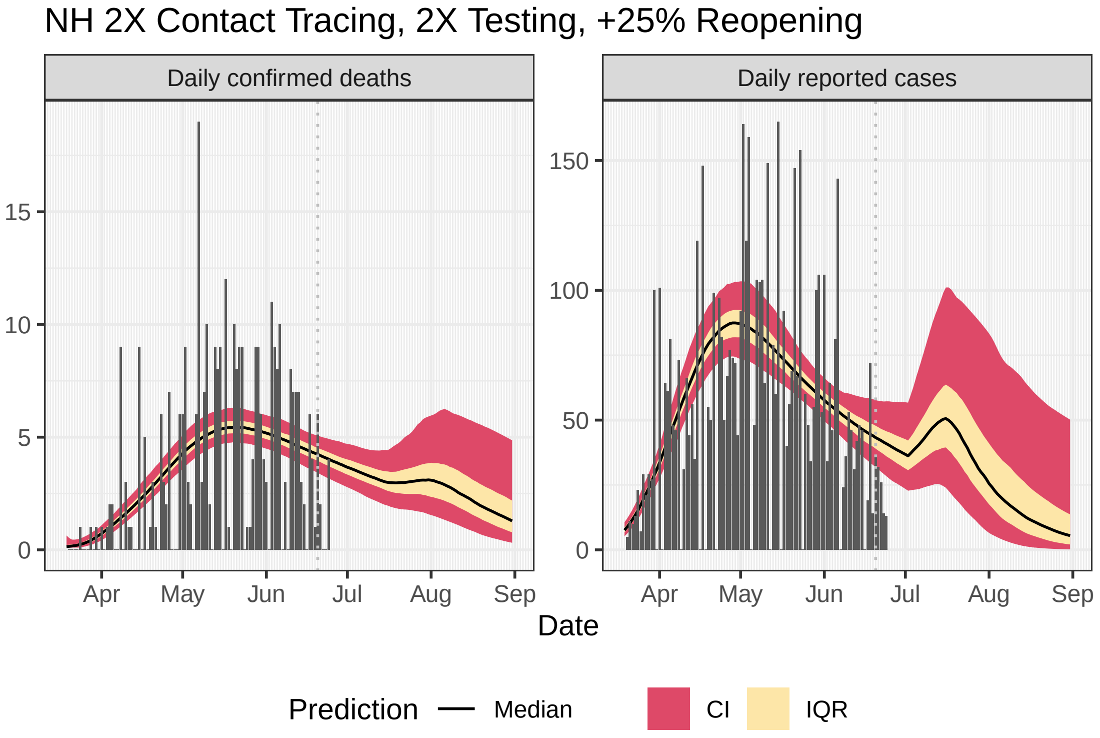

NH 2X Contact Tracing, 2X Testing, +25% Reopening
Daily confirmed deaths
Daily reported cases
0
5
10
15
50
100
150
Apr
May
Jun
Jul
Aug
Sep
Date
Prediction
Median
CI
IQR

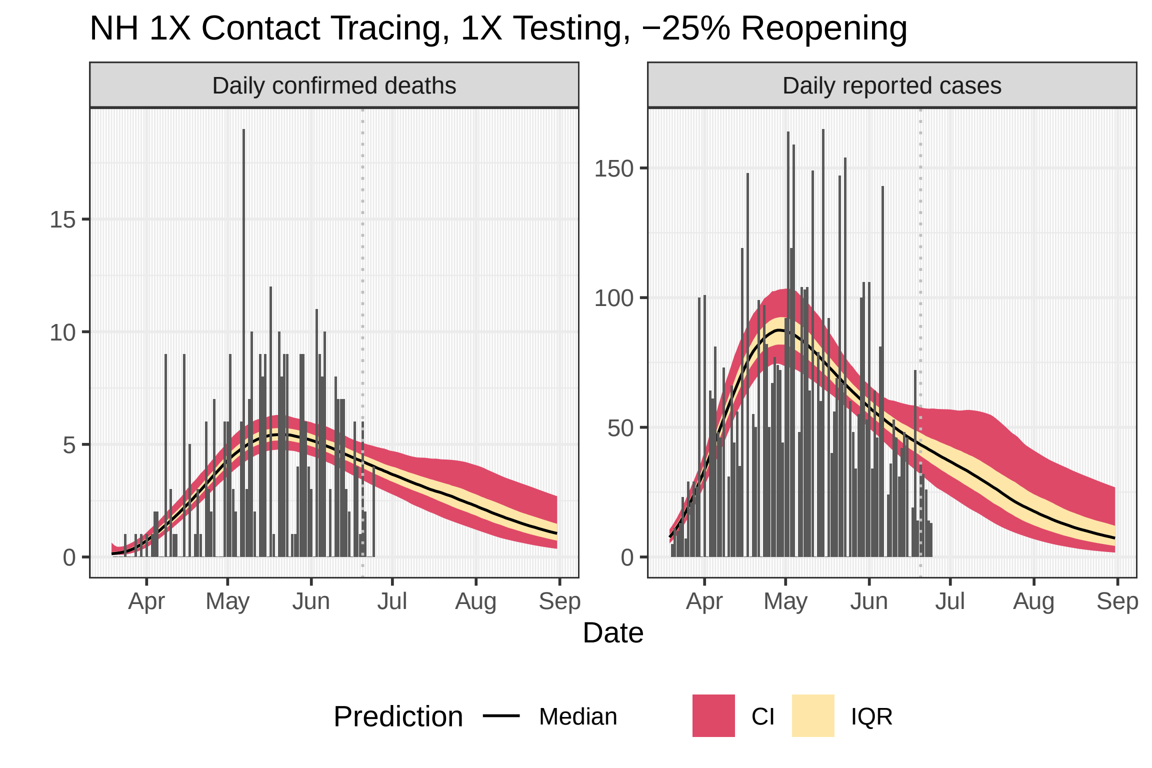
NH 1X Contact Tracing, 1X Testing, −25% Reopening
Daily confirmed deaths
Daily reported cases
0
5
10
15
50
100
150
Apr
May
Jun
Jul
Aug
Sep
Date
Prediction
Median
CI
IQR

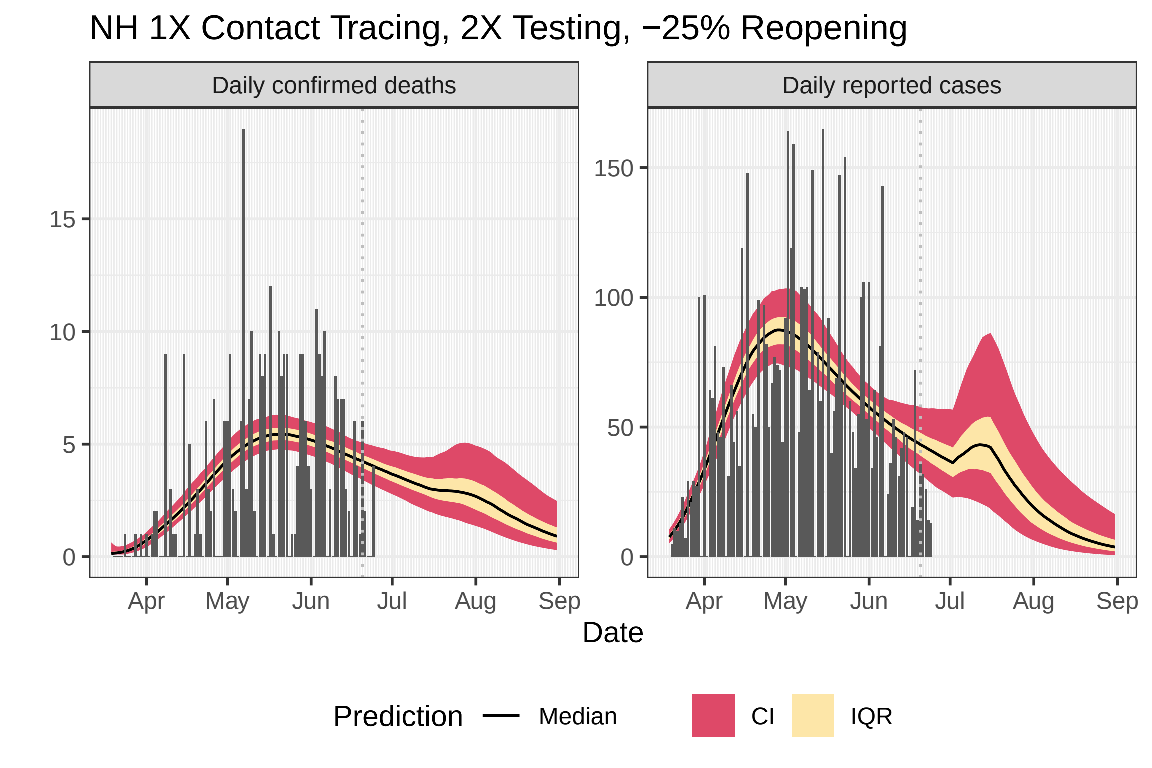

NH 1X Contact Tracing, 2X Testing, −25% Reopening
Daily confirmed deaths
Daily reported cases
0
5
10
15
0
50
100
150
Apr
May
Jun
Jul
Aug
Sep
Date
Prediction
Median
CI
IQR

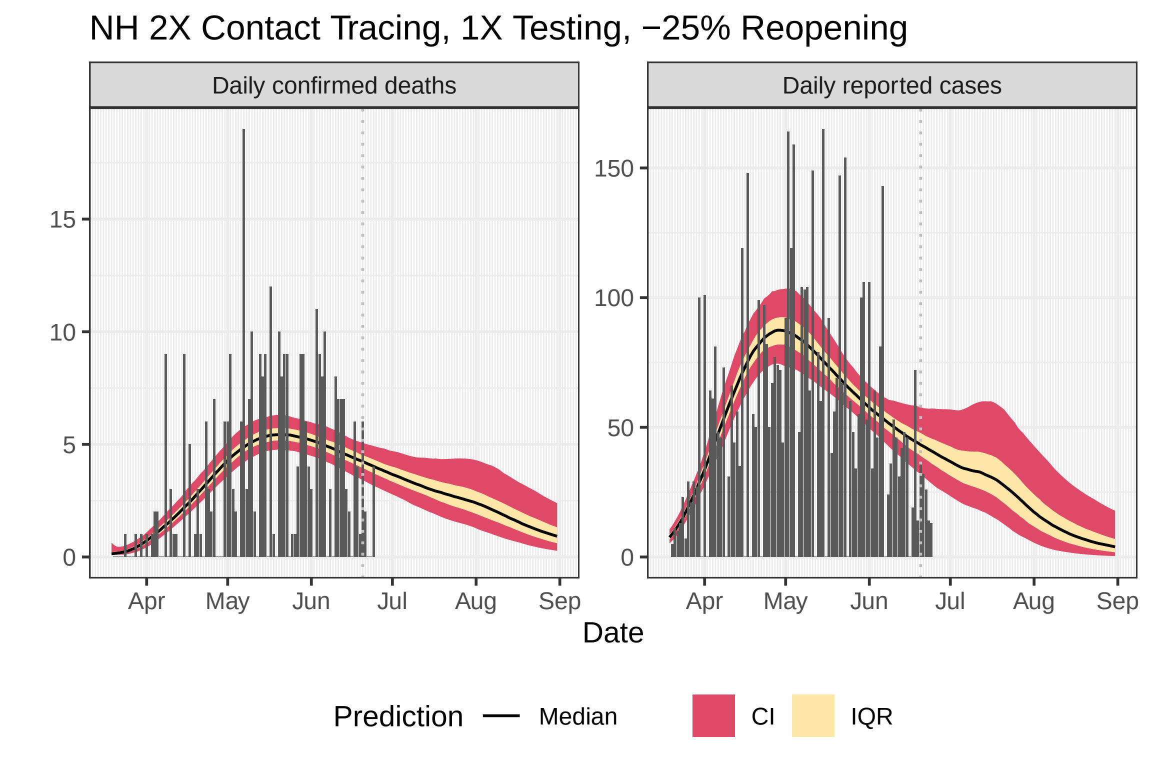

NH 2X Contact Tracing, 1X Testing, −25% Reopening
Daily confirmed deaths
Daily reported cases
0
5
10
15
50
100
150
Apr
May
Jun
Jul
Aug
Sep
Date
Prediction
Median
CI
IQR

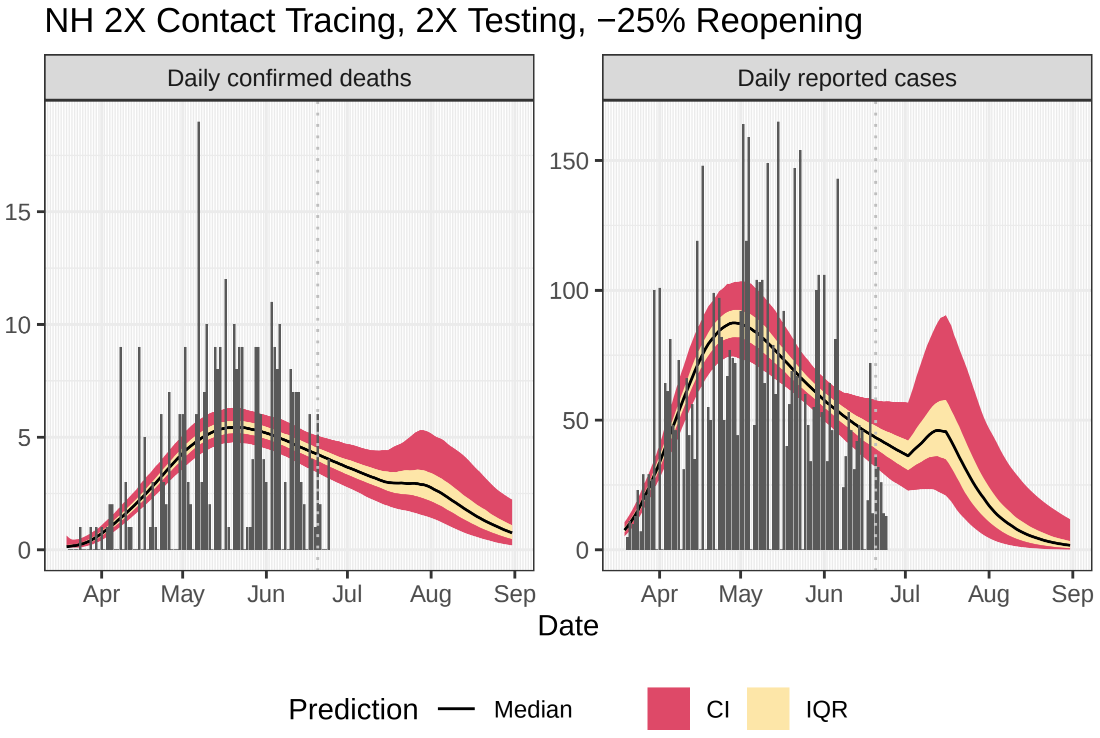

NH 2X Contact Tracing, 2X Testing, −25% Reopening
Daily confirmed deaths
Daily reported cases
0
5
10
15
0
50
100
150
Apr
May
Jun
Jul
Aug
Sep
Date
Prediction
Median
CI
IQR

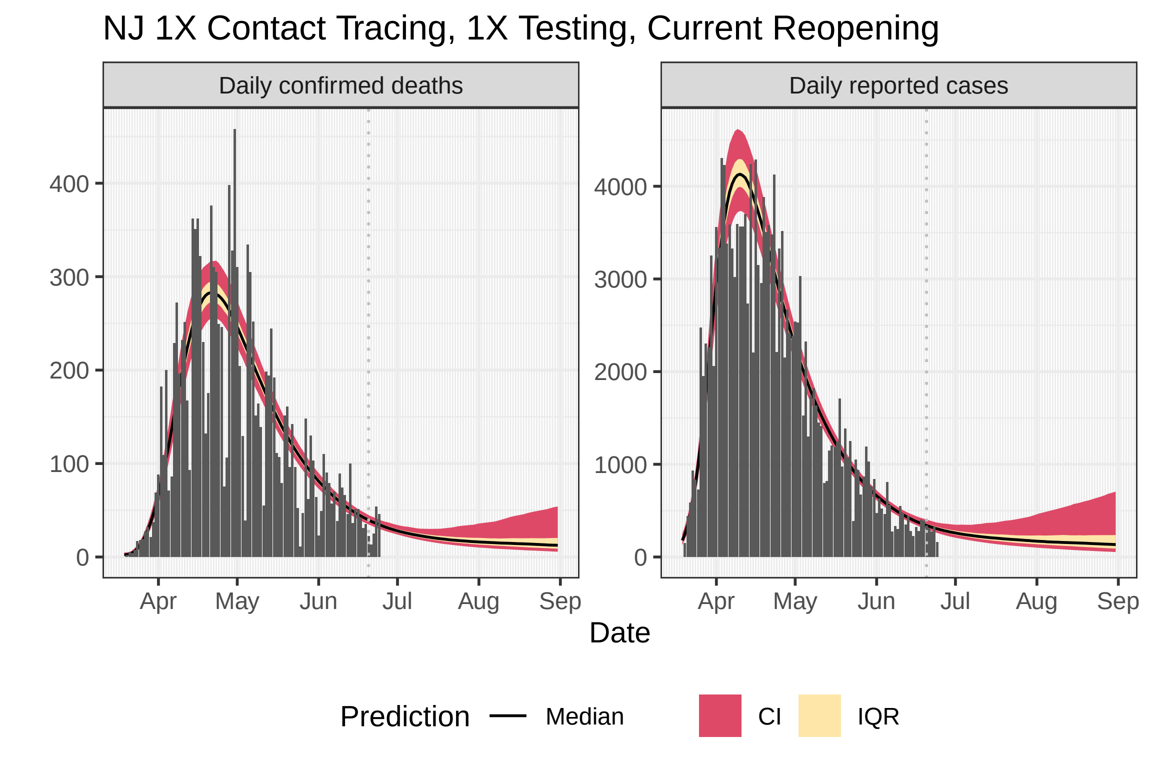
NJ 1X Contact Tracing, 1X Testing, Current Reopening
Daily confirmed deaths
Daily reported cases
0
100
200
300
400
1000
2000
3000
4000
Apr
May
Jun
Jul
Aug
Sep
Date
Prediction
Median
CI
IQR

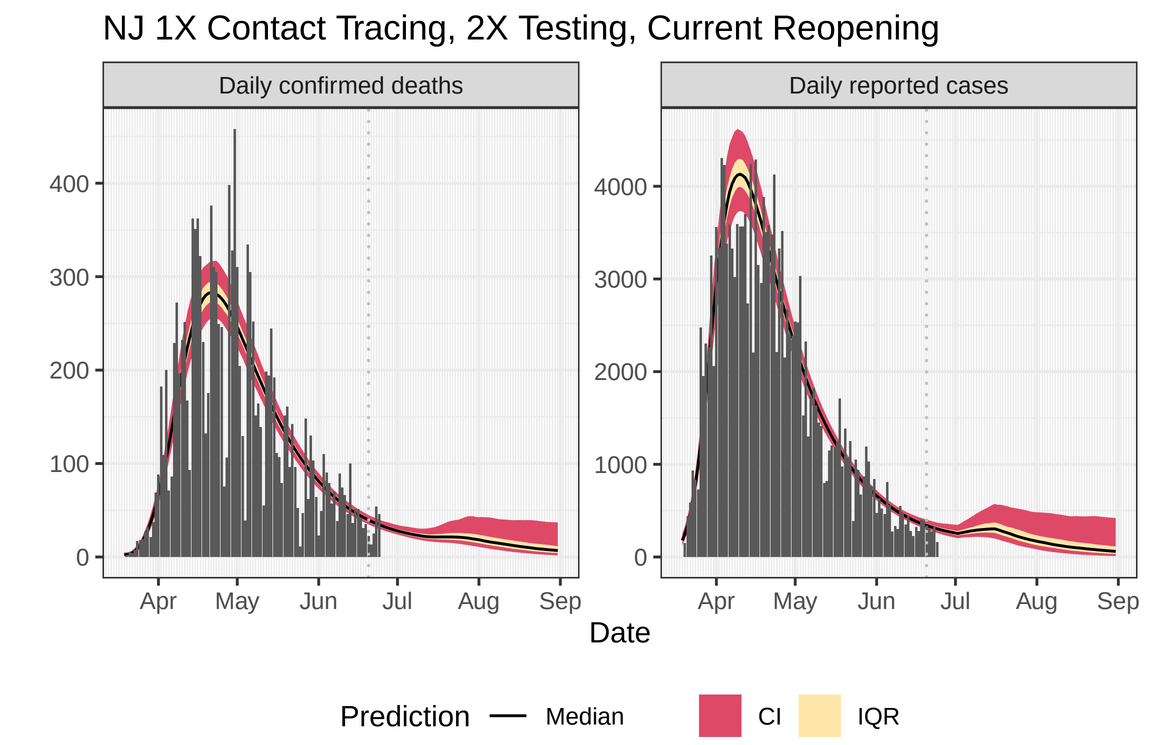

NJ 1X Contact Tracing, 2X Testing, Current Reopening
Daily confirmed deaths
Daily reported cases
0
100
200
300
400
0
1000
2000
3000
4000
Apr
May
Jun
Jul
Aug
Sep
Date
Prediction
Median
CI
IQR

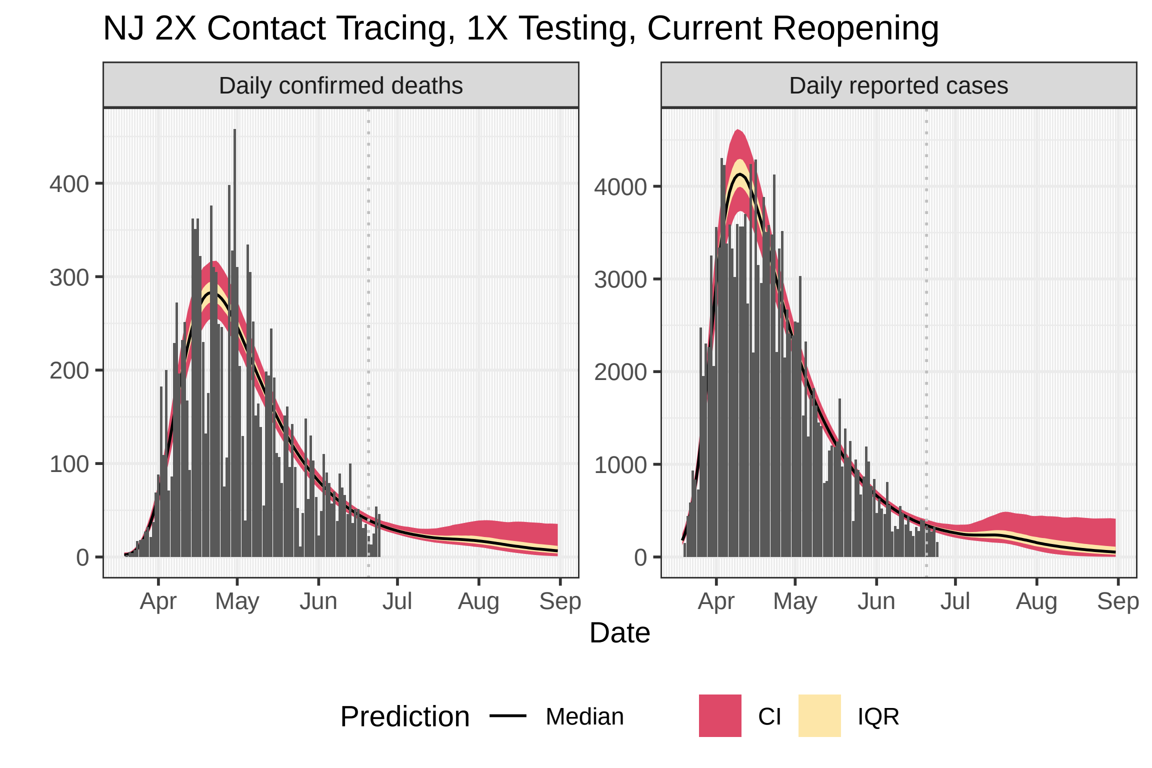

NJ 2X Contact Tracing, 1X Testing, Current Reopening
Daily confirmed deaths
Daily reported cases
0
100
200
300
400
1000
2000
3000
4000
Apr
May
Jun
Jul
Aug
Sep
Date
Prediction
Median
CI
IQR

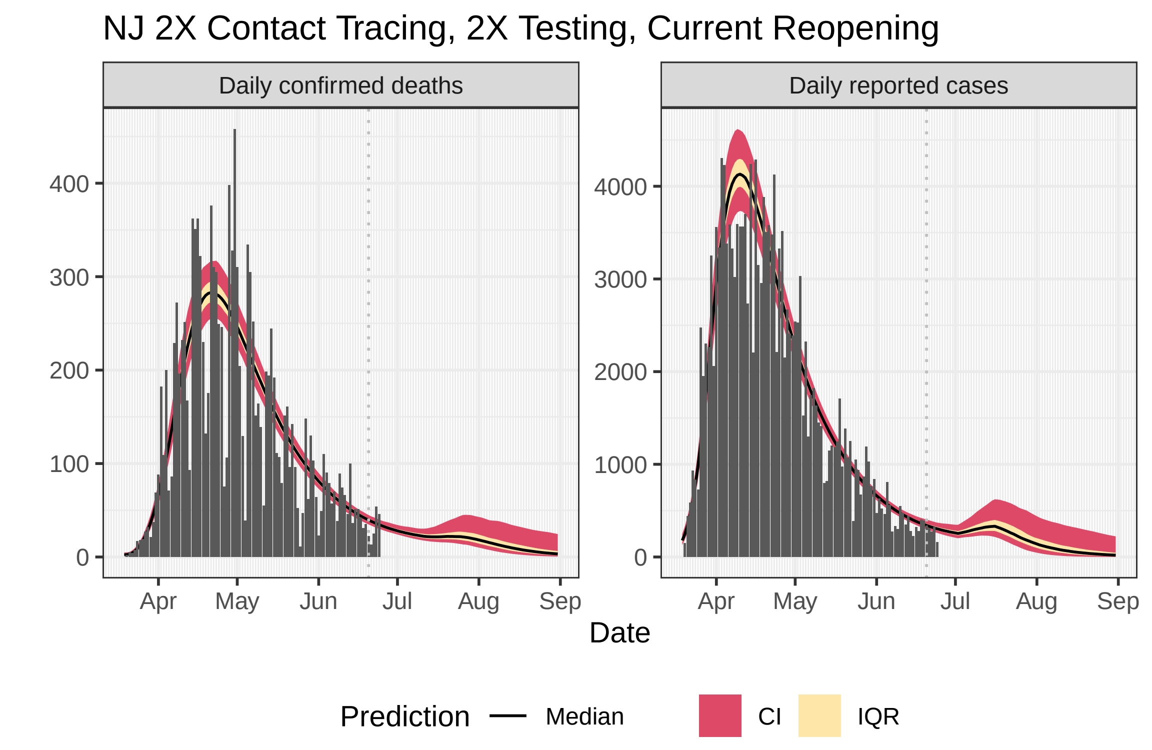

NJ 2X Contact Tracing, 2X Testing, Current Reopening
Daily confirmed deaths
Daily reported cases
400
300
200
100
0
4000
3000
2000
1000
0
Apr
May
Jun
Jul
Aug
Sep
Date
Prediction
Median
CI
IQR

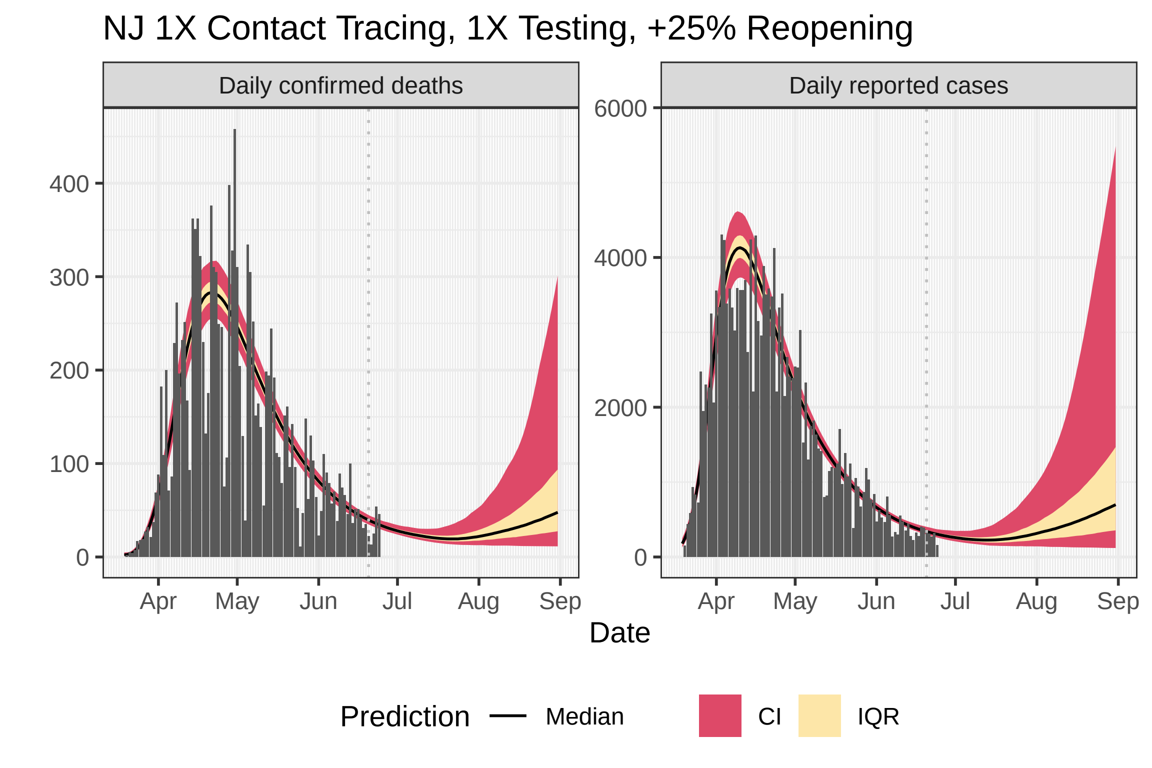

NJ 1X Contact Tracing, 1X Testing, +25% Reopening
Daily confirmed deaths
Daily reported cases
0
100
200
300
400
0
2000
4000
6000
Apr
May
Jun
Jul
Aug
Sep
Date
Prediction
Median
CI
IQR

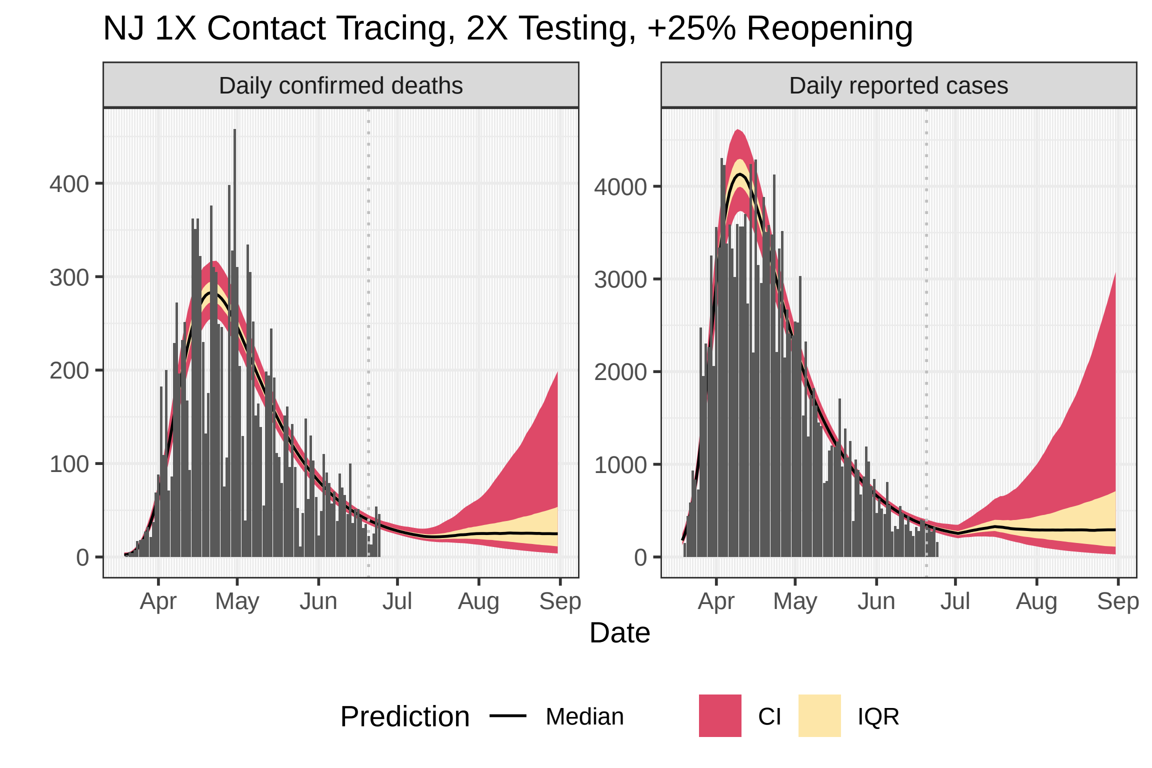
NJ 1X Contact Tracing, 2X Testing, +25% Reopening
Daily confirmed deaths
Daily reported cases
0
100
200
300
400
0
1000
2000
3000
4000
Apr
May
Jun
Jul
Aug
Sep
Date
Prediction
Median
CI
IQR

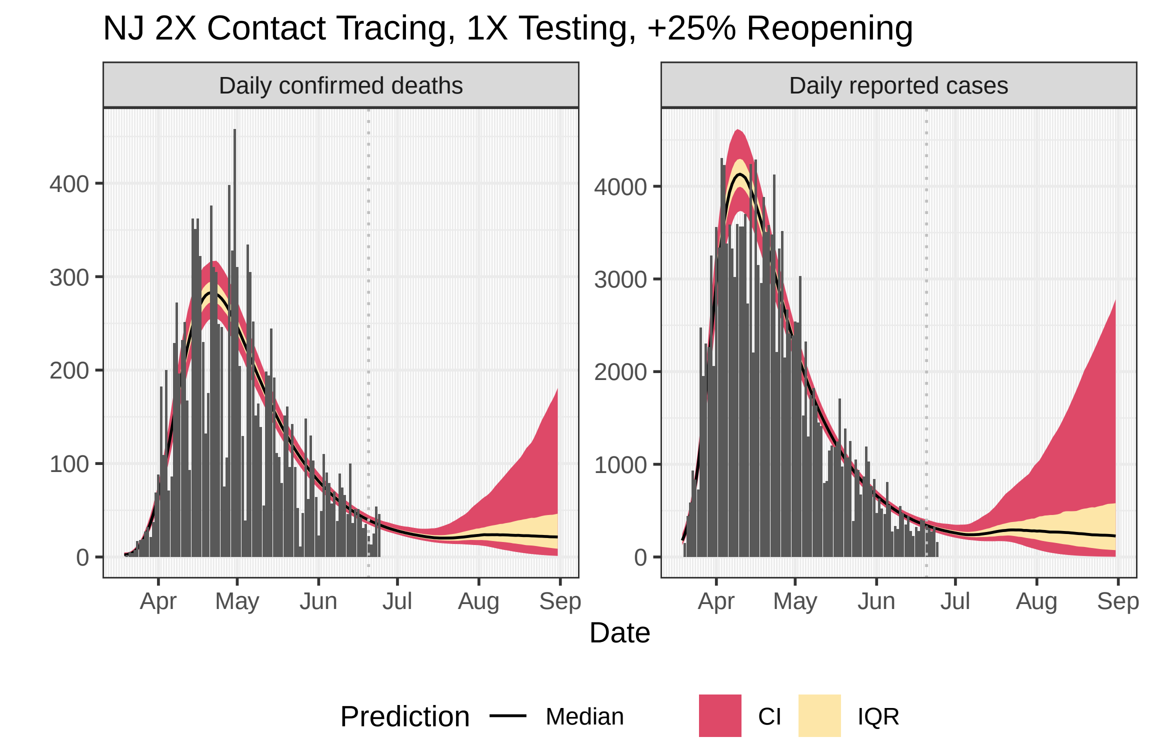

NJ 2X Contact Tracing, 1X Testing, +25% Reopening
Daily confirmed deaths
Daily reported cases
0
100
200
300
400
0
1000
2000
3000
4000
Apr
May
Jun
Jul
Aug
Sep
Date
Prediction
Median
CI
IQR

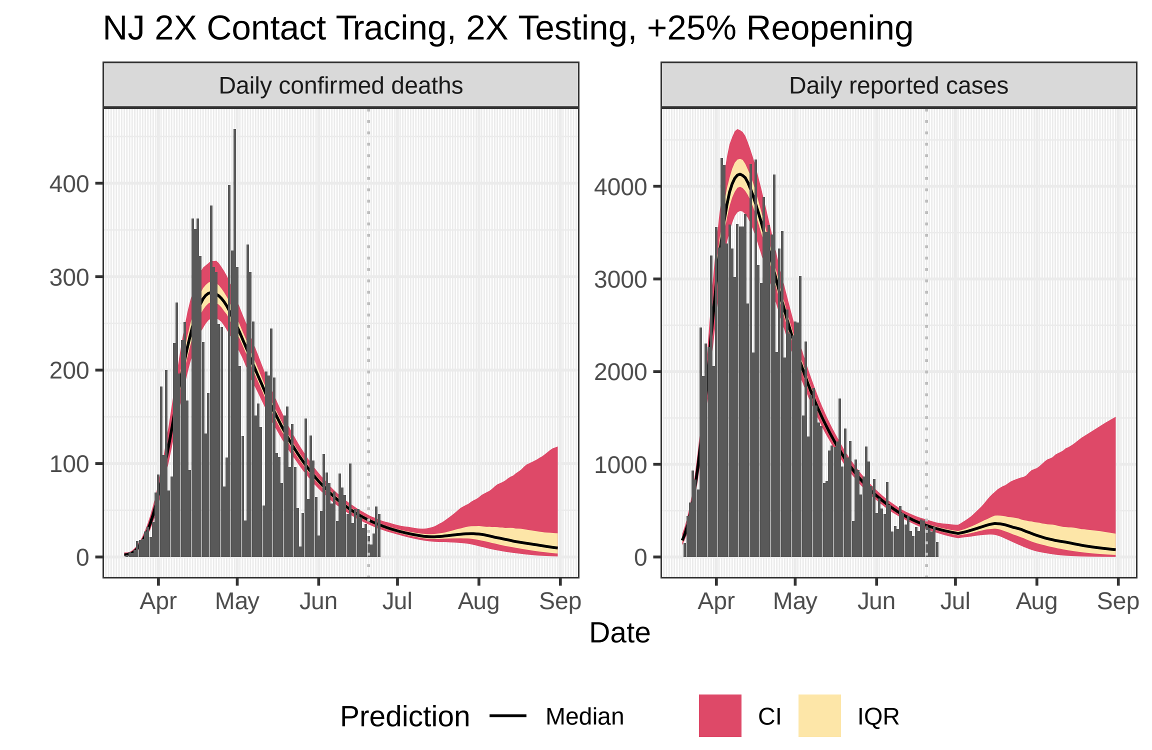
NJ 2X Contact Tracing, 2X Testing, +25% Reopening
Daily confirmed deaths
Daily reported cases
0
100
200
300
400
0
1000
2000
3000
4000
Apr
May
Jun
Jul
Aug
Sep
Date
Prediction
Median
CI
IQR

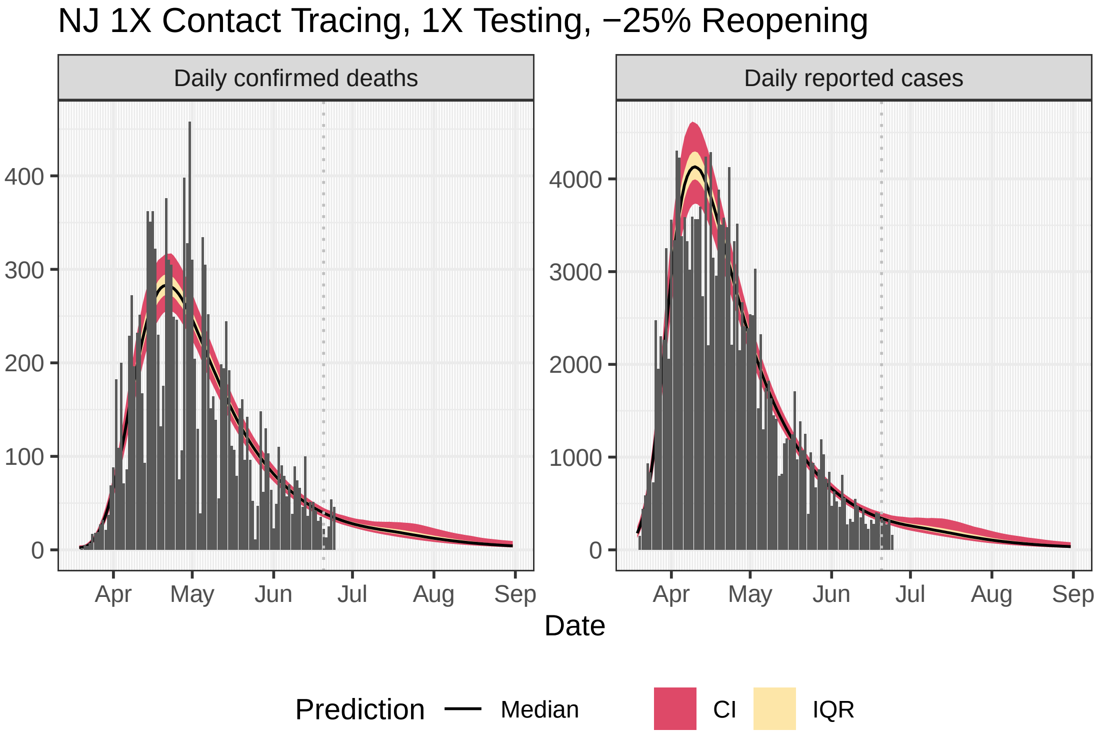
NJ 1X Contact Tracing, 1X Testing, −25% Reopening
Daily confirmed deaths
Daily reported cases
400
300
200
100
0
4000
3000
2000
1000
0
Apr
May
Jun
Jul
Aug
Sep
Date
Prediction
Median
CI
IQR

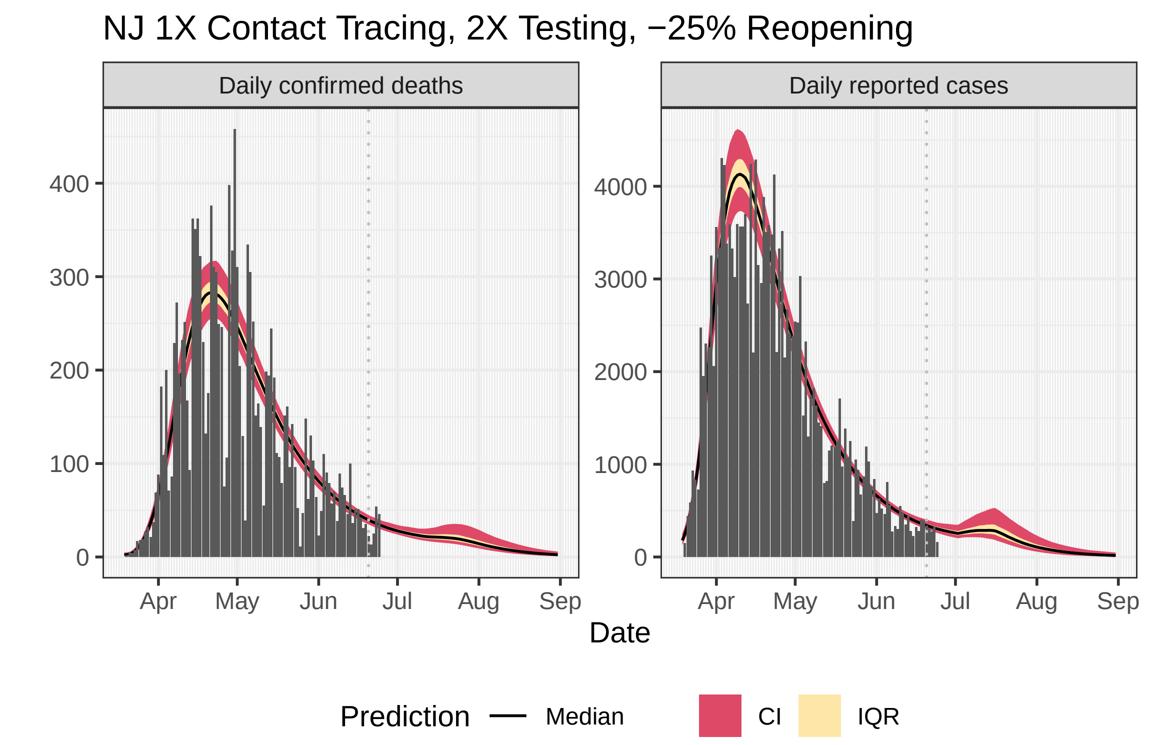

NJ 1X Contact Tracing, 2X Testing, −25% Reopening
Daily confirmed deaths
Daily reported cases
0
100
200
300
400
0
1000
2000
3000
4000
Apr
May
Jun
Jul
Aug
Sep
Date
Prediction
Median
CI
IQR

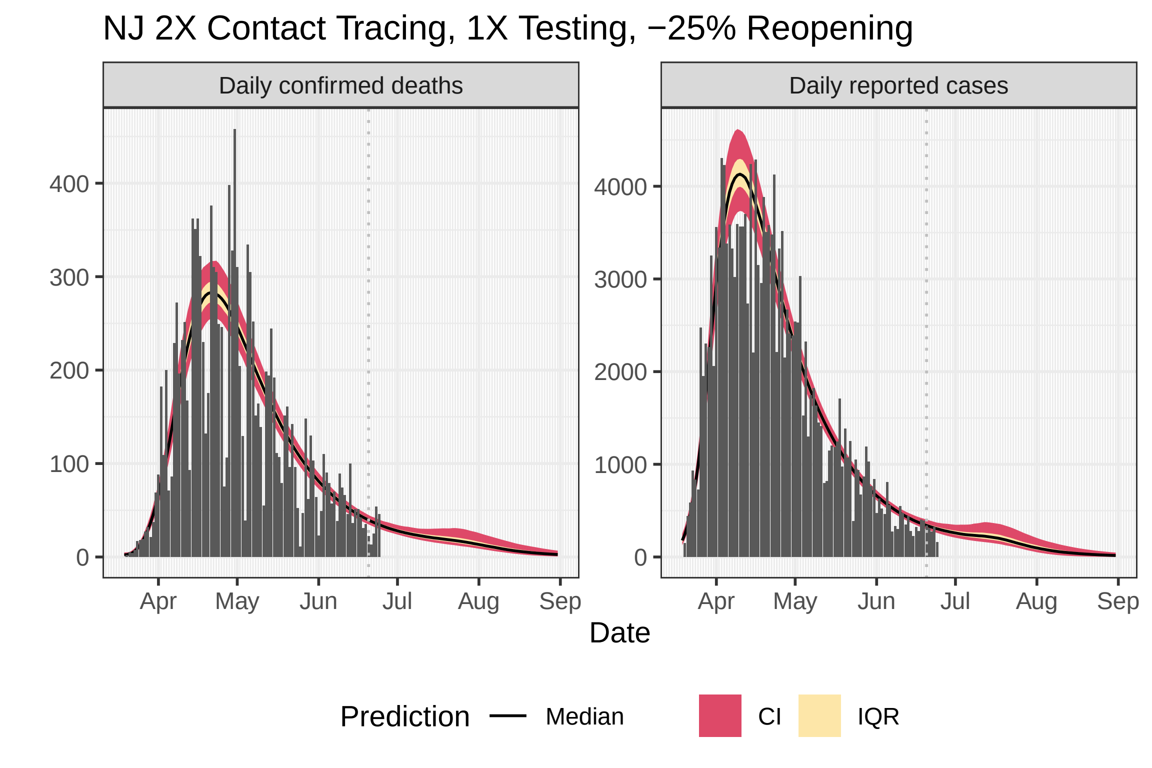
NJ 2X Contact Tracing, 1X Testing, −25% Reopening
Daily confirmed deaths
Daily reported cases
0
100
200
300
400
1000
2000
3000
4000
Apr
May
Jun
Jul
Aug
Sep
Date
Prediction
Median
CI
IQR

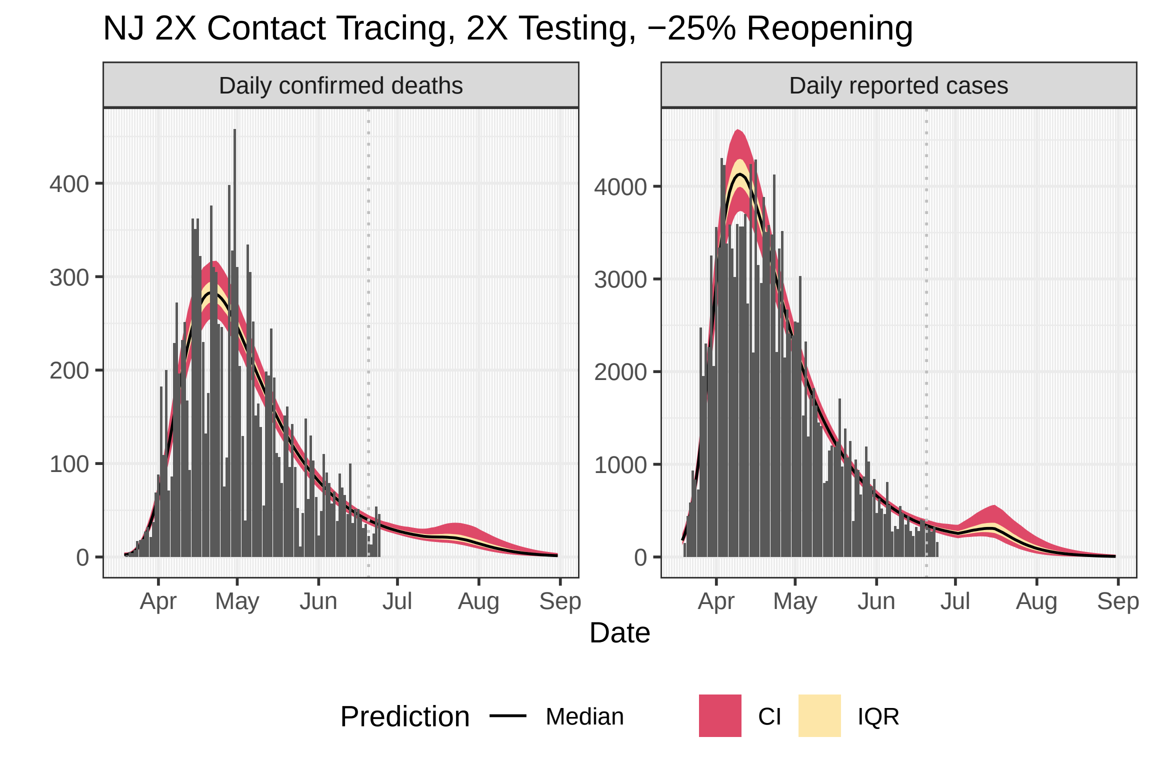
NJ 2X Contact Tracing, 2X Testing, −25% Reopening
Daily confirmed deaths
Daily reported cases
400
300
200
100
0
4000
3000
2000
1000
0
Apr
May
Jun
Jul
Aug
Sep
Date
Prediction
Median
CI
IQR

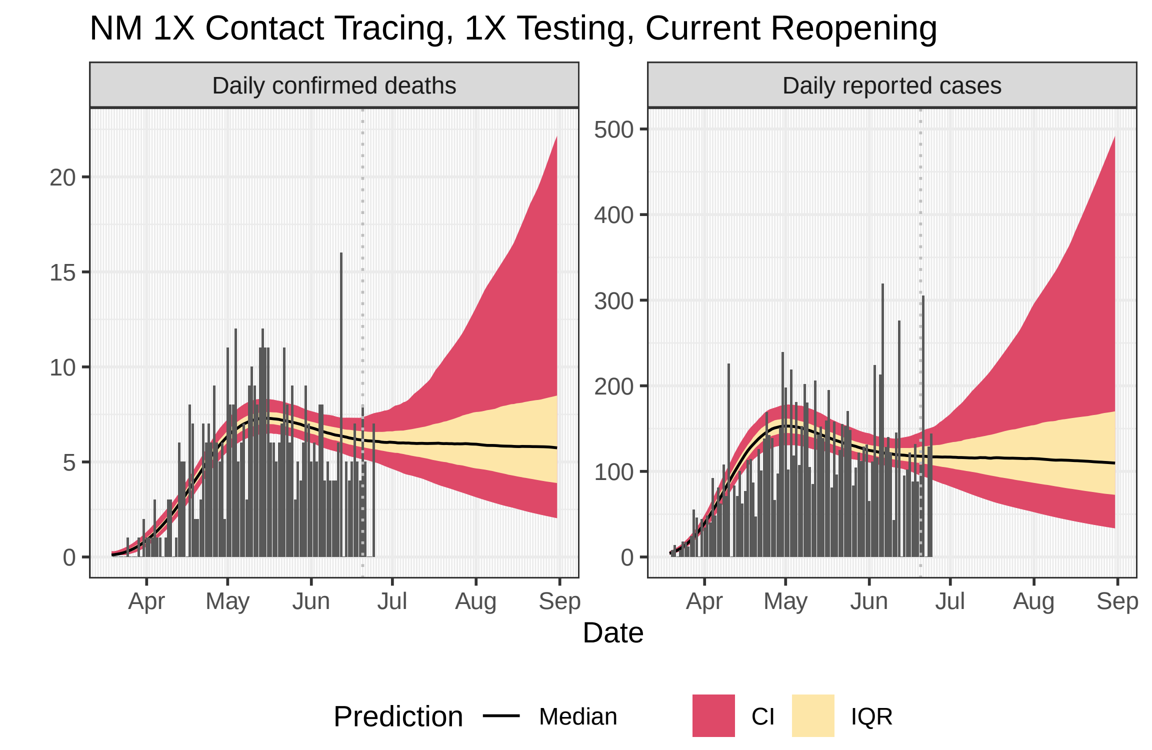

NM 1X Contact Tracing, 1X Testing, Current Reopening
Daily confirmed deaths
Daily reported cases
0
5
10
15
20
0
100
200
300
400
500
Apr
May
Jun
Jul
Aug
Sep
Date
Prediction
Median
CI
IQR

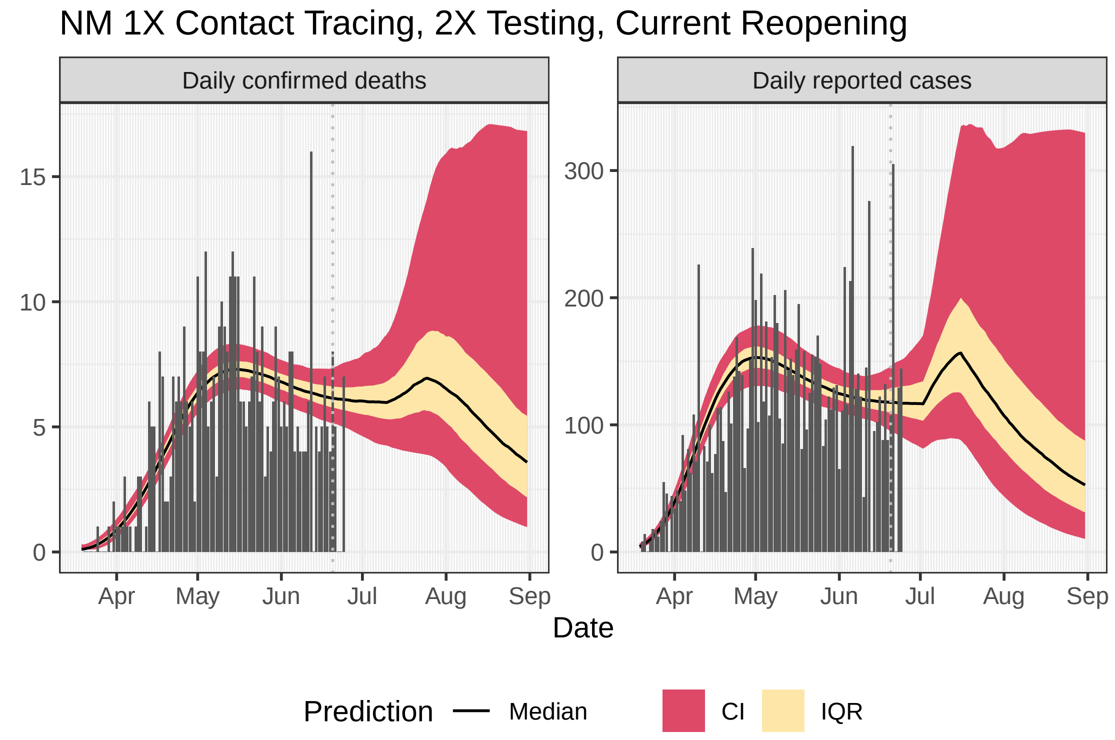

NM 1X Contact Tracing, 2X Testing, Current Reopening
Daily confirmed deaths
Daily reported cases
0
5
10
15
0
100
200
300
Apr
May
Jun
Jul
Aug
Sep
Date
Prediction
Median
CI
IQR

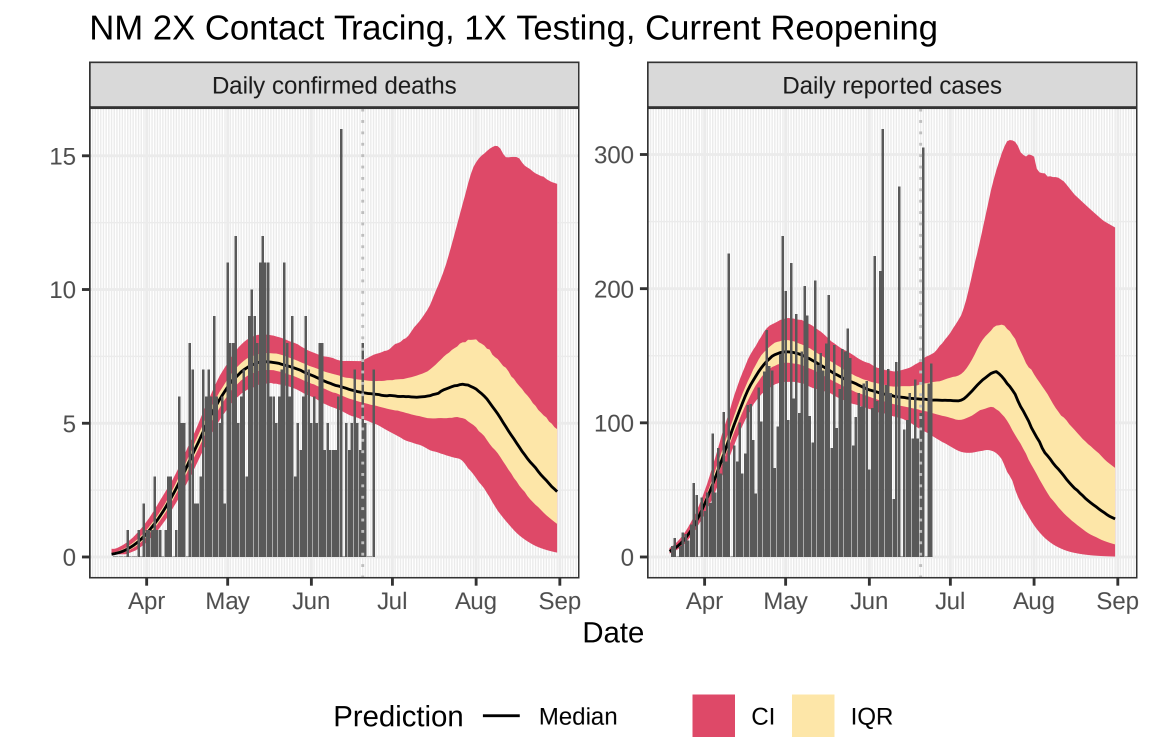

NM 2X Contact Tracing, 1X Testing, Current Reopening
Daily confirmed deaths
Daily reported cases
15
10
5
0
300
200
100
0
Apr
May
Jun
Jul
Aug
Sep
Date
Prediction
Median
CI
IQR

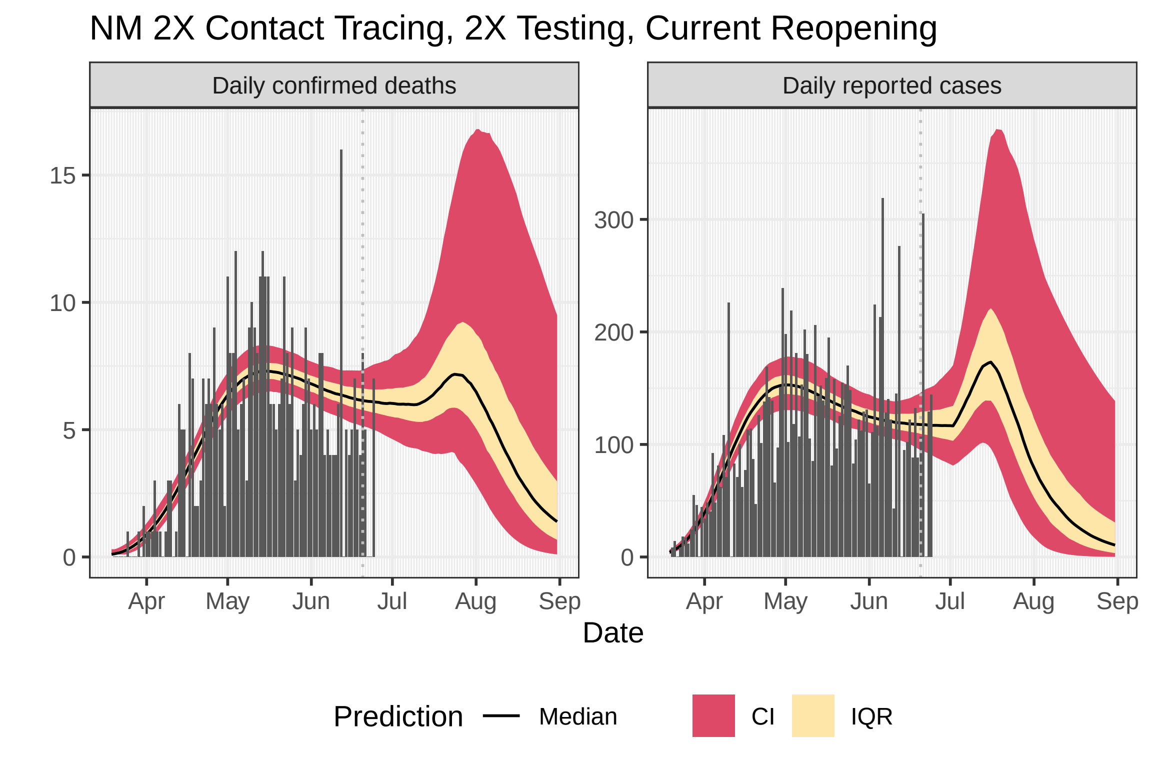

NM 2X Contact Tracing, 2X Testing, Current Reopening
Daily confirmed deaths
Daily reported cases
15
10
5
0
300
200
100
0
Apr
May
Jun
Jul
Aug
Sep
Date
Prediction
Median
CI
IQR

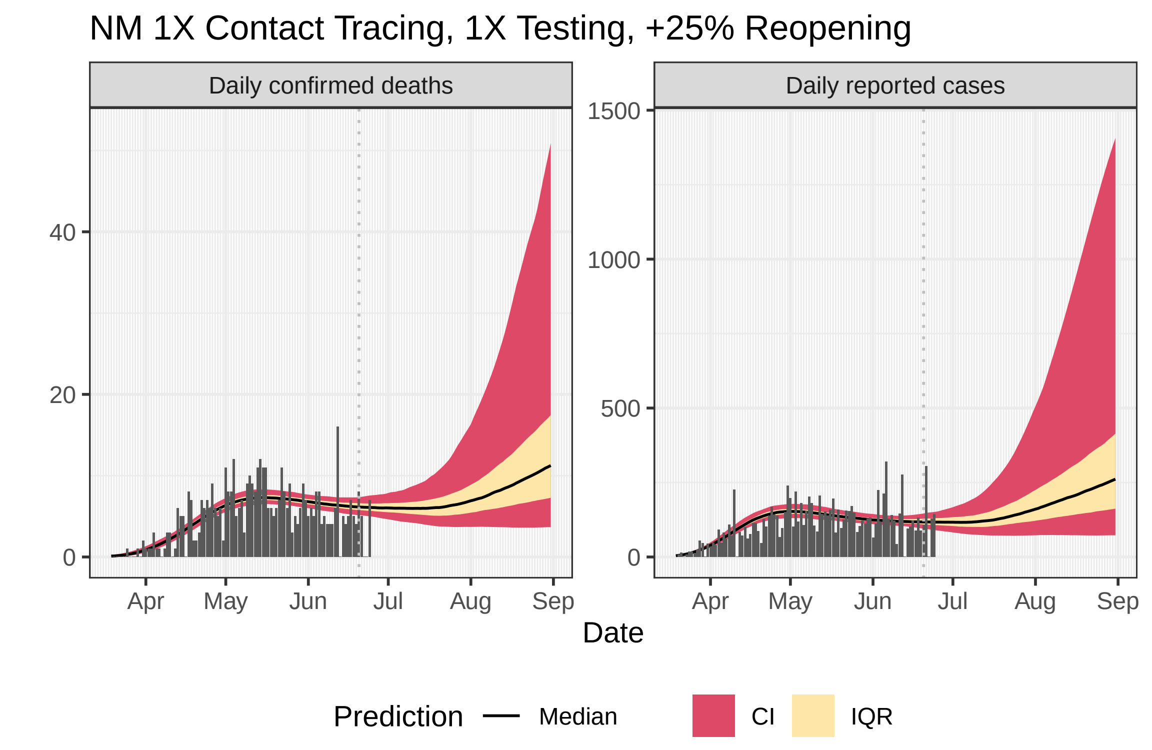

NM 1X Contact Tracing, 1X Testing, +25% Reopening
Daily confirmed deaths
Daily reported cases
0
20
40
0
500
1000
1500
Apr
May
Jun
Jul
Aug
Sep
Date
Prediction
Median
CI
IQR

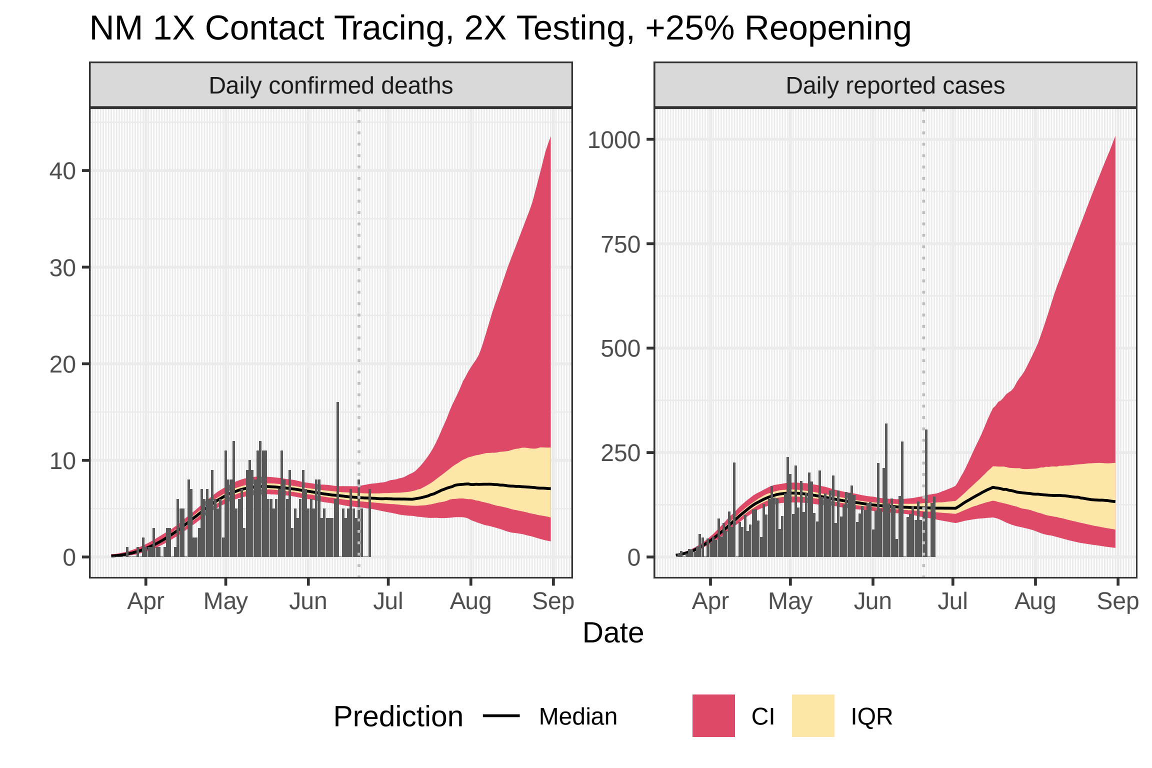
NM 1X Contact Tracing, 2X Testing, +25% Reopening
Daily confirmed deaths
Daily reported cases
40
30
20
10
0
1000
750
500
250
0
Apr
May
Jun
Jul
Aug
Sep
Date
Prediction
Median
CI
IQR

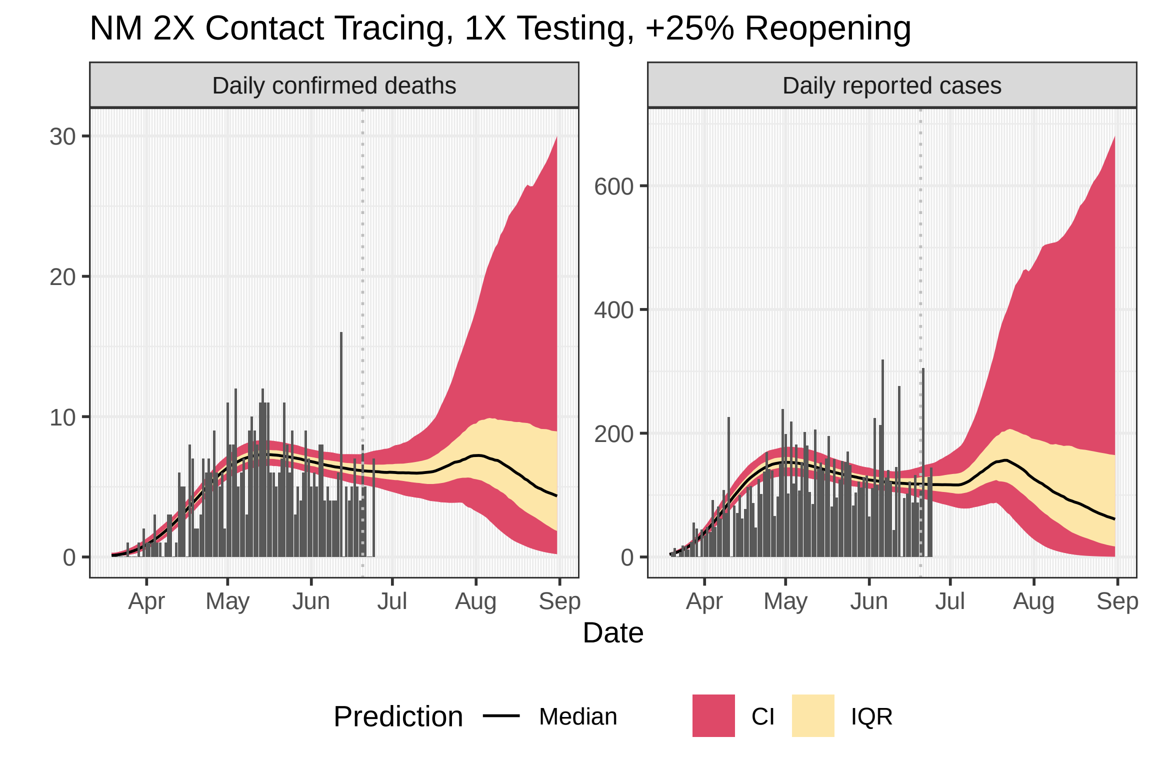

NM 2X Contact Tracing, 1X Testing, +25% Reopening
Daily confirmed deaths
Daily reported cases
30
20
10
0
600
400
200
0
Apr
May
Jun
Jul
Aug
Sep
Date
Prediction
Median
CI
IQR

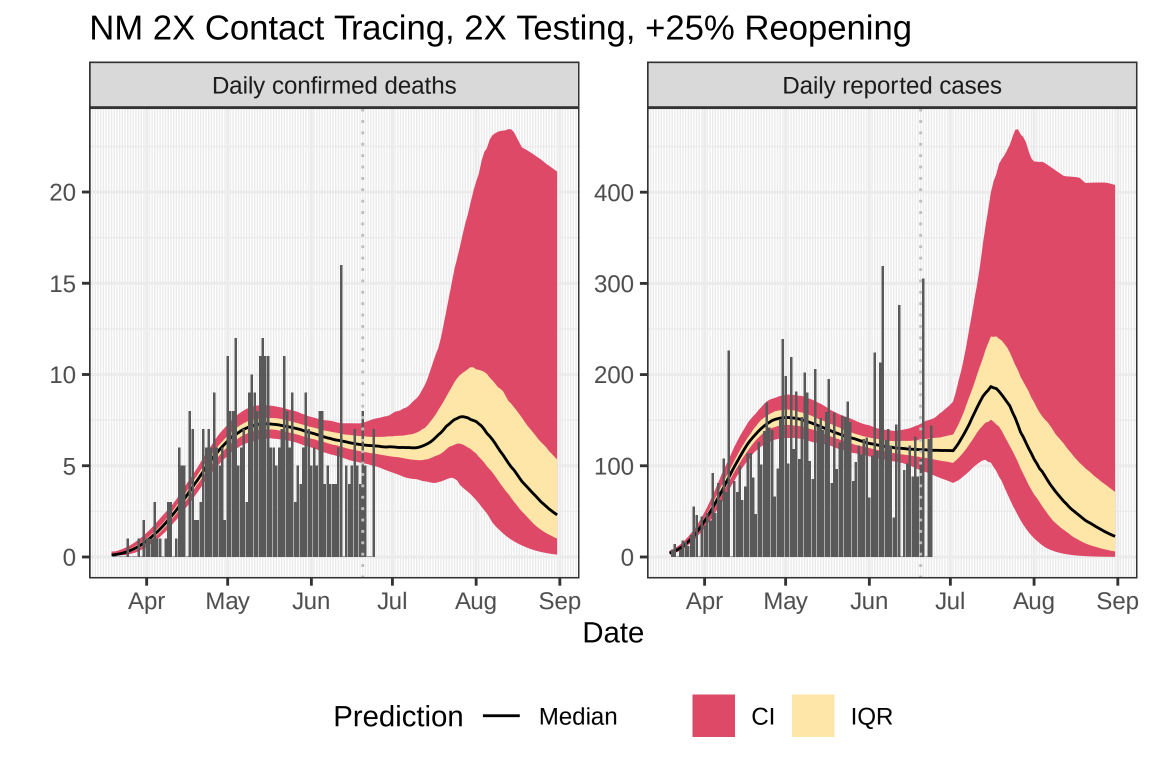
NM 2X Contact Tracing, 2X Testing, +25% Reopening
Daily confirmed deaths
Daily reported cases
0
5
10
15
20
0
100
200
300
400
Apr
May
Jun
Jul
Aug
Sep
Date
Prediction
Median
CI
IQR

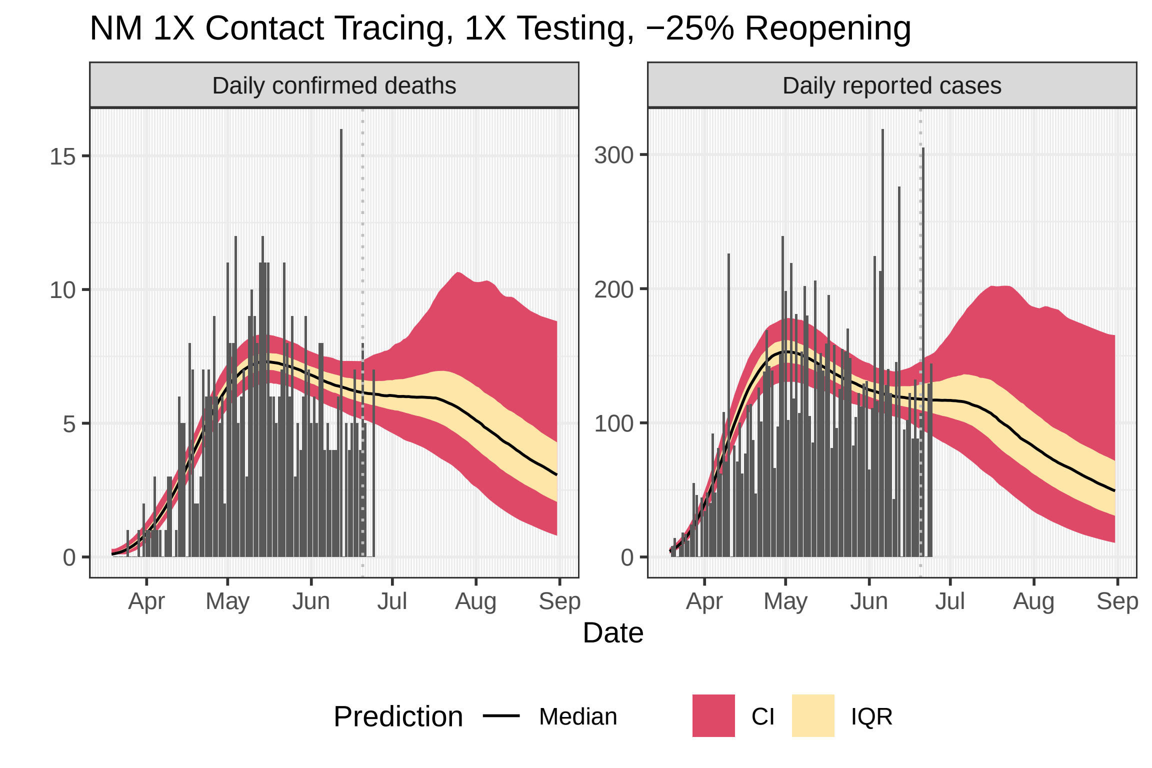
NM 1X Contact Tracing, 1X Testing, −25% Reopening
Daily confirmed deaths
Daily reported cases
15
10
5
0
300
200
100
0
Apr
May
Jun
Jul
Aug
Sep
Date
Prediction
Median
CI
IQR

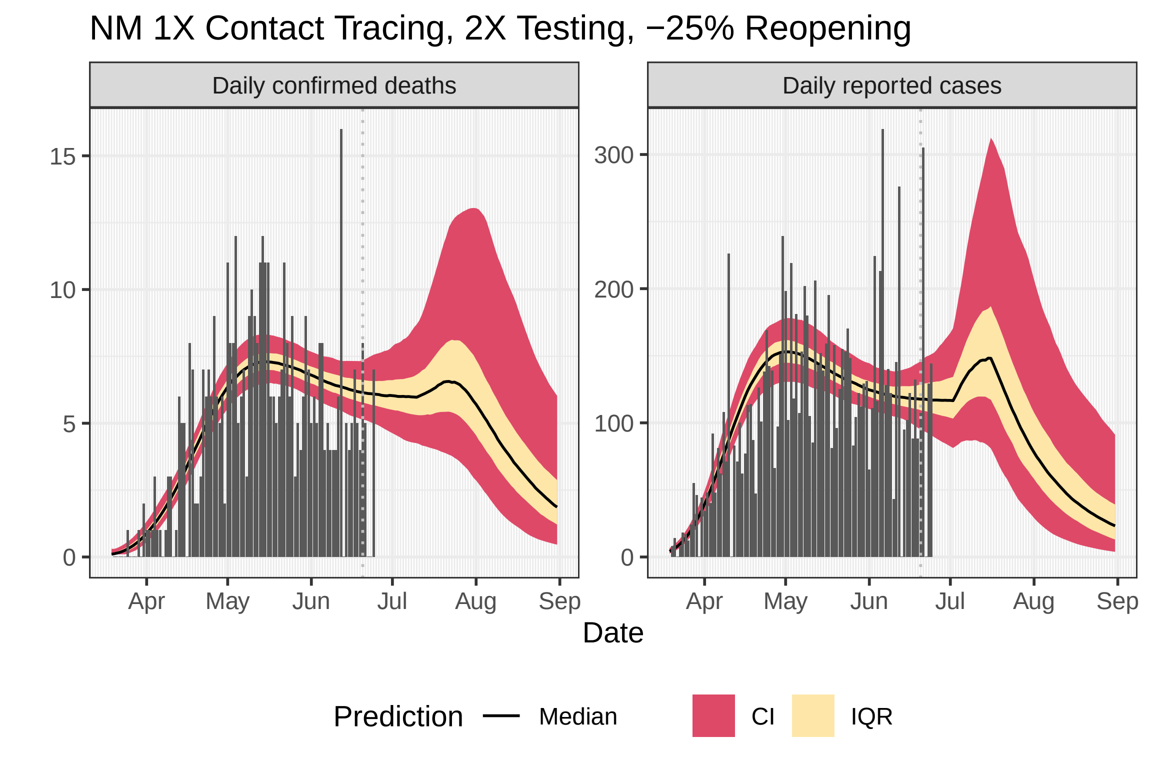
NM 1X Contact Tracing, 2X Testing, −25% Reopening
Daily confirmed deaths
Daily reported cases
15
10
5
0
300
200
100
0
Apr
May
Jun
Jul
Aug
Sep
Date
Prediction
Median
CI
IQR

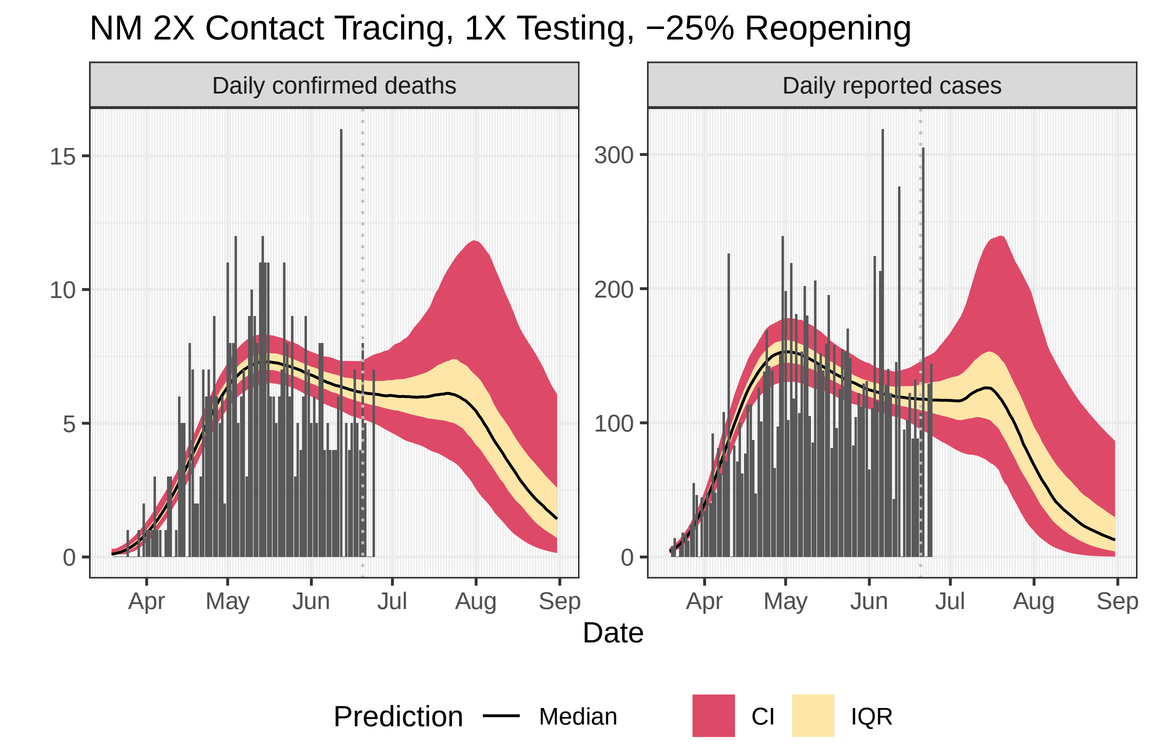
NM 2X Contact Tracing, 1X Testing, −25% Reopening
Daily confirmed deaths
Daily reported cases
0
5
10
15
0
100
200
300
Apr
May
Jun
Jul
Aug
Sep
Date
Prediction
Median
CI
IQR

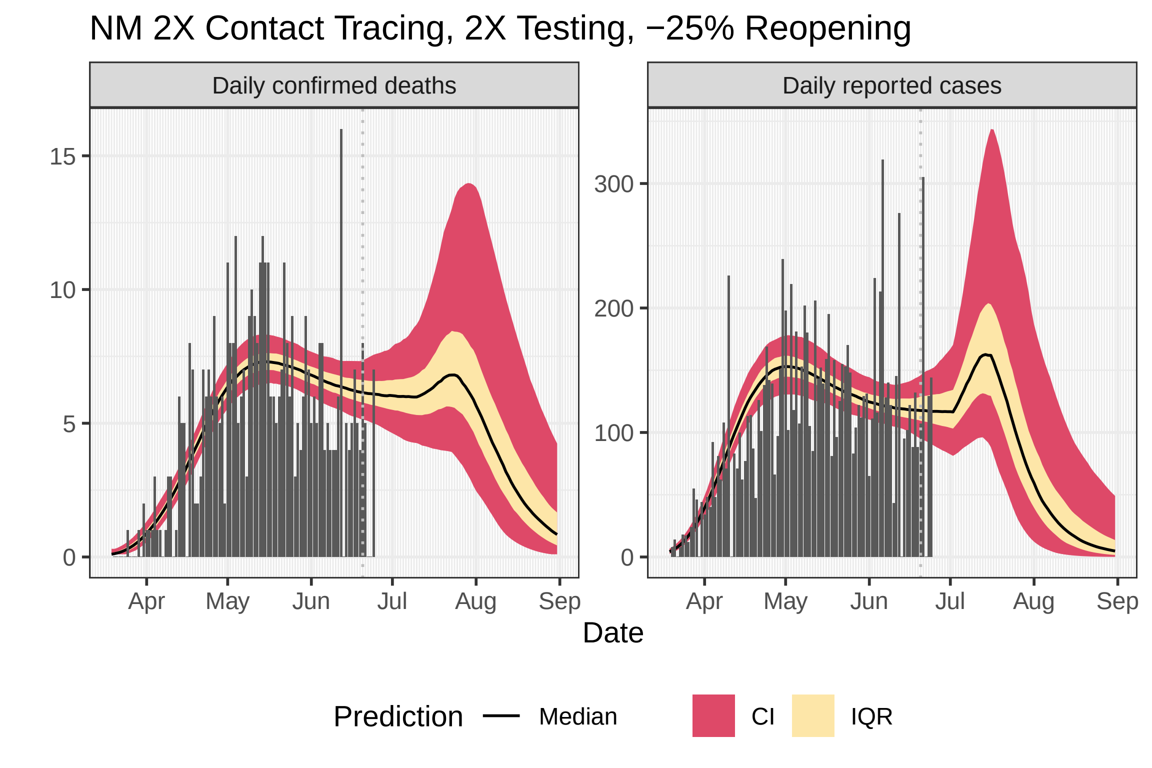

NM 2X Contact Tracing, 2X Testing, −25% Reopening
Daily confirmed deaths
Daily reported cases
15
10
5
0
300
200
100
0
Apr
May
Jun
Jul
Aug
Sep
Date
Prediction
Median
CI
IQR

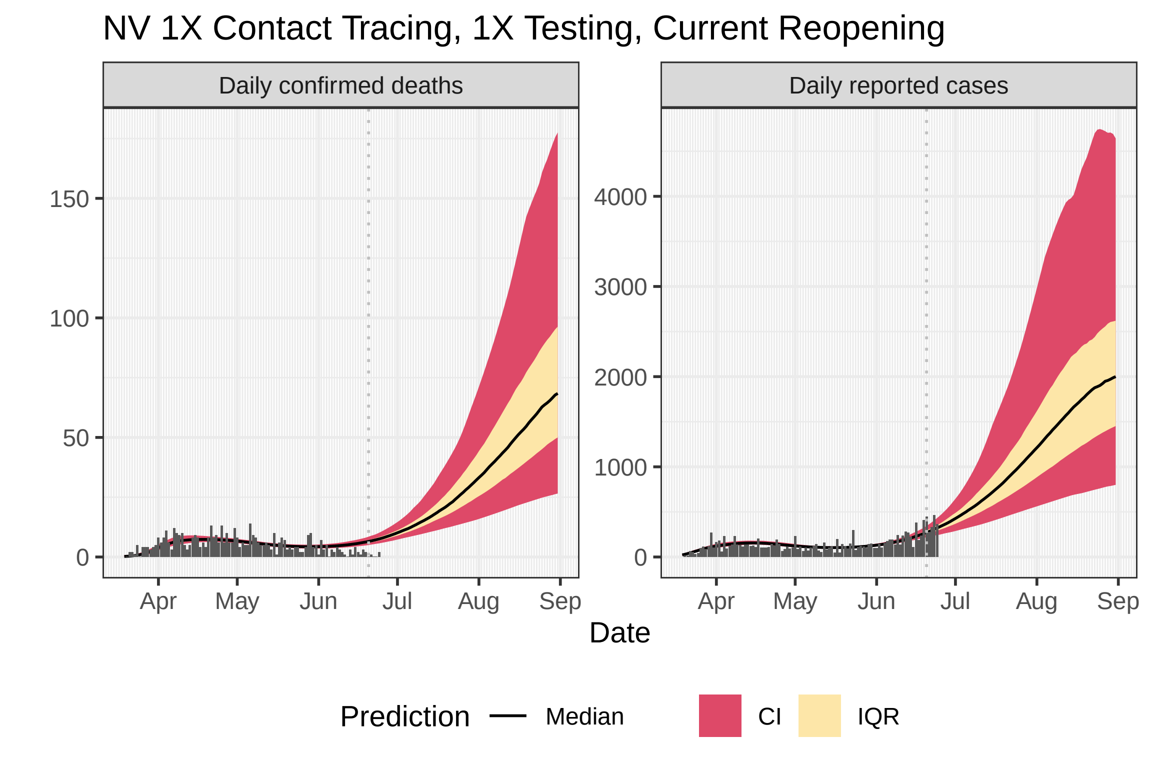
NV 1X Contact Tracing, 1X Testing, Current Reopening
Daily confirmed deaths
Daily reported cases
0
50
100
150
0
1000
2000
3000
4000
Apr
May
Jun
Jul
Aug
Sep
Date
Prediction
Median
CI
IQR

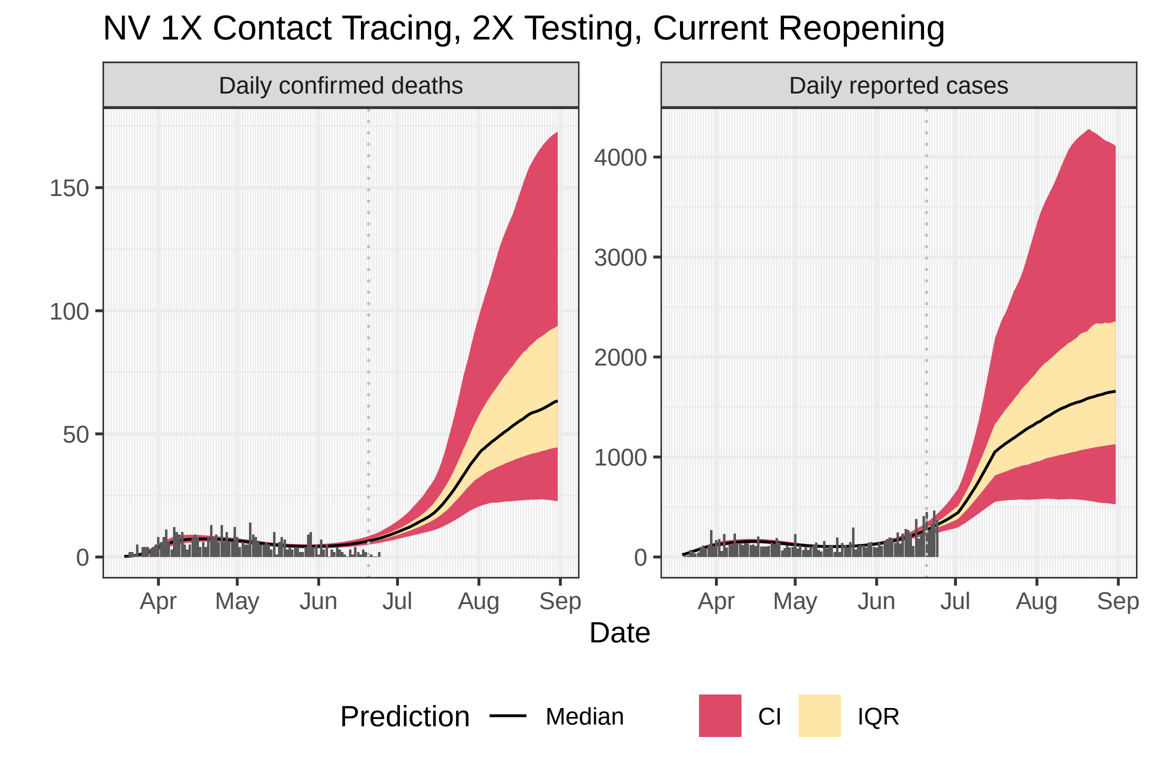
NV 1X Contact Tracing, 2X Testing, Current Reopening
Daily confirmed deaths
Daily reported cases
150
100
50
0
4000
3000
2000
1000
0
Apr
May
Jun
Jul
Aug
Sep
Date
Prediction
Median
CI
IQR

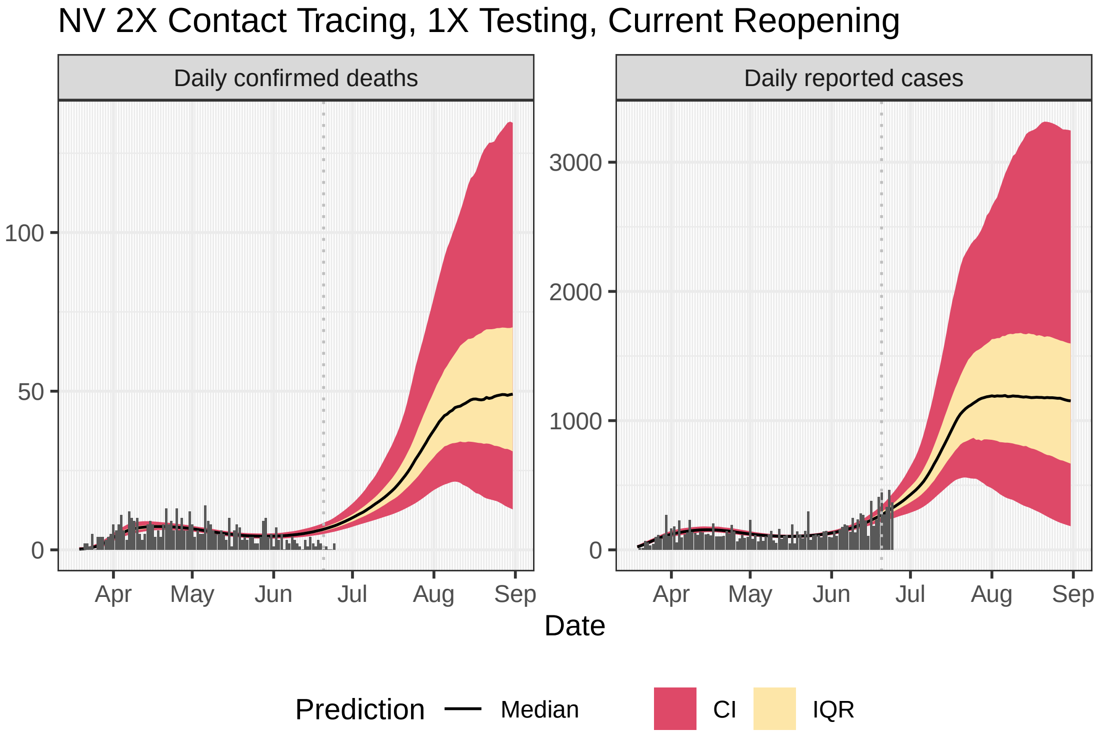

NV 2X Contact Tracing, 1X Testing, Current Reopening
Daily confirmed deaths
Daily reported cases
100
50
0
3000
2000
1000
0
Apr
May
Jun
Jul
Aug
Sep
Date
Prediction
Median
CI
IQR

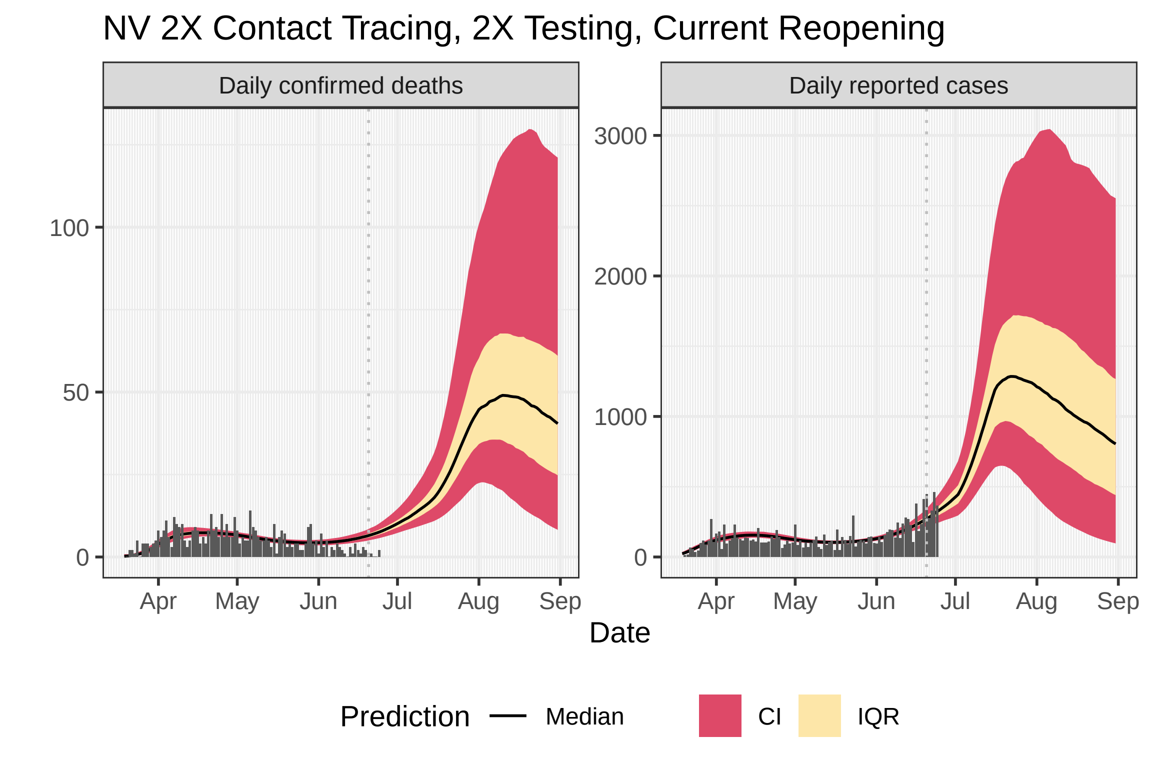
NV 2X Contact Tracing, 2X Testing, Current Reopening
Daily confirmed deaths
Daily reported cases
100
50
0
3000
2000
1000
0
Apr
May
Jun
Jul
Aug
Sep
Date
Prediction
Median
CI
IQR

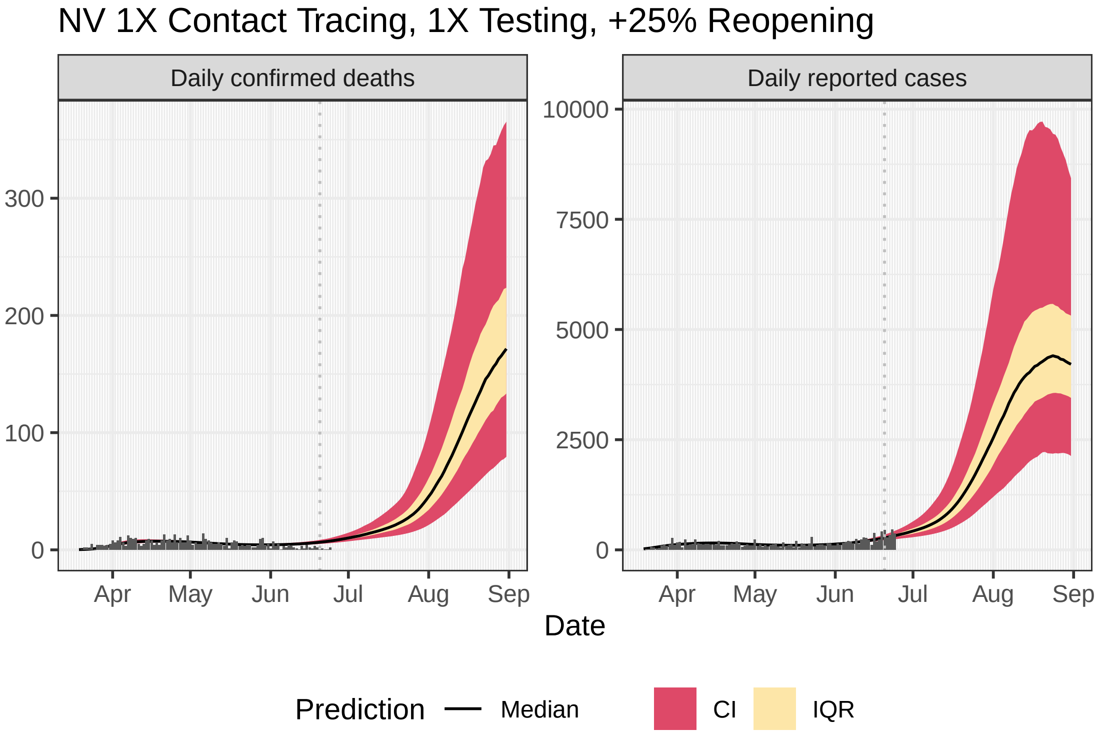

NV 1X Contact Tracing, 1X Testing, +25% Reopening
Daily confirmed deaths
Daily reported cases
300
200
100
0
10000
7500
5000
2500
0
Apr
May
Jun
Jul
Aug
Sep
Date
Prediction
Median
CI
IQR

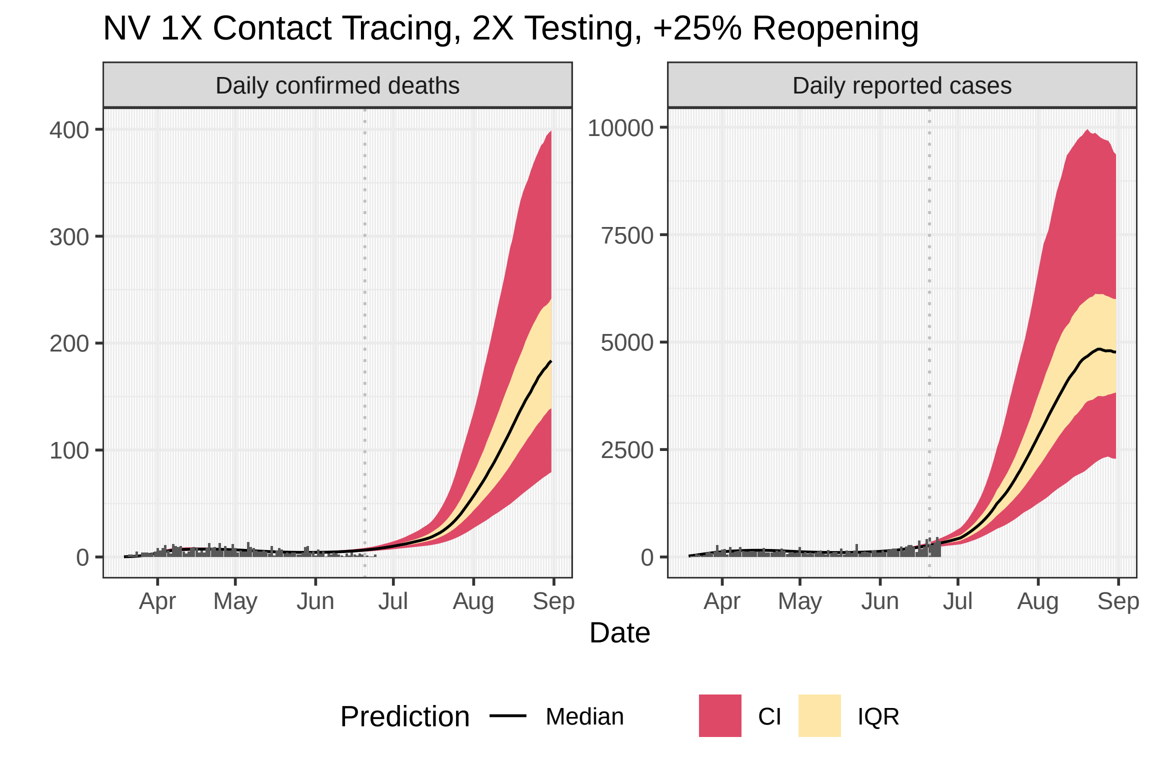

NV 1X Contact Tracing, 2X Testing, +25% Reopening
Daily confirmed deaths
Daily reported cases
400
300
200
100
0
10000
7500
5000
2500
0
Apr
May
Jun
Jul
Aug
Sep
Date
Prediction
Median
CI
IQR

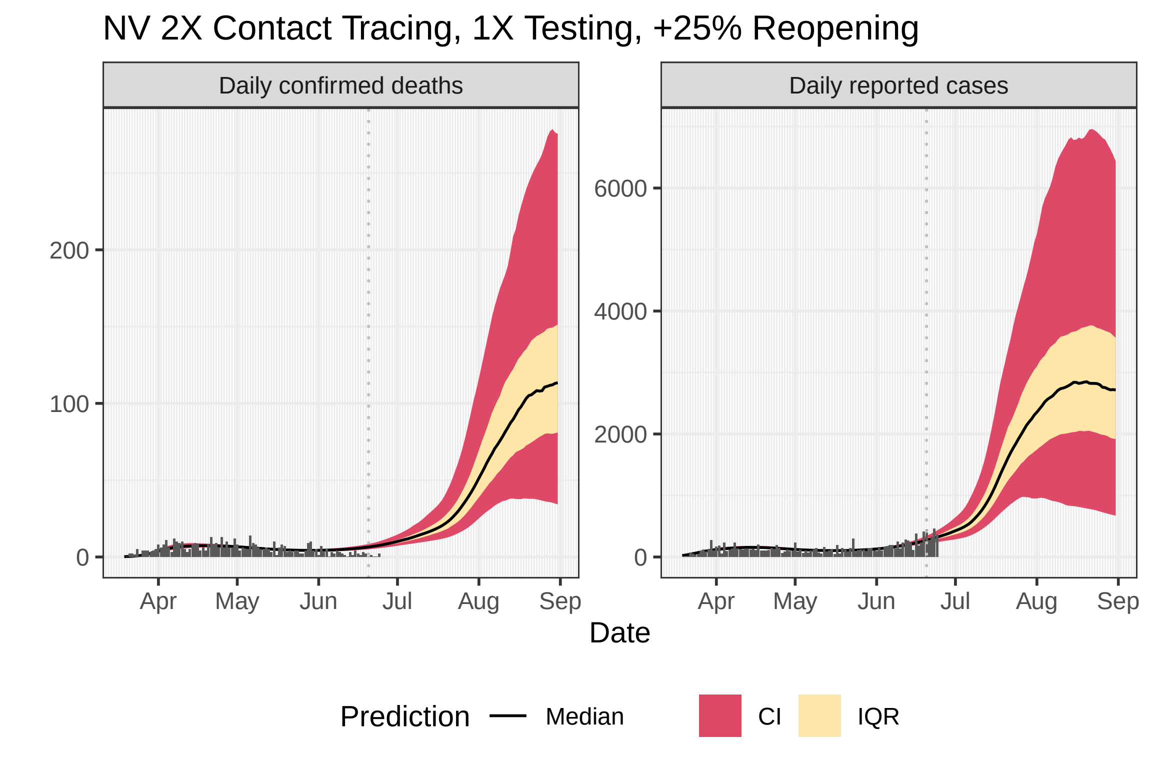
NV 2X Contact Tracing, 1X Testing, +25% Reopening
Daily confirmed deaths
Daily reported cases
0
100
200
0
2000
4000
6000
Apr
May
Jun
Jul
Aug
Sep
Date
Prediction
Median
CI
IQR

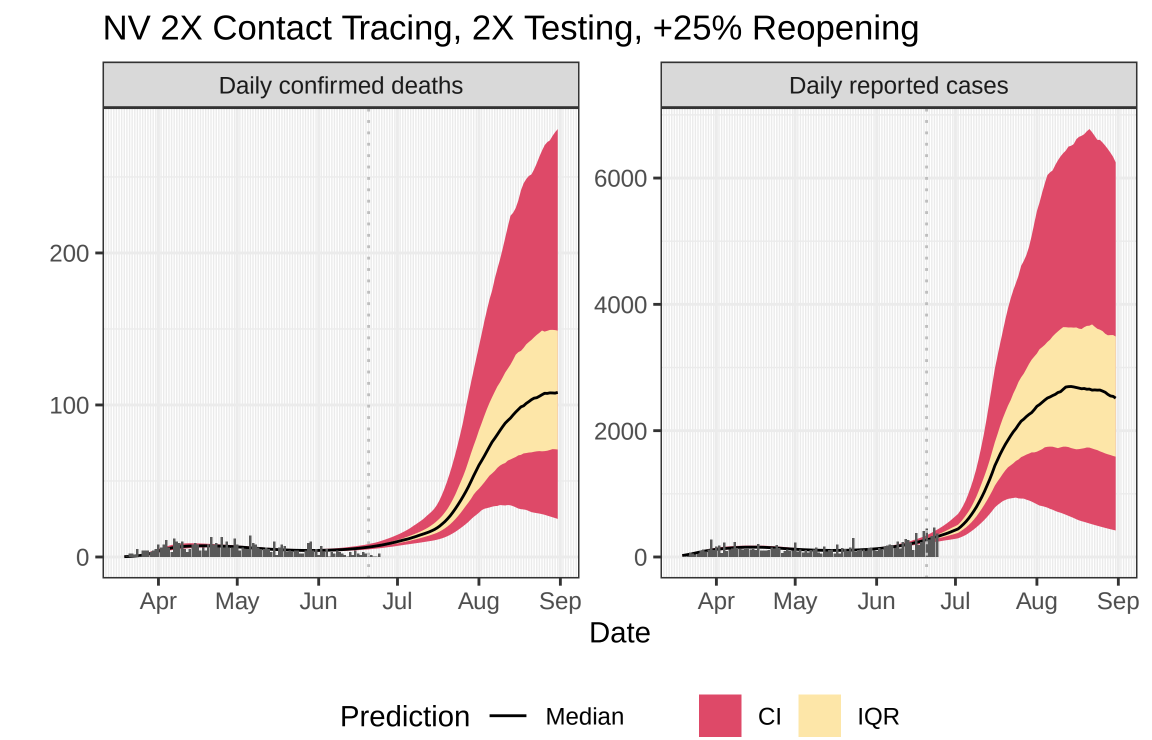

NV 2X Contact Tracing, 2X Testing, +25% Reopening
Daily confirmed deaths
Daily reported cases
0
100
200
2000
4000
6000
Apr
May
Jun
Jul
Aug
Sep
Date
Prediction
Median
CI
IQR

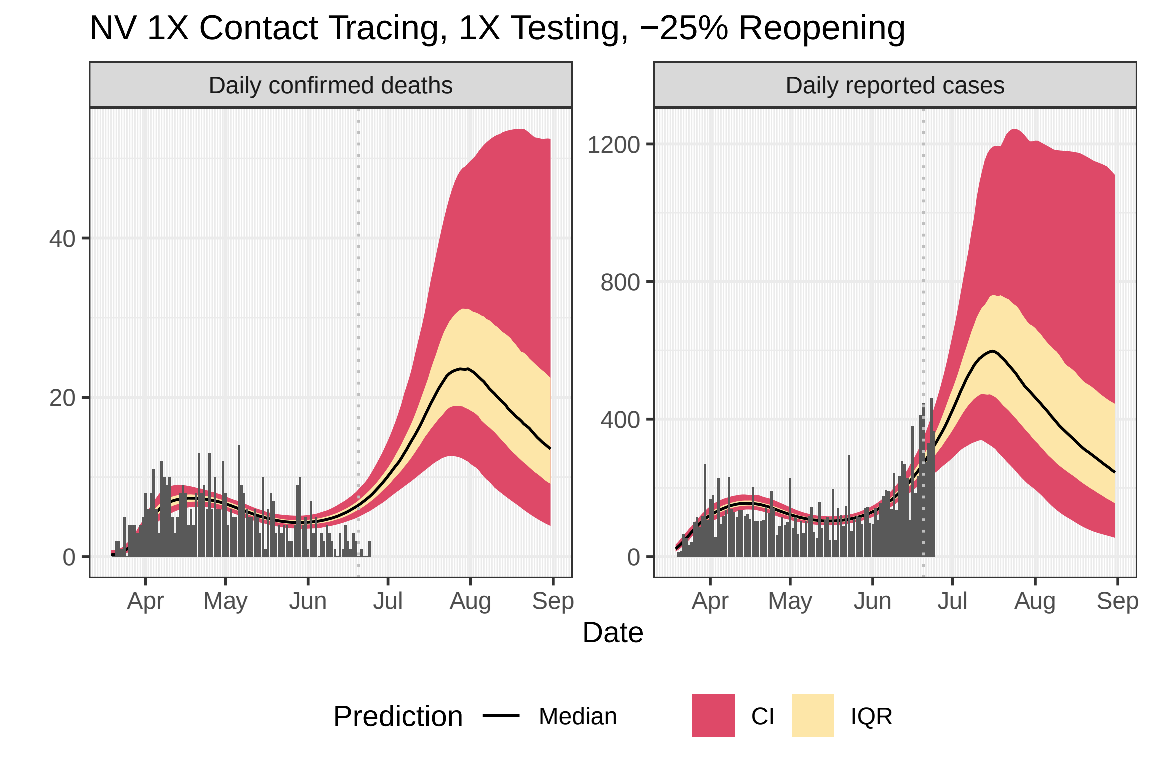
NV 1X Contact Tracing, 1X Testing, −25% Reopening
Daily confirmed deaths
Daily reported cases
0
20
40
0
400
800
1200
Apr
May
Jun
Jul
Aug
Sep
Apr
May
Jun
Jul
Aug
Sep
Date
Prediction
Median
CI
IQR

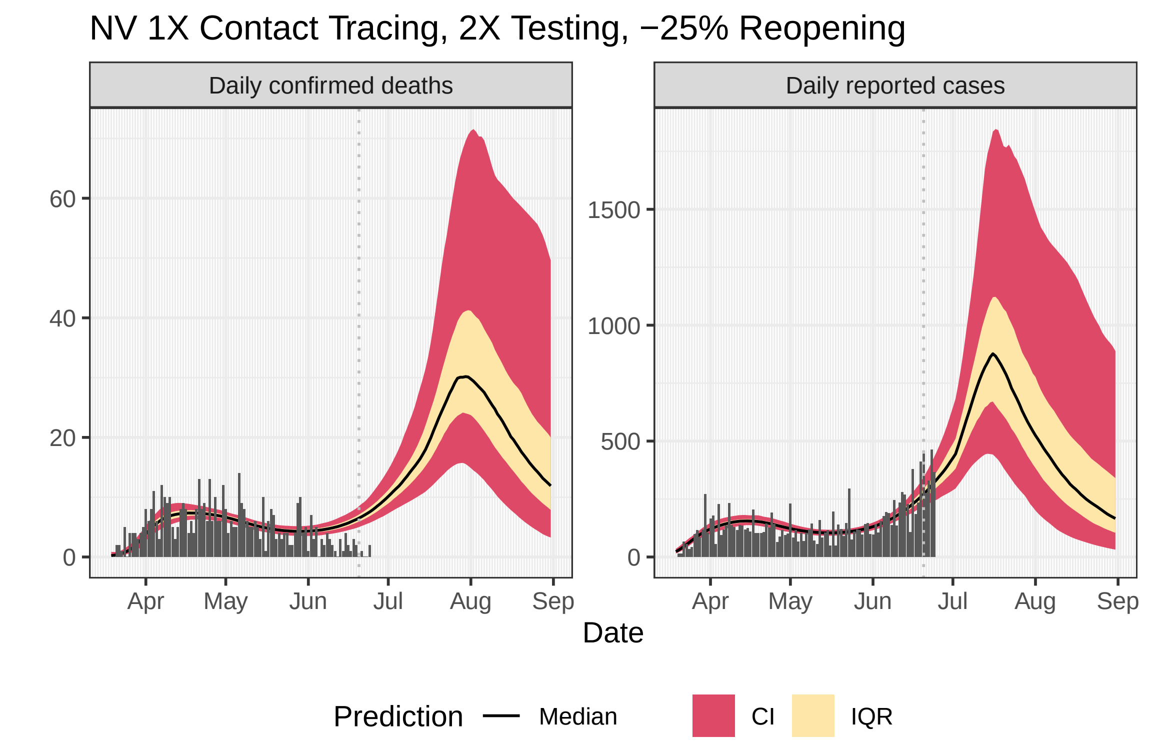

NV 1X Contact Tracing, 2X Testing, −25% Reopening
Daily confirmed deaths
Daily reported cases
0
20
40
60
0
500
1000
1500
Apr
May
Jun
Jul
Aug
Sep
Date
Prediction
Median
CI
IQR

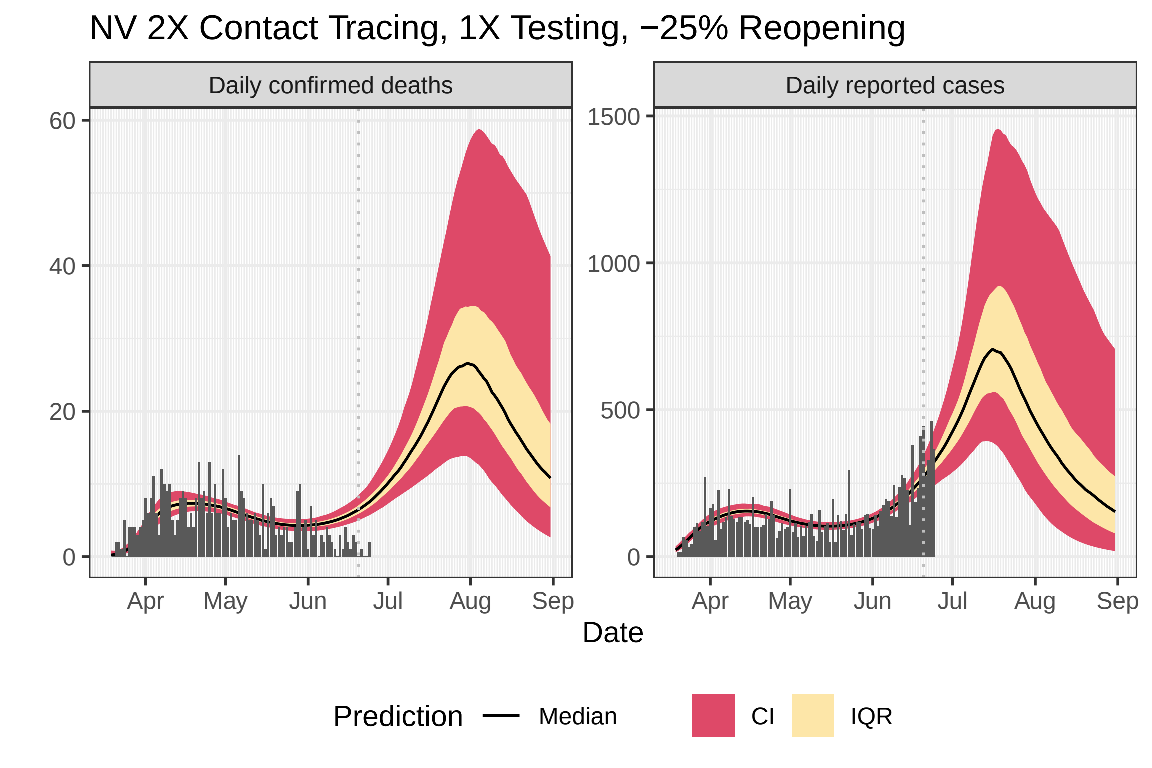

NV 2X Contact Tracing, 1X Testing, −25% Reopening
Daily confirmed deaths
Daily reported cases
60
40
20
0
1500
1000
500
0
Apr
May
Jun
Jul
Aug
Sep
Date
Prediction
Median
CI
IQR

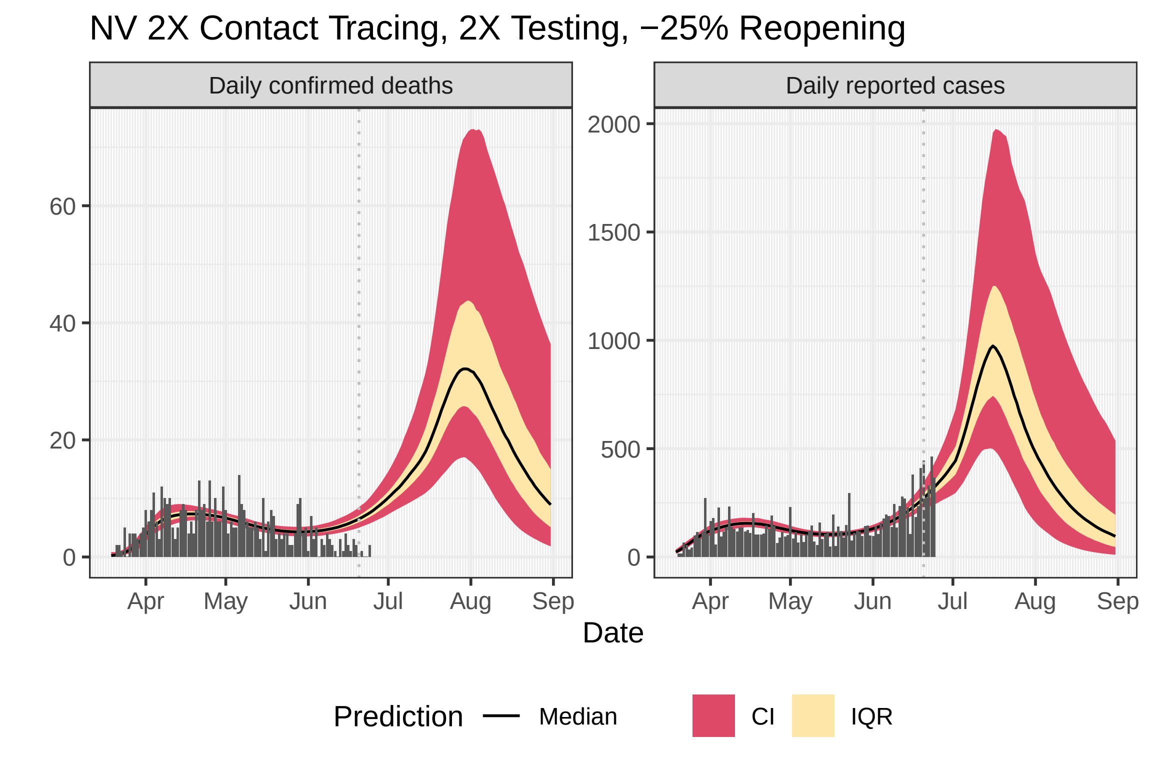

NV 2X Contact Tracing, 2X Testing, −25% Reopening
Daily confirmed deaths
Daily reported cases
0
20
40
60
0
500
1000
1500
2000
Apr
May
Jun
Jul
Aug
Sep
Date
Prediction
Median
CI
IQR

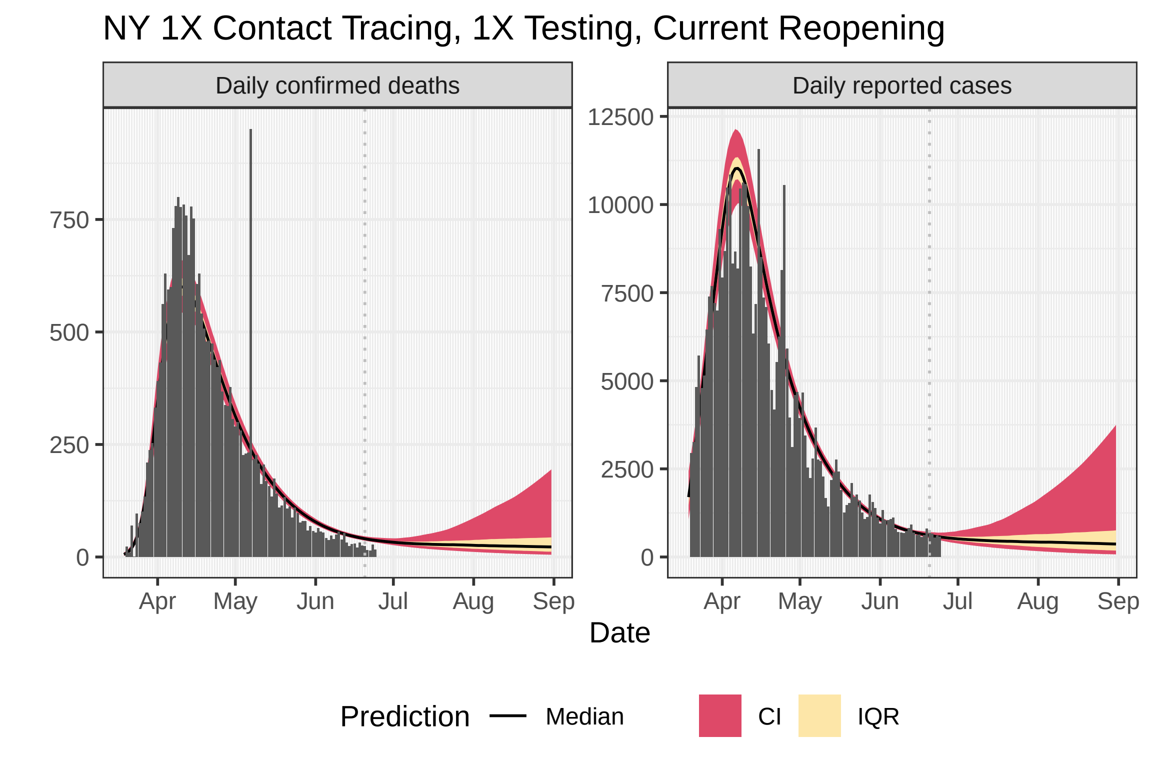

NY 1X Contact Tracing, 1X Testing, Current Reopening
Daily confirmed deaths
Daily reported cases
0
250
500
750
2500
5000
7500
10000
12500
Apr
May
Jun
Jul
Aug
Sep
Date
Prediction
Median
CI
IQR

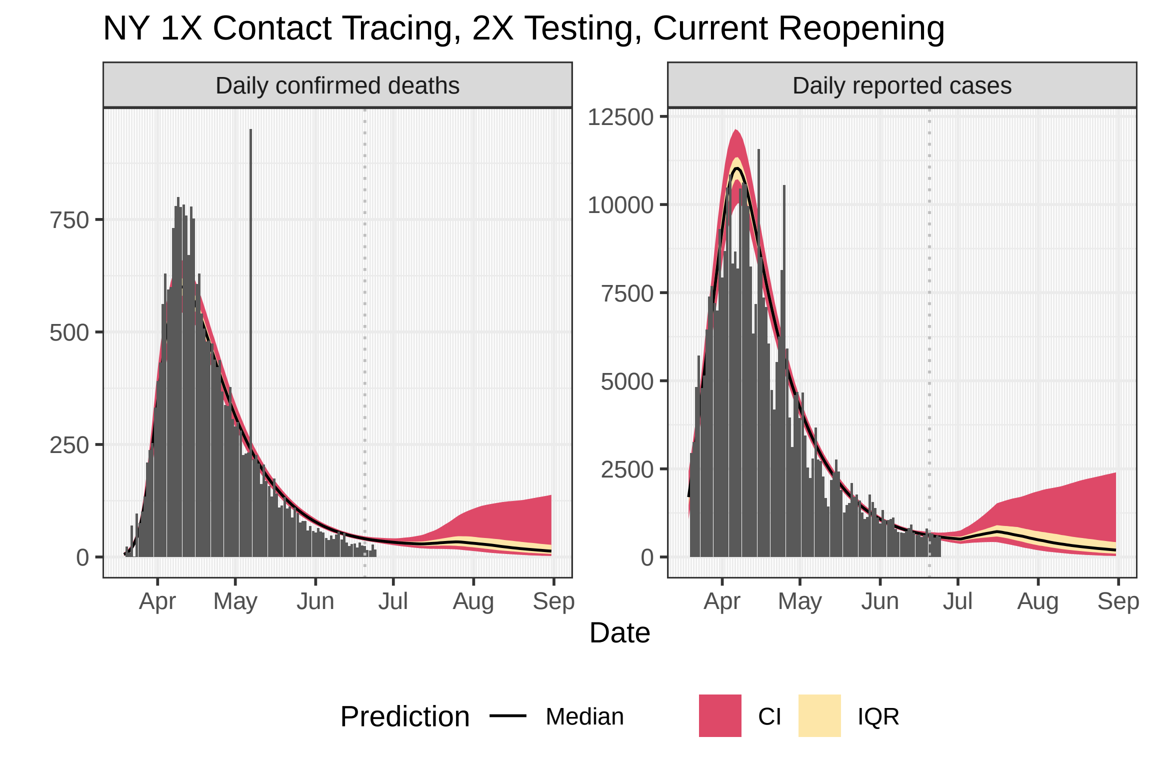

NY 1X Contact Tracing, 2X Testing, Current Reopening
Daily confirmed deaths
Daily reported cases
0
250
500
750
0
2500
5000
7500
10000
12500
Apr
May
Jun
Jul
Aug
Sep
Date
Prediction
Median
CI
IQR

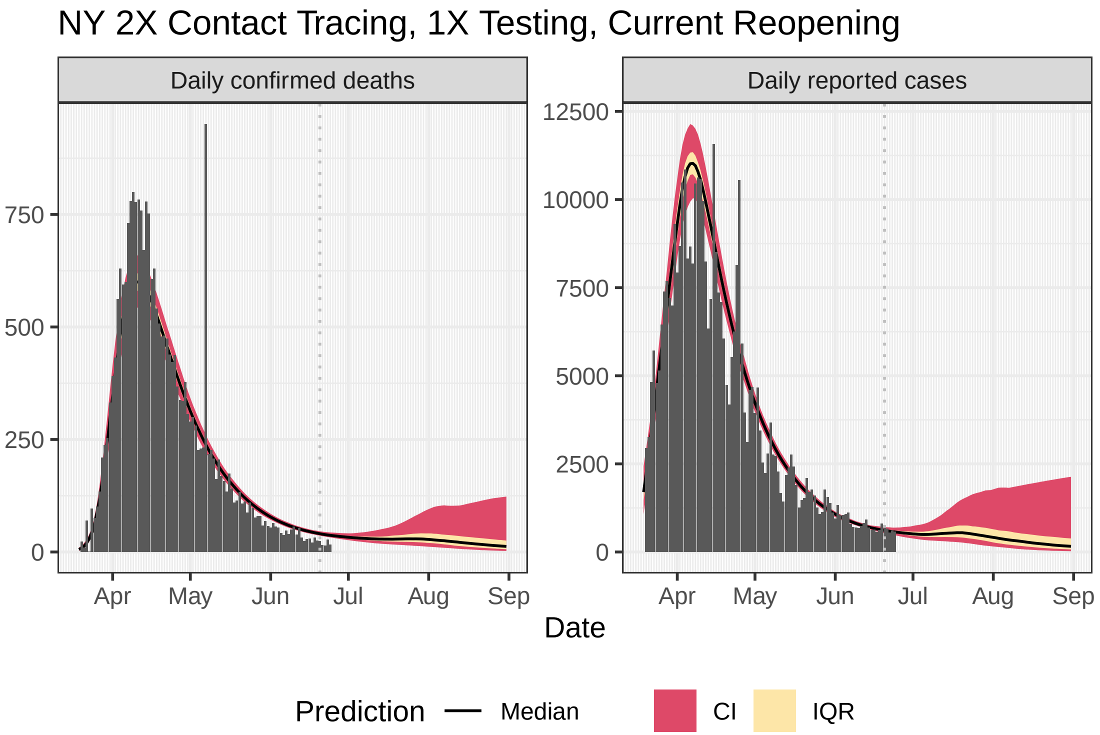
NY 2X Contact Tracing, 1X Testing, Current Reopening
Daily confirmed deaths
Daily reported cases
750
500
250
0
12500
10000
7500
5000
2500
0
Apr
May
Jun
Jul
Aug
Sep
Date
Prediction
Median
CI
IQR

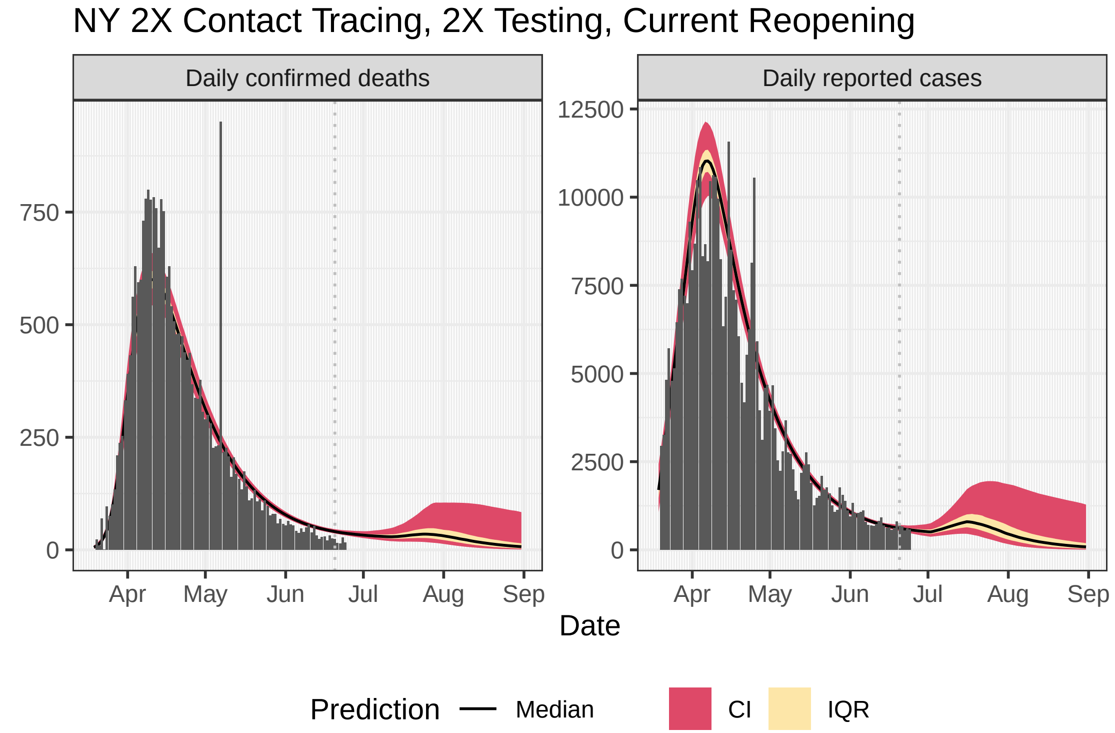
NY 2X Contact Tracing, 2X Testing, Current Reopening
Daily confirmed deaths
Daily reported cases
0
250
500
750
0
2500
5000
7500
10000
12500
Apr
May
Jun
Jul
Aug
Sep
Date
Prediction
Median
CI
IQR

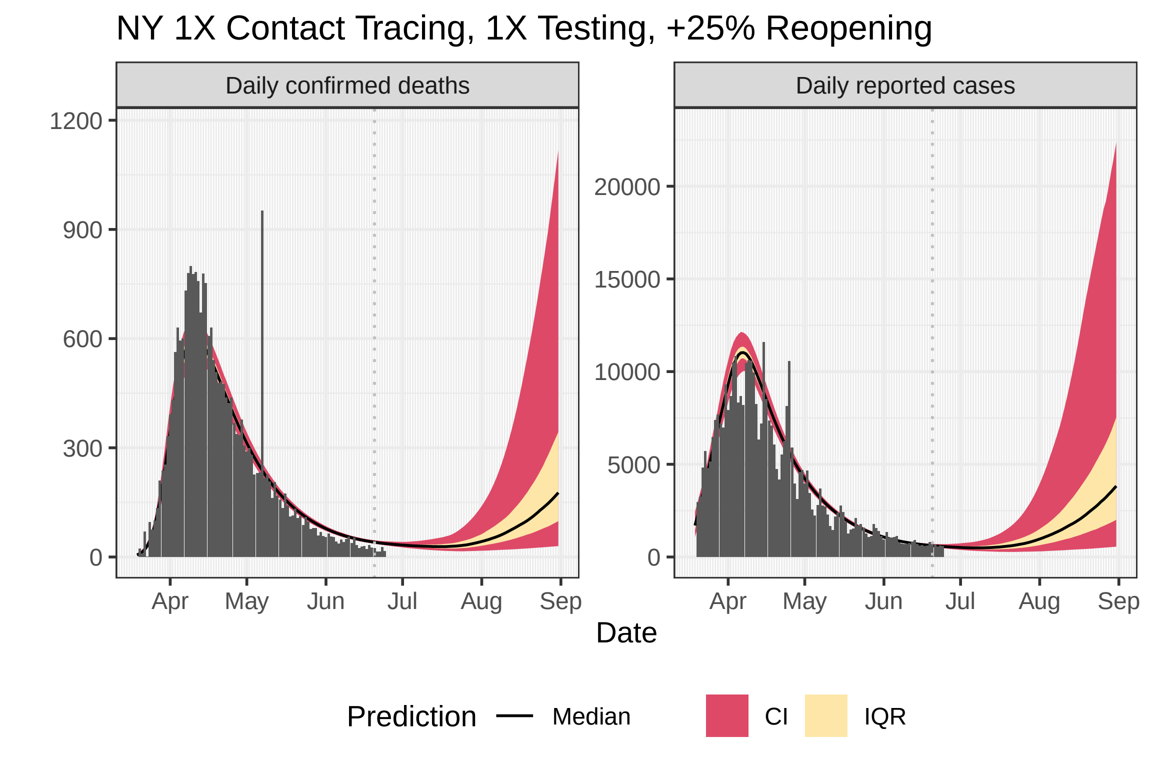

NY 1X Contact Tracing, 1X Testing, +25% Reopening
Daily confirmed deaths
Daily reported cases
1200
900
600
300
0
20000
15000
10000
5000
0
Apr
May
Jun
Jul
Aug
Sep
Date
Prediction
Median
CI
IQR

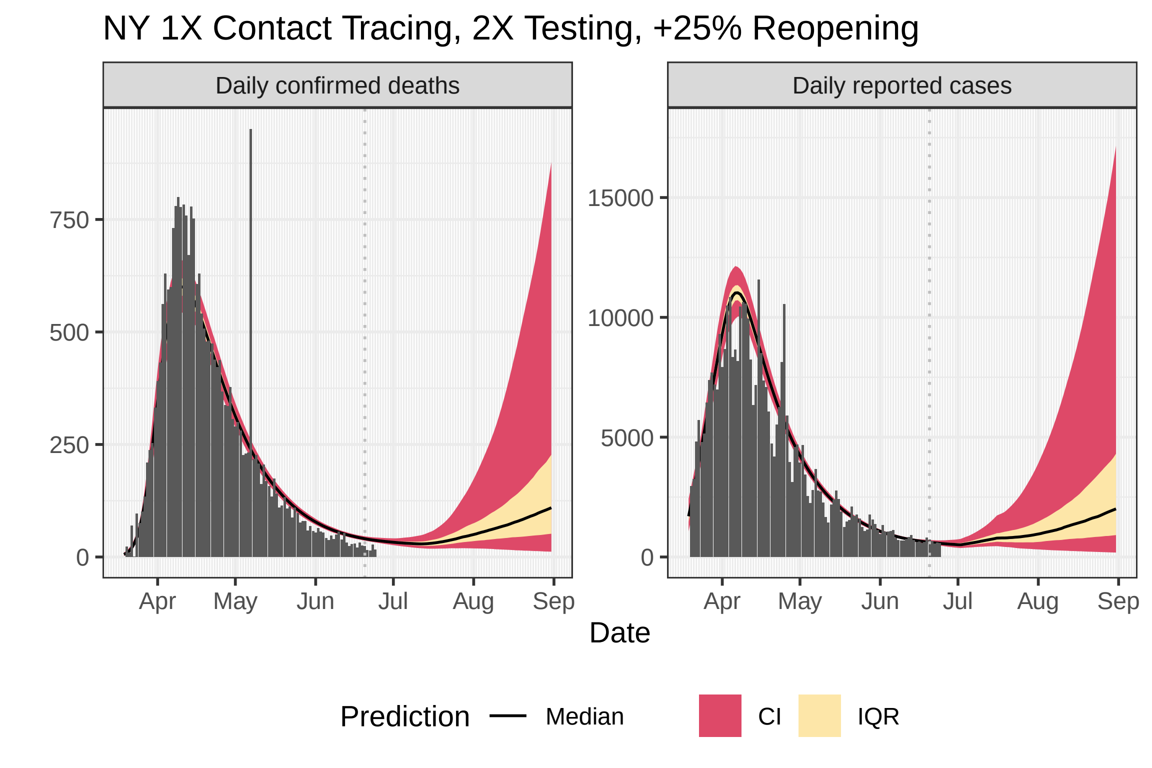
NY 1X Contact Tracing, 2X Testing, +25% Reopening
Daily confirmed deaths
Daily reported cases
750
500
250
0
15000
10000
5000
0
Apr
May
Jun
Jul
Aug
Sep
Date
Prediction
Median
CI
IQR

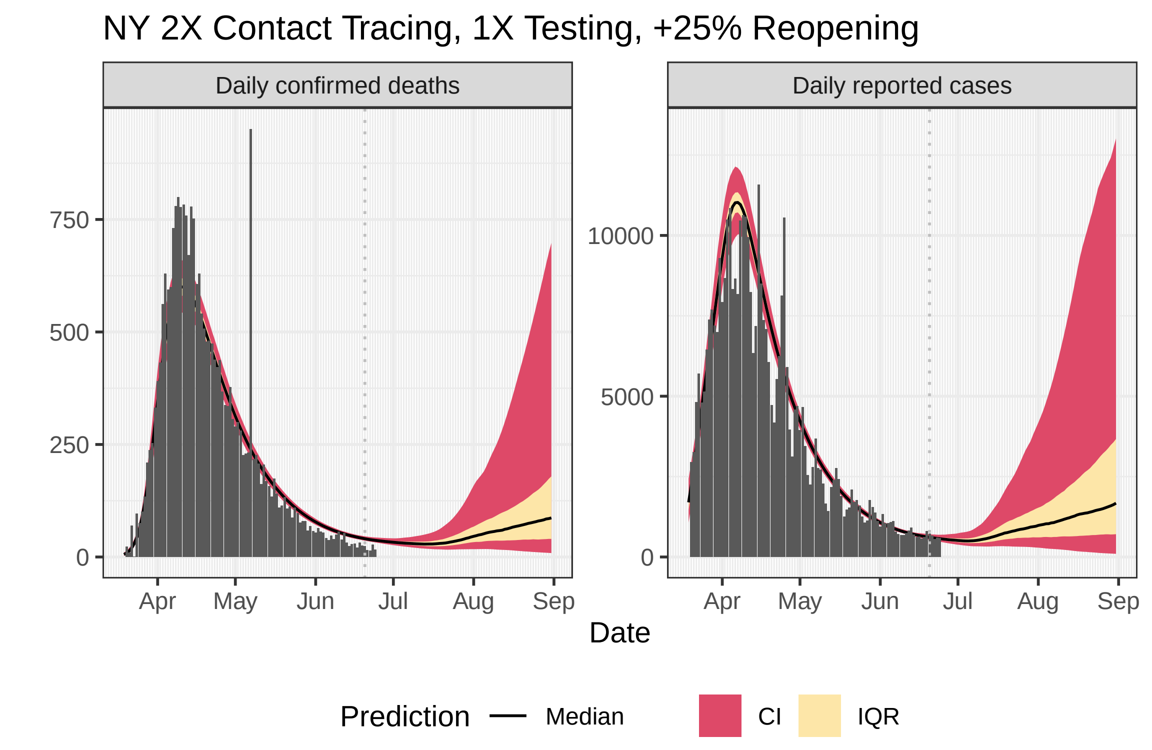
NY 2X Contact Tracing, 1X Testing, +25% Reopening
Daily confirmed deaths
Daily reported cases
750
500
250
0
10000
5000
0
Apr
May
Jun
Jul
Aug
Sep
Date
Prediction
Median
CI
IQR

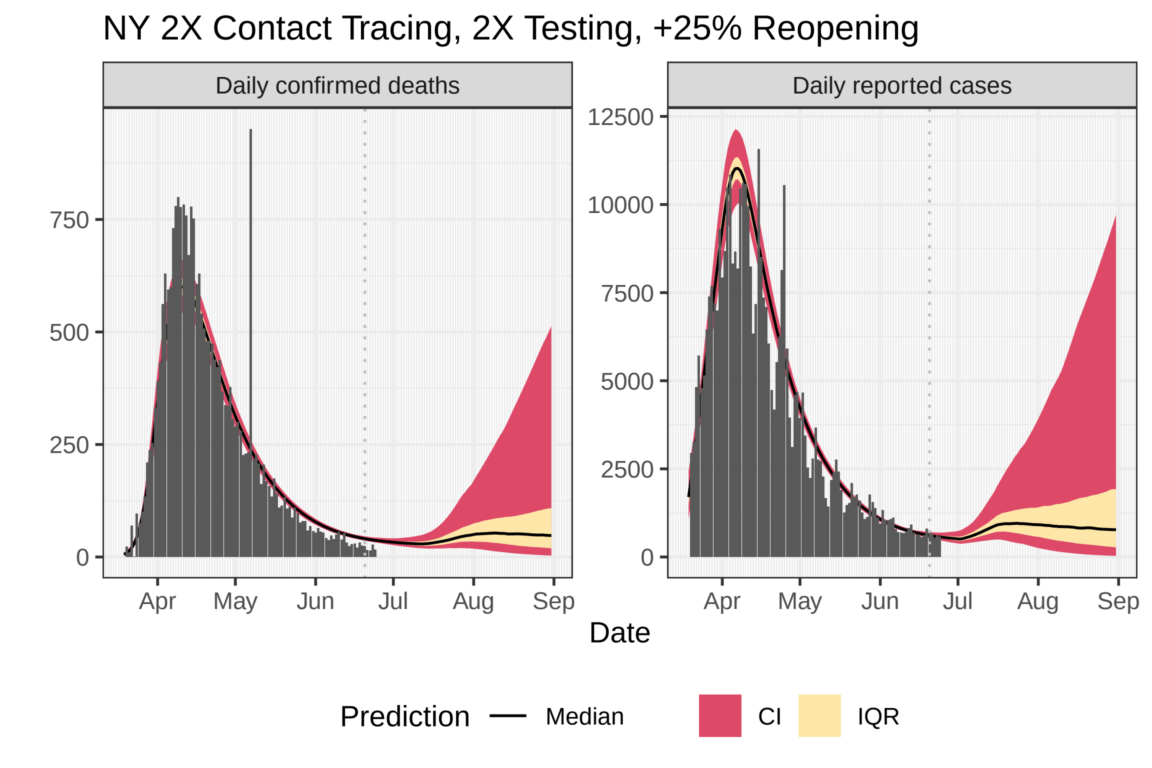
NY 2X Contact Tracing, 2X Testing, +25% Reopening
Daily confirmed deaths
Daily reported cases
0
250
500
750
0
2500
5000
7500
10000
12500
Apr
May
Jun
Jul
Aug
Sep
Date
Prediction
Median
CI
IQR

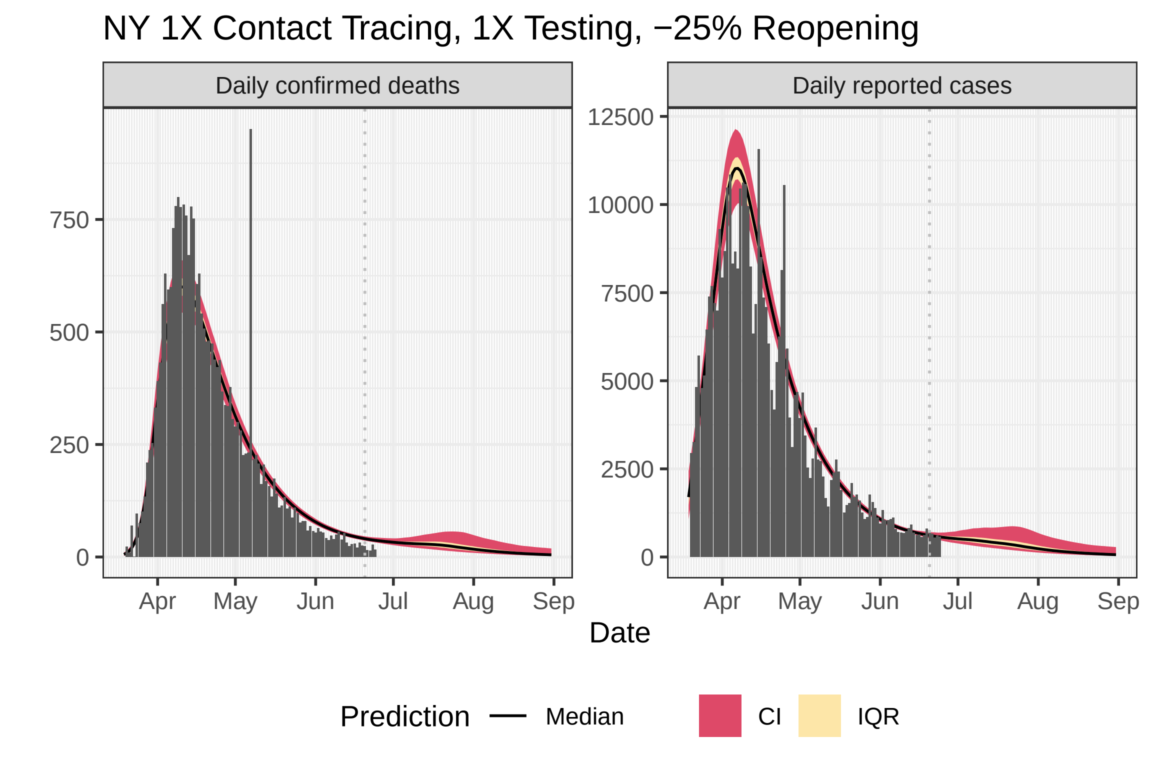

NY 1X Contact Tracing, 1X Testing, −25% Reopening
Daily confirmed deaths
Daily reported cases
750
500
250
0
12500
10000
7500
5000
2500
0
Apr
May
Jun
Jul
Aug
Sep
Date
Prediction
Median
CI
IQR

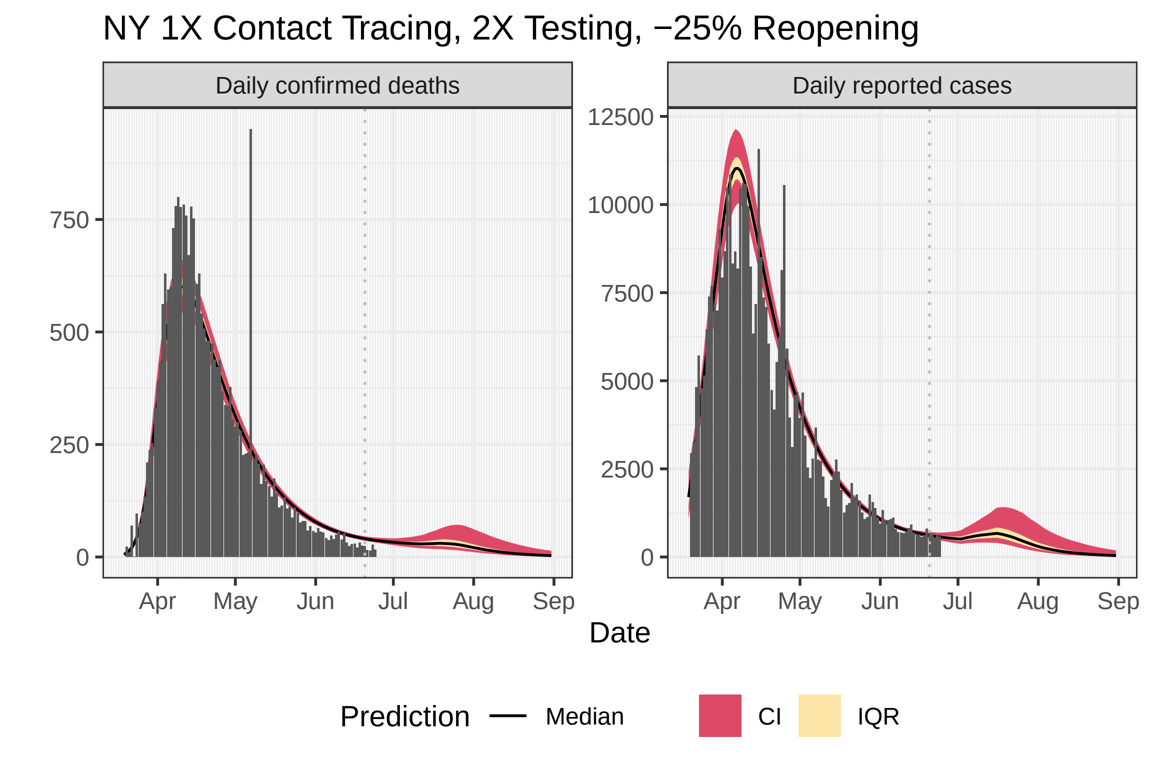
NY 1X Contact Tracing, 2X Testing, −25% Reopening
Daily confirmed deaths
Daily reported cases
0
250
500
750
0
2500
5000
7500
10000
12500
Apr
May
Jun
Jul
Aug
Sep
Date
Prediction
Median
CI
IQR

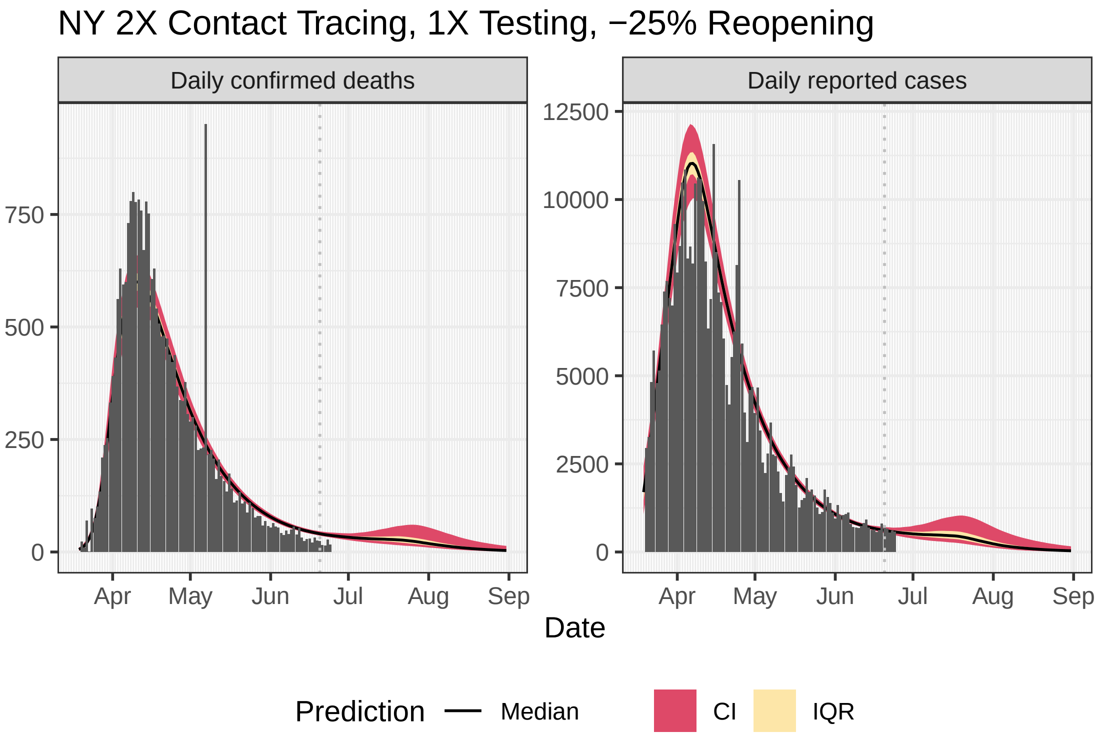
NY 2X Contact Tracing, 1X Testing, −25% Reopening
Daily confirmed deaths
Daily reported cases
0
250
500
750
0
2500
5000
7500
10000
12500
Apr
May
Jun
Jul
Aug
Sep
Date
Prediction
Median
CI
IQR

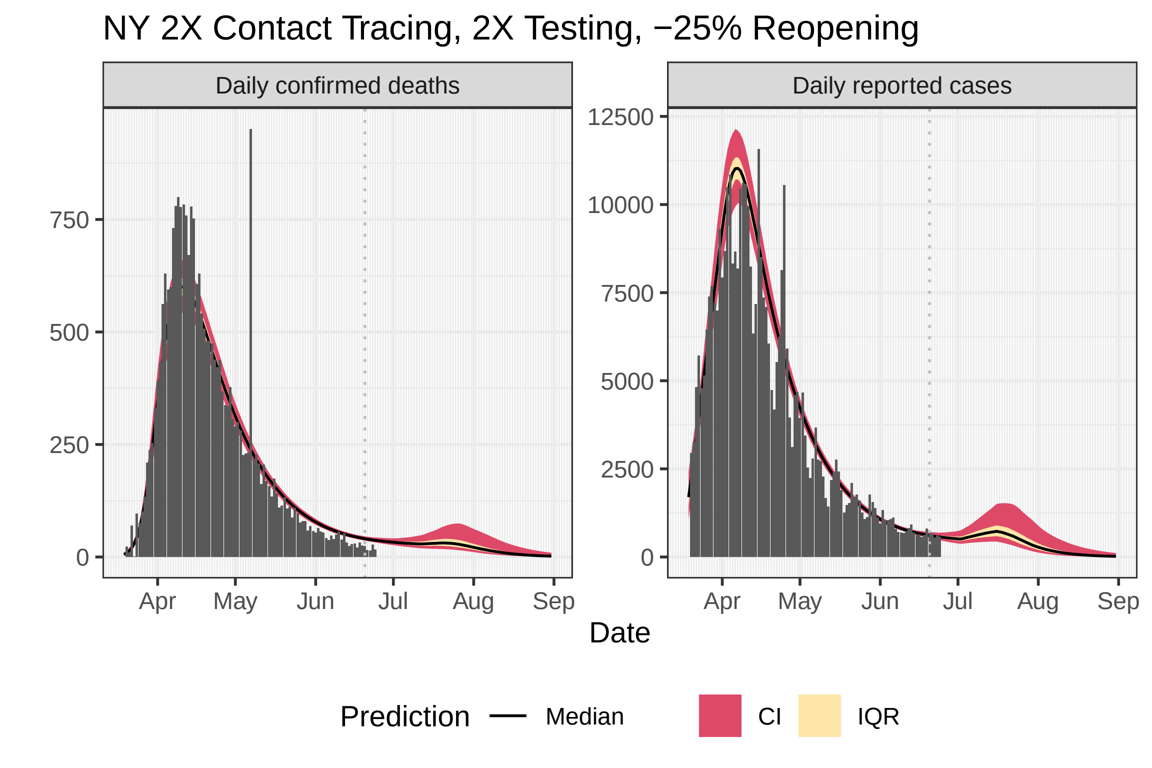

NY 2X Contact Tracing, 2X Testing, −25% Reopening
Daily confirmed deaths
Daily reported cases
750
500
250
0
12500
10000
7500
5000
2500
0
Apr
May
Jun
Jul
Aug
Sep
Date
Prediction
Median
CI
IQR

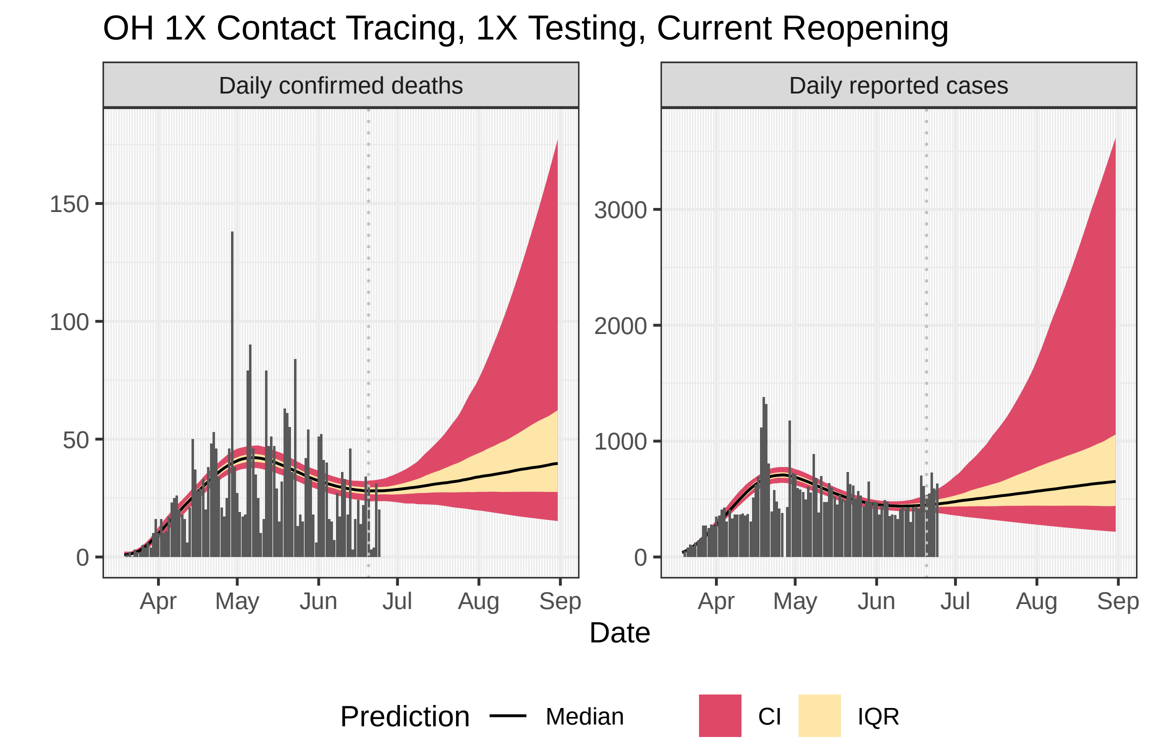
OH 1X Contact Tracing, 1X Testing, Current Reopening
Daily confirmed deaths
Daily reported cases
0
50
100
150
0
1000
2000
3000
Apr
May
Jun
Jul
Aug
Sep
Date
Prediction
Median
CI
IQR

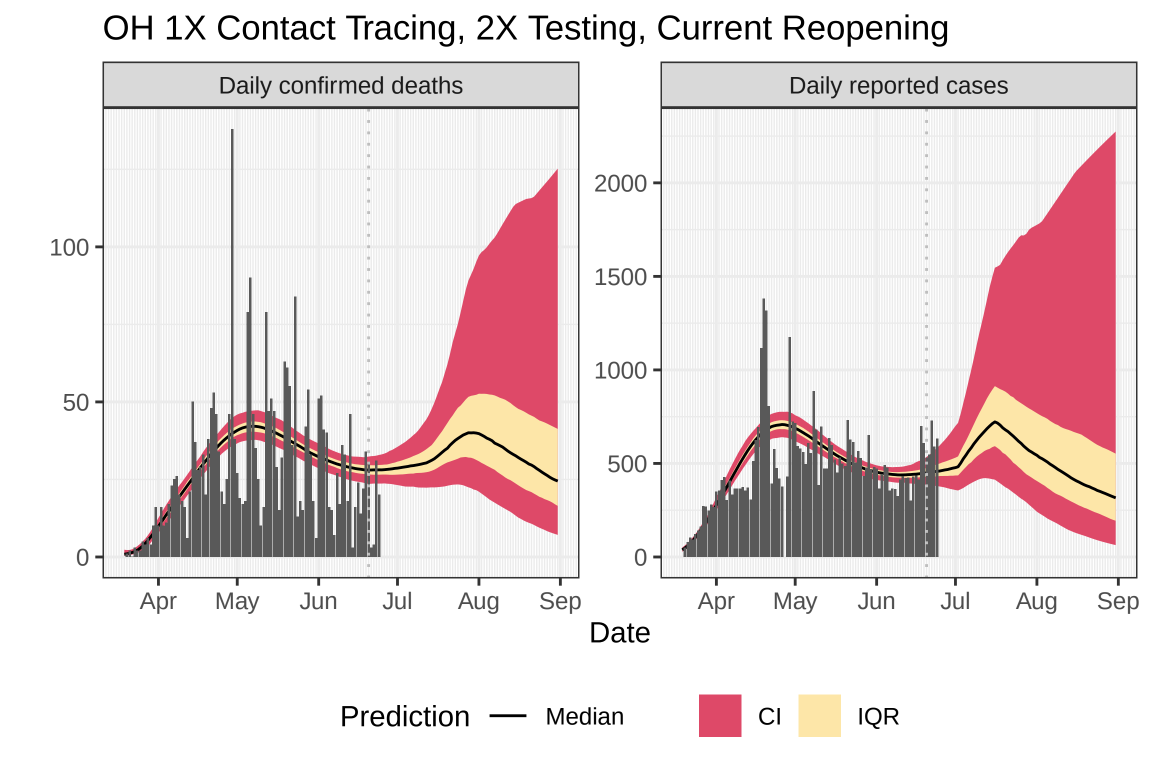
OH 1X Contact Tracing, 2X Testing, Current Reopening
Daily confirmed deaths
Daily reported cases
100
50
0
2000
1500
1000
500
0
Apr
May
Jun
Jul
Aug
Sep
Date
Prediction
Median
CI
IQR

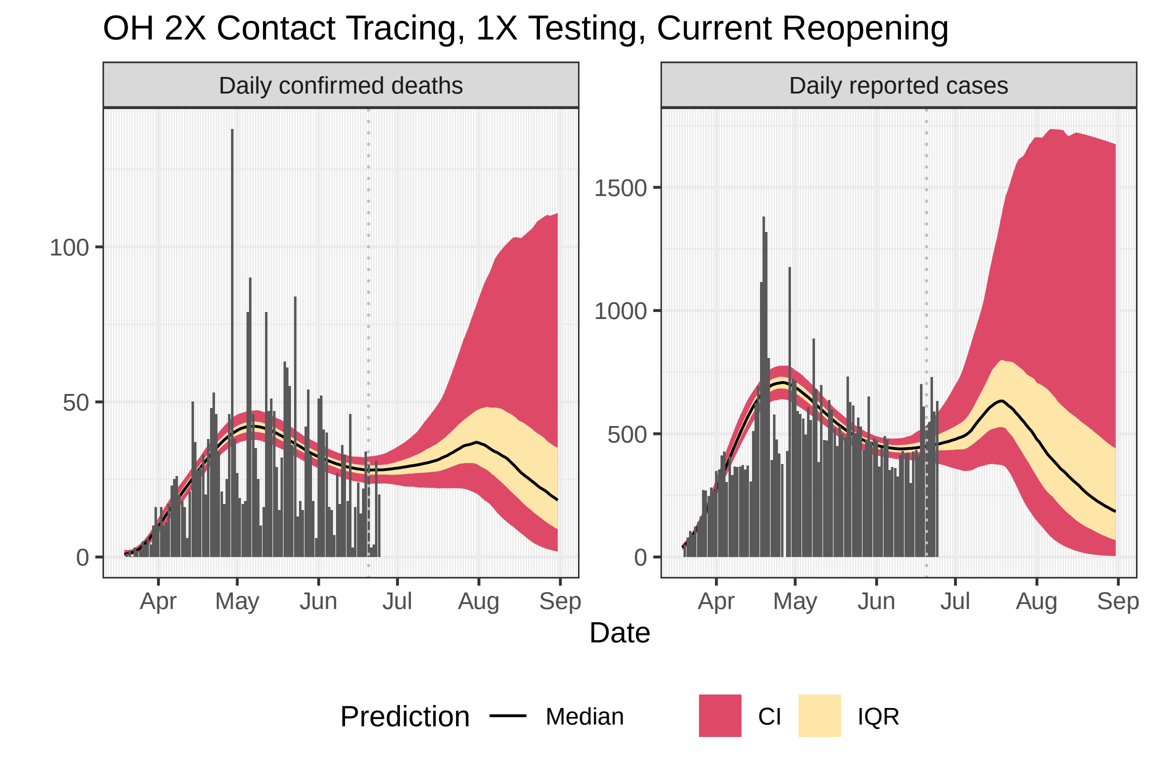
OH 2X Contact Tracing, 1X Testing, Current Reopening
Daily confirmed deaths
Daily reported cases
100
50
0
1500
1000
500
0
Apr
May
Jun
Jul
Aug
Sep
Date
Prediction
Median
CI
IQR

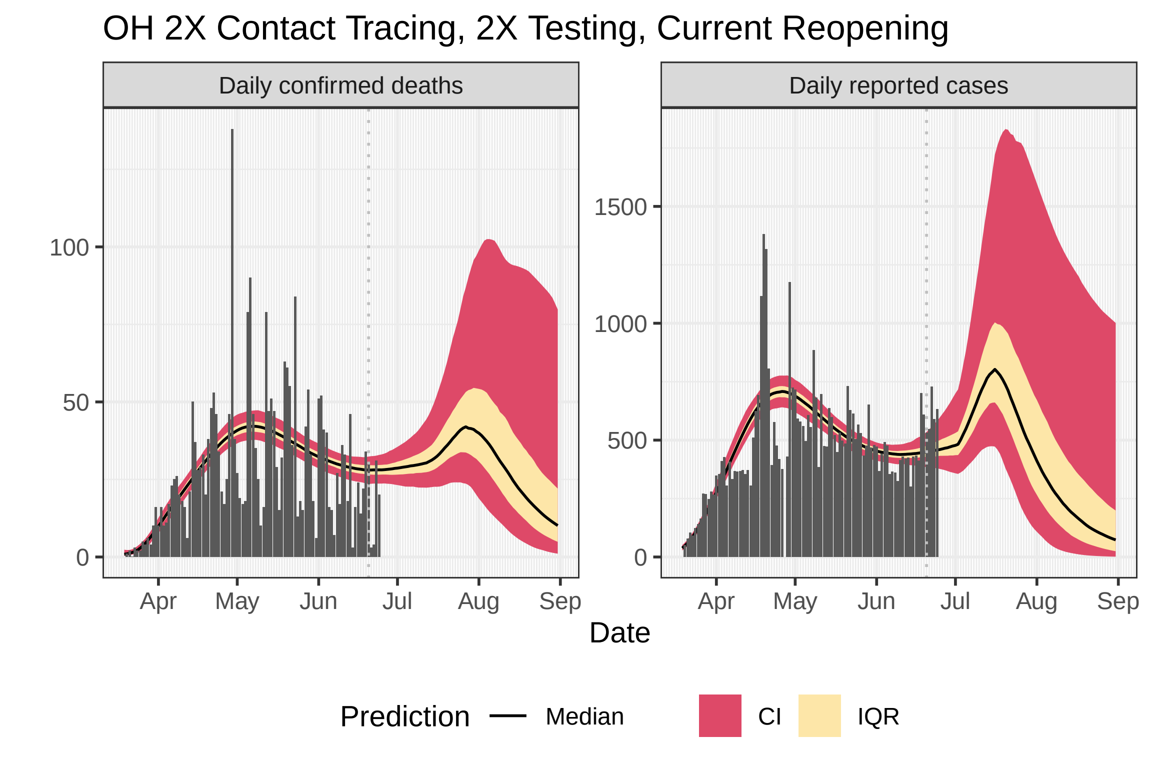
OH 2X Contact Tracing, 2X Testing, Current Reopening
Daily confirmed deaths
Daily reported cases
100
50
0
1500
1000
500
0
Apr
May
Jun
Jul
Aug
Sep
Date
Prediction
Median
CI
IQR

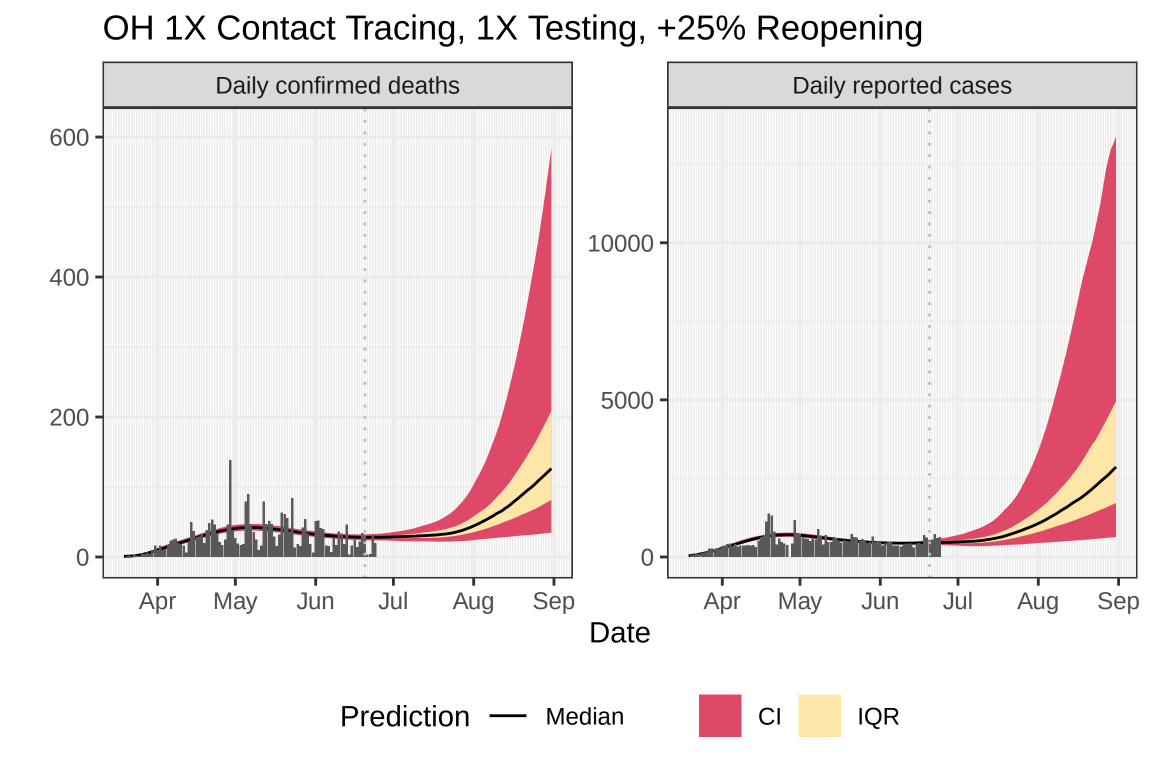

OH 1X Contact Tracing, 1X Testing, +25% Reopening
Daily confirmed deaths
Daily reported cases
600
400
200
0
10000
5000
0
Apr
May
Jun
Jul
Aug
Sep
Date
Prediction
Median
CI
IQR

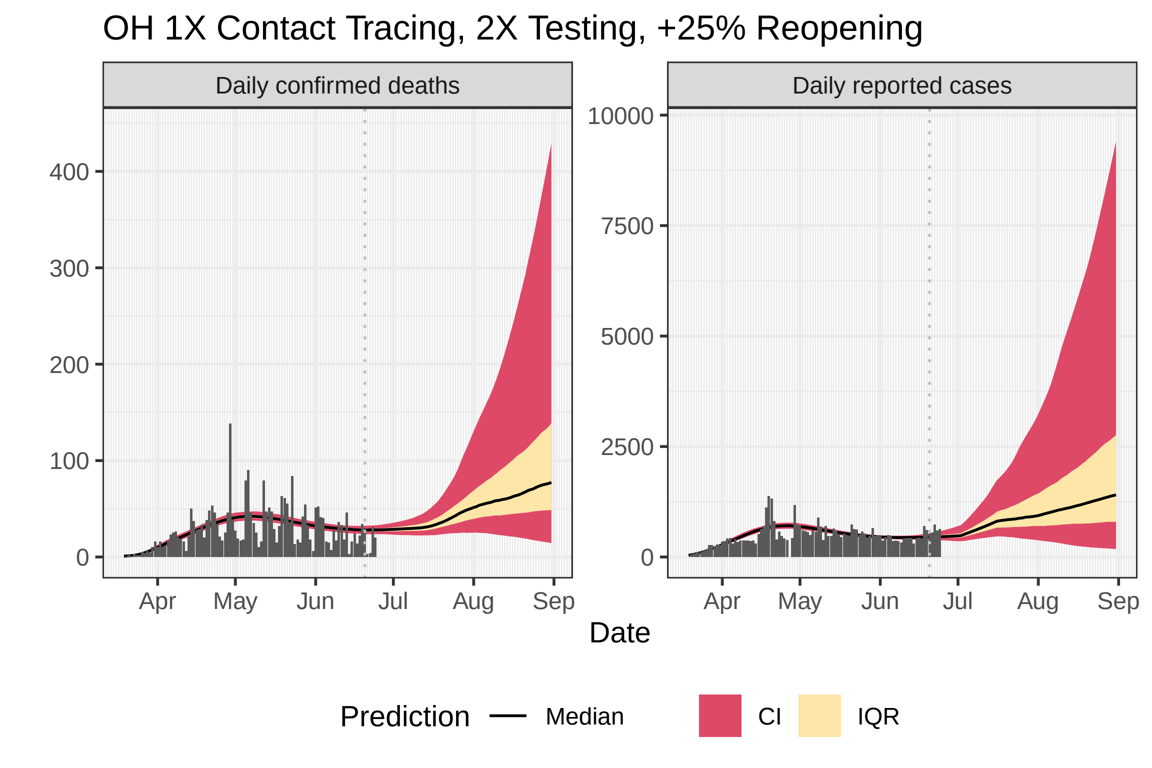
OH 1X Contact Tracing, 2X Testing, +25% Reopening
Daily confirmed deaths
Daily reported cases
0
100
200
300
400
0
2500
5000
7500
10000
Apr
May
Jun
Jul
Aug
Sep
Date
Prediction
Median
CI
IQR

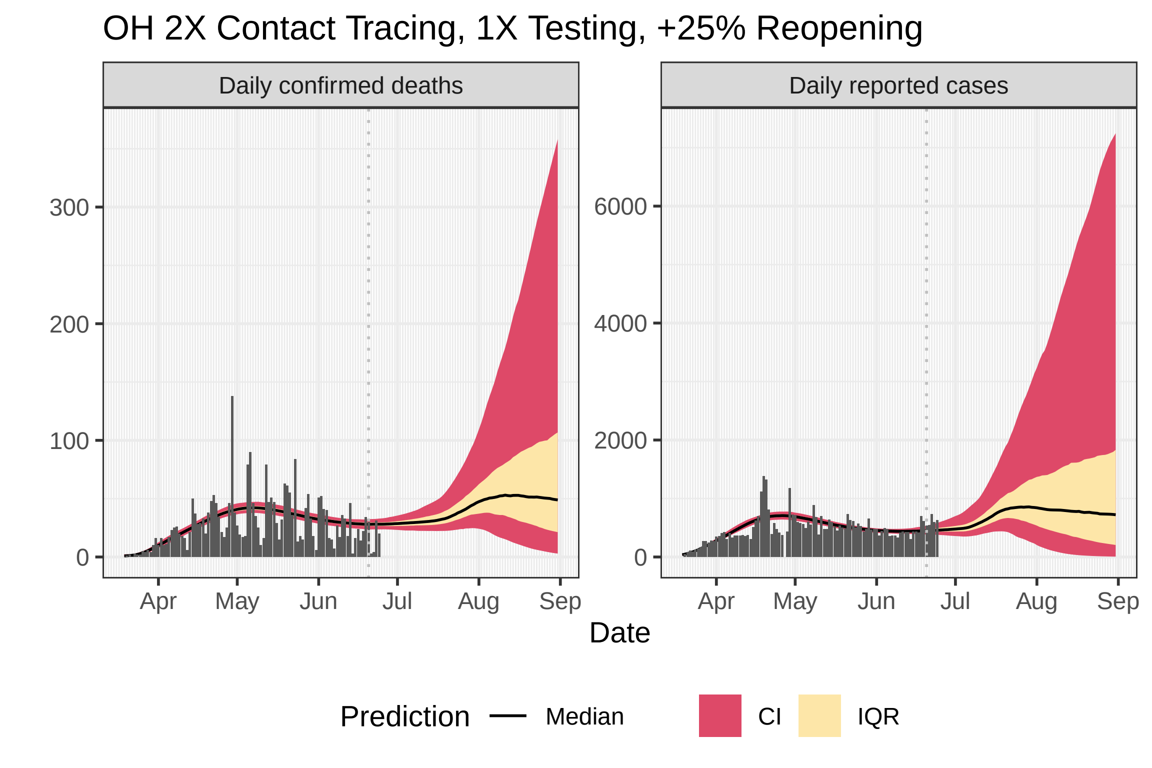

OH 2X Contact Tracing, 1X Testing, +25% Reopening
Daily confirmed deaths
Daily reported cases
300
200
100
0
6000
4000
2000
0
Apr
May
Jun
Jul
Aug
Sep
Date
Prediction
Median
CI
IQR

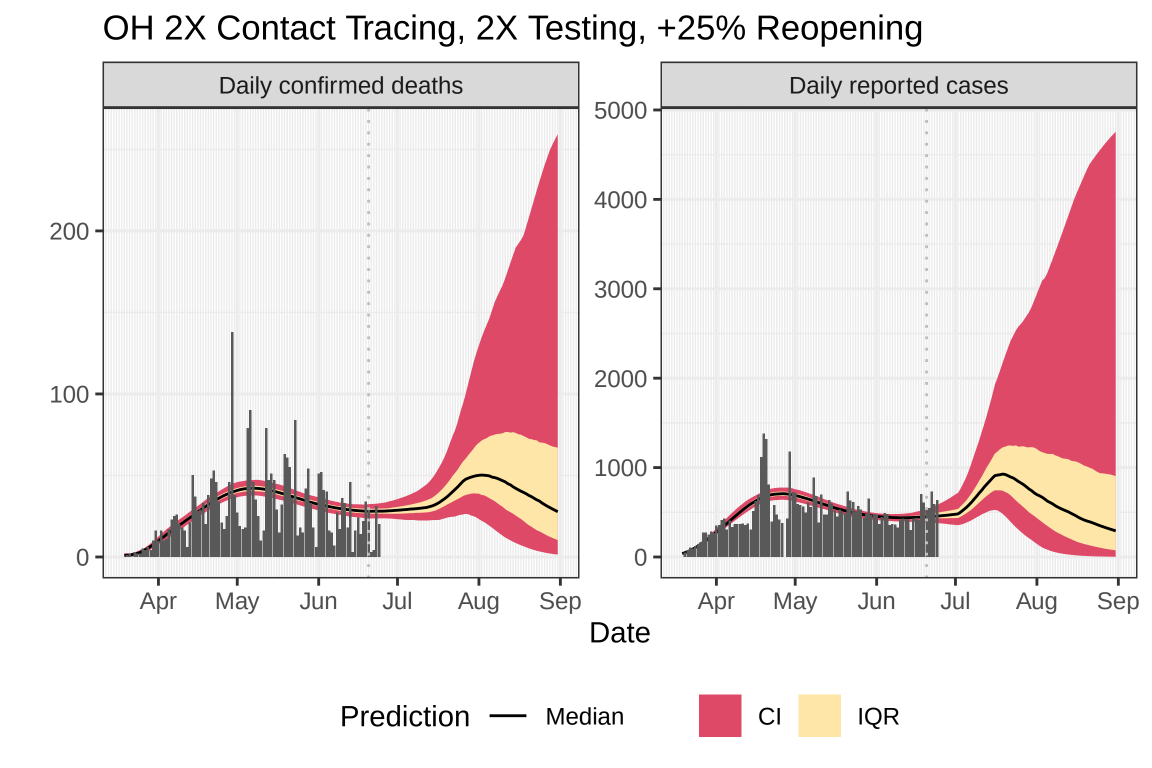

OH 2X Contact Tracing, 2X Testing, +25% Reopening
Daily confirmed deaths
Daily reported cases
0
100
200
0
1000
2000
3000
4000
5000
Apr
May
Jun
Jul
Aug
Sep
Date
Prediction
Median
CI
IQR

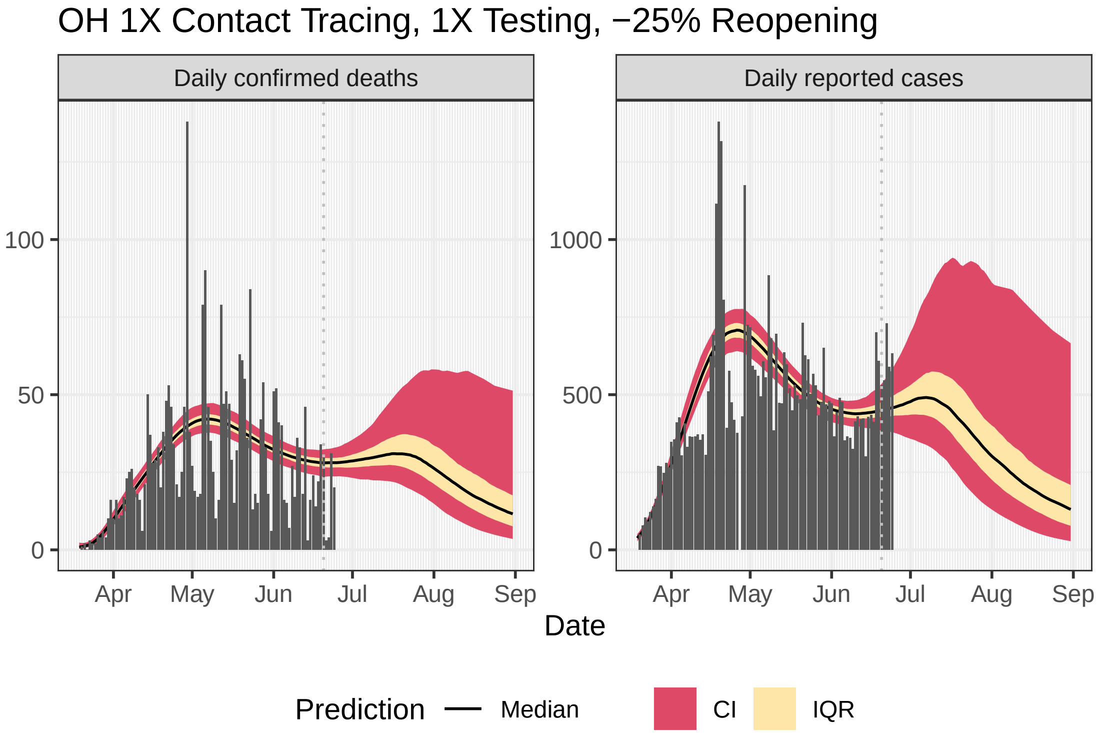

OH 1X Contact Tracing, 1X Testing, −25% Reopening
Daily confirmed deaths
Daily reported cases
100
50
0
1000
500
0
Apr
May
Jun
Jul
Aug
Sep
Date
Prediction
Median
CI
IQR

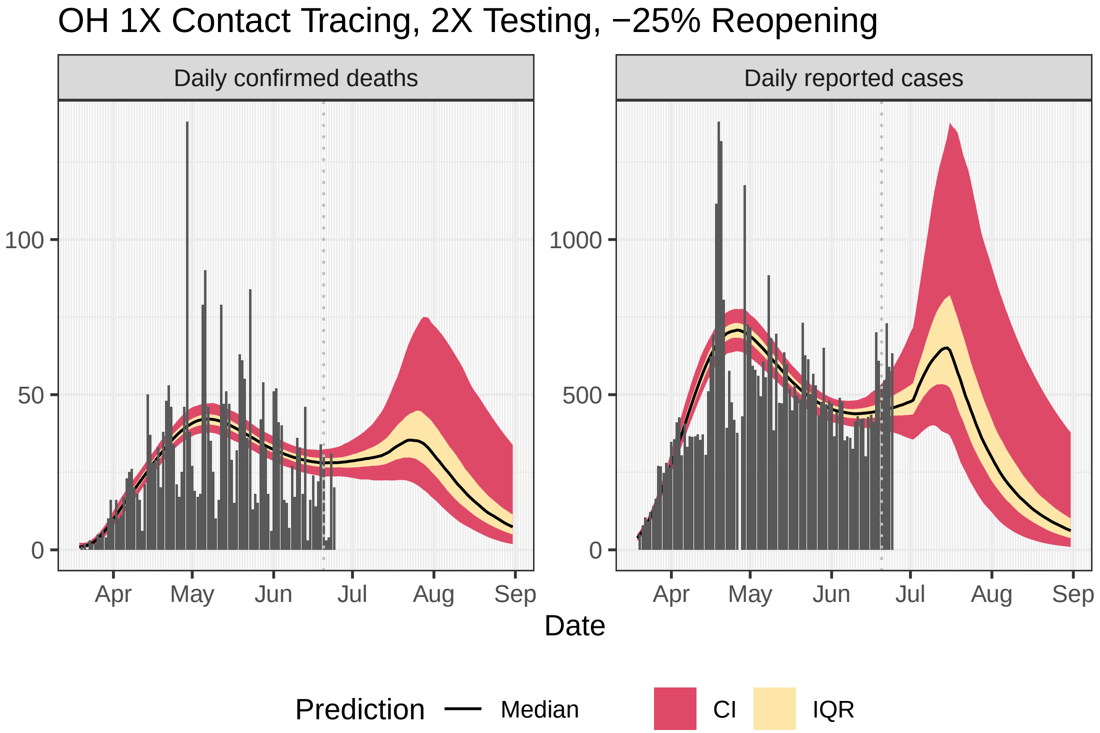

OH 1X Contact Tracing, 2X Testing, −25% Reopening
Daily confirmed deaths
Daily reported cases
100
50
0
1000
500
0
Apr
May
Jun
Jul
Aug
Sep
Date
Prediction
Median
CI
IQR

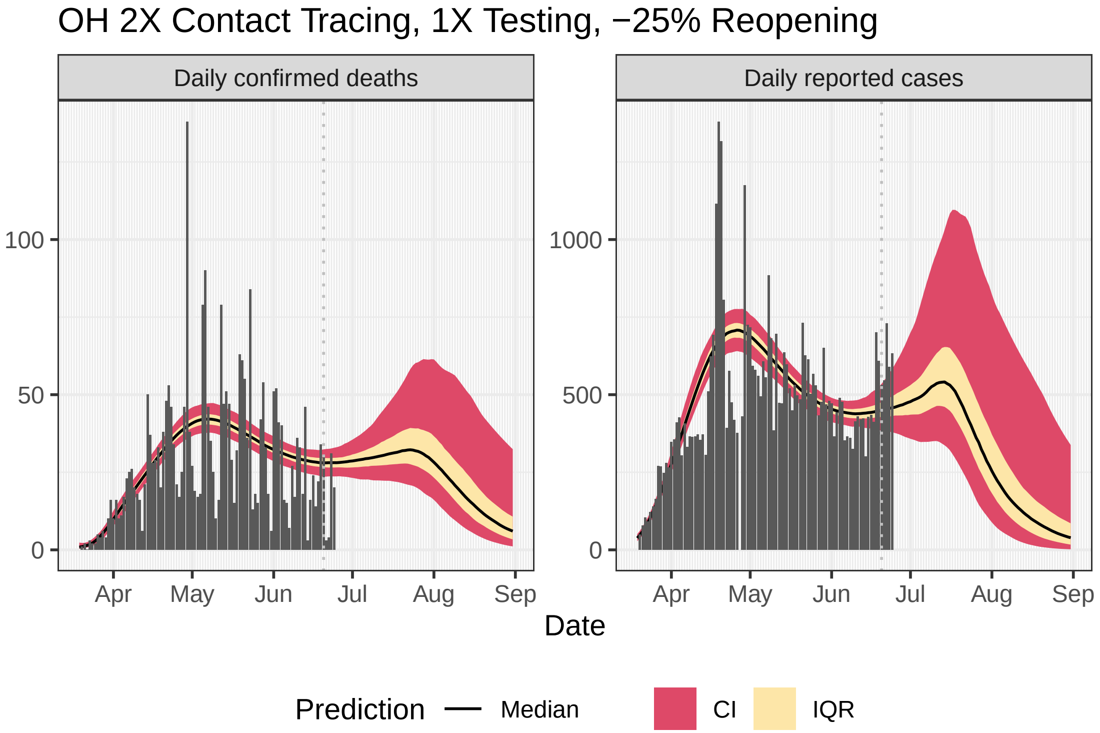
OH 2X Contact Tracing, 1X Testing, −25% Reopening
Daily confirmed deaths
Daily reported cases
100
50
0
1000
500
0
Apr
May
Jun
Jul
Aug
Sep
Date
Prediction
Median
CI
IQR

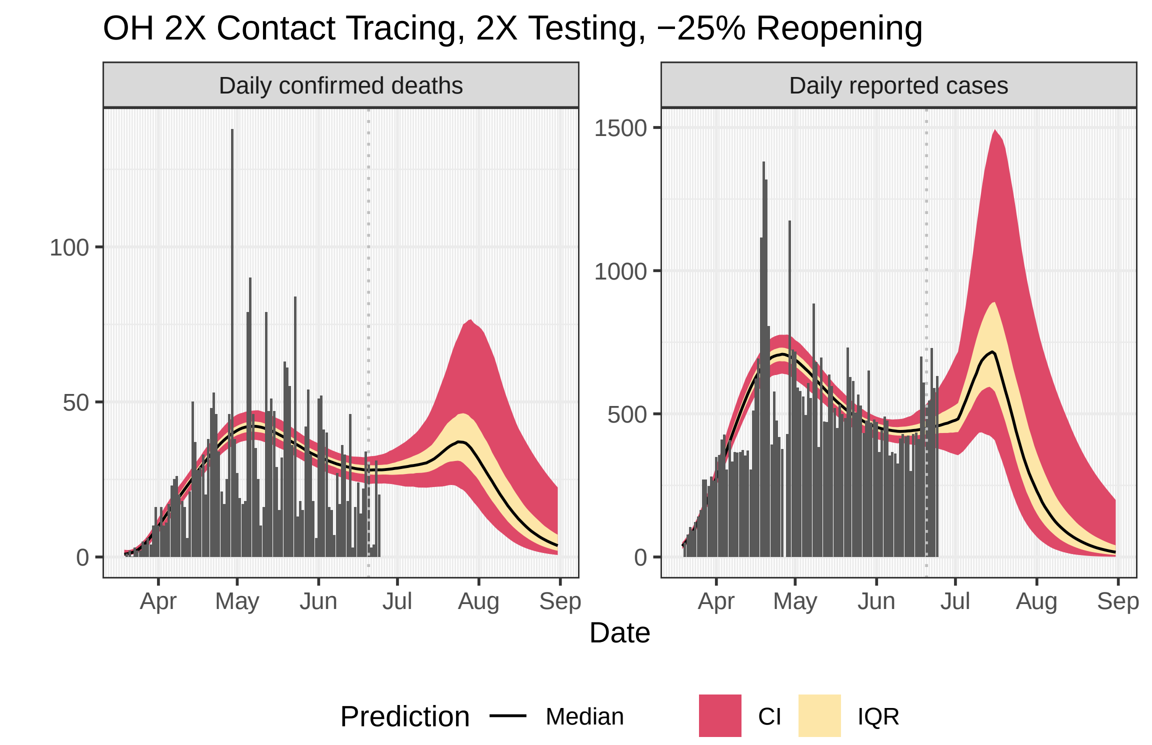

OH 2X Contact Tracing, 2X Testing, −25% Reopening
Daily confirmed deaths
Daily reported cases
100
50
0
1500
1000
500
0
Apr
May
Jun
Jul
Aug
Sep
Date
Prediction
Median
CI
IQR

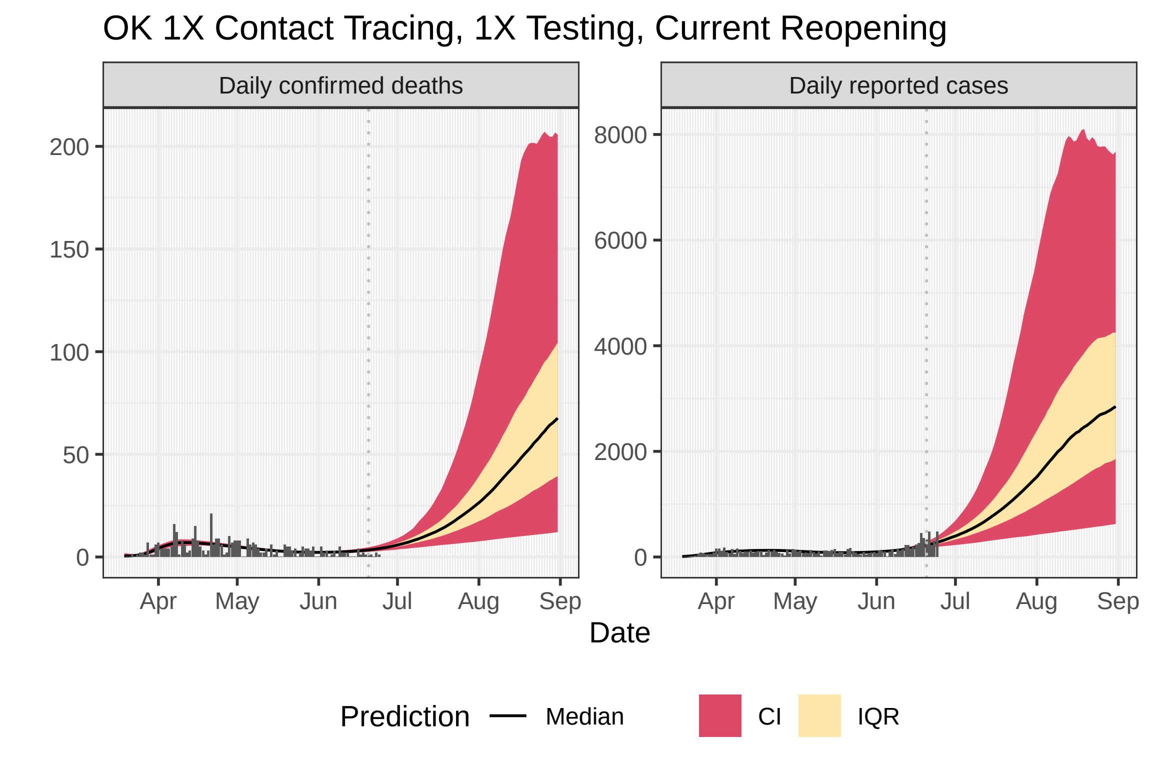
OK 1X Contact Tracing, 1X Testing, Current Reopening
Daily confirmed deaths
Daily reported cases
200
150
100
50
0
8000
6000
4000
2000
0
Apr
May
Jun
Jul
Aug
Sep
Date
Prediction
Median
CI
IQR

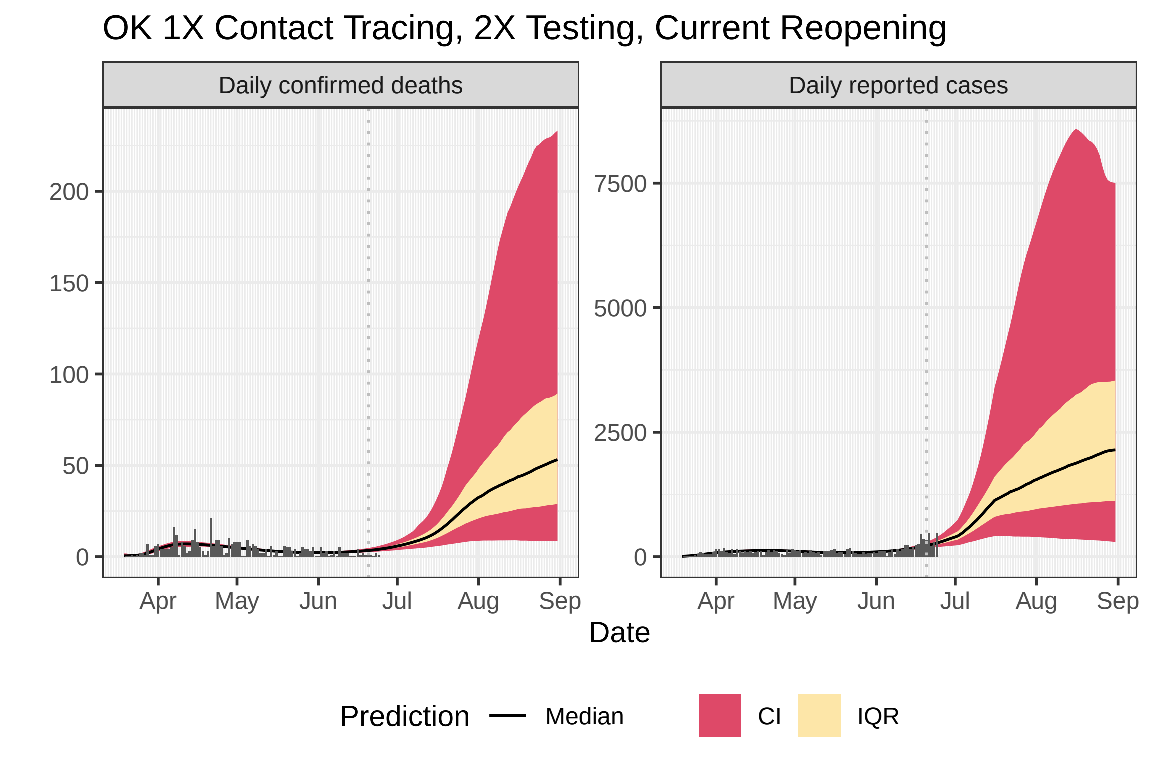

OK 1X Contact Tracing, 2X Testing, Current Reopening
Daily confirmed deaths
Daily reported cases
0
50
100
150
200
0
2500
5000
7500
Apr
May
Jun
Jul
Aug
Sep
Date
Prediction
Median
CI
IQR

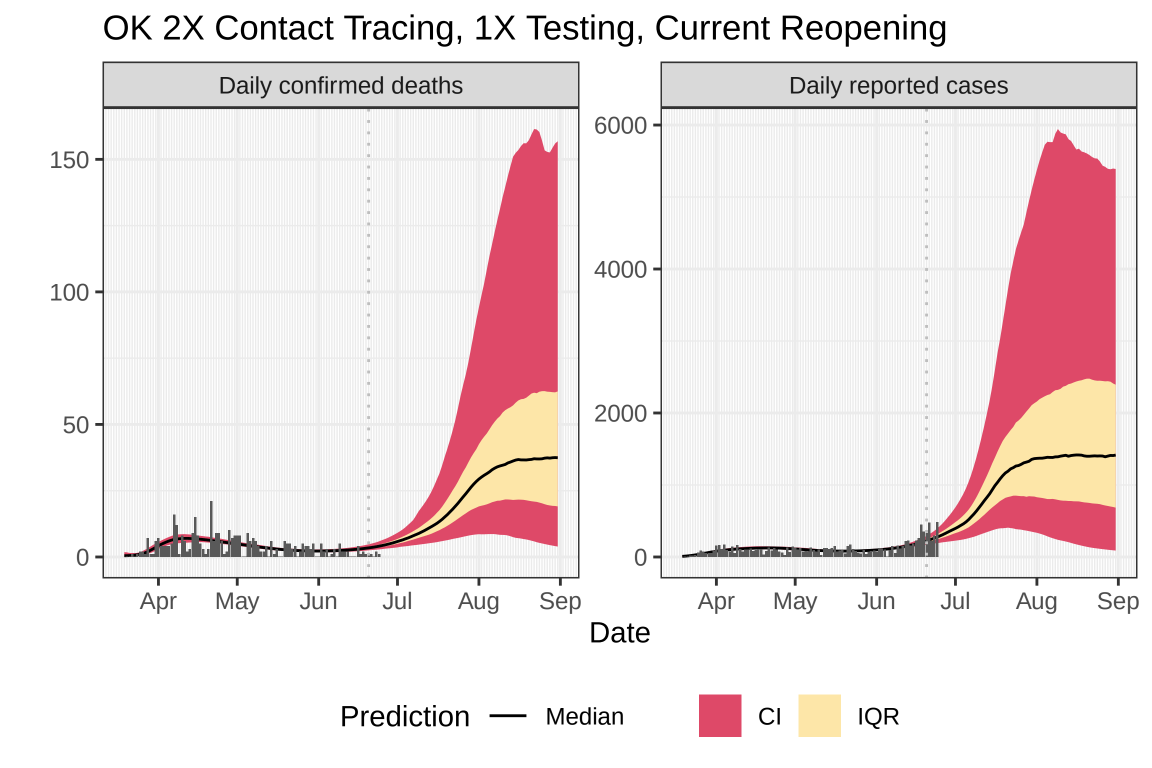

OK 2X Contact Tracing, 1X Testing, Current Reopening
Daily confirmed deaths
Daily reported cases
150
100
50
0
6000
4000
2000
0
Apr
May
Jun
Jul
Aug
Sep
Date
Prediction
Median
CI
IQR

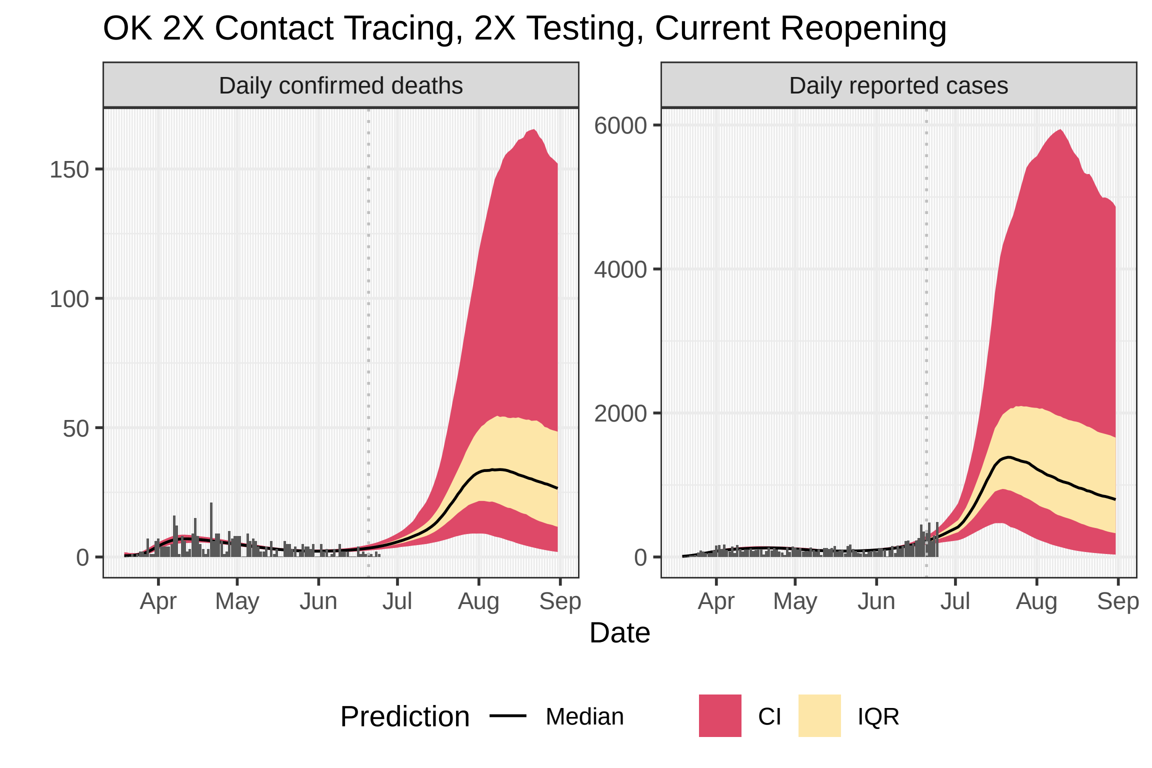
OK 2X Contact Tracing, 2X Testing, Current Reopening
Daily confirmed deaths
Daily reported cases
0
50
100
150
0
2000
4000
6000
Apr
May
Jun
Jul
Aug
Sep
Date
Prediction
Median
CI
IQR

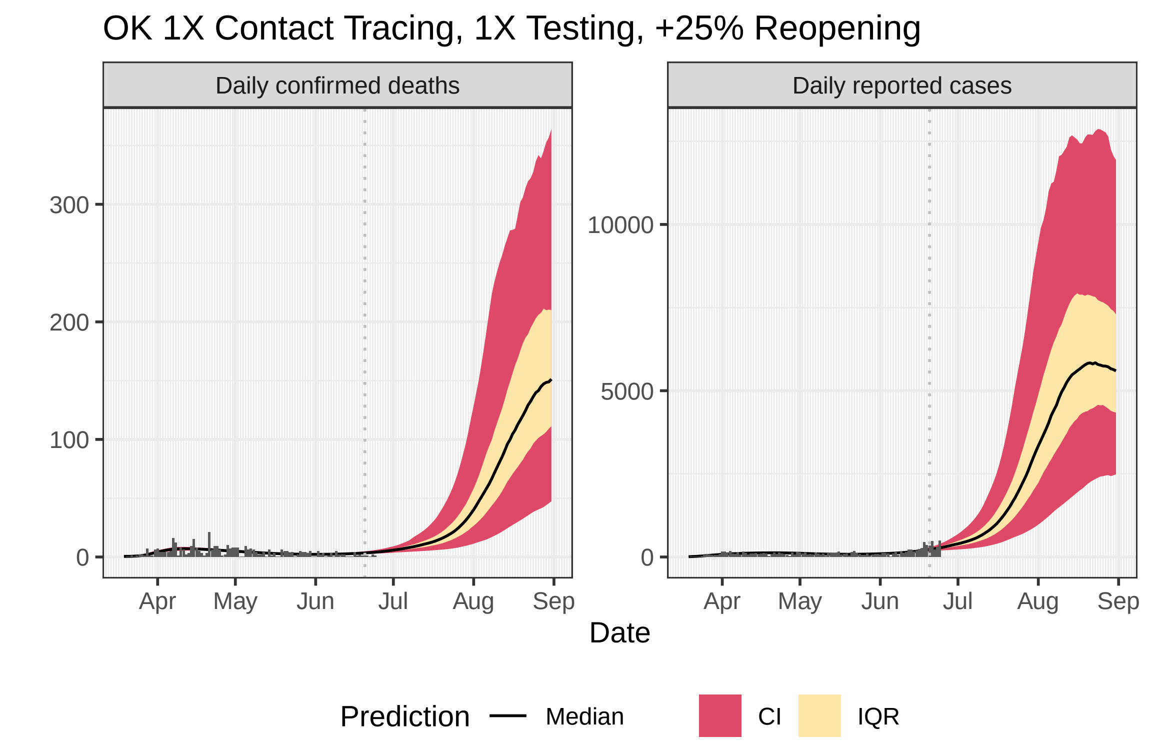
OK 1X Contact Tracing, 1X Testing, +25% Reopening
Daily confirmed deaths
Daily reported cases
0
100
200
300
0
5000
10000
Apr
May
Jun
Jul
Aug
Sep
Date
Prediction
Median
CI
IQR

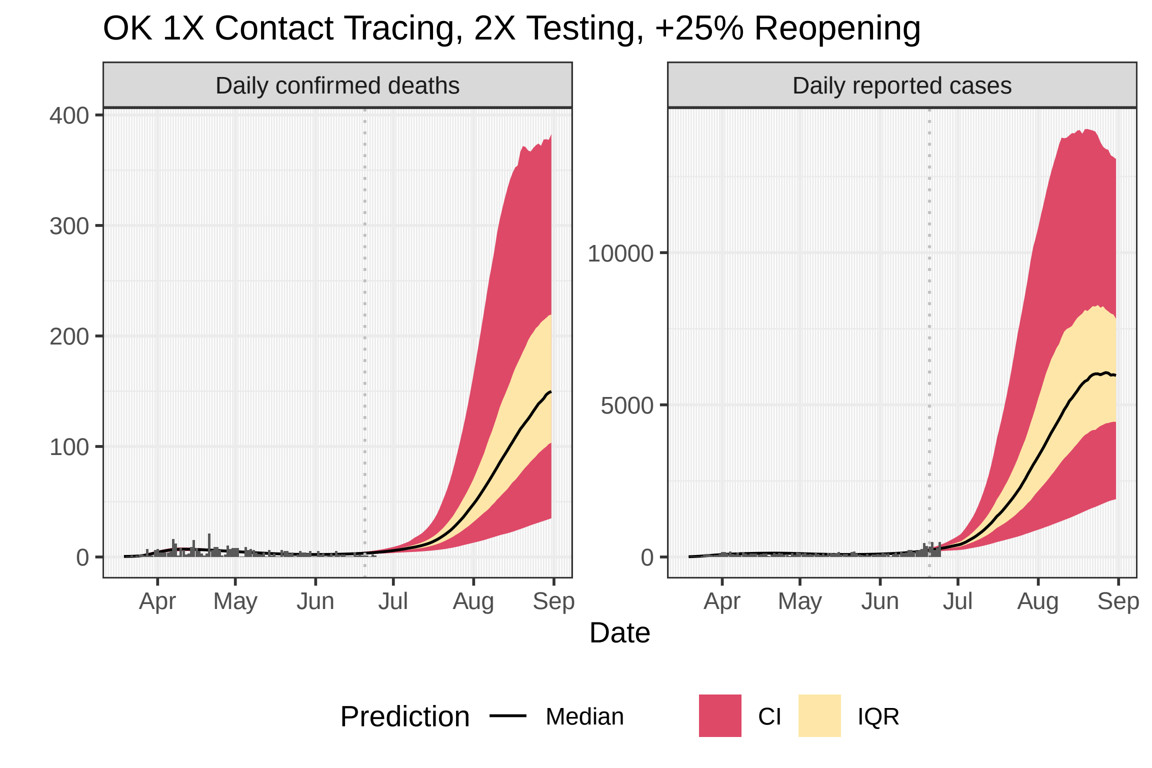

OK 1X Contact Tracing, 2X Testing, +25% Reopening
Daily confirmed deaths
Daily reported cases
400
300
200
100
0
10000
5000
0
Apr
May
Jun
Jul
Aug
Sep
Date
Prediction
Median
CI
IQR

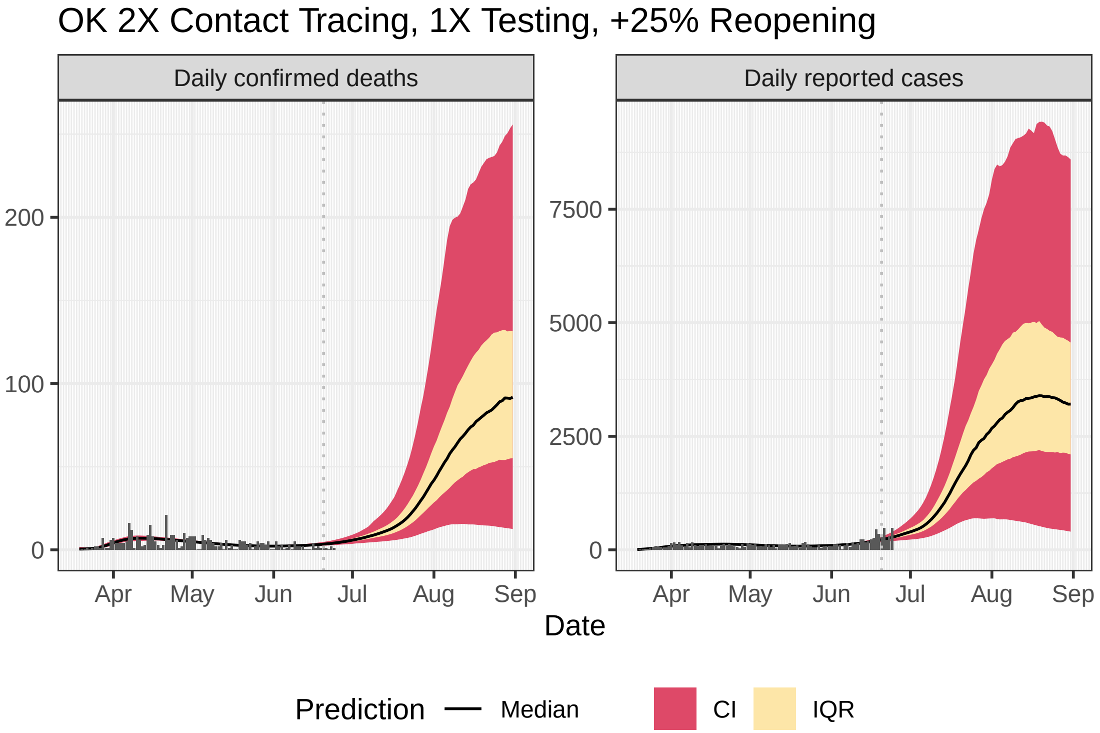

OK 2X Contact Tracing, 1X Testing, +25% Reopening
Daily confirmed deaths
Daily reported cases
200
100
0
7500
5000
2500
0
Apr
May
Jun
Jul
Aug
Sep
Date
Prediction
Median
CI
IQR

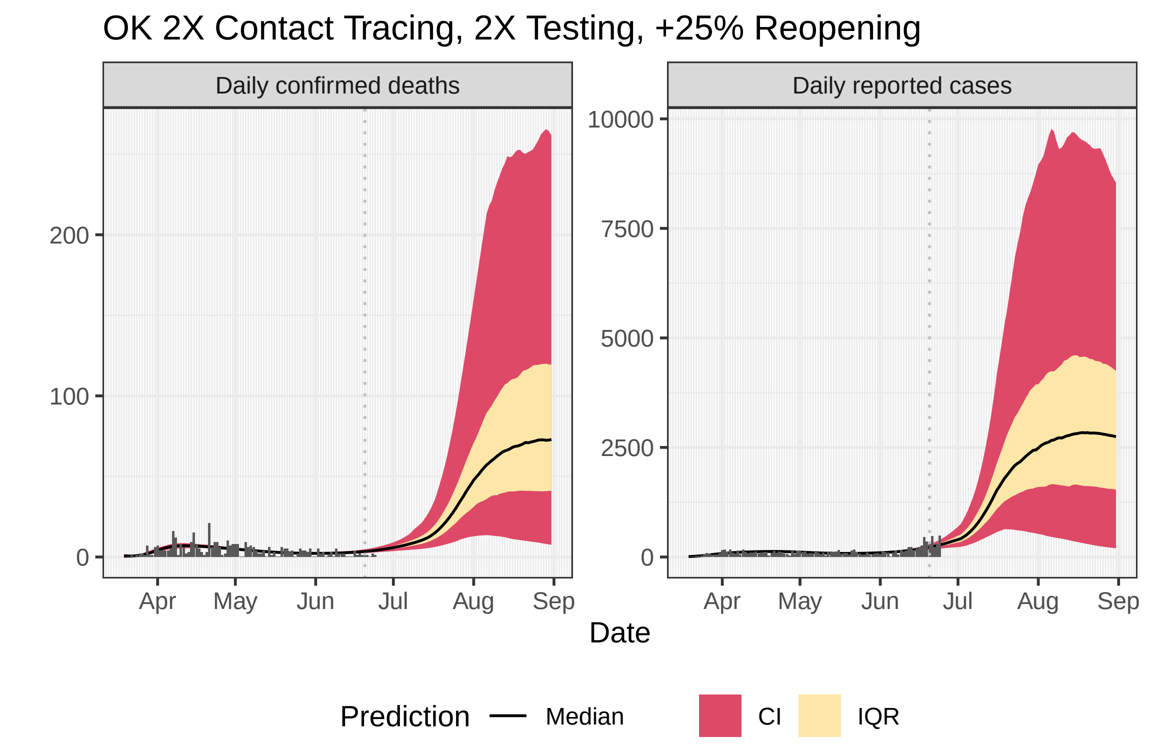

OK 2X Contact Tracing, 2X Testing, +25% Reopening
Daily confirmed deaths
Daily reported cases
0
100
200
0
2500
5000
7500
10000
Apr
May
Jun
Jul
Aug
Sep
Date
Prediction
Median
CI
IQR

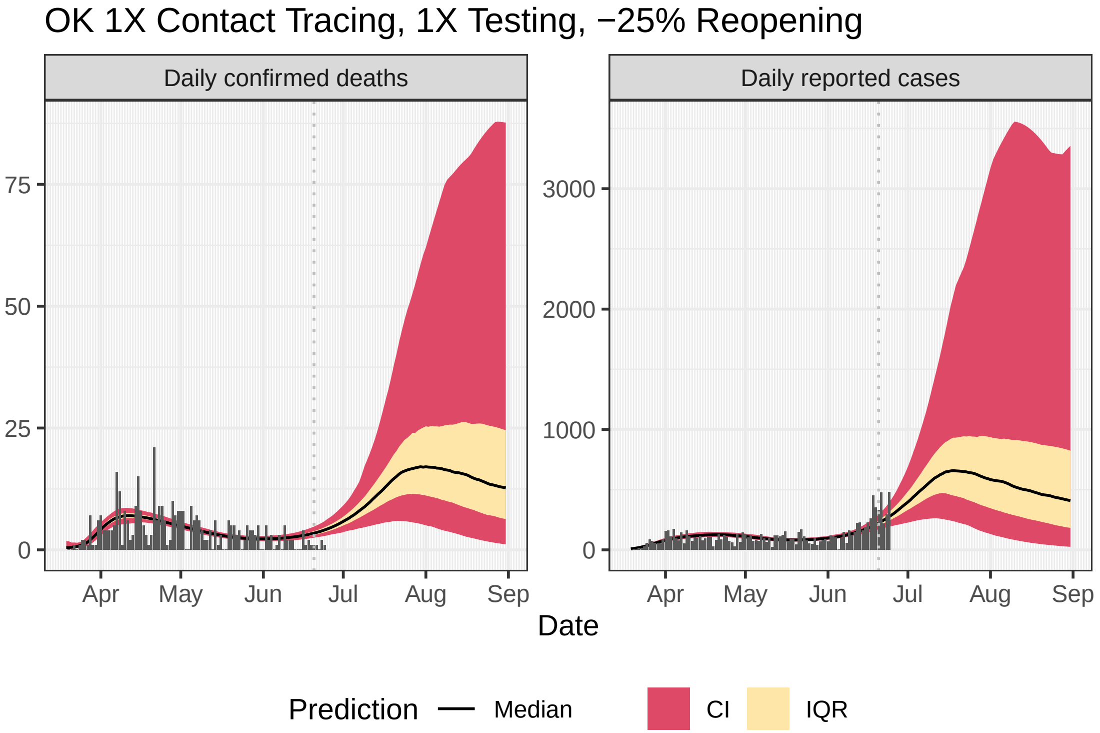

OK 1X Contact Tracing, 1X Testing, −25% Reopening
Daily confirmed deaths
Daily reported cases
0
25
50
75
0
1000
2000
3000
Apr
May
Jun
Jul
Aug
Sep
Date
Prediction
Median
CI
IQR

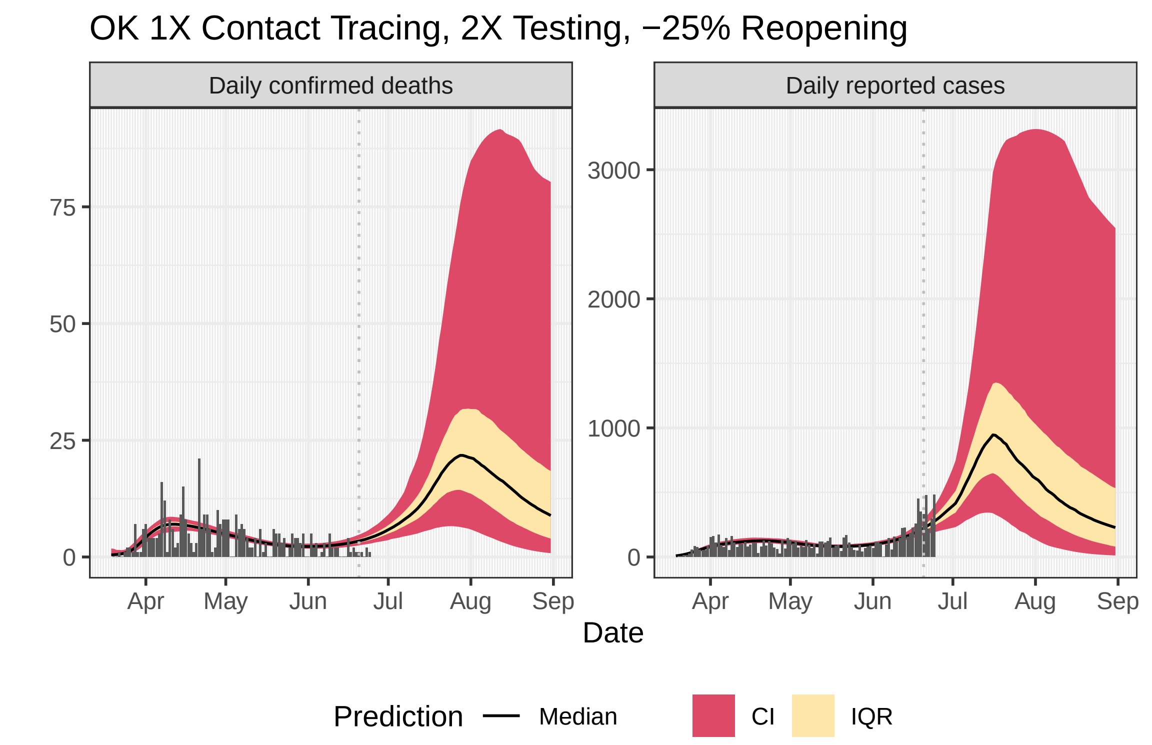

OK 1X Contact Tracing, 2X Testing, −25% Reopening
Daily confirmed deaths
Daily reported cases
75
50
25
0
3000
2000
1000
0
Apr
May
Jun
Jul
Aug
Sep
Date
Prediction
Median
CI
IQR

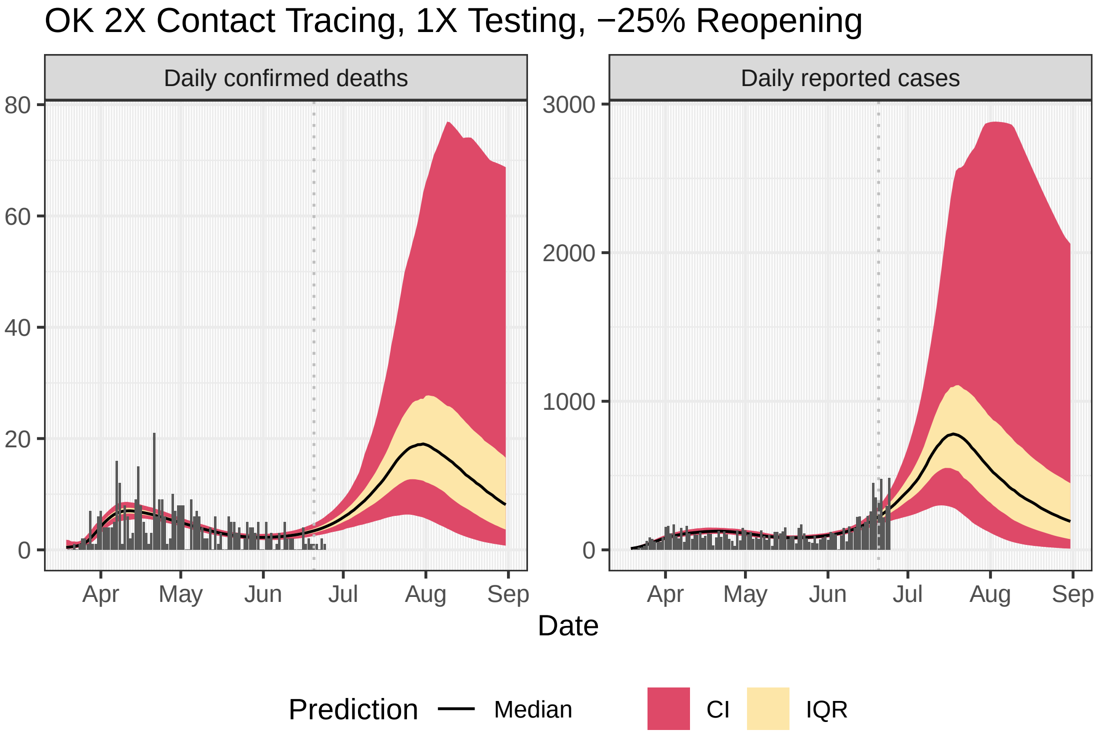
OK 2X Contact Tracing, 1X Testing, −25% Reopening
Daily confirmed deaths
Daily reported cases
80
60
40
20
0
3000
2000
1000
0
Apr
May
Jun
Jul
Aug
Sep
Date
Prediction
Median
CI
IQR

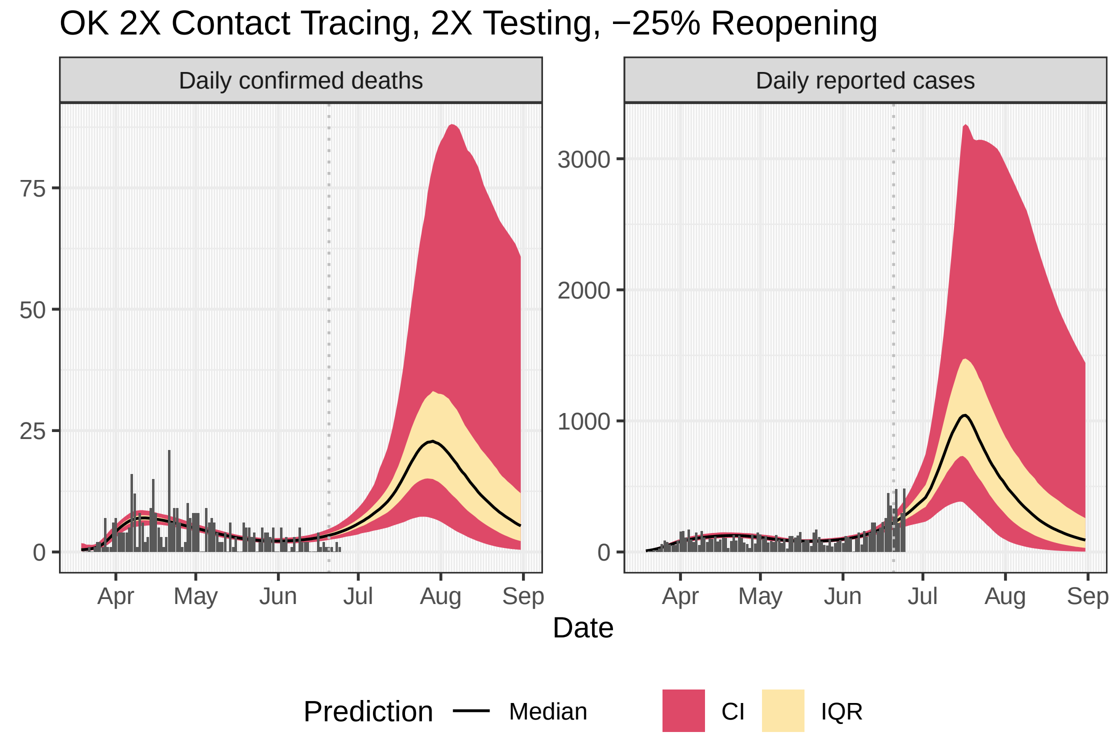

OK 2X Contact Tracing, 2X Testing, −25% Reopening
Daily confirmed deaths
Daily reported cases
75
50
25
0
3000
2000
1000
0
Apr
May
Jun
Jul
Aug
Sep
Date
Prediction
Median
CI
IQR

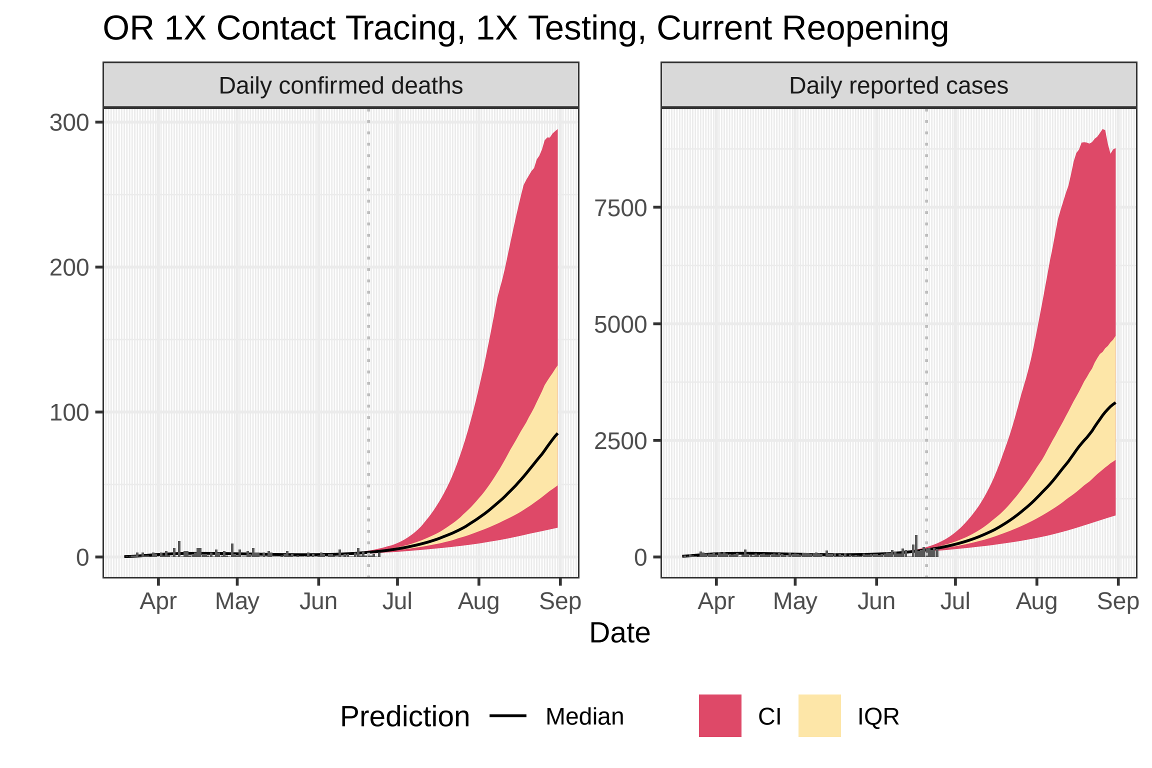

OR 1X Contact Tracing, 1X Testing, Current Reopening
Daily confirmed deaths
Daily reported cases
300
200
100
0
7500
5000
2500
0
Apr
May
Jun
Jul
Aug
Sep
Date
Prediction
Median
CI
IQR

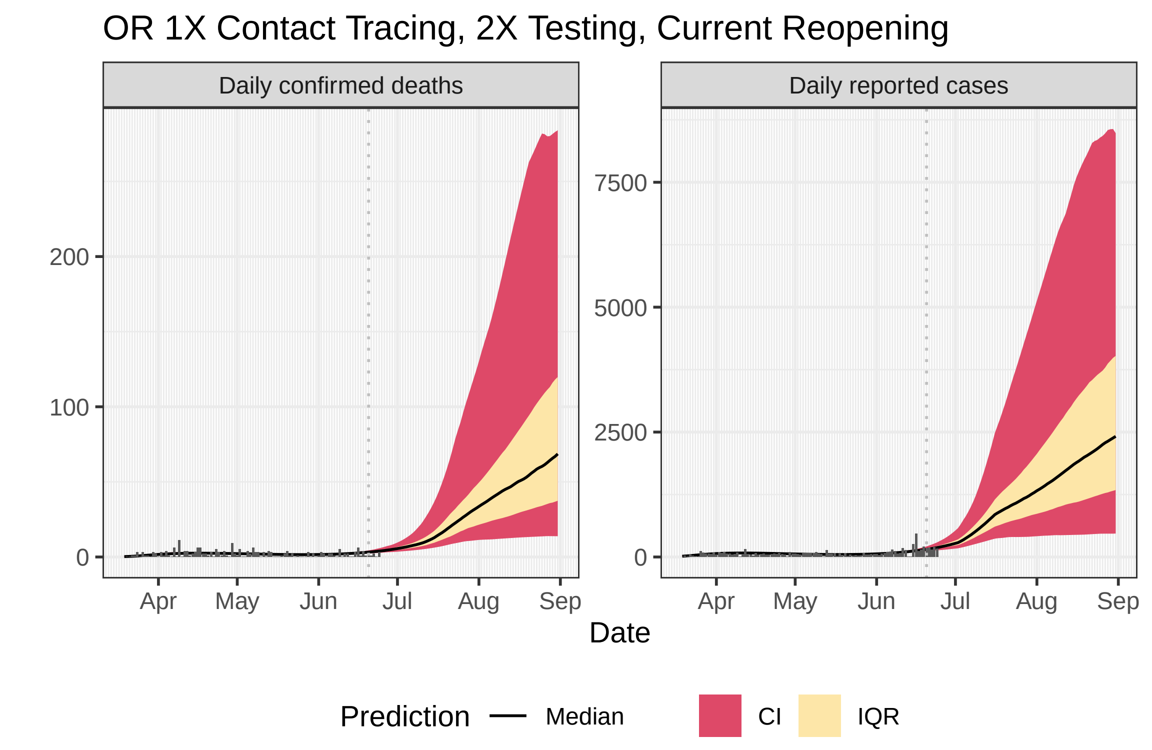

OR 1X Contact Tracing, 2X Testing, Current Reopening
Daily confirmed deaths
Daily reported cases
200
100
0
7500
5000
2500
0
Apr
May
Jun
Jul
Aug
Sep
Date
Prediction
Median
CI
IQR

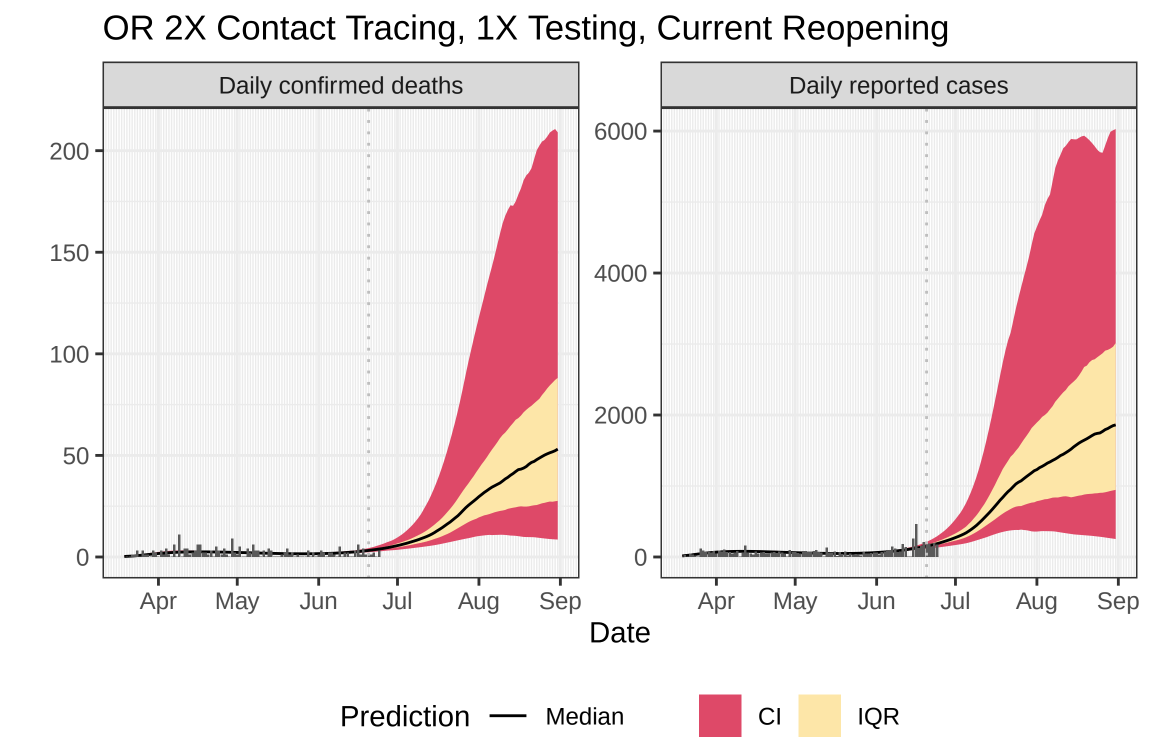

OR 2X Contact Tracing, 1X Testing, Current Reopening
Daily confirmed deaths
Daily reported cases
200
150
100
50
0
6000
4000
2000
0
Apr
May
Jun
Jul
Aug
Sep
Date
Prediction
Median
CI
IQR

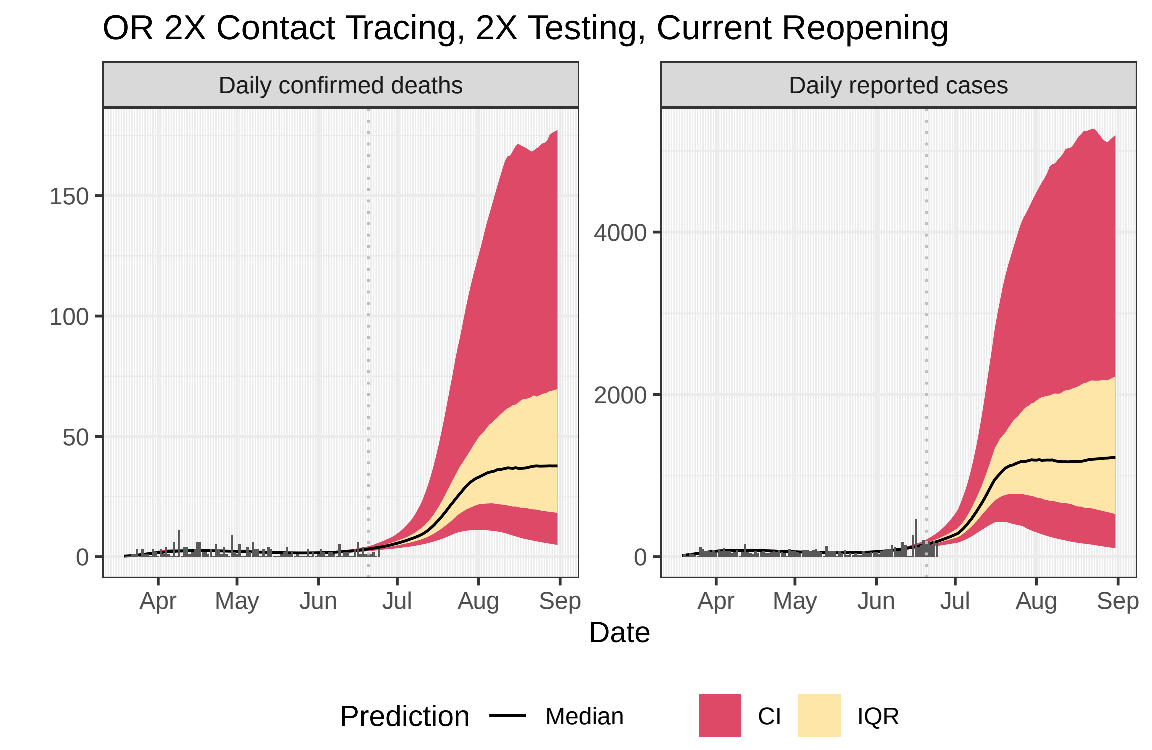

OR 2X Contact Tracing, 2X Testing, Current Reopening
Daily confirmed deaths
Daily reported cases
0
50
100
150
0
2000
4000
Apr
May
Jun
Jul
Aug
Sep
Date
Prediction
Median
CI
IQR

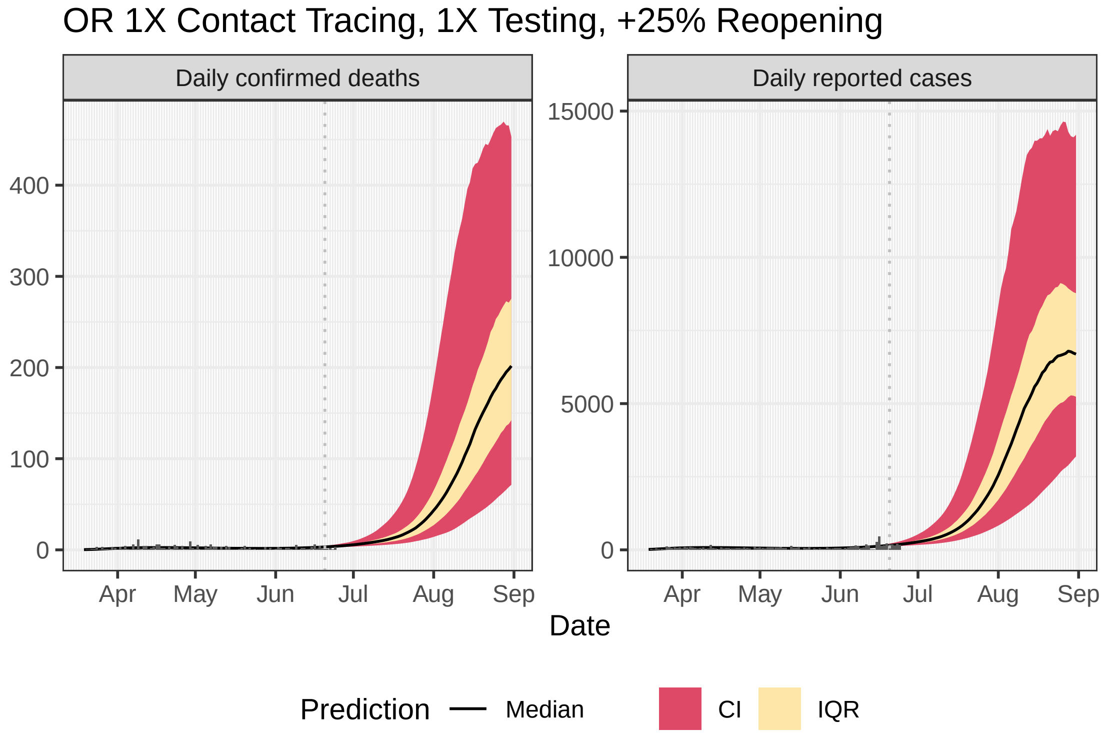

OR 1X Contact Tracing, 1X Testing, +25% Reopening
Daily confirmed deaths
Daily reported cases
400
300
200
100
0
15000
10000
5000
0
Apr
May
Jun
Jul
Aug
Sep
Date
Prediction
Median
CI
IQR

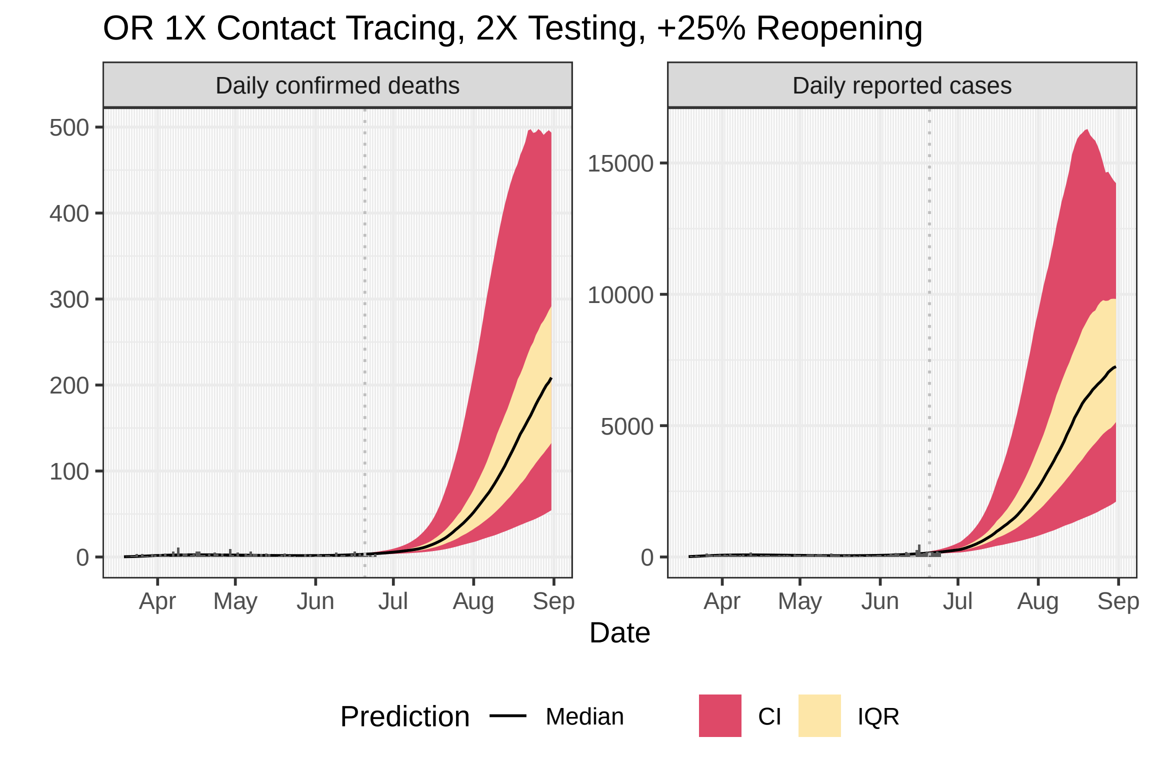

OR 1X Contact Tracing, 2X Testing, +25% Reopening
Daily confirmed deaths
Daily reported cases
500
400
300
200
100
0
15000
10000
5000
0
Apr
May
Jun
Jul
Aug
Sep
Date
Prediction
Median
CI
IQR

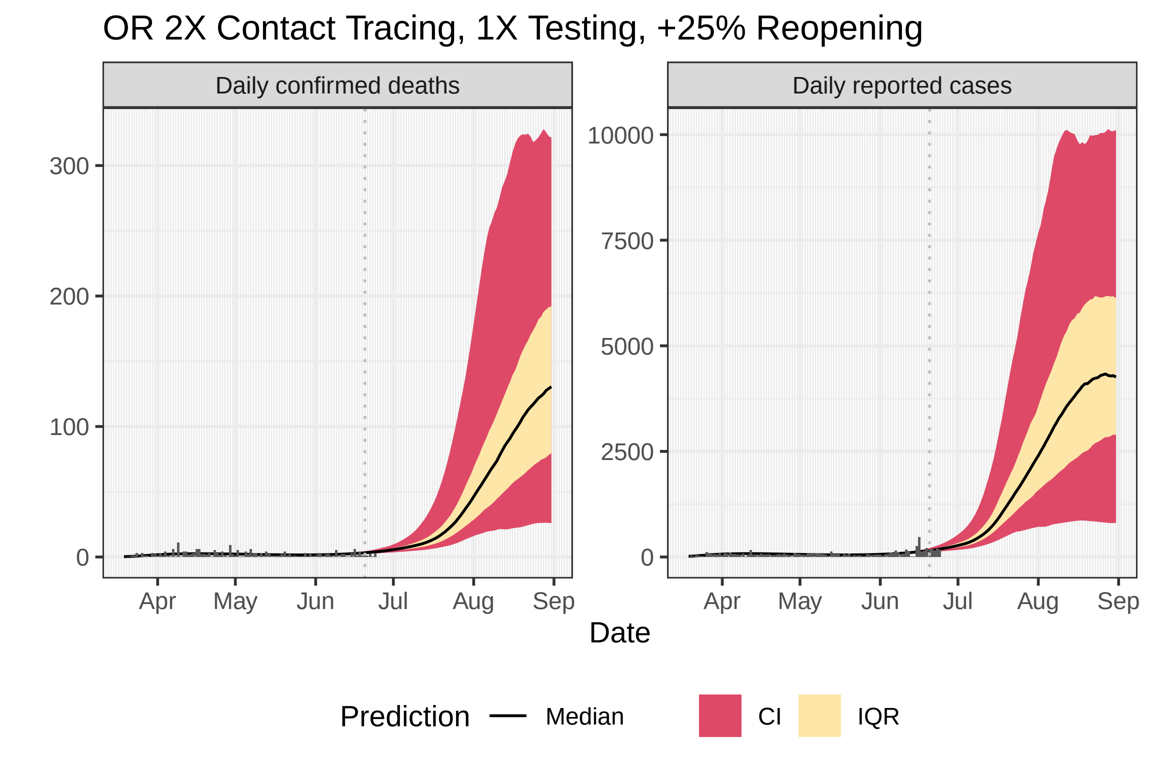

OR 2X Contact Tracing, 1X Testing, +25% Reopening
Daily confirmed deaths
Daily reported cases
300
200
100
0
10000
7500
5000
2500
0
Apr
May
Jun
Jul
Aug
Sep
Date
Prediction
Median
CI
IQR

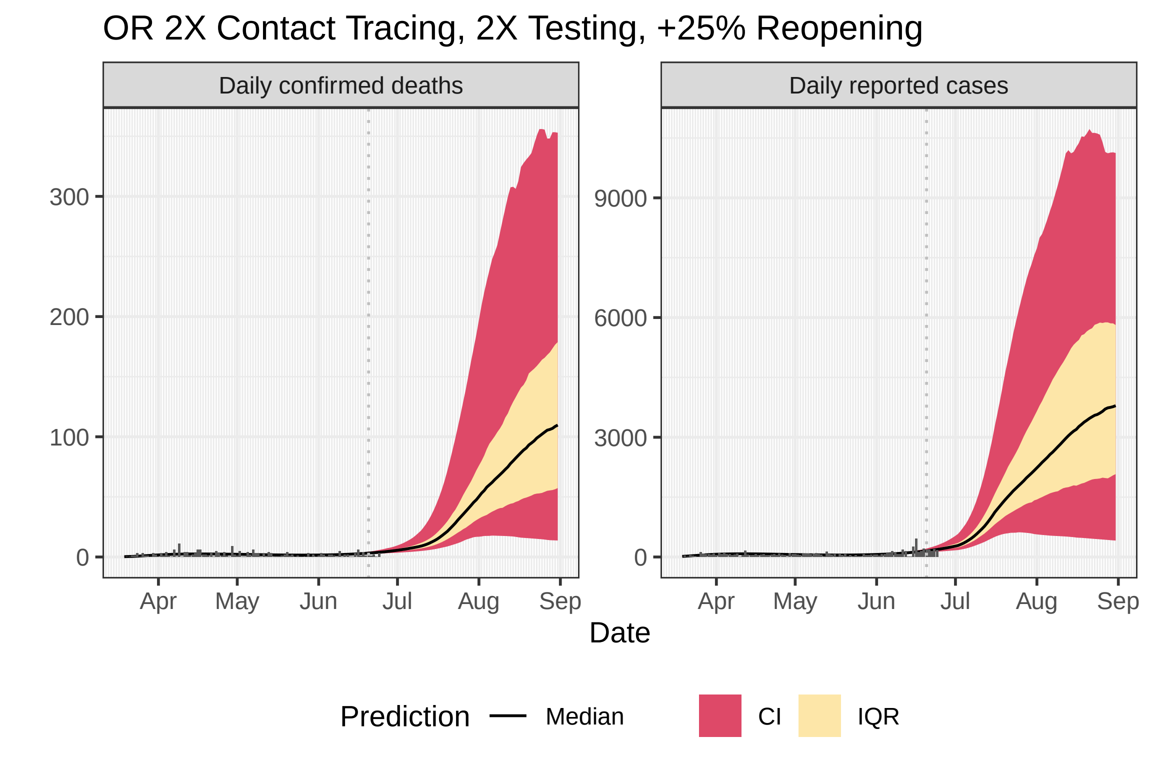
OR 2X Contact Tracing, 2X Testing, +25% Reopening
Daily confirmed deaths
Daily reported cases
300
200
100
0
9000
6000
3000
0
Apr
May
Jun
Jul
Aug
Sep
Date
Prediction
Median
CI
IQR

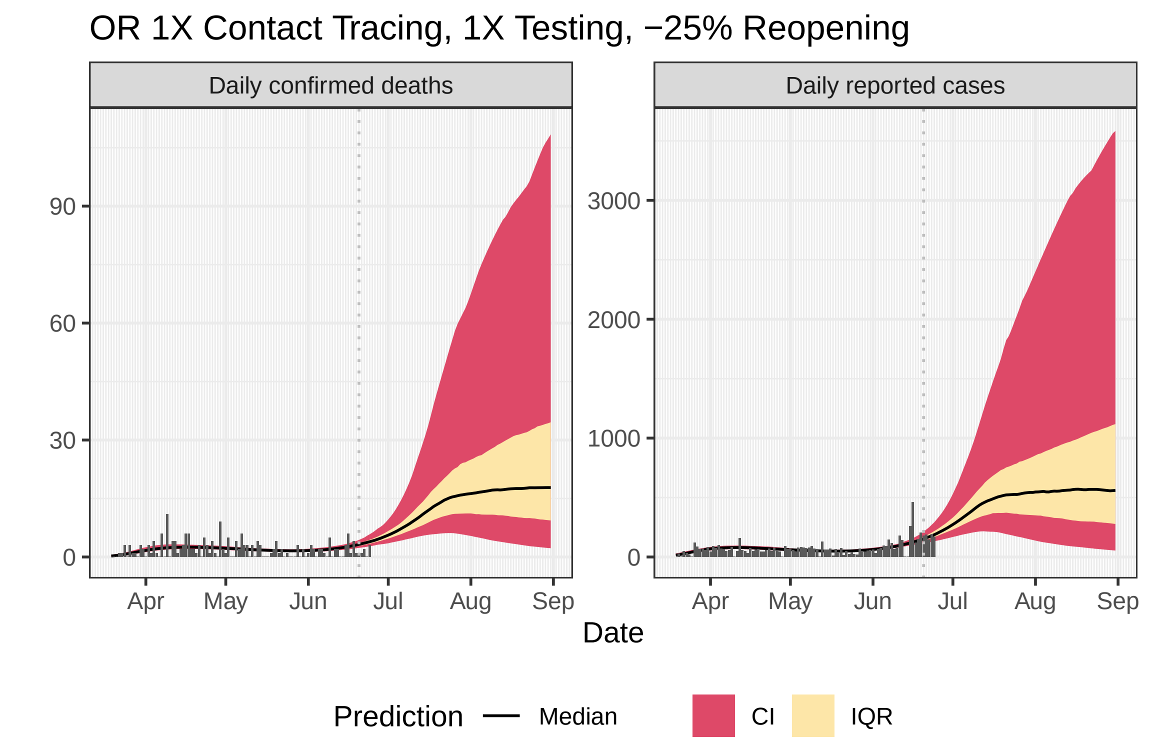

OR 1X Contact Tracing, 1X Testing, −25% Reopening
Daily confirmed deaths
Daily reported cases
90
60
30
0
3000
2000
1000
0
Apr
May
Jun
Jul
Aug
Sep
Date
Prediction
Median
CI
IQR

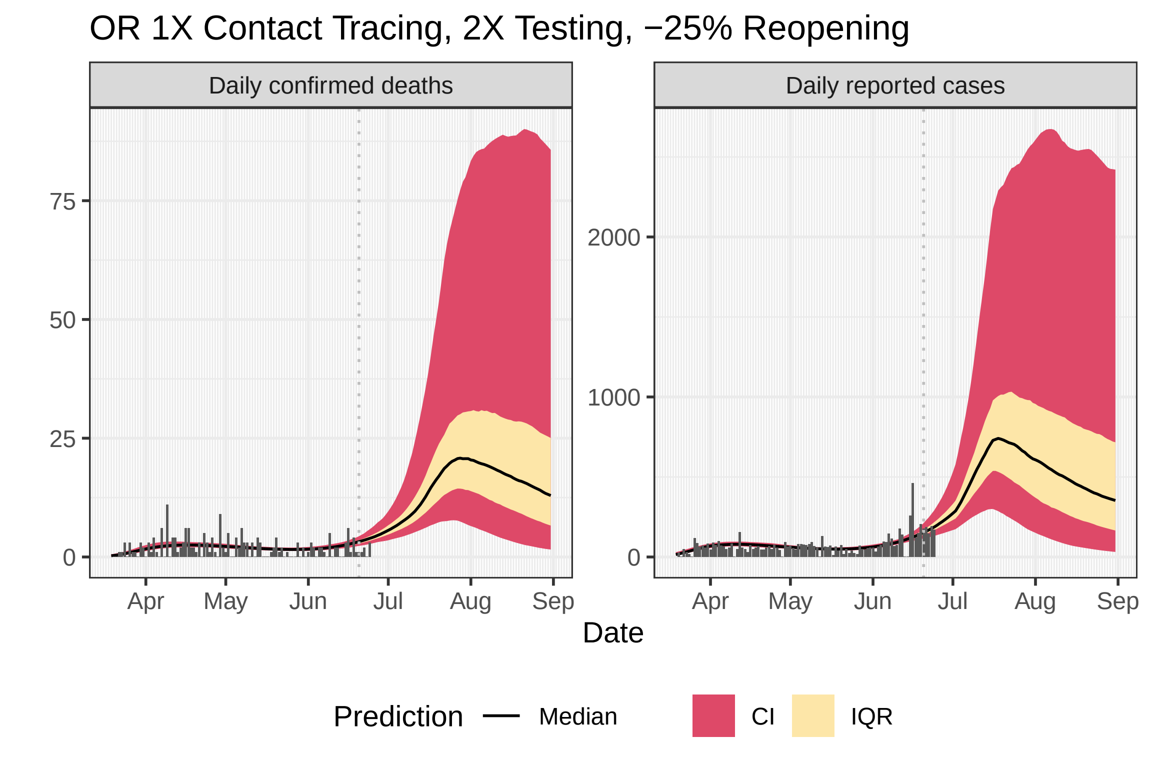
OR 1X Contact Tracing, 2X Testing, −25% Reopening
Daily confirmed deaths
Daily reported cases
75
50
25
0
2000
1000
0
Apr
May
Jun
Jul
Aug
Sep
Date
Prediction
Median
CI
IQR

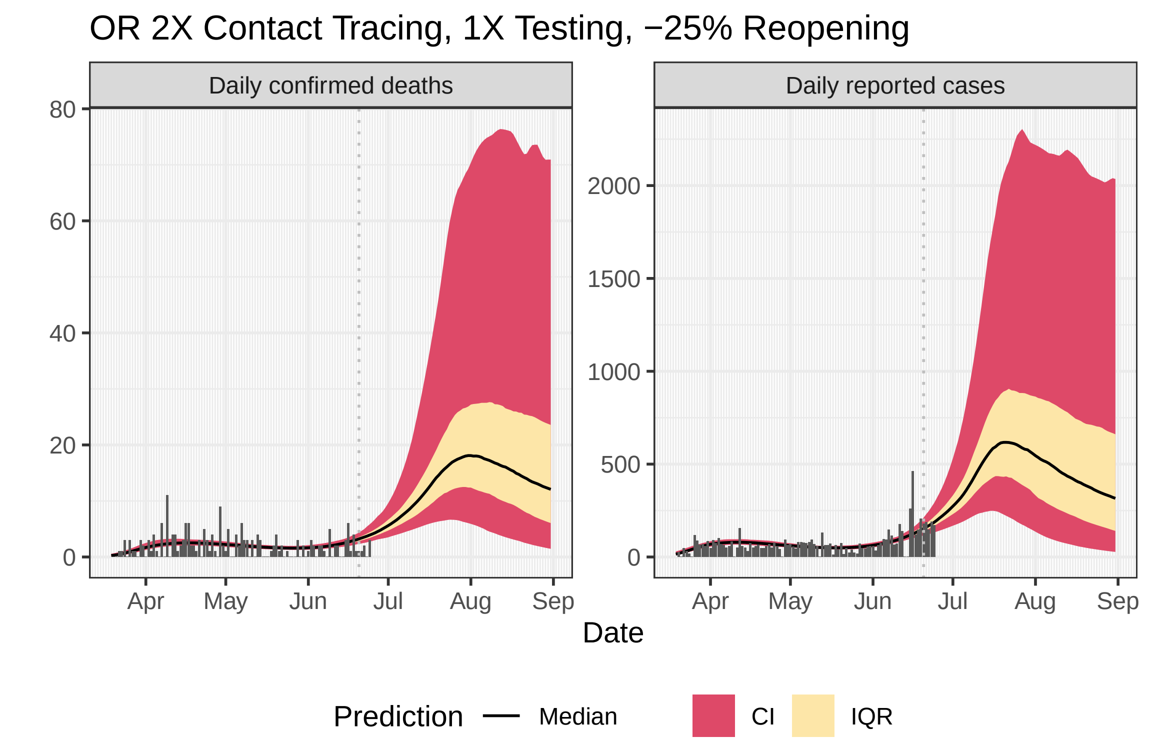
OR 2X Contact Tracing, 1X Testing, −25% Reopening
Daily confirmed deaths
Daily reported cases
80
60
40
20
0
2000
1500
1000
500
0
Apr
May
Jun
Jul
Aug
Sep
Date
Prediction
Median
CI
IQR

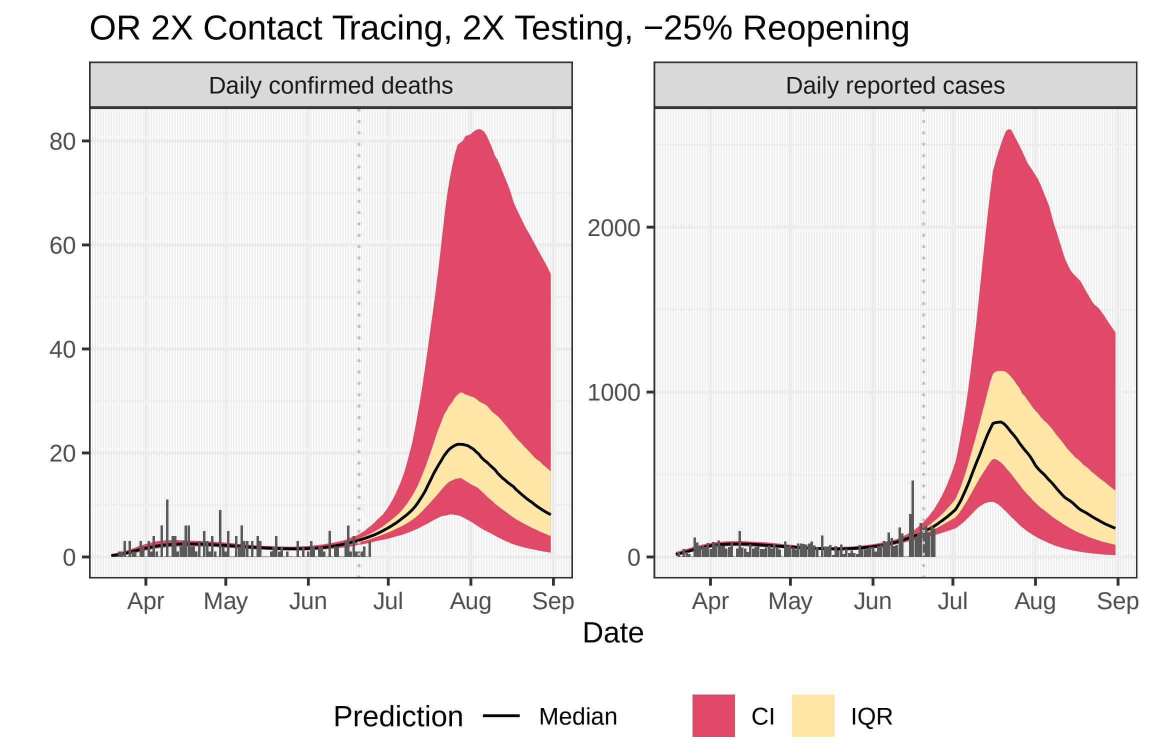

OR 2X Contact Tracing, 2X Testing, −25% Reopening
Daily confirmed deaths
Daily reported cases
80
60
40
20
0
2000
1000
0
Apr
May
Jun
Jul
Aug
Sep
Date
Prediction
Median
CI
IQR

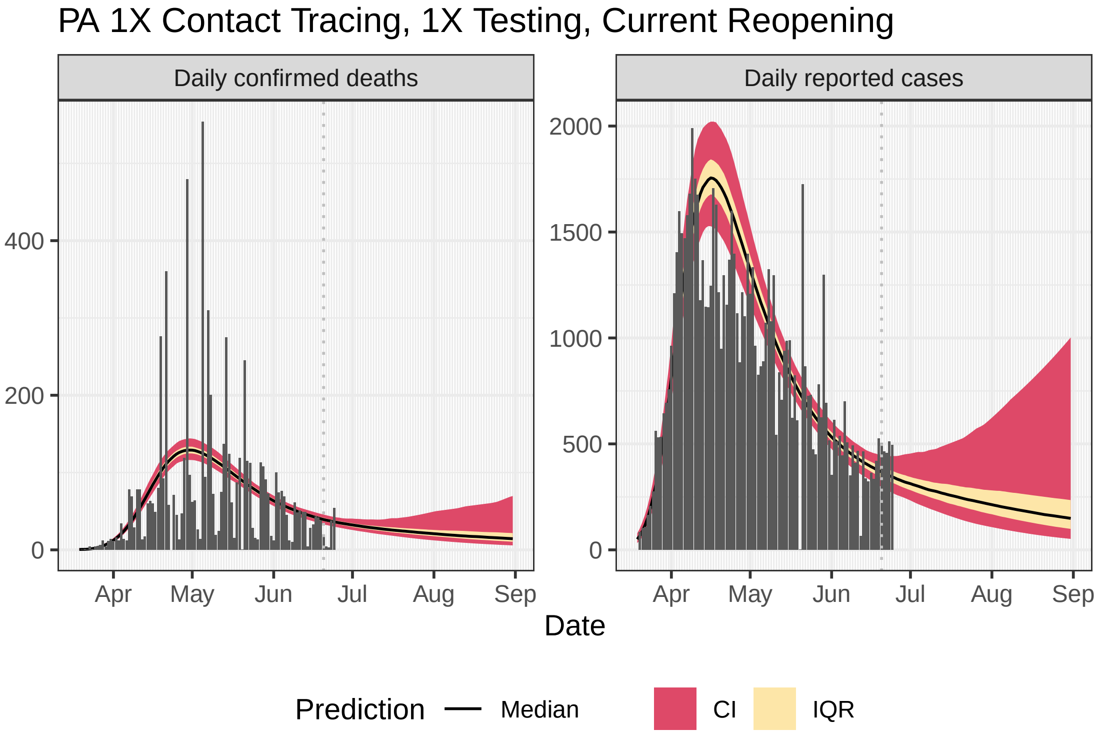
PA 1X Contact Tracing, 1X Testing, Current Reopening
Daily confirmed deaths
Daily reported cases
0
200
400
500
1000
1500
2000
Apr
May
Jun
Jul
Aug
Sep
Date
Prediction
Median
CI
IQR

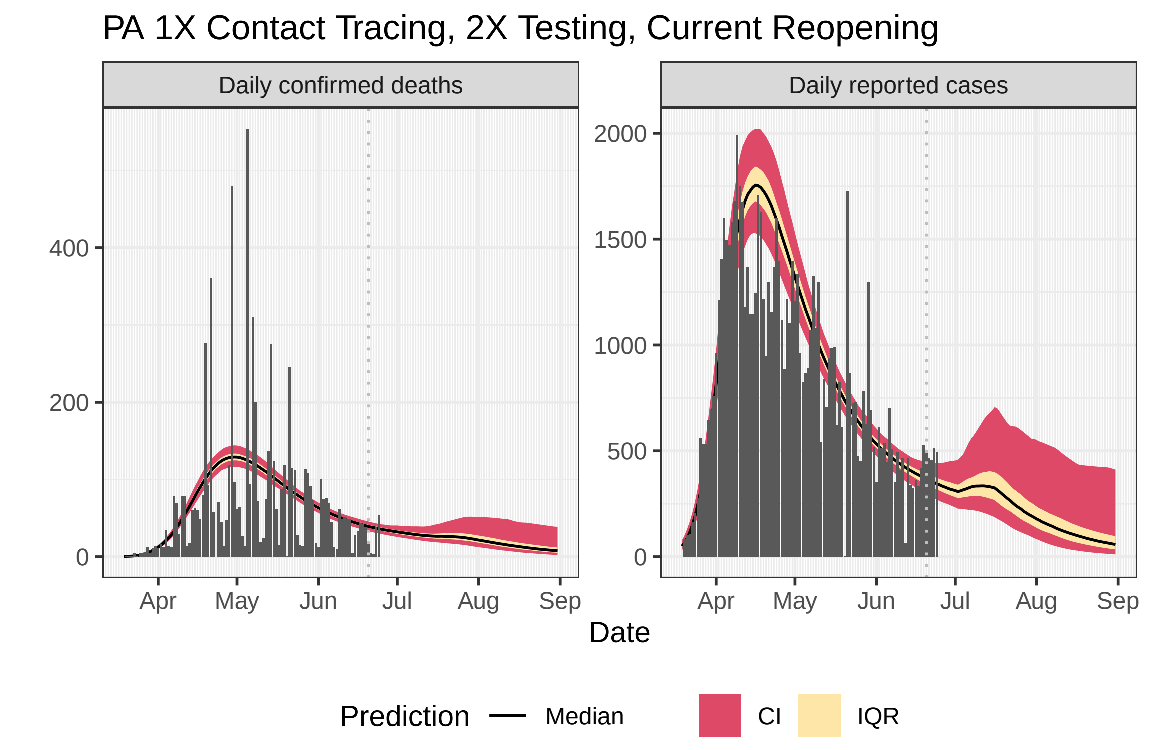
PA 1X Contact Tracing, 2X Testing, Current Reopening
Daily confirmed deaths
Daily reported cases
0
200
400
500
1000
1500
2000
Apr
May
Jun
Jul
Aug
Sep
Date
Prediction
Median
CI
IQR

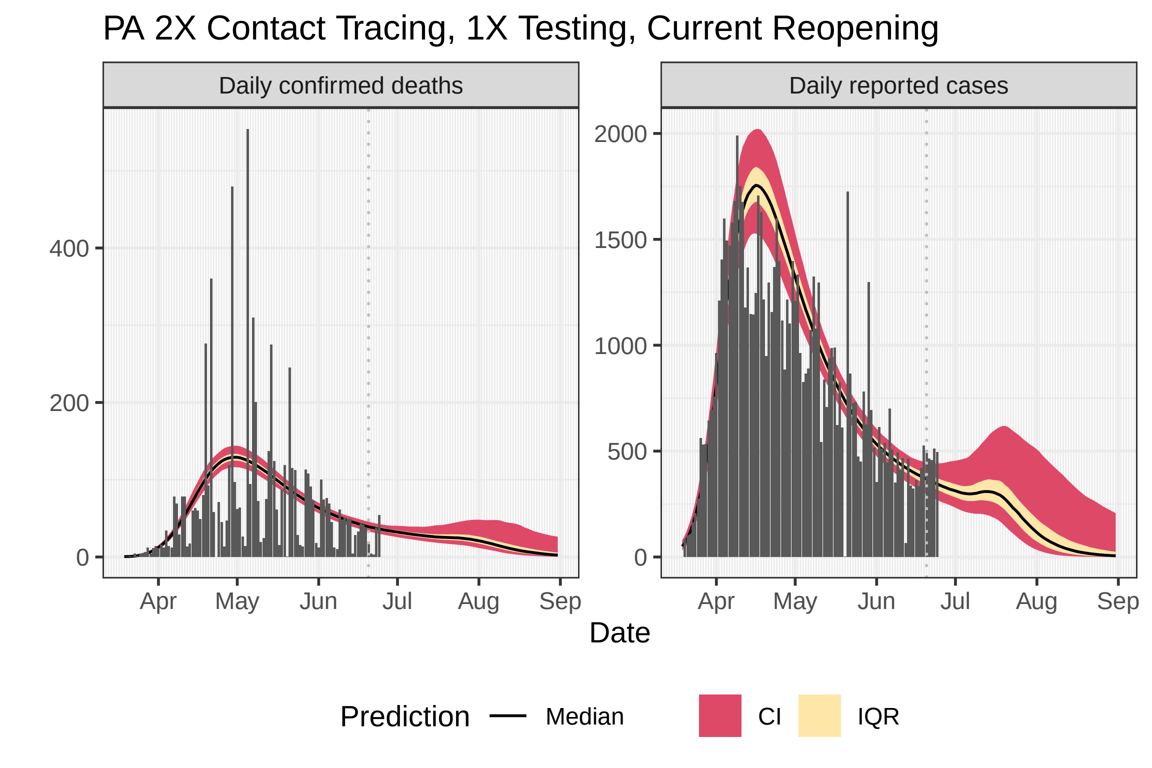
PA 2X Contact Tracing, 1X Testing, Current Reopening
Daily confirmed deaths
Daily reported cases
0
200
400
500
1000
1500
2000
Apr
May
Jun
Jul
Aug
Sep
Date
Prediction
Median
CI
IQR

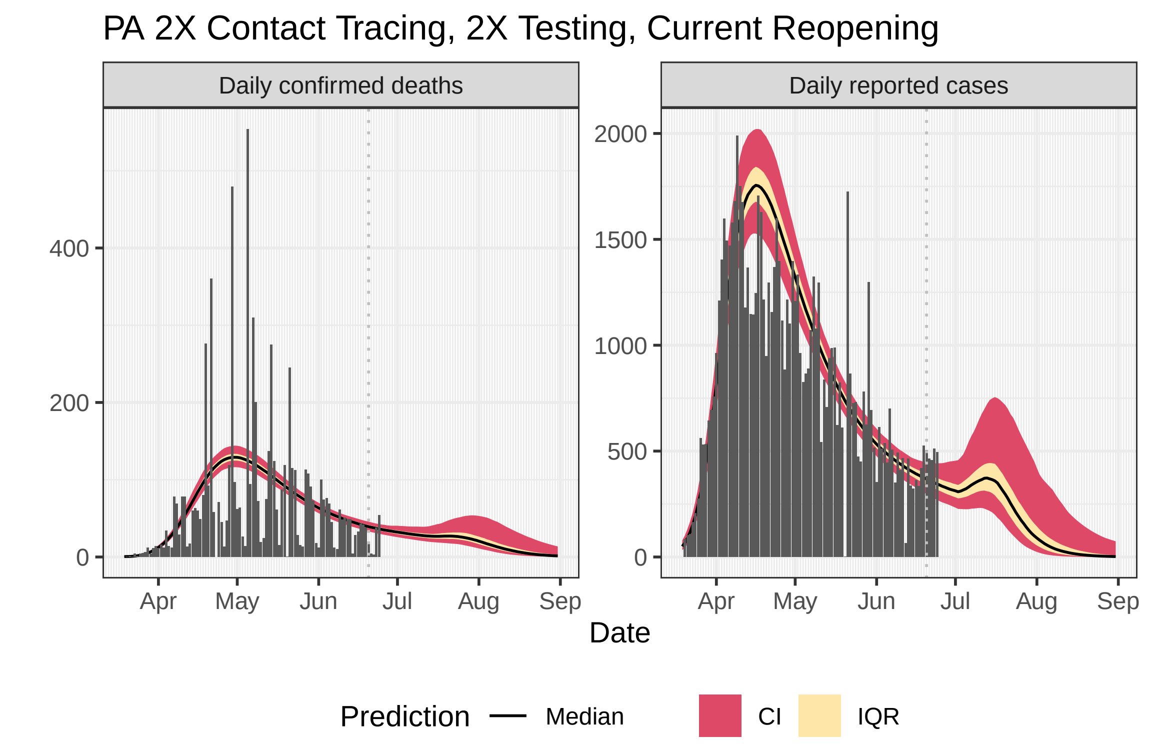

PA 2X Contact Tracing, 2X Testing, Current Reopening
Daily confirmed deaths
Daily reported cases
0
200
400
500
1000
1500
2000
Apr
May
Jun
Jul
Aug
Sep
Date
Prediction
Median
CI
IQR

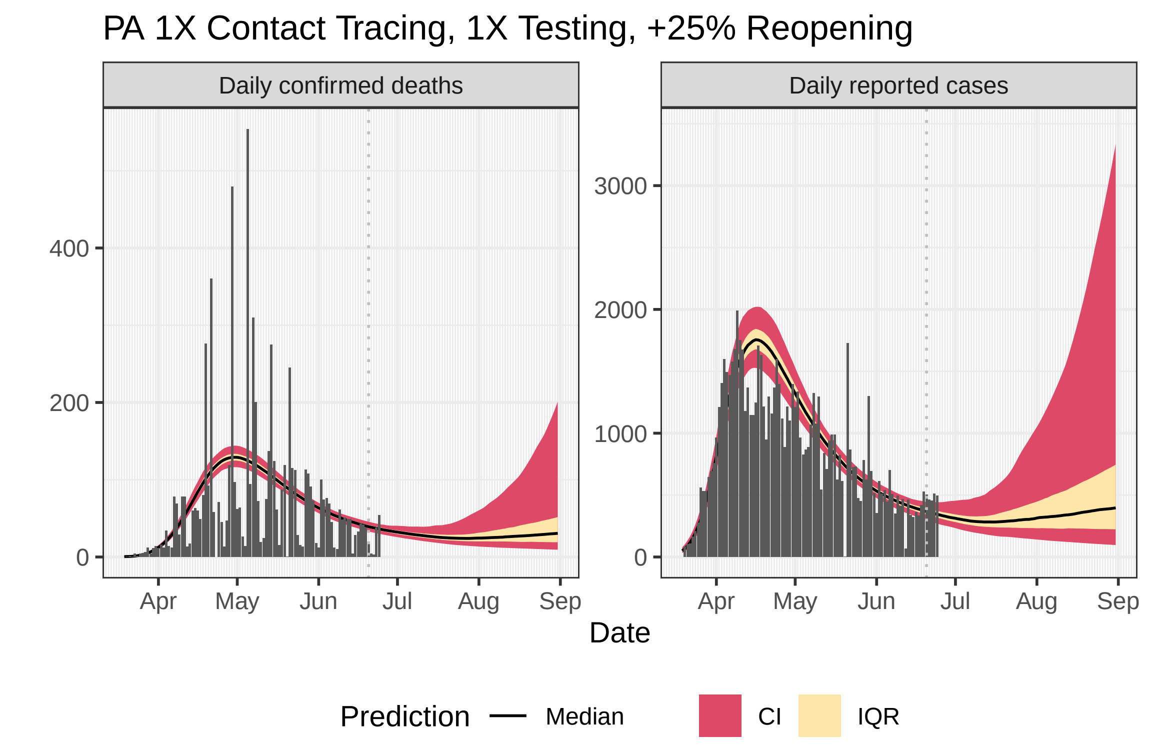
PA 1X Contact Tracing, 1X Testing, +25% Reopening
Daily confirmed deaths
Daily reported cases
0
200
400
1000
2000
3000
Apr
May
Jun
Jul
Aug
Sep
Date
Prediction
Median
CI
IQR

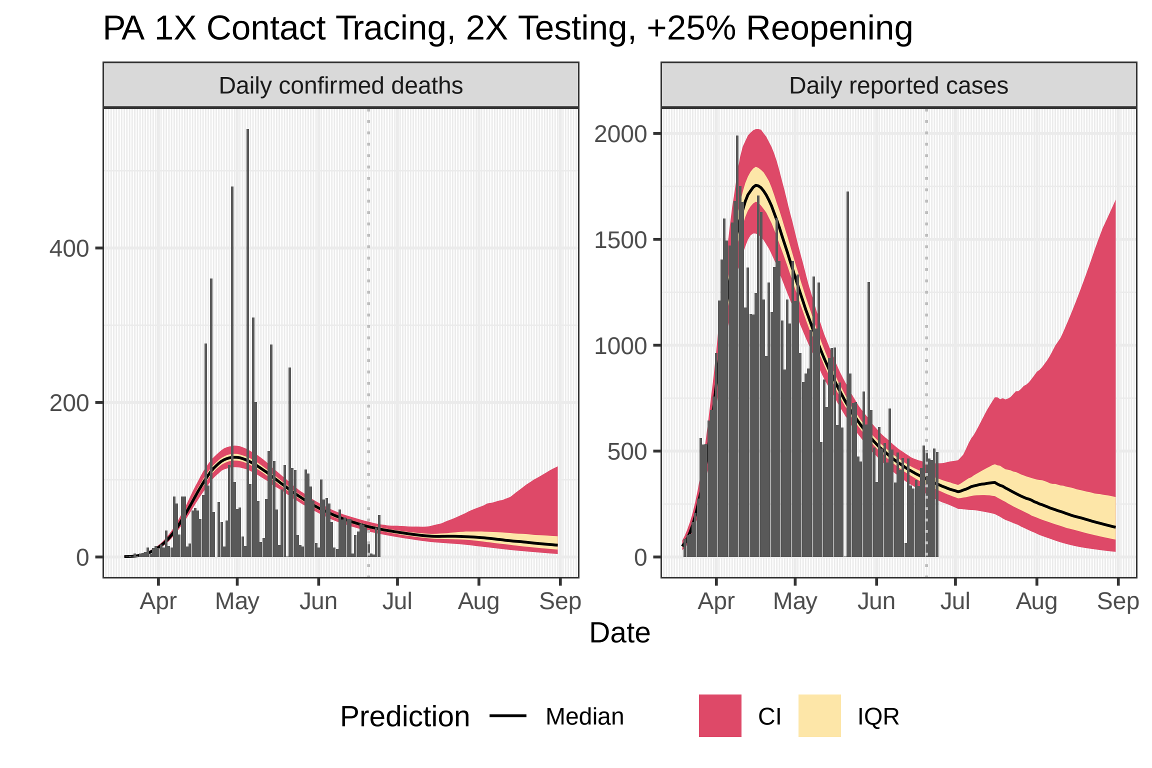
PA 1X Contact Tracing, 2X Testing, +25% Reopening
Daily confirmed deaths
Daily reported cases
400
200
0
2000
1500
1000
500
0
Apr
May
Jun
Jul
Aug
Sep
Date
Prediction
Median
CI
IQR

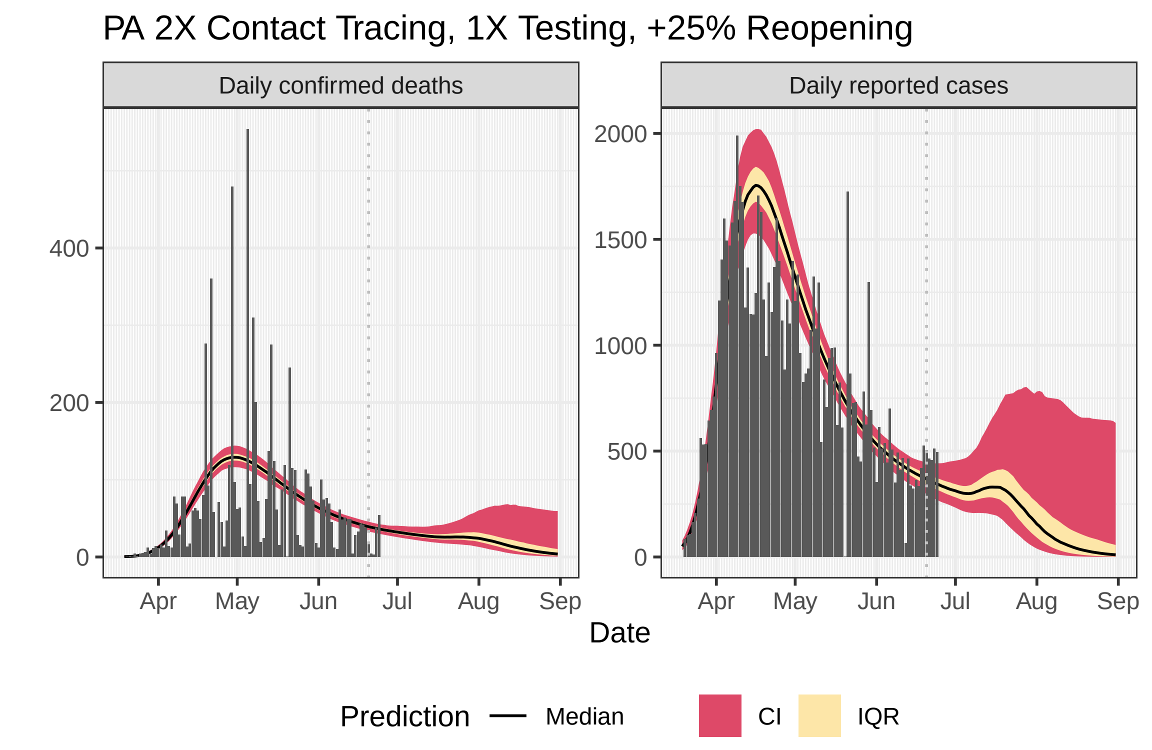

PA 2X Contact Tracing, 1X Testing, +25% Reopening
Daily confirmed deaths
Daily reported cases
0
200
400
500
1000
1500
2000
Apr
May
Jun
Jul
Aug
Sep
Date
Prediction
Median
CI
IQR

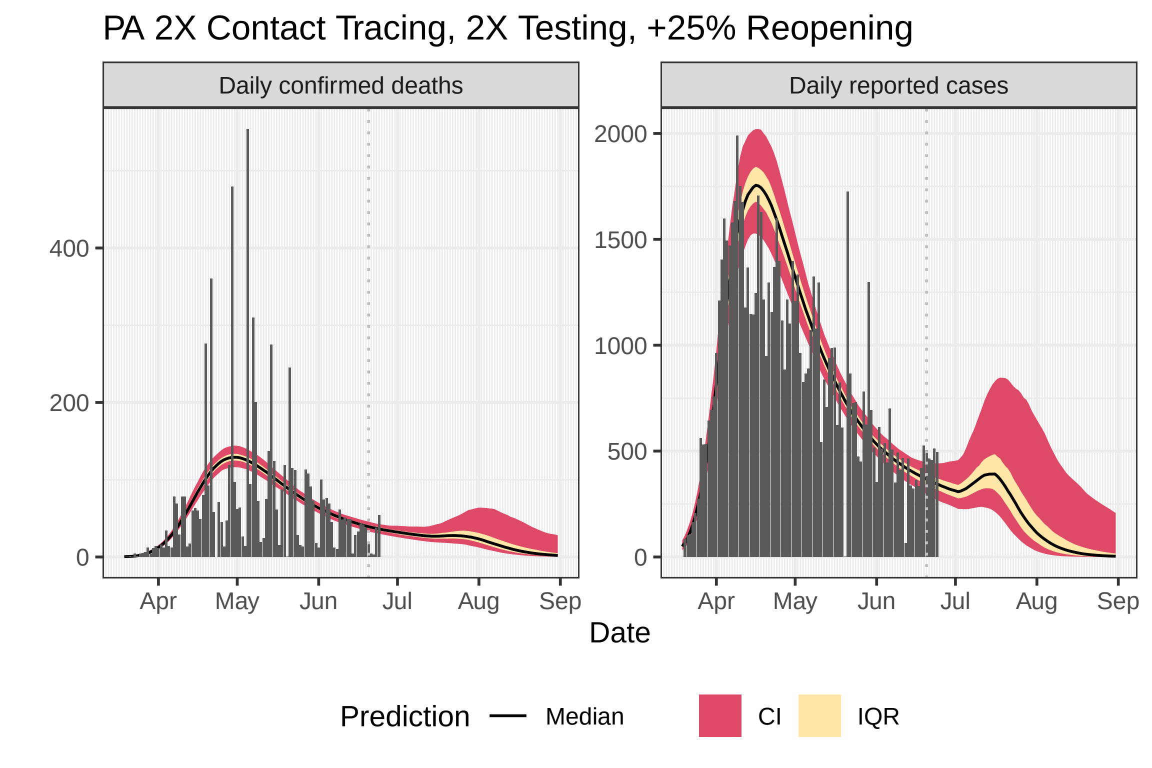

PA 2X Contact Tracing, 2X Testing, +25% Reopening
Daily confirmed deaths
Daily reported cases
400
200
0
2000
1500
1000
500
0
Apr
May
Jun
Jul
Aug
Sep
Date
Prediction
Median
CI
IQR

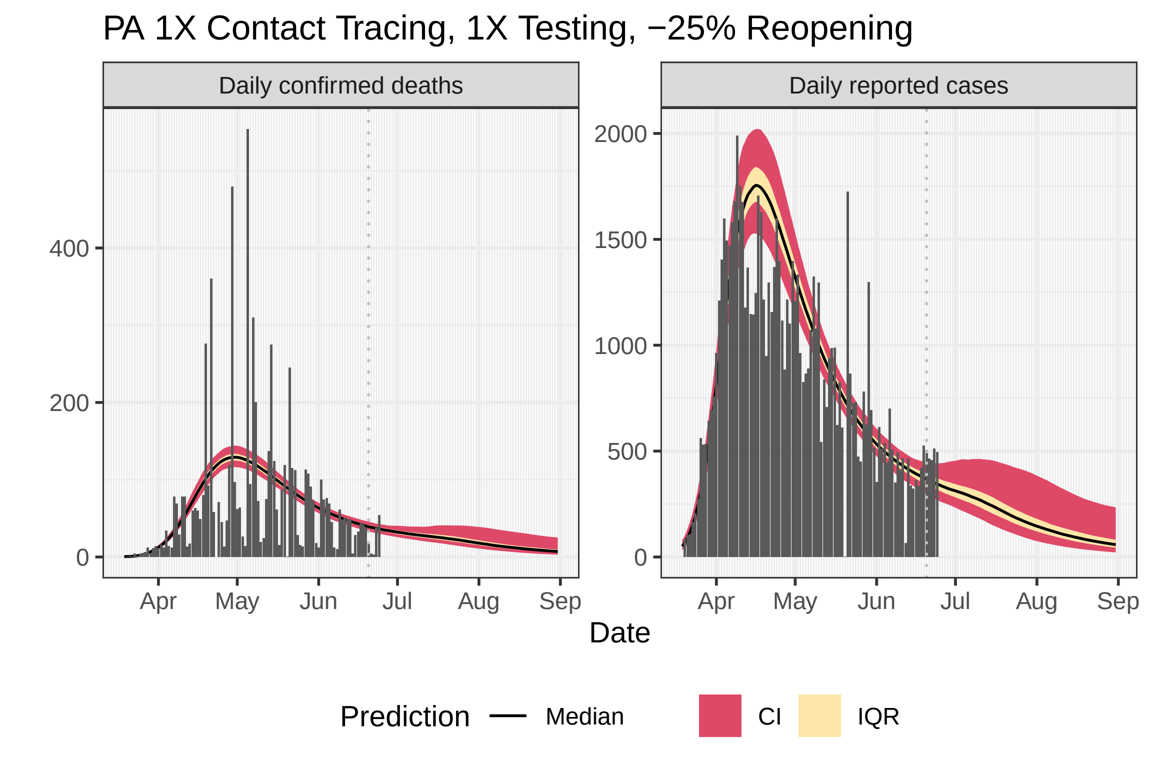

PA 1X Contact Tracing, 1X Testing, −25% Reopening
Daily confirmed deaths
Daily reported cases
0
200
400
0
500
1000
1500
2000
Apr
May
Jun
Jul
Aug
Sep
Date
Prediction
Median
CI
IQR

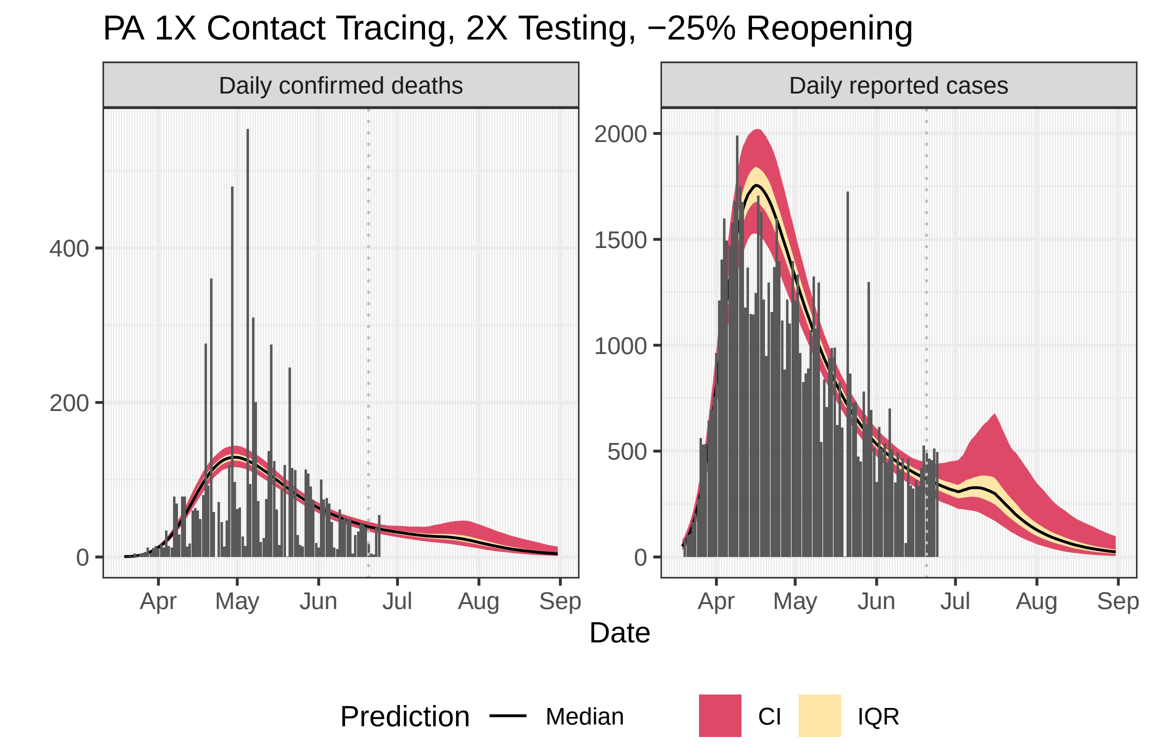
PA 1X Contact Tracing, 2X Testing, −25% Reopening
Daily confirmed deaths
Daily reported cases
0
200
400
500
1000
1500
2000
Apr
May
Jun
Jul
Aug
Sep
Date
Prediction
Median
CI
IQR

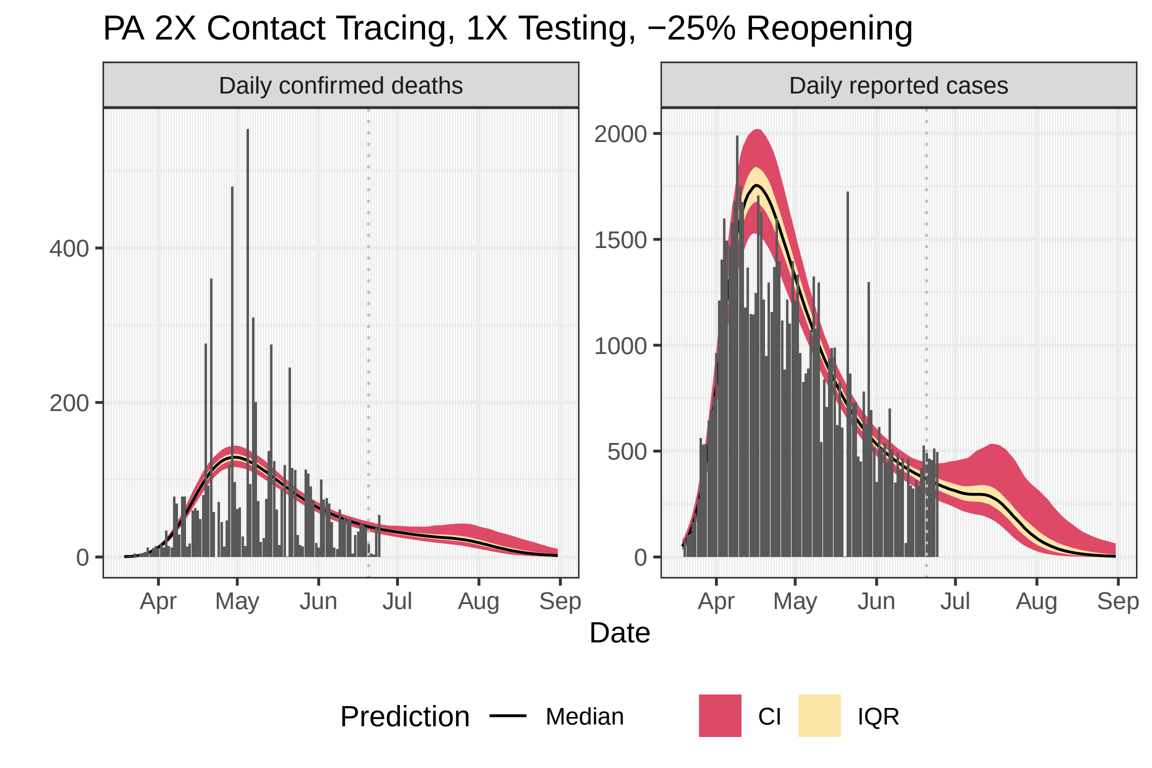

PA 2X Contact Tracing, 1X Testing, −25% Reopening
Daily confirmed deaths
Daily reported cases
0
200
400
500
1000
1500
2000
Apr
May
Jun
Jul
Aug
Sep
Date
Prediction
Median
CI
IQR

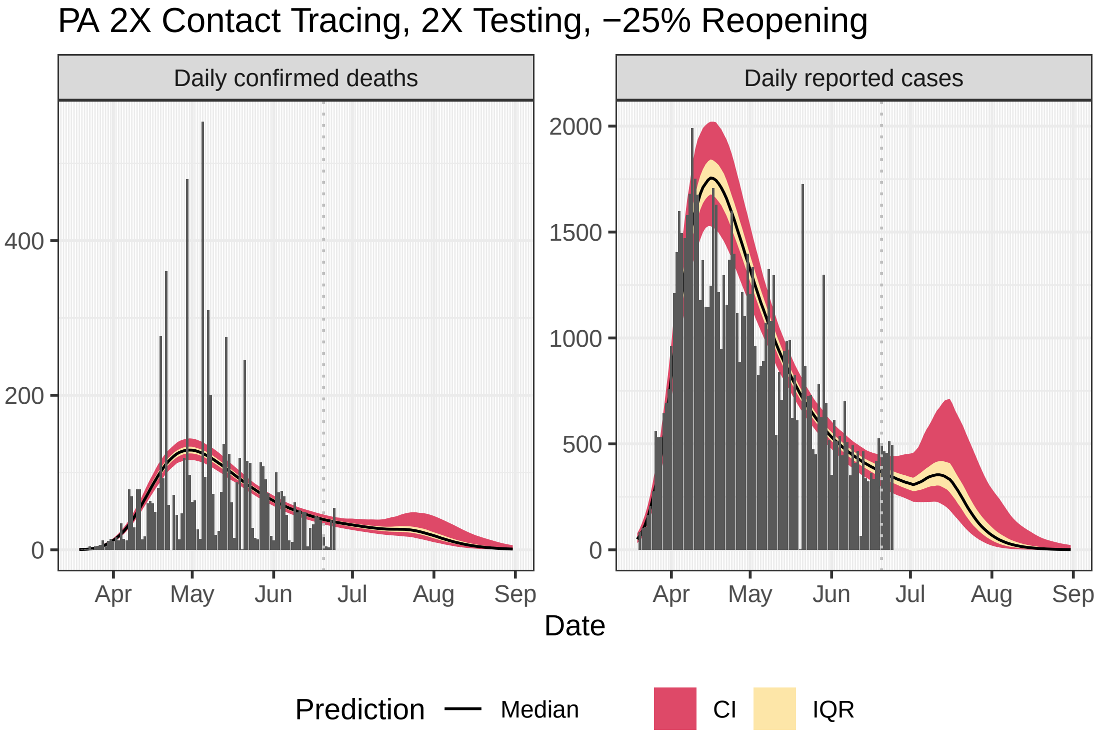

PA 2X Contact Tracing, 2X Testing, −25% Reopening
Daily confirmed deaths
Daily reported cases
0
200
400
500
1000
1500
2000
Apr
May
Jun
Jul
Aug
Sep
Date
Prediction
Median
CI
IQR

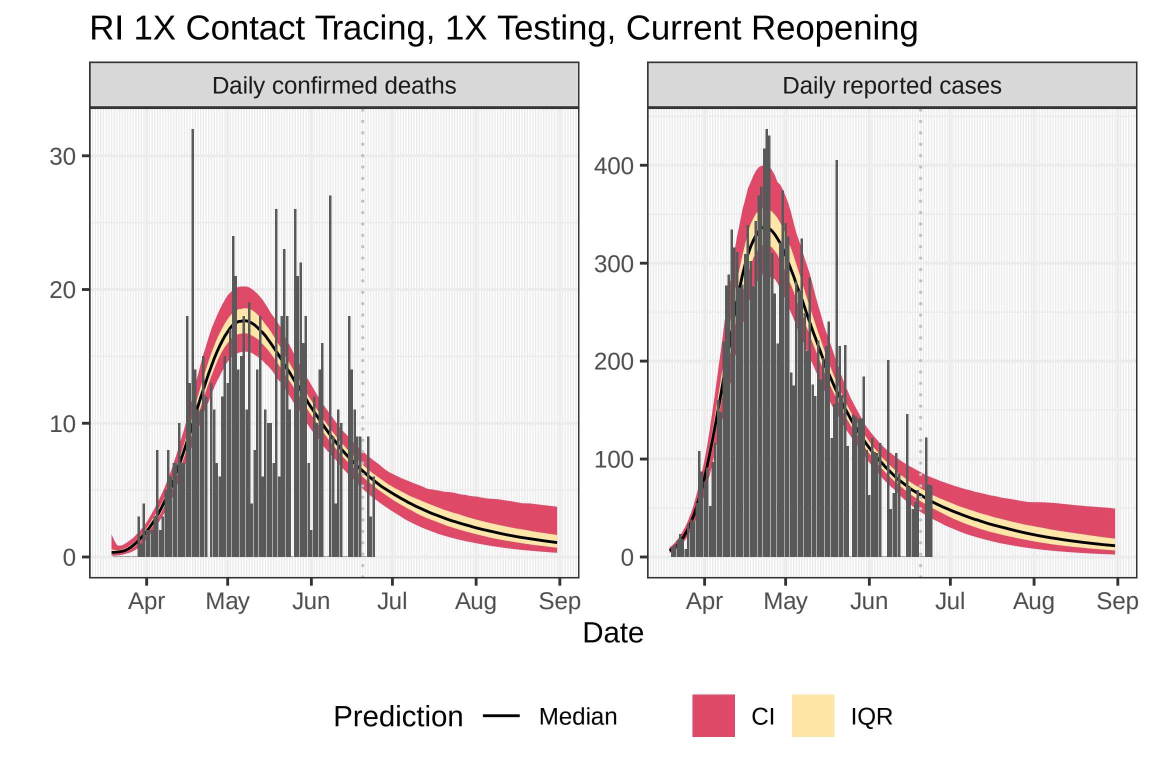
RI 1X Contact Tracing, 1X Testing, Current Reopening
Daily confirmed deaths
Daily reported cases
0
10
20
30
0
100
200
300
400
Apr
May
Jun
Jul
Aug
Sep
Date
Prediction
Median
CI
IQR

RI 1X Contact Tracing, 2X Testing, Current Reopening

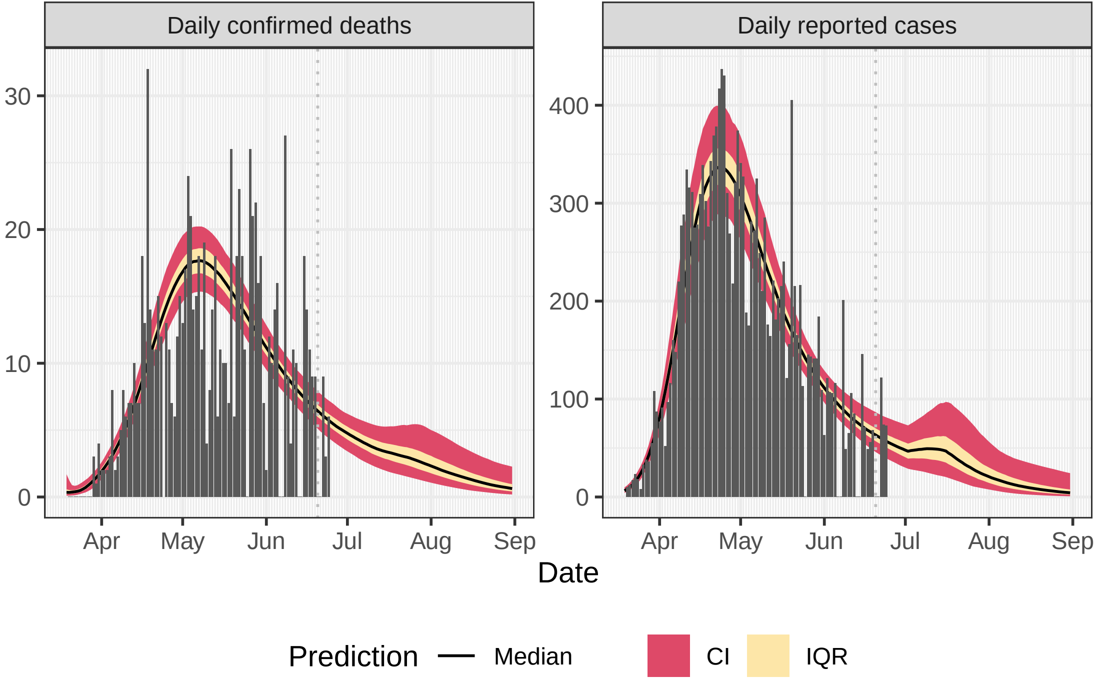

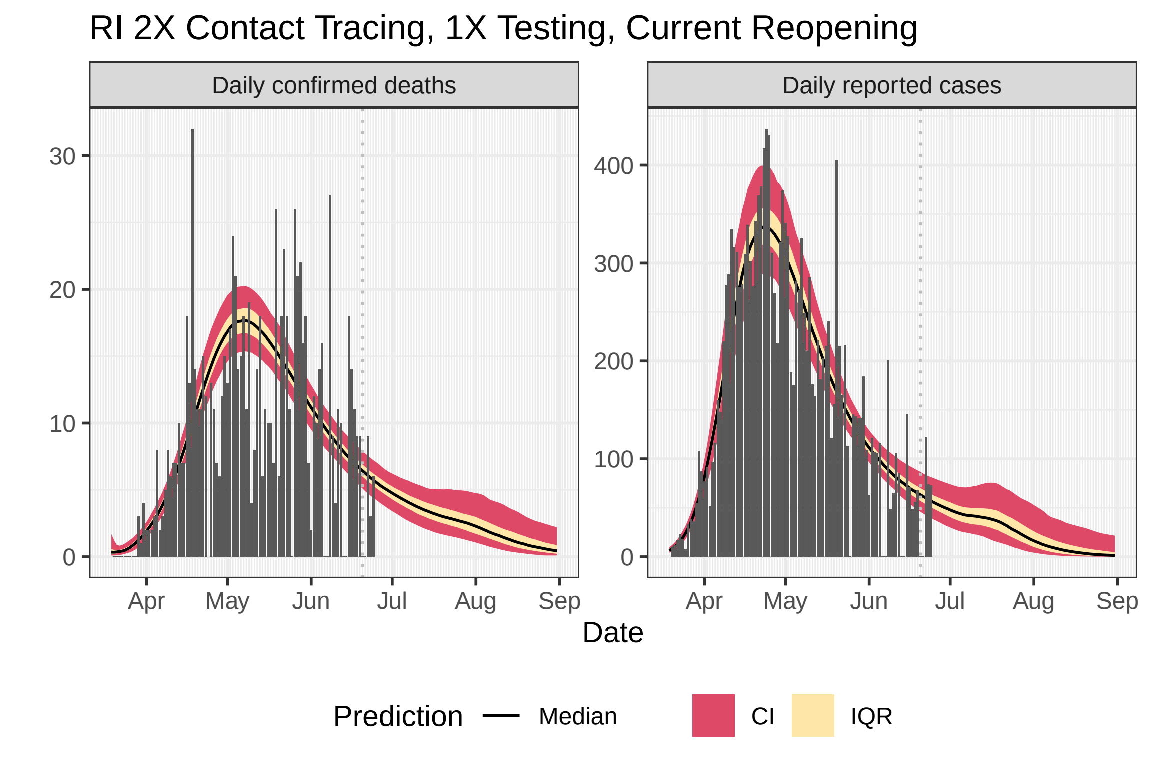
RI 2X Contact Tracing, 1X Testing, Current Reopening
Daily confirmed deaths
Daily reported cases
30
20
10
0
400
300
200
100
0
Apr
May
Jun
Jul
Aug
Sep
Date
Prediction
Median
CI
IQR

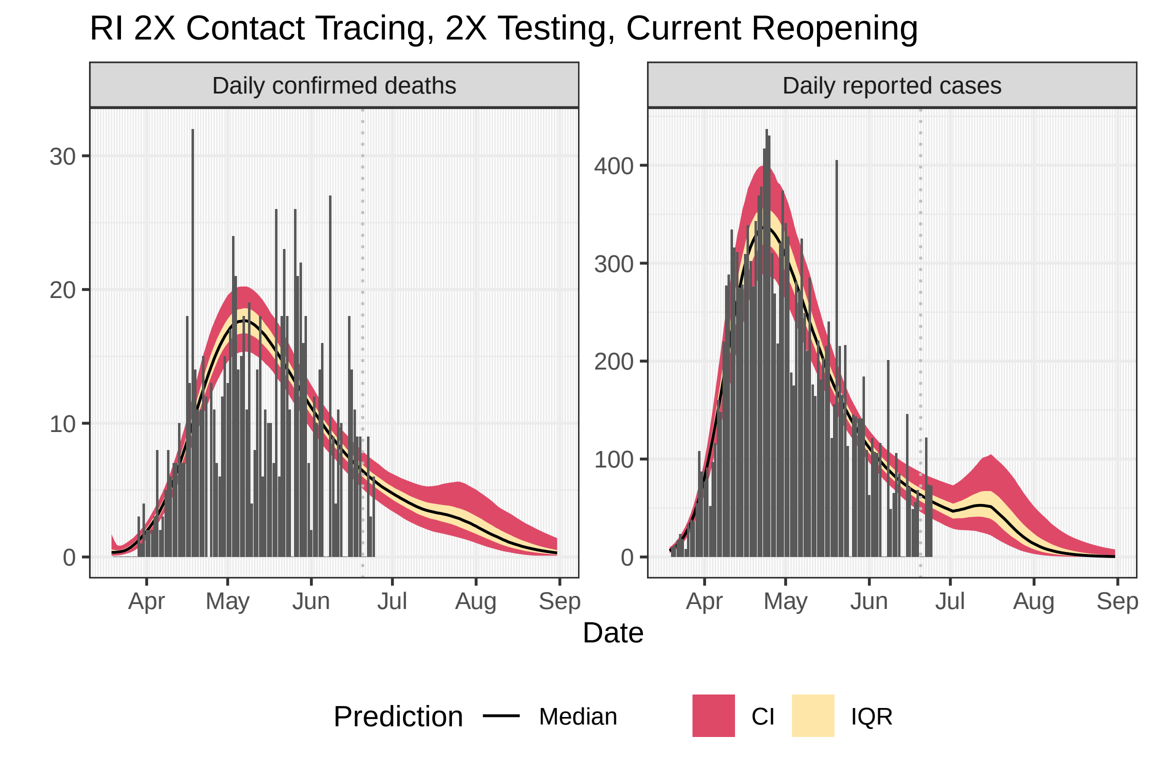

RI 2X Contact Tracing, 2X Testing, Current Reopening
Daily confirmed deaths
Daily reported cases
0
10
20
30
0
100
200
300
400
Apr
May
Jun
Jul
Aug
Sep
Date
Prediction
Median
CI
IQR

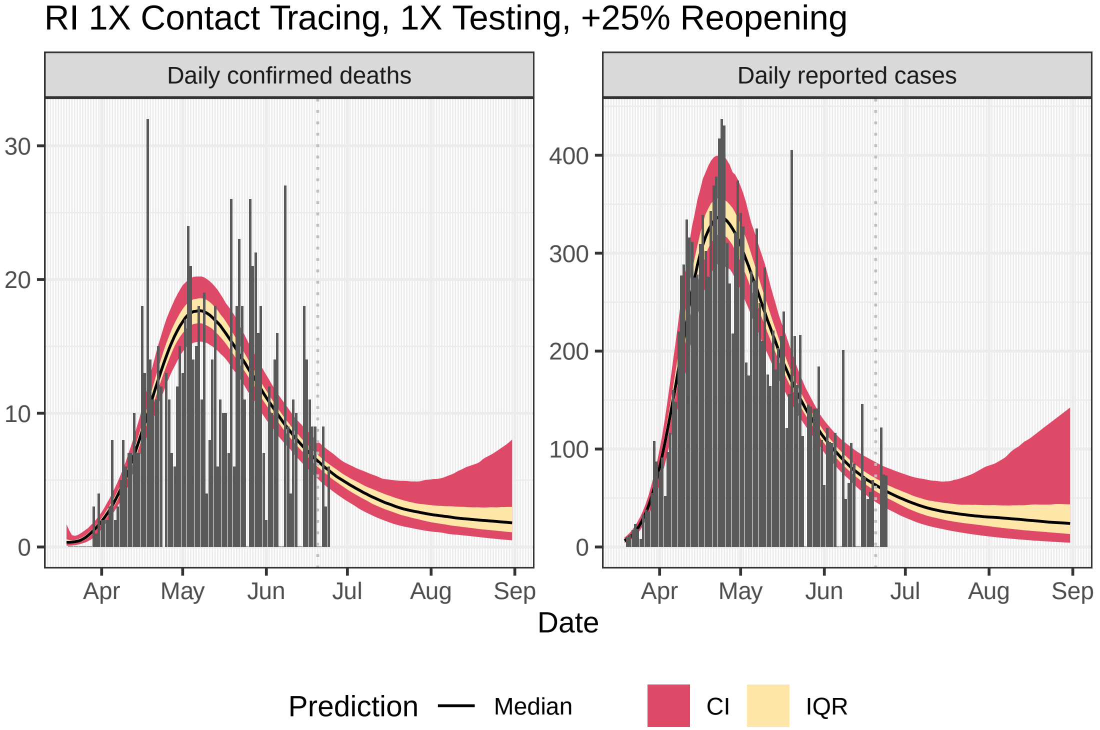

RI 1X Contact Tracing, 1X Testing, +25% Reopening
Daily confirmed deaths
Daily reported cases
0
10
20
30
0
100
200
300
400
Apr
May
Jun
Jul
Aug
Sep
Date
Prediction
Median
CI
IQR

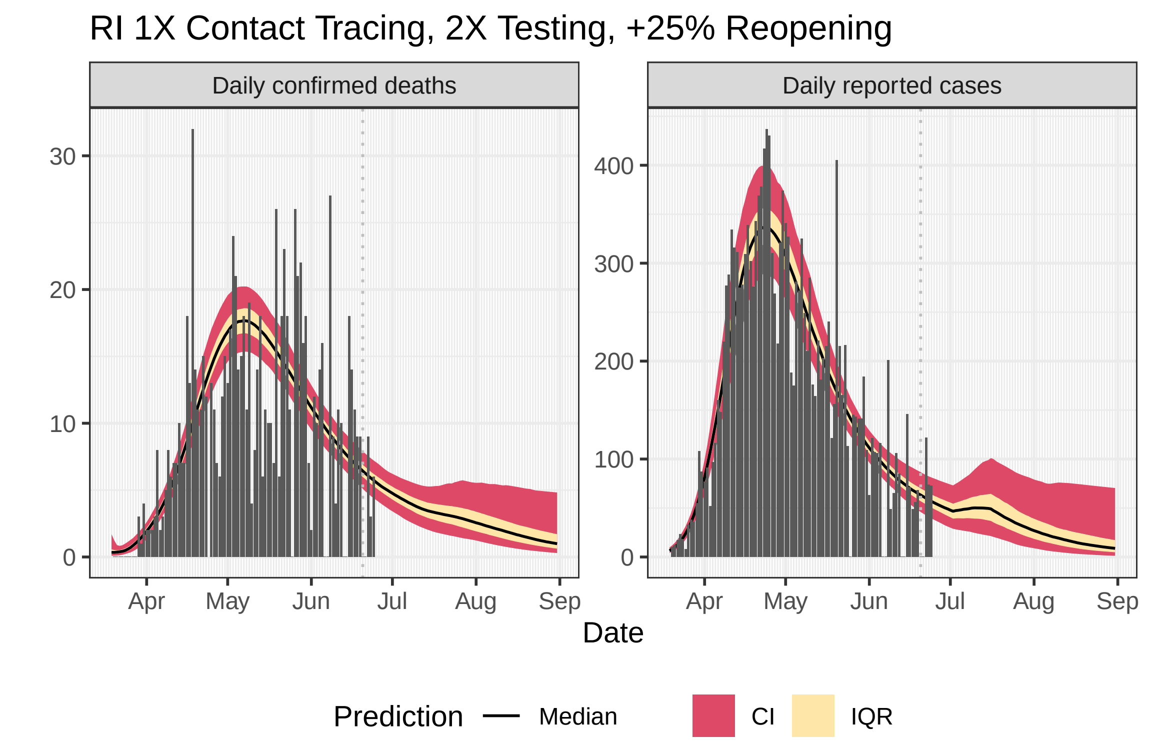

RI 1X Contact Tracing, 2X Testing, +25% Reopening
Daily confirmed deaths
Daily reported cases
0
10
20
30
0
100
200
300
400
Apr
May
Jun
Jul
Aug
Sep
Date
Prediction
Median
CI
IQR

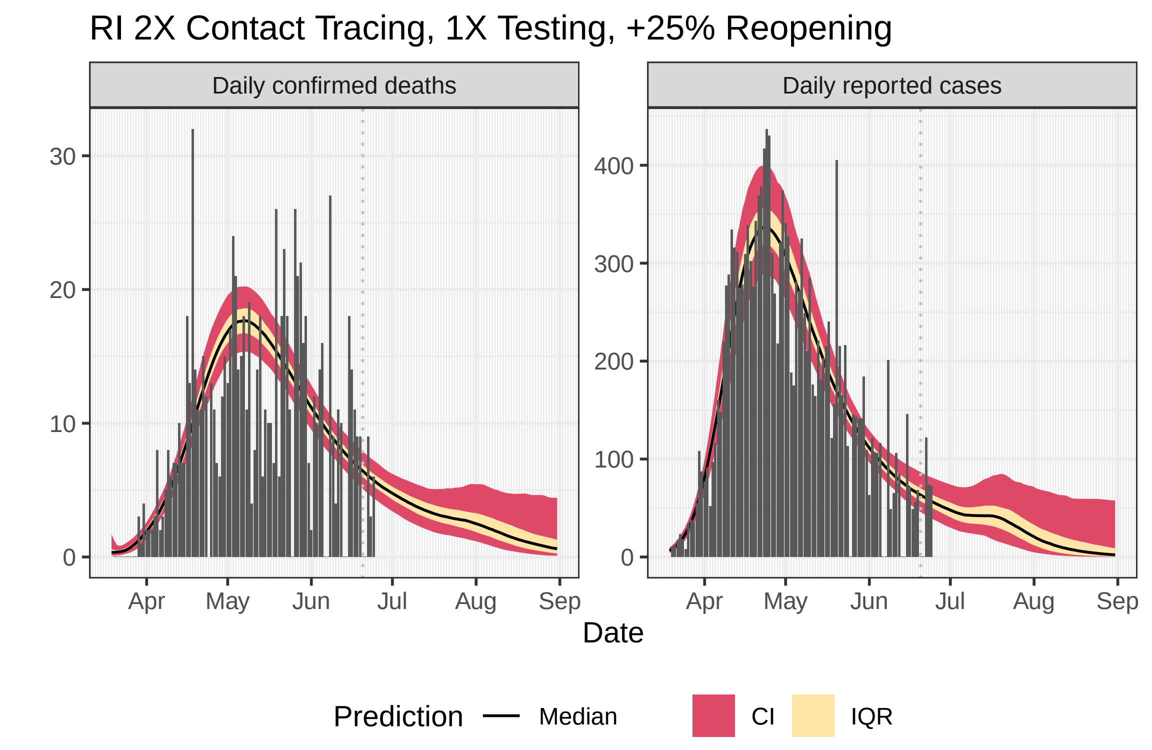
RI 2X Contact Tracing, 1X Testing, +25% Reopening
Daily confirmed deaths
Daily reported cases
30
20
10
0
400
300
200
100
0
Apr
May
Jun
Jul
Aug
Sep
Date
Prediction
Median
CI
IQR

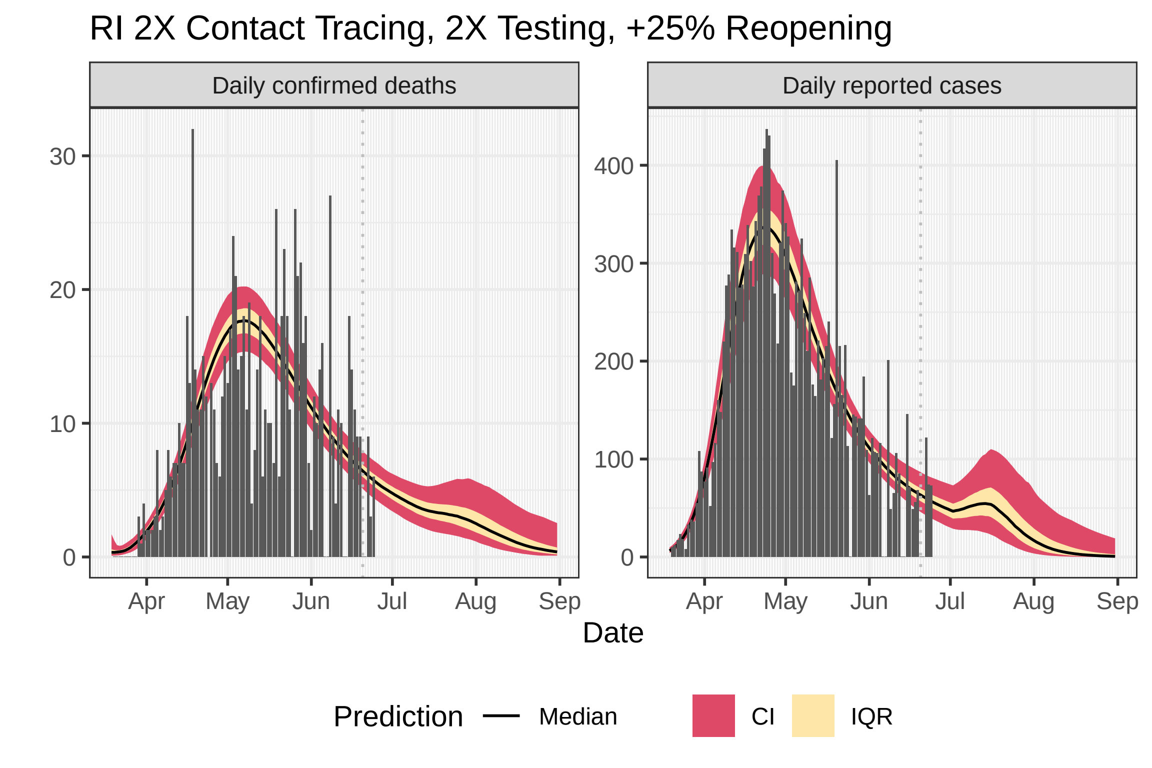

RI 2X Contact Tracing, 2X Testing, +25% Reopening
Daily confirmed deaths
Daily reported cases
30
20
10
0
400
300
200
100
0
Apr
May
Jun
Jul
Aug
Sep
Date
Prediction
Median
CI
IQR

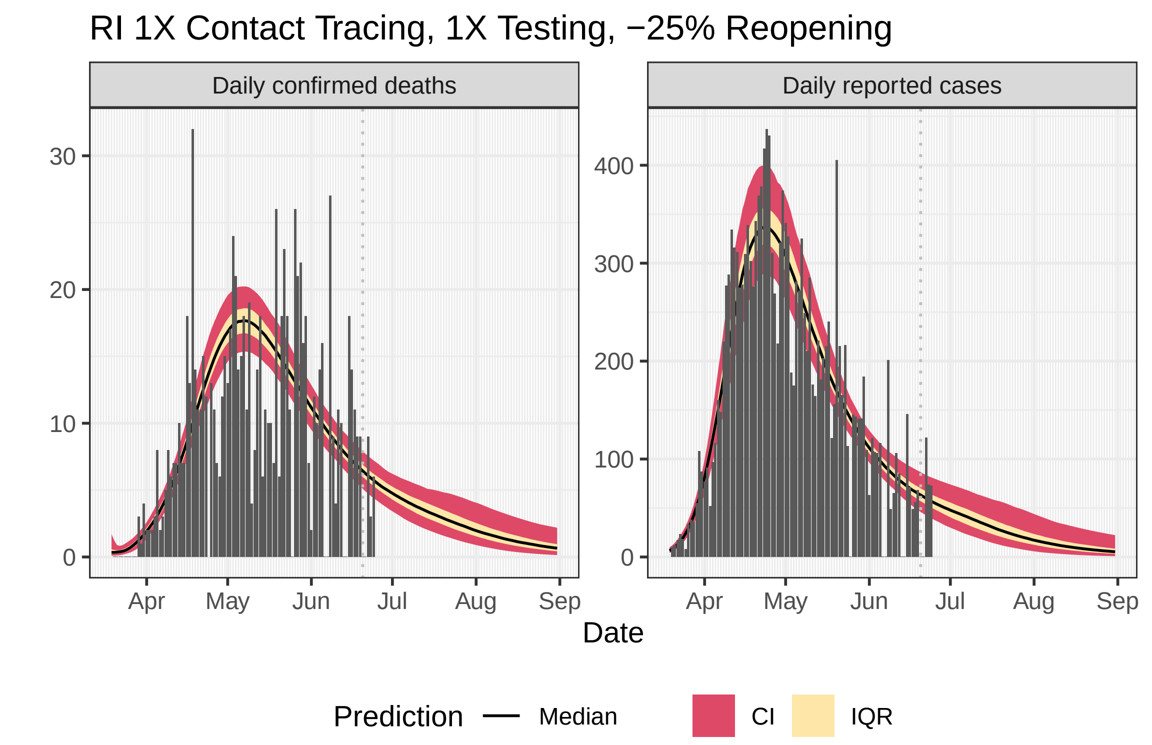
RI 1X Contact Tracing, 1X Testing, −25% Reopening
Daily confirmed deaths
Daily reported cases
0
10
20
30
0
100
200
300
400
Apr
May
Jun
Jul
Aug
Sep
Date
Prediction
Median
CI
IQR

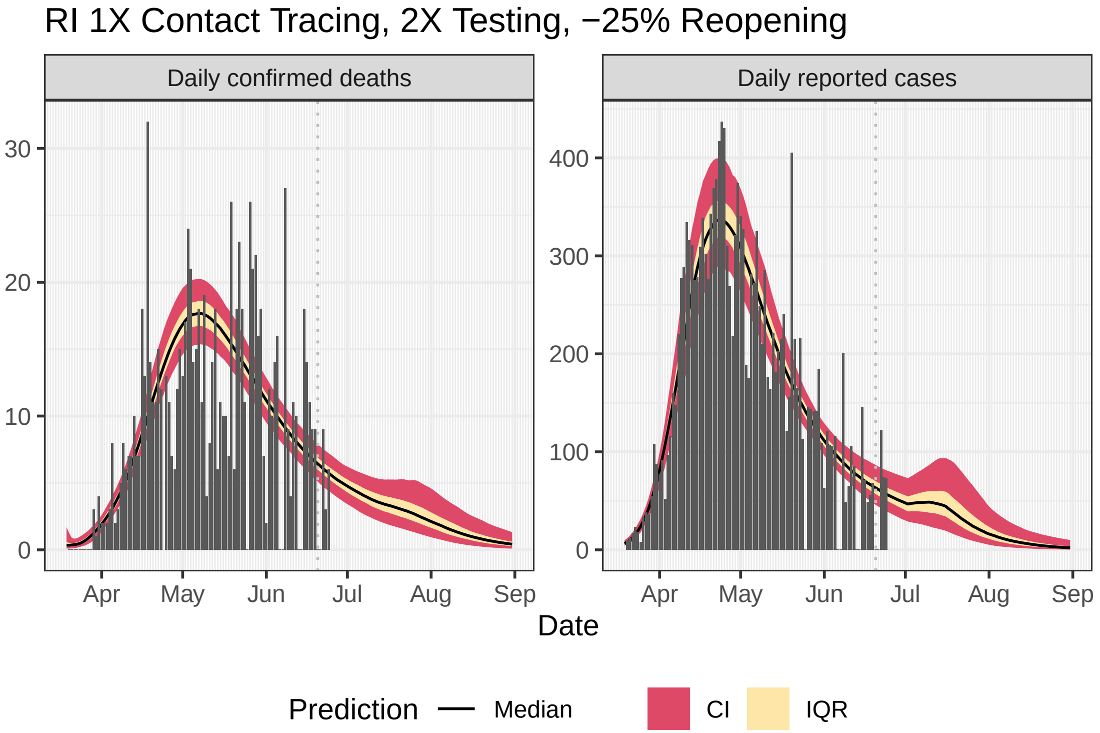
RI 1X Contact Tracing, 2X Testing, −25% Reopening
Daily confirmed deaths
Daily reported cases
0
10
20
30
0
100
200
300
400
Apr
May
Jun
Jul
Aug
Sep
Date
Prediction
Median
CI
IQR

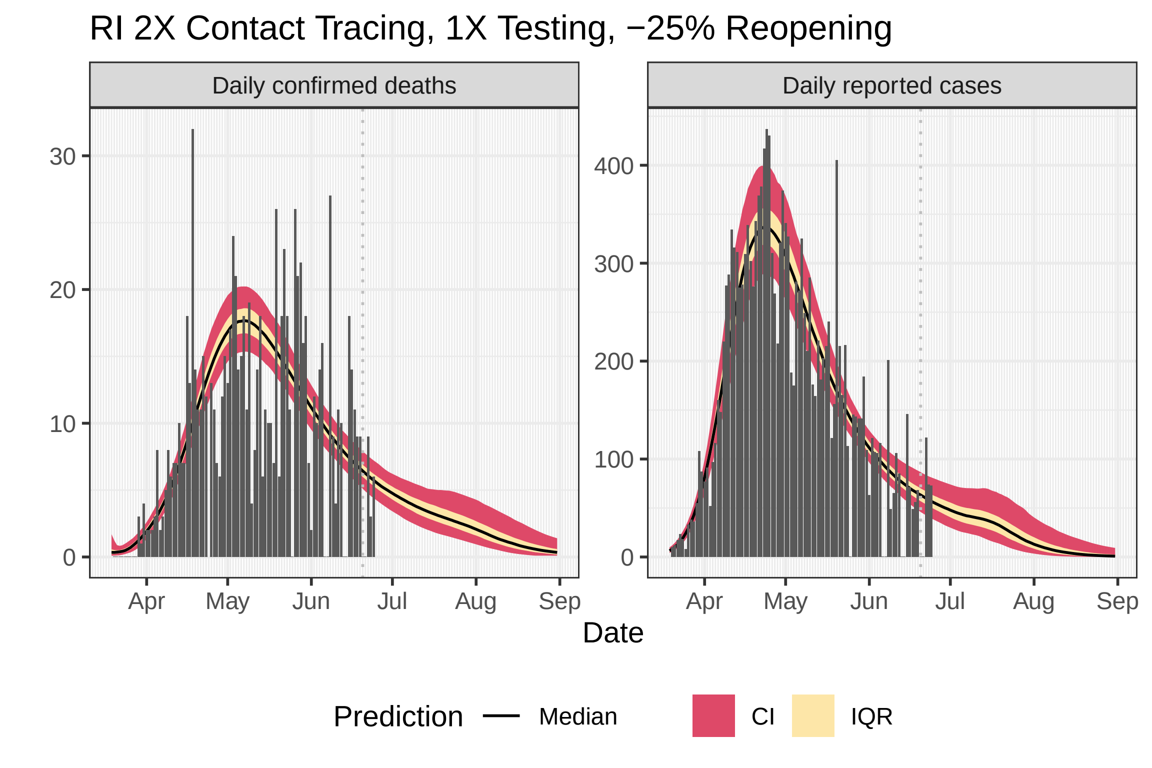

RI 2X Contact Tracing, 1X Testing, −25% Reopening
Daily confirmed deaths
Daily reported cases
0
10
20
30
0
100
200
300
400
Apr
May
Jun
Jul
Aug
Sep
Date
Prediction
Median
CI
IQR

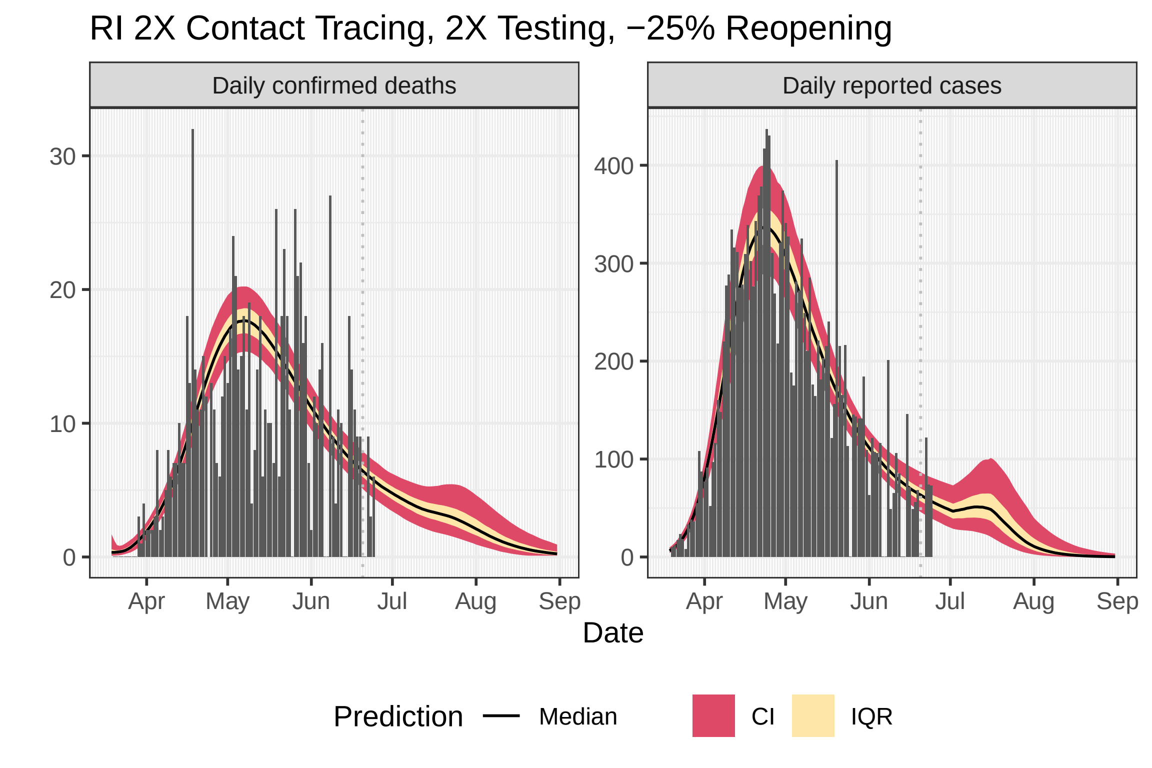

RI 2X Contact Tracing, 2X Testing, −25% Reopening
Daily confirmed deaths
Daily reported cases
30
20
10
0
400
300
200
100
0
Apr
May
Jun
Jul
Aug
Sep
Date
Prediction
Median
CI
IQR

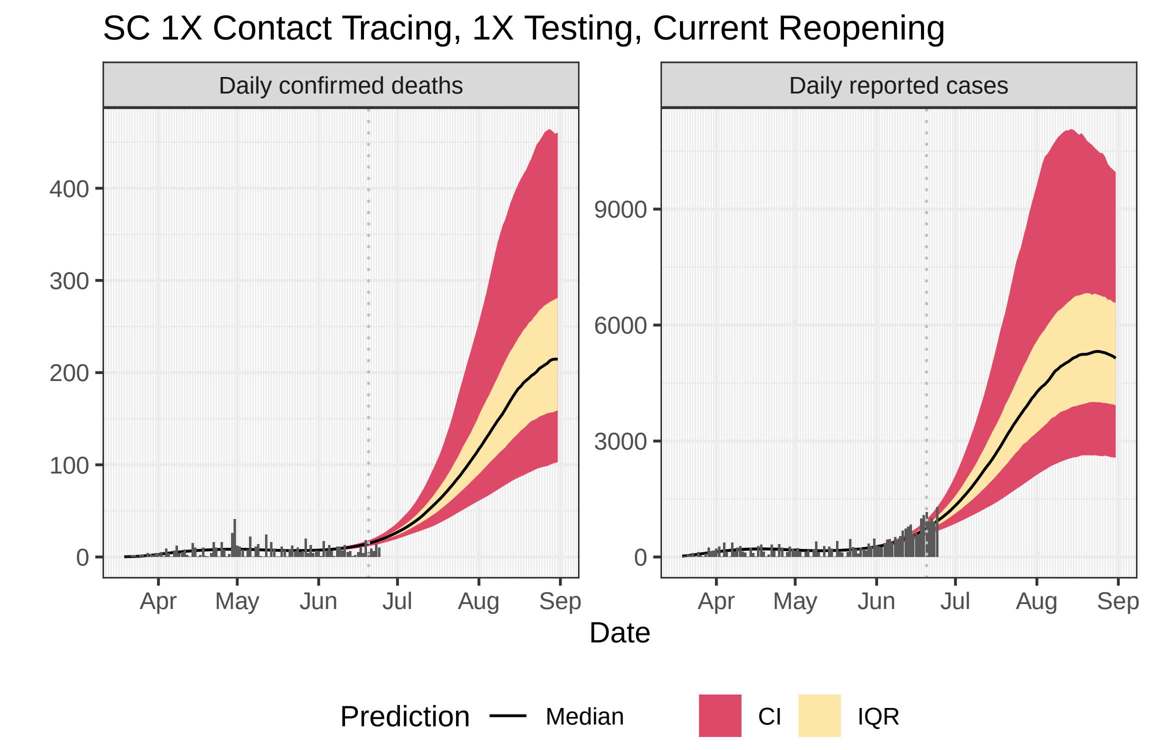
SC 1X Contact Tracing, 1X Testing, Current Reopening
Daily confirmed deaths
Daily reported cases
400
300
200
100
0
9000
6000
3000
0
Apr
May
Jun
Jul
Aug
Sep
Date
Prediction
Median
CI
IQR

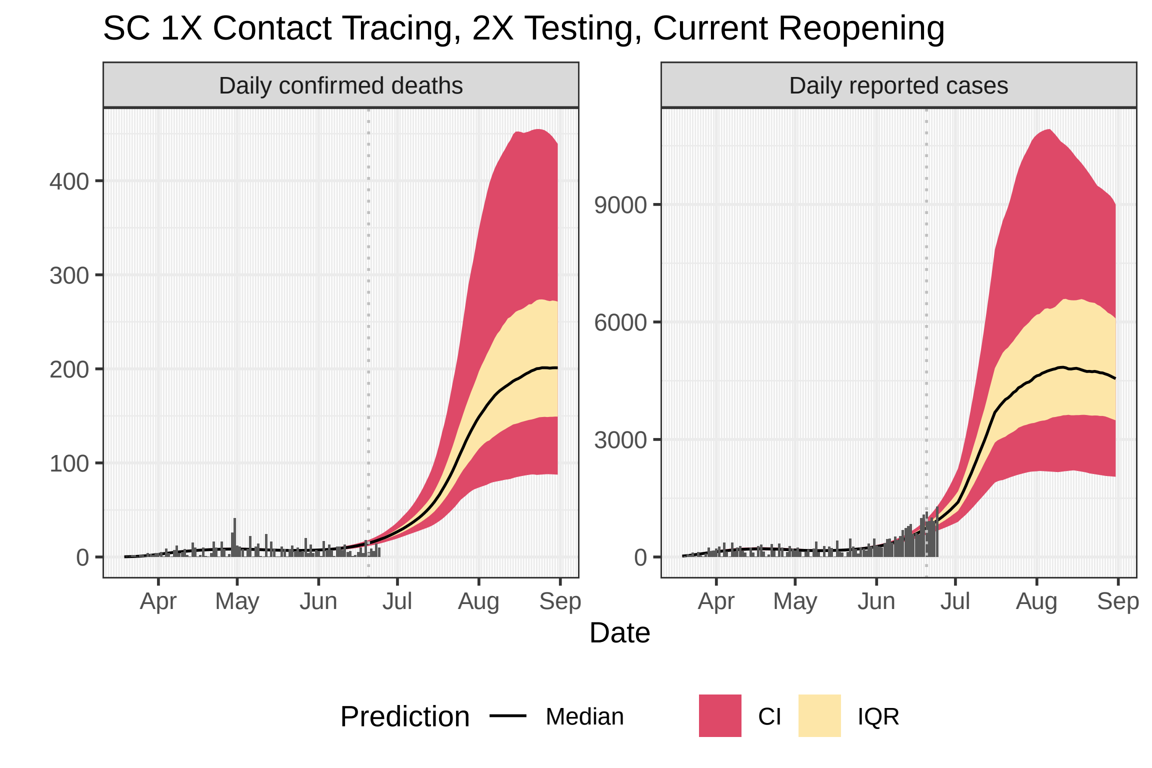
SC 1X Contact Tracing, 2X Testing, Current Reopening
Daily confirmed deaths
Daily reported cases
400
300
200
100
0
9000
6000
3000
0
Apr
May
Jun
Jul
Aug
Sep
Date
Prediction
Median
CI
IQR

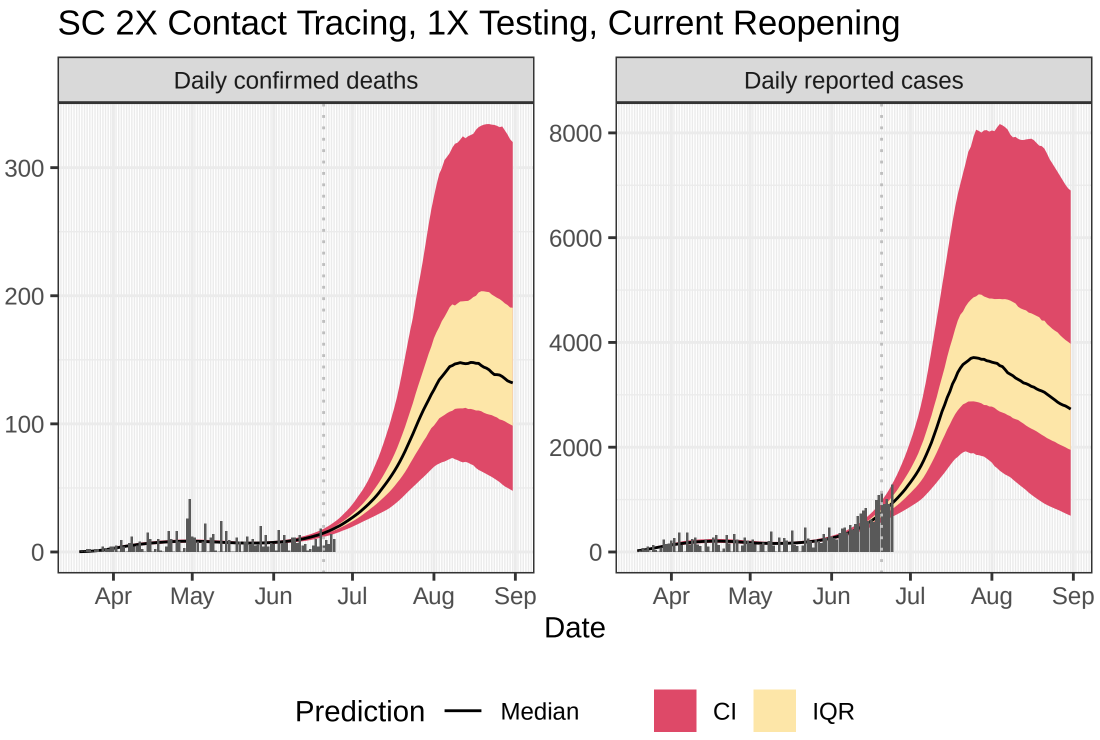
SC 2X Contact Tracing, 1X Testing, Current Reopening
Daily confirmed deaths
Daily reported cases
300
200
100
0
8000
6000
4000
2000
0
Apr
May
Jun
Jul
Aug
Sep
Date
Prediction
Median
CI
IQR

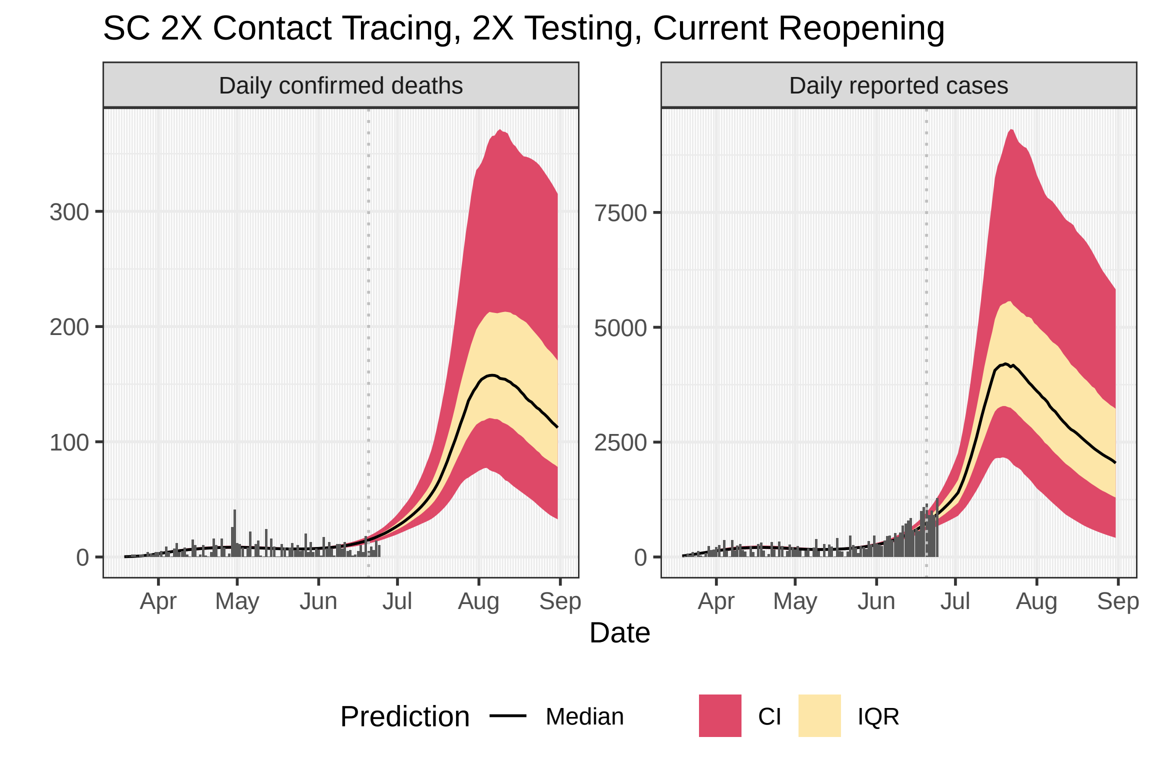
SC 2X Contact Tracing, 2X Testing, Current Reopening
Daily confirmed deaths
Daily reported cases
300
200
100
0
7500
5000
2500
0
Apr
May
Jun
Jul
Aug
Sep
Date
Prediction
Median
CI
IQR

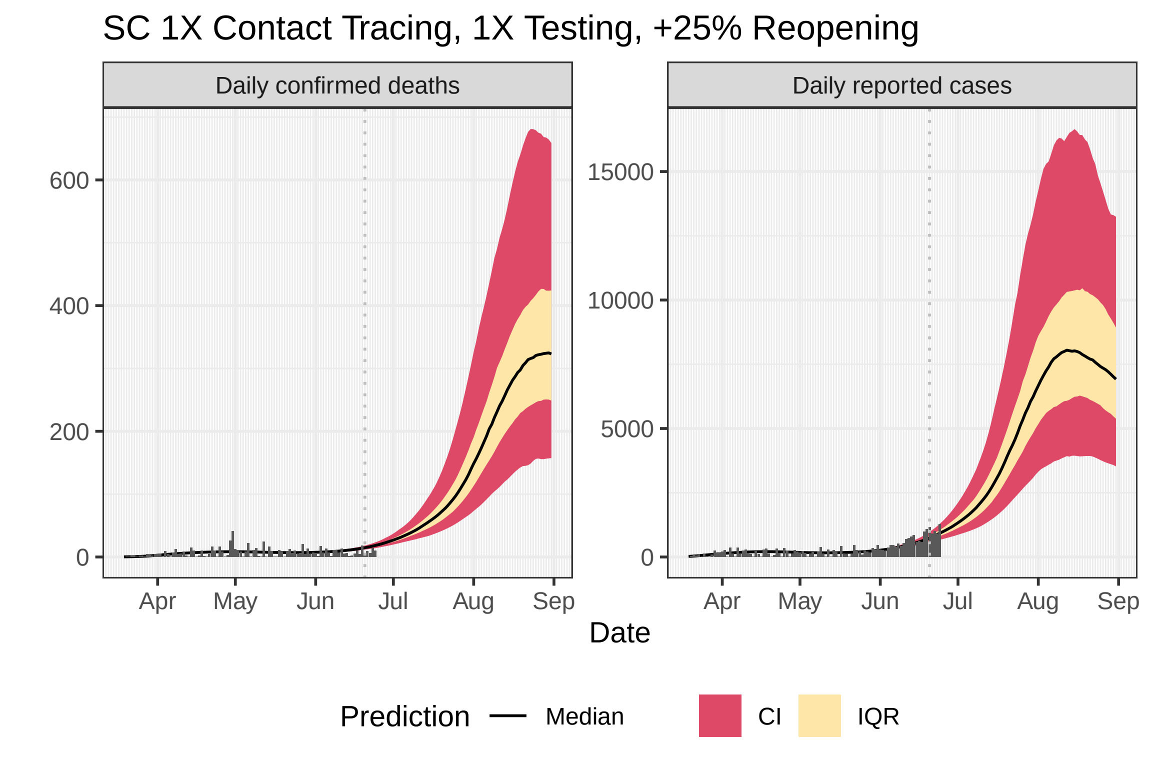

SC 1X Contact Tracing, 1X Testing, +25% Reopening
Daily confirmed deaths
Daily reported cases
0
200
400
600
0
5000
10000
15000
Apr
May
Jun
Jul
Aug
Sep
Date
Prediction
Median
CI
IQR

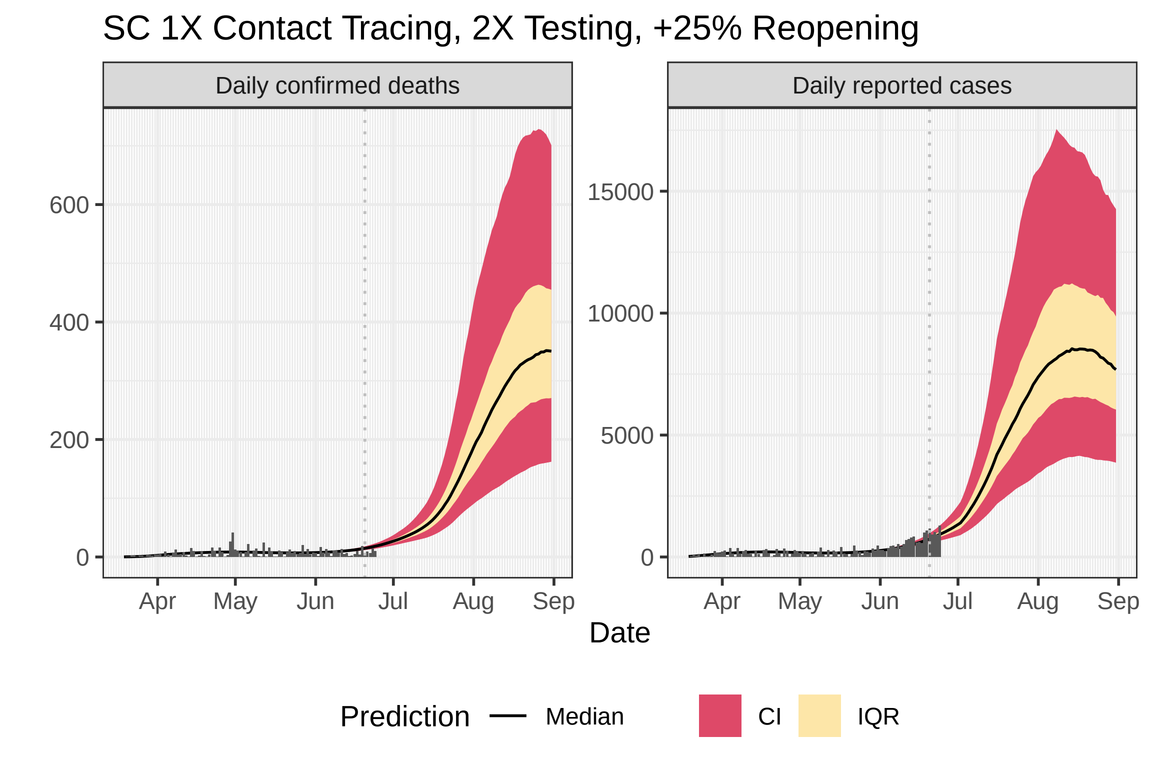

SC 1X Contact Tracing, 2X Testing, +25% Reopening
Daily confirmed deaths
Daily reported cases
600
400
200
0
15000
10000
5000
0
Apr
May
Jun
Jul
Aug
Sep
Date
Prediction
Median
CI
IQR

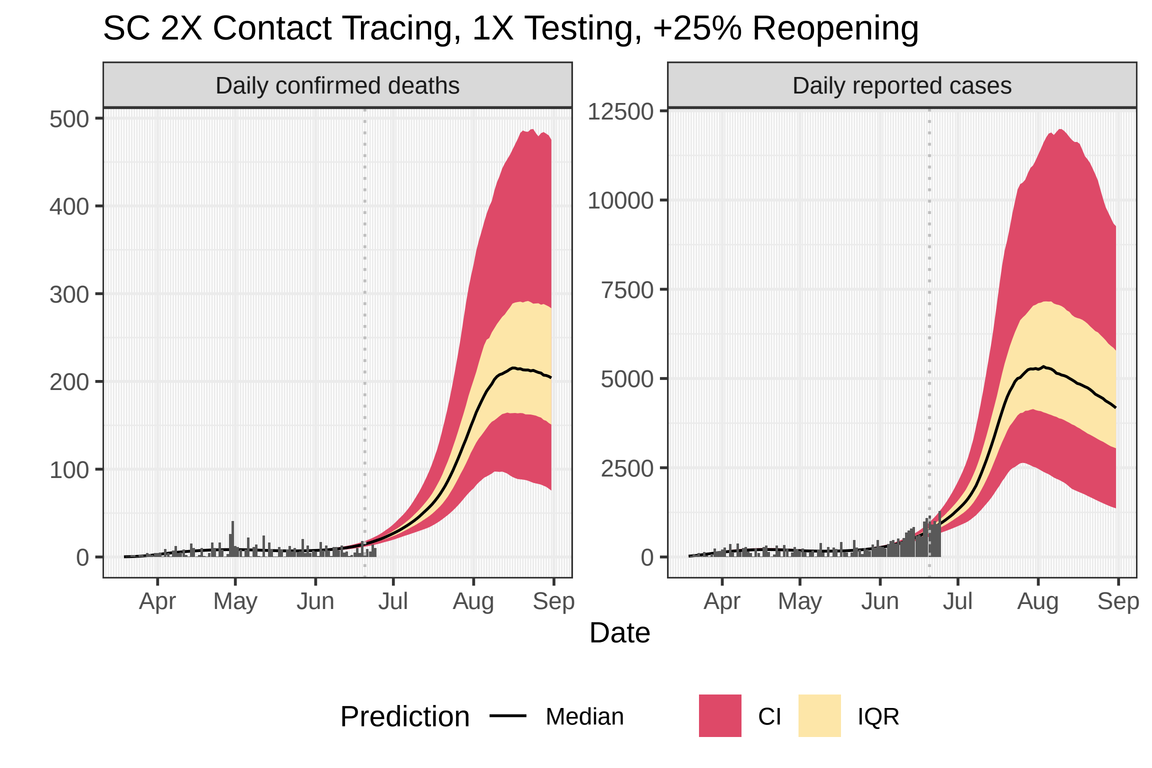

SC 2X Contact Tracing, 1X Testing, +25% Reopening
Daily confirmed deaths
Daily reported cases
500
400
300
200
100
0
12500
10000
7500
5000
2500
0
Apr
May
Jun
Jul
Aug
Sep
Date
Prediction
Median
CI
IQR

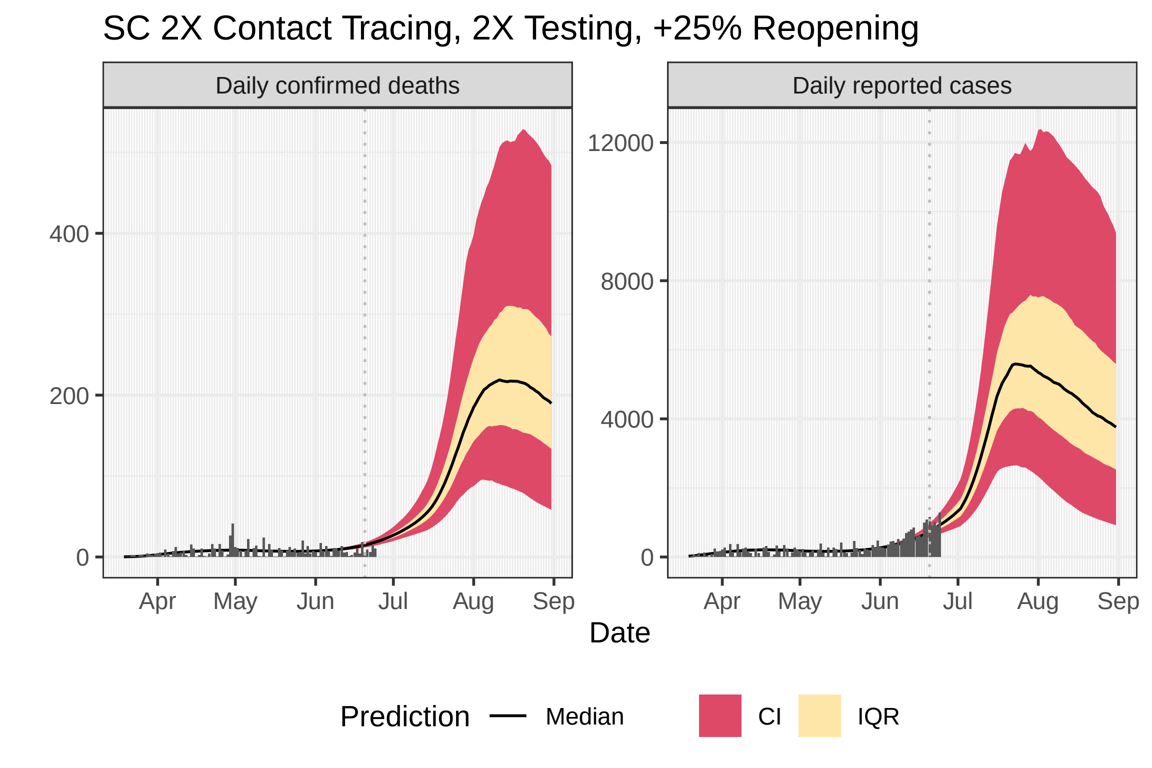

SC 2X Contact Tracing, 2X Testing, +25% Reopening
Daily confirmed deaths
Daily reported cases
0
200
400
0
4000
8000
12000
Apr
May
Jun
Jul
Aug
Sep
Date
Prediction
Median
CI
IQR

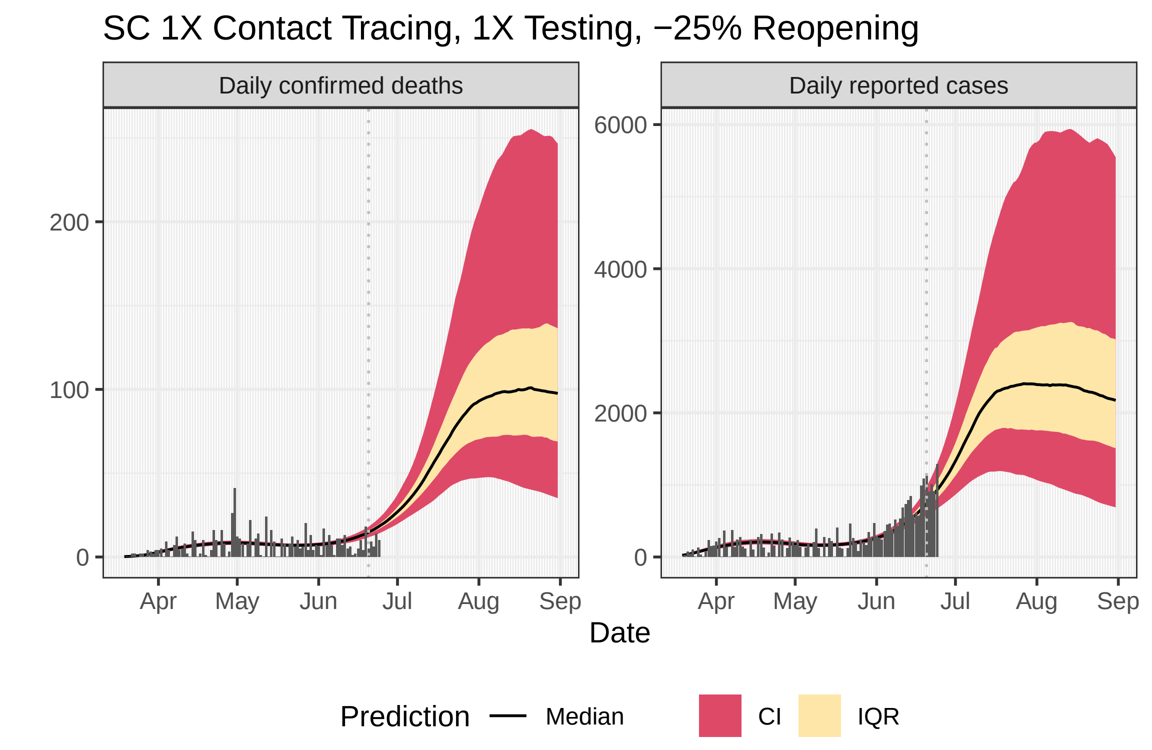
SC 1X Contact Tracing, 1X Testing, −25% Reopening
Daily confirmed deaths
Daily reported cases
0
100
200
0
2000
4000
6000
Apr
May
Jun
Jul
Aug
Sep
Date
Prediction
Median
CI
IQR

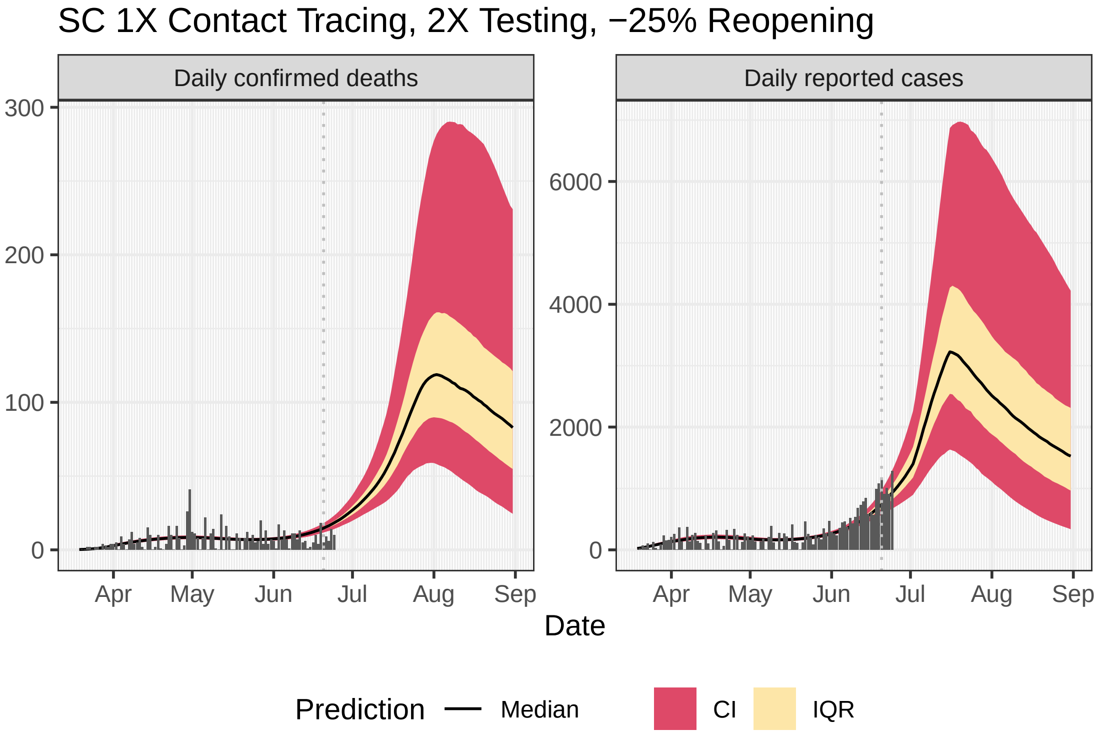

SC 1X Contact Tracing, 2X Testing, −25% Reopening
Daily confirmed deaths
Daily reported cases
300
200
100
0
6000
4000
2000
0
Apr
May
Jun
Jul
Aug
Sep
Date
Prediction
Median
CI
IQR

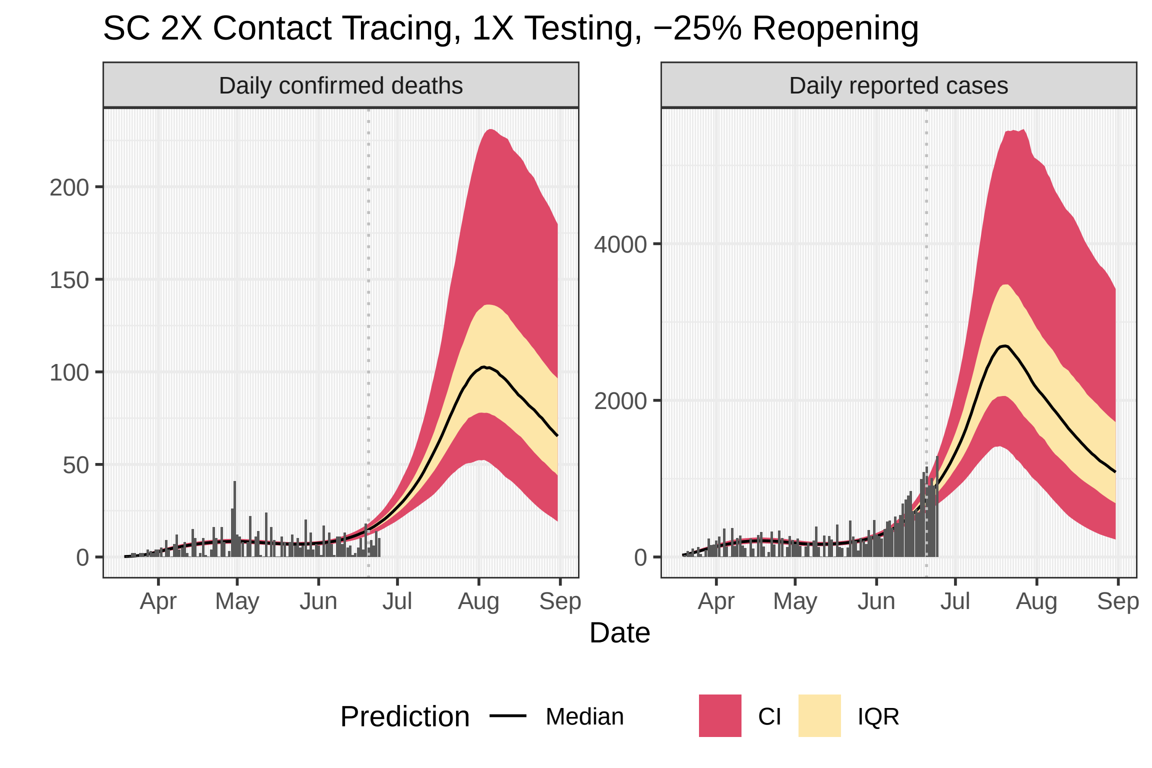
SC 2X Contact Tracing, 1X Testing, −25% Reopening
Daily confirmed deaths
Daily reported cases
0
50
100
150
200
0
2000
4000
Apr
May
Jun
Jul
Aug
Sep
Date
Prediction
Median
CI
IQR

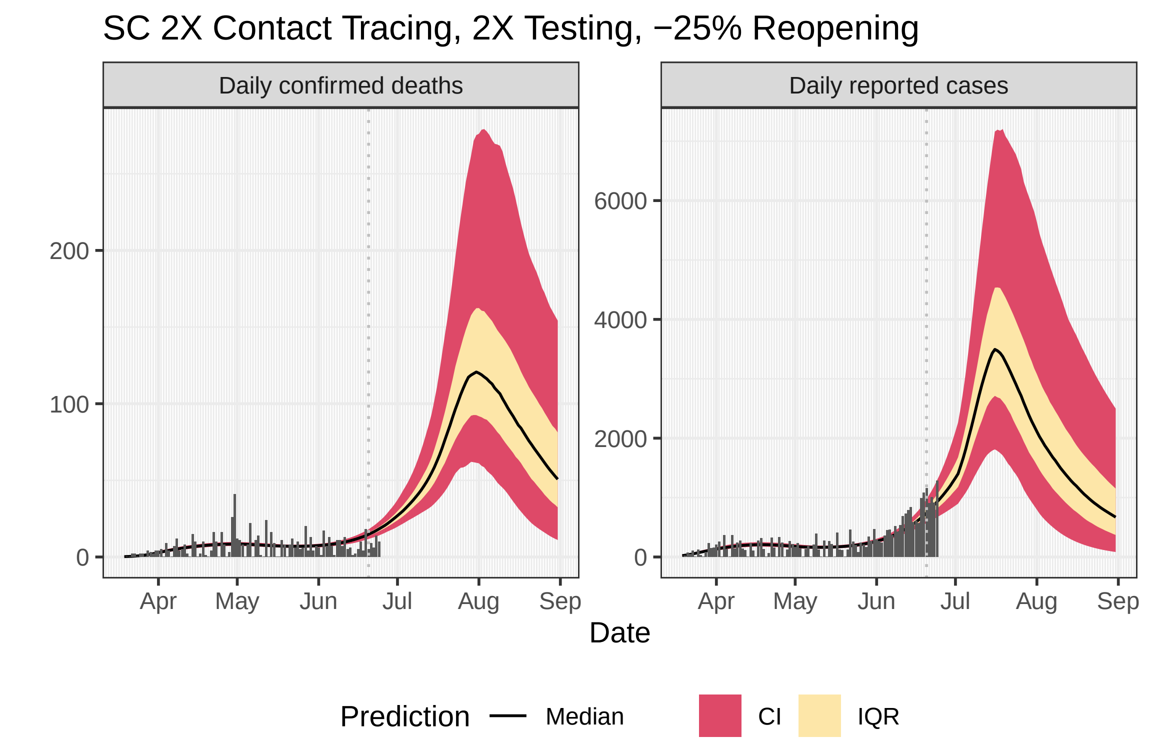

SC 2X Contact Tracing, 2X Testing, −25% Reopening
Daily confirmed deaths
Daily reported cases
200
100
0
6000
4000
2000
0
Apr
May
Jun
Jul
Aug
Sep
Date
Prediction
Median
CI
IQR

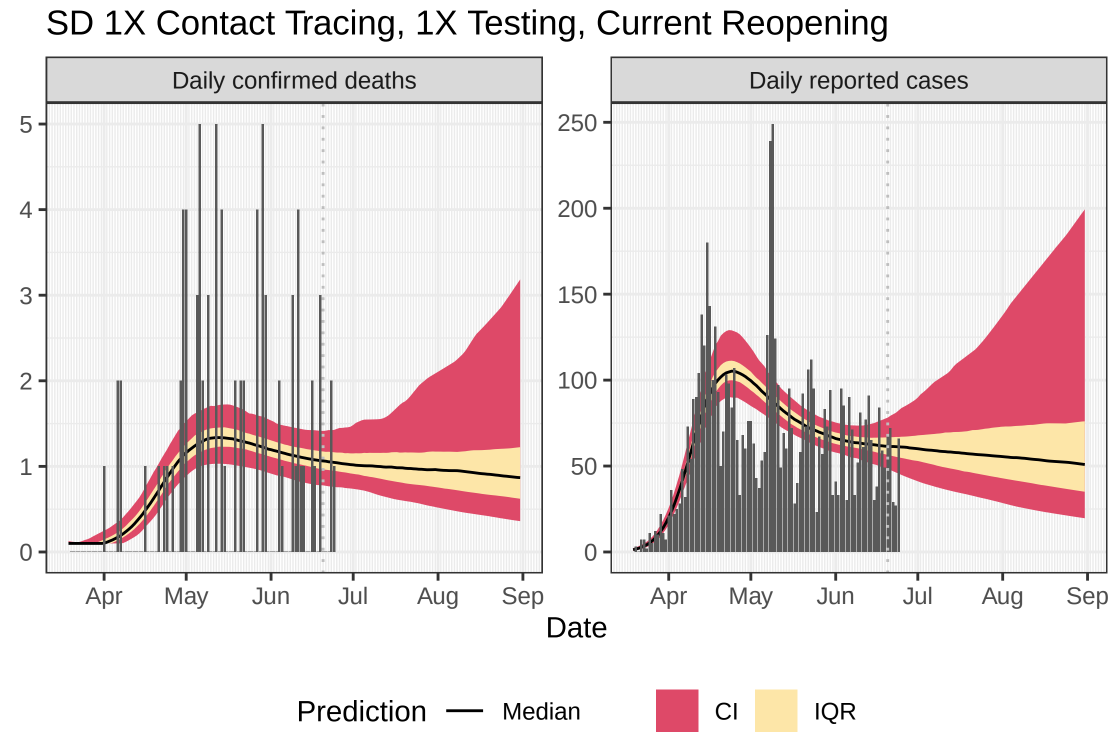

SD 1X Contact Tracing, 1X Testing, Current Reopening
Daily confirmed deaths
Daily reported cases
5
4
3
2
1
0
250
200
150
100
50
0
Apr
May
Jun
Jul
Aug
Sep
Date
Prediction
Median
CI
IQR

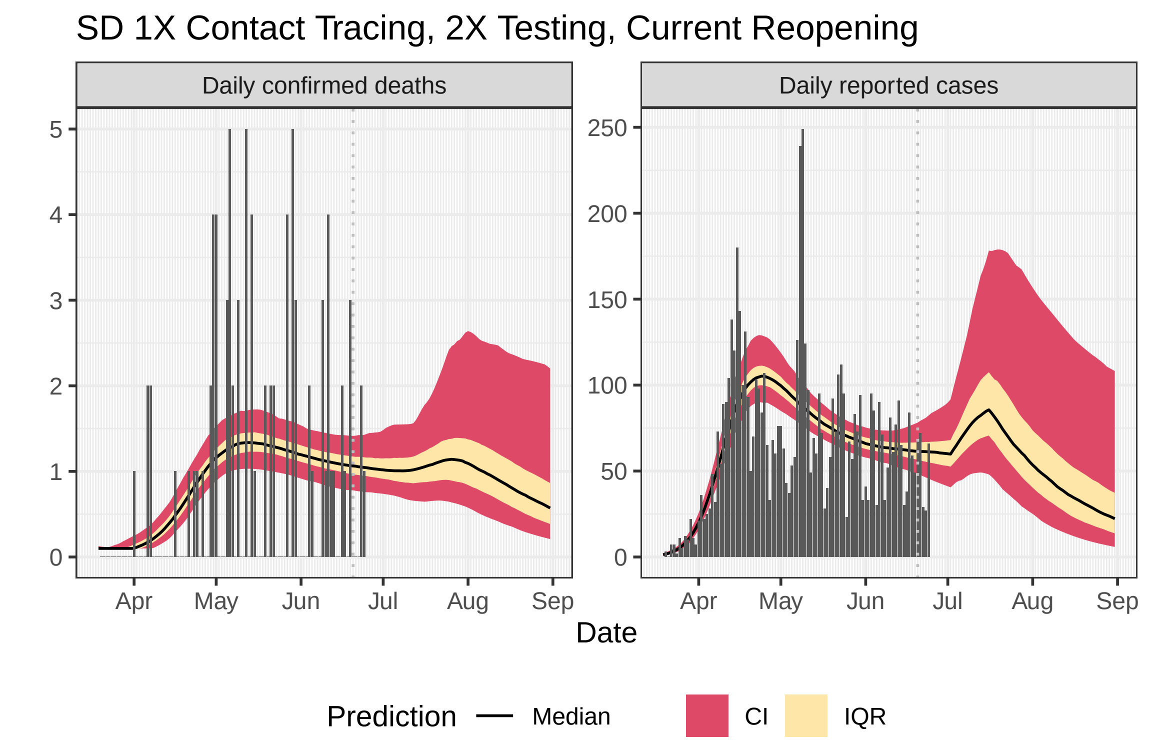
SD 1X Contact Tracing, 2X Testing, Current Reopening
Daily confirmed deaths
Daily reported cases
0
1
2
3
4
5
0
50
100
150
200
250
Apr
May
Jun
Jul
Aug
Sep
Date
Prediction
Median
CI
IQR

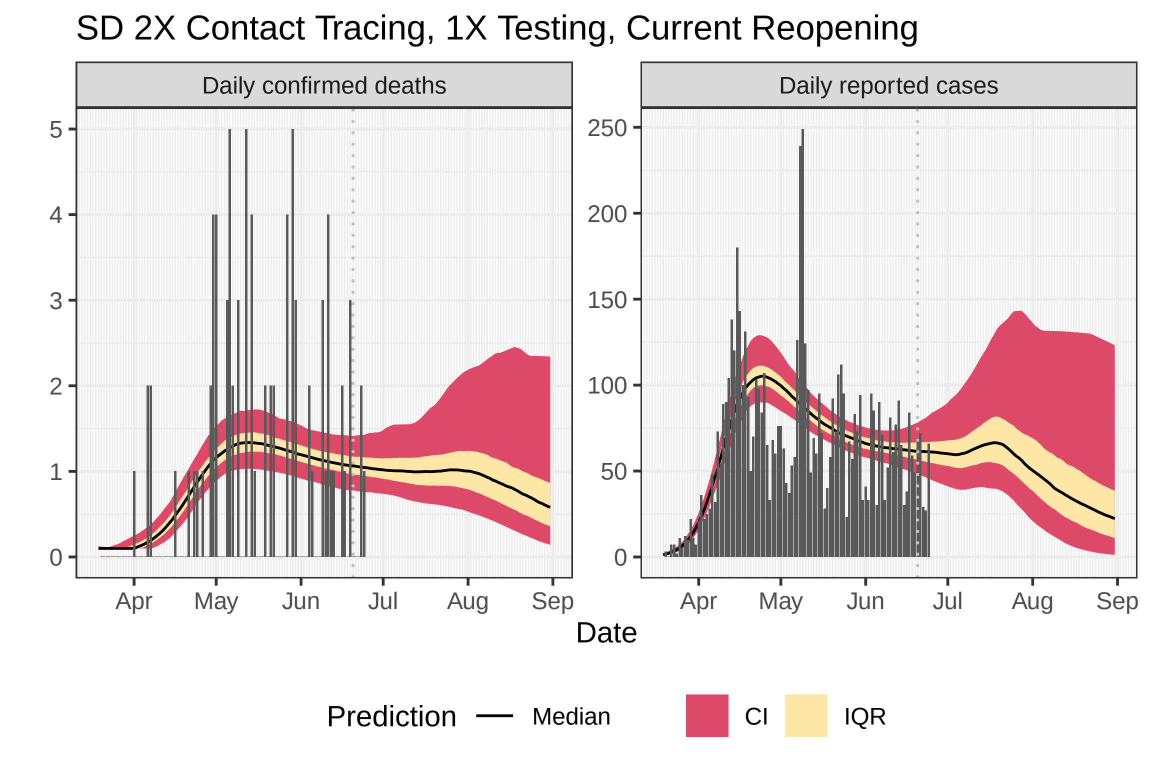
SD 2X Contact Tracing, 1X Testing, Current Reopening
Daily confirmed deaths
Daily reported cases
0
1
2
3
4
5
0
50
100
150
200
250
Apr
May
Jun
Jul
Aug
Sep
Date
Prediction
Median
CI
IQR

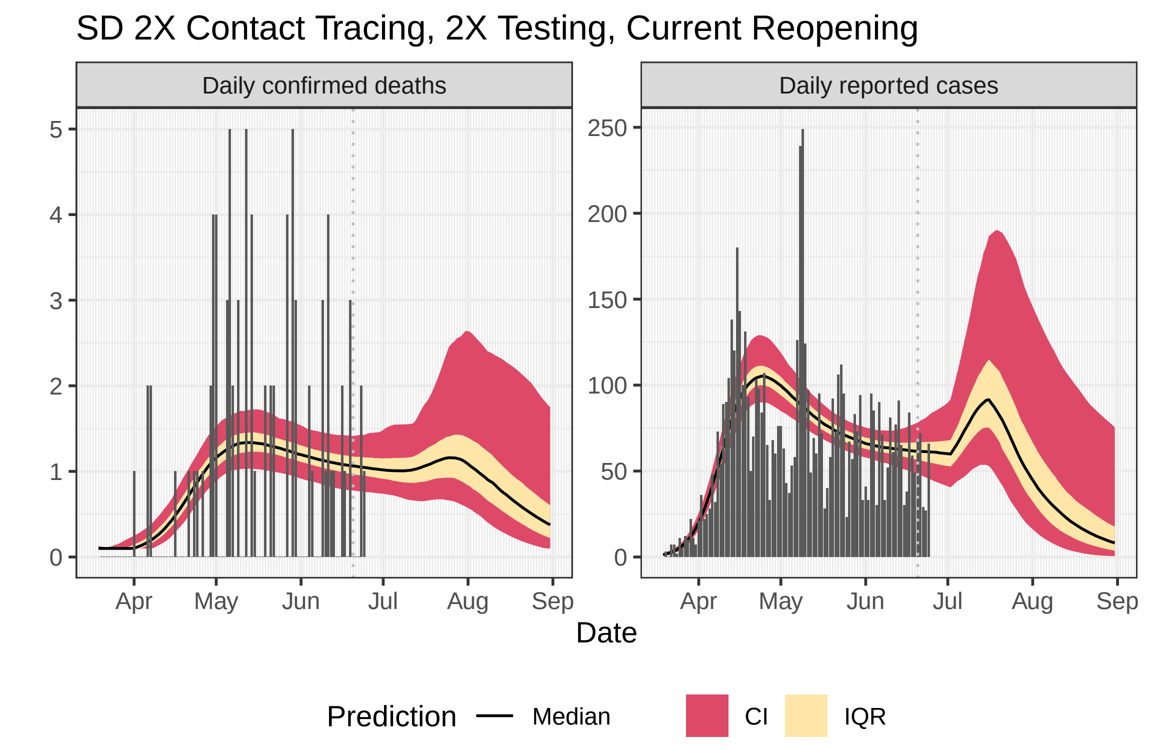
SD 2X Contact Tracing, 2X Testing, Current Reopening
Daily confirmed deaths
Daily reported cases
0
1
2
3
4
5
0
50
100
150
200
250
Apr
May
Jun
Jul
Aug
Sep
Date
Prediction
Median
CI
IQR

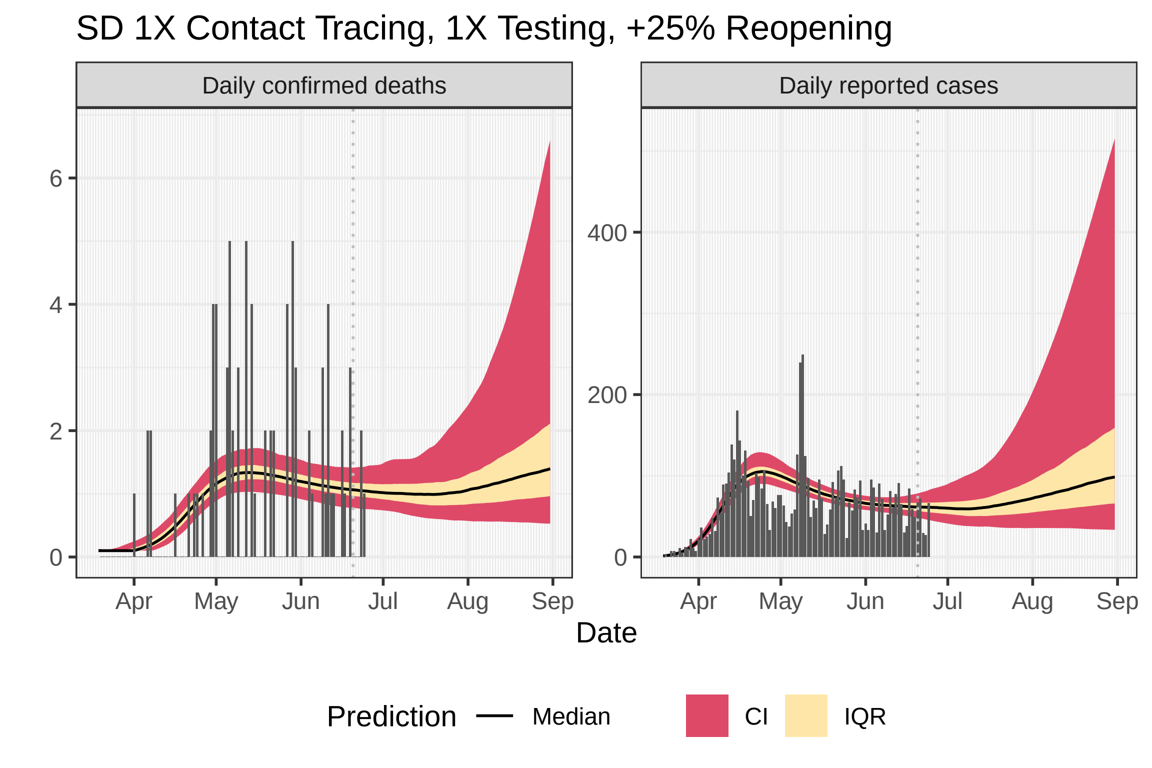
SD 1X Contact Tracing, 1X Testing, +25% Reopening
Daily confirmed deaths
Daily reported cases
0
2
4
6
0
200
400
Apr
May
Jun
Jul
Aug
Sep
Date
Prediction
Median
CI
IQR

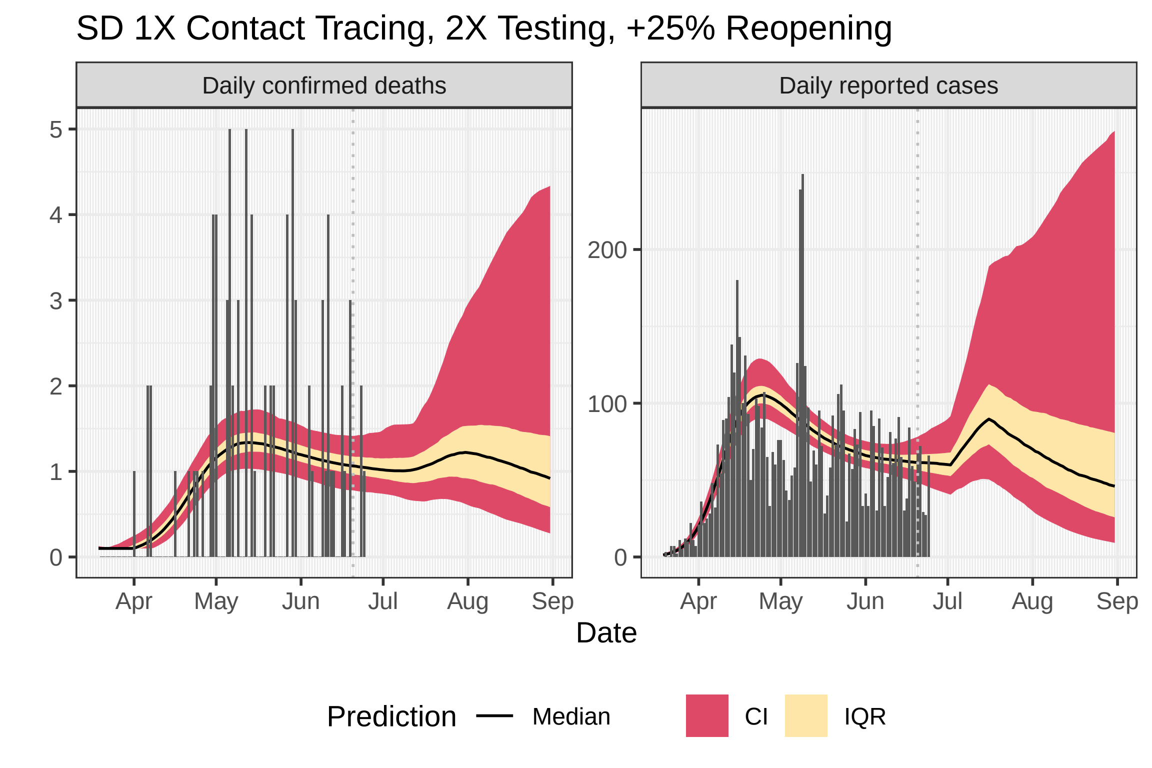

SD 1X Contact Tracing, 2X Testing, +25% Reopening
Daily confirmed deaths
Daily reported cases
5
4
3
2
1
0
200
100
0
Apr
May
Jun
Jul
Aug
Sep
Date
Prediction
Median
CI
IQR

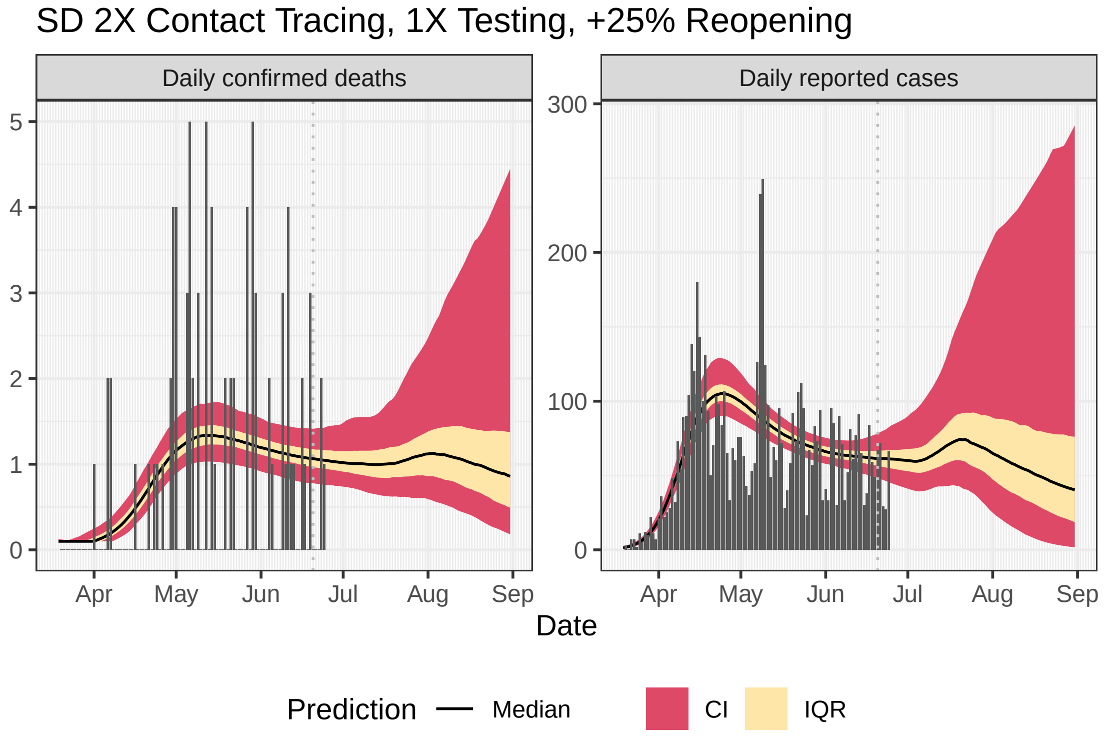
SD 2X Contact Tracing, 1X Testing, +25% Reopening
Daily confirmed deaths
Daily reported cases
0
1
2
3
4
5
0
100
200
300
Apr
May
Jun
Jul
Aug
Sep
Date
Prediction
Median
CI
IQR

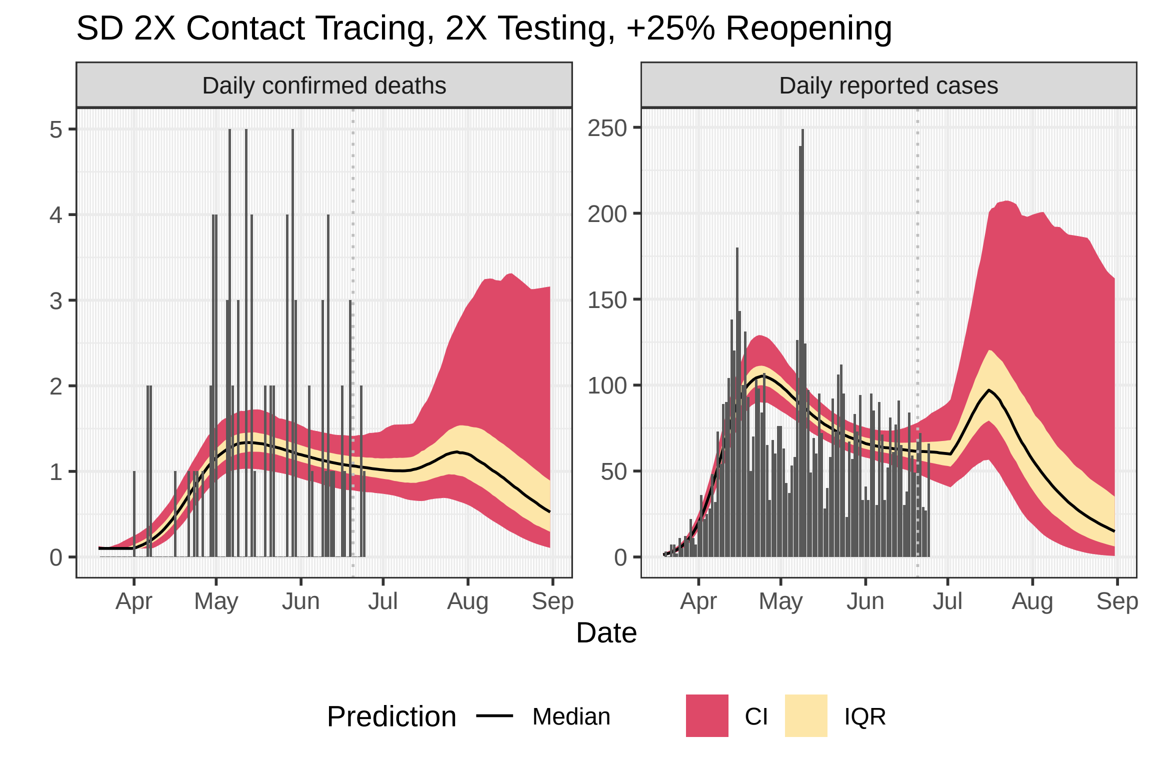

SD 2X Contact Tracing, 2X Testing, +25% Reopening
Daily confirmed deaths
Daily reported cases
0
1
2
3
4
5
0
50
100
150
200
250
Apr
May
Jun
Jul
Aug
Sep
Date
Prediction
Median
CI
IQR

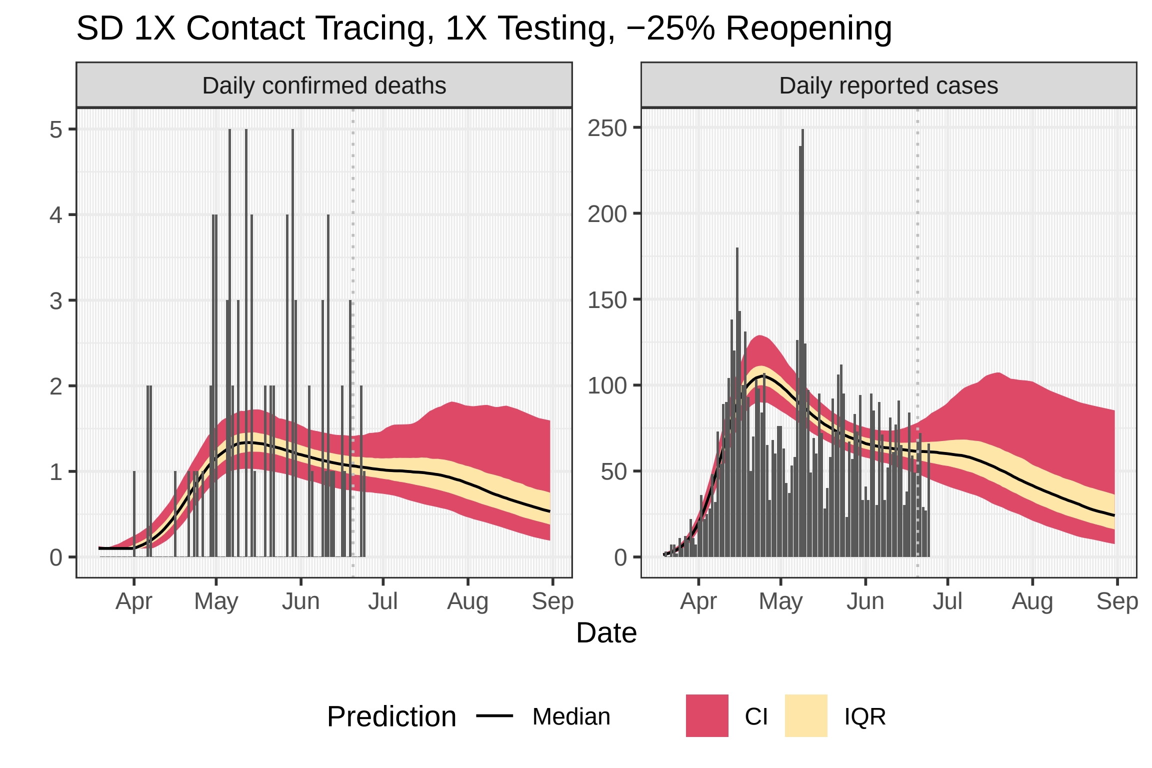
SD 1X Contact Tracing, 1X Testing, −25% Reopening
Daily confirmed deaths
Daily reported cases
0
1
2
3
4
5
0
50
100
150
200
250
Apr
May
Jun
Jul
Aug
Sep
Date
Prediction
Median
CI
IQR

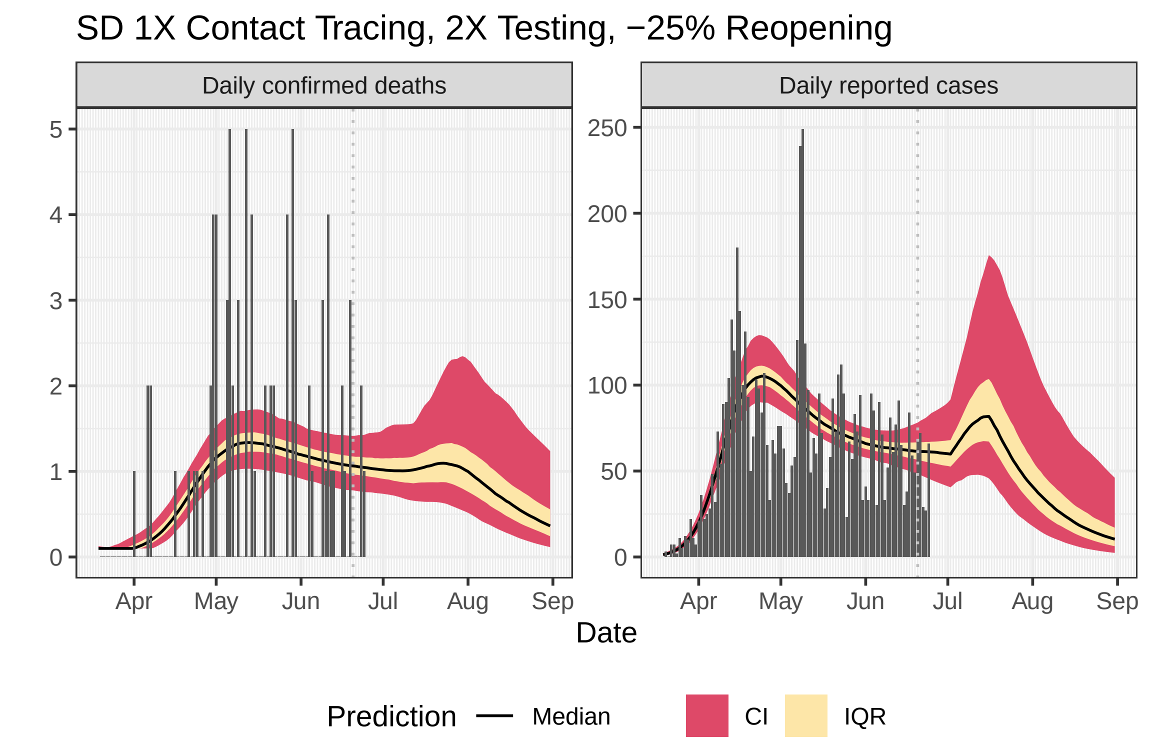
SD 1X Contact Tracing, 2X Testing, −25% Reopening
Daily confirmed deaths
Daily reported cases
0
1
2
3
4
5
0
50
100
150
200
250
Apr
May
Jun
Jul
Aug
Sep
Date
Prediction
Median
CI
IQR

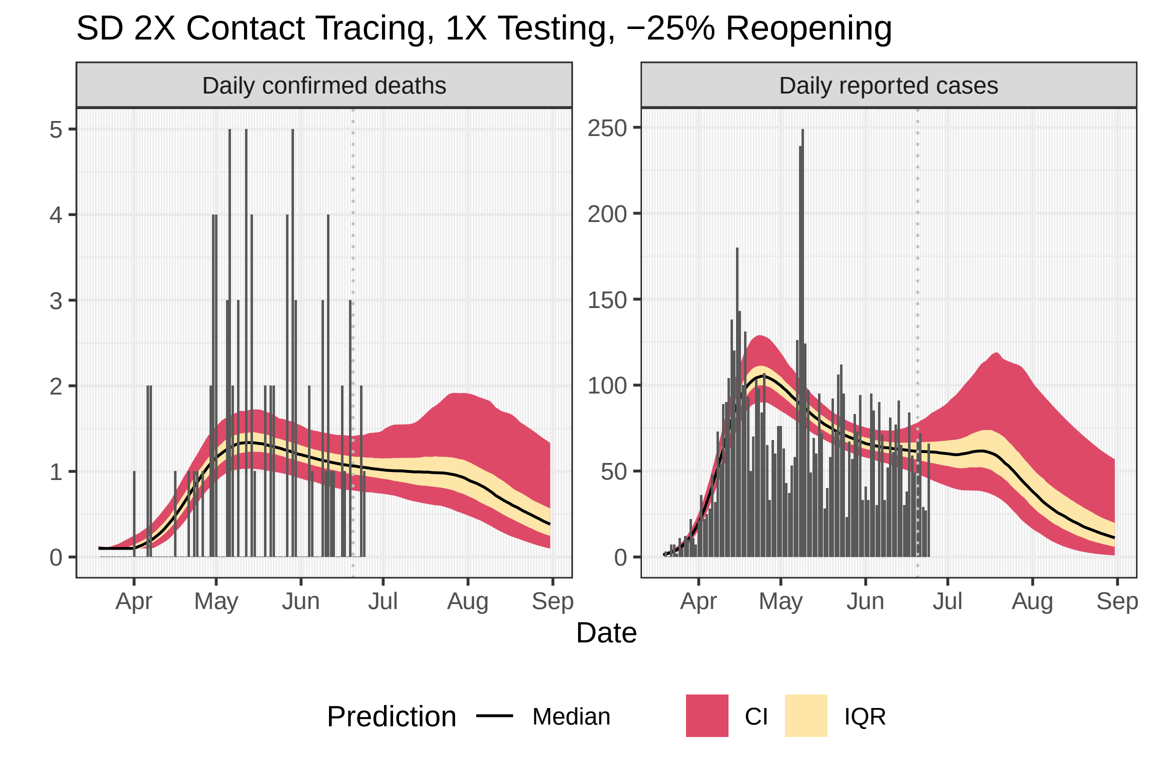

SD 2X Contact Tracing, 1X Testing, −25% Reopening
Daily confirmed deaths
Daily reported cases
0
1
2
3
4
5
0
50
100
150
200
250
Apr
May
Jun
Jul
Aug
Sep
Date
Prediction
Median
CI
IQR

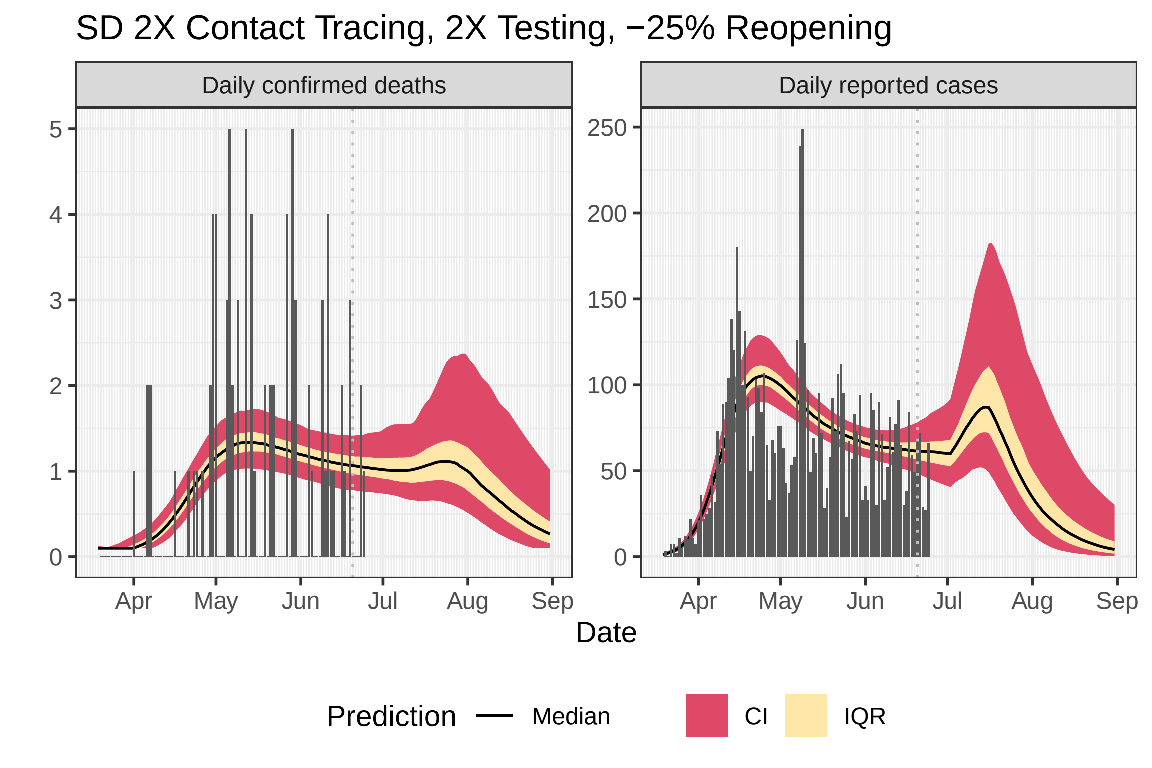

SD 2X Contact Tracing, 2X Testing, −25% Reopening
Daily confirmed deaths
Daily reported cases
0
1
2
3
4
5
0
50
100
150
200
250
Apr
May
Jun
Jul
Aug
Sep
Date
Prediction
Median
CI
IQR

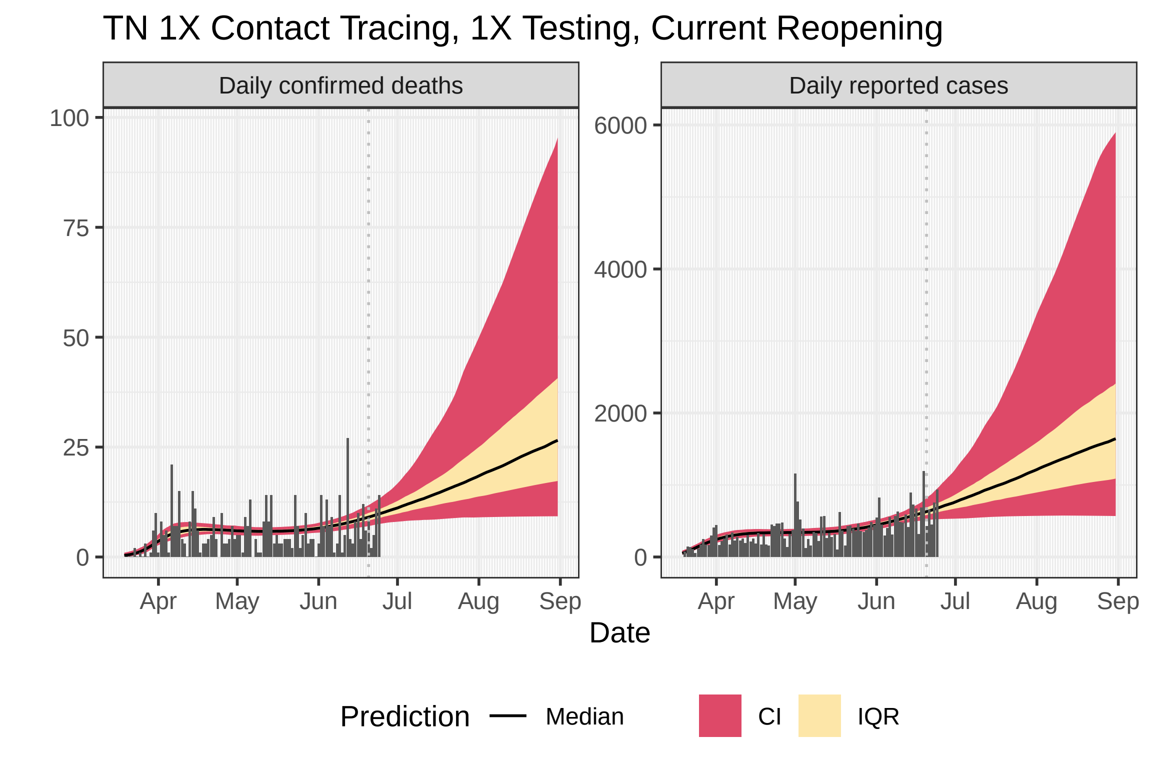

TN 1X Contact Tracing, 1X Testing, Current Reopening
Daily confirmed deaths
Daily reported cases
100
75
50
25
0
6000
4000
2000
0
Apr
May
Jun
Jul
Aug
Sep
Date
Prediction
Median
CI
IQR

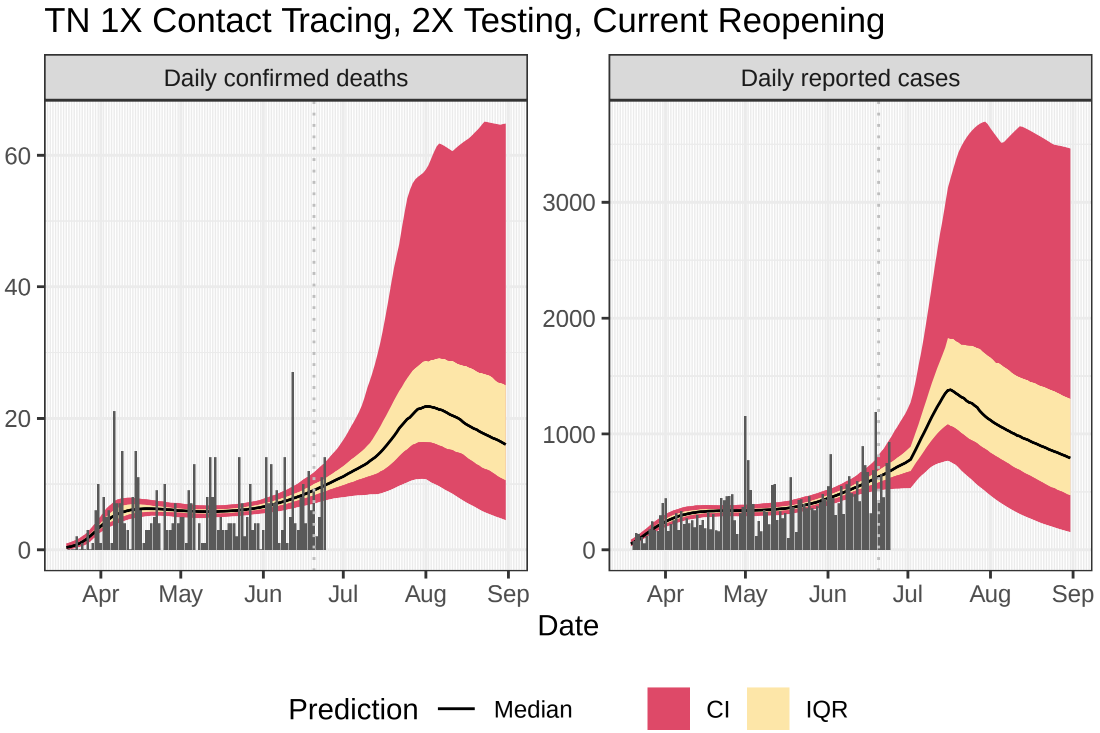

TN 1X Contact Tracing, 2X Testing, Current Reopening
Daily confirmed deaths
Daily reported cases
0
20
40
60
0
1000
2000
3000
Apr
May
Jun
Jul
Aug
Sep
Date
Prediction
Median
CI
IQR

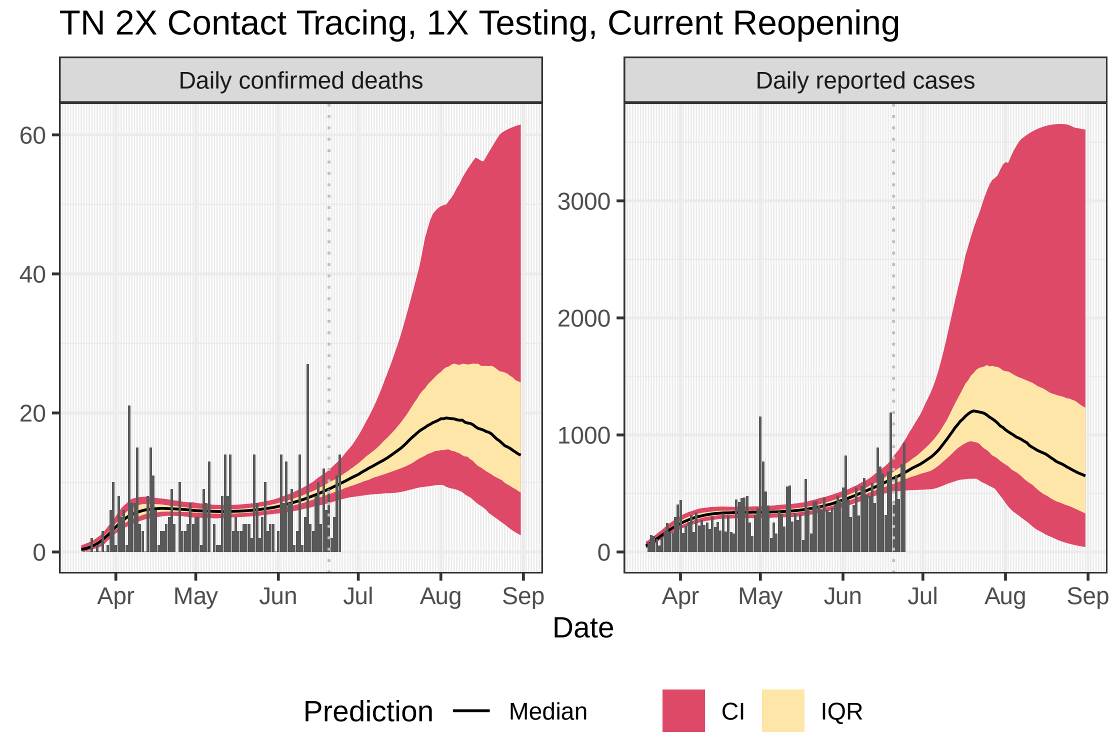
TN 2X Contact Tracing, 1X Testing, Current Reopening
Daily confirmed deaths
Daily reported cases
60
40
20
0
3000
2000
1000
0
Apr
May
Jun
Jul
Aug
Sep
Date
Prediction
Median
CI
IQR

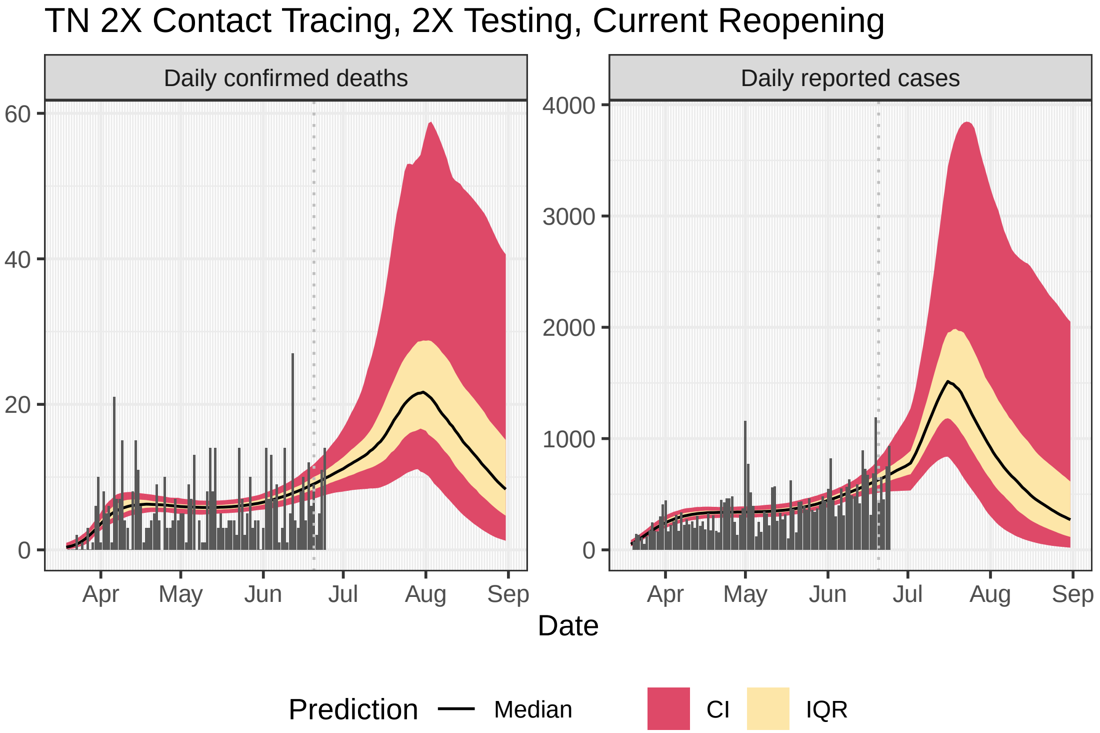
TN 2X Contact Tracing, 2X Testing, Current Reopening
Daily confirmed deaths
Daily reported cases
60
40
20
0
4000
3000
2000
1000
0
Apr
May
Jun
Jul
Aug
Sep
Date
Prediction
Median
CI
IQR

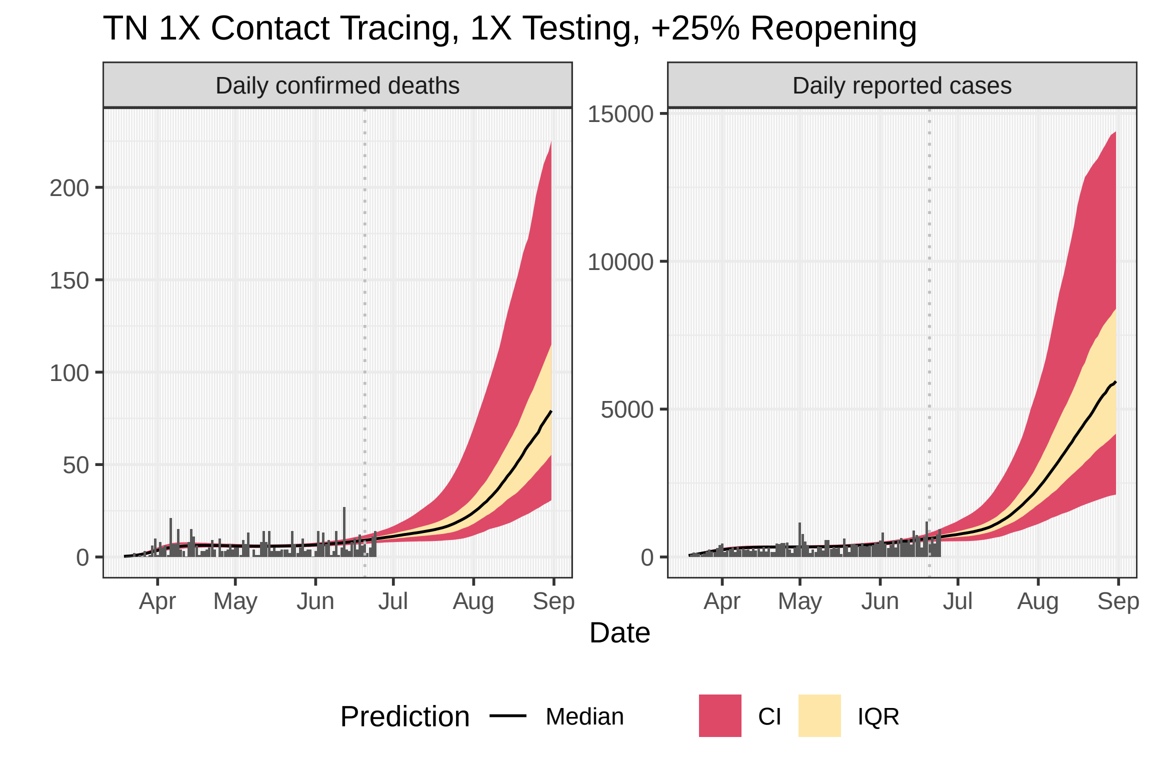

TN 1X Contact Tracing, 1X Testing, +25% Reopening
Daily confirmed deaths
Daily reported cases
200
150
100
50
0
15000
10000
5000
0
Apr
May
Jun
Jul
Aug
Sep
Date
Prediction
Median
CI
IQR

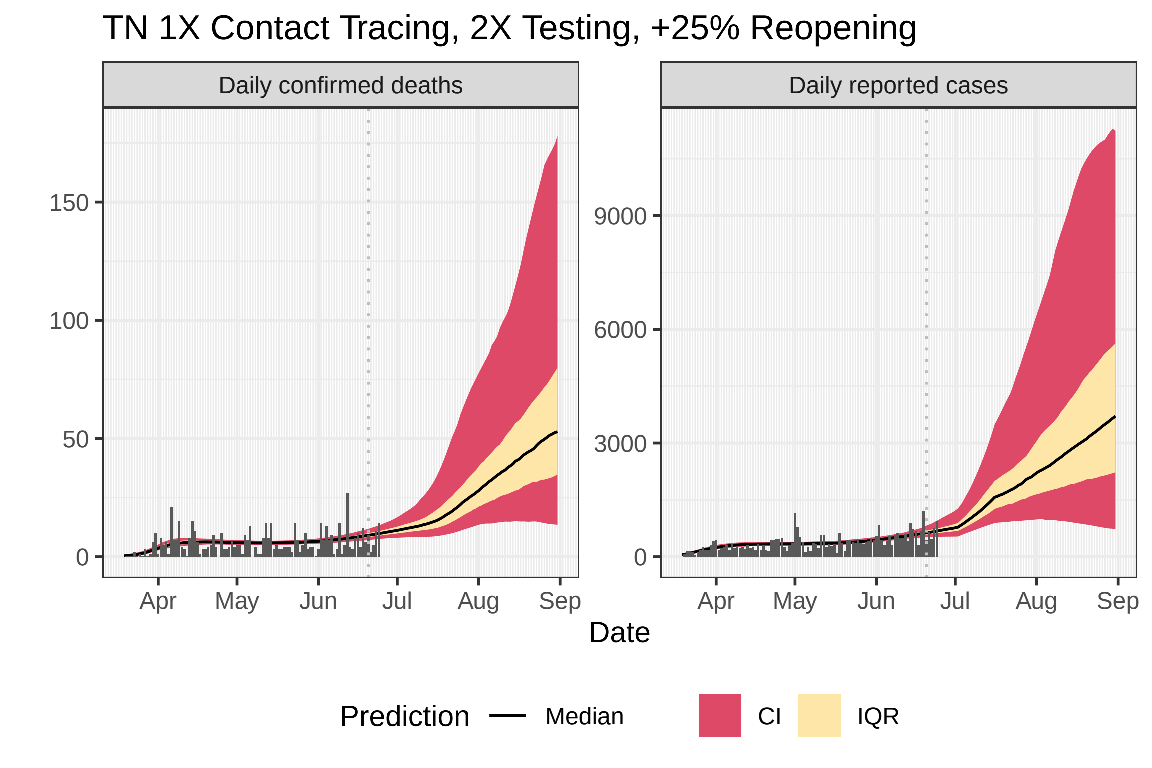

TN 1X Contact Tracing, 2X Testing, +25% Reopening
Daily confirmed deaths
Daily reported cases
150
100
50
0
9000
6000
3000
0
Apr
May
Jun
Jul
Aug
Sep
Date
Prediction
Median
CI
IQR

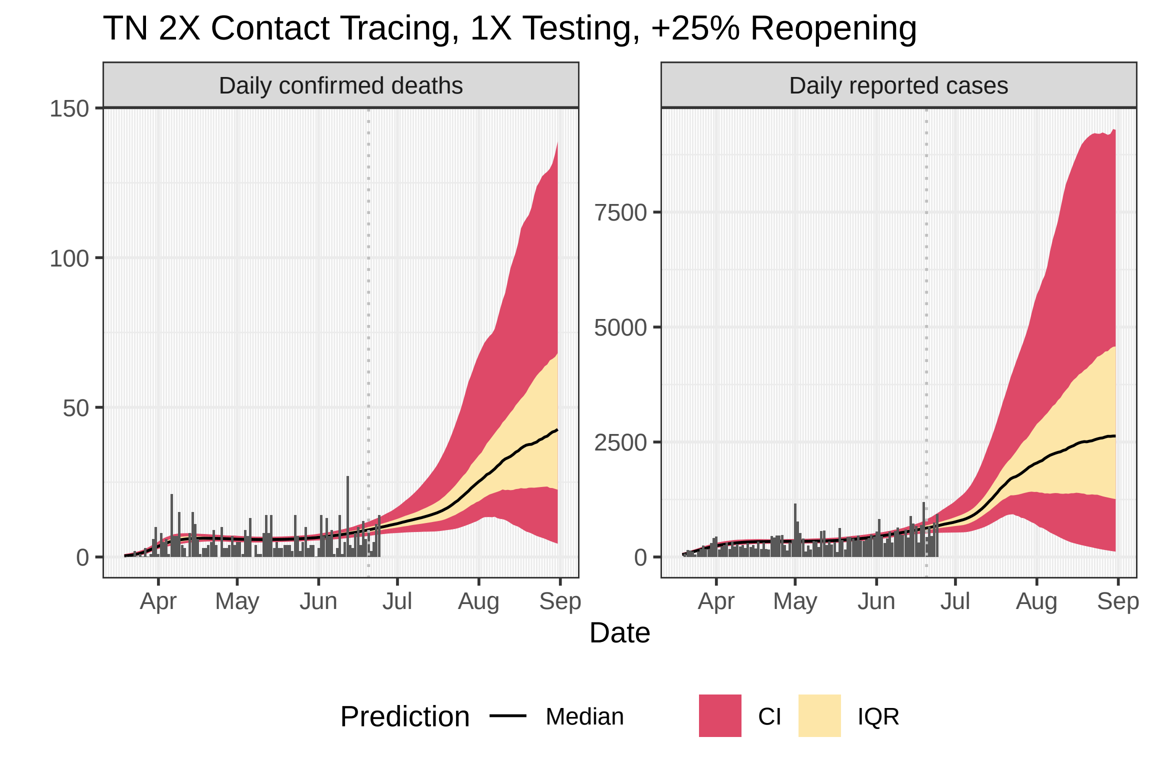

TN 2X Contact Tracing, 1X Testing, +25% Reopening
Daily confirmed deaths
Daily reported cases
150
100
50
0
7500
5000
2500
0
Apr
May
Jun
Jul
Aug
Sep
Date
Prediction
Median
CI
IQR

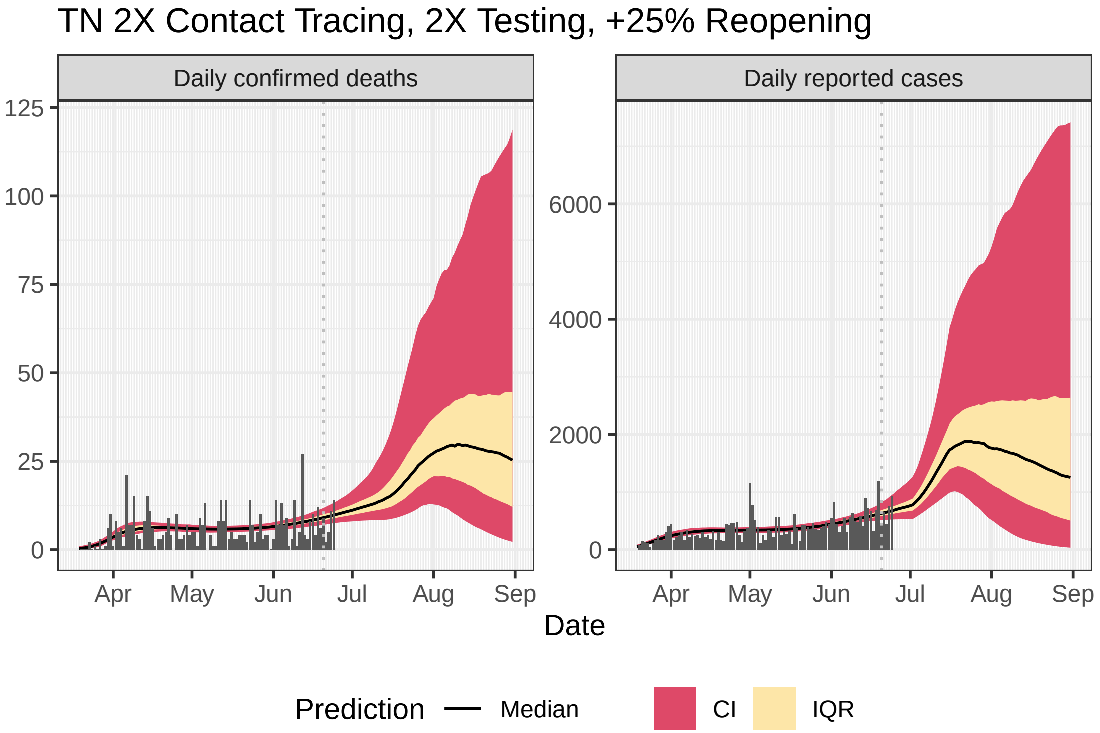

TN 2X Contact Tracing, 2X Testing, +25% Reopening
Daily confirmed deaths
Daily reported cases
125
100
75
50
25
0
6000
4000
2000
0
Apr
May
Jun
Jul
Aug
Sep
Date
Prediction
Median
CI
IQR

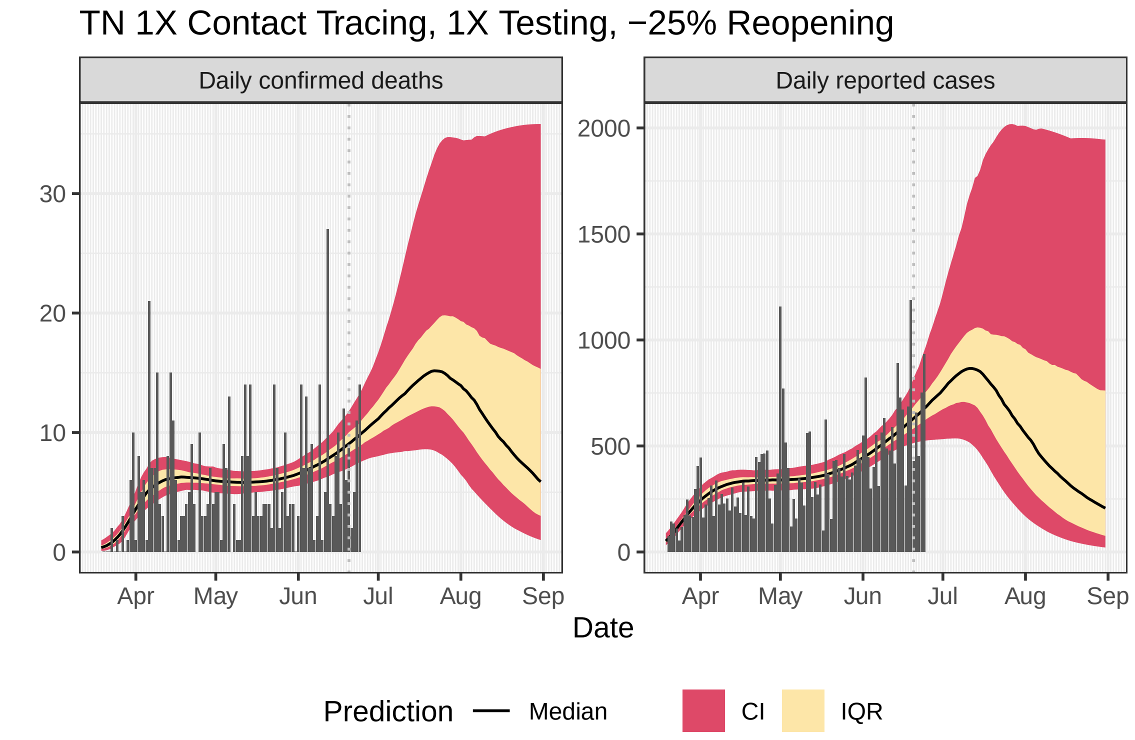
TN 1X Contact Tracing, 1X Testing, −25% Reopening
Daily confirmed deaths
Daily reported cases
0
10
20
30
0
500
1000
1500
2000
Apr
May
Jun
Jul
Aug
Sep
Date
Prediction
Median
CI
IQR

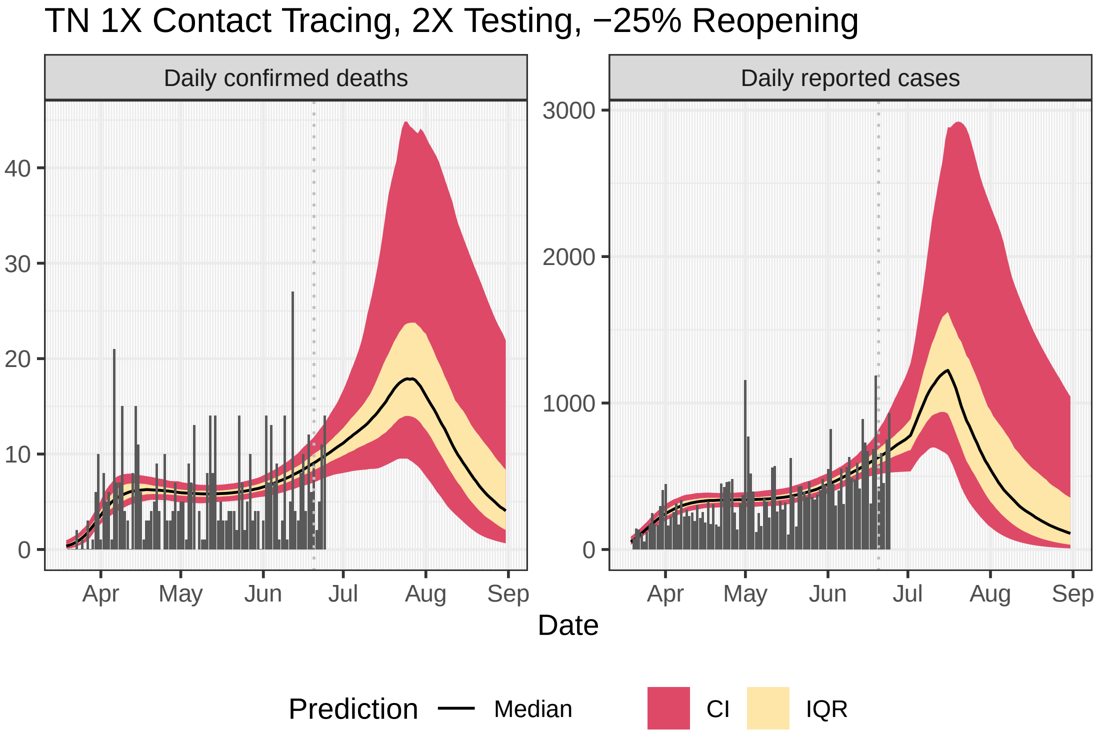

TN 1X Contact Tracing, 2X Testing, −25% Reopening
Daily confirmed deaths
Daily reported cases
0
10
20
30
40
0
1000
2000
3000
Apr
May
Jun
Jul
Aug
Sep
Date
Prediction
Median
CI
IQR

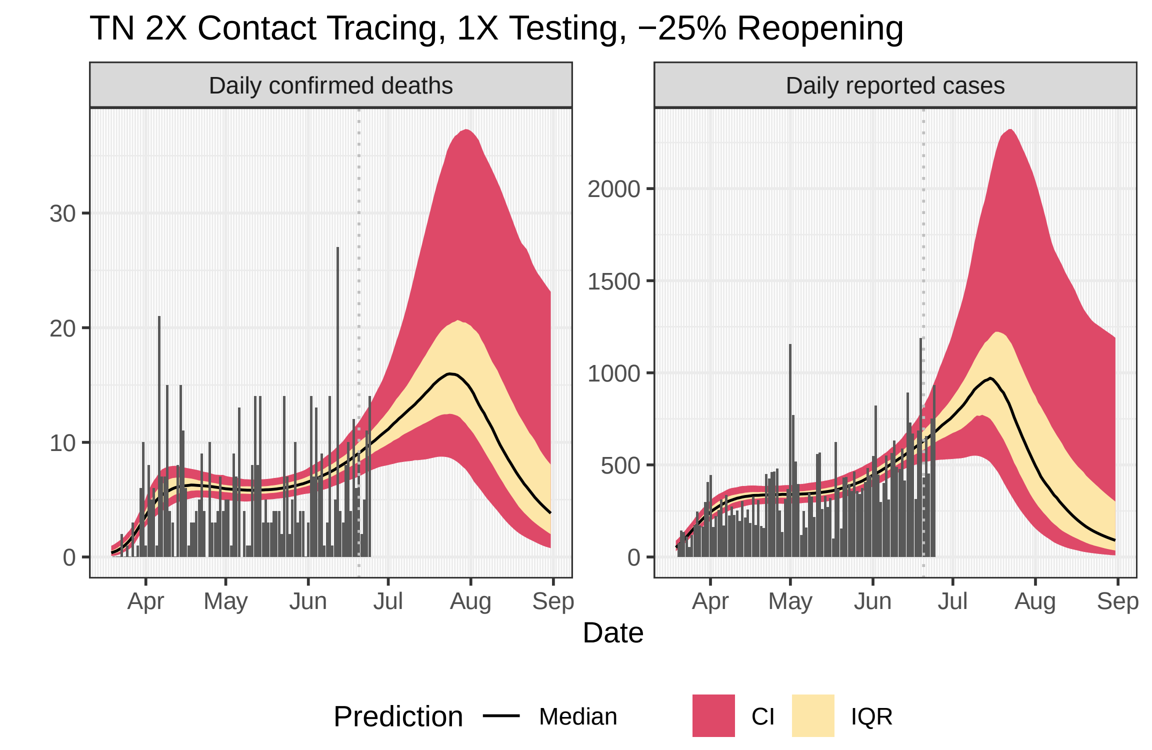

TN 2X Contact Tracing, 1X Testing, −25% Reopening
Daily confirmed deaths
Daily reported cases
0
10
20
30
0
500
1000
1500
2000
Apr
May
Jun
Jul
Aug
Sep
Date
Prediction
Median
CI
IQR

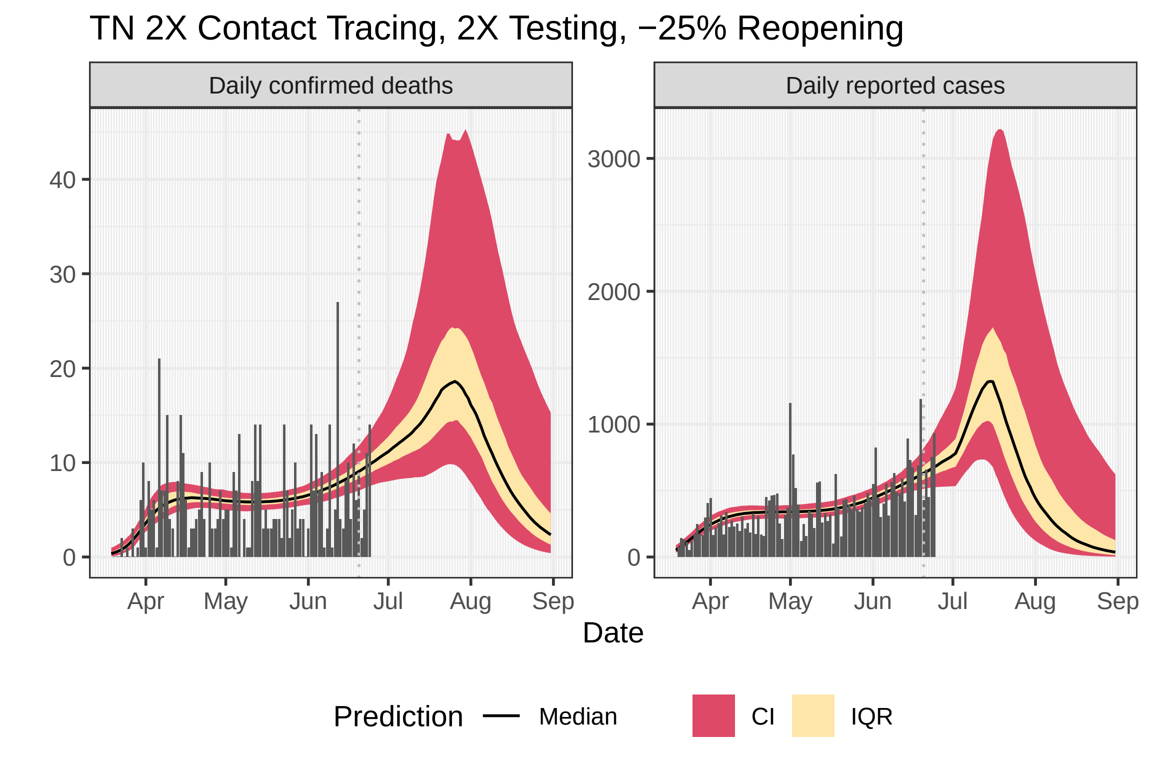

TN 2X Contact Tracing, 2X Testing, −25% Reopening
Daily confirmed deaths
Daily reported cases
0
10
20
30
40
0
1000
2000
3000
Apr
May
Jun
Jul
Aug
Sep
Date
Prediction
Median
CI
IQR

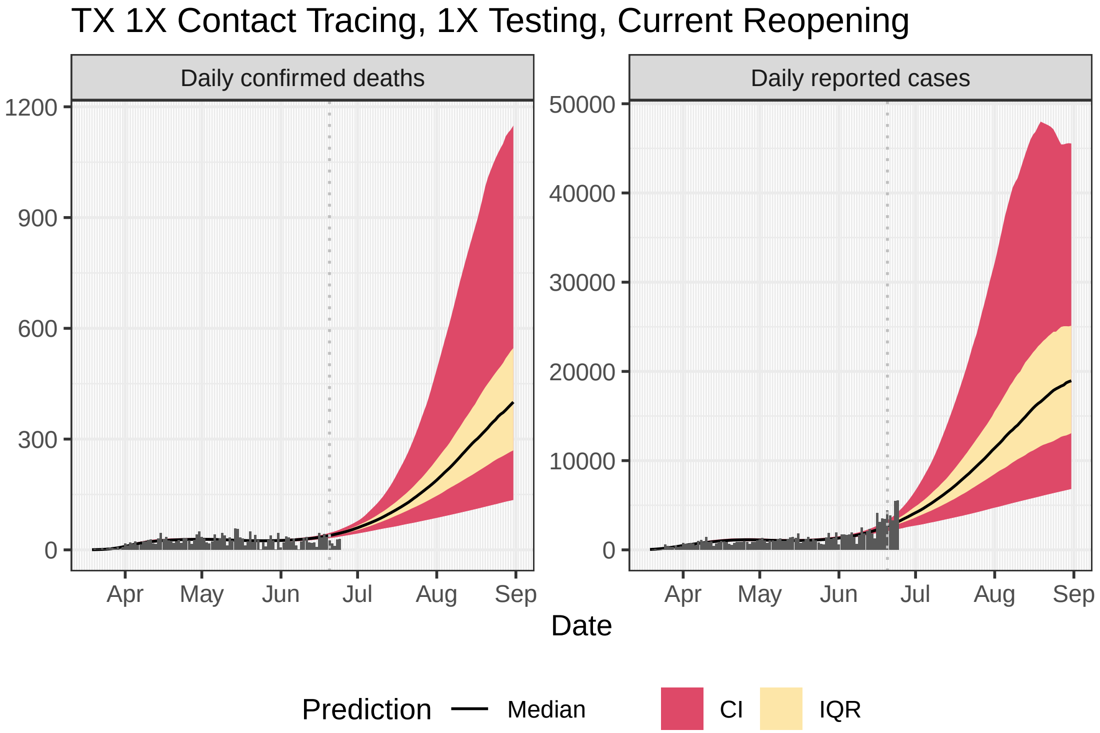

TX 1X Contact Tracing, 1X Testing, Current Reopening
Daily confirmed deaths
Daily reported cases
1200
900
600
300
0
50000
40000
30000
20000
10000
0
Apr
May
Jun
Jul
Aug
Sep
Date
Prediction
Median
CI
IQR

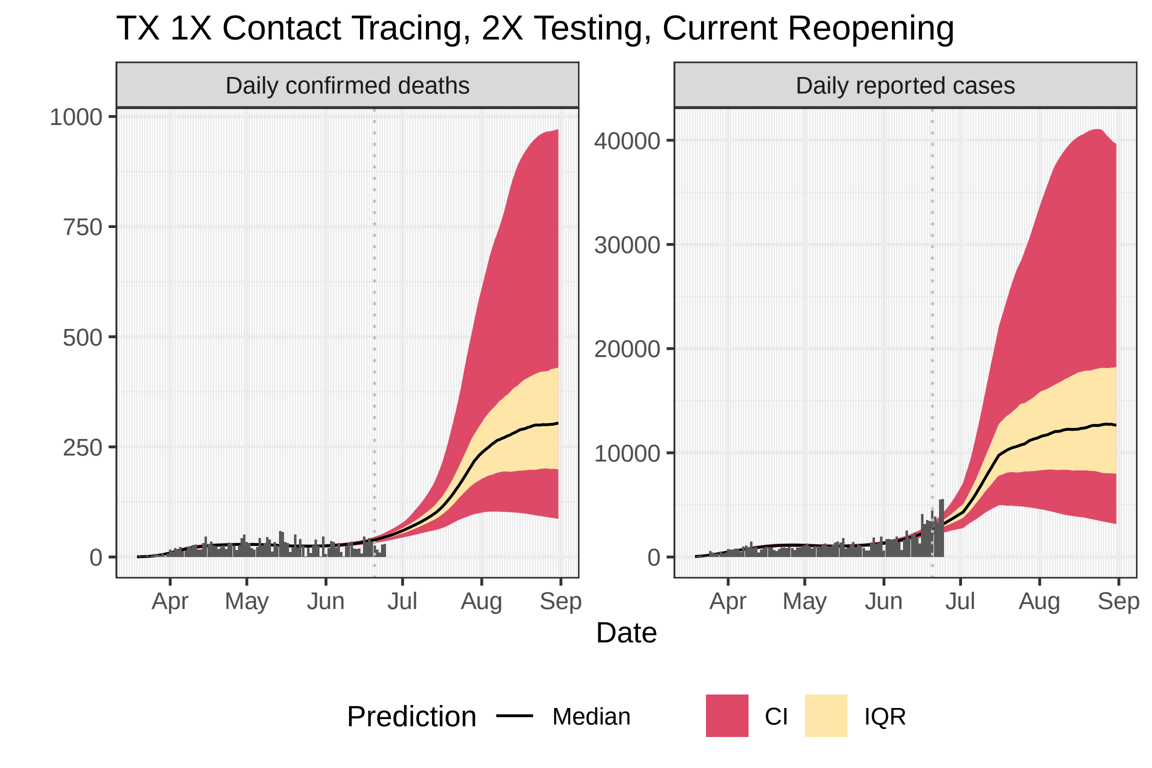
TX 1X Contact Tracing, 2X Testing, Current Reopening
Daily confirmed deaths
Daily reported cases
1000
750
500
250
0
40000
30000
20000
10000
0
Apr
May
Jun
Jul
Aug
Sep
Date
Prediction
Median
CI
IQR

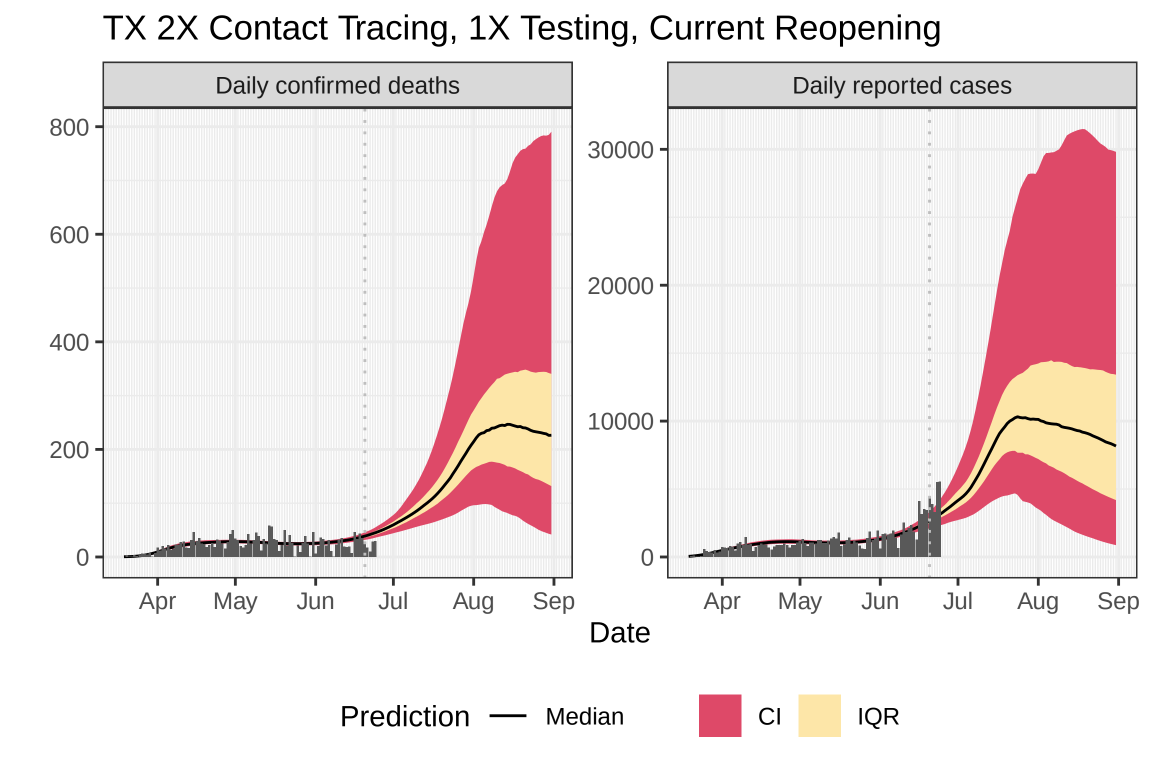

TX 2X Contact Tracing, 1X Testing, Current Reopening
Daily confirmed deaths
Daily reported cases
800
600
400
200
0
30000
20000
10000
0
Apr
May
Jun
Jul
Aug
Sep
Date
Prediction
Median
CI
IQR

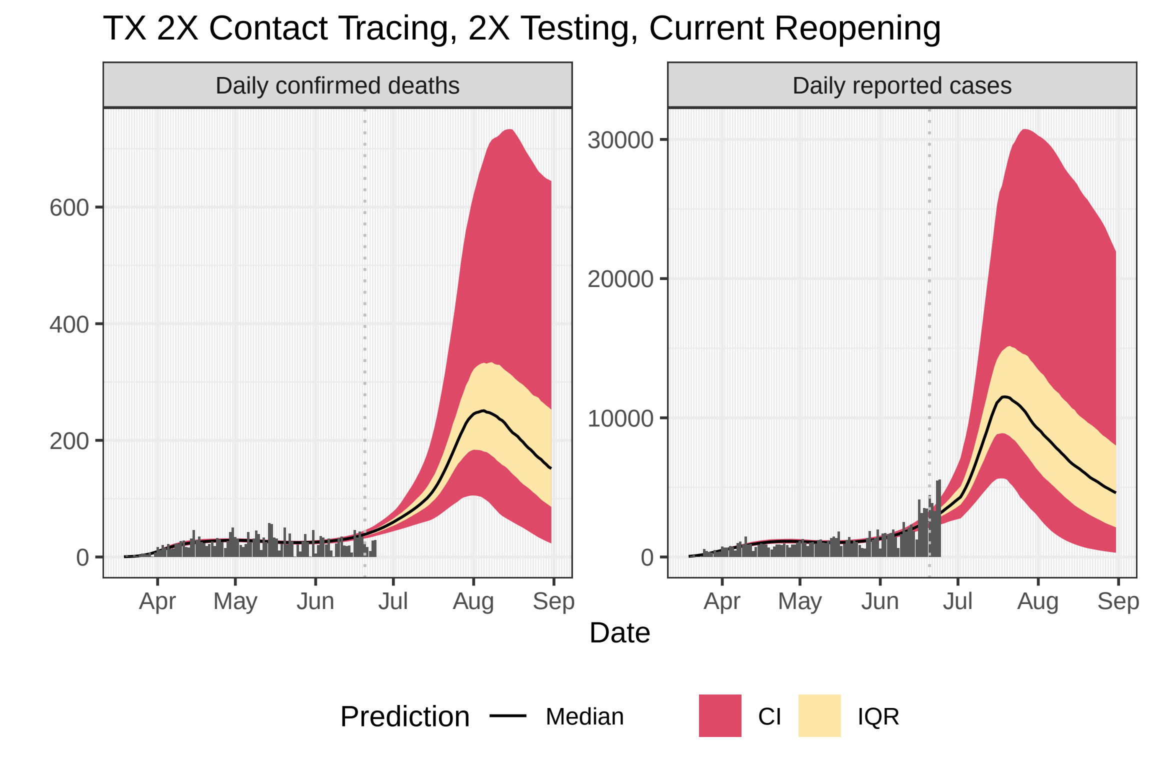

TX 2X Contact Tracing, 2X Testing, Current Reopening
Daily confirmed deaths
Daily reported cases
600
400
200
0
30000
20000
10000
0
Apr
May
Jun
Jul
Aug
Sep
Date
Prediction
Median
CI
IQR

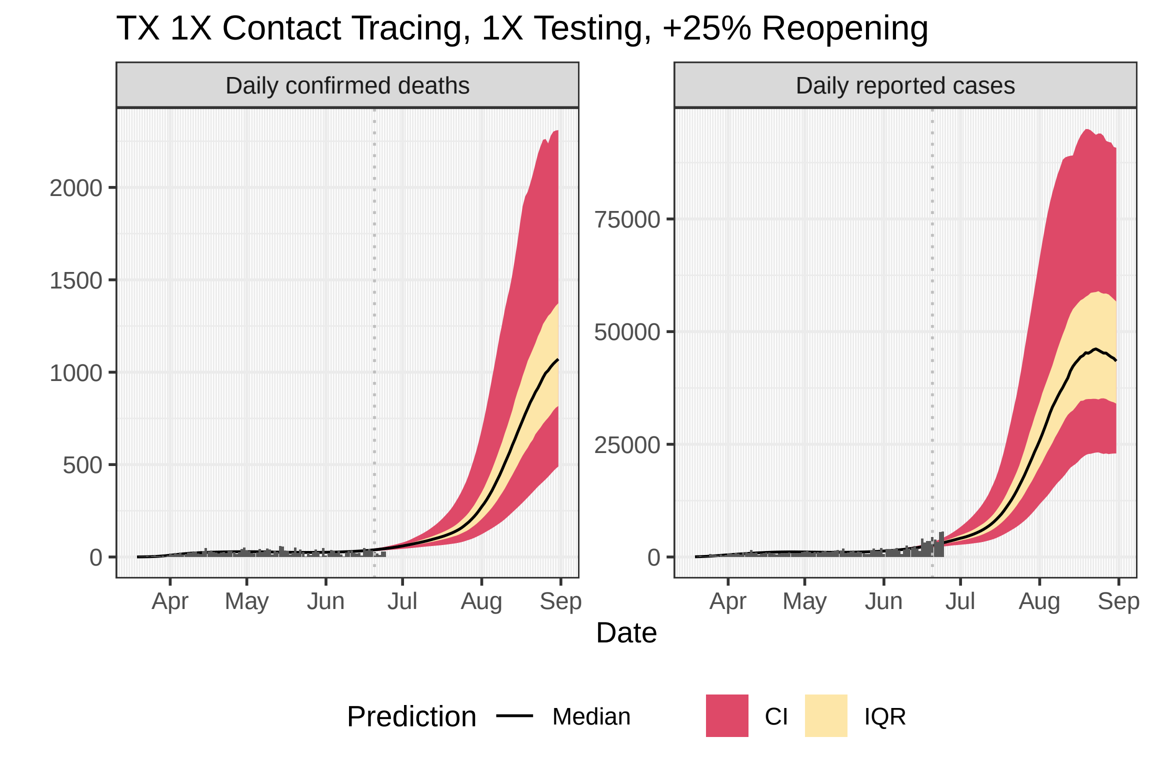
TX 1X Contact Tracing, 1X Testing, +25% Reopening
Daily confirmed deaths
Daily reported cases
2000
1500
1000
500
0
75000
50000
25000
0
Apr
May
Jun
Jul
Aug
Sep
Date
Prediction
Median
CI
IQR

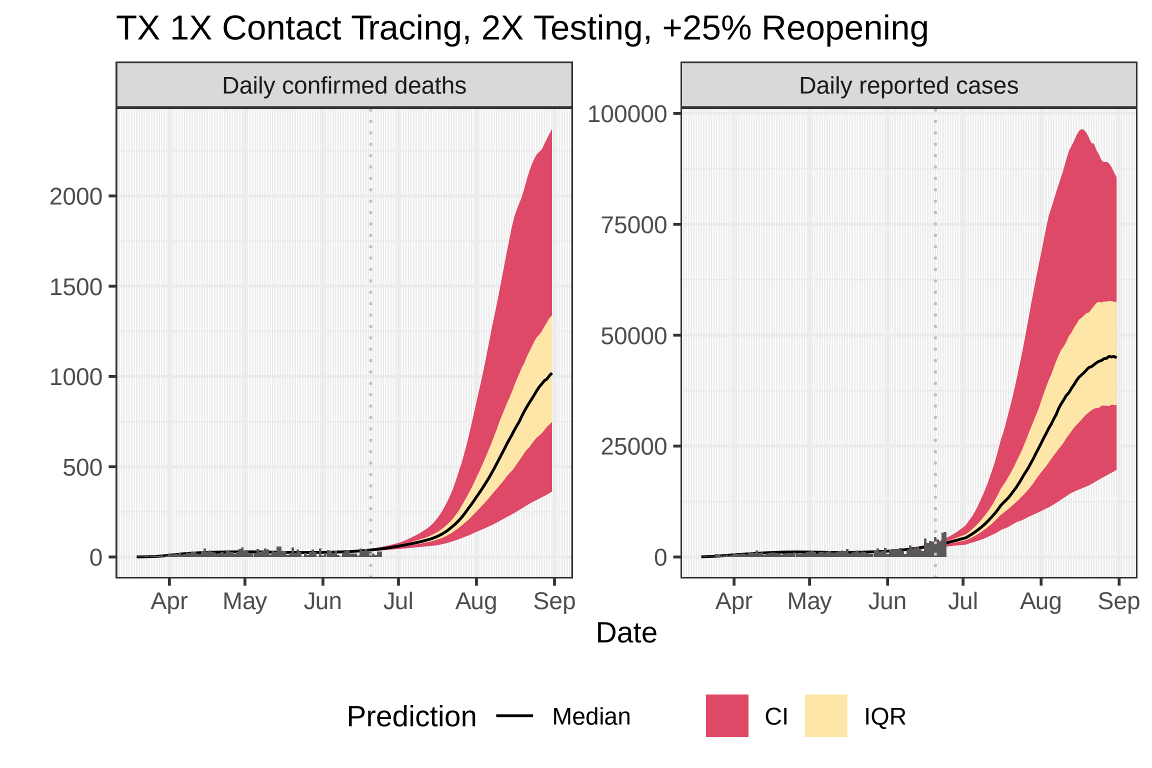
TX 1X Contact Tracing, 2X Testing, +25% Reopening
Daily confirmed deaths
Daily reported cases
0
500
1000
1500
2000
25000
50000
75000
100000
Apr
May
Jun
Jul
Aug
Sep
Date
Prediction
Median
CI
IQR

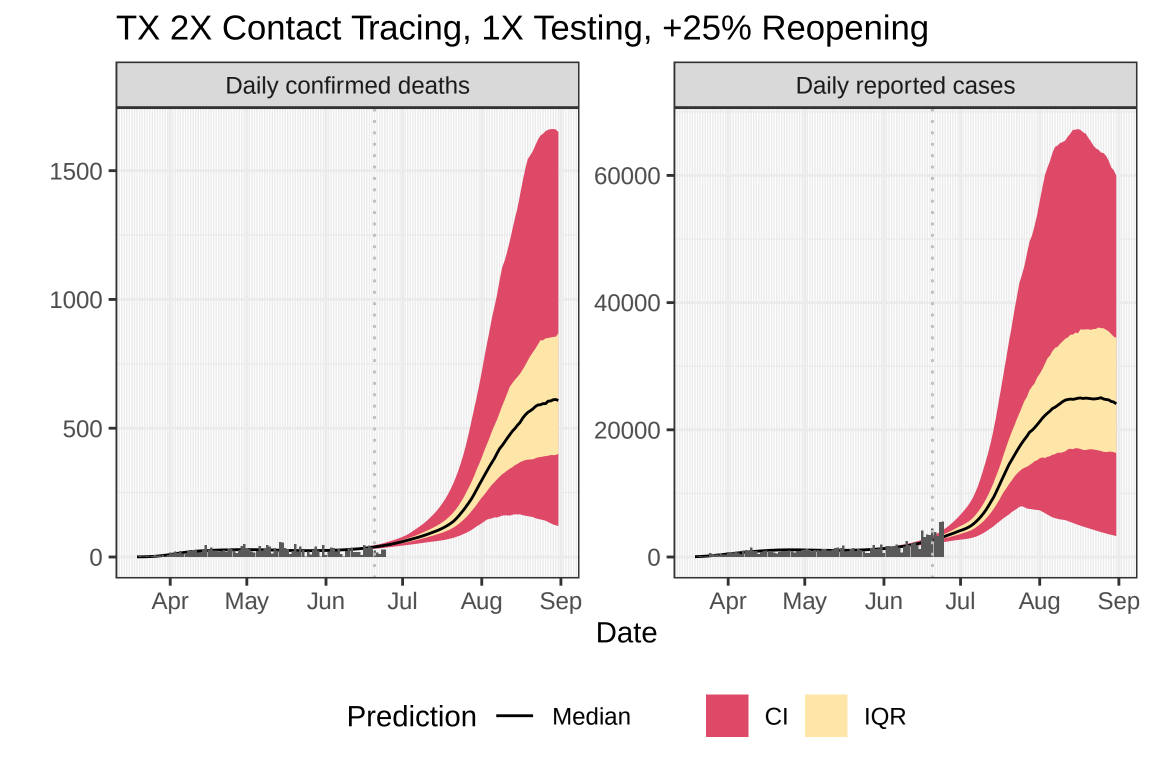
TX 2X Contact Tracing, 1X Testing, +25% Reopening
Daily confirmed deaths
Daily reported cases
1500
1000
500
0
60000
40000
20000
0
Apr
May
Jun
Jul
Aug
Sep
Date
Prediction
Median
CI
IQR

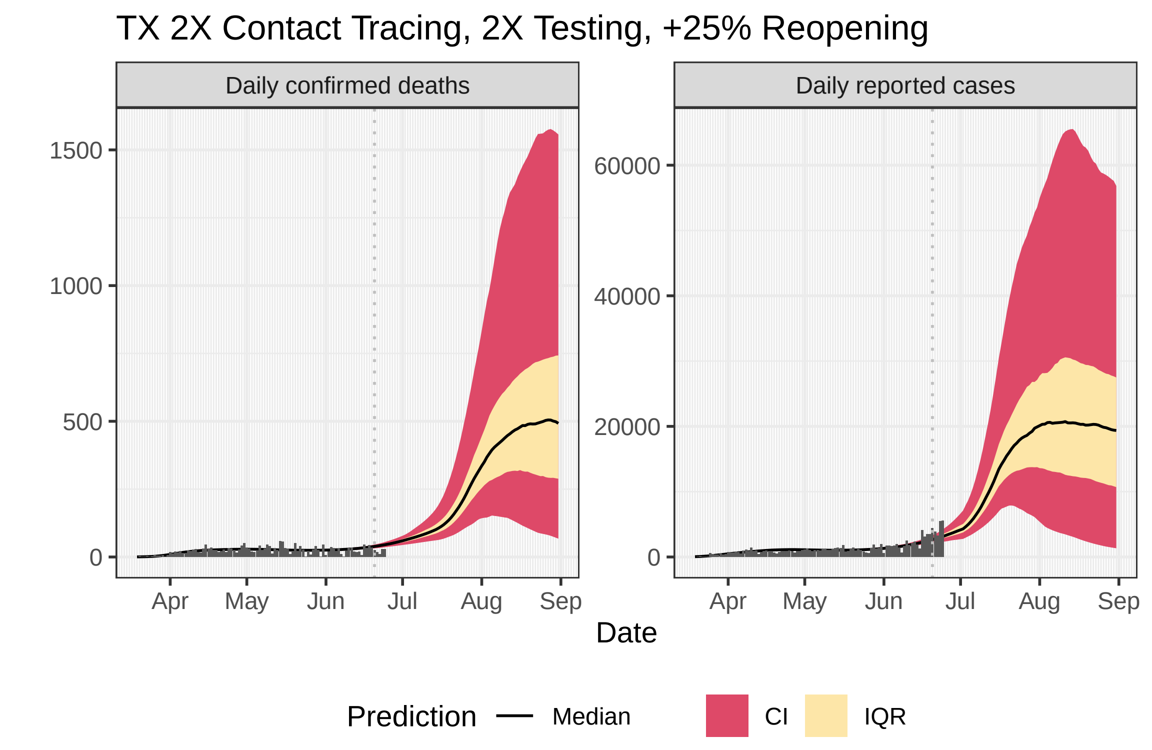
TX 2X Contact Tracing, 2X Testing, +25% Reopening
Daily confirmed deaths
Daily reported cases
1500
1000
500
0
60000
40000
20000
0
Apr
May
Jun
Jul
Aug
Sep
Date
Prediction
Median
CI
IQR

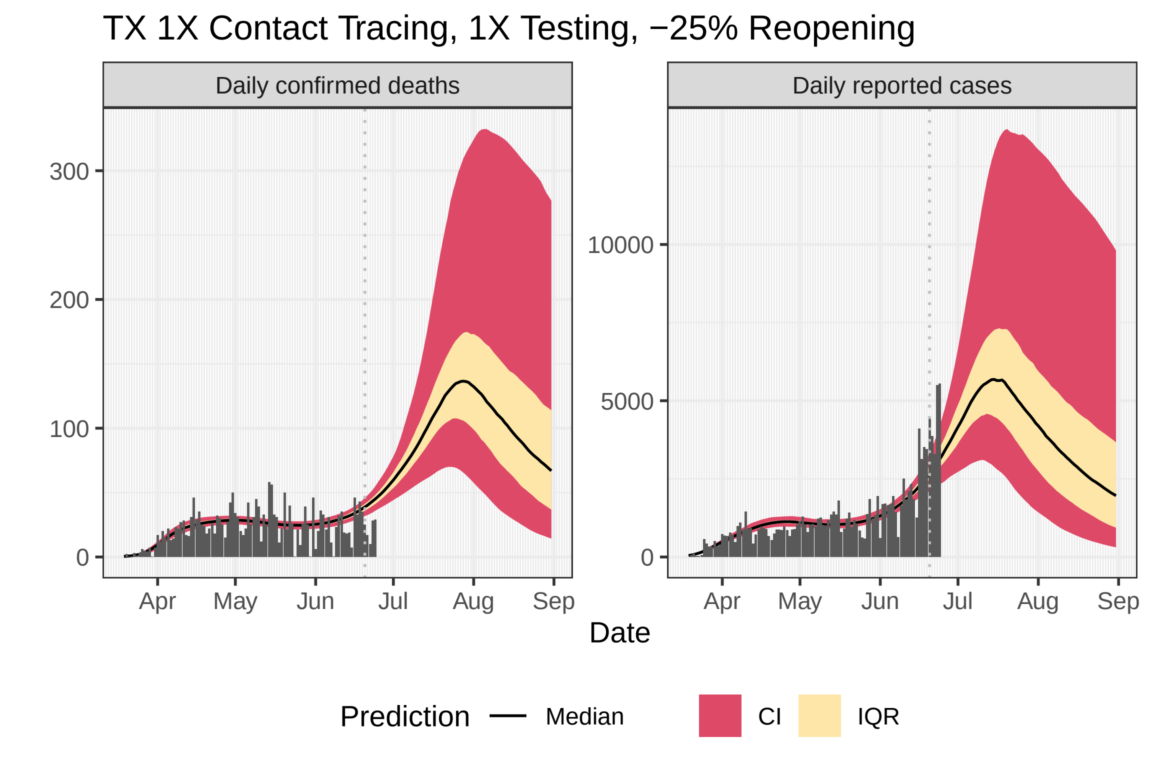

TX 1X Contact Tracing, 1X Testing, −25% Reopening
Daily confirmed deaths
Daily reported cases
300
200
100
0
10000
5000
0
Apr
May
Jun
Jul
Aug
Sep
Date
Prediction
Median
CI
IQR

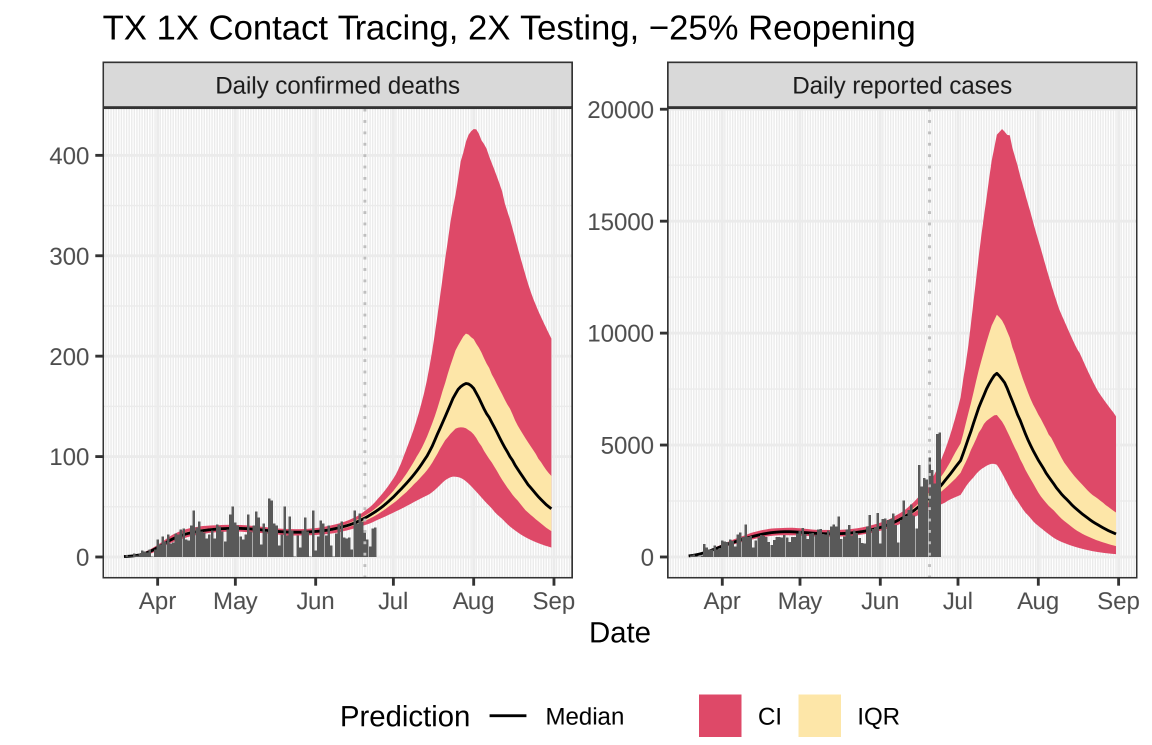

TX 1X Contact Tracing, 2X Testing, −25% Reopening
Daily confirmed deaths
Daily reported cases
400
300
200
100
0
20000
15000
10000
5000
0
Apr
May
Jun
Jul
Aug
Sep
Date
Prediction
Median
CI
IQR

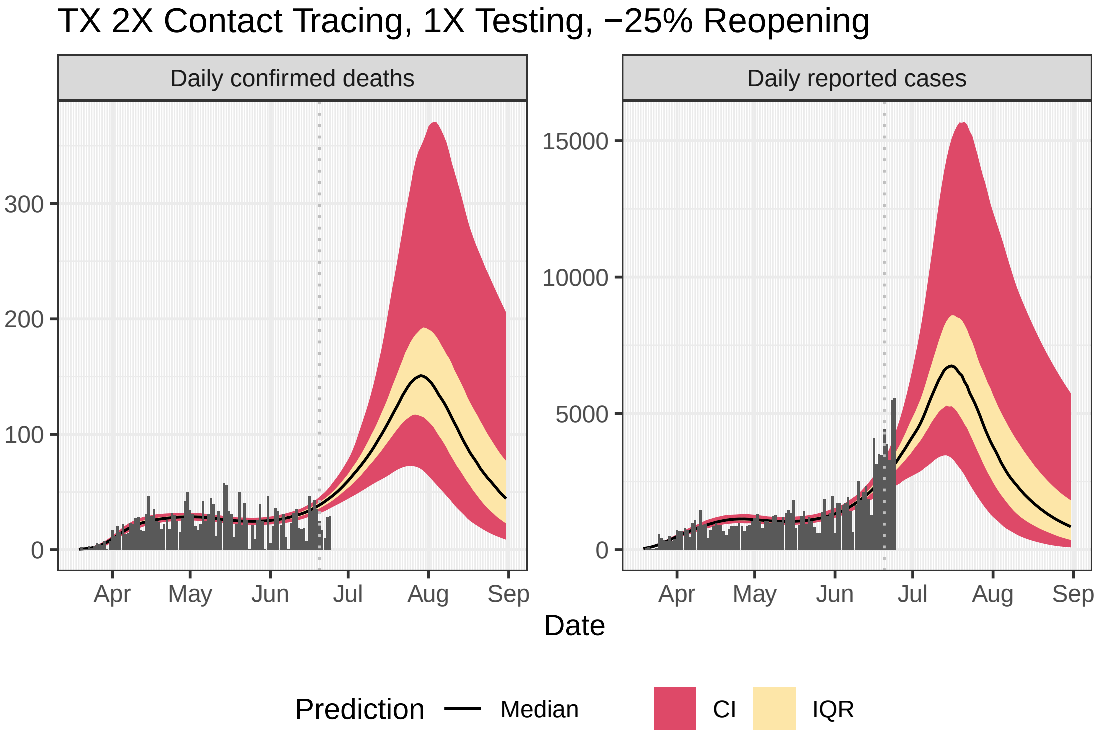

TX 2X Contact Tracing, 1X Testing, −25% Reopening
Daily confirmed deaths
Daily reported cases
300
200
100
0
15000
10000
5000
0
Apr
May
Jun
Jul
Aug
Sep
Date
Prediction
Median
CI
IQR

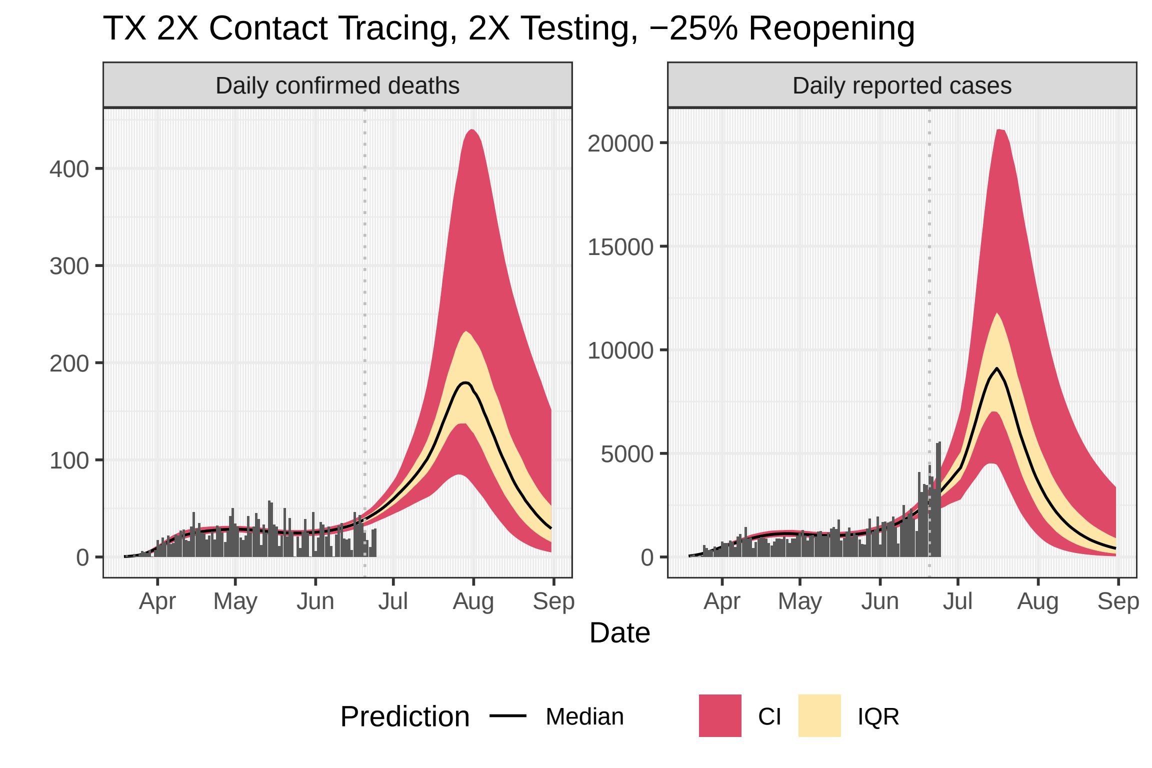
TX 2X Contact Tracing, 2X Testing, −25% Reopening
Daily confirmed deaths
Daily reported cases
0
100
200
300
400
0
5000
10000
15000
20000
Apr
May
Jun
Jul
Aug
Sep
Date
Prediction
Median
CI
IQR

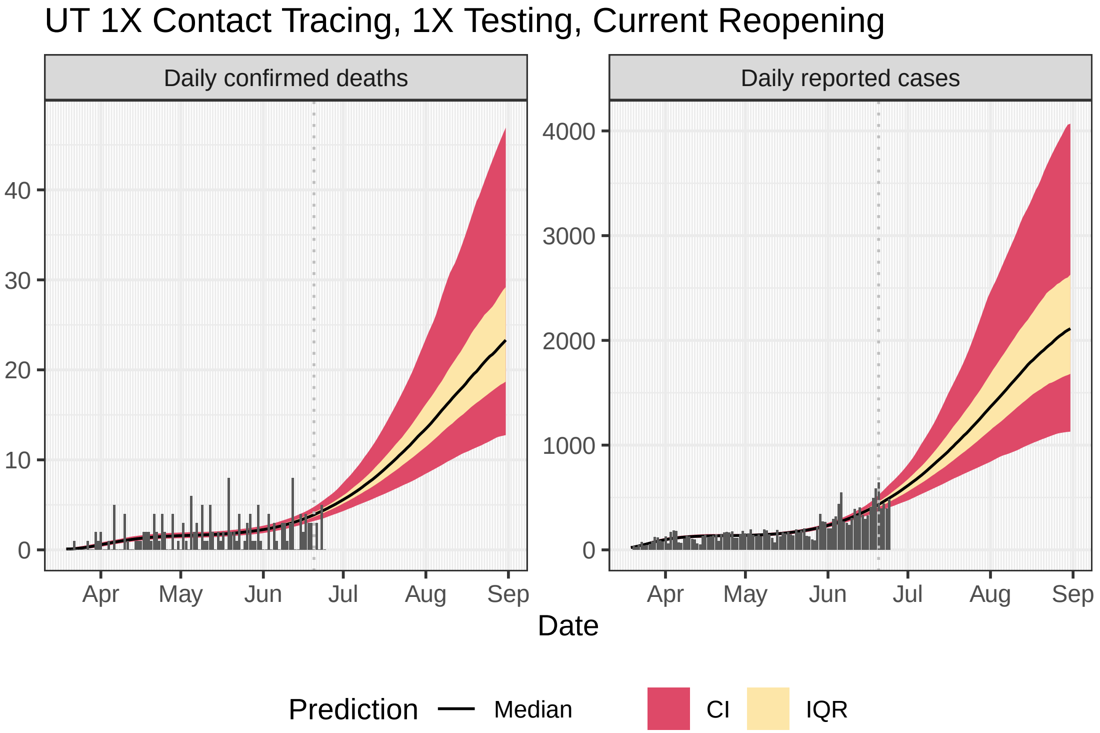

UT 1X Contact Tracing, 1X Testing, Current Reopening
Daily confirmed deaths
Daily reported cases
0
10
20
30
40
1000
2000
3000
4000
Apr
May
Jun
Jul
Aug
Sep
Date
Prediction
Median
CI
IQR

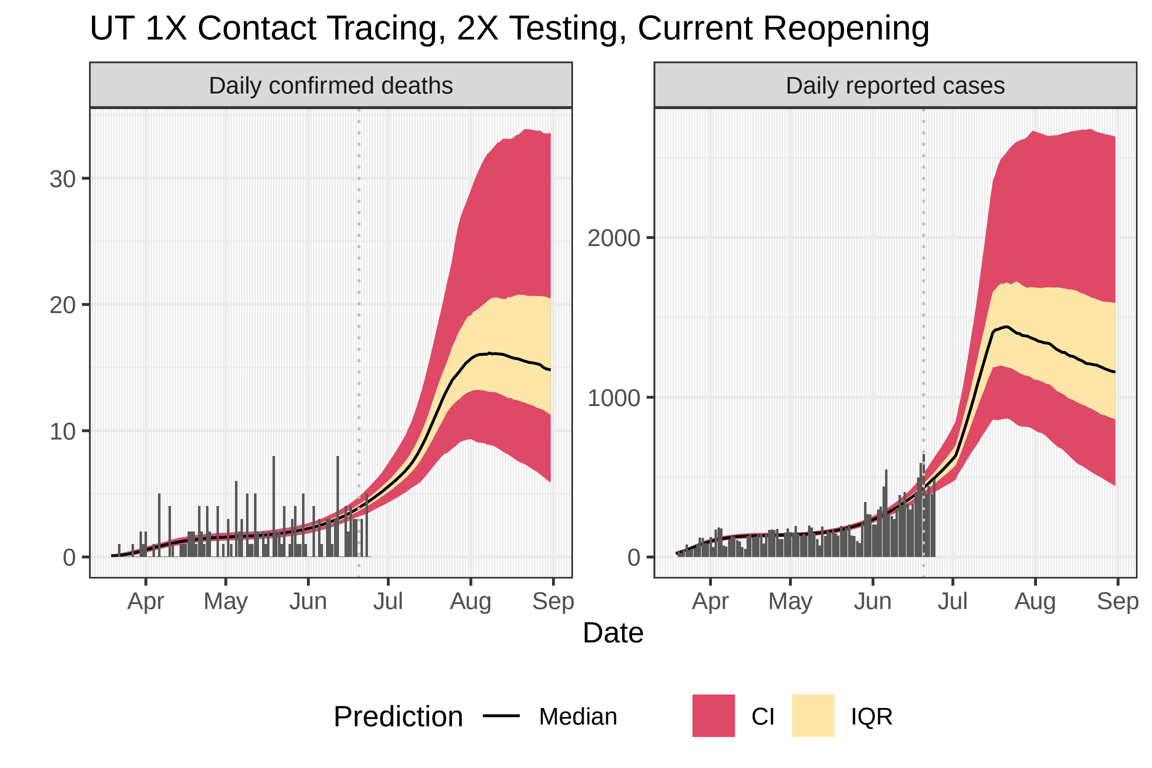
UT 1X Contact Tracing, 2X Testing, Current Reopening
Daily confirmed deaths
Daily reported cases
30
20
10
0
2000
1000
0
Apr
May
Jun
Jul
Aug
Sep
Date
Prediction
Median
CI
IQR

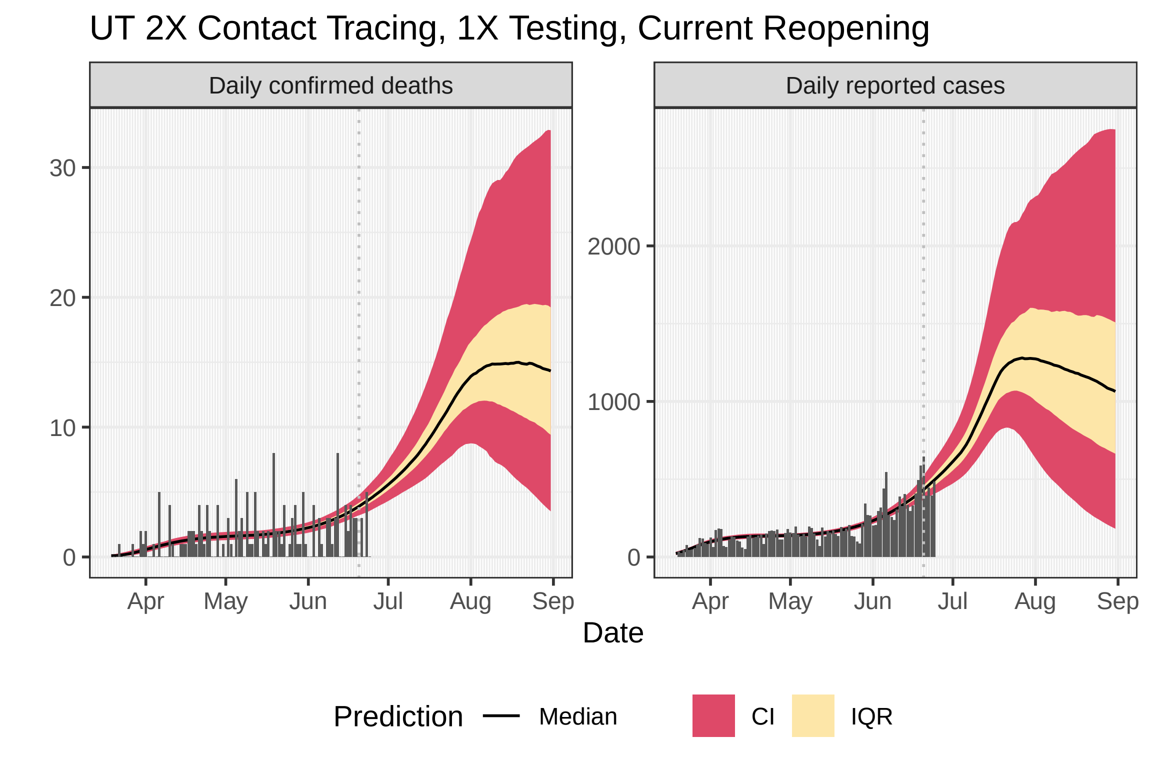

UT 2X Contact Tracing, 1X Testing, Current Reopening
Daily confirmed deaths
Daily reported cases
0
10
20
30
0
1000
2000
Apr
May
Jun
Jul
Aug
Sep
Date
Prediction
Median
CI
IQR

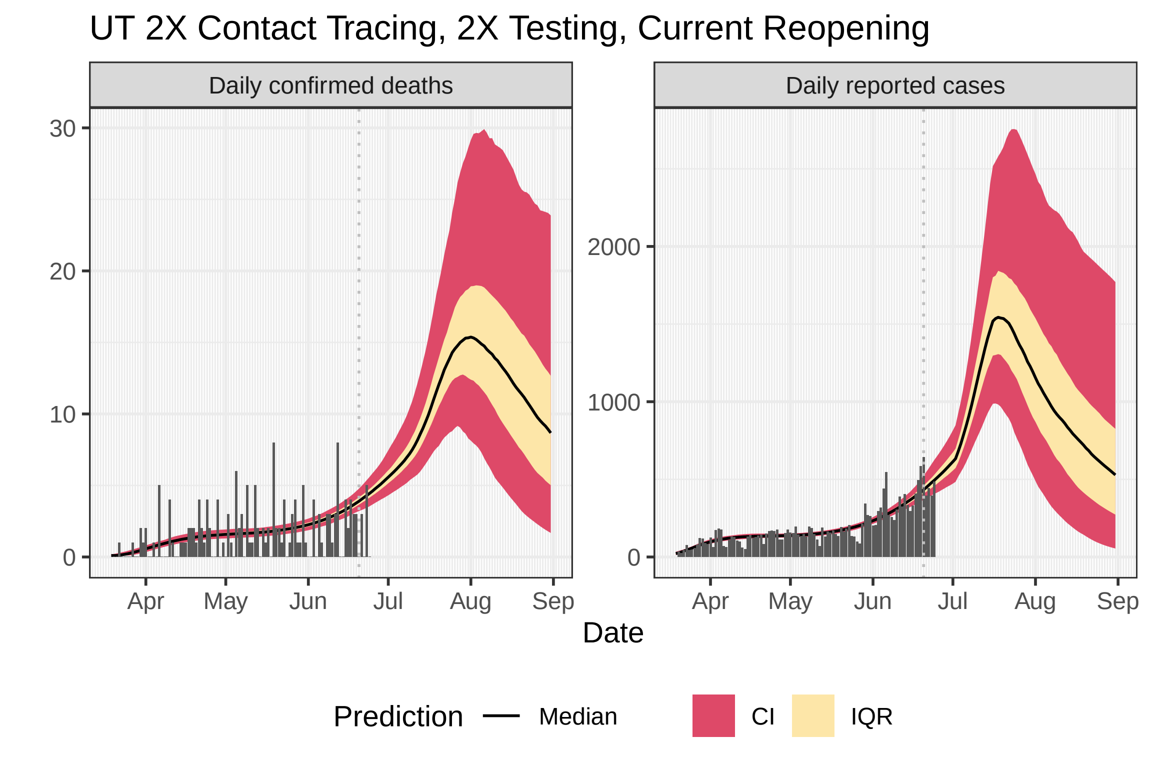
UT 2X Contact Tracing, 2X Testing, Current Reopening
Daily confirmed deaths
Daily reported cases
30
20
10
0
2000
1000
0
Apr
May
Jun
Jul
Aug
Sep
Date
Prediction
Median
CI
IQR

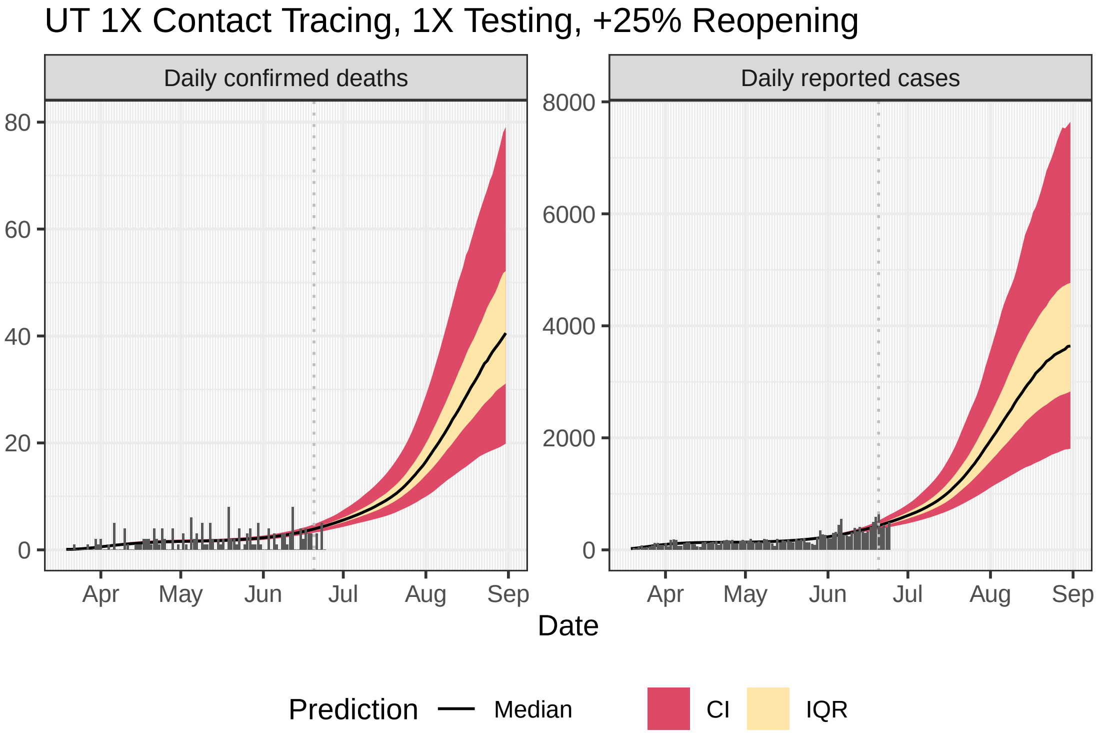

UT 1X Contact Tracing, 1X Testing, +25% Reopening
Daily confirmed deaths
Daily reported cases
0
20
40
60
80
0
2000
4000
6000
8000
Apr
May
Jun
Jul
Aug
Sep
Date
Prediction
Median
CI
IQR

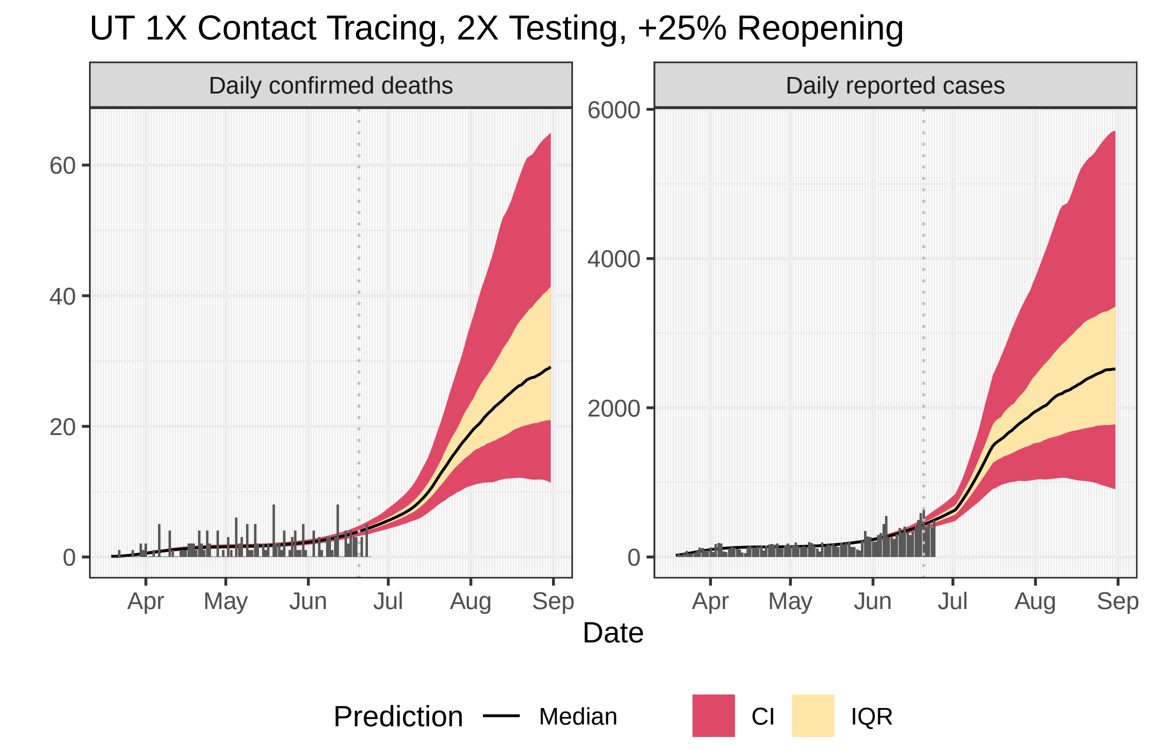

UT 1X Contact Tracing, 2X Testing, +25% Reopening
Daily confirmed deaths
Daily reported cases
0
20
40
60
0
2000
4000
6000
Apr
May
Jun
Jul
Aug
Sep
Date
Prediction
Median
CI
IQR

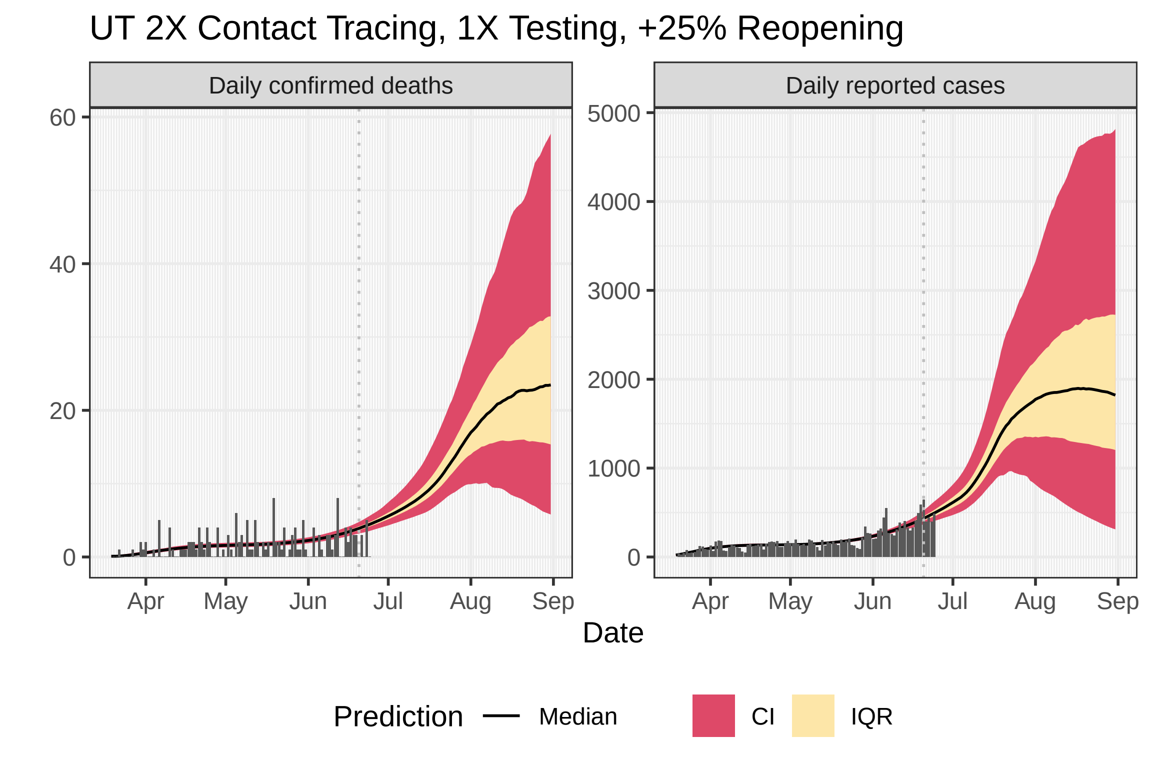
UT 2X Contact Tracing, 1X Testing, +25% Reopening
Daily confirmed deaths
Daily reported cases
60
40
20
0
5000
4000
3000
2000
1000
0
Apr
May
Jun
Jul
Aug
Sep
Date
Prediction
Median
CI
IQR

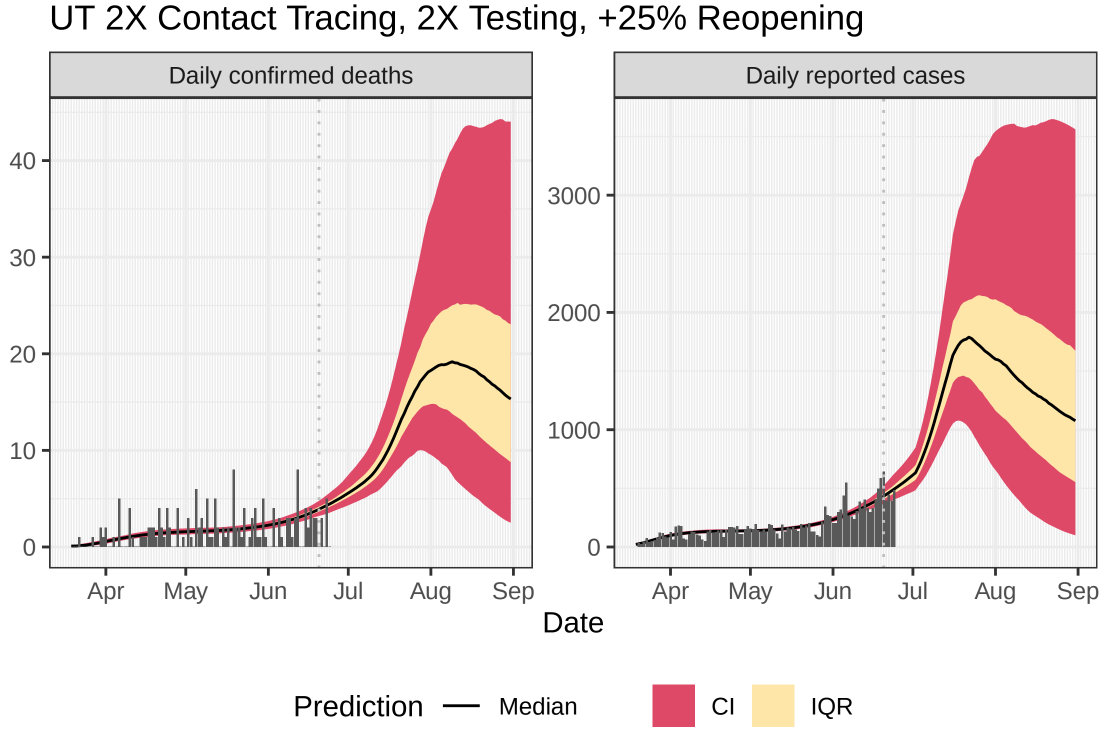

UT 2X Contact Tracing, 2X Testing, +25% Reopening
Daily confirmed deaths
Daily reported cases
0
10
20
30
40
0
1000
2000
3000
Apr
May
Jun
Jul
Aug
Sep
Date
Prediction
Median
CI
IQR

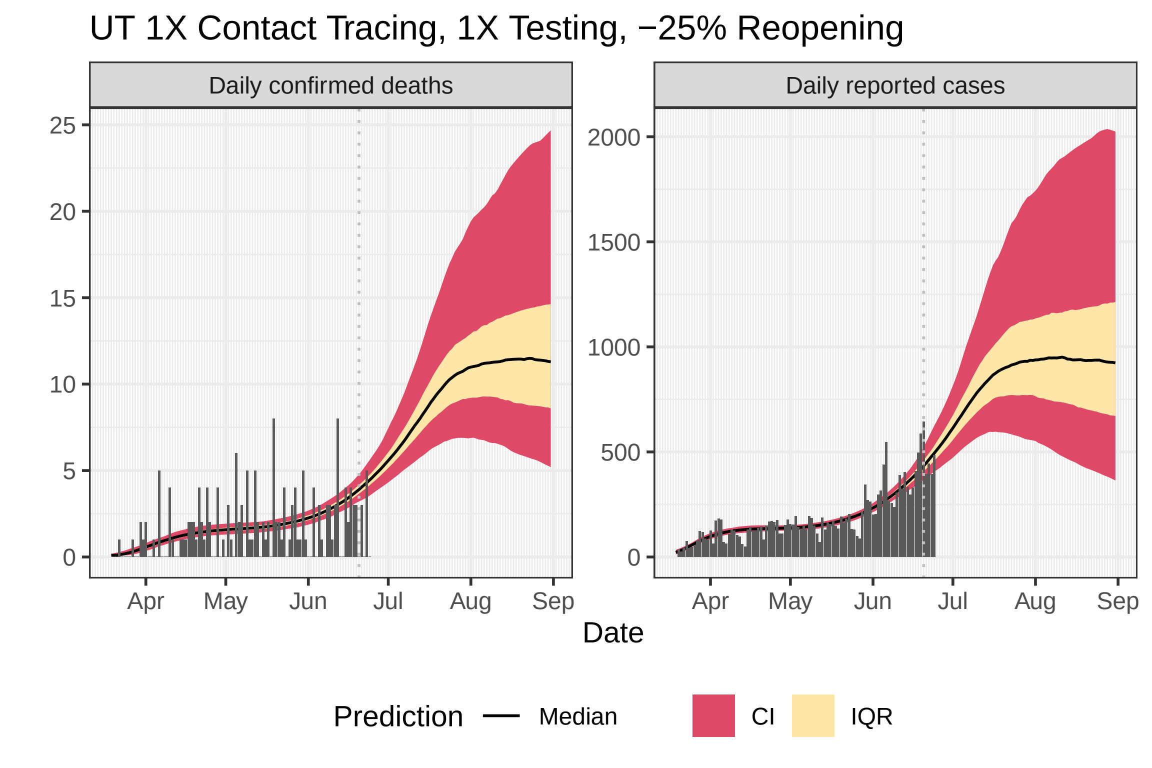

UT 1X Contact Tracing, 1X Testing, −25% Reopening
Daily confirmed deaths
Daily reported cases
0
5
10
15
20
25
500
1000
1500
2000
Apr
May
Jun
Jul
Aug
Sep
Date
Prediction
Median
CI
IQR

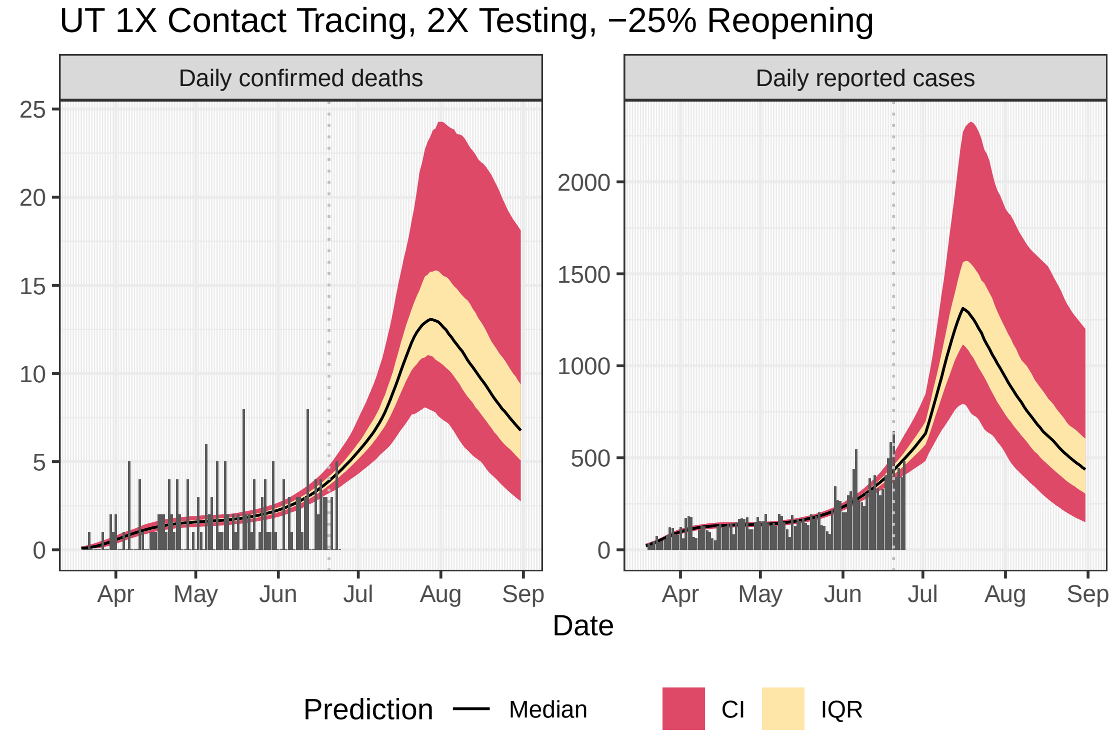
UT 1X Contact Tracing, 2X Testing, −25% Reopening
Daily confirmed deaths
Daily reported cases
25
20
15
10
5
0
2000
1500
1000
500
0
Apr
May
Jun
Jul
Aug
Sep
Date
Prediction
Median
CI
IQR

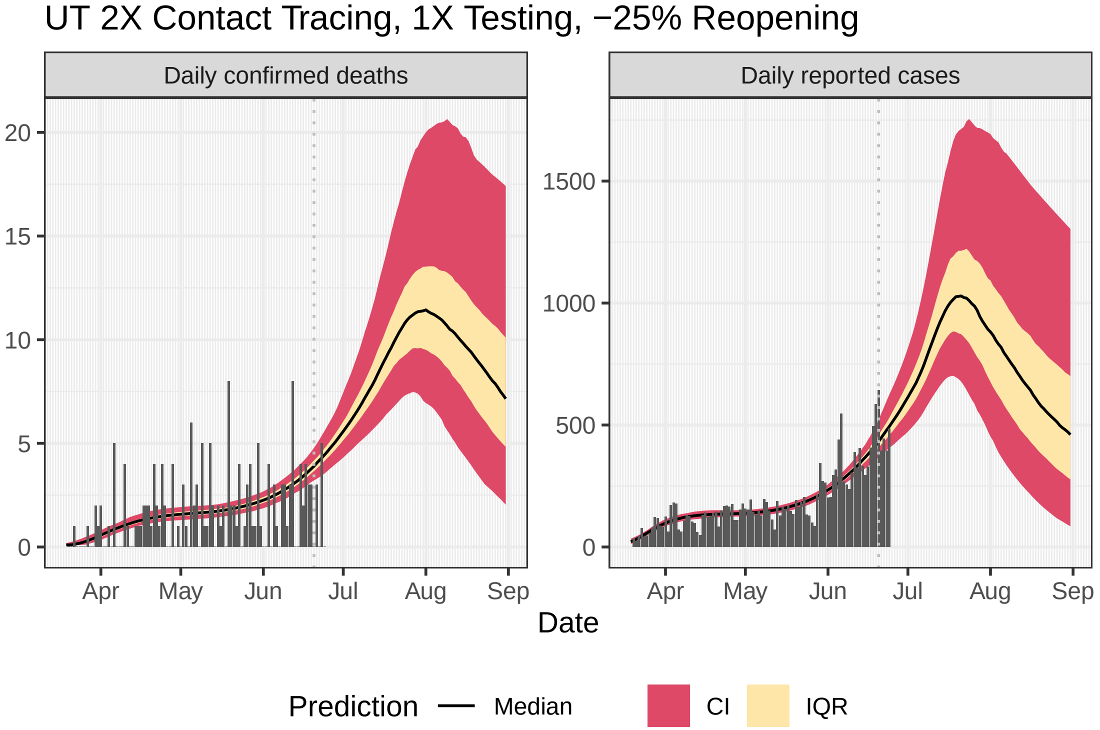

UT 2X Contact Tracing, 1X Testing, −25% Reopening
Daily confirmed deaths
Daily reported cases
20
15
10
5
0
1500
1000
500
0
Apr
May
Jun
Jul
Aug
Sep
Date
Prediction
Median
CI
IQR

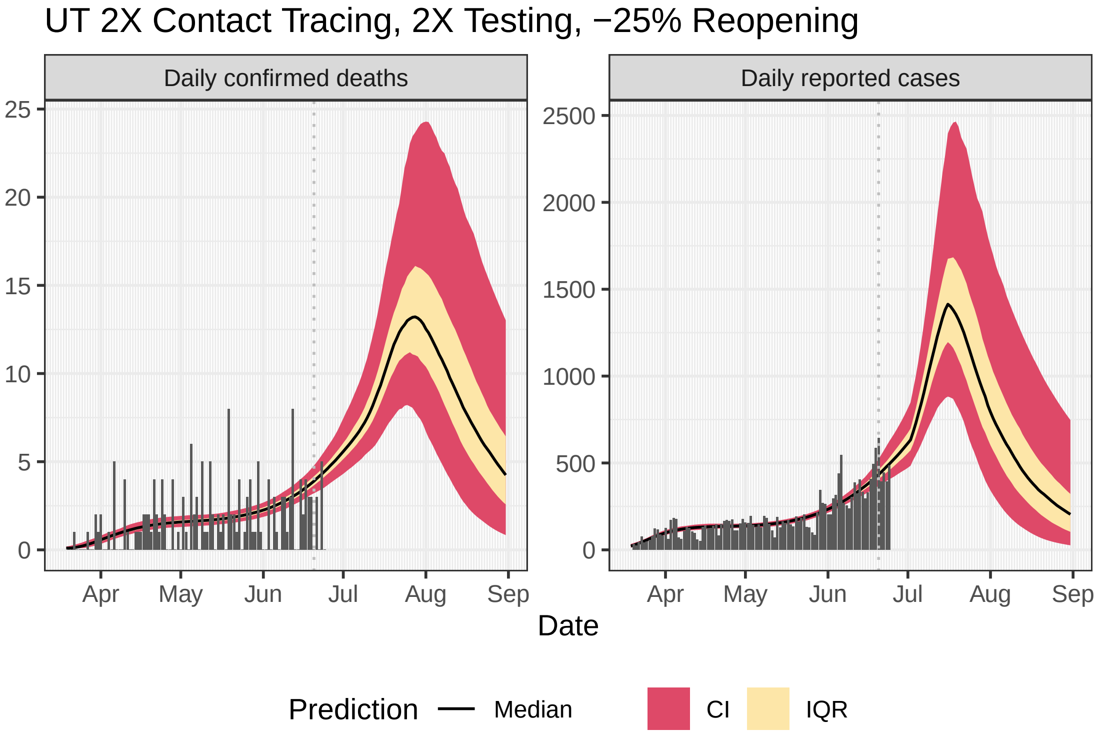
UT 2X Contact Tracing, 2X Testing, −25% Reopening
Daily confirmed deaths
Daily reported cases
25
20
15
10
5
0
2500
2000
1500
1000
500
0
Apr
May
Jun
Jul
Aug
Sep
Date
Prediction
Median
CI
IQR

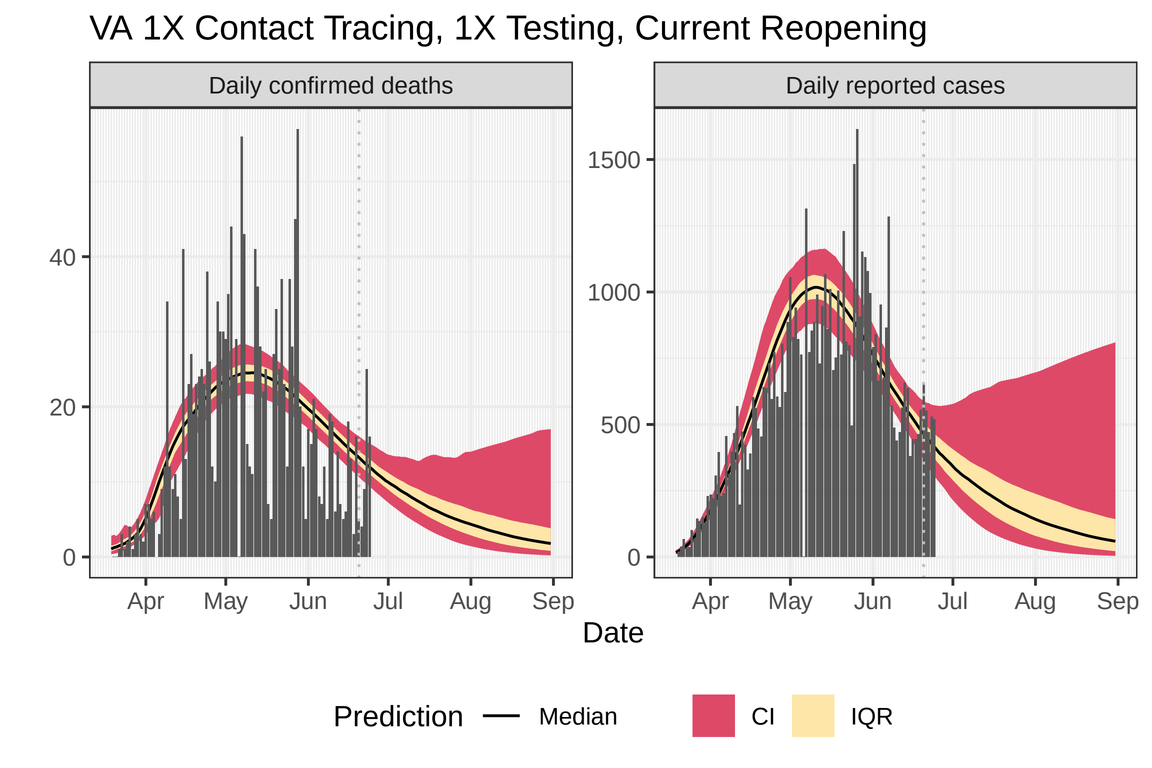
VA 1X Contact Tracing, 1X Testing, Current Reopening
Daily confirmed deaths
Daily reported cases
0
20
40
500
1000
1500
Apr
May
Jun
Jul
Aug
Sep
Date
Prediction
Median
CI
IQR

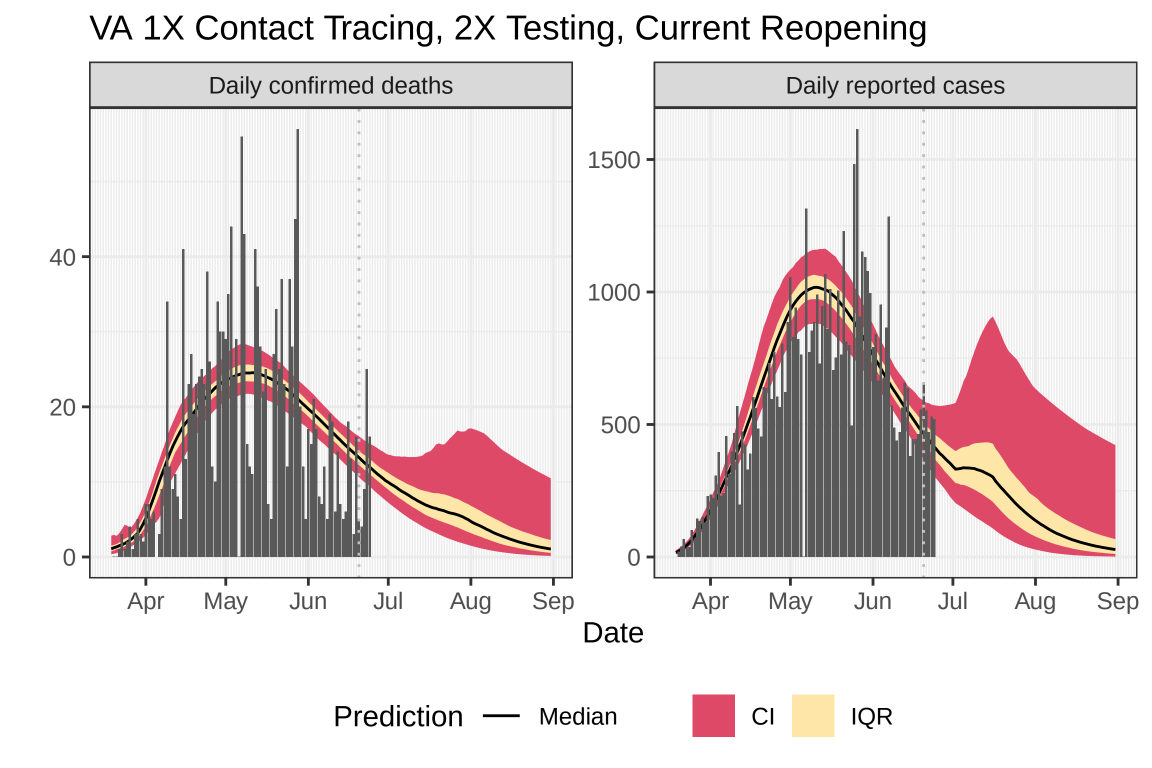
VA 1X Contact Tracing, 2X Testing, Current Reopening
Daily confirmed deaths
Daily reported cases
40
20
0
1500
1000
500
0
Apr
May
Jun
Jul
Aug
Sep
Date
Prediction
Median
CI
IQR

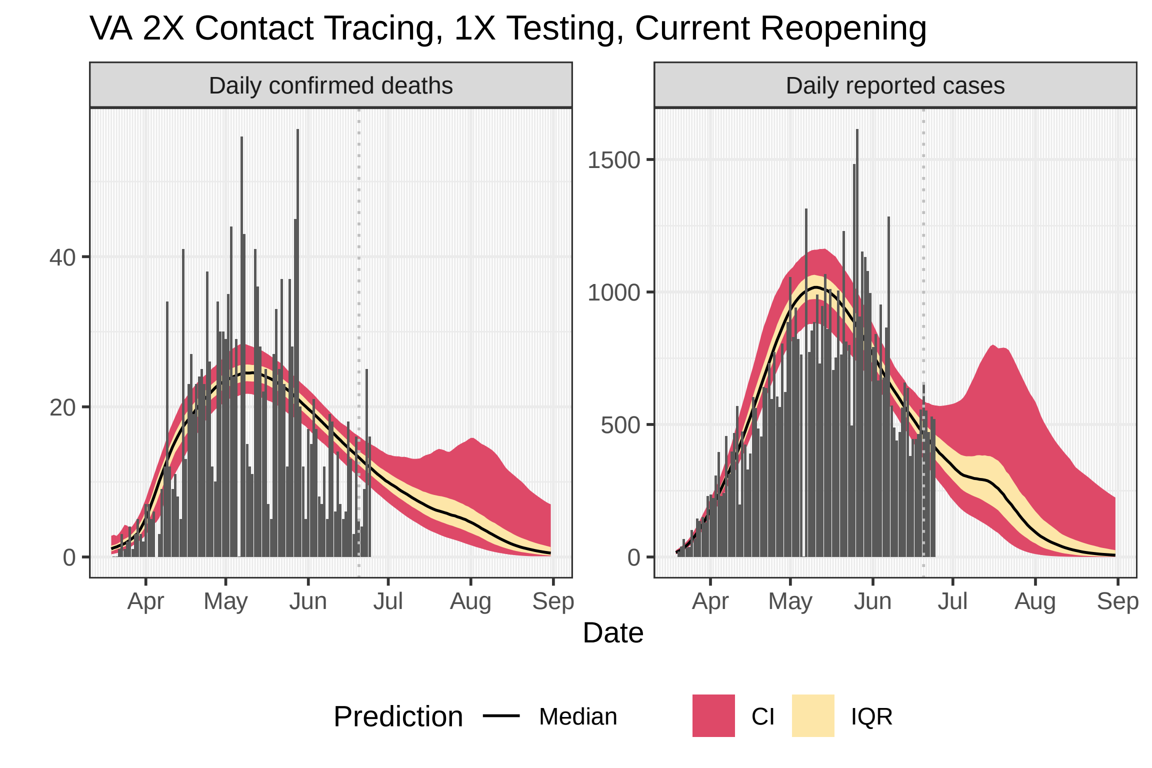
VA 2X Contact Tracing, 1X Testing, Current Reopening
Daily confirmed deaths
Daily reported cases
40
20
0
1500
1000
500
0
Apr
May
Jun
Jul
Aug
Sep
Date
Prediction
Median
CI
IQR

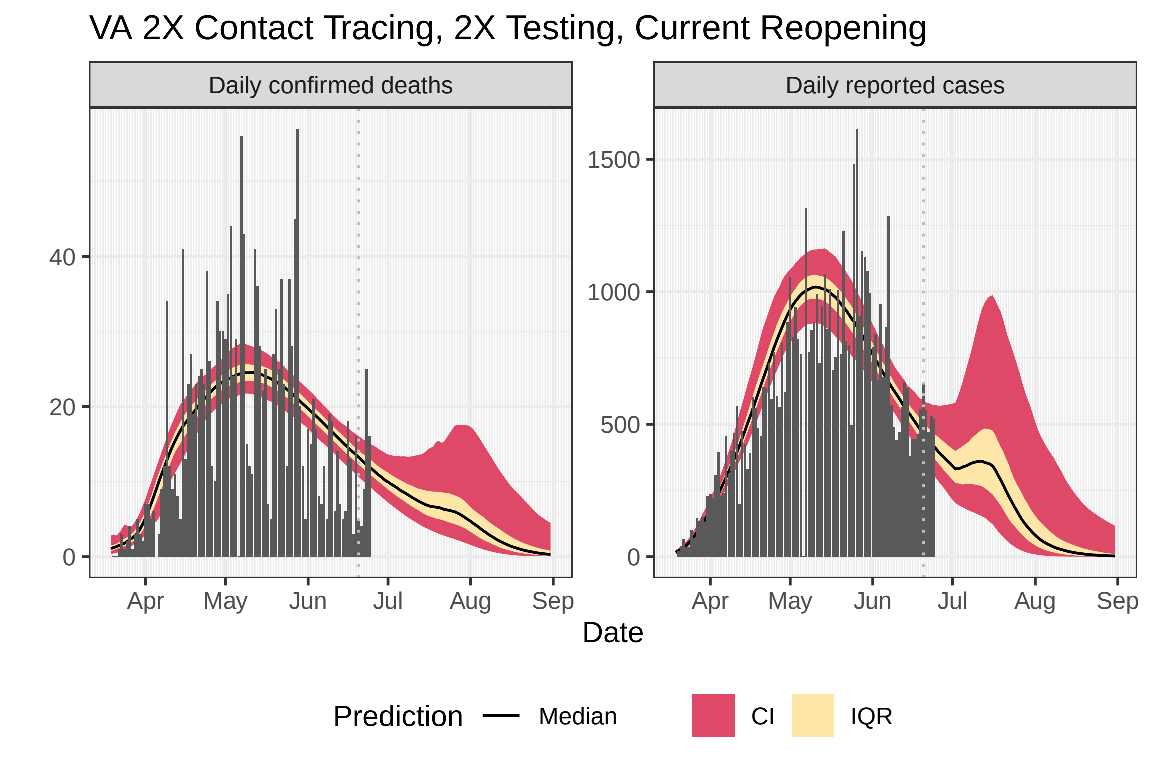

VA 2X Contact Tracing, 2X Testing, Current Reopening
Daily confirmed deaths
Daily reported cases
0
20
40
500
1000
1500
Apr
May
Jun
Jul
Aug
Sep
Date
Prediction
Median
CI
IQR

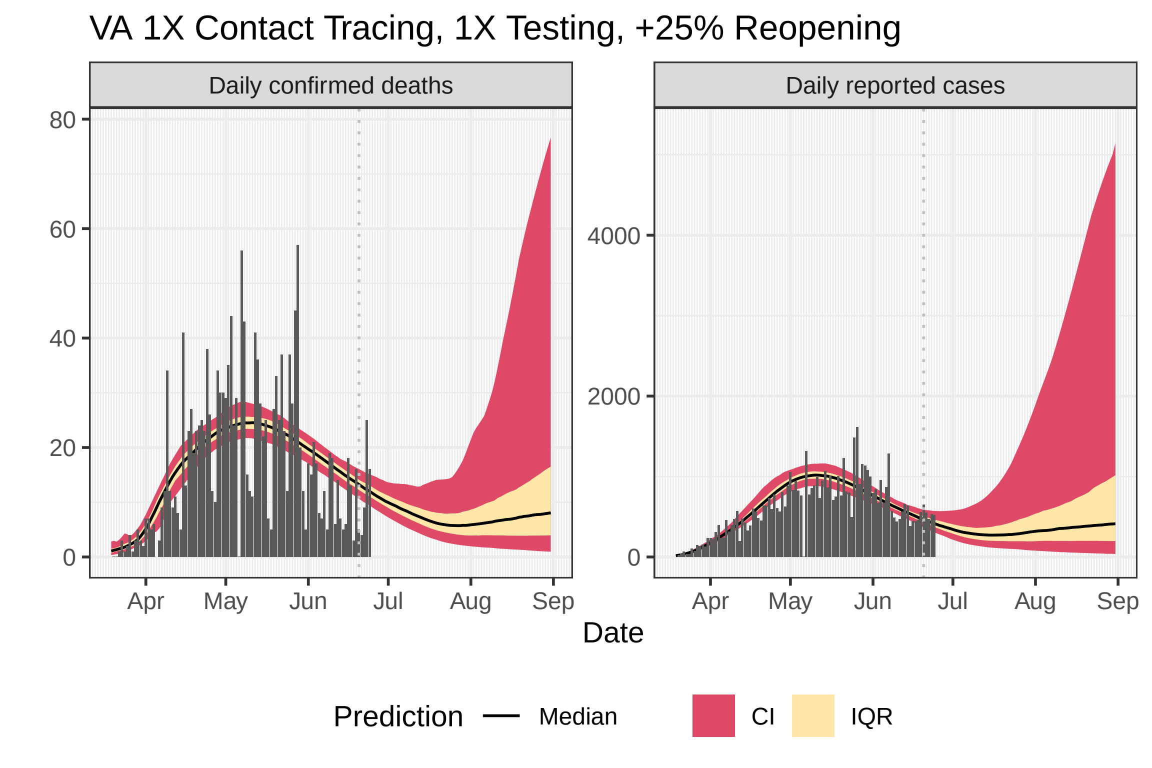
VA 1X Contact Tracing, 1X Testing, +25% Reopening
Daily confirmed deaths
Daily reported cases
80
60
40
20
0
4000
2000
0
Apr
May
Jun
Jul
Aug
Sep
Date
Prediction
Median
CI
IQR

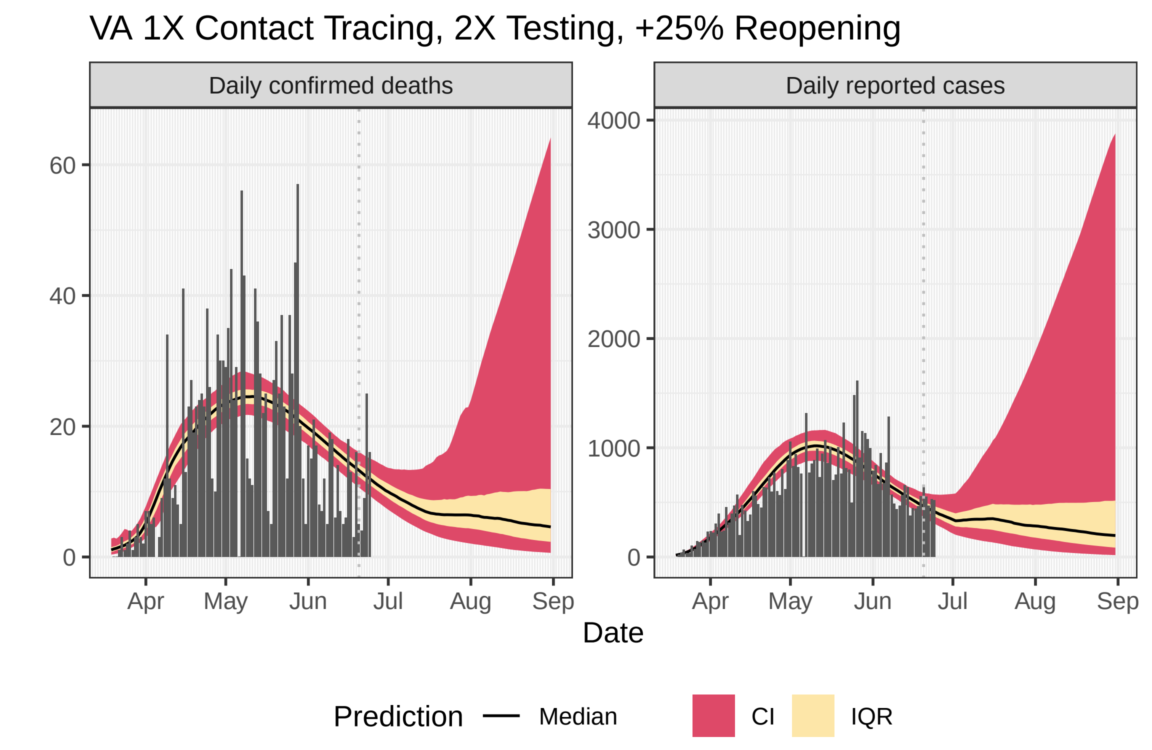

VA 1X Contact Tracing, 2X Testing, +25% Reopening
Daily confirmed deaths
Daily reported cases
0
20
40
60
0
1000
2000
3000
4000
Apr
May
Jun
Jul
Aug
Sep
Date
Prediction
Median
CI
IQR

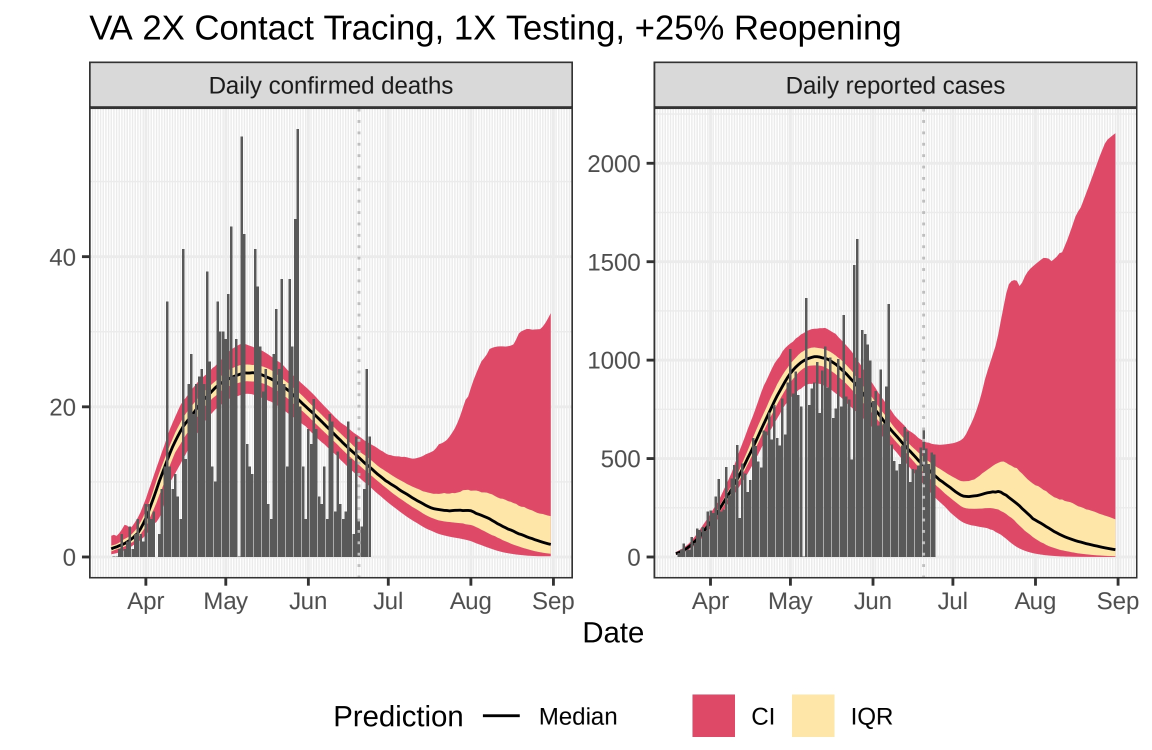

VA 2X Contact Tracing, 1X Testing, +25% Reopening
Daily confirmed deaths
Daily reported cases
0
20
40
500
1000
1500
2000
Apr
May
Jun
Jul
Aug
Sep
Date
Prediction
Median
CI
IQR

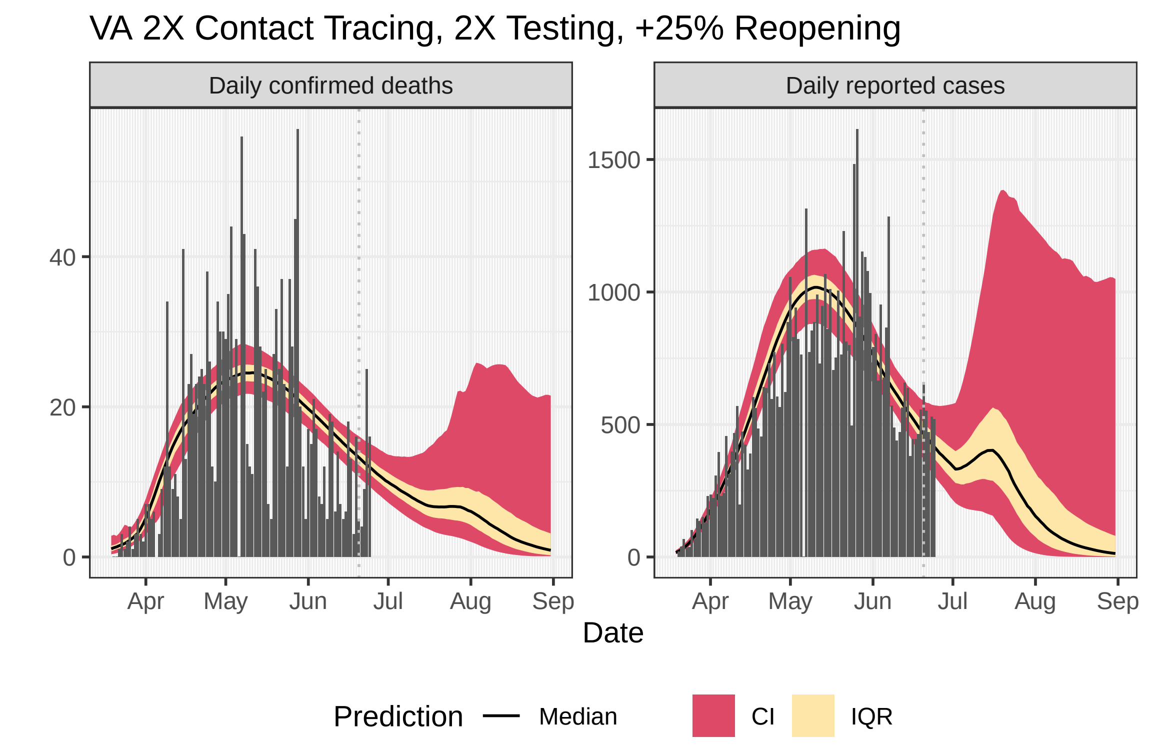

VA 2X Contact Tracing, 2X Testing, +25% Reopening
Daily confirmed deaths
Daily reported cases
0
20
40
500
1000
1500
Apr
May
Jun
Jul
Aug
Sep
Date
Prediction
Median
CI
IQR

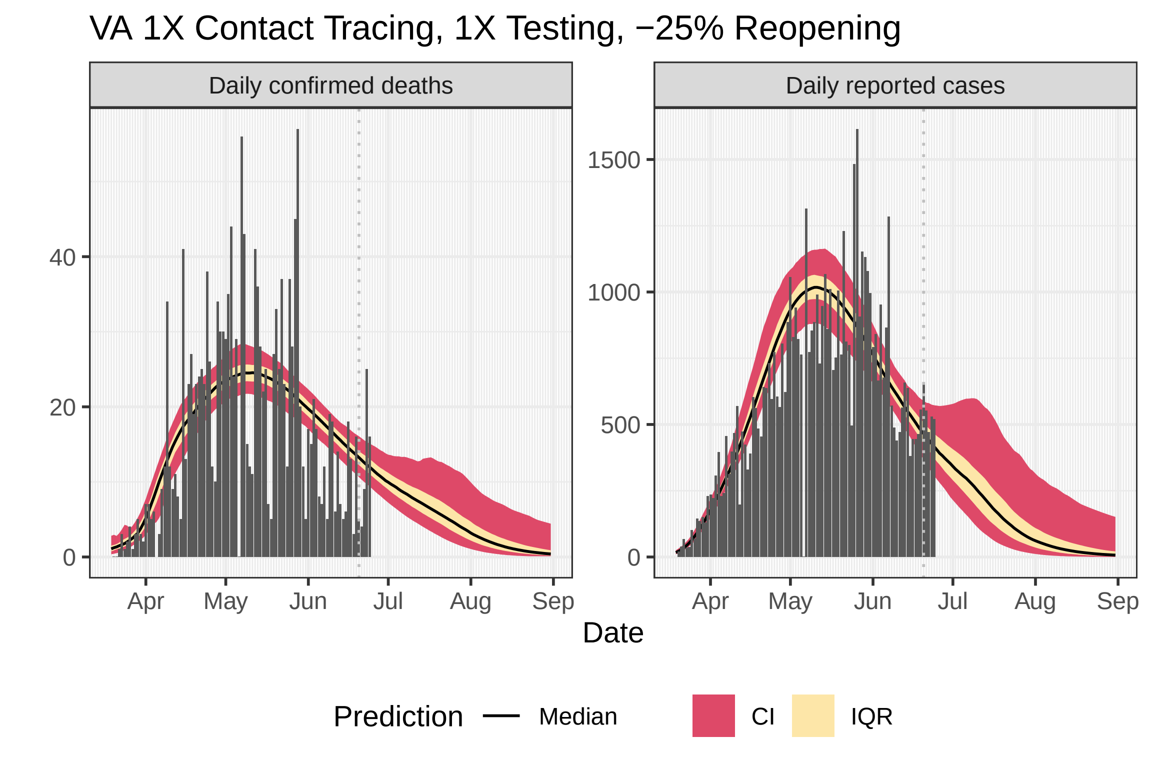

VA 1X Contact Tracing, 1X Testing, −25% Reopening
Daily confirmed deaths
Daily reported cases
0
20
40
500
1000
1500
Apr
May
Jun
Jul
Aug
Sep
Date
Prediction
Median
CI
IQR

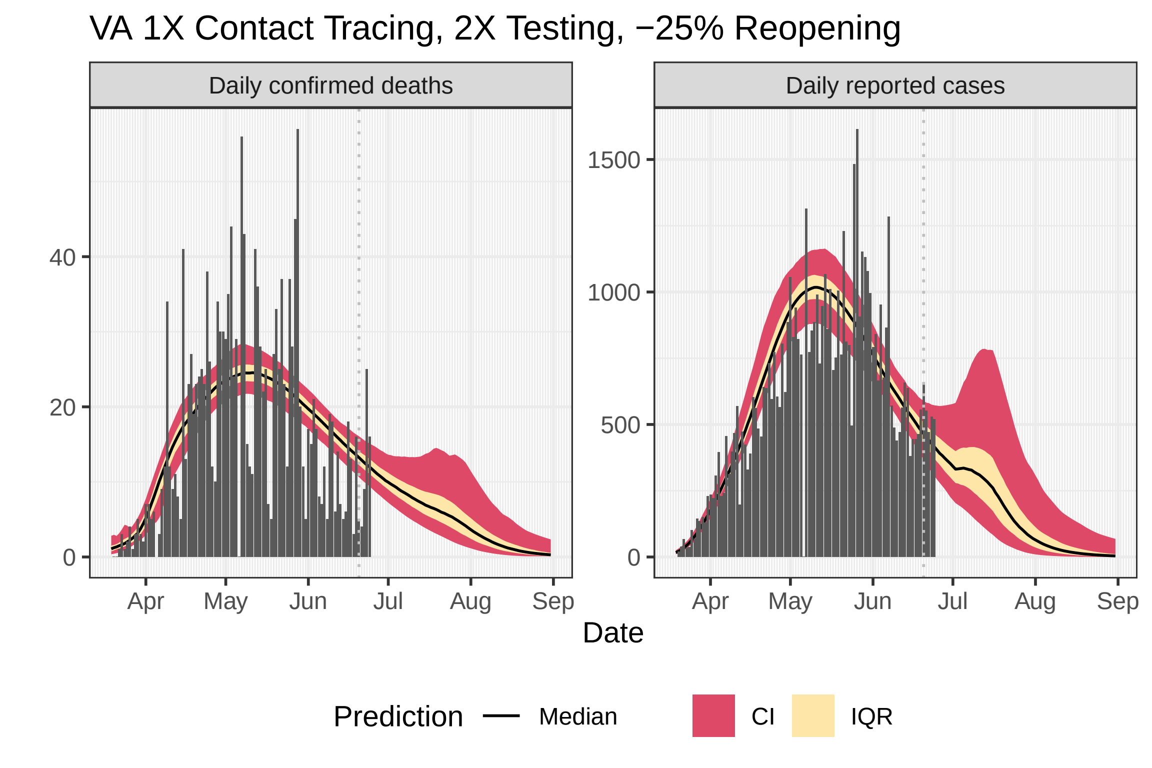
VA 1X Contact Tracing, 2X Testing, −25% Reopening
Daily confirmed deaths
Daily reported cases
0
20
40
500
1000
1500
Apr
May
Jun
Jul
Aug
Sep
Date
Prediction
Median
CI
IQR

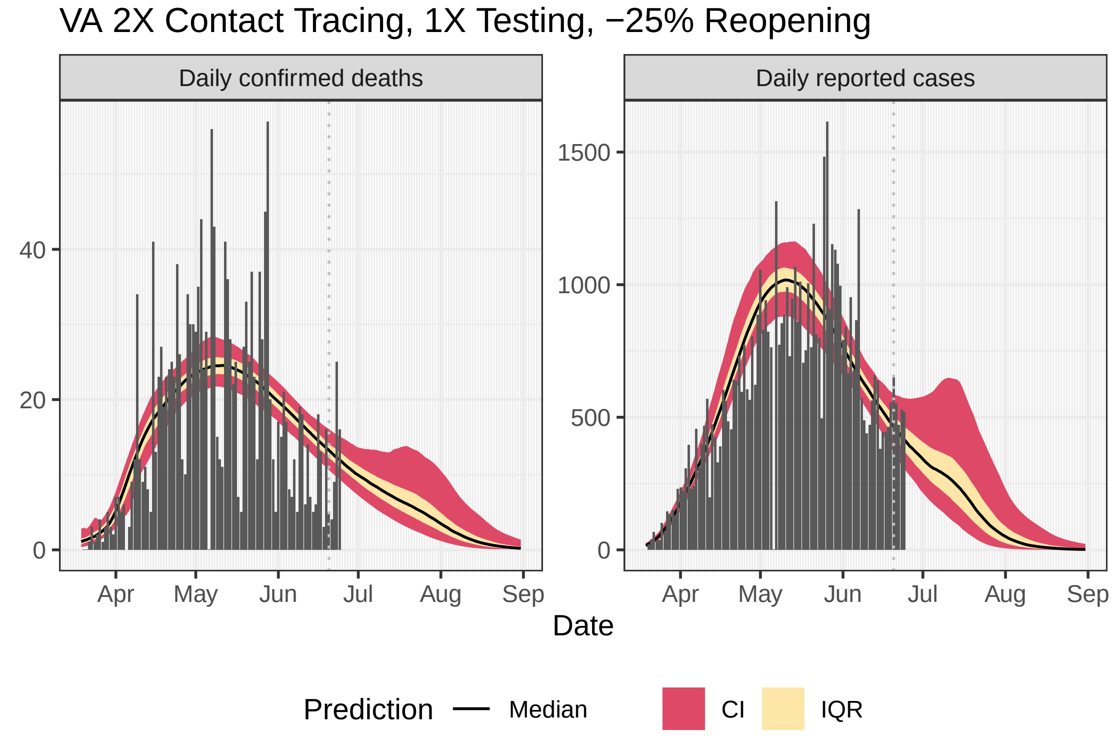

VA 2X Contact Tracing, 1X Testing, −25% Reopening
Daily confirmed deaths
Daily reported cases
0
20
40
500
1000
1500
Apr
May
Jun
Jul
Aug
Sep
Date
Prediction
Median
CI
IQR

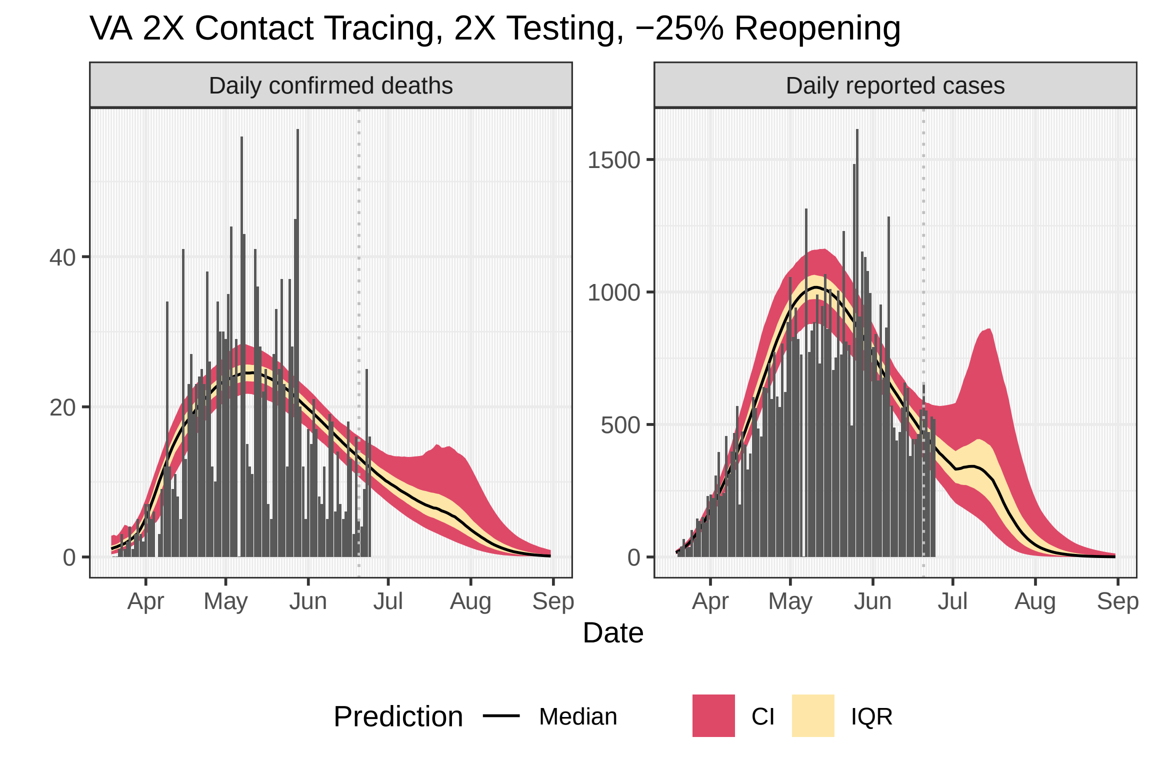
VA 2X Contact Tracing, 2X Testing, −25% Reopening
Daily confirmed deaths
Daily reported cases
0
20
40
500
1000
1500
Apr
May
Jun
Jul
Aug
Sep
Date
Prediction
Median
CI
IQR

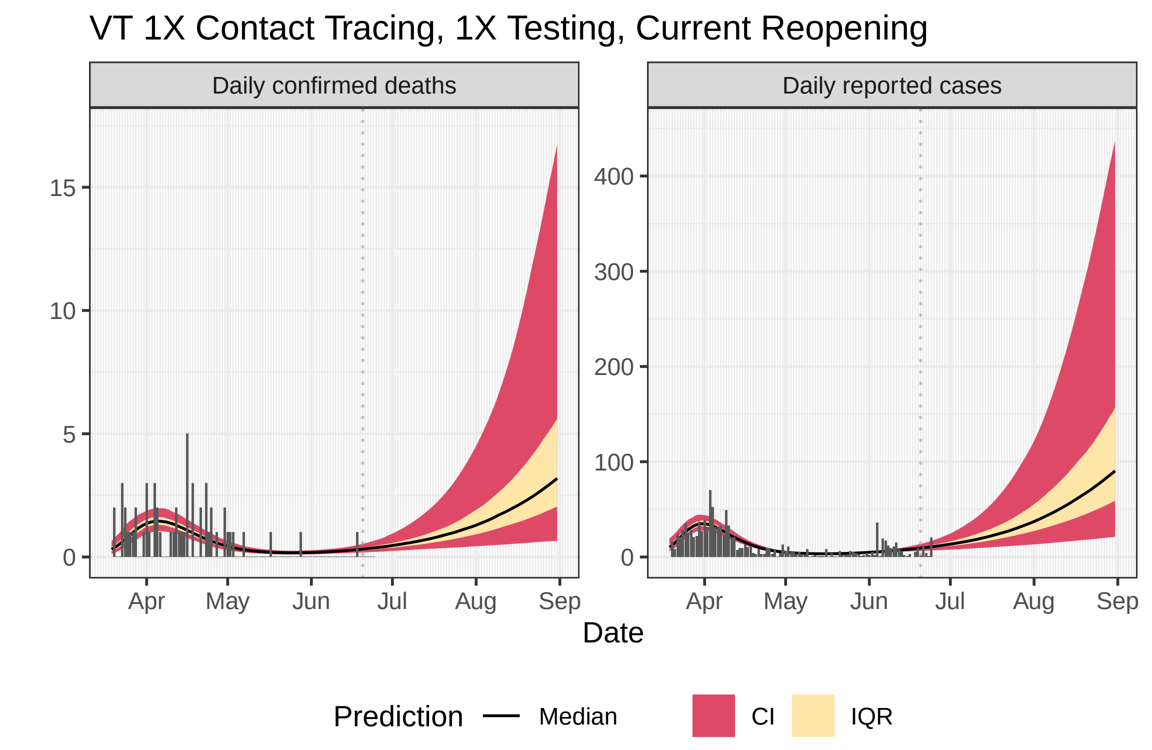

VT 1X Contact Tracing, 1X Testing, Current Reopening
Daily confirmed deaths
Daily reported cases
15
10
5
0
400
300
200
100
0
Apr
May
Jun
Jul
Aug
Sep
Date
Prediction
Median
CI
IQR

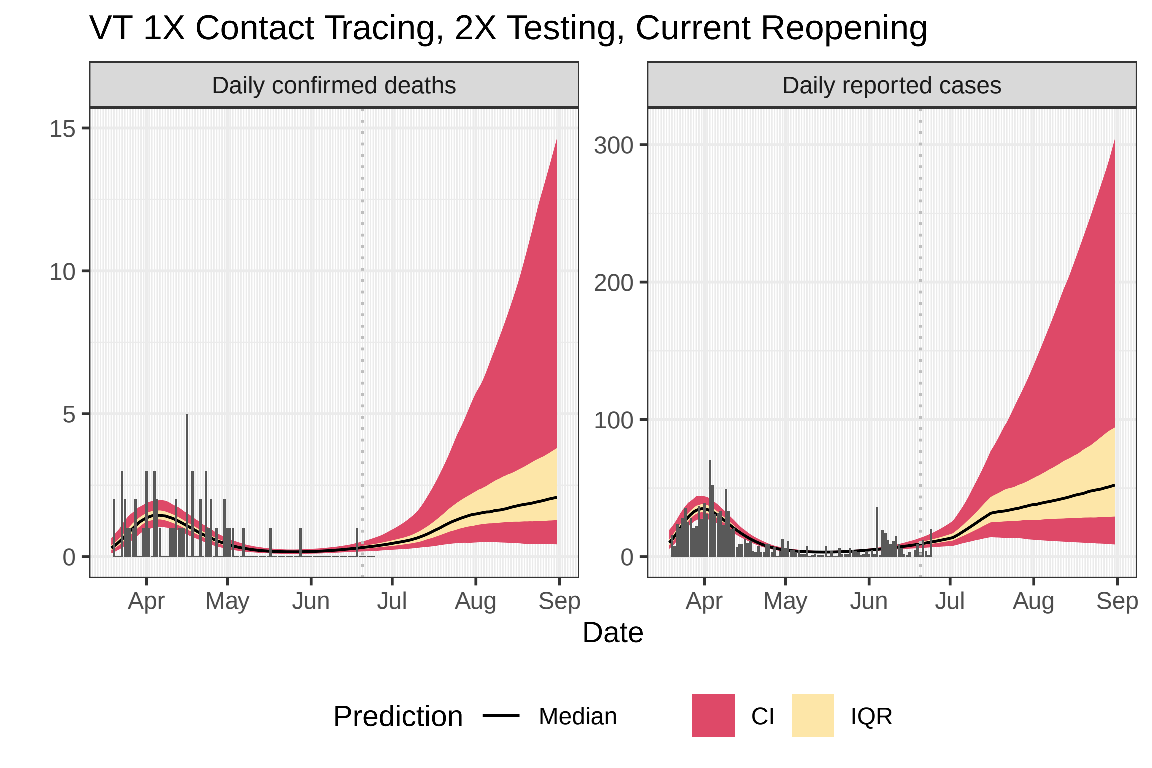

VT 1X Contact Tracing, 2X Testing, Current Reopening
Daily confirmed deaths
Daily reported cases
15
10
5
0
300
200
100
0
Apr
May
Jun
Jul
Aug
Sep
Date
Prediction
Median
CI
IQR

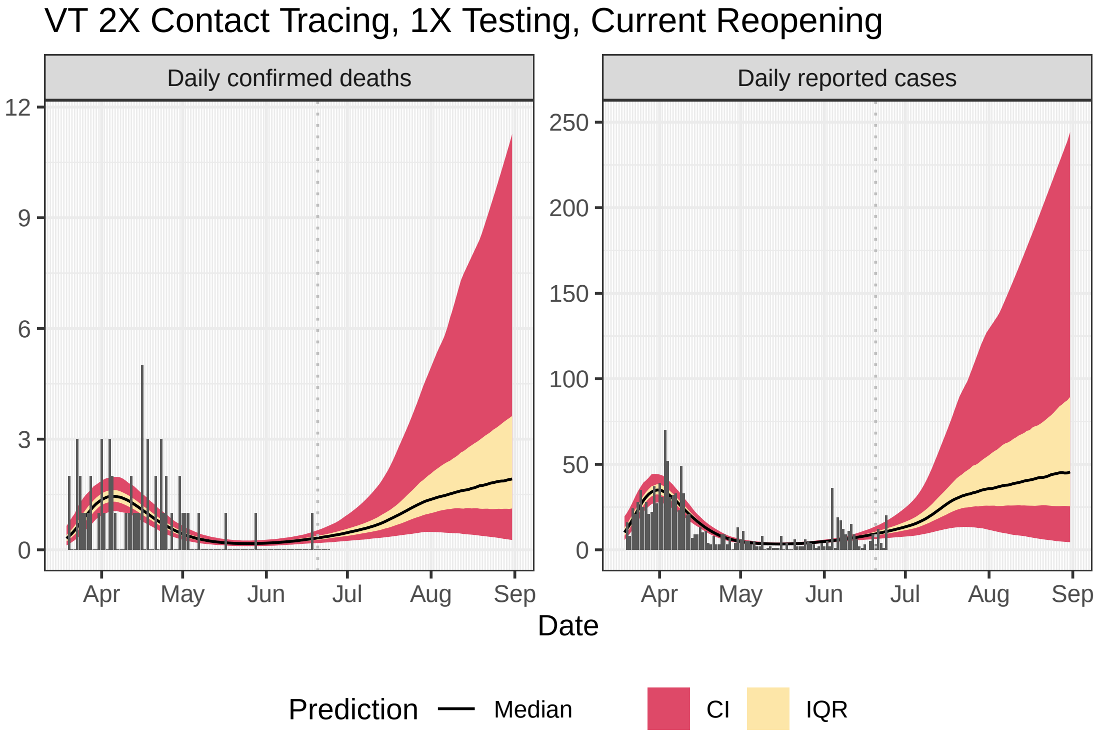
VT 2X Contact Tracing, 1X Testing, Current Reopening
Daily confirmed deaths
Daily reported cases
12
9
6
3
0
250
200
150
100
50
0
Apr
May
Jun
Jul
Aug
Sep
Date
Prediction
Median
CI
IQR

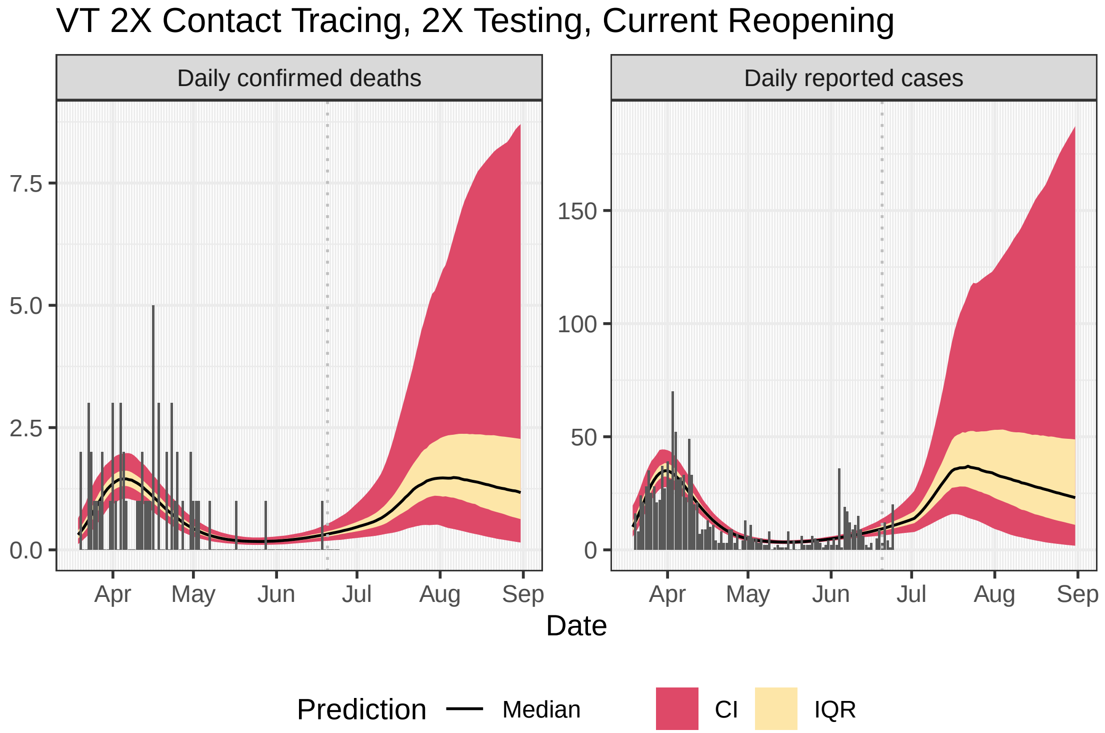

VT 2X Contact Tracing, 2X Testing, Current Reopening
Daily confirmed deaths
Daily reported cases
7.5
5.0
2.5
0.0
150
100
50
0
Apr
May
Jun
Jul
Aug
Sep
Date
Prediction
Median
CI
IQR

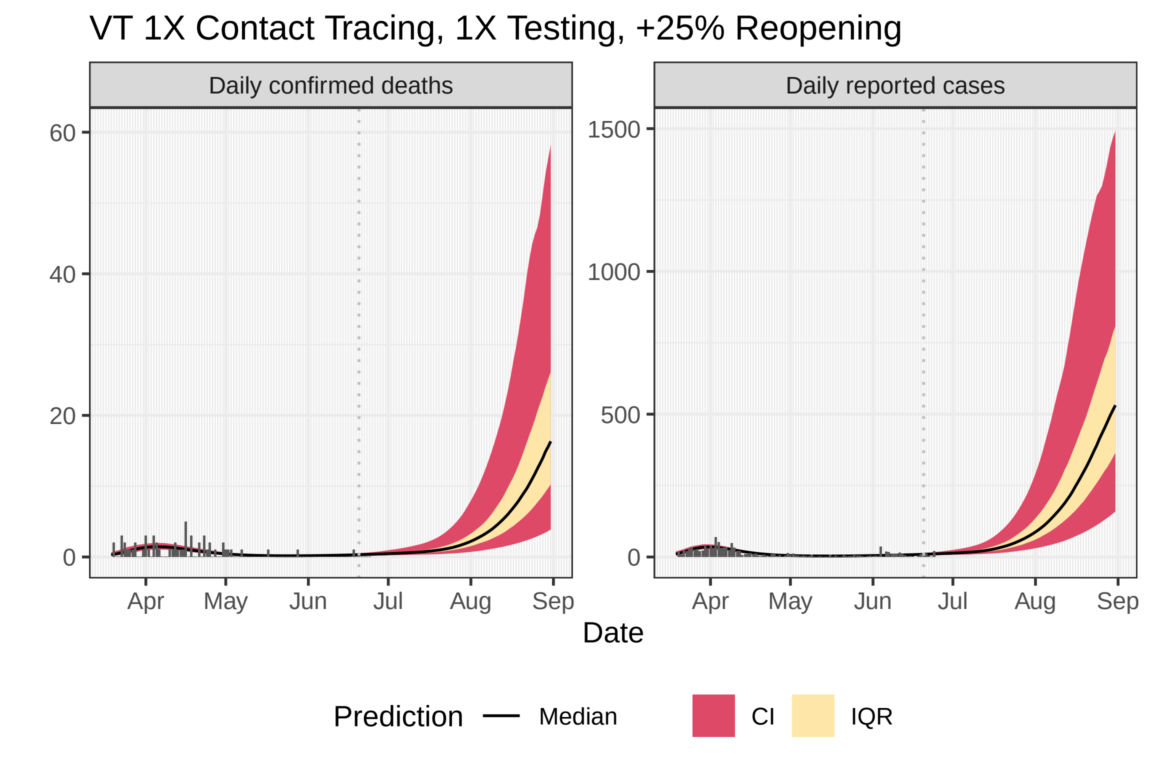

VT 1X Contact Tracing, 1X Testing, +25% Reopening
Daily confirmed deaths
Daily reported cases
60
40
20
0
1500
1000
500
0
Apr
May
Jun
Jul
Aug
Sep
Date
Prediction
Median
CI
IQR

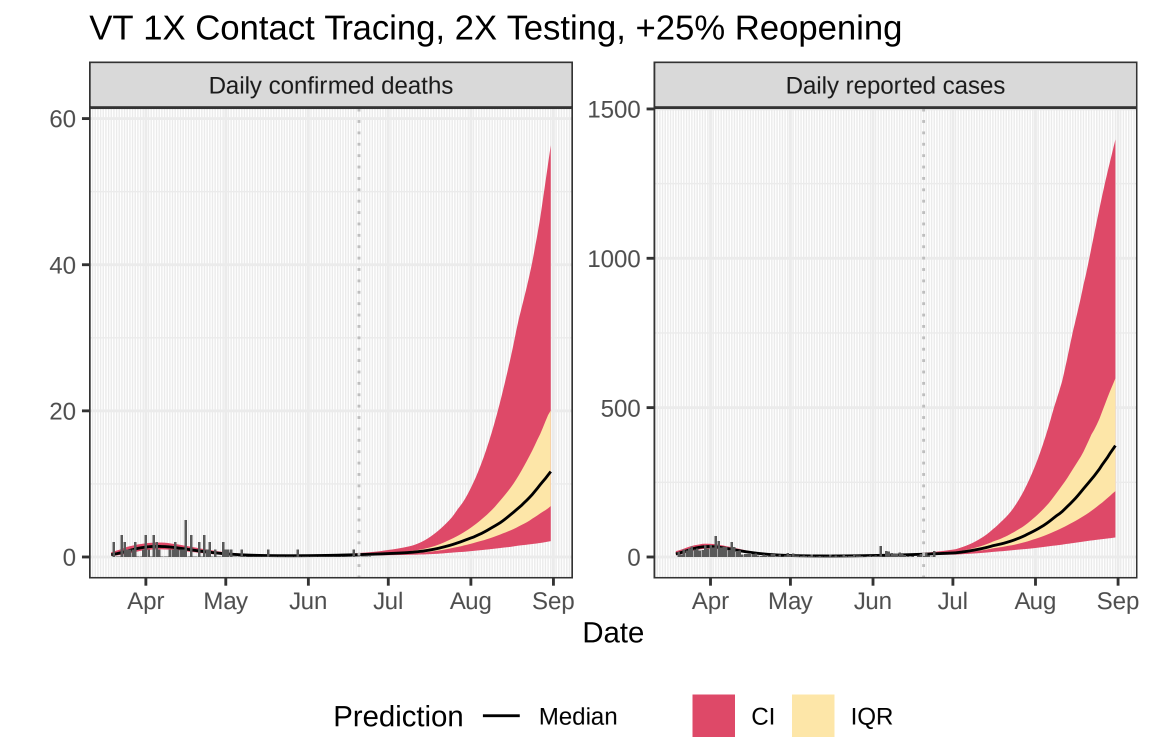
VT 1X Contact Tracing, 2X Testing, +25% Reopening
Daily confirmed deaths
Daily reported cases
60
40
20
0
1500
1000
500
0
Apr
May
Jun
Jul
Aug
Sep
Date
Prediction
Median
CI
IQR

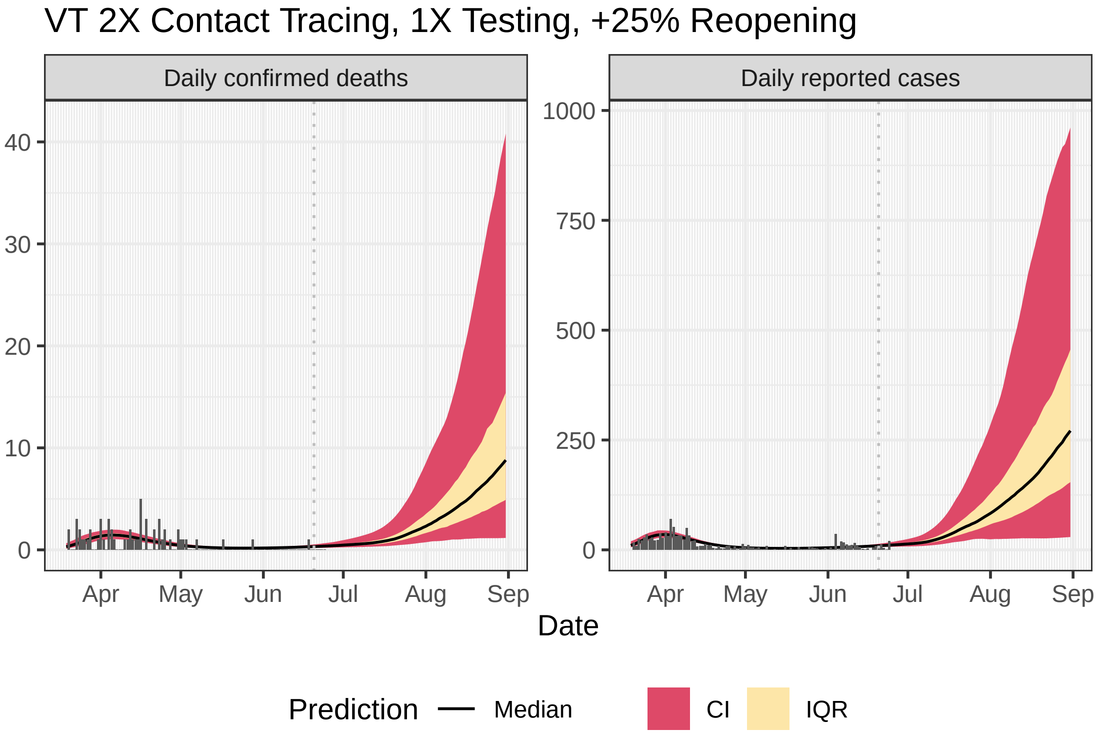
VT 2X Contact Tracing, 1X Testing, +25% Reopening
Daily confirmed deaths
Daily reported cases
0
10
20
30
40
0
250
500
750
1000
Apr
May
Jun
Jul
Aug
Sep
Date
Prediction
Median
CI
IQR

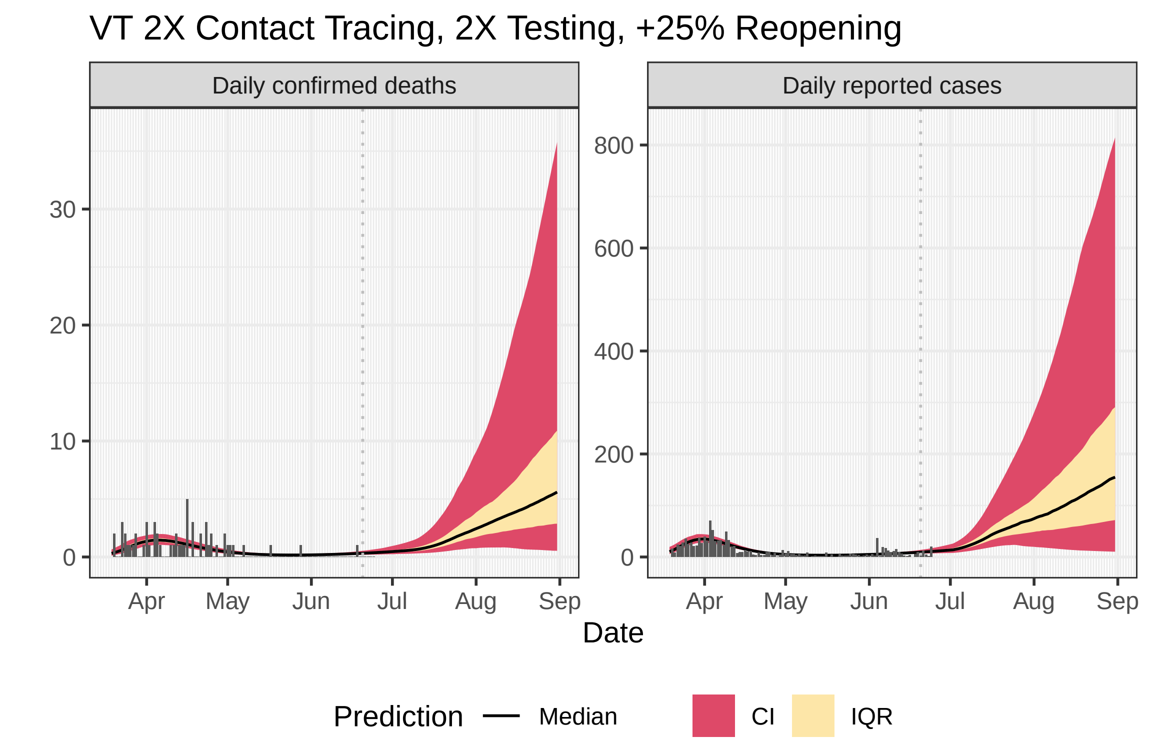

VT 2X Contact Tracing, 2X Testing, +25% Reopening
Daily confirmed deaths
Daily reported cases
0
10
20
30
0
200
400
600
800
Apr
May
Jun
Jul
Aug
Sep
Date
Prediction
Median
CI
IQR

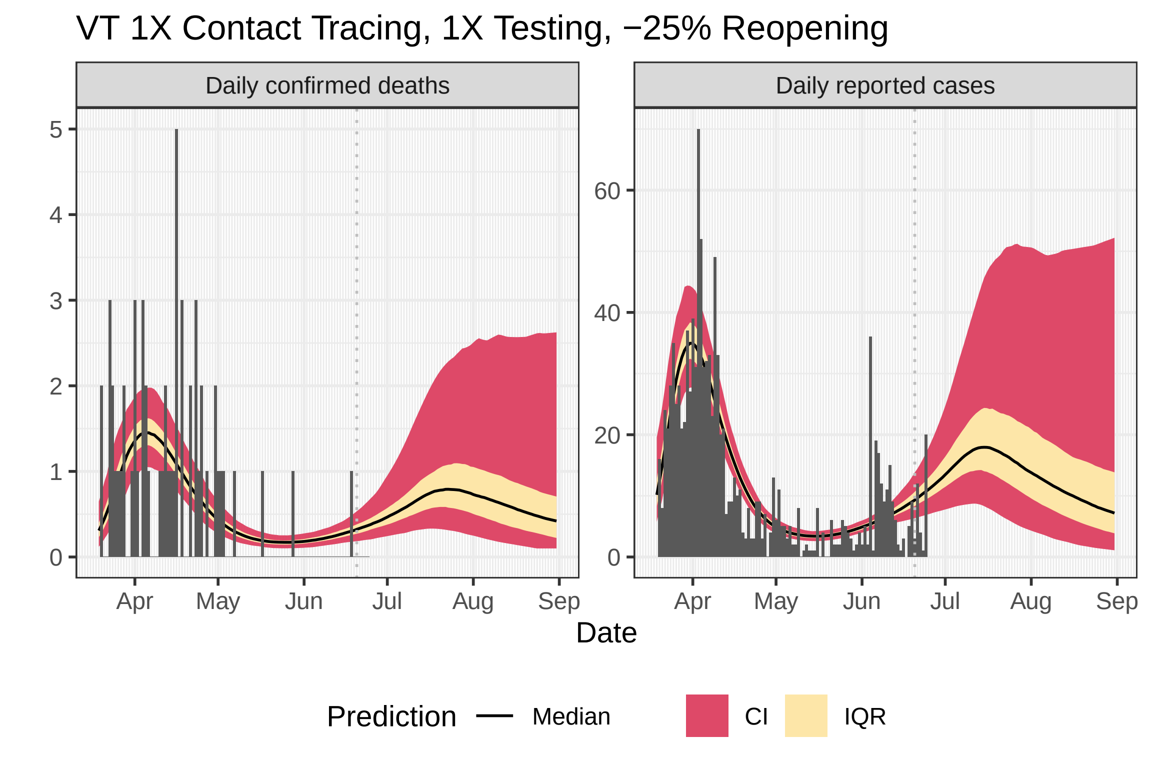
VT 1X Contact Tracing, 1X Testing, −25% Reopening
Daily confirmed deaths
Daily reported cases
0
1
2
3
4
5
0
20
40
60
Apr
May
Jun
Jul
Aug
Sep
Date
Prediction
Median
CI
IQR

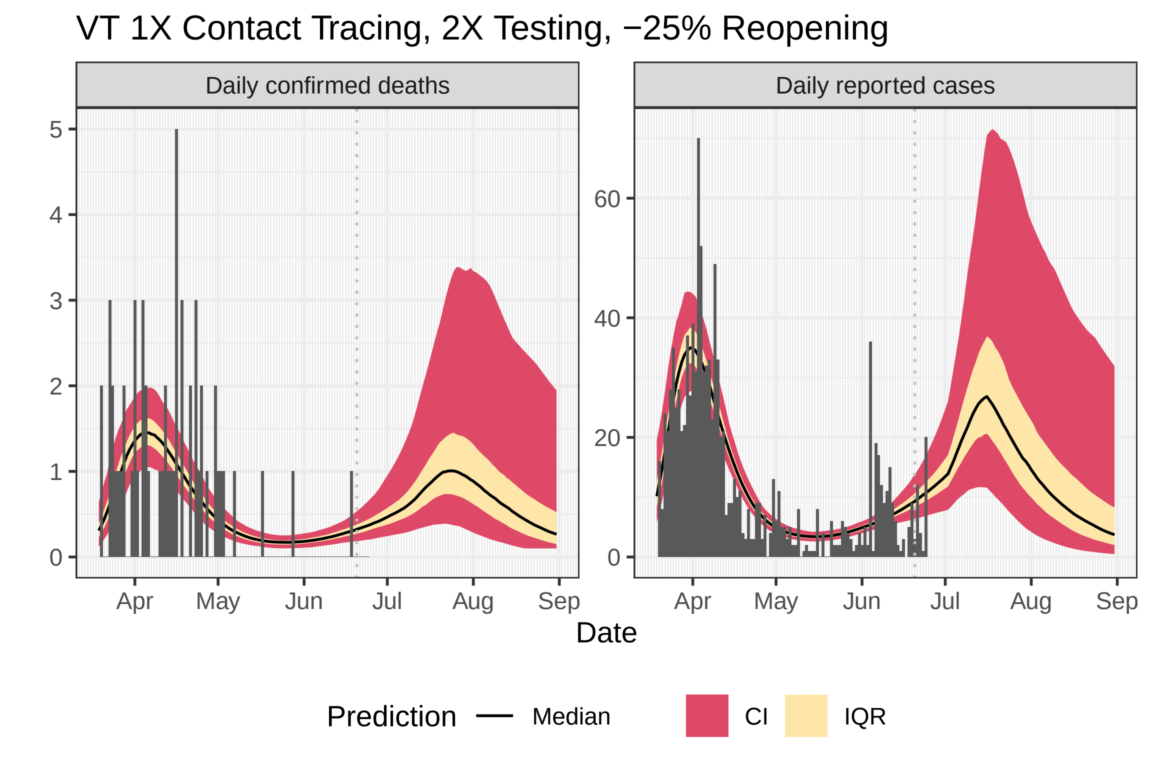

VT 1X Contact Tracing, 2X Testing, −25% Reopening
Daily confirmed deaths
Daily reported cases
0
1
2
3
4
5
0
20
40
60
Apr
May
Jun
Jul
Aug
Sep
Date
Prediction
Median
CI
IQR

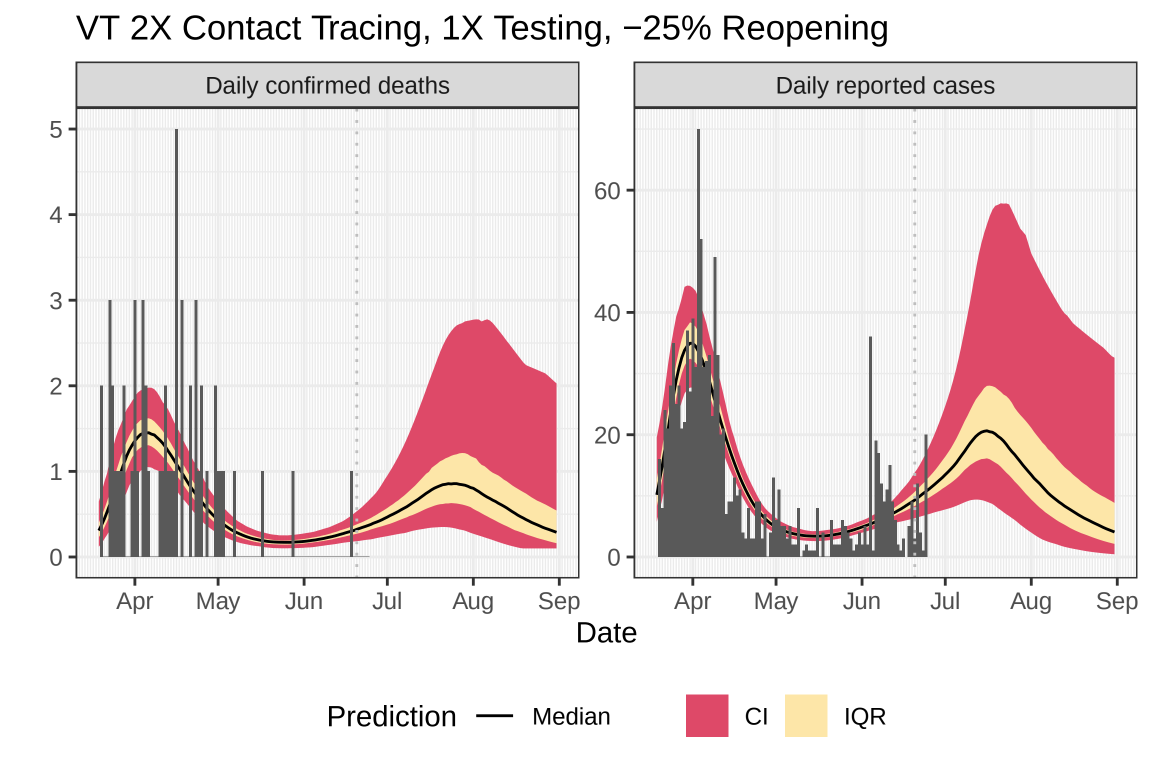

VT 2X Contact Tracing, 1X Testing, −25% Reopening
Daily confirmed deaths
Daily reported cases
5
4
3
2
1
0
60
40
20
0
Apr
May
Jun
Jul
Aug
Sep
Date
Prediction
Median
CI
IQR

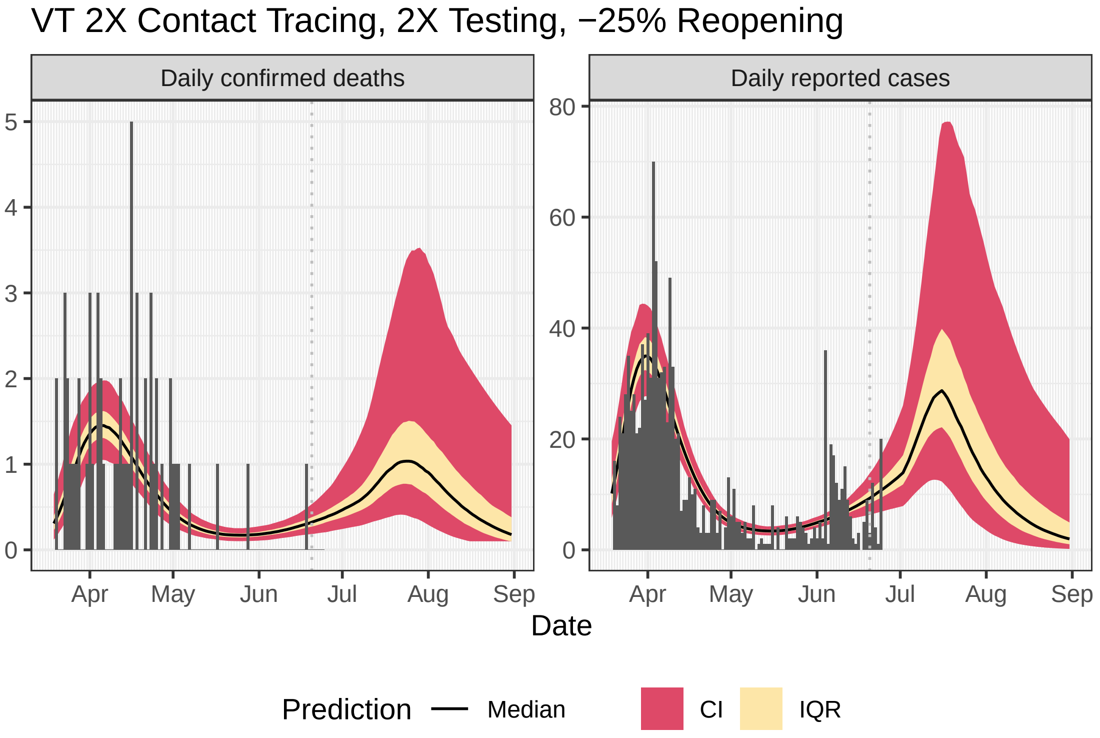

VT 2X Contact Tracing, 2X Testing, −25% Reopening
Daily confirmed deaths
Daily reported cases
0
1
2
3
4
5
0
20
40
60
80
Apr
May
Jun
Jul
Aug
Sep
Date
Prediction
Median
CI
IQR

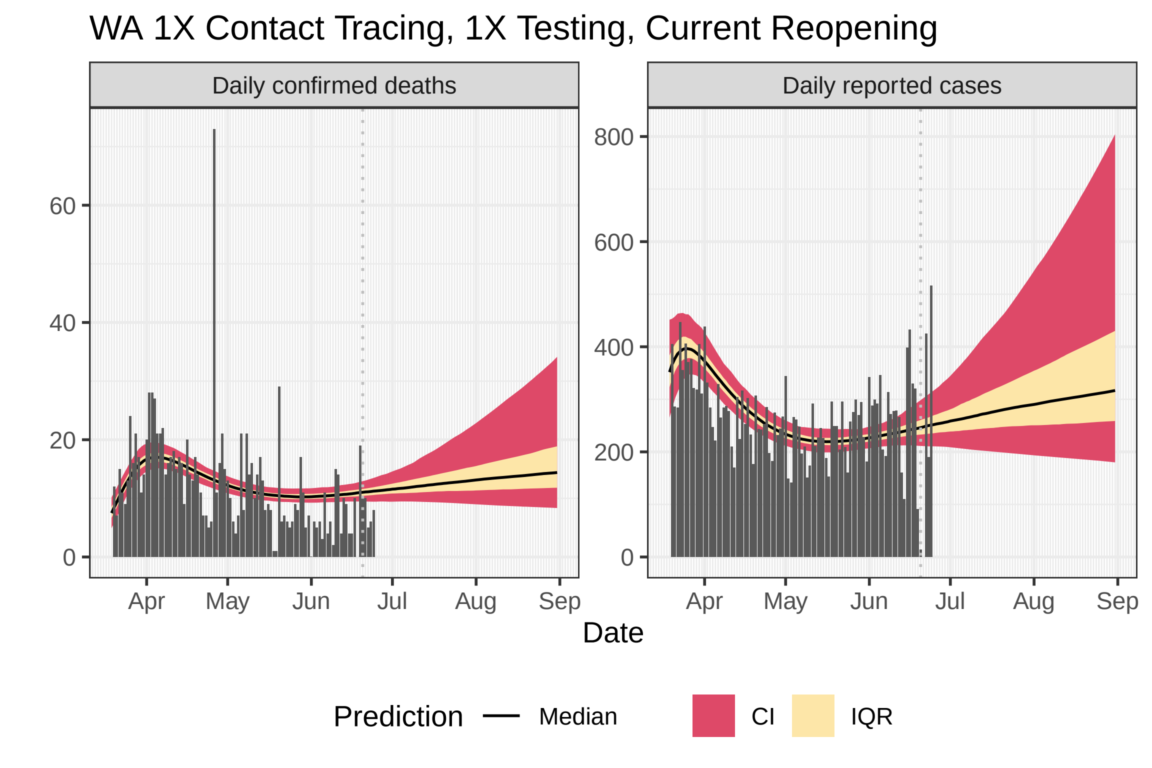
WA 1X Contact Tracing, 1X Testing, Current Reopening
Daily confirmed deaths
Daily reported cases
0
20
40
60
200
400
600
800
Apr
May
Jun
Jul
Aug
Sep
Date
Prediction
Median
CI
IQR

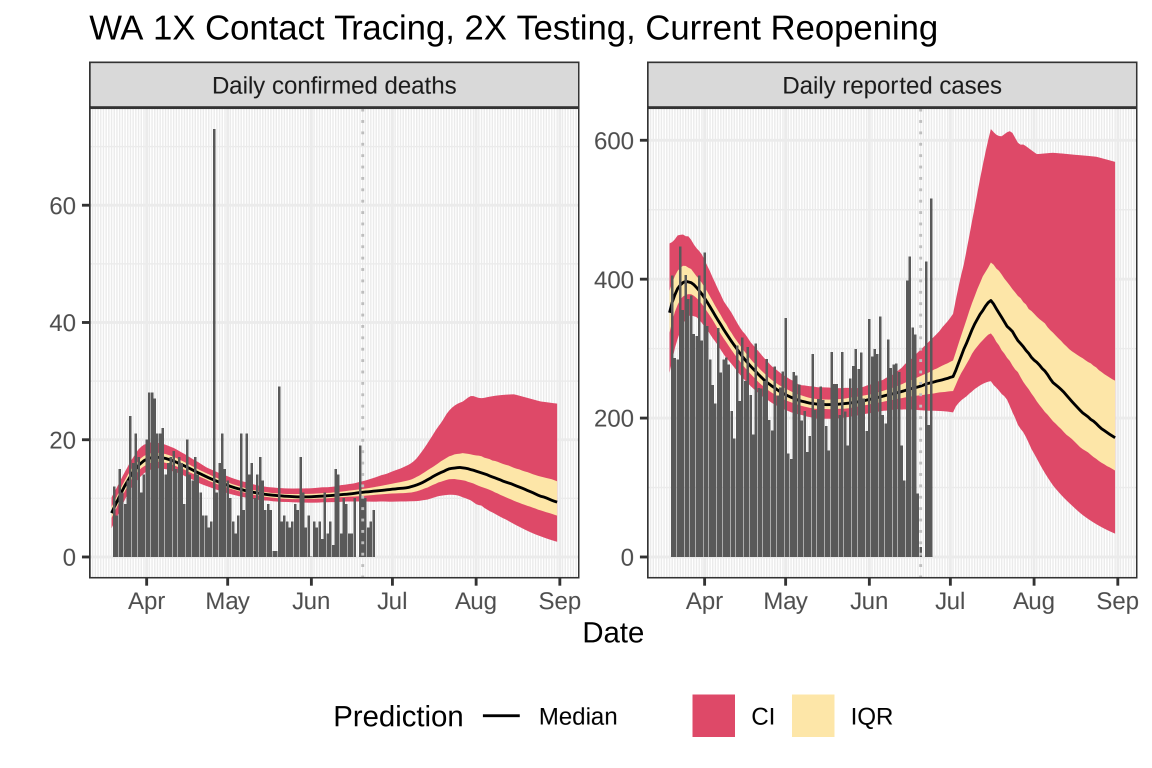

WA 1X Contact Tracing, 2X Testing, Current Reopening
Daily confirmed deaths
Daily reported cases
0
20
40
60
0
200
400
600
Apr
May
Jun
Jul
Aug
Sep
Date
Prediction
Median
CI
IQR

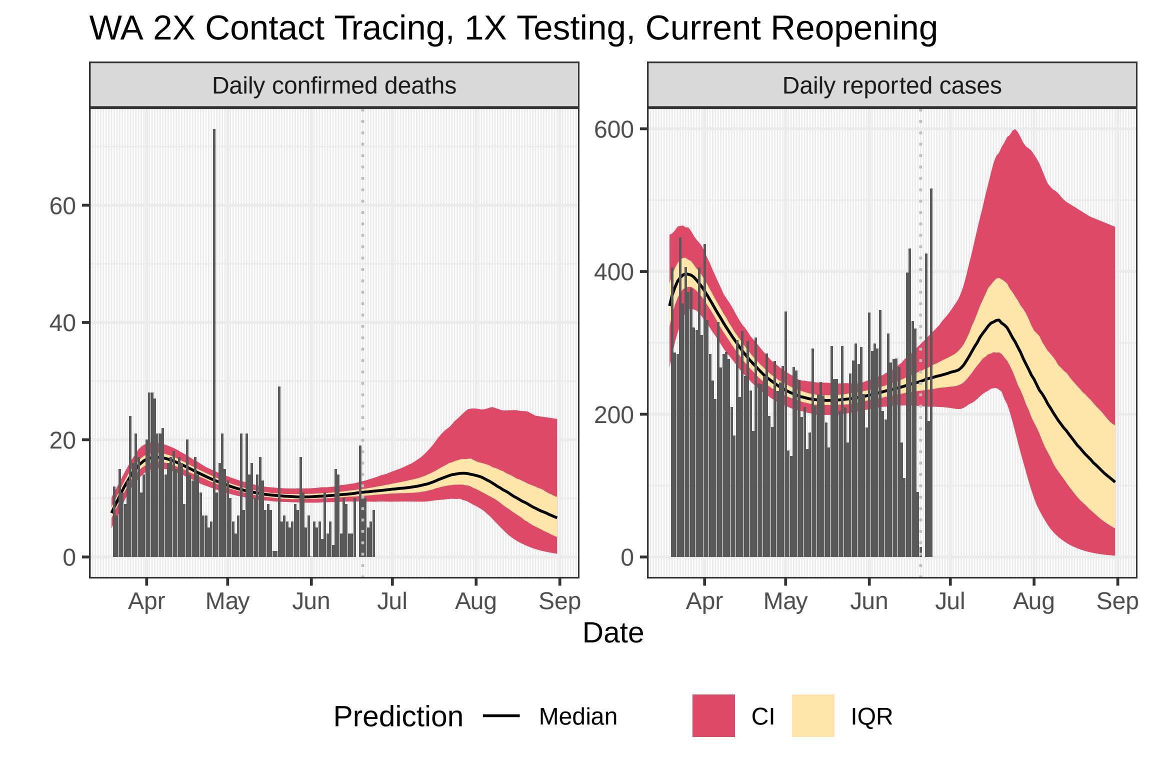
WA 2X Contact Tracing, 1X Testing, Current Reopening
Daily confirmed deaths
Daily reported cases
0
20
40
60
200
400
600
Apr
May
Jun
Jul
Aug
Sep
Date
Prediction
Median
CI
IQR

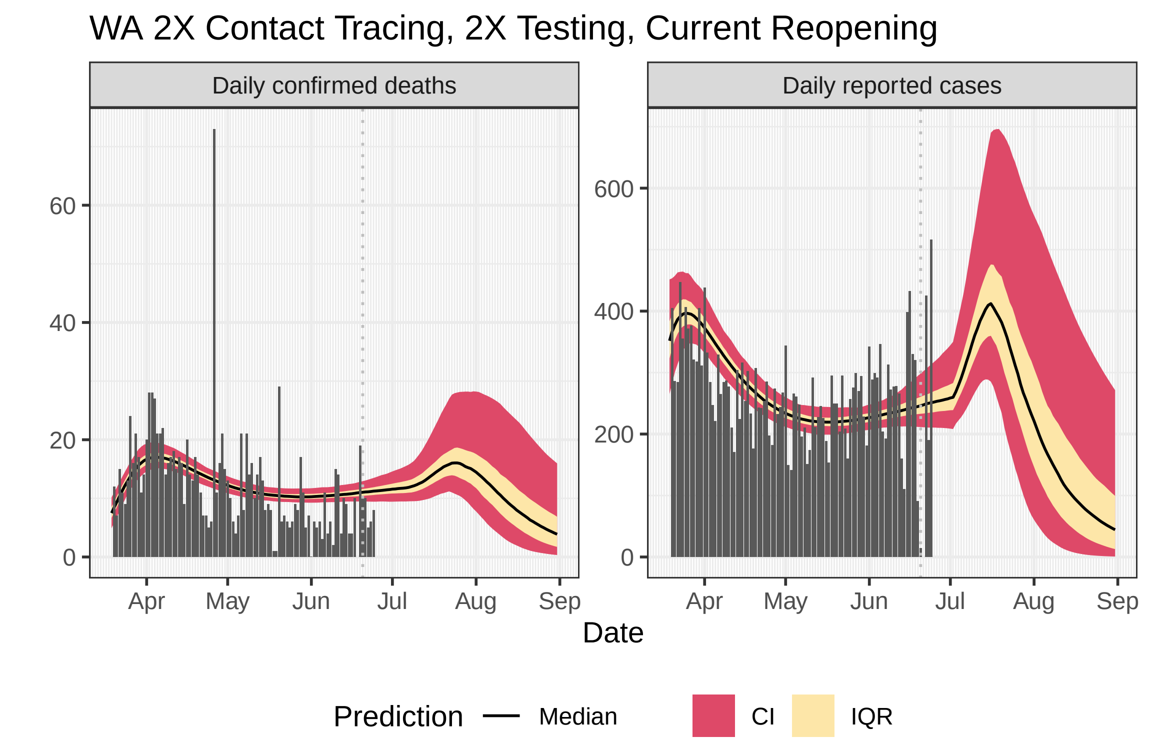
WA 2X Contact Tracing, 2X Testing, Current Reopening
Daily confirmed deaths
Daily reported cases
0
20
40
60
0
200
400
600
Apr
May
Jun
Jul
Aug
Sep
Date
Prediction
Median
CI
IQR

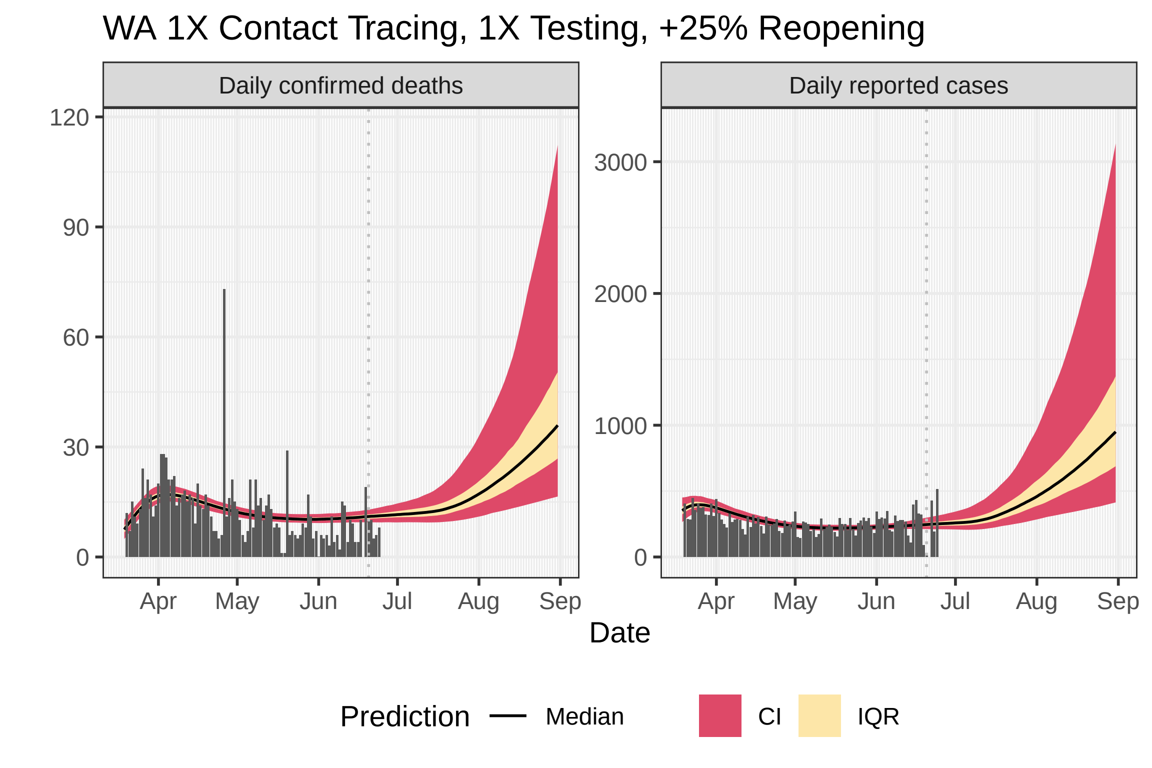
WA 1X Contact Tracing, 1X Testing, +25% Reopening
Daily confirmed deaths
Daily reported cases
120
90
60
30
0
3000
2000
1000
0
Apr
May
Jun
Jul
Aug
Sep
Date
Prediction
Median
CI
IQR

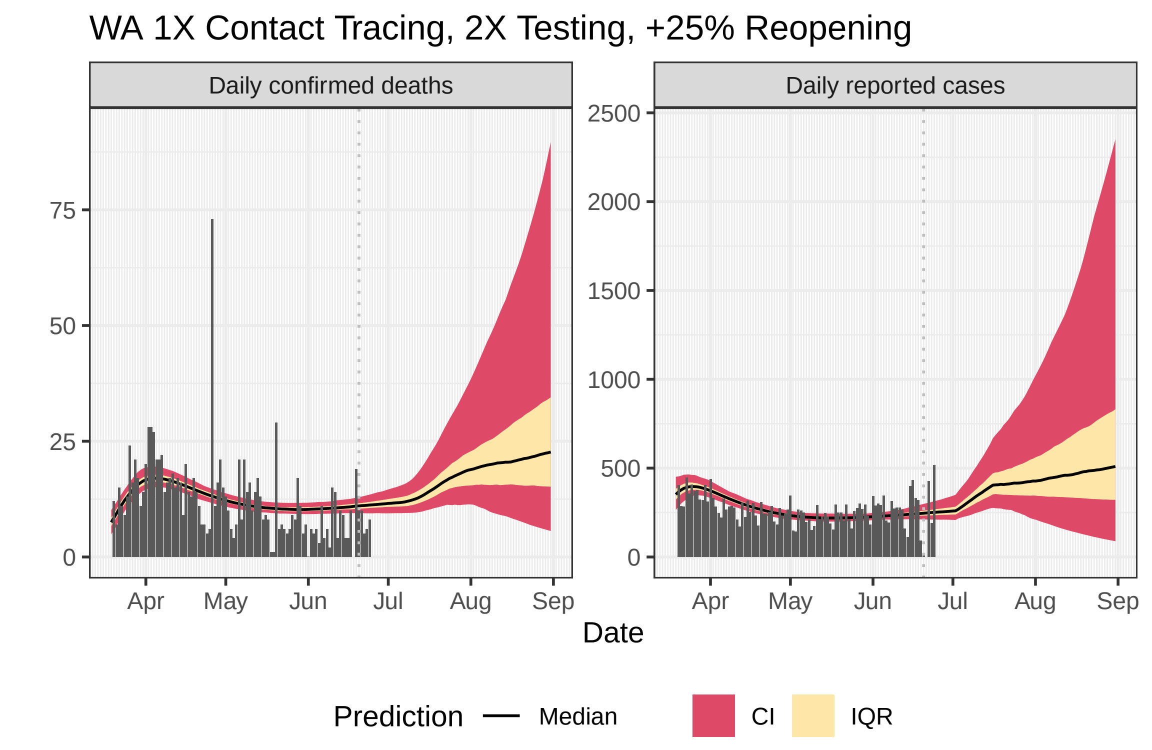

WA 1X Contact Tracing, 2X Testing, +25% Reopening
Daily confirmed deaths
Daily reported cases
0
25
50
75
0
500
1000
1500
2000
2500
Apr
May
Jun
Jul
Aug
Sep
Date
Prediction
Median
CI
IQR

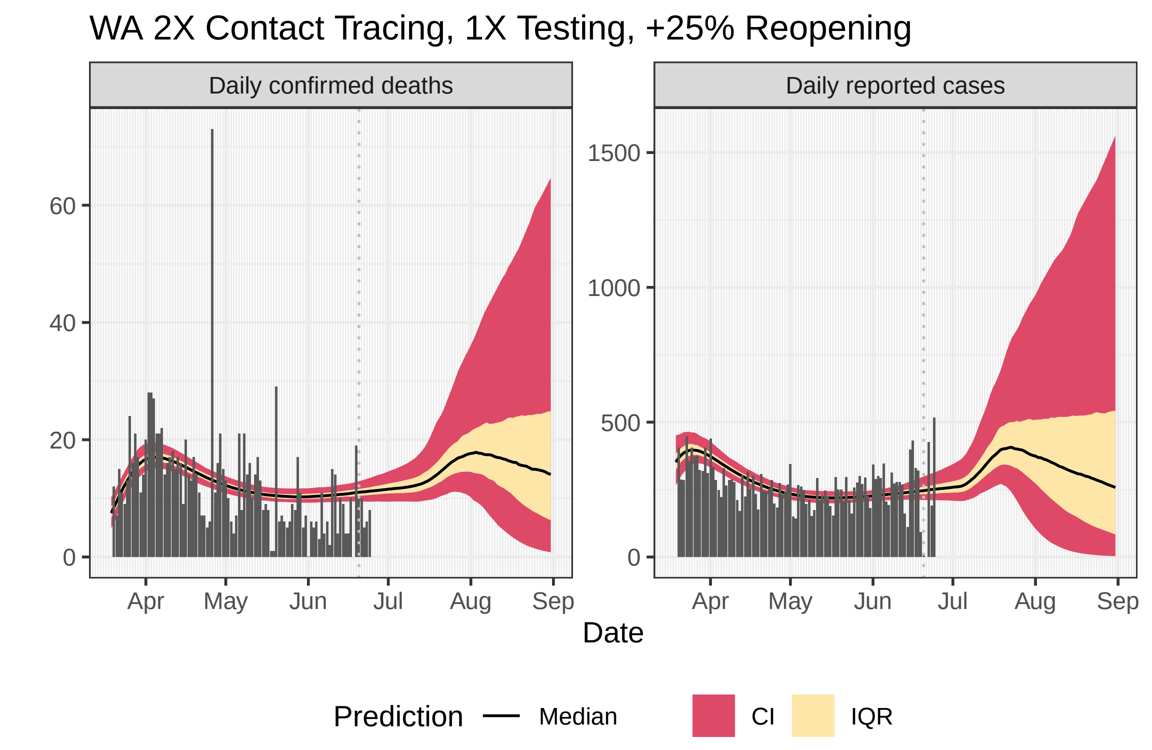

WA 2X Contact Tracing, 1X Testing, +25% Reopening
Daily confirmed deaths
Daily reported cases
0
20
40
60
0
500
1000
1500
Apr
May
Jun
Jul
Aug
Sep
Date
Prediction
Median
CI
IQR

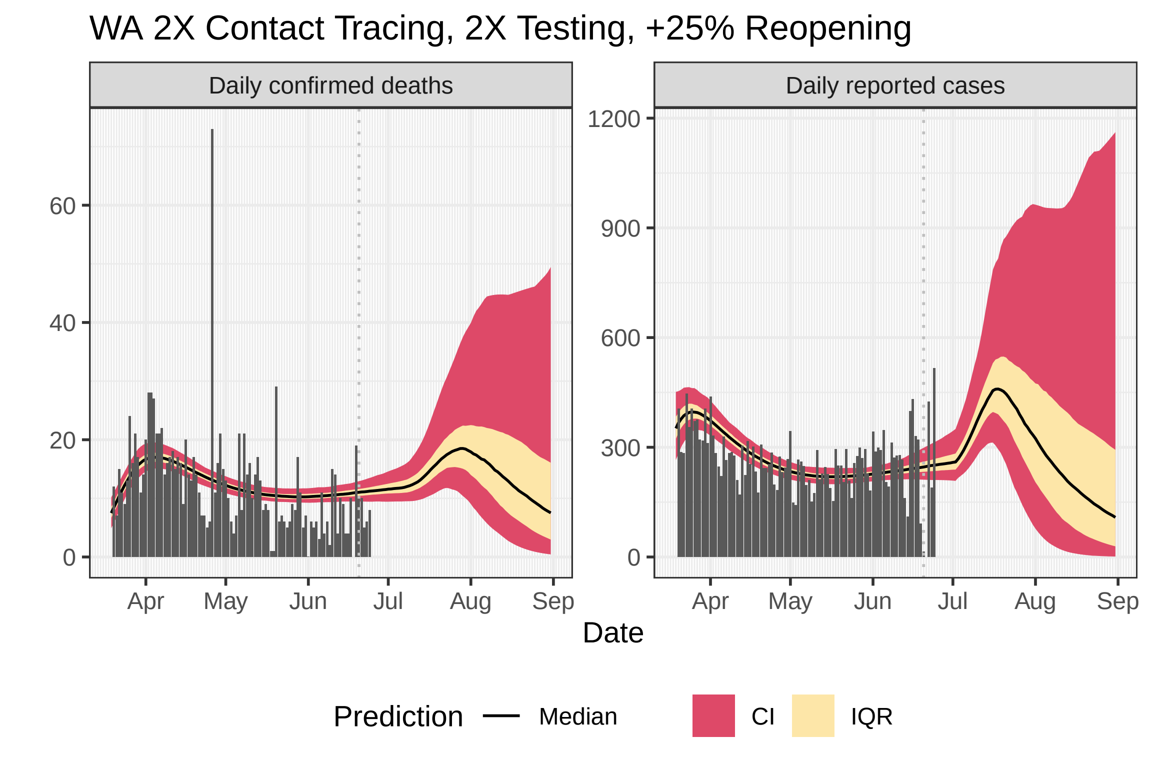

WA 2X Contact Tracing, 2X Testing, +25% Reopening
Daily confirmed deaths
Daily reported cases
0
20
40
60
0
300
600
900
1200
Apr
May
Jun
Jul
Aug
Sep
Date
Prediction
Median
CI
IQR

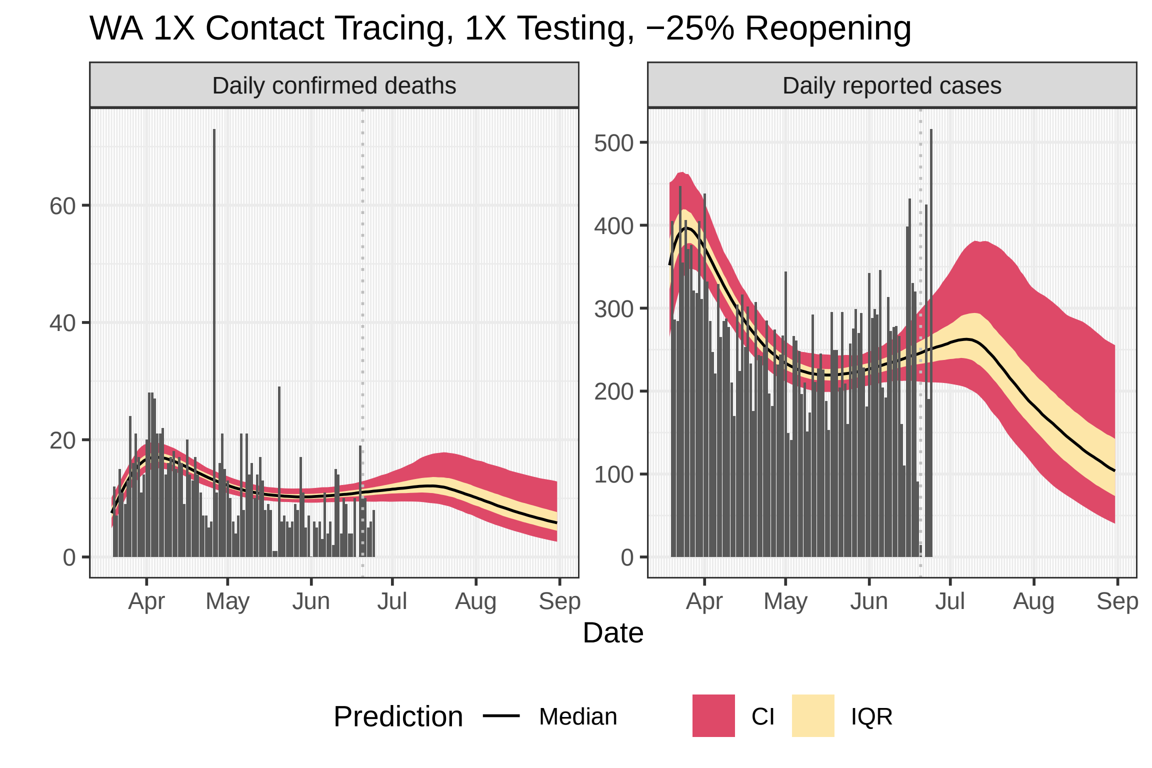

WA 1X Contact Tracing, 1X Testing, −25% Reopening
Daily confirmed deaths
Daily reported cases
0
20
40
60
0
100
200
300
400
500
Apr
May
Jun
Jul
Aug
Sep
Date
Prediction
Median
CI
IQR

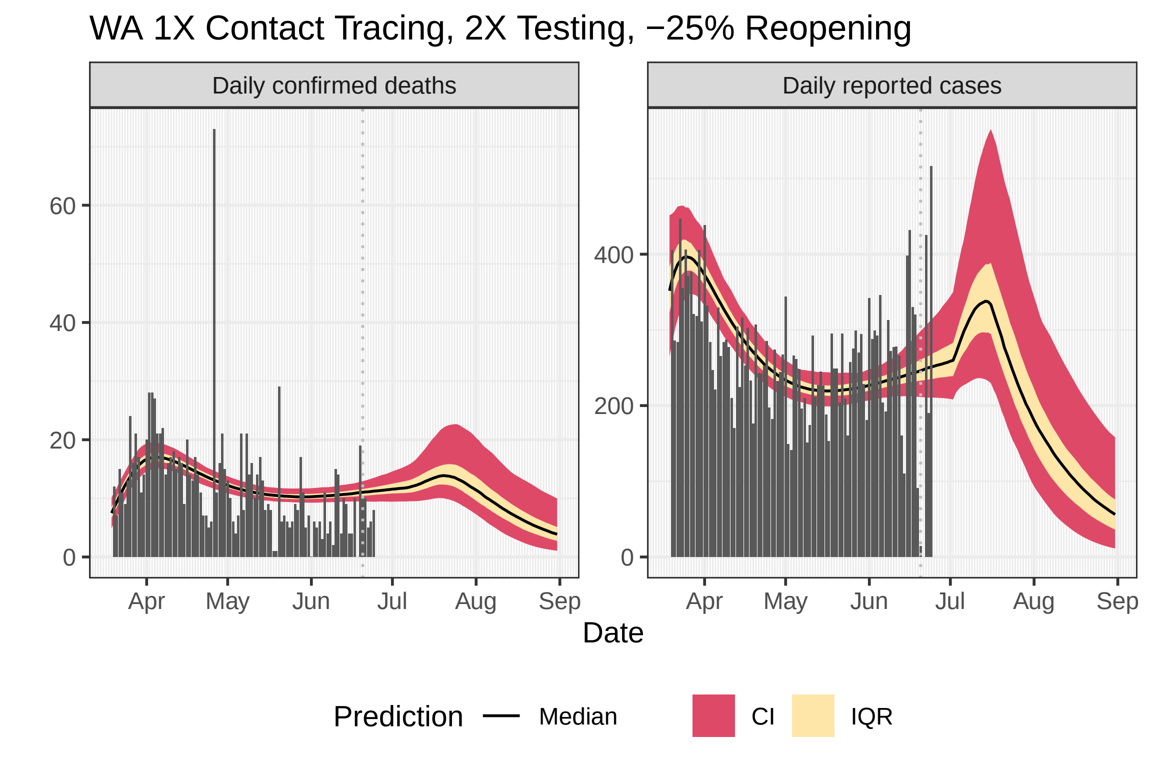

WA 1X Contact Tracing, 2X Testing, −25% Reopening
Daily confirmed deaths
Daily reported cases
0
20
40
60
0
200
400
Apr
May
Jun
Jul
Aug
Sep
Date
Prediction
Median
CI
IQR

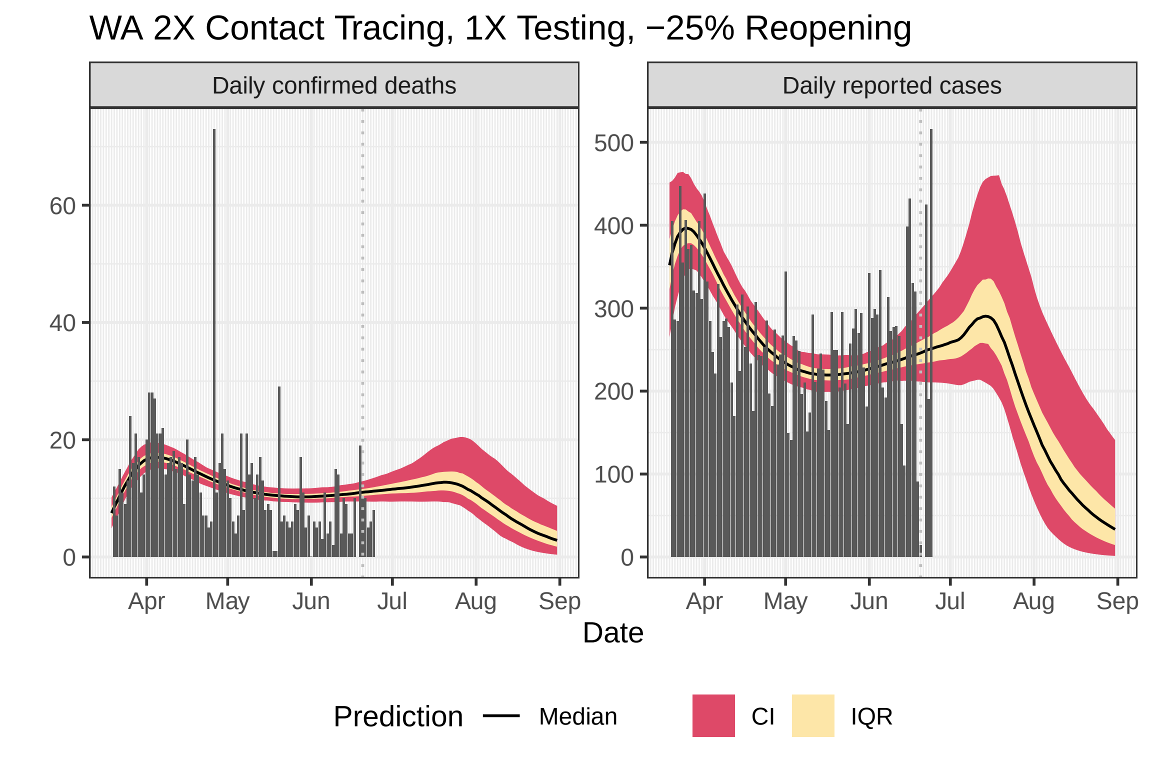
WA 2X Contact Tracing, 1X Testing, −25% Reopening
Daily confirmed deaths
Daily reported cases
0
20
40
60
100
200
300
400
500
Apr
May
Jun
Jul
Aug
Sep
Date
Prediction
Median
CI
IQR

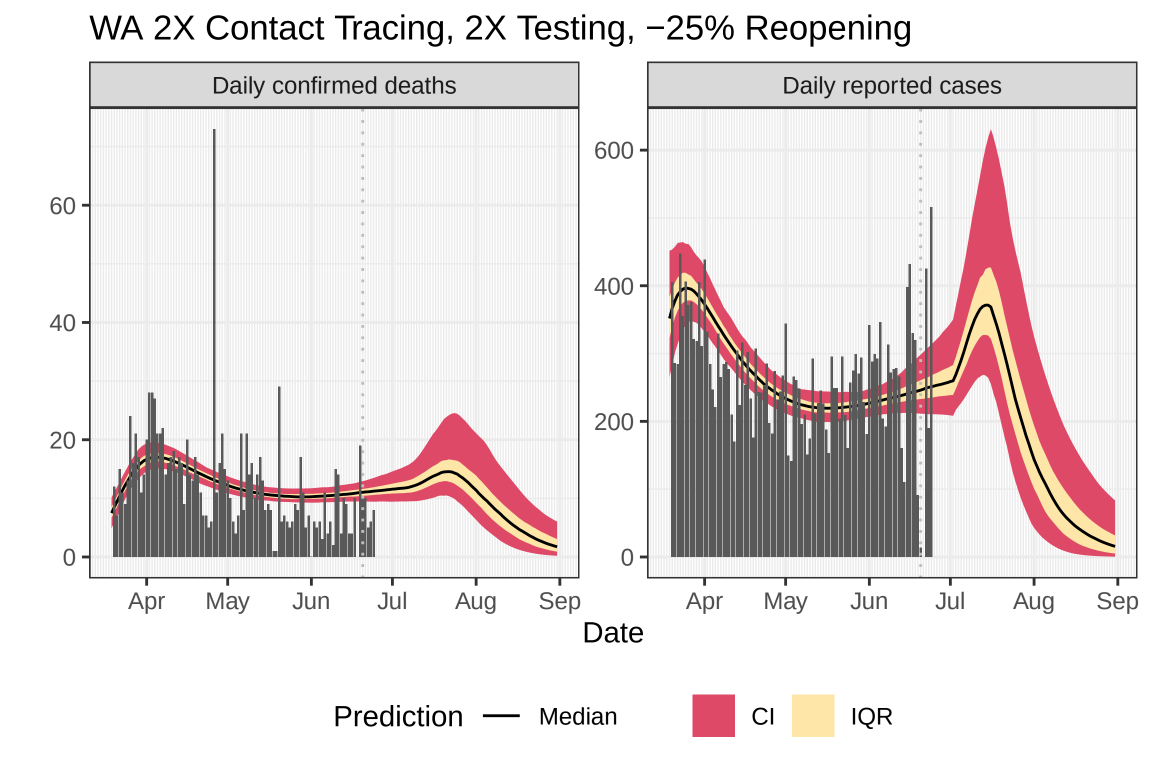

WA 2X Contact Tracing, 2X Testing, −25% Reopening
Daily confirmed deaths
Daily reported cases
0
20
40
60
0
200
400
600
Apr
May
Jun
Jul
Aug
Sep
Date
Prediction
Median
CI
IQR

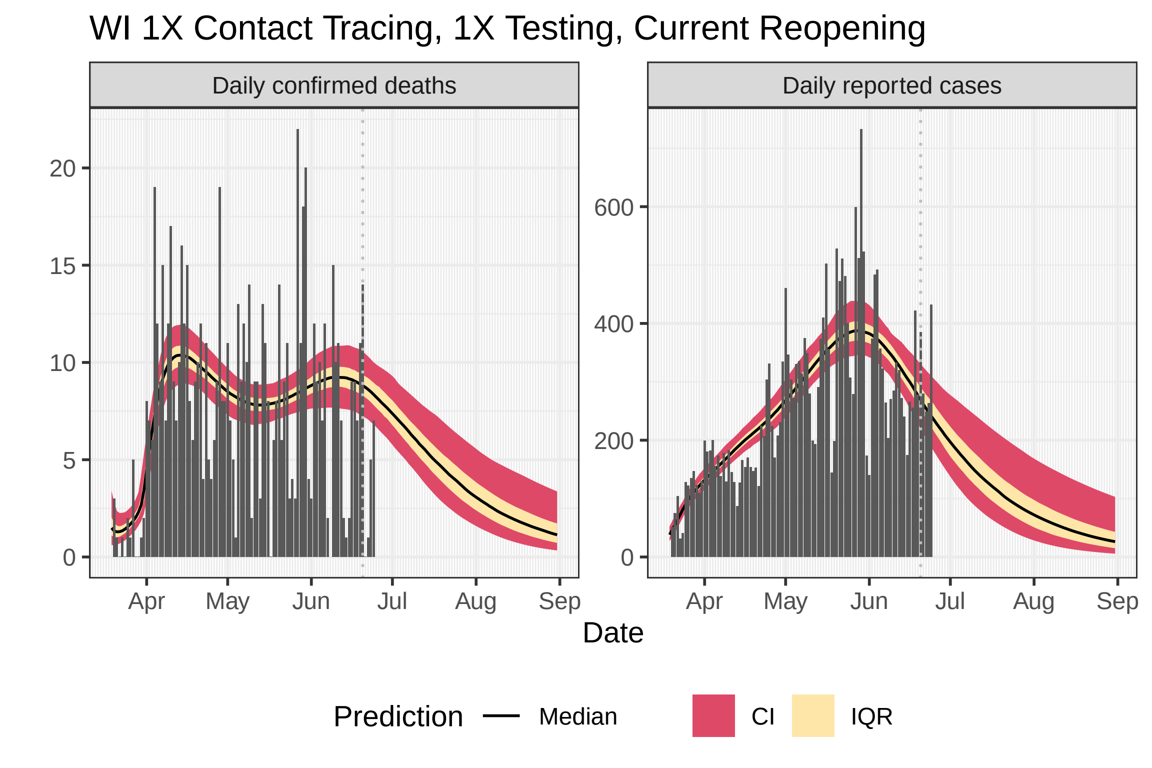

WI 1X Contact Tracing, 1X Testing, Current Reopening
Daily confirmed deaths
Daily reported cases
0
5
10
15
20
0
200
400
600
Apr
May
Jun
Jul
Aug
Sep
Date
Prediction
Median
CI
IQR

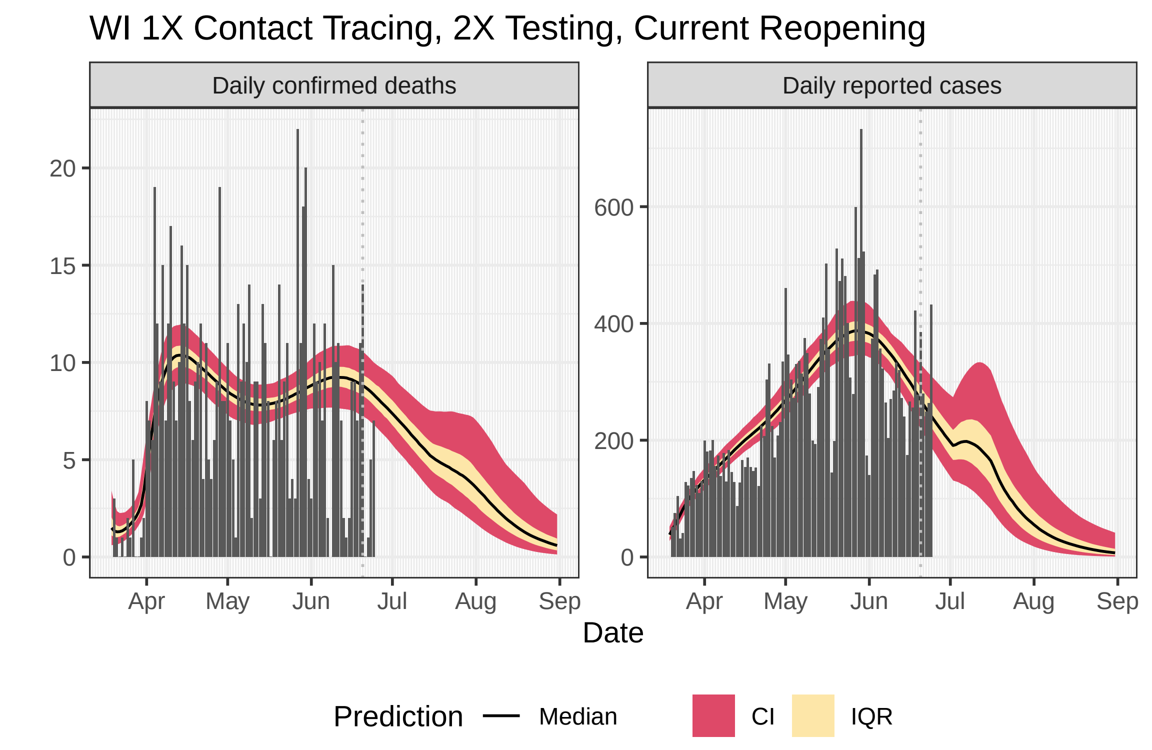

WI 1X Contact Tracing, 2X Testing, Current Reopening
Daily confirmed deaths
Daily reported cases
0
5
10
15
20
0
200
400
600
Apr
May
Jun
Jul
Aug
Sep
Date
Prediction
Median
CI
IQR

WI 2X Contact Tracing, 1X Testing, Current Reopening

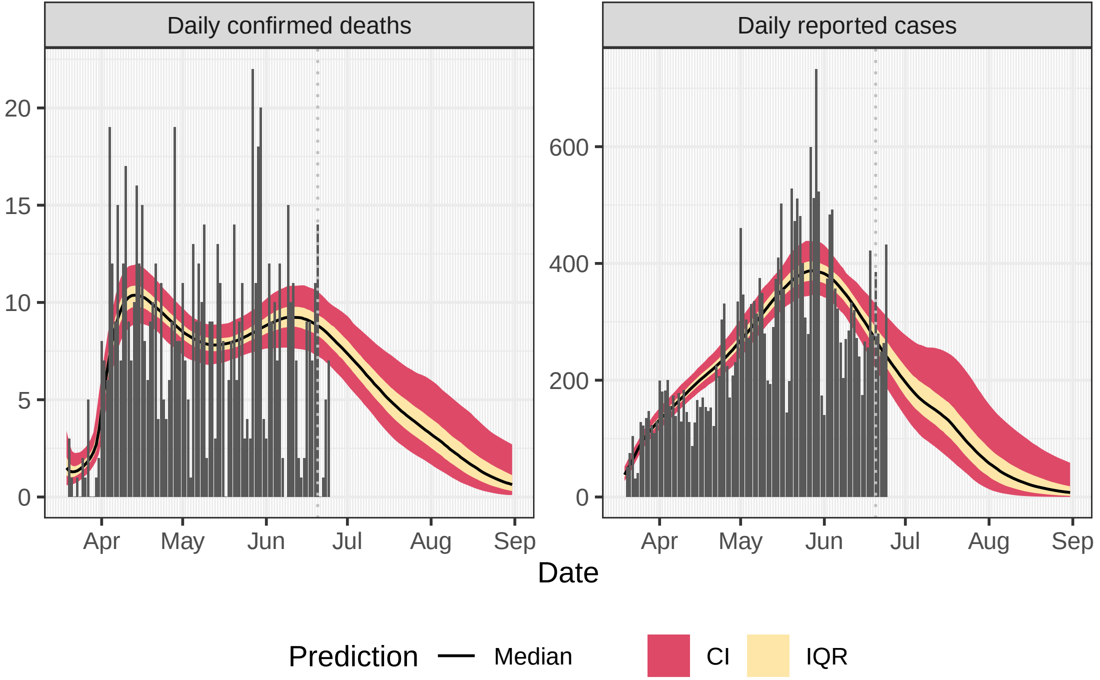

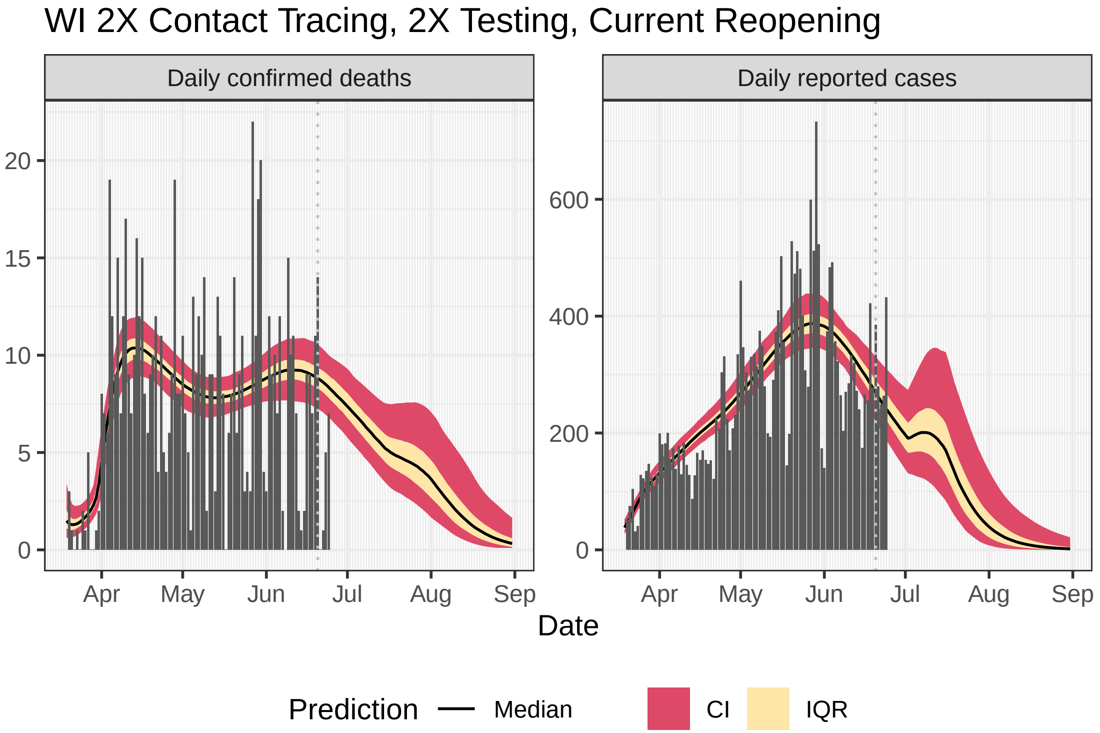
WI 2X Contact Tracing, 2X Testing, Current Reopening
Daily confirmed deaths
Daily reported cases
20
15
10
5
0
600
400
200
0
Apr
May
Jun
Jul
Aug
Sep
Date
Prediction
Median
CI
IQR

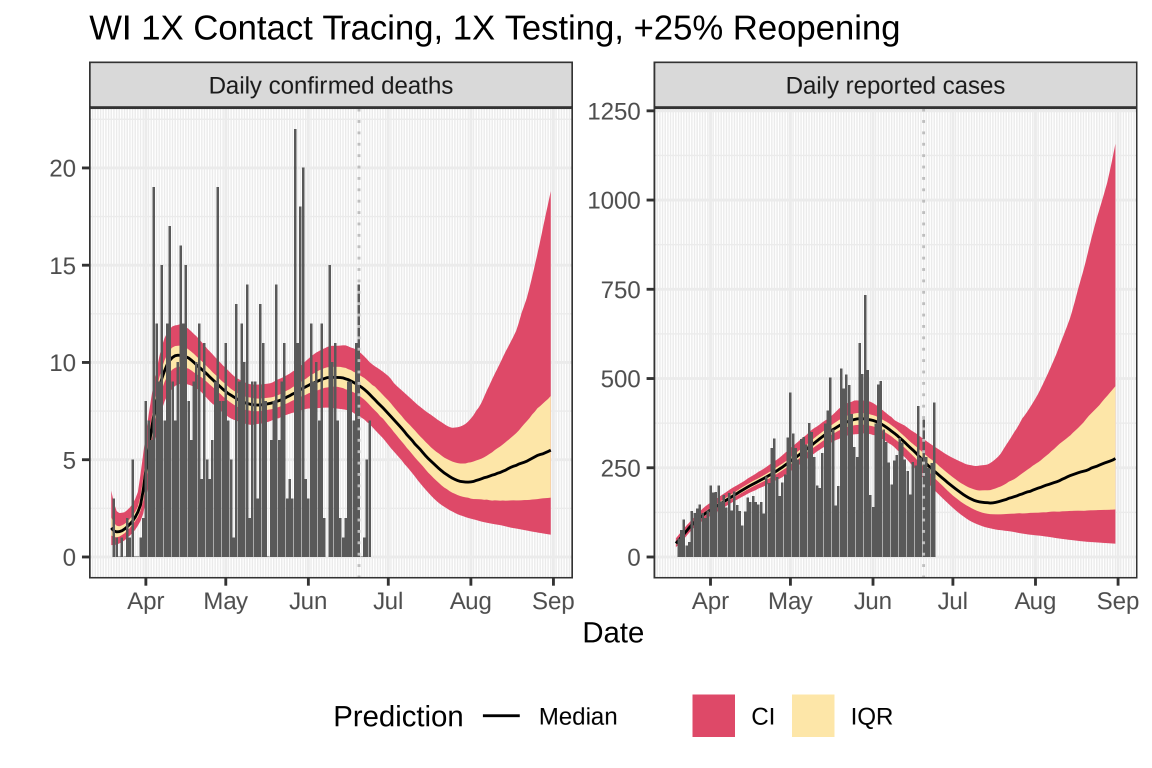

WI 1X Contact Tracing, 1X Testing, +25% Reopening
Daily confirmed deaths
Daily reported cases
0
5
10
15
20
0
250
500
750
1000
1250
Apr
May
Jun
Jul
Aug
Sep
Date
Prediction
Median
CI
IQR

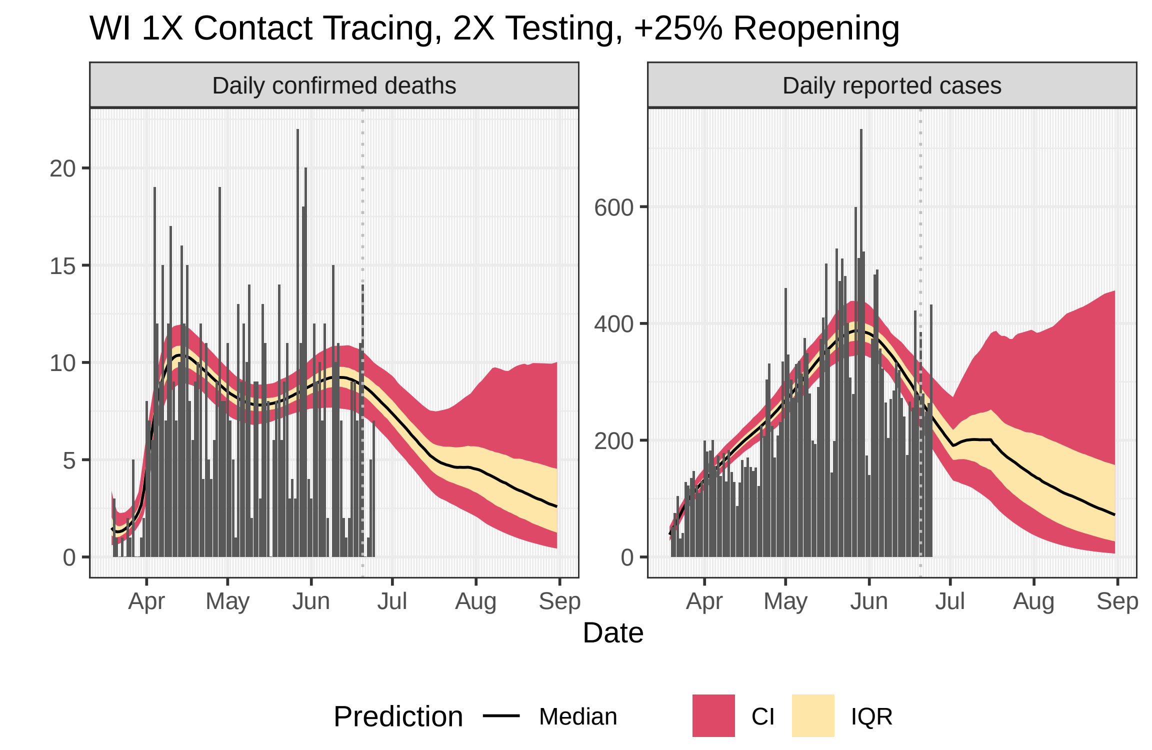
WI 1X Contact Tracing, 2X Testing, +25% Reopening
Daily confirmed deaths
Daily reported cases
0
5
10
15
20
0
200
400
600
Apr
May
Jun
Jul
Aug
Sep
Date
Prediction
Median
CI
IQR

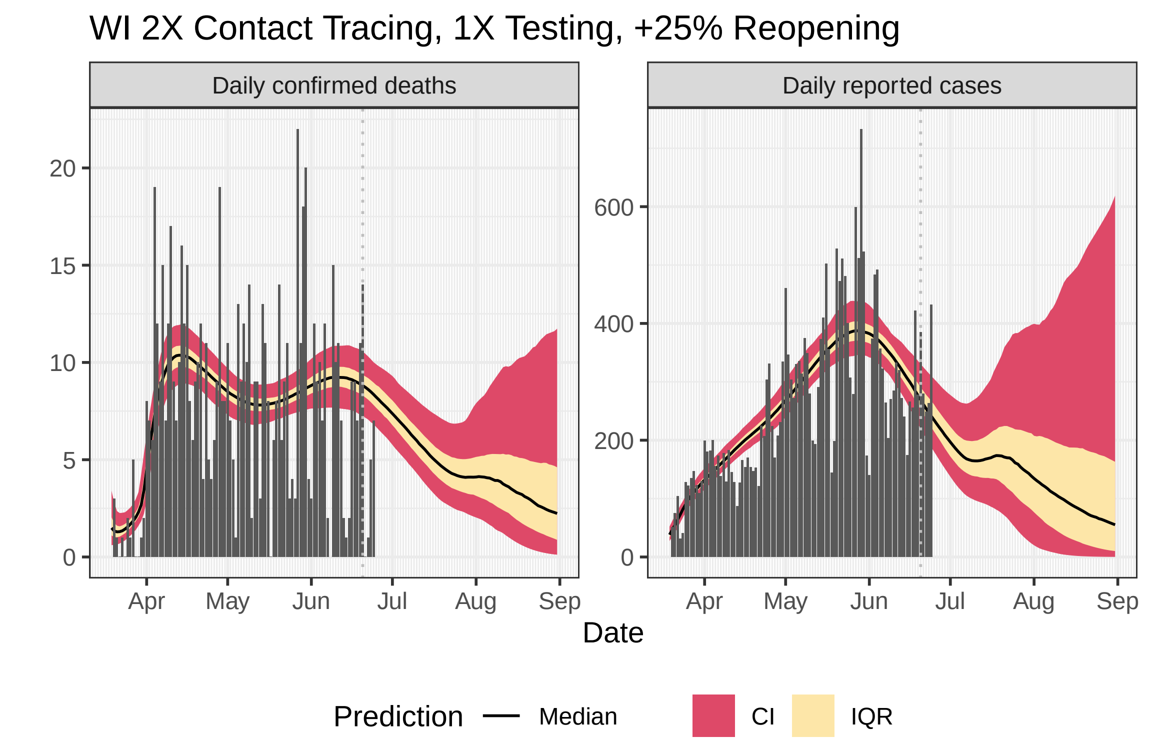

WI 2X Contact Tracing, 1X Testing, +25% Reopening
Daily confirmed deaths
Daily reported cases
0
5
10
15
20
0
200
400
600
Apr
May
Jun
Jul
Aug
Sep
Date
Prediction
Median
CI
IQR

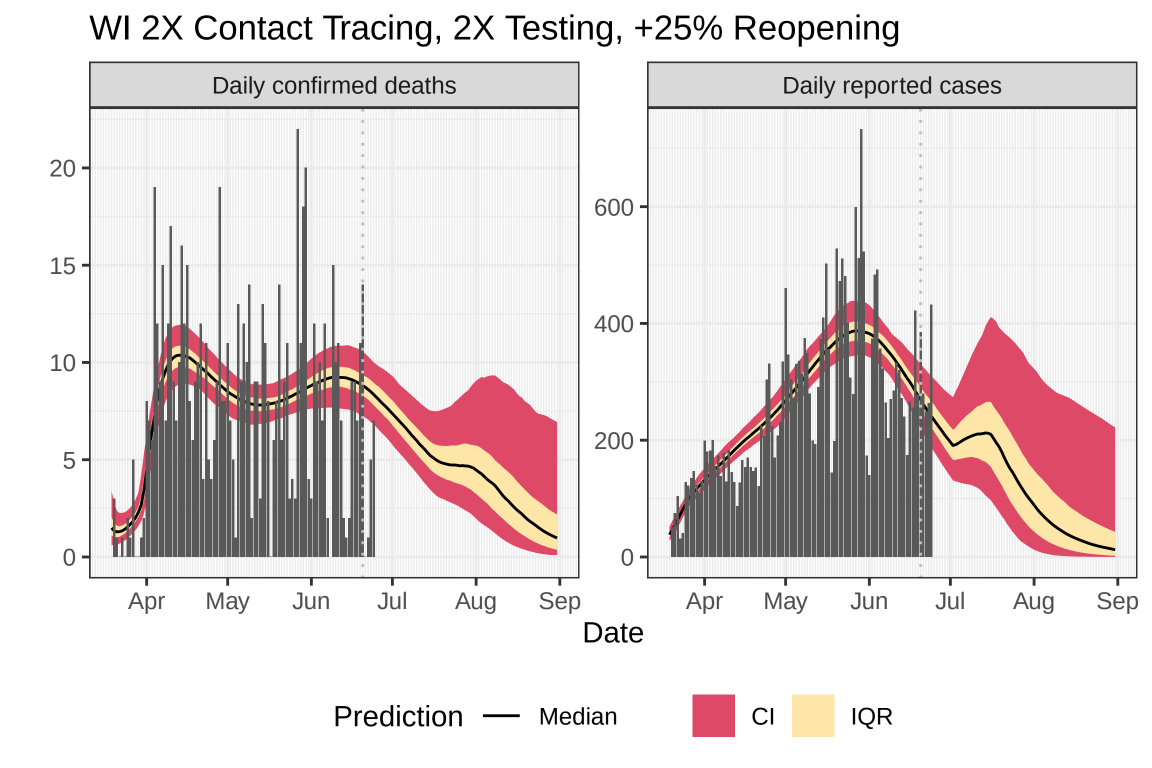
WI 2X Contact Tracing, 2X Testing, +25% Reopening
Daily confirmed deaths
Daily reported cases
0
5
10
15
20
0
200
400
600
Apr
May
Jun
Jul
Aug
Sep
Date
Prediction
Median
CI
IQR

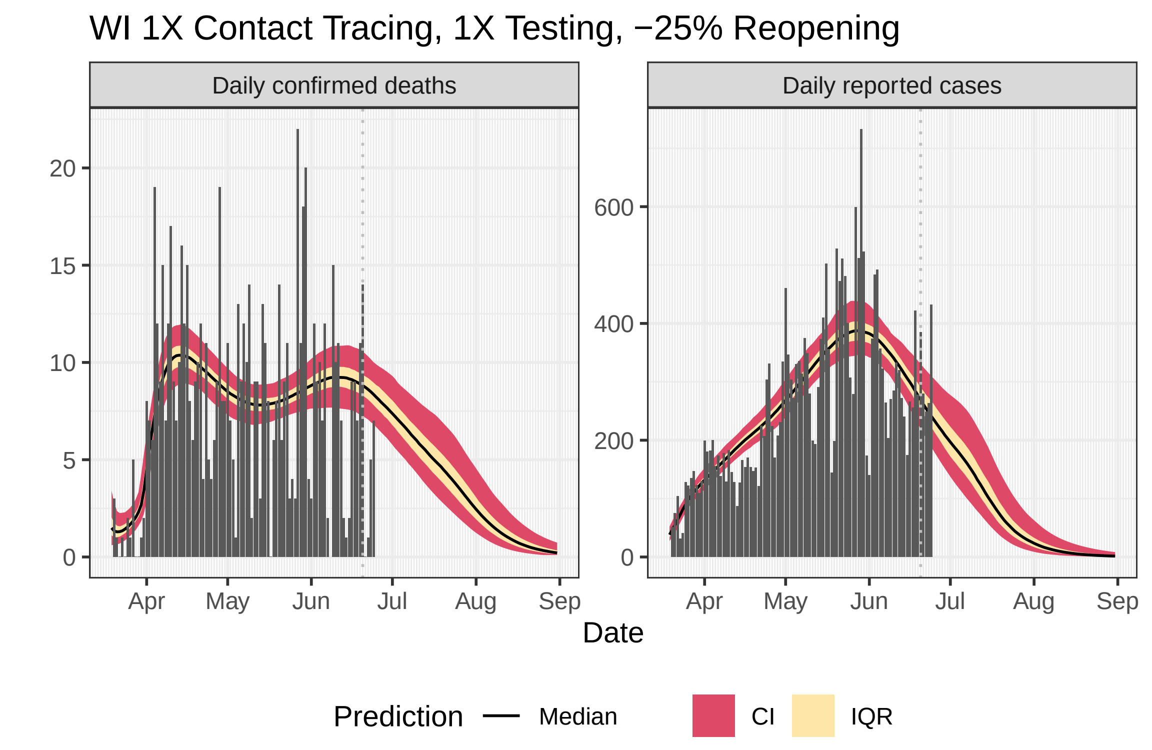
WI 1X Contact Tracing, 1X Testing, −25% Reopening
Daily confirmed deaths
Daily reported cases
0
5
10
15
20
0
200
400
600
Apr
May
Jun
Jul
Aug
Sep
Date
Prediction
Median
CI
IQR

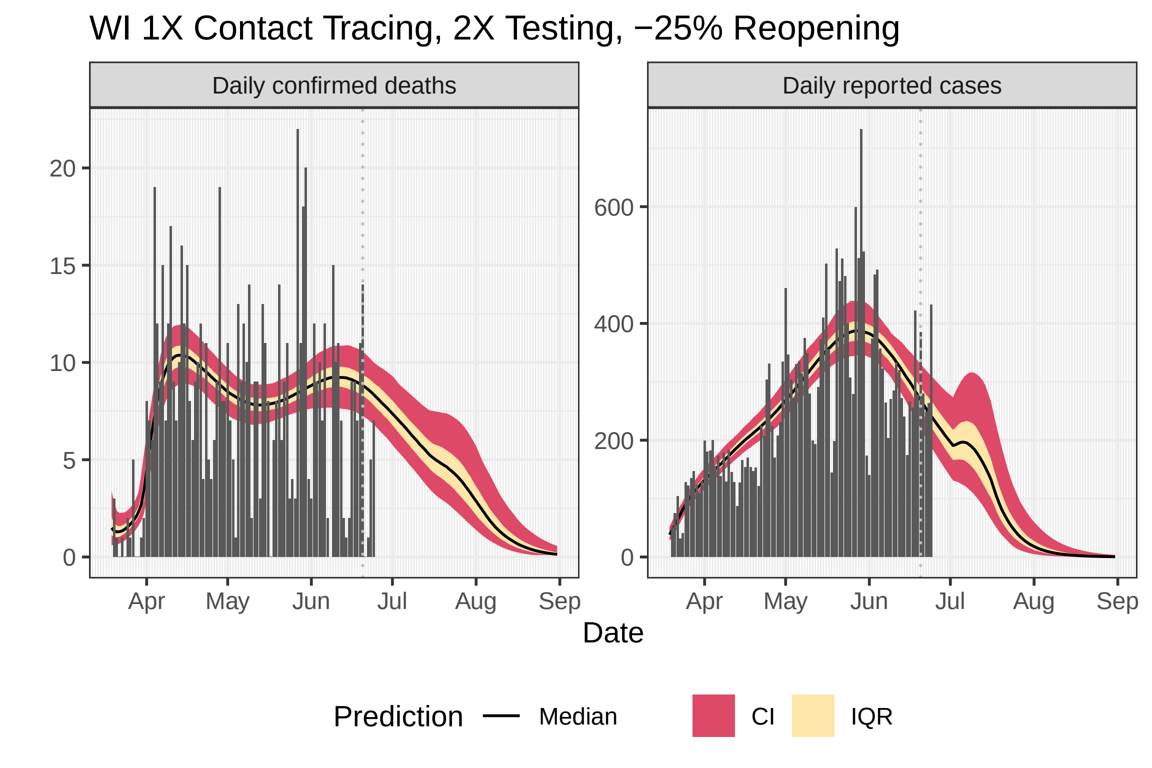

WI 1X Contact Tracing, 2X Testing, −25% Reopening
Daily confirmed deaths
Daily reported cases
20
15
10
5
0
600
400
200
0
Apr
May
Jun
Jul
Aug
Sep
Date
Prediction
Median
CI
IQR

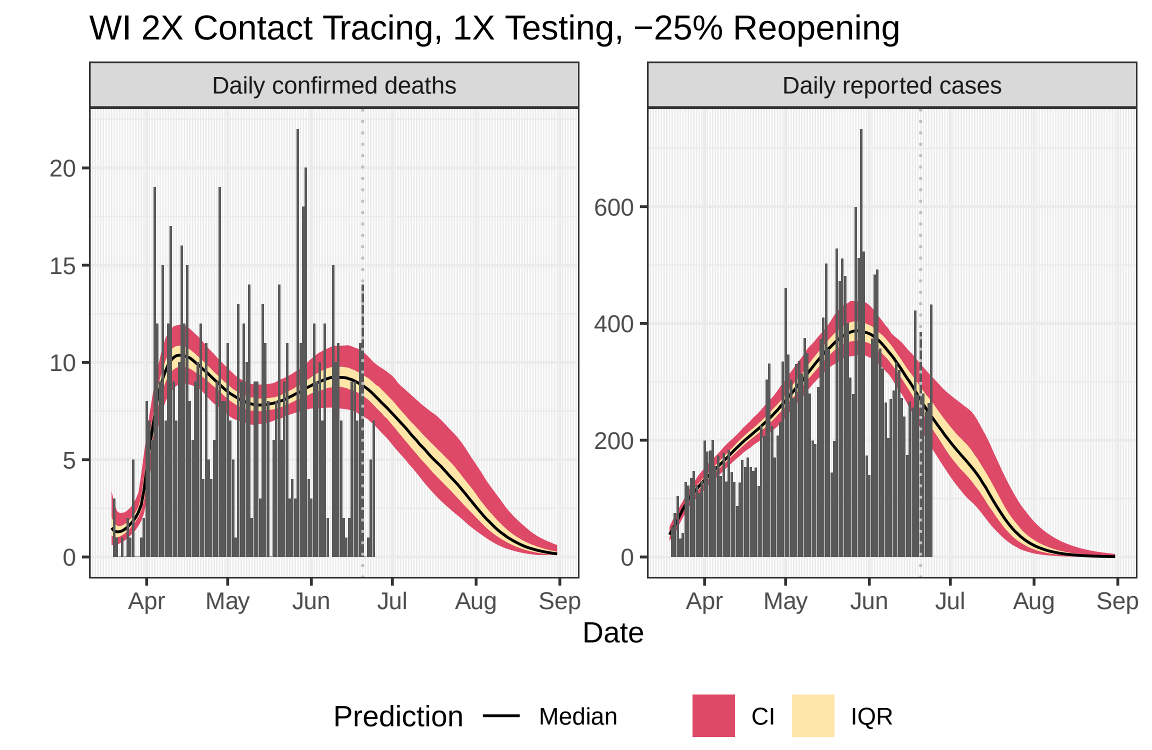
WI 2X Contact Tracing, 1X Testing, −25% Reopening
Daily confirmed deaths
Daily reported cases
0
5
10
15
20
0
200
400
600
Apr
May
Jun
Jul
Aug
Sep
Date
Prediction
Median
CI
IQR

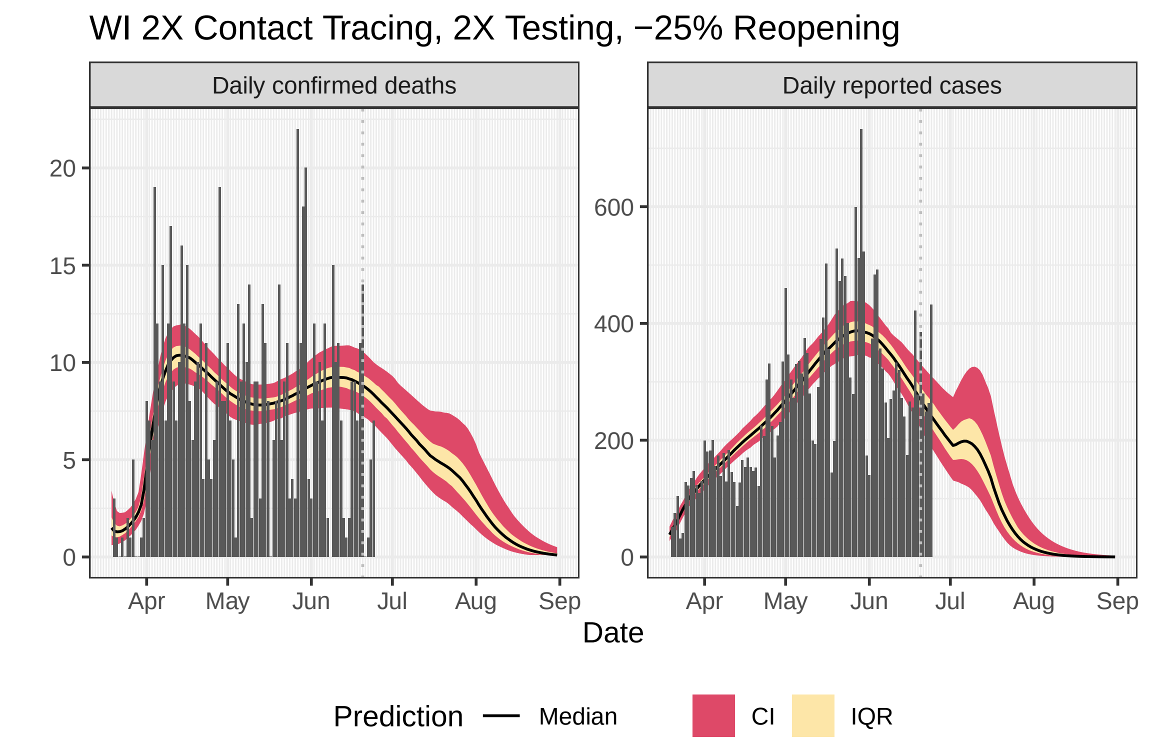
WI 2X Contact Tracing, 2X Testing, −25% Reopening
Daily confirmed deaths
Daily reported cases
0
5
10
15
20
0
200
400
600
Apr
May
Jun
Jul
Aug
Sep
Date
Prediction
Median
CI
IQR

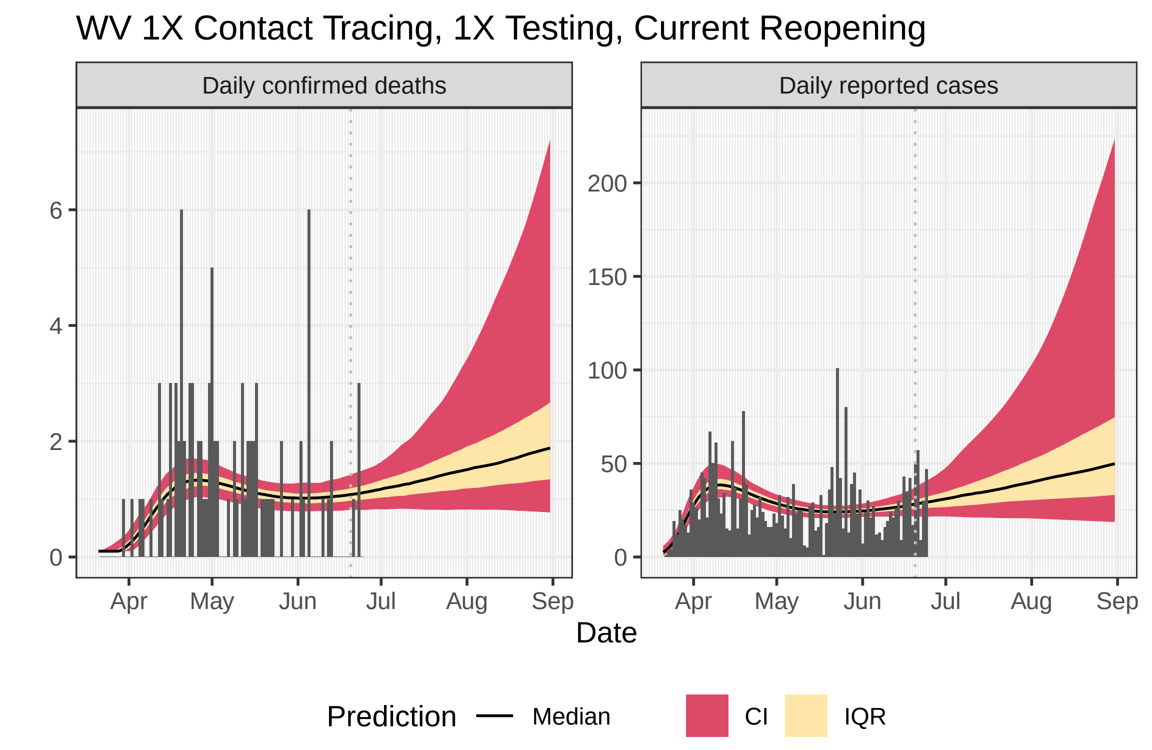

WV 1X Contact Tracing, 1X Testing, Current Reopening
Daily confirmed deaths
Daily reported cases
0
2
4
6
0
50
100
150
200
Apr
May
Jun
Jul
Aug
Sep
Date
Prediction
Median
CI
IQR

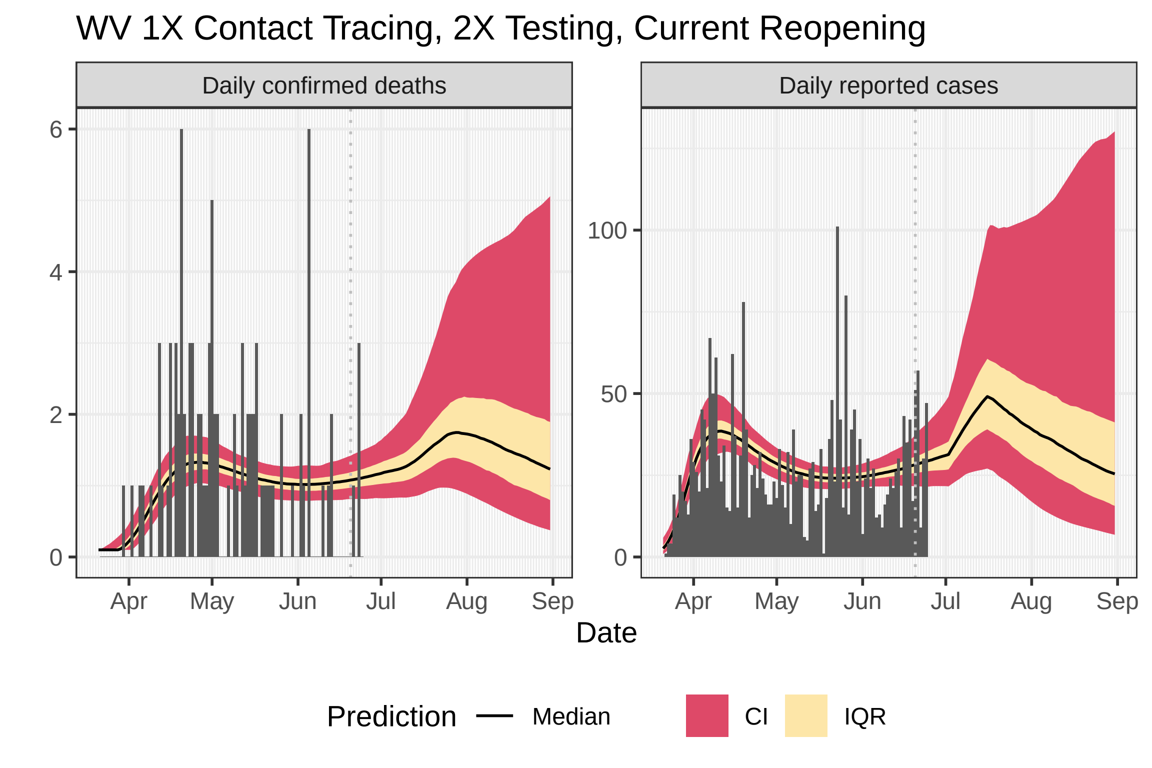

WV 1X Contact Tracing, 2X Testing, Current Reopening
Daily confirmed deaths
Daily reported cases
6
4
2
0
100
50
0
Apr
May
Jun
Jul
Aug
Sep
Date
Prediction
Median
CI
IQR

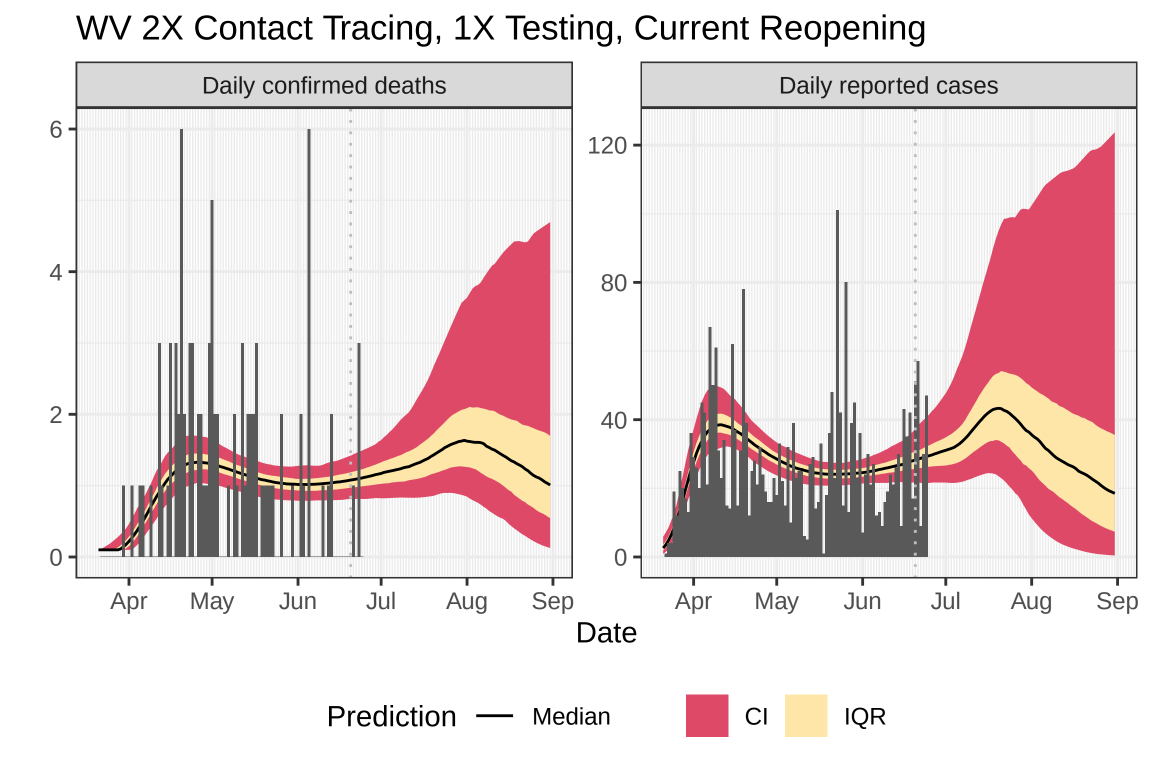

WV 2X Contact Tracing, 1X Testing, Current Reopening
Daily confirmed deaths
Daily reported cases
0
2
4
6
0
40
80
120
Apr
May
Jun
Jul
Aug
Sep
Date
Prediction
Median
CI
IQR

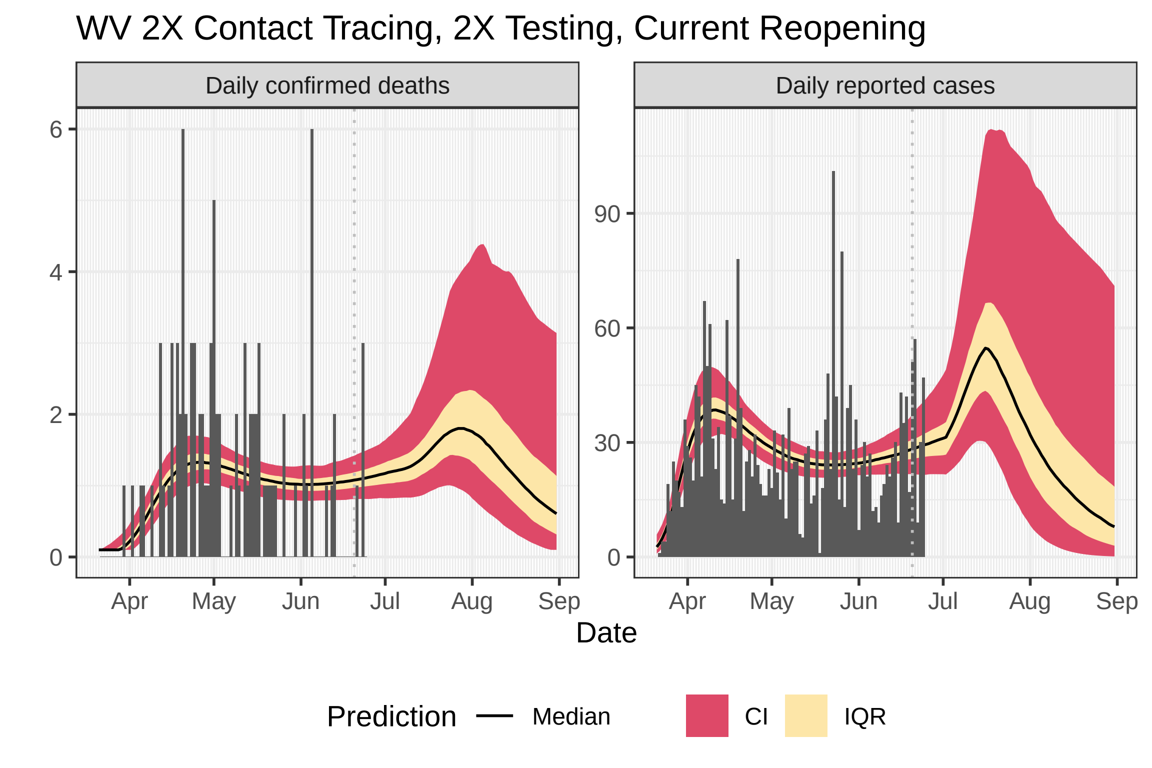
WV 2X Contact Tracing, 2X Testing, Current Reopening
Daily confirmed deaths
Daily reported cases
6
4
2
0
90
60
30
0
Apr
May
Jun
Jul
Aug
Sep
Date
Prediction
Median
CI
IQR

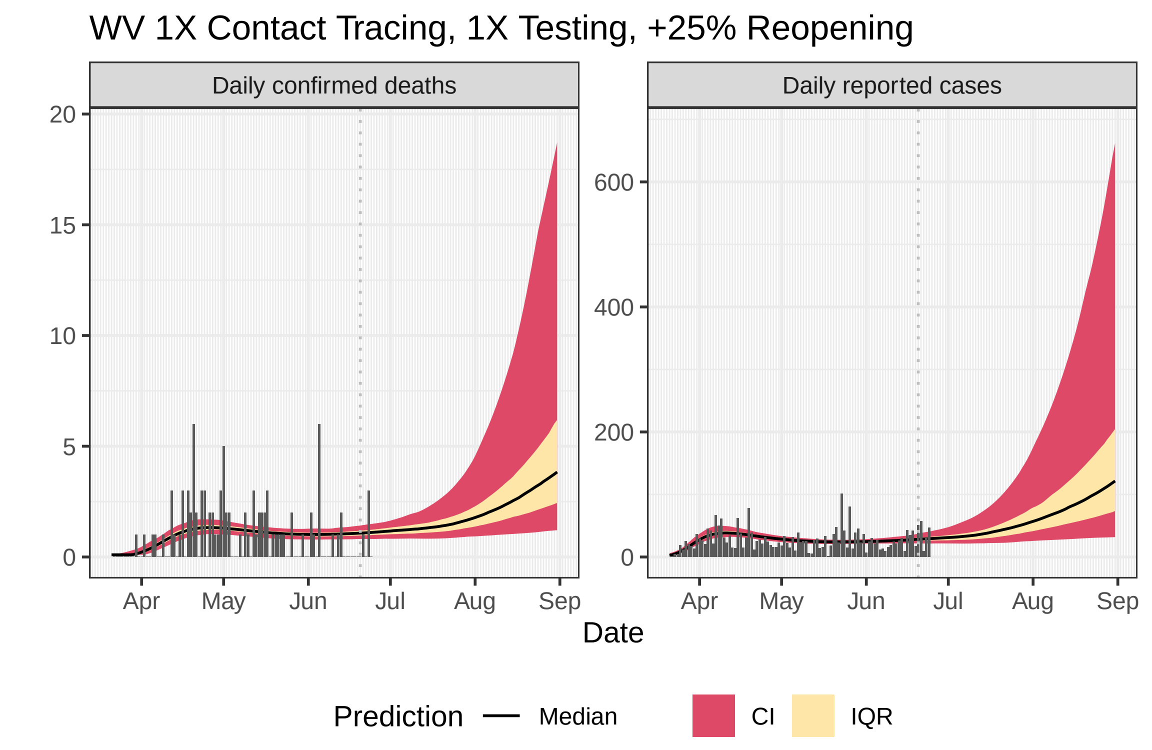
WV 1X Contact Tracing, 1X Testing, +25% Reopening
Daily confirmed deaths
Daily reported cases
0
5
10
15
20
0
200
400
600
Apr
May
Jun
Jul
Aug
Sep
Date
Prediction
Median
CI
IQR

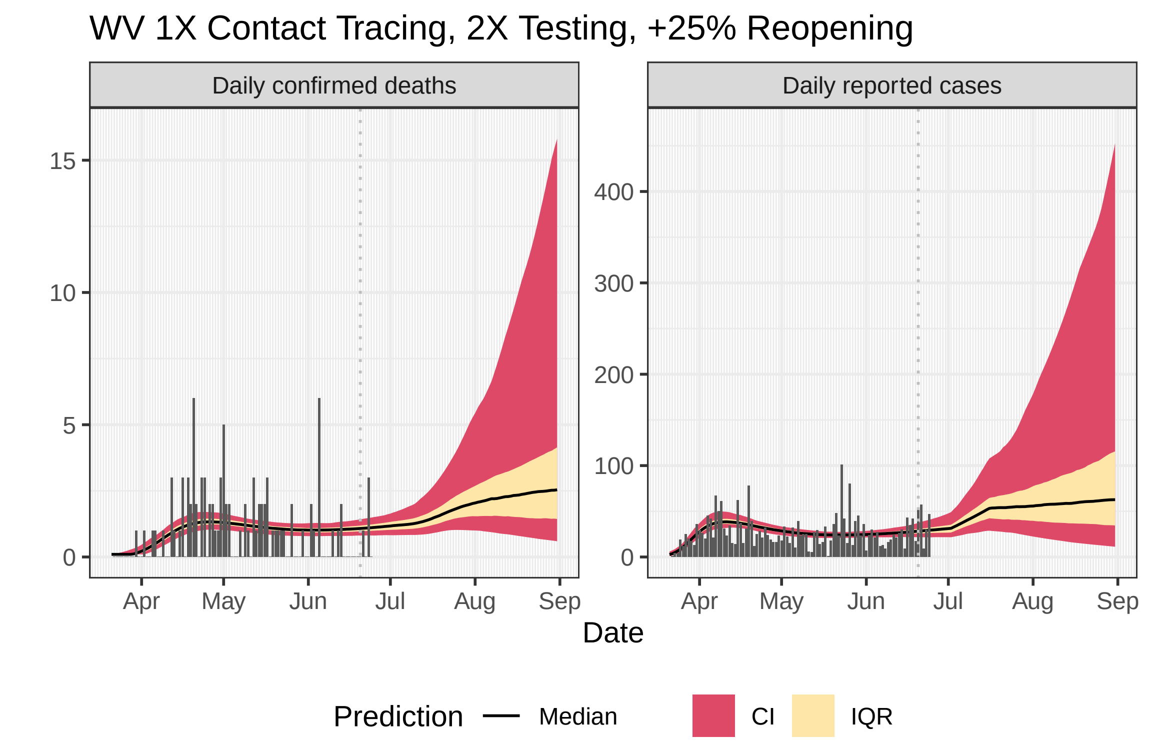

WV 1X Contact Tracing, 2X Testing, +25% Reopening
Daily confirmed deaths
Daily reported cases
0
5
10
15
0
100
200
300
400
Apr
May
Jun
Jul
Aug
Sep
Date
Prediction
Median
CI
IQR

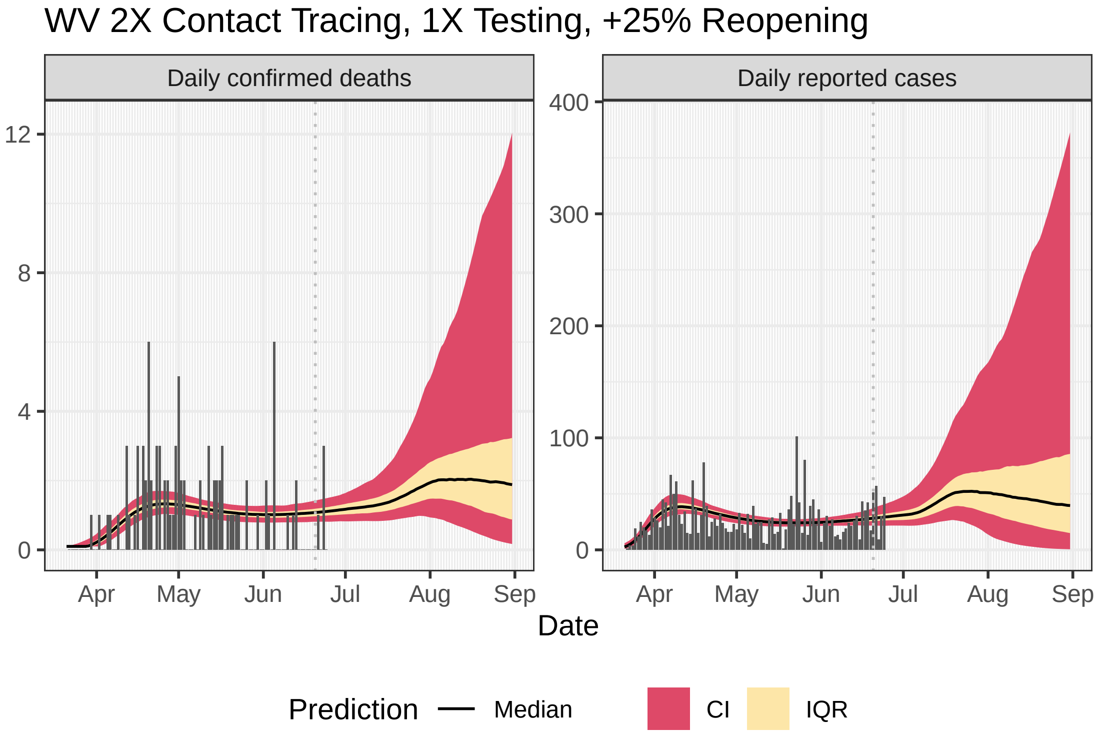
WV 2X Contact Tracing, 1X Testing, +25% Reopening
Daily confirmed deaths
Daily reported cases
0
4
8
12
0
100
200
300
400
Apr
May
Jun
Jul
Aug
Sep
Date
Prediction
Median
CI
IQR

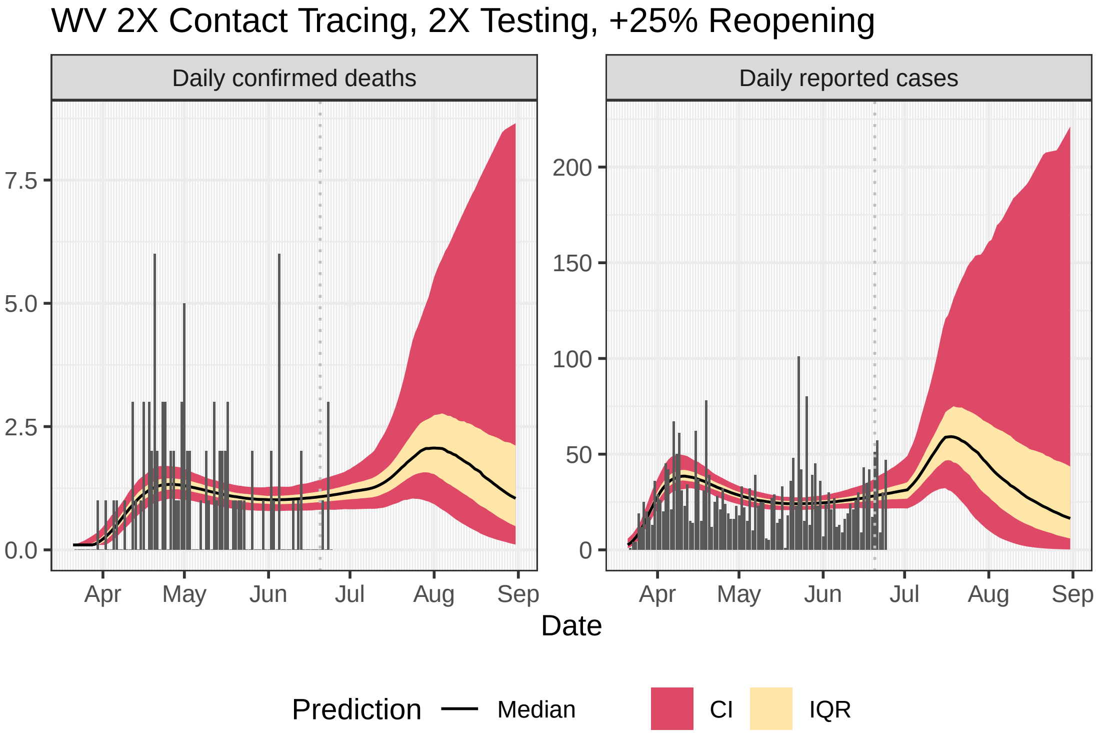

WV 2X Contact Tracing, 2X Testing, +25% Reopening
Daily confirmed deaths
Daily reported cases
0.0
2.5
5.0
7.5
0
50
100
150
200
Apr
May
Jun
Jul
Aug
Sep
Date
Prediction
Median
CI
IQR

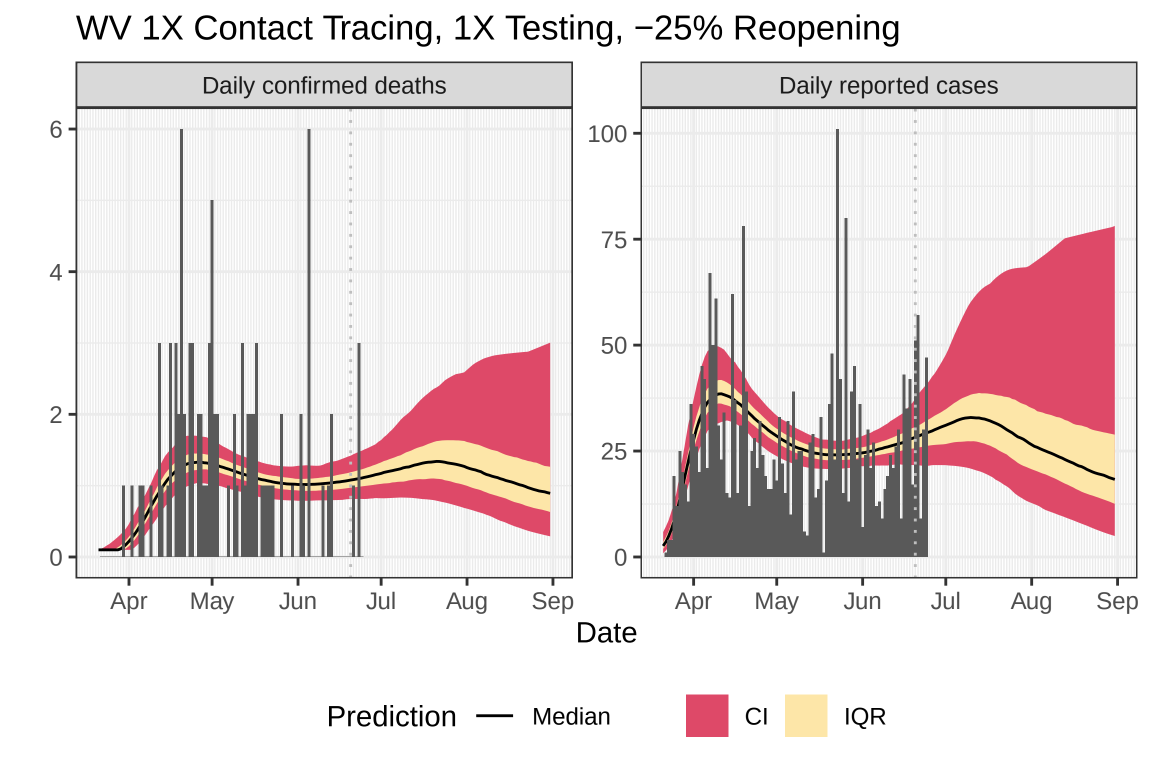

WV 1X Contact Tracing, 1X Testing, −25% Reopening
Daily confirmed deaths
Daily reported cases
0
2
4
6
0
25
50
75
100
Apr
May
Jun
Jul
Aug
Sep
Date
Prediction
Median
CI
IQR

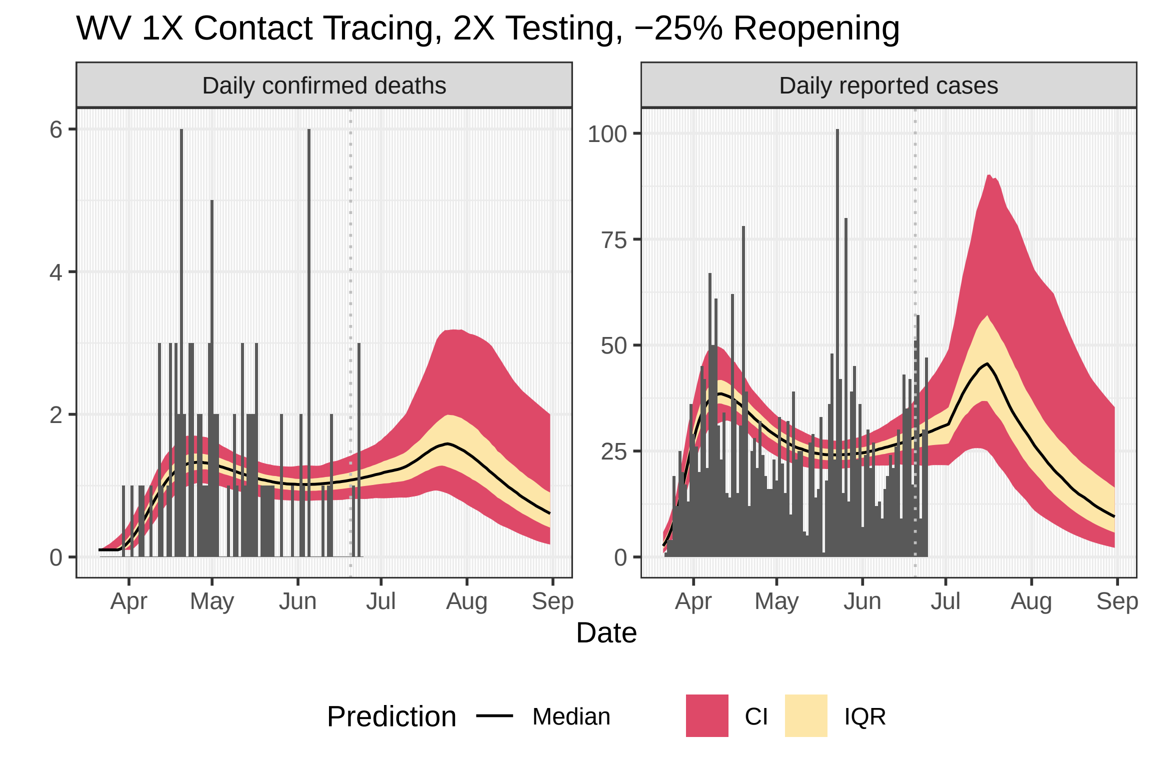
WV 1X Contact Tracing, 2X Testing, −25% Reopening
Daily confirmed deaths
Daily reported cases
0
2
4
6
0
25
50
75
100
Apr
May
Jun
Jul
Aug
Sep
Date
Prediction
Median
CI
IQR

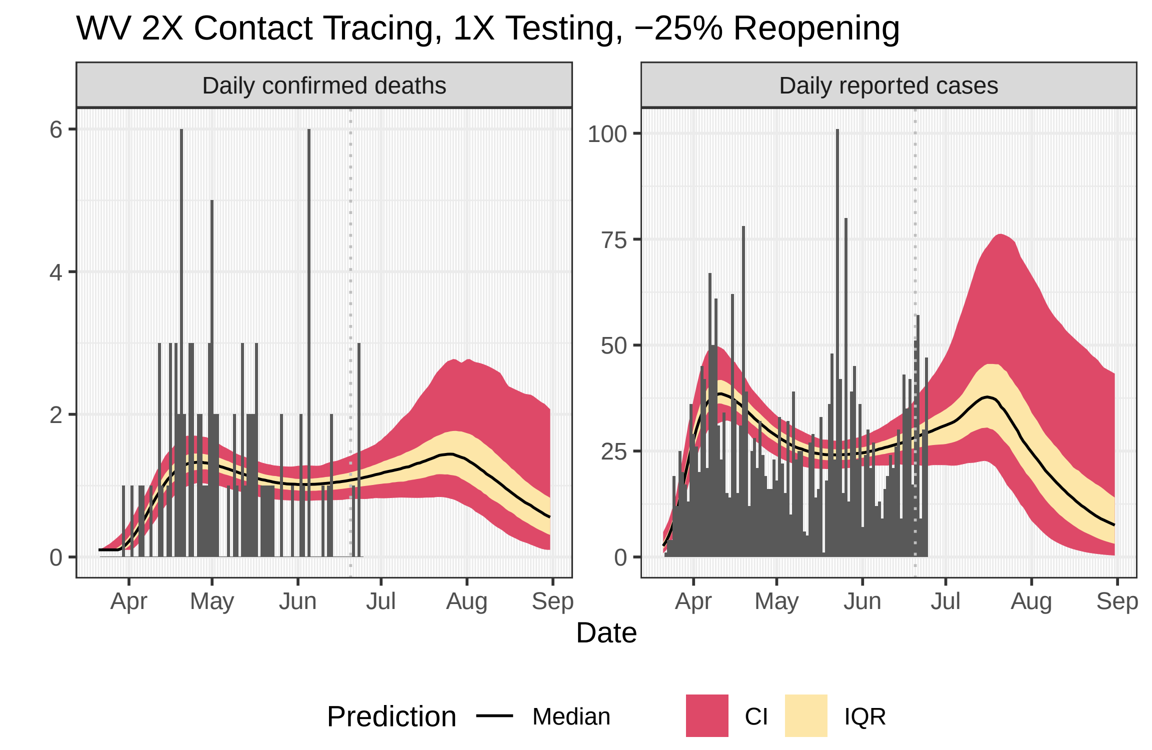

WV 2X Contact Tracing, 1X Testing, −25% Reopening
Daily confirmed deaths
Daily reported cases
6
4
2
0
100
75
50
25
0
Apr
May
Jun
Jul
Aug
Sep
Date
Prediction
Median
CI
IQR

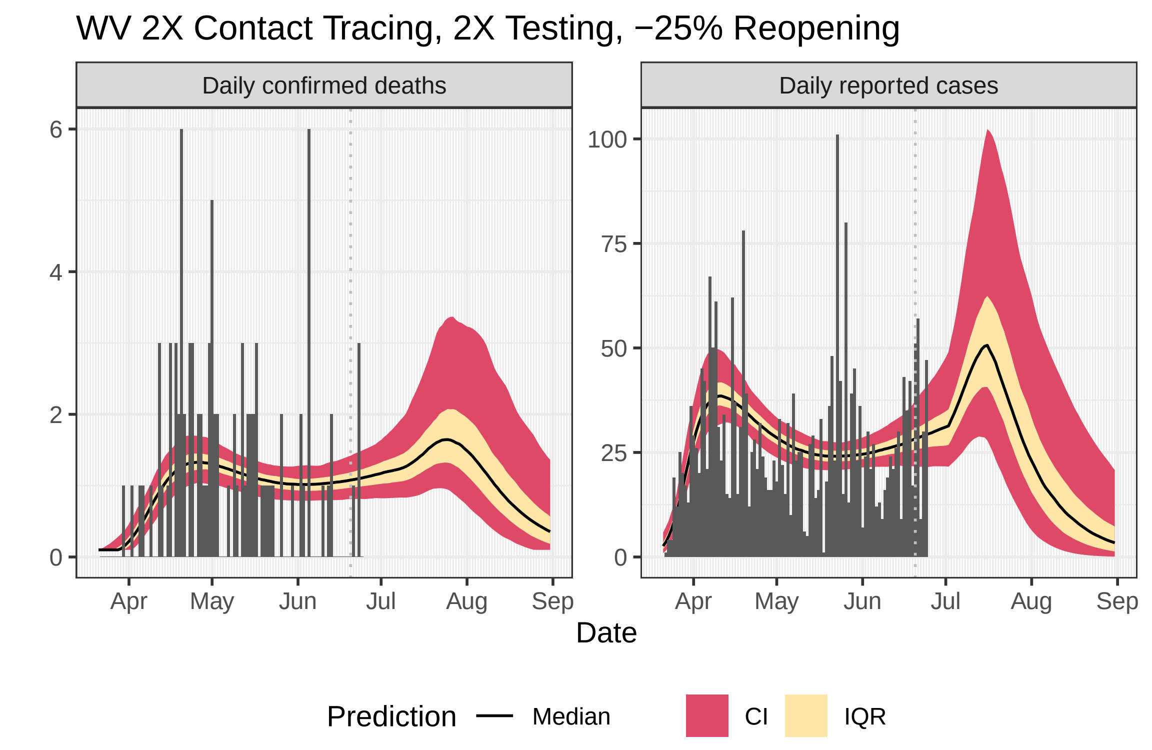
WV 2X Contact Tracing, 2X Testing, −25% Reopening
Daily confirmed deaths
Daily reported cases
0
2
4
6
0
25
50
75
100
Apr
May
Jun
Jul
Aug
Sep
Date
Prediction
Median
CI
IQR

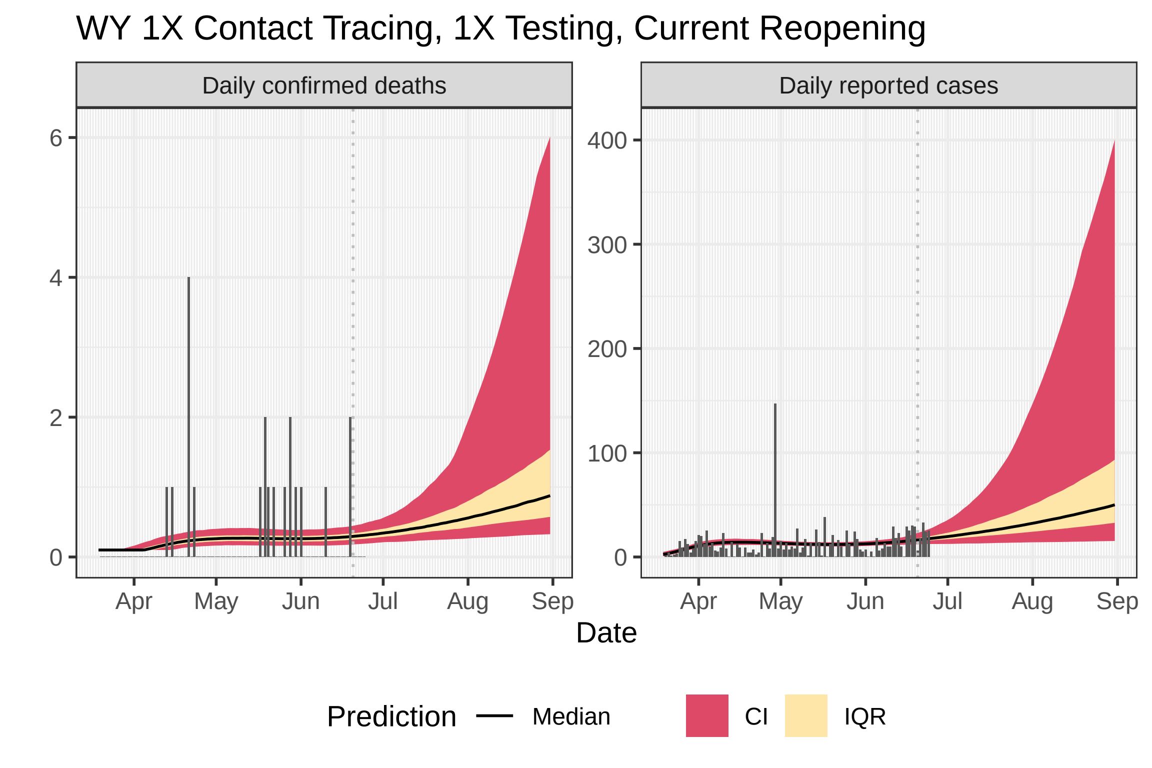
WY 1X Contact Tracing, 1X Testing, Current Reopening
Daily confirmed deaths
Daily reported cases
6
4
2
0
400
300
200
100
0
Apr
May
Jun
Jul
Aug
Sep
Date
Prediction
Median
CI
IQR

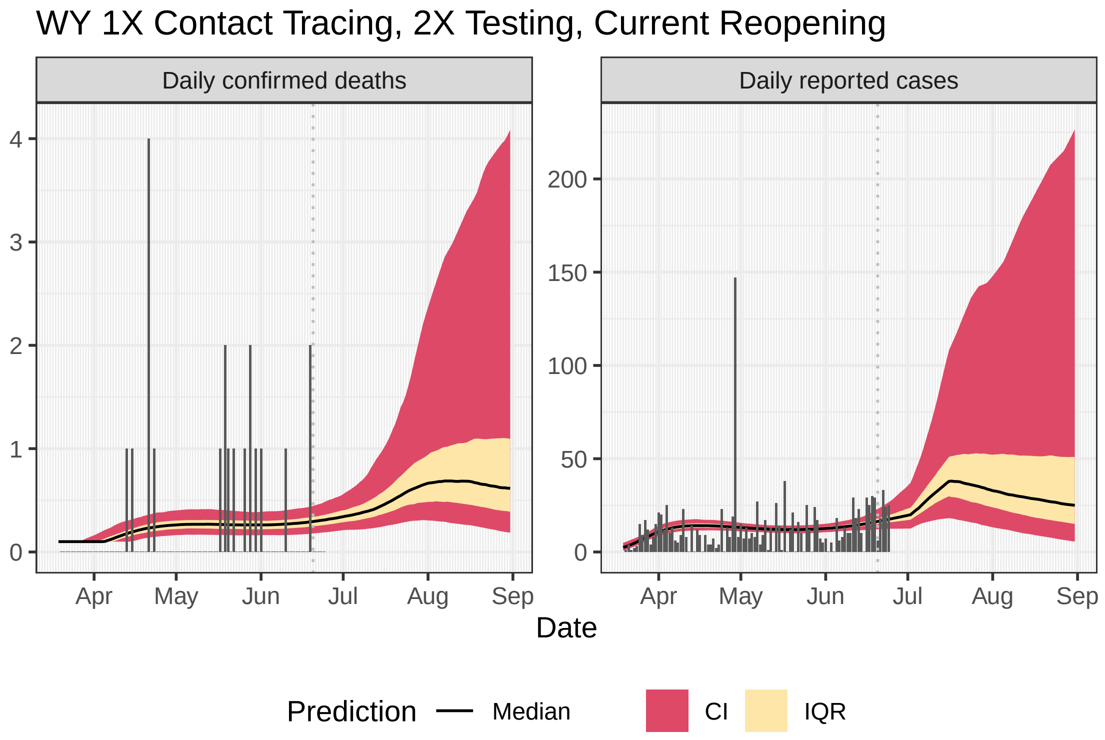

WY 1X Contact Tracing, 2X Testing, Current Reopening
Daily confirmed deaths
Daily reported cases
0
1
2
3
4
0
50
100
150
200
Apr
May
Jun
Jul
Aug
Sep
Date
Prediction
Median
CI
IQR

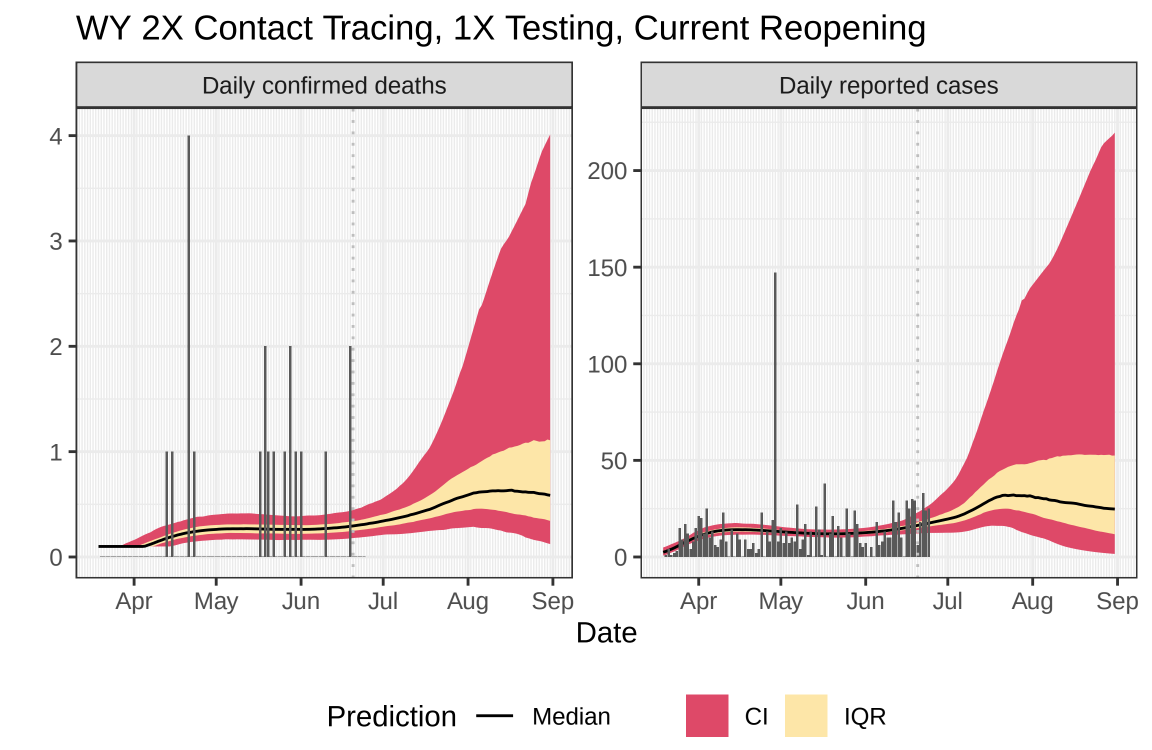

WY 2X Contact Tracing, 1X Testing, Current Reopening
Daily confirmed deaths
Daily reported cases
0
1
2
3
4
0
50
100
150
200
Apr
May
Jun
Jul
Aug
Sep
Date
Prediction
Median
CI
IQR

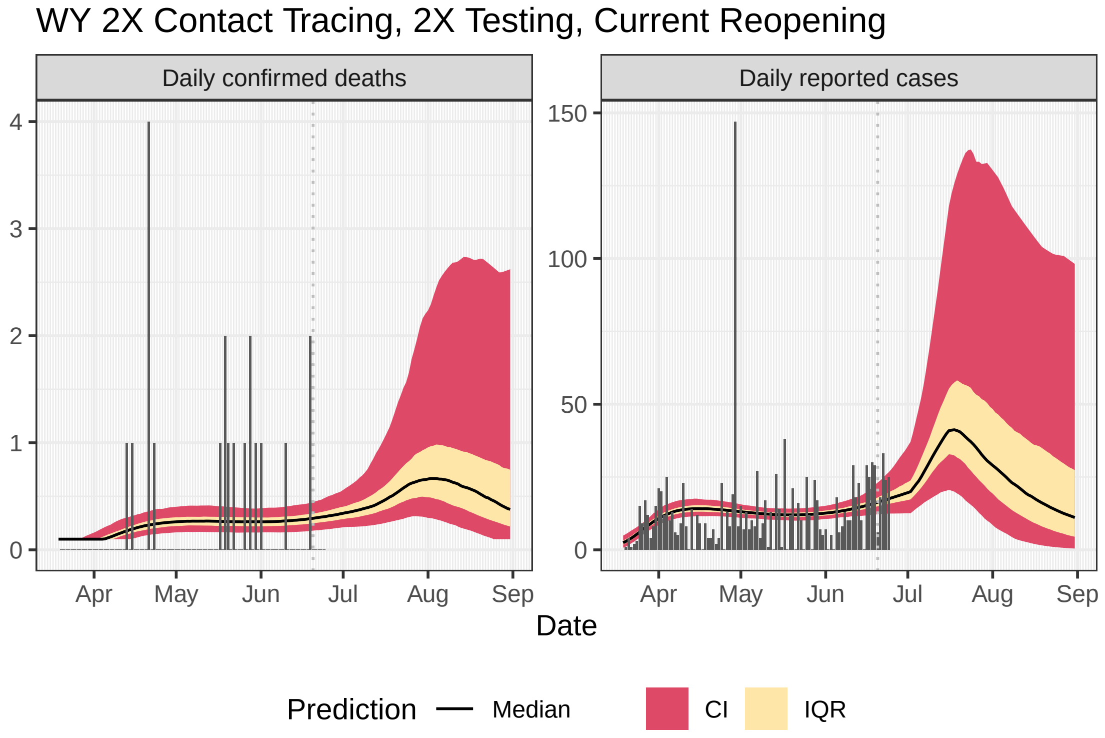
WY 2X Contact Tracing, 2X Testing, Current Reopening
Daily confirmed deaths
Daily reported cases
4
3
2
1
0
150
100
50
0
Apr
May
Jun
Jul
Aug
Sep
Date
Prediction
Median
CI
IQR

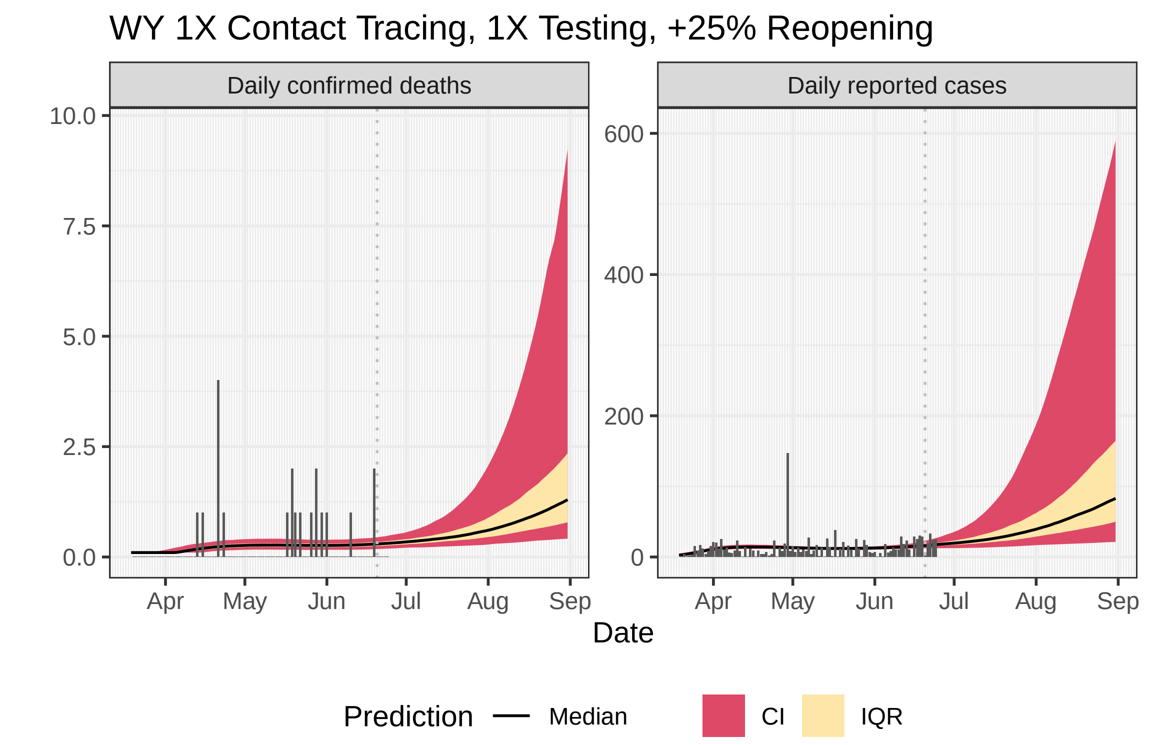

WY 1X Contact Tracing, 1X Testing, +25% Reopening
Daily confirmed deaths
Daily reported cases
10.0
7.5
5.0
2.5
0.0
600
400
200
0
Apr
May
Jun
Jul
Aug
Sep
Date
Prediction
Median
CI
IQR

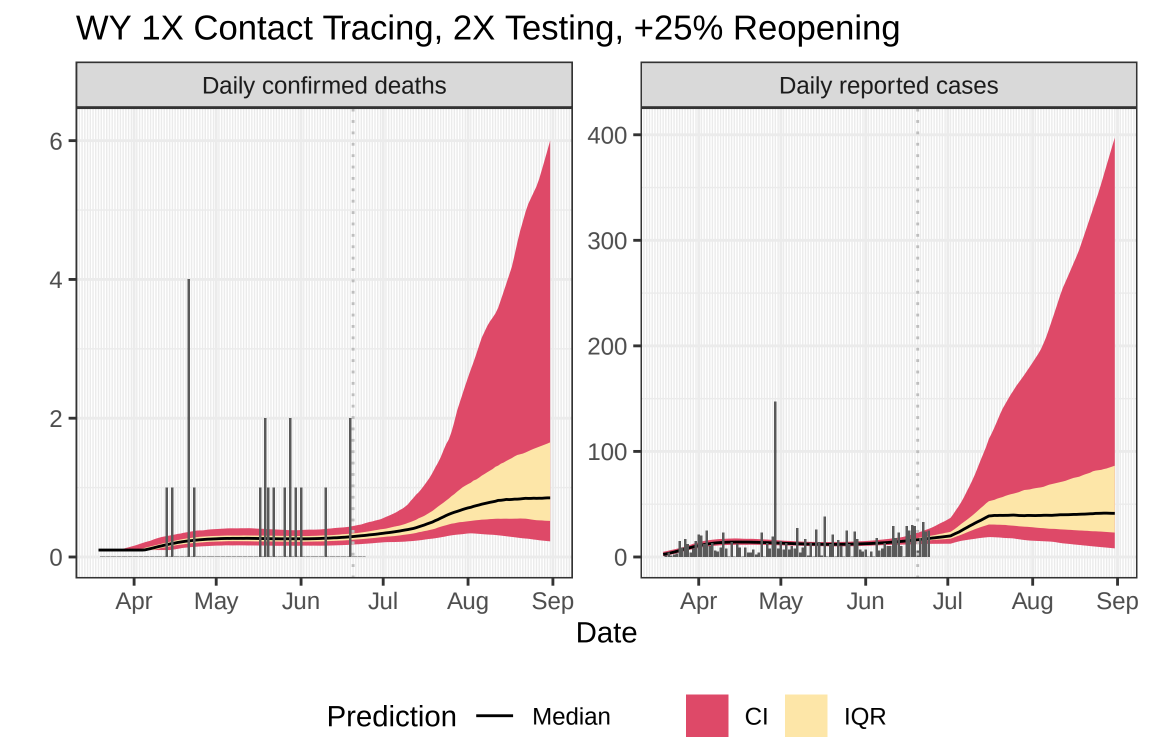

WY 1X Contact Tracing, 2X Testing, +25% Reopening
Daily confirmed deaths
Daily reported cases
0
2
4
6
0
100
200
300
400
Apr
May
Jun
Jul
Aug
Sep
Date
Prediction
Median
CI
IQR

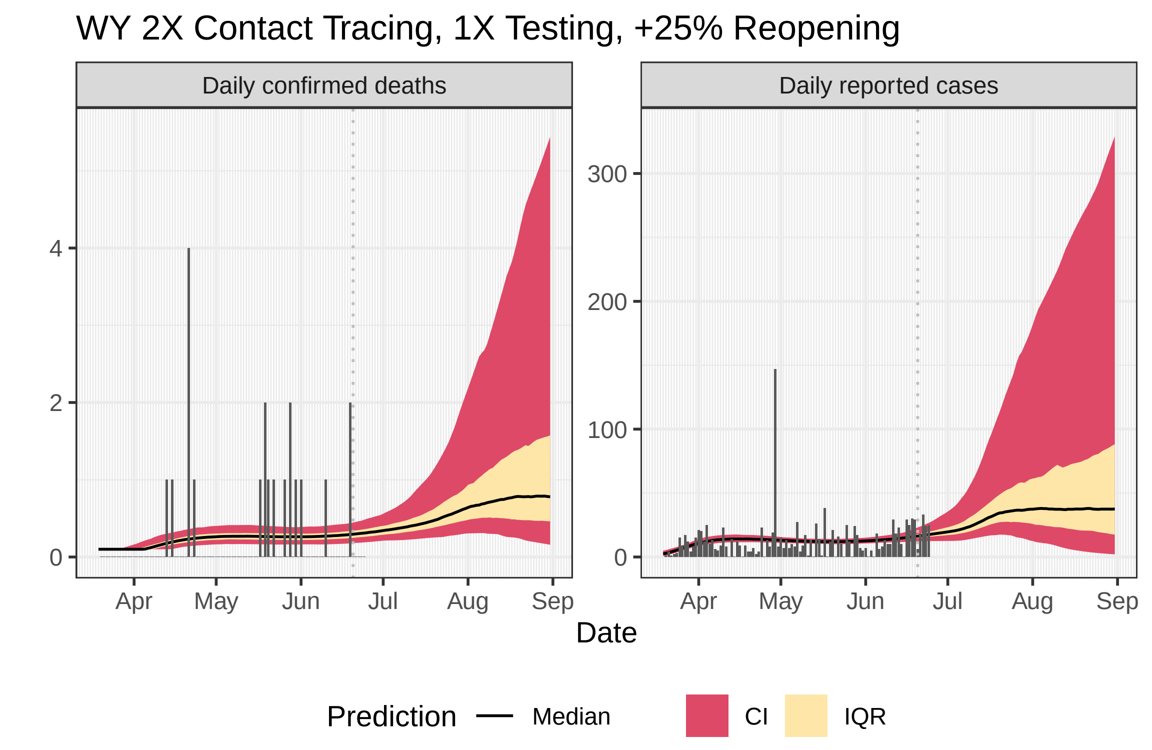

WY 2X Contact Tracing, 1X Testing, +25% Reopening
Daily confirmed deaths
Daily reported cases
0
2
4
0
100
200
300
Apr
May
Jun
Jul
Aug
Sep
Date
Prediction
Median
CI
IQR

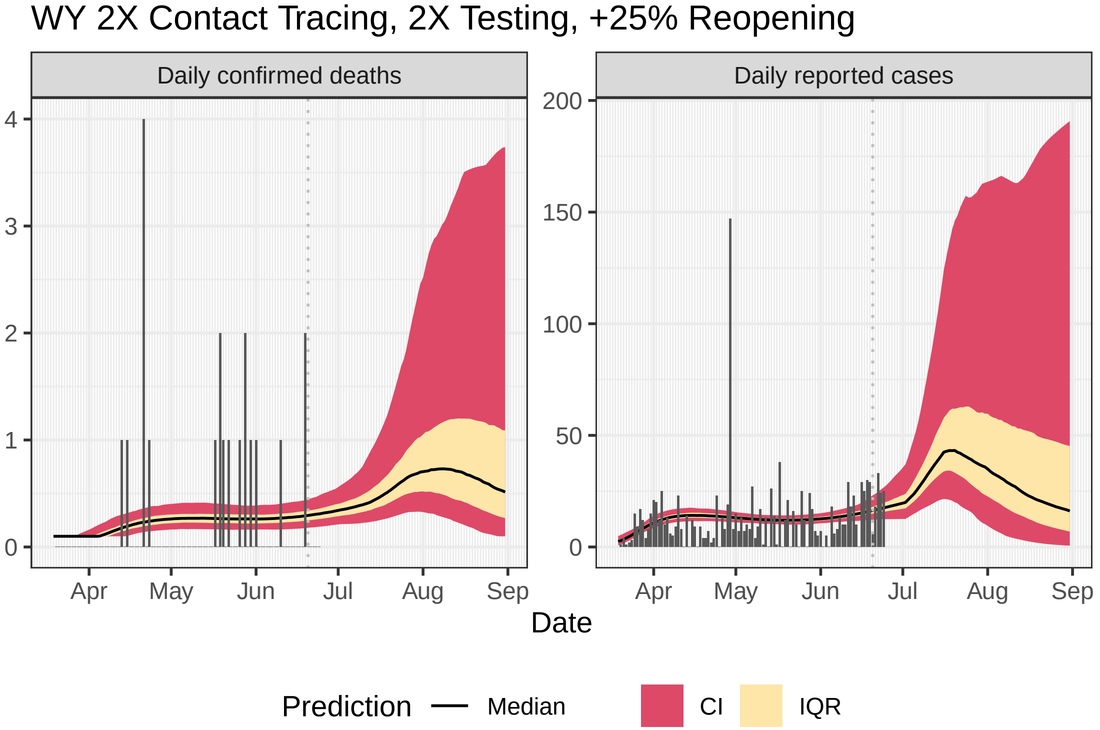
WY 2X Contact Tracing, 2X Testing, +25% Reopening
Daily confirmed deaths
Daily reported cases
0
1
2
3
4
0
50
100
150
200
Apr
May
Jun
Jul
Aug
Sep
Date
Prediction
Median
CI
IQR

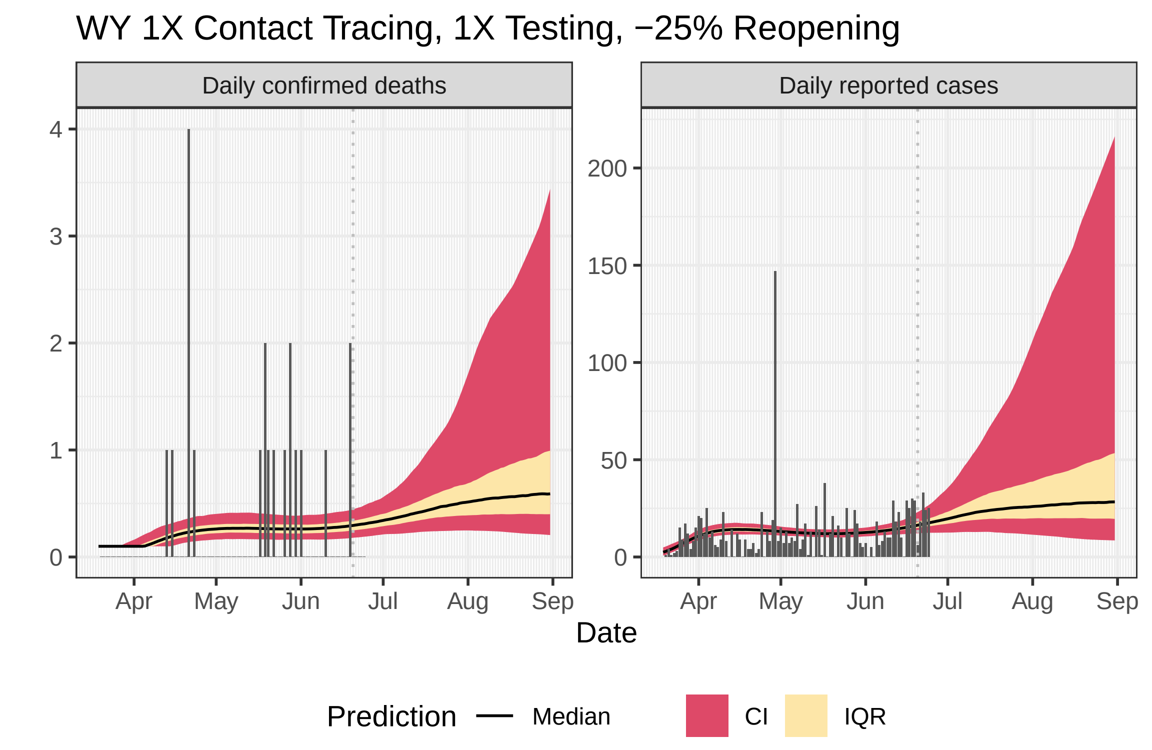

WY 1X Contact Tracing, 1X Testing, −25% Reopening
Daily confirmed deaths
Daily reported cases
4
3
2
1
0
200
150
100
50
0
Apr
May
Jun
Jul
Aug
Sep
Date
Prediction
Median
CI
IQR

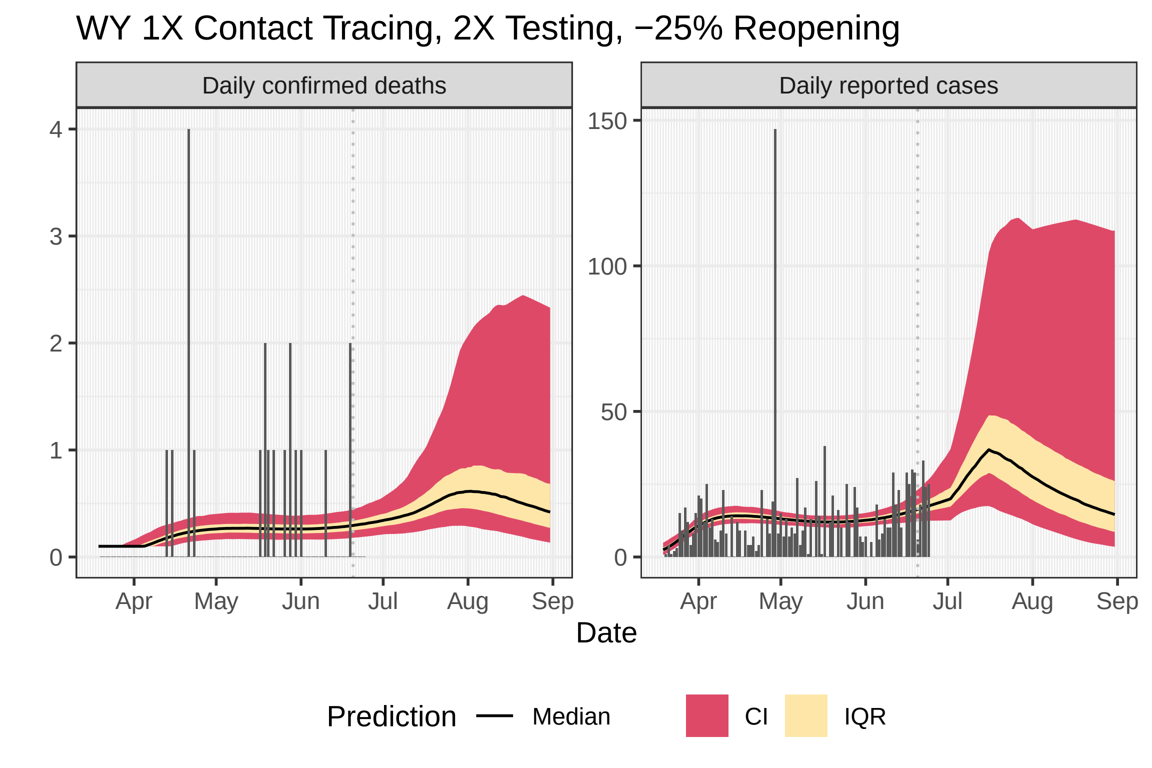
WY 1X Contact Tracing, 2X Testing, −25% Reopening
Daily confirmed deaths
Daily reported cases
0
1
2
3
4
0
50
100
150
Apr
May
Jun
Jul
Aug
Sep
Date
Prediction
Median
CI
IQR

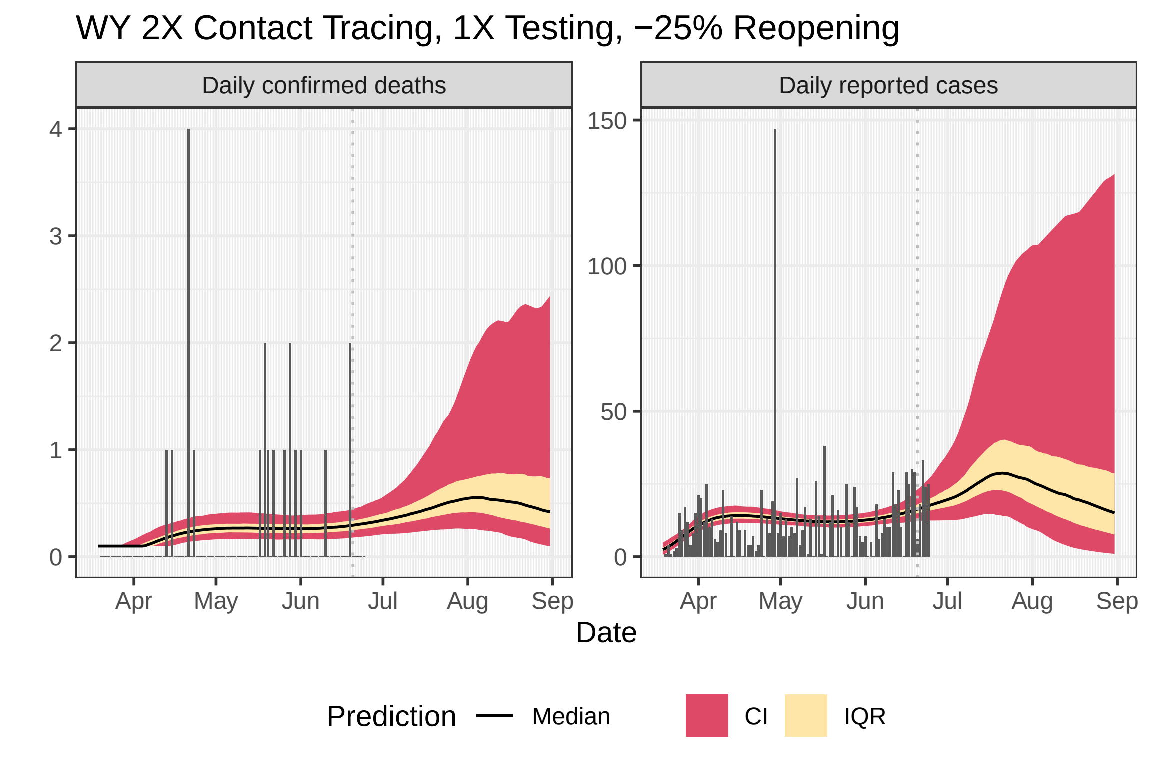

WY 2X Contact Tracing, 1X Testing, −25% Reopening
Daily confirmed deaths
Daily reported cases
0
1
2
3
4
0
50
100
150
Apr
May
Jun
Jul
Aug
Sep
Date
Prediction
Median
CI
IQR

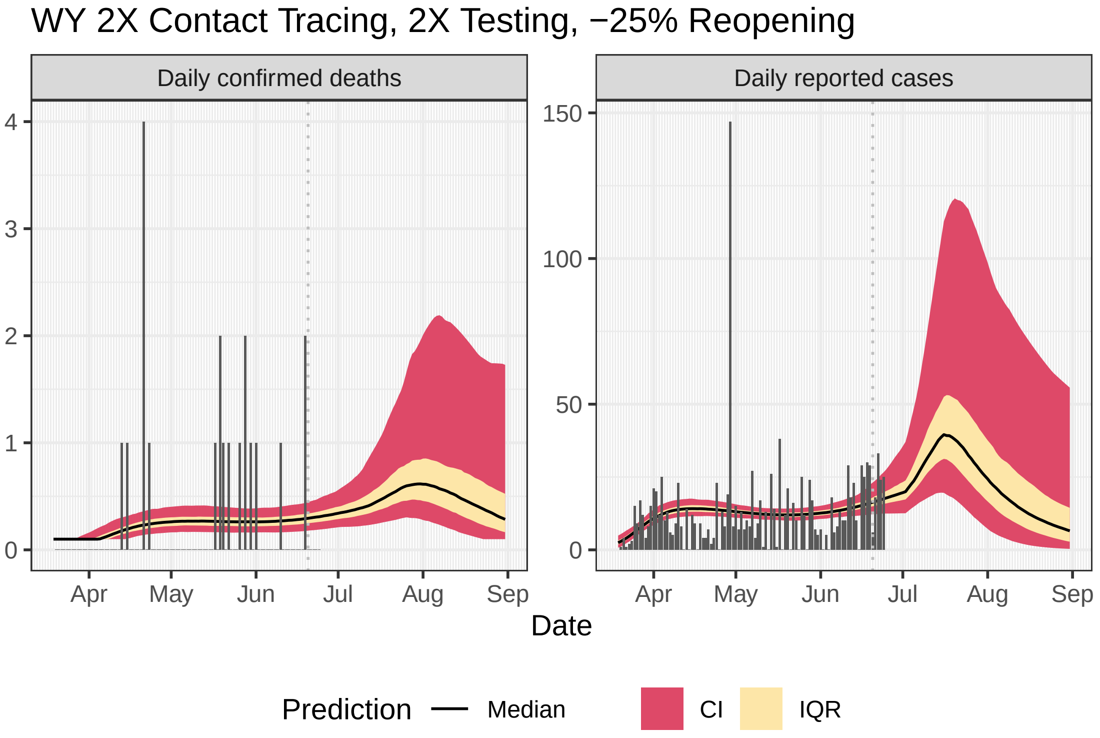

WY 2X Contact Tracing, 2X Testing, −25% Reopening
Daily confirmed deaths
Daily reported cases
0
1
2
3
4
0
50
100
150
Apr
May
Jun
Jul
Aug
Sep
Date
Prediction
Median
CI
IQR
