# Supplementary material for: State-level impact of social distancing and testing on COVID-19 in the United States
Source: Res Sq. 2020 Jul 7:rs.3.rs-40364. Preprint. [Version 1] doi: 10.21203/rs.3.rs-40364/v1 (PMC7362894; doi:10.21203/rs.3.rs-40364/v1)
Supplement: Supplementary file 2 [file FigS2validationdaily.pdf]

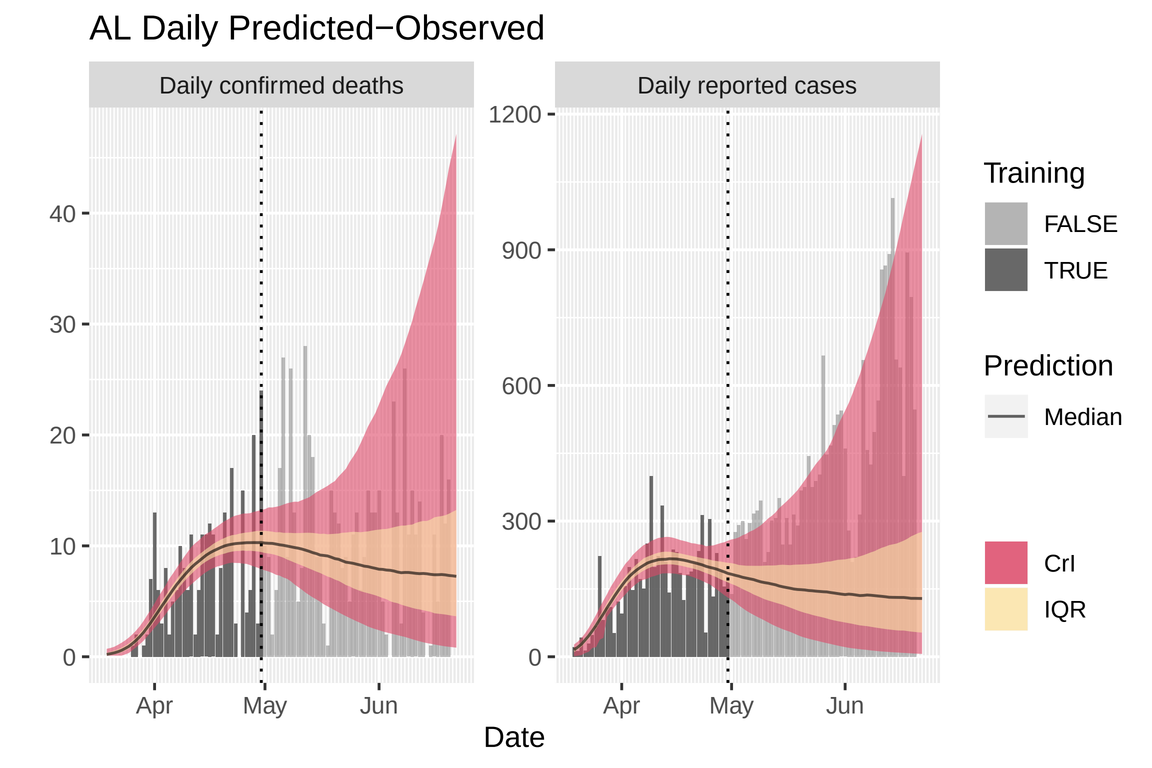
AL Daily Predicted−Observed
Daily confirmed deaths
Daily reported cases
0
10
20
30
40
0
300
600
900
1200
Apr
May
Jun
Date
Training
FALSE
TRUE
Prediction
Median
CrI
IQR

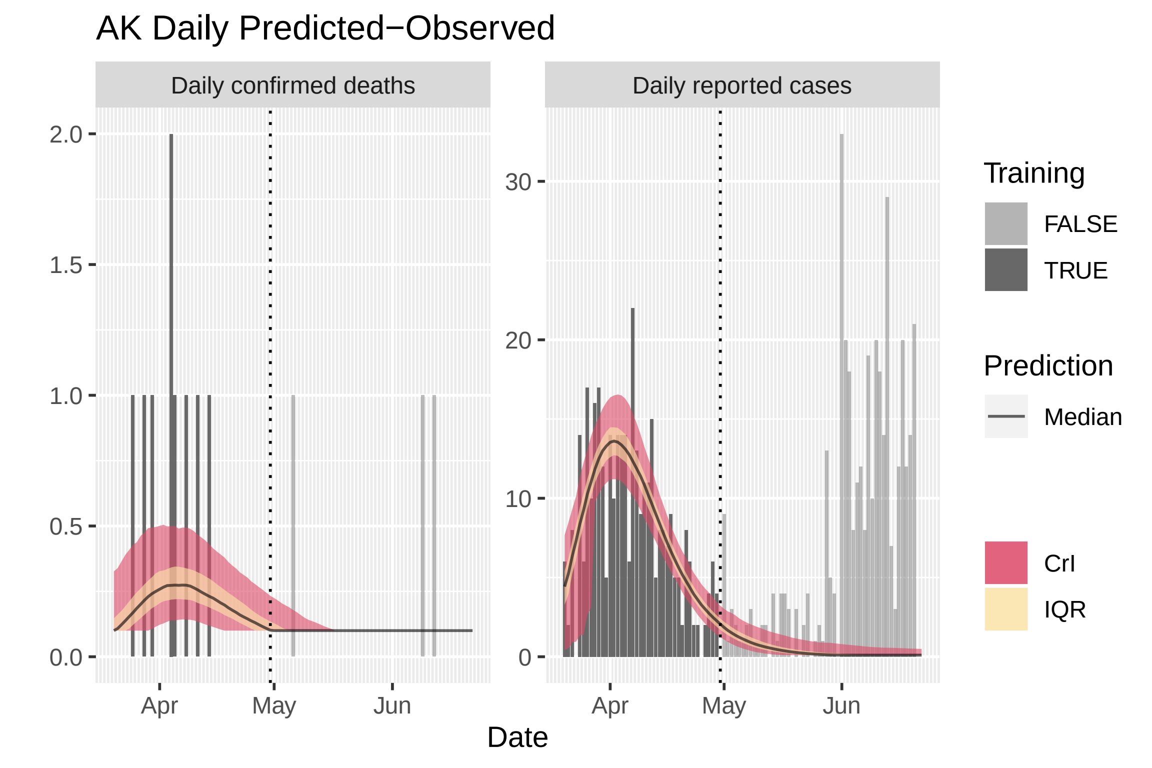
AK Daily Predicted−Observed
Daily confirmed deaths
Daily reported cases
2.0
1.5
1.0
0.5
0.0
30
20
10
0
Apr
May
Jun
Date
Training
FALSE
TRUE
Prediction
Median
CrI
IQR

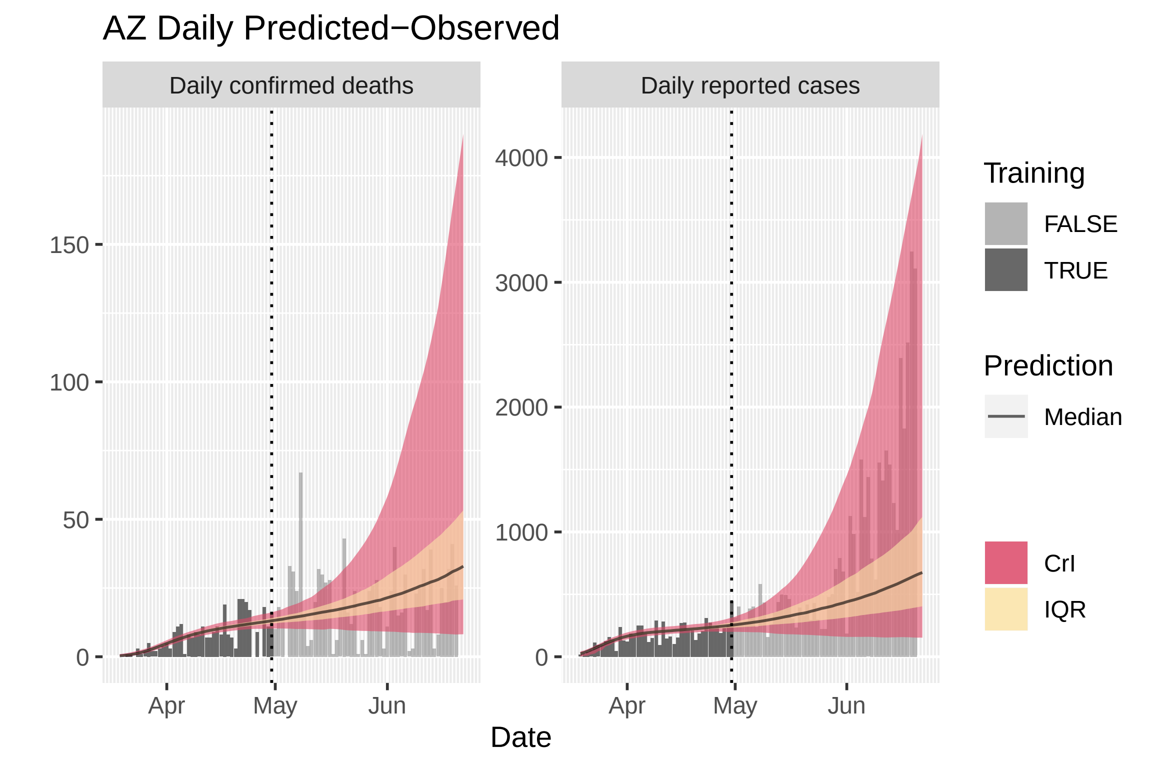

AZ Daily Predicted−Observed
Daily confirmed deaths
Daily reported cases
0
50
100
150
0
1000
2000
3000
4000
Apr
May
Jun
Apr
May
Jun
Date
Training
FALSE
TRUE
Prediction
Median
CrI
IQR

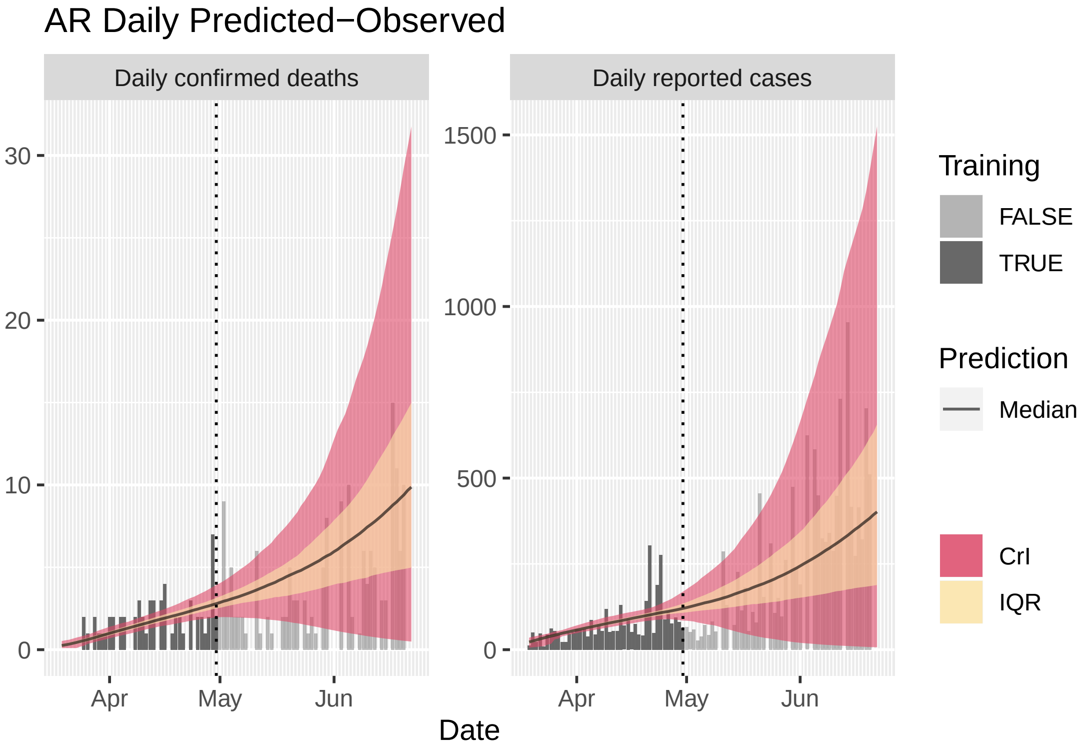

AR Daily Predicted−Observed
Daily confirmed deaths
Daily reported cases
30
20
10
0
1500
1000
500
0
Apr
May
Jun
Date
Training
FALSE
TRUE
Prediction
Median
CrI
IQR

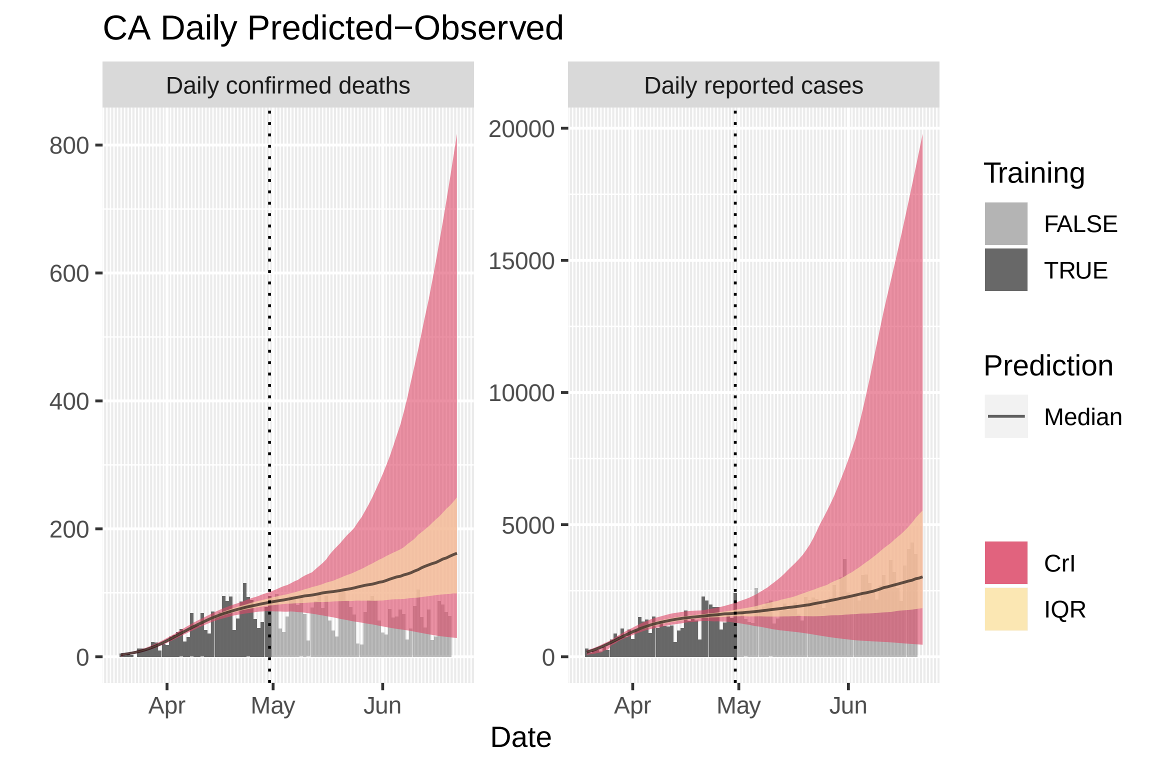
CA Daily Predicted−Observed
Daily confirmed deaths
Daily reported cases
800
600
400
200
0
20000
15000
10000
5000
0
Apr
May
Jun
Date
Training
FALSE
TRUE
Prediction
Median
CrI
IQR

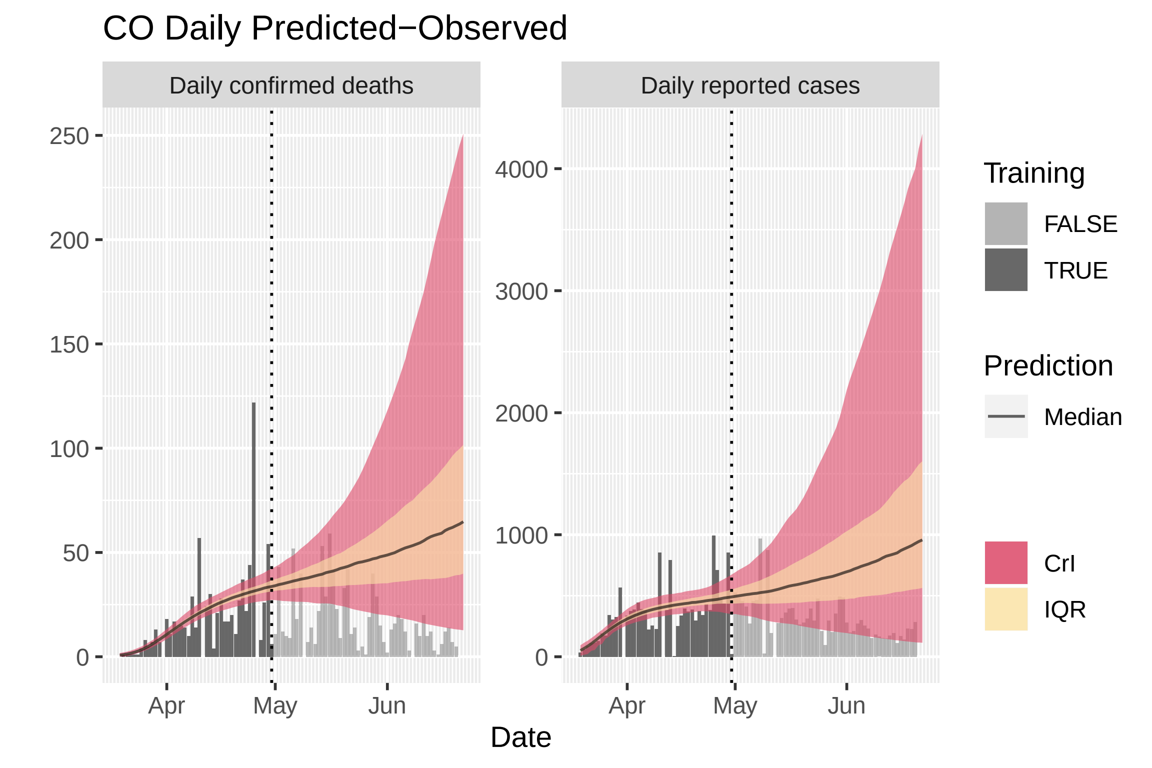

CO Daily Predicted−Observed
Daily confirmed deaths
Daily reported cases
250
200
150
100
50
0
4000
3000
2000
1000
0
Apr
May
Jun
Date
Training
FALSE
TRUE
Prediction
Median
CrI
IQR

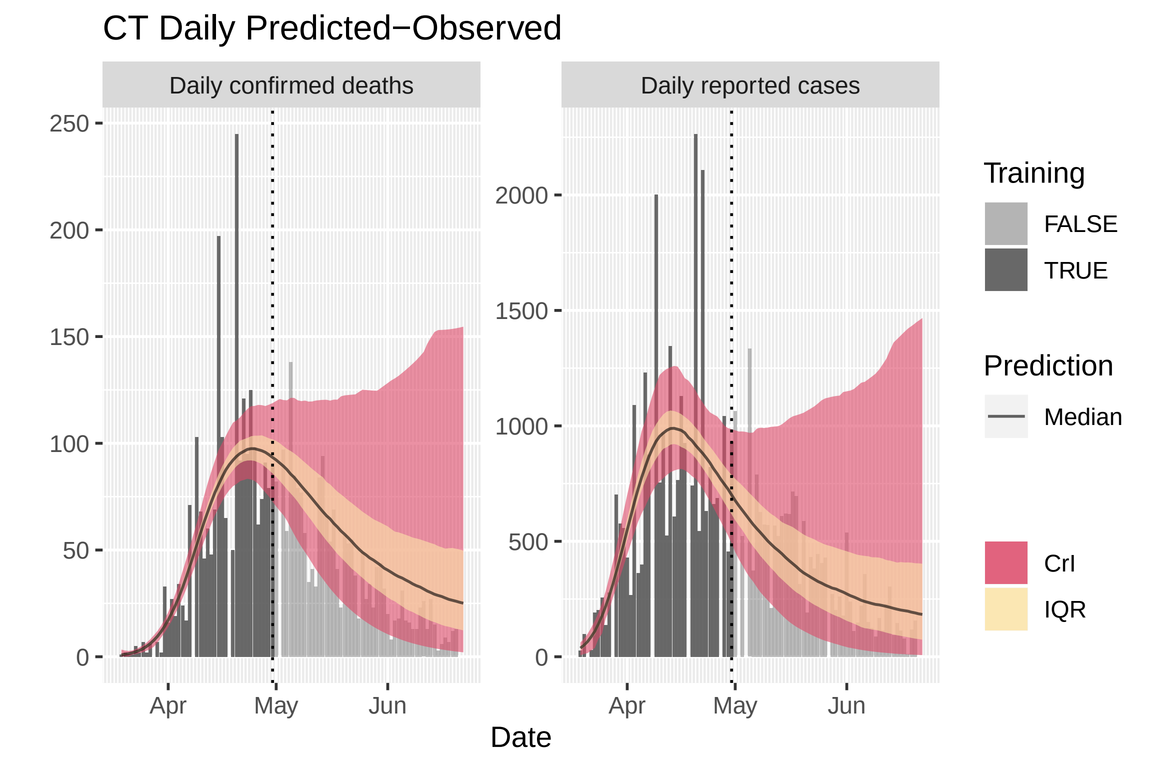

CT Daily Predicted−Observed
Daily confirmed deaths
Daily reported cases
250
200
150
100
50
0
2000
1500
1000
500
0
Apr
May
Jun
Date
Training
FALSE
TRUE
Prediction
Median
CrI
IQR

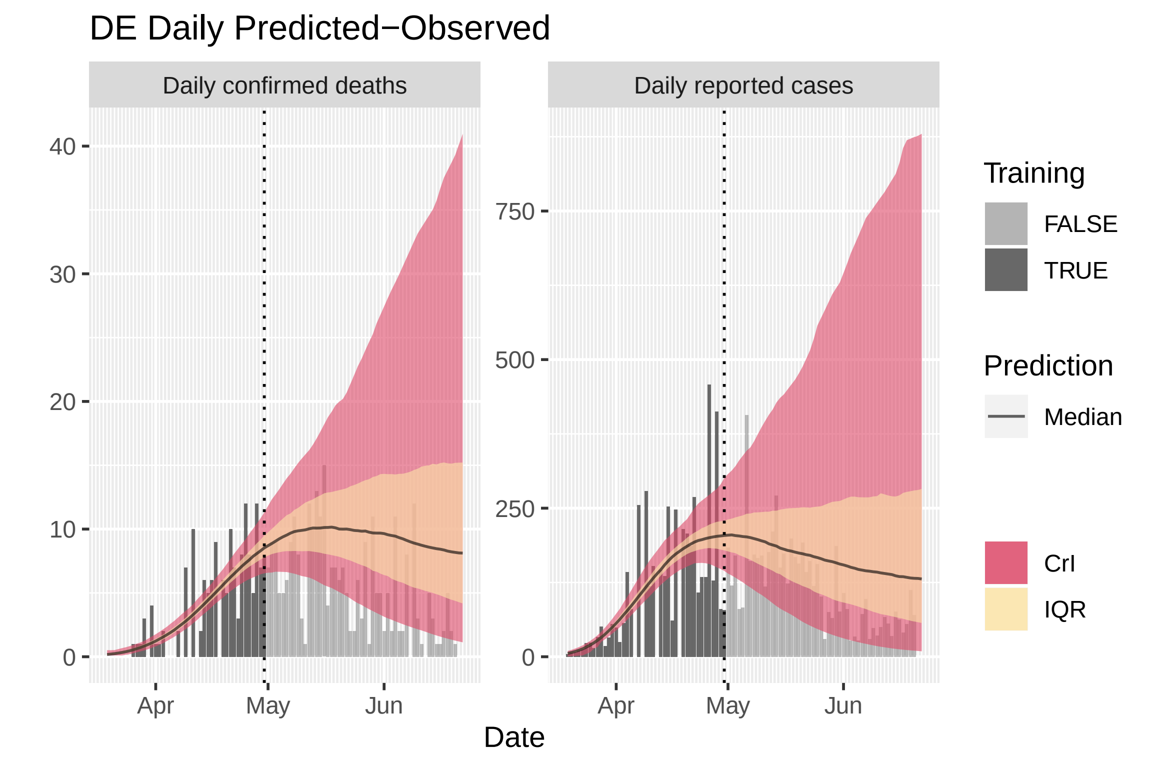
DE Daily Predicted−Observed
Daily confirmed deaths
Daily reported cases
40
30
20
10
0
750
500
250
0
Apr
May
Jun
Date
Training
FALSE
TRUE
Prediction
Median
CrI
IQR

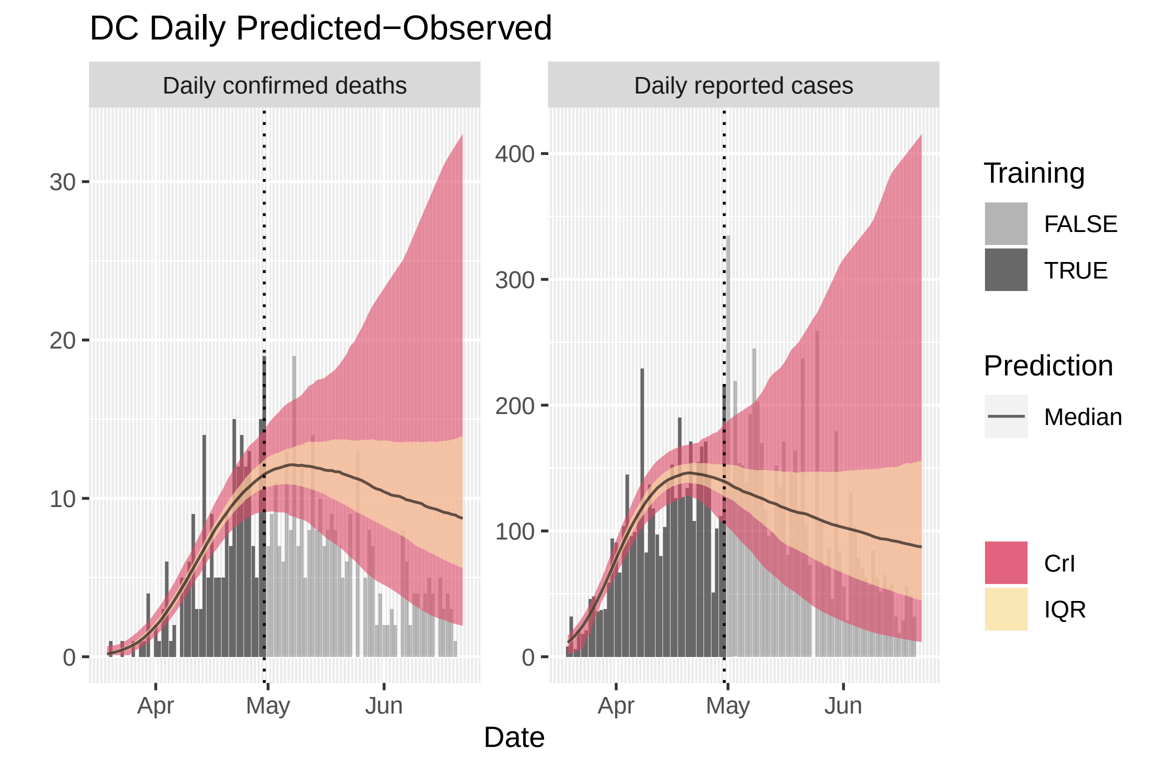
DC Daily Predicted−Observed
Daily confirmed deaths
Daily reported cases
30
20
10
0
400
300
200
100
0
Apr
May
Jun
Date
Training
FALSE
TRUE
Prediction
Median
CrI
IQR

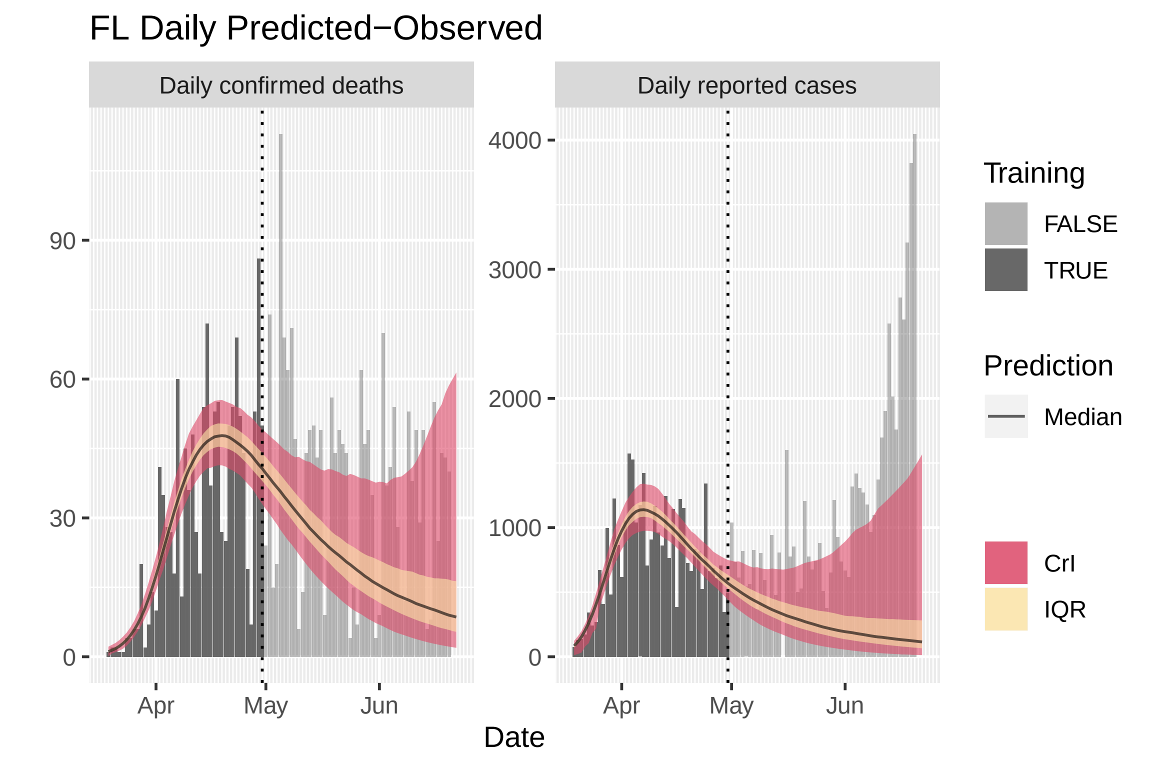

FL Daily Predicted−Observed
Daily confirmed deaths
Daily reported cases
0
30
60
90
0
1000
2000
3000
4000
Apr
May
Jun
Date
Training
FALSE
TRUE
Prediction
Median
CrI
IQR

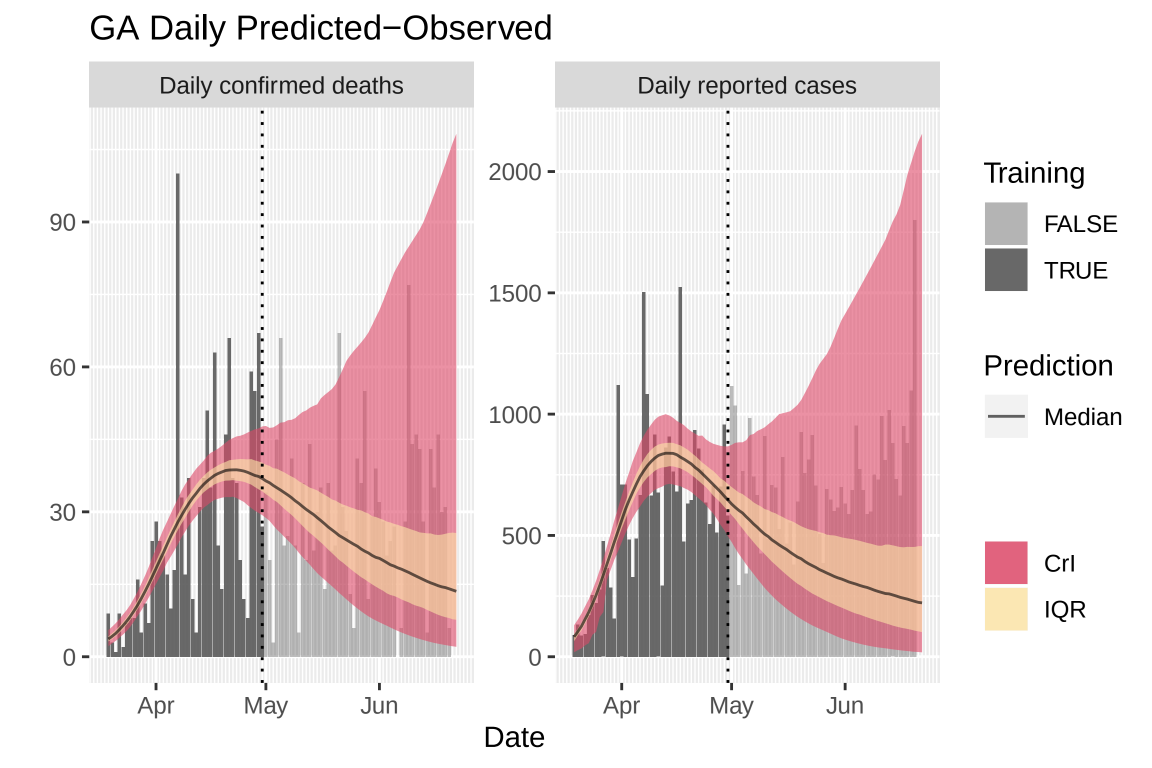
GA Daily Predicted−Observed
Daily confirmed deaths
Daily reported cases
0
30
60
90
0
500
1000
1500
2000
Apr
May
Jun
Date
Training
FALSE
TRUE
Prediction
Median
CrI
IQR

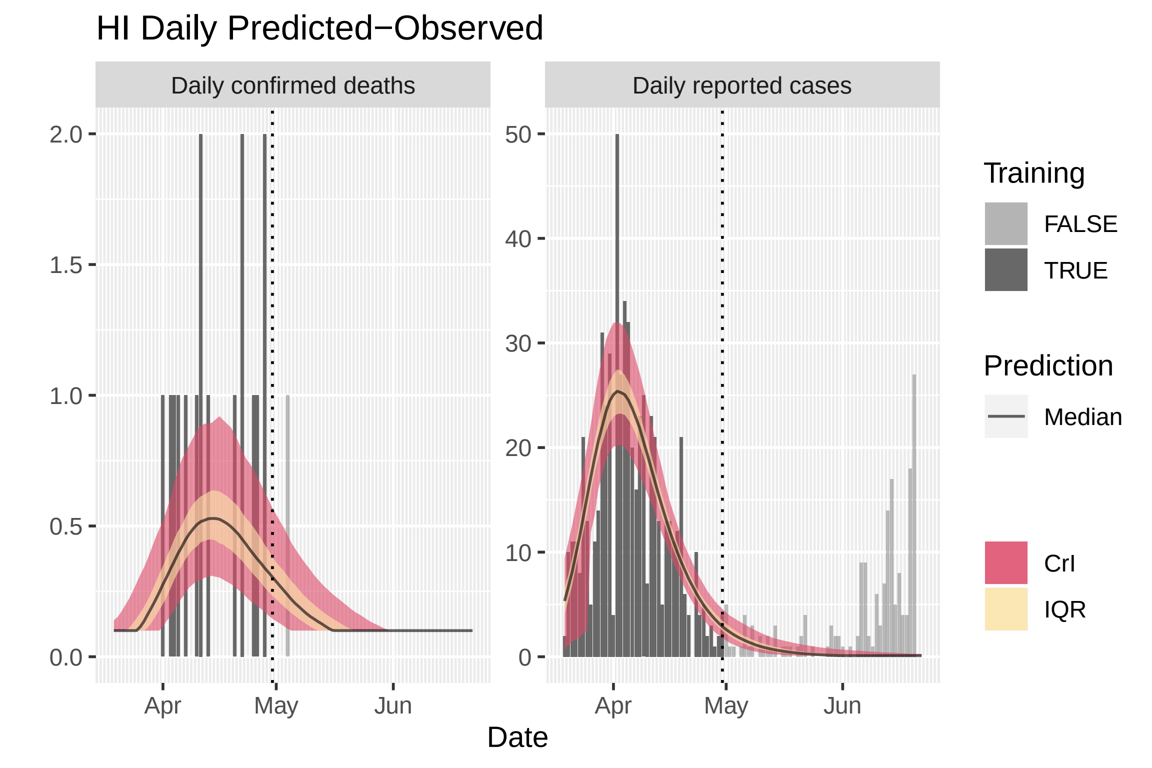

HI Daily Predicted−Observed
Daily confirmed deaths
Daily reported cases
2.0
1.5
1.0
0.5
0.0
50
40
30
20
10
0
Apr
May
Jun
Date
Training
FALSE
TRUE
Prediction
Median
CrI
IQR

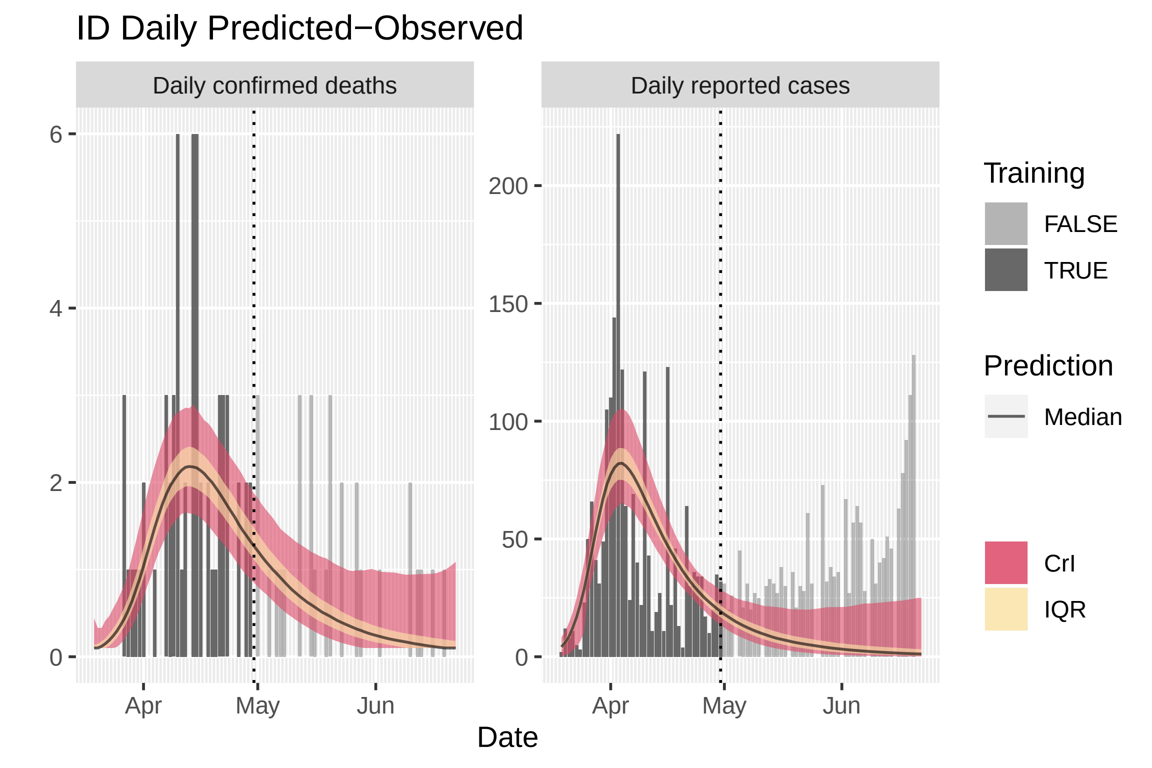

ID Daily Predicted−Observed
Daily confirmed deaths
Daily reported cases
0
2
4
6
50
100
150
200
Apr
May
Jun
Date
Training
FALSE
TRUE
Prediction
Median
CrI
IQR

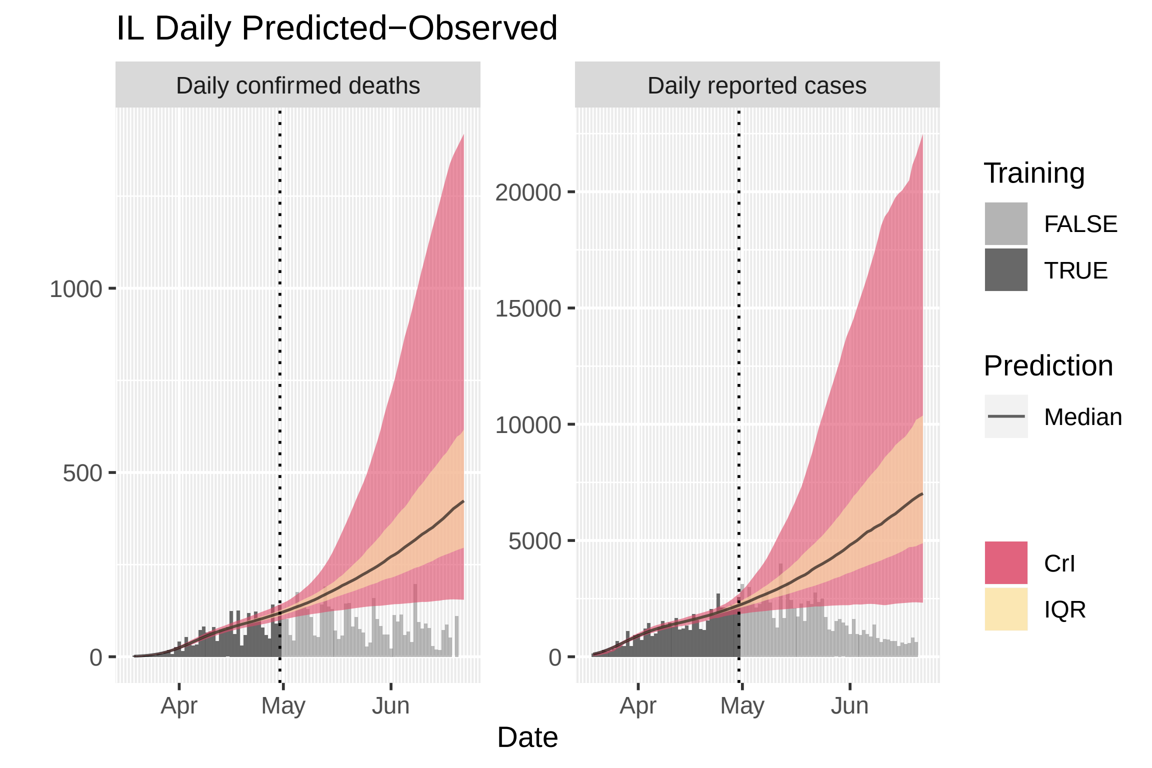
IL Daily Predicted−Observed
Daily confirmed deaths
Daily reported cases
1000
500
0
20000
15000
10000
5000
0
Apr
May
Jun
Date
Training
FALSE
TRUE
Prediction
Median
CrI
IQR

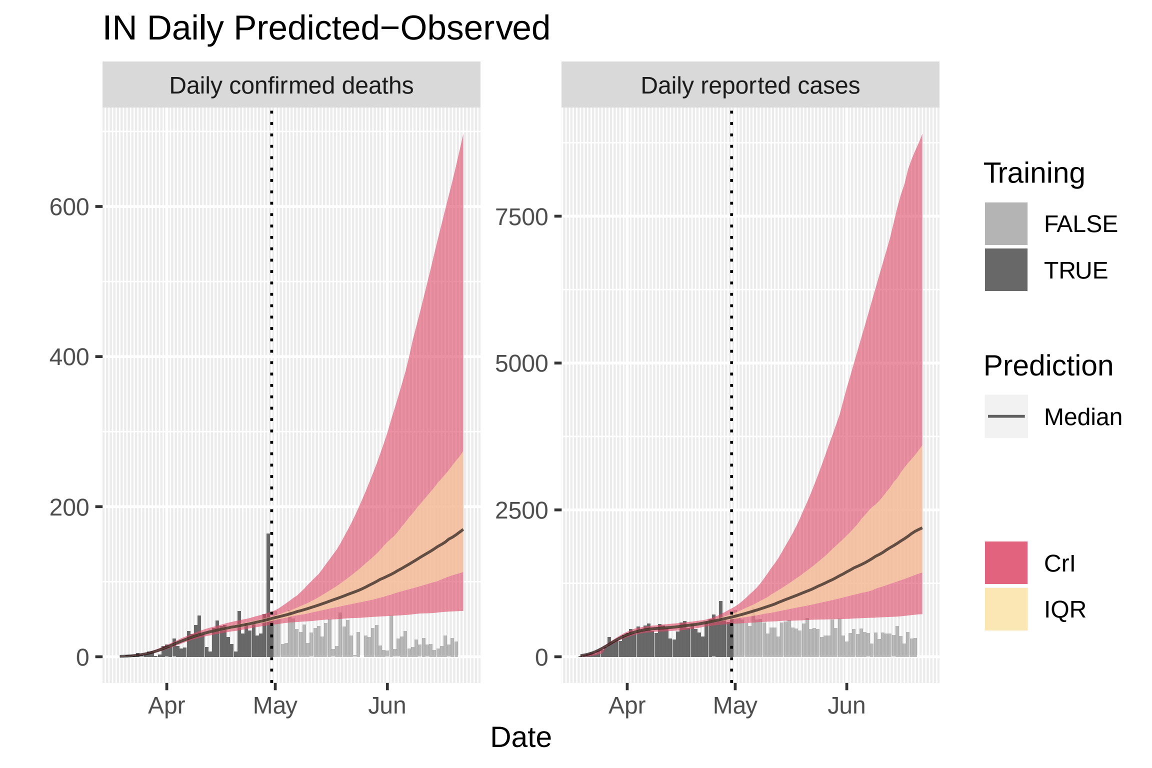
IN Daily Predicted−Observed
Daily confirmed deaths
Daily reported cases
600
400
200
0
7500
5000
2500
0
Apr
May
Jun
Date
Training
FALSE
TRUE
Prediction
Median
CrI
IQR

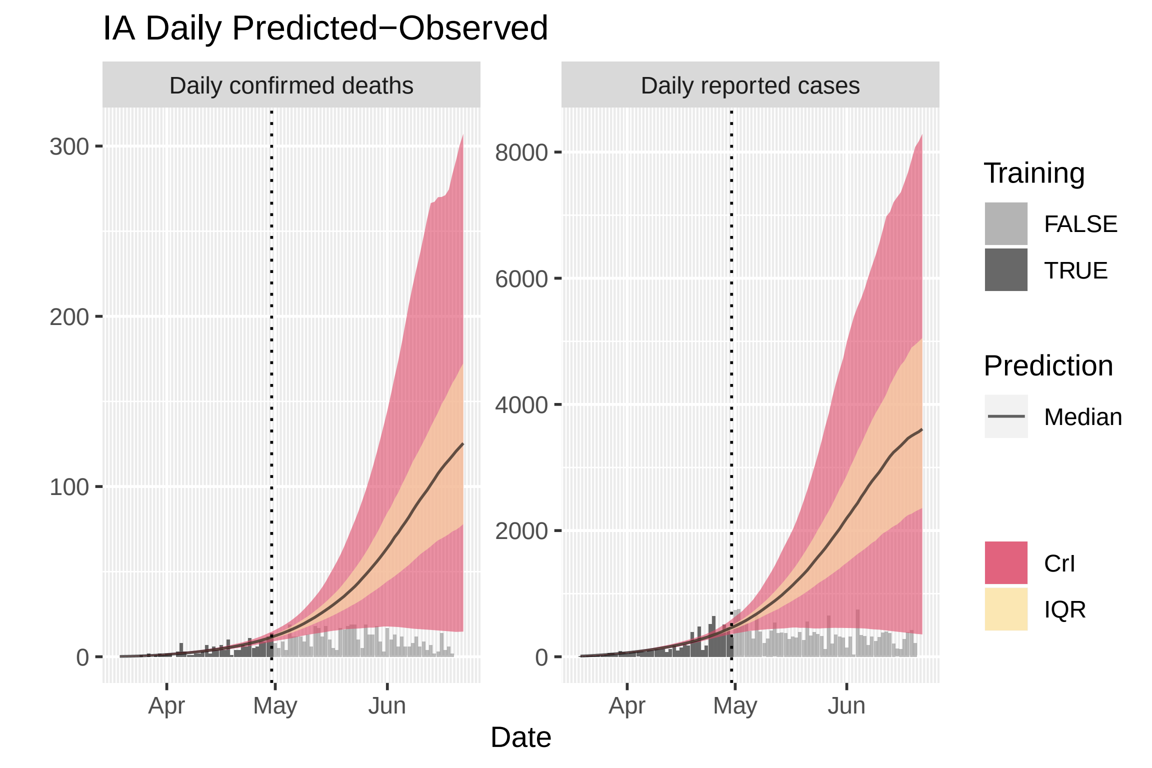
IA Daily Predicted−Observed
Daily confirmed deaths
Daily reported cases
300
200
100
0
8000
6000
4000
2000
0
Apr
May
Jun
Date
Training
FALSE
TRUE
Prediction
Median
CrI
IQR

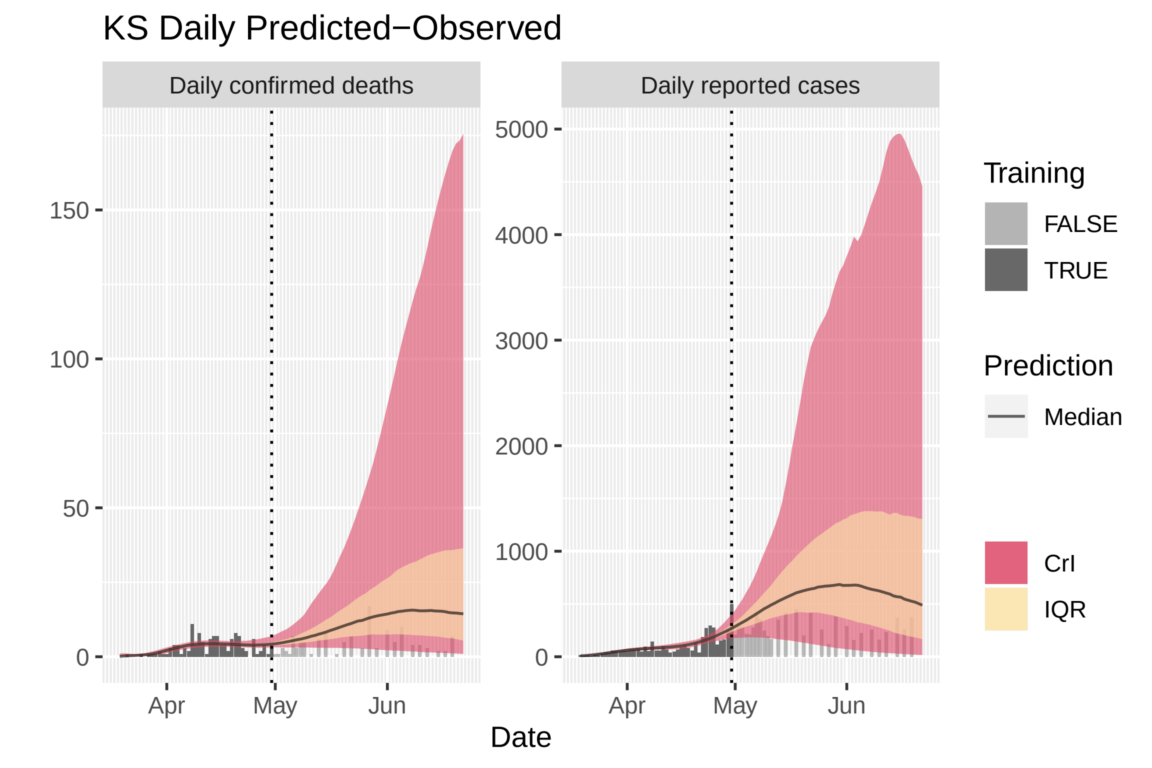

KS Daily Predicted−Observed
Daily confirmed deaths
Daily reported cases
0
50
100
150
0
1000
2000
3000
4000
5000
Apr
May
Jun
Date
Training
FALSE
TRUE
Prediction
Median
CrI
IQR

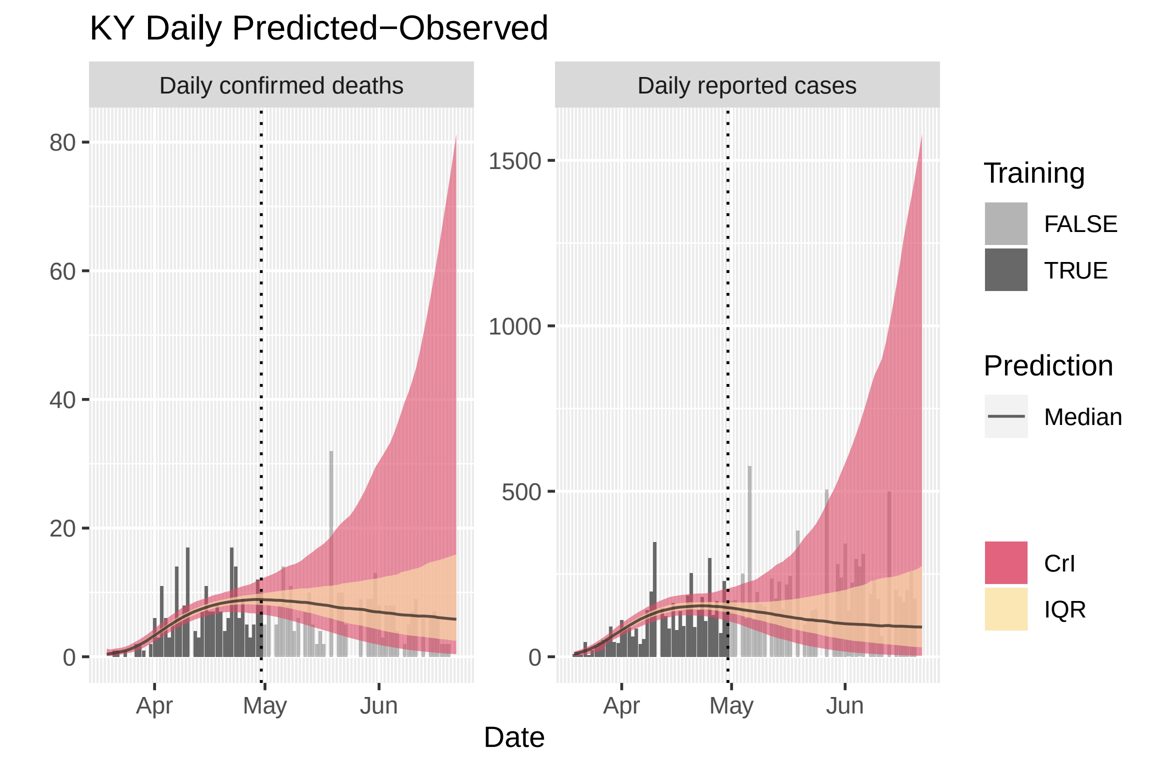

KY Daily Predicted−Observed
Daily confirmed deaths
Daily reported cases
80
60
40
20
0
1500
1000
500
0
Apr
May
Jun
Date
Training
FALSE
TRUE
Prediction
Median
CrI
IQR

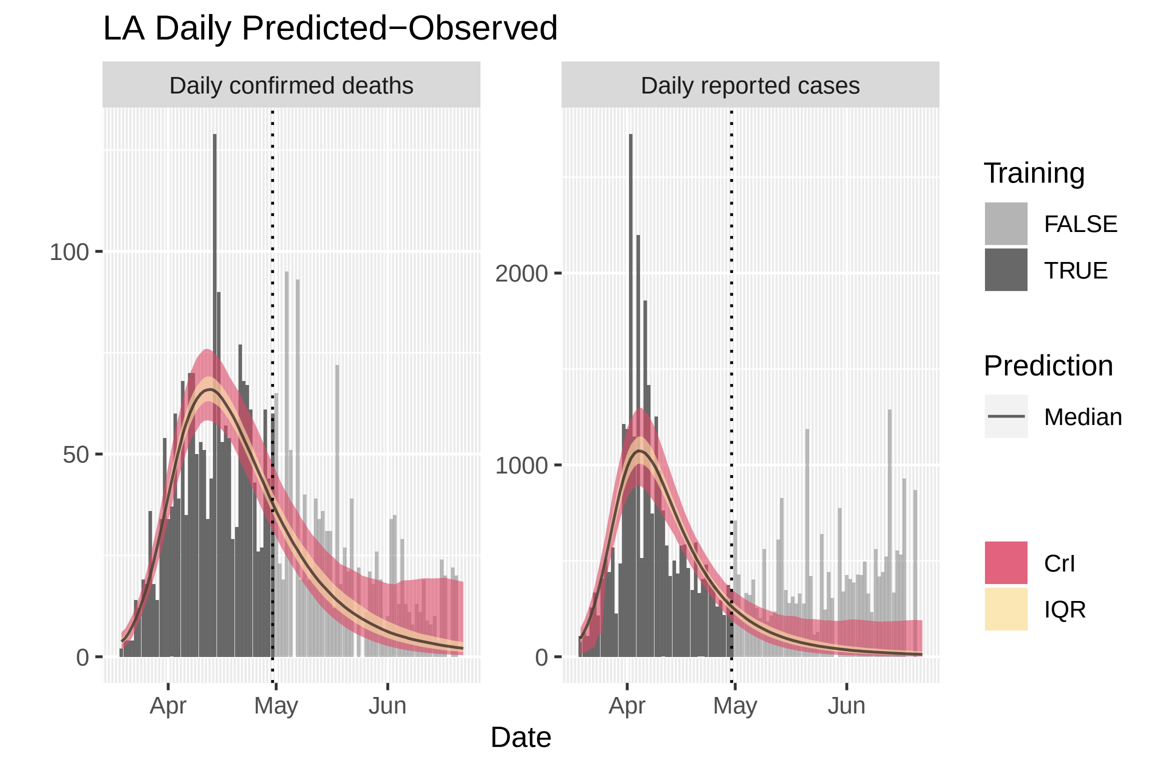
LA Daily Predicted−Observed
Daily confirmed deaths
Daily reported cases
100
50
0
2000
1000
0
Apr
May
Jun
Date
Training
FALSE
TRUE
Prediction
Median
CrI
IQR

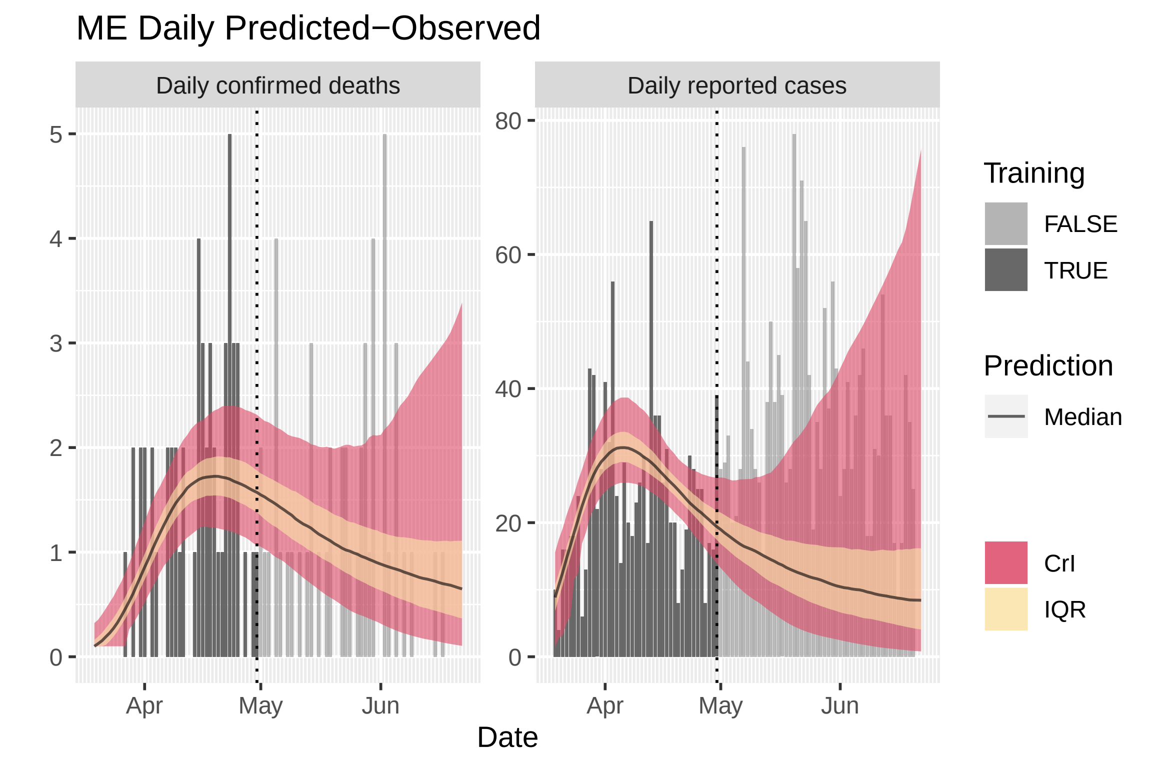
ME Daily Predicted−Observed
Daily confirmed deaths
Daily reported cases
0
1
2
3
4
5
0
20
40
60
80
Apr
May
Jun
Apr
May
Jun
Date
Training
FALSE
TRUE
Prediction
Median
CrI
IQR

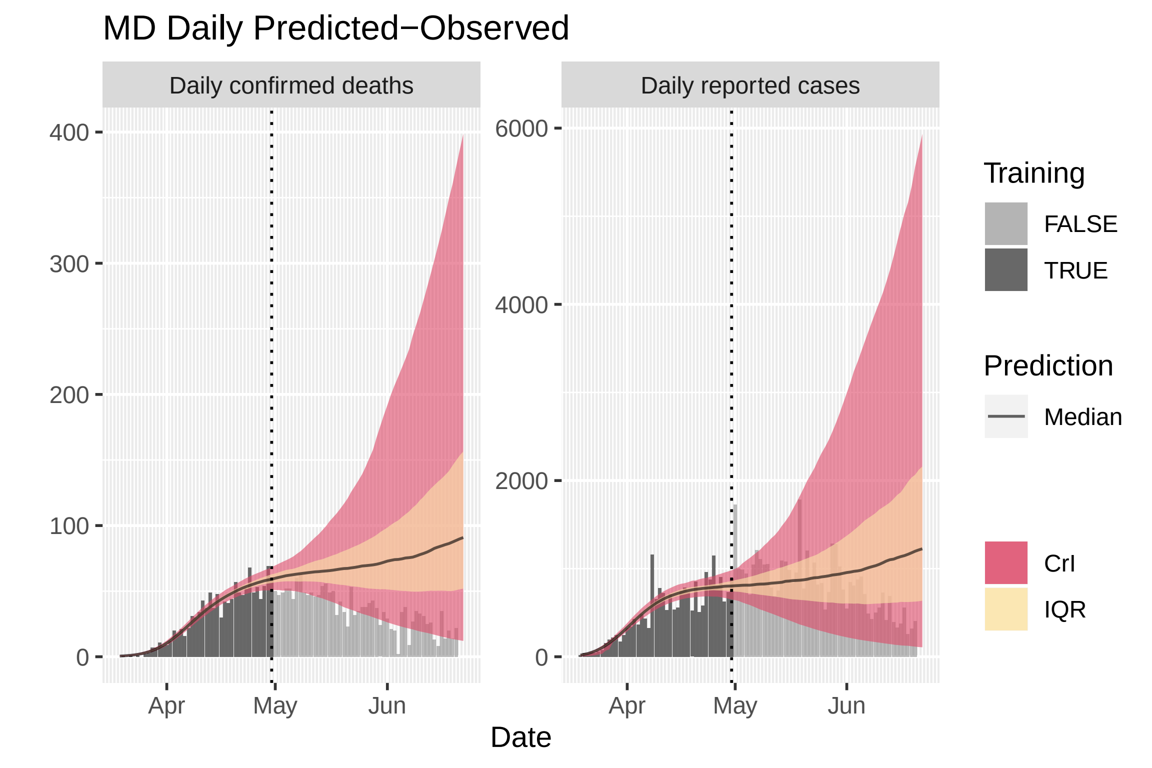

MD Daily Predicted−Observed
Daily confirmed deaths
Daily reported cases
400
300
200
100
0
6000
4000
2000
0
Apr
May
Jun
Date
Training
FALSE
TRUE
Prediction
Median
CrI
IQR

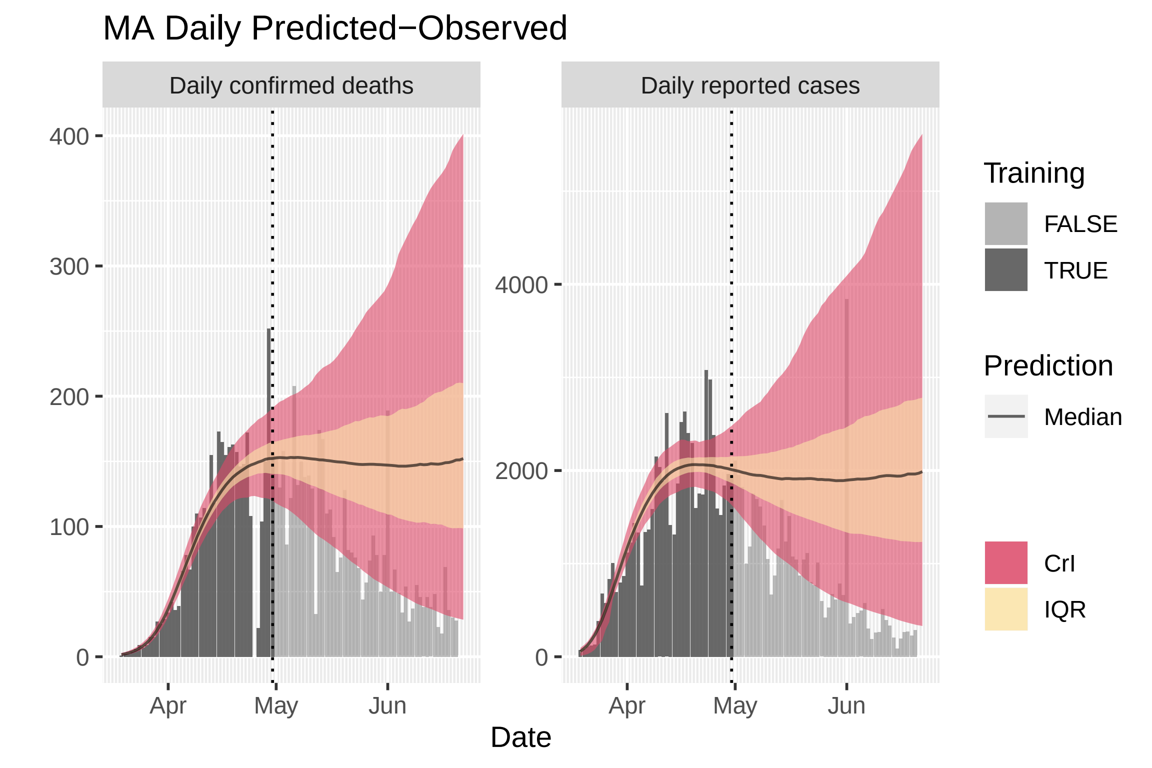

MA Daily Predicted−Observed
Daily confirmed deaths
Daily reported cases
400
300
200
100
0
4000
2000
0
Apr
May
Jun
Date
Training
FALSE
TRUE
Prediction
Median
CrI
IQR

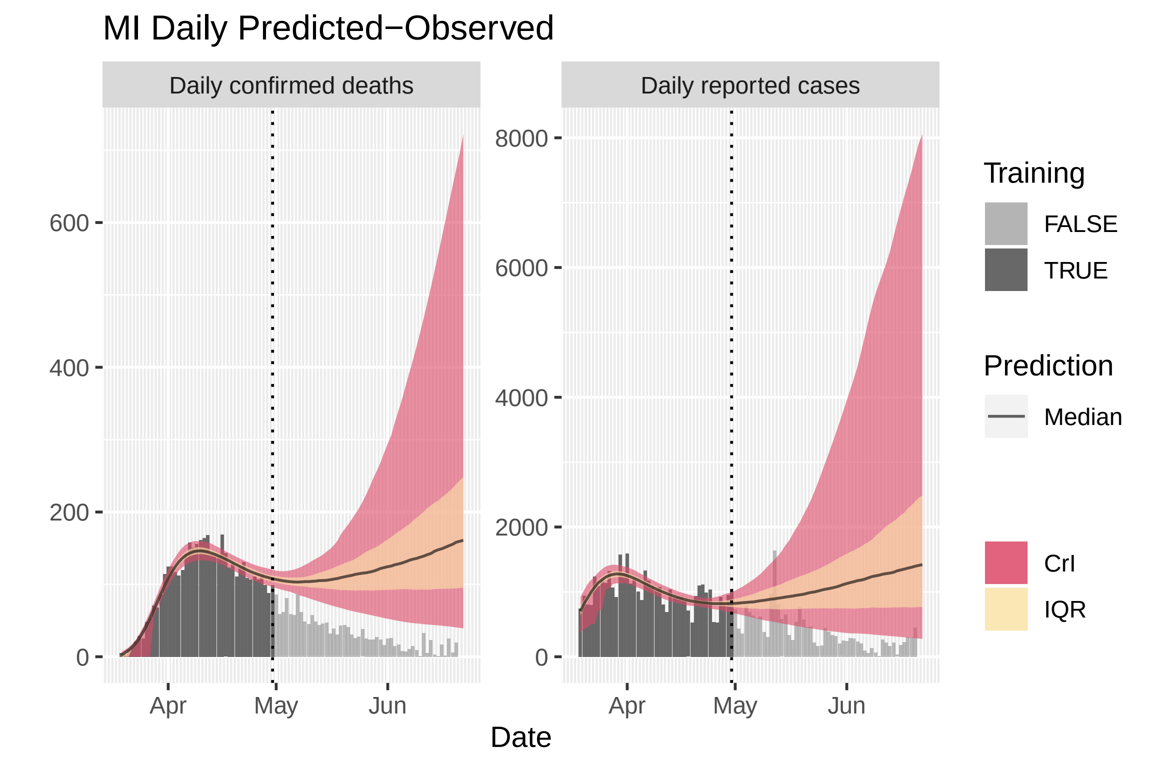
MI Daily Predicted−Observed
Daily confirmed deaths
Daily reported cases
600
400
200
0
8000
6000
4000
2000
0
Apr
May
Jun
Apr
May
Jun
Date
Training
FALSE
TRUE
Prediction
Median
CrI
IQR

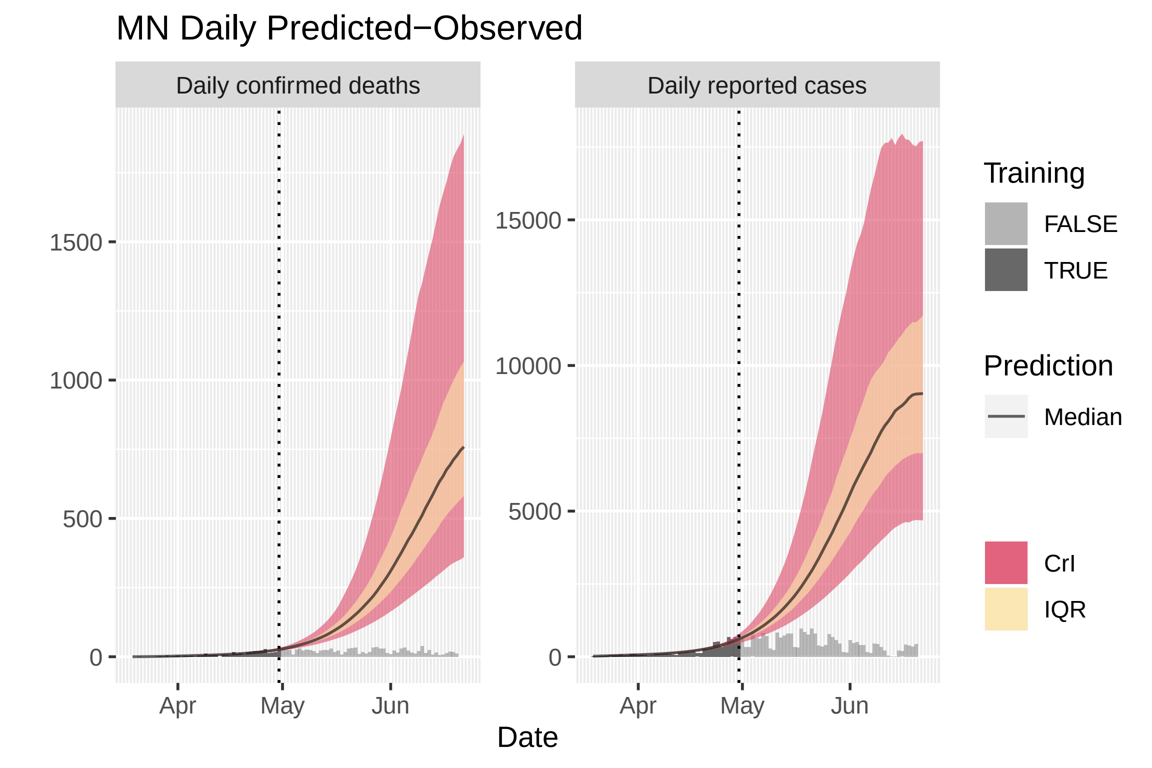

MN Daily Predicted−Observed
Daily confirmed deaths
Daily reported cases
1500
1000
500
0
15000
10000
5000
0
Apr
May
Jun
Date
Training
FALSE
TRUE
Prediction
Median
CrI
IQR

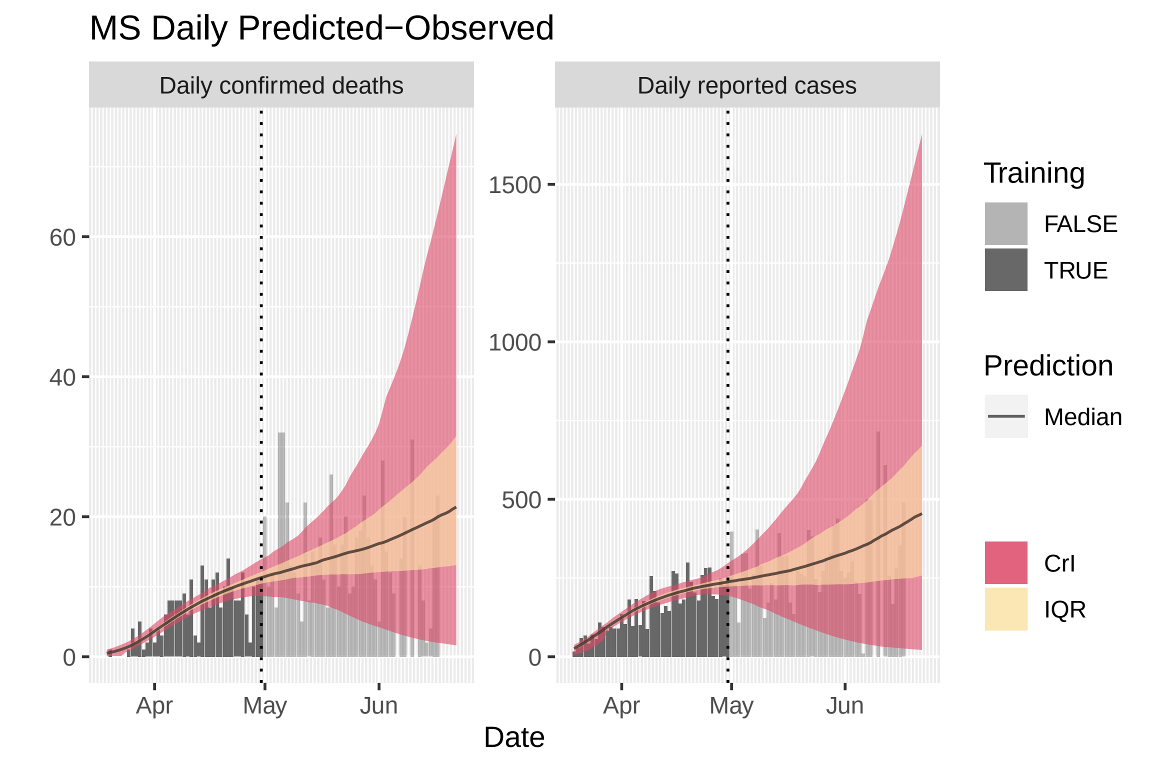

MS Daily Predicted−Observed
Daily confirmed deaths
Daily reported cases
0
20
40
60
500
1000
1500
Apr
May
Jun
Date
Training
FALSE
TRUE
Prediction
Median
CrI
IQR

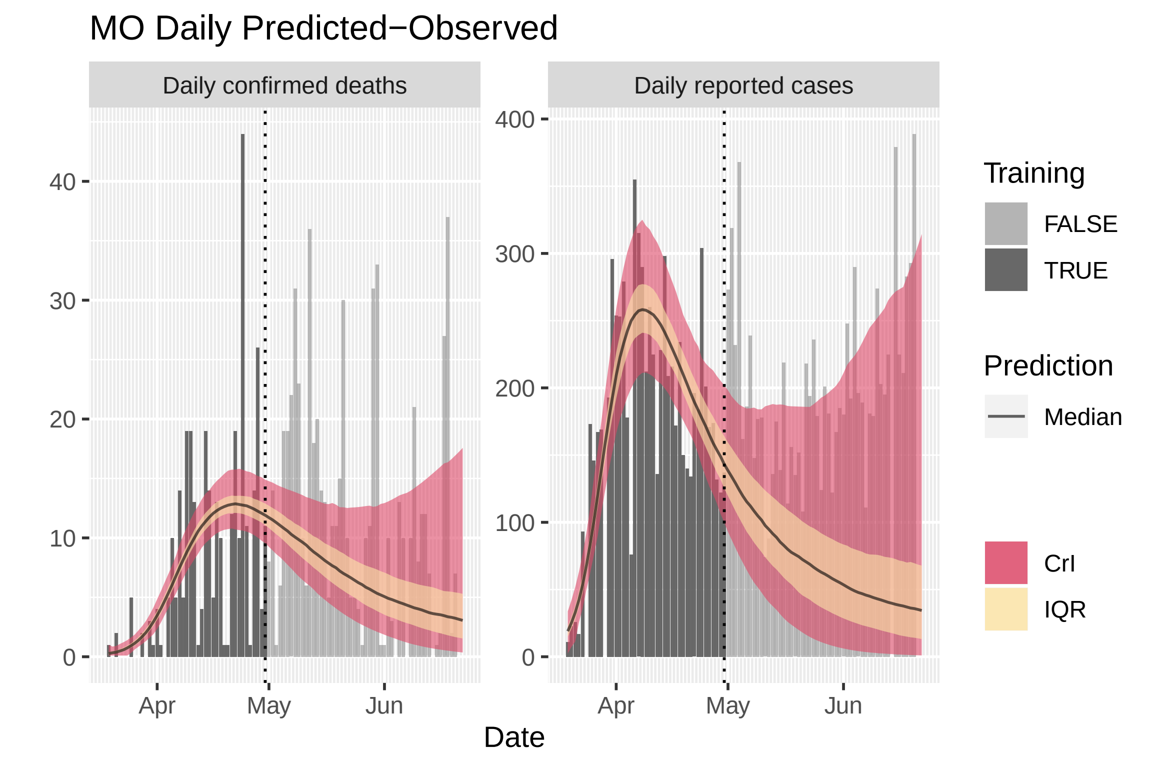
MO Daily Predicted−Observed
Daily confirmed deaths
Daily reported cases
40
30
20
10
0
400
300
200
100
0
Apr
May
Jun
Date
Training
FALSE
TRUE
Prediction
Median
CrI
IQR

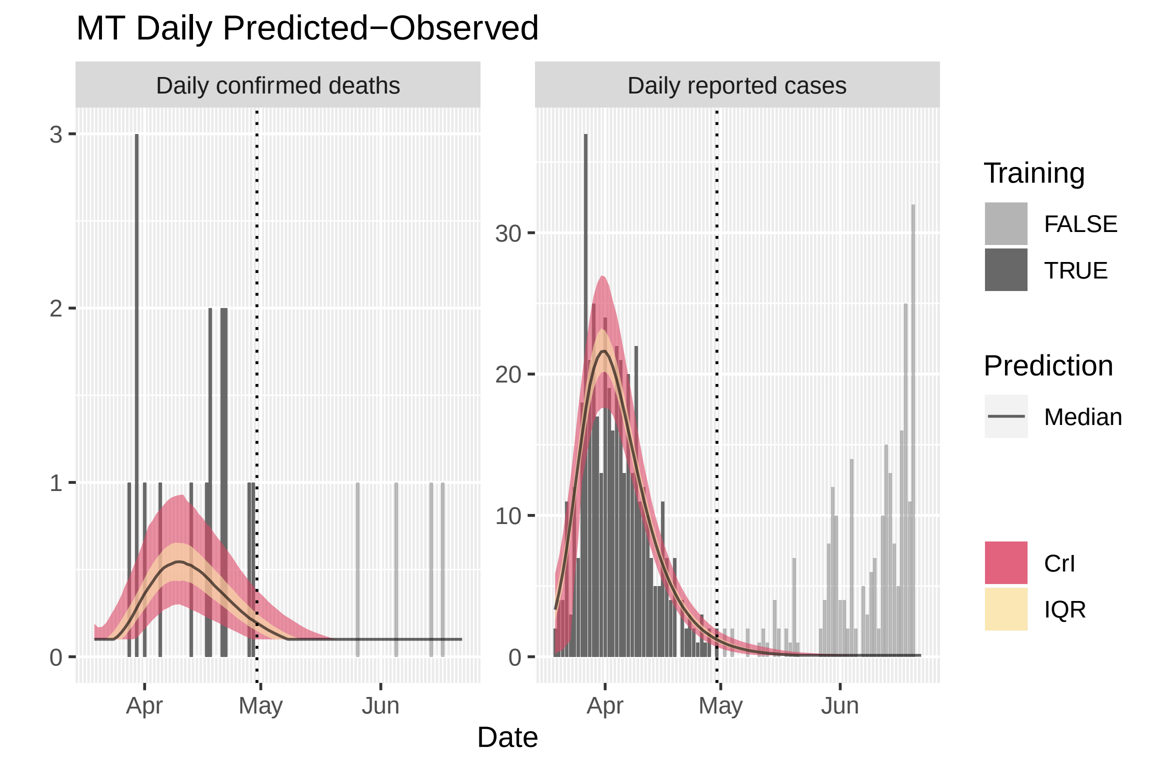

MT Daily Predicted−Observed
Daily confirmed deaths
Daily reported cases
0
1
2
3
10
20
30
Apr
May
Jun
Date
Training
FALSE
TRUE
Prediction
Median
CrI
IQR

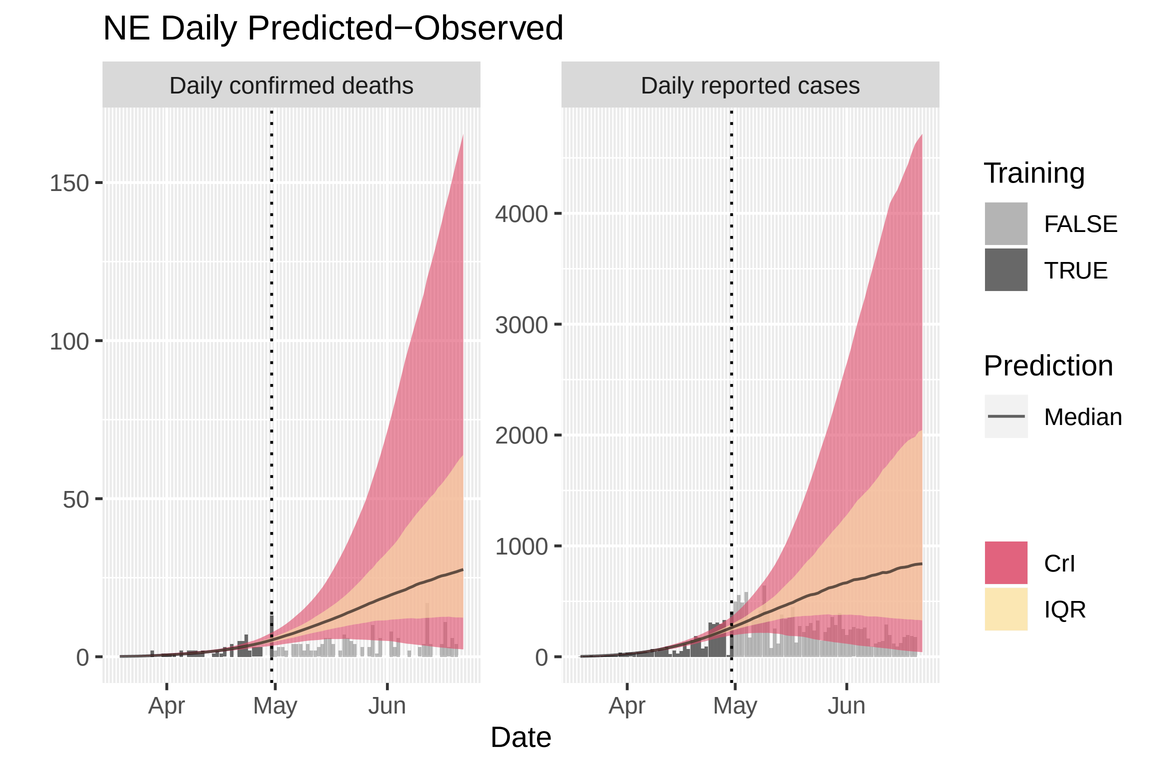

NE Daily Predicted−Observed
Daily confirmed deaths
Daily reported cases
150
100
50
0
4000
3000
2000
1000
0
Apr
May
Jun
Date
Training
FALSE
TRUE
Prediction
Median
CrI
IQR

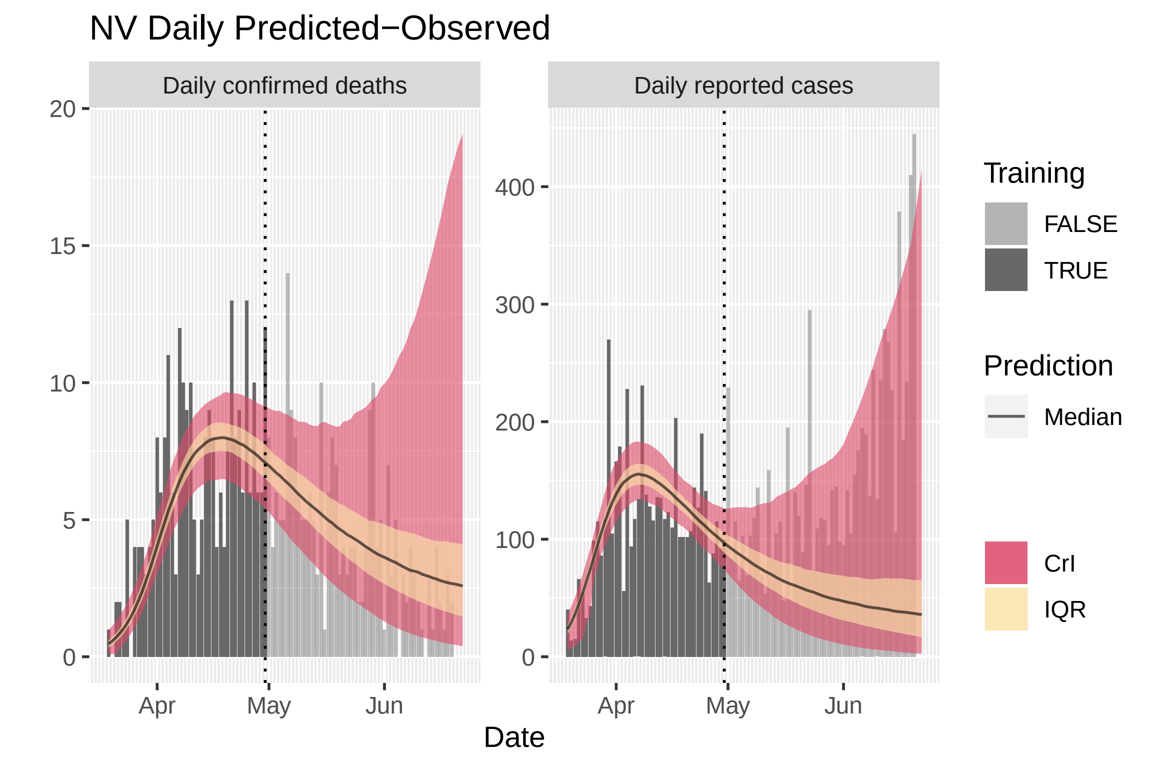

NV Daily Predicted−Observed
Daily confirmed deaths
Daily reported cases
20
15
10
5
0
400
300
200
100
0
Apr
May
Jun
Date
Training
FALSE
TRUE
Prediction
Median
CrI
IQR

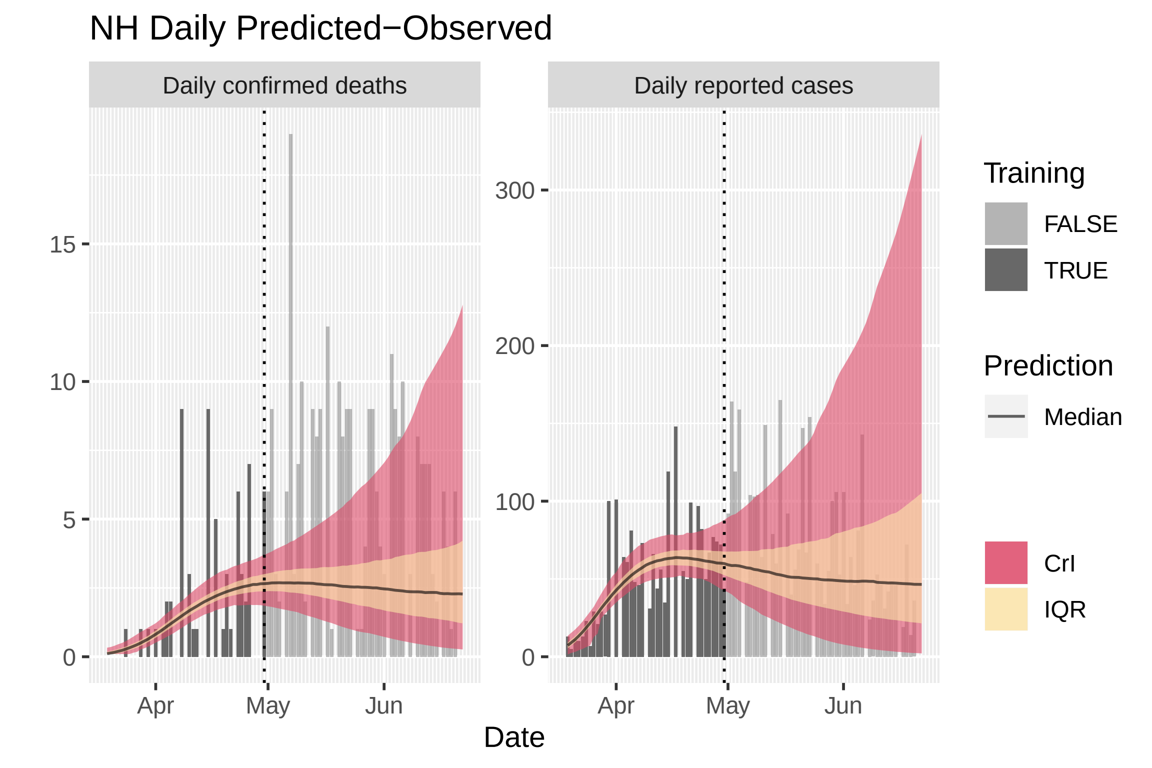

NH Daily Predicted−Observed
Daily confirmed deaths
Daily reported cases
0
5
10
15
0
100
200
300
Apr
May
Jun
Date
Training
FALSE
TRUE
Prediction
Median
CrI
IQR

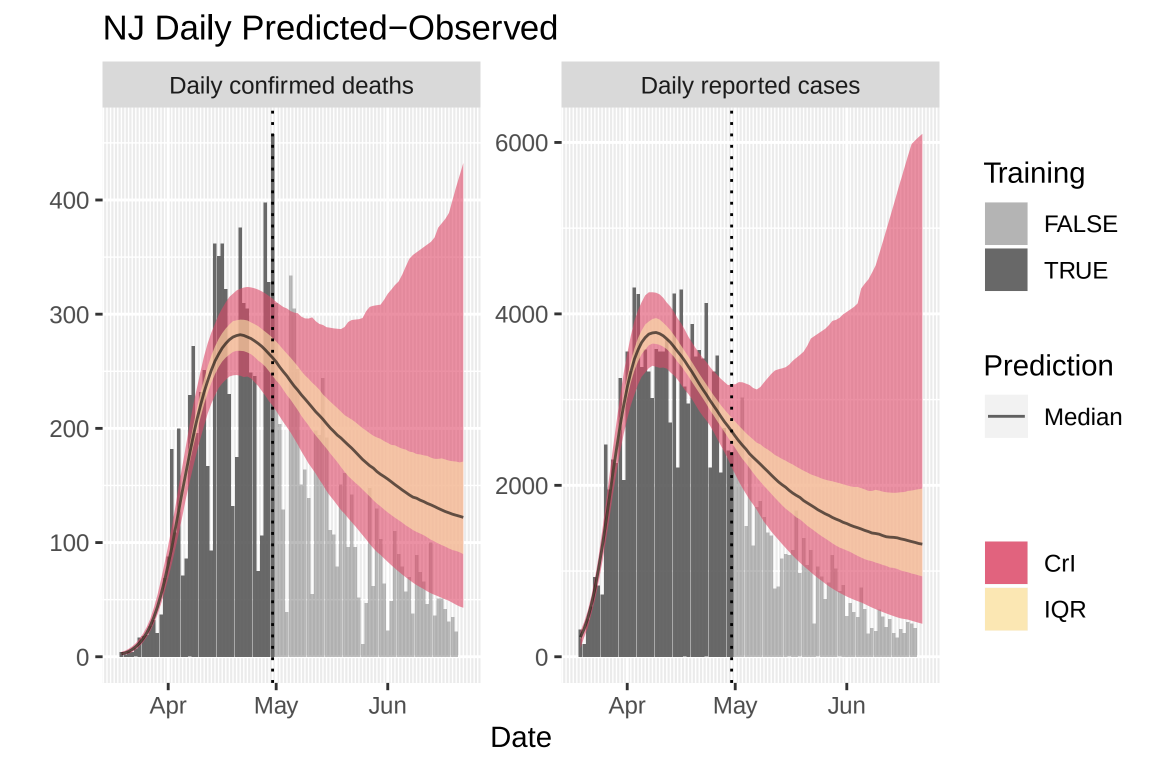
NJ Daily Predicted−Observed
Daily confirmed deaths
Daily reported cases
0
100
200
300
400
0
2000
4000
6000
Apr
May
Jun
Date
Training
FALSE
TRUE
Prediction
Median
CrI
IQR

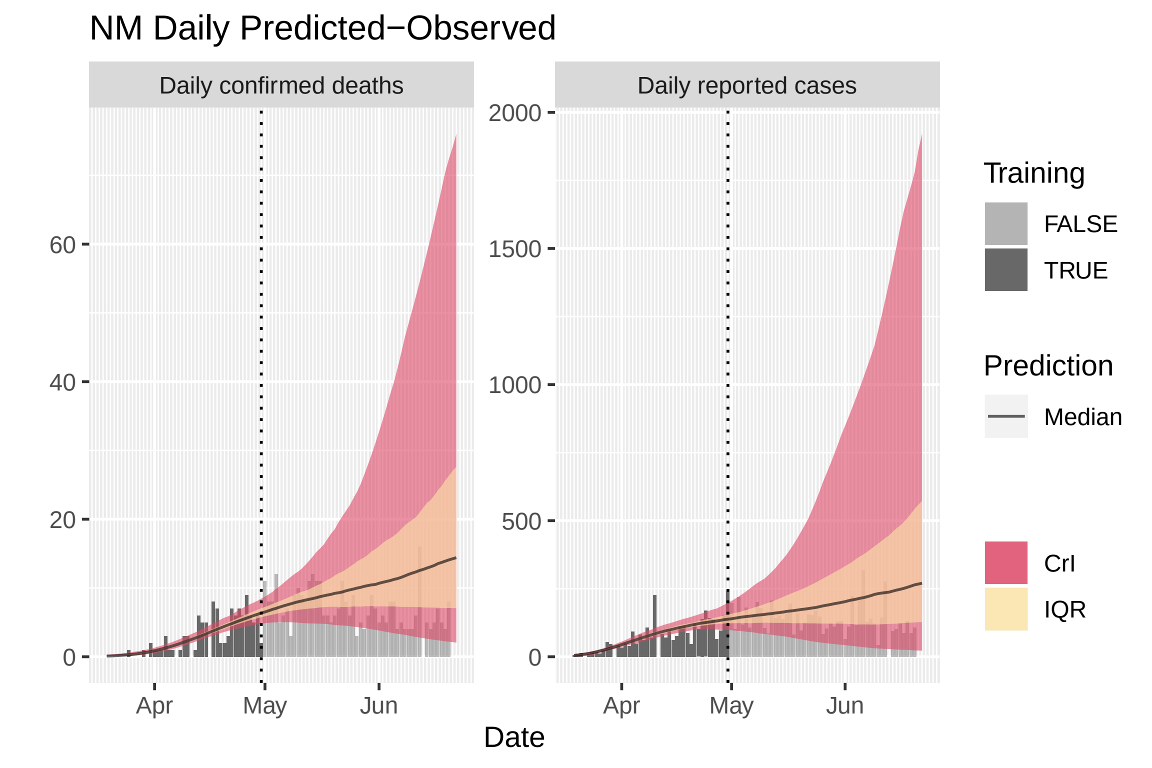
NM Daily Predicted−Observed
Daily confirmed deaths
Daily reported cases
0
20
40
60
0
500
1000
1500
2000
Apr
May
Jun
Date
Training
FALSE
TRUE
Prediction
Median
CrI
IQR

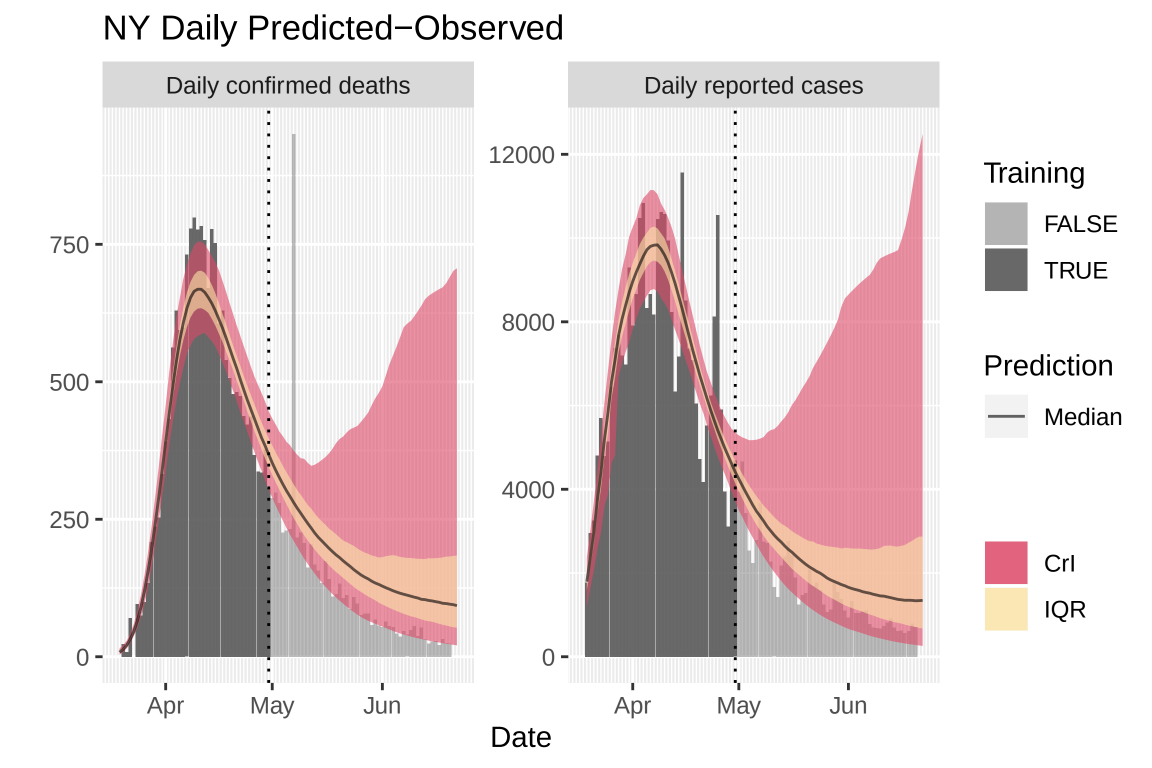

NY Daily Predicted−Observed
Daily confirmed deaths
Daily reported cases
750
500
250
0
12000
8000
4000
0
Apr
May
Jun
Date
Training
FALSE
TRUE
Prediction
Median
CrI
IQR

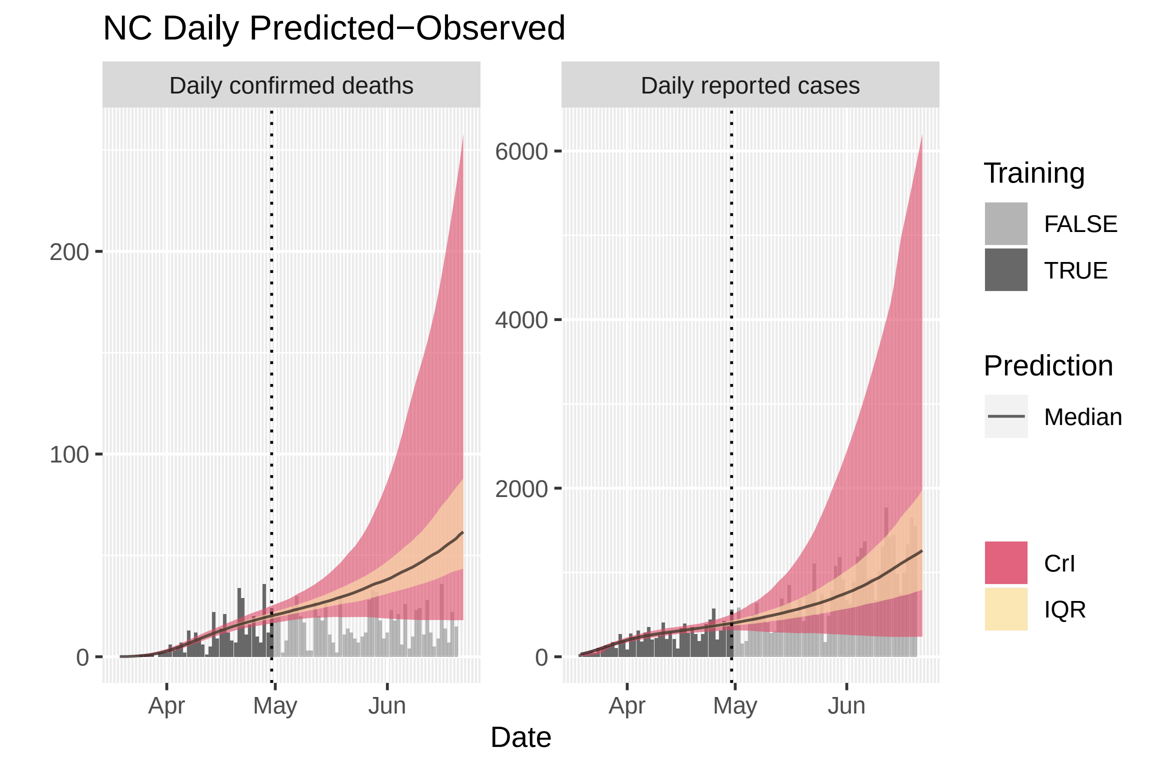
NC Daily Predicted−Observed
Daily confirmed deaths
Daily reported cases
0
100
200
2000
4000
6000
Apr
May
Jun
Date
Training
FALSE
TRUE
Prediction
Median
CrI
IQR

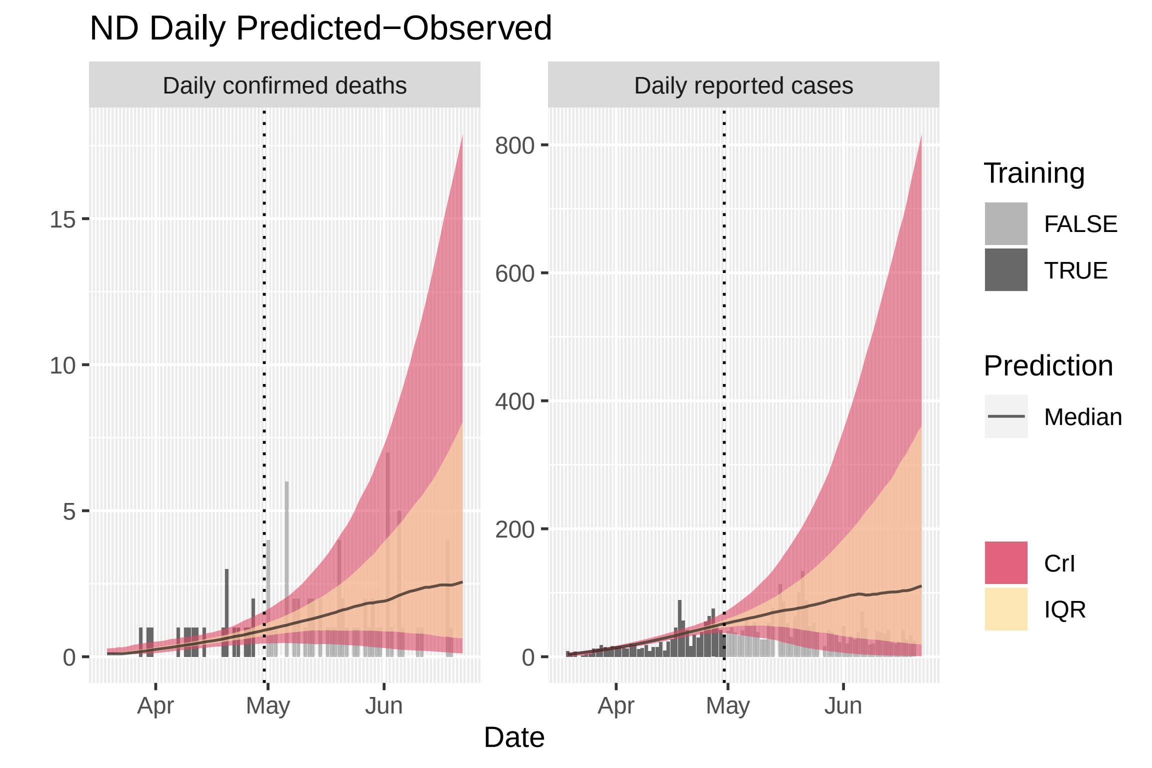

ND Daily Predicted−Observed
Daily confirmed deaths
Daily reported cases
15
10
5
0
800
600
400
200
0
Apr
May
Jun
Date
Training
FALSE
TRUE
Prediction
Median
CrI
IQR

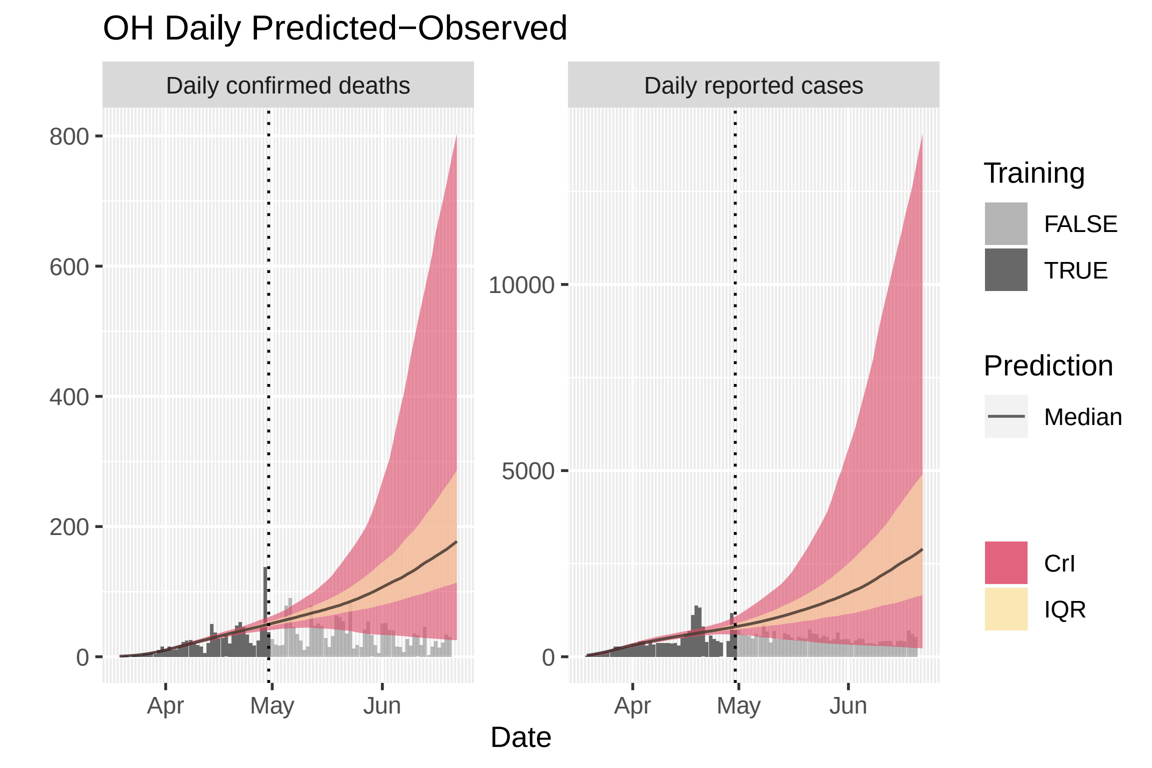

OH Daily Predicted−Observed
Daily confirmed deaths
Daily reported cases
800
600
400
200
0
10000
5000
0
Apr
May
Jun
Date
Training
FALSE
TRUE
Prediction
Median
CrI
IQR

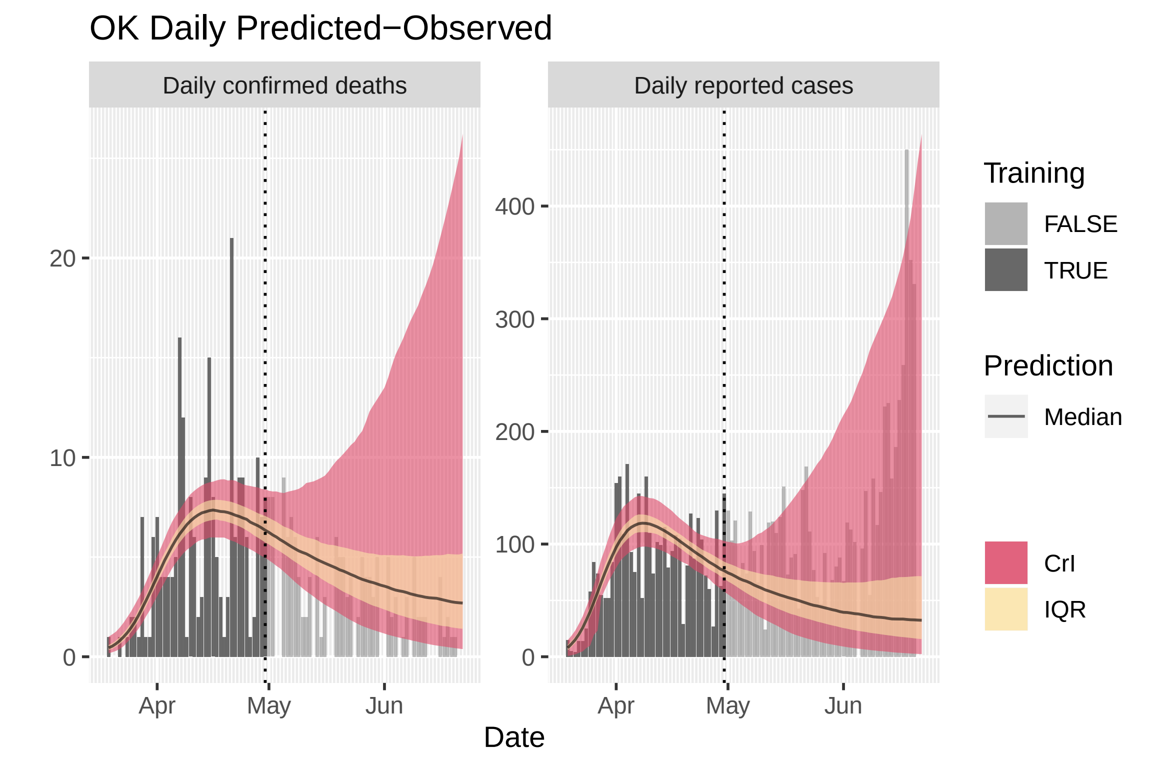

OK Daily Predicted−Observed
Daily confirmed deaths
Daily reported cases
0
10
20
0
100
200
300
400
Apr
May
Jun
Apr
May
Jun
Date
Training
FALSE
TRUE
Prediction
Median
CrI
IQR

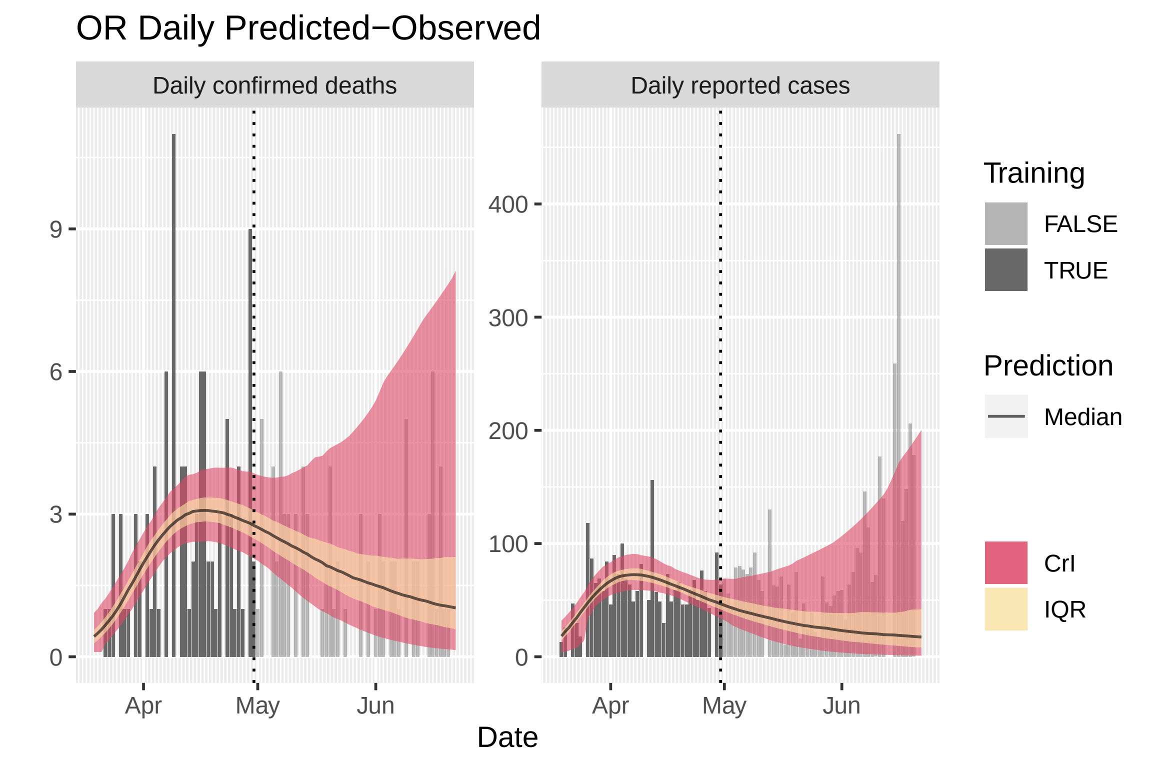

OR Daily Predicted−Observed
Daily confirmed deaths
Daily reported cases
0
3
6
9
0
100
200
300
400
Apr
May
Jun
Date
Training
FALSE
TRUE
Prediction
Median
CrI
IQR

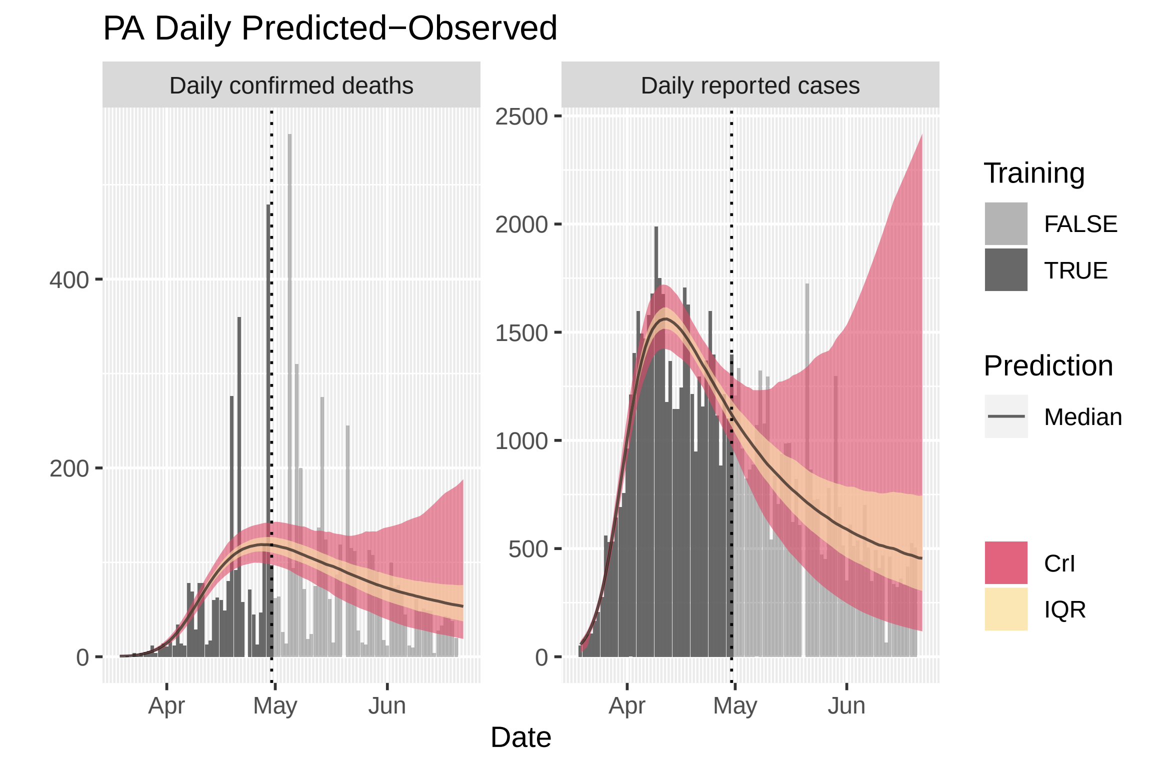
PA Daily Predicted−Observed
Daily confirmed deaths
Daily reported cases
0
200
400
500
1000
1500
2000
2500
Apr
May
Jun
Date
Training
FALSE
TRUE
Prediction
Median
CrI
IQR

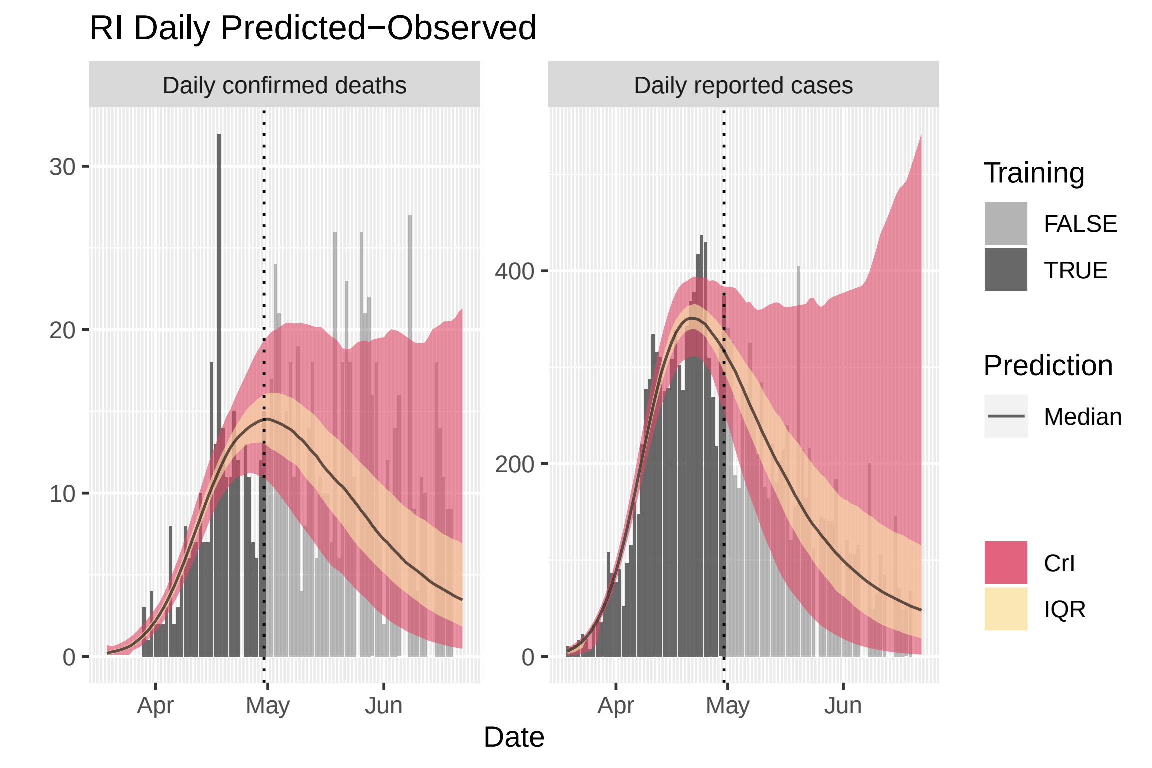
RI Daily Predicted−Observed
Daily confirmed deaths
Daily reported cases
0
10
20
30
0
200
400
Apr
May
Jun
Date
Training
FALSE
TRUE
Prediction
Median
CrI
IQR

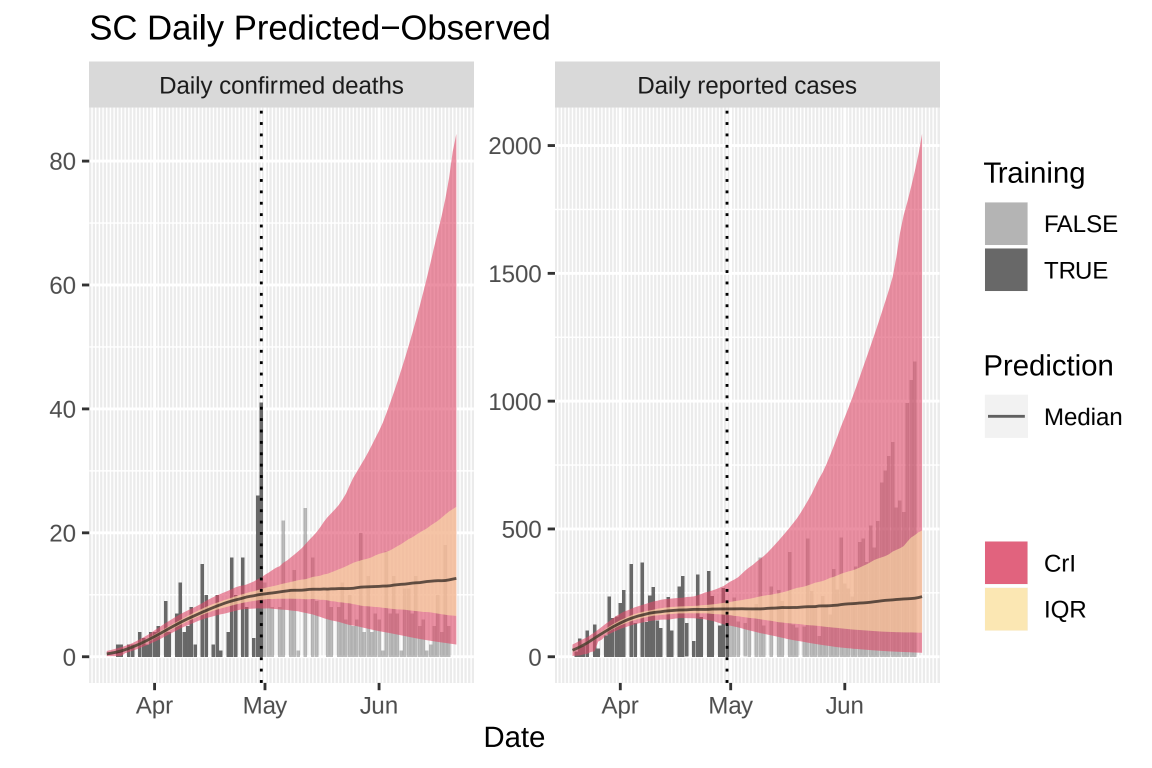

SC Daily Predicted−Observed
Daily confirmed deaths
Daily reported cases
80
60
40
20
0
2000
1500
1000
500
0
Apr
May
Jun
Apr
May
Jun
Date
Training
FALSE
TRUE
Prediction
Median
CrI
IQR

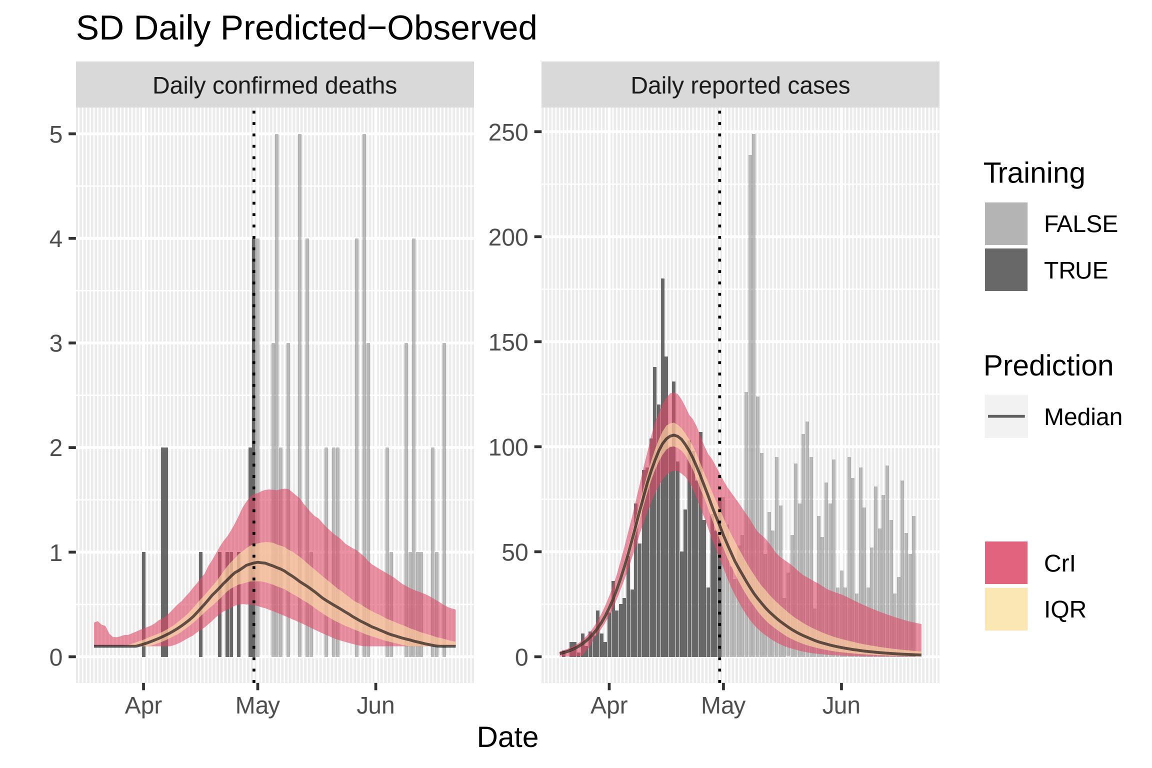

SD Daily Predicted−Observed
Daily confirmed deaths
Daily reported cases
5
4
3
2
1
0
250
200
150
100
50
0
Apr
May
Jun
Date
Training
FALSE
TRUE
Prediction
Median
CrI
IQR

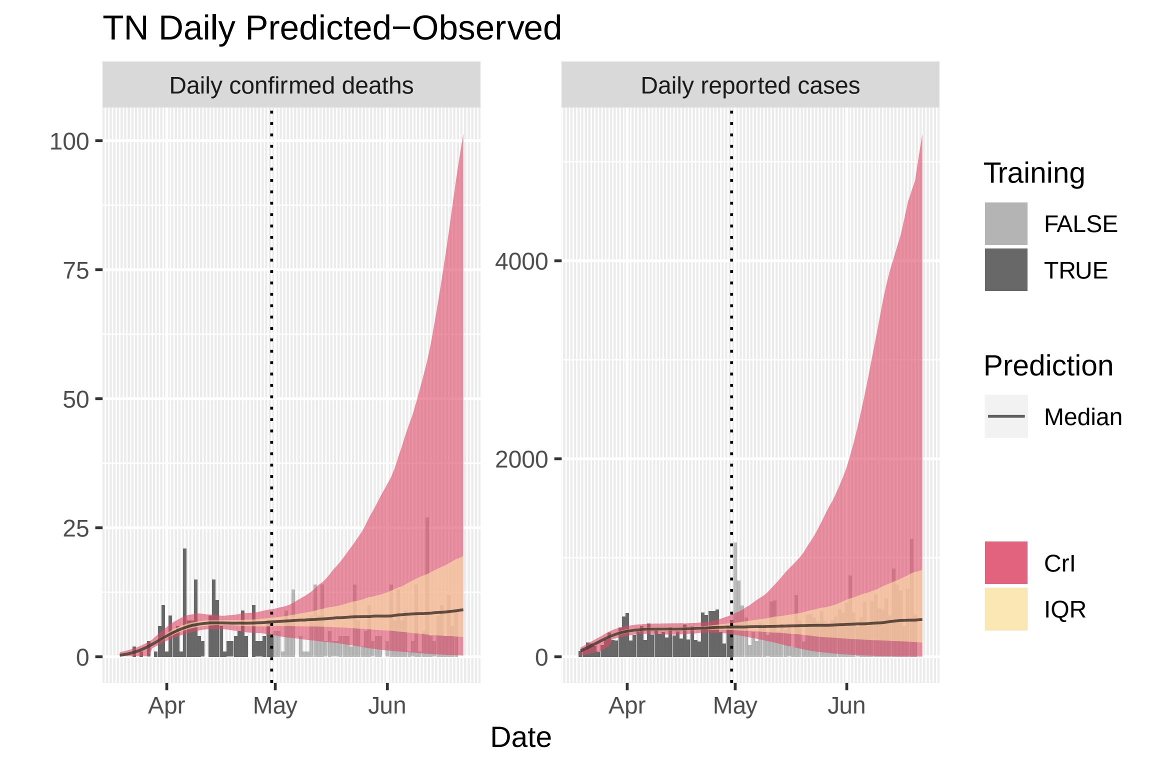

TN Daily Predicted−Observed
Daily confirmed deaths
Daily reported cases
100
75
50
25
0
4000
2000
0
Apr
May
Jun
Apr
May
Jun
Date
Training
FALSE
TRUE
Prediction
Median
CrI
IQR

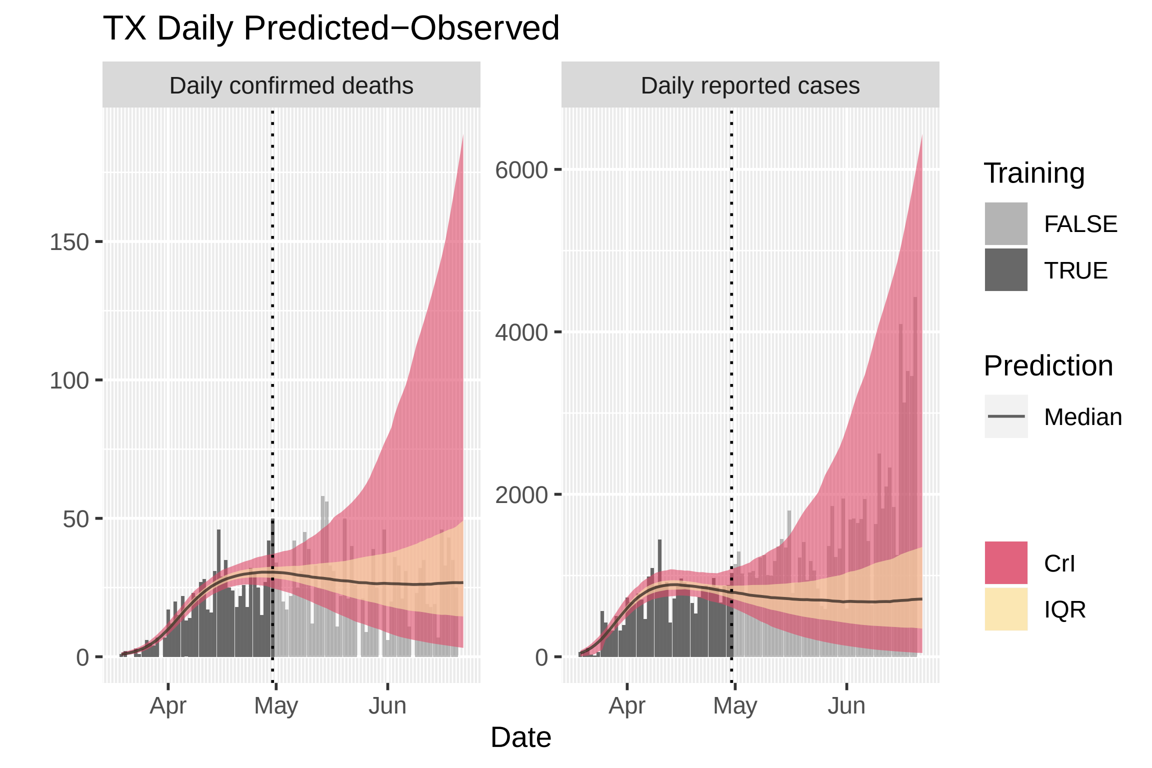

TX Daily Predicted−Observed
Daily confirmed deaths
Daily reported cases
150
100
50
0
6000
4000
2000
0
Apr
May
Jun
Apr
May
Jun
Date
Training
FALSE
TRUE
Prediction
Median
CrI
IQR

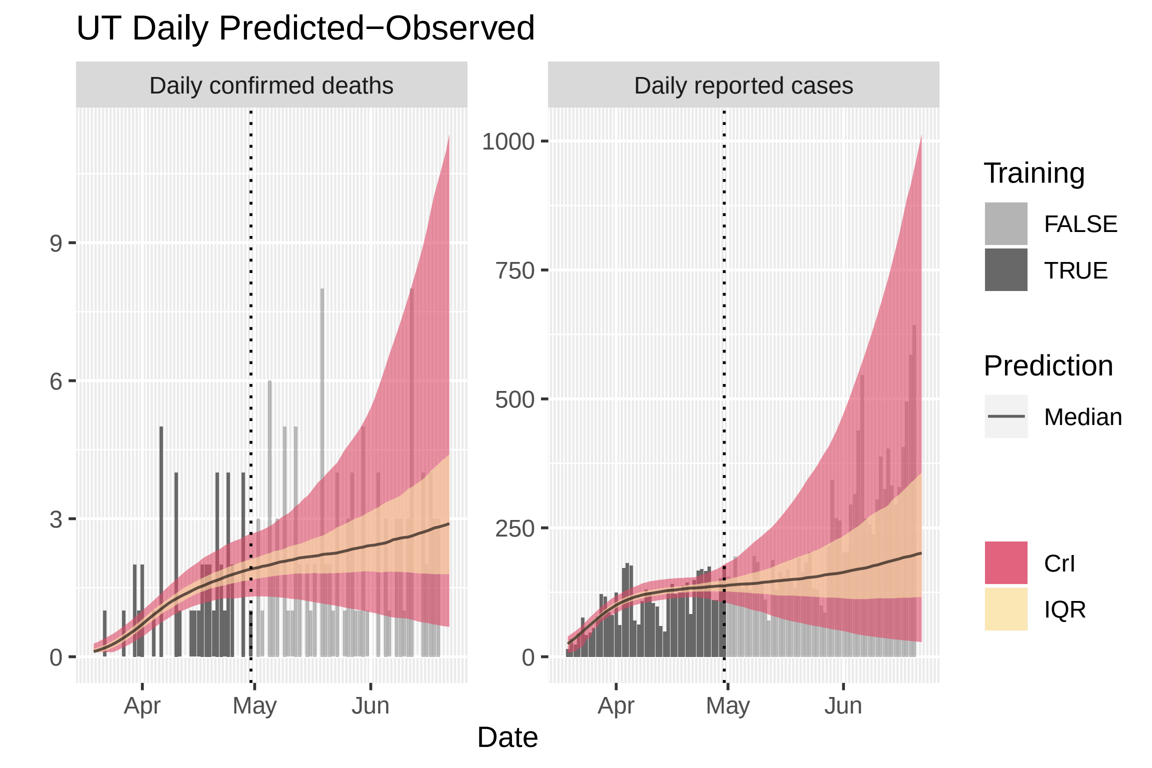

UT Daily Predicted−Observed
Daily confirmed deaths
Daily reported cases
0
3
6
9
0
250
500
750
1000
Apr
May
Jun
Apr
May
Jun
Date
Training
FALSE
TRUE
Prediction
Median
CrI
IQR

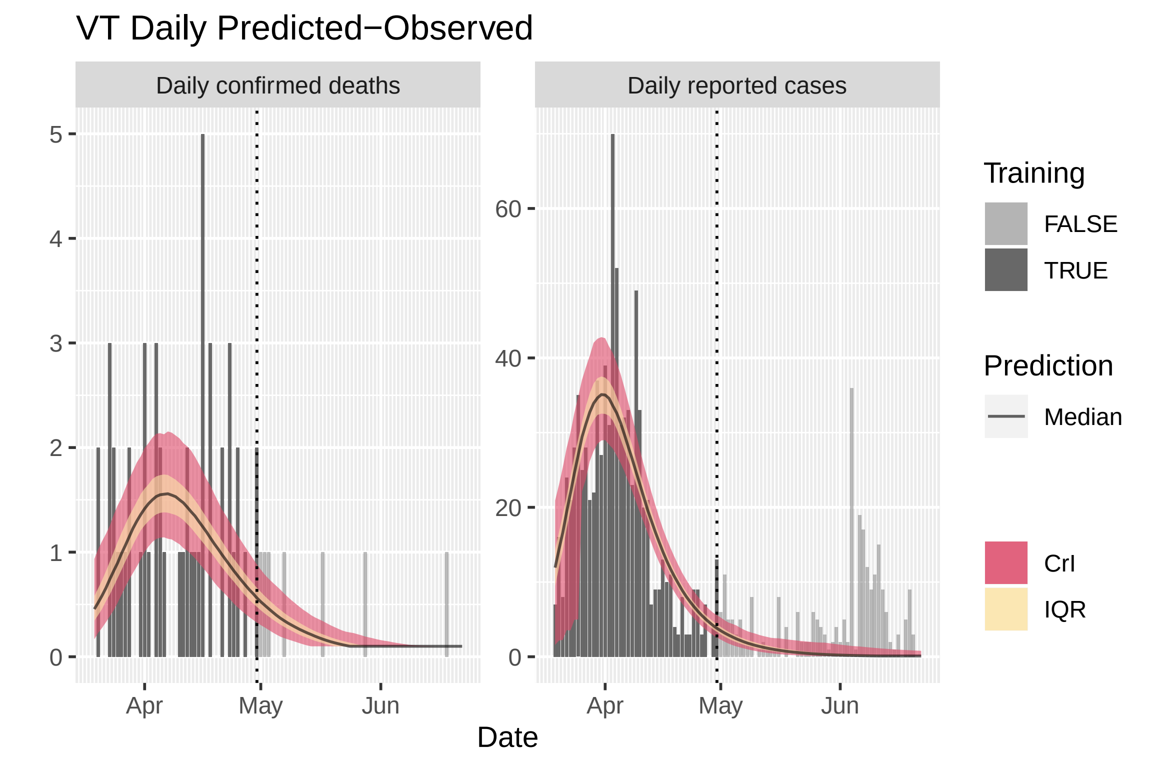

VT Daily Predicted−Observed
Daily confirmed deaths
Daily reported cases
0
1
2
3
4
5
0
20
40
60
Apr
May
Jun
Date
Training
FALSE
TRUE
Prediction
Median
CrI
IQR

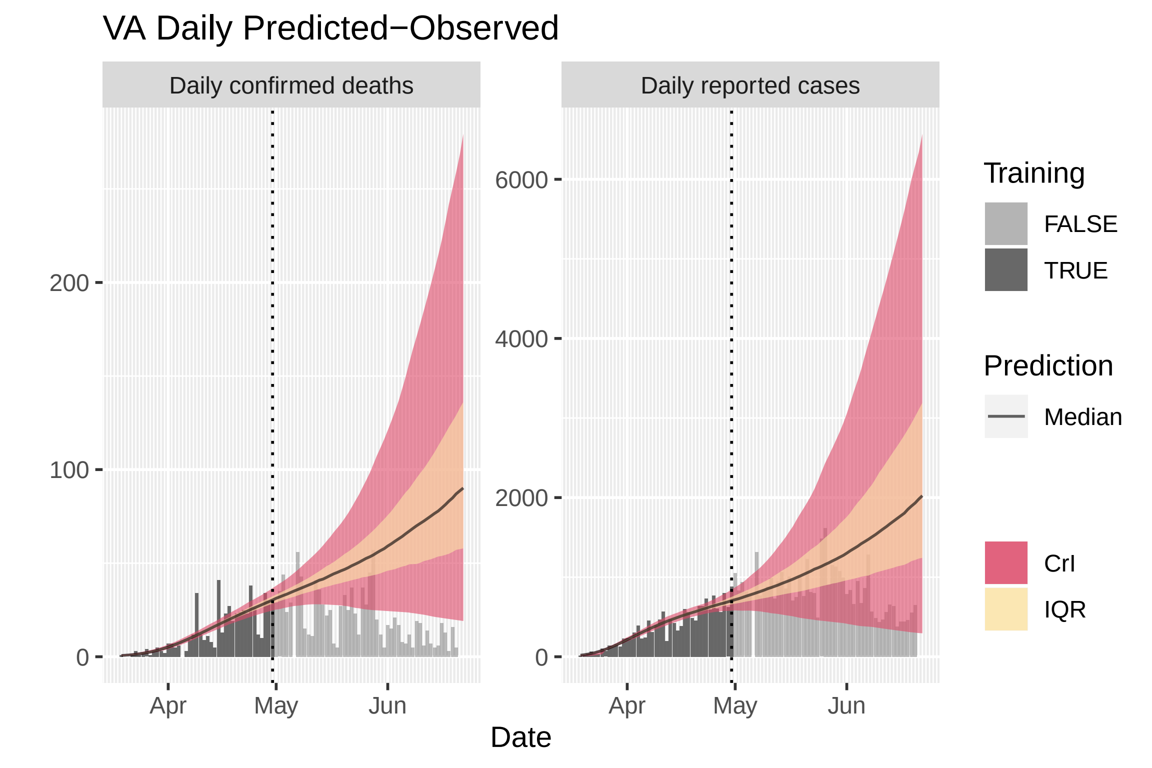

VA Daily Predicted−Observed
Daily confirmed deaths
Daily reported cases
0
100
200
2000
4000
6000
Apr
May
Jun
Date
Training
FALSE
TRUE
Prediction
Median
CrI
IQR

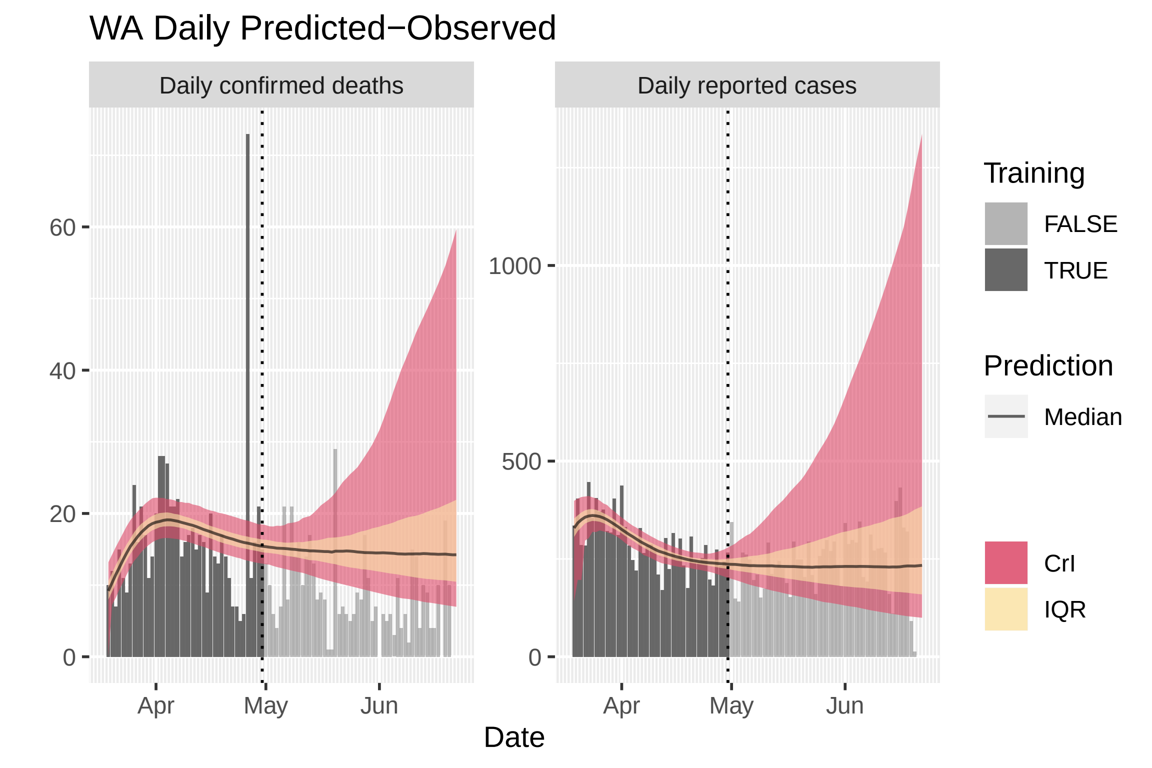

WA Daily Predicted−Observed
Daily confirmed deaths
Daily reported cases
60
40
20
0
1000
500
0
Apr
May
Jun
Date
Training
FALSE
TRUE
Prediction
Median
CrI
IQR

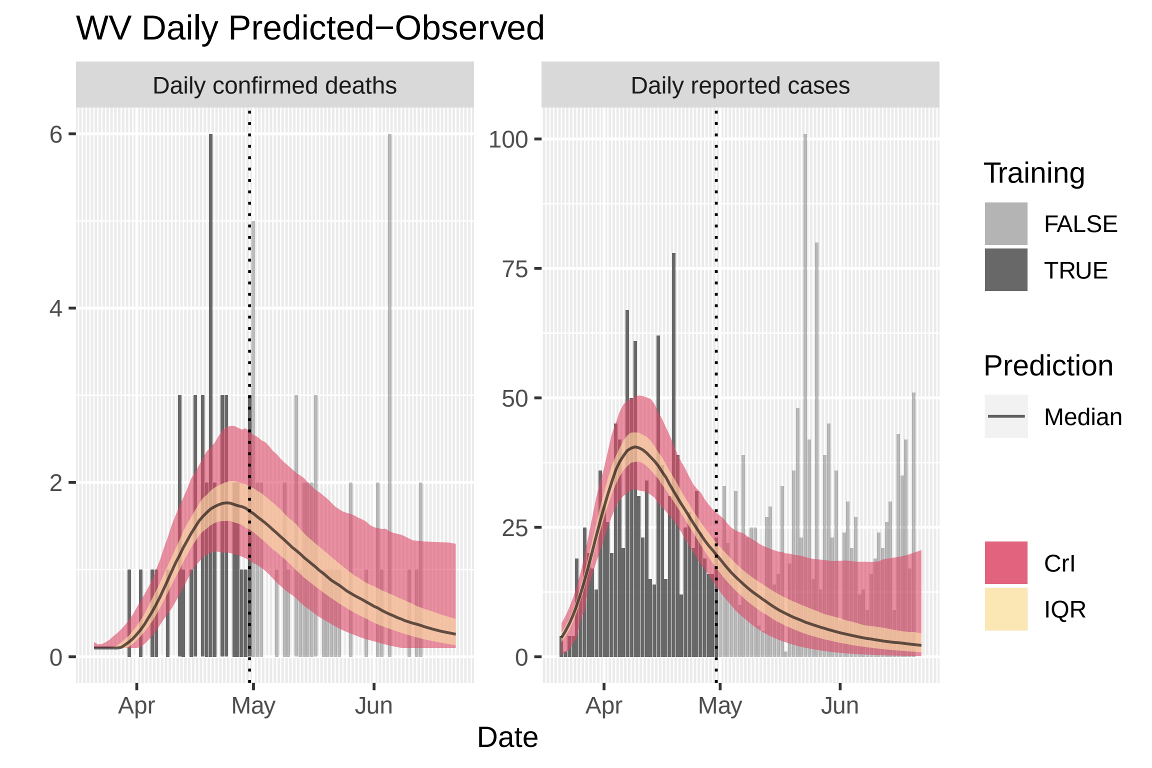
WV Daily Predicted−Observed
Daily confirmed deaths
Daily reported cases
0
2
4
6
0
25
50
75
100
Apr
May
Jun
Apr
May
Jun
Date
Training
FALSE
TRUE
Prediction
Median
CrI
IQR

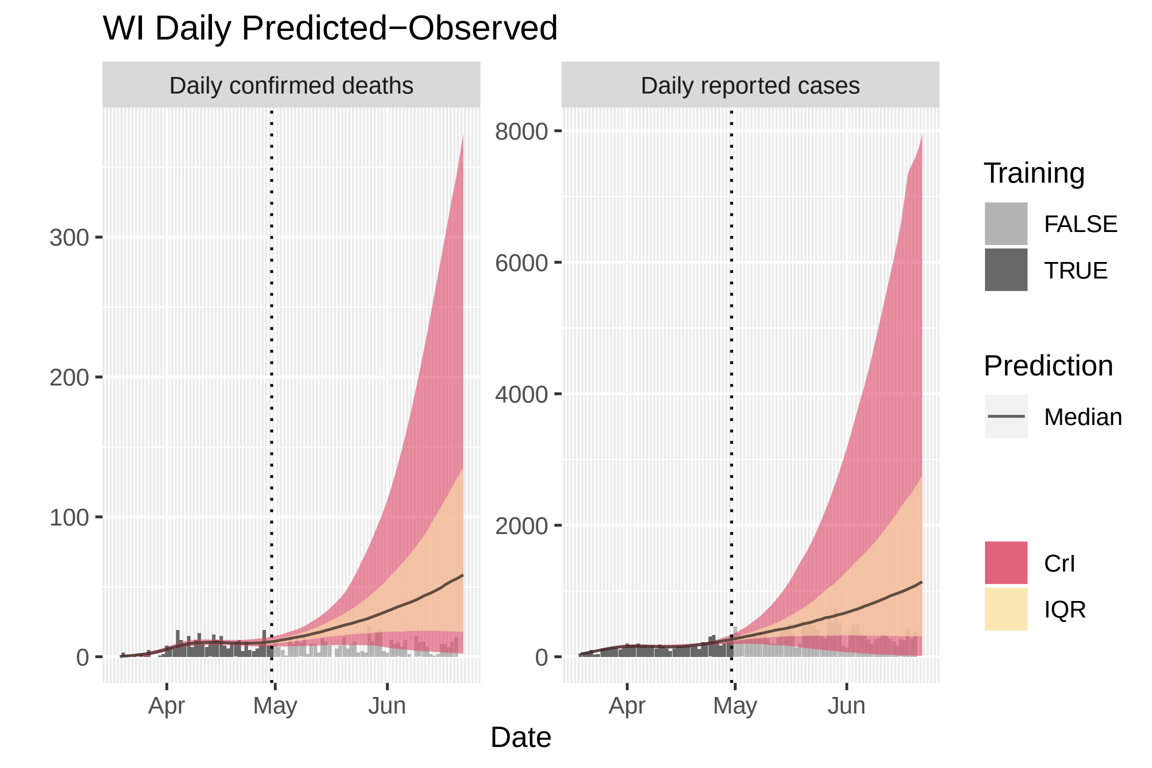

WI Daily Predicted−Observed
Daily confirmed deaths
Daily reported cases
300
200
100
0
8000
6000
4000
2000
0
Apr
May
Jun
Apr
May
Jun
Date
Training
FALSE
TRUE
Prediction
Median
CrI
IQR

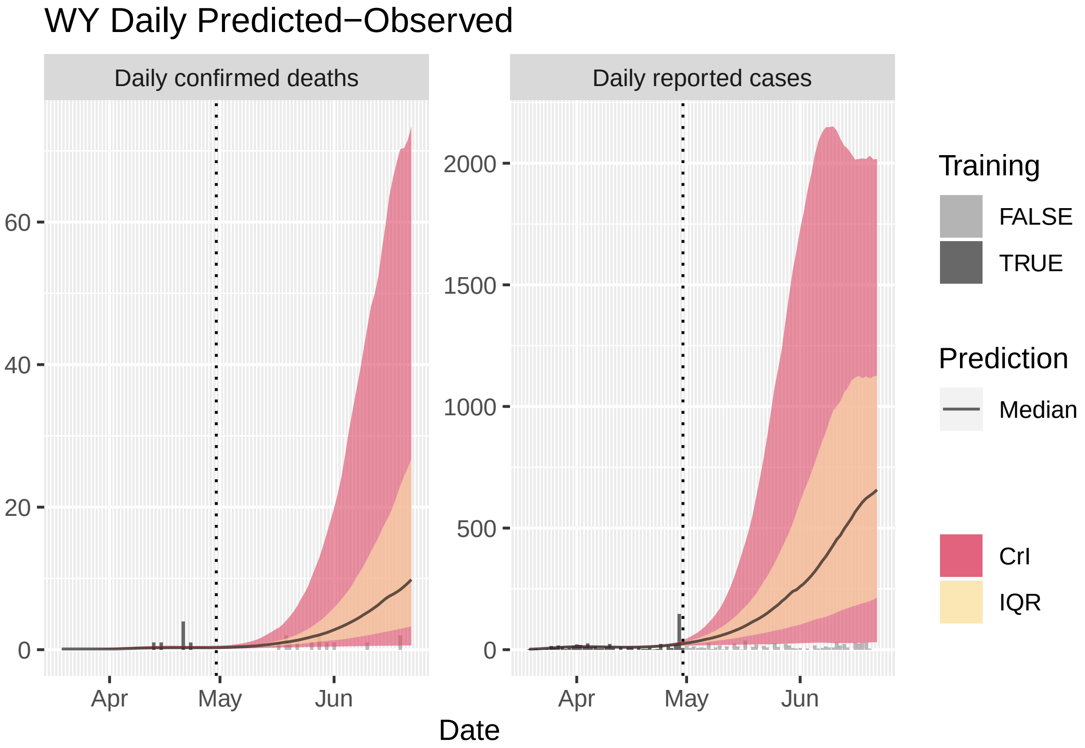

WY Daily Predicted−Observed
Daily confirmed deaths
Daily reported cases
60
40
20
0
2000
1500
1000
500
0
Apr
May
Jun
Date
Training
FALSE
TRUE
Prediction
Median
CrI
IQR
