# Supplementary material for: State-level impact of social distancing and testing on COVID-19 in the United States
Source: Res Sq. 2020 Jul 7:rs.3.rs-40364. Preprint. [Version 1] doi: 10.21203/rs.3.rs-40364/v1 (PMC7362894; doi:10.21203/rs.3.rs-40364/v1)
Supplement: Supplementary file 3 [file FigS4MobilityFitsbyState.pdf]

AL

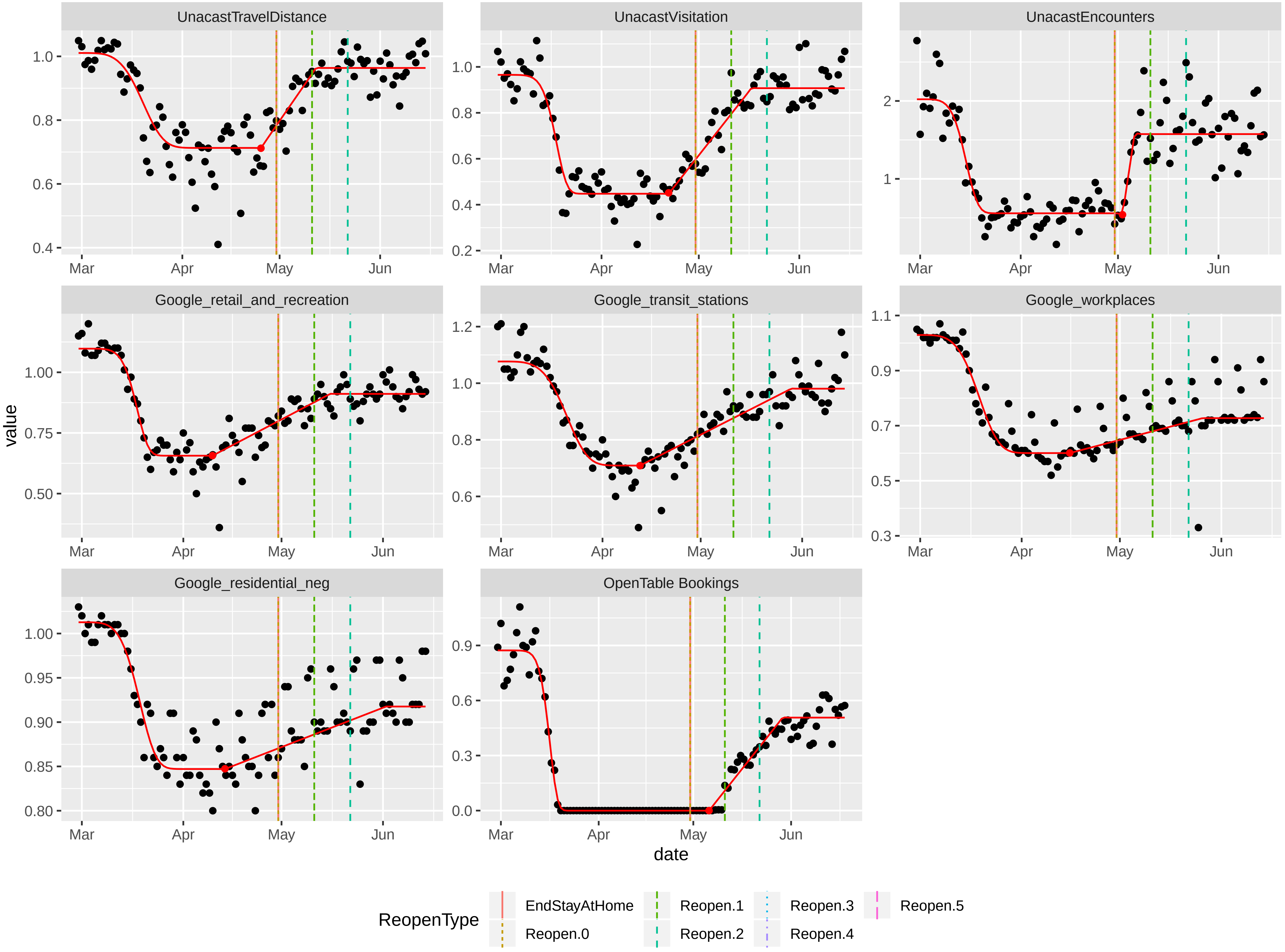
UnacastTravelDistance
UnacastVisitation
UnacastEncounters
Google_retail_and_recreation
Google_transit_stations
Google_workplaces
Google_residential_neg
OpenTable Bookings
value
date
Mar
Apr
May
Jun
ReopenType
EndStayAtHome
Reopen.0
Reopen.1
Reopen.2
Reopen.3
Reopen.4
Reopen.5

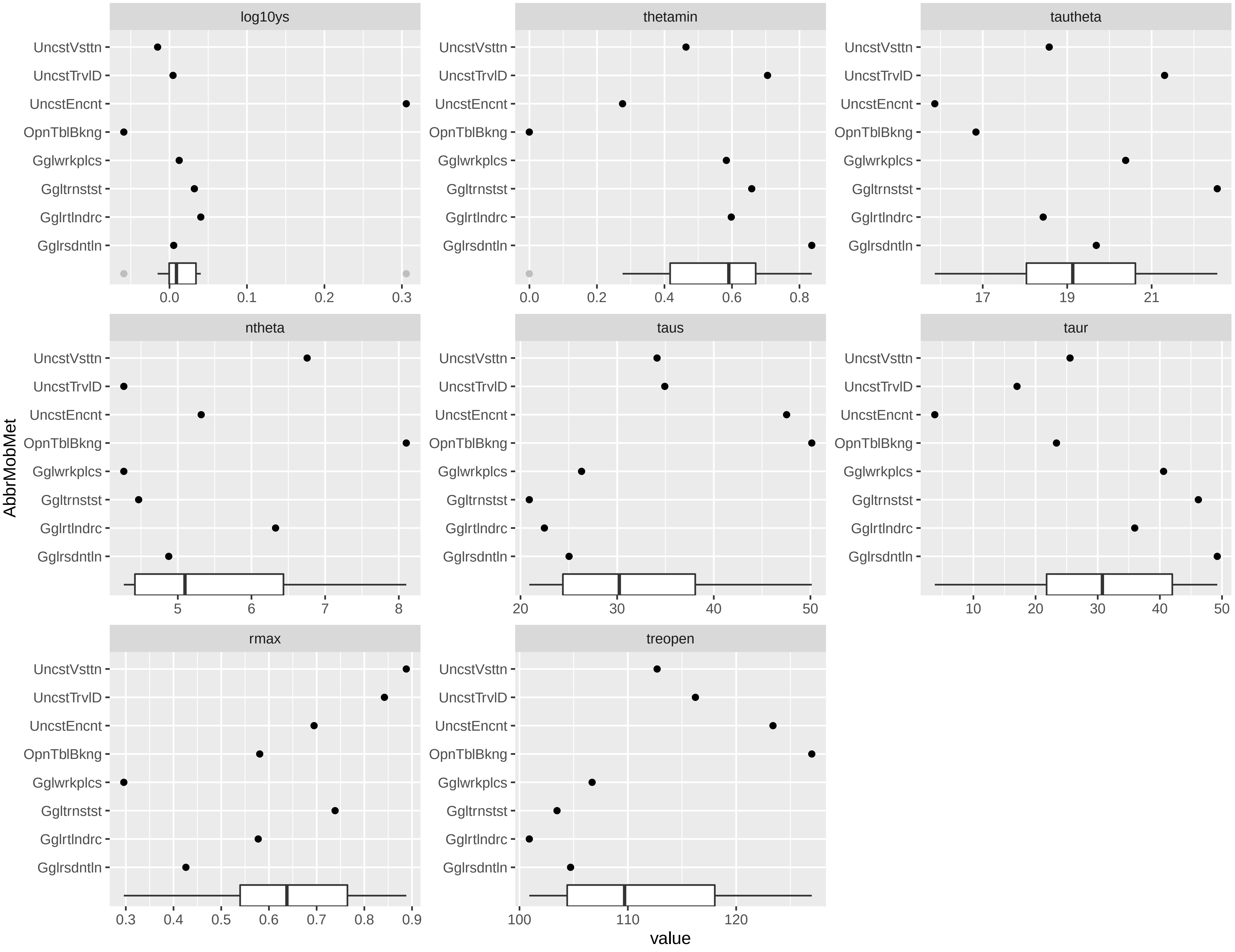

log10ys
thetamin
tautheta
ntheta
taus
taur
rmax
treopen
UncstVsttn
UncstTrvlD
UncstEncnt
OpnTblBkng
Gglwrkplcs
Ggltrnstst
Gglrtlndrc
Gglrsdntln
AbbrMobMet
value

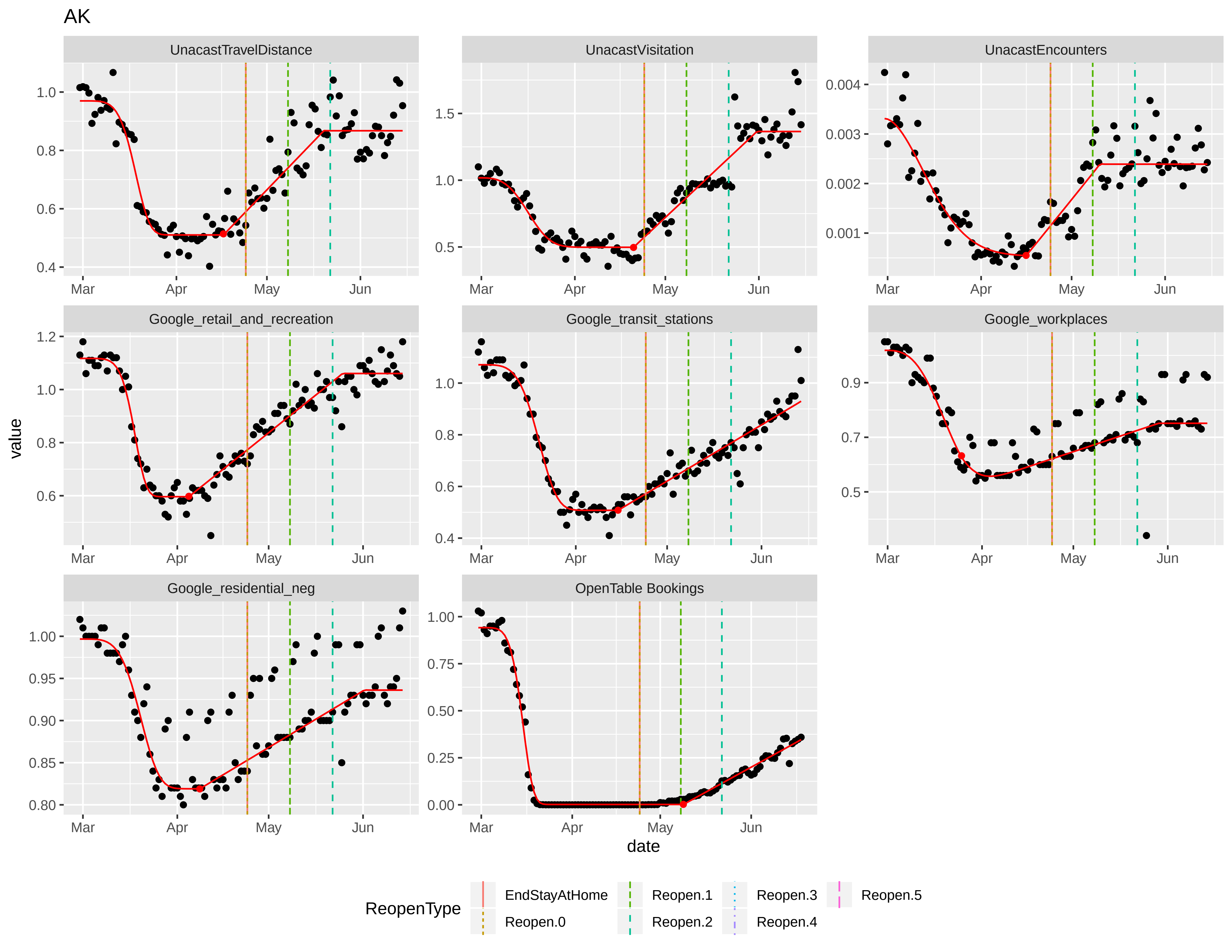
AK
UnacastTravelDistance
UnacastVisitation
UnacastEncounters
Google_retail_and_recreation
Google_transit_stations
Google_workplaces
Google_residential_neg
OpenTable Bookings
value
date
Mar
Apr
May
Jun
ReopenType
EndStayAtHome
Reopen.0
Reopen.1
Reopen.2
Reopen.3
Reopen.4
Reopen.5

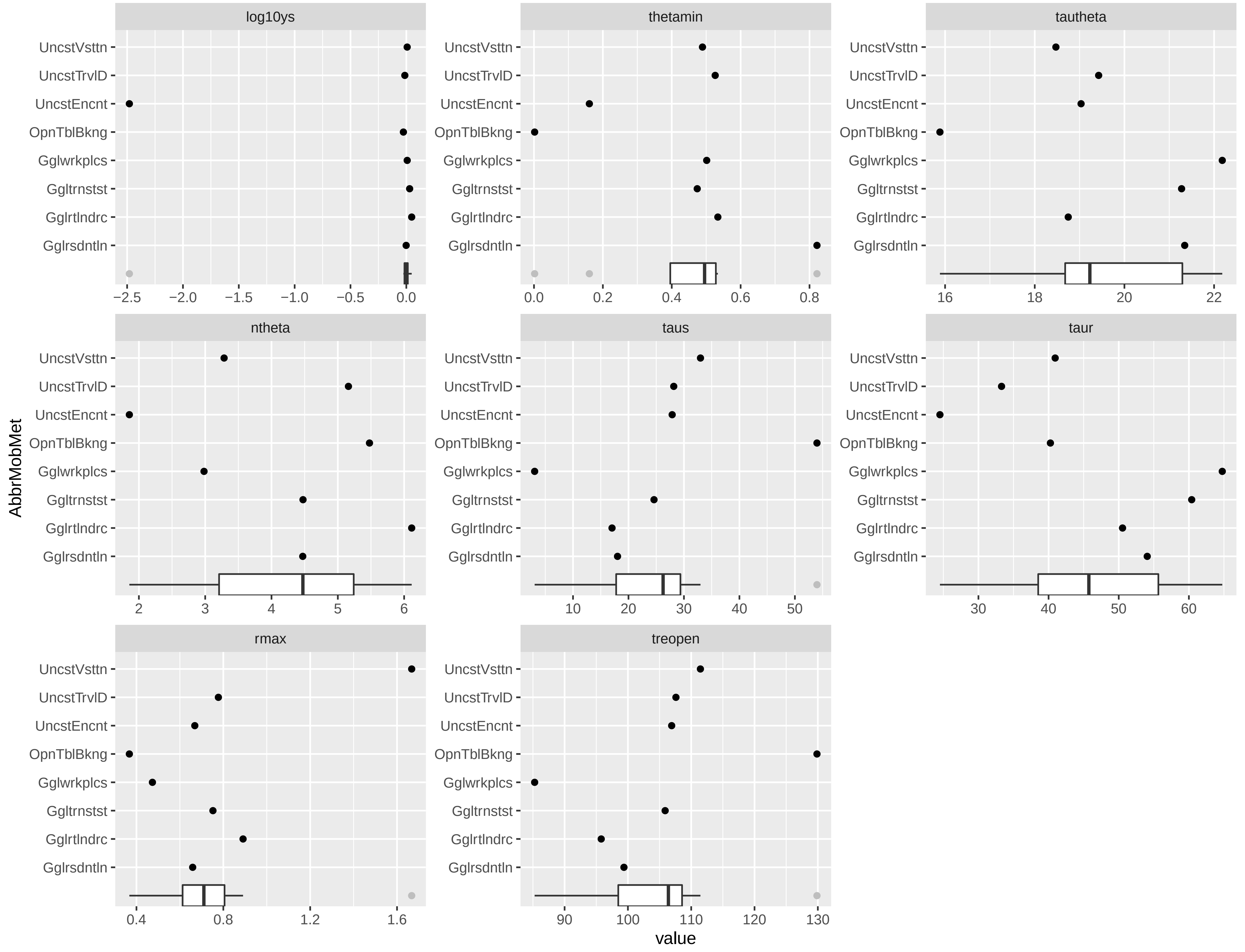

log10ys
thetamin
tautheta
ntheta
taus
taur
rmax
treopen
UncstVsttn
UncstTrvlD
UncstEncnt
OpnTblBkng
Gglwrkplcs
Ggltrnstst
Gglrtlndrc
Gglrsdntln
AbbrMobMet
value

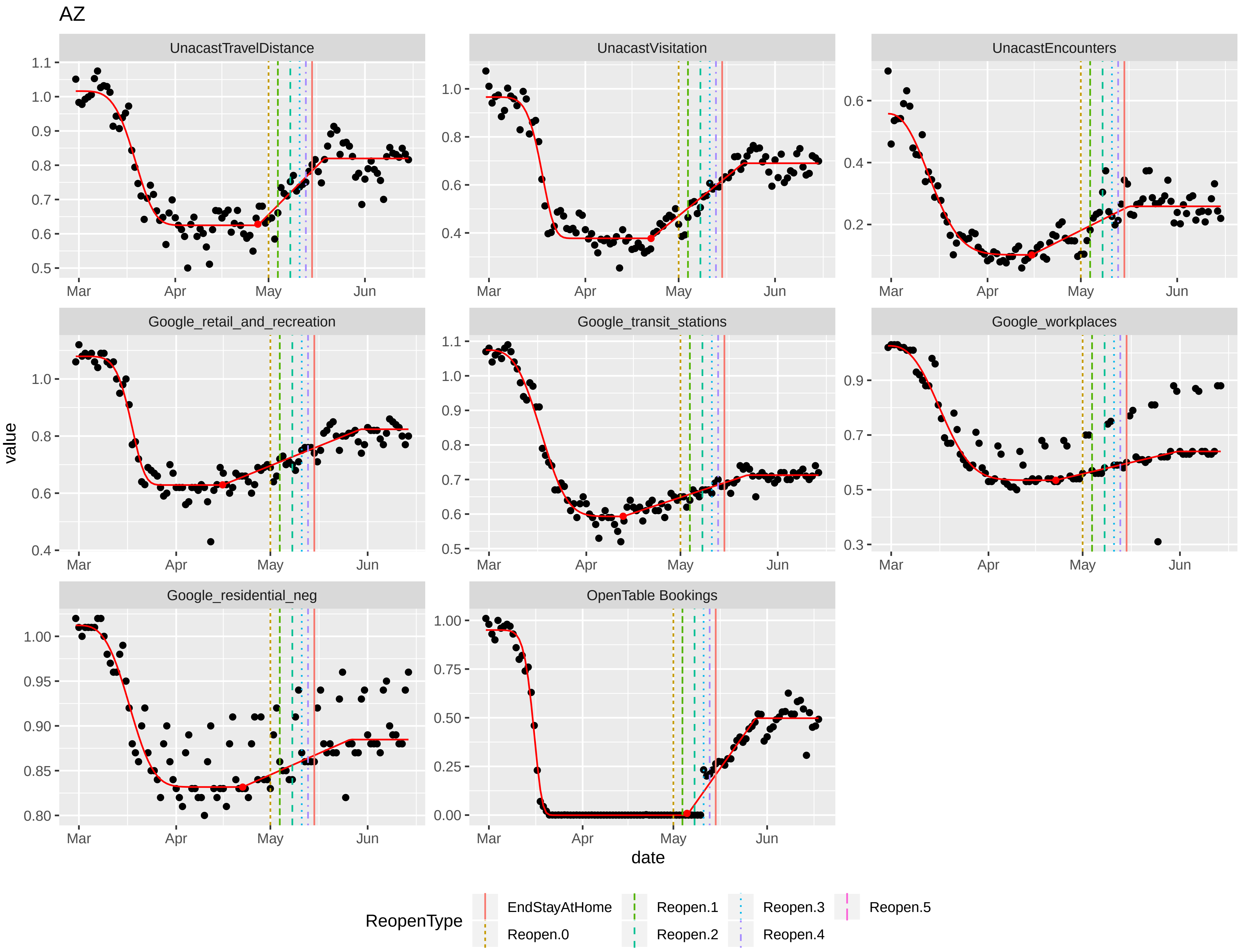

AZ
UnacastTravelDistance
UnacastVisitation
UnacastEncounters
Google_retail_and_recreation
Google_transit_stations
Google_workplaces
Google_residential_neg
OpenTable Bookings
value
date
Mar
Apr
May
Jun
ReopenType
EndStayAtHome
Reopen.0
Reopen.1
Reopen.2
Reopen.3
Reopen.4
Reopen.5

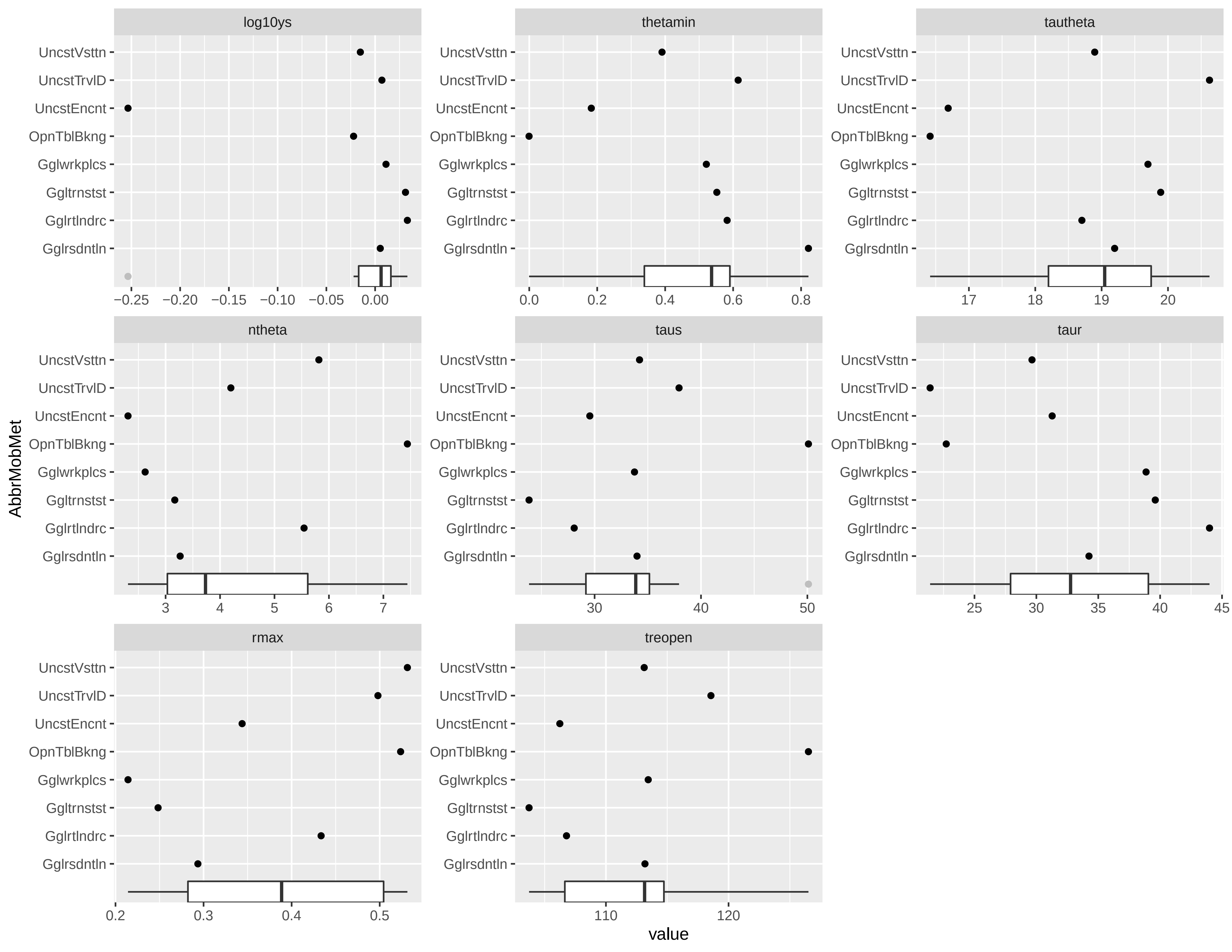

log10ys
thetamin
tautheta
ntheta
taus
taur
rmax
treopen
UncstVsttn
UncstTrvlD
UncstEncnt
OpnTblBkng
Gglwrkplcs
Ggltrnstst
Gglrtlndrc
Gglrsdntln
AbbrMobMet
value

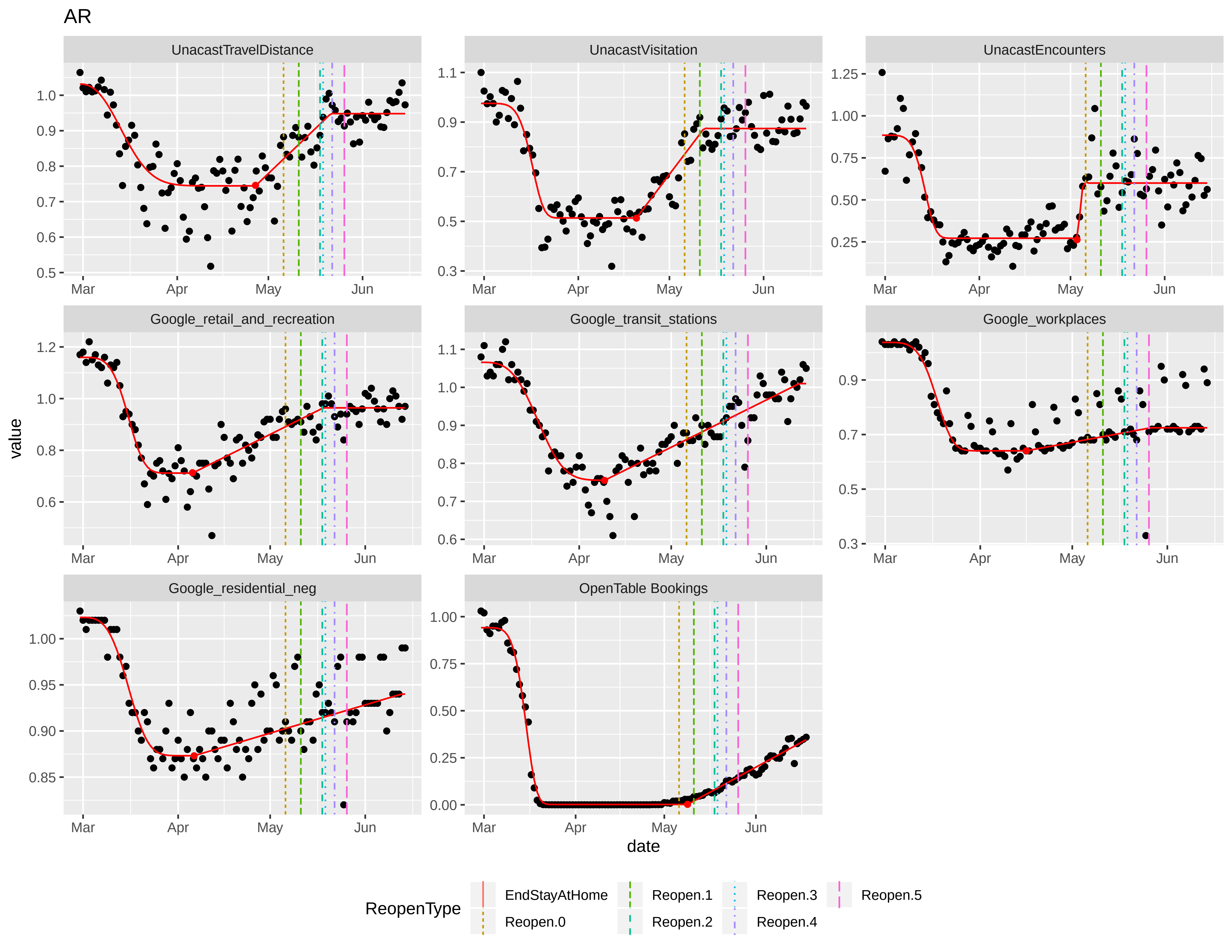

AR
UnacastTravelDistance
UnacastVisitation
UnacastEncounters
Google_retail_and_recreation
Google_transit_stations
Google_workplaces
Google_residential_neg
OpenTable Bookings
value
date
Mar
Apr
May
Jun
ReopenType
EndStayAtHome
Reopen.0
Reopen.1
Reopen.2
Reopen.3
Reopen.4
Reopen.5

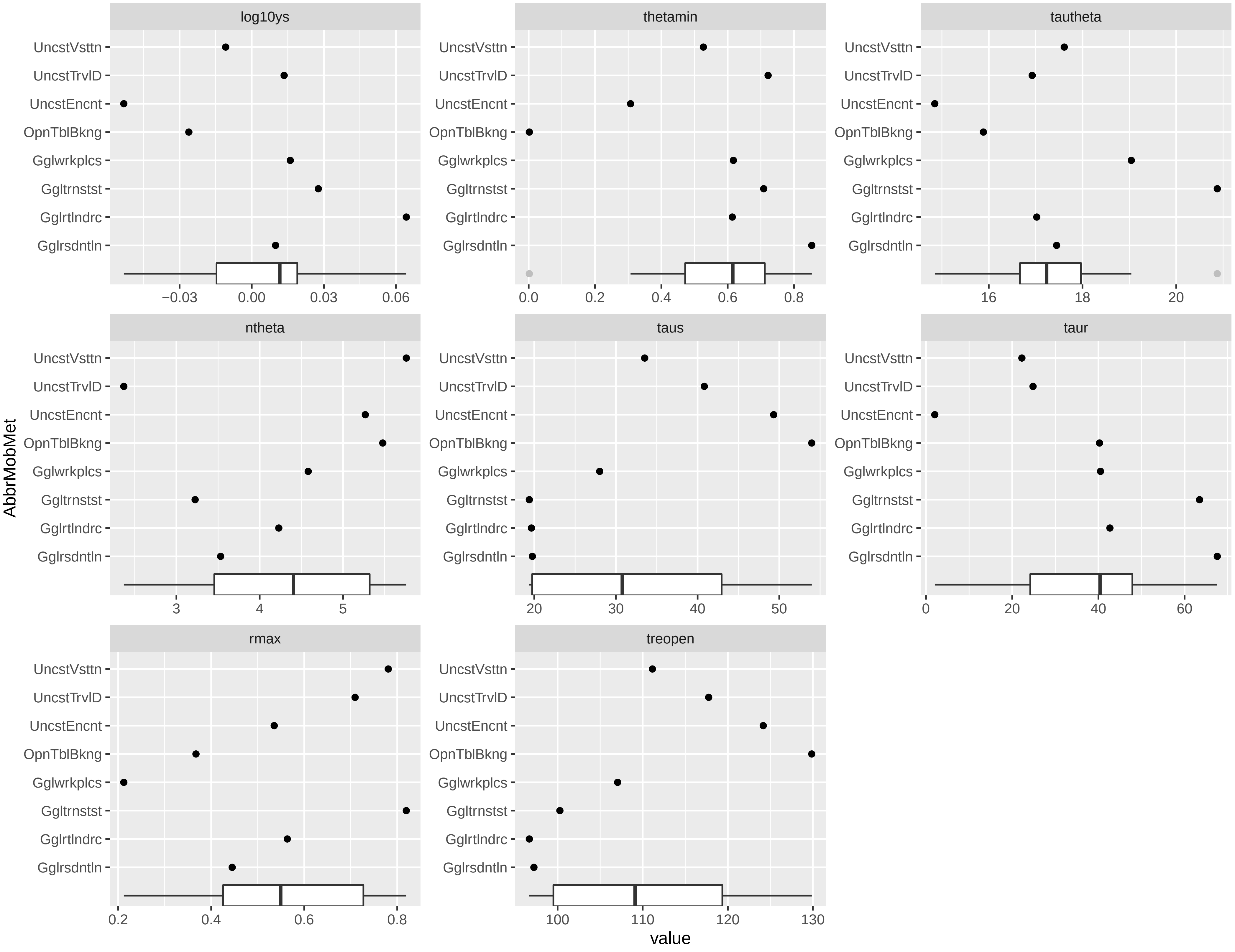

log10ys
thetamin
tautheta
ntheta
taus
taur
rmax
treopen
UncstVsttn
UncstTrvlD
UncstEncnt
OpnTblBkng
Gglwrkplcs
Ggltrnstst
Gglrtlndrc
Gglrsdntln
AbbrMobMet
value

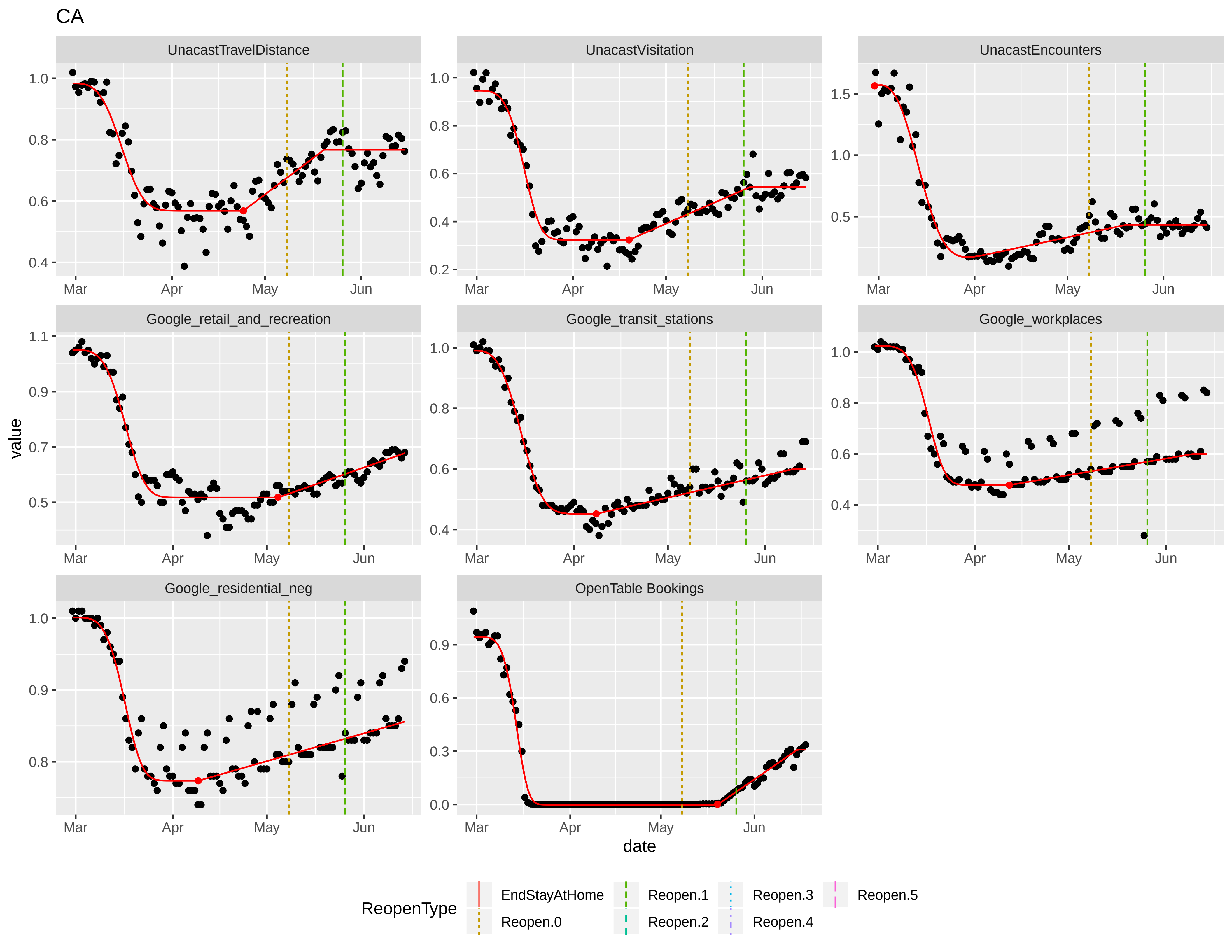
CA
UnacastTravelDistance
UnacastVisitation
UnacastEncounters
Google_retail_and_recreation
Google_transit_stations
Google_workplaces
Google_residential_neg
OpenTable Bookings
value
date
Mar
Apr
May
Jun
ReopenType
EndStayAtHome
Reopen.0
Reopen.1
Reopen.2
Reopen.3
Reopen.4
Reopen.5

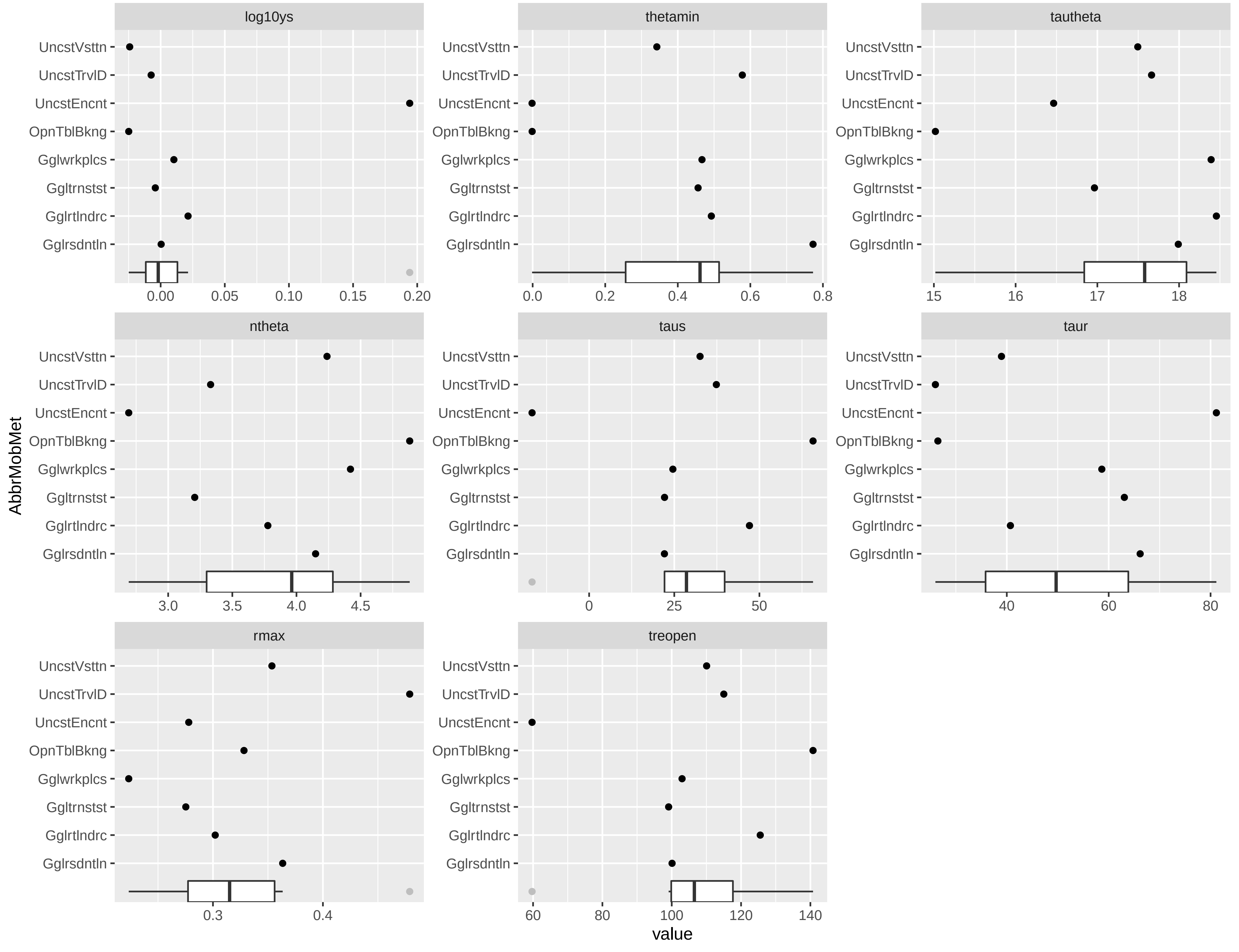

log10ys
thetamin
tautheta
ntheta
taus
taur
rmax
treopen
UncstVsttn
UncstTrvlD
UncstEncnt
OpnTblBkng
Gglwrkplcs
Ggltrnstst
Gglrtlndrc
Gglrsdntln
AbbrMobMet
value

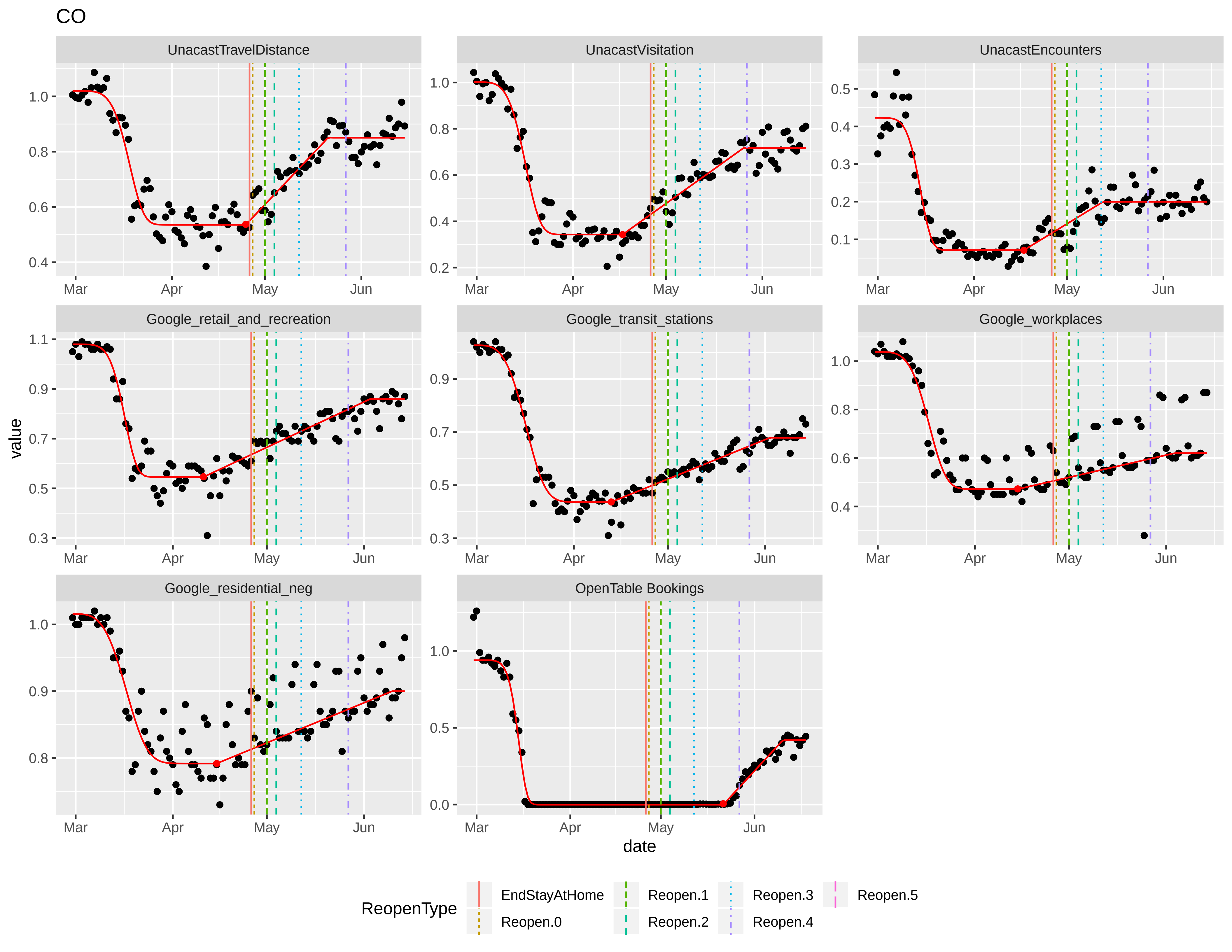

CO
UnacastTravelDistance
UnacastVisitation
UnacastEncounters
Google_retail_and_recreation
Google_transit_stations
Google_workplaces
Google_residential_neg
OpenTable Bookings
value
date
Mar
Apr
May
Jun
ReopenType
EndStayAtHome
Reopen.0
Reopen.1
Reopen.2
Reopen.3
Reopen.4
Reopen.5

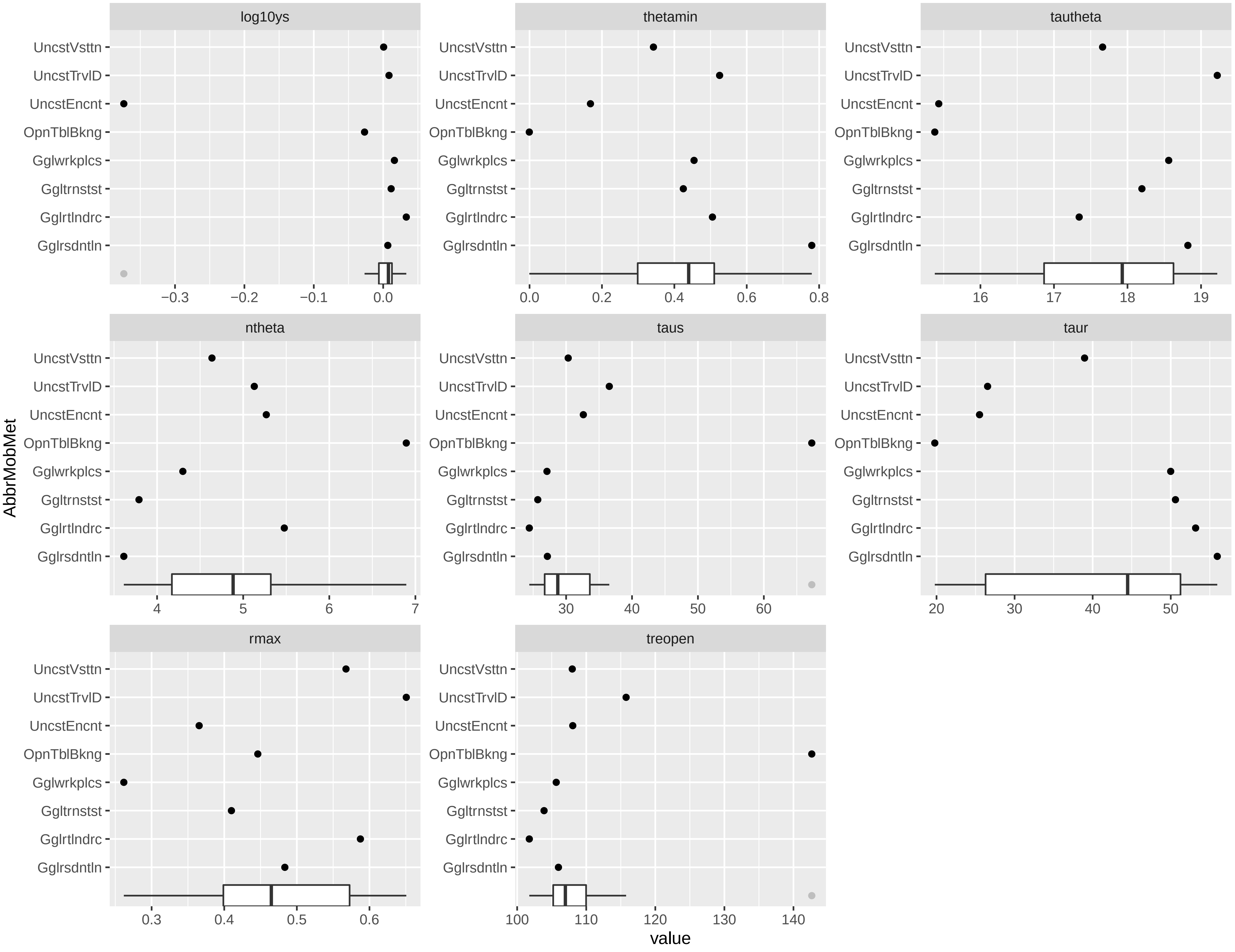
log10ys
thetamin
tautheta
ntheta
taus
taur
rmax
treopen
UncstVsttn
UncstTrvlD
UncstEncnt
OpnTblBkng
Gglwrkplcs
Ggltrnstst
Gglrtlndrc
Gglrsdntln
−0.3
−0.2
−0.1
0.0
0.2
0.4
0.6
0.8
16
17
18
19
4
5
6
7
30
40
50
60
20
0.3
0.5
100
110
120
130
140
AbbrMobMet
value

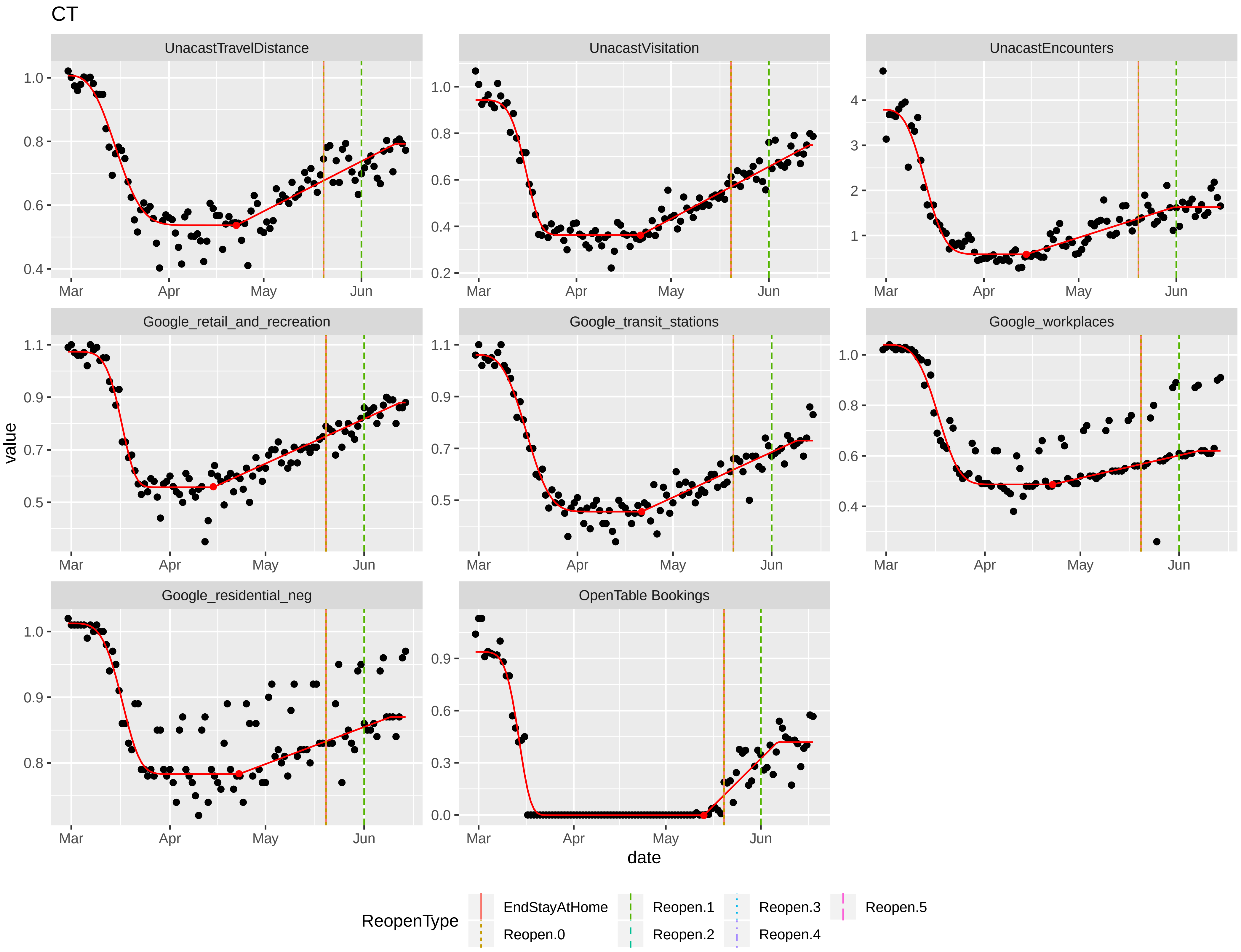

CT
UnacastTravelDistance
UnacastVisitation
UnacastEncounters
Google_retail_and_recreation
Google_transit_stations
Google_workplaces
Google_residential_neg
OpenTable Bookings
value
date
Mar
Apr
May
Jun
ReopenType
EndStayAtHome
Reopen.0
Reopen.1
Reopen.2
Reopen.3
Reopen.4
Reopen.5

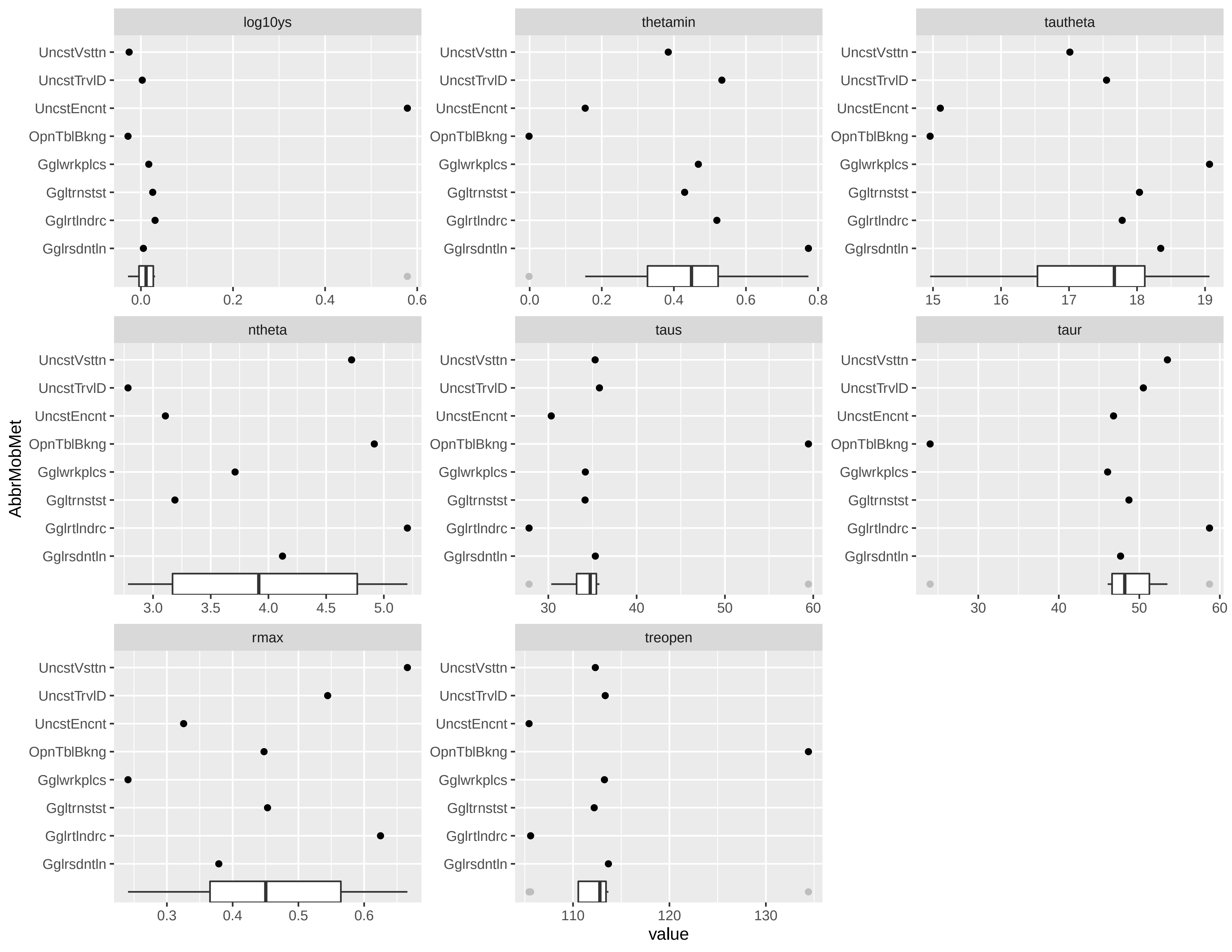

log10ys
thetamin
tautheta
ntheta
taus
taur
rmax
treopen
UncstVsttn
UncstTrvlD
UncstEncnt
OpnTblBkng
Gglwrkplcs
Ggltrnstst
Gglrtlndrc
Gglrsdntln
AbbrMobMet
value

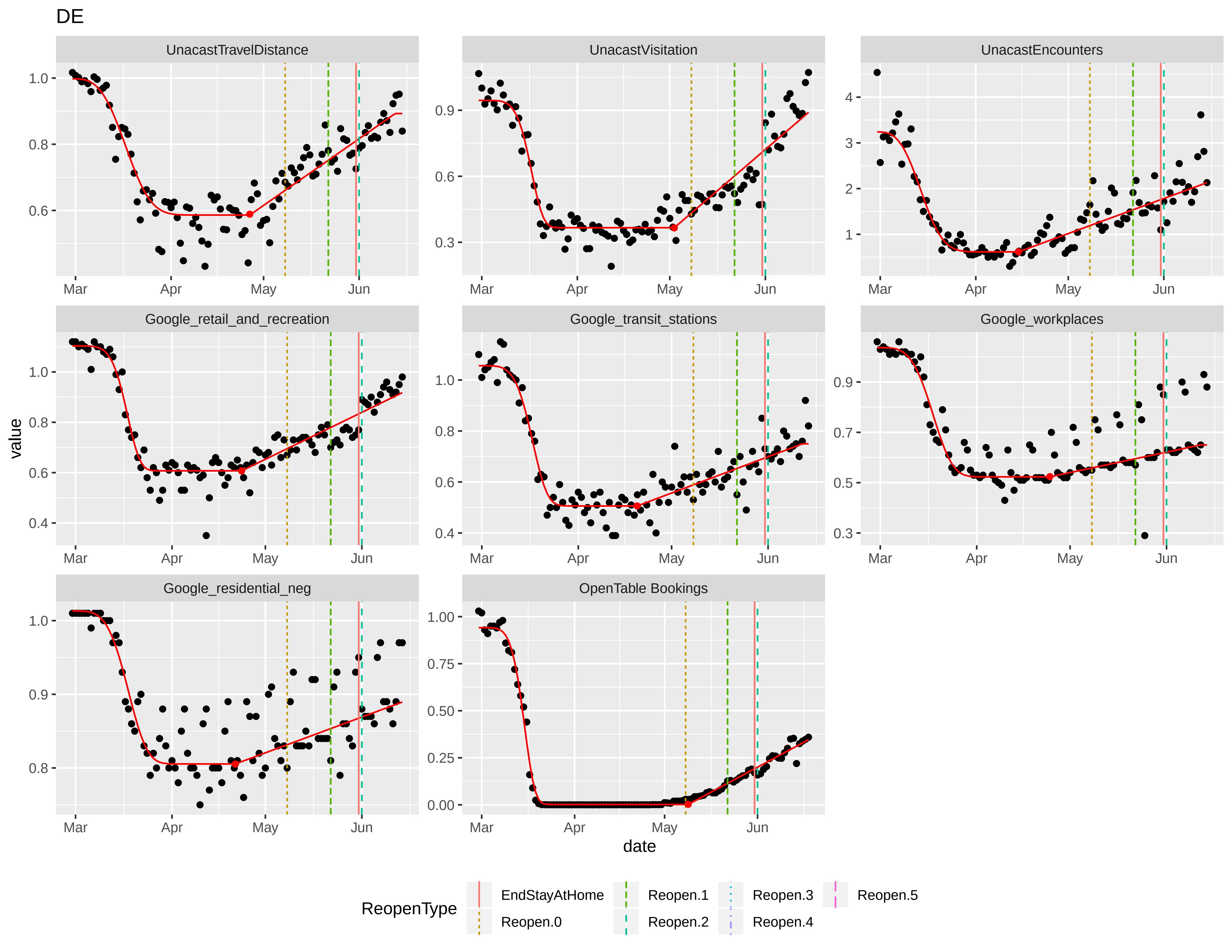

DE
UnacastTravelDistance
UnacastVisitation
UnacastEncounters
Google_retail_and_recreation
Google_transit_stations
Google_workplaces
Google_residential_neg
OpenTable Bookings
value
date
Mar
Apr
May
Jun
ReopenType
EndStayAtHome
Reopen.0
Reopen.1
Reopen.2
Reopen.3
Reopen.4
Reopen.5

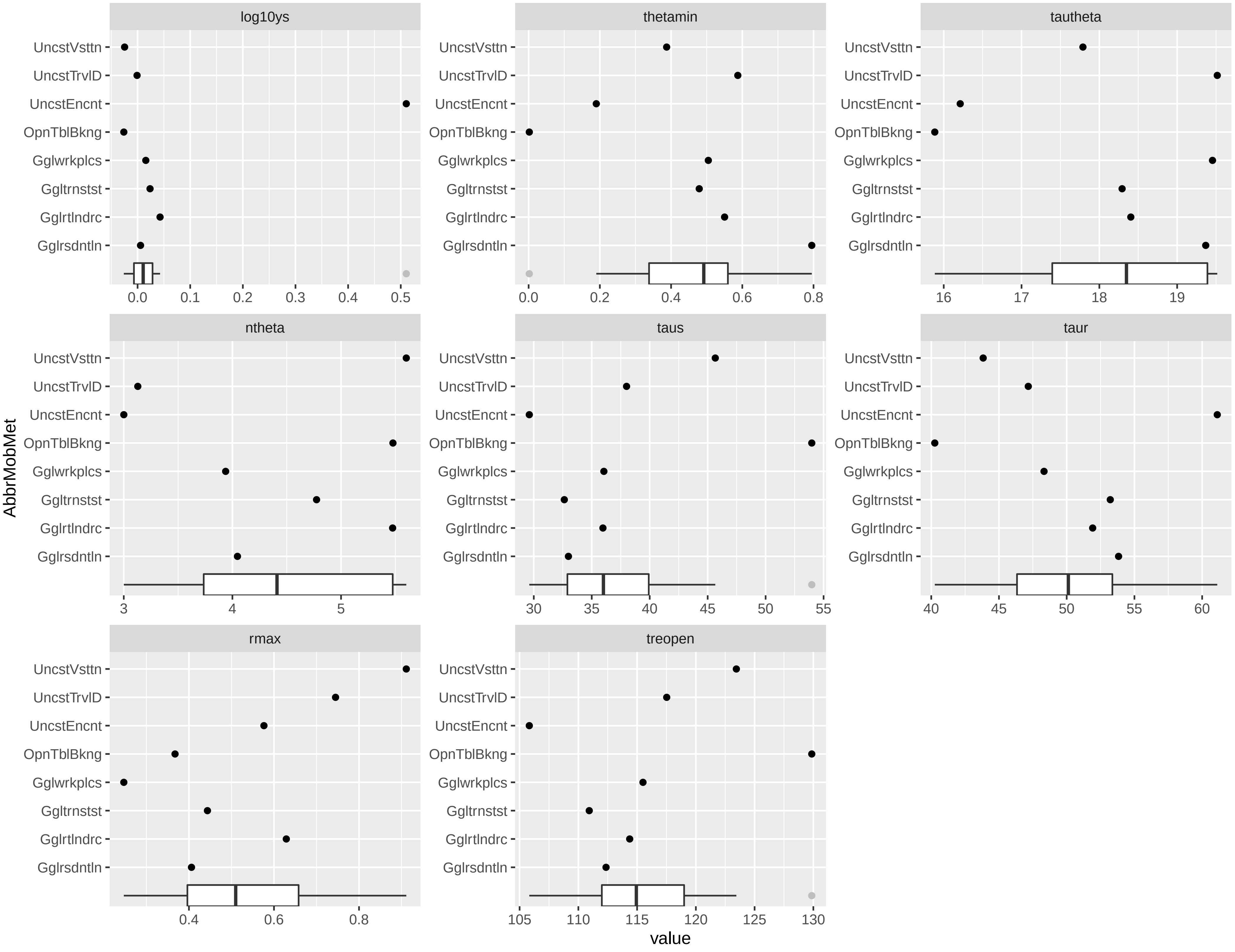
log10ys
thetamin
tautheta
ntheta
taus
taur
rmax
treopen
UncstVsttn
UncstTrvlD
UncstEncnt
OpnTblBkng
Gglwrkplcs
Ggltrnstst
Gglrtlndrc
Gglrsdntln
AbbrMobMet
value

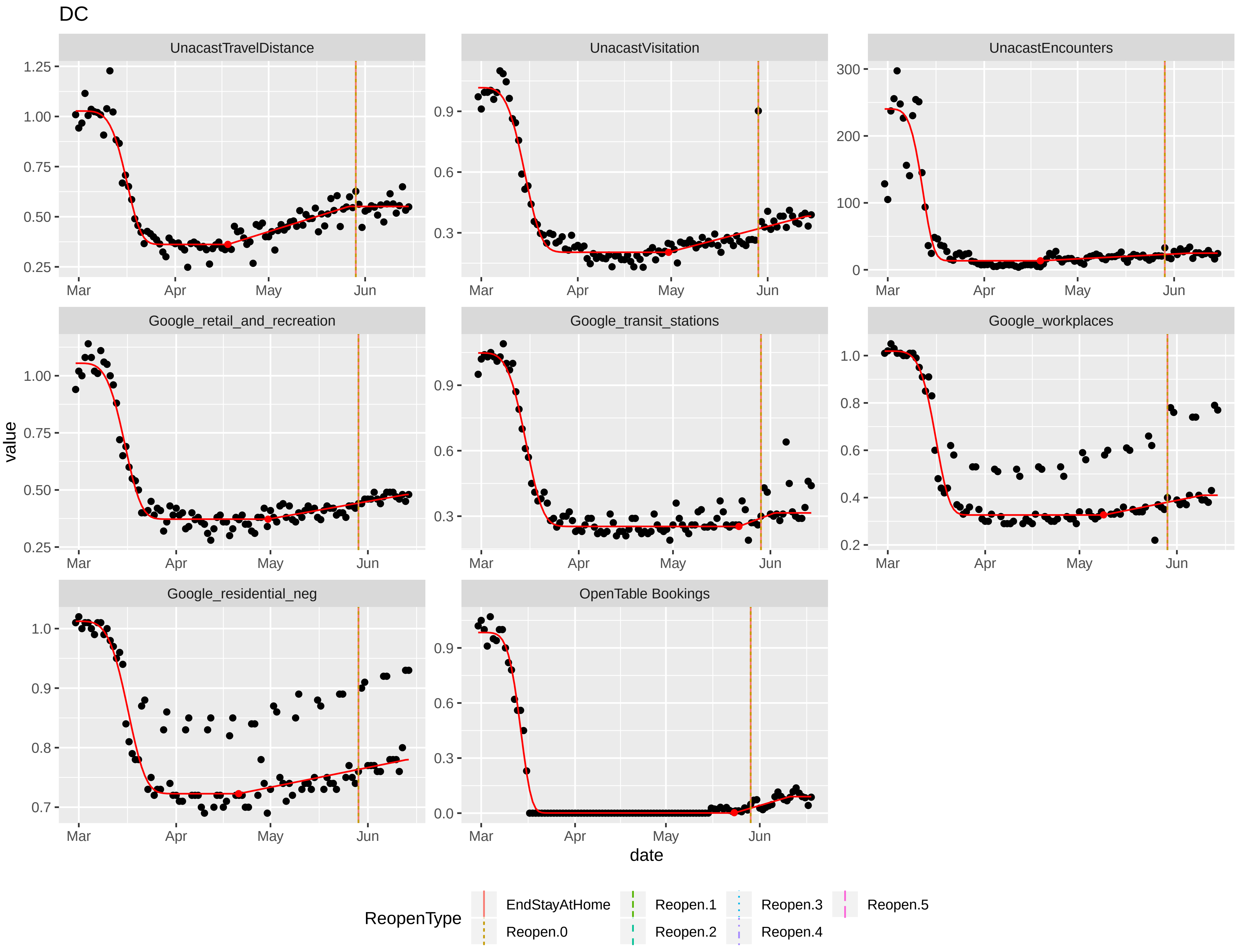
DC
UnacastTravelDistance
UnacastVisitation
UnacastEncounters
Google_retail_and_recreation
Google_transit_stations
Google_workplaces
Google_residential_neg
OpenTable Bookings
value
date
Mar
Apr
May
Jun
ReopenType
EndStayAtHome
Reopen.0
Reopen.1
Reopen.2
Reopen.3
Reopen.4
Reopen.5

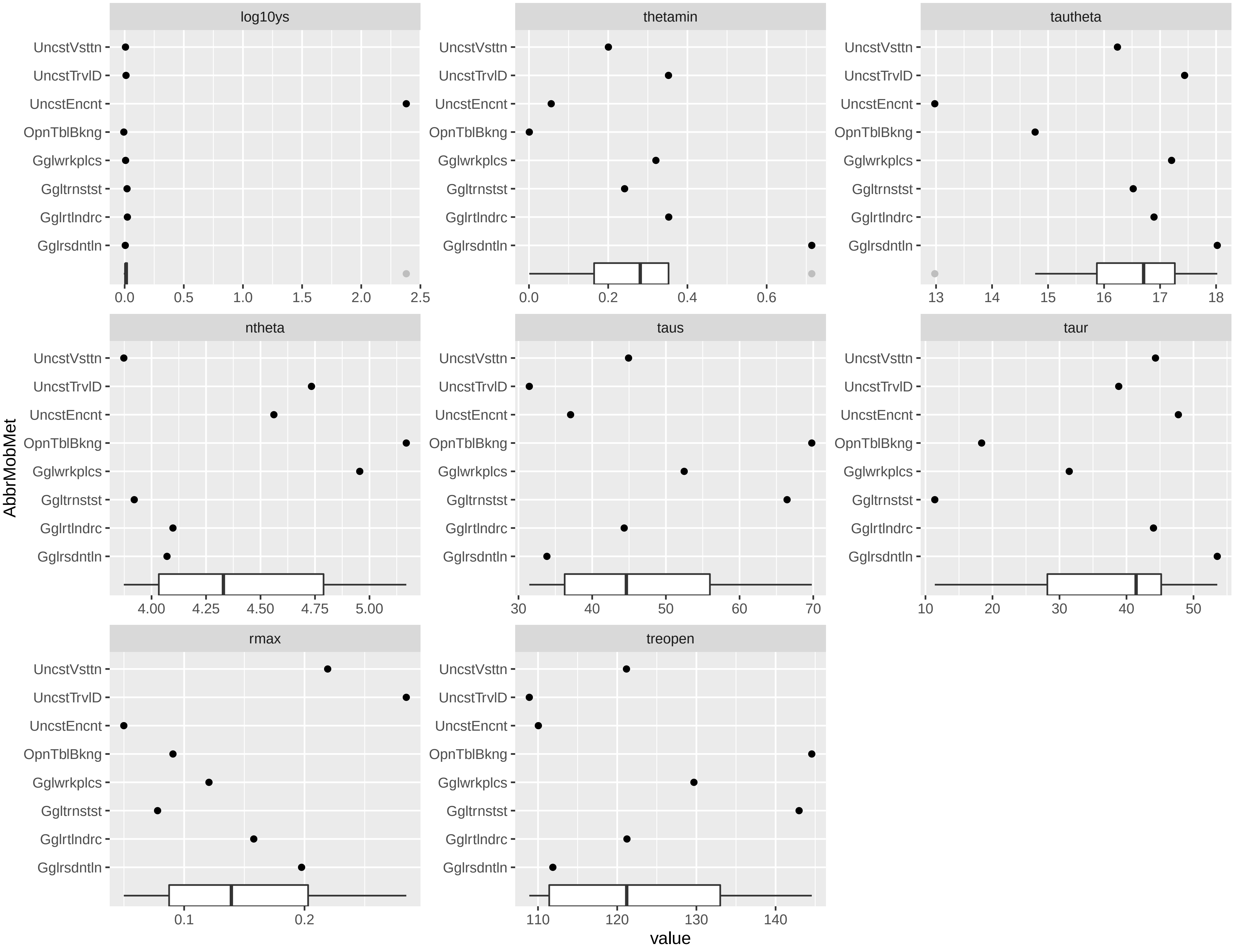
log10ys
thetamin
tautheta
ntheta
taus
taur
rmax
treopen
UncstVsttn
UncstTrvlD
UncstEncnt
OpnTblBkng
Gglwrkplcs
Ggltrnstst
Gglrtlndrc
Gglrsdntln
AbbrMobMet
value

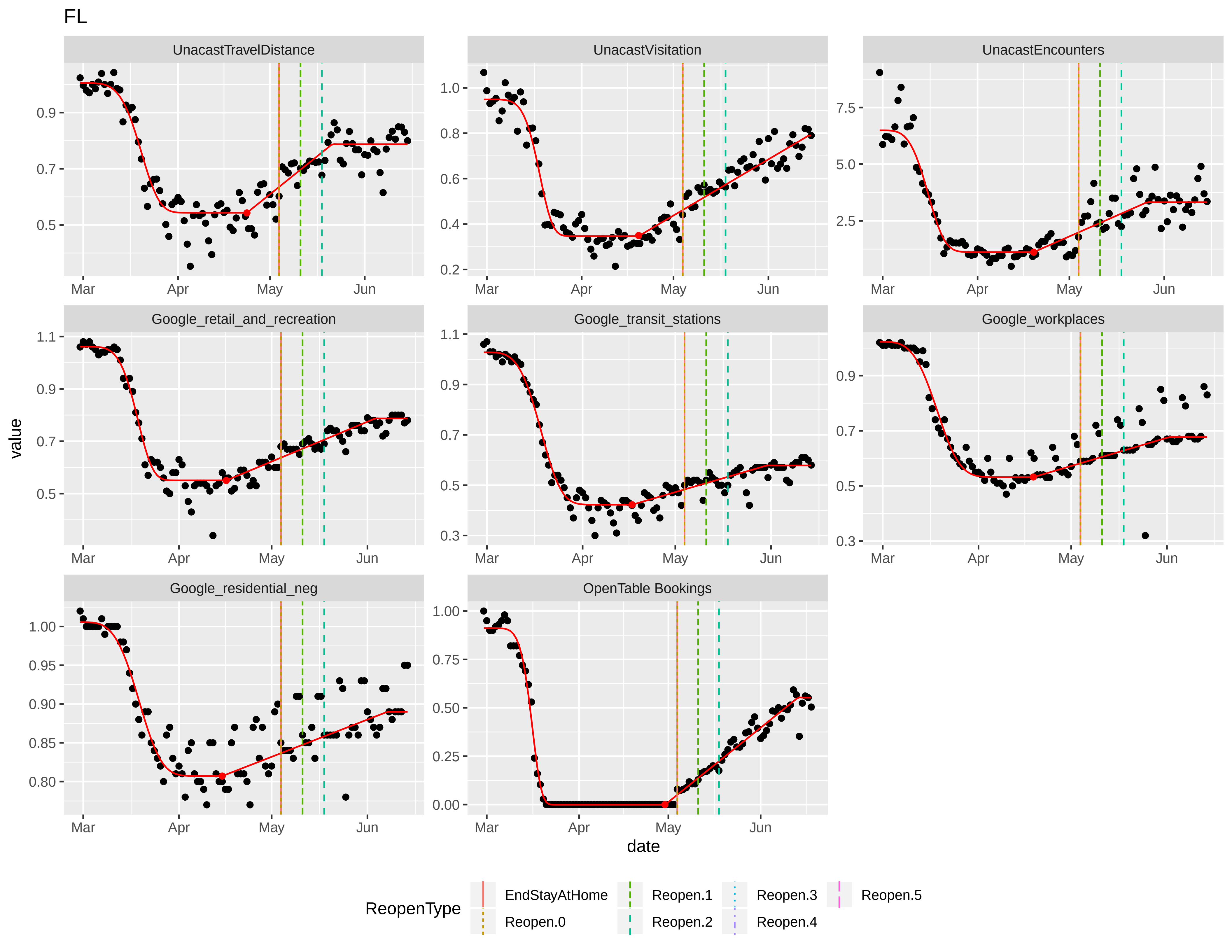
FL
UnacastTravelDistance
UnacastVisitation
UnacastEncounters
Google_retail_and_recreation
Google_transit_stations
Google_workplaces
Google_residential_neg
OpenTable Bookings
value
date
Mar
Apr
May
Jun
ReopenType
EndStayAtHome
Reopen.0
Reopen.1
Reopen.2
Reopen.3
Reopen.4
Reopen.5

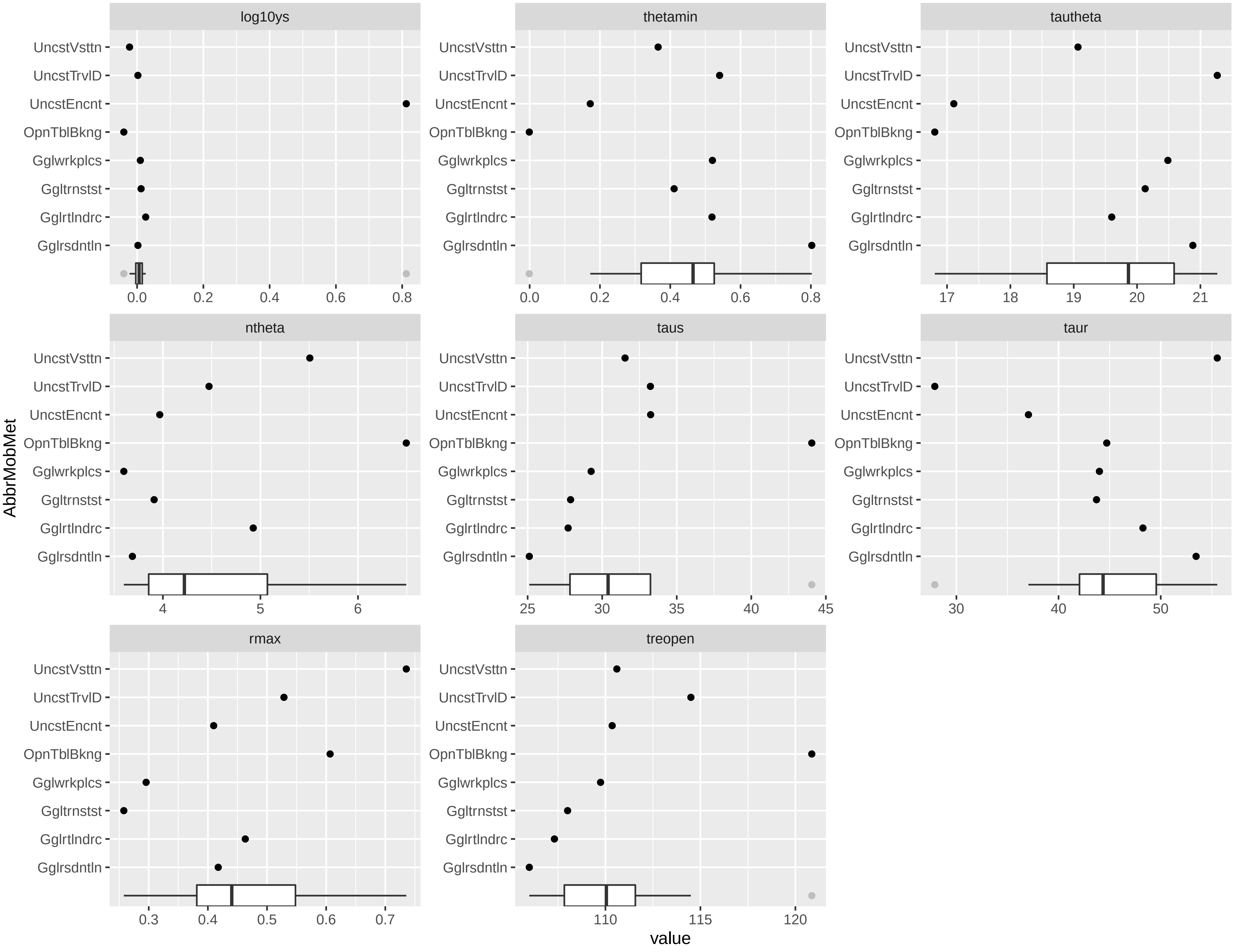
log10ys
thetamin
tautheta
ntheta
taus
taur
rmax
treopen
UncstVsttn
UncstTrvlD
UncstEncnt
OpnTblBkng
Gglwrkplcs
Ggltrnstst
Gglrtlndrc
Gglrsdntln
AbbrMobMet
value

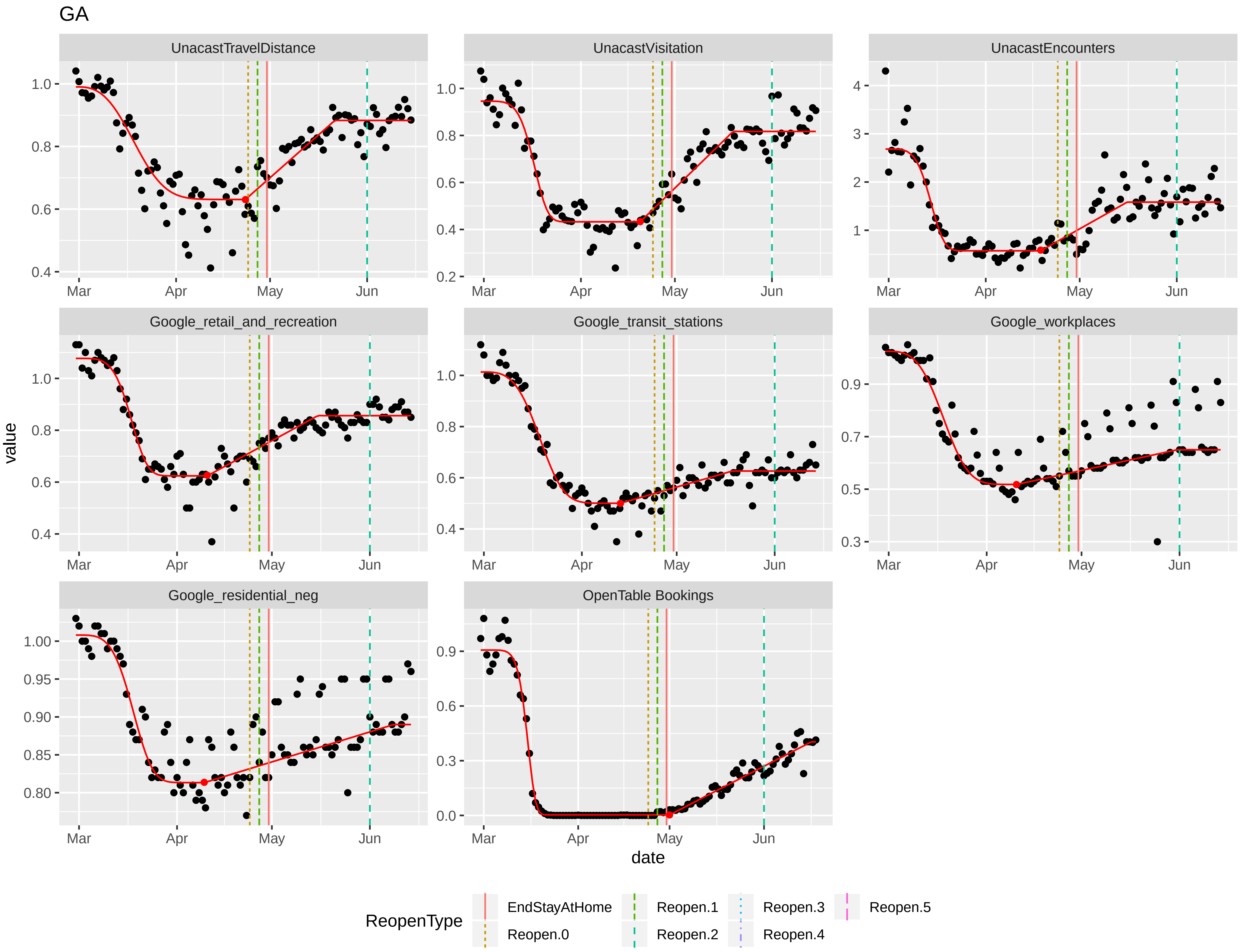

GA
UnacastTravelDistance
UnacastVisitation
UnacastEncounters
Google_retail_and_recreation
Google_transit_stations
Google_workplaces
Google_residential_neg
OpenTable Bookings
value
date
Mar
Apr
May
Jun
ReopenType
EndStayAtHome
Reopen.0
Reopen.1
Reopen.2
Reopen.3
Reopen.4
Reopen.5

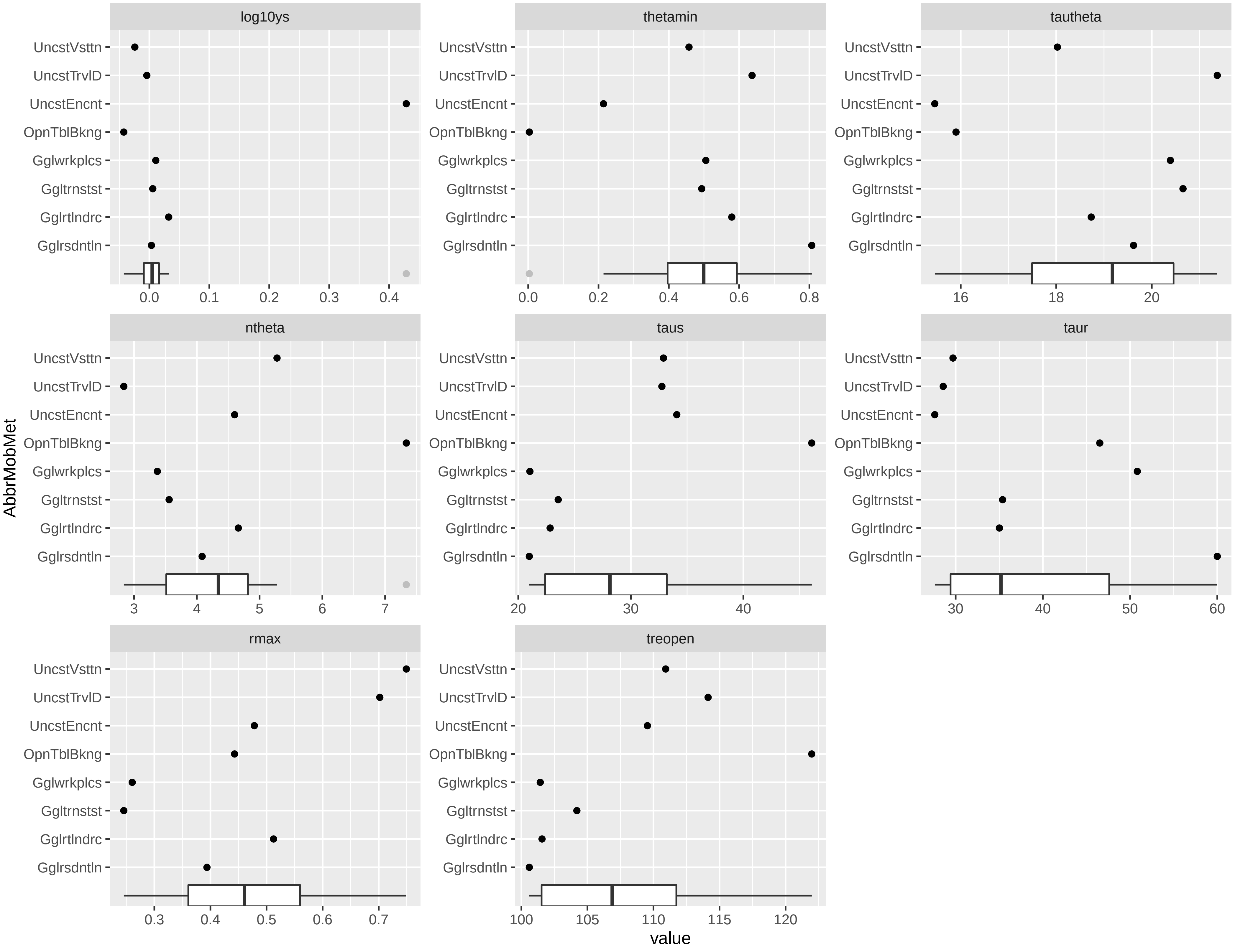
log10ys
thetamin
tautheta
ntheta
taus
taur
rmax
treopen
UncstVsttn
UncstTrvlD
UncstEncnt
OpnTblBkng
Gglwrkplcs
Reviewed: Ggltrnstst
Gglrtlndrc
Gglrsdntln
AbbrMobMet
value

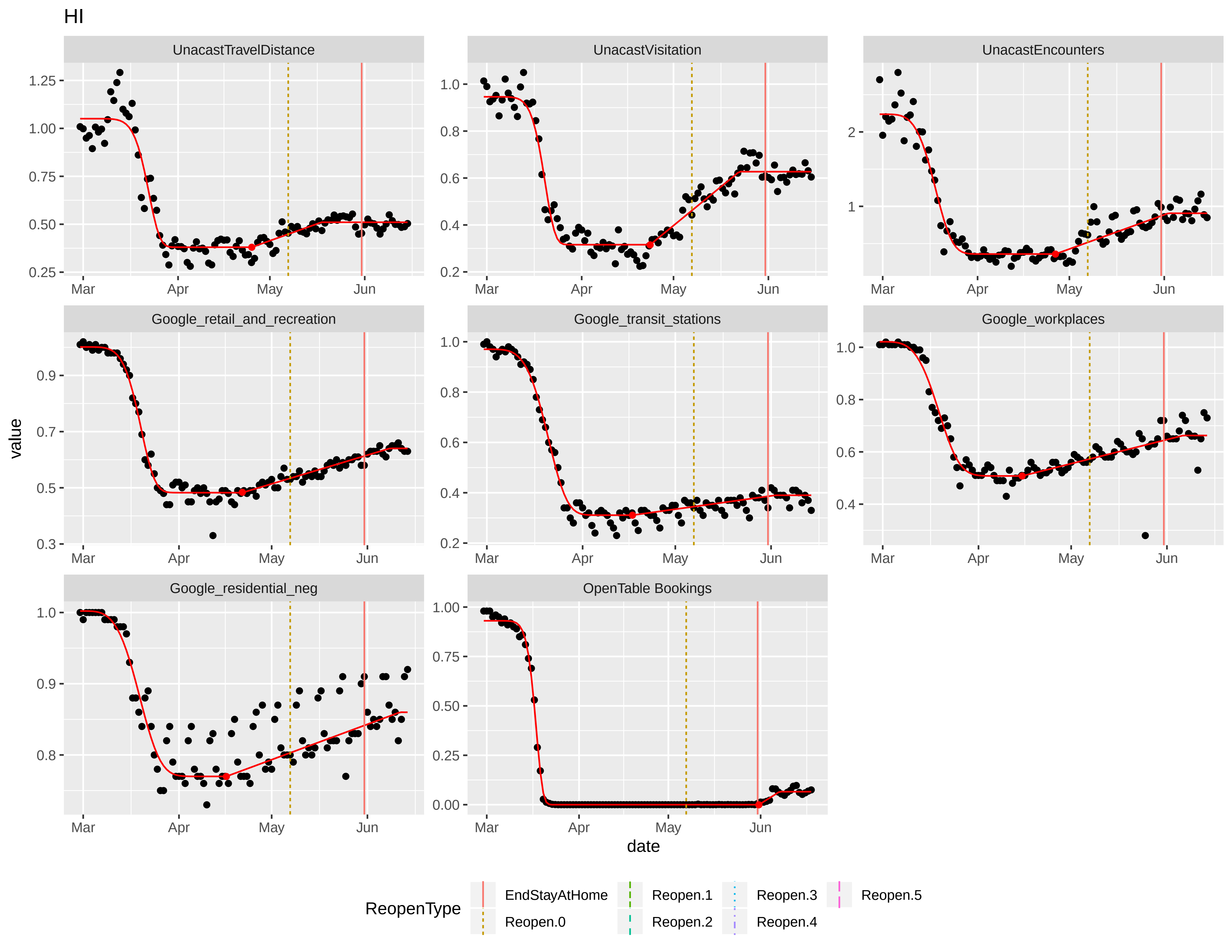

HI
UnacastTravelDistance
UnacastVisitation
UnacastEncounters
Google_retail_and_recreation
Google_transit_stations
Google_workplaces
Google_residential_neg
OpenTable Bookings
value
date
Mar
Apr
May
Jun
ReopenType
EndStayAtHome
Reopen.0
Reopen.1
Reopen.2
Reopen.3
Reopen.4
Reopen.5

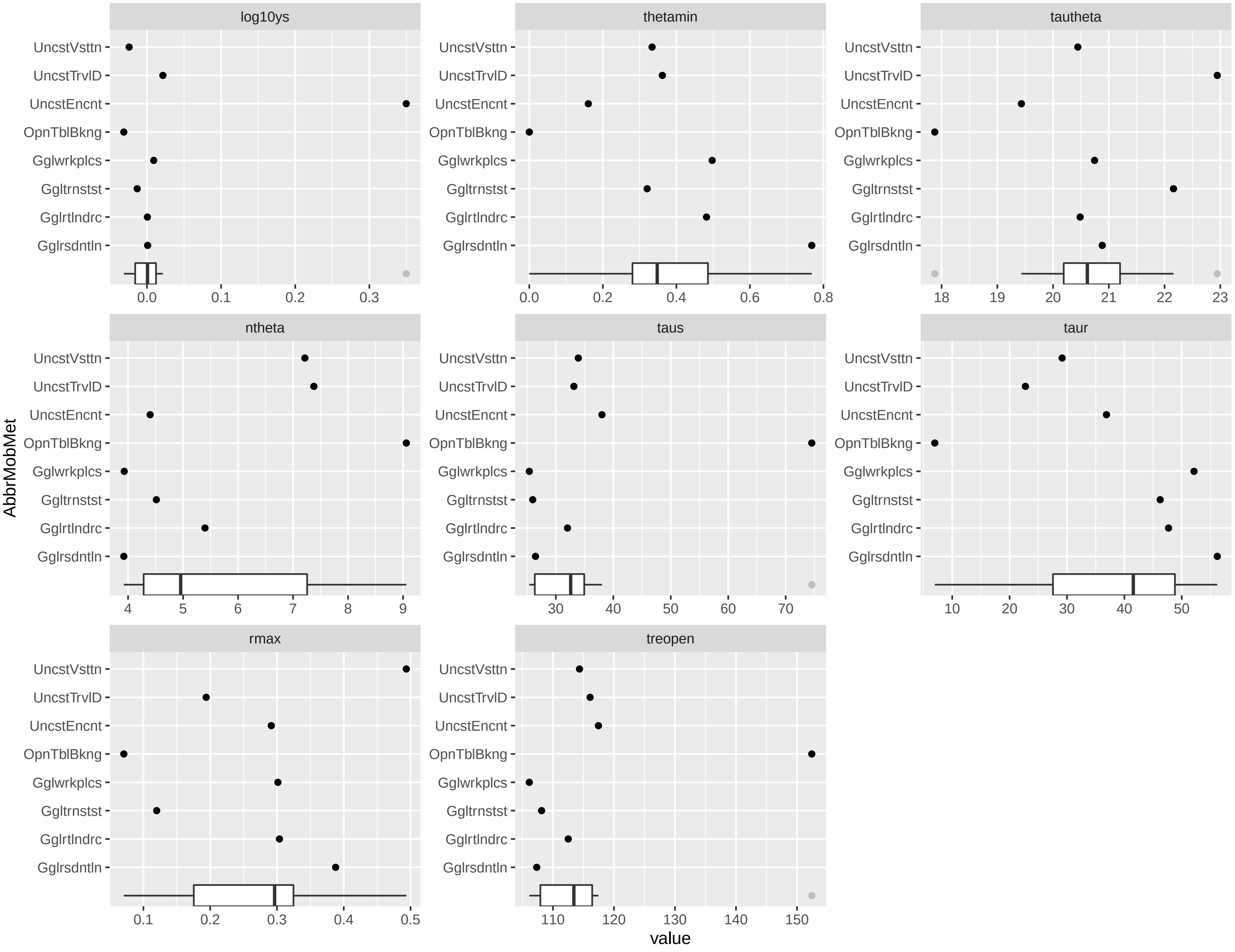
log10ys
thetamin
tautheta
ntheta
taus
taur
rmax
treopen
UncstVsttn
UncstTrvlD
UncstEncnt
OpnTblBkng
Gglwrkplcs
Ggltrnstst
Gglrtlndrc
Gglrsdntln
AbbrMobMet
value

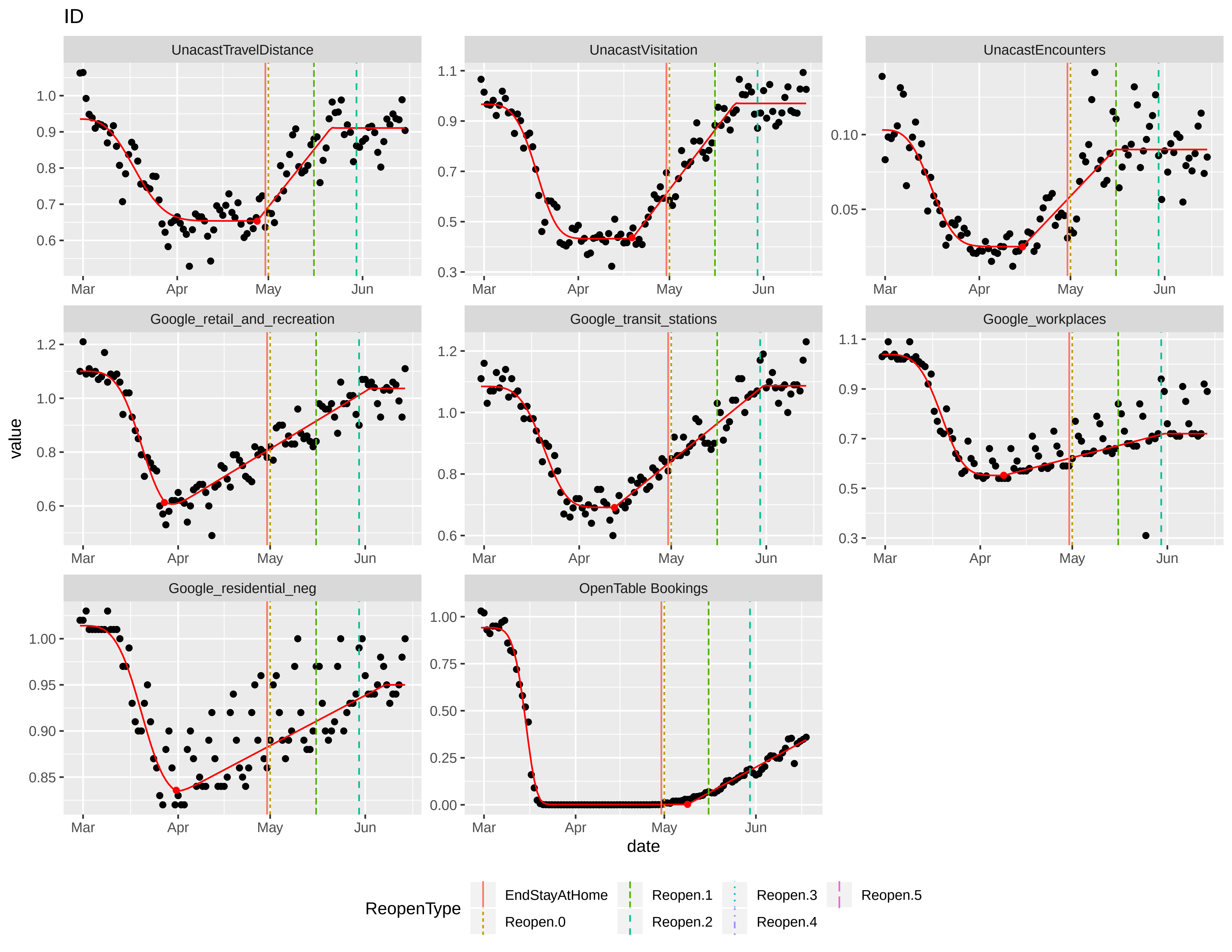

ID
UnacastTravelDistance
UnacastVisitation
UnacastEncounters
Google_retail_and_recreation
Google_transit_stations
Google_workplaces
Google_residential_neg
OpenTable Bookings
value
date
Mar
Apr
May
Jun
ReopenType
EndStayAtHome
Reopen.0
Reopen.1
Reopen.2
Reopen.3
Reopen.4
Reopen.5

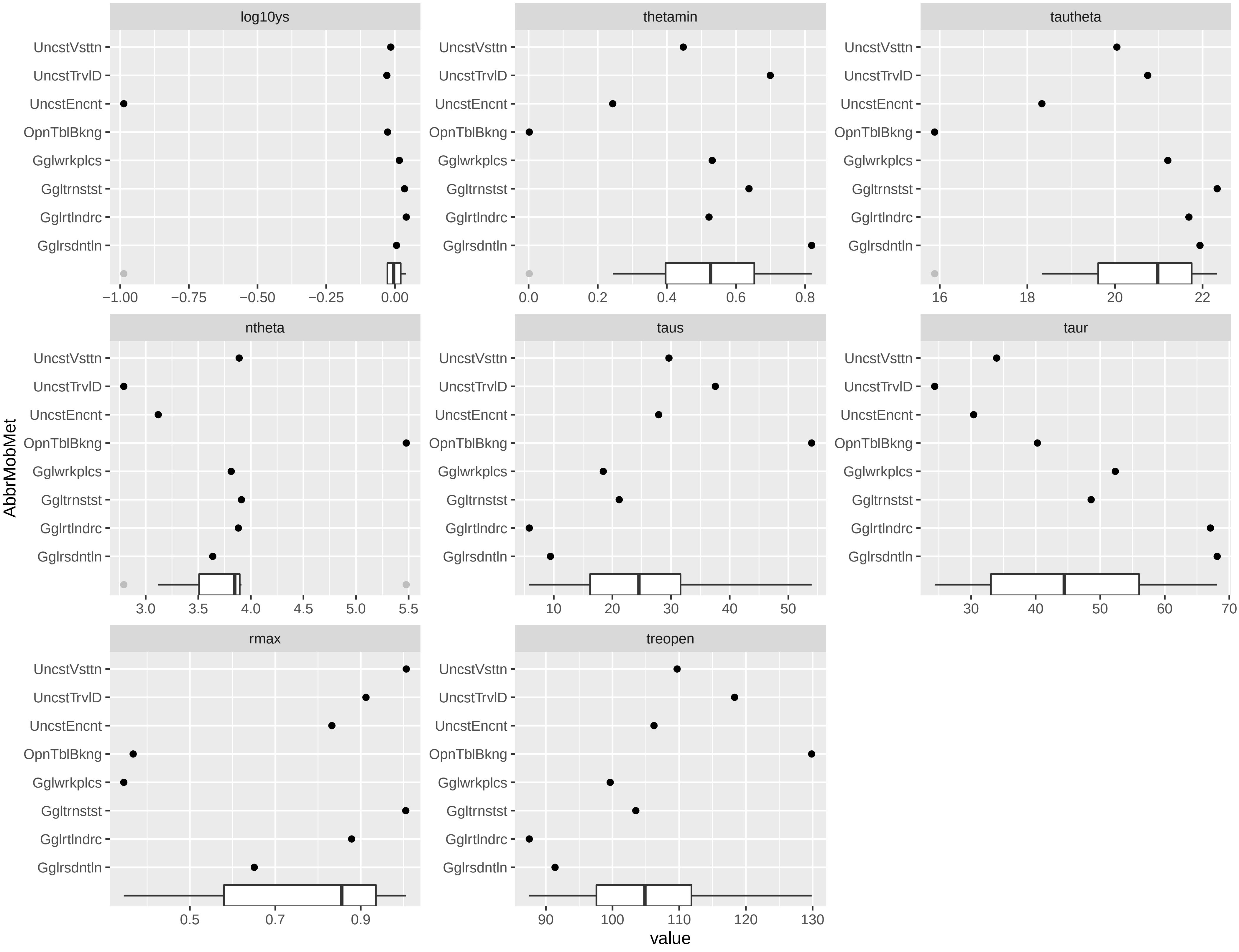

log10ys
thetamin
tautheta
ntheta
taus
taur
rmax
treopen
UncstVsttn
UncstTrvlD
UncstEncnt
OpnTblBkng
Gglwrkplcs
Ggltrnstst
Gglrtlndrc
Gglrsdntln
AbbrMobMet
value

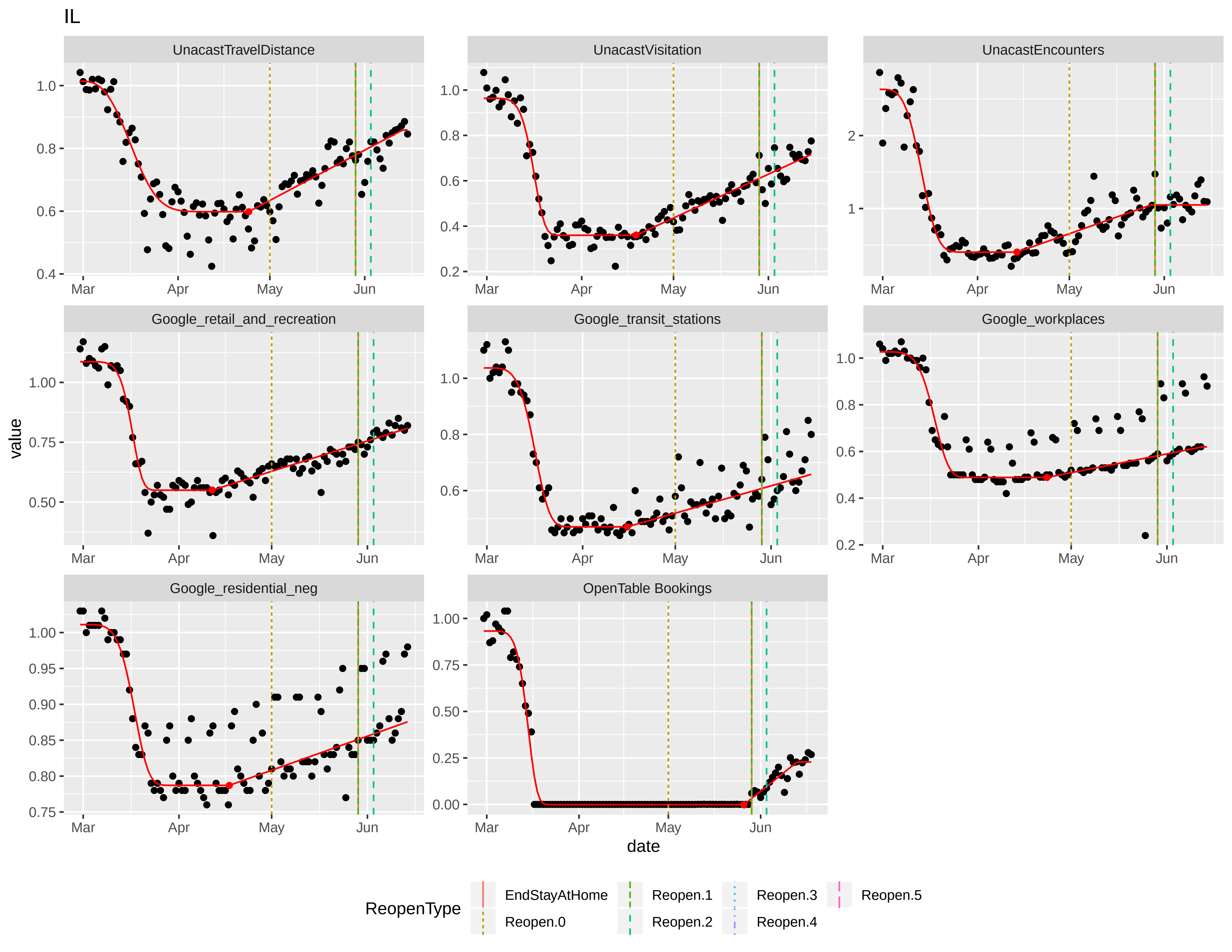

IL
UnacastTravelDistance
UnacastVisitation
UnacastEncounters
Google_retail_and_recreation
Google_transit_stations
Google_workplaces
Google_residential_neg
OpenTable Bookings
value
date
Mar
Apr
May
Jun
ReopenType
EndStayAtHome
Reopen.0
Reopen.1
Reopen.2
Reopen.3
Reopen.4
Reopen.5

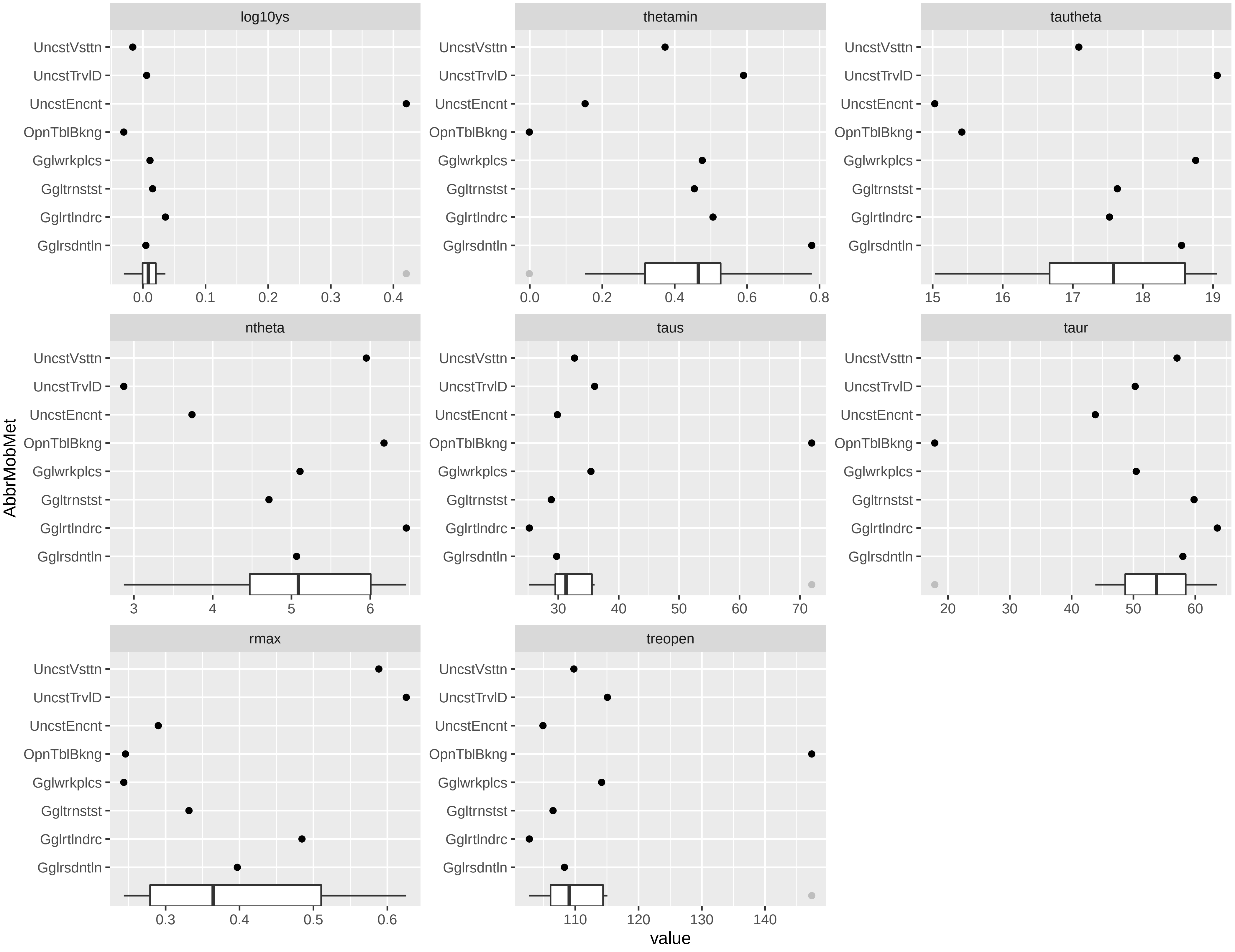

log10ys
thetamin
tautheta
ntheta
taus
taur
rmax
treopen
UncstVsttn
UncstTrvlD
UncstEncnt
OpnTblBkng
Gglwrkplcs
Ggltrnstst
Gglrtlndrc
Gglrsdntln
AbbrMobMet
value

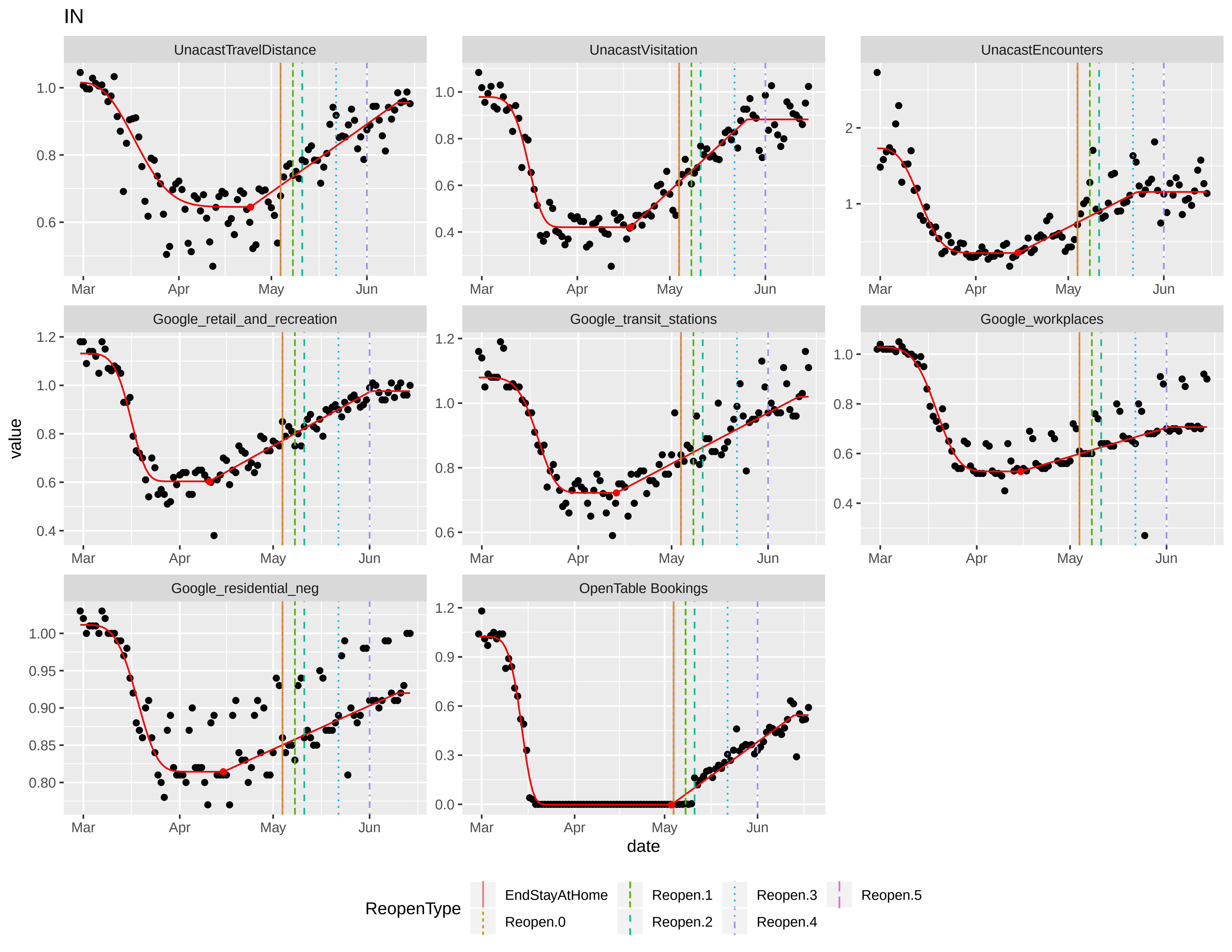

IN
UnacastTravelDistance
UnacastVisitation
UnacastEncounters
Google_retail_and_recreation
Google_transit_stations
Google_workplaces
Google_residential_neg
OpenTable Bookings
value
date
Mar
Apr
May
Jun
ReopenType
EndStayAtHome
Reopen.0
Reopen.1
Reopen.2
Reopen.3
Reopen.4
Reopen.5

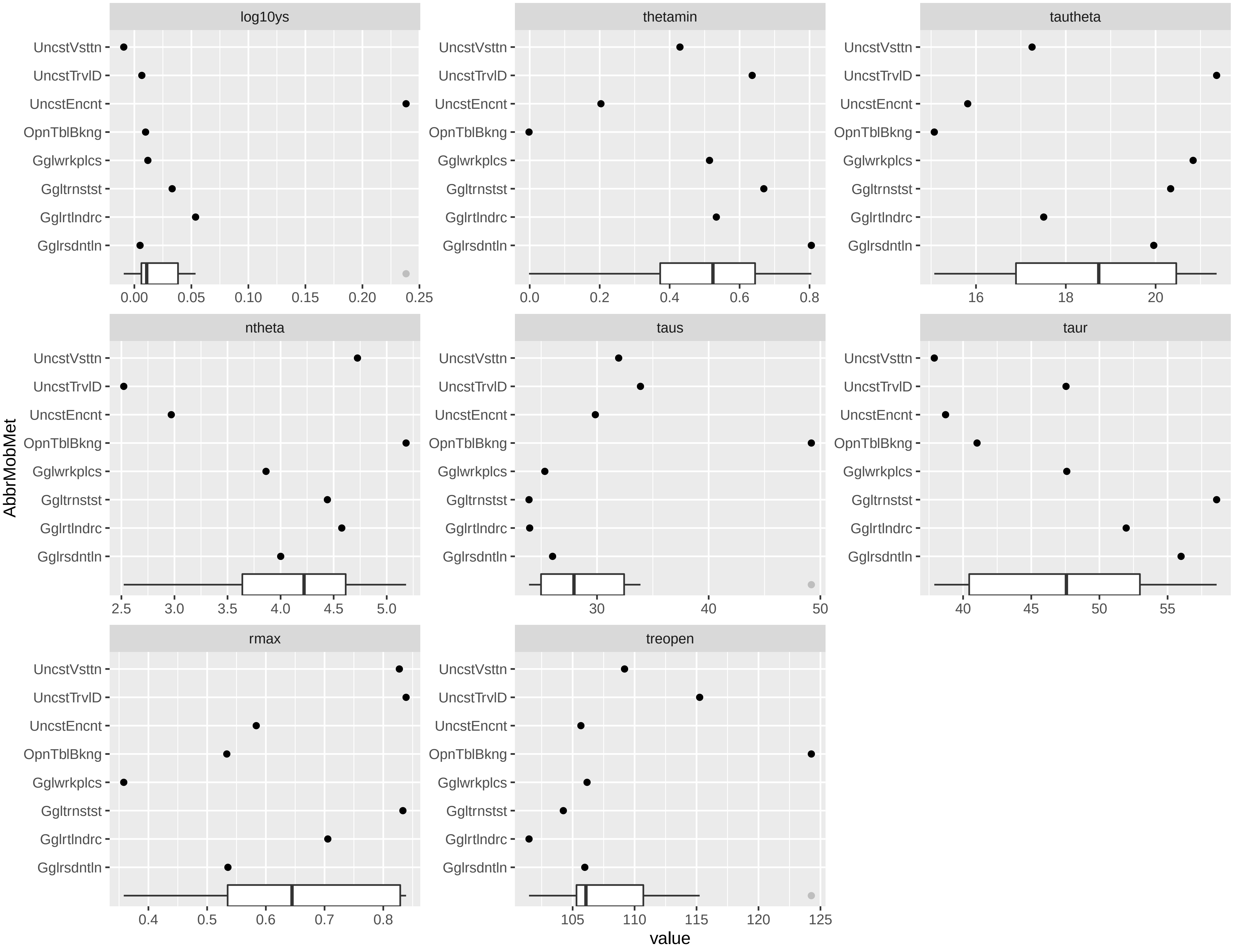

log10ys
thetamin
tautheta
ntheta
taus
taur
rmax
treopen
UncstVsttn
UncstTrvlD
UncstEncnt
OpnTblBkng
Gglwrkplcs
Ggltrnstst
Gglrtlndrc
Gglrsdntln
0.00
0.05
0.10
0.15
0.20
0.25
0.0
0.2
0.4
0.6
0.8
16
18
20
2.5
3.0
3.5
4.0
4.5
5.0
30
40
50
40
45
50
55
0.4
0.5
0.6
0.7
0.8
105
110
115
120
125
AbbrMobMet
value

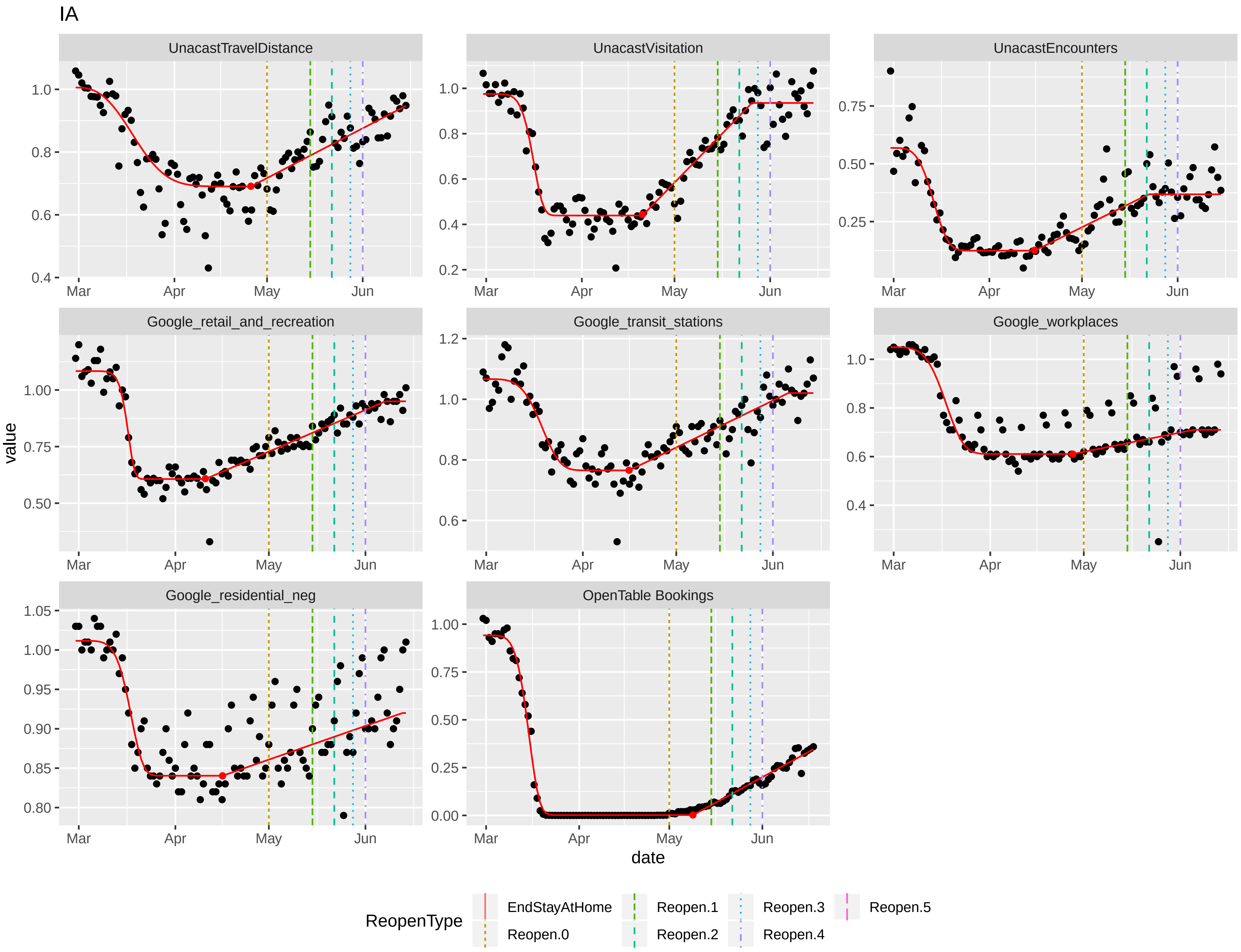
IA
UnacastTravelDistance
UnacastVisitation
UnacastEncounters
Google_retail_and_recreation
Google_transit_stations
Google_workplaces
Google_residential_neg
OpenTable Bookings
value
date
Mar
Apr
May
Jun
ReopenType
EndStayAtHome
Reopen.0
Reopen.1
Reopen.2
Reopen.3
Reopen.4
Reopen.5

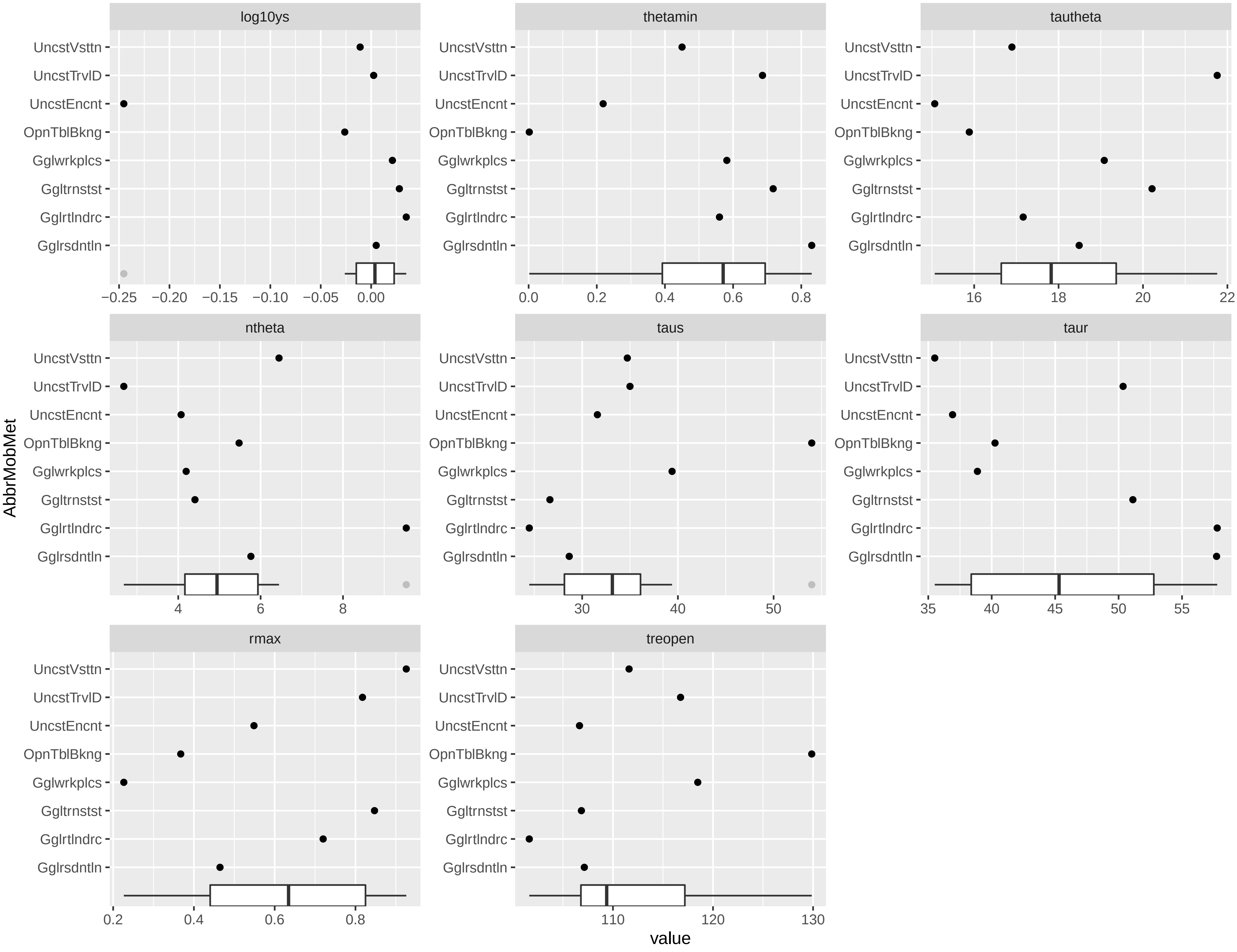
log10ys
thetamin
tautheta
ntheta
taus
taur
rmax
treopen
UncstVsttn
UncstTrvlD
UncstEncnt
OpnTblBkng
Gglwrkplcs
Ggltrnstst
Gglrtlndrc
Gglrsdntln
AbbrMobMet
value

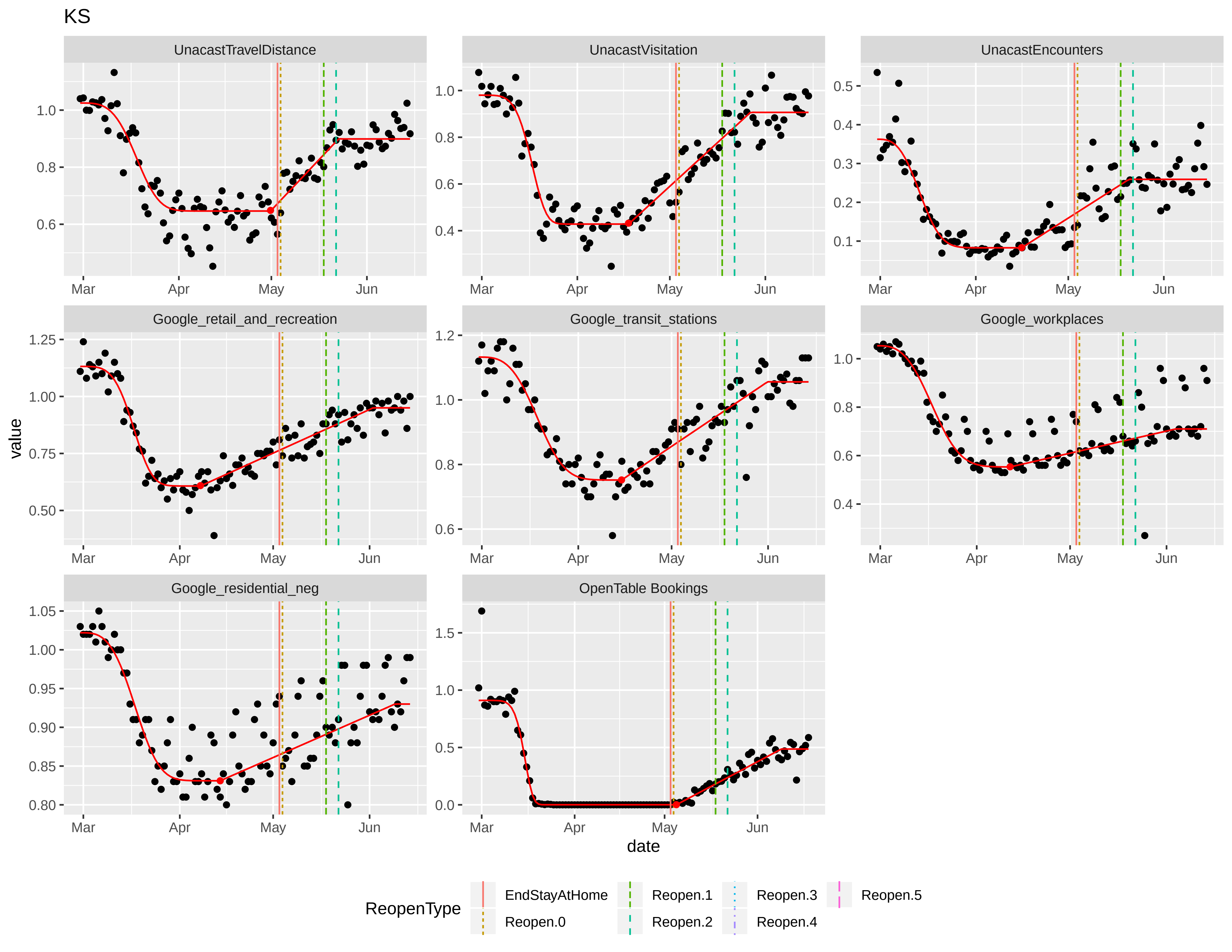
KS
UnacastTravelDistance
UnacastVisitation
UnacastEncounters
Google_retail_and_recreation
Google_transit_stations
Google_workplaces
Google_residential_neg
OpenTable Bookings
value
date
Mar
Apr
May
Jun
ReopenType
EndStayAtHome
Reopen.0
Reopen.1
Reopen.2
Reopen.3
Reopen.4
Reopen.5

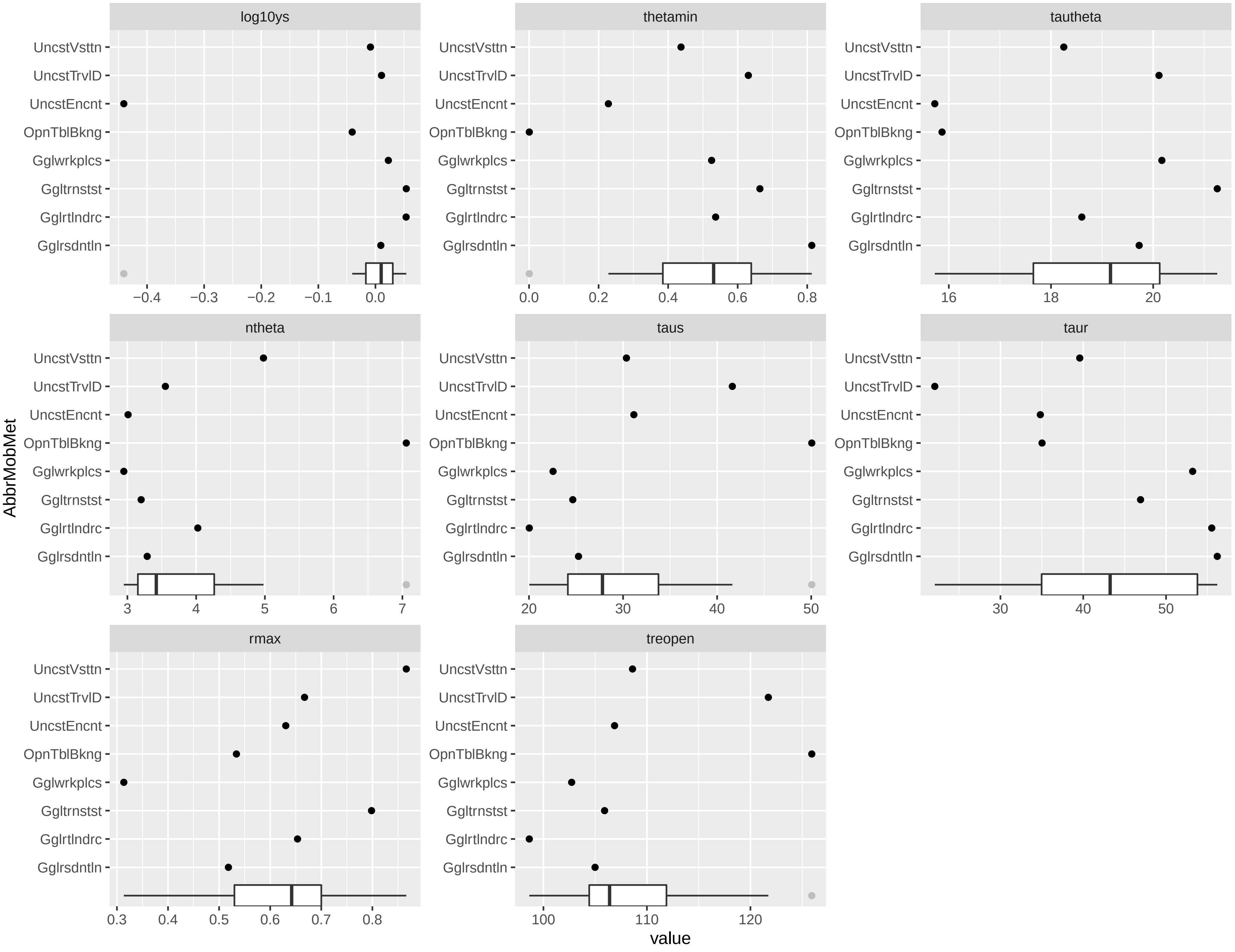

log10ys
thetamin
tautheta
ntheta
taus
taur
rmax
treopen
UncstVsttn
UncstTrvlD
UncstEncnt
OpnTblBkng
Gglwrkplcs
Ggltrnstst
Gglrtlndrc
Gglrsdntln
AbbrMobMet
value

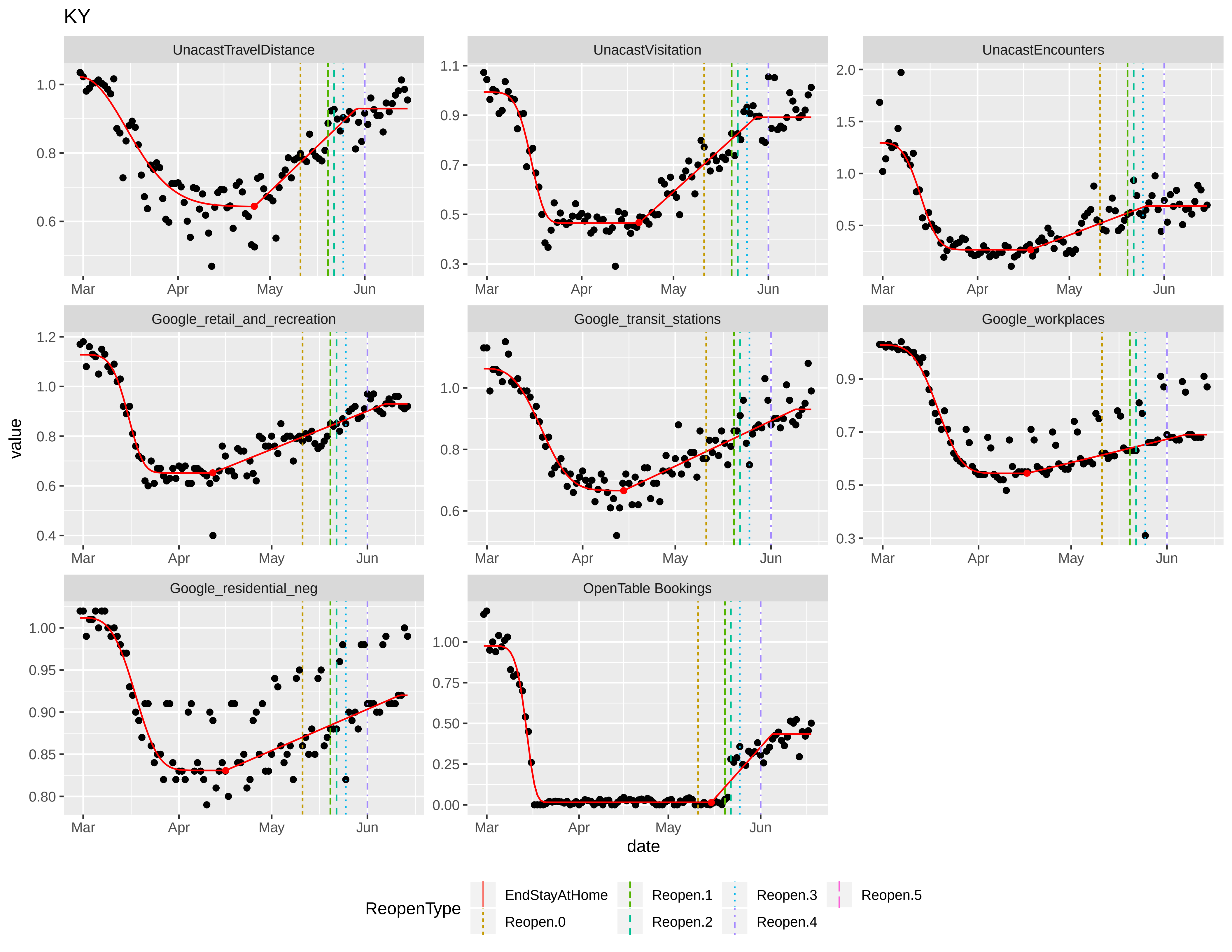

KY
UnacastTravelDistance
UnacastVisitation
UnacastEncounters
Google_retail_and_recreation
Google_transit_stations
Google_workplaces
Google_residential_neg
OpenTable Bookings
value
date
Mar
Apr
May
Jun
ReopenType
EndStayAtHome
Reopen.0
Reopen.1
Reopen.2
Reopen.3
Reopen.4
Reopen.5

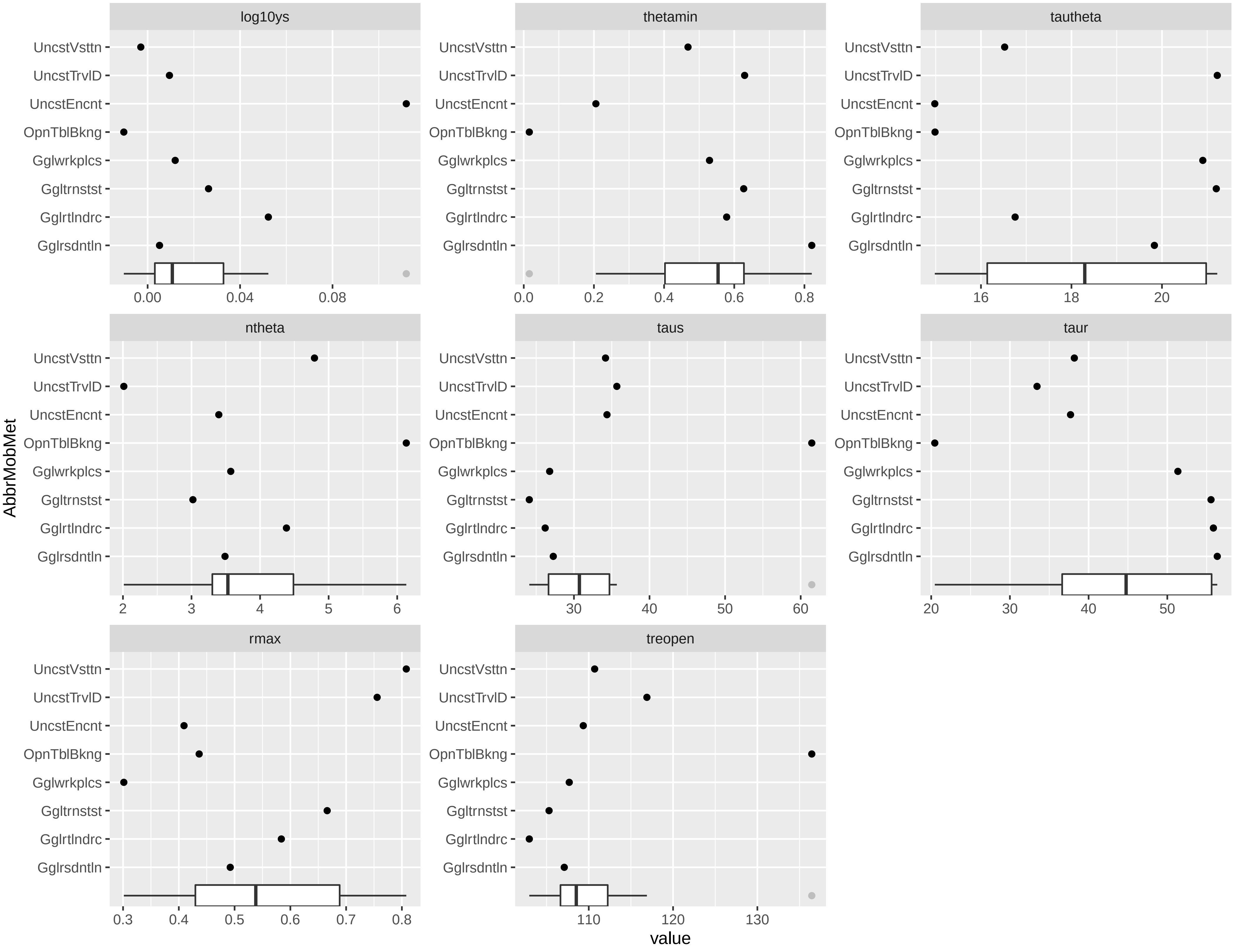
log10ys
thetamin
tautheta
ntheta
taus
taur
rmax
treopen
UncstVsttn
UncstTrvlD
UncstEncnt
OpnTblBkng
Gglwrkplcs
Ggltrnstst
Gglrtlndrc
Gglrsdntln
AbbrMobMet
value
0.00
0.04
0.08
0.0
0.2
0.4
0.6
0.8
16
18
20
2
3
4
5
6
30
40
50
60
20
30
40
50
0.3
0.4
0.5
0.6
0.7
0.8
110
120
130

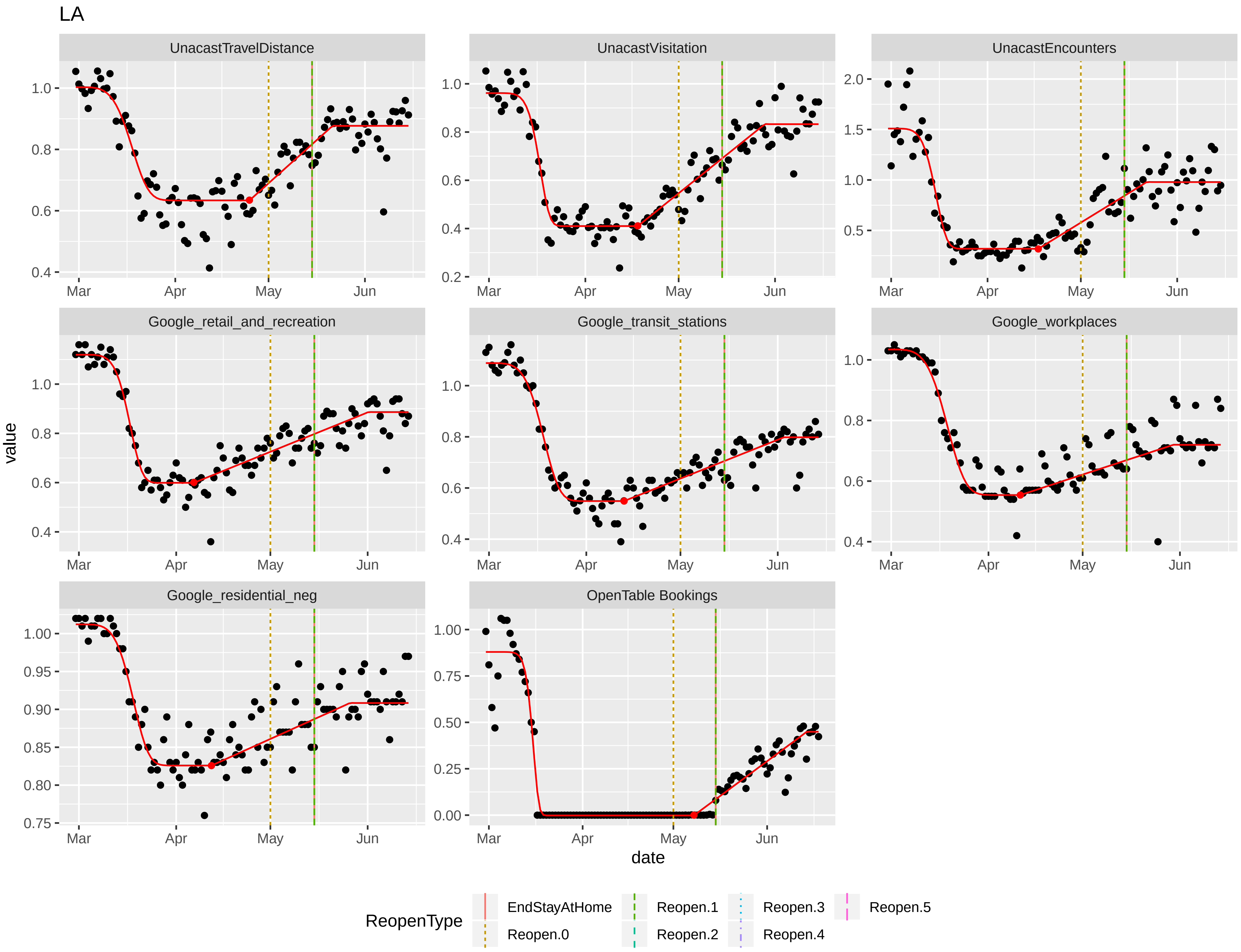
LA
UnacastTravelDistance
UnacastVisitation
UnacastEncounters
Google_retail_and_recreation
Google_transit_stations
Google_workplaces
Google_residential_neg
OpenTable Bookings
value
date
Mar
Apr
May
Jun
ReopenType
EndStayAtHome
Reopen.0
Reopen.1
Reopen.2
Reopen.3
Reopen.4
Reopen.5

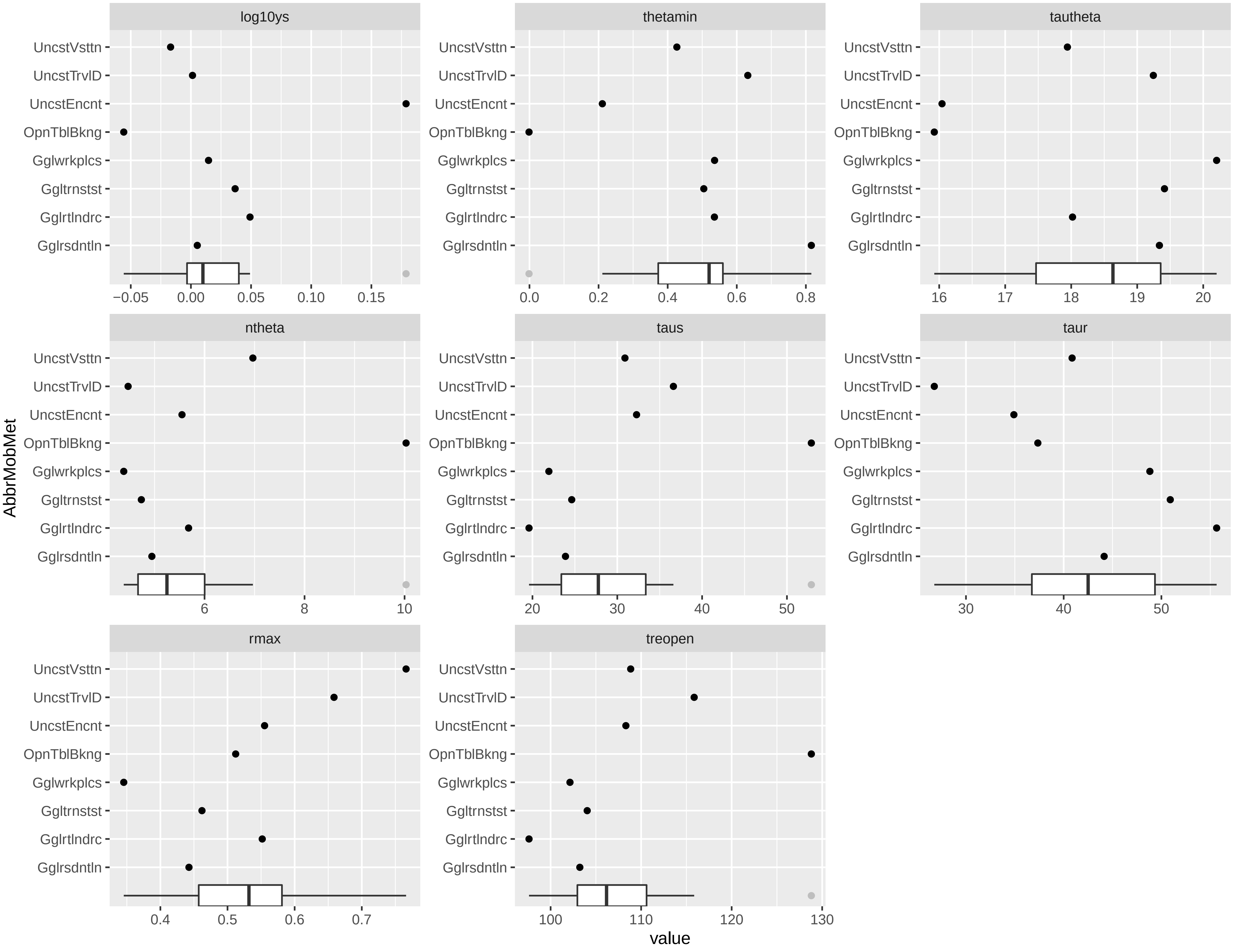
log10ys
thetamin
tautheta
ntheta
taus
taur
rmax
treopen
UncstVsttn
UncstTrvlD
UncstEncnt
OpnTblBkng
Gglwrkplcs
Ggltrnstst
Gglrtlndrc
Gglrsdntln
AbbrMobMet
value

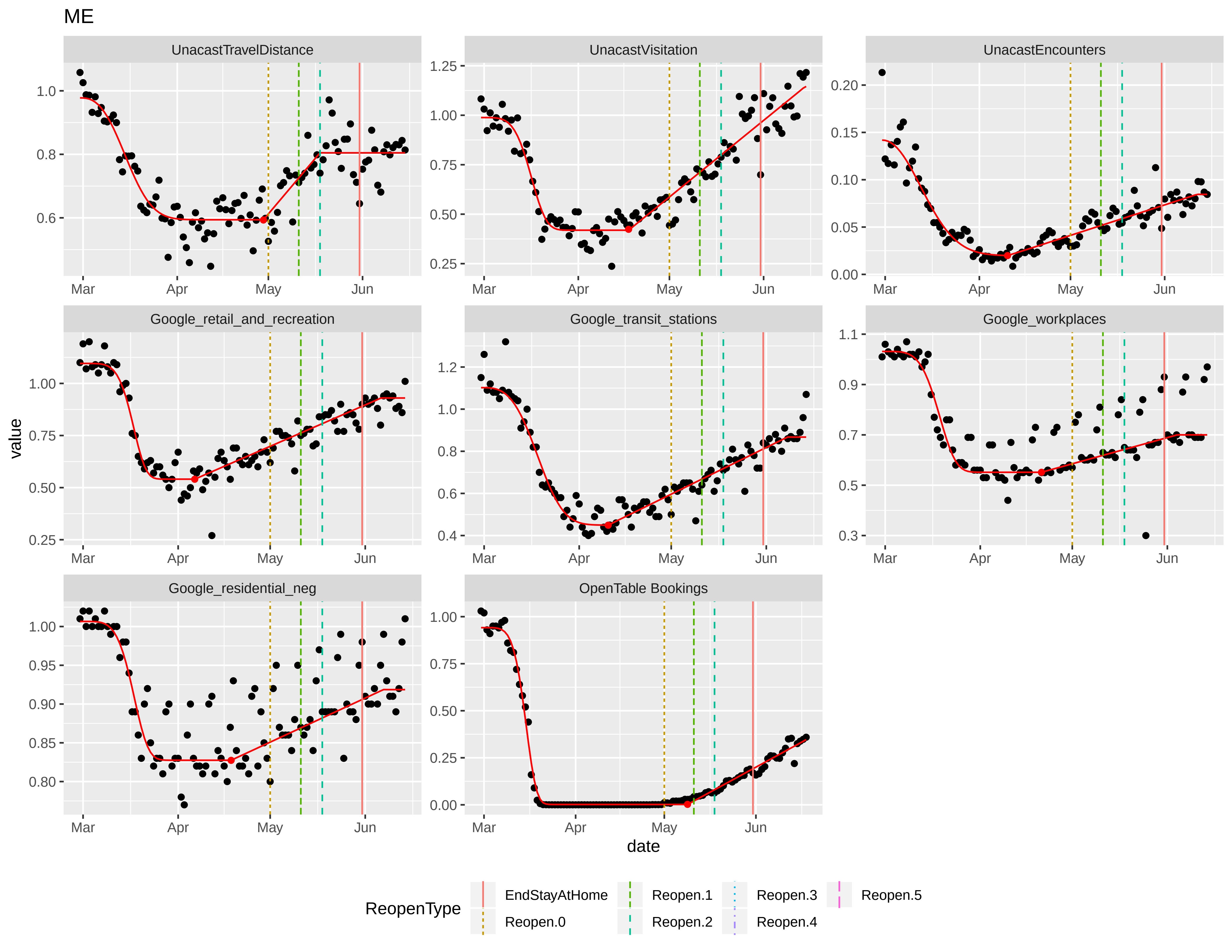

ME
UnacastTravelDistance
UnacastVisitation
UnacastEncounters
Google_retail_and_recreation
Google_transit_stations
Google_workplaces
Google_residential_neg
OpenTable Bookings
value
date
Mar
Apr
May
Jun
ReopenType
EndStayAtHome
Reopen.0
Reopen.1
Reopen.2
Reopen.3
Reopen.4
Reopen.5

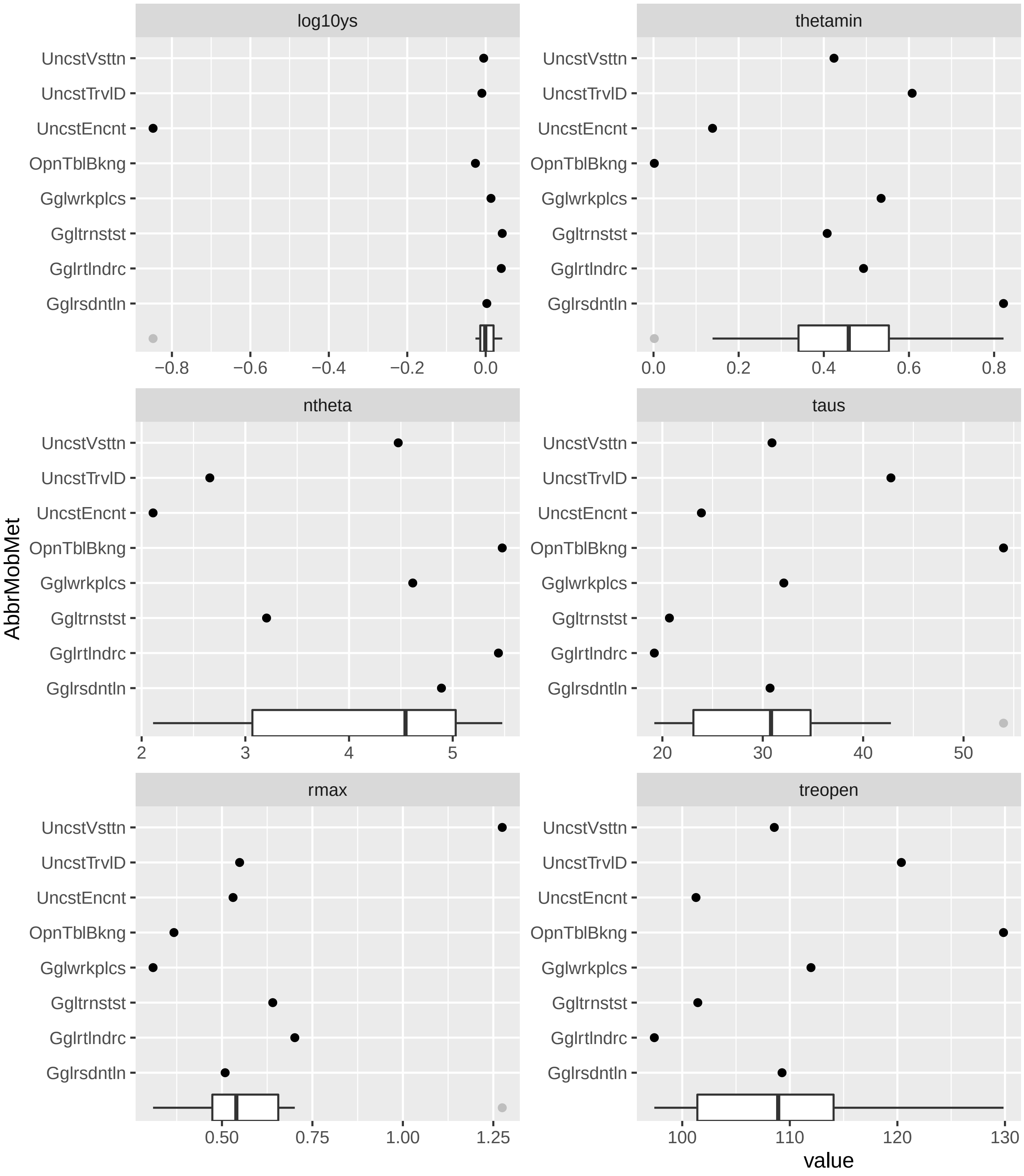
log10ys
thetamin
tautheta
ntheta
taus
taur
rmax
treopen
UncstVsttn
UncstTrvlD
UncstEncnt
OpnTblBkng
Gglwrkplcs
Ggltrnstst
Gglrtlndrc
Gglrsdntln
AbbrMobMet
value

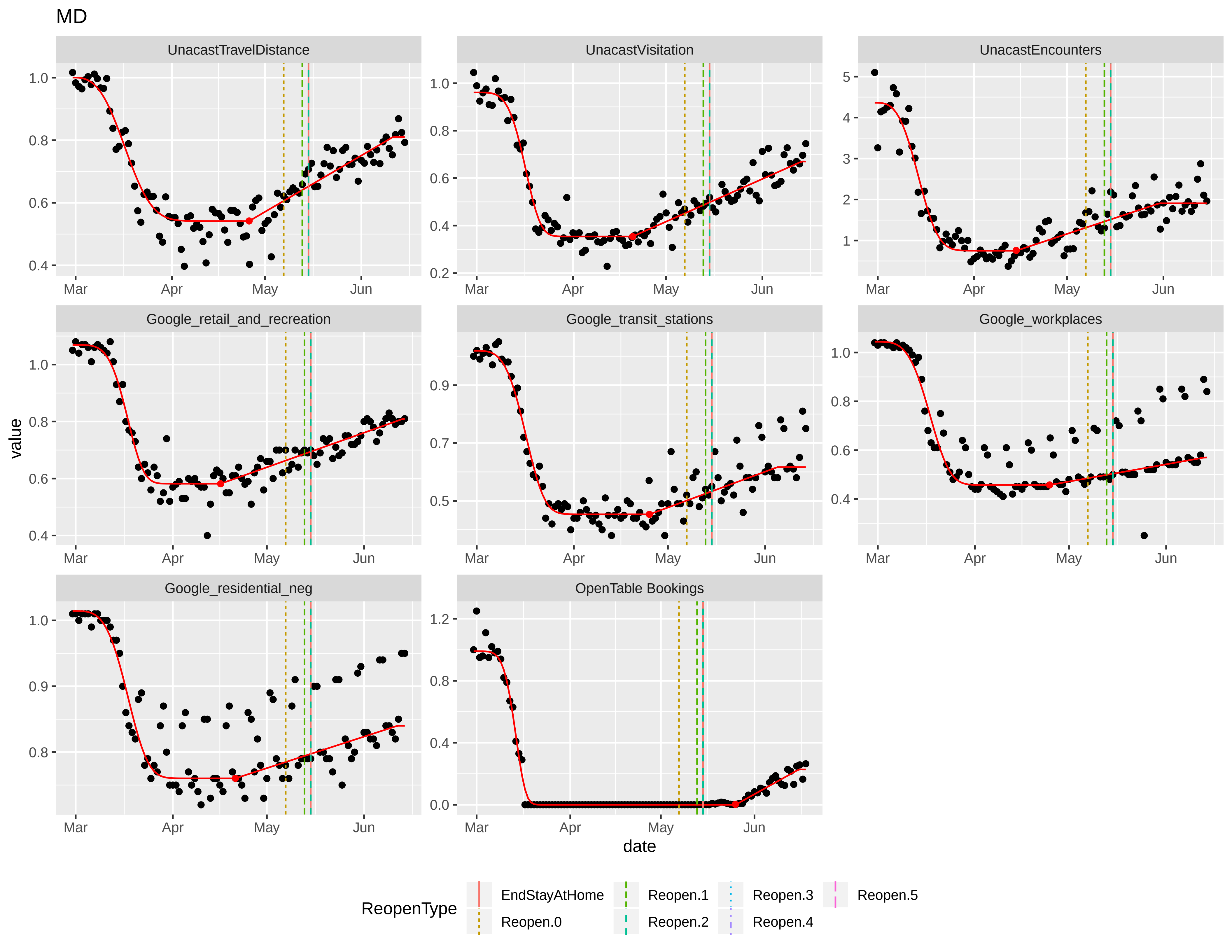
MD
UnacastTravelDistance
UnacastVisitation
UnacastEncounters
Google_retail_and_recreation
Google_transit_stations
Google_workplaces
Google_residential_neg
OpenTable Bookings
value
date
Mar
Apr
May
Jun
ReopenType
EndStayAtHome
Reopen.0
Reopen.1
Reopen.2
Reopen.3
Reopen.4
Reopen.5

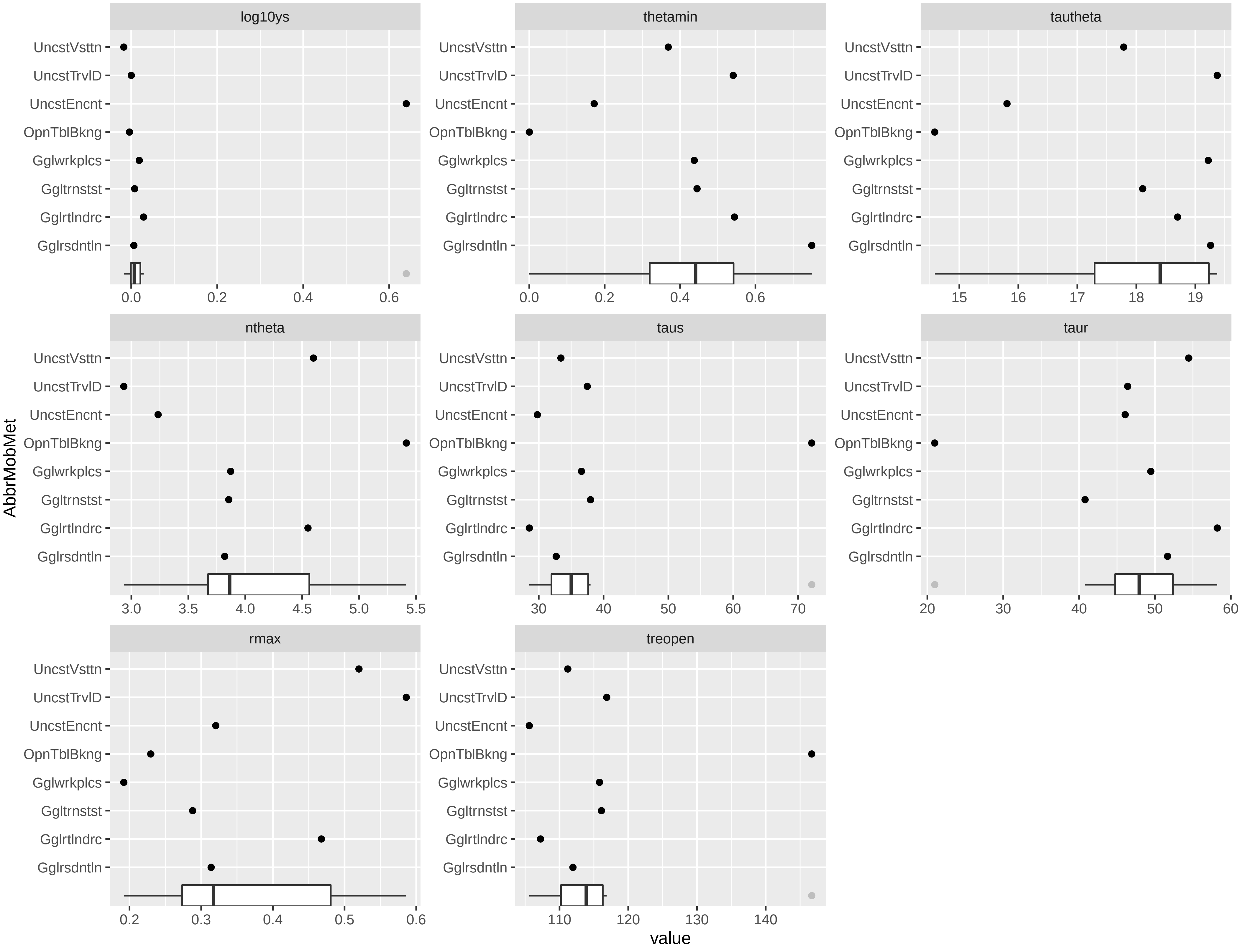

log10ys
thetamin
tautheta
ntheta
taus
taur
rmax
treopen
UncstVsttn
UncstTrvlD
UncstEncnt
OpnTblBkng
Gglwrkplcs
Ggltrnstst
Gglrtlndrc
Gglrsdntln
AbbrMobMet
value

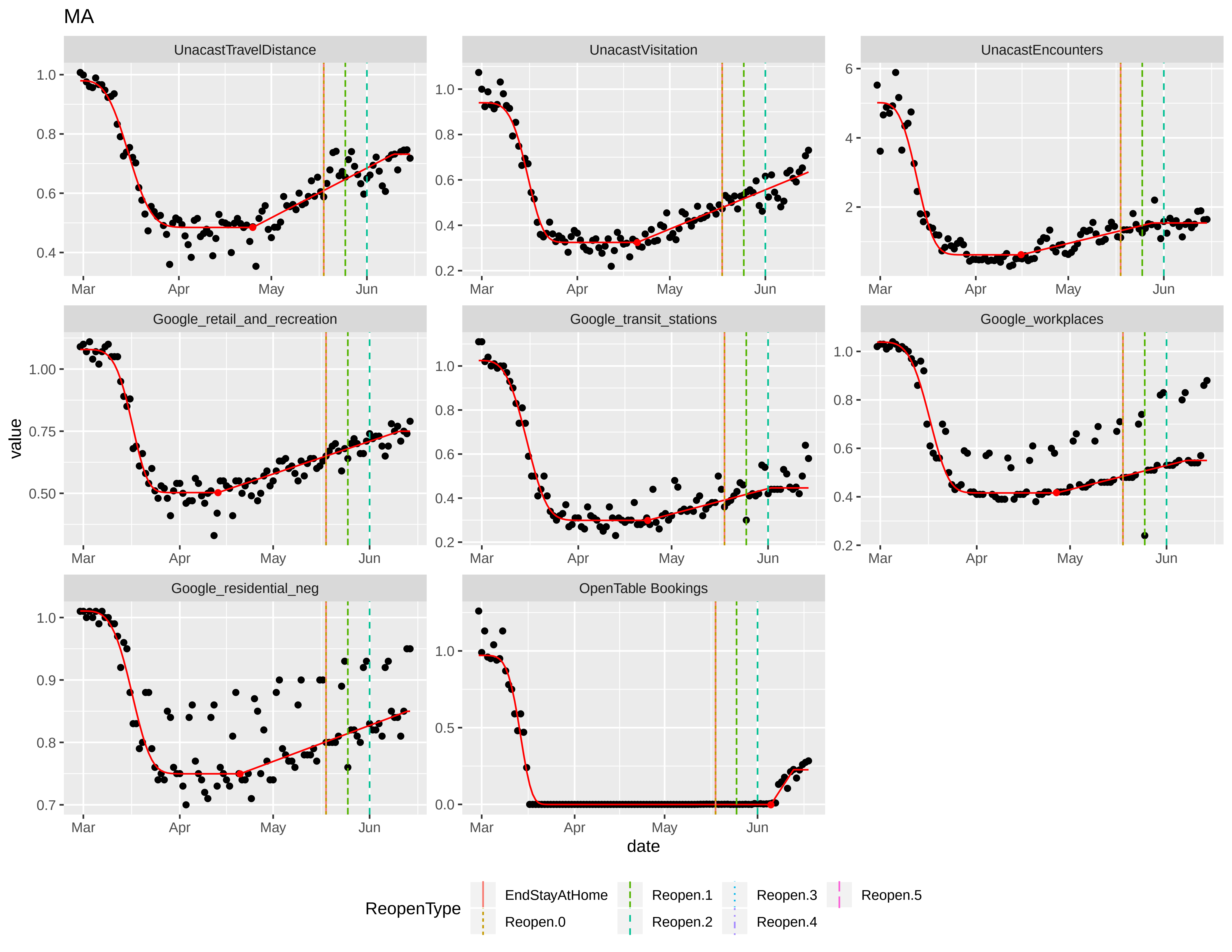

MA
UnacastTravelDistance
UnacastVisitation
UnacastEncounters
Google_retail_and_recreation
Google_transit_stations
Google_workplaces
Google_residential_neg
OpenTable Bookings
value
date
Mar
Apr
May
Jun
ReopenType
EndStayAtHome
Reopen.0
Reopen.1
Reopen.2
Reopen.3
Reopen.4
Reopen.5

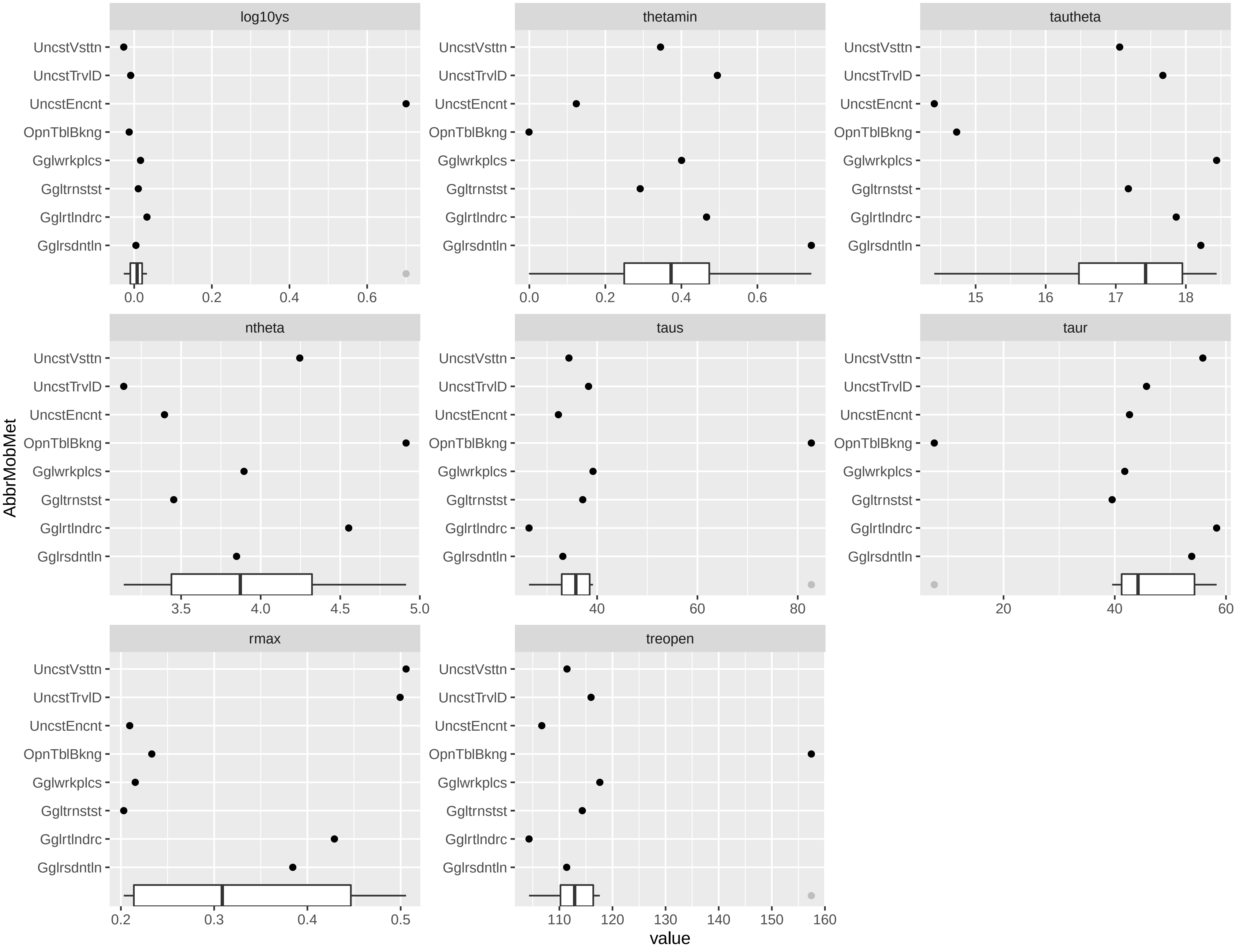
log10ys
thetamin
tautheta
ntheta
taus
taur
rmax
treopen
UncstVsttn
UncstTrvlD
UncstEncnt
OpnTblBkng
Gglwrkplcs
Ggltrnstst
Gglrtlndrc
Gglrsdntln
AbbrMobMet
value
0.0
0.2
0.4
0.6
15
16
17
18
3.5
4.0
4.5
5.0
40
60
80
20
0.3
0.5
110
120
130
140
150
160

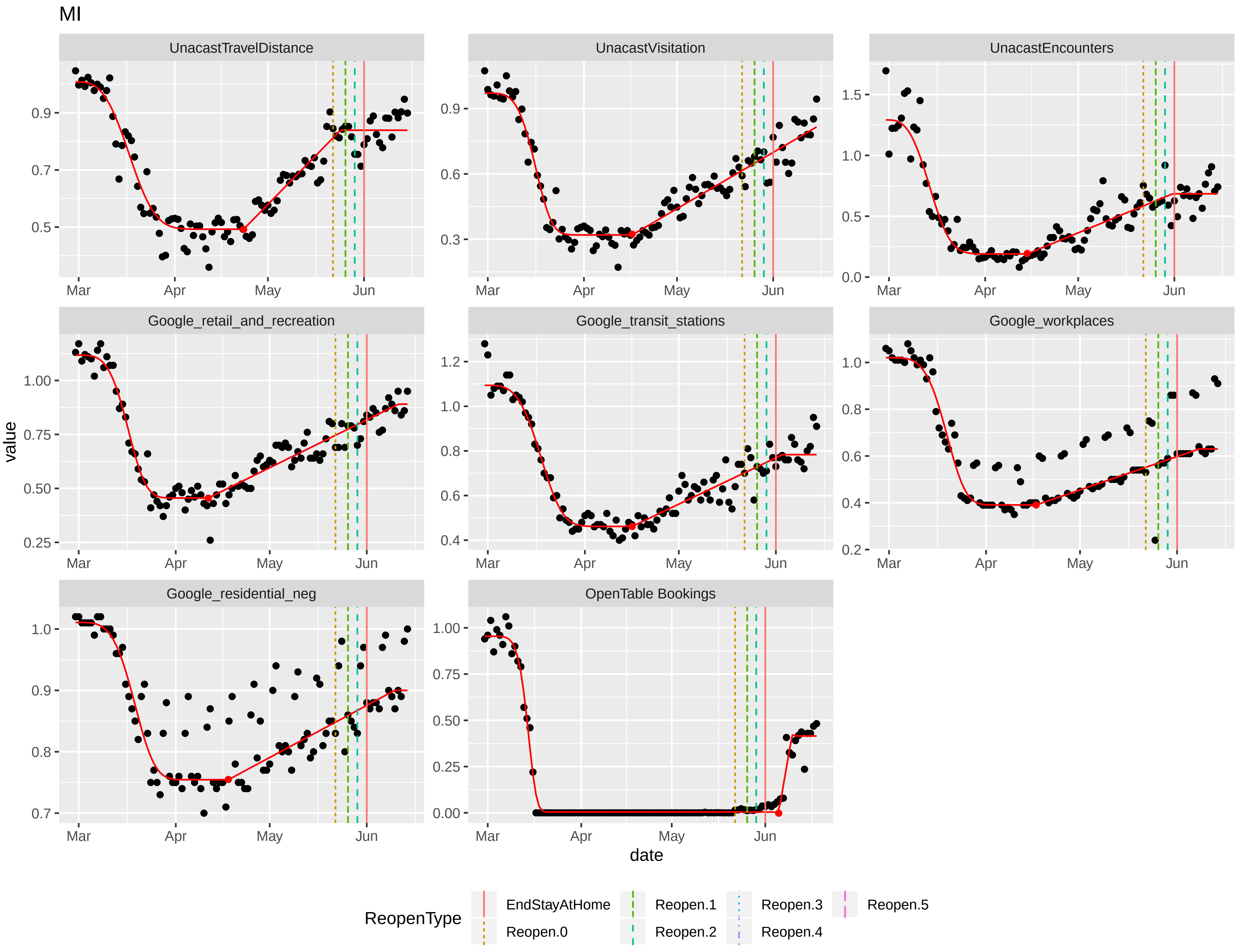
MI
UnacastTravelDistance
UnacastVisitation
UnacastEncounters
Google_retail_and_recreation
Google_transit_stations
Google_workplaces
Google_residential_neg
OpenTable Bookings
value
date
Mar
Apr
May
Jun
ReopenType
EndStayAtHome
Reopen.0
Reopen.1
Reopen.2
Reopen.3
Reopen.4
Reopen.5

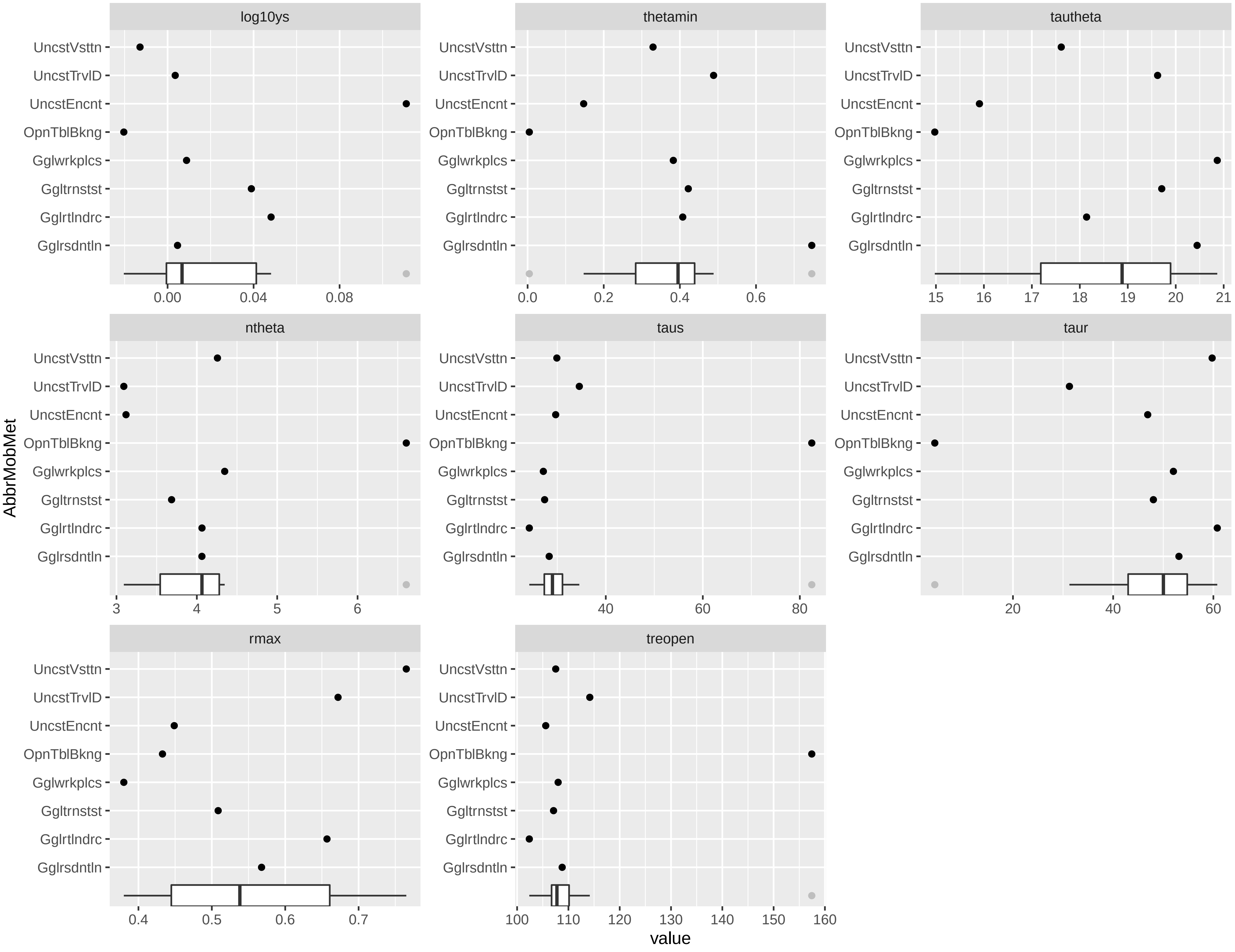
log10ys
thetamin
tautheta
ntheta
taus
taur
rmax
treopen
UncstVsttn
UncstTrvlD
UncstEncnt
OpnTblBkng
Gglwrkplcs
Miglitrnststst
Gglrtlndrc
Gglrsdntln
AbbrMobMet
value
0.00
0.04
0.08
0.0
0.2
0.4
0.6
15
16
17
18
19
20
21
3
4
5
6
40
60
80
20
0.4
0.5
0.6
0.7
100
110
120
130
140
150
160

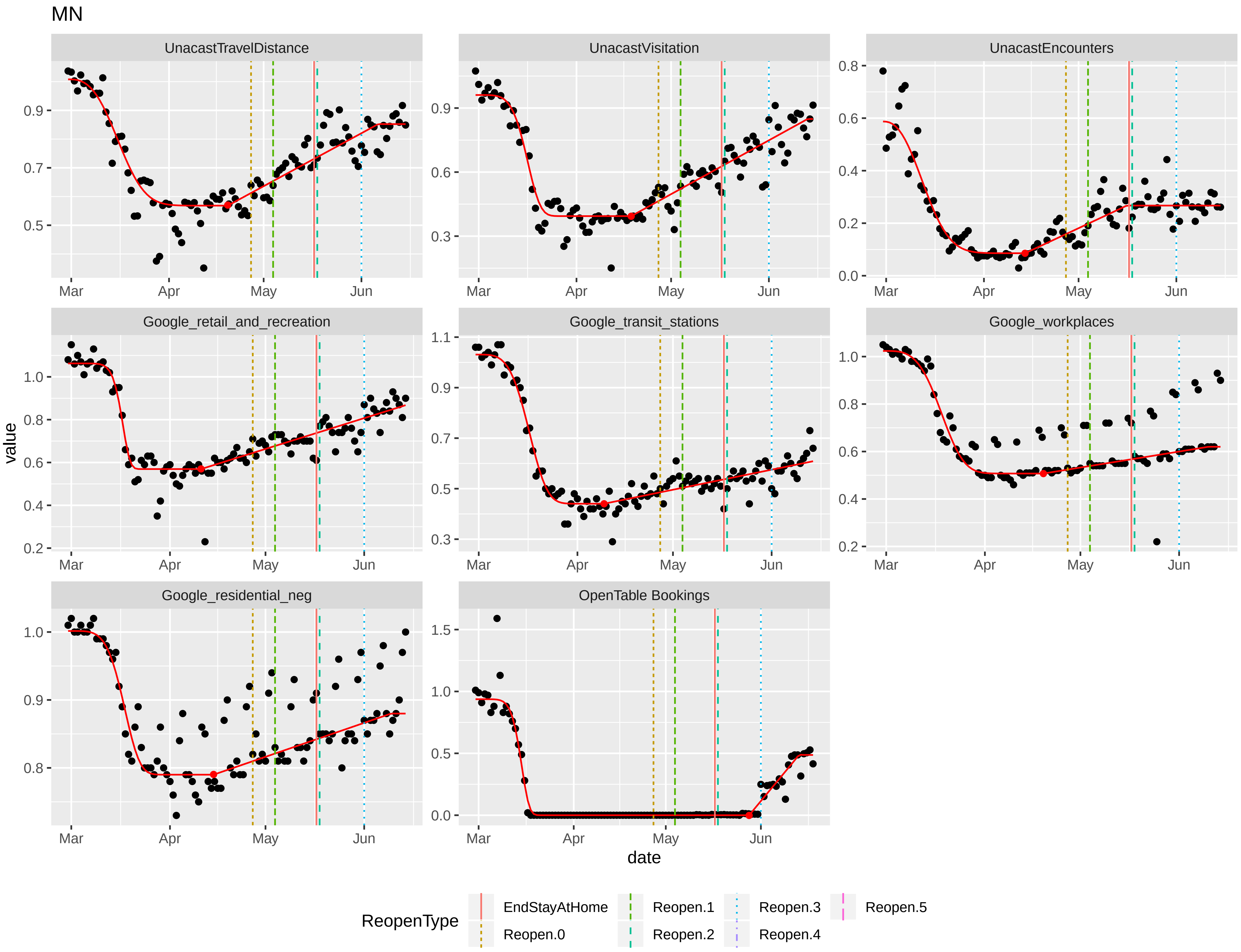

MN
UnacastTravelDistance
UnacastVisitation
UnacastEncounters
Google_retail_and_recreation
Google_transit_stations
Google_workplaces
Google_residential_neg
OpenTable Bookings
value
date
Mar
Apr
May
Jun
ReopenType
EndStayAtHome
Reopen.0
Reopen.1
Reopen.2
Reopen.3
Reopen.4
Reopen.5

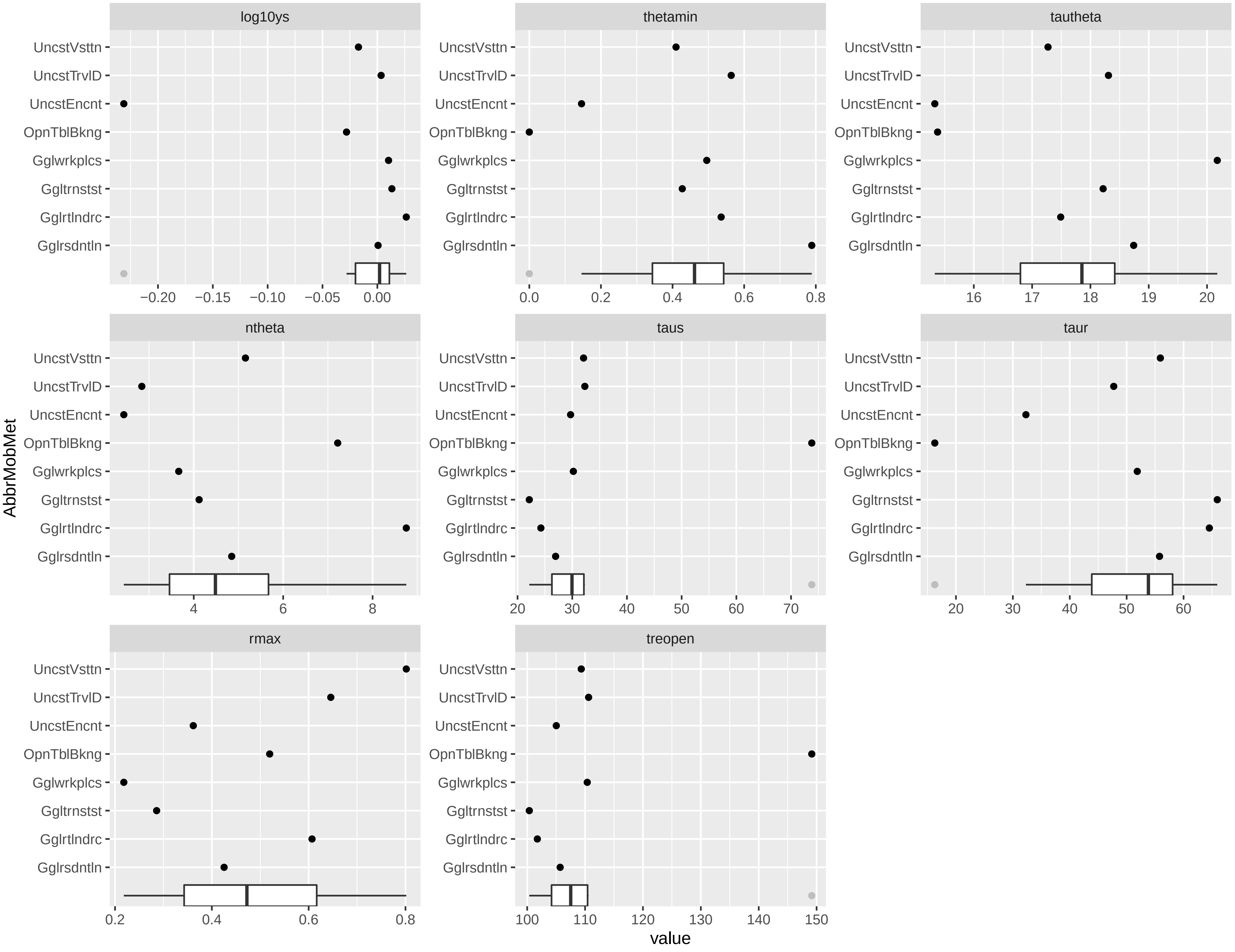

log10ys
thetamin
tautheta
ntheta
taus
taur
rmax
treopen
UncstVsttn
UncstTrvlD
UncstEncnt
OpnTblBkng
Gglwrkplcs
Ggltrnstst
Gglrtlndrc
Gglrsdntln
AbbrMobMet
value

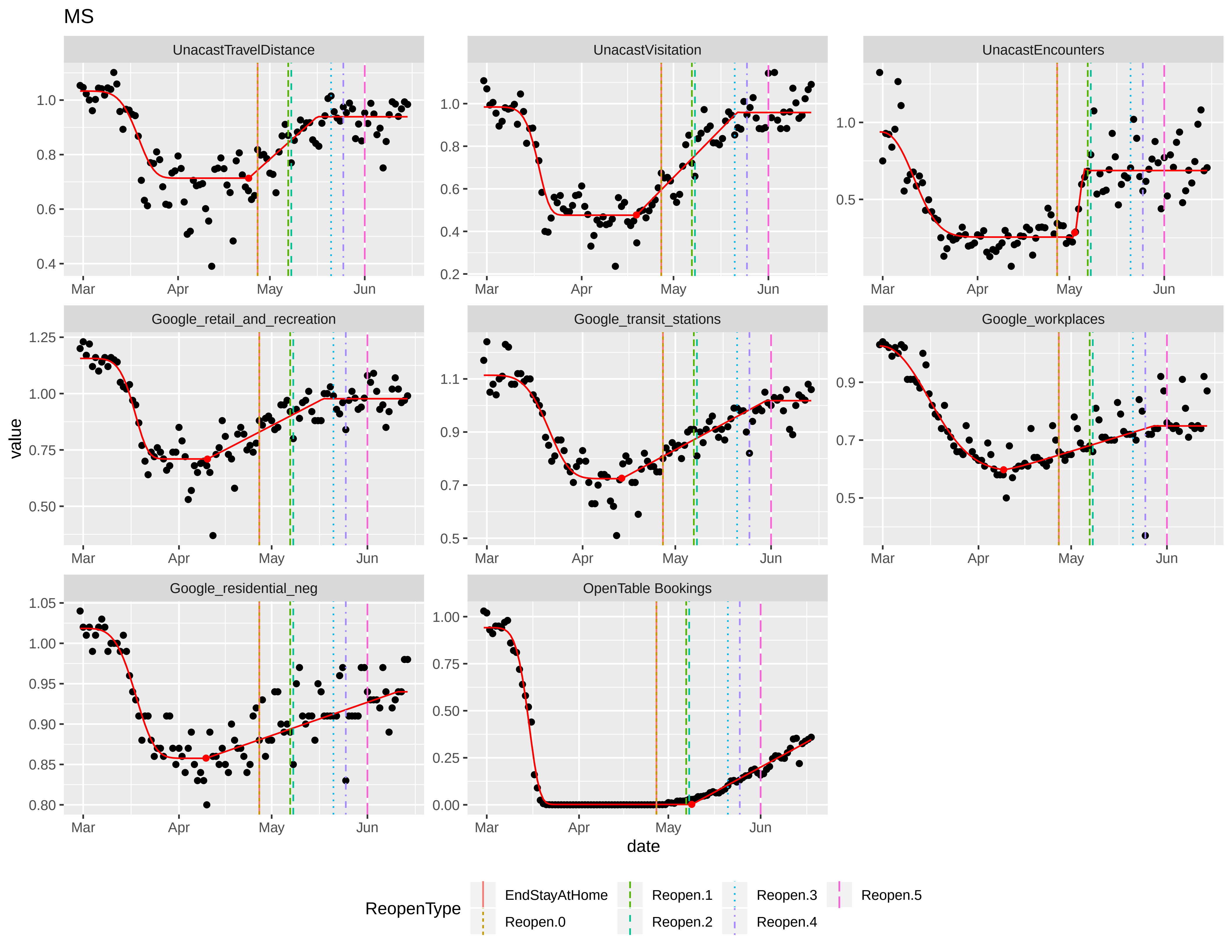
MS
UnacastTravelDistance
UnacastVisitation
UnacastEncounters
Google_retail_and_recreation
Google_transit_stations
Google_workplaces
Google_residential_neg
OpenTable Bookings
value
date
Mar
Apr
May
Jun
ReopenType
EndStayAtHome
Reopen.0
Reopen.1
Reopen.2
Reopen.3
Reopen.4
Reopen.5

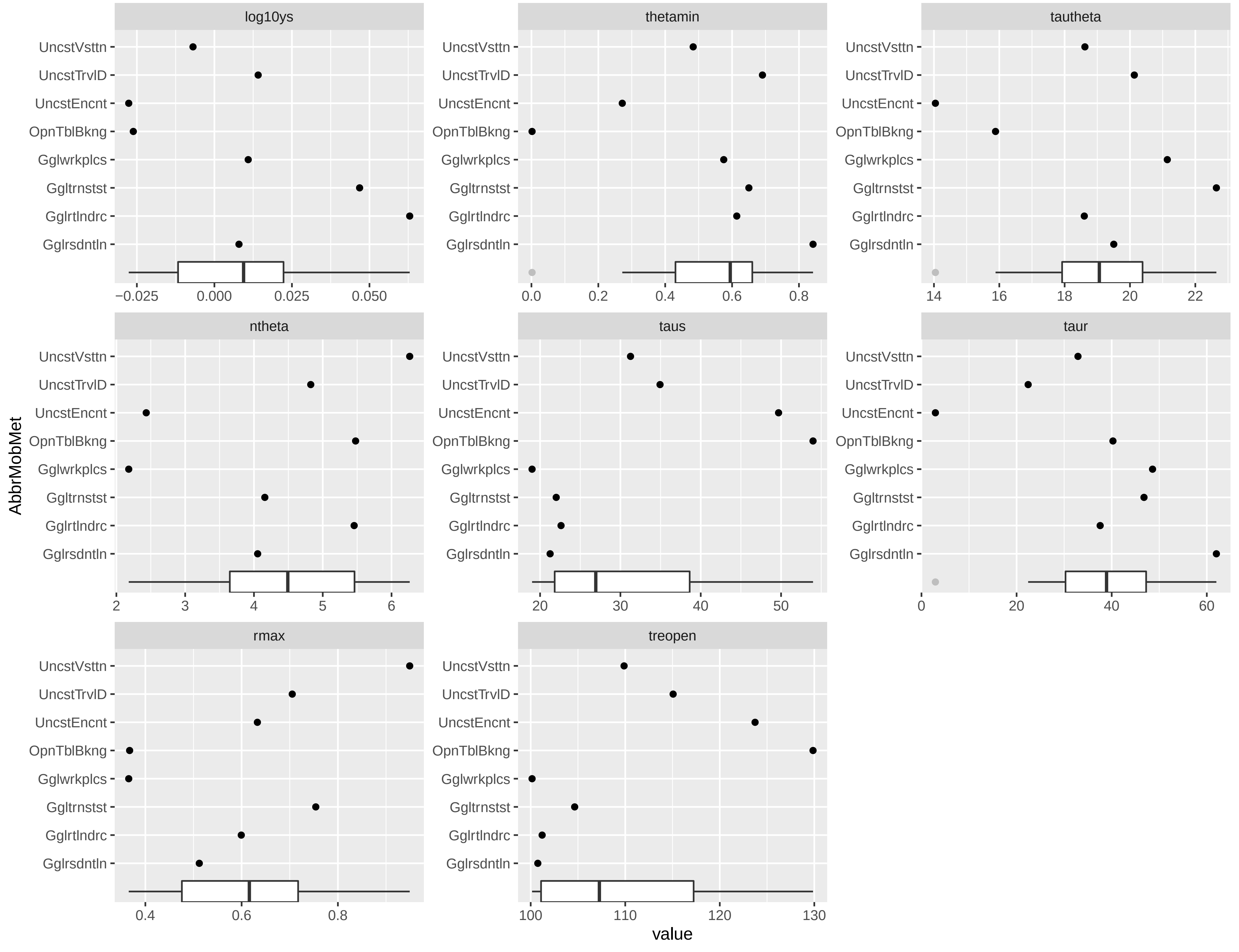

log10ys
thetamin
tautheta
ntheta
taus
taur
rmax
treopen
UncstVsttn
UncstTrvlD
UncstEncnt
OpnTblBkng
Gglwrkplcs
Ggltrnstst
Gglrtlndrc
Gglrsdntln
AbbrMobMet
value

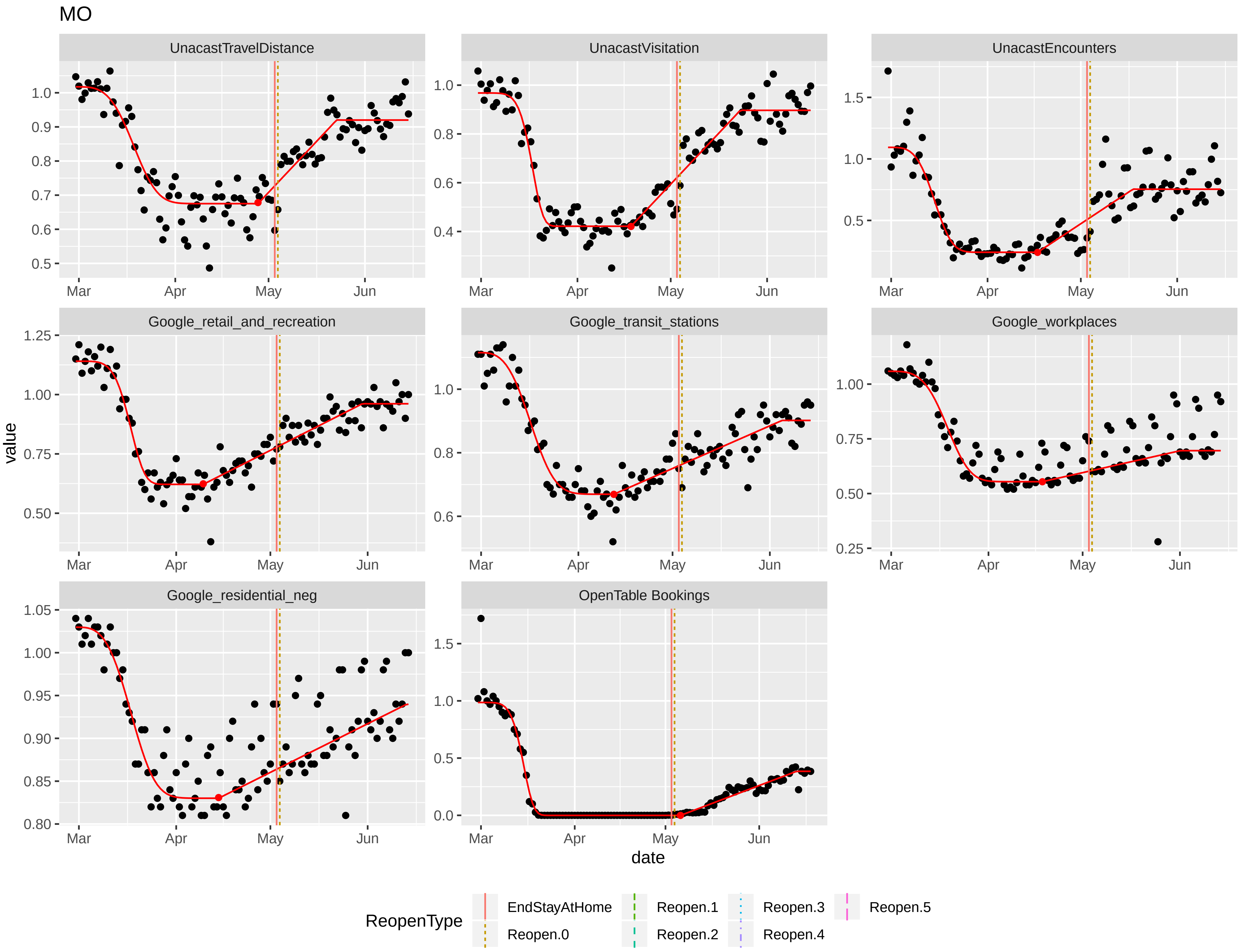
MO
UnacastTravelDistance
UnacastVisitation
UnacastEncounters
Google_retail_and_recreation
Google_transit_stations
Google_workplaces
Google_residential_neg
OpenTable Bookings
value
date
Mar
Apr
May
Jun
ReopenType
EndStayAtHome
Reopen.0
Reopen.1
Reopen.2
Reopen.3
Reopen.4
Reopen.5

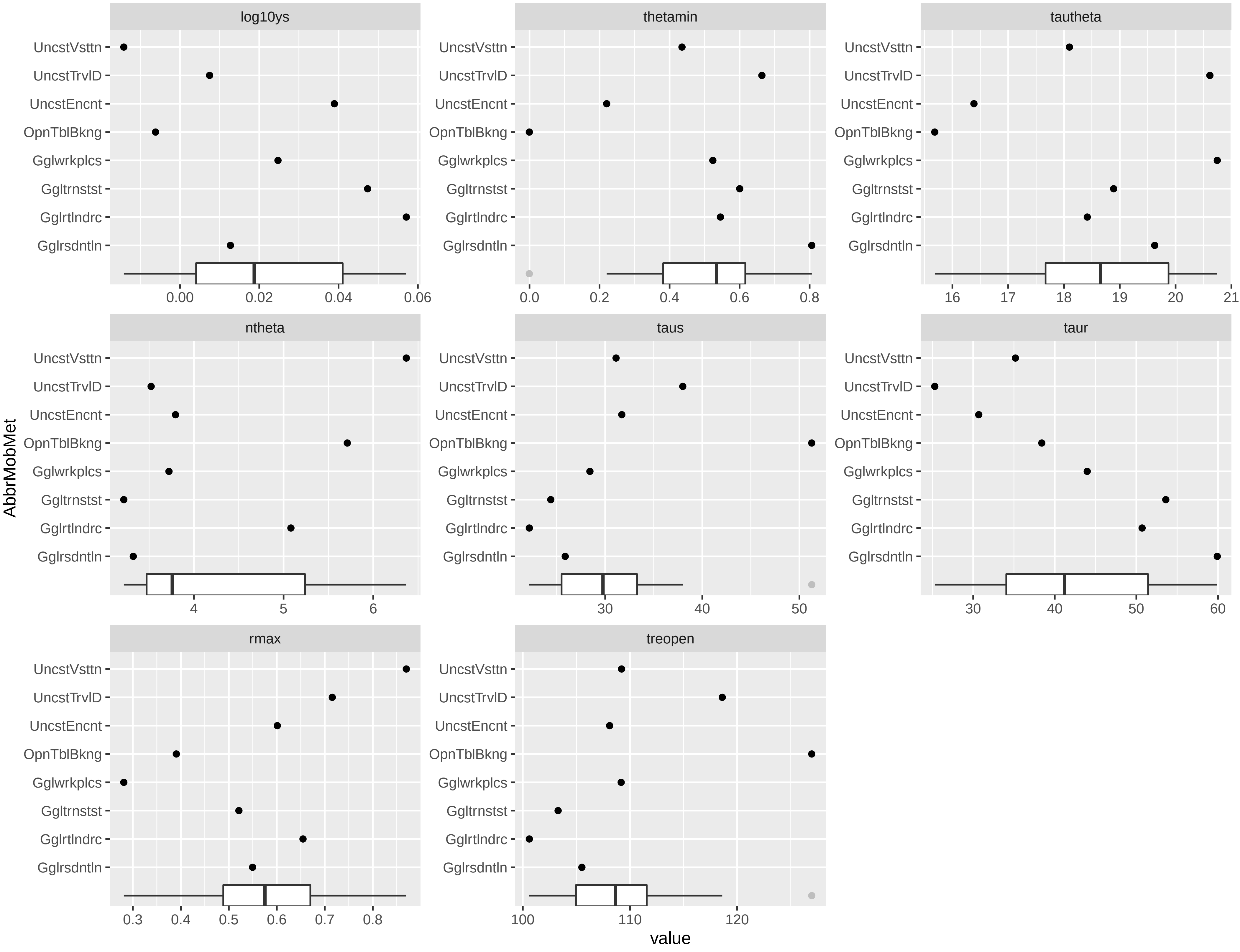

log10ys
thetamin
tautheta
ntheta
taus
taur
rmax
treopen
UncstVsttn
UncstTrvlD
UncstEncnt
OpnTblBkng
Gglwrkplcs
Ggltrnstst
Gglrtlndrc
Gglrsdntln
0.00
0.02
0.04
0.06
0.0
0.2
0.4
0.6
0.8
16
17
18
19
20
21
4
5
6
30
40
50
60
0.3
0.4
0.5
0.6
0.7
0.8
100
110
120
AbbrMobMet
value

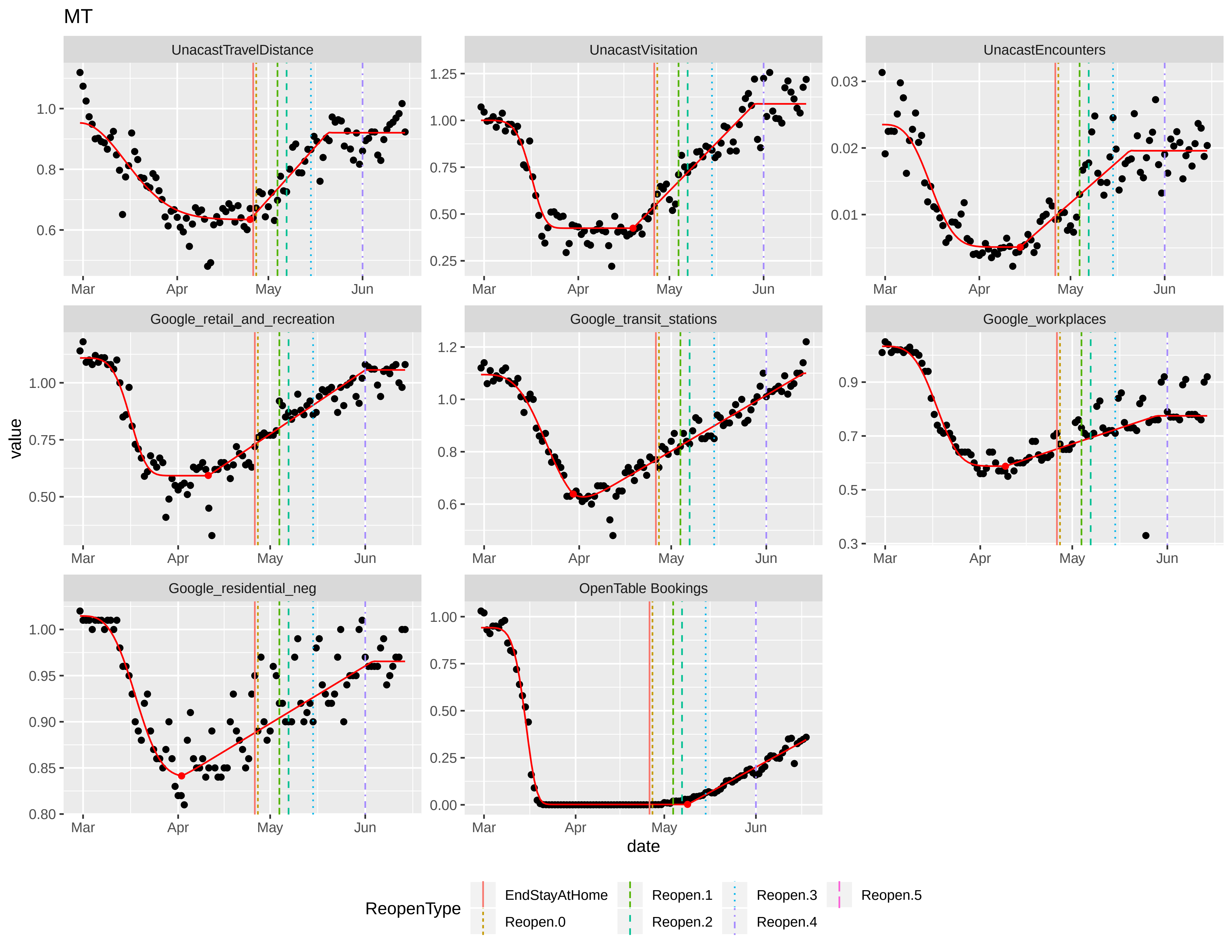

MT
UnacastTravelDistance
UnacastVisitation
UnacastEncounters
Google_retail_and_recreation
Google_transit_stations
Google_workplaces
Google_residential_neg
OpenTable Bookings
value
date
Mar
Apr
May
Jun
ReopenType
EndStayAtHome
Reopen.0
Reopen.1
Reopen.2
Reopen.3
Reopen.4
Reopen.5

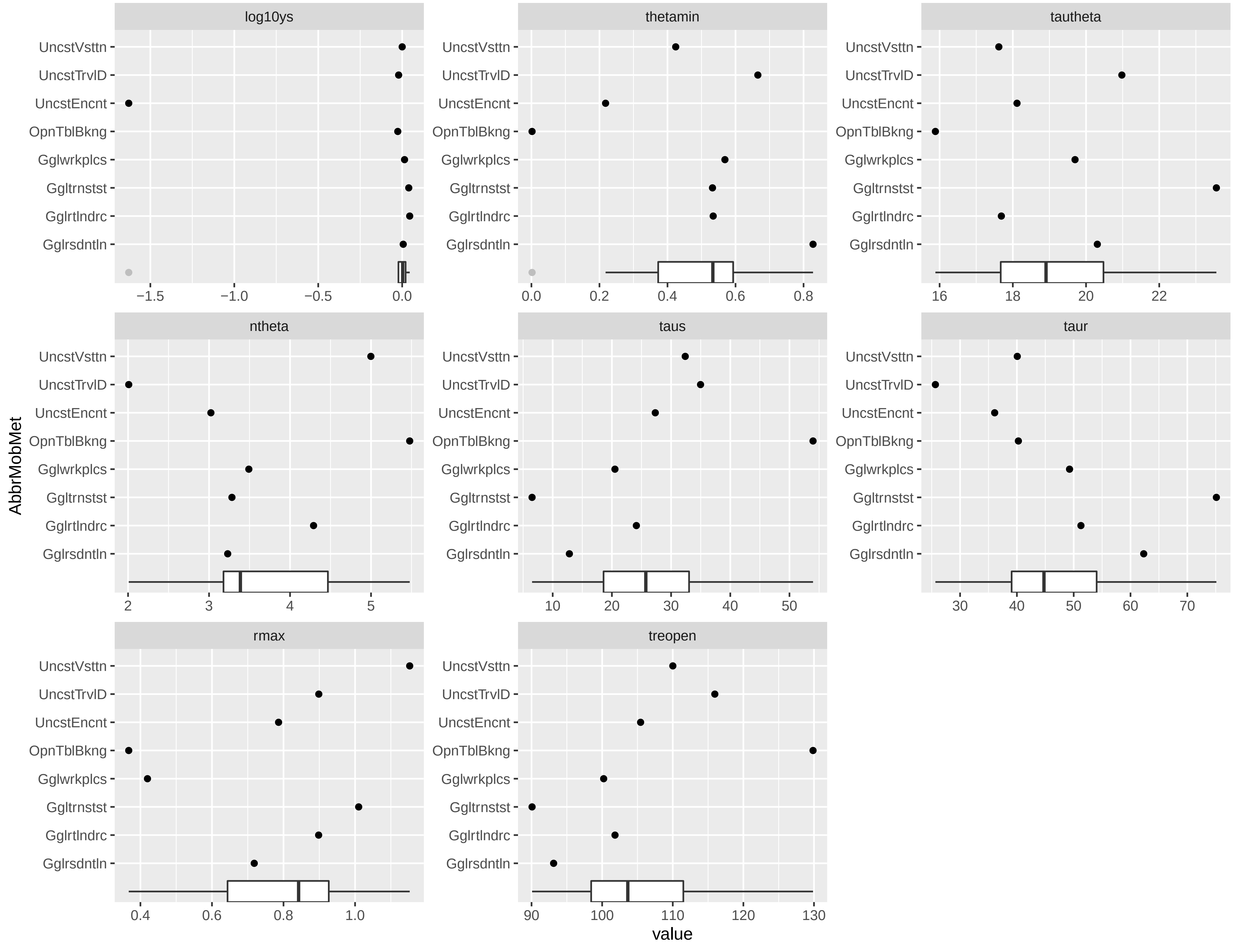
log10ys
thetamin
tautheta
ntheta
taus
taur
rmax
treopen
UncstVsttn
UncstTrvlD
UncstEncnt
OpnTblBkng
Gglwrkplcs
Ggltrnstst
Gglrtlndrc
Gglrsdntln
AbbrMobMet
value

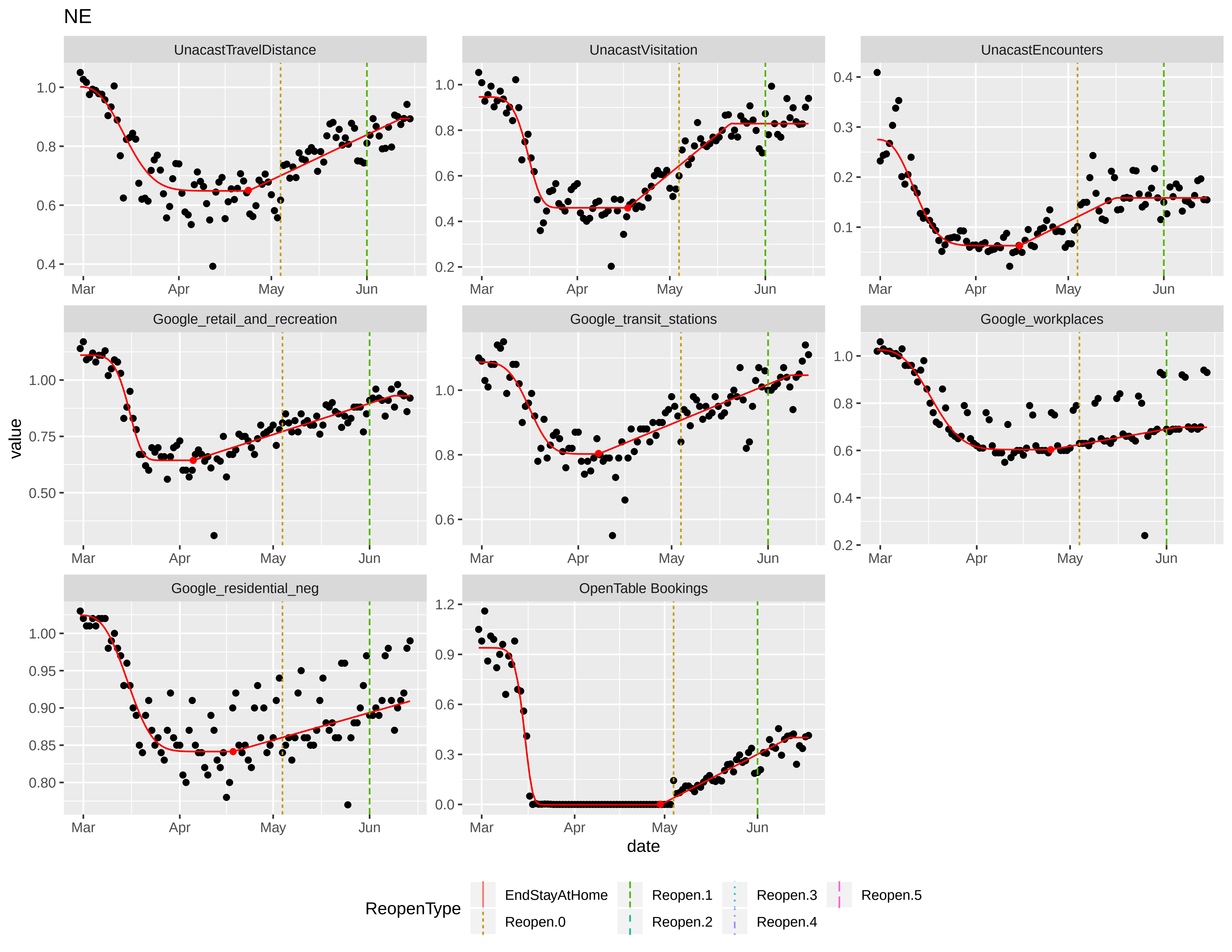

NE
UnacastTravelDistance
UnacastVisitation
UnacastEncounters
Google_retail_and_recreation
Google_transit_stations
Google_workplaces
Google_residential_neg
OpenTable Bookings
value
date
Mar
Apr
May
Jun
ReopenType
EndStayAtHome
Reopen.0
Reopen.1
Reopen.2
Reopen.3
Reopen.4
Reopen.5

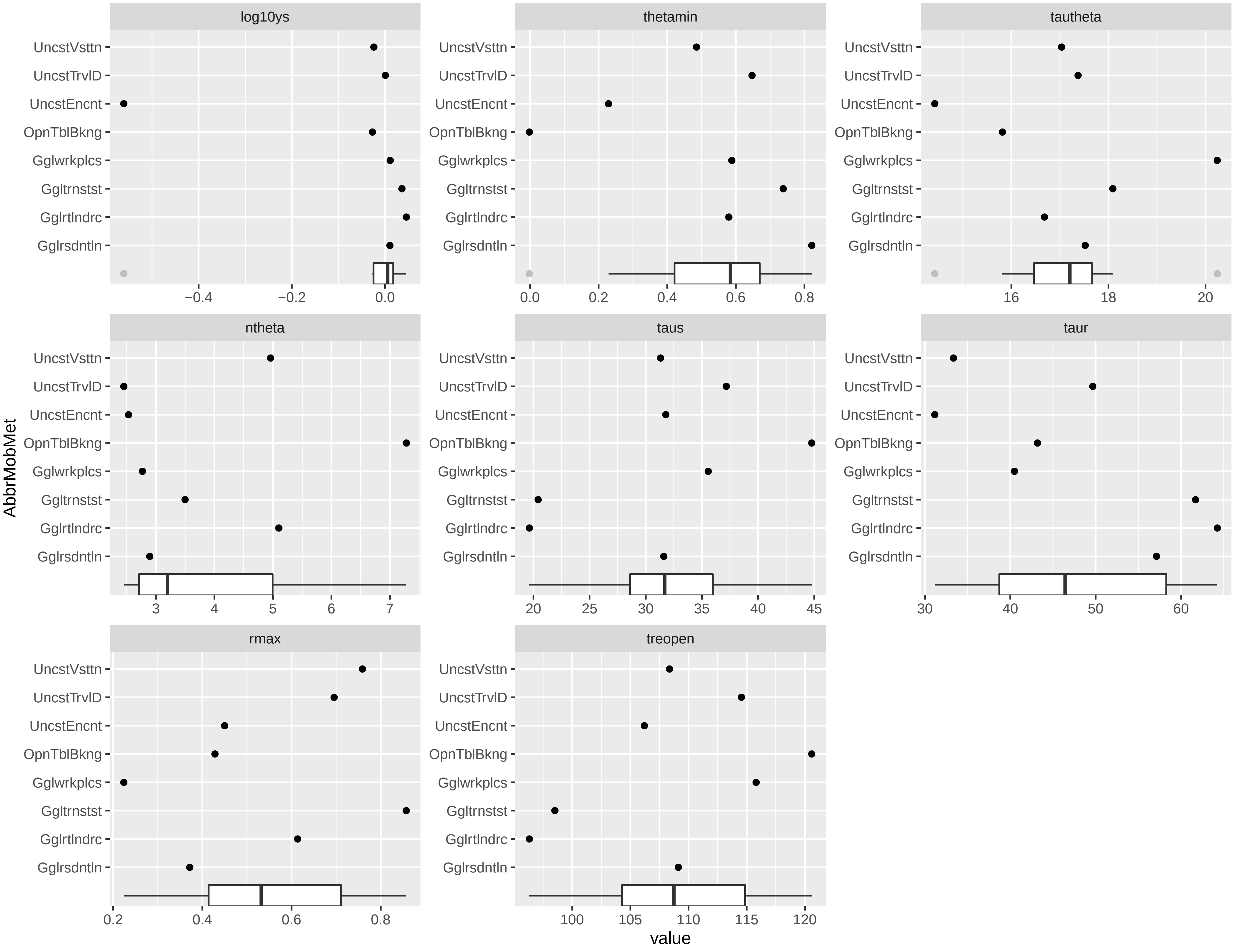
log10ys
thetamin
tautheta
ntheta
taus
taur
rmax
treopen
UncstVsttn
UncstTrvlD
UncstEncnt
OpnTblBkng
Gglwrkplcs
Ggltrnstst
Gglrtlndrc
Gglrsdntln
AbbrMobMet
value

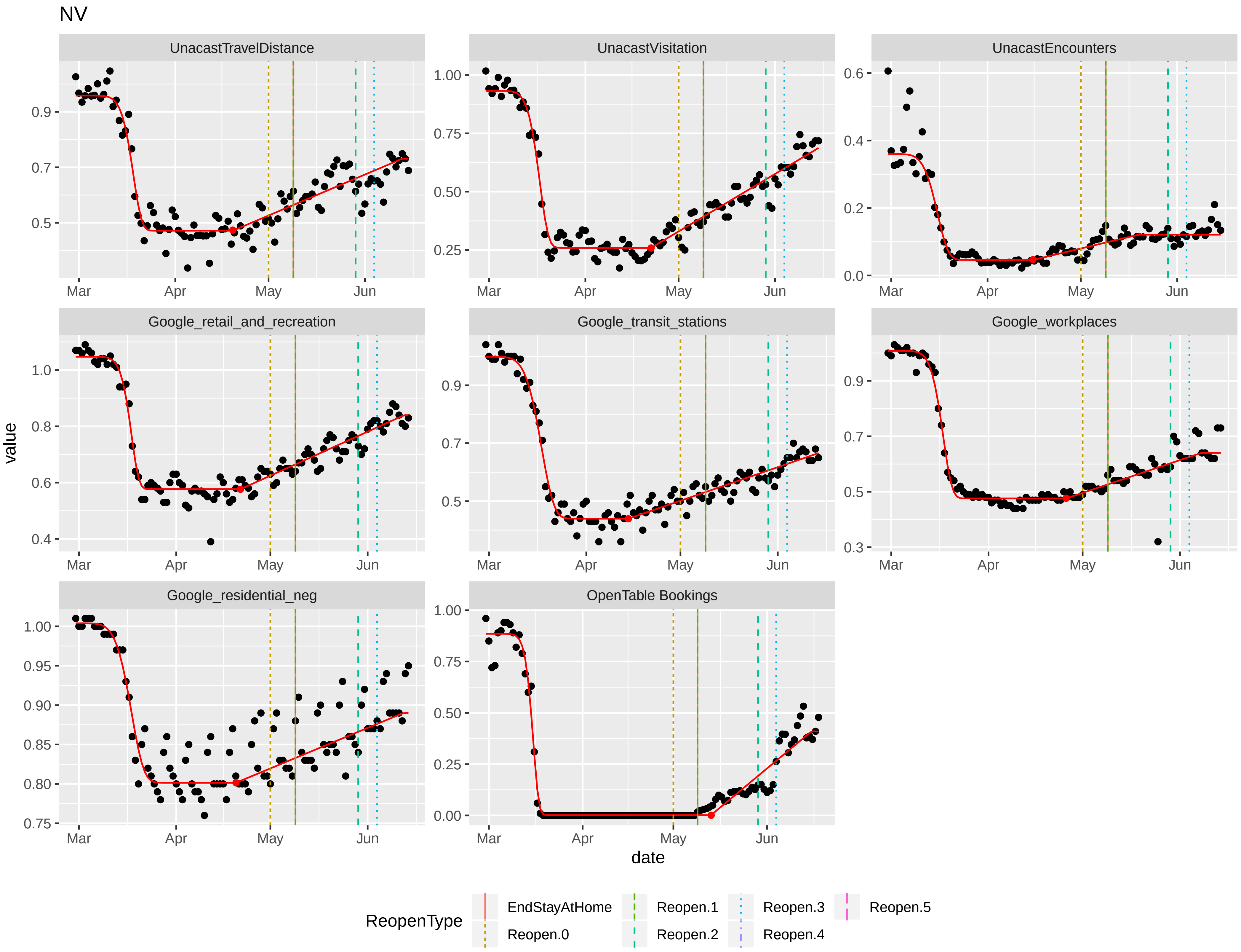
NV
UnacastTravelDistance
UnacastVisitation
UnacastEncounters
Google_retail_and_recreation
Google_transit_stations
Google_workplaces
Google_residential_neg
OpenTable Bookings
value
date
Mar
Apr
May
Jun
ReopenType
EndStayAtHome
Reopen.0
Reopen.1
Reopen.2
Reopen.3
Reopen.4
Reopen.5

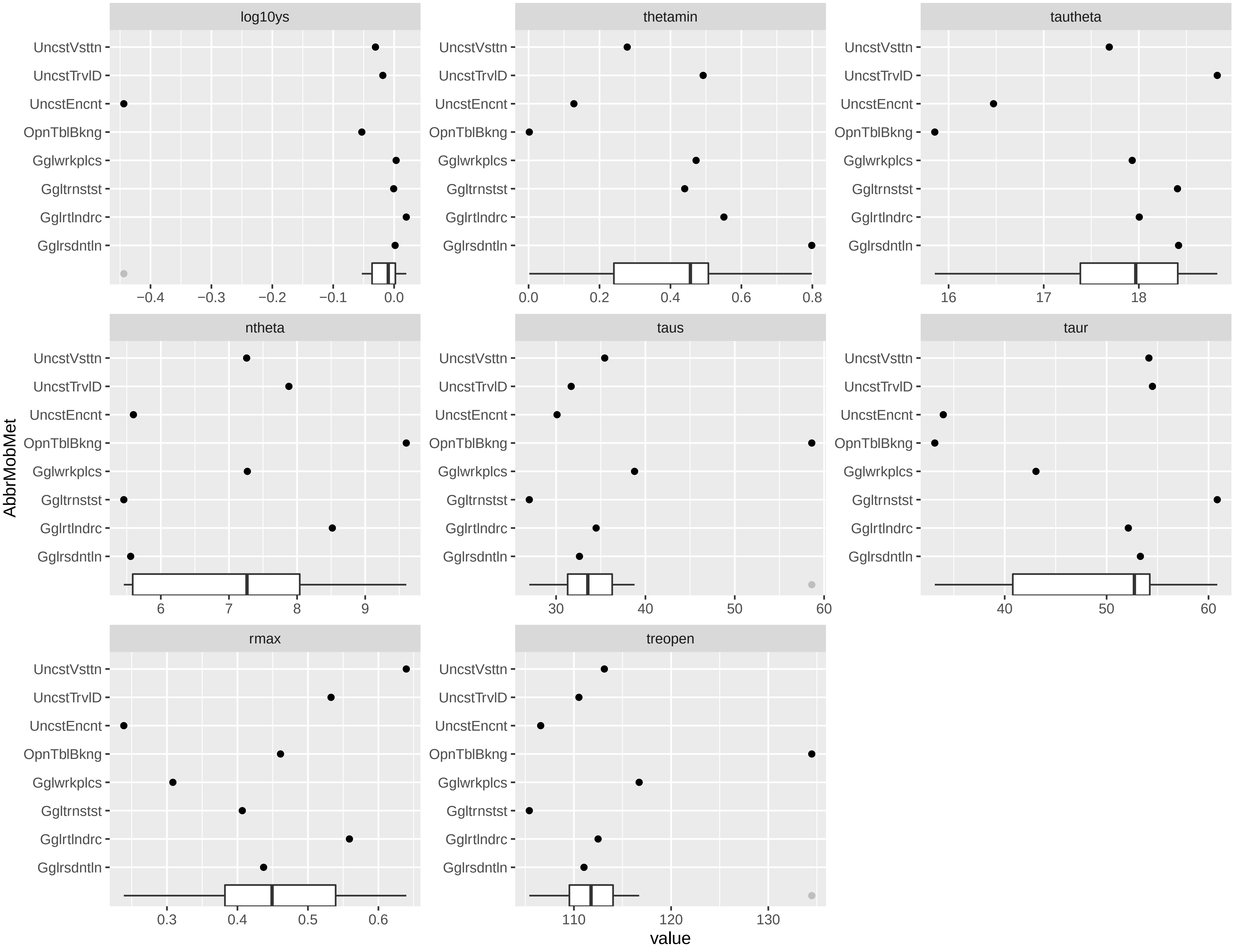

log10ys
thetamin
tautheta
ntheta
taus
taur
rmax
treopen
UncstVsttn
UncstTrvlD
UncstEncnt
OpnTblBkng
Gglwrkplcs
Heltrnstst
Gglrtlndrc
Gglrsdntln
−0.4
−0.3
−0.2
−0.1
0.0
0.2
0.4
0.6
0.8
16
17
18
6
7
8
9
30
40
50
60
0.3
0.5
110
120
130
AbbrMobMet
value

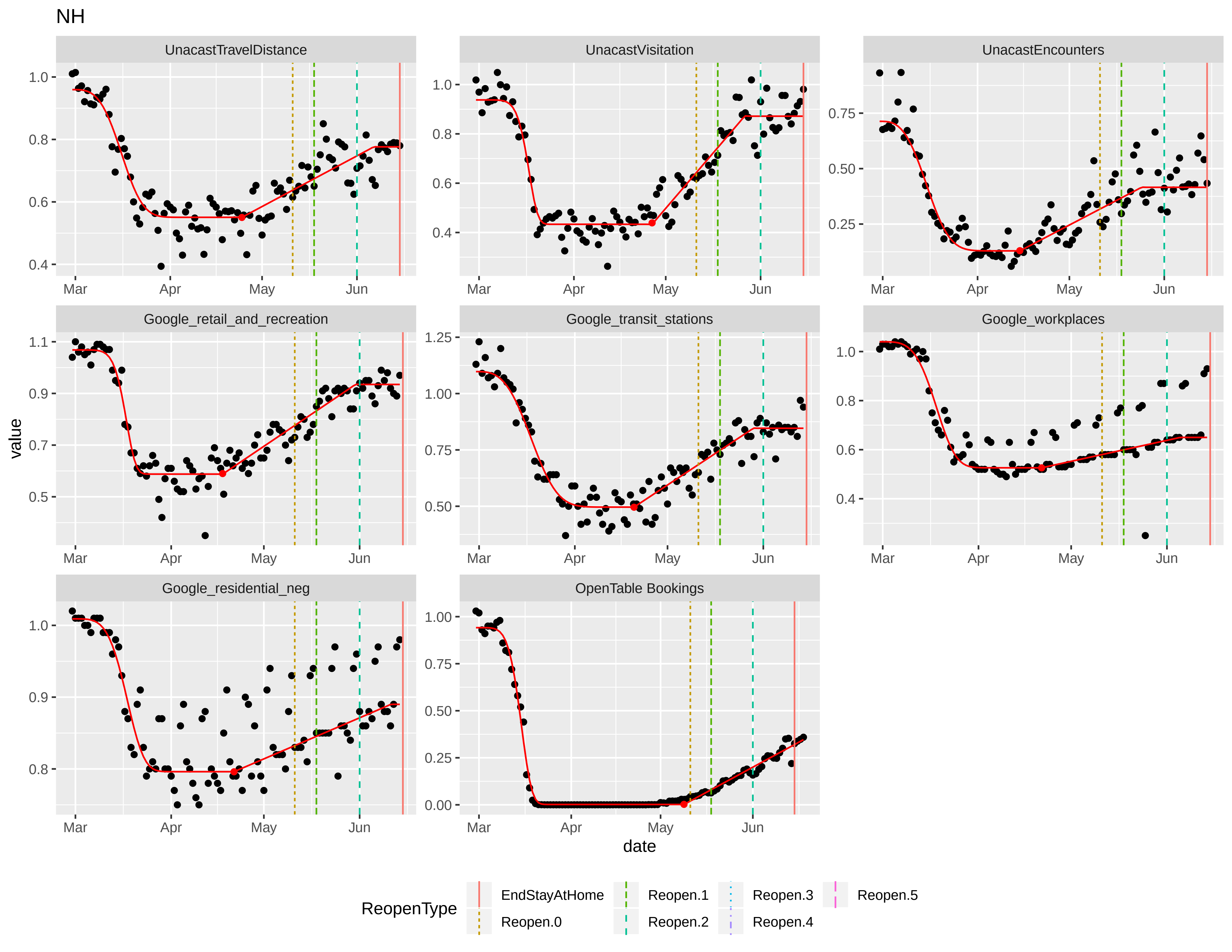

NH
UnacastTravelDistance
UnacastVisitation
UnacastEncounters
Google_retail_and_recreation
Google_transit_stations
Google_workplaces
Google_residential_neg
OpenTable Bookings
value
date
Mar
Apr
May
Jun
ReopenType
EndStayAtHome
Reopen.0
Reopen.1
Reopen.2
Reopen.3
Reopen.4
Reopen.5

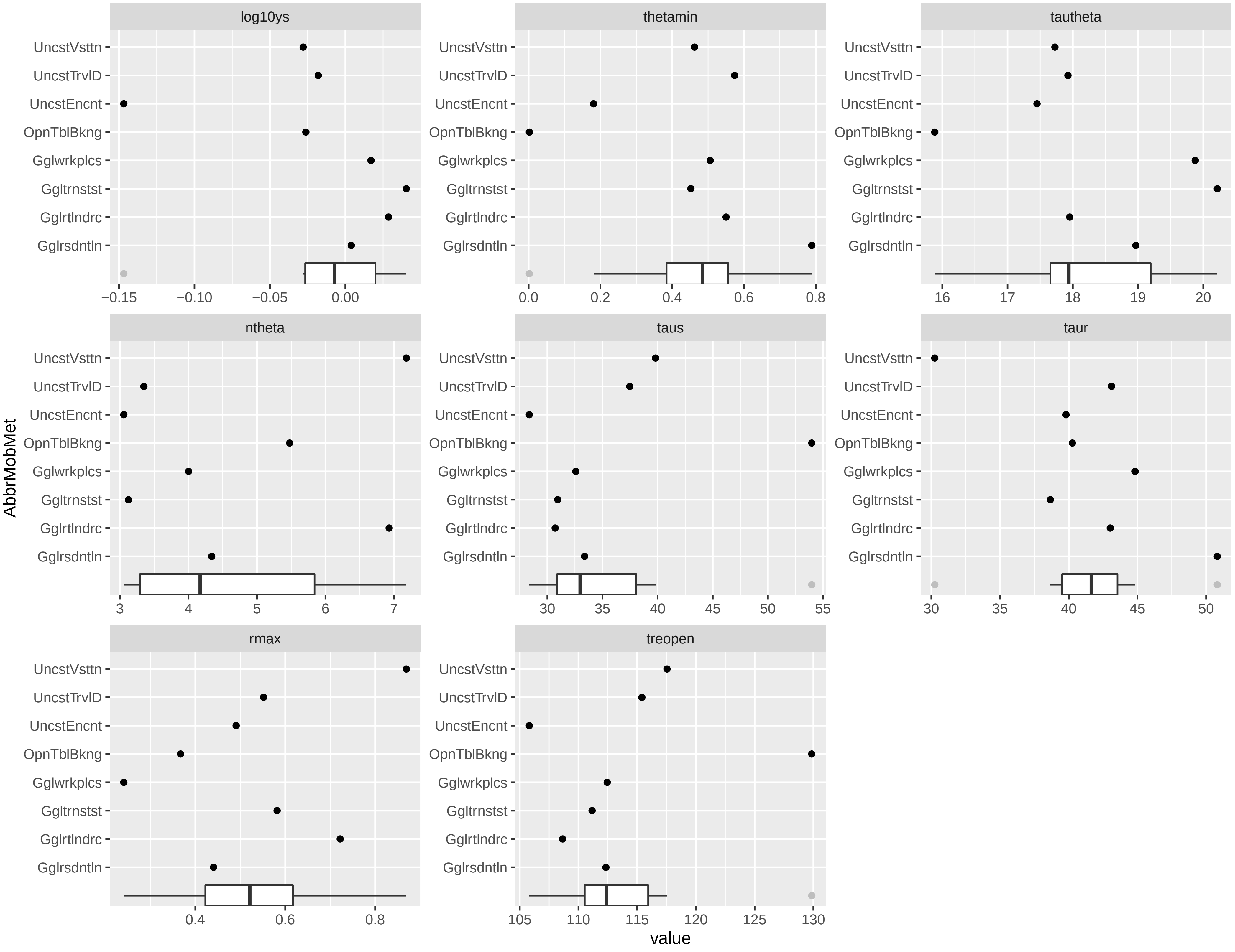

log10ys
thetamin
tautheta
ntheta
taus
taur
rmax
treopen
UncstVsttn
UncstTrvlD
UncstEncnt
OpnTblBkng
Gglwrkplcs
Ggltrnstst
Gglrtlndrc
Gglrsdntln
AbbrMobMet
value

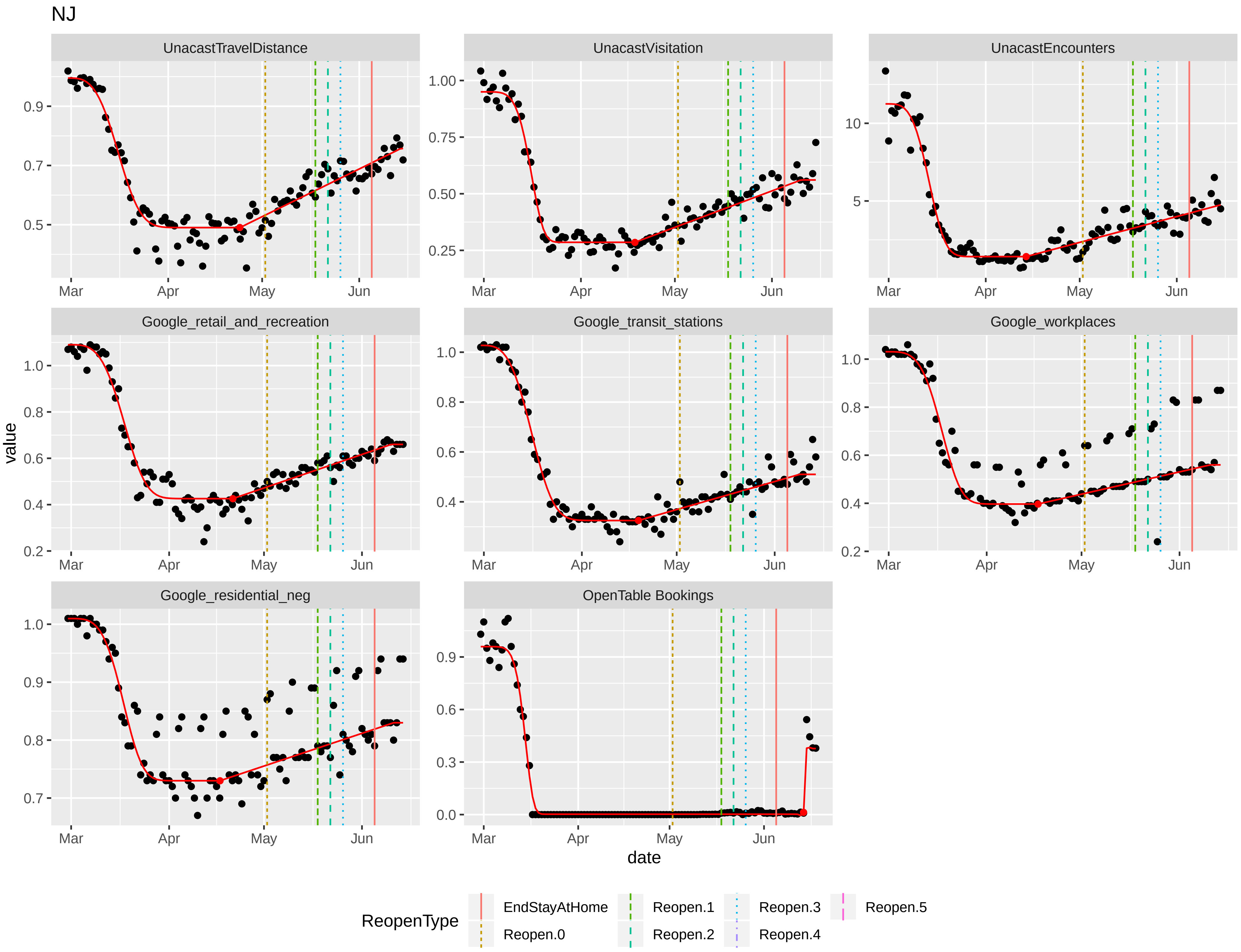

NJ
UnacastTravelDistance
UnacastVisitation
UnacastEncounters
Google_retail_and_recreation
Google_transit_stations
Google_workplaces
Google_residential_neg
OpenTable Bookings
value
date
Mar
Apr
May
Jun
ReopenType
EndStayAtHome
Reopen.0
Reopen.1
Reopen.2
Reopen.3
Reopen.4
Reopen.5

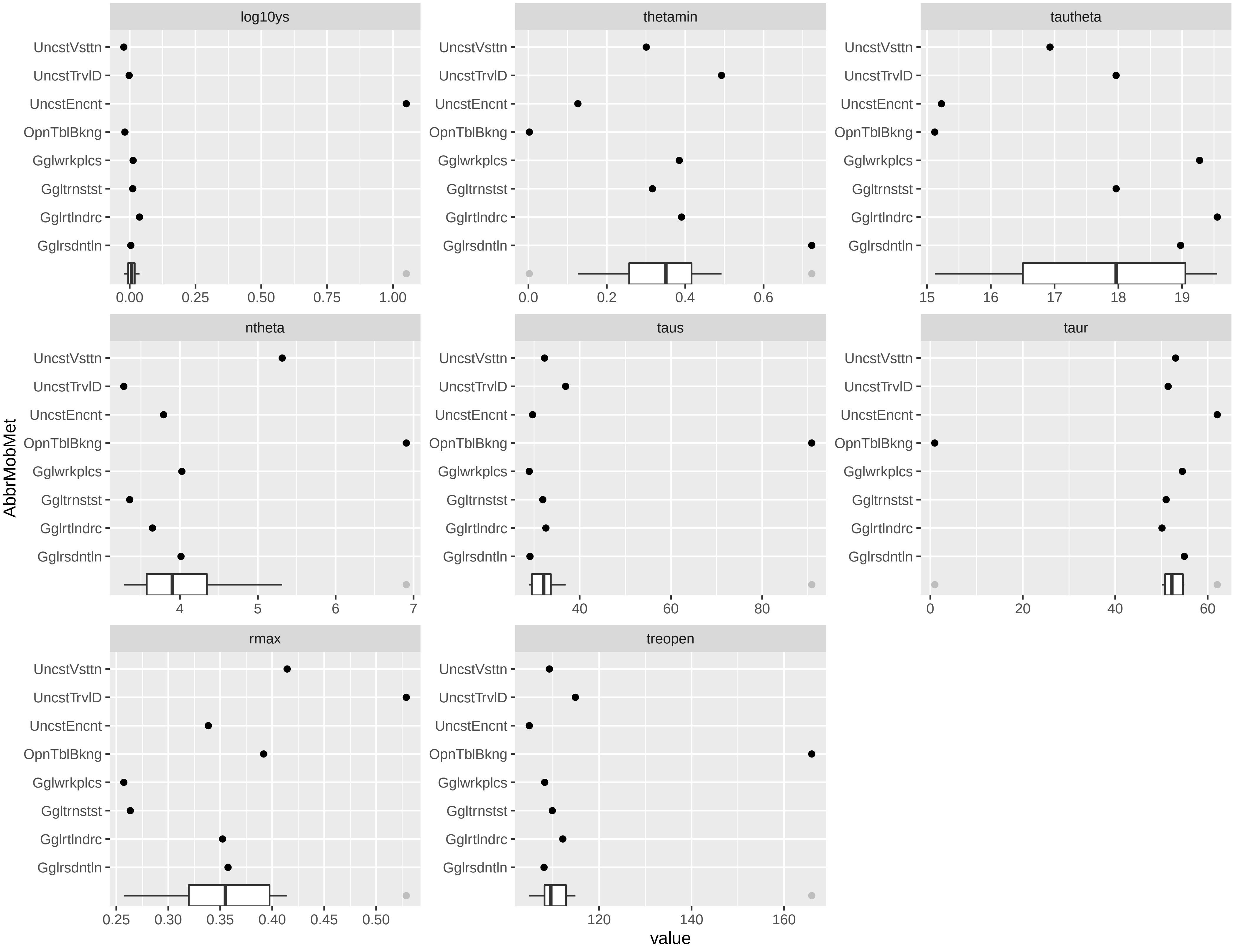

log10ys
thetamin
tautheta
ntheta
taus
taur
rmax
treopen
UncstVsttn
UncstTrvlD
UncstEncnt
OpnTblBkng
Gglwrkplcs
Ggltrnstst
Gglrtlndrc
Gglrsdntln
0.00
0.25
0.50
0.75
1.00
0.0
0.2
0.4
0.6
15
16
17
18
19
4
5
6
7
40
60
80
0
20
40
60
0.25
0.30
0.35
0.40
0.45
0.50
120
140
160
AbbrMobMet
value

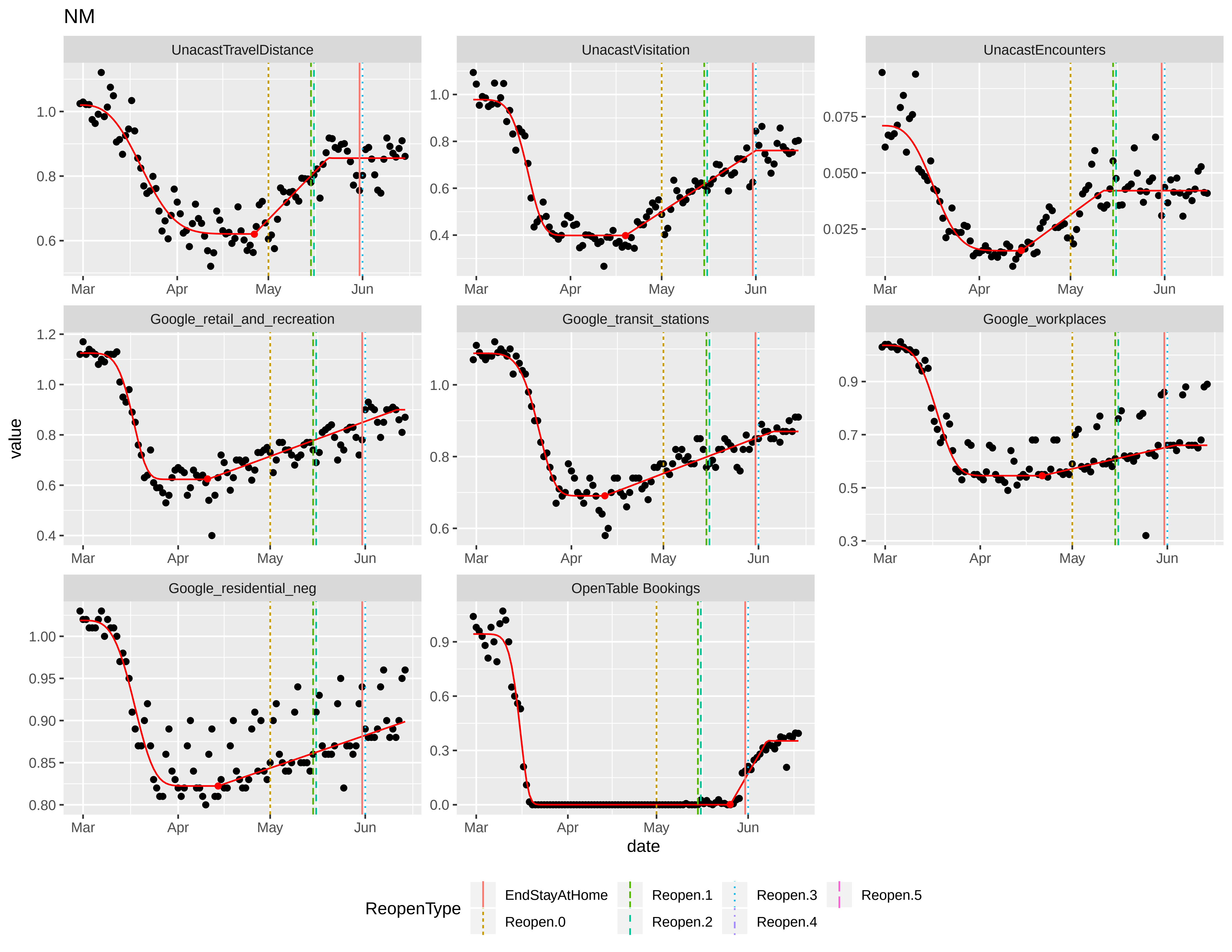
NM
UnacastTravelDistance
UnacastVisitation
UnacastEncounters
Google_retail_and_recreation
Google_transit_stations
Google_workplaces
Google_residential_neg
OpenTable Bookings
value
date
Mar
Apr
May
Jun
ReopenType
EndStayAtHome
Reopen.0
Reopen.1
Reopen.2
Reopen.3
Reopen.4
Reopen.5

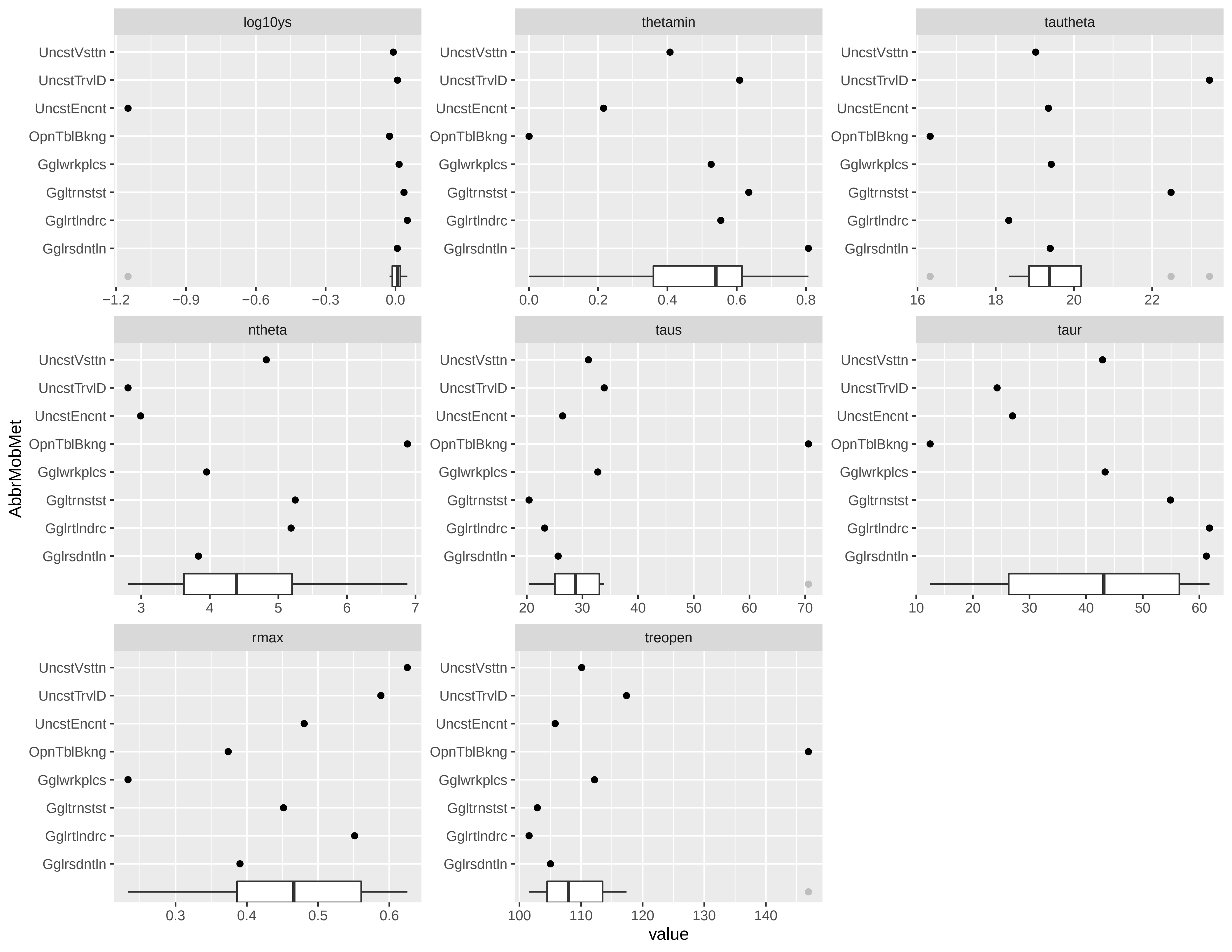

log10ys
thetamin
tautheta
ntheta
taus
taur
rmax
treopen
UncstVsttn
UncstTrvlD
UncstEncnt
OpnTblBkng
Gglwrkplcs
Ggltrnstst
Gglrtlndrc
Gglrsdntln
−1.2
−0.9
−0.6
−0.3
0.0
0.2
0.4
0.6
0.8
16
18
20
22
3
4
5
6
7
10
30
40
50
60
70
0.3
0.5
100
110
120
130
140
AbbrMobMet
value

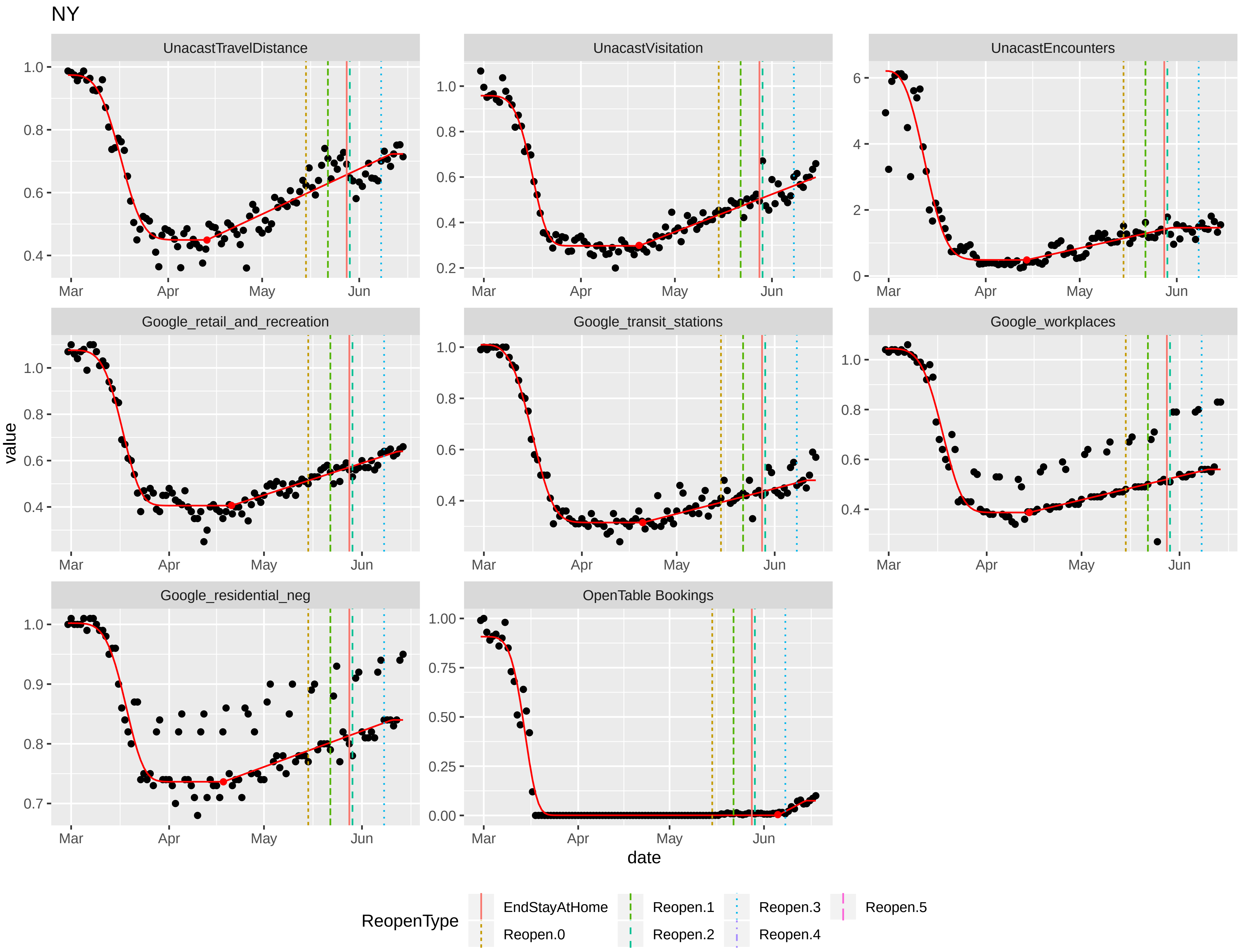

NY
UnacastTravelDistance
UnacastVisitation
UnacastEncounters
Google_retail_and_recreation
Google_transit_stations
Google_workplaces
Google_residential_neg
OpenTable Bookings
value
date
Mar
Apr
May
Jun
ReopenType
EndStayAtHome
Reopen.0
Reopen.1
Reopen.2
Reopen.3
Reopen.4
Reopen.5

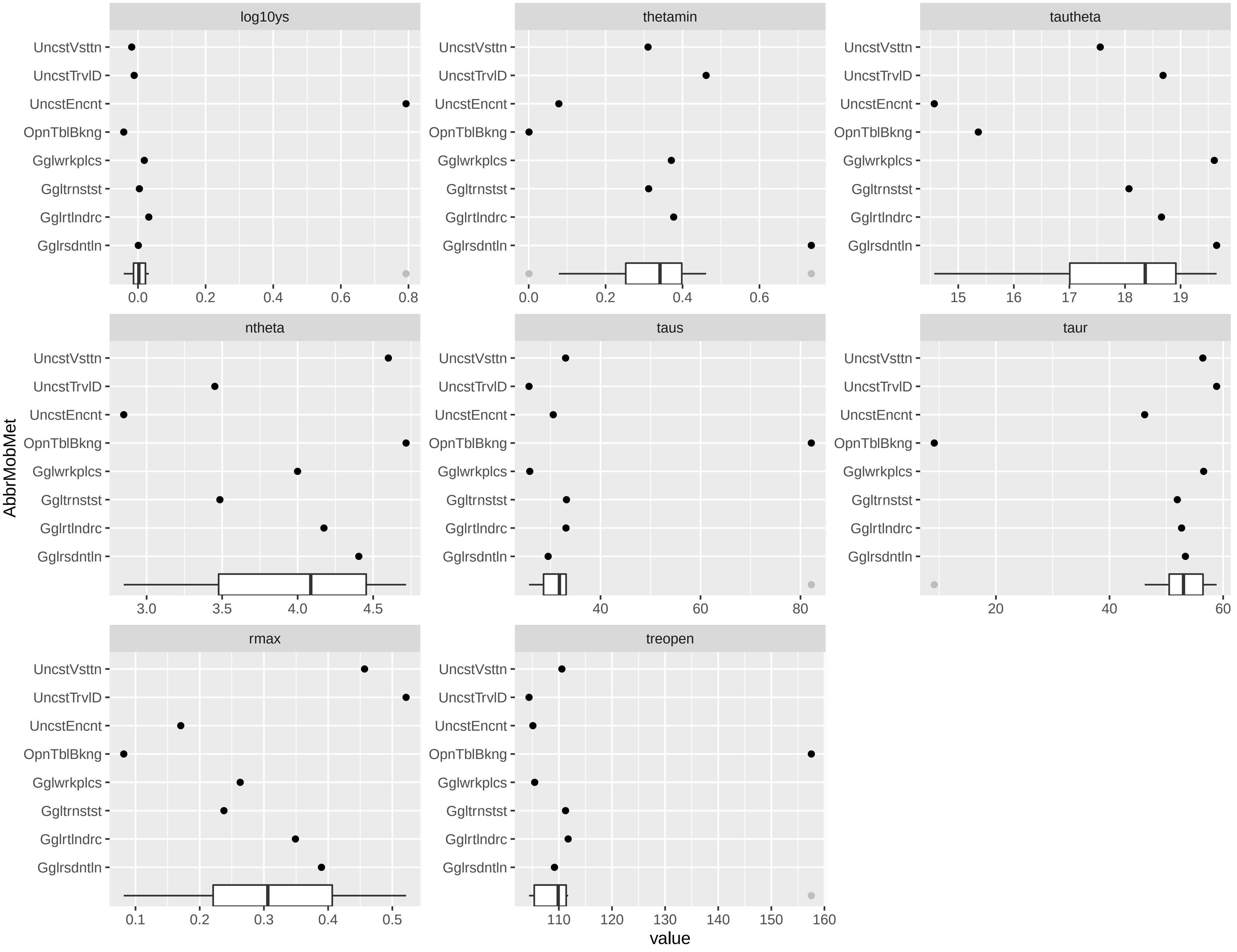

log10ys
thetamin
tautheta
ntheta
taus
taur
rmax
treopen
UncstVsttn
UncstTrvlD
UncstEncnt
OpnTblBkng
Gglwrkplcs
Buffltrnstst
Gglrtlndrc
Gglrsdntln
AbbrMobMet
value

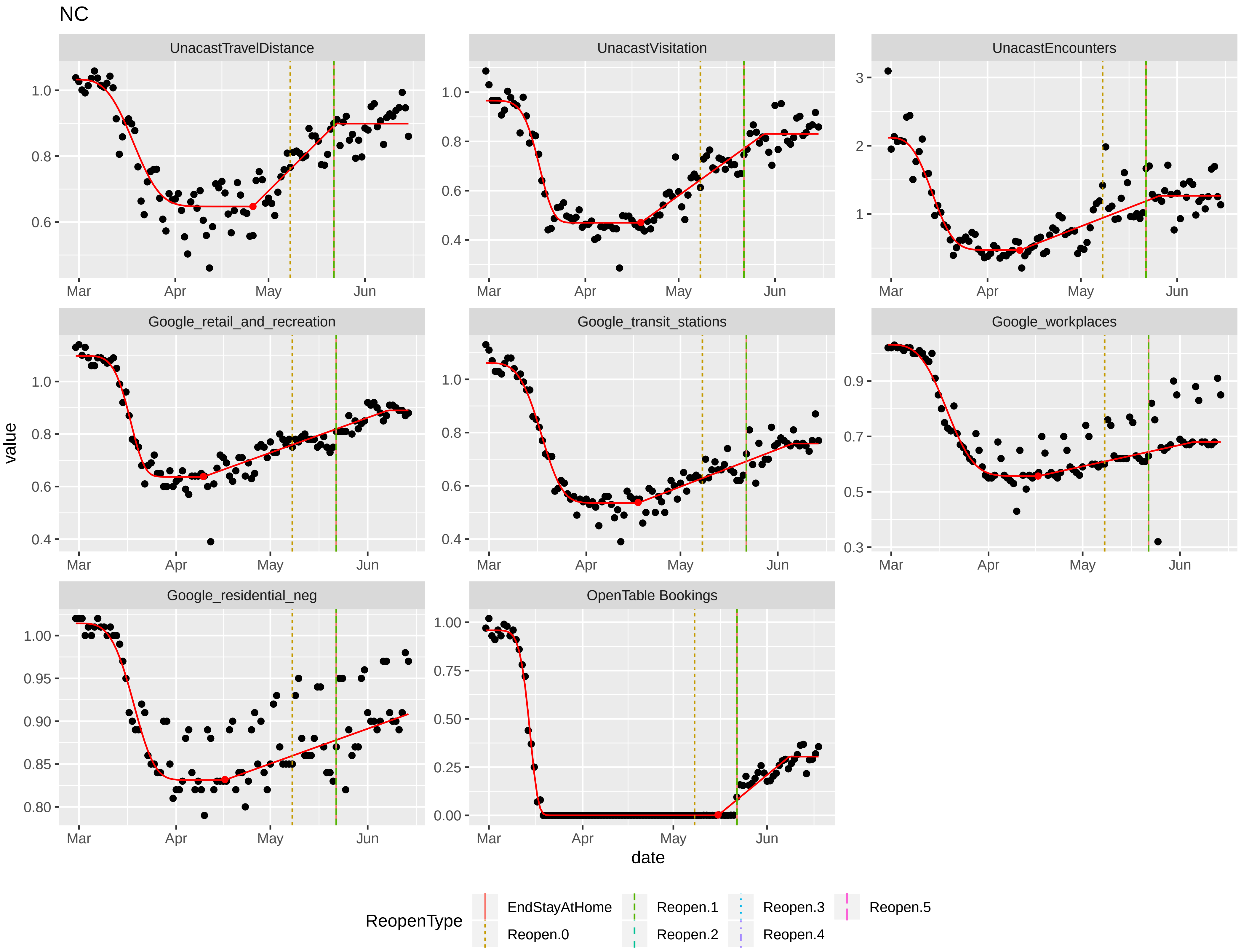
NC
UnacastTravelDistance
UnacastVisitation
UnacastEncounters
Google_retail_and_recreation
Google_transit_stations
Google_workplaces
Google_residential_neg
OpenTable Bookings
value
date
Mar
Apr
May
Jun
ReopenType
EndStayAtHome
Reopen.0
Reopen.1
Reopen.2
Reopen.3
Reopen.4
Reopen.5

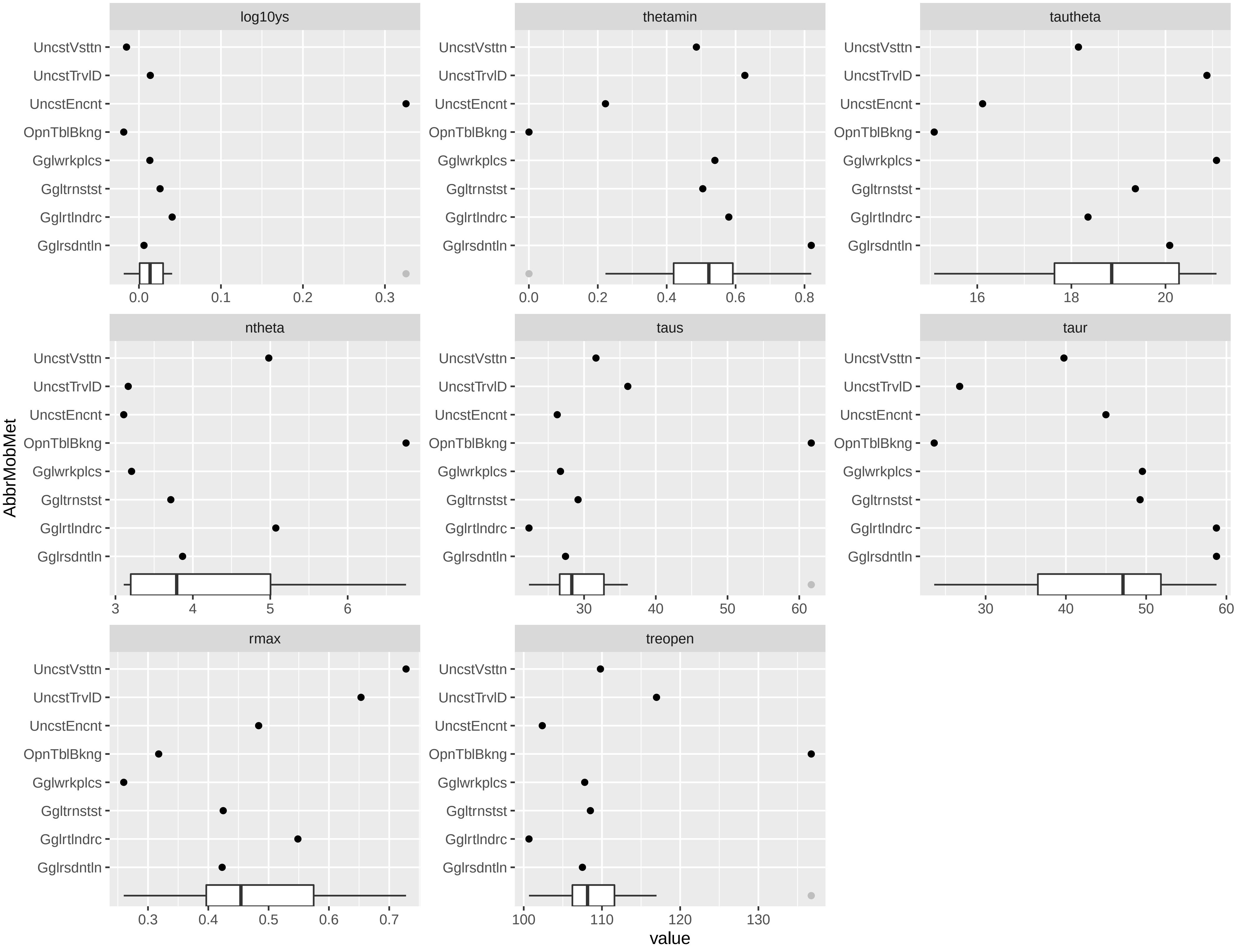
log10ys
thetamin
tautheta
ntheta
taus
taur
rmax
treopen
UncstVsttn
UncstTrvlD
UncstEncnt
OpnTblBkng
Gglwrkplcs
Ggltrnstst
Gglrtlndrc
Gglrsdntln
AbbrMobMet
value

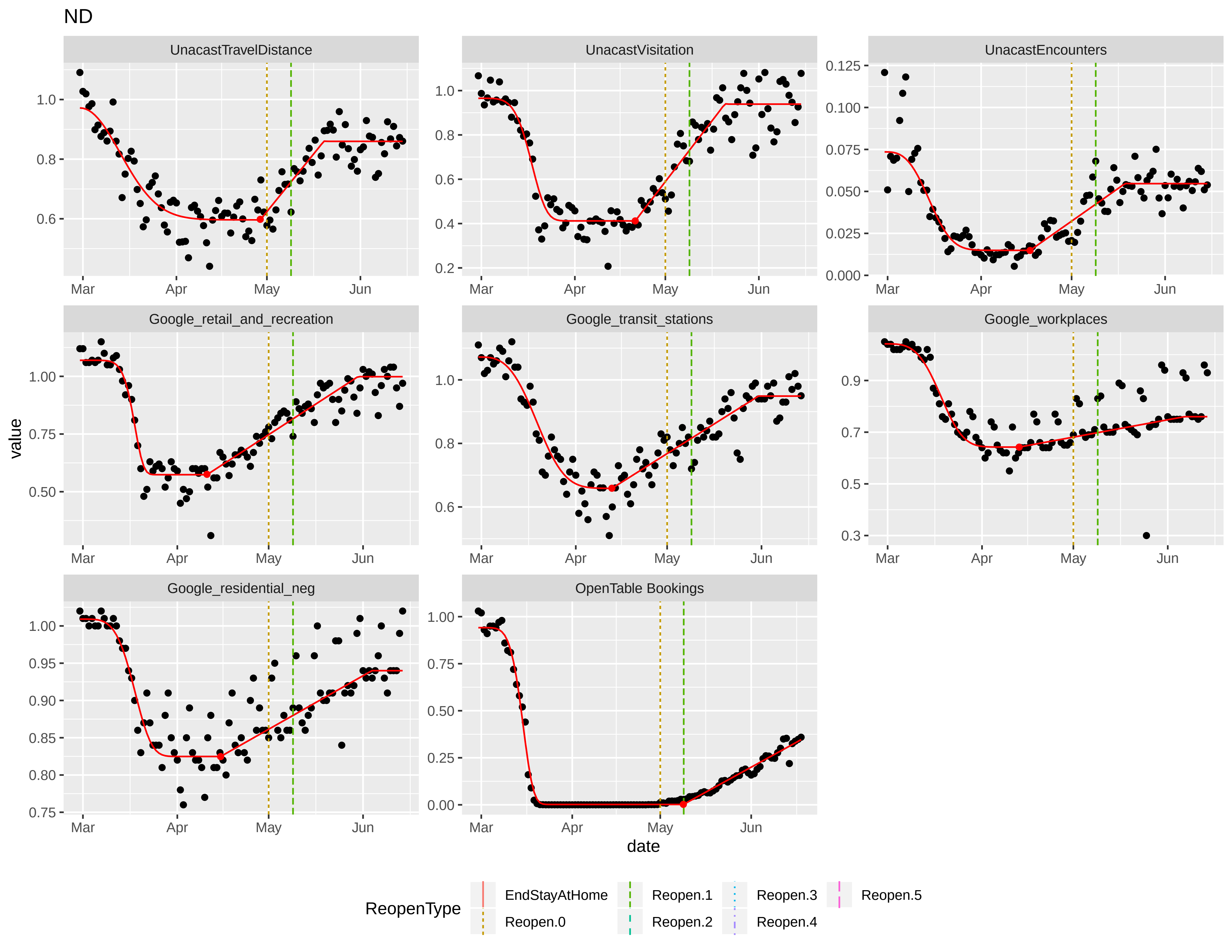

ND
UnacastTravelDistance
UnacastVisitation
UnacastEncounters
Google_retail_and_recreation
Google_transit_stations
Google_workplaces
Google_residential_neg
OpenTable Bookings
value
date
Mar
Apr
May
Jun
ReopenType
EndStayAtHome
Reopen.0
Reopen.1
Reopen.2
Reopen.3
Reopen.4
Reopen.5

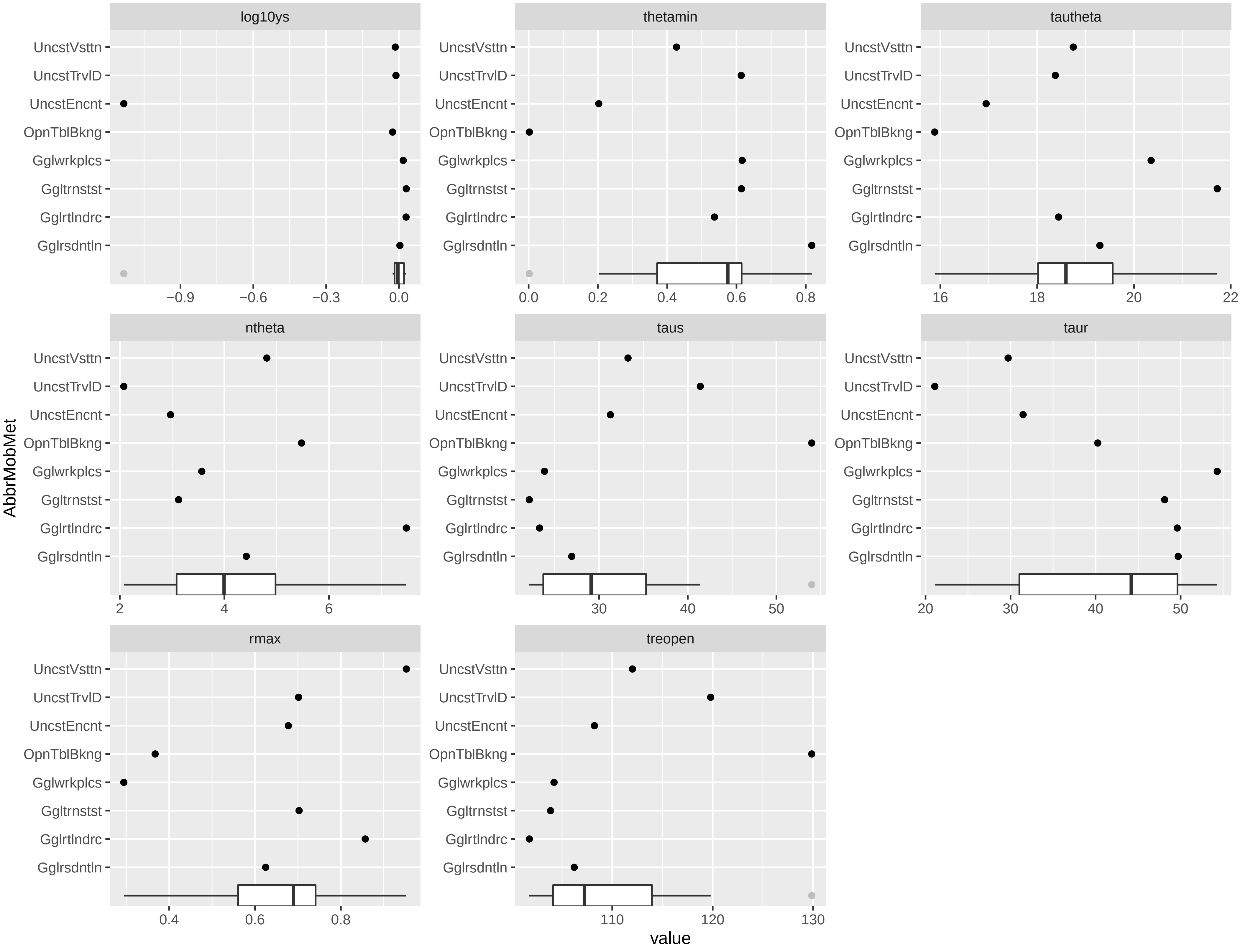
log10ys
thetamin
tautheta
ntheta
taus
taur
rmax
treopen
UncstVsttn
UncstTrvlD
UncstEncnt
OpnTblBkng
Gglwrkplcs
Ggltrnstst
Gglrtlndrc
Gglrsdntln
AbbrMobMet
value
−0.9
−0.6
−0.3
0.0
0.2
0.4
0.6
0.8
16
18
20
22
2
4
6
30
40
50
20
110
120
130

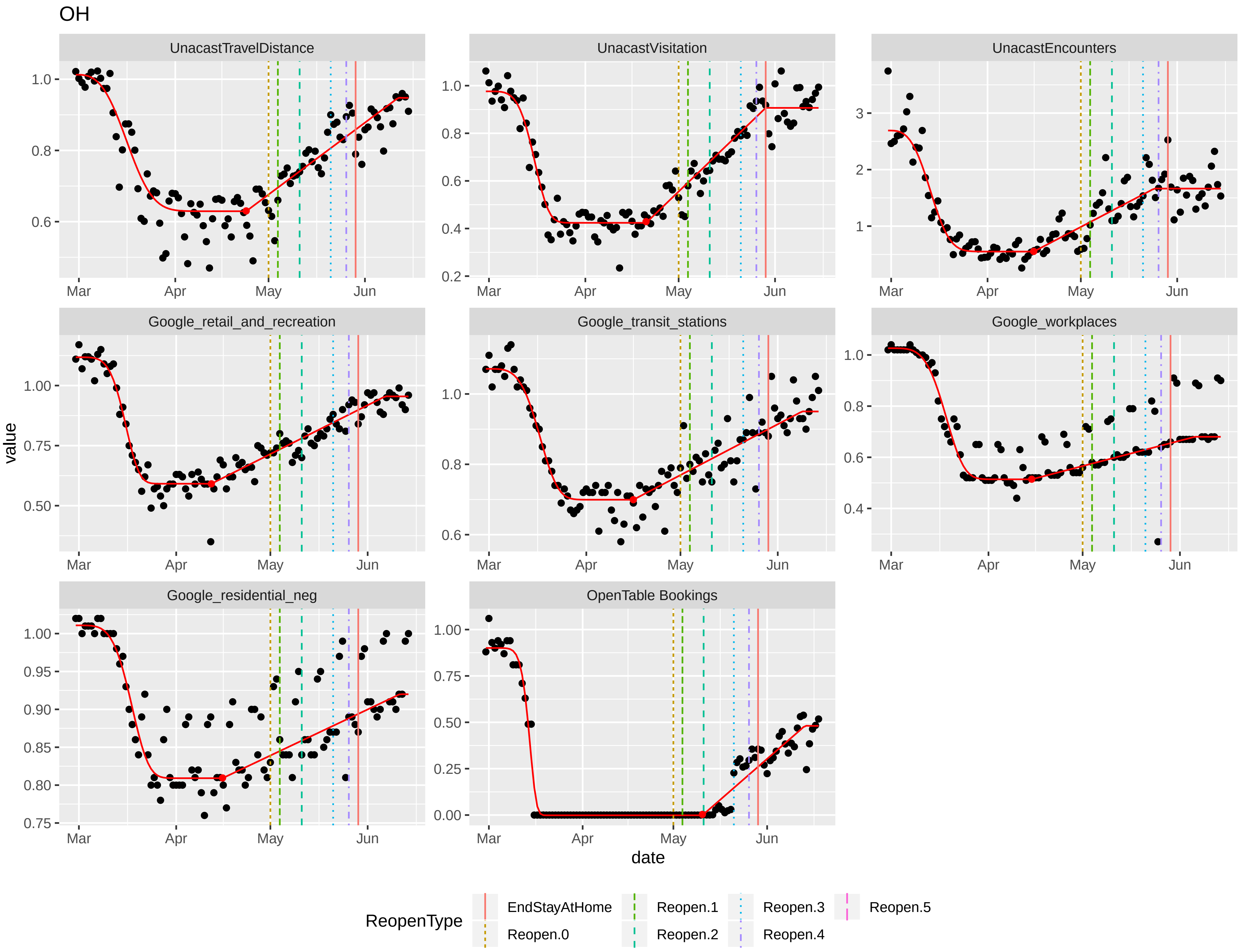
OH
UnacastTravelDistance
UnacastVisitation
UnacastEncounters
Google_retail_and_recreation
Google_transit_stations
Google_workplaces
Google_residential_neg
OpenTable Bookings
value
date
Mar
Apr
May
Jun
ReopenType
EndStayAtHome
Reopen.0
Reopen.1
Reopen.2
Reopen.3
Reopen.4
Reopen.5

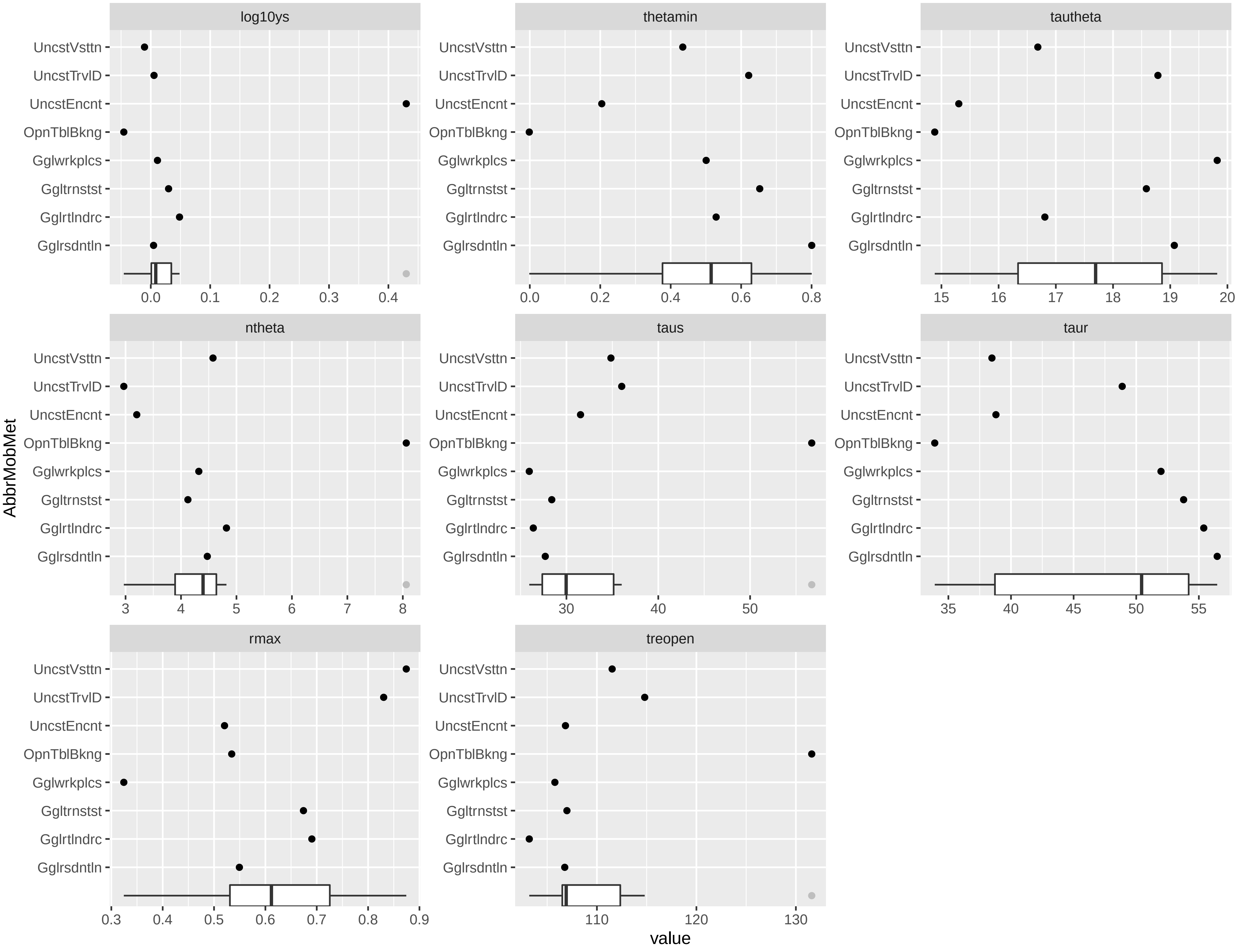

log10ys
thetamin
tautheta
ntheta
taus
taur
rmax
treopen
UncstVsttn
UncstTrvlD
UncstEncnt
OpnTblBkng
Gglwrkplcs
Ggltrnstst
Gglrtlndrc
Gglrsdntln
AbbrMobMet
value

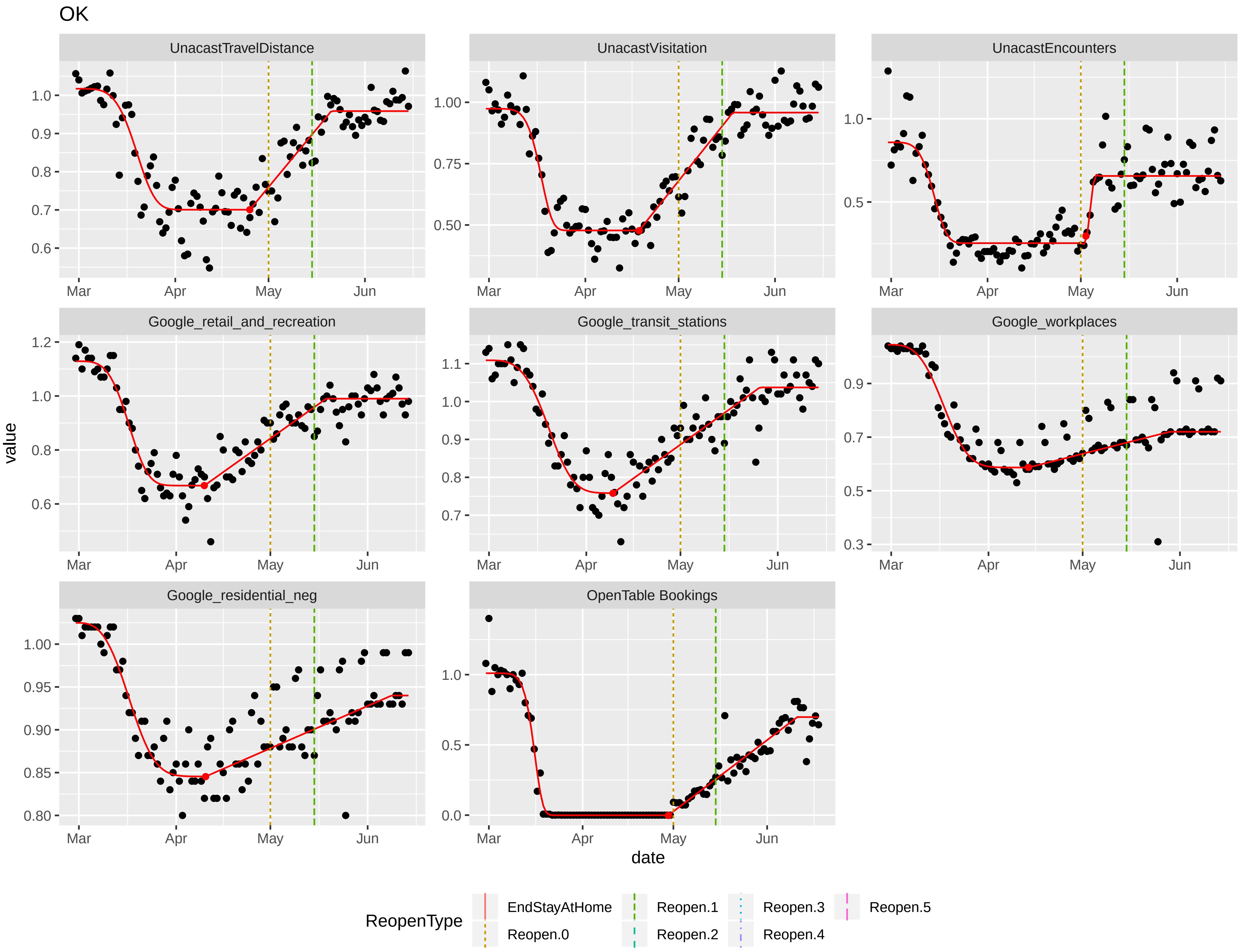
OK
UnacastTravelDistance
UnacastVisitation
UnacastEncounters
Google_retail_and_recreation
Google_transit_stations
Google_workplaces
Google_residential_neg
OpenTable Bookings
value
date
Mar
Apr
May
Jun
ReopenType
EndStayAtHome
Reopen.0
Reopen.1
Reopen.2
Reopen.3
Reopen.4
Reopen.5

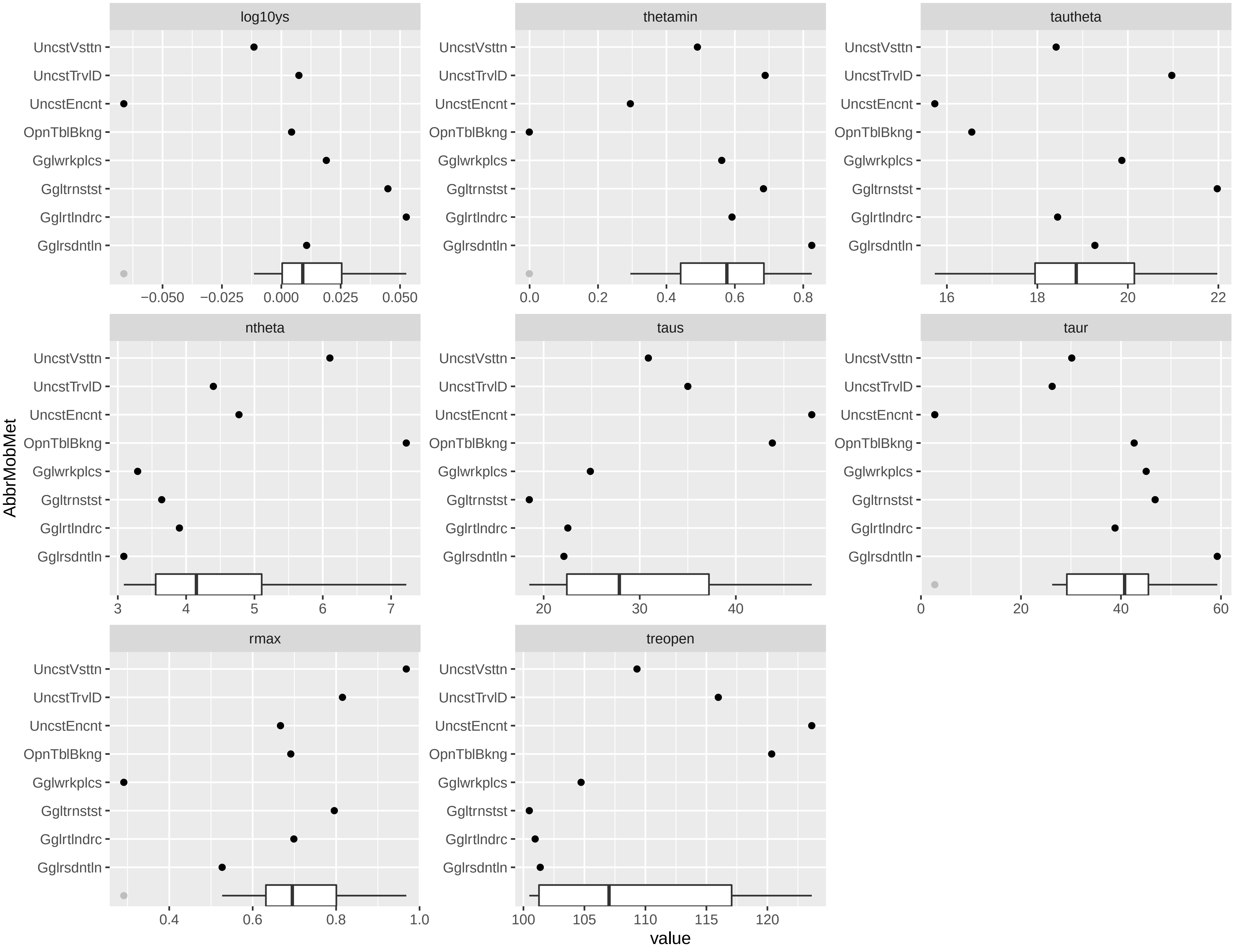

log10ys
thetamin
tautheta
ntheta
taus
taur
rmax
treopen
UncstVsttn
UncstTrvlD
UncstEncnt
OpnTblBkng
Gglwrkplcs
Goltrnstst
Gglrtlndrc
Gglrsdntln
−0.050
−0.025
0.000
0.025
0.050
0.0
0.2
0.4
0.6
0.8
16
18
20
22
3
4
5
6
7
20
30
40
0
20
40
60
0.4
0.6
0.8
1.0
100
105
110
115
120
AbbrMobMet
value

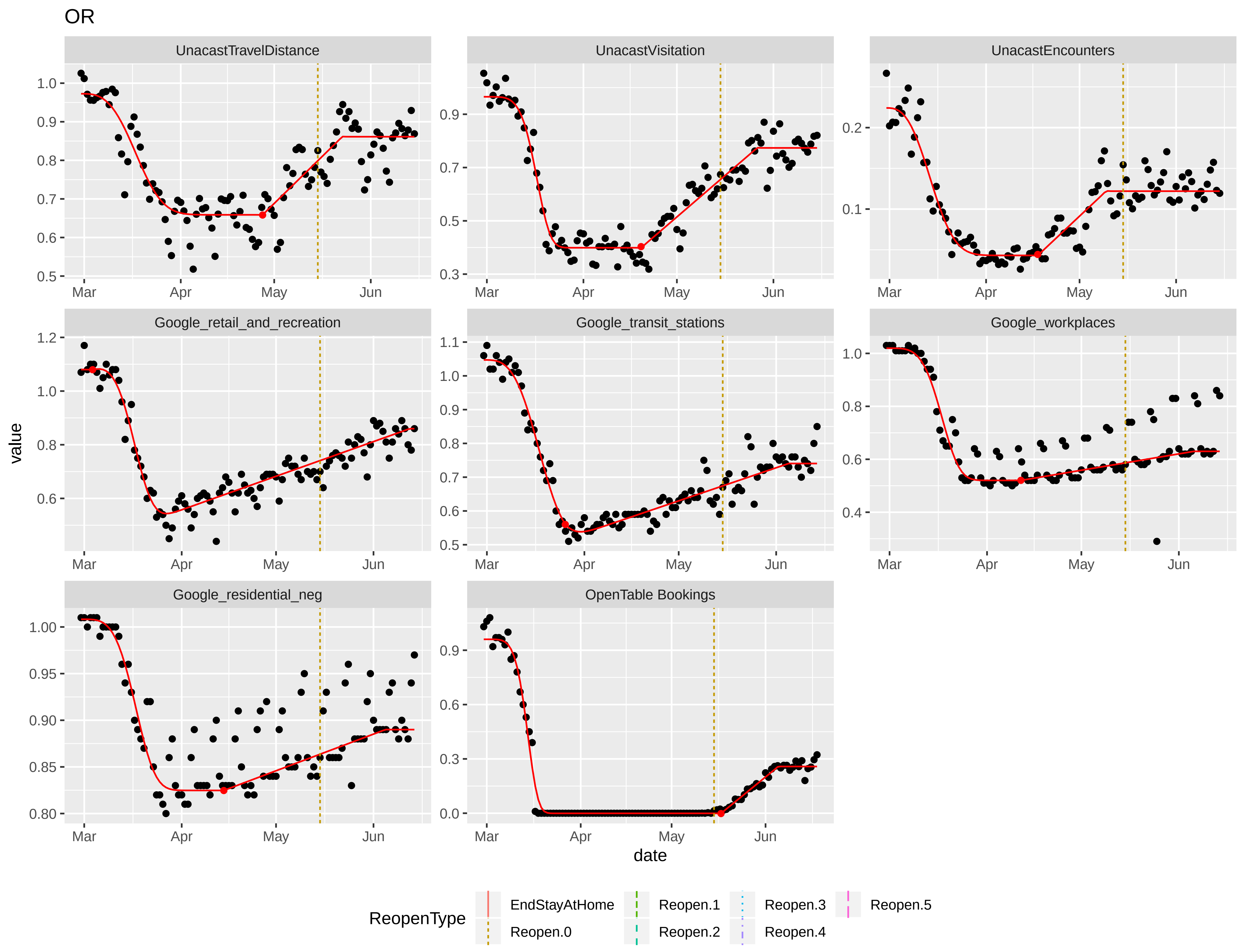
OR
UnacastTravelDistance
UnacastVisitation
UnacastEncounters
Google_retail_and_recreation
Google_transit_stations
Google_workplaces
Google_residential_neg
OpenTable Bookings
value
date
Mar
Apr
May
Jun
ReopenType
EndStayAtHome
Reopen.0
Reopen.1
Reopen.2
Reopen.3
Reopen.4
Reopen.5

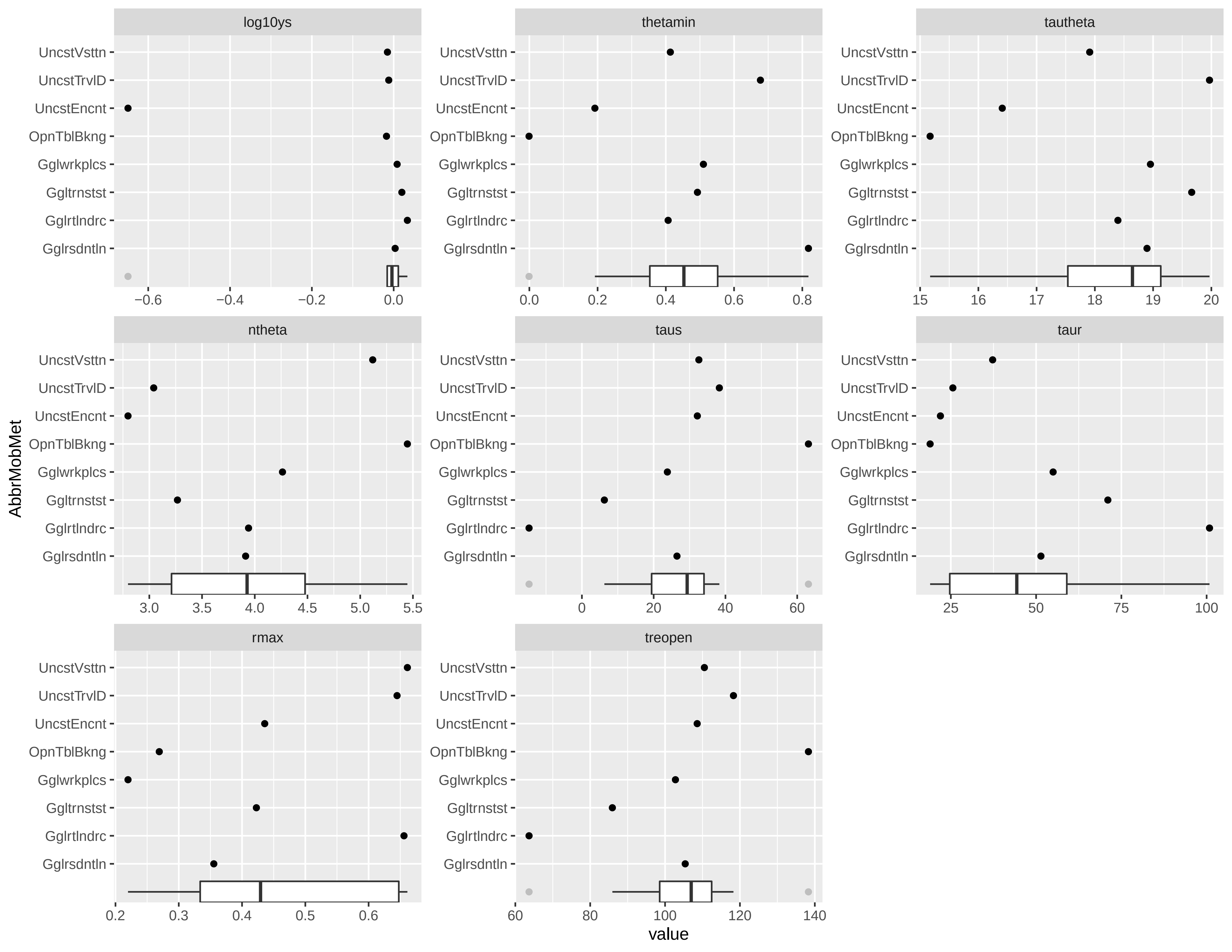
log10ys
thetamin
tautheta
ntheta
taus
taur
rmax
treopen
UncstVsttn
UncstTrvlD
UncstEncnt
OpnTblBkng
Gglwrkplcs
Ggltrnstst
Gglrtlndrc
Gglrsdntln
AbbrMobMet
value

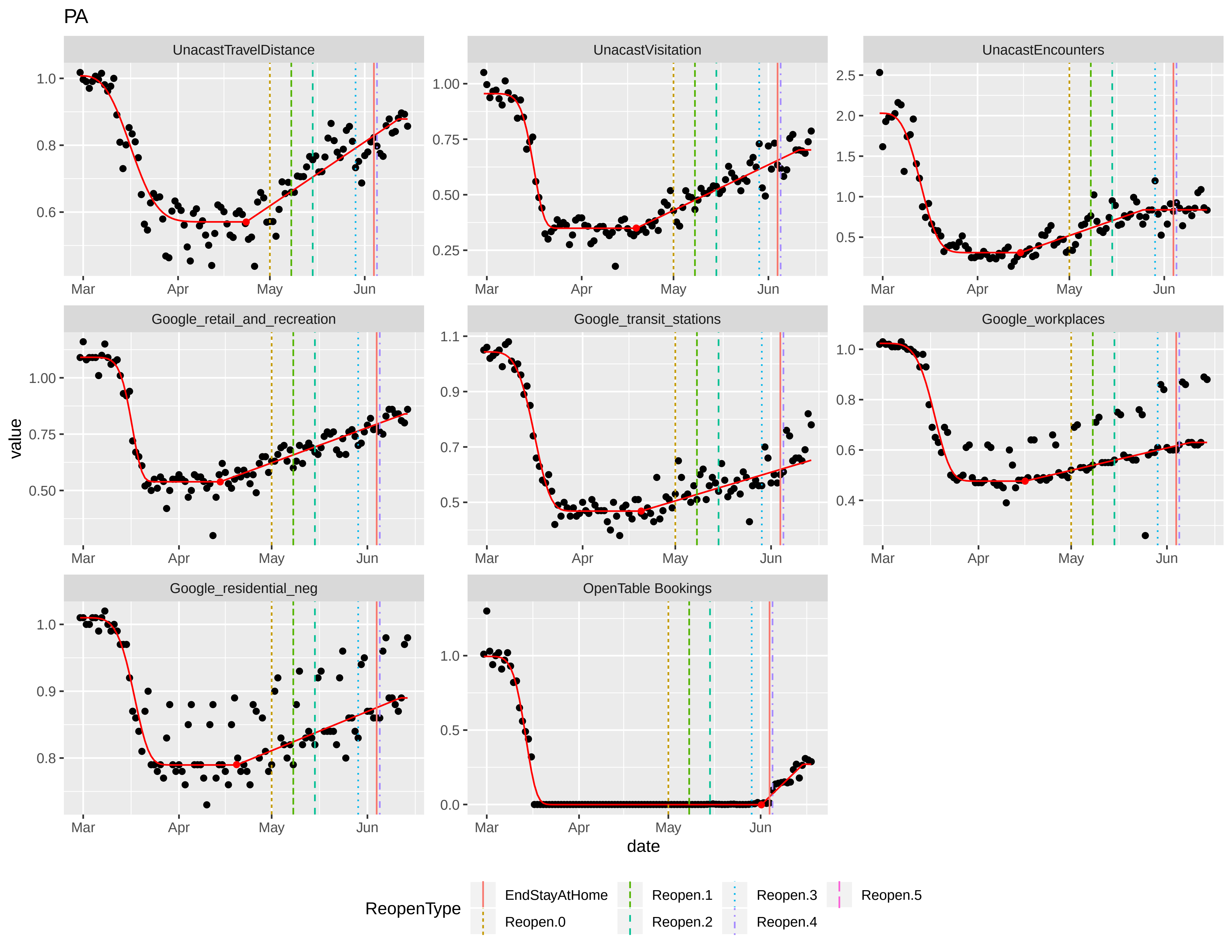

PA
UnacastTravelDistance
UnacastVisitation
UnacastEncounters
Google_retail_and_recreation
Google_transit_stations
Google_workplaces
Google_residential_neg
OpenTable Bookings
value
date
Mar
Apr
May
Jun
ReopenType
EndStayAtHome
Reopen.0
Reopen.1
Reopen.2
Reopen.3
Reopen.4
Reopen.5

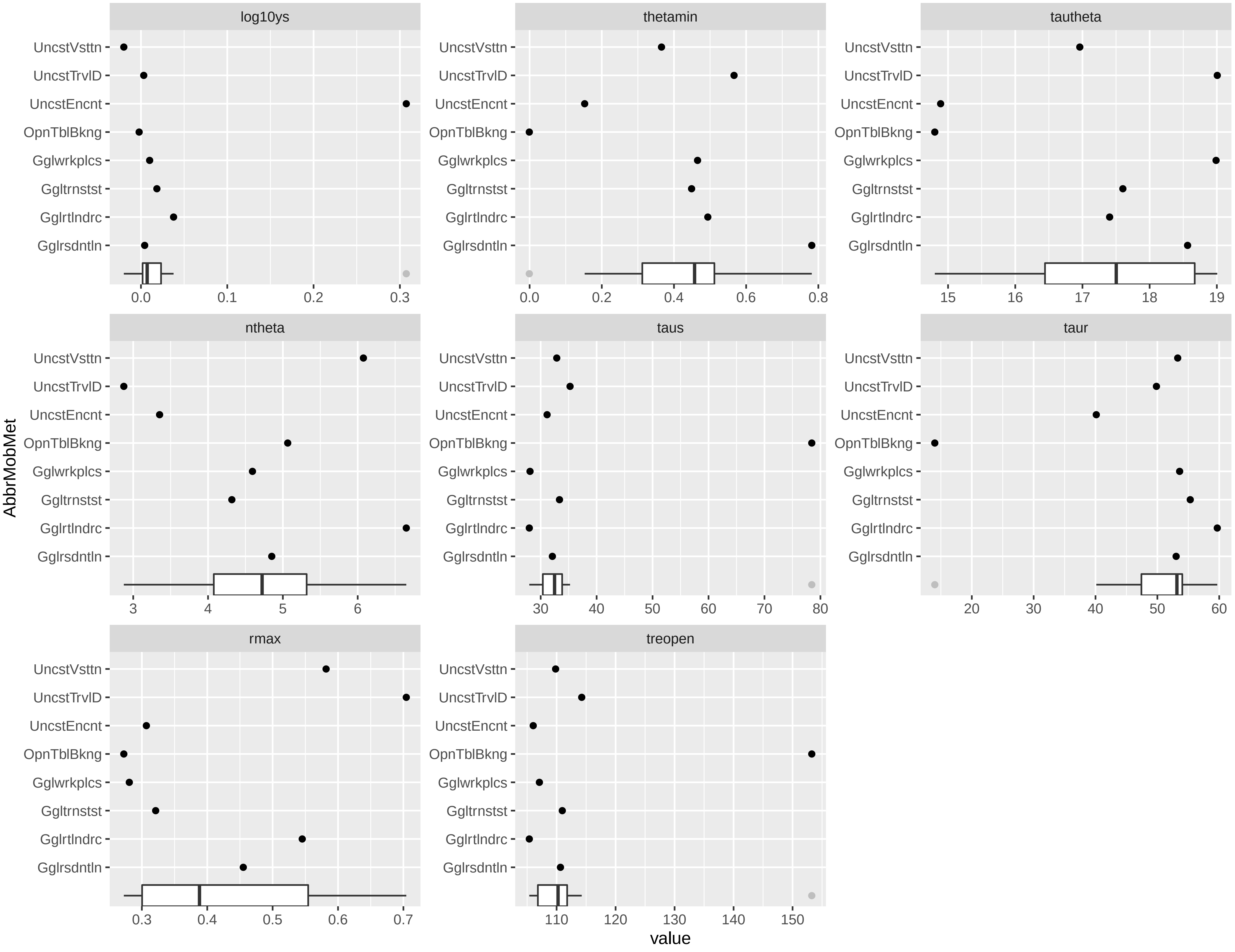
log10ys
thetamin
tautheta
ntheta
taus
taur
rmax
treopen
UncstVsttn
UncstTrvlD
UncstEncnt
OpnTblBkng
Gglwrkplcs
Ggltrnstst
Gglrtlndrc
Gglrsdntln
AbbrMobMet
value

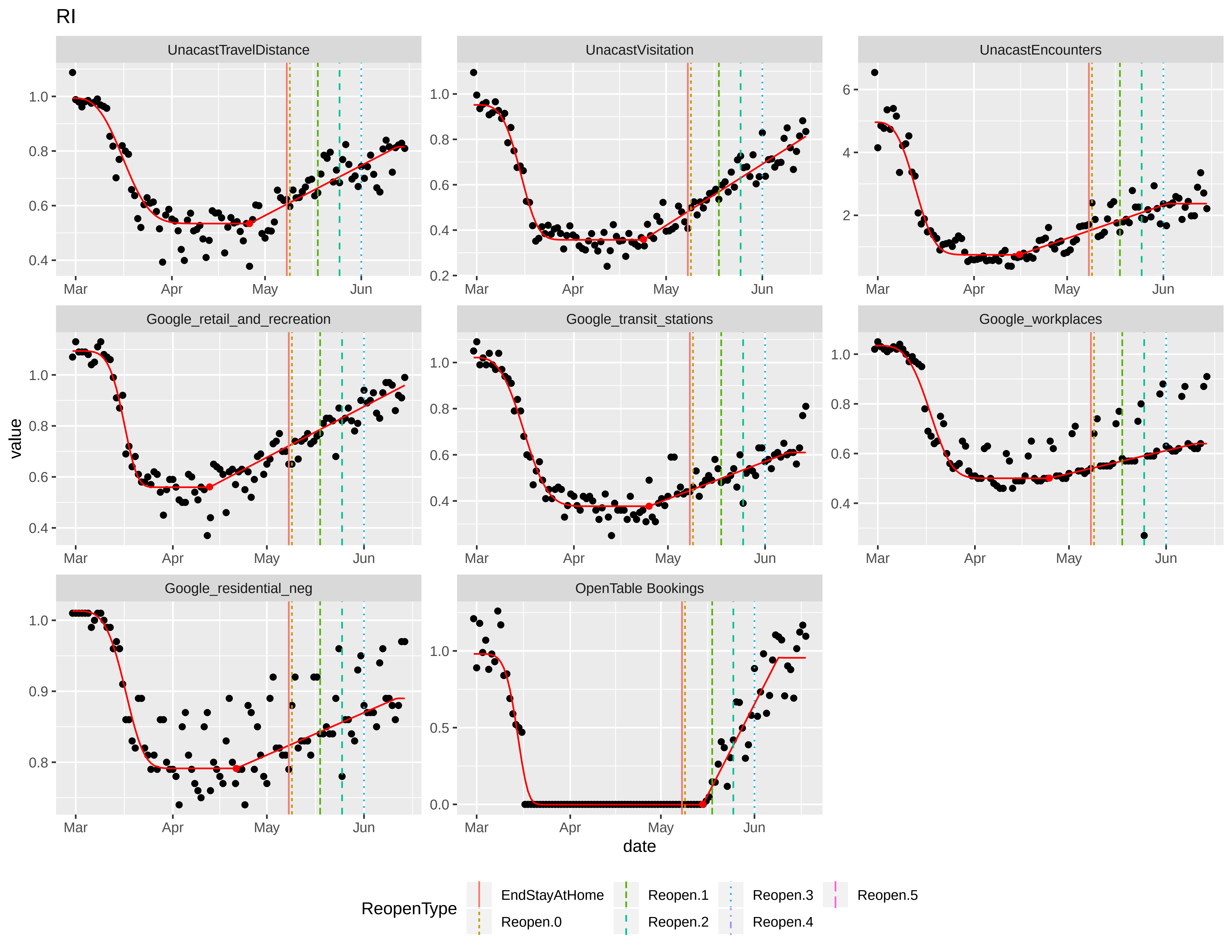

RI
UnacastTravelDistance
UnacastVisitation
UnacastEncounters
Google_retail_and_recreation
Google_transit_stations
Google_workplaces
Google_residential_neg
OpenTable Bookings
value
date
Mar
Apr
May
Jun
ReopenType
EndStayAtHome
Reopen.0
Reopen.1
Reopen.2
Reopen.3
Reopen.4
Reopen.5

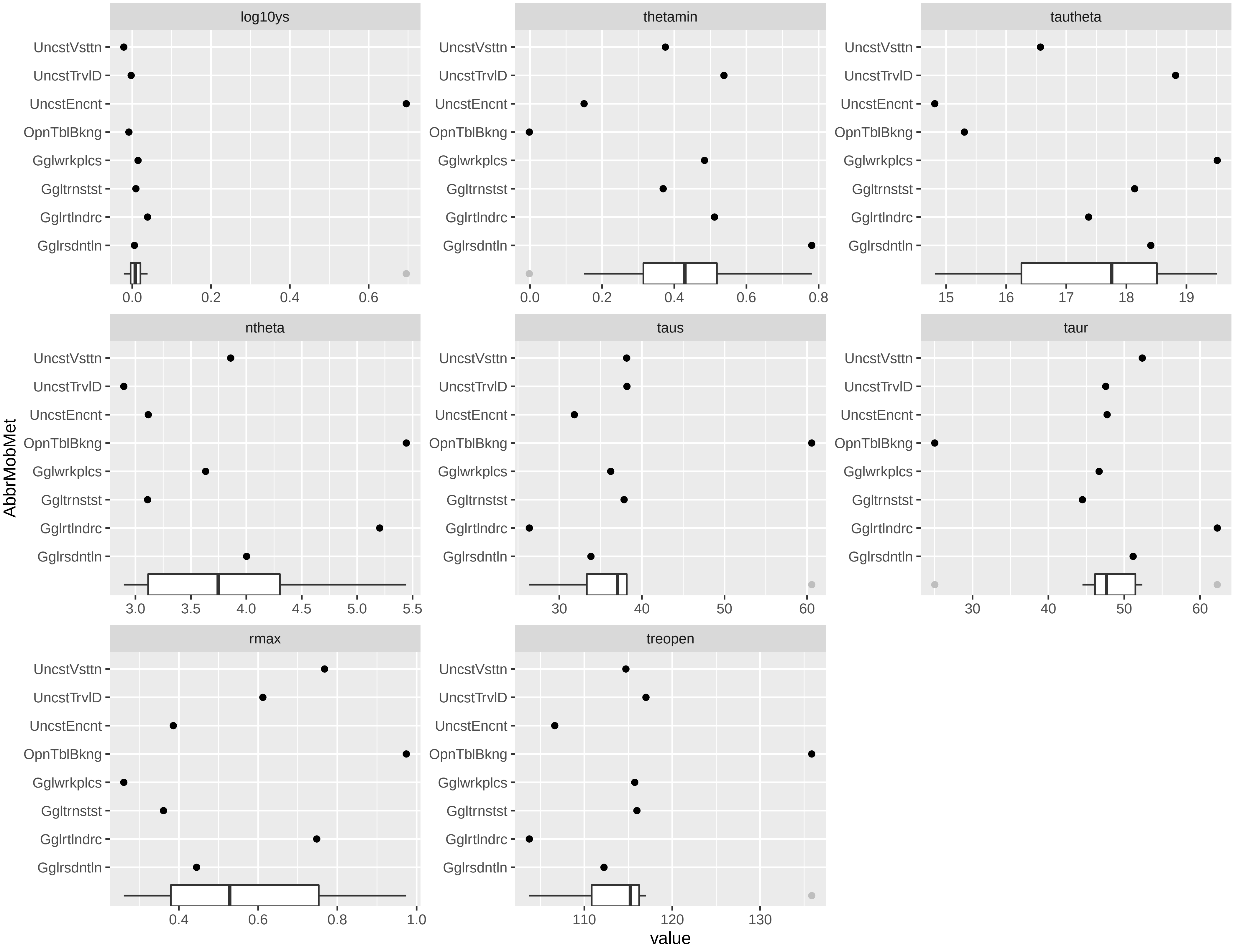

log10ys
thetamin
tautheta
ntheta
taus
taur
rmax
treopen
UncstVsttn
UncstTrvlD
UncstEncnt
OpnTblBkng
Gglwrkplcs
Ggltrnstst
Gglrtlndrc
Gglrsdntln
AbbrMobMet
value

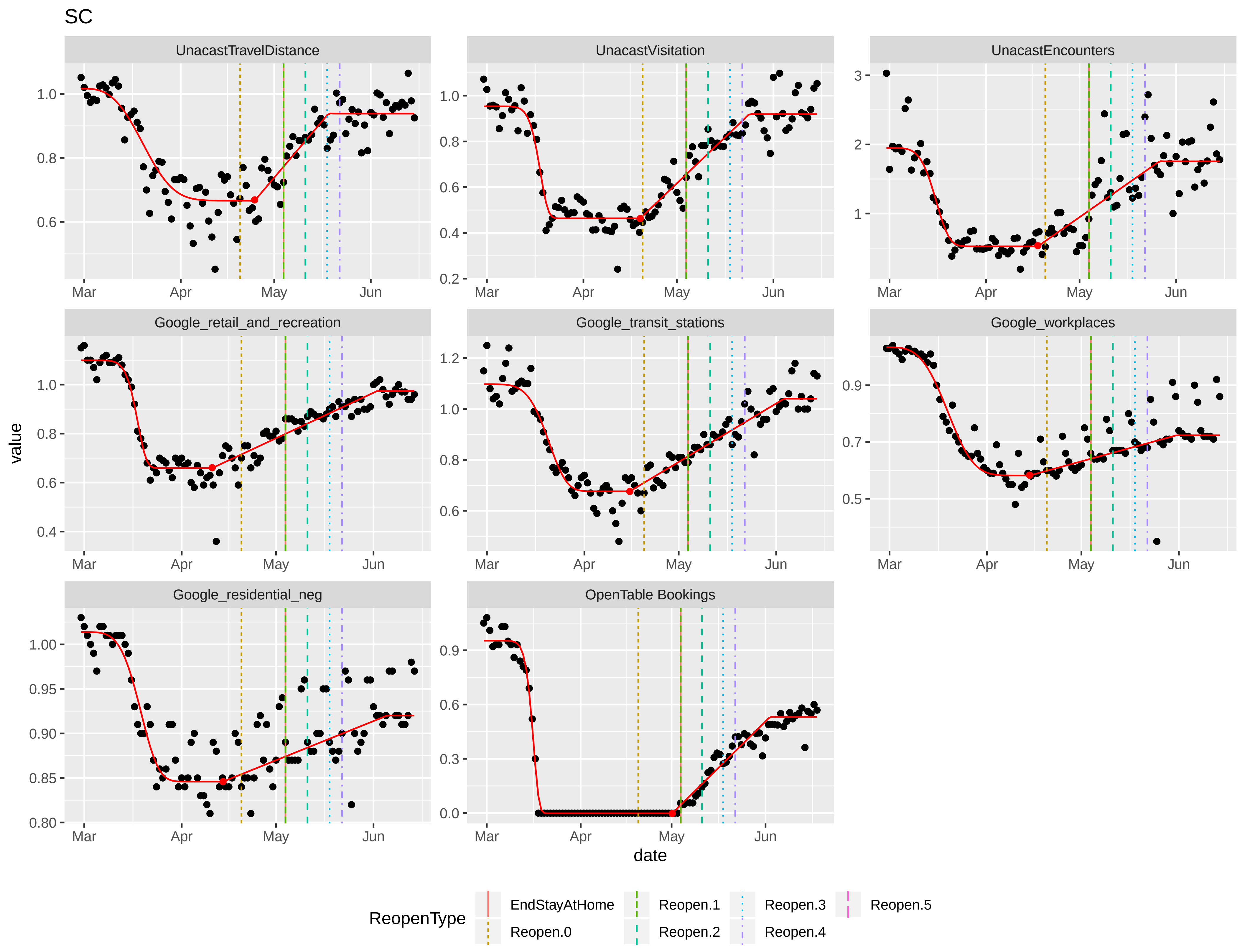

SC
UnacastTravelDistance
UnacastVisitation
UnacastEncounters
Google_retail_and_recreation
Google_transit_stations
Google_workplaces
Google_residential_neg
OpenTable Bookings
value
date
Mar
Apr
May
Jun
ReopenType
EndStayAtHome
Reopen.0
Reopen.1
Reopen.2
Reopen.3
Reopen.4
Reopen.5

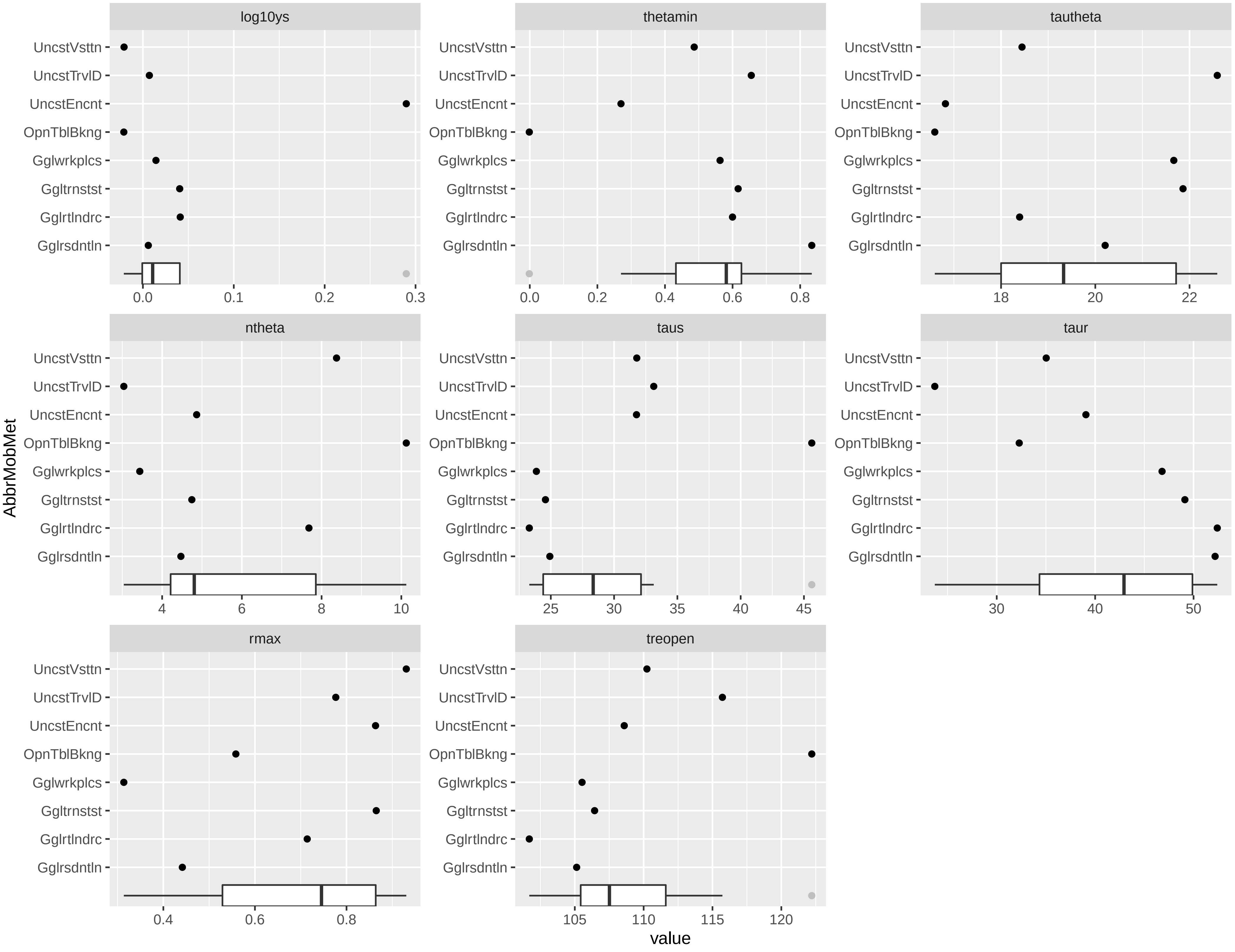

log10ys
thetamin
tautheta
ntheta
taus
taur
rmax
treopen
UncstVsttn
UncstTrvlD
UncstEncnt
OpnTblBkng
Gglwrkplcs
Ggltrnstst
Gglrtlndrc
Gglrsdntln
AbbrMobMet
value

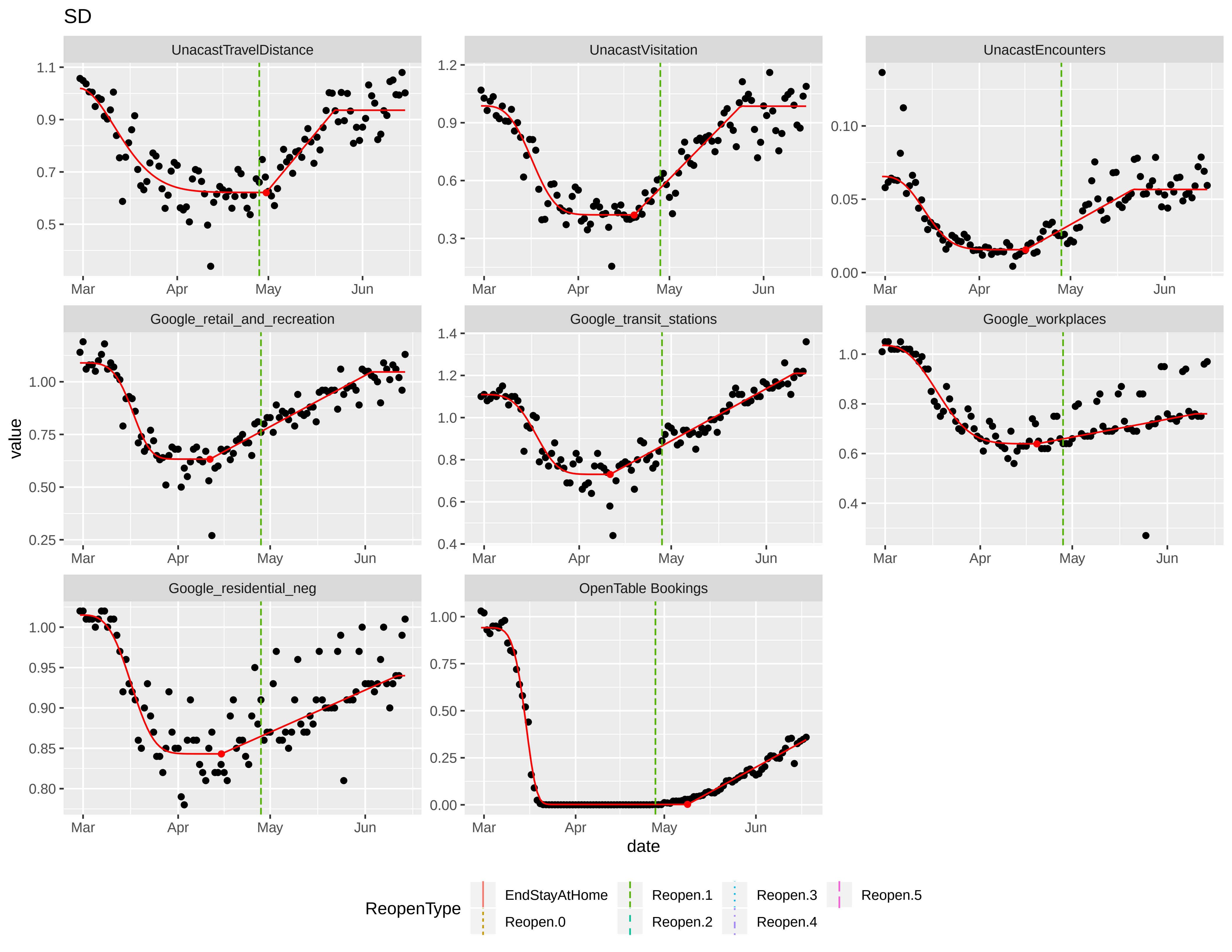
SD
UnacastTravelDistance
UnacastVisitation
UnacastEncounters
Google_retail_and_recreation
Google_transit_stations
Google_workplaces
Google_residential_neg
OpenTable Bookings
value
date
Mar
Apr
May
Jun
ReopenType
EndStayAtHome
Reopen.0
Reopen.1
Reopen.2
Reopen.3
Reopen.4
Reopen.5

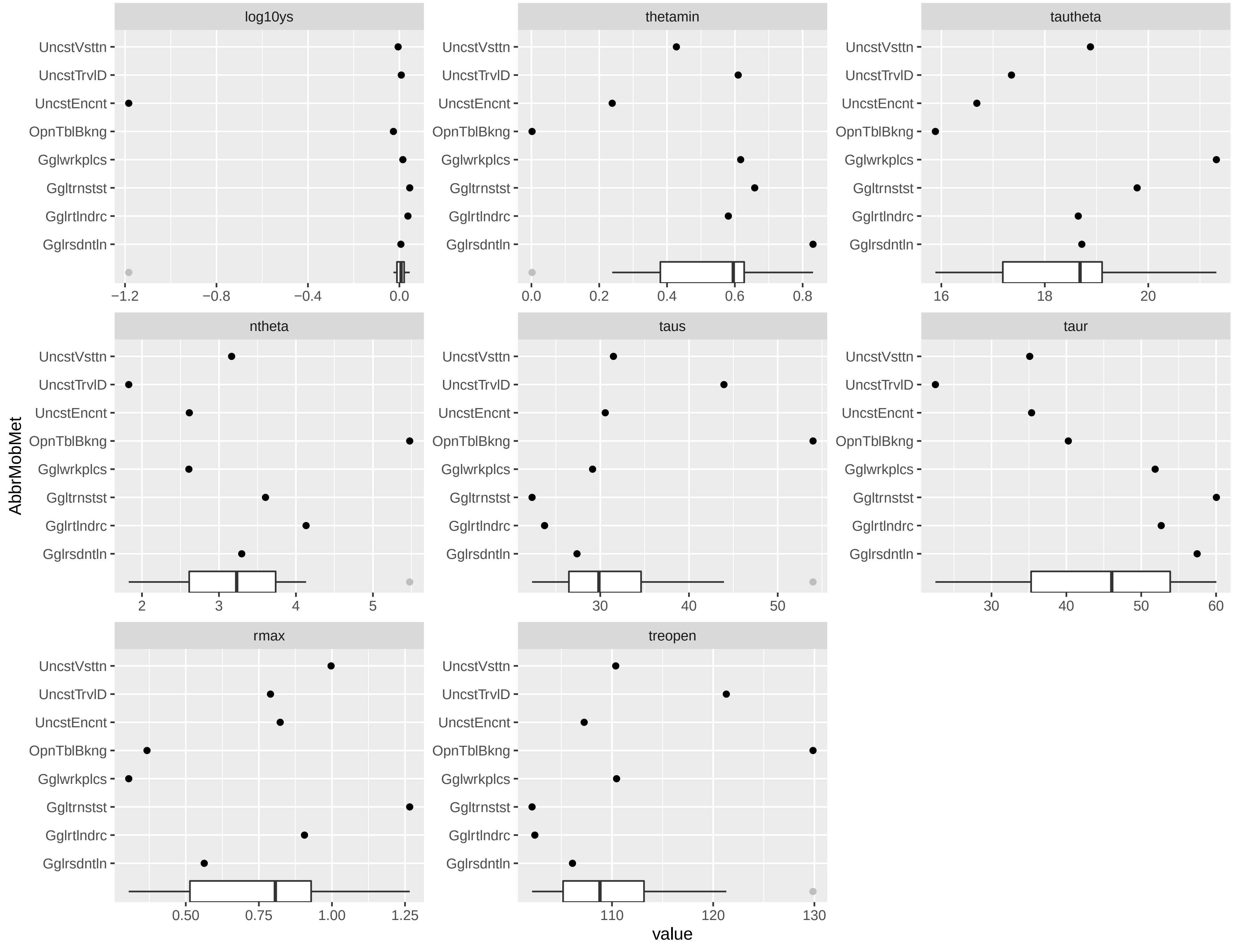

log10ys
thetamin
tautheta
ntheta
taus
taur
rmax
treopen
UncstVsttn
UncstTrvlD
UncstEncnt
OpnTblBkng
Gglwrkplcs
Ggltrnstst
Gglrtlndrc
Gglrsdntln
AbbrMobMet
value

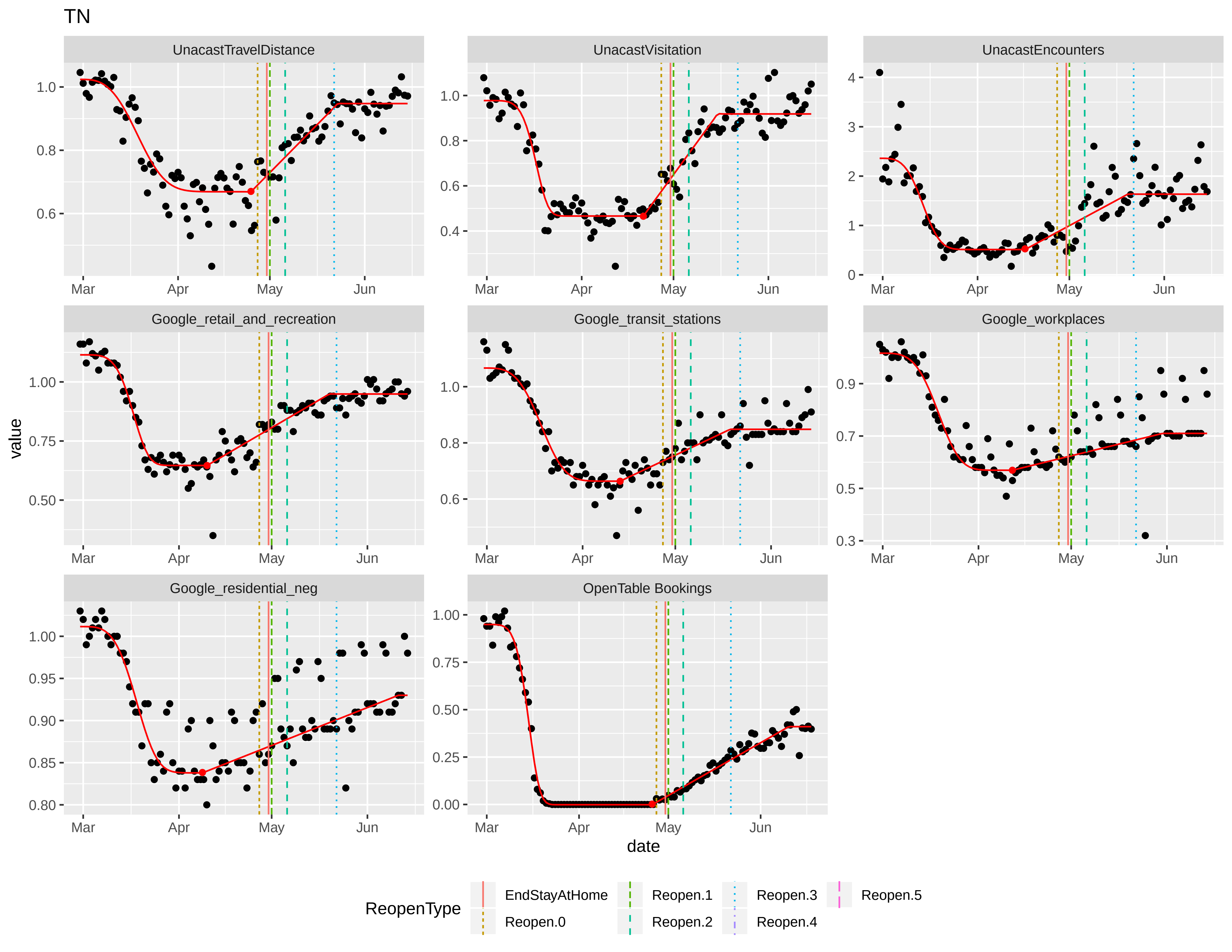
TN
UnacastTravelDistance
UnacastVisitation
UnacastEncounters
Google_retail_and_recreation
Google_transit_stations
Google_workplaces
Google_residential_neg
OpenTable Bookings
value
date
Mar
Apr
May
Jun
ReopenType
EndStayAtHome
Reopen.0
Reopen.1
Reopen.2
Reopen.3
Reopen.4
Reopen.5

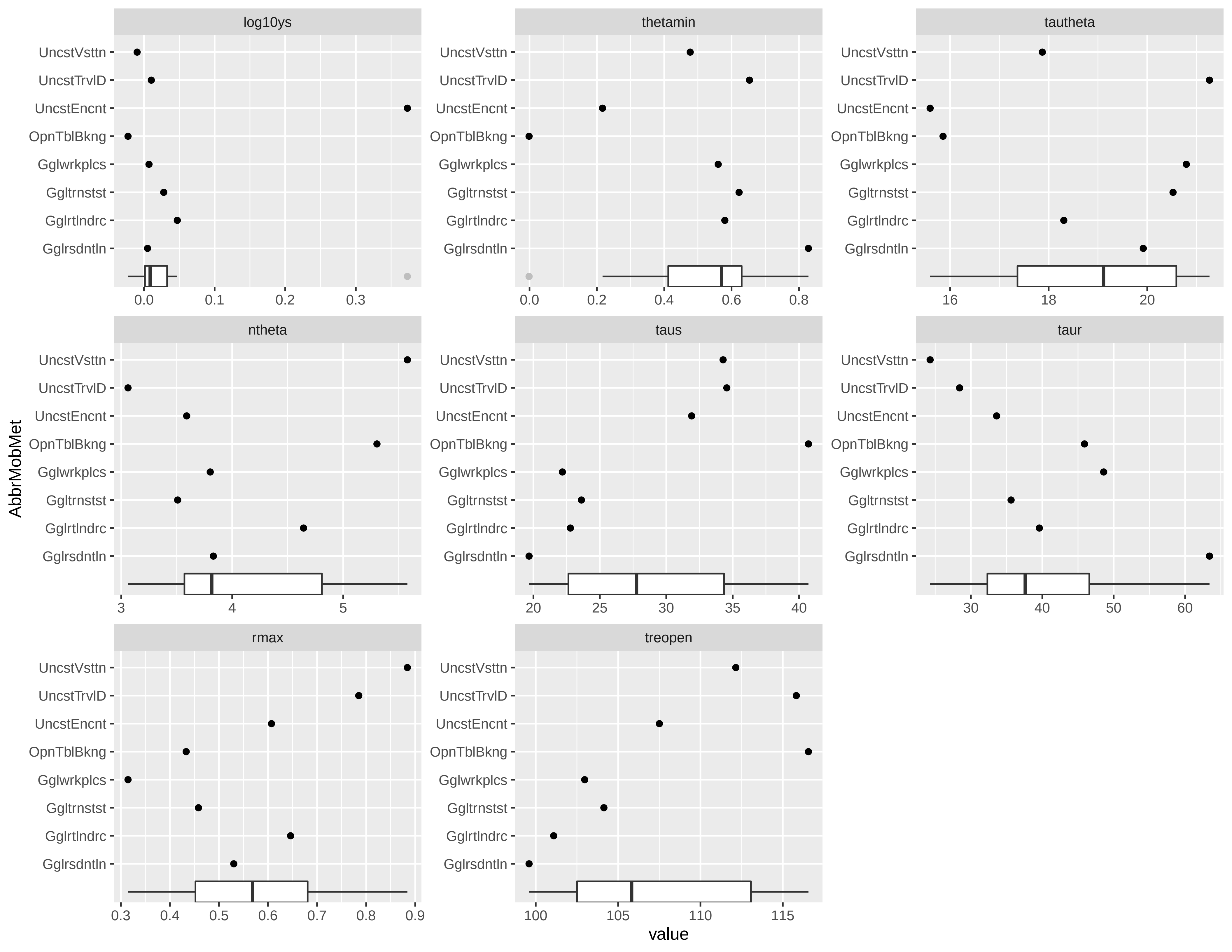

log10ys
thetamin
tautheta
ntheta
taus
taur
rmax
treopen
UncstVsttn
UncstTrvlD
UncstEncnt
OpnTblBkng
Gglwrkplcs
Ggltrnstst
Gglrtlndrc
Gglrsdntln
AbbrMobMet
value

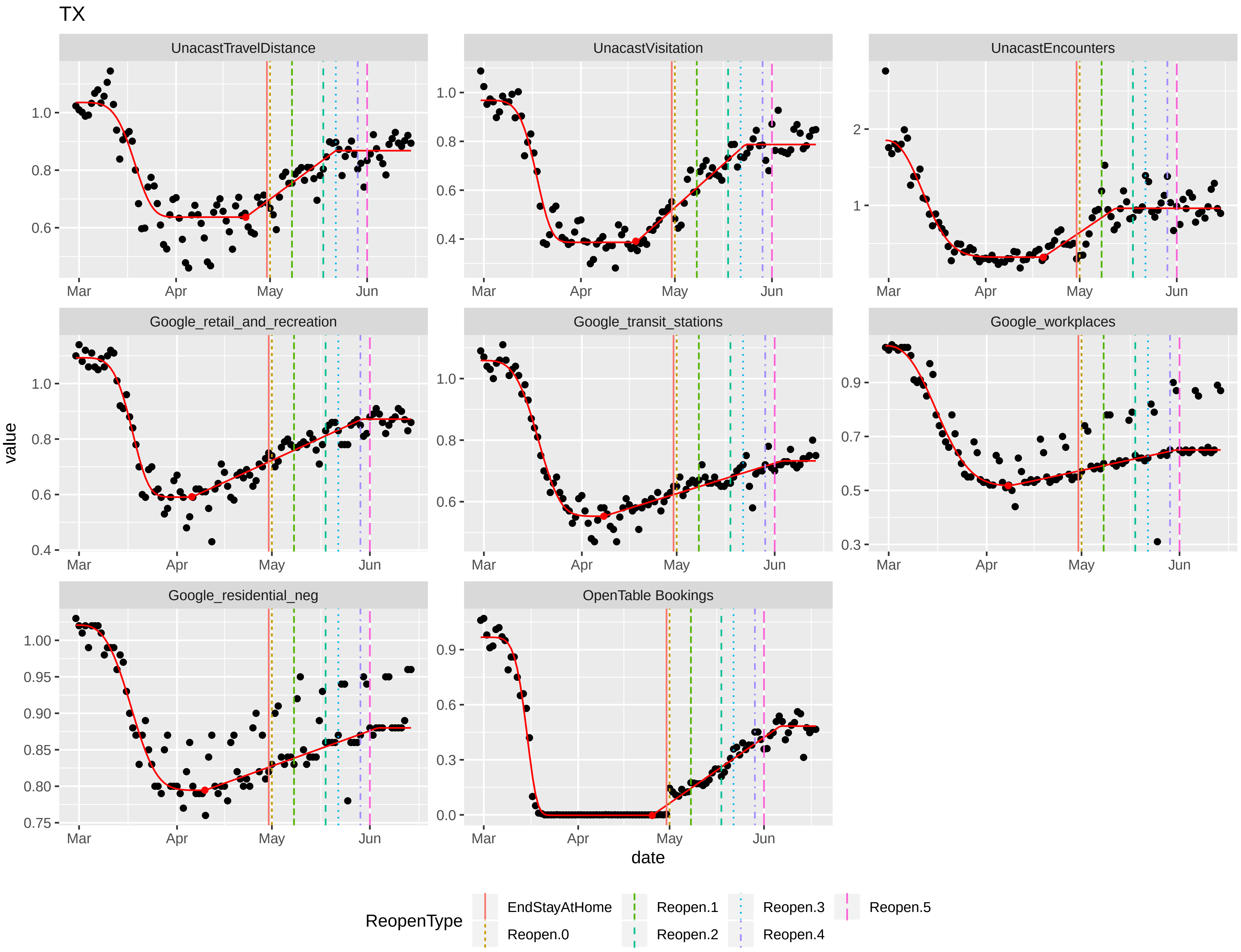

TX
UnacastTravelDistance
UnacastVisitation
UnacastEncounters
Google_retail_and_recreation
Google_transit_stations
Google_workplaces
Google_residential_neg
OpenTable Bookings
value
date
Mar
Apr
May
Jun
ReopenType
EndStayAtHome
Reopen.0
Reopen.1
Reopen.2
Reopen.3
Reopen.4
Reopen.5

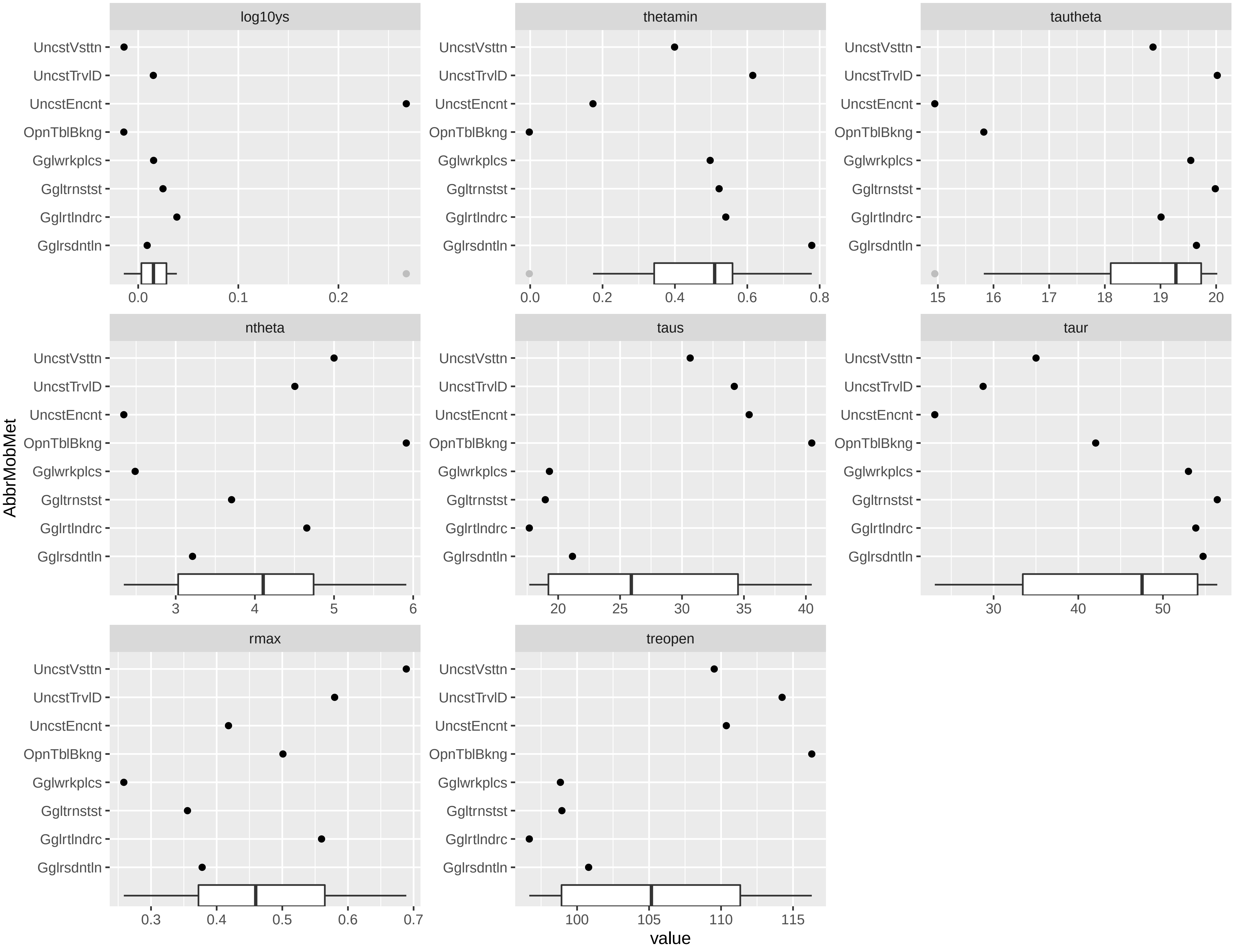
log10ys
thetamin
tautheta
ntheta
taus
taur
rmax
treopen
UncstVsttn
UncstTrvlD
UncstEncnt
OpnTblBkng
Gglwrkplcs
Ggltrnstst
Gglrtlndrc
Gglrsdntln
AbbrMobMet
value

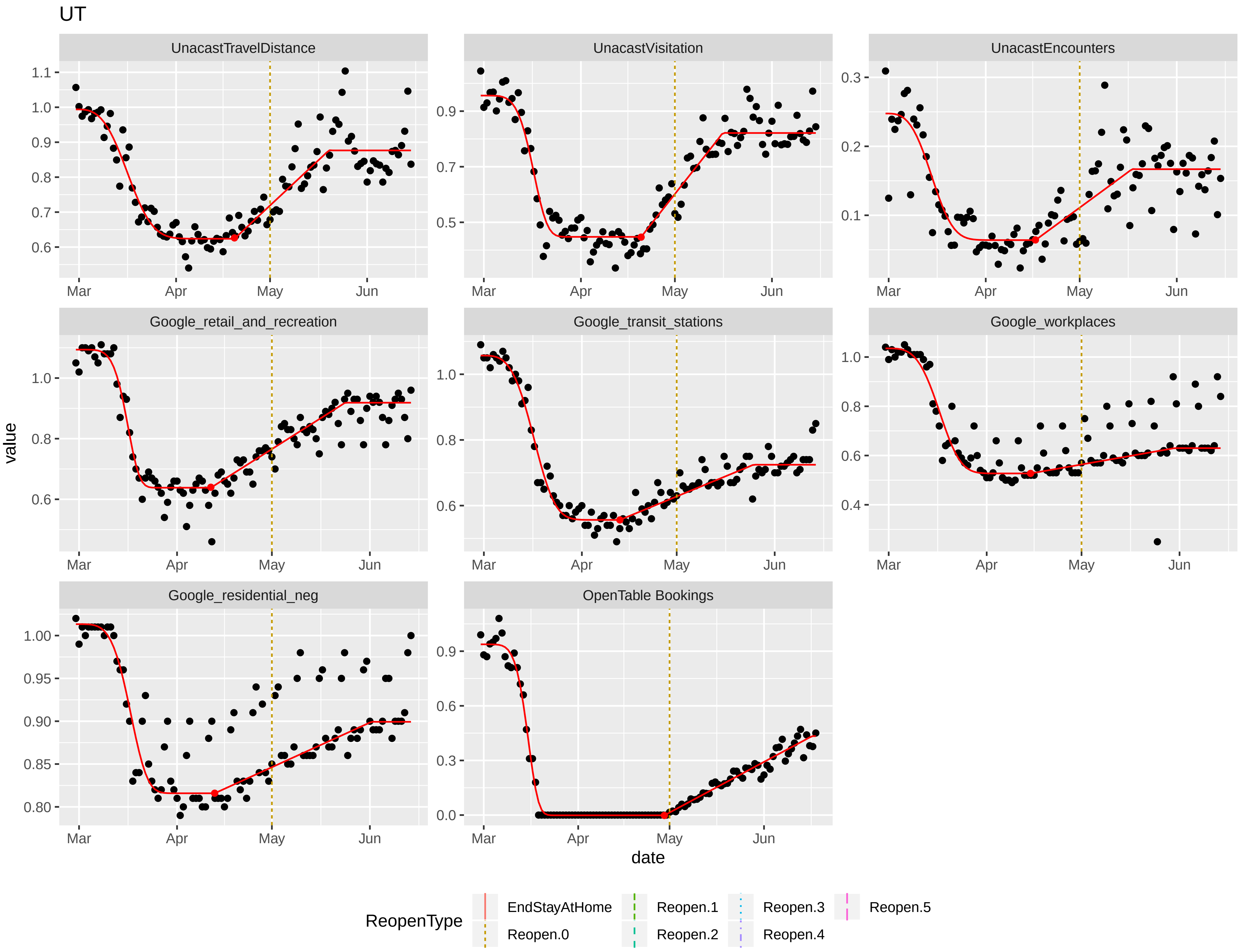
UT
UnacastTravelDistance
UnacastVisitation
UnacastEncounters
Google_retail_and_recreation
Google_transit_stations
Google_workplaces
Google_residential_neg
OpenTable Bookings
value
date
Mar
Apr
May
Jun
ReopenType
EndStayAtHome
Reopen.0
Reopen.1
Reopen.2
Reopen.3
Reopen.4
Reopen.5

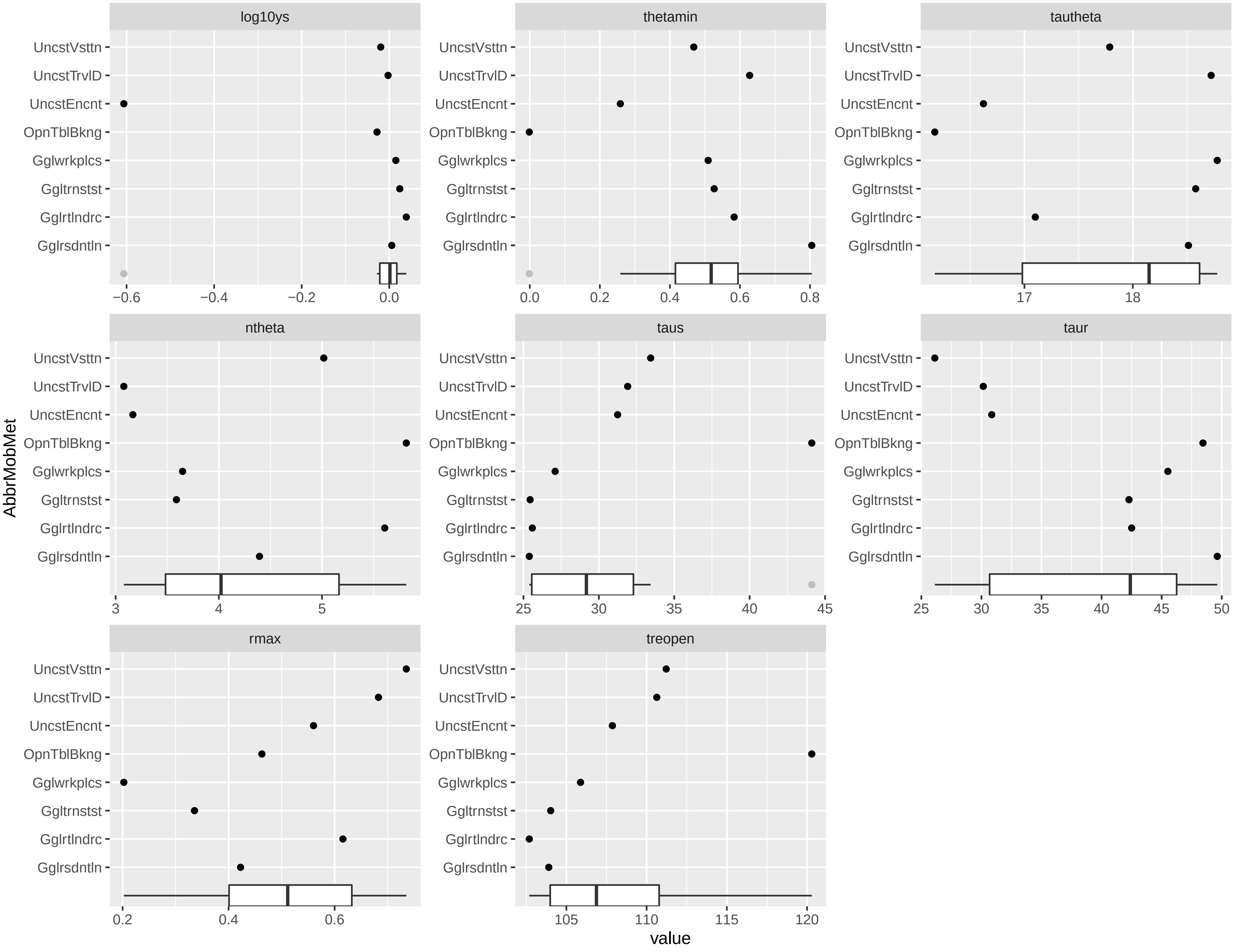

log10ys
thetamin
tautheta
ntheta
taus
taur
rmax
treopen
UncstVsttn
UncstTrvlD
UncstEncnt
OpnTblBkng
Gglwrkplcs
Ggltrnstst
Gglrtlndrc
Gglrsdntln
AbbrMobMet
value
−0.6
−0.4
−0.2
0.0
0.2
0.4
0.6
0.8
17
18
3
4
5
25
30
35
40
45
50
105
110
115
120

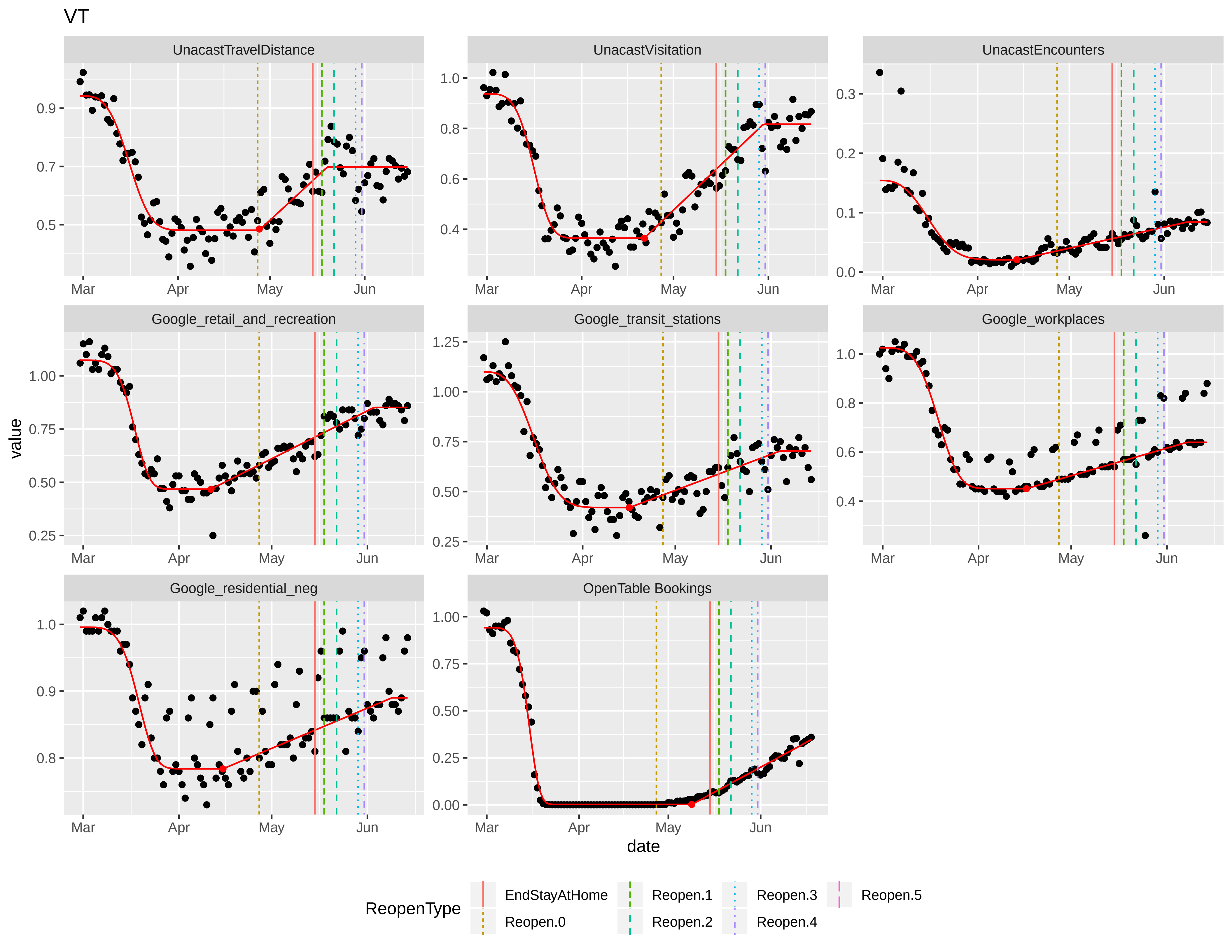

VT
UnacastTravelDistance
UnacastVisitation
UnacastEncounters
Google_retail_and_recreation
Google_transit_stations
Google_workplaces
Google_residential_neg
OpenTable Bookings
value
date
Mar
Apr
May
Jun
ReopenType
EndStayAtHome
Reopen.0
Reopen.1
Reopen.2
Reopen.3
Reopen.4
Reopen.5

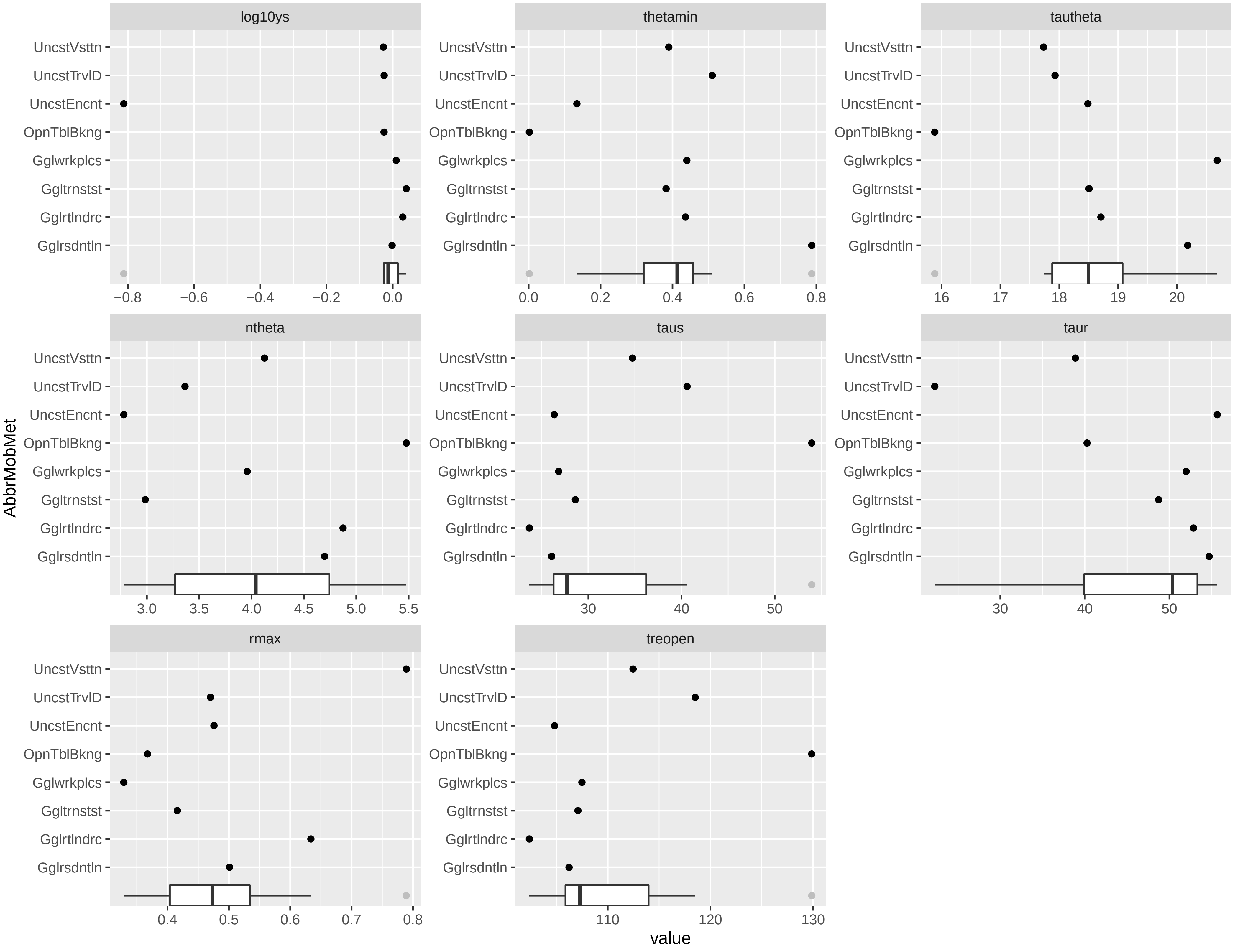

log10ys
thetamin
tautheta
ntheta
taus
taur
rmax
treopen
UncstVsttn
UncstTrvlD
UncstEncnt
OpnTblBkng
Gglwrkplcs
Ggltrnstst
Gglrtlndrc
Gglrsdntln
AbbrMobMet
value

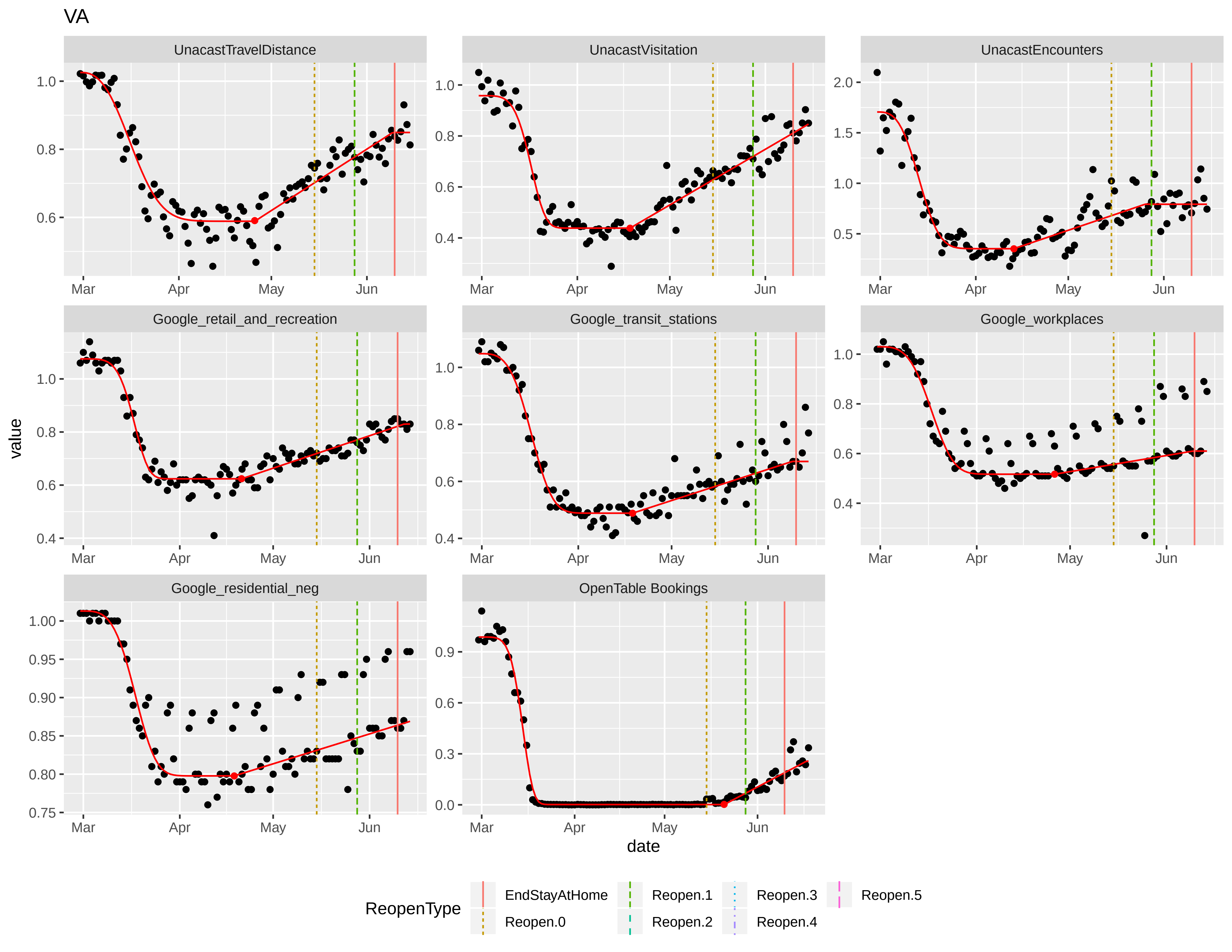

VA
UnacastTravelDistance
UnacastVisitation
UnacastEncounters
Google_retail_and_recreation
Google_transit_stations
Google_workplaces
Google_residential_neg
OpenTable Bookings
value
date
Mar
Apr
May
Jun
ReopenType
EndStayAtHome
Reopen.0
Reopen.1
Reopen.2
Reopen.3
Reopen.4
Reopen.5

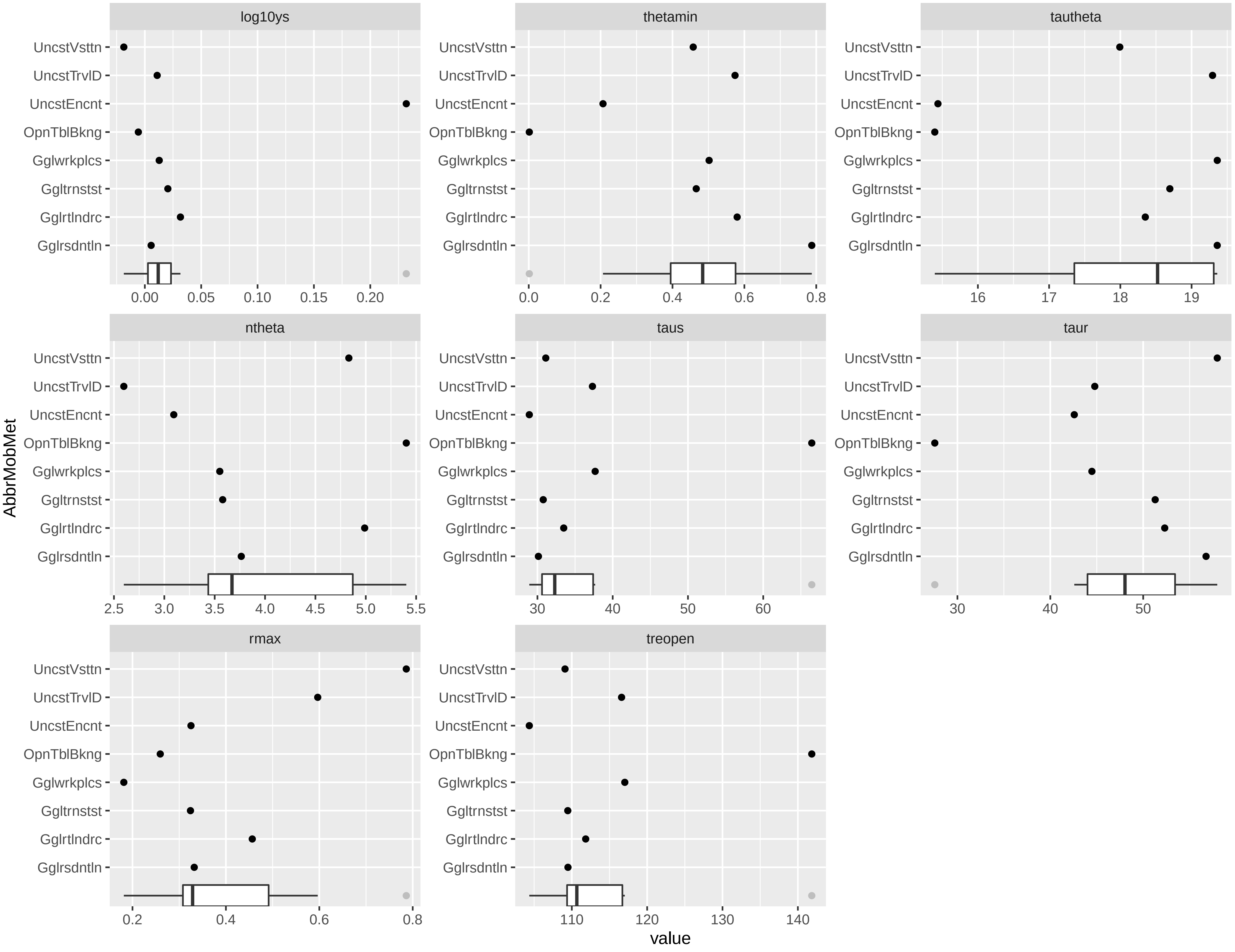

log10ys
thetamin
tautheta
ntheta
taus
taur
rmax
treopen
UncstVsttn
UncstTrvlD
UncstEncnt
OpnTblBkng
Gglwrkplcs
Ggltrnstst
Gglrtlndrc
Gglrsdntln
AbbrMobMet
value

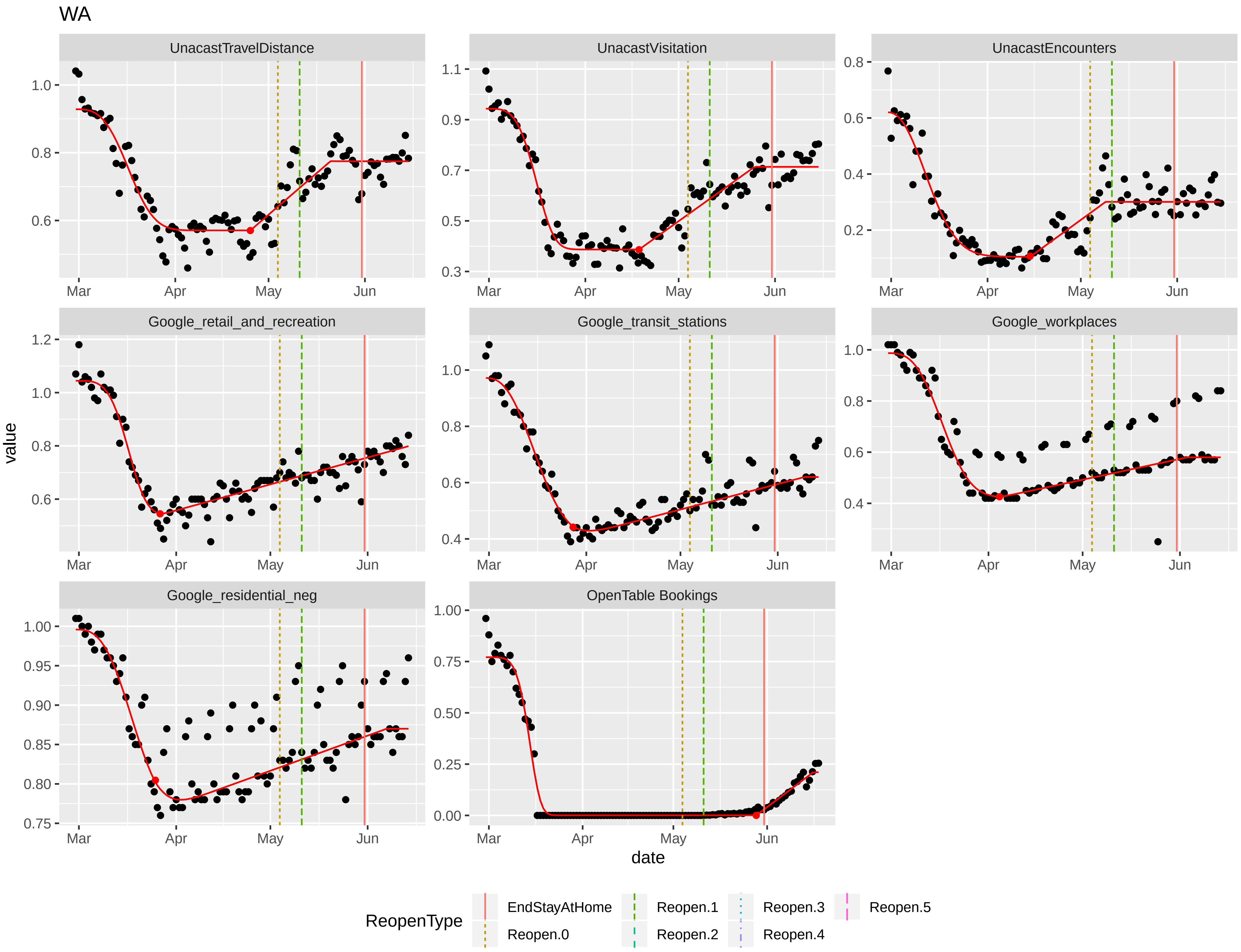
WA
UnacastTravelDistance
UnacastVisitation
UnacastEncounters
Google_retail_and_recreation
Google_transit_stations
Google_workplaces
Google_residential_neg
OpenTable Bookings
value
date
Mar
Apr
May
Jun
ReopenType
EndStayAtHome
Reopen.0
Reopen.1
Reopen.2
Reopen.3
Reopen.4
Reopen.5

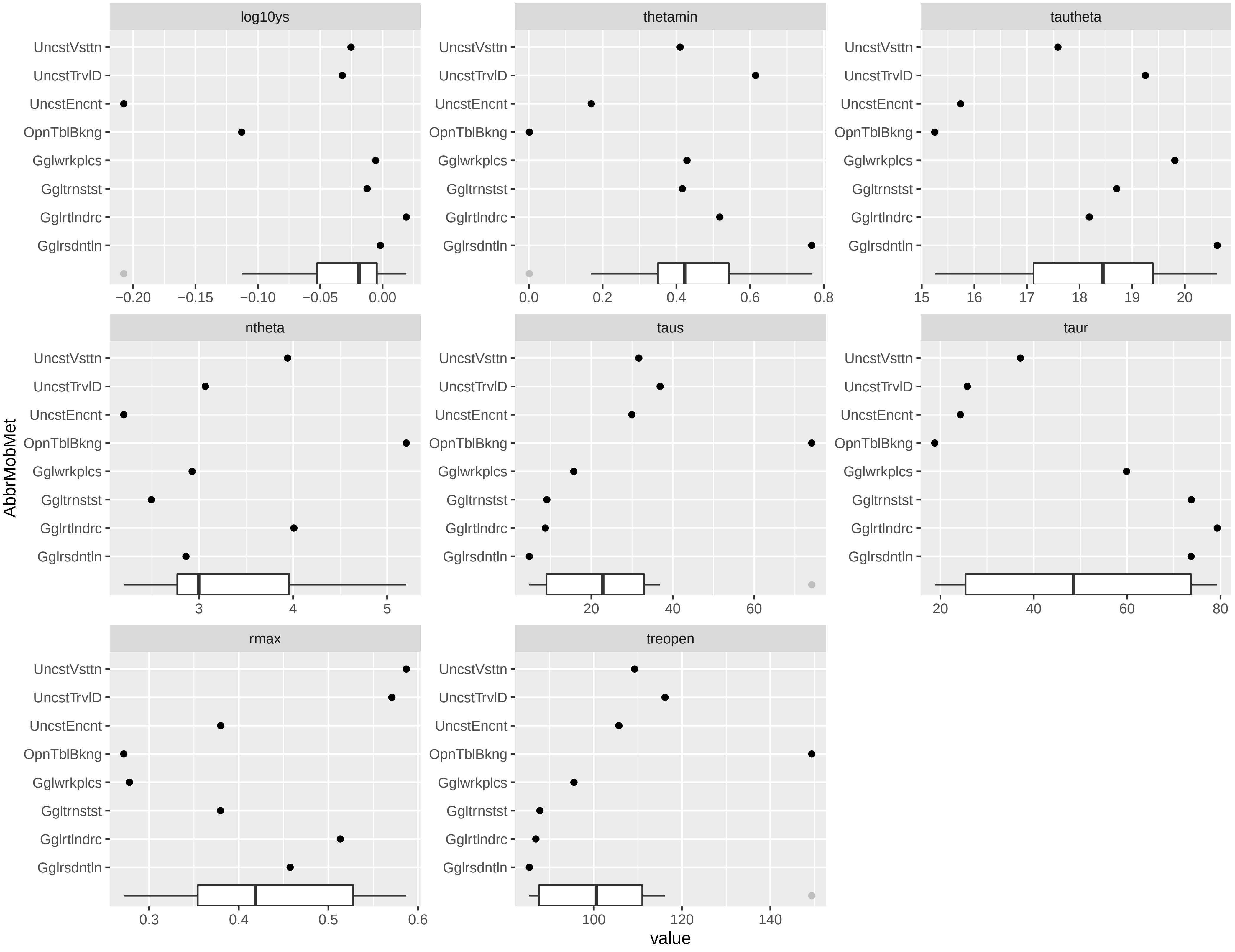

log10ys
thetamin
tautheta
ntheta
taus
taur
rmax
treopen
UncstVsttn
UncstTrvlD
UncstEncnt
OpnTblBkng
Gglwrkplcs
Ggltrnstst
Gglrtlndrc
Gglrsdntln
−0.20
−0.15
−0.10
−0.05
0.00
0.0
0.2
0.4
0.6
0.8
15
16
17
18
19
20
3
4
5
20
40
60
20
40
60
80
0.3
0.4
0.5
0.6
100
120
140
AbbrMobMet
value

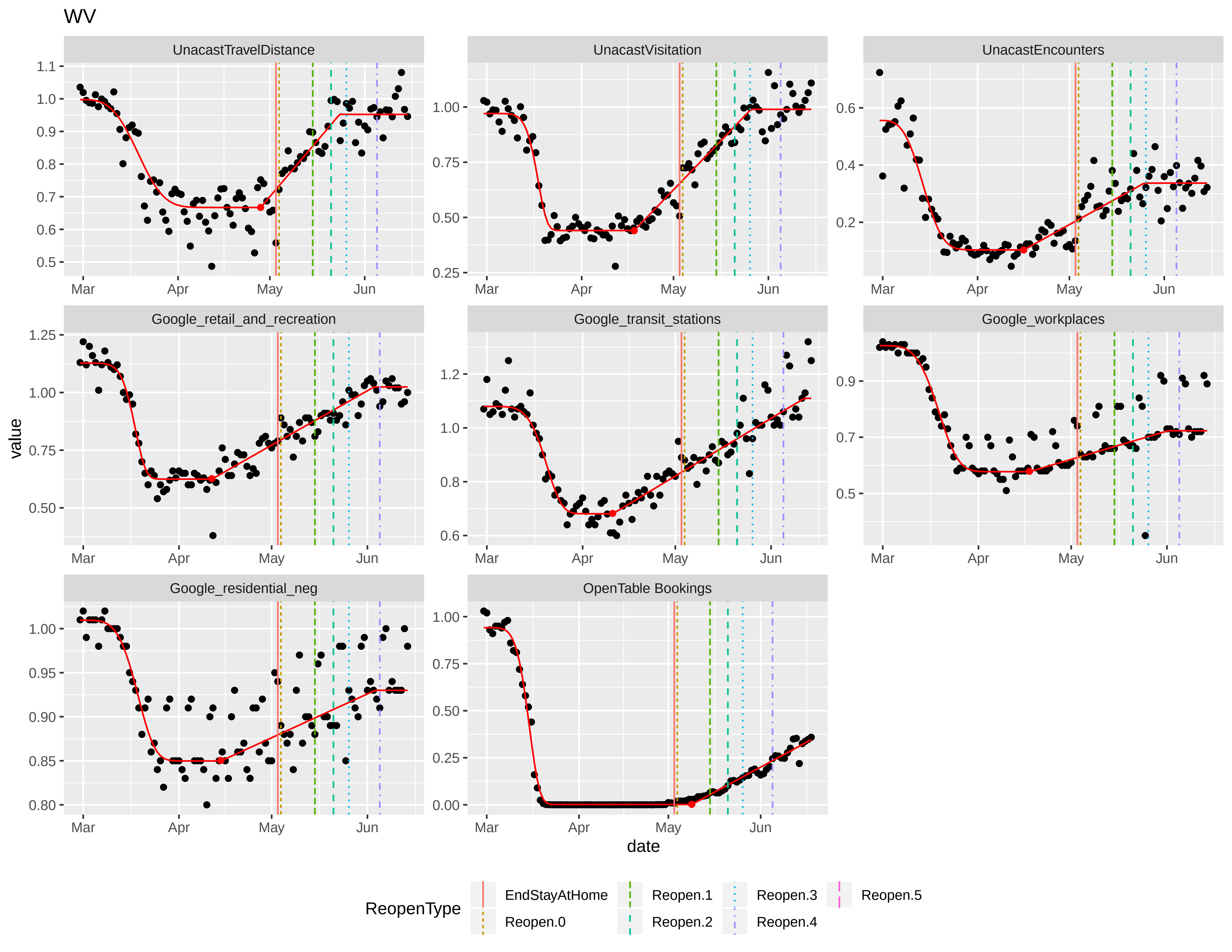

WV
UnacastTravelDistance
UnacastVisitation
UnacastEncounters
Google_retail_and_recreation
Google_transit_stations
Google_workplaces
Google_residential_neg
OpenTable Bookings
value
date
Mar
Apr
May
Jun
ReopenType
EndStayAtHome
Reopen.0
Reopen.1
Reopen.2
Reopen.3
Reopen.4
Reopen.5

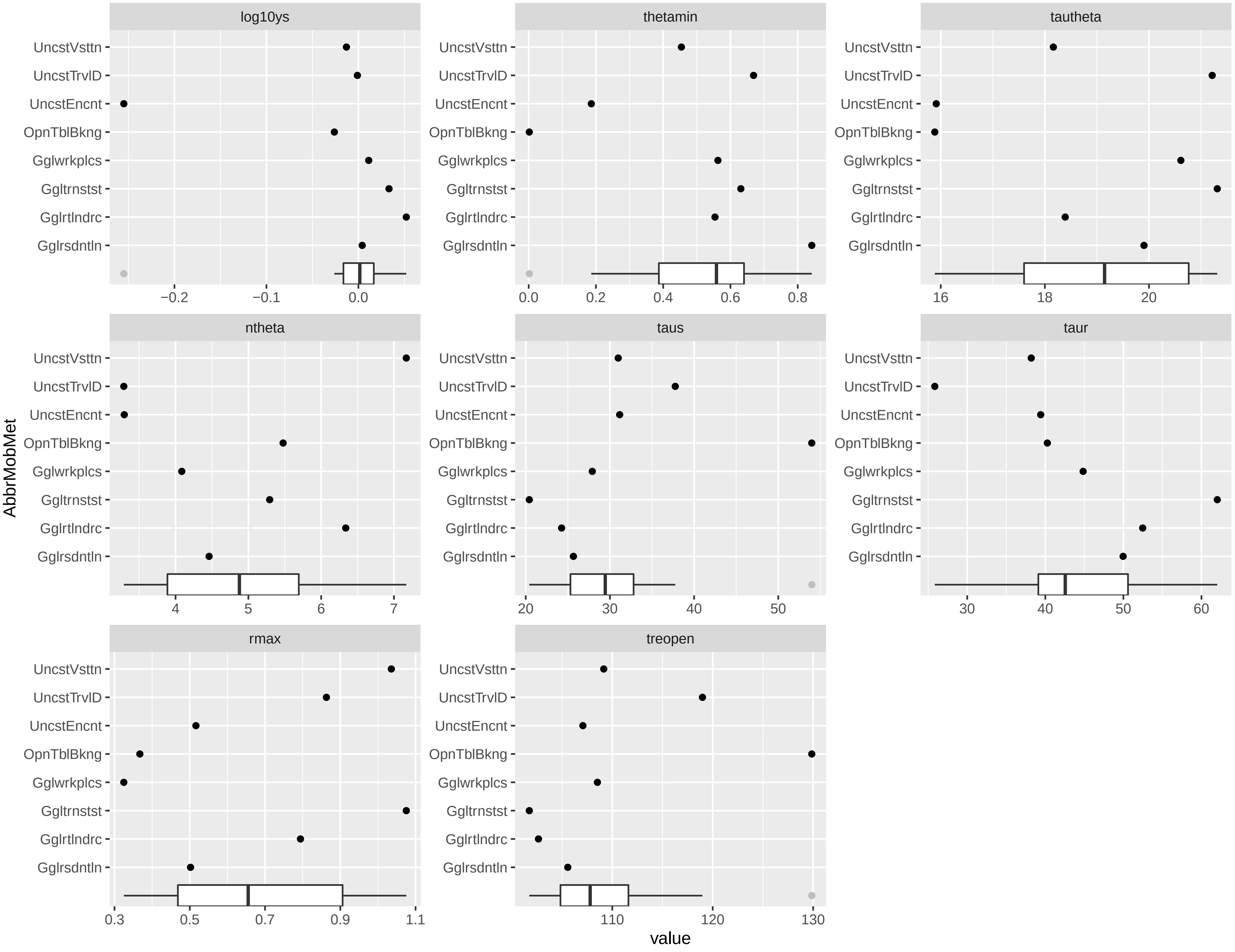

log10ys
thetamin
tautheta
ntheta
taus
taur
rmax
treopen
UncstVsttn
UncstTrvlD
UncstEncnt
OpnTblBkng
Gglwrkplcs
Ggltrnstst
Gglrtlndrc
Gglrsdntln
−0.2
−0.1
0.0
0.2
0.4
0.6
0.8
16
18
20
4
5
6
7
20
30
40
50
30
40
50
60
0.3
0.5
0.7
0.9
1.1
110
120
130
AbbrMobMet
value

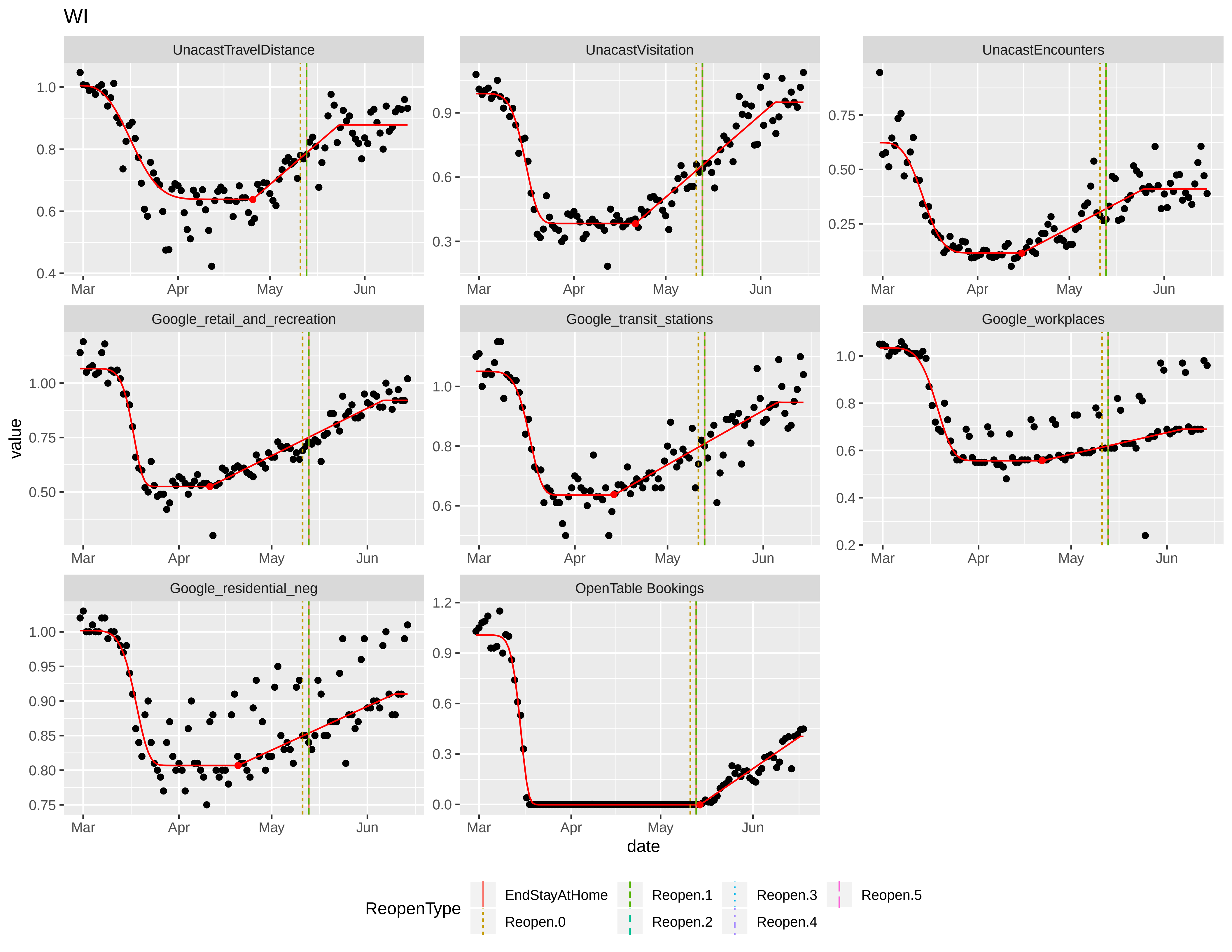
WI
UnacastTravelDistance
UnacastVisitation
UnacastEncounters
Google_retail_and_recreation
Google_transit_stations
Google_workplaces
Google_residential_neg
OpenTable Bookings
value
date
Mar
Apr
May
Jun
ReopenType
EndStayAtHome
Reopen.0
Reopen.1
Reopen.2
Reopen.3
Reopen.4
Reopen.5

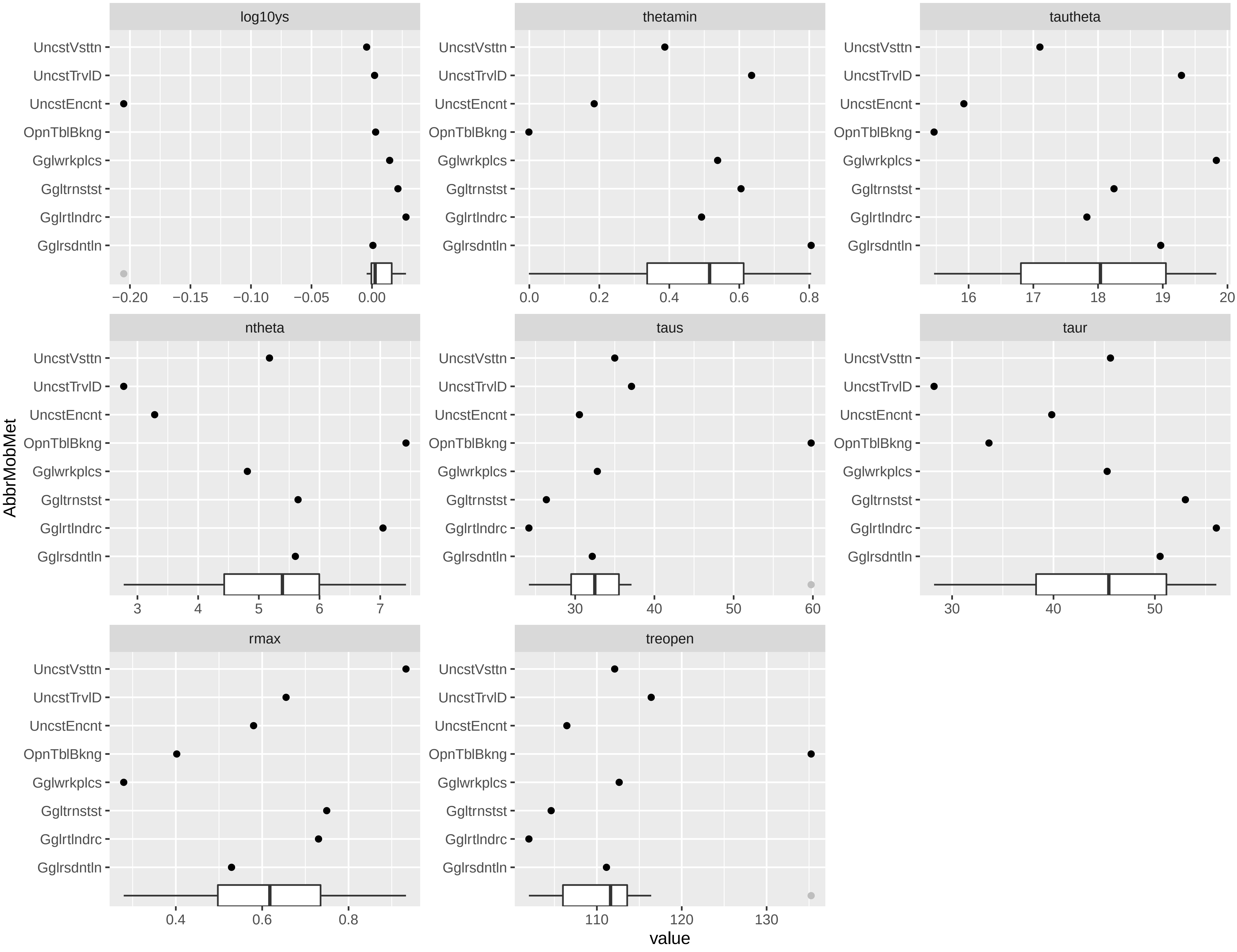

log10ys
thetamin
tautheta
ntheta
taus
taur
rmax
treopen
UncstVsttn
UncstTrvlD
UncstEncnt
OpnTblBkng
Gglwrkplcs
Ggltrnstst
Gglrtlndrc
Gglrsdntln
AbbrMobMet
value

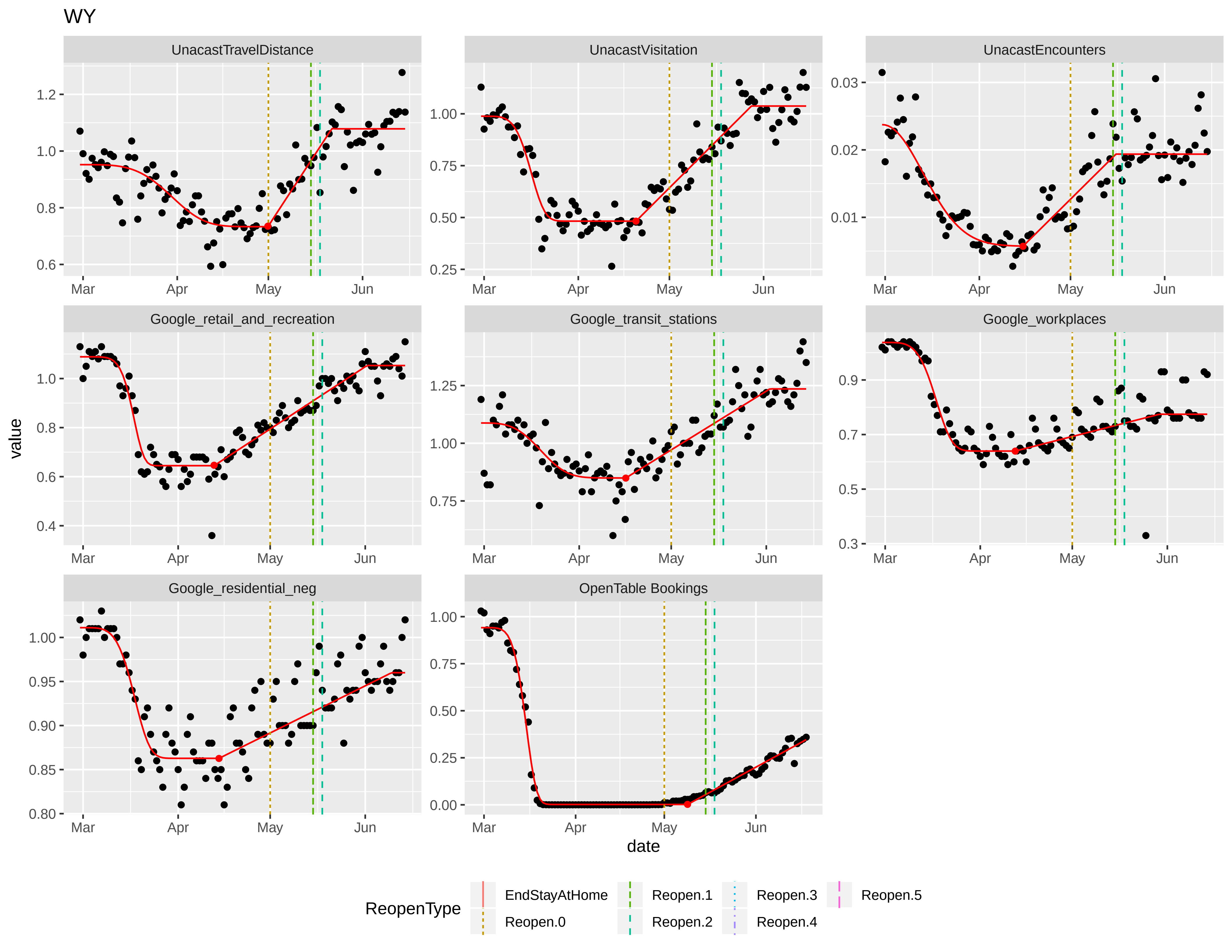

WY
UnacastTravelDistance
UnacastVisitation
UnacastEncounters
Google_retail_and_recreation
Google_transit_stations
Google_workplaces
Google_residential_neg
OpenTable Bookings
value
date
Mar
Apr
May
Jun
ReopenType
EndStayAtHome
Reopen.0
Reopen.1
Reopen.2
Reopen.3
Reopen.4
Reopen.5

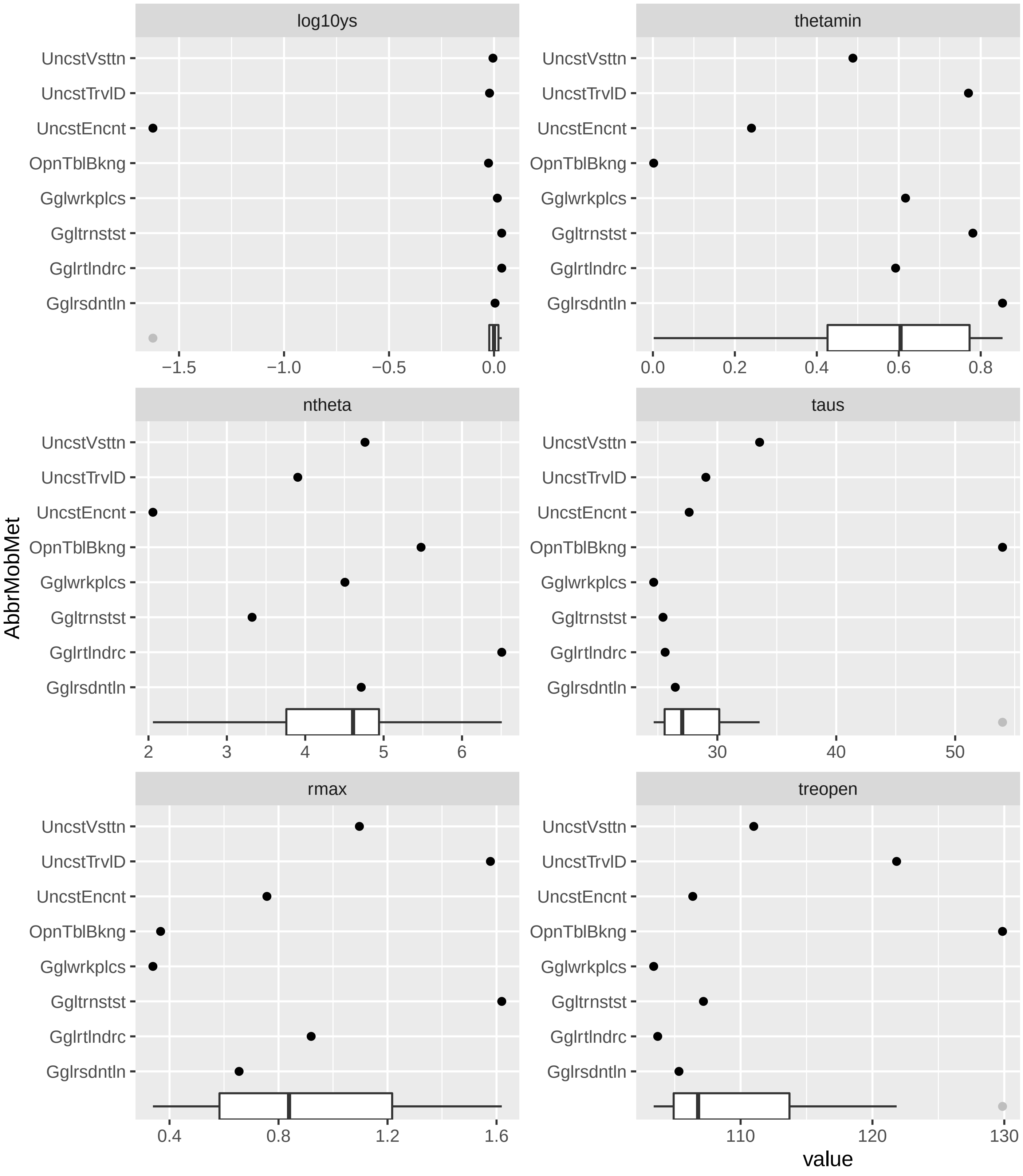

log10ys
thetamin
tautheta
ntheta
taus
taur
rmax
treopen
UncstVsttn
UncstTrvlD
UncstEncnt
OpnTblBkng
Gglwrkplcs
Ggltrnstst
Gglrtlndrc
Gglrsdntln
AbbrMobMet
value
