# Supplementary material for: State-level impact of social distancing and testing on COVID-19 in the United States
Source: Res Sq. 2020 Jul 7:rs.3.rs-40364. Preprint. [Version 1] doi: 10.21203/rs.3.rs-40364/v1 (PMC7362894; doi:10.21203/rs.3.rs-40364/v1)
Supplement: Supplementary file 4 [file FigS7PriorsPosteriorsbyState0620.pdf]

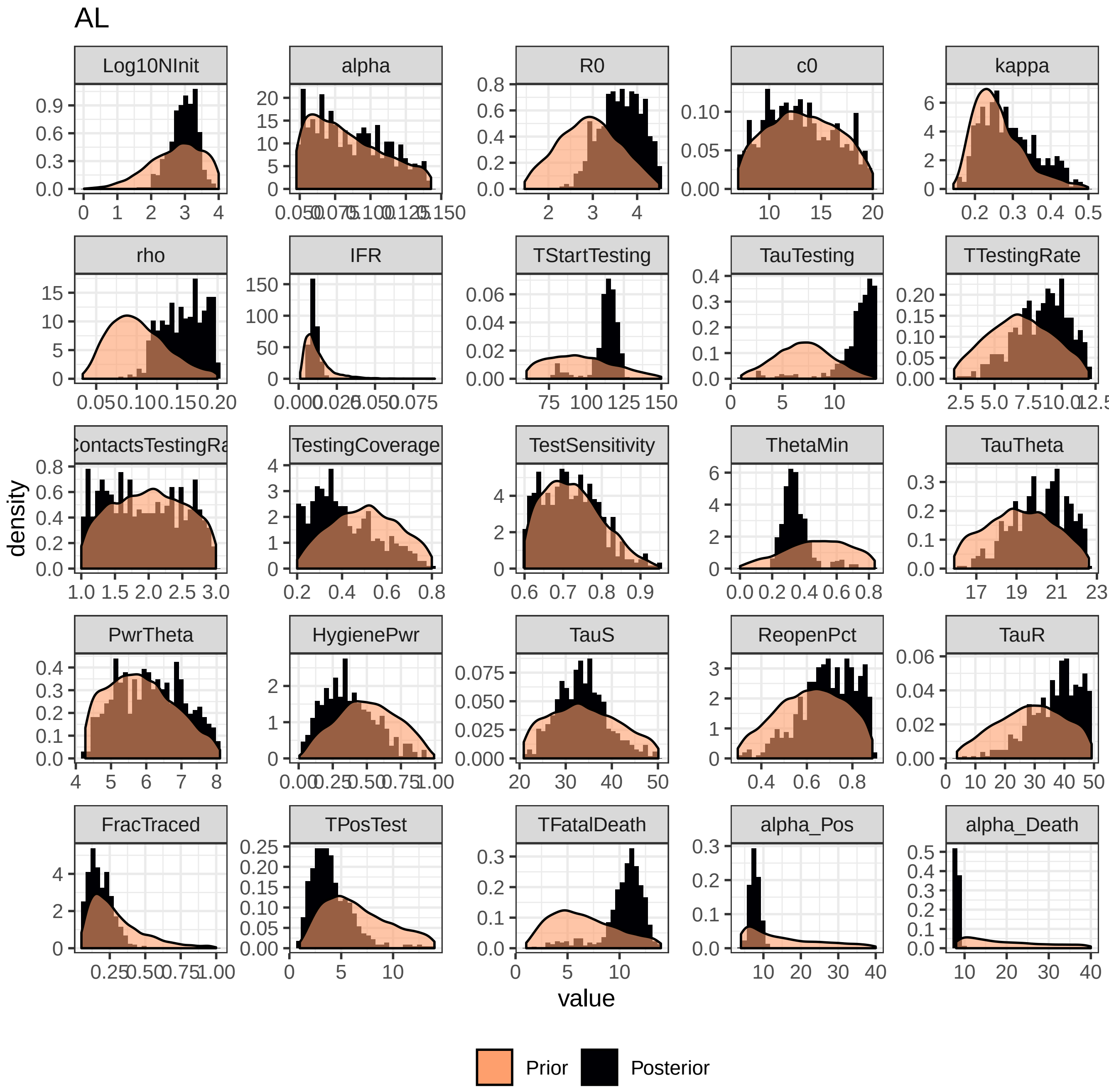

AL
Log10NInit
alpha
R0
c0
kappa
rho
IFR
TStartTesting
TauTesting
TTestingRate
ContactsTestingRa
TestingCoverage
TestSensitivity
ThetaMin
TauTheta
PwrTheta
HygienePwr
TauS
ReopenPct
TauR
FracTraced
TPosTest
TFatalDeath
alpha_Pos
alpha_Death
density
value
Prior
Posterior

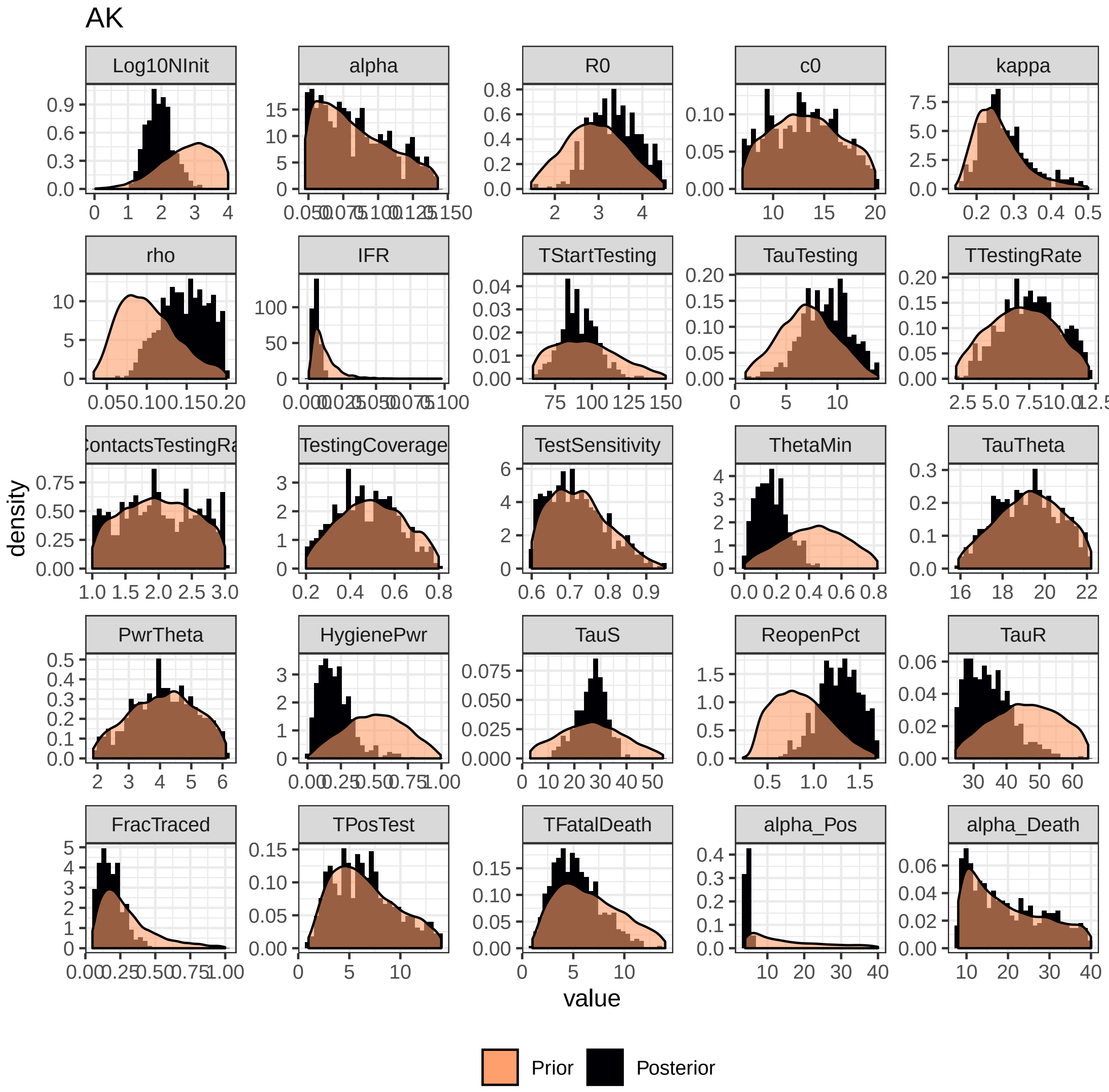
AK
Log10NInit
alpha
R0
c0
kappa
rho
IFR
TStartTesting
TauTesting
TTestingRate
ContactsTestingRa
TestingCoverage
TestSensitivity
ThetaMin
TauTheta
PwrTheta
HygienePwr
TauS
ReopenPct
TauR
FracTraced
TPosTest
TFatalDeath
alpha_Pos
alpha_Death
density
value
Prior
Posterior

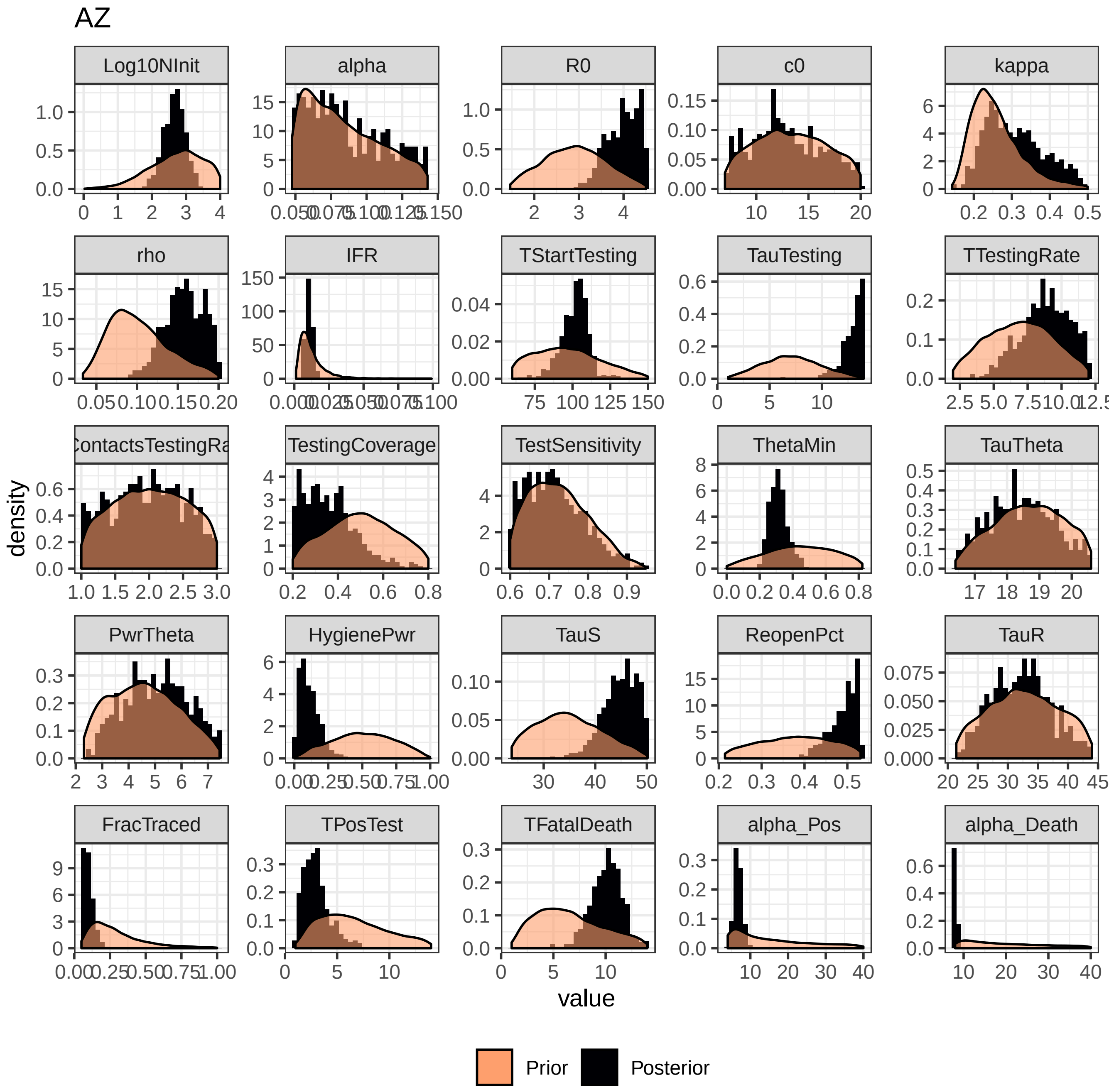
AZ
Log10NInit
alpha
R0
c0
kappa
rho
IFR
TStartTesting
TauTesting
TTestingRate
ContactsTestingRa
TestingCoverage
TestSensitivity
ThetaMin
TauTheta
PwrTheta
HygienePwr
TauS
ReopenPct
TauR
FracTraced
TPosTest
TFatalDeath
alpha_Pos
alpha_Death
density
value
Prior
Posterior

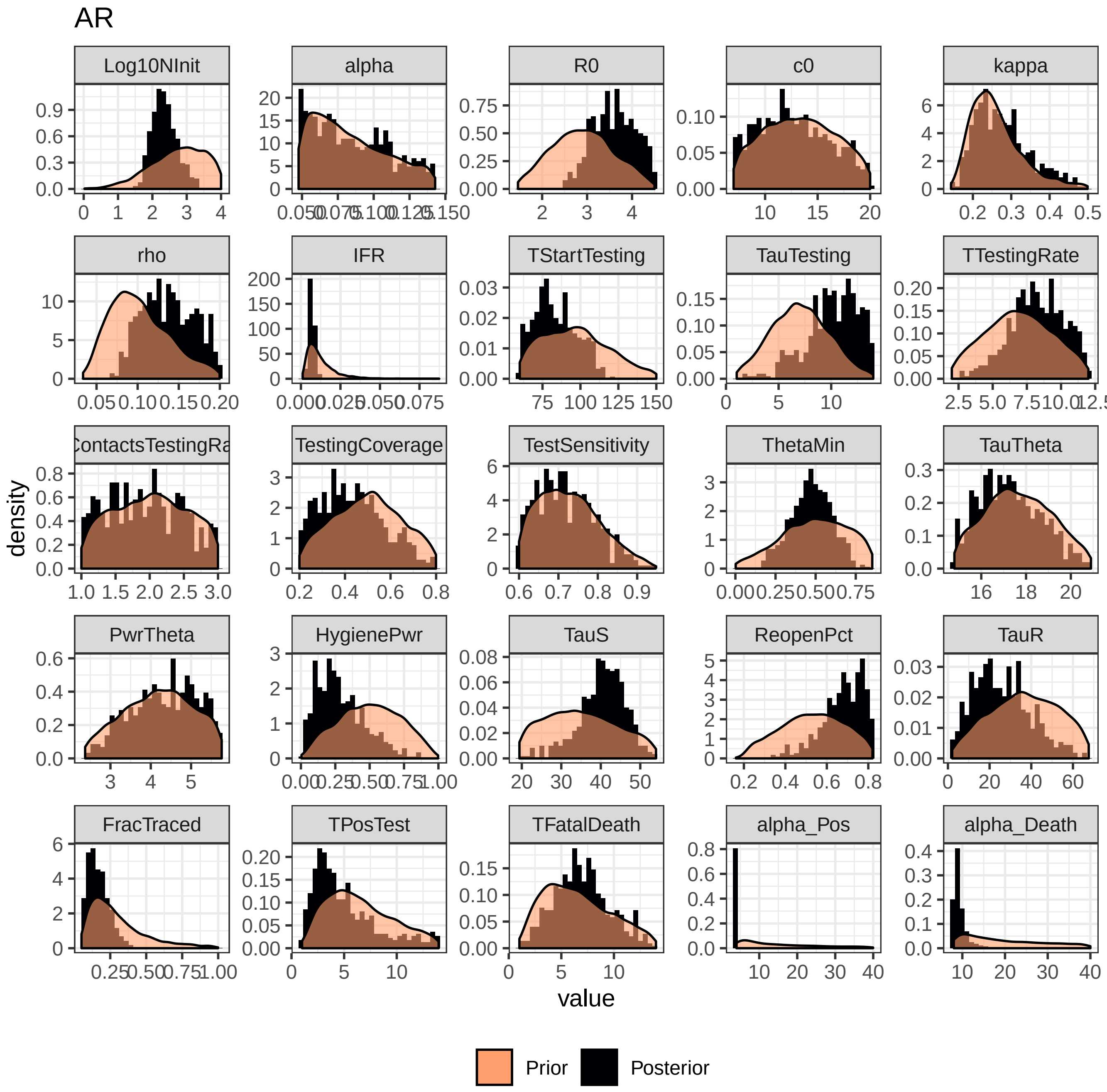
AR
Log10NInit
alpha
R0
c0
kappa
rho
IFR
TStartTesting
TauTesting
TTestingRate
ContactsTestingRa
TestingCoverage
TestSensitivity
ThetaMin
TauTheta
PwrTheta
HygienePwr
TauS
ReopenPct
TauR
FracTraced
TPosTest
TFatalDeath
alpha_Pos
alpha_Death
density
value
Prior
Posterior

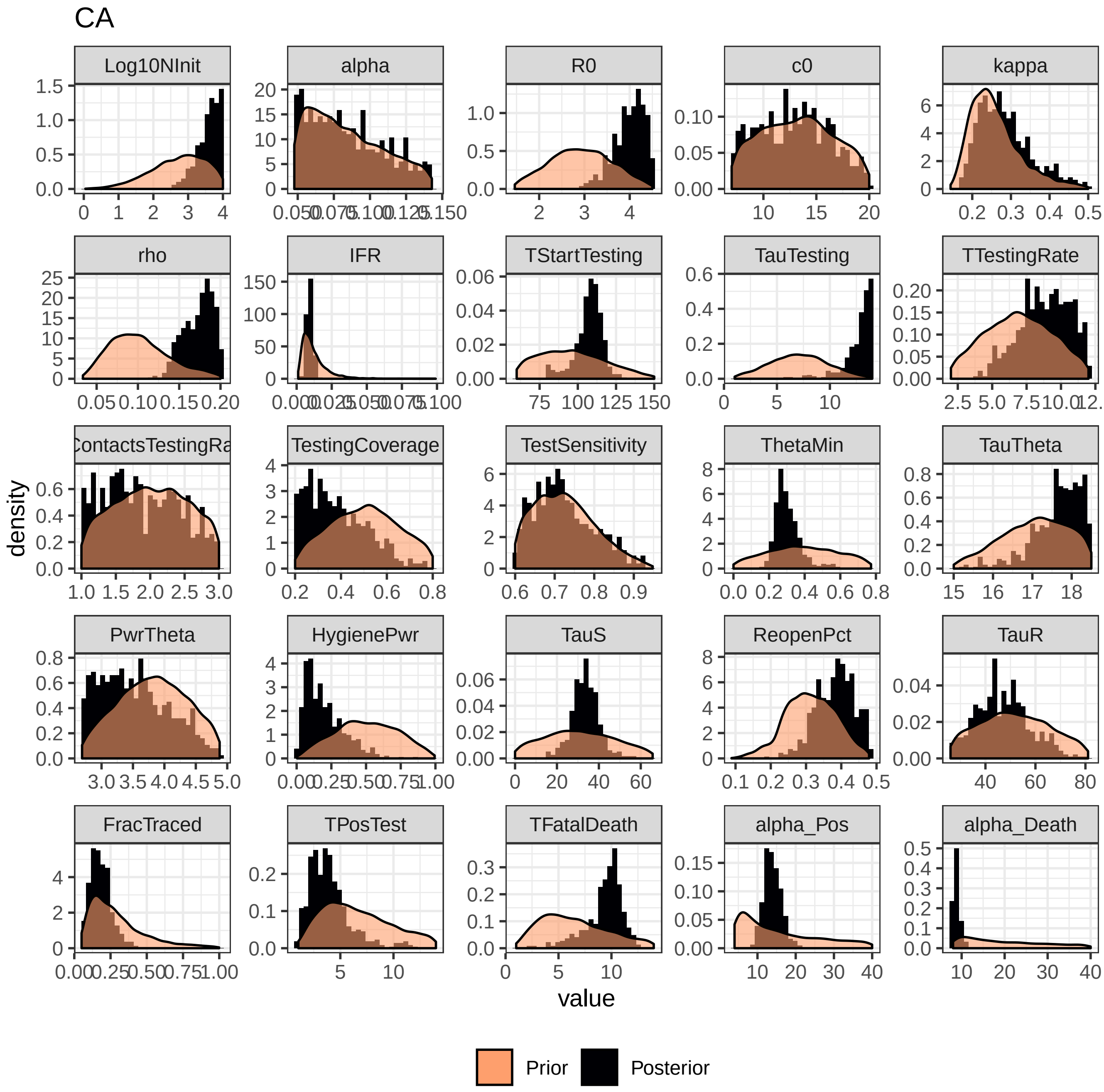
CA
Log10NInit
alpha
R0
c0
kappa
rho
IFR
TStartTesting
TauTesting
TTestingRate
ContactsTestingRa
TestingCoverage
TestSensitivity
ThetaMin
TauTheta
PwrTheta
HygienePwr
TauS
ReopenPct
TauR
FracTraced
TPosTest
TFatalDeath
alpha_Pos
alpha_Death
density
value
Prior
Posterior

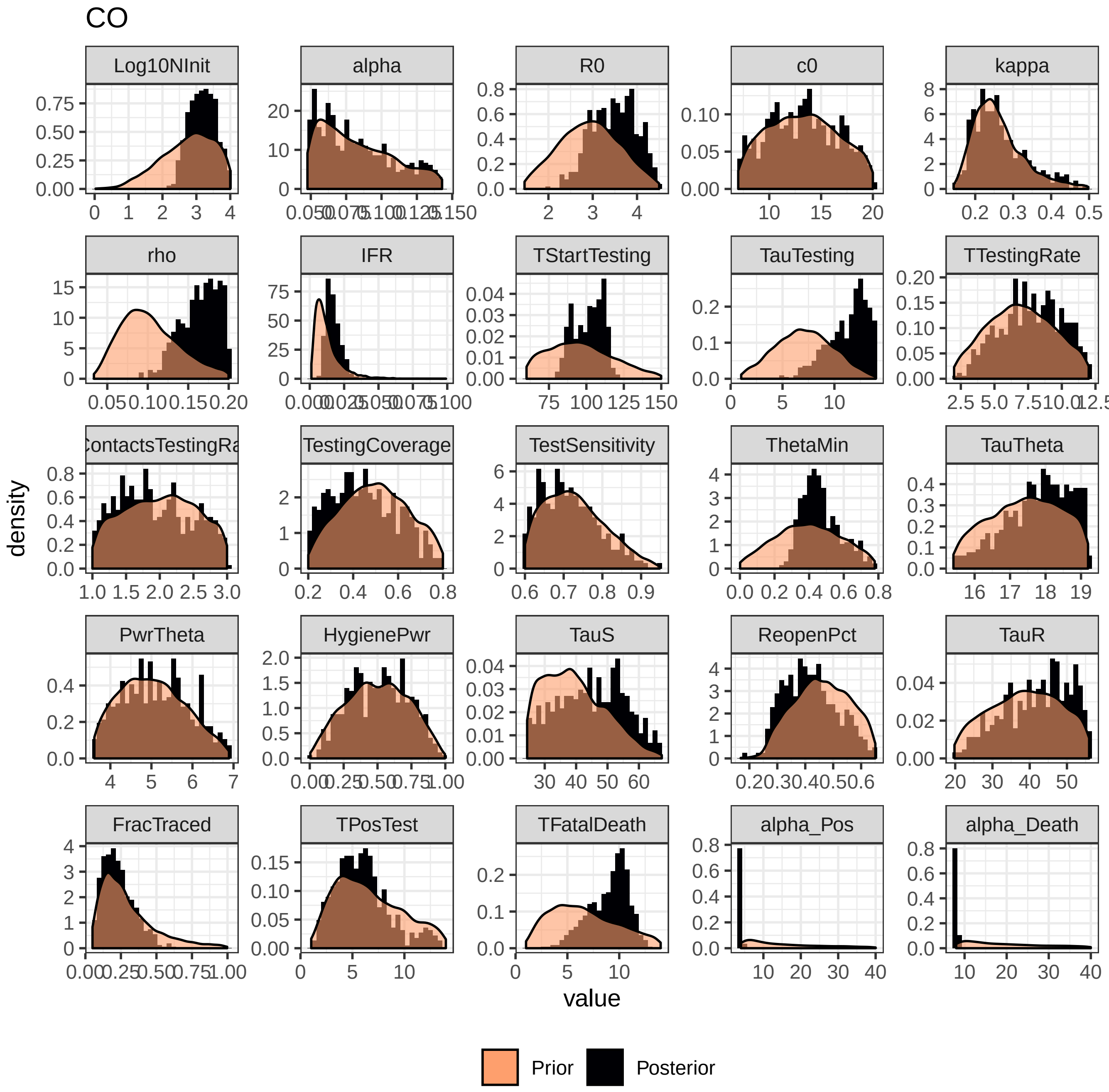
CO
Log10NInit
alpha
R0
c0
kappa
rho
IFR
TStartTesting
TauTesting
TTestingRate
ContactsTestingRa
TestingCoverage
TestSensitivity
ThetaMin
TauTheta
PwrTheta
HygienePwr
TauS
ReopenPct
TauR
FracTraced
TPosTest
TFatalDeath
alpha_Pos
alpha_Death
density
value
Prior
Posterior

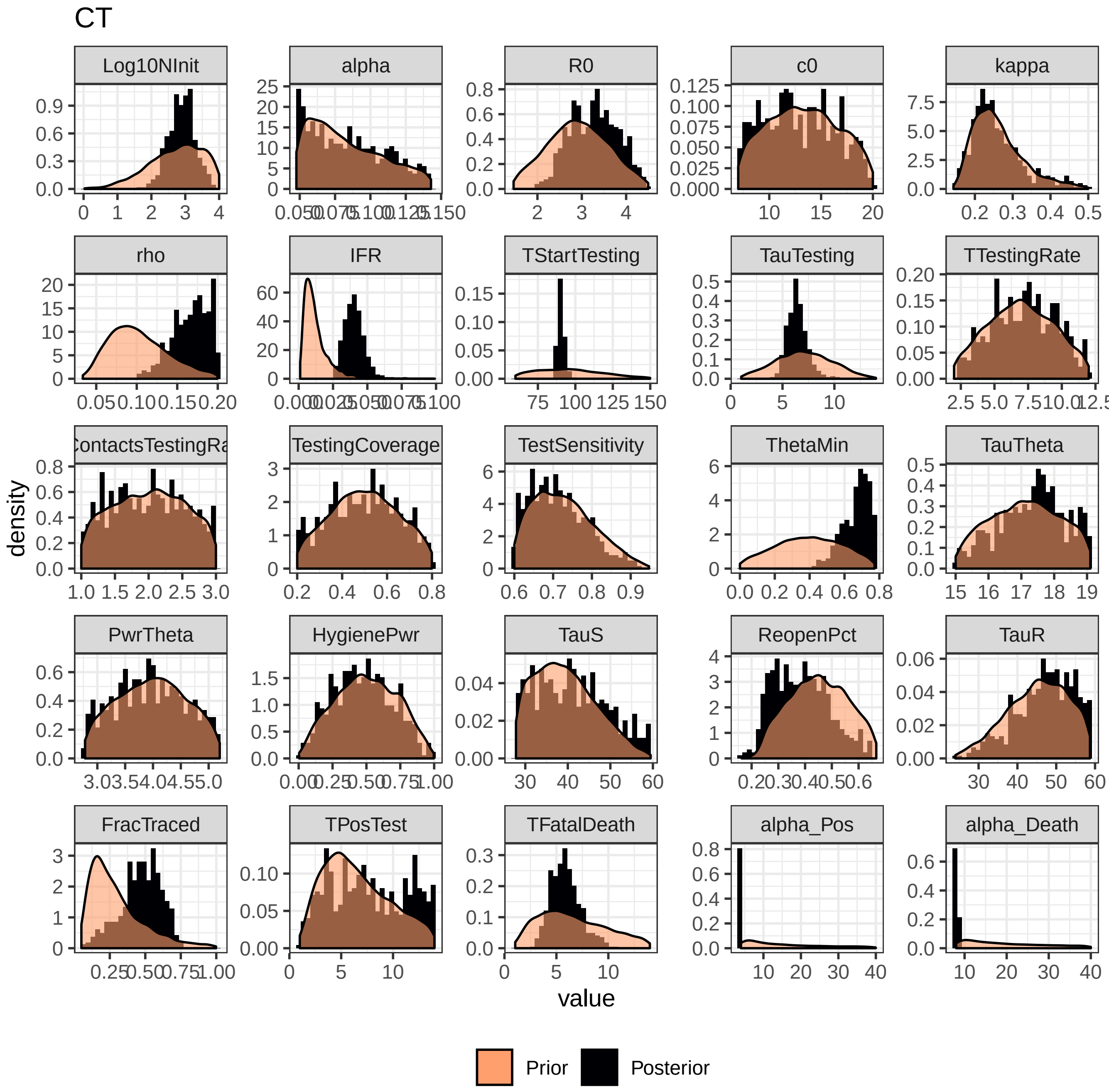
CT
Log10NInit
alpha
R0
c0
kappa
rho
IFR
TStartTesting
TauTesting
TTestingRate
ContactsTestingRa
TestingCoverage
TestSensitivity
ThetaMin
TauTheta
PwrTheta
HygienePwr
TauS
ReopenPct
TauR
FracTraced
TPosTest
TFatalDeath
alpha_Pos
alpha_Death
density
value
Prior
Posterior

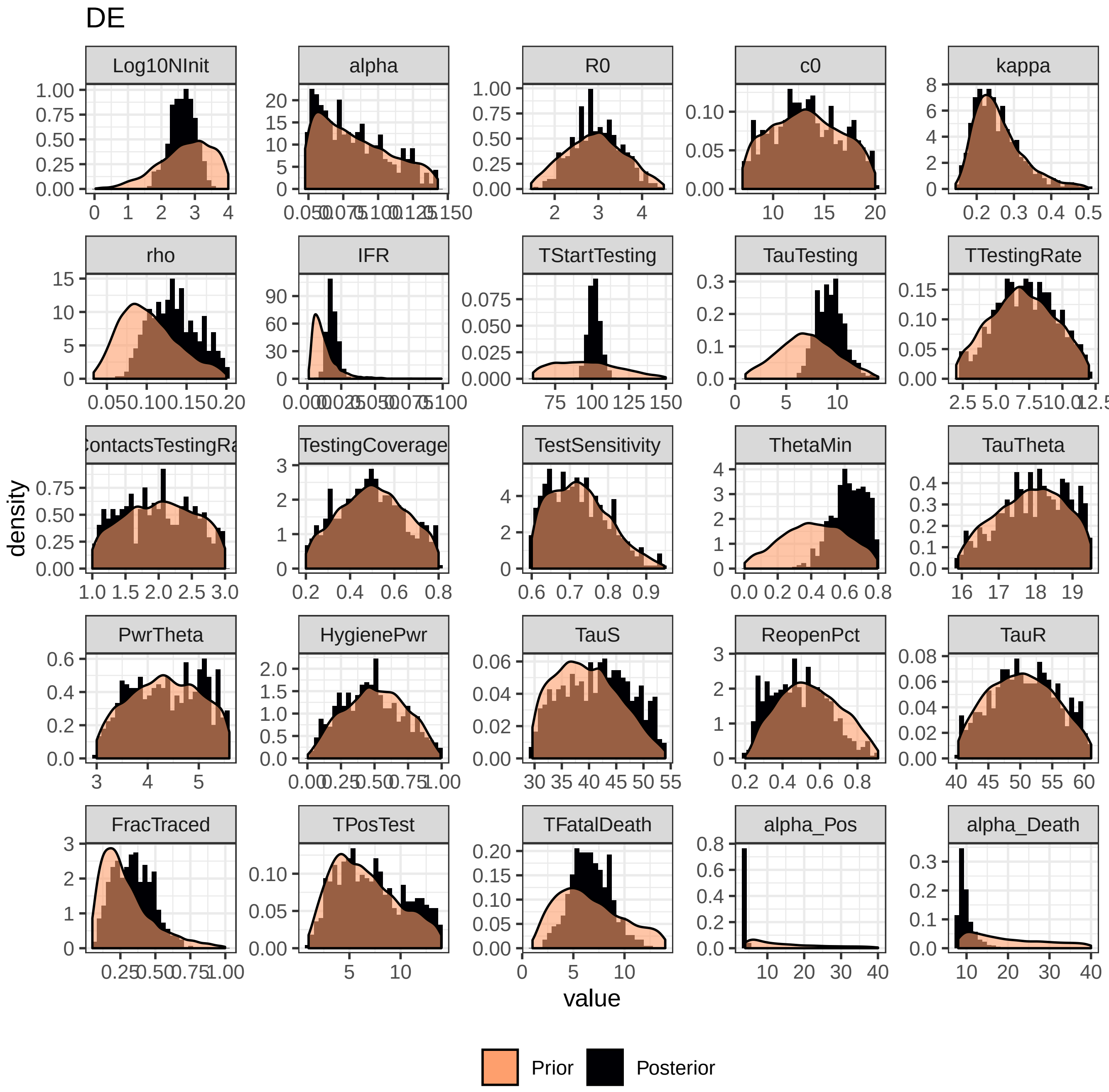

DE
Log10NInit
alpha
R0
c0
kappa
rho
IFR
TStartTesting
TauTesting
TTestingRate
ContactsTestingRa
TestingCoverage
TestSensitivity
ThetaMin
TauTheta
PwrTheta
HygienePwr
TauS
ReopenPct
TauR
FracTraced
TPosTest
TFatalDeath
alpha_Pos
alpha_Death
density
value
Prior
Posterior

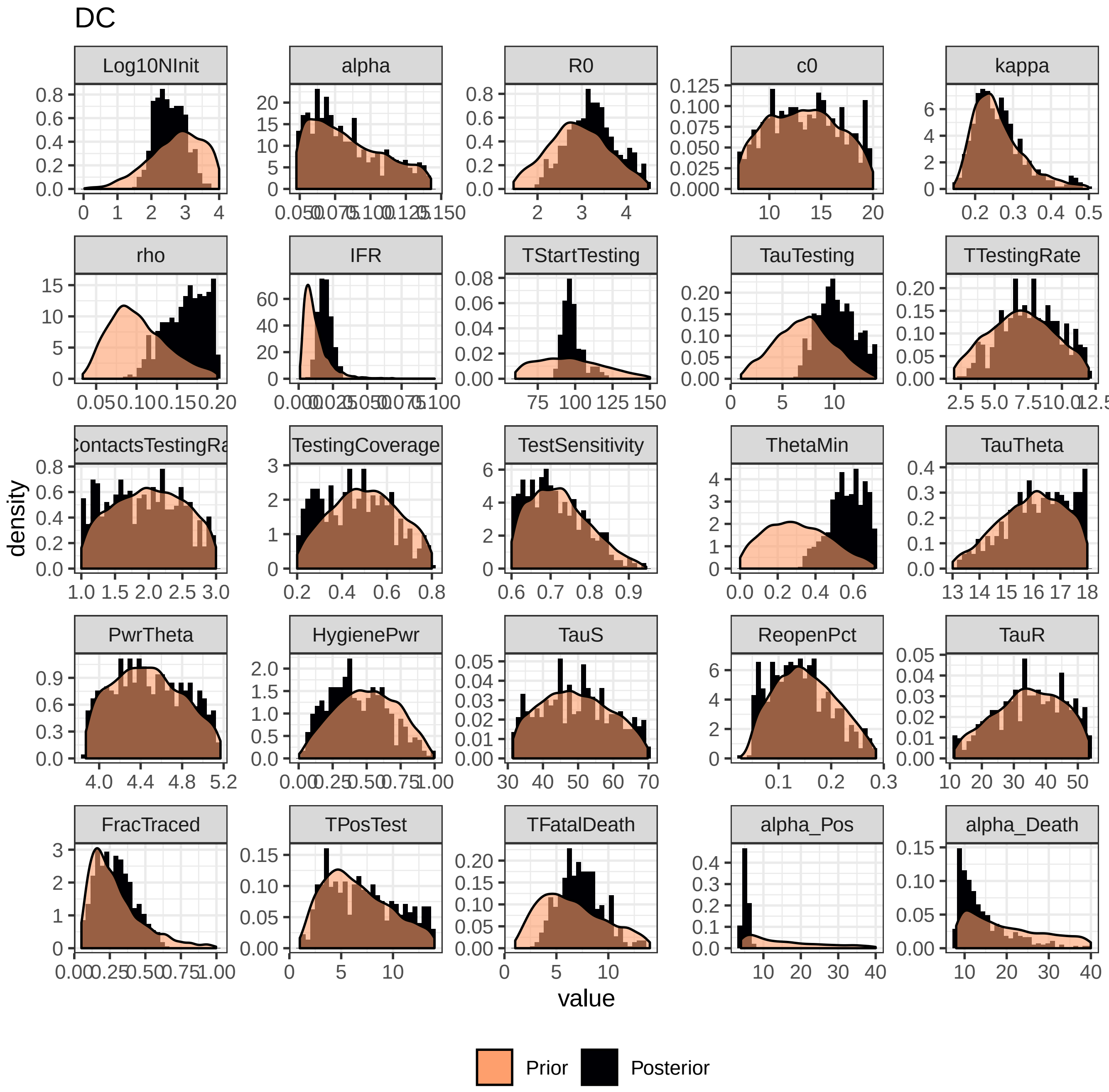
DC
Log10NInit
alpha
R0
c0
kappa
rho
IFR
TStartTesting
TauTesting
TTestingRate
ContactsTestingRa
TestingCoverage
TestSensitivity
ThetaMin
TauTheta
PwrTheta
HygienePwr
TauS
ReopenPct
TauR
FracTraced
TPosTest
TFatalDeath
alpha_Pos
alpha_Death
density
value
Prior
Posterior

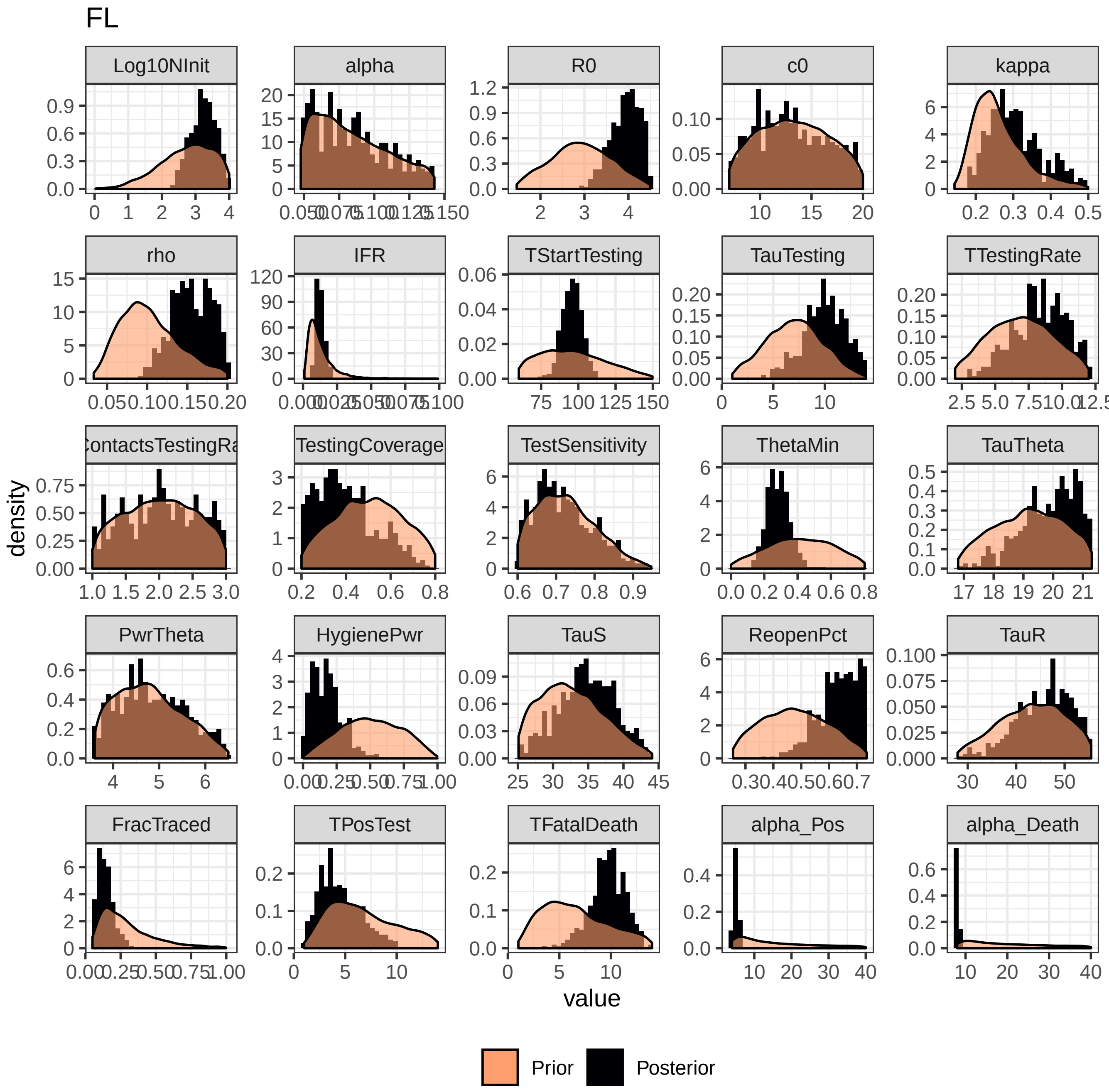

FL
Log10NInit
alpha
R0
c0
kappa
rho
IFR
TStartTesting
TauTesting
TTestingRate
ContactsTestingRa
TestingCoverage
TestSensitivity
ThetaMin
TauTheta
PwrTheta
HygienePwr
TauS
ReopenPct
TauR
FracTraced
TPosTest
TFatalDeath
alpha_Pos
alpha_Death
density
value
Prior
Posterior

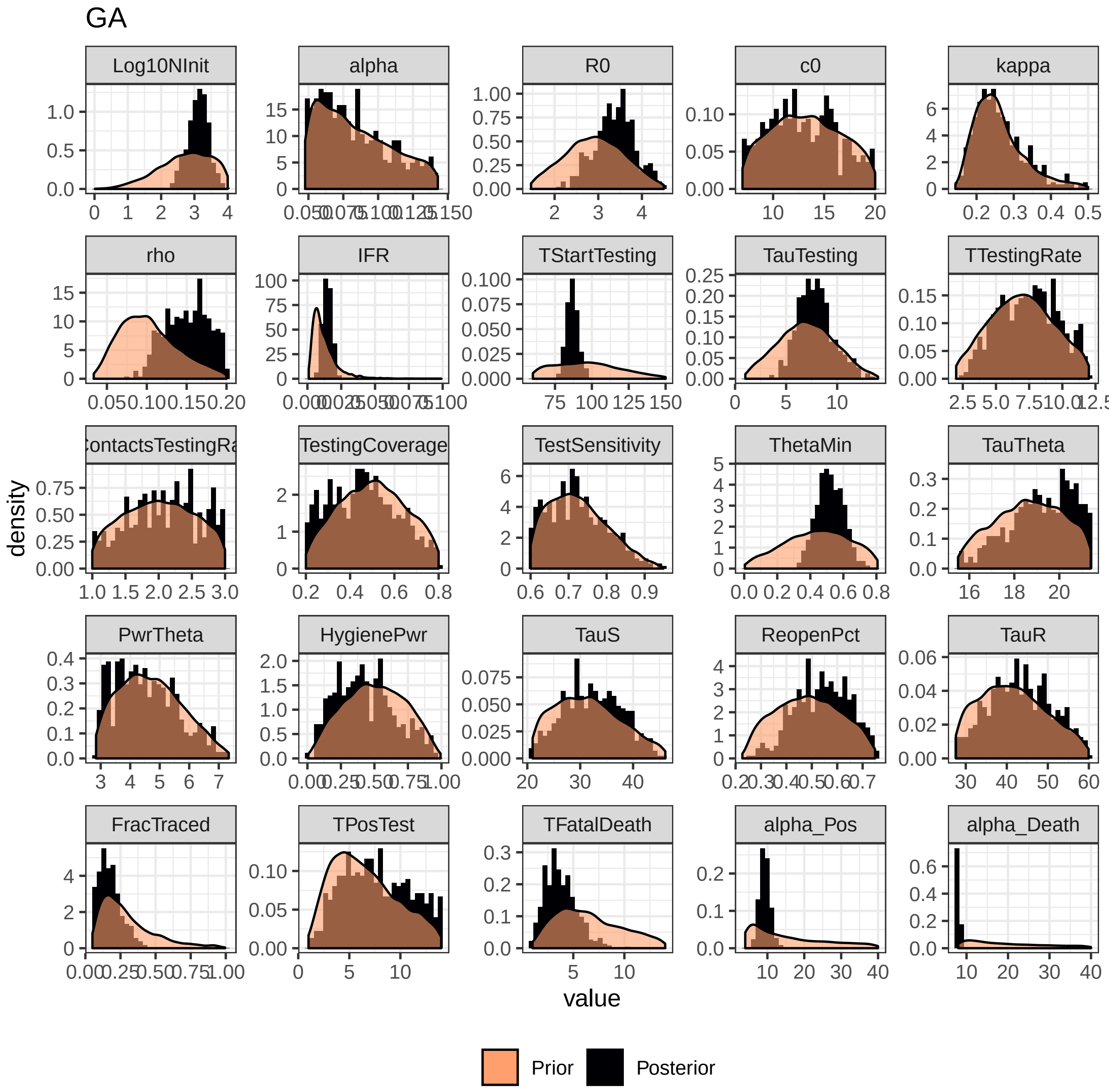
GA
Log10NInit
alpha
R0
c0
kappa
rho
IFR
TStartTesting
TauTesting
TTestingRate
ContactsTestingRa
TestingCoverage
TestSensitivity
ThetaMin
TauTheta
PwrTheta
HygienePwr
TauS
ReopenPct
TauR
FracTraced
TPosTest
TFatalDeath
alpha_Pos
alpha_Death
density
value
Prior
Posterior

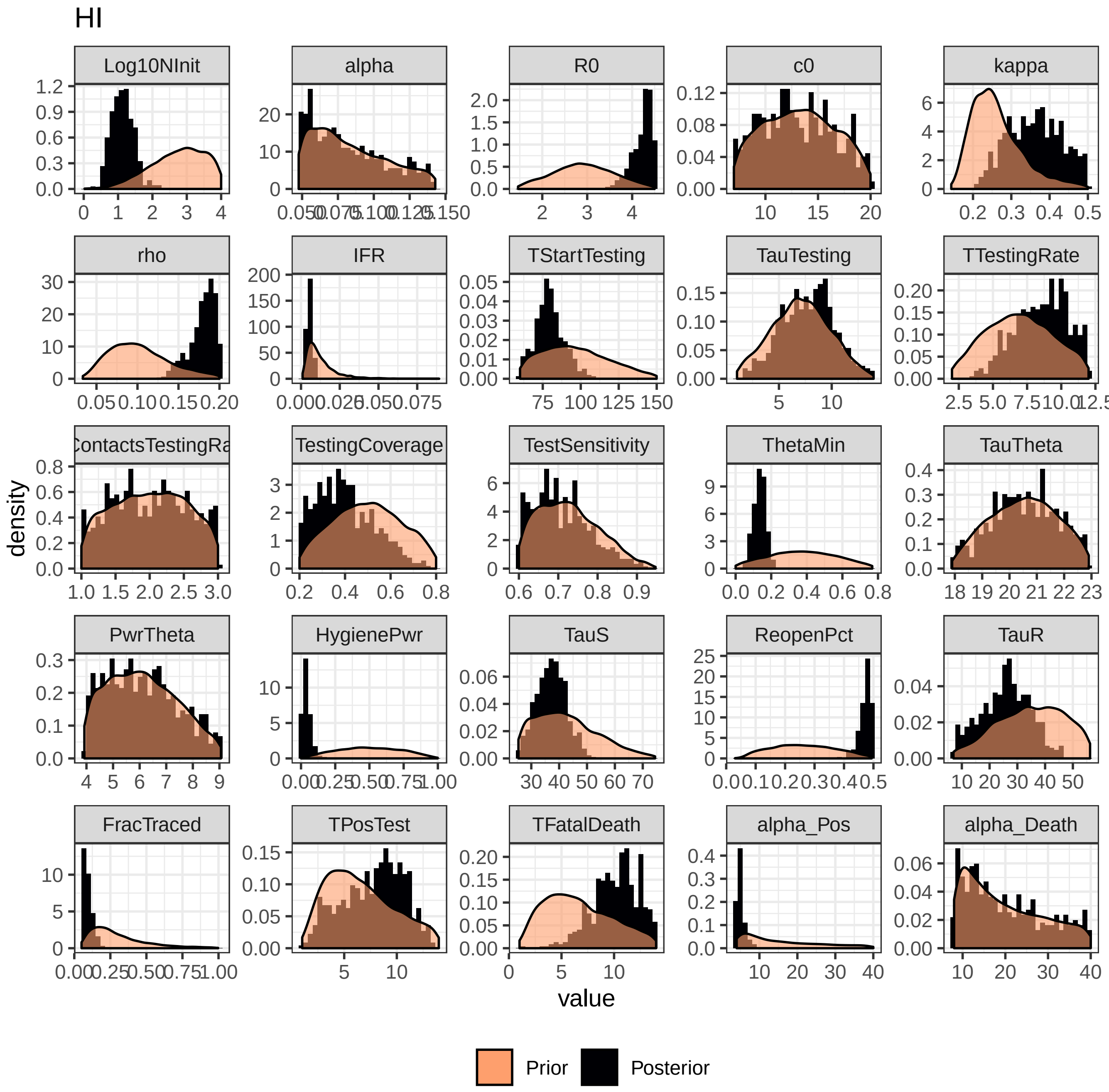

HI
Log10NInit
alpha
R0
c0
kappa
rho
IFR
TStartTesting
TauTesting
TTestingRate
ContactsTestingRa
TestingCoverage
TestSensitivity
ThetaMin
TauTheta
PwrTheta
HygienePwr
TauS
ReopenPct
TauR
FracTraced
TPosTest
TFatalDeath
alpha_Pos
alpha_Death
density
value
Prior
Posterior

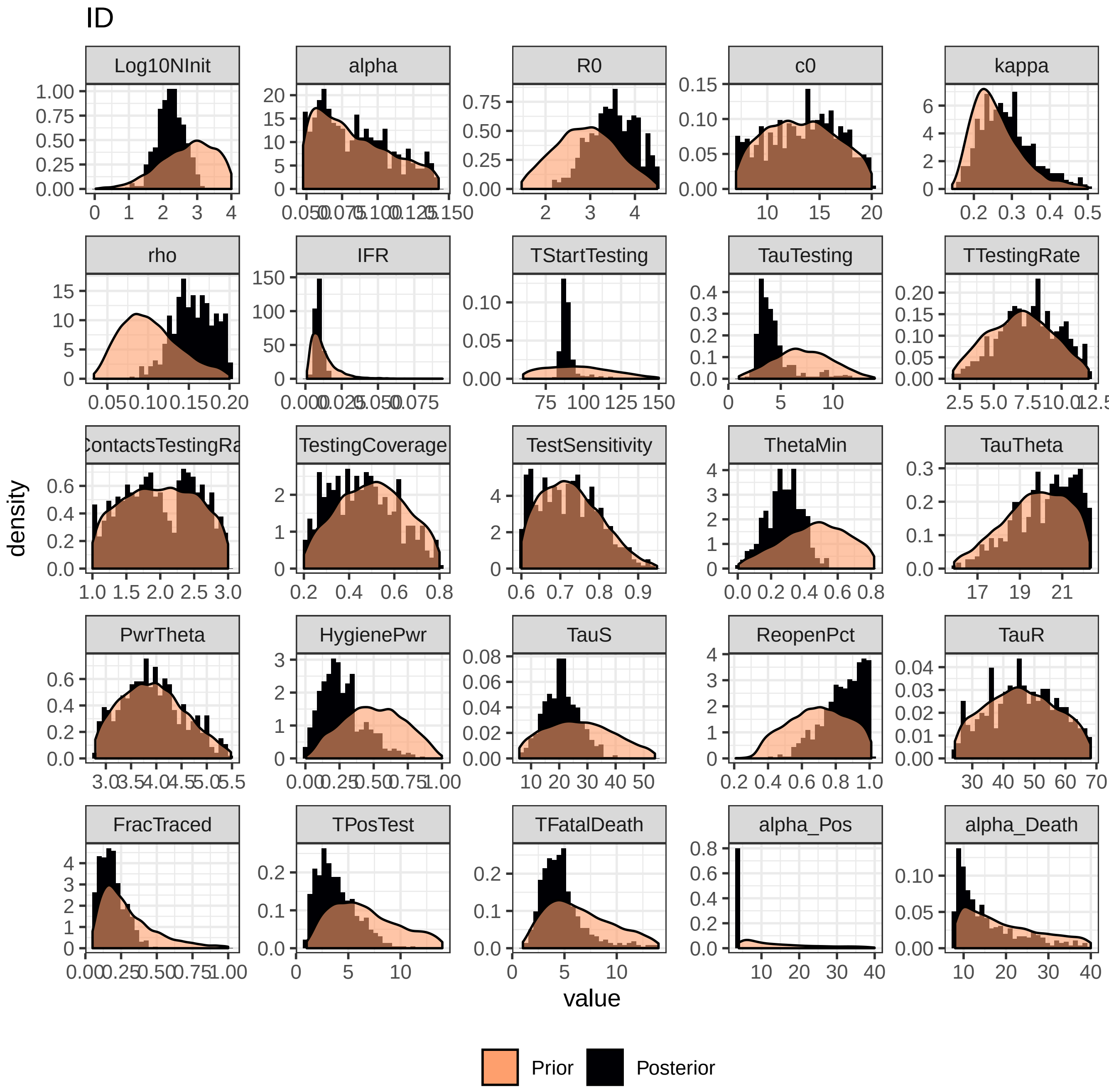
ID
Log10NInit
alpha
R0
c0
kappa
rho
IFR
TStartTesting
TauTesting
TTestingRate
ContactsTestingRa
TestingCoverage
TestSensitivity
ThetaMin
TauTheta
PwrTheta
HygienePwr
TauS
ReopenPct
TauR
FracTraced
TPosTest
TFatalDeath
alpha_Pos
alpha_Death
density
value
Prior
Posterior

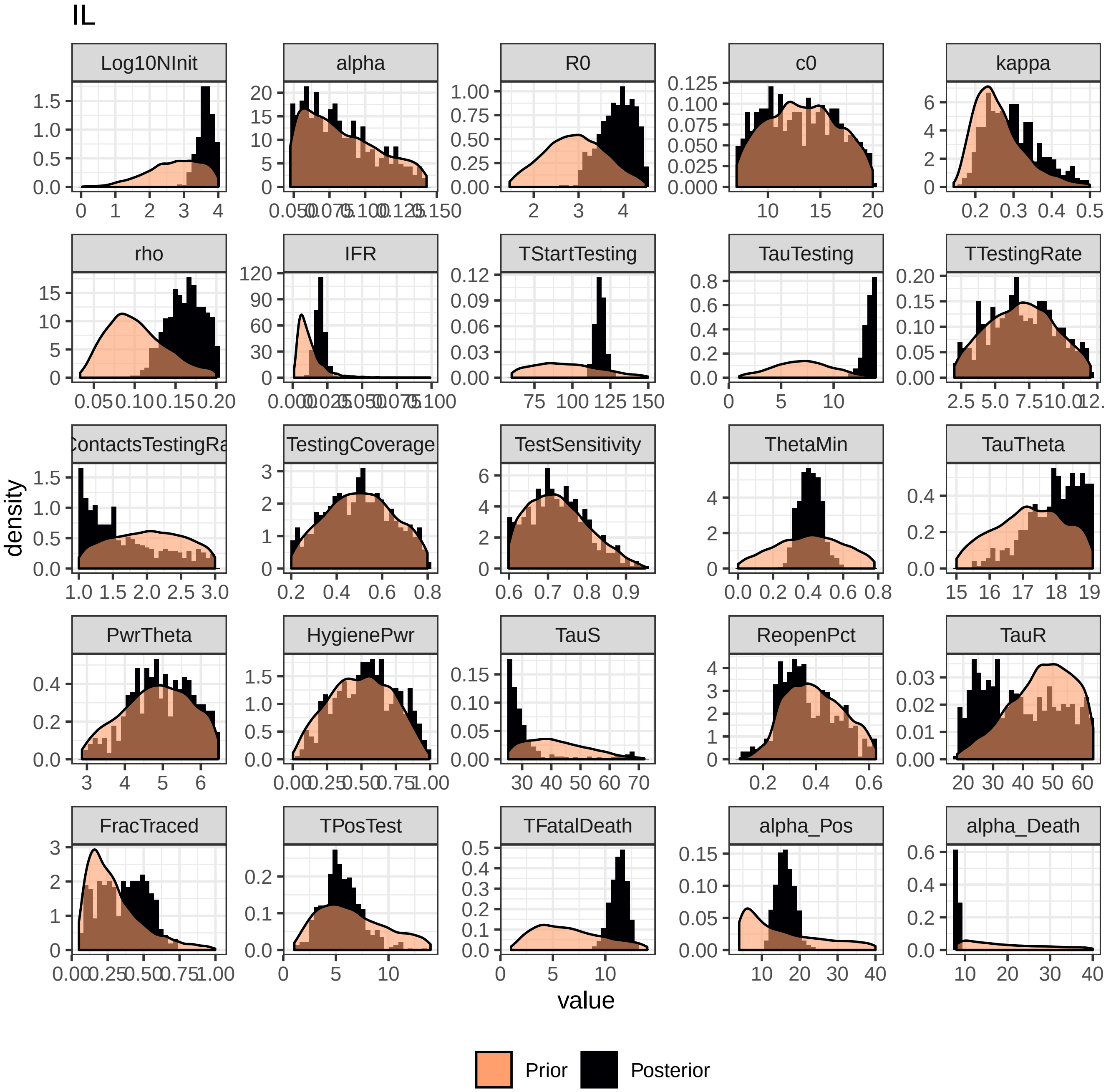
IL
Log10NInit
alpha
R0
c0
kappa
rho
IFR
TStartTesting
TauTesting
TTestingRate
ContactsTestingRa
TestingCoverage
TestSensitivity
ThetaMin
TauTheta
PwrTheta
HygienePwr
TauS
ReopenPct
TauR
FracTraced
TPosTest
TFatalDeath
alpha_Pos
alpha_Death
density
value
Prior
Posterior

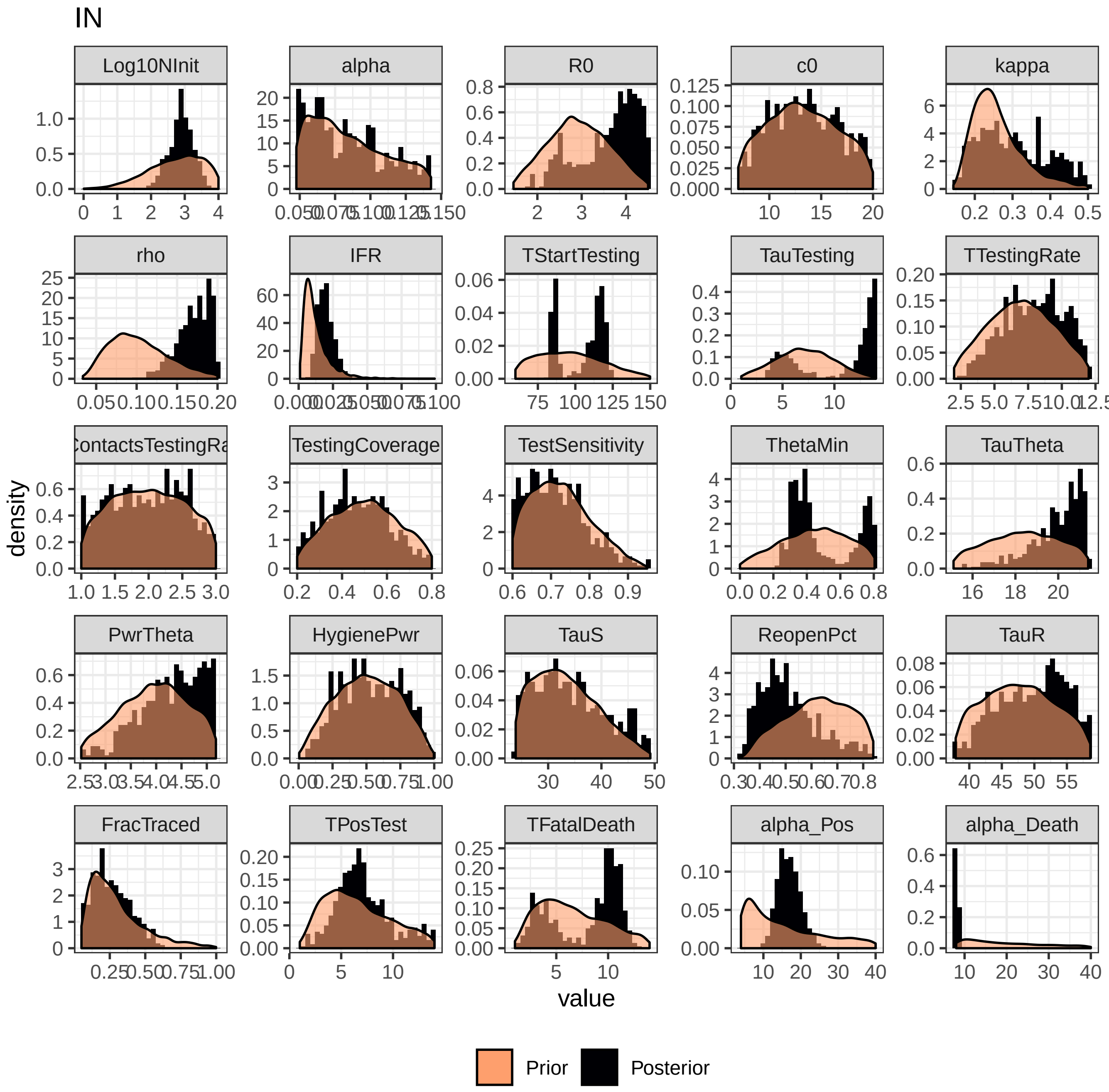
IN
Log10NInit
alpha
R0
c0
kappa
rho
IFR
TStartTesting
TauTesting
TTestingRate
ContactsTestingRa
TestingCoverage
TestSensitivity
ThetaMin
TauTheta
PwrTheta
HygienePwr
TauS
ReopenPct
TauR
FracTraced
TPosTest
TFatalDeath
alpha_Pos
alpha_Death
density
value
Prior
Posterior

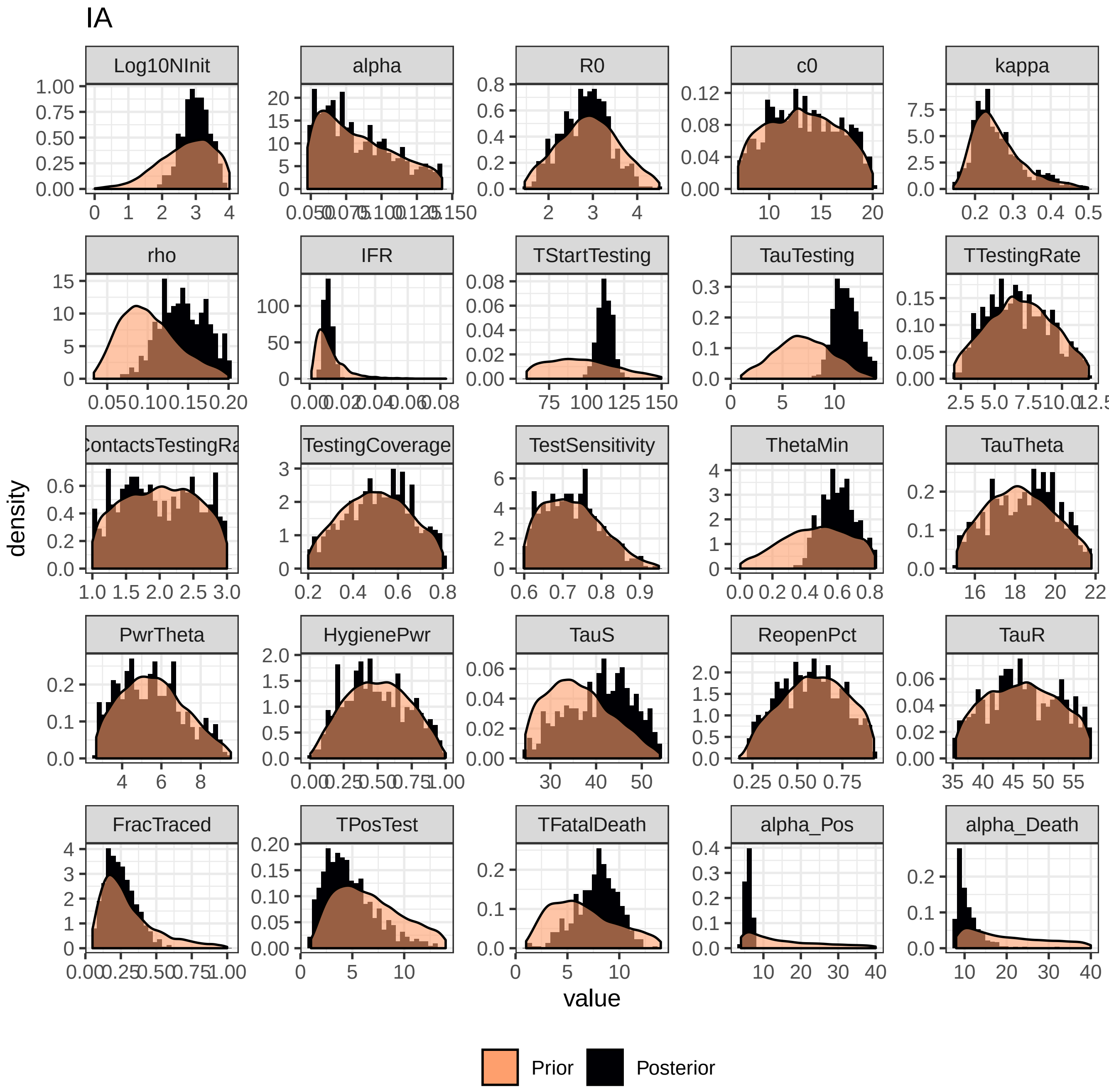
IA
Log10NInit
alpha
R0
c0
kappa
rho
IFR
TStartTesting
TauTesting
TTestingRate
ContactsTestingRa
TestingCoverage
TestSensitivity
ThetaMin
TauTheta
PwrTheta
HygienePwr
TauS
ReopenPct
TauR
FracTraced
TPosTest
TFatalDeath
alpha_Pos
alpha_Death
density
value
Prior
Posterior

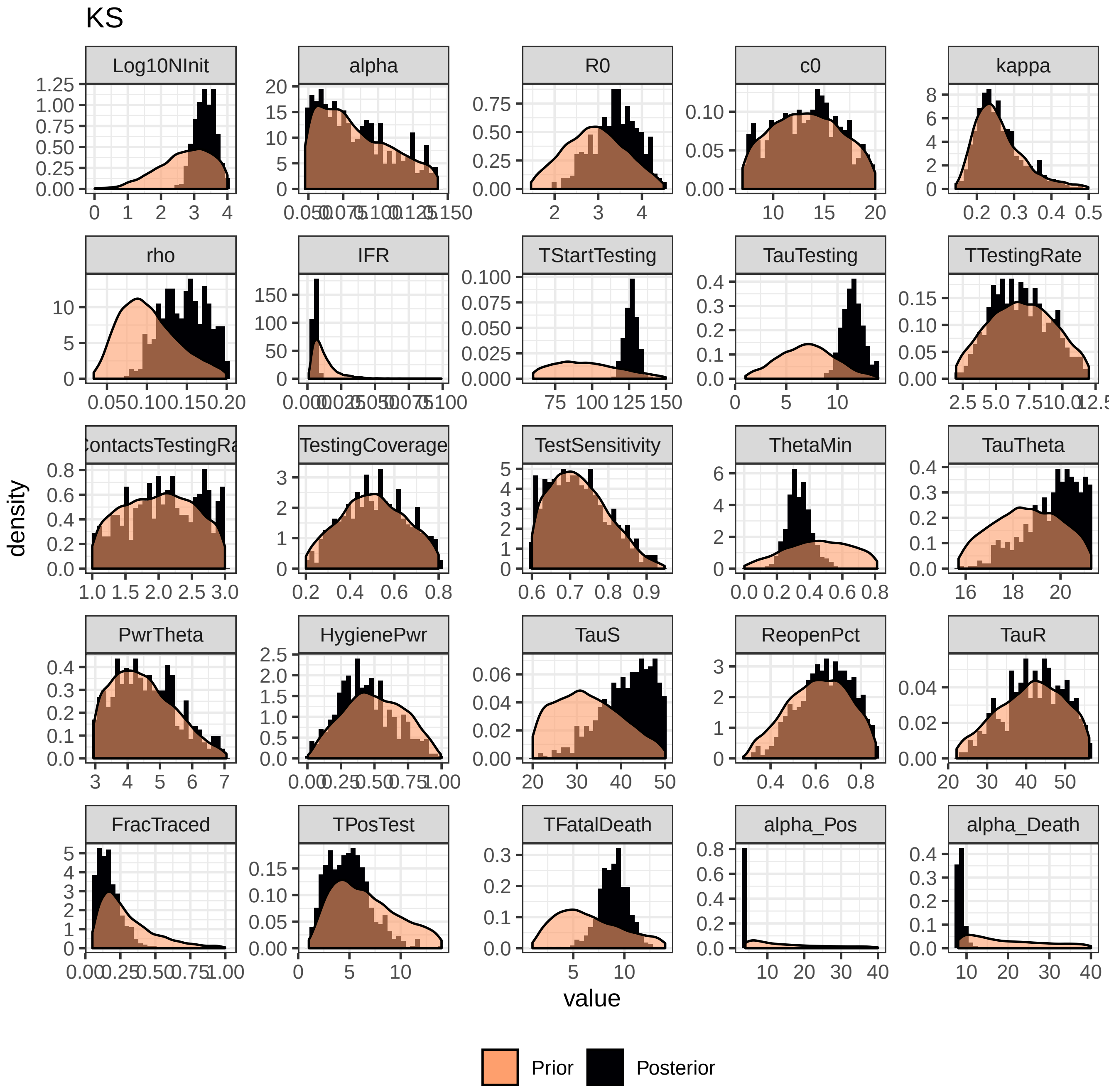
KS
Log10NInit
alpha
R0
c0
kappa
rho
IFR
TStartTesting
TauTesting
TTestingRate
ContactsTestingRa
TestingCoverage
TestSensitivity
ThetaMin
TauTheta
PwrTheta
HygienePwr
TauS
ReopenPct
TauR
FracTraced
TPosTest
TFatalDeath
alpha_Pos
alpha_Death
density
value
Prior
Posterior

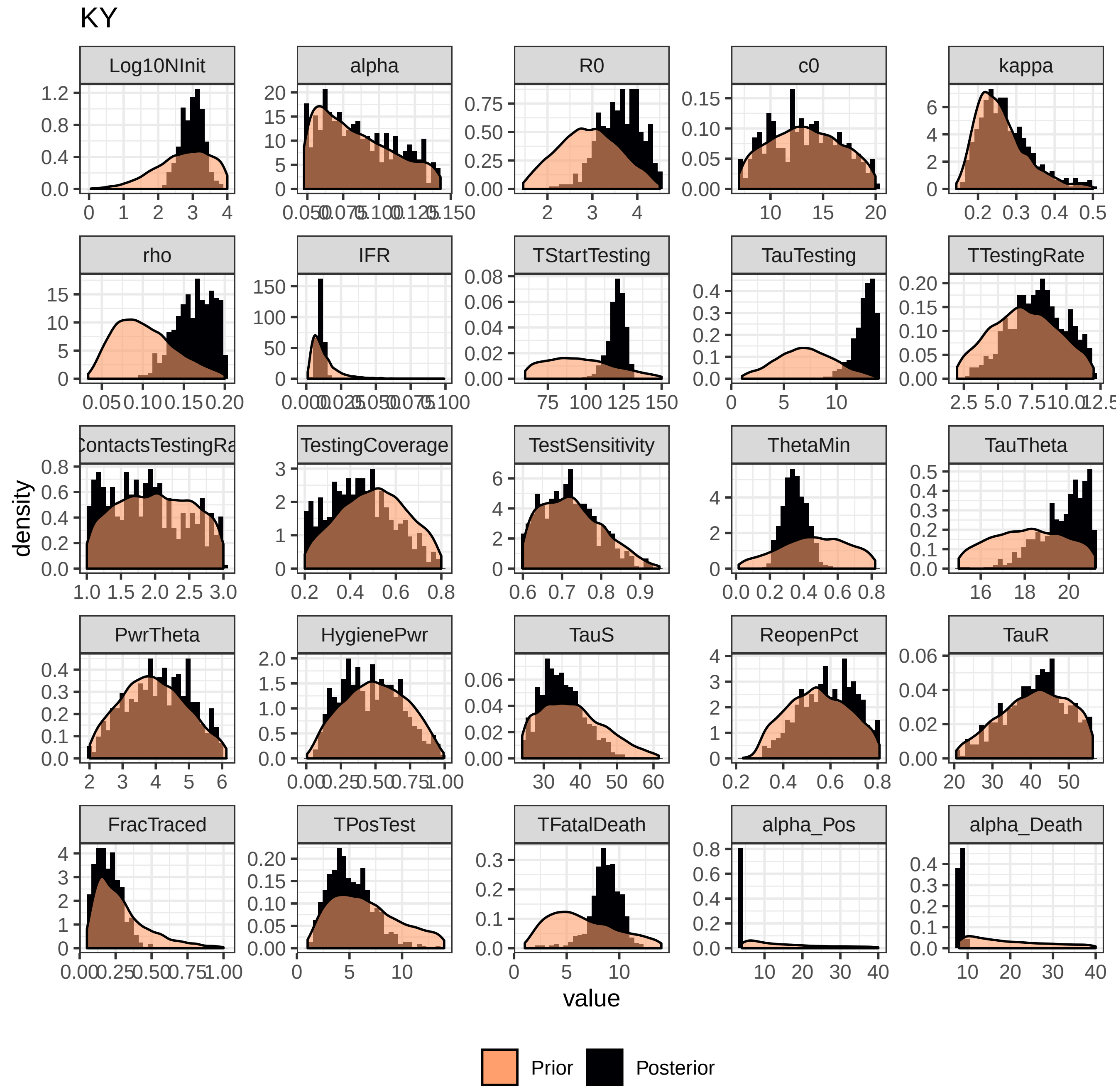
KY
Log10NInit
alpha
R0
c0
kappa
rho
IFR
TStartTesting
TauTesting
TTestingRate
ContactsTestingRa
TestingCoverage
TestSensitivity
ThetaMin
TauTheta
PwrTheta
HygienePwr
TauS
ReopenPct
TauR
FracTraced
TPosTest
TFatalDeath
alpha_Pos
alpha_Death
density
value
Prior
Posterior

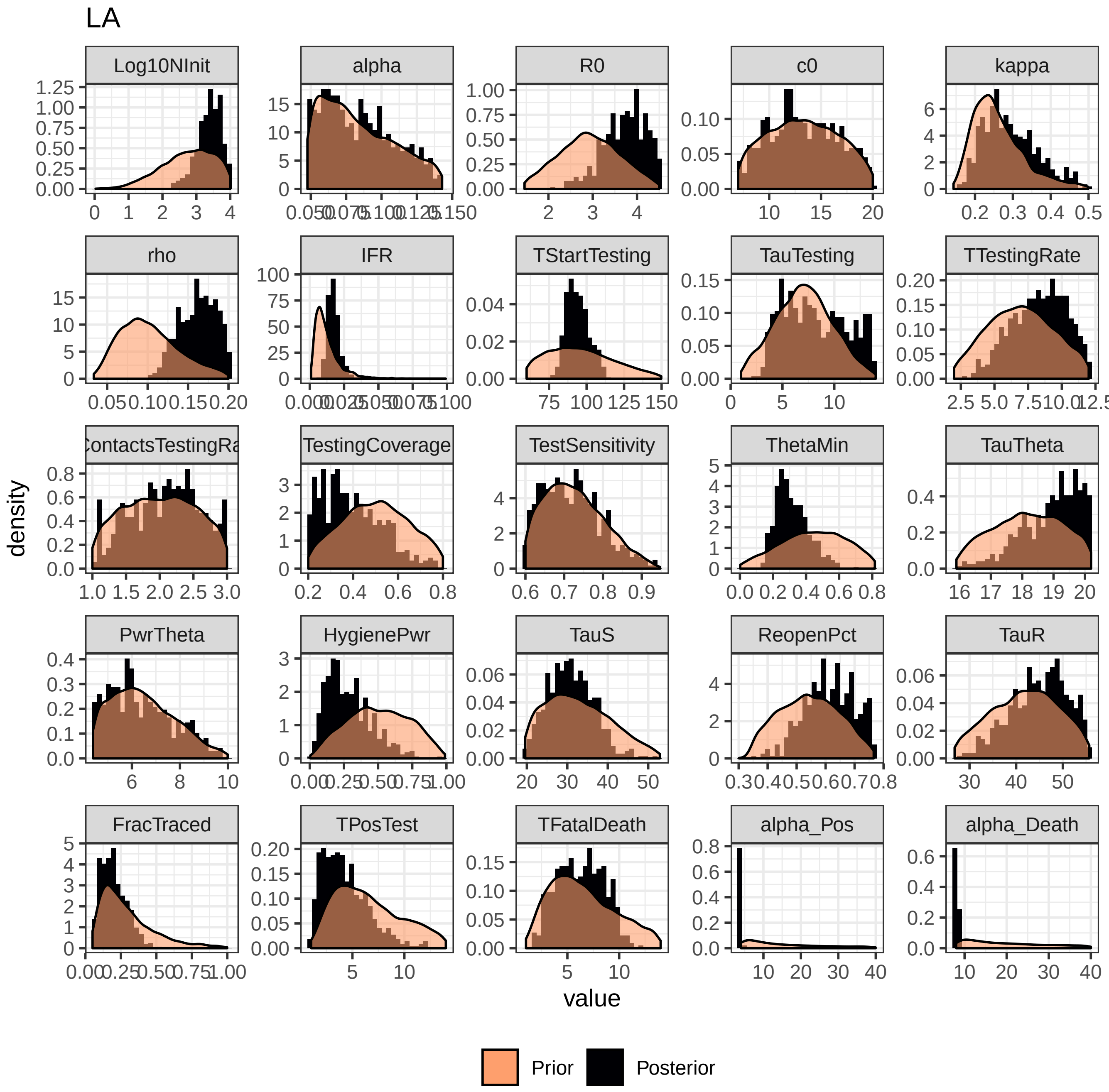
LA
Log10NInit
alpha
R0
c0
kappa
rho
IFR
TStartTesting
TauTesting
TTestingRate
ContactsTestingRa
TestingCoverage
TestSensitivity
ThetaMin
TauTheta
PwrTheta
HygienePwr
TauS
ReopenPct
TauR
FracTraced
TPosTest
TFatalDeath
alpha_Pos
alpha_Death
density
value
Prior
Posterior

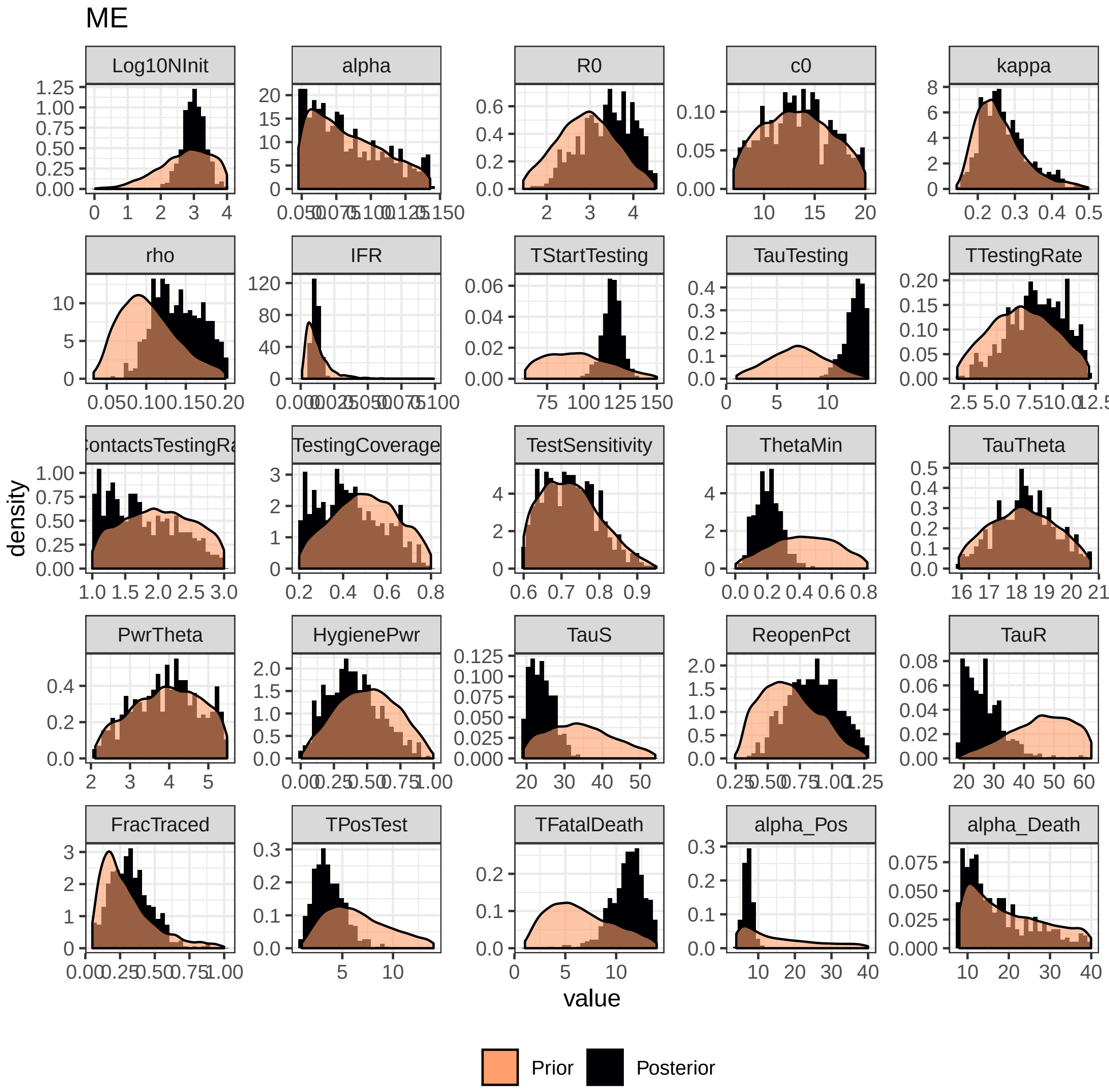

ME
Log10NInit
alpha
R0
c0
kappa
rho
IFR
TStartTesting
TauTesting
TTestingRate
ContactsTestingRa
TestingCoverage
TestSensitivity
ThetaMin
TauTheta
PwrTheta
HygienePwr
TauS
ReopenPct
TauR
FracTraced
TPosTest
TFatalDeath
alpha_Pos
alpha_Death
density
value
Prior
Posterior

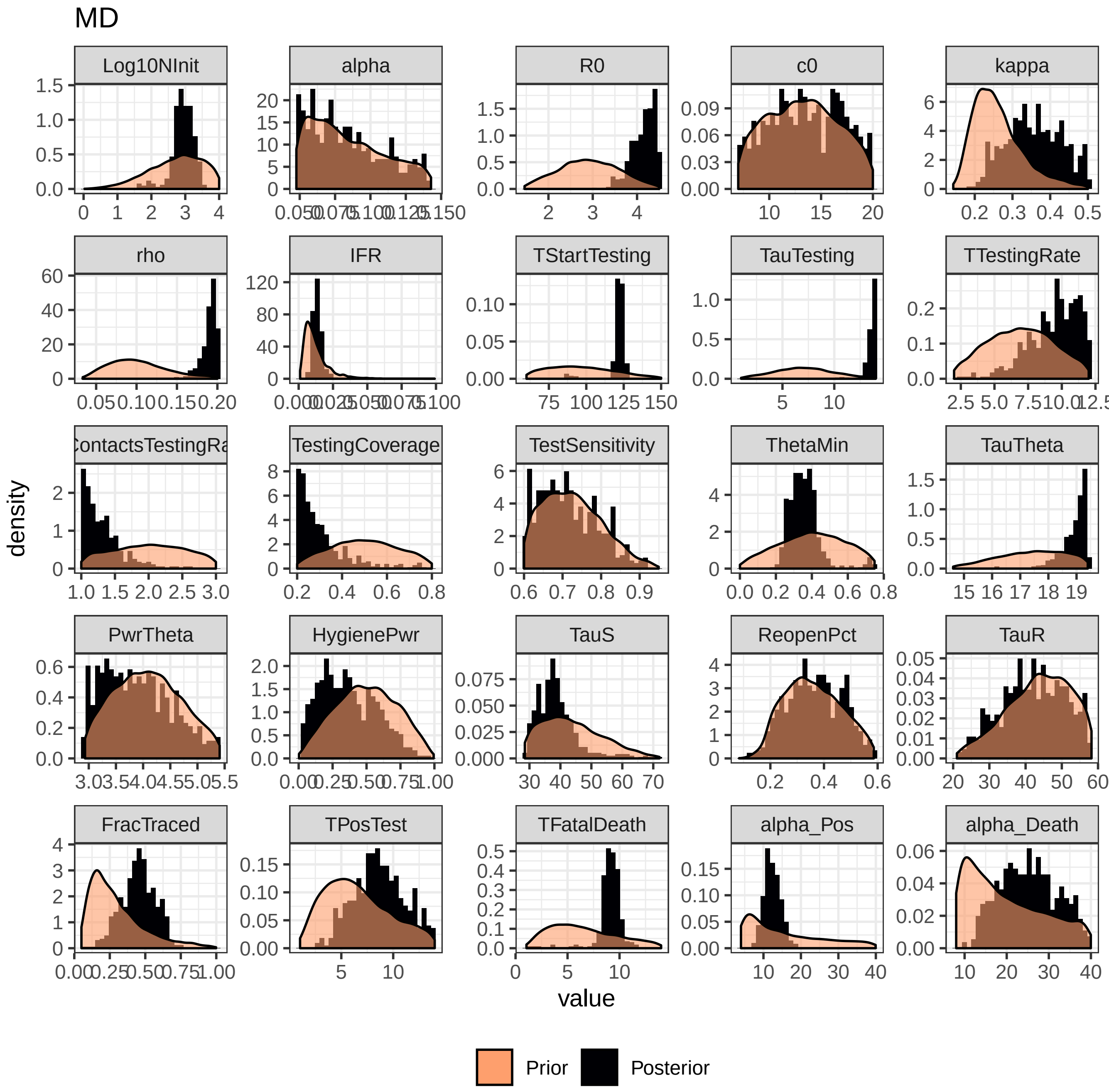

MD
Log10NInit
alpha
R0
c0
kappa
rho
IFR
TStartTesting
TauTesting
TTestingRate
ContactsTestingRa
TestingCoverage
TestSensitivity
ThetaMin
TauTheta
PwrTheta
HygienePwr
TauS
ReopenPct
TauR
FracTraced
TPosTest
TFatalDeath
alpha_Pos
alpha_Death
density
value
Prior
Posterior

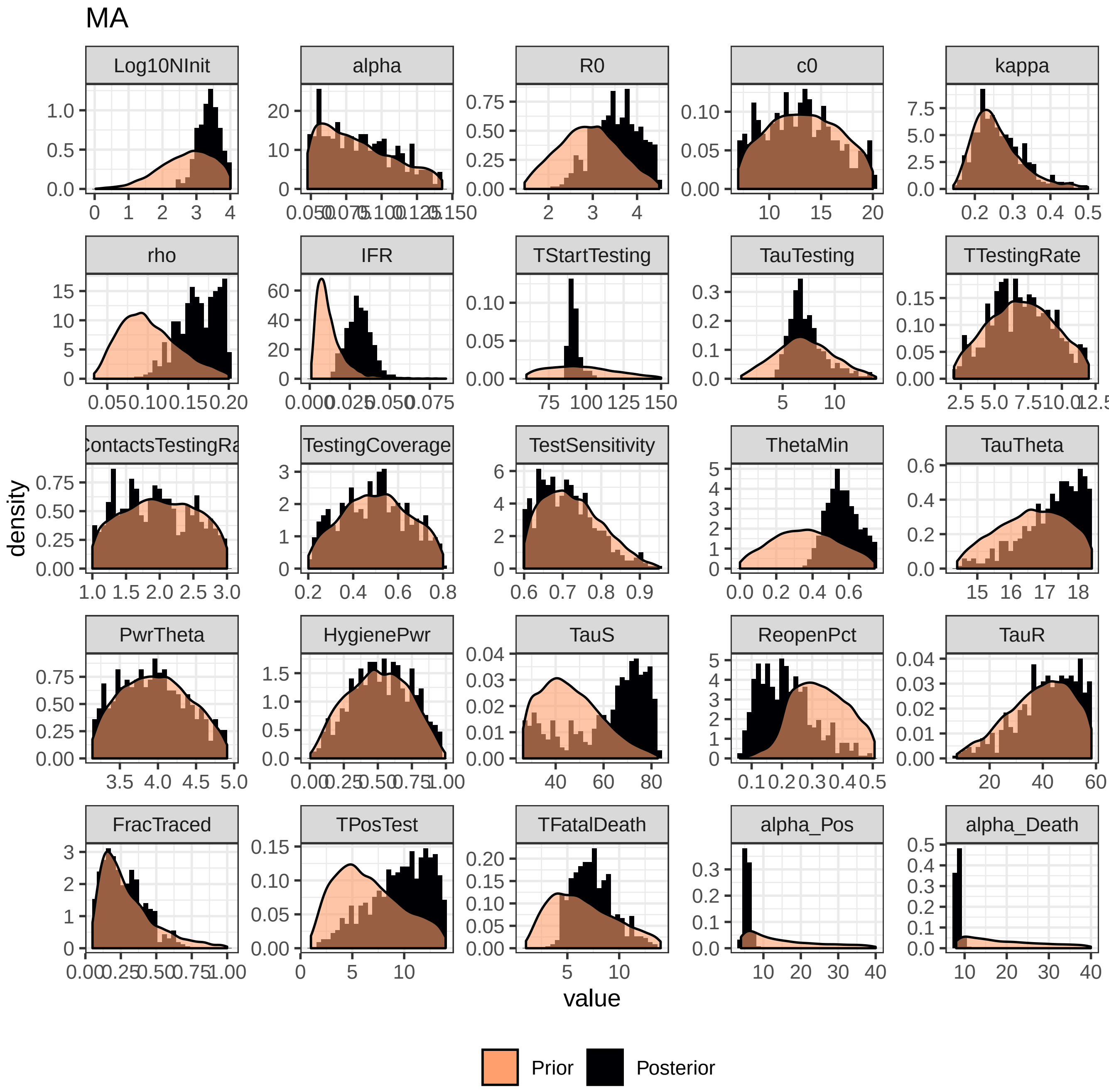
MA
Log10NInit
alpha
R0
c0
kappa
rho
IFR
TStartTesting
TauTesting
TTestingRate
ContactsTestingRa
TestingCoverage
TestSensitivity
ThetaMin
TauTheta
PwrTheta
HygienePwr
TauS
ReopenPct
TauR
FracTraced
TPosTest
TFatalDeath
alpha_Pos
alpha_Death
density
value
Prior
Posterior

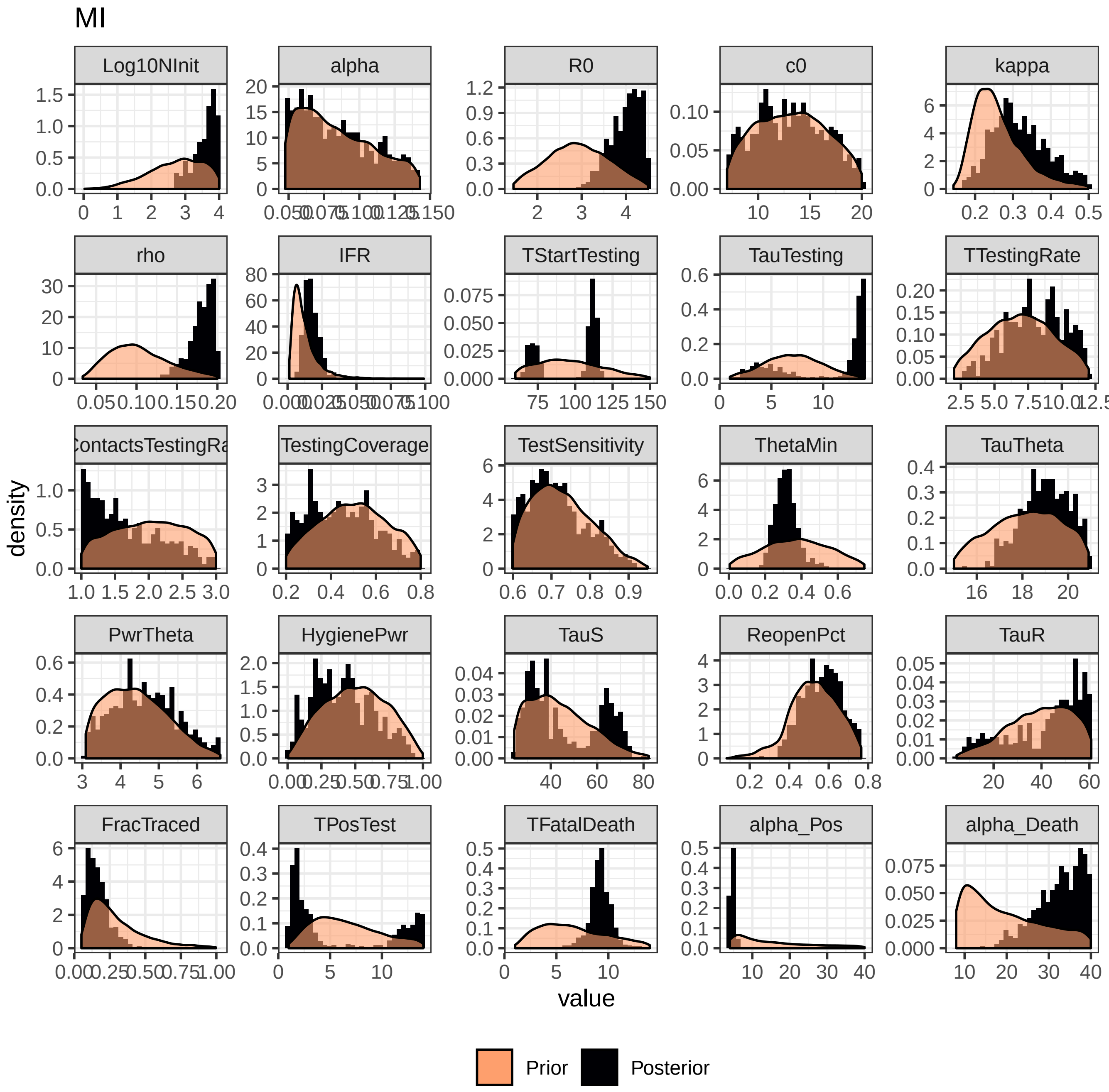

MI
Log10NInit
alpha
R0
c0
kappa
rho
IFR
TStartTesting
TauTesting
TTestingRate
ContactsTestingRa
TestingCoverage
TestSensitivity
ThetaMin
TauTheta
PwrTheta
HygienePwr
TauS
ReopenPct
TauR
FracTraced
TPosTest
TFatalDeath
alpha_Pos
alpha_Death
density
value
Prior
Posterior

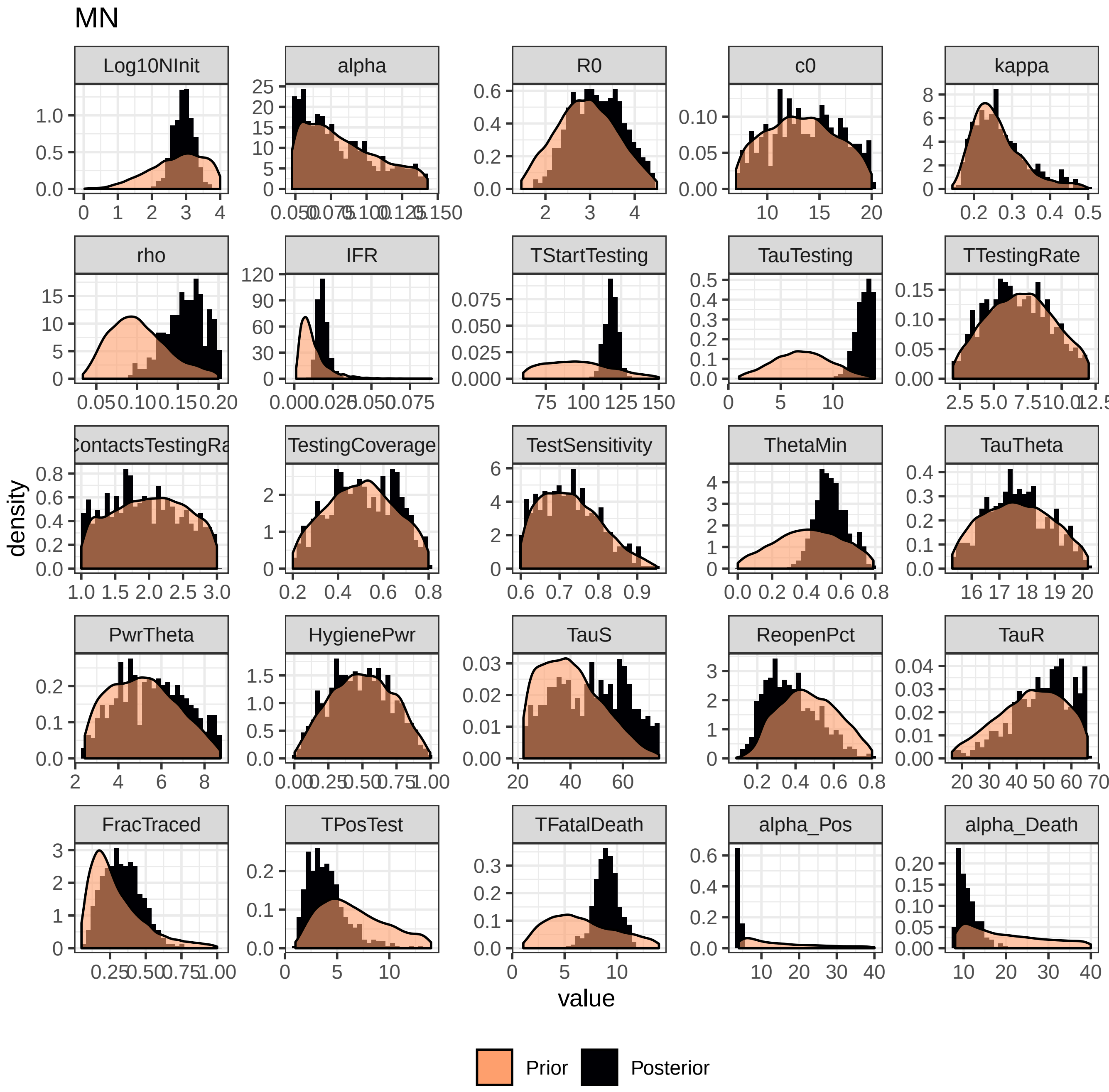

MN
Log10NInit
alpha
R0
c0
kappa
rho
IFR
TStartTesting
TauTesting
TTestingRate
ContactsTestingRa
TestingCoverage
TestSensitivity
ThetaMin
TauTheta
PwrTheta
HygienePwr
TauS
ReopenPct
TauR
FracTraced
TPosTest
TFatalDeath
alpha_Pos
alpha_Death
density
value
Prior
Posterior

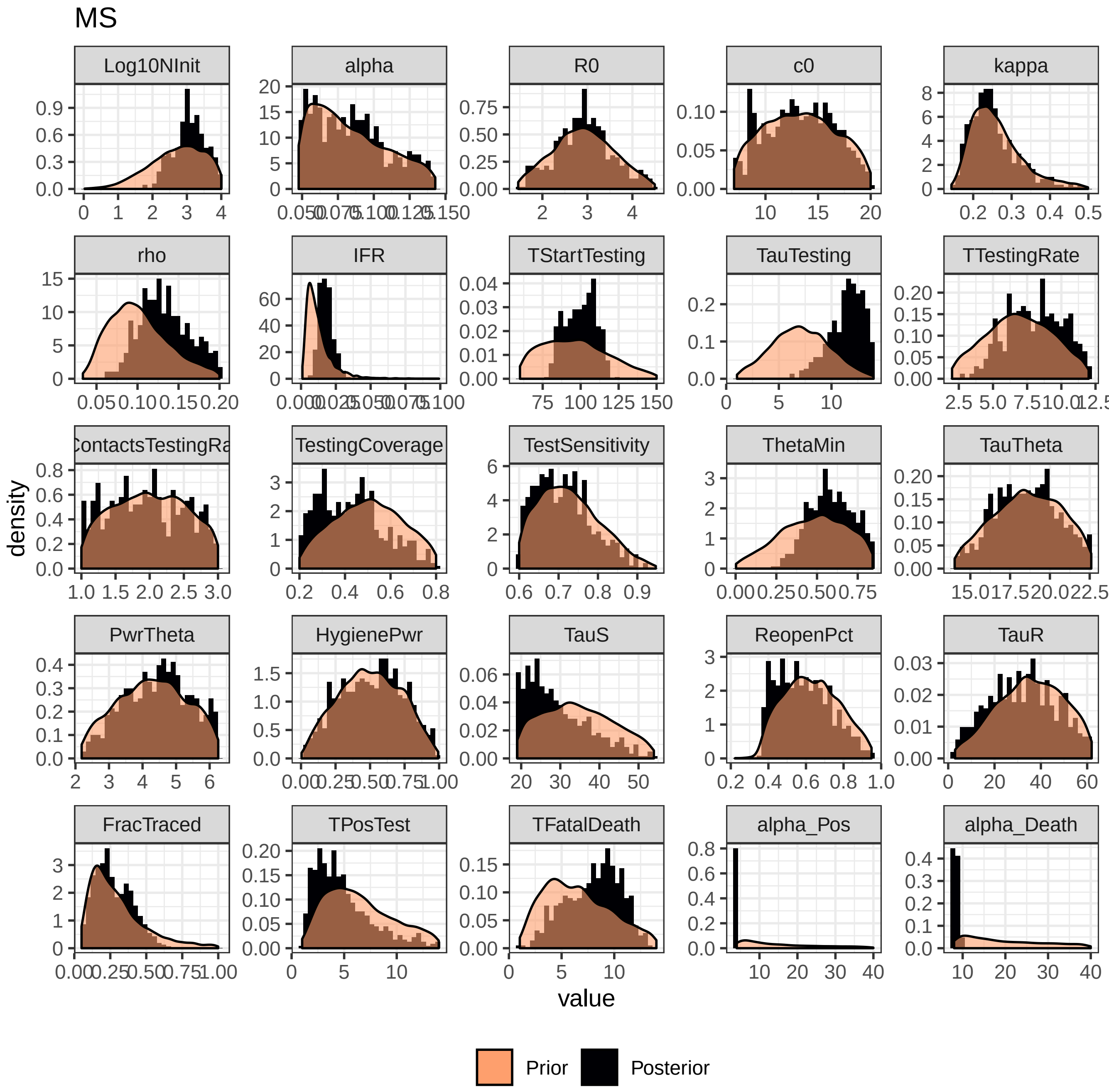
MS
Log10NInit
alpha
R0
c0
kappa
rho
IFR
TStartTesting
TauTesting
TTestingRate
ContactsTestingRa
TestingCoverage
TestSensitivity
ThetaMin
TauTheta
PwrTheta
HygienePwr
TauS
ReopenPct
TauR
FracTraced
TPosTest
TFatalDeath
alpha_Pos
alpha_Death
density
value
Prior
Posterior

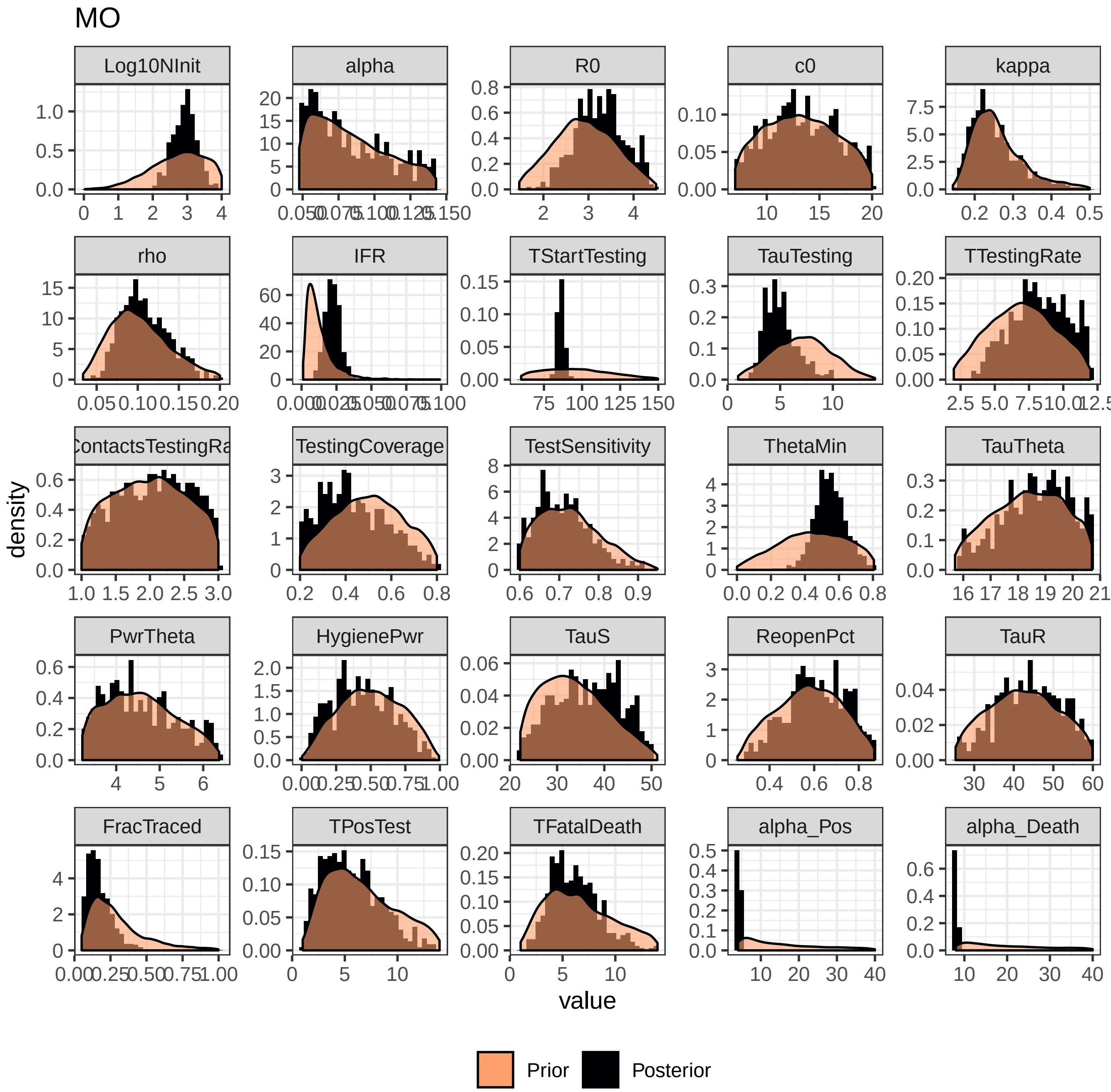
MO
Log10NInit
alpha
R0
c0
kappa
rho
IFR
TStartTesting
TauTesting
TTestingRate
ContactsTestingRa
TestingCoverage
TestSensitivity
ThetaMin
TauTheta
PwrTheta
HygienePwr
TauS
ReopenPct
TauR
FracTraced
TPosTest
TFatalDeath
alpha_Pos
alpha_Death
density
value
Prior
Posterior

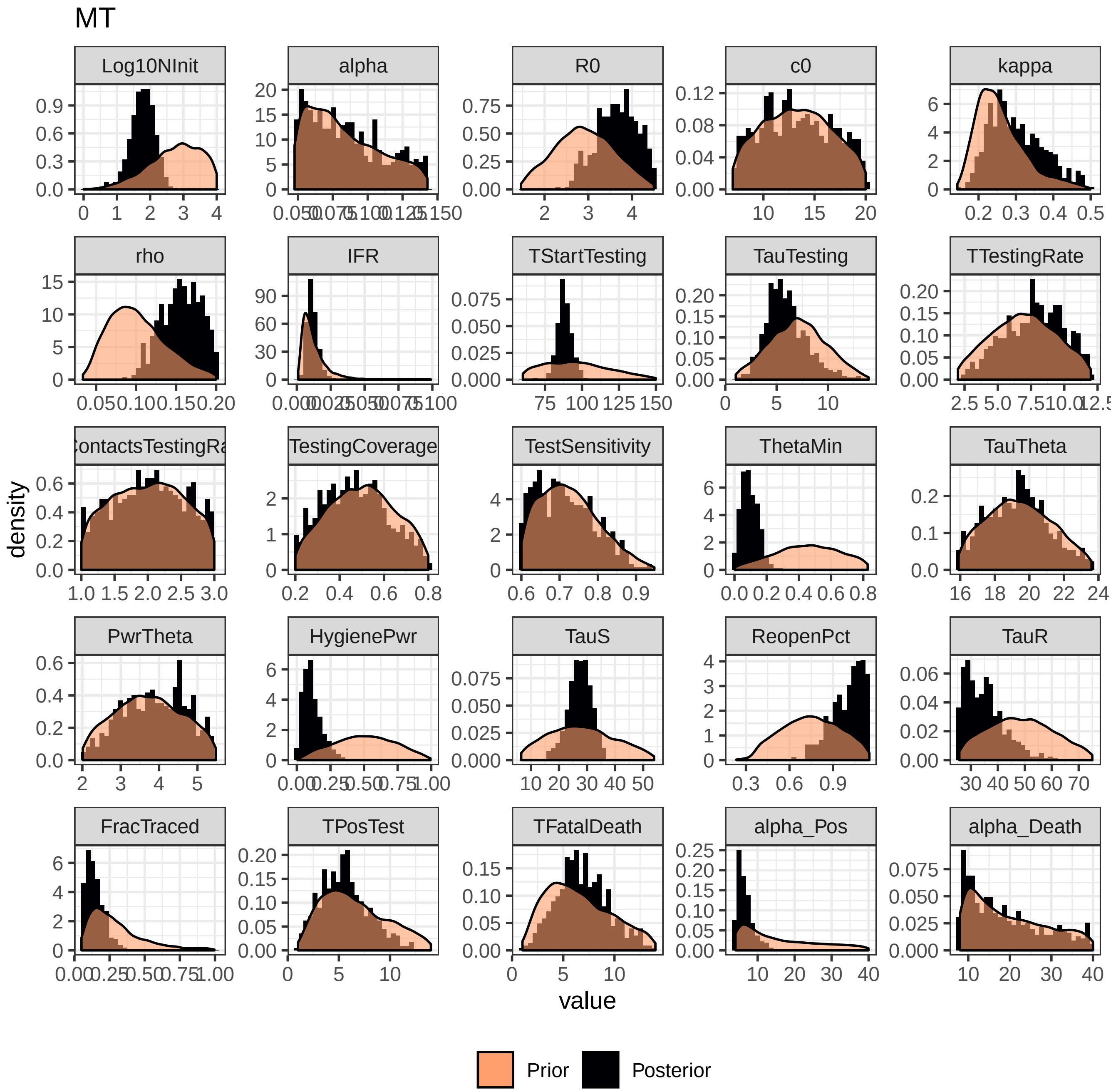

MT
Log10NInit
alpha
R0
c0
kappa
rho
IFR
TStartTesting
TauTesting
TTestingRate
ContactsTestingRa
TestingCoverage
TestSensitivity
ThetaMin
TauTheta
PwrTheta
HygienePwr
TauS
ReopenPct
TauR
FracTraced
TPosTest
TFatalDeath
alpha_Pos
alpha_Death
density
value
Prior
Posterior

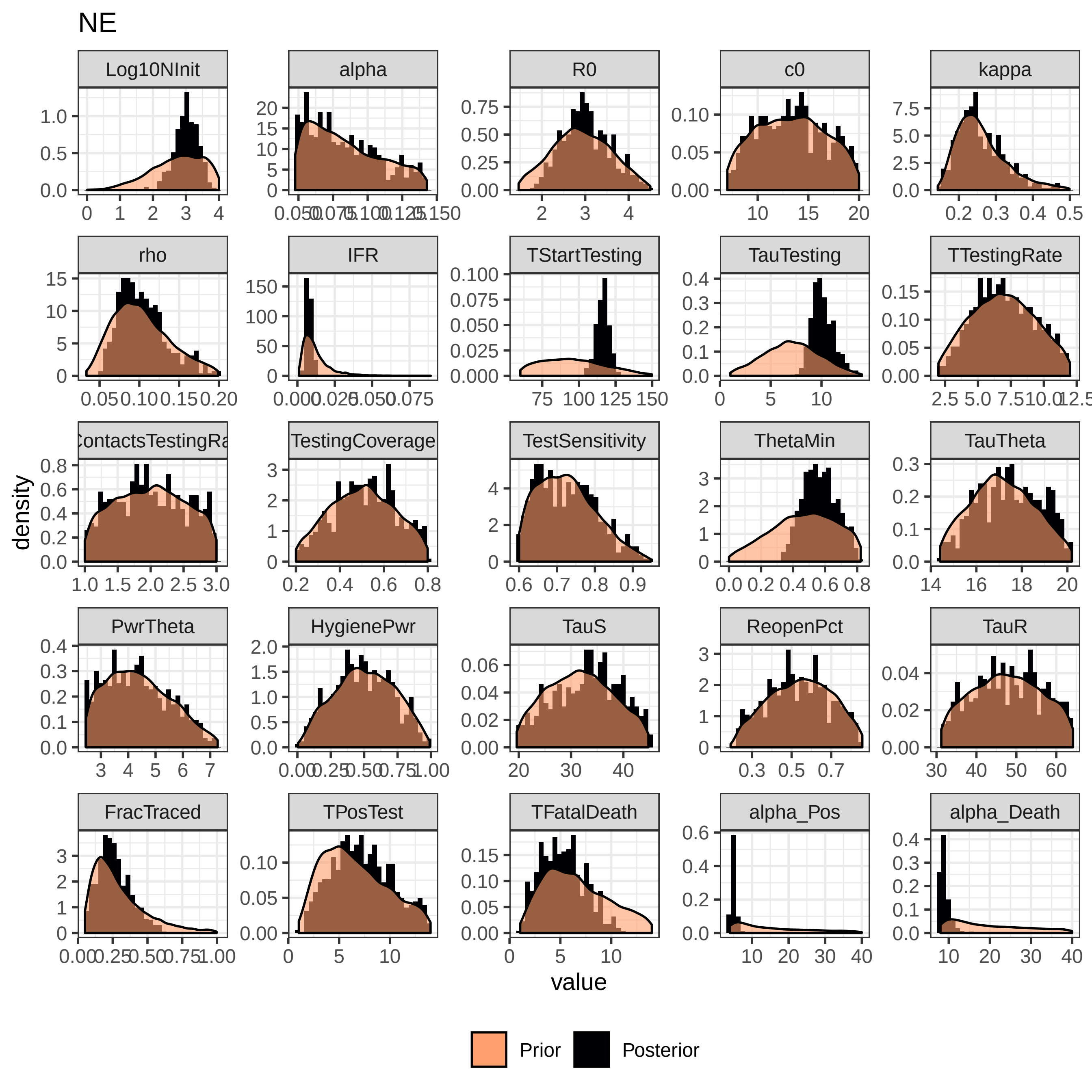
NE
Log10NInit
alpha
R0
c0
kappa
rho
IFR
TStartTesting
TauTesting
TTestingRate
ContactsTestingRa
TestingCoverage
TestSensitivity
ThetaMin
TauTheta
PwrTheta
HygienePwr
TauS
ReopenPct
TauR
FracTraced
TPosTest
TFatalDeath
alpha_Pos
alpha_Death
density
value
Prior
Posterior

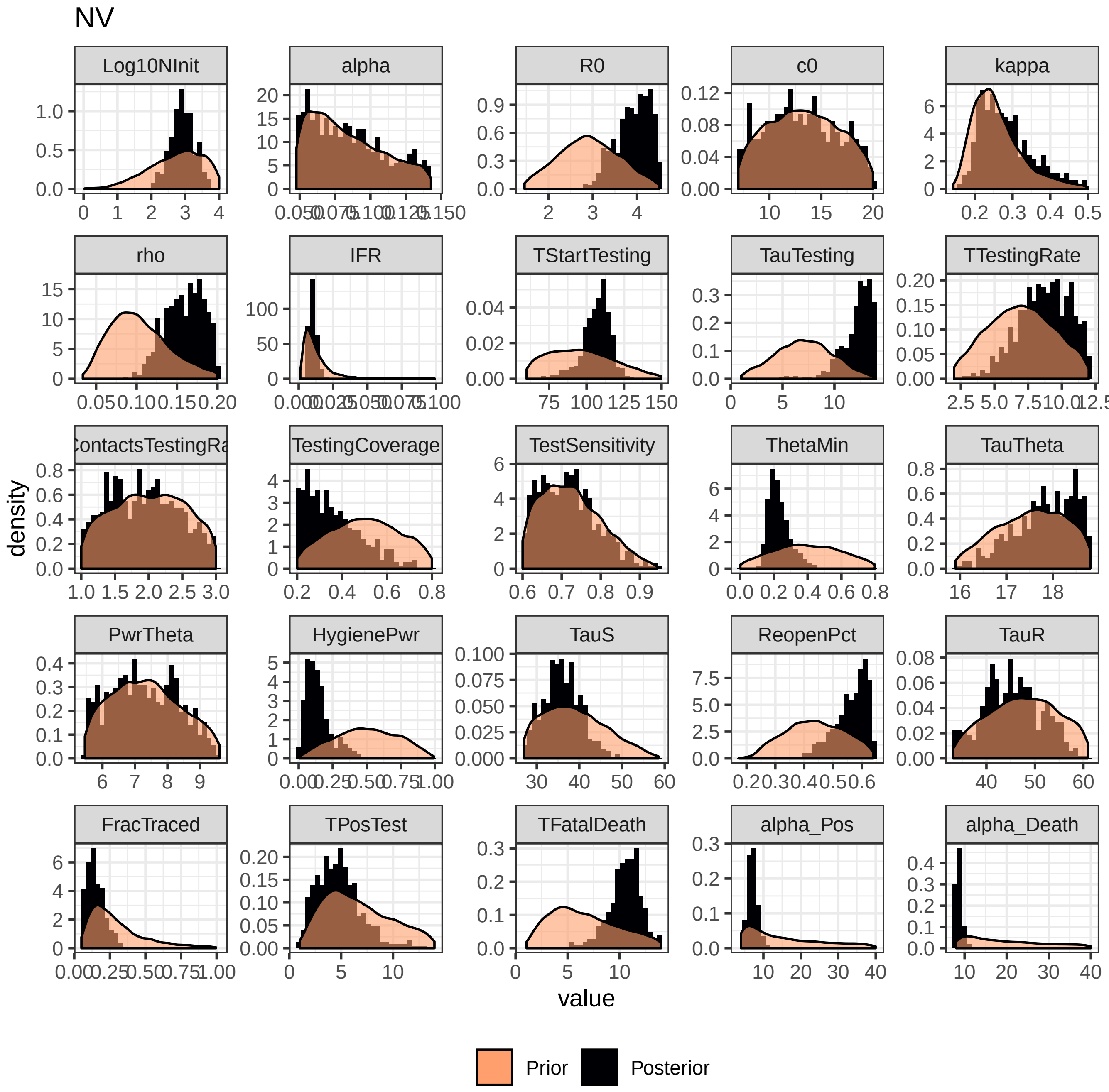
NV
Log10NInit
alpha
R0
c0
kappa
rho
IFR
TStartTesting
TauTesting
TTestingRate
ContactsTestingRa
TestingCoverage
TestSensitivity
ThetaMin
TauTheta
PwrTheta
HygienePwr
TauS
ReopenPct
TauR
FracTraced
TPosTest
TFatalDeath
alpha_Pos
alpha_Death
density
value
Prior
Posterior

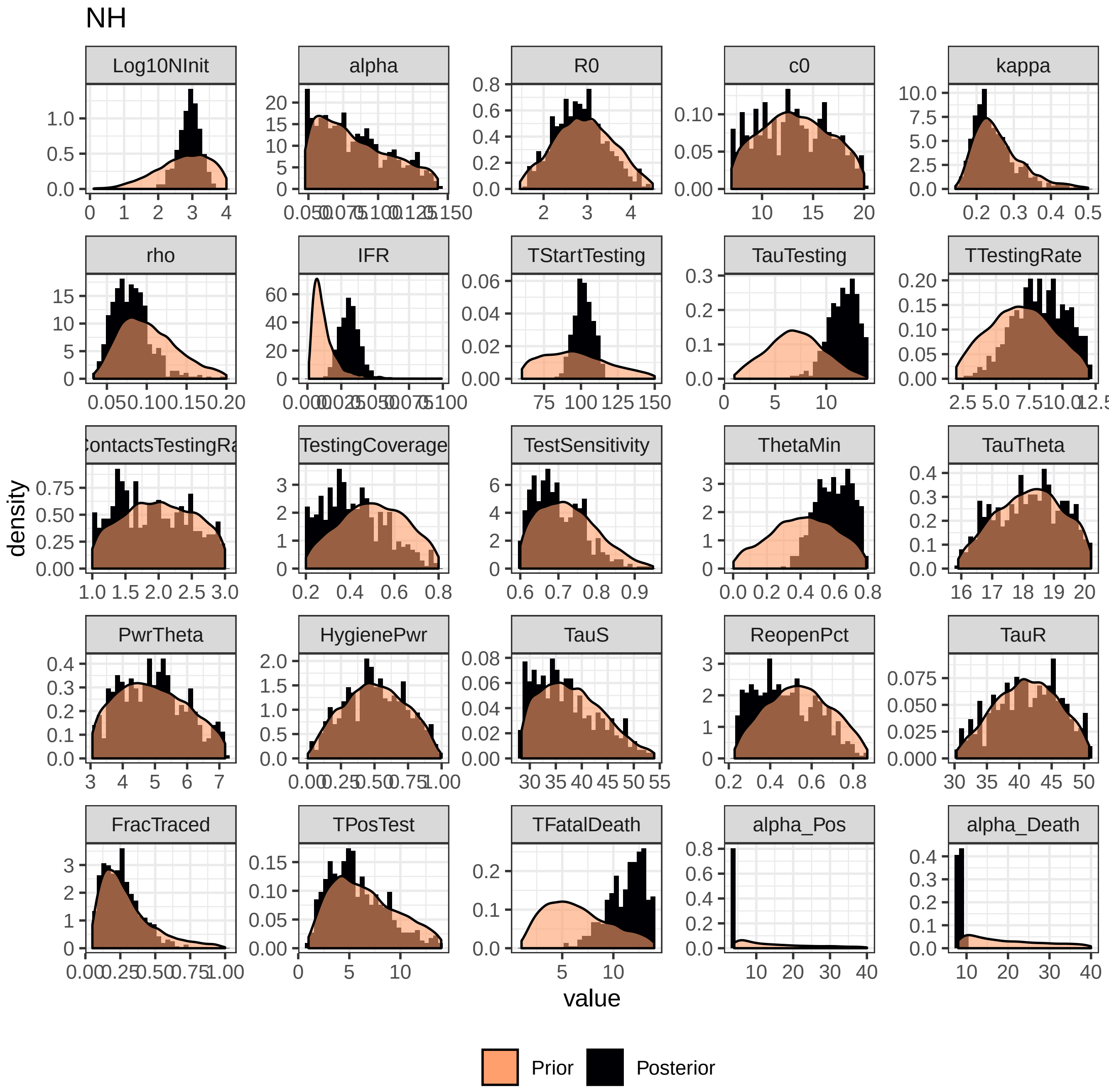

NH
Log10NInit
alpha
R0
c0
kappa
rho
IFR
TStartTesting
TauTesting
TTestingRate
TestingCoverage
TestSensitivity
ThetaMin
TauTheta
PwrTheta
HygienePwr
TauS
ReopenPct
TauR
FracTraced
TPosTest
TFatalDeath
alpha_Pos
alpha_Death
density
value
Prior
Posterior

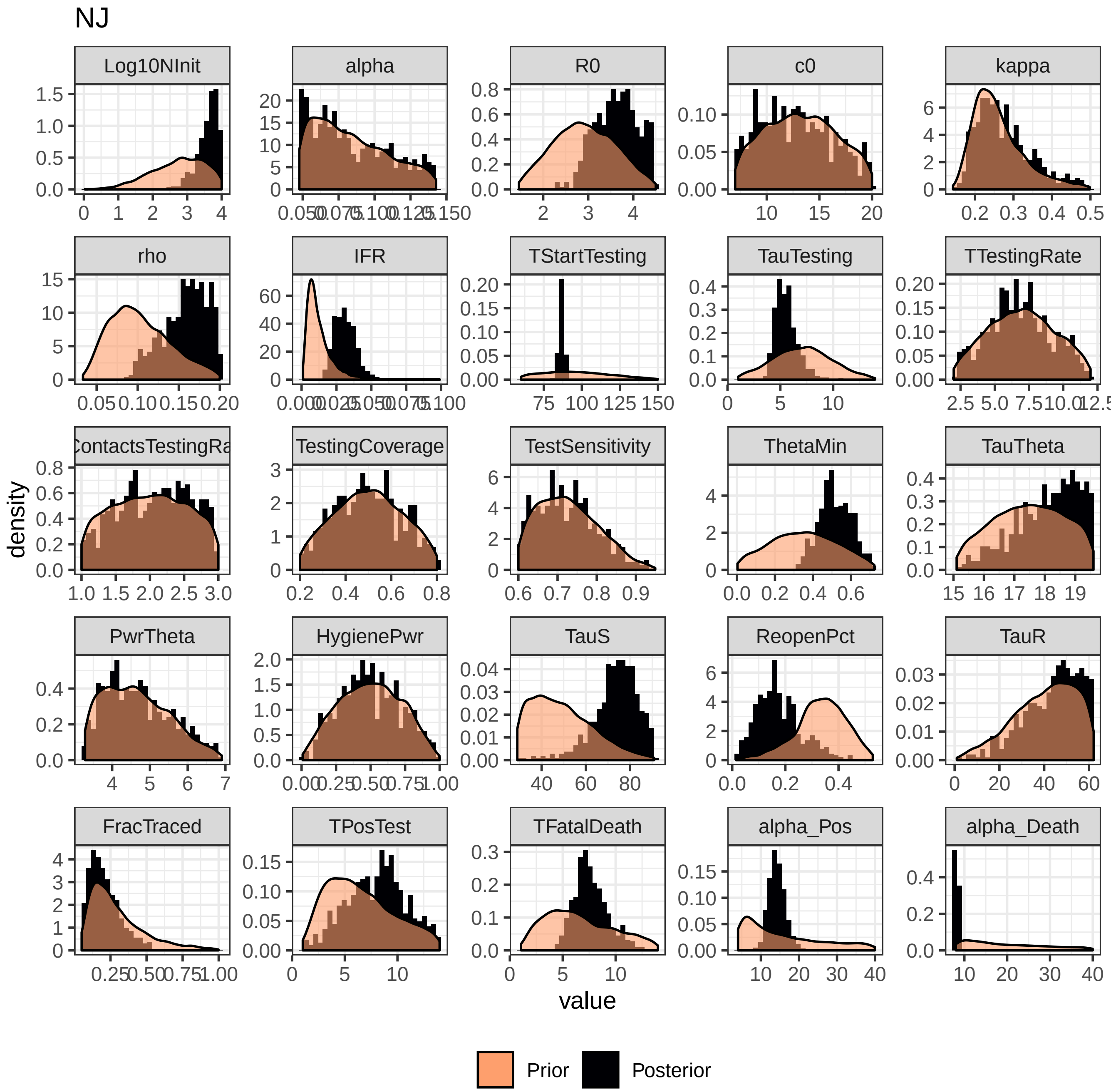
NJ
Log10NInit
alpha
R0
c0
kappa
rho
IFR
TStartTesting
TauTesting
TTestingRate
ContactsTestingRa
TestingCoverage
TestSensitivity
ThetaMin
TauTheta
PwrTheta
HygienePwr
TauS
ReopenPct
TauR
FracTraced
TPosTest
TFatalDeath
alpha_Pos
alpha_Death
density
value
Prior
Posterior

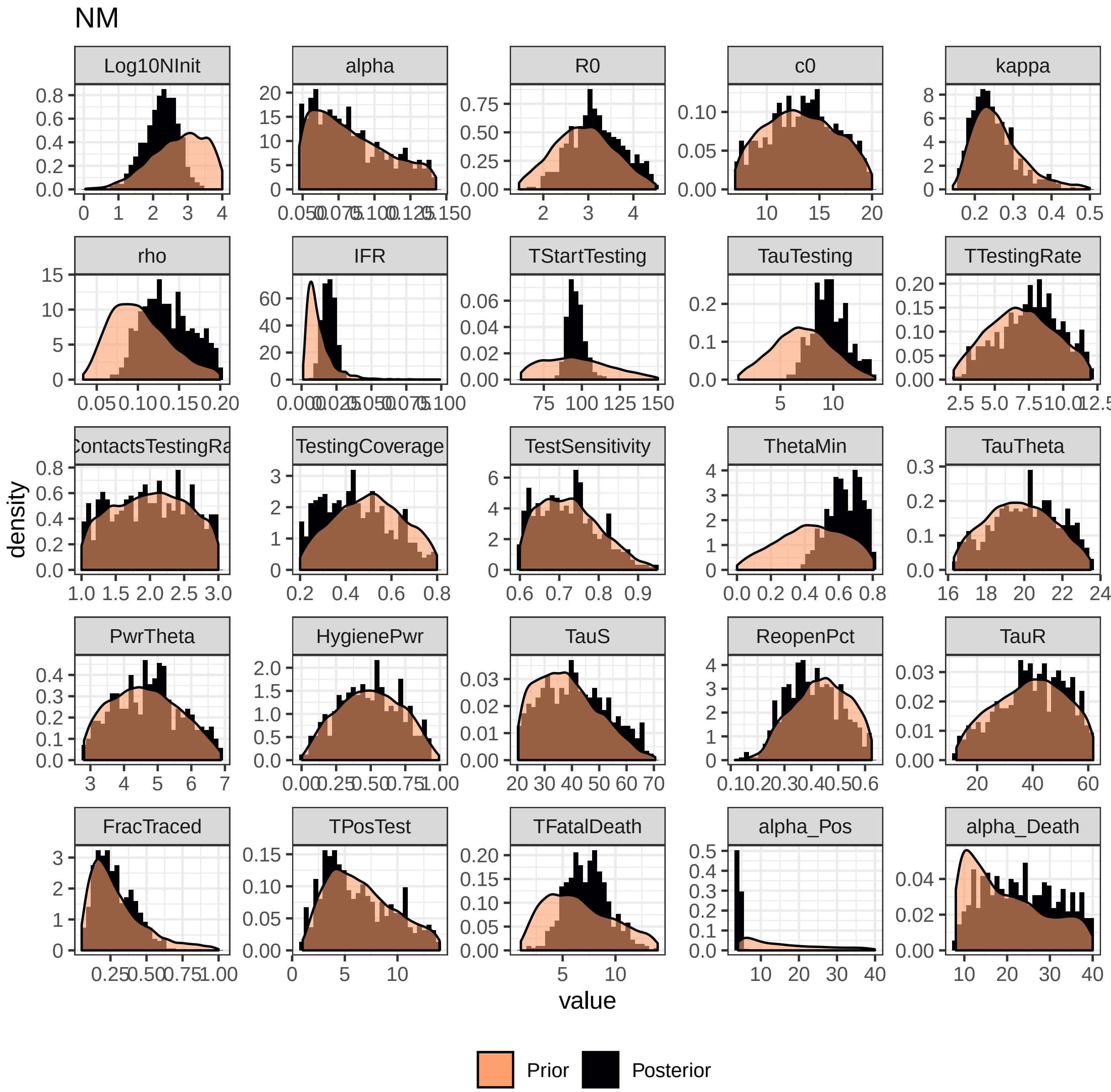
NM
Log10NInit
alpha
R0
c0
kappa
rho
IFR
TStartTesting
TauTesting
TTestingRate
ContactsTestingRa
TestingCoverage
TestSensitivity
ThetaMin
TauTheta
PwrTheta
HygienePwr
TauS
ReopenPct
TauR
FracTraced
TPosTest
TFatalDeath
alpha_Pos
alpha_Death
density
value
Prior
Posterior

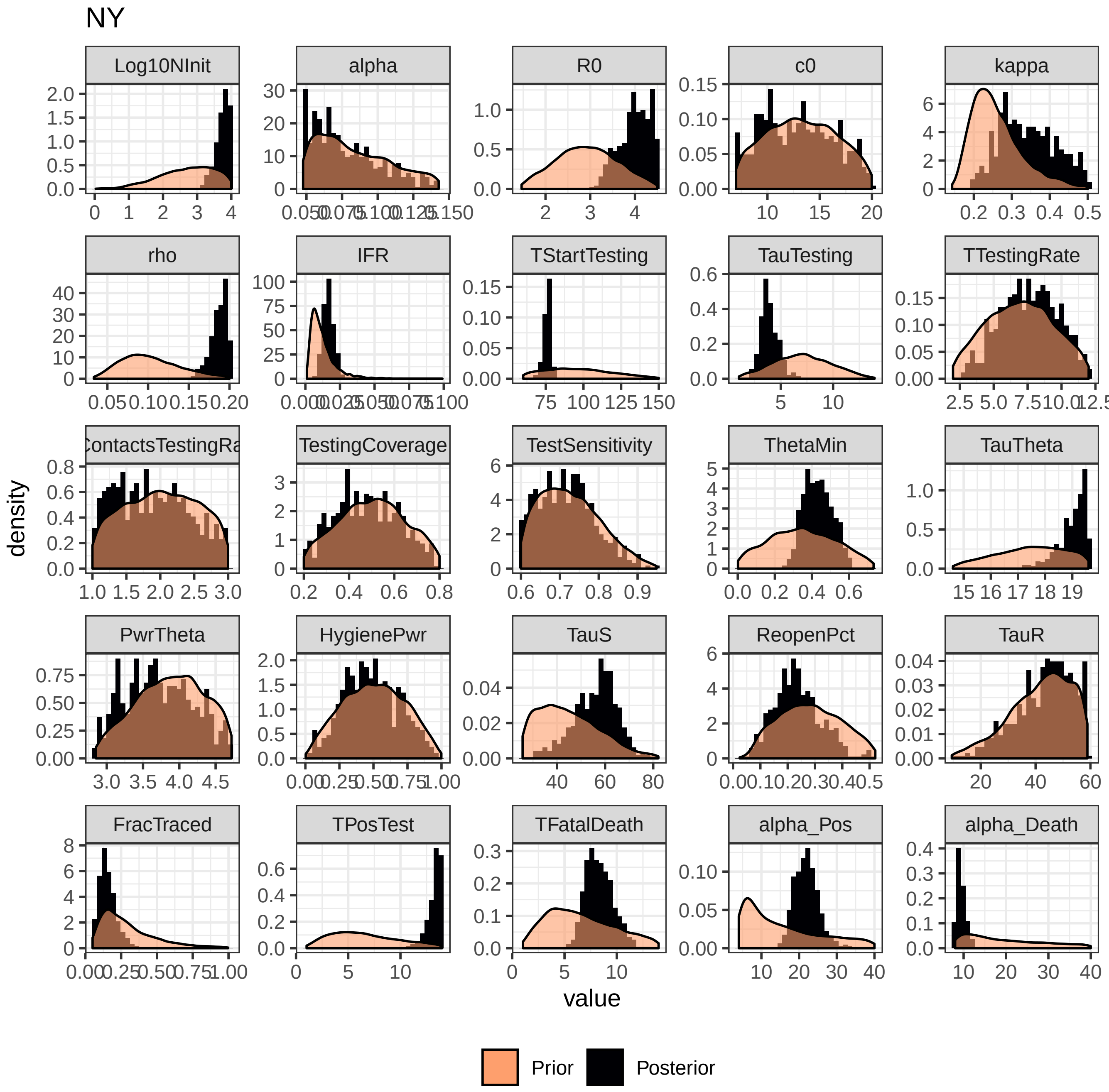

NY
Log10NInit
alpha
R0
c0
kappa
rho
IFR
TStartTesting
TauTesting
TTestingRate
ContactsTestingRa
TestingCoverage
TestSensitivity
ThetaMin
TauTheta
PwrTheta
HygienePwr
TauS
ReopenPct
TauR
FracTraced
TPosTest
TFatalDeath
alpha_Pos
alpha_Death
density
value
Prior
Posterior

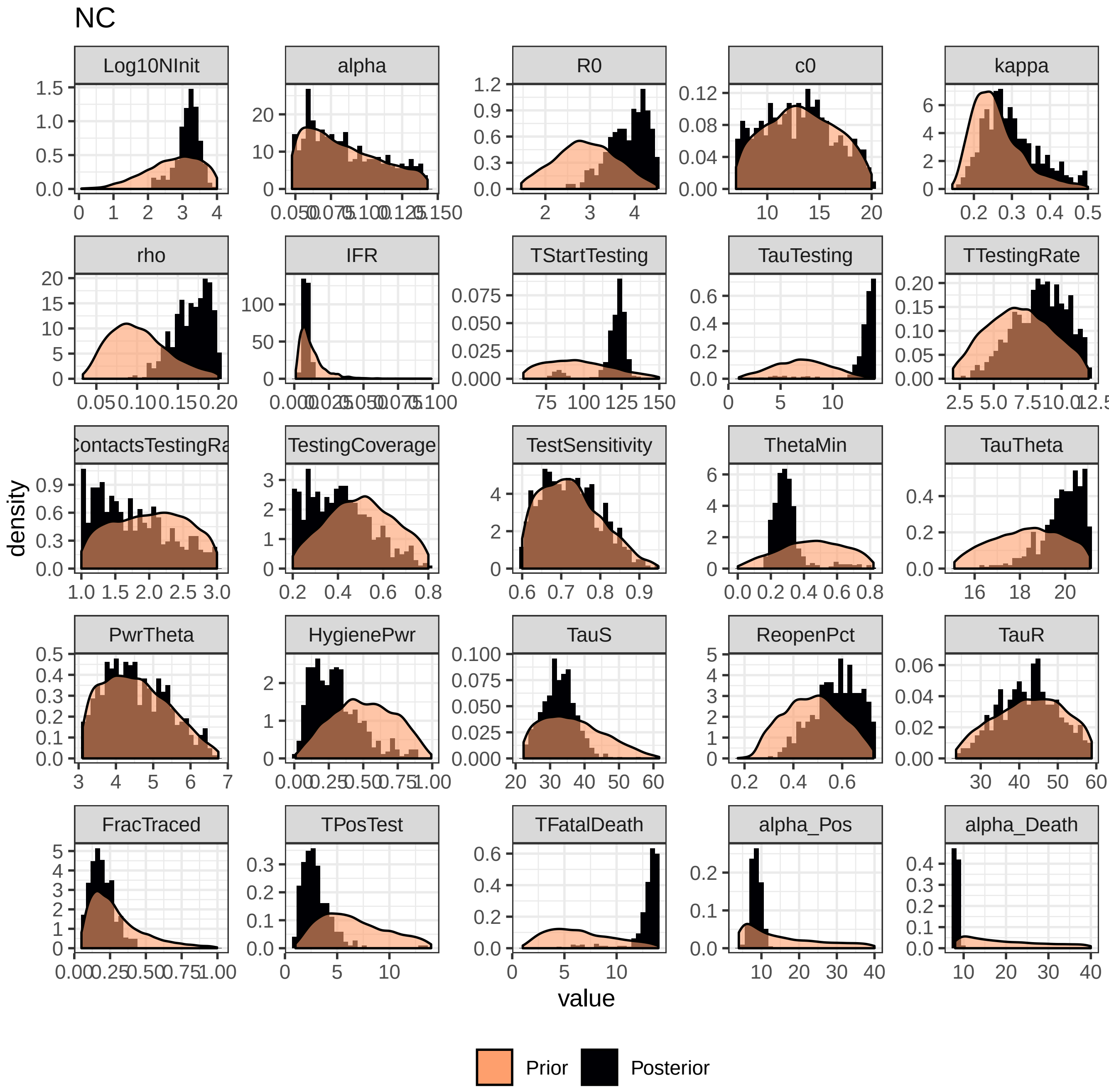
NC
Log10NInit
alpha
R0
c0
kappa
rho
IFR
TStartTesting
TauTesting
TTestingRate
ContactsTestingRa
TestingCoverage
TestSensitivity
ThetaMin
TauTheta
PwrTheta
HygienePwr
TauS
ReopenPct
TauR
FracTraced
TPosTest
TFatalDeath
alpha_Pos
alpha_Death
density
value
Prior
Posterior

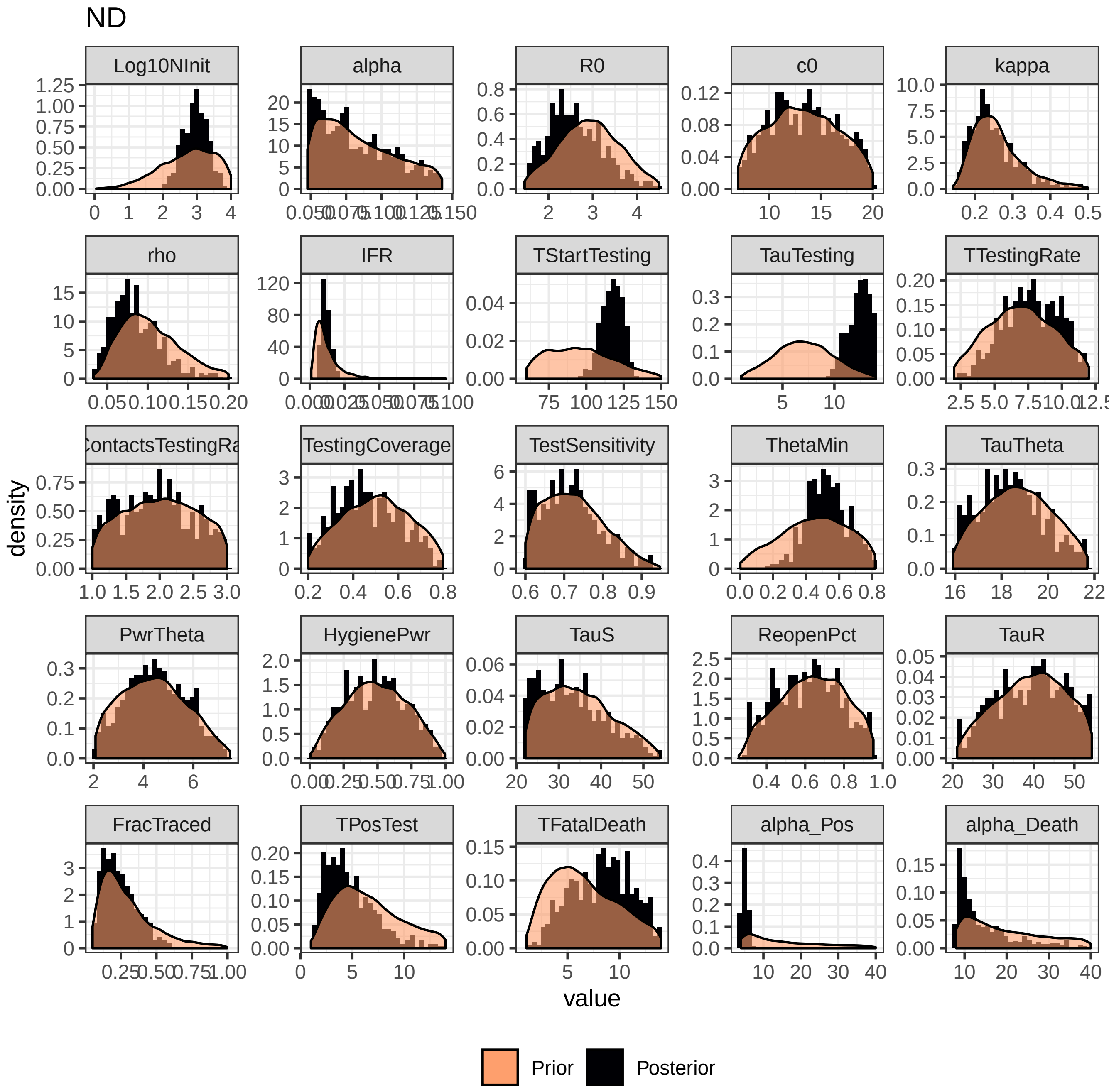

ND
Log10NInit
alpha
R0
c0
kappa
rho
IFR
TStartTesting
TauTesting
TTestingRate
ContactsTestingRa
TestingCoverage
TestSensitivity
ThetaMin
TauTheta
PwrTheta
HygienePwr
TauS
ReopenPct
TauR
FracTraced
TPosTest
TFatalDeath
alpha_Pos
alpha_Death
density
value
Prior
Posterior

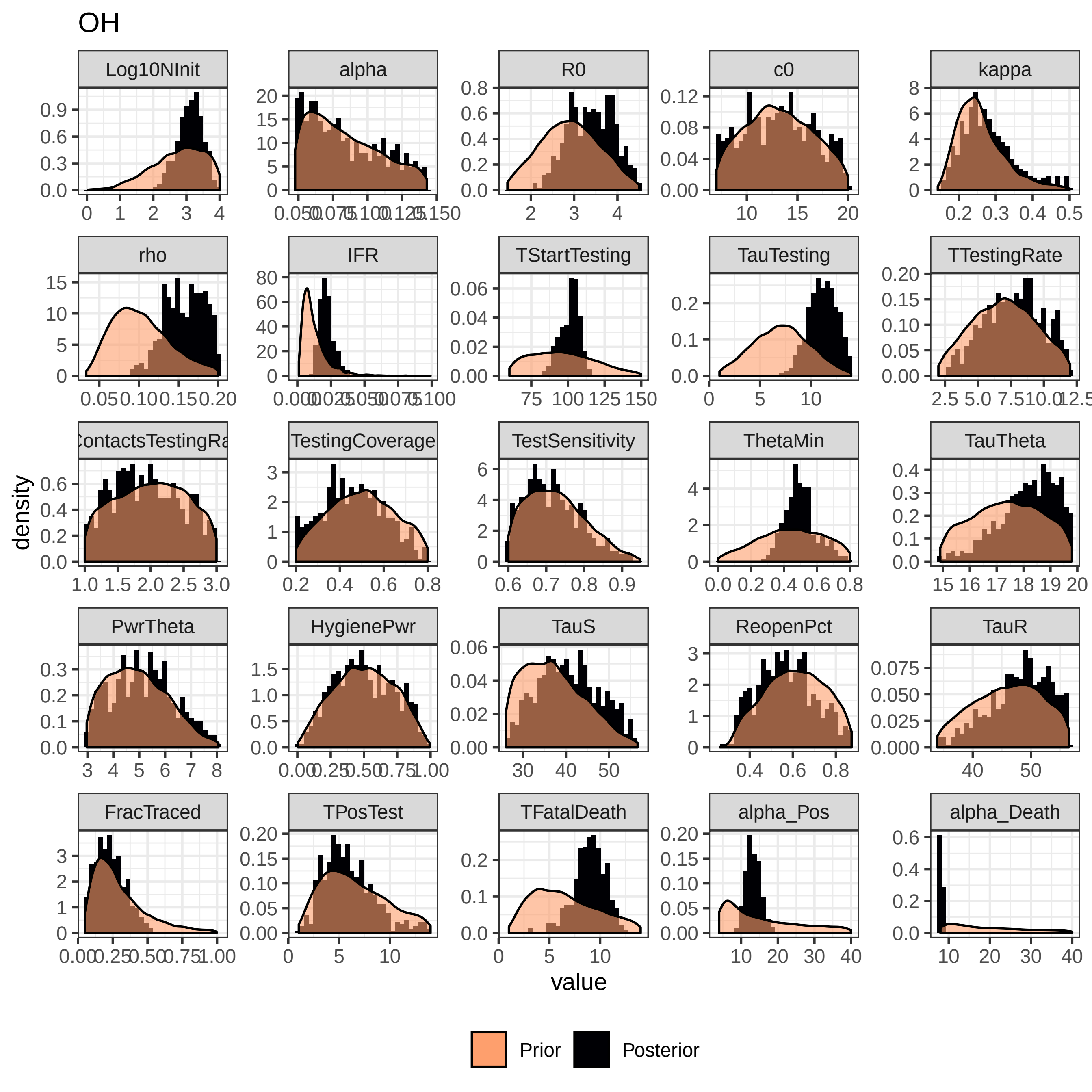
OH
Log10NInit
alpha
R0
c0
kappa
rho
IFR
TStartTesting
TauTesting
TTestingRate
ContactsTestingRa
TestingCoverage
TestSensitivity
ThetaMin
TauTheta
PwrTheta
HygienePwr
TauS
ReopenPct
TauR
FracTraced
TPosTest
TFatalDeath
alpha_Pos
alpha_Death
density
value
Prior
Posterior

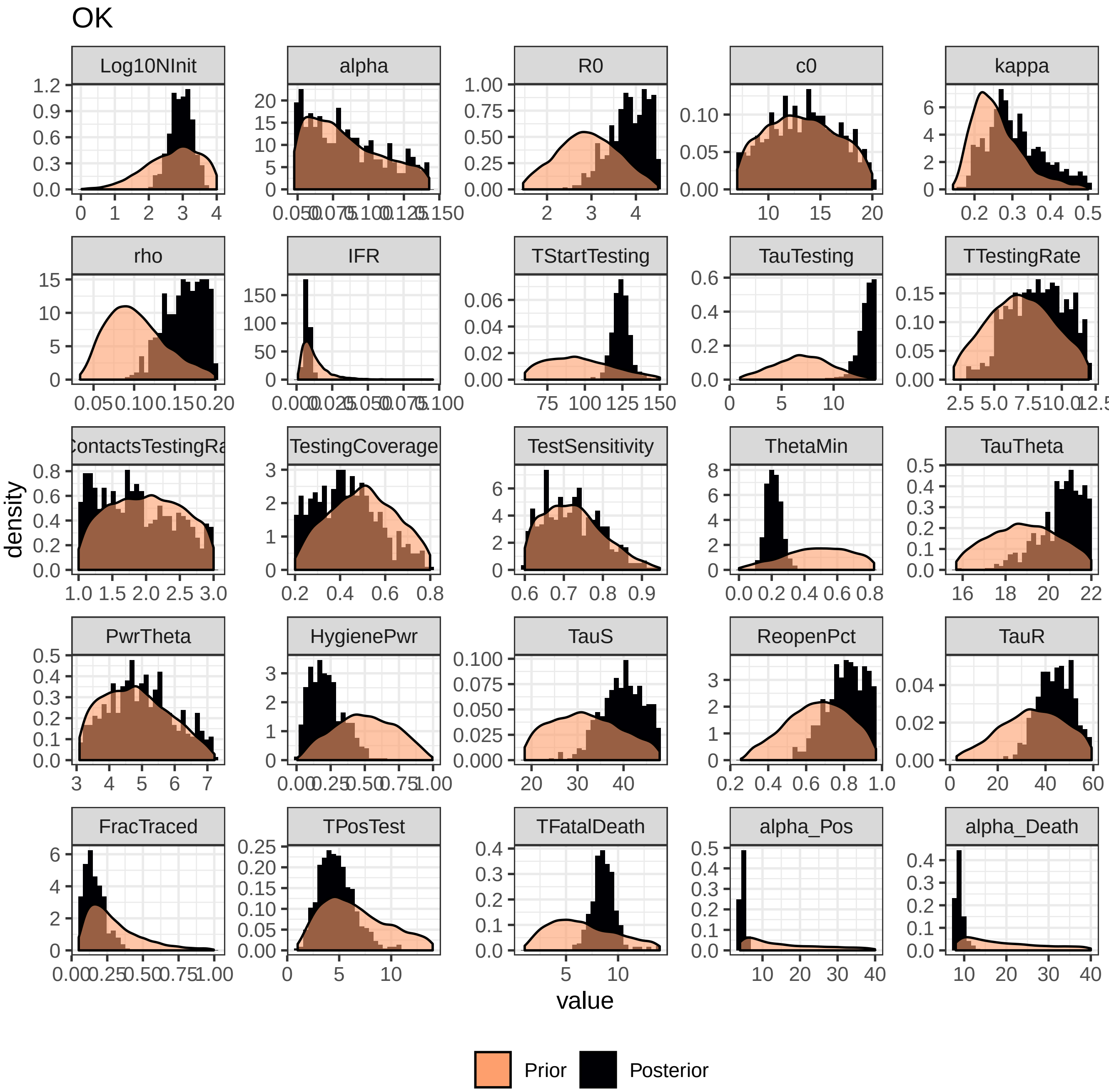
OK
Log10NInit
alpha
R0
c0
kappa
rho
IFR
TStartTesting
TauTesting
TTestingRate
ContactsTestingRa
TestingCoverage
TestSensitivity
ThetaMin
TauTheta
PwrTheta
HygienePwr
TauS
ReopenPct
TauR
FracTraced
TPosTest
TFatalDeath
alpha_Pos
alpha_Death
density
value
Prior
Posterior

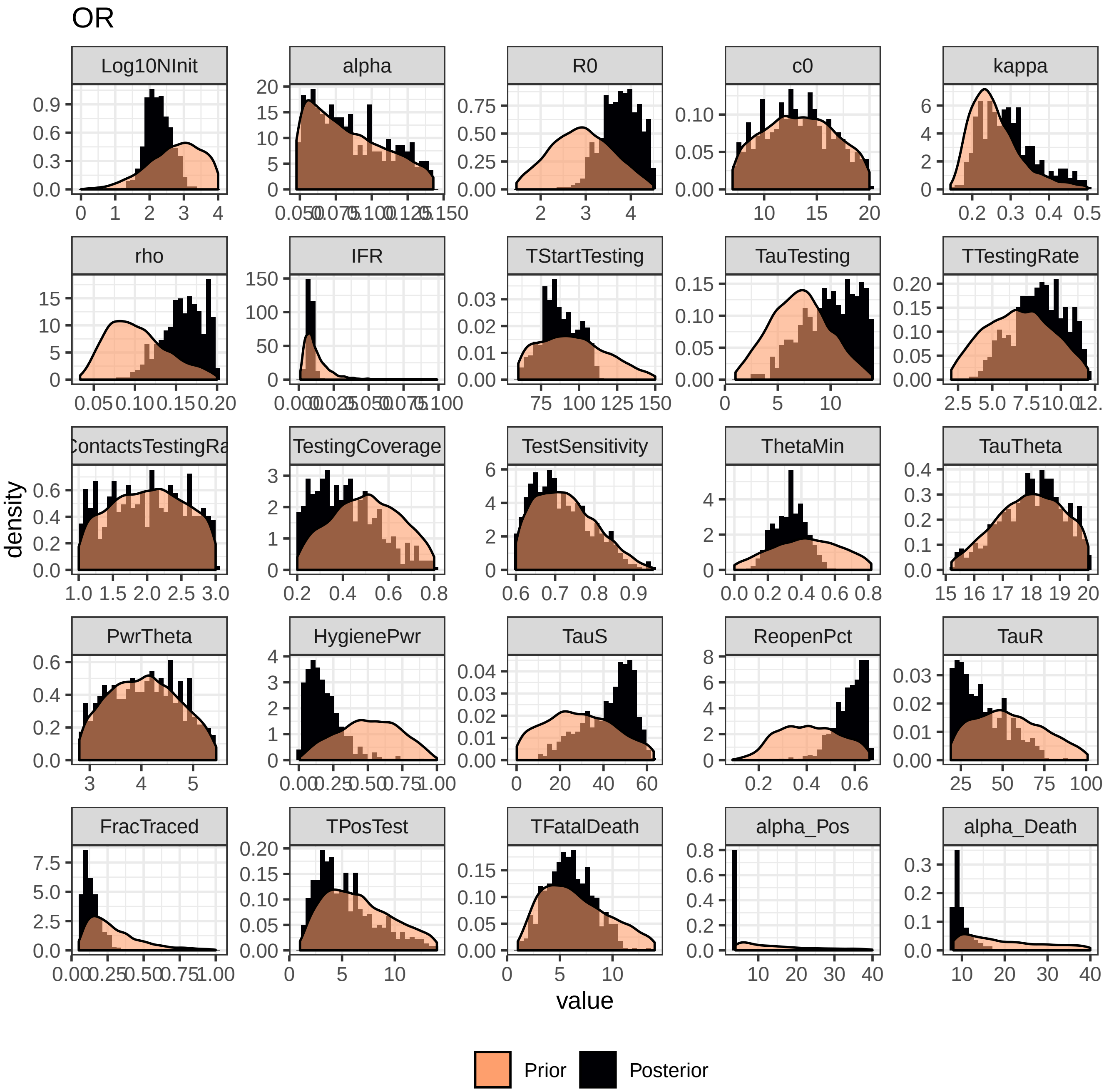

OR
Log10NInit
alpha
R0
c0
kappa
rho
IFR
TStartTesting
TauTesting
TTestingRate
ContactsTestingRa
TestingCoverage
TestSensitivity
ThetaMin
TauTheta
PwrTheta
HygienePwr
TauS
ReopenPct
TauR
FracTraced
TPosTest
TFatalDeath
alpha_Pos
alpha_Death
density
value
Prior
Posterior

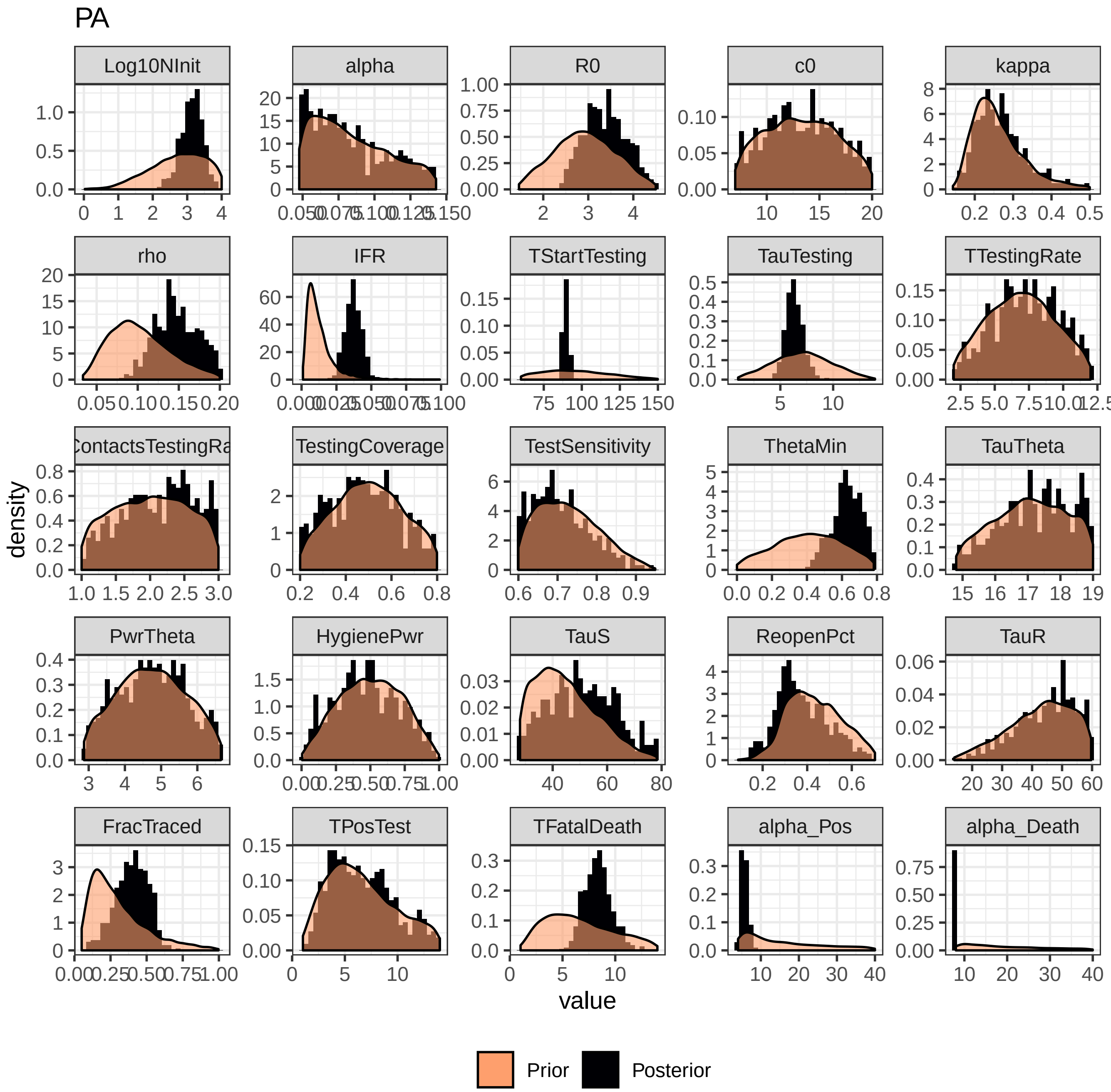
PA
Log10NInit
alpha
R0
c0
kappa
rho
IFR
TStartTesting
TauTesting
TTestingRate
ContactsTestingRa
TestingCoverage
TestSensitivity
ThetaMin
TauTheta
PwrTheta
HygienePwr
TauS
ReopenPct
TauR
FracTraced
TPosTest
TFatalDeath
alpha_Pos
alpha_Death
density
value
Prior
Posterior

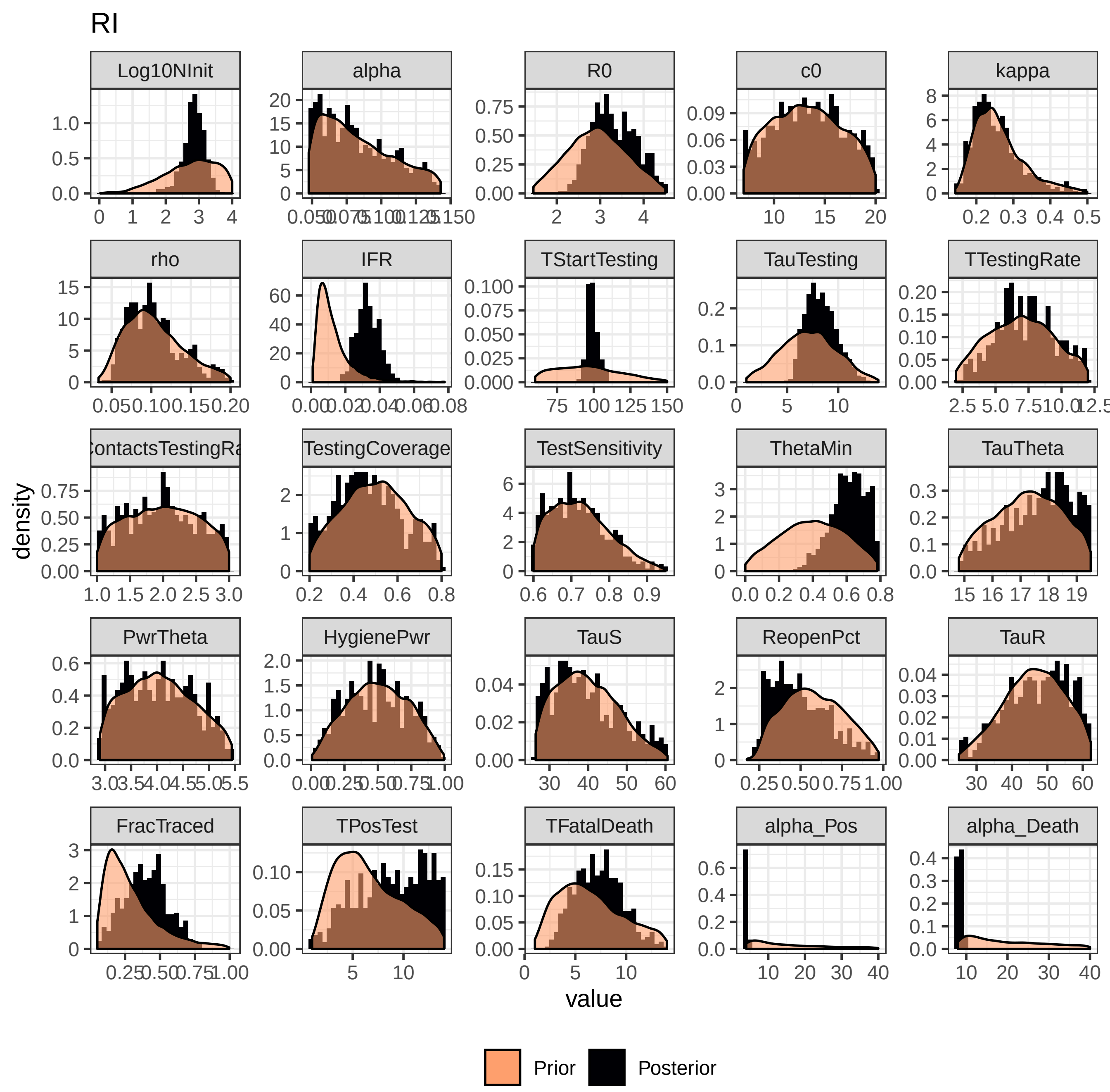

RI
Log10NInit
alpha
R0
c0
kappa
rho
IFR
TStartTesting
TauTesting
TTestingRate
TestingCoverage
TestSensitivity
ThetaMin
TauTheta
PwrTheta
HygienePwr
TauS
ReopenPct
TauR
FracTraced
TPosTest
TFatalDeath
alpha_Pos
alpha_Death
density
value
Prior
Posterior

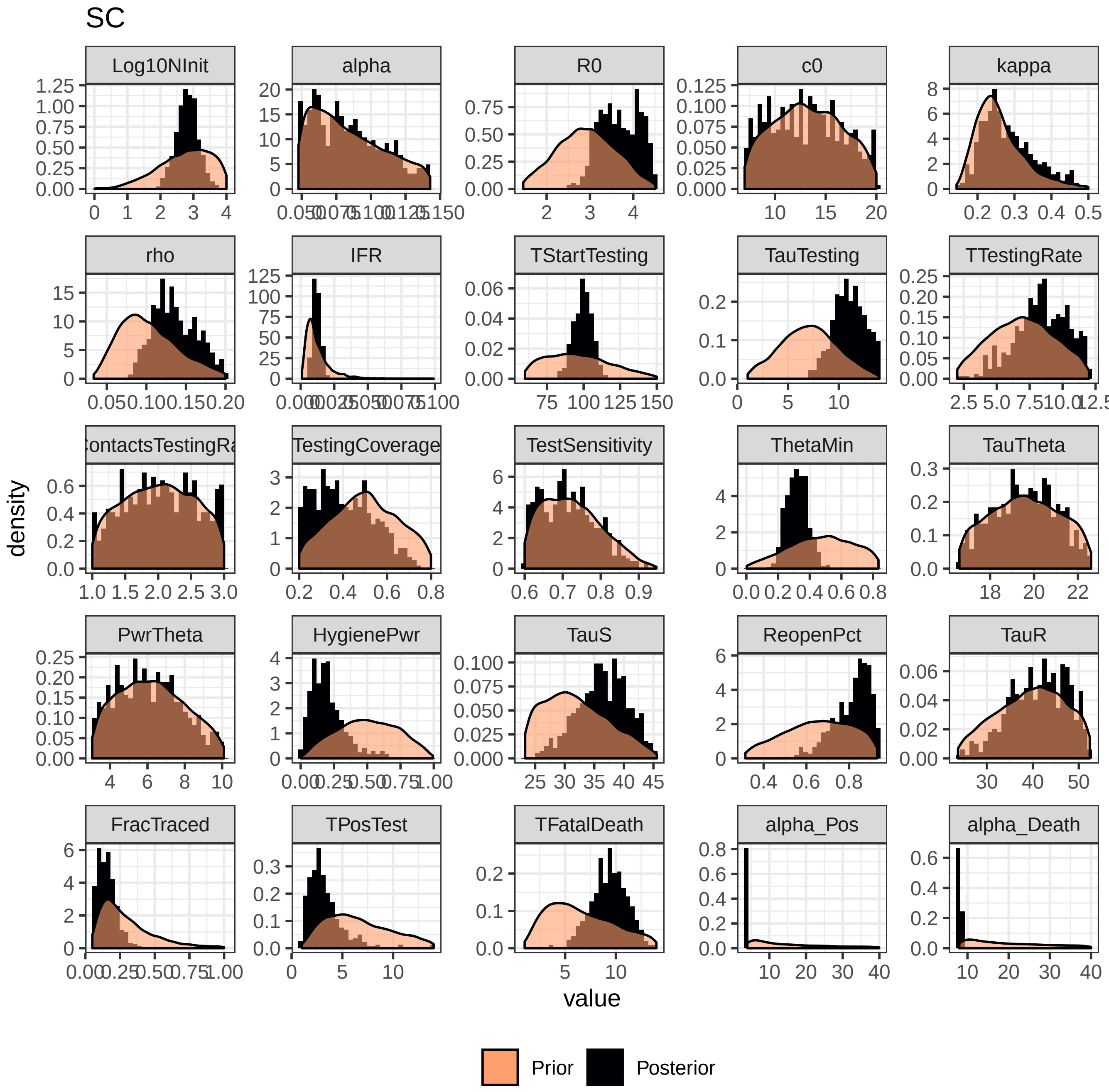
SC
Log10NInit
alpha
R0
c0
kappa
rho
IFR
TStartTesting
TauTesting
TTestingRate
TestingCoverage
TestSensitivity
ThetaMin
TauTheta
PwrTheta
HygienePwr
TauS
ReopenPct
TauR
FracTraced
TPosTest
TFatalDeath
alpha_Pos
alpha_Death
density
value
Prior
Posterior

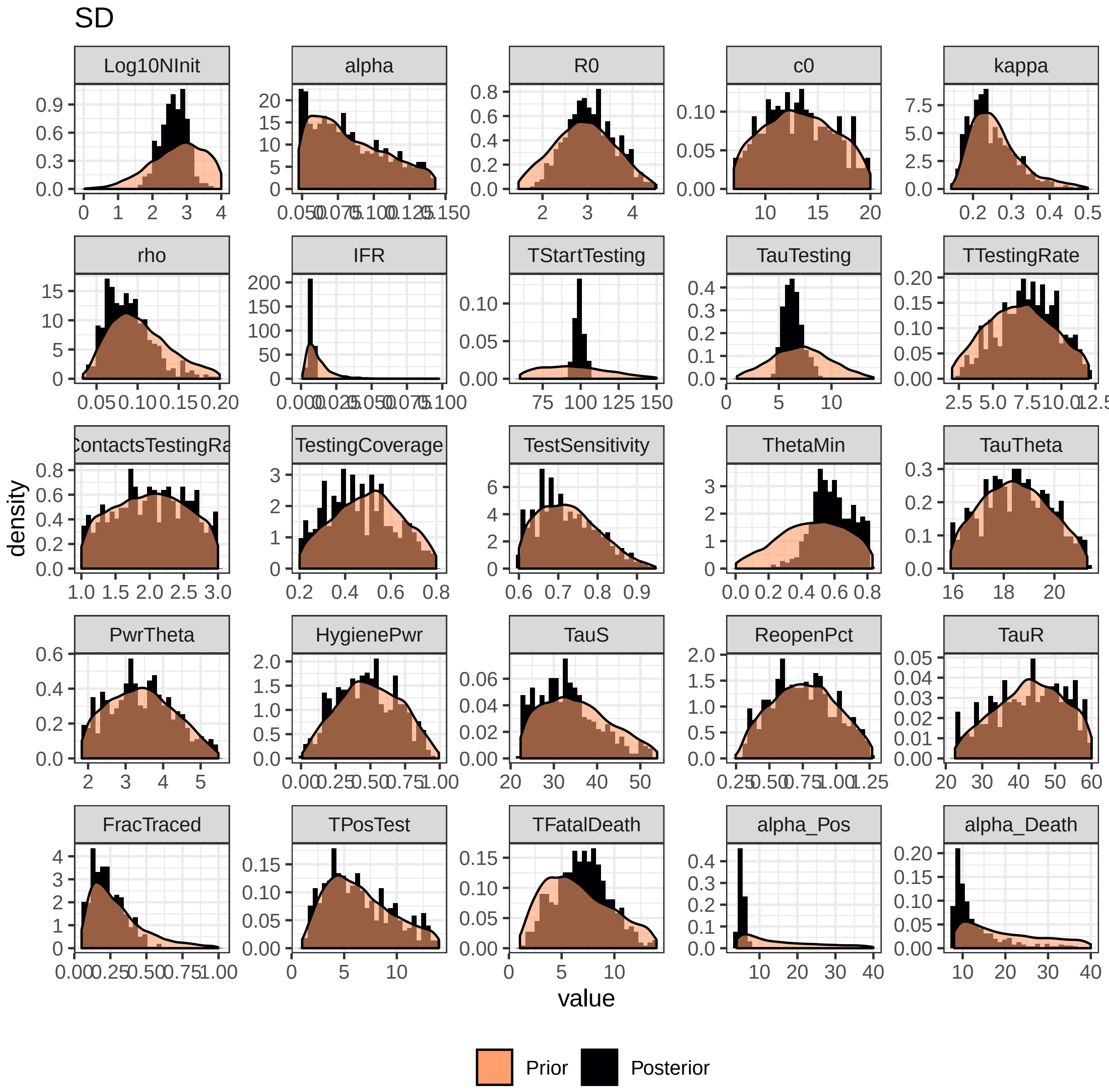
SD
Log10NInit
alpha
R0
c0
kappa
rho
IFR
TStartTesting
TauTesting
TTestingRate
ContactsTestingRa
TestingCoverage
TestSensitivity
ThetaMin
TauTheta
PwrTheta
HygienePwr
TauS
ReopenPct
TauR
FracTraced
TPosTest
TFatalDeath
alpha_Pos
alpha_Death
density
value
Prior
Posterior

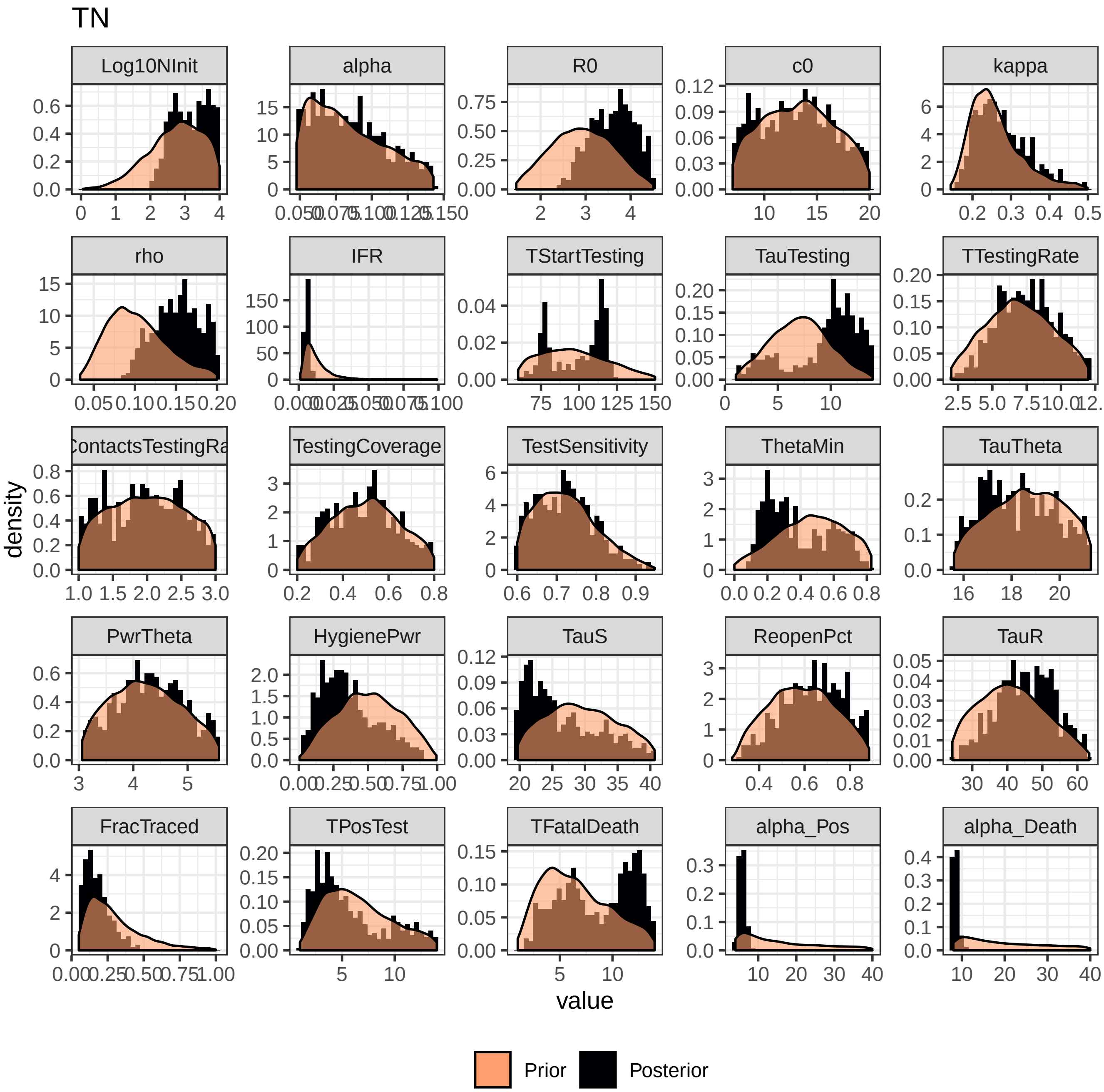
TN
Log10NInit
alpha
R0
c0
kappa
rho
IFR
TStartTesting
TauTesting
TTestingRate
ContactsTestingRa
TestingCoverage
TestSensitivity
ThetaMin
TauTheta
PwrTheta
HygienePwr
TauS
ReopenPct
TauR
FracTraced
TPosTest
TFatalDeath
alpha_Pos
alpha_Death
density
value
Prior
Posterior

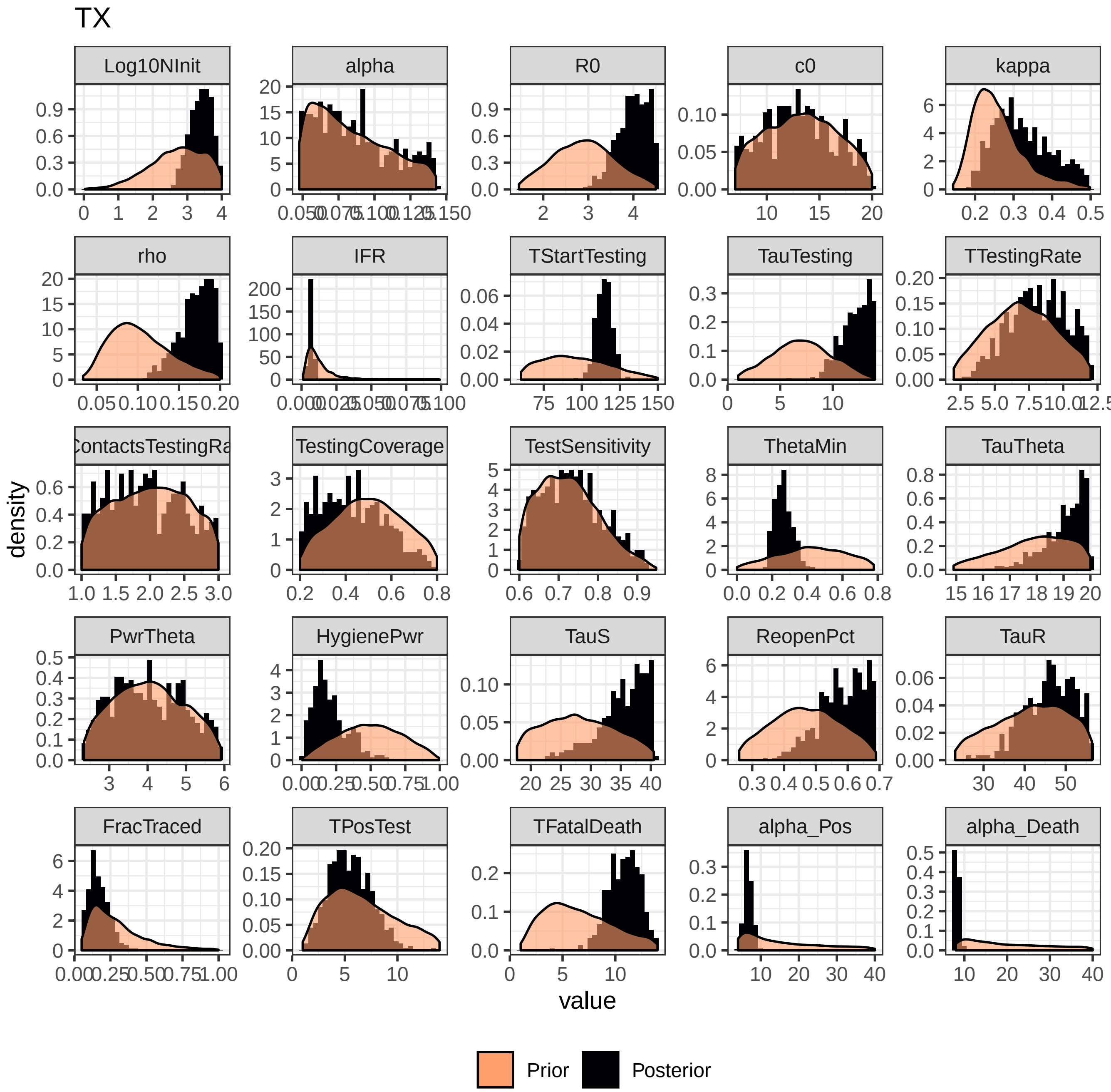

TX
Log10NInit
alpha
R0
c0
kappa
rho
IFR
TStartTesting
TauTesting
TTestingRate
ContactsTestingRa
TestingCoverage
TestSensitivity
ThetaMin
TauTheta
PwrTheta
HygienePwr
TauS
ReopenPct
TauR
FracTraced
TPosTest
TFatalDeath
alpha_Pos
alpha_Death
density
value
Prior
Posterior

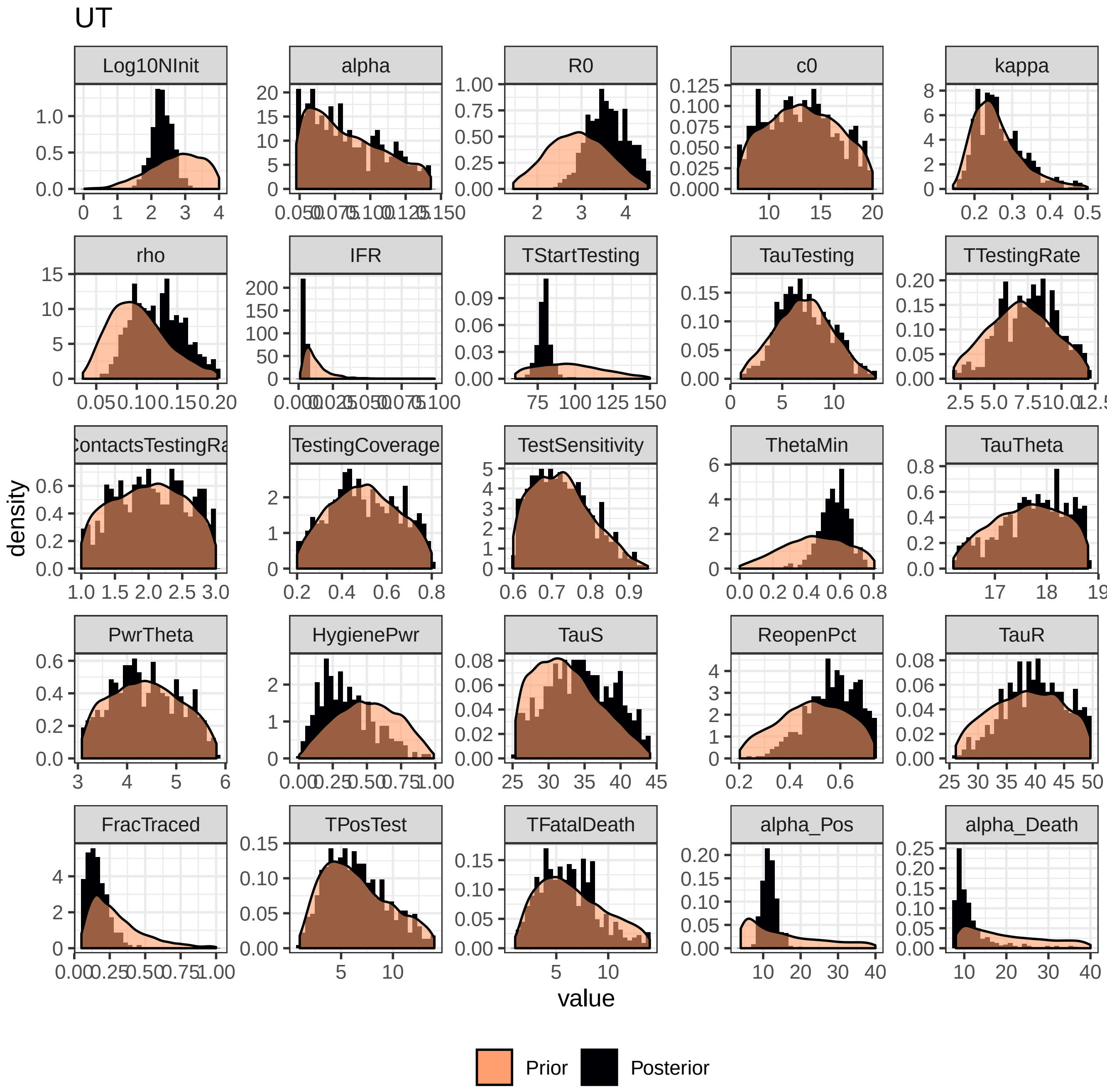
UT
Log10NInit
alpha
R0
c0
kappa
rho
IFR
TStartTesting
TauTesting
TTestingRate
ContactsTestingRa
TestingCoverage
TestSensitivity
ThetaMin
TauTheta
PwrTheta
HygienePwr
TauS
ReopenPct
TauR
FracTraced
TPosTest
TFatalDeath
alpha_Pos
alpha_Death
density
value
Prior
Posterior

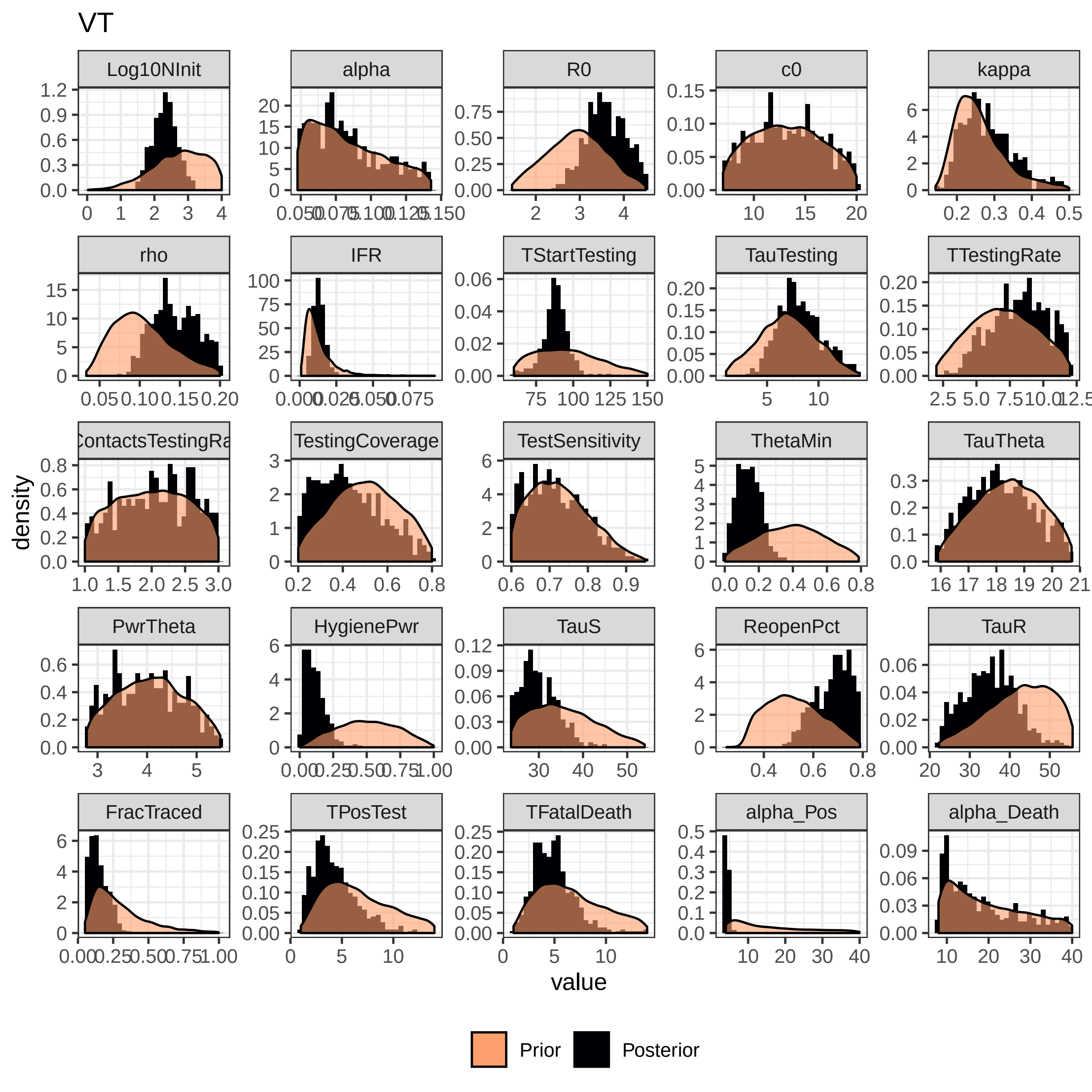
VT
Log10NInit
alpha
R0
c0
kappa
rho
IFR
TStartTesting
TauTesting
TTestingRate
ContactsTestingRa
TestingCoverage
TestSensitivity
ThetaMin
TauTheta
PwrTheta
HygienePwr
TauS
ReopenPct
TauR
FracTraced
TPosTest
TFatalDeath
alpha_Pos
alpha_Death
density
value
Prior
Posterior

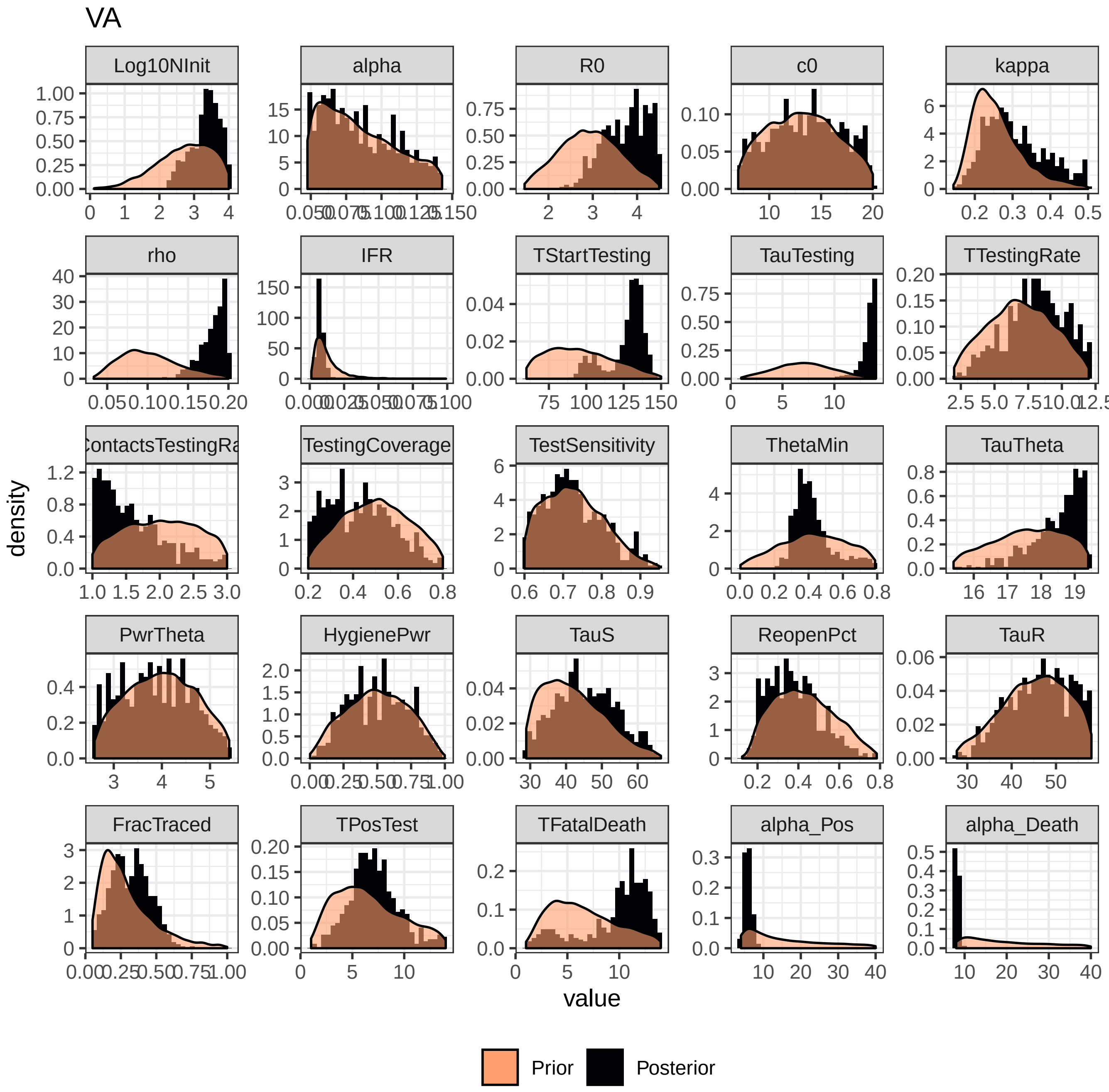
VA
Log10NInit
alpha
R0
c0
kappa
rho
IFR
TStartTesting
TauTesting
TTestingRate
ContactsTestingRa
TestingCoverage
TestSensitivity
ThetaMin
TauTheta
PwrTheta
HygienePwr
TauS
ReopenPct
TauR
FracTraced
TPosTest
TFatalDeath
alpha_Pos
alpha_Death
density
value
Prior
Posterior

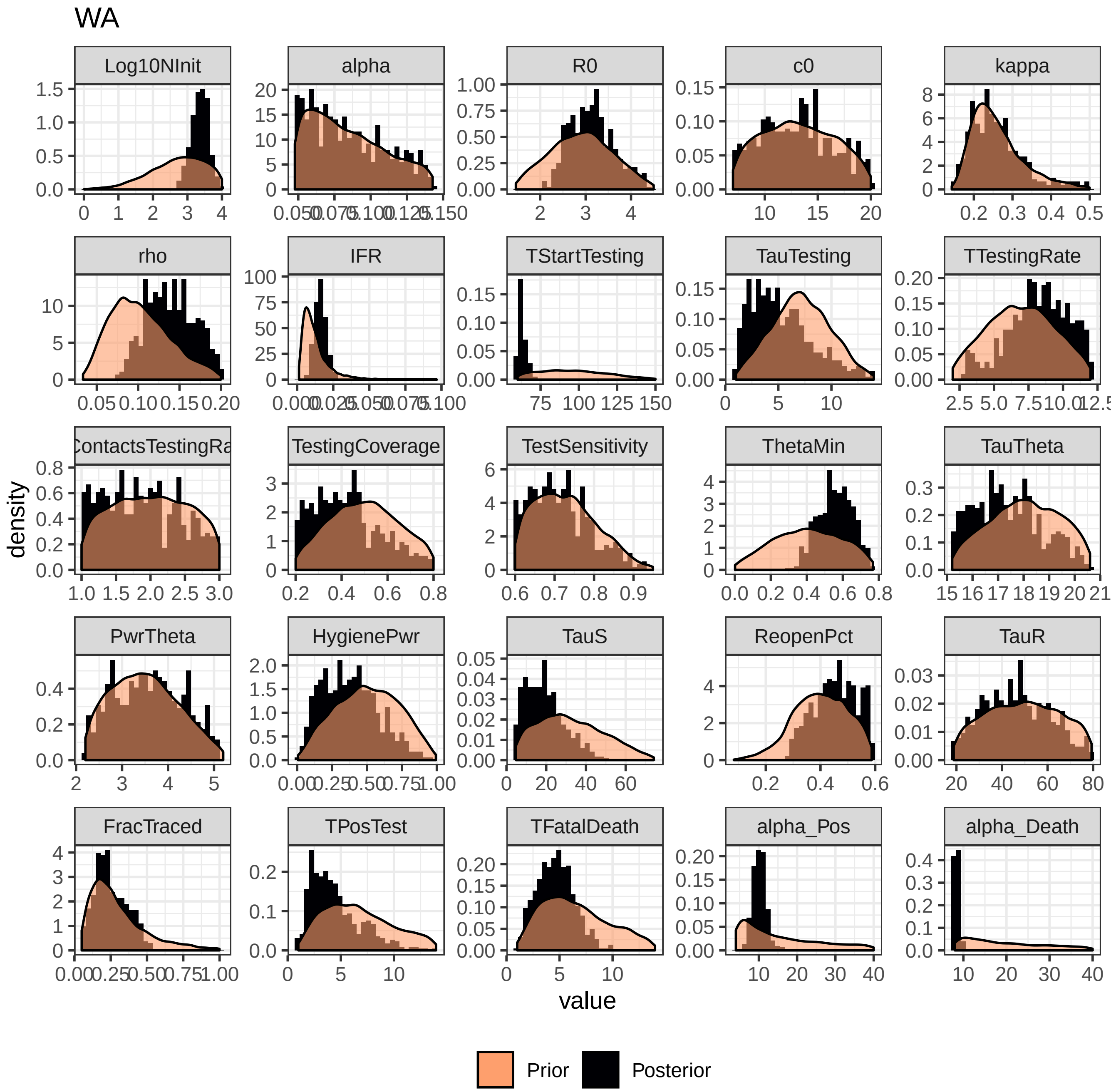
WA
Log10NInit
alpha
R0
c0
kappa
rho
IFR
TStartTesting
TauTesting
TTestingRate
ContactsTestingRa
TestingCoverage
TestSensitivity
ThetaMin
TauTheta
PwrTheta
HygienePwr
TauS
ReopenPct
TauR
FracTraced
TPosTest
TFatalDeath
alpha_Pos
alpha_Death
density
value
Prior
Posterior

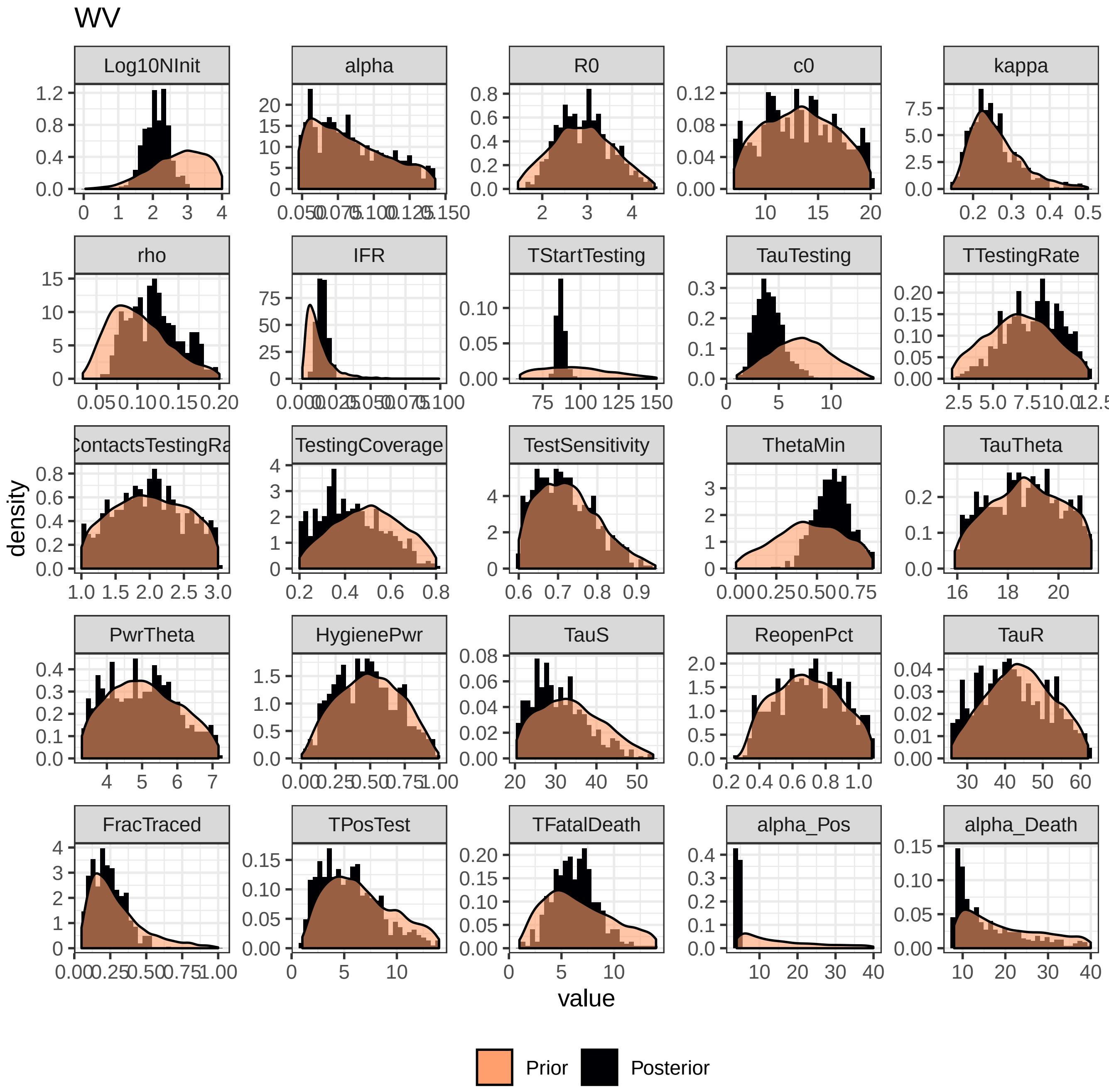
WV
Log10NInit
alpha
R0
c0
kappa
rho
IFR
TStartTesting
TauTesting
TTestingRate
ContactsTestingRa
TestingCoverage
TestSensitivity
ThetaMin
TauTheta
PwrTheta
HygienePwr
TauS
ReopenPct
TauR
FracTraced
TPosTest
TFatalDeath
alpha_Pos
alpha_Death
density
value
Prior
Posterior

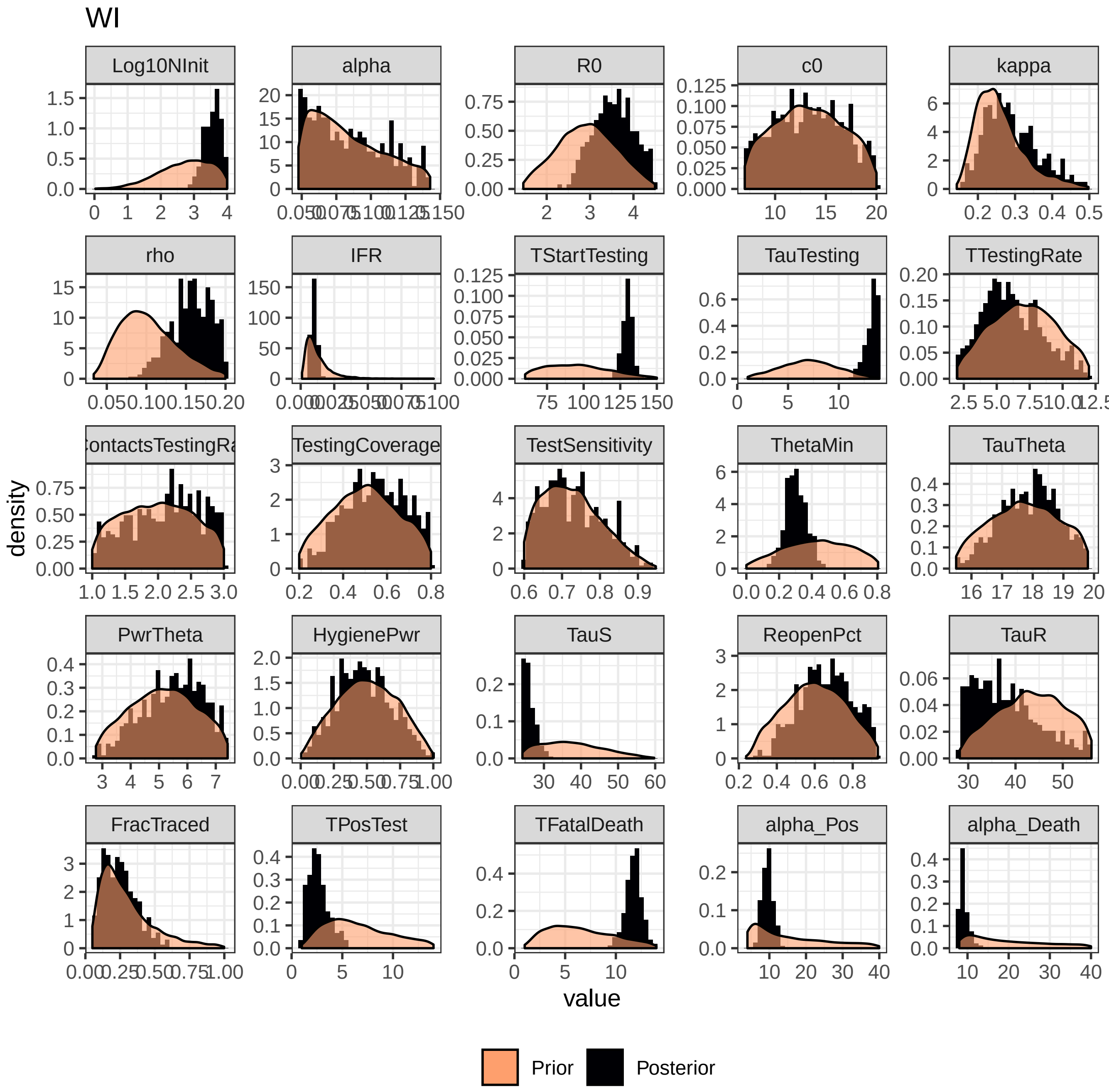
WI
Log10NInit
alpha
R0
c0
kappa
rho
IFR
TStartTesting
TauTesting
TTestingRate
ContactsTestingRa
TestingCoverage
TestSensitivity
ThetaMin
TauTheta
PwrTheta
HygienePwr
TauS
ReopenPct
TauR
FracTraced
TPosTest
TFatalDeath
alpha_Pos
alpha_Death
density
value
Prior
Posterior

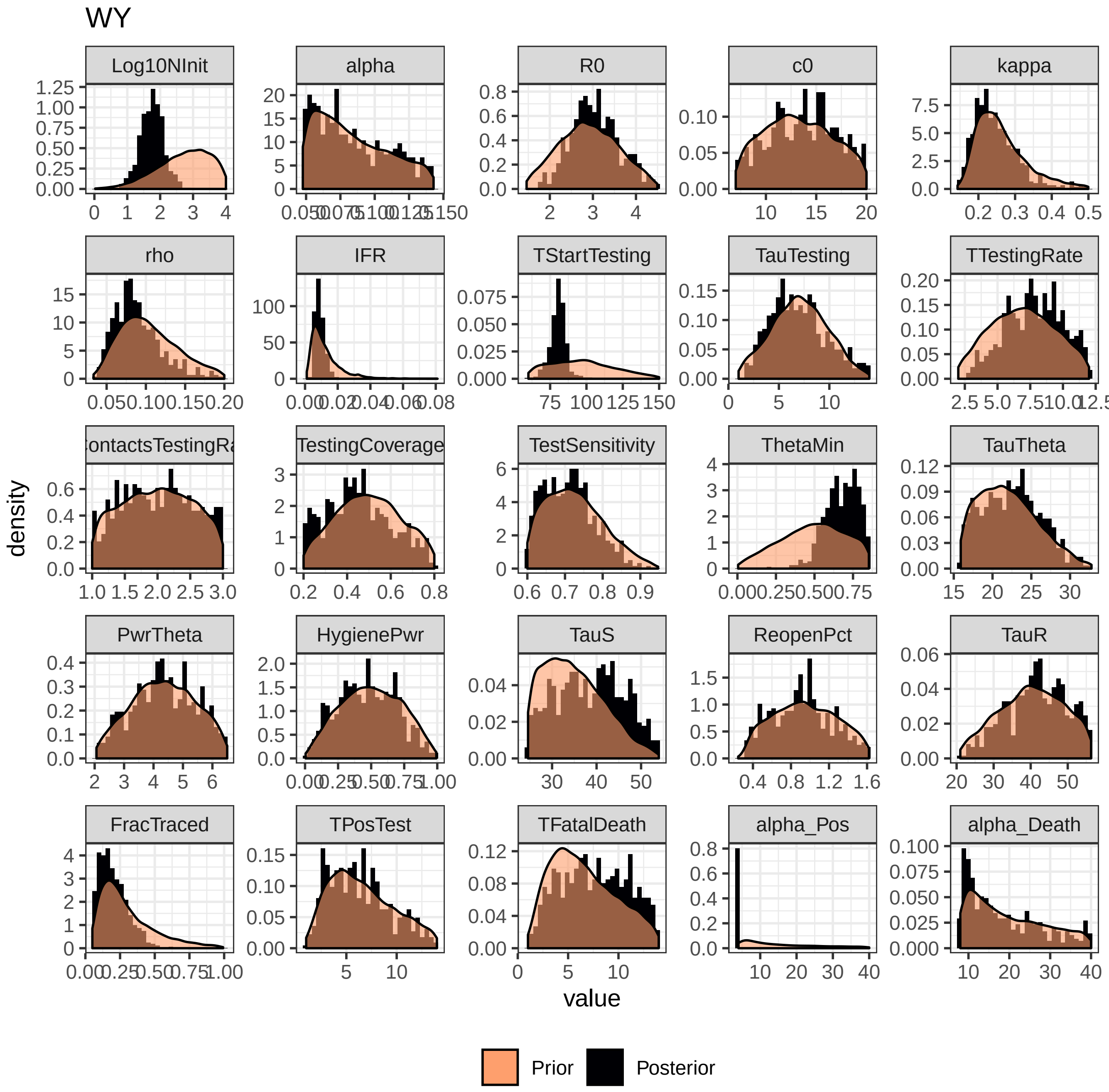
WY
Log10NInit
alpha
R0
c0
kappa
rho
IFR
TStartTesting
TauTesting
TTestingRate
TestingCoverage
TestSensitivity
ThetaMin
TauTheta
PwrTheta
HygienePwr
TauS
ReopenPct
TauR
FracTraced
TPosTest
TFatalDeath
alpha_Pos
alpha_Death
density
value
Prior
Posterior
